# Untargeted metabolomic analysis investigating links between unprocessed red meat intake and markers of inflammation

**DOI:** 10.1016/j.ajcnut.2023.08.018

**Published:** 2023-09-01

**Authors:** Alexis C. Wood, Goncalo Graca, Meghana Gadgil, Mackenzie K. Senn, Matthew A. Allison, Ioanna Tzoulaki, Philip Greenland, Timothy Ebbels, Paul Elliott, Mark O. Goodarzi, Russell Tracy, Jerome I. Rotter, David Herrington

**Affiliations:** 1 United States Department of Agriculture (USDA)/ARS Children’s Nutrition Research Center, Baylor College of Medicine, TX, United States; 2 Section of Bioinformatics, Division of Systems Medicine, Department of Metabolism, Digestion and Reproduction, Faculty of Medicine, Imperial College London, London, United Kingdom; 3 Division of General Internal Medicine, Department of Medicine, University of California, San Francisco, CA, United States; 4 Department of Family Medicine, University of California, San Diego, La Jolla, CA, United States; 5 Department of Hygiene and Epidemiology, University of Ioannina Medical School, Ioannina, Greece; 6 Department of Epidemiology and Biostatistics, Imperial College London School of Public Health, London*,* United Kingdom; 7 Departments of Preventive Medicine and Medicine, Feinberg School of Medicine, Northwestern University, Chicago, IL, United States; 8 Biomolecular Medicine, Department of Surgery and Cancer, Imperial College London, London, United Kingdom; 9 Division of Endocrinology, Diabetes, and Metabolism, Department of Medicine, Cedars-Sinai Medical Center, Los Angeles, CA, United States; 10 Laboratory for Clinical Biochemistry Research, University of Vermont, Burlington, VT, United States; 11 The Institute for Translational Genomics and Population Sciences, Department of Pediatrics, The Lundquist Institute for Biomedical Innovation at Harbor-UCLA Medical Center, Torrance, CA, United States; 12 Section on Cardiovascular Medicine, Department of Internal Medicine, Wake Forest School of Medicine; Medical Center Boulevard, Winston-Salem, NC, United States

**Keywords:** Red meat, inflammation, C-reactive protein, metabolomics, metabolome-wide association study, adiposity, BMI, biomarker

## Abstract

**Background:**

Whether red meat consumption is associated with higher inflammation or confounded by increased adiposity remains unclear. Plasma metabolites capture the effects of diet after food is processed, digested, and absorbed, and correlate with markers of inflammation, so they can help clarify diet-health relationships.

**Objective:**

To identify whether any metabolites associated with red meat intake are also associated with inflammation.

**Methods:**

A cross-sectional analysis of observational data from older adults (52.84% women, mean age 63 ± 0.3 y) participating in the Multi-Ethnic Study of Atherosclerosis (MESA). Dietary intake was assessed by food-frequency questionnaire, alongside C-reactive protein (CRP), interleukin-2, interleukin-6, fibrinogen, homocysteine, and tumor necrosis factor alpha, and untargeted proton nuclear magnetic resonance (^1^H NMR) metabolomic features. Associations between these variables were examined using linear regression models, adjusted for demographic factors, lifestyle behaviors, and body mass index (BMI).

**Results:**

In analyses that adjust for BMI, neither processed nor unprocessed forms of red meat were associated with any markers of inflammation (all *P* > 0.01). However, when adjusting for BMI, unprocessed red meat was inversely associated with spectral features representing the metabolite glutamine (sentinel hit: *β* = −0.09 ± 0.02, *P* = 2.0 × 10^−5^), an amino acid which was also inversely associated with CRP level (*β* = −0.11 ± 0.01, *P* = 3.3 × 10^−10^).

**Conclusions:**

Our analyses were unable to support a relationship between either processed or unprocessed red meat and inflammation, over and above any confounding by BMI. Glutamine, a plasma correlate of lower unprocessed red meat intake, was associated with lower CRP levels. The differences in diet-inflammation associations, compared with diet metabolite-inflammation associations, warrant further investigation to understand the extent that these arise from the following: *1)* a reduction in measurement error with metabolite measures; *2)* the extent that which factors other than unprocessed red meat intake contribute to glutamine levels; and *3)* the ability of plasma metabolites to capture individual differences in how food intake is metabolized.

## Introduction

Every 5 years, the USDA releases the Dietary Guidelines for Americans containing an expert panel’s dietary advice for good health [1]. The most current Dietary Guidelines for Americans recommend that red meat intake is minimized, which mirrors reduced red meat intake in healthy dietary patterns, such as the Dietary Approaches to Stop Hypertension diet [2] and a Mediterranean-style diet [3]. One of the reasons for recommending that red meat consumption is minimized is because individuals with cardiovascular disease (CVD) often report consuming higher amounts of red meat [4,5]. However, some, but not all, observational studies report the increased risk is only associated with processed, and not unprocessed, red meat (e.g., [6,7]). Others have reported that the association is confounded by higher BMI (kg/m^2^) [8,9]. Meanwhile, a meta-analysis of results from randomized controlled trials, in which the amount of red meat intake participants consume (including zero intake) is randomly assigned, does not report differences in conventional CVD risk factors associated with intake levels of either processed or unprocessed meat intake [10,11].

One potential pathway between red meat intake and CVD risk involves higher levels of inflammation [[12], [13], [14], [15], [16]]. Plasma metabolites reflect, in part, dietary intake [[17], [18], [19], [20], [21], [22]], where metabolites capture the effects of diet after food is processed, digested, and absorbed, and markers of inflammation [[23], [24], [25]]. Thus, metabolomic investigations could provide unique information about the relationship between red meat intake and risk for CVD. Despite this promise, we are not aware of any large-scale investigations using metabolomic analysis to examine relationships between red meat intake and markers of inflammation.

The overarching goal of the current analyses was to gain insights into the association of red meat with inflammation. Using data on a large, multiethnic sample of older US adults participating in the MultiEthnic Study of Atherosclerosis (MESA), we sought to: *1)* examine whether processed and unprocessed meat intake are each associated with markers of inflammation; *2)* separate conduct metabolome-wide associations studies (MWAS) with unprocessed and processed red meat intake; *3)* examine whether any molecules associated with unprocessed and/or processed red meat intake were also associated with plasma markers of inflammation; and *4)* establish the extent that BMI confounded any relationships identified.

## Methods

### Study population

The MESA is a longitudinal cohort study of US adults designed to identify factors that influence the conversion of subclinical atherosclerosis to overt CVD. Participants were recruited from 6 metropolitan areas across the United States (Baltimore County, Maryland; Chicago, Illinois; Forsyth County, North Carolina; New York, New York; Los Angeles County, California; and St. Paul, Minnesota) for a baseline examination conducted between 2000 and 2002, for which recruitment began in 1999. Exclusion criteria for participation in MESA included: age <45 or >84 y; self-reported race other than African-American Black, Asian, or Caucasian/White (see below); the presence of overt CVD; active treatment of cancer; pregnancy; any serious medical condition which would prevent long-term participation; weight >300 pounds (136 kilograms); cognitive inability; living in a nursing home or on the waiting list for a nursing home; plans to leave the community within 5 y; lack of fluency in either English, Spanish, Cantonese or Mandarin; and having undergone a chest computed tomography scan in the past year. Exclusion criteria were ascertained through self-report via a screening form that was administered over the phone (except for a small number of cases when it was administered in person at the participant’s home). As part of the screening form, participants were first asked: “Are you Spanish, Hispanic, or Latino?” Subsequently, participants were asked, “Which of the following best describes your race?” for which respondents could select more than one answer from the following options: African-American or Black; Asian (with suboptions Chinese, Filipino, Japanese, Korean, Vietnamese, and Asian Indian); Caucasian or White; Native Hawaiian or other Pacific Islander (suboptions: Guamanian or Cham, Samoan, Micronesia, and Tahitian); American Indian or Alaska Native; and “Did not identify.” The baseline participant population for the MESA cohort consisted of 6814 men and women aged 45–84 y, with self-reported White (*N* = 2,623), Black (*N* = 1891), Hispanic (*N* = 1496), and Chinese (*N* = 804) ancestry [26]. A subset of 3955 randomly selected MESA participants had their plasma metabolome assessed by untargeted ^1^H NMR analysis [27,28]. Further details on the recruitment methods and procedures for MESA are available elsewhere [26].

**FIGURE 1:**
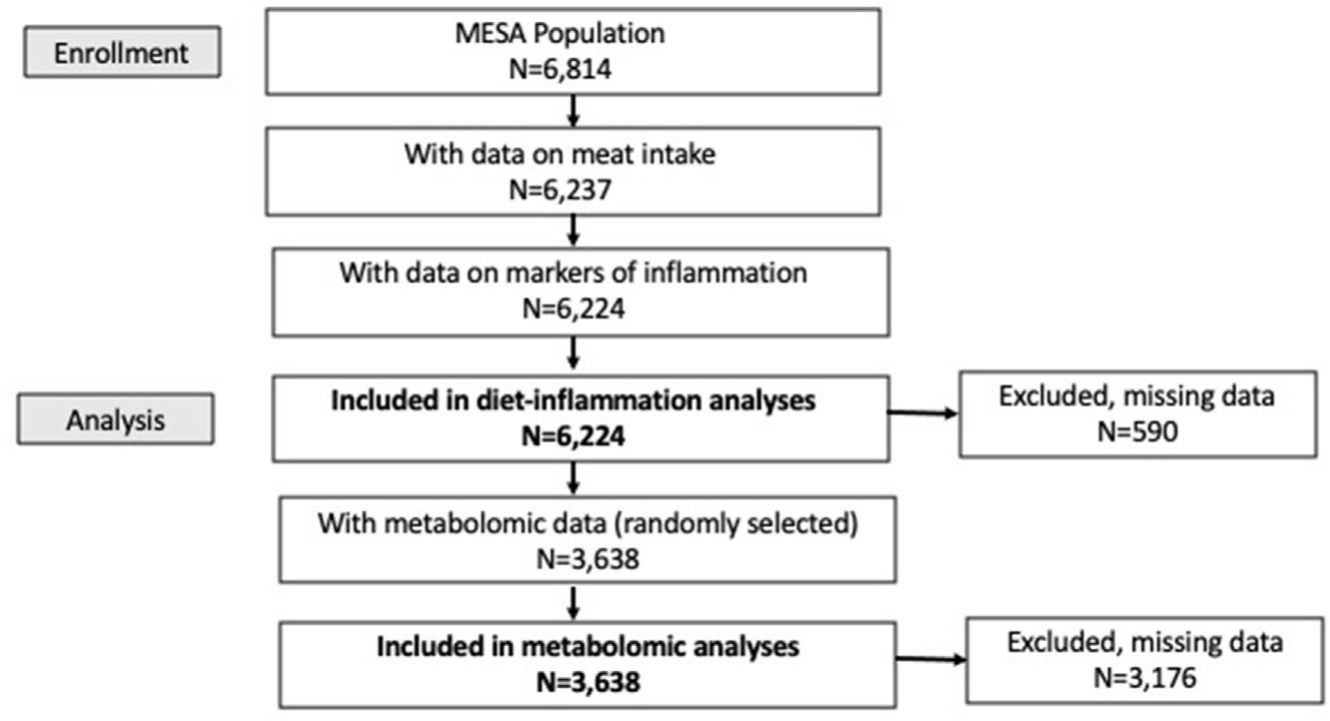
Participant flow diagram.

We excluded 577 participants with missing information on red meat intake and 13 participants with missing information for all markers of inflammation, leaving a final sample of *N* = 6224 for analysis (*N* = 3638 for analysis involving the untargeted metabolomic data, Figure 1), with no further exclusion criteria.

*Ethics.* The protocol was approved by the institutional review board (IRB) at each of the study centers (Wake Forest University IRB, Columbia University IRB, Johns Hopkins University IRB, University of Minnesota IRB, Northwestern University IRB, and University of California at Los Angeles IRB), and all participants gave informed consent.

### Procedures

During the baseline examination, clinical characteristics and anthropometric measurements were obtained by trained personnel using standardized protocols. A fasting blood sample was also drawn (after a minimum 8-h fast) and stored at −80°C until analysis. Questionnaires were administered to collect self-reported demographic data, including age, gender, race, dietary information, and health behaviors.

### Measures

*Dietary intake.* The MESA food-frequency questionnaire asked about intake frequency and average serving size for 120 foods (including mixed dishes such as chow mein) over the past 12 mo. For each item, participants could choose from 9 frequency options, which ranged from “rare or never” to a maximum of “≥2 times/d” for foods and a maximum of “≥6 times/d” for beverages. The individual foods were coalesced into average servings per day for each of 49 food groups (Supplementary Table 1), which included unprocessed red meat (hamburger, cheeseburger, meat loaf, hash, beef, pork or lamb steaks, roasts, barbeque or ribs, picadillo, carne guisada, menudo, chili with meat and beans, oriental noodles with meat [saimen, ramen, and wonton mein], red chile con carne with meat, green chili con carne with meat, pasta with tomato sauce and meat, poultry or seafood, including spaghetti and lasagna, pasta with cream sauce, cheese and meat, poultry or seafood, including tuna noodle casserole, stir-fried beef, pork or chicken with vegetables, including beef broccoli, burritos, quesadillas or fajitas with meat, enchilada, tamales, tacos or nachos with meat, meat, chicken or turkey stew, and pot pie or empanada), and processed red meat (ham, hot dogs, bologna, salami, other, lunch meats; liver, including chicken livers, other organ meats, ham hocks, pigs' feet, chicharones, sausage, chorizo, scrapple, and bacon).

^*1*^*H NMR untargeted metabolomics.* Metabolomics data were available for *N* = 3638 randomly selected MESA participants. Characteristics of the participants with and without metabolomic data were highly similar, with nominally significant differences found for only a few traits (Supplementary Table 2).

Untargeted metabolomic profiles were generated via a standard ^1^H NMR one-dimensional spectrum with water suppression and a T2-edited spectrum that used a Carr-Purcell-Meiboom-Gill sequence using stored fasting serum samples from the baseline clinic visit. After thawing, 300 μl of serum were mixed with 300-μl phosphate buffer in Eppendorfs and subjected to centrifugation, then kept at 4°C until analysis. For each 96-tube rack, an additional sample was included for quality control [27,28].

Bruker DRX600 spectrometer (Bruker Biospin) operating at 600 MHz was used for acquiring All ^1^H NMR spectra. Standard water suppressed a 1-dimensional spectrum, and a Carr-Purcell-Meiboom-Gill spectrum was obtained for each sample [27]. The spectra were automatically phased and baseline corrected, and the chemical shifts were calibrated to the glucose signal at 5.233 ppm using TOPSPIN 3.1 (Bruker Biospin). Spectral data were imported into MATLAB (version 8.3 [R2014a] Mathworks Inc), spectral intensities organized into rectangular matrices (samples as rows, chemical shifts as columns), the regions containing the residual water peak (4.5–5.0 ppm) and those containing only baseline were removed (the spectral range from 0.5–10 ppm was kept). Further processing was performed, including peak alignment and normalization using the recursive segment-wise peak alignment algorithm [28] and probabilistic quotient normalization [29] methods, respectively.

*Metabolite identification.* Metabolites were annotated using with the aid of additional spectral information gathered from 2D NMR experiments (2D JRES, Correlation Spectroscopy, Total Correlation Spectroscopy, and Heteronuclear single quantum correlation spectroscopy) and statistical correlation methods (Statistical Total Correlation Spectroscopy and Subset Optimization by Reference Matching). This information was then compared with available inhouse and publicly available databases (Human Metabolome Database [30]) as well as with published data on human serum and plasma metabolite components. Spike-in experiments were used to confirm metabolite identities when feasible. The annotation information was organized into manually defined bins. For this purpose, spectra were divided into smaller spectral regions enclosing each of the detected peaks, which were annotated to one or more metabolites or macromolecules according to the information gathered using the 2D NMR experiments and statistical correlation methods. Overall, ∼75% of molecules have been annotated with at least one associated metabolite.

### Covariates

*Demographic factors, lifestyle behaviors, and physical activity.* Age*,* sex*,* household income, highest education levels, and smoking status were obtained through inperson interviews with trained assessors. Participants’ smoking information was categorized into current smokers/former smokers/never smokers. Physical activity was assessed using a detailed, semiquantitative questionnaire adapted from the Cross-Cultural Activity Participation Study (B. Ainsworth, personal communication, San Diego State University).

*Anthropometric measures.* Height and weight were measured in duplicate by trained study staff. A mean of both measurements was used to calculate BMI as weight in kilograms (kg) divided by height in meters (m) squared (kg/m^2^).

### Analyses

All analyses were conducted using the latest version of R software (version 4.0.5, 64-bit) [31]. Like all 64-bit software, double-precision floating-point format (FP64 or float64) and 11 bits for the exponent are employed. The Institute of Electrical and Electronics Engineers’ standard value for FP64 machine epsilon (ε) is 2.22 × 10^-16^. Therefore, all *P* values in the current analyses are censored at 2.0 × 10^-16^ [32].

*Data preparation.* Distributions of all continuous variables (except spectral features) were examined using measures of central tendency and a visual inspection of histograms. Normality was defined as having both a skew and kurtosis in the range of −1 to +1. Where distributions did not meet these criteria, variables were transformed to normality using an inverse normal transformation prior to use as an outcome in linear regressions and/or before use in t-tests. For spectral feature data, each batch was log-transformed, median-centered, and scaled to unit median absolute deviation prior to analysis. The data thereafter was back-scaled to a pooled median absolute deviation and back-centered to the mean of the batch medians (see [27] for more details and validation of the quality control, harmonization, and preparation of the spectral feature data).

*Participant characteristics.* Raw (untransformed) data were used to provide means (±SD) for continuous variables, or total number (*N*) and percentage (%) for categorical or ordinal variables, stratified by quintile of total red meat intake (the sum of processed and unprocessed red meat intake). Differences in demographic, dietary, and health information between quintiles were examined using linear regression models for continuous variables and chi-squared tests of difference for categorical variables.

*Associations of processed and unprocessed red meat intake with markers of inflammation.* In separate linear regression models controlled for age, sex, race/ethnicity, data collection site, smoking status, highest education level, income level, daily energy intake, and physical activity level as fixed effect covariates, unprocessed meat intake, and processed meat intake were specified as predictors, and markers of inflammation as outcomes. Significance was set at a Bonferroni corrected *P* < 0.004 (0.05 / [2 dietary intake variables × 6 markers of inflammation = 0.004]). Standardized parameter estimates (effect sizes [*β*] and SEs) are presented in the text and tables. However, to facilitate interpretability for any significant associations, the change in outcome per serving of red meat is also presented, which is analogous to information that can be derived from non-standardized parameter estimates. For any significant associations, the proportion of variance in the inflammatory marker explained by the dietary intake variable was calculated as the difference in the *R*^*2*^ between the following: *1)* the linear regression model with the dietary intake variable and all covariates as predictors, and *2)* the linear regression model with only the covariates as predictors.

*MWASsof processed and unprocessed red meat intake.* The associations of each spectral feature with each of processed and unprocessed red meat intake were analyzed using separate linear regression models, which controlled for age, race, gender, and data collection site as fixed effect covariates. Significance was determined using permutation analysis. For this procedure, as described in [29,33], for each MWAS (i.e., for each processed and unprocessed red meat intake), 10,000 permutations were conducted in which the outcome was randomly shuffled among the participants (to simulate the null hypothesis). The highest *P* value which satisfied:

$$\alpha =\mathrm{Pr}\left(\min\left\{p\right\}<\;\alpha^{\prime }\right)$$

where α′ is the MWSL, p denotes the *P* value from the i-th variable, and min{p} denotes the minimum *P* value across all associations across all permutations, at a family-wide error rate of 5% was used to determine the threshold for significance. Standardized estimates are presented for all significant associations in models, which additionally controlled for smoking status, highest education level, income level, daily energy intake, and physical activity levels as fixed effects.

To avoid problems with multicollinearity and reduce the number of frequentist statistical tests, when either MWAS showed significant associations between meat intake and groups of correlated spectral features (*r* > 0.8), only the spectral feature with the lowest *P* value for association (the sentinel spectral feature) was retained for subsequent analyses.

*Associations of sentinel spectral features with markers of inflammation.* Separate linear models were constructed for each of the inflammation markers. For each marker, 2 sets of models were analyzed; the first set included the 2 sentinel features as predictors as well as age, sex, race/ethnicity, data collection site, smoking status, highest education level, income level, daily energy intake, and physical activity level as fixed effect covariates. The second set of models additionally included the intake of unprocessed meat (i.e., the dietary variable significantly associated with the spectral features in the MWAS). Significance was determined by a Bonferroni correction. For any significant associations, the proportion of variance in the inflammatory marker explained by the spectral feature (partial *R*^*2*^) was calculated as the difference in the *R*^*2*^ between the following: *1)* a linear regression model with the spectral feature and all covariates as predictors, and *2)* a linear regression model with only the covariates as predictors.

*Exploration of dietary associations with sentinel spectral features.* Additional analyses were conducted to examine whether other dietary food groups contributed to the concentrations of any sentinel spectral features. All food groups in MESA were included, excluding those for processed and unprocessed red meat (*N* = 45; Supplementary Table 1). Linear regression models specified the sentinel spectral features as the outcomes and each of the 45 food groups as predictors in separate linear regression models, which controlled for age, sex, race/ethnicity, data collection site, smoking status, highest education level, income level, daily energy intake, and physical activity level as fixed effect covariates. Significance was determined by a Bonferroni correction.

*The role of BMI.* If BMI is involved in any relationship between red meat intake and inflammation, whether it serves as a mediator, moderator, or confounder is not clear in the existing literature. Therefore, rather than include BMI as a covariate in our main models (and potentially control for the mechanism of interest), we ran a second set of models to quantify the contribution of BMI (as either a mediator, moderator, or confounder) to any observed associations.

First, we confirmed the expected associations between BMI and processed/ unprocessed meat intake, sentinel spectral features, and markers of inflammation via parameter estimates from linear regression models specifying BMI as a predictor and age, sex, race/ethnicity, data collection site, smoking status, highest education level, income level, daily energy intake, and physical activity level as fixed effect covariates. For any significant associations, the linear regression models above were recomputed with the inclusion of BMI as a fixed effect. After this step, the “mediation” package in *R* was used to decompose any observed significant associations between red meat intake (processed or unprocessed), markers of inflammation, and spectral features into the proportion attributable to BMI and the remaining “direct” effects. *P* values around the parameter representing the proportion of any associations attributable to BMI were calculated via bootstrap-based confidence intervals (*N* = 1000 resamples; see [34] for further details).

## Results

### Participant characteristics

The analytic sample had a mean age of 63.0 ± 10.3 y and was almost equally split between genders (52.84% women, Table 1). A total of 39.6% self-reported their ancestry as White, 12.7% as Chinese, 25.8% as African-American, and 21.9% as Hispanic (Table 1).

**TABLE 1:** Baseline characteristics of Multi-Ethnic Study of Atherosclerosis participants, for the whole sample and stratified by quintile of total red meat intake

	Full sample (N = 6224)	Quintile of total red meat intake				
		1st (N = 1250)	2nd (N = 1245)	3rd (N = 1250)	4th (N = 1234)	5th (N = 1245)
*Demographics*						
Age, y^3^	63.0 (10.3)	64.6 (10.0)	63.5 (10.2)	62.3 (10.4)	61 (10.13)	59.41 (9.9)
Gender, women, *N* (%)^3^	3289 (52.84%)	816 (62.3%)	744 (59.8%)	663 (45.1%)	678 (45.1%)	510 (41.0%)
Race/ethnicity^3^						
White, *N* (%)	2467 (39.6%)	459 (36.7%)	515 (41.4%)	509 (40.7%)	524 (42.5%)	460 (37.0%)
Chinese, *N* (%)	791 (12.7%)	104 (8.3%)	185 (14.9%)	208 (16.6%)	182 (14.8%)	112 (9.0%)
African-American, *N* (%)	1606 (25.8%)	341 (27.3%)	300 (24.1%)	285 (22.8%)	301 (24.4%)	379 (30.4%)
Hispanic, *N* (%)	1360 (21.9%)	346 (27.7%)	245 (19.7%)	248 (19.8%)	227 (18.4%)	294 (23.6%)
Household income category^3^						
<$25,000, *N* (%)	1866 (31.1%)	427 (35.%)	374 (30.9%)	408 (33.9%)	331 (27.5%)	326 (27.3%)
$25,000–$49,999, *N* (%)	1731 (28.8%)	355 (29.7%)	352 (29.1%)	304 (25.3%)	365 (30.4%)	355 (29.7%)
> $50,000, *N* (%)	2406 (40.8%)	412 (34.5%)	483 (40.0%)	490 (40.8%)	506 (42.1%)	515 (43.1%)
*Health behaviors*						
BMI, kg/m^2 3^	28.0 (5.3)	27.4 (5.24)	27.60 (5.0)	28.1 (5.4)	28.4 (5.3)	29.6 (5.8)
Physical activity, MET mins/wk^3^	5554.0 (5667.3)	5438.4 (5799.7)	5361.9 (5301.0)	5638.7 (6351.6)	5514.8 (5178.8)	6594.2 (6556.7)
Smoking status^3^						
Never smoker, *N* (%)	3177 (51.1%)	706 (56.5%)	683 (55.0%)	656 (52.6%)	591 (47.9%)	541 (43.5%)
Former smoker, *N* (%)	2273 (36.6%)	458 (36.7%)	433 (34.4%)	453 (36.3%)	473 (38.3%)	456 (36.7%)
Current smoker, *N* (%)	767 (12.3%)	85 (6.8%)	139 (11.1%)	139 (11.1%)	170 (13.8%)	246 (19.8%)
*Dietary intake*						
Processed meat, svg/d^3^	0.02 (0.03)	0.02 (0.03)	0.05 (0.0)	0.10 (0.10)	0.20 (0.17)	0.51 (0.44)
Unprocessed red meat, svg/d^3^	0.06 (0.04)	0.06 (0.04)	0.18 (0.07)	0.31 (0.10)	0.46 (0.18)	0.87 (0.52)
*Markers of inflammation*						
CRP, mg/dL^1^	3.6 (5.6)	3.6 (6.3)	3.6 (5.2)	3.5 (4.8)	3.6 (5.6)	4.3 (7.1)
IL-2, pg/mL	1023.2 (443.5)	1021.1 (493.0)	985.84 (416.9)	1000.4 (414.1)	952.1 (394.6)	1022.5 (468.5)
IL-6, pg/mL	1.5 (1.2)	1.6 (1.2)	1.5 (1.2)	1.5 (1.1)	1.5 (1.2)	1.7 (1.3)
Fibrinogen (mg/dL)^1^	343.7 (72.2)	350.1 (74.9)	346.9 (75.1)	344.6 (72.6)	342.6 (70.9)	344.1 (72.1)
Homocysteine, umol/L	9.3 (4.1)	9.4 (3.2)	9.2 (4.0)	9.2 (4.3)	9.3 (3.4)	9.3 (3.7)
TNF-α, pg/mL^1^	1420.6 (439.9)	1412.9 (478.7)	1388.8 (415.2)	1370.0 (416.5)	1339.1 (396.9)	1372.2 (457.8)

^1^*P* < 0.05, ^2^*P* < 0.001, ^3^*P* < 0.001 for tests of differences by quintile of total red meat intake.

Abbreviations: BMI: Body mass index, CRP: C-Reactive Protein; MET min: Metabolic equivalent minutes, TNF-α: Tumor Necrosis Factor-α Soluble Receptors

Comparing the sample by quintile of red meat intake, the quintiles had different race/ethnicity distributions (χ^2^ = 116; df = 12; *P* < 2.0 × 10^-16^; Table 1). Participants with a higher red meat intake were more likely to be younger (*β* = −1.65 ± 0.26; *P* =3.5 × 10^−10^), male (χ^2^ = 202; df = 4; *P* < 2.0 × 10^−16^) and a smoker (χ^2^ = 125; df = 8; *P* < 2.0 × 10^−16^; Table 1), as well as have a higher income (χ^2^ = 42; df = 8; *P* < 2.0 × 10^−16^). Higher red meat intake was also associated with a higher BMI (*β* = 0.53 ±0.05; *P* < 2.0 × 10^−16^; Table 1) and higher levels of physical activity (*β* = 246.60 ± 52.60; *P* < 2.0 × 10^−16^; Table 1).

### Associations of processed and unprocessed red meat intake with markers of inflammation

After a Bonferroni correction (0.05/2 red meat intakes [processed and unprocessed] × 6 markers of inflammation = 0.004), unprocessed red meat take was significantly associated with C-reactive protein (CRP), with each daily serving of unprocessed red meat associated with 0.73 mg/dL higher CRP (*β* = 0.08 ±0.02; *P* = 7.6 × 10^−5^; Table 2). When adjusted for demographic and lifestyle covariates, unprocessed red meat intake explained 0.39% of the variance in CRP. Meanwhile, processed red meat intake was not associated with markers of inflammation (all *P* > 0.004; Table 2).

**TABLE 2:** Standardized parameter estimates from linear regression models examining the associations between 6 markers of inflammation with unprocessed and processed red meat intake

	Unprocessed red meat			Processed red meat		
	*β*	SE	P	*β*	SE	P
C-Reactive protein	0.08^1^	0.02^1^	7.6 × 10^-5 1^	0.05	0.02	0.01
IL-2	0.07	0.03	0.02	0.04	0.03	0.22
IL-6	0.05	0.02	0.01	0.05	0.02	0.01
Fibrinogen	0.14	0.02	0.03	0.02	0.02	0.24
Homocysteine	-0.002	0.02	0.90	0.03	0.02	0.13
TNF-α	0.03	0.03	0.36	0.02	0.03	0.57

^1^Significant results (*P* < 0.004, Bonferroni corrected).

All models control for age, sex, race/ethnicity, data collection site, smoking status, highest education level, income level, daily energy intake and physical activity level

### MWASs of processed and unprocessed red meat intake

**FIGURE 2:**
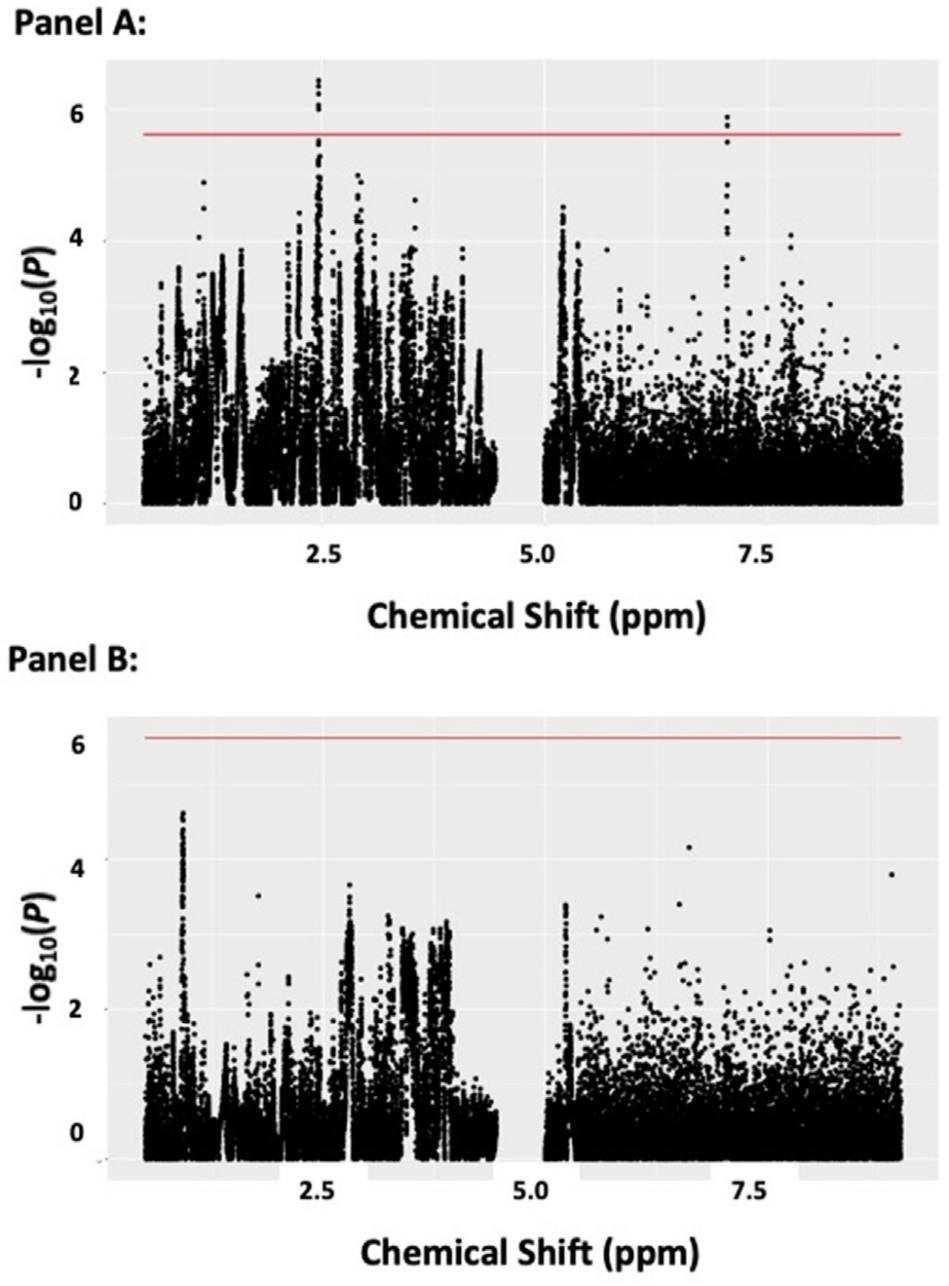
Manhattan-style plots for metabolome-wide associations studies with processed and unprocessed red meat intake. Panel A: Associations between spectral features and unprocessed red meat intake. Panel B: Associations between spectral features and processed red meat intake. *Note*: Models control for age, sex, race/ethnicity, and data collection site. Solid lines indicate metabolome-wide significance level (*P* < 2.4 × 10^– 6^ for unprocessed red meat intake and *P* < 2.1 × 10^– 6^ for processed red meat intake). *Abbreviations*: ppm: chemical shift in parts per million.

After permutation analyses (*N* = 10,000 permutations), significance was set at *P* < 2.4 × 10^−6^ for the MWAS of unprocessed meat intake and *P* < 2.1 × 10^−6^ for the MWAS of processed red meat intake. Seven spectral features were significantly associated with unprocessed red meat intake at metabolome-wide significance levels (*P* < 2.4 × 10^−6^; Figure 2, Supplementary Table 3). When additionally adjusted for smoking status, highest education level, income level, daily energy intake, and physical activity levels, 6 of these remained significant, 5 of which were annotated as glutamine or proline betaine (*P* = 2.2 × 10^−7^ to 1.7 × 10^−6^; Table 3), and one annotated as histidine (*P* = 2.3 × 10^−6^; Table 3).

**TABLE 3:** Standardized parameter estimates for significant associations from the metabolome-wide association study with either processed or unprocessed red meat intake

Chemical shift (ppm)	Annotation(s)	Unprocessed red meat			Processed red meat		
		*β*	SE	P	*β*	SE	P
2.463823342	glutamine/proline betaine	−0.04	0.02	0.02	−0.11^1^	0.02^1^	2.2 × 10^−7 1^
2.463150477		−0.02	0.02	0.20	−0.10^1^	0.02^1^	3.8 × 10^−7 1^
2.46348691		−0.03	0.02	0.09	−0.10^1^	0.02^1^	9.4 × 10^−7 1^
2.462814045		−0.02	0.02	0.30	−0.10^1^	0.02^1^	1.7 × 10^−6 1^
2.464159775		−0.05	0.02	0.004	−0.10^1^	0.02^1^	3.2 × 10^−7 1^
7.052427453	histidine	−0.04	0.02	0.06	−0.10^1^	0.02^1^	2.3 × 10^−6 1^
7.052763885		−0.01	.02	0.44	−0.08	0.02	0.0001

^1^Significant results (*P* < 2.4 × 10^−6^ for unprocessed red meat intake and 2.1 × 10^−6^ for processed red meat intake after permutation-based correction to maintain family-wide error rate at 5%)

Abbreviations: ppm: parts per million

All models control for age, sex, race/ethnicity, data collection site, smoking status, income level, education levels, total energy intake, and physical activity level

### Associations of sentinel spectral features with markers of inflammation

There were strong correlations among the metabolites annotated as glutamine/proline betaine (all *r* = 0.88–0.97; Supplementary Table 4) and annotated among the 2 metabolites as histidine (*r* = 0.98; Supplementary Table 4), but not between the metabolites annotated as glutamine/proline betaine and histidine (*r* = 0.35–0.56; Supplementary Table 4). As such, the sentinel spectral feature for each group of correlated features (i.e., for each annotation) was taken forward for analyses (ppm: 2.463823342 for glutamine/proline betaine and ppm: 7.052427453 for histidine).

When examining the associations of spectral features with markers of inflammation, the inclusion of unprocessed red meat intake as a fixed effect covariate did not notably impact results; therefore, parameter estimates from the first set of models which did not include unprocessed red meat intake are presented in the supplementary material (Supplementary Table 5), and estimates from the second set of models which included unprocessed red meat intake included in the main text (Table 4).

**TABLE 4:** Standardized parameter estimates from linear regression models examining the associations between 6 markers of inflammation with spectral features when controlling for unprocessed red meat

	Glutamine/proline betaine ppm: 2.463823342			Histidine ppm: 7.052427453			Unprocessed red meat		
	*β*	SE	*P*	*β*^1^	SE^1^	*P*^1^	*β*	SE	*P*
C-Reactive Protein	−0.15^1^	0.02^1^	<2.0 × 10^−16 1^	−0.16	0.02	<2.0 × 10^−16^	0.04	0.02	0.01
IL-2	0.03	0.03	0.32	−0.12	0.03	0.0001	0.07	0.03	0.02
IL-6	−0.07^1^	0.02^1^	0.0002^1^	−0.14	0.02	2.7 × 10^−14^	0.03	0.02	0.12
Fibrinogen	−0.03	0.02	0.07	−0.22	0.02	<2.0 × 10^−16^	0.02	0.02	0.25
Homocysteine	0.004	0.02	0.79	−0.07	0.02	2.0 × 10^−5^	−0.01	0.02	0.68
TNF-α	−0.04	0.03	0.17	−0.13	0.03	1.2 × 10^−5^	0.02	0.03	0.55

^1^Significant results (P<.004, Bonferroni corrected).

*Abbreviations*: ppm: chemical shift in parts per million

All models control for age, sex, race/ethnicity, data collection site, smoking status, education level, income level, total energy intake, and physical activity level

When the significance threshold was set using a Bonferroni correction for multiple testing (.05/2 features × 6 markers of inflammation =.004), the sentinel feature annotated as glutamine/proline betaine (ppm: 2.463823342) was inversely associated with CRP and IL-6, and the feature annotated as histidine was inversely associated with all markers of inflammation (Table 4). In these models, glutamine/proline betaine explained 5.5% of the variance in CRP (*β* = −0.15 ±0.02; *P* < 2.0 × 10^−16^; Table 4); and 1.6% of the variance in IL-6 (*β* = −0.07 ± 0.02; *P* = 0.0002; Table 4). Histidine explained 5.2% of the variance in CRP (*β* = −0.16 ± 0.02; *P* < 2.0 × 10^−16^; Table 4); 1.0% of the variance in IL-2 (*β* = −0.12 ± 0.03; *P*=0.0001; Table 4); 2.7% of the variance in IL-6 (*β* = −0.14 ± 0.02; *P* = 2.7 × 10^−14^; Table 4); 5.0% of the variance in fibrinogen (*β* = −0.22 ± 0.02; *P* < 2.0 × 10^−16^; Table 4); 0.5% of the variance in homocysteine (*β* = −0.07 ± 0.02; *P* = 2.0 × 10^−15^; Table 4); and 2.06% of the variance in TNF-α (*β* = −0.13 ± 0.03; *P* = 1.2 × 10^−5^; Table 4). Together, the 2 amino acids explained 10.3% of the variance in CRP, 1.0% in IL-2, 4.2% in IL-6, 5.0% in fibrinogen, 0.5% in homocysteine, and 2.1% in TNF-α.

### Post hoc exploration of dietary associations with sentinel spectral features

Given the strong associations between spectral features and markers of intake relative to those between unprocessed red meat intake and either a spectral feature or any marker of inflammation, we ran sensitivity analyses to examine whether other food groups were associated with the sentinel spectral features. After a Bonferroni correction (0.05/2 spectral features × 45 food groups = 0.001), no other food groups were associated with either feature (all *P* > 0.001; Supplementary Table 6).

### The role of BMI

BMI was strongly associated with both forms of red meat intake, with sentinel spectral features annotated as glutamine/proline betaine and histidine, and with markers of inflammation (Supplementary Table 7).

The examination of the role BMI plays in diet-inflammation, diet metabolite, and metabolite-inflammation relationships was seen as an exploration of our prior models; thus, the significance threshold was set via a Bonferroni correction for the number of models analyzed (0.05/5 models [Table 5] = 0.001).

**TABLE 5:** Standardized estimates from mediation models examining the effect of BMI on observed relationships between unprocessed red meat intake, sentinel spectral features, and markers of inflammation

	Effect of x on y when not controlling for BMI				Remaining effect of x on y when controlling for BMI				Proportion of effect of x on y, which is due to BMI	
	*β*^1^	SE^1^	*P*^1^	*R*^*2*1^	*β*	SE	*P*	*R*^*2*^	%^1^	*P*^1^
*Unprocessed red meat intake (x) and markers of inflammation (y)*										
*y*: CRP	.08	.02	7.6 × 10^−5^	0.39%	0.12	0.05	0.02	0.13%	43%	<2.0 × 10^−16^
*Unprocessed red meat intake (x) and sentinel spectral features (y)*										
*y*: Glutamine/proline betaine	−0.10	.02	3.2 × 10^−7^	0.69%	−0.09^1^	0.02^1^	2.0 × 10^−5 1^	0.47%^1^	17%	<2.0 × 10^−16^
*y*: Histidine	−0.08	0.02	4.8 × 10^−6^	0.44%	−0.06	0.02	0.002	0.26%^1^	24%	<2.0 × 10^−16^
*Glutamine/proline betaine (x) and markers of inflammation (y)*										
*y*: CRP	−0.15	0.02	<2.0 × 10^−16^	5.51%	−0.11^1^	0.01^1^	3.3 × 10−10^1^	2.35%^1^	25%	<2.0 × 10^−16^
*y*: IL-6	−0.07	0.02	.0002	1.56%	−0.25	0.08	0.002	0.29%^1^	41%	<2.0 × 10^−16^
*Histidine (x) and markers of inflammation (y)*										
*y*: CRP	−0.16	0.02	<2.0 × 10^−16^	5.18%	−0.14^1^	0.02^1^	<2.0 × 10^−16 1^	2.99%^1^	22%	<2.0 × 10^−16^
*y*: IL-2	−0.12	0.03	0.0001	0.96%	−0.68^1^	0.02^1^	0.0004^1^	0.71%^1^	18%	0.0004
*y*: IL-6	−0.14	0.02	2.7 × 10^−14^	2.67%	−0.92^1^	0.01^1^	<2.0 × 10^−16 1^	1.09%^1^	31%	<2.0 × 10^−16^
*y*: Fibrinogen	−0.22	0.02	<2.0 × 10^−16^	5.03%	−1.50^1^	0.11^1^	<2.0 × 10^−16 1^	3.40%^1^	13%	<2.0 × 10^−16^
*y*: Homocysteine	−0.07	0.02	2.0 × 10^−5^	0.46%	−0.48^1^	0.11^1^	2.5 × 10^−5 1^	0.28%^1^	17%	0.03
*y*: TNF-α	−0.13	0.03	1.2 × 10^−^5	2.06%	−0.74^1^	0.19^1^	8.5 × 10^−5 1^	1.21%^1^	17%	<2.0 × 10^−16^

^1^Significant results (*P* < 0.001, Bonferroni corrected).

All models control for age, sex, race/ethnicity, data collection site, smoking status, income level, daily energy intake and physical activity level

Columns 6 to 9 (subheading: “remaining effect of x on y when controlling for BMI”) of Table 5 quantify the strength of various associations when additionally controlling for BMI. When controlling for BMI, unprocessed red meat was no longer significantly associated with CRP (*β* = 0.12 ± 0.05; *P* = 0.02; Table 5), with ∼43% of the unadjusted association (unadjusted for BMI) between unprocessed red meat intake and CRP attributable to BMI (*P* < 2.0 × 10^-16^; Table 5). Columns 10 and 11 of Table 5 (subheading: “proportion of the effect of x on y which is due to BMI”) quantify the proportion of the various associations that can be attributable to BMI. In similar BMI-adjusted models, the association between unprocessed red meat intake and glutamine/proline betaine was attenuated by ∼17% (*P* < 2.0 × 10^−16^; Table 5) compared with models that did not adjust for BMI and remained significant (*β* = −0.09 ± 0.02; *P* = 2.0 × 10^−5^; Table 5), with unprocessed red meat intake explaining 0.47% of the variance in glutamine/proline betaine. However, when adjusted for BMI, the remaining relationship between unprocessed red meat intake and histidine (∼24% of the unadjusted association; *P* < 2.0 × 10^−16^; Table 5) was no longer significant (*β* = −0.06 ± 0.02; *P* = 0.002; Table 5).

Although the relationships between sentinel spectral features and markers of inflammation were all significantly attenuated when controlling for BMI (which accounted for 13%–31% of the unadjusted associations, *P* = 0.03 to *P* = 2.0 × 10^−16^; Table 5), almost all of the feature-inflammation associations remained significant (*P* = 0.0002 to *P* = 2.0 × 10^−16^; Table 5), with the exception of the association between glutamine/proline betaine at IL-6, which approached significance (*β* = −0.25 ± 0.08; *P* = 0.002; Table 5). Column 9 of Table 5 quantifies the variance in outcomes attributable to dietary and/or spectral features when controlling for BMI. In models that controlled for BMI, glutamine/proline betaine explained 2.3% of the variance in CRP (*β* = −0.11 ± 0.01; *P* < 3.3 × 10^−10^; Table 5) and 0.3% of the variance in IL-6 (*β* = −0.25 ± 0.08; *P* = 0.002; Table 5). In similar models (i.e., models that also controlled for BMI, as well as demographic and behavioral factors), histidine explained 3.0% of the variance in CRP (*β* = −0.14 ± 0.02,;*P* = 2.0 × 10^−16^; Table 5); 0.7% of the variance in IL-2 (*β* = −0.68 ± 0.02; *P* = 0.0004, Table 5); 1.1% of the variance in IL-6 (*β* = −0.92 ± 0.01; *P* < 2.0 × 10^−16^; Table 5); 3.4% of the variance in fibrinogen (*β* = −1.50 ± 0.11; *P* < 2.0 × 10^−16^; Table 5); 0.3% of the variance in homocysteine (*β* = −0.48 ± 0.11; *P* < 2.5 × 10^−5^; Table 5); and 1.2% of the variance in TNF-α (*β* = −0.74 ± 0.19; *P* < 8.5 × 10^−5^; Table 5).

## Discussion

Using a large multiethnic sample of older adults, the current analyses sought to use metabolomic data to explore the relationships between red meat intake and inflammation. The pattern of results differed according to whether BMI was included as a covariate or not. When our models did not adjust for BMI, we found that intake of unprocessed red meat was positively associated with CRP and inversely associated with glutamine and histidine, 2 amino acids with established anti-inflammatory properties, which were inversely associated with various markers of inflammation in our population. When our models controlled for BMI, we did not observe a significant association between unprocessed red meat and CRP, nor did we observe the inverse association between unprocessed red meat and histidine. In these BMI-adjusted analyses, the associations that remained significant were the inverse association between unprocessed red meat and glutamine and the inverse association between glutamine and CRP. The lack of association between unprocessed red meat and CRP when controlling for BMI, plus the observation that unprocessed red meat accounted for <1% of the variance in glutamine, indicated that these analyses did not provide support for the role of red meat intake in inflammation.

Our finding that processed red meat was not associated with inflammation in analyses was unexpected. However, the association between unprocessed red meat and CRP, a marker of inflammation that is elevated in CVD [[34], [35], [36], [37]], has been previously reported [see 12–16]. In our analyses, the association between unprocessed red meat and CRP was small (accounting for 0.4% of the variance in CRP concentrations) and was attenuated to nonsignificance when controlling for BMI, indicating that our analyses did not support a relationship between red meat and inflammation over and above confounding by BMI. A meta-analysis of randomized controlled trials into the effects of red meat intake on inflammation also reported a nonsignificant relationship between markers of inflammation and both processed and unprocessed red meat intake [38]. In this latter analysis, the presence/absence of red meat intake, or the level of participants’ red meat intake, was randomly assigned within the constituent studies, with the goal of reducing residual confounding, such as differences in BMI. Thus, the difference between these results compared with those from observational studies supports our conclusion that population associations between unprocessed red meat and inflammation are confounded by BMI.

The small magnitude of association in the association of unprocessed red meat with CRP could be attributable to heterogeneity in the population-level associations. As metabolites in the plasma capture the effects of dietary intake after it has been digested, processed, and absorbed if there are individual differences in the metabolism of unprocessed red meat, the incorporation of metabolomic data would offer promise for detecting relationships between red meat intake and inflammation. Thus, we conducted a metabolome-wide analysis of unprocessed red meat intake, which revealed inverse associations between unprocessed red meat intake and 2 sets of spectral features. One set of spectral features was annotated as histidine, an α-amino acid found in red meat [39,40], as well as poultry [41,42]. Histidine increases acutely in human urine after ingestion of fish, poultry, beef, and pork (i.e., peaking within ∼7 h and returning to baseline within 40 h [43], supporting our confidence this molecule was correctly annotated. However, the inverse association of histidine with unprocessed red meat intake was attenuated to nonsignificance when adjusting for BMI, suggesting it is not a biomarker of unprocessed red meat over and above confounding by BMI.

When additionally controlling for BMI, the inverse association between unprocessed red meat and a second set of spectral features remained strongly significant. However, the annotation associated with these features was less clear, as it included both glutamine and proline betaine. Glutamine is largely derived from branched-chain amino acids [44,45], of which red meat is a major dietary source. Meanwhile, proline betaine is not linked to meat intake in the literature but is considered a strong marker of citrus consumption [[46], [47], [48], [49]]. As this set of spectral features was not associated with citrus consumption in our post hoc analyses, we conclude that these features are most likely to represent glutamine, which, in our data, served as a biomarker of red meat intake.

In our analyses, glutamine was inversely associated with CRP and IL-6, which is supported by previous data that have shown that glutamine has powerful anti-inflammatory effects at the local and systemic levels [[50], [51], [52], [53], [54], [55]]. The inverse relationship between glutamine and CRP was much larger in magnitude than that between glutamine and IL-2 and remained significant when controlling for BMI. Glutamine accounted for ∼3% to 5% of the variance in CRP, and unprocessed red meat was only associated with ∼0.1% to 0.5% of the variance in CRP. We have previously observed similar differences in the magnitude of association between health indicators and dietary intake compared with the magnitude of association between the same health indicator and metabolomic correlates of food intake [56,57]. Why the magnitude of relationships with health differs for self-reported food intake compared with metabolites associated with self-reported food intake is beyond the scope of the current investigation, but the stronger metabolite-CRP relationships may reflect measurement issues [58], as metabolomic data are also not subject to the widely recognized reporting errors of self-reported dietary data [[59], [60], [61], [62]]. A second explanation is that multiple other environmental exposures or genetic vulnerabilities may also contribute to levels of these amino acids to explain the relatively stronger associations with CRP. For this reason, we probed our data for other dietary associations with spectral features, but none reached significance. A third possibility is that metabolites reflect the physiologic effects of dietary intake at the individual level (i.e., after accounting for differences in food metabolism), and this could be a major driver of stronger associations. Ultimately, the reasons, which likely reflect a combination of these possibilities, are beyond the scope of the current investigation but serve to highlight the value of metabolomic information when ascertaining diet-health associations.

The pattern of results differed according to whether the models adjusted for BMI or not. Comparisons between these 2 sets of models indicated that BMI played a role in up to one-fourth of the observed relationships between unprocessed red meat intake, glutamine, and CRP. The inflammatory effects of white adipose tissue are well established [63,64], and excess adipose tissue is considered a direct cause of the lower levels of glutamine and histidine seen in adults with obesity [[65], [66], [67], [68]]. However, supplementing glutamine or histidine reduces adiposity in animal studies and one small human study [[69], [70], [71], [72], [73]], making it difficult to judge whether BMI is likely to be a mediator or a confounder – or even a moderator given that obesity directly affects the expression of glutamine synthase genes [74] and that histidine shows greater associations with lower inflammation in adults with obesity compared to those with a normal weight [66].

Our study benefited from a multiethnic population and data on untargeted spectral features, the latter of which allowed for hypothesis-free discovery of molecules associated with unprocessed red meat consumption. However, this approach also necessitated a stringent correction for multiple testing, and our conclusions relate only to those spectral features where associations with unprocessed red meat met (or exceeded) this threshold. Future investigations that investigate other pathways by which red meat may influence inflammation are therefore warranted. Although our food-frequency questionnaire has shown some evidence of criterion and predictive validity [e.g., 77,78], the usual limitations of self-reported nutritional data apply, such as a tendency to under-report intake [[60], [61], [62], [63]]. We also did not distinguish between frequency of consumption and portion size when estimating average servings per week. In addition, we were unable to conclusively confirm molecule annotations, as evidenced by annotations for one set of metabolites indicating both glutamine or proline betaine, although the former seems more likely based on the known biological origins and functions of the 2 molecules. Our observational study, despite adjustments for potential confounders, also cannot rule out possible residual confounding, and its cross-sectional design precluded causal inferences. Finally, the markedly stronger associations between the metabolite data and markers of inflammation than those between the dietary intake data and markers of inflammation were unexpected and should be investigated in other studies for replication and to elucidate the underlying mechanisms.

In the context of these limitations, the results of the current study lend themselves to 2 preliminary conclusions. First, the lack of any associations between unprocessed red meat and markers of inflammation, considered alongside the differences in associations between CRP and unprocessed red meat when controlling for BMI compared with when not controlling for BMI, together provide additional evidence that red meat does not associate with inflammation over and above confounding by BMI at a population level. Second, the differences in the magnitude of association between CRP and unprocessed red meat compared with the magnitude of the inverse association between CRP and glutamine, a plasma correlate of red meat intake, suggests that unprocessed red meat is not a major driver of glutamine, and therefore in the glutamine-inflammation pathway; and/or that there are individual differences in the association of red meat with CRP, heterogeneity that is captured, in part, by individual differences in the extent that the metabolism of unprocessed red meat is associated with plasma levels of glutamine.

The current analyses did not provide evidence that intake of red meat is associated with markers of inflammation independently of BMI. Future investigations into this relationship may be enhanced by including metabolomic markers of red meat intake, as these may subsume aggregate etiologic effects and/or account for individuals’ metabolic responses to their red meat intake.

In summary, our preliminary conclusions from these data are that red meat is not associated with inflammation when controlling for BMI. However, glutamine is an anti-inflammatory metabolite that is associated with red meat intake when controlling for BMI; it also shows associations with lower CRP when controlling for BMI.

## Acknowledgments

We thank the other investigators, the staff, and the participants of the MESA study for their valuable contributions. A full list of participating MESA investigators and institutions can be found at http://www.mesa-nhlbi.org.

### Author contributions

The authors’ responsibilities were as follows – ACW: designed the research; MAA, MOG, IT, PG, TE, PE, RT, JIR, DH: conducted research, provided essential reagents or provided essential materials; ACW, MKS: analyzed data and performed statistical analysis; ACW, JIR, DH: wrote the paper; ACW: had primary responsibility for final content. All authors have read and approved the final manuscript.

### Conflict of interest

ACCW has received funding from Sabra Dipping Company, Hass Avocado Board, and Ionis Pharmaceuticals for studies unrelated to the current analyses. All other authors report no conflicts of interest.

### Funding

The study was supported by the Beef Checkoff to ACW. A.C.W'. was supported, in part, by the USDA/ARS (Cooperative Agreement 58-3092-5-001). M.O.G. was supported by the Eris M. Field Chair in Diabetes Research. J.I.R. was supported, in part, by the National Institutes of Health grants from the National Institute of Diabetes and Digestive and Kidney Disease (DK063491), from the National Center for Advancing Translational Sciences (UL1TR001881), the CHARGE Consortium, and the National Heart, Lung, and Blood Institute (NHLBI; R01HL105756).

The Multi-Ethnic Study of Atherosclerosis (MESA) projects are conducted and supported by the National Heart, Lung, and Blood Institute (NHLBI) in collaboration with MESA investigators. Support for MESA is provided by contracts 75N92020D00001, HHSN268201500003I, N01-HC-95159, 75N92020D00005, N01-HC-95160, 75N92020D00002, N01-HC-95161, 75N92020D00003, N01-HC-95162, 75N92020D00006, N01-HC-95163, 75N92020D00004, N01-HC-95164, 75N92020D00007, N01-HC-95165, N01-HC-95166, N01-HC-95167, N01-HC-95168, N01-HC-95169, UL1-TR-000040, UL1-TR-001079, UL1-TR-001420, UL1TR001881, DK063491, R01HL133932 and R01HL105756. Additional support for the metabolomics data was provided by the EU COMBI-BIO project (FP7, 305422.

The contents of this publication do not necessarily reflect the views or policies of the USDA, nor does mention of trade names, commercial products, or organizations imply endorsement from the US government.

### Data Availability

Data described in the manuscript, code book, and analytic code will be made available upon request pending approval by the MESA Genetic Publications and Presentations committee and a signed and executive materials transfer agreement.
