# Supplementary material for: Untargeted metabolomic analysis investigating links between unprocessed red meat intake and markers of inflammation
Source: Am J Clin Nutr. 2023 Sep 1;118(5):989–99. doi: 10.1016/j.ajcnut.2023.08.018 (PMC10797554; doi:10.1016/j.ajcnut.2023.08.018)
Supplement: Multimedia component 1 [file mmc1.pdf]

Untargeted Metabolomic Analysis Investigating Links Between Unprocessed Red Meat Intake and Markers of Inflammation

Alexis C. Wood et al

Supplementary Information

Supplementary Table 1: Relationship of Foods from the Food Frequency Questionnaire to 49 Food groups in the Multi Ethnic Study of Atherosclerosis

Fruit
peaches, apricots, nectarines, plums
cantaloupe, mango, papaya
strawberries, blueberries, other berries
apples, applesauce, pears
bananas, plantains
oranges, grapefruit, tangerines, kiwi
dried fruits including raisins, prunes, figs, apricots
any other fruits (pineapples, persimmon, grapes, other melon, canned peaches, fruit cocktail, etc.)
Fruit Juices
orange juice, grapefruit juice
any other fruit juice (apple, grape, punch, kool-aid, guava juice, etc.)
Eggs and Egg Products
eggs, omelets, huevos rancheros
Dark/Whole Grains
dark, whole grain breads or rolls (hamburger buns, bagels, pita, English muffins, etc.)
bran muffins
brown or wild rice
oatmeal
cold cereal- high fiber
Refined Grains
white bread or rolls (hamburger buns, bagels, pita, English muffins, etc.)
white, Mexican or sticky rice
flour or corn tortilla on the side
other hot cereal (grits, cream of wheat, mush, congee)
cold cereal- low fiber
oriental noodles with meat (saimen, ramen, wonton mein)
fish stew or seafood gumbo, paella
pasta with tomato sauce (no meat), including spaghetti and lasagna
pasta with tomato sauce and meat, poultry or seafood, including spaghetti and lasagna
pasta with cream sauce or cheese (no meat), including macaroni and cheese quiche, pesto
pasta with cream sauce, cheese and meat, poultry or seafood, including tuna noodle casserole
arroz con pollo
burritos, quesadillas or fajitas with meat, poultry or seafood
enchilada, tamales, tacos or nachos with meat, poultry or seafood
enchiladas, tamales, tacos or nachos with no meat
burritos or quesadillas with no meat
meat, chicken or turkey stew, pot pie or empanada
Sweet Breads
biscuits, other muffins, croissants, corn bread, hush puppies
pancakes, waffles, French toast
Desserts

pies
pudding, custard, flan
white doughnuts, cookies, cakes, pastries, Pop Tarts, Chinese desserts, Mexican desserts
chocolate doughnuts, cookies, cakes, brownies or candy
dessert made with tofu

Salty Snacks

potato, corn or tortilla chips
crackers, pretzels, popcorn

Seeds, Nuts and Peanut Butter

almonds, walnuts, pecans, other nuts
sunflower, pinyon, other seeds
peanuts, peanut butter

Yogurt

plain yogurt (unflavored)
flavored yogurt

Low-Fat Dairy Desserts

frozen yogurt, low-fat ice cream, ice milk, sherbet
sweetened condensed milk

Ice Cream

regular ice cream

Cottage / Ricotta Cheese

cottage or ricotta cheese

Low-Fat Milk

2% milk and beverages made with 2% milk including café latte, café au lait
skim milk, 1% or buttermilk, or beverages made with these including café latte, café au lait
cold cereal- high fiber (when milk bev response = skim or 2%)
cold cereal- low fiber (when milk bev response = skim or 2%)

Coffee-Tea Creamers

cream, half-and-half or non-dairy creamer in coffee or tea
milk in coffee or tea (not including café latte, café au lait)

High-Fat Dairy

whole milk and beverages made with whole milk including café latte, café au lait
cheddar, American, Chihuahua, Swiss, cream cheese, cheese spreads, any other cheese
cold cereal- high fiber (when milk bev response = whole)
cold cereal- low fiber (when milk bev response = whole)
pasta with cream sauce or cheese (no meat), including macaroni and cheese quiche, pesto
pasta with cream sauce, cheese and meat, poultry or seafood, including tuna noodle casserole
burritos, quesadillas or fajitas with meat, poultry or seafood
enchiladas, tamales, tacos or nachos with meat, poultry or seafood
enchiladas, tamales, tacos or nachos with no meat
burritos or quesadillas with no meat

Cream-based Soups

cream soups including chowders, potato and cheese soups

Other Soups

other soups including vegetable beef, tomato, egg drop, chicken noodle

meat, chicken or turkey stew, pot pie or empanada

Legumes

pea, lentil, black bean, potajes soups
pinto, black, baked, butter or red beans, pork and beans, black-eyed peas
refried beans as a side dish
chile with meat and beans
enchiladas, tamales, tacos or nachos with no meat
burritos or quesadillas with no meat

Green Leafy Vegetables

tossed salad with spinach, romaine or dark greens, cooked spinach, turnip greens, collards
Cruciferous Vegetables
broccoli, cabbage, cauliflower, brussel sprouts, sauerkraut, kimchee
stir-fried vegetables (no meat)
stir-fried shrimp or fish with vegetables
stir-fried beef, pork or chicken with vegetables, including beef broccoli
stir-fried tofu or tempeh with vegetables

Dark Yellow Vegetables

stir-fried vegetables (no meat)
stir-fried shrimp or fish with vegetables
stir-fried beef, pork or chicken with vegetables, including beef broccoli
stir-fried tofu or tempeh with vegetables
red chile con carne with meat
carrots
winter squash, acorn squash
sweet potatoes, yams
meat, chicken or turkey stew, pot pie or empanada

Starchy Vegetables

boiled, baked, mashed or other potatoes, turnips
meat, chicken or turkey stew, pot pie or empanada

Other Vegetables

corn hominy
green beans, peas, snow peas
any other vegetables including summer squash, zucchini, asparagus, mixed vegetables
tossed salad with iceberg or light green lettuce
burritos, quesadillas or fajitas with meat, poultry or seafood
enchiladas, tamales, tacos or nachos with meat, poultry or seafood
enchiladas, tamales, tacos or nachos with no meat
burritos or quesadillas with no meat
meat, chicken or turkey stew, pot pie or empanada
stir-fried vegetables (no meat)
green chile con carne with meat
red chile con carne with meat
stir-fried tofu or tempeh with vegetables

Avocados / Guacamole

avocado, guacamole

Tomatoes

tomatoes (cooked or raw), tomato juice
salsa, pico de gallo
chile with meat and beans
picadillo, carne quisada, menudo
fish stew or seafood gumbo, paella
pasta with tomato sauce (no meat), including spaghetti and lasagna
pasta with tomato sauce and meat, poultry or seafood, including spaghetti and lasagna
red chile con carne with meat
green chile con carne with meat
burritos, quesadillas or fajitas with meat, poultry or seafood
enchilada, tamales, tacos or nachos with meat, poultry or seafood
enchiladas, tamales, tacos or nachos with no meat
burritos or quesadillas with no meat

Fried Potatoes

French fries, fried potatoes, hash browns

Soy Foods and Beverages

soy milk
miso soup or sauce with soybean paste
stir-fried tofu or tempeh with vegetables
dessert made with tofu

Pizza

pizza

Starchy Foods with Mayonnaise

pasta salad, macaroni salad, potato salad, cole slaw

Protein Foods with mayonnaise

chicken salad, tuna salad or egg salad

Processed Red Meat

ham, hot dogs, bologna, salami, other, lunch meats
liver including chicken livers, other organ meats
ham hocks, pigs' feet, chicarones
sausage, chorizo, scrapple, bacon

High-Fat Chinese Dishes

fried rice
chow mein
Chinese dumplings, spring roll, dim sum (not fried), Chinese bun with meat, sausage and vegetables

Unprocessed Red Meat

hamburger, cheeseburger, meat loaf, hash
beef, pork or lamb steaks, roasts, barbeque or ribs
picadillo, carne quisada, menudo
chile with meat and beans
oriental noodles with meat (saimen, ramen, wonton mein)
red chile con carne with meat
green chile con carne with meat
pasta with tomato sauce and meat, poultry or seafood, including spaghetti and lasagna

pasta with cream sauce, cheese and meat, poultry or seafood, including tuna noodle casserole
stir-fried beef, pork or chicken with vegetables, including beef broccoli
burritos, quesadillas or fajitas with meat, poultry or seafood
enchilada, tamales, tacos or nachos with meat, poultry or seafood
meat, chicken or turkey stew, pot pie or empanada

Poultry

roasted, broiled, baked or ground chicken or turkey
fried chicken
arroz con pollo
pasta with cream sauce, cheese and meat, poultry or seafood, including tuna noodle casserole
stir-fried beef, pork or chicken with vegetables, including beef broccoli
burritos, quesadillas or fajitas with meat, poultry or seafood
enchilada, tamales, tacos or nachos with meat, poultry or seafood
meat, chicken or turkey stew, pot pie or empanada

Fish

shrimp, lobster, crab, oysters, mussels (not fried)
tuna, salmon, sardines (including sashimi or sushi)
other broiled, steamed, baked or raw fish (trout, sole, halibut, poke, grouper)
fried fish or fish sandwich, fried shrimp, calamari
fish stew or seafood gumbo, paella
stir-fried shrimp or fish with vegetables
pasta with cream sauce, cheese and meat, poultry or seafood, including tuna noodle casserole

Sweet Extras

sugar or honey in coffee or tea (not including artificial sweeteners)
sugar, jelly, jam, molasses on bread or cereal
other candy including hard candy, licorice, other candy bars

Fats and Oils

margarine or mayonnaise on bread or rolls
butter on bread or rolls
butter, margarine or oil on vegetables, rice or potatoes
refried beans as a side dish
fried chicken
fried fish or fish sandwich, fried shrimp, calamari

Gravy

gravies made with meat or poultry drippings

Meal Replacement Drinks

instant breakfast, Ensure, Slimfast

Hot Chocolate

hot chocolate

Coffee

coffee (regular or decaffeinated) not including latte, café au lait

Tea

herbal tea
black or green tea

Non-Diet Soft Drinks

regular soft drinks, soda, sweetened mineral water (not diet), non-alcoholic beer
Diet Soft Drinks
diet soft drinks, unsweetened mineral water
Beer
beer
Other Alcoholic Beverages
wine
liquor or mixed drinks

Supplementary Table 2. Baseline Characteristics of Participants in the Multi-Ethnic Study of Atherosclerosis overall, and separately, those without metabolomics data

	Overall<br>(N=6,224)	With metabolomics data<br>(N= 3,638)
Demographics		
Age, y ***	63.0 (10.3)	61.3 (10.1)
Gender, N (%) female ***	1144 (50.1%)	1843 (50.7%)
Race / Ethnicity ***		
White, N (%)	1025 (39.6%)	1142 (39.6%)
Chinese, N (%)	266 (10.3%)	525 (14.4%)
African-American , N (%)	778 (30.1%)	828 (22.8%)
Hispanic, N (%)	517 (20.0%)	843 (23.2%)
Household income category**		
<$25,000, N (%)	712 (28.8%)	1154 (32.7%)
$25,000- $49,999 , N (%)	737 (29.8%)	994 (28.2%)
> $50,000 , N (%)	1026 (41.5%)	1380 (39.1%)
Health Behaviors		
BMI, kg/m^2 **	28.0 (5.3)	28.5 (5.5)
Physical Activity, MET mins / week *	5554.0 (5667.3)	5928.5 (6161.5)
Smoking status		
Never Smoker, N (%)	1316 (50.9%)	1861 (51.2%)
Former Smoker, N (%)	925 (35.8%)	1348 (37.1%)
Current smoker, N (%)	423 (13.3%)	344 (11.7%)
Dietary Intake		
Processed meat, svg / day	0.17 (0.26)	0.18 (0.30)
Unprocessed red meat, svg / day	0.38 (0.39)	0.37 (0.37)
Markers of Immune functioning		
CRP, mg/dL *	3.6 (5.6)	3.9 (6.2)
IL-2, pg/ml **	1023.2 (443.5)	971.8 (432.1)
IL-6 , pg/ml	1.5 (1.2)	1.5 (1.2)
Fibrinogen antigen (mg/dL) *	343.7 (72.2)	348.4 (74.4)
Total Homocysteine, umol/L ***	9.4 (4.1)	9.1 (3.3)
TNF-α, pg/ml ***	1420.6 (439.9)	1337.0 (423.9)

Note: *P<.05, **P<.001, ***P<.001 for tests of differences between those with and those without metabolomics data.

Abbreviations: BMI: Body mass index, CRP: C-Reactive Protein, IL-2: Interleukin-2, IL-6: Interleukin-6, MET min: Metabolic equivalent minutes, TNF-α: Tumor Necrosis Factor-α Soluble Receptors

Supplementary Table 3: Parameter Estimates From Metabolome Wide Association Studies With Proces

	Unprocessed Red Meat Intake			Processed Red Meat Intake		
Chemical shift (ppm)	*b*	SE	P	*b*	SE	P
2.463150477	**-0.043**	**0.009**	**4.44E-07**	-0.008	0.013	5.10E-01
2.462814045	**-0.042**	**0.009**	**8.66E-07**	-0.007	0.013	5.61E-01
2.46348691	**-0.042**	**0.008**	**5.80E-07**	-0.013	0.013	3.08E-01
2.463823342	**-0.042**	**0.008**	**3.64E-07**	-0.016	0.012	1.83E-01
7.052427	**-0.030**	**0.006**	**1.31E-06**	-0.020	0.009	3.20E-02
2.464159775	**-0.039**	**0.008**	**9.98E-07**	-0.022	0.012	6.82E-02
2.460795449	-0.038	0.008	6.59E-06	-0.008	0.012	4.99E-01
2.462477612	-0.040	0.009	3.43E-06	-0.007	0.013	5.79E-01
2.461131882	-0.038	0.008	6.20E-06	-0.009	0.013	4.89E-01
7.052764	**-0.032**	**0.007**	**1.76E-06**	-0.012	0.010	2.40E-01
2.460459016	-0.036	0.008	1.78E-05	-0.008	0.012	5.44E-01
2.461804747	-0.038	0.008	5.84E-06	-0.003	0.013	7.94E-01
2.462141179	-0.039	0.009	7.07E-06	-0.005	0.013	7.25E-01
7.0531	-0.036	0.008	3.15E-06	-0.015	0.011	1.84E-01
2.464496207	-0.036	0.008	3.03E-06	-0.024	0.012	4.25E-02
2.461468314	-0.037	0.008	1.07E-05	-0.009	0.013	4.90E-01
2.449693173	-0.036	0.009	2.03E-05	-0.021	0.013	9.43E-02
2.938866	-0.023	0.005	1.28E-05	-0.011	0.008	1.57E-01
2.458103988	-0.038	0.009	1.94E-05	-0.012	0.013	3.59E-01
2.458440421	-0.038	0.009	2.02E-05	-0.012	0.013	3.63E-01
2.448347443	-0.035	0.008	2.23E-05	-0.002	0.012	8.72E-01
7.053437	-0.038	0.009	1.41E-05	-0.020	0.013	1.27E-01
2.460122584	-0.034	0.008	6.89E-05	-0.004	0.013	7.45E-01
2.452384634	-0.035	0.008	2.16E-05	-0.020	0.012	1.01E-01
2.890756	-0.025	0.006	4.27E-05	-0.001	0.009	9.15E-01
2.448011011	-0.032	0.008	4.89E-05	-0.004	0.012	7.32E-01
2.939203	-0.022	0.005	3.43E-05	-0.009	0.008	2.39E-01
2.448683876	-0.036	0.009	4.63E-05	-0.001	0.013	9.10E-01
2.457767556	-0.037	0.009	2.92E-05	-0.013	0.013	3.32E-01
2.451375336	-0.035	0.008	2.08E-05	-0.017	0.012	1.55E-01
2.904214	-0.029	0.007	1.02E-05	-0.022	0.010	2.90E-02
2.451711769	-0.035	0.008	1.97E-05	-0.018	0.012	1.45E-01
2.891093	-0.025	0.006	5.06E-05	-0.006	0.009	4.85E-01
2.459449719	-0.034	0.009	9.36E-05	-0.002	0.013	8.61E-01
2.452721067	-0.034	0.008	4.00E-05	-0.021	0.012	9.35E-02
2.458776853	-0.036	0.009	5.85E-05	-0.008	0.013	5.64E-01
2.452048202	-0.034	0.008	2.84E-05	-0.019	0.012	1.30E-01
2.46483264	-0.033	0.008	1.79E-05	-0.024	0.012	3.43E-02
2.479635674	-0.035	0.008	5.17E-06	-0.002	0.011	8.40E-01

7.047717	-0.027	0.006	3.59E-05	-0.018	0.010	5.55E-02
2.467524101	-0.034	0.008	1.41E-05	-0.021	0.012	7.43E-02
3.546127	0.044	0.010	2.40E-05	0.039	0.016	1.33E-02
2.627329582	-0.030	0.008	7.45E-05	-0.018	0.011	1.20E-01
5.20945	0.034	0.008	3.07E-05	0.007	0.012	5.41E-01
2.480308539	-0.034	0.008	1.17E-05	0.000	0.012	9.84E-01
2.480981404	-0.036	0.008	1.57E-05	-0.002	0.012	8.81E-01
2.90455	-0.028	0.007	2.04E-05	-0.020	0.010	4.19E-02
5.209113	0.034	0.008	3.10E-05	0.005	0.012	6.55E-01
2.451038904	-0.034	0.008	5.37E-05	-0.018	0.013	1.57E-01
2.453057499	-0.033	0.008	8.41E-05	-0.020	0.012	9.90E-02
7.052091	-0.025	0.006	6.43E-05	-0.024	0.009	1.10E-02
2.459786151	-0.032	0.009	1.86E-04	-0.001	0.013	9.63E-01
2.479972107	-0.033	0.008	1.08E-05	-0.002	0.011	8.83E-01
2.903877	-0.028	0.007	2.26E-05	-0.021	0.010	3.19E-02
7.053773	-0.040	0.010	7.73E-05	-0.029	0.015	5.55E-02
5.210123	0.035	0.008	4.71E-05	0.004	0.013	7.50E-01
2.455412528	-0.033	0.008	9.38E-05	-0.024	0.012	5.62E-02
2.459113286	-0.034	0.009	1.22E-04	-0.005	0.013	7.12E-01
2.93853	-0.021	0.005	5.16E-05	-0.007	0.008	3.43E-01
5.209786	0.035	0.009	4.25E-05	0.007	0.013	6.09E-01
2.455076095	-0.033	0.008	8.51E-05	-0.026	0.013	4.12E-02
2.467860533	-0.035	0.008	1.48E-05	-0.014	0.012	2.50E-01
2.45574896	-0.031	0.008	1.61E-04	-0.023	0.012	6.43E-02
5.204067	0.053	0.013	4.08E-05	0.012	0.019	5.17E-01
3.089588	-0.024	0.006	8.39E-05	-0.017	0.009	5.83E-02
2.481317837	-0.037	0.009	2.92E-05	-0.004	0.013	7.75E-01
7.047381	-0.030	0.007	2.08E-05	-0.017	0.010	1.08E-01
3.506764	0.036	0.009	1.27E-04	0.047	0.014	9.93E-04
2.89042	-0.023	0.006	1.89E-04	-0.006	0.009	5.27E-01
2.454739662	-0.033	0.008	9.72E-05	-0.027	0.013	3.27E-02
5.214496	0.022	0.006	1.51E-04	-0.004	0.009	6.39E-01
2.457431123	-0.034	0.009	1.08E-04	-0.016	0.013	2.11E-01
3.491625	0.037	0.010	1.41E-04	0.047	0.014	1.06E-03
1.121120871	0.025	0.006	8.86E-05	0.011	0.009	2.26E-01
2.467187668	-0.032	0.008	6.58E-05	-0.022	0.012	6.70E-02
5.208777	0.034	0.008	5.28E-05	0.008	0.013	5.25E-01
5.702323	0.031	0.008	1.38E-04	0.012	0.012	2.98E-01
2.442628089	-0.031	0.008	1.46E-04	-0.024	0.012	5.19E-02
3.491961	0.036	0.010	1.81E-04	0.047	0.015	1.38E-03
3.476149	0.036	0.010	1.61E-04	0.047	0.014	1.18E-03
2.442964522	-0.031	0.008	2.34E-04	-0.025	0.012	4.55E-02
1.174950086	0.018	0.004	1.30E-05	0.008	0.006	2.12E-01

3.420301	0.036	0.010	1.71E-04	0.045	0.014	1.72E-03
2.447674578	-0.029	0.008	2.16E-04	-0.008	0.012	5.04E-01
5.397516	0.034	0.009	2.31E-04	-0.010	0.014	4.55E-01
2.937184	-0.021	0.005	7.05E-05	-0.013	0.008	1.13E-01
2.456085393	-0.031	0.008	2.55E-04	-0.023	0.013	7.19E-02
2.442291656	-0.031	0.008	1.13E-04	-0.023	0.012	5.63E-02
3.506428	0.036	0.009	1.50E-04	0.045	0.014	1.37E-03
2.449020308	-0.033	0.009	3.05E-04	-0.006	0.014	6.70E-01
2.932138	-0.023	0.006	1.00E-04	-0.024	0.009	7.24E-03
3.476485	0.036	0.010	2.37E-04	0.047	0.015	1.31E-03
5.20373	0.051	0.013	5.37E-05	0.011	0.019	5.57E-01
7.768692	-0.051	0.013	8.24E-05	-0.037	0.019	5.18E-02
3.089924	-0.024	0.006	1.09E-04	-0.016	0.009	7.39E-02
2.480644972	-0.033	0.008	3.32E-05	0.000	0.012	9.84E-01
3.545791	0.042	0.011	6.39E-05	0.038	0.016	1.64E-02
2.89513	-0.022	0.006	2.68E-04	-0.015	0.009	1.05E-01
2.45709469	-0.032	0.008	1.96E-04	-0.020	0.013	1.16E-01
7.680547	0.029	0.008	4.52E-04	0.006	0.012	6.16E-01
2.906232	-0.030	0.007	4.18E-05	-0.022	0.011	4.02E-02
2.45440323	-0.033	0.009	1.50E-04	-0.027	0.013	3.65E-02
2.938193	-0.020	0.005	1.92E-04	-0.005	0.008	5.04E-01
5.205076	0.044	0.011	7.13E-05	0.012	0.017	4.56E-01
2.893111	-0.022	0.006	1.95E-04	-0.022	0.009	1.43E-02
5.205413	0.043	0.011	5.01E-05	0.015	0.016	3.50E-01
2.437245168	-0.036	0.010	2.08E-04	0.013	0.015	3.77E-01
3.507101	0.035	0.010	2.80E-04	0.045	0.014	1.54E-03
2.453393932	-0.031	0.008	2.37E-04	-0.020	0.013	1.06E-01
5.207095	0.042	0.010	5.23E-05	0.009	0.015	5.82E-01
2.441955224	-0.032	0.008	1.15E-04	-0.022	0.012	7.47E-02
2.939539	-0.019	0.005	2.48E-04	-0.005	0.008	5.17E-01
7.680884	0.029	0.009	9.28E-04	0.007	0.013	6.08E-01
2.935838	-0.021	0.005	1.60E-04	-0.009	0.008	2.90E-01
7.048054	-0.024	0.006	2.57E-04	-0.019	0.010	5.50E-02
2.443300954	-0.030	0.009	4.80E-04	-0.027	0.013	3.99E-02
2.939875	-0.019	0.005	2.37E-04	-0.004	0.008	6.20E-01
3.420638	0.034	0.010	3.26E-04	0.044	0.014	1.92E-03
5.375311	0.034	0.009	1.12E-04	0.020	0.013	1.20E-01
2.940212	-0.020	0.005	1.33E-04	-0.003	0.008	7.29E-01
3.090261	-0.023	0.006	1.83E-04	-0.016	0.009	8.62E-02
2.120325667	-0.026	0.007	1.16E-04	-0.014	0.010	1.69E-01
2.456421825	-0.030	0.008	3.72E-04	-0.021	0.013	9.65E-02
2.450702471	-0.032	0.009	2.38E-04	-0.017	0.013	1.91E-01
2.906569	-0.029	0.007	8.30E-05	-0.019	0.011	7.59E-02

3.419965	0.035	0.009	2.37E-04	0.043	0.014	2.15E-03
2.450029606	-0.031	0.009	3.89E-04	-0.017	0.013	1.89E-01
5.21416	0.021	0.006	3.12E-04	-0.001	0.009	8.73E-01
5.204403	0.050	0.013	9.12E-05	0.011	0.019	5.78E-01
3.492298	0.035	0.010	4.13E-04	0.045	0.015	2.09E-03
2.120662099	-0.026	0.007	1.13E-04	-0.015	0.010	1.36E-01
2.905559	-0.028	0.007	9.81E-05	-0.019	0.011	7.33E-02
3.28842	-0.059	0.017	4.67E-04	-0.070	0.025	5.51E-03
2.454066797	-0.032	0.009	2.55E-04	-0.025	0.013	5.66E-02
3.491288	0.034	0.009	2.78E-04	0.044	0.014	1.81E-03
2.626656716	-0.029	0.008	1.48E-04	-0.013	0.011	2.56E-01
2.453730365	-0.031	0.009	3.22E-04	-0.021	0.013	9.96E-02
2.907241	-0.029	0.007	9.55E-05	-0.021	0.011	5.60E-02
3.405162	0.034	0.009	3.28E-04	0.044	0.014	1.70E-03
3.546463	0.039	0.010	1.38E-04	0.036	0.015	1.60E-02
3.28741	-0.057	0.016	3.23E-04	-0.056	0.024	1.74E-02
2.468196966	-0.034	0.008	7.00E-05	-0.008	0.013	5.30E-01
5.20474	0.049	0.013	1.09E-04	0.016	0.019	4.08E-01
3.288083	-0.059	0.017	4.19E-04	-0.068	0.025	6.13E-03
2.441618791	-0.032	0.008	1.44E-04	-0.022	0.012	7.48E-02
1.174613653	0.017	0.004	3.22E-05	0.007	0.006	2.67E-01
5.207431	0.036	0.009	1.01E-04	0.002	0.014	8.80E-01
3.287747	-0.058	0.016	3.36E-04	-0.065	0.024	7.33E-03
2.901859	-0.024	0.006	1.93E-04	-0.012	0.009	2.00E-01
2.891766	-0.022	0.006	2.99E-04	-0.009	0.009	3.04E-01
3.405498	0.034	0.010	4.26E-04	0.045	0.014	1.61E-03
2.903541	-0.026	0.007	8.03E-05	-0.016	0.010	9.52E-02
2.120998532	-0.025	0.007	1.51E-04	-0.017	0.010	9.50E-02
5.205749	0.043	0.011	8.45E-05	0.016	0.017	3.23E-01
2.627666014	-0.028	0.008	3.32E-04	-0.017	0.012	1.38E-01
5.847999	0.032	0.009	5.55E-04	-0.011	0.014	4.11E-01
7.051755	-0.024	0.007	3.52E-04	-0.024	0.010	2.03E-02
3.475813	0.034	0.009	3.10E-04	0.044	0.014	1.82E-03
3.476822	0.034	0.010	5.60E-04	0.045	0.015	2.02E-03
2.905896	-0.028	0.007	9.02E-05	-0.019	0.011	7.24E-02
2.456758258	-0.030	0.008	4.40E-04	-0.022	0.013	8.33E-02
2.479299241	-0.033	0.008	2.35E-05	-0.005	0.011	6.88E-01
2.449356741	-0.031	0.009	3.92E-04	-0.011	0.013	4.03E-01
2.248506484	0.047	0.012	3.77E-05	0.009	0.017	6.02E-01
2.910269	-0.033	0.008	1.04E-04	-0.019	0.013	1.28E-01
3.288756	-0.058	0.017	6.28E-04	-0.071	0.025	5.64E-03
5.199693	0.056	0.015	1.11E-04	0.021	0.022	3.35E-01
2.250188647	0.044	0.011	6.90E-05	0.012	0.016	4.68E-01

2.626993149	-0.027	0.008	2.84E-04	-0.015	0.011	1.87E-01
3.389013	0.033	0.009	4.92E-04	0.043	0.014	2.26E-03
5.20844	0.034	0.009	1.43E-04	0.009	0.013	5.18E-01
2.444646685	-0.028	0.008	7.29E-04	-0.022	0.012	8.19E-02
2.4375816	-0.035	0.010	2.53E-04	0.009	0.014	5.17E-01
2.894793	-0.021	0.006	5.56E-04	-0.016	0.009	7.27E-02
5.206758	0.040	0.010	1.09E-04	0.009	0.015	5.58E-01
2.447338145	-0.028	0.008	5.42E-04	-0.012	0.012	3.07E-01
2.892775	-0.021	0.006	4.17E-04	-0.014	0.009	1.23E-01
2.249852214	0.044	0.011	7.28E-05	0.014	0.017	3.97E-01
2.48165427	-0.035	0.009	1.67E-04	-0.007	0.014	6.40E-01
2.940548	-0.019	0.005	1.57E-04	-0.002	0.008	7.61E-01
2.904886	-0.026	0.007	1.68E-04	-0.024	0.010	1.97E-02
3.777256	0.029	0.008	3.80E-04	0.035	0.012	4.66E-03
2.121334965	-0.025	0.007	2.22E-04	-0.018	0.010	6.77E-02
2.465169073	-0.030	0.008	2.34E-04	-0.025	0.012	4.03E-02
5.848335	0.027	0.008	1.01E-03	0.004	0.012	7.39E-01
2.441282359	-0.032	0.009	2.19E-04	-0.023	0.013	7.50E-02
5.206085	0.042	0.011	1.23E-04	0.014	0.016	3.93E-01
2.444310252	-0.028	0.008	7.98E-04	-0.023	0.013	6.80E-02
5.374638	0.037	0.010	1.23E-04	0.029	0.014	4.62E-02
6.085857	0.027	0.008	9.74E-04	0.022	0.012	7.19E-02
5.202721	0.054	0.014	1.80E-04	0.013	0.022	5.46E-01
3.77692	0.031	0.009	3.65E-04	0.038	0.013	3.41E-03
2.901522	-0.023	0.006	3.85E-04	-0.014	0.009	1.50E-01
2.445655982	-0.026	0.008	1.19E-03	-0.022	0.012	6.74E-02
2.895803	-0.022	0.006	6.04E-04	-0.017	0.010	7.58E-02
3.089252	-0.021	0.006	3.56E-04	-0.009	0.009	2.99E-01
2.119989234	-0.025	0.007	2.72E-04	-0.012	0.010	2.33E-01
5.20003	0.056	0.015	1.53E-04	0.023	0.022	2.89E-01
5.200366	0.061	0.016	1.89E-04	0.028	0.024	2.48E-01
2.478626376	-0.032	0.008	4.11E-05	-0.007	0.012	5.28E-01
3.455963	0.033	0.009	4.68E-04	0.043	0.014	2.07E-03
2.247833619	0.048	0.012	5.95E-05	0.015	0.018	4.13E-01
3.450244	0.033	0.009	5.03E-04	0.045	0.014	1.64E-03
2.94223	-0.018	0.005	3.52E-04	-0.007	0.007	3.64E-01
3.459664	0.033	0.009	4.56E-04	0.044	0.014	1.63E-03
2.478962809	-0.032	0.008	3.48E-05	-0.006	0.012	5.94E-01
2.435226572	-0.024	0.007	9.85E-04	0.002	0.011	8.45E-01
5.202048	0.058	0.015	1.50E-04	0.018	0.023	4.16E-01
2.940885	-0.019	0.005	2.70E-04	-0.003	0.008	6.93E-01
2.903204	-0.023	0.007	3.98E-04	-0.012	0.010	2.37E-01
7.056128	-0.038	0.012	1.08E-03	-0.016	0.017	3.71E-01

3.404825	0.032	0.009	4.83E-04	0.042	0.014	1.98E-03
2.891429	-0.021	0.006	6.39E-04	-0.005	0.009	6.17E-01
1.121457304	0.024	0.007	3.40E-04	0.007	0.010	4.79E-01
2.895466	-0.022	0.006	5.46E-04	-0.017	0.009	7.33E-02
5.202385	0.055	0.015	1.85E-04	0.014	0.022	5.37E-01
5.198684	0.059	0.016	1.70E-04	0.020	0.024	3.90E-01
5.3669	0.044	0.012	3.19E-04	0.026	0.018	1.51E-01
5.376993	0.044	0.012	2.01E-04	-0.009	0.018	5.91E-01
2.931465	-0.024	0.006	1.65E-04	-0.025	0.009	8.66E-03
3.446543	0.032	0.009	6.06E-04	0.043	0.014	1.96E-03
2.952323	-0.017	0.005	3.06E-04	-0.005	0.007	5.14E-01
2.413022021	-0.032	0.009	7.63E-04	0.009	0.014	5.25E-01
3.289092	-0.058	0.017	8.89E-04	-0.070	0.026	6.79E-03
5.846989	0.029	0.009	1.15E-03	-0.001	0.013	9.61E-01
5.200702	0.060	0.016	2.03E-04	0.025	0.024	3.03E-01
5.212478	0.024	0.007	3.52E-04	-0.004	0.010	7.12E-01
3.388677	0.032	0.009	5.67E-04	0.041	0.014	3.07E-03
2.896476	-0.022	0.007	1.02E-03	-0.022	0.010	2.47E-02
5.208104	0.036	0.010	2.05E-04	0.002	0.014	9.12E-01
2.906905	-0.027	0.007	2.07E-04	-0.021	0.011	5.48E-02
2.931128	-0.024	0.006	1.94E-04	-0.022	0.010	1.93E-02
3.46	0.032	0.010	7.20E-04	0.045	0.014	1.50E-03
3.776583	0.032	0.009	4.68E-04	0.041	0.014	3.29E-03
2.450366039	-0.031	0.009	6.63E-04	-0.015	0.013	2.53E-01
2.900513	-0.022	0.006	4.15E-04	-0.019	0.009	3.71E-02
3.420974	0.032	0.009	8.51E-04	0.042	0.014	2.74E-03
5.847326	0.033	0.010	8.28E-04	-0.006	0.015	6.86E-01
5.183208	0.048	0.013	1.54E-04	0.024	0.019	1.98E-01
2.439263763	-0.030	0.008	3.71E-04	-0.008	0.013	5.16E-01
5.379012	0.035	0.010	2.85E-04	0.020	0.014	1.58E-01
3.088242	-0.022	0.006	3.37E-04	-0.010	0.009	2.44E-01
5.397852	0.028	0.008	8.31E-04	-0.005	0.013	6.85E-01
5.206422	0.040	0.011	1.87E-04	0.011	0.016	4.78E-01
2.445992415	-0.026	0.008	1.72E-03	-0.020	0.012	9.72E-02
3.287074	-0.052	0.015	6.62E-04	-0.045	0.023	4.80E-02
5.210459	0.028	0.008	4.74E-04	-0.001	0.012	9.23E-01
2.930792	-0.023	0.006	2.70E-04	-0.022	0.010	1.93E-02
2.910606	-0.030	0.008	2.54E-04	-0.021	0.012	9.10E-02
3.768509	0.031	0.009	4.69E-04	0.032	0.013	1.55E-02
3.507437	0.031	0.009	9.18E-04	0.043	0.014	2.41E-03
3.908128	0.030	0.009	6.10E-04	0.039	0.013	2.67E-03
5.211468	0.027	0.008	4.22E-04	-0.001	0.011	9.50E-01
3.090597	-0.022	0.006	5.63E-04	-0.014	0.009	1.37E-01

5.183881	0.047	0.012	1.71E-04	0.024	0.019	1.90E-01
3.449907	0.032	0.009	4.94E-04	0.042	0.014	2.25E-03
3.405835	0.031	0.010	9.63E-04	0.044	0.014	2.18E-03
2.932474	-0.021	0.006	2.94E-04	-0.022	0.009	1.31E-02
2.62968461	-0.026	0.008	8.71E-04	-0.012	0.012	3.07E-01
2.25052508	0.041	0.011	1.31E-04	0.014	0.016	3.66E-01
2.444983117	-0.027	0.008	1.16E-03	-0.020	0.012	1.09E-01
3.492634	0.032	0.010	9.97E-04	0.043	0.015	3.08E-03
3.456299	0.032	0.010	8.87E-04	0.043	0.014	2.31E-03
2.438590898	-0.034	0.009	2.43E-04	-0.005	0.014	7.34E-01
2.629348177	-0.027	0.008	8.14E-04	-0.011	0.012	3.79E-01
5.183544	0.047	0.012	1.75E-04	0.023	0.019	2.07E-01
2.93752	-0.019	0.005	4.14E-04	-0.010	0.008	2.09E-01
2.436908735	-0.033	0.010	8.58E-04	0.021	0.015	1.49E-01
2.438927331	-0.031	0.009	3.04E-04	-0.006	0.013	6.62E-01
2.956697	-0.015	0.005	6.81E-04	-0.007	0.007	2.79E-01
2.440945926	-0.031	0.009	4.47E-04	-0.023	0.013	7.57E-02
2.248170051	0.046	0.012	7.36E-05	0.011	0.017	5.10E-01
2.443973819	-0.028	0.009	1.20E-03	-0.024	0.013	6.20E-02
3.081177	-0.020	0.006	4.08E-04	-0.008	0.008	3.62E-01
2.936175	-0.020	0.005	3.58E-04	-0.008	0.008	3.56E-01
4.080718	0.076	0.020	1.34E-04	0.044	0.030	1.41E-01
2.44531955	-0.026	0.008	1.40E-03	-0.020	0.012	1.09E-01
2.246487888	0.051	0.013	1.68E-04	0.039	0.020	5.12E-02
2.411676291	-0.035	0.010	7.54E-04	-0.015	0.016	3.48E-01
5.214833	0.020	0.006	5.51E-04	-0.004	0.009	6.40E-01
2.900849	-0.022	0.006	4.64E-04	-0.015	0.009	1.15E-01
2.439600196	-0.029	0.008	6.07E-04	-0.013	0.012	2.89E-01
3.777593	0.027	0.008	6.74E-04	0.034	0.012	4.09E-03
2.247160754	0.048	0.013	1.28E-04	0.025	0.019	1.90E-01
2.905223	-0.025	0.007	2.85E-04	-0.022	0.010	3.31E-02
2.443637387	-0.028	0.009	1.35E-03	-0.025	0.013	5.35E-02
2.438254465	-0.035	0.009	2.62E-04	0.005	0.014	7.39E-01
5.198347	0.061	0.017	2.36E-04	0.020	0.025	4.09E-01
5.182872	0.048	0.013	1.84E-04	0.024	0.019	2.06E-01
3.446207	0.031	0.009	5.89E-04	0.041	0.014	2.52E-03
3.466392	0.032	0.009	6.60E-04	0.043	0.014	2.14E-03
5.203057	0.049	0.013	2.87E-04	0.014	0.020	4.80E-01
0.894365305	0.048	0.013	2.57E-04	0.021	0.020	2.79E-01
2.898494	-0.020	0.006	9.61E-04	-0.015	0.009	8.70E-02
3.477158	0.032	0.010	1.24E-03	0.043	0.015	3.60E-03
2.941221	-0.018	0.005	5.69E-04	-0.004	0.008	6.21E-01
3.455627	0.032	0.009	5.43E-04	0.041	0.014	2.87E-03

0.894028872	0.049	0.013	2.60E-04	0.024	0.020	2.19E-01
3.904428	0.029	0.009	6.93E-04	0.038	0.013	3.05E-03
5.845644	0.032	0.010	2.07E-03	0.009	0.015	5.40E-01
3.908465	0.030	0.009	8.03E-04	0.042	0.013	1.53E-03
2.959725	-0.016	0.005	6.21E-04	-0.011	0.007	8.97E-02
2.935502	-0.019	0.005	3.57E-04	-0.014	0.008	8.19E-02
3.389349	0.031	0.009	1.17E-03	0.043	0.014	2.37E-03
2.121671397	-0.023	0.007	4.44E-04	-0.021	0.010	3.75E-02
5.845307	0.026	0.008	2.28E-03	0.008	0.013	5.02E-01
5.212141	0.025	0.007	4.40E-04	-0.006	0.011	5.69E-01
8.208746	0.029	0.009	9.27E-04	0.018	0.013	1.53E-01
5.367237	0.041	0.012	4.11E-04	0.024	0.017	1.59E-01
2.907578	-0.028	0.007	1.65E-04	-0.023	0.011	3.49E-02
3.081514	-0.021	0.006	3.00E-04	-0.006	0.009	4.57E-01
1.108672865	0.019	0.006	8.24E-04	0.010	0.008	2.17E-01
2.90926	-0.031	0.008	3.07E-04	-0.020	0.013	1.07E-01
7.873996	0.028	0.009	1.01E-03	0.011	0.013	3.92E-01
3.414582	0.028	0.008	1.10E-03	0.040	0.013	1.60E-03
5.207768	0.033	0.009	3.10E-04	-0.003	0.014	8.22E-01
2.248842917	0.044	0.011	1.05E-04	0.010	0.017	5.69E-01
2.245815023	0.053	0.014	1.95E-04	0.038	0.021	7.09E-02
5.84598	0.026	0.008	1.16E-03	0.013	0.012	2.80E-01
2.893448	-0.020	0.006	6.71E-04	-0.020	0.009	2.17E-02
2.439936628	-0.028	0.008	8.63E-04	-0.019	0.013	1.21E-01
2.9281	-0.023	0.006	4.38E-04	-0.024	0.010	1.50E-02
2.930119	-0.022	0.006	5.55E-04	-0.016	0.009	8.06E-02
2.412685588	-0.031	0.010	9.79E-04	0.003	0.014	8.38E-01
1.59616369	0.061	0.016	1.39E-04	0.030	0.024	2.01E-01
2.246151456	0.052	0.014	1.90E-04	0.038	0.021	6.74E-02
5.376657	0.032	0.009	3.31E-04	0.010	0.013	4.52E-01
2.249515782	0.043	0.011	1.40E-04	0.014	0.017	4.20E-01
7.223672	-0.036	0.009	1.88E-04	-0.016	0.014	2.52E-01
2.931801	-0.022	0.006	3.23E-04	-0.022	0.009	1.70E-02
3.414245	0.028	0.008	1.05E-03	0.040	0.013	1.69E-03
3.462692	0.030	0.009	9.21E-04	0.042	0.014	2.05E-03
3.506092	0.031	0.009	7.08E-04	0.041	0.014	3.21E-03
3.466729	0.032	0.010	1.05E-03	0.043	0.014	2.57E-03
0.893692439	0.050	0.014	2.82E-04	0.027	0.021	1.97E-01
2.246824321	0.049	0.013	1.85E-04	0.034	0.019	8.19E-02
3.446879	0.030	0.009	1.21E-03	0.042	0.014	2.23E-03
5.366564	0.045	0.013	4.34E-04	0.027	0.019	1.65E-01
7.76701	-0.034	0.009	1.27E-04	-0.035	0.013	8.61E-03
5.369255	0.035	0.010	3.60E-04	0.028	0.014	5.03E-02

3.709297	0.029	0.009	7.63E-04	0.035	0.013	5.78E-03
2.481990702	-0.036	0.010	2.51E-04	-0.013	0.015	3.76E-01
2.434890139	-0.024	0.007	1.38E-03	-0.007	0.011	5.48E-01
5.369592	0.038	0.011	3.81E-04	0.023	0.016	1.47E-01
0.894701737	0.047	0.013	2.96E-04	0.018	0.019	3.52E-01
3.45058	0.031	0.010	1.17E-03	0.043	0.014	2.57E-03
2.934829	-0.020	0.005	1.97E-04	-0.010	0.008	2.41E-01
4.080382	0.076	0.020	1.83E-04	0.050	0.030	9.67E-02
5.201712	0.056	0.015	2.85E-04	0.019	0.023	4.00E-01
2.466851236	-0.029	0.008	7.51E-04	-0.023	0.013	7.35E-02
3.768172	0.032	0.009	5.49E-04	0.035	0.014	1.25E-02
5.230309	0.028	0.009	1.25E-03	0.032	0.013	1.23E-02
1.595827257	0.061	0.016	1.69E-04	0.031	0.024	1.94E-01
1.120784438	0.020	0.006	5.88E-04	0.008	0.009	3.52E-01
5.19902	0.058	0.016	3.49E-04	0.027	0.024	2.61E-01
5.199357	0.055	0.015	3.40E-04	0.024	0.023	2.92E-01
3.459327	0.031	0.009	7.07E-04	0.040	0.014	2.89E-03
2.247497186	0.046	0.012	1.44E-04	0.018	0.018	3.25E-01
3.887606	0.026	0.008	8.57E-04	0.036	0.012	2.02E-03
3.490952	0.031	0.009	8.56E-04	0.042	0.014	2.60E-03
1.381856129	0.040	0.011	1.71E-04	0.028	0.016	7.72E-02
2.892102	-0.020	0.006	8.60E-04	-0.013	0.009	1.41E-01
0.893356007	0.052	0.014	2.92E-04	0.027	0.021	2.11E-01
3.610386	-0.024	0.007	1.40E-03	0.006	0.011	6.06E-01
3.413236	0.027	0.008	1.20E-03	0.039	0.013	1.94E-03
2.901186	-0.021	0.006	6.67E-04	-0.012	0.009	2.03E-01
2.892438	-0.020	0.006	9.11E-04	-0.008	0.009	3.45E-01
2.936848	-0.019	0.005	4.67E-04	-0.011	0.008	1.58E-01
2.437918033	-0.034	0.010	4.11E-04	0.013	0.014	3.52E-01
2.890083	-0.020	0.006	1.42E-03	-0.010	0.009	2.78E-01
1.593472229	0.067	0.018	2.01E-04	0.031	0.027	2.47E-01
3.904764	0.029	0.009	1.01E-03	0.041	0.013	1.46E-03
3.768845	0.028	0.008	9.16E-04	0.029	0.012	1.81E-02
3.776247	0.033	0.010	8.41E-04	0.044	0.015	2.64E-03
2.896139	-0.020	0.007	1.85E-03	-0.020	0.010	3.91E-02
7.712508	-0.033	0.010	7.00E-04	-0.020	0.014	1.70E-01
2.249179349	0.043	0.011	1.58E-04	0.009	0.017	6.00E-01
2.440609494	-0.029	0.009	8.84E-04	-0.023	0.013	7.76E-02
5.37632	0.028	0.008	5.22E-04	0.008	0.012	5.14E-01
2.245478591	0.054	0.015	2.38E-04	0.038	0.022	8.61E-02
3.413909	0.027	0.008	1.18E-03	0.039	0.013	2.16E-03
5.230645	0.027	0.009	1.55E-03	0.029	0.013	2.33E-02
3.419628	0.031	0.009	7.75E-04	0.040	0.014	3.47E-03

5.184217	0.046	0.013	2.88E-04	0.020	0.019	2.99E-01
6.419934	0.024	0.008	2.22E-03	0.008	0.012	5.02E-01
5.201039	0.057	0.016	3.75E-04	0.023	0.024	3.43E-01
3.530651	0.029	0.009	1.40E-03	0.036	0.014	8.04E-03
1.596500122	0.059	0.016	1.80E-04	0.031	0.023	1.90E-01
5.182535	0.047	0.013	2.74E-04	0.024	0.019	2.20E-01
3.413573	0.027	0.008	1.22E-03	0.039	0.013	2.02E-03
0.89503817	0.047	0.013	3.42E-04	0.016	0.020	4.16E-01
2.925745	-0.023	0.006	3.53E-04	-0.025	0.010	8.88E-03
2.947613	-0.016	0.005	1.29E-03	-0.010	0.007	1.62E-01
3.403816	0.029	0.009	1.25E-03	0.044	0.013	1.03E-03
1.385556888	0.031	0.009	2.47E-04	0.022	0.013	9.02E-02
5.195656	0.061	0.017	2.82E-04	0.026	0.025	2.96E-01
3.463028	0.029	0.009	1.48E-03	0.042	0.014	2.18E-03
3.907792	0.029	0.009	9.93E-04	0.037	0.013	3.79E-03
5.229972	0.027	0.009	1.33E-03	0.035	0.013	6.00E-03
3.460337	0.030	0.010	1.66E-03	0.043	0.014	2.47E-03
2.124026425	-0.024	0.007	4.99E-04	-0.008	0.010	4.55E-01
5.236701	0.026	0.008	1.61E-03	0.030	0.013	1.63E-02
3.887943	0.026	0.008	1.05E-03	0.038	0.012	1.14E-03
3.472448	0.029	0.009	1.49E-03	0.042	0.013	1.68E-03
5.375984	0.029	0.008	4.95E-04	0.008	0.012	5.22E-01
2.909597	-0.030	0.009	4.53E-04	-0.018	0.013	1.53E-01
2.902195	-0.021	0.006	9.19E-04	-0.011	0.010	2.58E-01
3.078149	-0.021	0.006	6.40E-04	-0.010	0.009	2.72E-01
2.937857	-0.017	0.005	1.13E-03	-0.008	0.008	2.94E-01
2.435899437	-0.024	0.008	2.40E-03	0.017	0.012	1.52E-01
5.198011	0.061	0.017	3.60E-04	0.023	0.026	3.77E-01
2.626320284	-0.026	0.008	8.09E-04	-0.015	0.012	1.93E-01
3.018264	-0.014	0.004	1.23E-03	-0.008	0.006	2.24E-01
3.414918	0.027	0.008	1.61E-03	0.039	0.013	1.94E-03
1.385220455	0.032	0.009	2.76E-04	0.022	0.013	9.41E-02
2.94795	-0.016	0.005	1.06E-03	-0.008	0.007	2.66E-01
3.4129	0.027	0.008	1.37E-03	0.039	0.013	1.87E-03
3.524259	0.029	0.009	1.40E-03	0.035	0.014	1.02E-02
2.468533399	-0.031	0.009	2.69E-04	-0.007	0.013	5.69E-01
2.478289944	-0.030	0.008	1.16E-04	-0.012	0.012	3.05E-01
3.709633	0.029	0.009	8.86E-04	0.035	0.013	5.82E-03
7.046708	-0.032	0.009	4.77E-04	-0.014	0.014	2.92E-01
3.404489	0.029	0.009	1.08E-03	0.041	0.013	2.22E-03
2.933483	-0.019	0.006	8.21E-04	-0.011	0.008	1.84E-01
5.236364	0.026	0.008	1.54E-03	0.033	0.012	8.54E-03
3.962294	-0.027	0.008	9.71E-04	-0.016	0.012	1.83E-01

2.411339858	-0.036	0.011	1.01E-03	-0.016	0.016	3.24E-01
5.203394	0.047	0.013	4.31E-04	0.015	0.020	4.49E-01
3.777929	0.026	0.008	1.08E-03	0.036	0.012	2.91E-03
2.242114265	0.073	0.020	2.86E-04	0.048	0.030	1.11E-01
3.462355	0.029	0.009	1.11E-03	0.040	0.013	2.32E-03
3.530315	0.029	0.009	1.22E-03	0.034	0.013	1.03E-02
2.242450697	0.071	0.020	2.78E-04	0.046	0.029	1.13E-01
1.593808662	0.065	0.018	2.33E-04	0.029	0.026	2.78E-01
6.153816	0.026	0.008	7.00E-04	0.039	0.012	8.53E-04
2.12200783	-0.023	0.007	7.16E-04	-0.023	0.010	2.14E-02
1.382192562	0.039	0.010	1.92E-04	0.024	0.015	1.18E-01
3.289429	-0.056	0.018	1.50E-03	-0.070	0.026	7.63E-03
2.929782	-0.020	0.006	9.86E-04	-0.015	0.009	9.81E-02
5.211132	0.027	0.008	6.70E-04	-0.001	0.012	9.04E-01
1.376136775	0.050	0.014	2.98E-04	0.024	0.021	2.45E-01
3.472112	0.029	0.009	1.22E-03	0.041	0.013	2.14E-03
2.951987	-0.016	0.005	9.66E-04	-0.004	0.007	6.11E-01
3.258477	0.028	0.009	1.58E-03	0.034	0.013	1.04E-02
7.510985	-0.026	0.009	2.88E-03	-0.021	0.013	1.04E-01
1.38589332	0.030	0.008	2.22E-04	0.018	0.012	1.38E-01
3.018601	-0.014	0.004	1.32E-03	-0.007	0.006	2.61E-01
1.381183264	0.040	0.011	2.43E-04	0.026	0.016	1.16E-01
2.440273061	-0.027	0.009	1.33E-03	-0.023	0.013	7.23E-02
5.195992	0.060	0.017	4.20E-04	0.023	0.026	3.61E-01
2.894457	-0.019	0.006	1.39E-03	-0.014	0.009	1.14E-01
0.893019574	0.053	0.015	3.60E-04	0.026	0.022	2.36E-01
3.08185	-0.020	0.006	5.82E-04	-0.004	0.009	6.70E-01
3.398097	0.026	0.009	2.29E-03	0.034	0.013	7.24E-03
3.39776	0.027	0.009	1.88E-03	0.035	0.013	6.55E-03
1.377146073	0.048	0.013	3.15E-04	0.025	0.020	2.18E-01
1.394640568	0.025	0.007	2.57E-04	0.018	0.010	6.57E-02
2.910942	-0.028	0.008	5.58E-04	-0.018	0.012	1.42E-01
2.959389	-0.015	0.005	1.07E-03	-0.014	0.007	4.62E-02
2.629011745	-0.026	0.008	1.64E-03	-0.013	0.012	2.89E-01
3.456636	0.029	0.009	2.06E-03	0.041	0.014	3.52E-03
5.197338	0.062	0.018	5.11E-04	0.020	0.027	4.48E-01
3.849926	0.026	0.008	1.45E-03	0.034	0.012	6.40E-03
2.24278713	0.068	0.019	3.40E-04	0.044	0.028	1.23E-01
3.904091	0.028	0.009	1.17E-03	0.035	0.013	6.62E-03
2.435563005	-0.022	0.007	2.66E-03	0.008	0.011	4.72E-01
5.197675	0.063	0.018	4.35E-04	0.020	0.027	4.45E-01
2.92911	-0.021	0.006	9.56E-04	-0.015	0.009	1.09E-01
0.895374602	0.046	0.013	4.08E-04	0.014	0.019	4.80E-01

5.196329	0.058	0.016	4.08E-04	0.019	0.024	4.32E-01
2.124362858	-0.025	0.007	5.41E-04	-0.006	0.011	5.47E-01
5.230981	0.026	0.009	2.43E-03	0.027	0.013	3.71E-02
2.412012723	-0.032	0.010	1.56E-03	-0.004	0.015	8.00E-01
2.935165	-0.019	0.005	4.40E-04	-0.013	0.008	1.01E-01
3.611058	-0.027	0.008	1.08E-03	0.006	0.012	6.12E-01
5.369928	0.047	0.014	5.79E-04	0.024	0.020	2.46E-01
3.729819	0.028	0.009	1.64E-03	0.036	0.013	6.04E-03
5.197002	0.062	0.018	5.54E-04	0.025	0.027	3.48E-01
5.181526	0.044	0.012	3.52E-04	0.020	0.018	2.87E-01
1.394977	0.024	0.007	2.76E-04	0.018	0.010	7.25E-02
3.404152	0.028	0.009	1.50E-03	0.042	0.013	1.70E-03
2.896812	-0.020	0.007	2.16E-03	-0.020	0.010	4.11E-02
3.430058	0.026	0.008	2.00E-03	0.036	0.013	4.31E-03
3.080168	-0.019	0.006	9.79E-04	0.000	0.009	9.89E-01
2.117297773	-0.023	0.007	9.57E-04	-0.003	0.010	7.53E-01
2.136474431	-0.031	0.009	3.41E-04	-0.008	0.013	5.31E-01
3.018937	-0.013	0.004	1.68E-03	-0.007	0.006	2.60E-01
3.406171	0.029	0.009	2.27E-03	0.042	0.014	3.13E-03
1.598182285	0.052	0.014	3.09E-04	0.018	0.022	4.16E-01
3.836468	0.025	0.008	1.50E-03	0.035	0.012	3.54E-03
2.897821	-0.019	0.006	1.91E-03	-0.016	0.009	8.45E-02
1.27285197	0.074	0.020	3.20E-04	0.032	0.031	2.95E-01
5.215842	0.018	0.005	1.13E-03	0.005	0.008	5.66E-01
1.272515537	0.074	0.020	3.26E-04	0.032	0.031	2.94E-01
1.271842672	0.074	0.021	3.35E-04	0.032	0.031	2.92E-01
2.930455	-0.021	0.006	8.95E-04	-0.018	0.009	5.11E-02
3.42131	0.028	0.009	2.11E-03	0.040	0.014	4.14E-03
2.898158	-0.019	0.006	1.68E-03	-0.013	0.009	1.62E-01
2.243123563	0.066	0.018	3.80E-04	0.045	0.028	1.02E-01
1.272179104	0.074	0.021	3.34E-04	0.032	0.031	2.94E-01
3.477495	0.030	0.010	2.50E-03	0.041	0.015	4.68E-03
2.902868	-0.021	0.006	1.15E-03	-0.012	0.010	2.10E-01
5.374975	0.033	0.009	5.58E-04	0.021	0.014	1.45E-01
3.021965	-0.013	0.004	1.06E-03	-0.008	0.006	1.90E-01
1.273188402	0.073	0.020	3.24E-04	0.032	0.030	2.98E-01
5.182199	0.046	0.013	3.86E-04	0.022	0.019	2.61E-01
2.447001713	-0.026	0.009	2.24E-03	-0.015	0.013	2.57E-01
2.241777832	0.074	0.021	3.46E-04	0.045	0.031	1.40E-01
7.047045	-0.028	0.008	3.70E-04	-0.024	0.012	4.35E-02
5.184554	0.047	0.013	4.29E-04	0.017	0.020	4.04E-01
1.273524835	0.073	0.020	3.26E-04	0.031	0.030	3.00E-01
3.09127	-0.022	0.007	7.24E-04	-0.020	0.010	4.68E-02

3.492971	0.030	0.010	2.20E-03	0.041	0.015	5.04E-03
1.273861267	0.072	0.020	3.22E-04	0.031	0.030	2.99E-01
1.591453633	0.070	0.019	3.21E-04	0.035	0.029	2.23E-01
3.258814	0.028	0.009	1.02E-03	0.039	0.013	2.90E-03
5.229636	0.027	0.009	1.73E-03	0.038	0.013	3.19E-03
5.379348	0.024	0.007	5.26E-04	0.009	0.011	3.96E-01
1.271506239	0.074	0.021	3.55E-04	0.032	0.031	2.92E-01
2.244805725	0.056	0.016	3.85E-04	0.033	0.023	1.58E-01
3.961621	-0.027	0.008	7.43E-04	-0.017	0.012	1.52E-01
1.393967702	0.025	0.007	2.60E-04	0.018	0.010	7.34E-02
5.18018	0.044	0.012	3.16E-04	0.024	0.018	1.91E-01
3.429721	0.026	0.008	1.91E-03	0.036	0.013	4.60E-03
5.181189	0.043	0.012	3.57E-04	0.026	0.018	1.53E-01
1.598518718	0.051	0.014	3.28E-04	0.017	0.021	4.34E-01
2.24413286	0.060	0.017	3.49E-04	0.039	0.025	1.17E-01
3.849589	0.026	0.008	1.48E-03	0.034	0.012	6.35E-03
4.080046	0.072	0.020	3.57E-04	0.046	0.030	1.23E-01
2.245142158	0.054	0.015	3.94E-04	0.036	0.023	1.17E-01
2.929446	-0.020	0.006	1.30E-03	-0.017	0.009	7.28E-02
3.70896	0.027	0.009	1.62E-03	0.036	0.013	4.97E-03
5.180853	0.043	0.012	3.16E-04	0.028	0.018	1.20E-01
3.908801	0.028	0.009	1.83E-03	0.044	0.014	1.15E-03
3.961958	-0.027	0.008	9.81E-04	-0.016	0.012	1.89E-01
3.836132	0.025	0.008	1.72E-03	0.035	0.012	3.56E-03
1.2741977	0.072	0.020	3.32E-04	0.031	0.030	3.00E-01
3.714343	0.026	0.008	1.64E-03	0.037	0.012	2.12E-03
3.530988	0.027	0.009	2.43E-03	0.036	0.013	7.14E-03
3.103718	-0.030	0.009	5.05E-04	-0.028	0.013	3.30E-02
1.381519697	0.039	0.011	2.81E-04	0.026	0.016	1.06E-01
3.853626	0.025	0.008	1.85E-03	0.032	0.012	7.81E-03
5.709052	0.027	0.009	2.49E-03	0.002	0.013	8.53E-01
5.237037	0.025	0.008	2.61E-03	0.027	0.013	3.52E-02
5.211805	0.024	0.007	1.02E-03	-0.004	0.011	7.41E-01
7.681893	0.025	0.008	1.67E-03	0.008	0.012	5.05E-01
3.258141	0.026	0.009	3.32E-03	0.031	0.013	2.05E-02
3.087906	-0.020	0.006	1.05E-03	-0.006	0.009	4.94E-01
3.466056	0.030	0.009	1.14E-03	0.039	0.014	3.77E-03
1.601210179	0.046	0.013	2.89E-04	0.011	0.019	5.61E-01
3.447216	0.027	0.009	2.52E-03	0.040	0.013	2.91E-03
1.271169807	0.073	0.021	3.87E-04	0.032	0.031	2.95E-01
6.154489	0.029	0.009	1.04E-03	0.004	0.013	7.53E-01
3.523922	0.028	0.009	1.49E-03	0.034	0.013	1.09E-02
3.017928	-0.013	0.004	1.56E-03	-0.008	0.006	2.18E-01

1.274534133	0.071	0.020	3.46E-04	0.031	0.030	3.04E-01
1.372772449	0.055	0.016	4.52E-04	0.031	0.024	1.94E-01
3.507774	0.028	0.009	2.39E-03	0.041	0.014	3.45E-03
3.730156	0.028	0.009	1.65E-03	0.035	0.013	6.95E-03
2.470888427	-0.027	0.008	1.10E-03	-0.015	0.012	2.09E-01
3.832095	0.026	0.008	1.87E-03	0.038	0.013	2.47E-03
1.596836555	0.056	0.015	3.05E-04	0.028	0.023	2.25E-01
1.372099584	0.056	0.016	4.30E-04	0.029	0.024	2.16E-01
2.922045	-0.022	0.006	5.61E-04	-0.010	0.009	2.93E-01
2.941894	-0.017	0.005	9.35E-04	-0.005	0.008	4.74E-01
3.719053	0.027	0.008	1.19E-03	0.033	0.012	8.73E-03
3.289765	-0.055	0.017	1.52E-03	-0.068	0.026	9.46E-03
5.356807	0.053	0.016	6.42E-04	0.015	0.023	5.20E-01
3.88727	0.025	0.008	1.48E-03	0.035	0.012	2.69E-03
3.836805	0.025	0.008	1.77E-03	0.035	0.012	3.83E-03
3.850262	0.026	0.008	2.13E-03	0.035	0.012	5.14E-03
3.286737	-0.047	0.015	1.34E-03	-0.039	0.022	6.98E-02
1.380846831	0.040	0.011	3.36E-04	0.025	0.017	1.35E-01
3.96835	-0.026	0.008	1.09E-03	-0.011	0.012	3.65E-01
3.71468	0.025	0.008	1.75E-03	0.036	0.012	3.29E-03
1.39228554	0.025	0.007	3.69E-04	0.016	0.011	1.41E-01
3.610049	-0.022	0.007	1.94E-03	0.001	0.011	9.20E-01
1.591790066	0.069	0.019	3.48E-04	0.034	0.029	2.35E-01
7.712172	-0.030	0.010	1.95E-03	-0.012	0.015	4.25E-01
1.274870565	0.070	0.020	3.64E-04	0.030	0.030	3.08E-01
3.884242	0.025	0.008	1.79E-03	0.038	0.012	1.36E-03
2.928437	-0.021	0.006	8.93E-04	-0.025	0.010	1.01E-02
1.371763151	0.056	0.016	4.60E-04	0.032	0.024	1.77E-01
5.236028	0.026	0.008	2.08E-03	0.035	0.012	4.84E-03
5.18489	0.050	0.014	4.46E-04	0.016	0.021	4.45E-01
1.270833374	0.073	0.021	4.23E-04	0.032	0.031	2.99E-01
5.196665	0.056	0.016	5.29E-04	0.019	0.024	4.37E-01
2.936511	-0.018	0.005	8.27E-04	-0.006	0.008	4.65E-01
3.610722	-0.024	0.008	1.82E-03	0.004	0.012	7.13E-01
3.883905	0.025	0.008	1.64E-03	0.035	0.012	3.13E-03
2.630021042	-0.024	0.008	1.80E-03	-0.008	0.012	4.98E-01
1.589435038	0.073	0.021	4.12E-04	0.034	0.031	2.63E-01
1.373781747	0.054	0.015	4.54E-04	0.033	0.023	1.51E-01
8.130357	0.028	0.009	2.33E-03	0.000	0.014	9.77E-01
1.37680964	0.048	0.014	4.59E-04	0.024	0.020	2.27E-01
2.922381	-0.021	0.006	1.01E-03	-0.012	0.009	1.97E-01
2.946268	-0.016	0.005	1.33E-03	-0.005	0.007	4.74E-01
2.2414414	0.075	0.021	3.97E-04	0.046	0.031	1.46E-01

5.201375	0.053	0.016	6.72E-04	0.016	0.023	5.02E-01
1.376473208	0.048	0.014	4.50E-04	0.024	0.021	2.51E-01
3.090934	-0.021	0.007	1.07E-03	-0.016	0.010	1.11E-01
5.180517	0.043	0.012	3.46E-04	0.026	0.018	1.48E-01
2.908924	-0.029	0.009	7.59E-04	-0.019	0.013	1.45E-01
3.415255	0.026	0.008	2.43E-03	0.039	0.013	2.09E-03
1.275206998	0.070	0.020	3.77E-04	0.030	0.029	3.10E-01
3.962631	-0.026	0.008	1.59E-03	-0.014	0.013	2.61E-01
2.631366773	-0.025	0.008	1.23E-03	-0.013	0.011	2.58E-01
1.595490825	0.059	0.016	3.62E-04	0.029	0.025	2.40E-01
2.243796428	0.061	0.017	4.32E-04	0.041	0.026	1.13E-01
8.389747	0.022	0.008	3.21E-03	0.024	0.011	3.68E-02
3.40348	0.028	0.009	1.91E-03	0.043	0.013	1.47E-03
3.430394	0.025	0.008	2.69E-03	0.035	0.012	5.59E-03
1.589098605	0.073	0.021	4.16E-04	0.039	0.031	2.06E-01
1.377482506	0.047	0.013	3.89E-04	0.023	0.020	2.36E-01
3.475476	0.030	0.009	1.45E-03	0.040	0.014	4.11E-03
2.941558	-0.017	0.005	1.05E-03	-0.003	0.008	7.30E-01
3.837141	0.025	0.008	1.96E-03	0.035	0.012	3.38E-03
1.386229753	0.028	0.008	2.88E-04	0.017	0.012	1.45E-01
3.082186	-0.019	0.006	9.16E-04	-0.006	0.009	4.62E-01
3.088915	-0.020	0.006	9.03E-04	-0.011	0.009	2.10E-01
3.831758	0.026	0.008	2.06E-03	0.038	0.012	2.33E-03
2.243459995	0.063	0.018	4.56E-04	0.043	0.027	1.05E-01
1.373445314	0.054	0.016	4.88E-04	0.031	0.023	1.80E-01
1.378155371	0.046	0.013	3.62E-04	0.028	0.019	1.48E-01
3.44587	0.029	0.009	1.30E-03	0.037	0.013	5.85E-03
3.38834	0.029	0.009	1.62E-03	0.038	0.014	5.28E-03
1.270496942	0.072	0.020	4.62E-04	0.032	0.031	3.03E-01
5.19532	0.063	0.018	4.91E-04	0.025	0.027	3.53E-01
1.594145094	0.062	0.017	3.43E-04	0.027	0.026	2.90E-01
3.022301	-0.014	0.004	1.17E-03	-0.008	0.006	1.84E-01
2.446328848	-0.025	0.009	4.23E-03	-0.017	0.013	1.77E-01
3.450917	0.029	0.010	2.80E-03	0.042	0.014	3.13E-03
2.902531	-0.020	0.006	1.76E-03	-0.012	0.010	2.06E-01
1.378828236	0.045	0.013	4.09E-04	0.028	0.019	1.32E-01
7.055792	-0.028	0.009	2.39E-03	-0.008	0.014	5.70E-01
3.85329	0.025	0.008	2.03E-03	0.033	0.012	5.86E-03
5.194983	0.064	0.018	4.86E-04	0.023	0.027	3.92E-01
3.463365	0.027	0.009	2.98E-03	0.042	0.014	2.58E-03
1.27554343	0.069	0.020	4.00E-04	0.029	0.029	3.12E-01
3.092279	-0.024	0.007	7.72E-04	-0.018	0.011	9.80E-02
1.373108882	0.054	0.016	5.35E-04	0.031	0.023	1.90E-01

3.718717	0.026	0.008	1.56E-03	0.033	0.012	7.52E-03
0.697215806	0.046	0.013	4.47E-04	0.013	0.019	5.17E-01
2.95266	-0.015	0.005	1.06E-03	-0.006	0.007	4.00E-01
3.460673	0.027	0.009	3.27E-03	0.042	0.014	2.59E-03
3.467065	0.029	0.010	2.85E-03	0.042	0.014	4.02E-03
1.378491803	0.045	0.013	4.18E-04	0.028	0.019	1.43E-01
3.905101	0.027	0.009	2.19E-03	0.043	0.013	1.04E-03
3.449571	0.029	0.009	1.33E-03	0.037	0.013	5.81E-03
3.097999	-0.031	0.009	8.15E-04	-0.035	0.014	1.29E-02
2.945931	-0.015	0.005	1.60E-03	-0.005	0.007	4.80E-01
2.949968	-0.016	0.005	1.37E-03	-0.005	0.007	5.35E-01
2.244469293	0.057	0.016	4.71E-04	0.034	0.024	1.64E-01
3.524595	0.027	0.009	2.60E-03	0.035	0.014	1.08E-02
1.386566185	0.027	0.008	3.11E-04	0.016	0.011	1.64E-01
1.372436017	0.055	0.016	5.41E-04	0.028	0.024	2.36E-01
1.374791045	0.052	0.015	4.26E-04	0.030	0.022	1.77E-01
5.373965	0.033	0.009	5.28E-04	0.023	0.014	9.60E-02
1.593135796	0.065	0.018	3.84E-04	0.030	0.027	2.64E-01
1.595154392	0.059	0.017	4.08E-04	0.025	0.025	3.06E-01
3.714007	0.025	0.008	2.08E-03	0.038	0.012	1.94E-03
1.590780768	0.070	0.020	4.38E-04	0.034	0.030	2.51E-01
3.853963	0.024	0.008	2.56E-03	0.033	0.012	6.43E-03
5.421739	0.023	0.008	2.73E-03	0.012	0.012	3.12E-01
0.895711035	0.044	0.013	5.61E-04	0.012	0.019	5.32E-01
1.275879863	0.068	0.019	4.30E-04	0.029	0.029	3.12E-01
0.892683142	0.054	0.016	5.53E-04	0.026	0.023	2.70E-01
3.019274	-0.013	0.004	2.40E-03	-0.006	0.006	2.98E-01
0.925317103	-0.013	0.004	2.23E-03	-0.026	0.006	4.04E-05
5.368919	0.033	0.010	8.29E-04	0.018	0.015	2.13E-01
5.193637	0.066	0.019	5.41E-04	0.023	0.028	4.25E-01
5.181862	0.044	0.013	5.43E-04	0.019	0.019	3.11E-01
2.107204796	-0.019	0.006	3.54E-03	-0.019	0.010	4.84E-02
2.250861512	0.037	0.010	4.55E-04	0.012	0.016	4.26E-01
2.909933	-0.029	0.008	5.92E-04	-0.013	0.013	2.98E-01
1.38320186	0.036	0.010	3.46E-04	0.022	0.015	1.47E-01
1.592462931	0.066	0.019	4.22E-04	0.033	0.028	2.35E-01
3.715016	0.025	0.008	2.09E-03	0.034	0.012	4.85E-03
1.380510399	0.040	0.011	4.09E-04	0.024	0.017	1.54E-01
2.106868363	-0.020	0.007	3.64E-03	-0.019	0.010	5.73E-02
3.021292	-0.013	0.004	1.71E-03	-0.009	0.006	1.59E-01
7.497191	0.019	0.006	1.05E-03	0.022	0.009	1.12E-02
0.886627355	0.075	0.021	5.16E-04	0.039	0.032	2.22E-01
1.591117201	0.069	0.020	4.52E-04	0.034	0.029	2.52E-01

1.270160509	0.071	0.020	5.27E-04	0.031	0.030	3.07E-01
0.886290922	0.075	0.022	5.23E-04	0.040	0.032	2.11E-01
1.37411818	0.053	0.015	5.21E-04	0.035	0.023	1.23E-01
3.921922	0.036	0.013	3.77E-03	0.061	0.019	1.11E-03
1.375800343	0.049	0.014	5.06E-04	0.030	0.021	1.60E-01
2.922717	-0.020	0.006	1.36E-03	-0.016	0.009	9.71E-02
3.429385	0.026	0.008	2.26E-03	0.035	0.013	5.10E-03
4.081055	0.069	0.020	3.97E-04	0.043	0.029	1.43E-01
1.600537313	0.046	0.013	4.74E-04	0.019	0.019	3.25E-01
7.883416	-0.033	0.009	4.34E-04	0.003	0.014	8.10E-01
3.397424	0.026	0.009	2.54E-03	0.034	0.013	7.96E-03
1.276216296	0.068	0.019	4.53E-04	0.029	0.029	3.14E-01
0.885618057	0.075	0.022	5.52E-04	0.042	0.032	1.97E-01
0.697552239	0.052	0.015	5.12E-04	0.014	0.022	5.41E-01
0.88595449	0.075	0.022	5.40E-04	0.041	0.032	2.08E-01
3.922259	0.036	0.012	4.08E-03	0.061	0.019	9.47E-04
4.079709	0.068	0.019	4.47E-04	0.043	0.029	1.36E-01
1.590444336	0.070	0.020	4.97E-04	0.034	0.030	2.50E-01
1.37546391	0.050	0.014	5.38E-04	0.029	0.022	1.82E-01
1.58842574	0.073	0.021	4.99E-04	0.036	0.031	2.52E-01
3.142071	-0.030	0.009	1.20E-03	-0.024	0.014	8.55E-02
2.115952043	-0.020	0.007	2.30E-03	-0.003	0.010	7.23E-01
3.830413	0.026	0.008	2.24E-03	0.036	0.013	3.88E-03
3.77591	0.030	0.010	1.91E-03	0.040	0.014	4.58E-03
1.375127477	0.051	0.015	4.86E-04	0.029	0.022	1.80E-01
3.778265	0.025	0.008	1.77E-03	0.036	0.012	2.85E-03
3.888279	0.024	0.008	2.21E-03	0.040	0.012	6.84E-04
0.920607047	-0.013	0.004	1.33E-03	-0.021	0.006	7.63E-04
1.592799364	0.065	0.018	4.46E-04	0.031	0.028	2.57E-01
1.594481527	0.061	0.017	4.05E-04	0.028	0.026	2.69E-01
2.123689993	-0.022	0.007	1.16E-03	-0.011	0.010	2.98E-01
1.590107903	0.071	0.020	4.72E-04	0.036	0.030	2.32E-01
1.377818938	0.046	0.013	4.30E-04	0.025	0.020	1.96E-01
5.372283	0.029	0.009	9.57E-04	0.013	0.013	3.02E-01
6.613383	0.030	0.010	3.29E-03	0.014	0.015	3.56E-01
3.080504	-0.018	0.006	1.74E-03	-0.004	0.008	6.47E-01
2.119652802	-0.022	0.007	1.52E-03	-0.010	0.010	3.19E-01
3.398433	0.024	0.009	4.44E-03	0.034	0.013	8.23E-03
1.592126499	0.066	0.019	4.54E-04	0.034	0.028	2.29E-01
7.230064	-0.033	0.011	2.53E-03	-0.022	0.016	1.84E-01
2.898831	-0.018	0.006	2.31E-03	-0.017	0.009	5.91E-02
0.886963788	0.074	0.021	5.58E-04	0.038	0.032	2.28E-01
2.631703205	-0.024	0.008	1.84E-03	-0.014	0.012	2.20E-01

1.276552728	0.067	0.019	4.80E-04	0.029	0.029	3.19E-01
2.953333	-0.014	0.005	2.34E-03	-0.003	0.007	6.22E-01
1.59750942	0.053	0.015	4.17E-04	0.024	0.022	2.79E-01
1.589771471	0.071	0.020	4.89E-04	0.036	0.030	2.39E-01
5.194647	0.063	0.018	6.09E-04	0.021	0.027	4.38E-01
3.088579	-0.019	0.006	1.26E-03	-0.010	0.009	2.84E-01
1.394304135	0.024	0.007	4.11E-04	0.018	0.010	7.40E-02
2.946941	-0.015	0.005	2.34E-03	-0.005	0.007	4.75E-01
0.885281625	0.074	0.022	6.01E-04	0.042	0.032	1.93E-01
2.965108	-0.014	0.005	1.54E-03	-0.011	0.007	1.12E-01
5.397179	0.025	0.008	1.99E-03	-0.003	0.012	7.98E-01
2.240432102	0.077	0.022	5.28E-04	0.044	0.033	1.87E-01
2.951651	-0.015	0.005	1.63E-03	-0.005	0.007	4.94E-01
5.37262	0.029	0.009	1.13E-03	0.001	0.013	9.27E-01
3.020956	-0.013	0.004	1.96E-03	-0.009	0.006	1.36E-01
1.269824076	0.070	0.020	5.99E-04	0.031	0.030	3.13E-01
5.231318	0.024	0.008	4.49E-03	0.025	0.013	4.85E-02
1.588762173	0.072	0.021	5.31E-04	0.037	0.031	2.37E-01
3.430731	0.025	0.008	3.41E-03	0.034	0.013	6.25E-03
2.899167	-0.019	0.006	1.80E-03	-0.014	0.009	1.09E-01
1.597172988	0.054	0.015	4.23E-04	0.026	0.023	2.49E-01
1.597845853	0.051	0.015	5.07E-04	0.020	0.022	3.53E-01
5.422075	0.026	0.008	1.62E-03	0.008	0.012	5.39E-01
6.25811	0.020	0.007	4.87E-03	0.014	0.011	1.80E-01
3.412563	0.026	0.008	2.49E-03	0.038	0.013	2.60E-03
2.116288476	-0.020	0.006	1.81E-03	-0.002	0.010	8.76E-01
2.89984	-0.019	0.006	1.78E-03	-0.010	0.009	2.65E-01
0.884945192	0.074	0.022	6.44E-04	0.043	0.032	1.88E-01
1.387575483	0.026	0.007	3.92E-04	0.014	0.011	1.83E-01
2.949632	-0.016	0.005	1.67E-03	-0.006	0.007	4.06E-01
3.832431	0.026	0.009	2.86E-03	0.039	0.013	2.13E-03
0.88730022	0.073	0.021	5.86E-04	0.038	0.032	2.31E-01
1.371426719	0.055	0.016	6.36E-04	0.032	0.024	1.88E-01
1.387911916	0.026	0.007	4.45E-04	0.013	0.011	2.37E-01
3.545454	0.035	0.010	8.88E-04	0.034	0.016	2.82E-02
1.389594079	0.026	0.008	5.69E-04	0.014	0.011	2.28E-01
1.588089308	0.072	0.021	5.59E-04	0.036	0.031	2.52E-01
3.531324	0.025	0.009	3.81E-03	0.035	0.013	7.32E-03
3.739576	0.026	0.008	2.48E-03	0.039	0.013	2.28E-03
3.021629	-0.013	0.004	1.73E-03	-0.009	0.006	1.60E-01
1.276889161	0.066	0.019	5.25E-04	0.028	0.028	3.23E-01
8.134731	0.018	0.006	5.13E-03	0.004	0.010	7.13E-01
3.830076	0.026	0.008	2.46E-03	0.036	0.013	3.83E-03

0.925653536	-0.013	0.004	2.44E-03	-0.026	0.006	7.71E-05
2.908587	-0.027	0.008	9.09E-04	-0.018	0.012	1.36E-01
3.25915	0.028	0.009	1.26E-03	0.041	0.013	1.63E-03
3.477831	0.028	0.010	4.00E-03	0.041	0.015	4.79E-03
3.091943	-0.023	0.007	1.16E-03	-0.017	0.010	1.09E-01
0.887636653	0.072	0.021	5.94E-04	0.036	0.031	2.42E-01
3.831422	0.025	0.008	2.78E-03	0.038	0.012	2.36E-03
3.389686	0.027	0.009	4.15E-03	0.041	0.014	3.46E-03
1.384884023	0.031	0.009	5.51E-04	0.024	0.014	8.13E-02
1.601546611	0.044	0.012	4.74E-04	0.010	0.019	5.81E-01
2.899504	-0.019	0.006	1.74E-03	-0.010	0.009	2.96E-01
3.017591	-0.013	0.004	2.16E-03	-0.007	0.006	2.50E-01
1.17427722	0.014	0.004	3.24E-04	0.005	0.006	3.61E-01
1.389930511	0.026	0.008	5.45E-04	0.015	0.011	1.83E-01
3.810563	0.022	0.007	3.29E-03	0.031	0.011	5.36E-03
1.112373624	0.020	0.007	3.48E-03	0.003	0.010	7.52E-01
0.887973085	0.071	0.021	5.96E-04	0.036	0.031	2.48E-01
7.768356	-0.038	0.012	1.12E-03	-0.035	0.017	4.16E-02
1.389257646	0.026	0.008	5.94E-04	0.013	0.011	2.53E-01
5.464466	0.025	0.009	3.13E-03	0.009	0.013	4.92E-01
1.383538292	0.034	0.010	4.81E-04	0.024	0.015	1.06E-01
3.715353	0.025	0.008	2.45E-03	0.033	0.012	5.62E-03
0.884608759	0.073	0.022	6.92E-04	0.042	0.032	1.88E-01
3.835795	0.023	0.008	3.07E-03	0.035	0.012	3.21E-03
2.43623587	-0.024	0.009	5.03E-03	0.021	0.013	1.03E-01
3.529978	0.027	0.009	1.91E-03	0.034	0.013	8.73E-03
5.355125	0.051	0.015	9.59E-04	0.005	0.023	8.13E-01
2.240768534	0.075	0.022	5.85E-04	0.042	0.033	1.97E-01
5.709389	0.026	0.009	4.46E-03	0.007	0.013	6.19E-01
1.277225593	0.066	0.019	5.59E-04	0.028	0.028	3.25E-01
0.925989968	-0.013	0.004	2.79E-03	-0.025	0.006	8.55E-05
2.900176	-0.019	0.006	1.98E-03	-0.015	0.009	1.07E-01
5.229299	0.026	0.009	2.84E-03	0.040	0.013	1.72E-03
5.356134	0.053	0.016	9.22E-04	0.029	0.024	2.22E-01
3.244347	0.023	0.008	4.37E-03	0.037	0.012	1.95E-03
1.58708001	0.073	0.021	6.08E-04	0.035	0.032	2.65E-01
3.082523	-0.019	0.006	9.28E-04	-0.008	0.009	3.32E-01
1.600873746	0.044	0.013	5.21E-04	0.014	0.019	4.58E-01
1.594817959	0.059	0.017	4.90E-04	0.024	0.025	3.39E-01
3.540071	0.027	0.009	2.89E-03	0.034	0.013	1.04E-02
1.122130169	0.021	0.007	1.81E-03	0.006	0.010	5.27E-01
3.830749	0.025	0.008	2.86E-03	0.037	0.012	3.33E-03
3.244011	0.023	0.008	3.07E-03	0.040	0.012	6.51E-04

1.374454612	0.052	0.015	6.17E-04	0.033	0.023	1.39E-01
0.888309518	0.070	0.020	6.27E-04	0.034	0.030	2.65E-01
0.884272327	0.073	0.021	7.37E-04	0.042	0.032	1.92E-01
1.269487644	0.068	0.020	6.97E-04	0.030	0.030	3.22E-01
2.241104967	0.074	0.021	6.15E-04	0.044	0.032	1.73E-01
5.235691	0.025	0.008	3.06E-03	0.038	0.013	2.32E-03
3.70997	0.027	0.009	2.07E-03	0.037	0.013	4.40E-03
5.353443	0.056	0.017	1.02E-03	0.025	0.025	3.28E-01
3.462019	0.026	0.009	2.58E-03	0.038	0.013	3.63E-03
2.950978	-0.016	0.005	1.18E-03	-0.003	0.007	7.24E-01
2.697643993	-0.023	0.006	2.40E-04	-0.016	0.010	9.05E-02
1.585734279	0.074	0.022	6.65E-04	0.039	0.033	2.27E-01
2.924736	-0.020	0.006	1.80E-03	-0.016	0.010	1.05E-01
5.237374	0.024	0.008	5.05E-03	0.024	0.013	6.05E-02
1.585397847	0.075	0.022	6.60E-04	0.040	0.033	2.18E-01
0.892346709	0.055	0.016	7.17E-04	0.026	0.024	2.83E-01
3.415591	0.025	0.008	3.59E-03	0.038	0.013	2.44E-03
3.810227	0.022	0.008	3.39E-03	0.030	0.011	8.01E-03
3.456972	0.026	0.009	4.60E-03	0.039	0.014	3.77E-03
1.277562026	0.065	0.019	5.99E-04	0.027	0.028	3.30E-01
3.729483	0.026	0.009	3.98E-03	0.036	0.013	6.23E-03
7.510649	-0.026	0.009	3.17E-03	-0.011	0.013	4.02E-01
5.949938	0.026	0.008	2.09E-03	0.018	0.012	1.42E-01
3.098672	-0.037	0.011	9.60E-04	-0.036	0.017	2.94E-02
1.59885515	0.048	0.014	5.92E-04	0.014	0.021	4.92E-01
3.767836	0.031	0.010	1.23E-03	0.037	0.014	1.14E-02
2.960061	-0.014	0.005	2.16E-03	-0.010	0.007	1.47E-01
7.874332	0.024	0.008	1.60E-03	0.017	0.011	1.26E-01
3.097662	-0.029	0.009	8.75E-04	-0.031	0.013	1.71E-02
3.406507	0.026	0.009	4.45E-03	0.040	0.014	3.95E-03
5.19431	0.062	0.018	7.77E-04	0.022	0.027	4.21E-01
1.388584781	0.025	0.007	6.42E-04	0.012	0.011	2.84E-01
1.587416442	0.072	0.021	6.49E-04	0.033	0.032	3.00E-01
5.949601	0.031	0.010	2.27E-03	0.004	0.015	7.72E-01
3.698867	0.024	0.008	2.65E-03	0.033	0.012	5.37E-03
2.47526205	-0.034	0.010	8.38E-04	-0.005	0.015	7.50E-01
3.96364	-0.026	0.008	2.00E-03	-0.001	0.012	9.11E-01
1.587752875	0.072	0.021	6.50E-04	0.034	0.031	2.78E-01
3.769182	0.024	0.008	3.10E-03	0.029	0.012	1.97E-02
2.117634206	-0.023	0.007	1.64E-03	-0.007	0.011	5.35E-01
1.388248348	0.025	0.007	6.04E-04	0.012	0.011	2.81E-01
3.968013	-0.026	0.008	1.57E-03	-0.009	0.012	4.76E-01
2.697307561	-0.024	0.006	2.19E-04	-0.016	0.009	9.54E-02

3.472785	0.026	0.009	4.57E-03	0.040	0.013	2.71E-03
3.883569	0.024	0.008	2.48E-03	0.033	0.012	5.43E-03
2.122344262	-0.021	0.007	1.84E-03	-0.022	0.010	3.26E-02
0.962997553	0.013	0.004	2.48E-03	0.014	0.006	2.80E-02
3.000433	-0.012	0.004	4.14E-03	0.001	0.006	8.40E-01
6.670576	0.032	0.009	7.25E-04	0.021	0.014	1.34E-01
7.497864	0.024	0.008	3.84E-03	0.004	0.012	7.57E-01
3.837478	0.024	0.008	3.19E-03	0.036	0.012	2.59E-03
1.583715684	0.077	0.023	7.28E-04	0.040	0.034	2.35E-01
2.946604	-0.015	0.005	2.39E-03	-0.005	0.007	4.56E-01
5.193974	0.063	0.019	7.99E-04	0.024	0.028	3.85E-01
2.239422804	0.077	0.023	7.48E-04	0.040	0.034	2.37E-01
3.884578	0.023	0.008	3.34E-03	0.039	0.012	7.85E-04
3.831085	0.024	0.008	3.25E-03	0.038	0.012	2.49E-03
1.277898459	0.064	0.019	6.42E-04	0.027	0.028	3.33E-01
3.907456	0.026	0.009	2.80E-03	0.038	0.013	3.46E-03
2.116961341	-0.021	0.007	2.18E-03	0.001	0.010	9.50E-01
3.257804	0.024	0.009	6.35E-03	0.031	0.013	2.08E-02
2.240095669	0.076	0.022	7.06E-04	0.044	0.033	1.92E-01
5.210795	0.026	0.008	1.64E-03	-0.002	0.012	8.47E-01
1.586743577	0.072	0.021	7.01E-04	0.036	0.032	2.57E-01
1.371090286	0.054	0.016	7.63E-04	0.032	0.024	1.91E-01
3.290102	-0.053	0.017	2.15E-03	-0.065	0.026	1.15E-02
1.379164668	0.043	0.012	6.28E-04	0.027	0.019	1.51E-01
1.379501101	0.042	0.012	5.94E-04	0.029	0.018	1.15E-01
3.097326	-0.028	0.008	6.58E-04	-0.029	0.012	2.00E-02
3.243338	0.025	0.008	2.24E-03	0.040	0.012	1.15E-03
3.099345	-0.042	0.013	1.46E-03	-0.048	0.019	1.46E-02
3.71367	0.025	0.008	3.10E-03	0.039	0.012	1.61E-03
0.883935894	0.071	0.021	8.40E-04	0.041	0.032	1.95E-01
7.767347	-0.032	0.009	7.72E-04	-0.026	0.014	6.46E-02
3.244683	0.023	0.008	5.54E-03	0.034	0.013	6.47E-03
3.243674	0.024	0.008	2.44E-03	0.041	0.012	6.83E-04
5.190946	0.064	0.019	8.43E-04	0.023	0.029	4.25E-01
3.611395	-0.026	0.008	2.19E-03	0.006	0.013	6.27E-01
3.810899	0.021	0.007	4.22E-03	0.032	0.011	3.79E-03
3.079831	-0.018	0.006	2.13E-03	-0.004	0.009	6.51E-01
3.142408	-0.030	0.009	1.38E-03	-0.027	0.014	5.45E-02
1.583042819	0.078	0.023	7.54E-04	0.041	0.034	2.38E-01
3.849253	0.025	0.009	2.81E-03	0.034	0.013	7.50E-03
3.822338	0.022	0.008	3.32E-03	0.033	0.011	3.51E-03
3.01961	-0.012	0.004	3.08E-03	-0.005	0.006	3.90E-01
3.827721	0.023	0.008	3.13E-03	0.036	0.012	2.43E-03

1.585061414	0.075	0.022	7.48E-04	0.039	0.033	2.41E-01
0.888645951	0.068	0.020	7.10E-04	0.033	0.030	2.67E-01
2.251197945	0.035	0.010	6.70E-04	0.012	0.015	4.21E-01
1.269151211	0.067	0.020	8.12E-04	0.029	0.030	3.34E-01
2.107541228	-0.018	0.006	4.36E-03	-0.020	0.009	2.66E-02
1.121793736	0.021	0.007	2.27E-03	0.004	0.010	6.94E-01
1.600200881	0.045	0.013	6.27E-04	0.024	0.020	2.29E-01
0.926662833	-0.013	0.004	3.69E-03	-0.026	0.007	8.85E-05
6.154152	0.024	0.008	1.36E-03	0.021	0.011	5.66E-02
0.70293516	0.027	0.008	1.20E-03	0.024	0.013	5.41E-02
3.730492	0.026	0.009	3.06E-03	0.035	0.013	6.97E-03
1.586070712	0.073	0.022	7.46E-04	0.039	0.032	2.31E-01
2.951314	-0.015	0.005	1.36E-03	-0.005	0.007	4.96E-01
1.602892342	0.040	0.011	4.50E-04	0.016	0.017	3.45E-01
2.238749939	0.077	0.023	8.35E-04	0.039	0.034	2.58E-01
3.850598	0.024	0.008	4.25E-03	0.036	0.013	4.20E-03
1.382528994	0.036	0.010	5.15E-04	0.020	0.015	1.86E-01
3.540408	0.025	0.009	6.71E-03	0.037	0.014	6.92E-03
2.400574015	-0.026	0.010	6.32E-03	0.002	0.014	9.03E-01
1.278234891	0.064	0.019	6.94E-04	0.027	0.028	3.36E-01
3.099008	-0.039	0.012	1.20E-03	-0.046	0.018	1.14E-02
3.822002	0.022	0.008	3.56E-03	0.033	0.011	3.61E-03
1.583379251	0.077	0.023	7.83E-04	0.041	0.034	2.32E-01
2.239759237	0.076	0.023	7.76E-04	0.042	0.034	2.12E-01
7.797289	-0.031	0.009	9.16E-04	-0.016	0.014	2.50E-01
1.584052116	0.076	0.023	7.75E-04	0.038	0.034	2.58E-01
3.827385	0.023	0.008	3.14E-03	0.036	0.012	2.27E-03
3.075458	-0.019	0.006	2.90E-03	-0.026	0.010	7.23E-03
3.828057	0.023	0.008	3.38E-03	0.036	0.012	2.50E-03
0.896047468	0.042	0.013	8.35E-04	0.010	0.019	6.12E-01
2.239086371	0.077	0.023	8.27E-04	0.038	0.034	2.63E-01
2.628675312	-0.024	0.008	4.50E-03	-0.021	0.013	9.01E-02
4.081391	0.066	0.019	6.11E-04	0.041	0.029	1.60E-01
3.017255	-0.013	0.004	2.23E-03	-0.007	0.006	2.97E-01
3.739239	0.025	0.009	4.00E-03	0.040	0.013	1.85E-03
3.490616	0.027	0.009	2.92E-03	0.040	0.014	3.46E-03
6.087202	0.030	0.011	4.09E-03	0.026	0.016	1.02E-01
3.45529	0.028	0.009	2.31E-03	0.035	0.014	1.11E-02
2.482327135	-0.034	0.010	7.71E-04	-0.009	0.015	5.51E-01
0.892010276	0.055	0.017	8.31E-04	0.026	0.025	2.89E-01
0.920270614	-0.013	0.004	1.66E-03	-0.021	0.006	9.63E-04
3.022974	-0.014	0.004	1.80E-03	-0.009	0.007	1.82E-01
5.185227	0.050	0.015	8.54E-04	0.012	0.022	5.78E-01

2.411003425	-0.035	0.011	2.17E-03	-0.009	0.017	5.93E-01
3.698531	0.023	0.008	3.34E-03	0.034	0.012	3.92E-03
1.391949107	0.024	0.007	6.99E-04	0.017	0.011	1.12E-01
3.098335	-0.033	0.010	1.33E-03	-0.034	0.015	2.65E-02
1.386902618	0.026	0.007	5.49E-04	0.014	0.011	2.00E-01
2.697980426	-0.023	0.006	3.11E-04	-0.016	0.010	9.36E-02
3.091607	-0.022	0.007	1.32E-03	-0.017	0.010	8.62E-02
2.928773	-0.020	0.006	1.88E-03	-0.019	0.009	4.00E-02
2.942567	-0.015	0.005	2.40E-03	-0.008	0.007	2.86E-01
1.39363127	0.024	0.007	5.62E-04	0.016	0.010	1.18E-01
1.278571324	0.063	0.019	7.38E-04	0.027	0.028	3.39E-01
3.245356	0.025	0.008	2.76E-03	0.036	0.013	4.10E-03
0.888982383	0.066	0.020	7.78E-04	0.032	0.029	2.70E-01
3.493307	0.027	0.010	4.88E-03	0.039	0.015	6.64E-03
1.586407145	0.072	0.021	8.33E-04	0.037	0.032	2.54E-01
3.421647	0.025	0.009	4.48E-03	0.039	0.013	3.63E-03
2.911279	-0.025	0.008	1.70E-03	-0.006	0.012	6.01E-01
3.834113	0.023	0.008	4.24E-03	0.038	0.012	1.84E-03
1.580687791	0.079	0.024	8.88E-04	0.041	0.036	2.45E-01
2.959052	-0.014	0.005	2.92E-03	-0.010	0.007	1.32E-01
0.883599462	0.070	0.021	9.65E-04	0.041	0.032	1.97E-01
3.447552	0.024	0.009	5.45E-03	0.038	0.013	3.15E-03
0.963333986	0.013	0.004	2.47E-03	0.013	0.007	5.69E-02
3.471775	0.027	0.009	2.74E-03	0.037	0.013	5.33E-03
0.891673844	0.057	0.017	8.56E-04	0.027	0.025	2.88E-01
3.24502	0.024	0.009	4.87E-03	0.033	0.013	9.49E-03
2.893784	-0.018	0.006	2.91E-03	-0.016	0.009	8.08E-02
0.924307805	-0.013	0.004	3.23E-03	-0.026	0.006	5.18E-05
3.826712	0.022	0.008	3.33E-03	0.034	0.011	2.82E-03
1.581360656	0.079	0.024	9.09E-04	0.043	0.035	2.21E-01
5.192965	0.065	0.020	1.00E-03	0.026	0.030	3.89E-01
1.601883044	0.042	0.012	6.11E-04	0.011	0.018	5.47E-01
3.083196	-0.019	0.006	1.69E-03	-0.006	0.009	5.00E-01
1.382865427	0.035	0.010	5.30E-04	0.022	0.015	1.48E-01
1.582033521	0.078	0.023	8.96E-04	0.042	0.035	2.36E-01
2.413358454	-0.026	0.009	4.20E-03	0.014	0.014	2.94E-01
3.080841	-0.018	0.006	2.04E-03	-0.008	0.008	3.46E-01
0.932382187	-0.015	0.005	2.18E-03	-0.009	0.007	1.87E-01
0.924644238	-0.013	0.004	3.26E-03	-0.027	0.006	2.45E-05
3.431067	0.024	0.009	4.95E-03	0.035	0.013	6.53E-03
2.927427	-0.021	0.006	1.42E-03	-0.022	0.010	2.09E-02
3.458991	0.027	0.009	2.73E-03	0.036	0.013	7.26E-03
3.015909	-0.013	0.004	1.69E-03	-0.005	0.006	4.30E-01

3.46101	0.025	0.009	6.03E-03	0.040	0.013	2.49E-03
0.93271862	-0.015	0.005	1.99E-03	-0.011	0.007	1.32E-01
3.020619	-0.012	0.004	2.76E-03	-0.008	0.006	2.17E-01
5.357144	0.040	0.012	1.28E-03	0.006	0.019	7.54E-01
1.599191583	0.047	0.014	6.97E-04	0.017	0.021	4.02E-01
5.350751	0.056	0.018	1.37E-03	0.024	0.026	3.68E-01
1.278907756	0.062	0.019	7.93E-04	0.026	0.028	3.42E-01
3.827048	0.023	0.008	3.37E-03	0.035	0.012	2.64E-03
0.889318816	0.065	0.019	8.25E-04	0.032	0.029	2.73E-01
6.613719	0.025	0.010	1.06E-02	0.002	0.015	8.83E-01
3.71939	0.026	0.008	2.48E-03	0.033	0.013	1.04E-02
5.367573	0.033	0.010	1.39E-03	0.023	0.015	1.39E-01
1.379837534	0.041	0.012	7.07E-04	0.027	0.018	1.36E-01
1.603228774	0.038	0.011	6.87E-04	0.016	0.017	3.30E-01
1.581697088	0.078	0.024	9.60E-04	0.042	0.035	2.30E-01
1.581024223	0.079	0.024	9.51E-04	0.042	0.035	2.39E-01
1.268814779	0.065	0.020	9.72E-04	0.028	0.030	3.46E-01
0.92498067	-0.013	0.004	3.30E-03	-0.027	0.006	3.03E-05
1.584724982	0.074	0.022	8.95E-04	0.038	0.033	2.60E-01
1.582706386	0.077	0.023	9.08E-04	0.040	0.035	2.48E-01
1.395649865	0.022	0.007	9.64E-04	0.017	0.010	8.04E-02
3.822675	0.022	0.008	3.88E-03	0.034	0.011	2.79E-03
2.136810864	-0.029	0.009	1.16E-03	-0.007	0.013	6.14E-01
1.109009298	0.018	0.006	2.57E-03	0.014	0.009	1.15E-01
3.903755	0.026	0.009	3.25E-03	0.034	0.013	8.87E-03
0.926326401	-0.013	0.004	4.01E-03	-0.026	0.006	7.23E-05
2.950641	-0.015	0.005	1.87E-03	-0.001	0.007	8.75E-01
8.20841	0.020	0.007	5.24E-03	0.009	0.011	3.77E-01
0.928681429	-0.013	0.005	5.75E-03	-0.026	0.007	1.17E-04
5.356471	0.051	0.016	1.28E-03	0.023	0.023	3.24E-01
4.079036	0.065	0.020	8.33E-04	0.043	0.029	1.39E-01
3.832768	0.025	0.009	4.60E-03	0.041	0.013	1.68E-03
2.238413506	0.076	0.023	1.02E-03	0.038	0.034	2.74E-01
5.375647	0.029	0.009	1.85E-03	0.016	0.014	2.36E-01
3.968686	-0.024	0.008	2.44E-03	-0.013	0.012	2.92E-01
5.389778	0.025	0.008	2.36E-03	0.019	0.012	1.25E-01
3.86742	-0.014	0.005	6.60E-03	0.001	0.007	9.08E-01
3.909138	0.026	0.009	5.04E-03	0.043	0.014	1.71E-03
0.891337411	0.058	0.017	9.04E-04	0.028	0.026	2.86E-01
3.828394	0.023	0.008	4.24E-03	0.036	0.012	2.23E-03
3.083532	-0.018	0.006	2.22E-03	-0.001	0.009	8.73E-01
7.304415	-0.031	0.010	1.28E-03	-0.031	0.014	3.15E-02
2.93382	-0.017	0.006	1.98E-03	-0.010	0.008	2.11E-01

0.963670418	0.014	0.005	2.55E-03	0.012	0.007	7.76E-02
1.095888427	0.021	0.007	4.35E-03	-0.002	0.011	8.24E-01
2.237404208	0.076	0.023	9.55E-04	0.035	0.034	3.13E-01
0.699907267	0.036	0.011	9.89E-04	0.024	0.016	1.40E-01
2.238077074	0.076	0.023	9.84E-04	0.037	0.035	2.88E-01
2.926082	-0.021	0.007	1.38E-03	-0.028	0.010	3.98E-03
5.498782	0.016	0.005	3.36E-03	-0.002	0.008	7.96E-01
1.279244189	0.062	0.019	8.64E-04	0.026	0.028	3.48E-01
1.582369954	0.077	0.023	9.71E-04	0.040	0.035	2.52E-01
3.395742	0.023	0.008	6.06E-03	0.038	0.013	2.51E-03
8.104116	0.024	0.008	3.78E-03	0.008	0.012	5.21E-01
3.961285	-0.026	0.008	1.77E-03	-0.017	0.012	1.59E-01
1.602555909	0.040	0.012	6.11E-04	0.014	0.017	4.08E-01
3.016919	-0.013	0.004	2.52E-03	-0.006	0.006	3.47E-01
3.082859	-0.019	0.006	1.32E-03	-0.009	0.009	3.11E-01
2.229329826	0.084	0.026	1.11E-03	0.048	0.039	2.15E-01
3.905437	0.024	0.009	4.96E-03	0.041	0.013	1.26E-03
1.584388549	0.074	0.022	9.52E-04	0.037	0.034	2.65E-01
1.370753854	0.053	0.016	1.04E-03	0.032	0.024	1.92E-01
0.883263029	0.069	0.021	1.12E-03	0.040	0.031	2.00E-01
3.80989	0.021	0.008	4.46E-03	0.028	0.011	1.19E-02
3.415928	0.024	0.008	5.11E-03	0.038	0.013	2.27E-03
0.926999266	-0.012	0.004	5.03E-03	-0.026	0.007	9.01E-05
1.204892586	0.015	0.005	4.44E-03	0.011	0.008	1.67E-01
2.228993394	0.085	0.026	1.14E-03	0.048	0.039	2.18E-01
3.079495	-0.017	0.006	2.65E-03	-0.006	0.009	4.53E-01
2.630357475	-0.023	0.008	2.47E-03	-0.010	0.011	3.78E-01
2.952996	-0.014	0.005	3.58E-03	-0.006	0.007	4.29E-01
0.889655248	0.063	0.019	9.13E-04	0.031	0.028	2.75E-01
3.524932	0.025	0.009	4.83E-03	0.033	0.013	1.19E-02
2.237740641	0.076	0.023	9.87E-04	0.036	0.035	2.91E-01
0.889991681	0.062	0.019	9.13E-04	0.030	0.028	2.77E-01
1.580014925	0.079	0.024	1.03E-03	0.040	0.036	2.67E-01
3.243001	0.024	0.008	4.31E-03	0.035	0.013	6.14E-03
7.795943	-0.031	0.010	1.93E-03	-0.013	0.015	3.85E-01
2.471897724	-0.025	0.008	2.51E-03	-0.007	0.012	5.88E-01
3.699204	0.023	0.008	3.57E-03	0.031	0.012	8.55E-03
2.434553707	-0.022	0.008	4.88E-03	-0.015	0.012	1.87E-01
5.366227	0.040	0.013	1.90E-03	0.037	0.019	5.56E-02
2.465505505	-0.026	0.009	2.77E-03	-0.025	0.013	5.48E-02
5.193301	0.064	0.020	1.18E-03	0.023	0.030	4.38E-01
3.813591	0.021	0.008	4.99E-03	0.031	0.011	5.31E-03
2.953669	-0.014	0.005	2.50E-03	-0.007	0.007	3.26E-01

3.467402	0.026	0.010	6.62E-03	0.041	0.014	4.12E-03
1.364361634	0.062	0.019	1.15E-03	0.036	0.028	2.05E-01
7.859866	0.026	0.010	6.97E-03	0.012	0.014	3.90E-01
2.698316858	-0.023	0.006	3.51E-04	-0.017	0.010	7.95E-02
1.205901884	0.015	0.005	4.42E-03	0.012	0.008	1.50E-01
0.891000979	0.059	0.018	9.49E-04	0.028	0.026	2.83E-01
2.632039638	-0.023	0.008	3.46E-03	-0.014	0.012	2.48E-01
2.954006	-0.014	0.005	2.47E-03	-0.009	0.007	1.76E-01
1.580351358	0.078	0.024	1.05E-03	0.040	0.036	2.66E-01
1.579678493	0.079	0.024	1.03E-03	0.041	0.036	2.58E-01
3.075794	-0.019	0.006	2.75E-03	-0.020	0.010	3.28E-02
1.395313433	0.022	0.007	9.58E-04	0.016	0.010	1.08E-01
3.813927	0.021	0.008	4.67E-03	0.032	0.011	4.67E-03
3.022638	-0.013	0.004	2.24E-03	-0.008	0.006	2.17E-01
0.890328114	0.061	0.018	9.52E-04	0.029	0.027	2.85E-01
3.451253	0.025	0.009	6.25E-03	0.040	0.014	4.12E-03
2.12469929	-0.025	0.008	1.48E-03	-0.003	0.012	8.07E-01
2.881	-0.020	0.007	5.47E-03	0.000	0.011	9.87E-01
1.599864448	0.045	0.013	8.16E-04	0.023	0.020	2.49E-01
3.523586	0.026	0.009	2.93E-03	0.034	0.013	9.87E-03
2.106531931	-0.018	0.007	8.14E-03	-0.017	0.010	9.36E-02
2.237067776	0.075	0.023	1.06E-03	0.035	0.034	3.09E-01
3.092616	-0.023	0.007	1.85E-03	-0.014	0.011	2.15E-01
5.190609	0.064	0.019	9.88E-04	0.021	0.029	4.63E-01
7.051418	-0.022	0.007	3.27E-03	-0.022	0.011	4.50E-02
1.393294837	0.023	0.007	6.82E-04	0.018	0.010	8.37E-02
3.852954	0.023	0.008	4.48E-03	0.033	0.012	5.60E-03
1.107663567	0.018	0.006	2.67E-03	0.008	0.009	3.79E-01
3.429048	0.025	0.008	3.76E-03	0.034	0.013	6.71E-03
3.463701	0.025	0.009	6.82E-03	0.040	0.014	2.93E-03
3.715689	0.023	0.008	3.77E-03	0.032	0.012	8.99E-03
0.928344996	-0.012	0.005	6.06E-03	-0.026	0.007	1.56E-04
2.44666528	-0.024	0.009	6.90E-03	-0.015	0.013	2.47E-01
5.846317	0.024	0.008	4.89E-03	0.013	0.013	2.87E-01
3.854299	0.023	0.008	5.59E-03	0.035	0.012	4.37E-03
1.366043797	0.059	0.018	1.21E-03	0.030	0.027	2.70E-01
1.279580621	0.061	0.018	9.66E-04	0.026	0.028	3.55E-01
2.698653291	-0.023	0.006	3.75E-04	-0.017	0.010	7.25E-02
1.205229019	0.015	0.005	4.71E-03	0.011	0.008	1.45E-01
5.179844	0.042	0.013	9.35E-04	0.020	0.019	2.88E-01
2.471561292	-0.025	0.008	2.54E-03	-0.005	0.012	6.78E-01
2.227984096	0.085	0.026	1.26E-03	0.046	0.039	2.36E-01
3.821665	0.021	0.008	4.55E-03	0.034	0.011	2.82E-03

4.081728	0.062	0.018	7.94E-04	0.035	0.028	2.00E-01
2.228320529	0.084	0.026	1.26E-03	0.046	0.039	2.40E-01
3.53166	0.024	0.008	5.44E-03	0.033	0.013	8.43E-03
1.061572302	0.014	0.005	1.02E-02	-0.006	0.008	4.86E-01
3.009517	-0.012	0.004	1.71E-03	-0.004	0.006	5.31E-01
3.962967	-0.025	0.009	3.85E-03	-0.011	0.013	3.96E-01
2.229666259	0.083	0.026	1.23E-03	0.046	0.038	2.25E-01
3.143417	-0.031	0.010	2.18E-03	-0.033	0.015	2.79E-02
3.82974	0.024	0.008	4.48E-03	0.037	0.012	3.17E-03
3.833777	0.023	0.008	5.45E-03	0.037	0.012	2.56E-03
7.68122	0.023	0.009	9.86E-03	0.001	0.013	9.12E-01
3.83445	0.023	0.008	5.27E-03	0.038	0.012	1.78E-03
0.890664546	0.060	0.018	9.94E-04	0.029	0.027	2.87E-01
1.380173966	0.039	0.012	8.05E-04	0.024	0.018	1.72E-01
0.962661121	0.013	0.004	3.28E-03	0.015	0.006	1.86E-02
2.907914	-0.025	0.008	1.27E-03	-0.021	0.011	6.83E-02
3.539735	0.027	0.009	2.23E-03	0.032	0.013	1.63E-02
5.374302	0.027	0.008	1.12E-03	0.028	0.012	2.04E-02
4.079373	0.068	0.021	9.88E-04	0.044	0.031	1.56E-01
1.599528016	0.046	0.014	8.22E-04	0.022	0.020	2.76E-01
1.268478346	0.064	0.020	1.18E-03	0.027	0.029	3.60E-01
2.925072	-0.020	0.006	1.63E-03	-0.021	0.009	2.29E-02
3.00077	-0.011	0.004	5.61E-03	0.002	0.006	7.22E-01
5.350415	0.055	0.018	1.72E-03	0.028	0.026	2.84E-01
3.099681	-0.042	0.014	2.38E-03	-0.046	0.021	2.63E-02
2.475934916	-0.033	0.010	9.54E-04	-0.008	0.015	5.71E-01
0.882926597	0.067	0.021	1.27E-03	0.040	0.031	2.02E-01
1.361333741	0.064	0.020	1.43E-03	0.038	0.030	2.03E-01
2.230002691	0.082	0.025	1.26E-03	0.045	0.038	2.39E-01
1.364025202	0.062	0.019	1.27E-03	0.036	0.029	2.14E-01
5.189264	0.060	0.018	9.65E-04	0.016	0.027	5.60E-01
3.826375	0.022	0.008	4.24E-03	0.034	0.011	2.55E-03
2.9244	-0.019	0.006	2.59E-03	-0.007	0.010	4.49E-01
2.228656961	0.084	0.026	1.30E-03	0.046	0.039	2.35E-01
5.179507	0.044	0.013	1.01E-03	0.018	0.020	3.79E-01
3.708624	0.024	0.008	5.06E-03	0.033	0.013	7.89E-03
0.8963839	0.040	0.012	1.12E-03	0.008	0.018	6.60E-01
5.355461	0.046	0.015	1.73E-03	0.011	0.022	6.00E-01
2.925409	-0.020	0.006	1.41E-03	-0.024	0.009	1.15E-02
1.602219476	0.040	0.012	8.12E-04	0.011	0.018	5.23E-01
2.894121	-0.017	0.006	3.97E-03	-0.013	0.009	1.43E-01
3.963976	-0.025	0.008	2.28E-03	0.003	0.012	8.33E-01
3.259486	0.026	0.009	2.42E-03	0.040	0.013	1.92E-03

1.363352337	0.063	0.020	1.39E-03	0.035	0.029	2.30E-01
2.897149	-0.018	0.006	6.27E-03	-0.022	0.010	2.27E-02
1.279917054	0.060	0.018	1.04E-03	0.025	0.028	3.62E-01
2.881336	-0.020	0.007	6.72E-03	0.000	0.011	9.98E-01
3.146109	-0.065	0.021	1.79E-03	-0.091	0.031	3.71E-03
2.227647663	0.084	0.026	1.37E-03	0.045	0.039	2.46E-01
1.360997309	0.064	0.020	1.47E-03	0.038	0.030	2.03E-01
1.3650345	0.060	0.019	1.28E-03	0.035	0.028	2.15E-01
7.04839	-0.019	0.007	5.80E-03	-0.023	0.010	2.38E-02
1.36638023	0.058	0.018	1.26E-03	0.030	0.027	2.64E-01
1.383874725	0.033	0.010	8.50E-04	0.020	0.015	1.62E-01
3.397087	0.024	0.009	4.77E-03	0.033	0.013	1.00E-02
2.136137999	-0.030	0.009	9.99E-04	-0.010	0.014	4.69E-01
1.578669195	0.078	0.024	1.16E-03	0.037	0.036	2.97E-01
0.694860778	0.051	0.017	2.21E-03	0.006	0.025	7.99E-01
5.228963	0.024	0.009	4.88E-03	0.043	0.013	8.90E-04
2.412349156	-0.027	0.010	4.92E-03	0.004	0.015	7.61E-01
1.395986298	0.021	0.007	1.04E-03	0.018	0.010	6.40E-02
1.207247614	0.016	0.006	4.85E-03	0.012	0.008	1.51E-01
3.245693	0.026	0.008	2.37E-03	0.042	0.013	1.04E-03
3.50811	0.025	0.009	6.11E-03	0.037	0.014	6.08E-03
2.630693908	-0.023	0.008	2.78E-03	-0.014	0.011	2.25E-01
3.023311	-0.014	0.005	1.87E-03	-0.011	0.007	1.17E-01
5.394488	0.026	0.009	4.09E-03	-0.013	0.014	3.44E-01
1.388921214	0.025	0.008	1.07E-03	0.013	0.011	2.60E-01
0.933055053	-0.014	0.005	2.66E-03	-0.013	0.007	6.29E-02
1.38454759	0.031	0.009	9.23E-04	0.022	0.014	1.18E-01
5.929415	0.024	0.009	5.99E-03	0.004	0.013	7.43E-01
1.204556154	0.014	0.005	5.11E-03	0.011	0.008	1.68E-01
5.235355	0.024	0.008	5.18E-03	0.041	0.013	1.14E-03
5.191282	0.064	0.020	1.38E-03	0.023	0.030	4.52E-01
0.928008564	-0.012	0.005	6.36E-03	-0.026	0.007	1.38E-04
1.578332762	0.078	0.024	1.23E-03	0.038	0.036	2.90E-01
1.579005628	0.078	0.024	1.21E-03	0.038	0.036	2.89E-01
2.963089	-0.013	0.005	3.50E-03	-0.005	0.007	4.31E-01
3.814264	0.021	0.008	5.35E-03	0.033	0.011	3.53E-03
3.270925	-0.035	0.014	1.04E-02	-0.023	0.020	2.57E-01
2.227311231	0.083	0.026	1.43E-03	0.045	0.039	2.49E-01
1.363688769	0.062	0.019	1.47E-03	0.035	0.029	2.20E-01
1.363015904	0.063	0.020	1.43E-03	0.033	0.029	2.63E-01
3.811236	0.020	0.007	6.75E-03	0.033	0.011	3.20E-03
1.577323465	0.077	0.024	1.31E-03	0.039	0.036	2.81E-01
3.609713	-0.020	0.007	4.31E-03	0.000	0.010	9.79E-01

2.489055787	-0.044	0.014	1.33E-03	-0.003	0.020	8.96E-01
0.882590164	0.066	0.020	1.38E-03	0.039	0.031	2.01E-01
3.478168	0.026	0.010	7.01E-03	0.040	0.014	5.30E-03
3.016246	-0.013	0.004	2.49E-03	-0.005	0.006	4.58E-01
1.364698067	0.060	0.019	1.36E-03	0.035	0.028	2.17E-01
5.844971	0.020	0.008	6.59E-03	0.008	0.011	4.54E-01
1.360660876	0.064	0.020	1.51E-03	0.039	0.030	2.01E-01
5.420393	0.019	0.007	4.95E-03	0.018	0.010	7.31E-02
1.205565451	0.015	0.005	5.25E-03	0.011	0.008	1.51E-01
3.835459	0.022	0.008	5.94E-03	0.036	0.012	2.14E-03
0.920943479	-0.012	0.004	2.82E-03	-0.021	0.006	6.22E-04
3.431403	0.023	0.009	6.97E-03	0.035	0.013	7.05E-03
2.897485	-0.017	0.006	5.88E-03	-0.018	0.009	5.43E-02
1.366716663	0.058	0.018	1.33E-03	0.032	0.027	2.29E-01
1.392958405	0.023	0.007	9.78E-04	0.016	0.010	1.18E-01
5.231654	0.021	0.008	9.35E-03	0.024	0.012	4.71E-02
5.192628	0.064	0.020	1.34E-03	0.025	0.030	4.11E-01
3.077813	-0.019	0.006	1.94E-03	-0.009	0.009	3.36E-01
1.206238317	0.015	0.005	5.10E-03	0.011	0.008	1.60E-01
2.105859065	-0.018	0.007	1.11E-02	-0.011	0.011	3.06E-01
1.57934206	0.078	0.024	1.25E-03	0.039	0.036	2.75E-01
3.396078	0.023	0.009	7.44E-03	0.038	0.013	2.81E-03
5.370264	0.040	0.013	1.78E-03	0.018	0.019	3.32E-01
4.078363	0.069	0.021	1.15E-03	0.045	0.032	1.57E-01
5.370601	0.036	0.012	1.65E-03	0.006	0.017	7.17E-01
1.206574749	0.015	0.005	5.09E-03	0.012	0.008	1.53E-01
3.868766	-0.013	0.005	7.19E-03	0.001	0.007	9.09E-01
5.999393	0.026	0.010	6.58E-03	-0.002	0.014	9.09E-01
1.280253487	0.060	0.018	1.14E-03	0.025	0.027	3.68E-01
0.927672131	-0.012	0.004	6.53E-03	-0.027	0.007	6.03E-05
5.188591	0.061	0.019	1.25E-03	0.015	0.028	5.88E-01
5.353106	0.051	0.017	2.05E-03	0.022	0.025	3.82E-01
1.387239051	0.024	0.007	8.59E-04	0.013	0.011	2.41E-01
2.231011989	0.078	0.024	1.38E-03	0.043	0.037	2.42E-01
0.921616345	-0.012	0.004	2.96E-03	-0.023	0.006	2.32E-04
6.192842	0.022	0.008	7.23E-03	0.017	0.012	1.63E-01
5.843289	0.021	0.007	3.98E-03	0.021	0.011	5.38E-02
1.206911182	0.015	0.006	5.23E-03	0.012	0.008	1.54E-01
3.457309	0.023	0.009	8.55E-03	0.039	0.013	2.85E-03
1.207584047	0.016	0.006	5.08E-03	0.012	0.008	1.46E-01
5.357816	0.047	0.015	1.89E-03	0.014	0.023	5.47E-01
3.8146	0.021	0.008	5.56E-03	0.033	0.011	3.13E-03
0.933391485	-0.014	0.005	3.21E-03	-0.015	0.007	2.76E-02

3.22988	0.023	0.008	3.58E-03	0.035	0.012	2.59E-03
7.781477	-0.032	0.011	2.18E-03	-0.001	0.016	9.62E-01
1.362679471	0.063	0.020	1.54E-03	0.034	0.030	2.57E-01
3.823011	0.021	0.008	5.24E-03	0.035	0.011	1.84E-03
4.076008	0.071	0.022	1.29E-03	0.038	0.033	2.49E-01
3.698195	0.022	0.008	5.61E-03	0.035	0.012	3.33E-03
0.923971373	-0.012	0.004	4.35E-03	-0.025	0.006	8.34E-05
3.002452	-0.013	0.004	2.01E-03	0.001	0.006	9.11E-01
1.576987032	0.077	0.024	1.42E-03	0.038	0.036	2.83E-01
5.701987	0.022	0.008	4.06E-03	0.010	0.012	3.99E-01
3.461346	0.023	0.009	7.45E-03	0.040	0.013	2.09E-03
3.83344	0.023	0.008	6.66E-03	0.038	0.012	2.42E-03
3.833104	0.023	0.009	6.50E-03	0.039	0.013	2.07E-03
2.230339124	0.080	0.025	1.45E-03	0.043	0.037	2.55E-01
2.957034	-0.013	0.005	3.82E-03	-0.005	0.007	4.40E-01
2.926755	-0.020	0.006	1.90E-03	-0.020	0.010	4.36E-02
3.775574	0.025	0.009	4.44E-03	0.035	0.013	7.84E-03
3.71838	0.023	0.008	4.62E-03	0.031	0.012	9.37E-03
1.365707365	0.059	0.018	1.51E-03	0.032	0.028	2.48E-01
3.016582	-0.012	0.004	3.26E-03	-0.005	0.006	4.73E-01
0.526980915	0.019	0.007	6.28E-03	-0.006	0.011	5.70E-01
1.577659897	0.077	0.024	1.39E-03	0.038	0.036	2.85E-01
5.1896	0.062	0.019	1.27E-03	0.018	0.029	5.35E-01
2.698989724	-0.022	0.006	4.86E-04	-0.016	0.010	9.68E-02
5.364545	0.031	0.010	2.73E-03	0.020	0.015	1.99E-01
6.420271	0.019	0.008	1.23E-02	0.003	0.012	8.23E-01
1.268141913	0.062	0.019	1.45E-03	0.026	0.029	3.77E-01
3.778602	0.024	0.008	3.31E-03	0.035	0.012	3.58E-03
4.078027	0.073	0.022	1.26E-03	0.047	0.034	1.62E-01
2.920362	-0.021	0.007	1.68E-03	-0.009	0.010	3.73E-01
1.280589919	0.059	0.018	1.23E-03	0.024	0.027	3.74E-01
2.889747	-0.017	0.006	6.39E-03	-0.007	0.009	4.75E-01
0.932045755	-0.013	0.005	4.32E-03	-0.008	0.007	2.16E-01
1.361670174	0.063	0.020	1.67E-03	0.036	0.030	2.24E-01
2.230675557	0.079	0.025	1.49E-03	0.042	0.037	2.52E-01
2.950305	-0.015	0.005	2.35E-03	-0.003	0.007	7.16E-01
2.945258	-0.014	0.005	4.83E-03	-0.004	0.007	5.53E-01
3.020283	-0.012	0.004	3.81E-03	-0.006	0.006	2.90E-01
4.0787	0.066	0.020	1.18E-03	0.047	0.030	1.23E-01
0.962324688	0.012	0.004	3.84E-03	0.013	0.006	3.62E-02
3.531997	0.023	0.008	5.23E-03	0.032	0.012	8.67E-03
3.713334	0.023	0.008	5.52E-03	0.040	0.012	1.32E-03
3.710306	0.025	0.009	4.29E-03	0.039	0.013	2.31E-03

3.739912	0.024	0.009	5.20E-03	0.038	0.013	2.91E-03
8.949234	-0.030	0.010	4.11E-03	-0.031	0.016	4.61E-02
2.945595	-0.014	0.005	4.66E-03	-0.004	0.007	5.94E-01
3.922595	0.032	0.012	7.90E-03	0.059	0.018	1.05E-03
5.846653	0.027	0.010	8.19E-03	0.005	0.015	7.41E-01
1.365370932	0.059	0.019	1.55E-03	0.033	0.028	2.31E-01
3.886933	0.021	0.008	4.97E-03	0.034	0.011	2.94E-03
0.702262295	0.034	0.011	1.53E-03	0.025	0.016	1.19E-01
2.106195498	-0.018	0.007	1.09E-02	-0.014	0.011	1.84E-01
1.20792048	0.016	0.006	5.31E-03	0.012	0.008	1.44E-01
5.191619	0.064	0.020	1.52E-03	0.026	0.030	3.81E-01
3.461682	0.024	0.009	6.18E-03	0.038	0.013	3.02E-03
2.628002447	-0.023	0.008	4.54E-03	-0.020	0.012	9.86E-02
2.696971128	-0.022	0.006	4.48E-04	-0.015	0.009	1.04E-01
4.084756	0.045	0.014	8.56E-04	0.028	0.020	1.61E-01
3.416264	0.022	0.008	7.31E-03	0.038	0.012	2.26E-03
1.576650599	0.076	0.024	1.55E-03	0.039	0.036	2.78E-01
4.082401	0.060	0.019	1.20E-03	0.034	0.028	2.20E-01
7.046035	-0.038	0.012	1.66E-03	-0.014	0.018	4.24E-01
1.57799633	0.076	0.024	1.46E-03	0.038	0.036	2.87E-01
5.35748	0.043	0.014	2.10E-03	0.007	0.021	7.21E-01
0.882253731	0.064	0.020	1.63E-03	0.038	0.030	2.05E-01
2.12335356	-0.020	0.007	3.46E-03	-0.014	0.010	1.76E-01
1.175286518	0.019	0.006	7.60E-04	-0.001	0.008	9.41E-01
3.271262	-0.033	0.013	1.18E-02	-0.013	0.019	4.87E-01
3.5468	0.030	0.010	1.74E-03	0.032	0.014	2.44E-02
1.208256912	0.016	0.006	5.20E-03	0.012	0.008	1.45E-01
3.419292	0.026	0.009	4.40E-03	0.037	0.014	6.78E-03
3.921586	0.033	0.012	7.07E-03	0.058	0.018	1.78E-03
2.107877661	-0.016	0.006	7.53E-03	-0.022	0.009	1.90E-02
3.412227	0.024	0.009	5.44E-03	0.037	0.013	3.18E-03
5.351088	0.057	0.018	2.13E-03	0.024	0.028	3.73E-01
1.392621972	0.023	0.007	1.25E-03	0.014	0.010	1.78E-01
7.76802	-0.036	0.012	2.19E-03	-0.030	0.017	8.43E-02
3.395405	0.023	0.008	7.88E-03	0.036	0.013	4.37E-03
2.116624908	-0.019	0.007	4.00E-03	0.002	0.010	8.42E-01
2.251534377	0.032	0.010	1.51E-03	0.013	0.015	4.00E-01
5.188927	0.062	0.019	1.47E-03	0.013	0.029	6.42E-01
1.301785172	0.060	0.019	1.49E-03	0.029	0.028	3.08E-01
1.280926352	0.059	0.018	1.34E-03	0.024	0.027	3.79E-01
5.358153	0.059	0.019	1.40E-03	0.025	0.028	3.67E-01
0.919934182	-0.012	0.004	3.16E-03	-0.019	0.006	2.51E-03
3.406844	0.024	0.009	8.47E-03	0.038	0.014	4.88E-03

5.37733	0.030	0.010	1.99E-03	-0.005	0.015	7.40E-01
5.354452	0.048	0.015	1.68E-03	0.005	0.023	8.42E-01
1.302121605	0.060	0.019	1.48E-03	0.029	0.028	3.11E-01
3.015573	-0.013	0.004	2.93E-03	-0.006	0.006	3.80E-01
3.82873	0.021	0.008	6.66E-03	0.036	0.012	2.50E-03
5.70266	0.020	0.007	3.65E-03	0.008	0.010	4.13E-01
3.270589	-0.035	0.014	1.23E-02	-0.025	0.021	2.22E-01
0.921952777	-0.012	0.004	3.41E-03	-0.024	0.006	1.05E-04
3.867084	-0.013	0.005	8.24E-03	0.001	0.007	9.32E-01
7.681556	0.022	0.008	7.61E-03	0.002	0.012	8.83E-01
3.079159	-0.017	0.006	2.90E-03	-0.006	0.009	4.72E-01
5.355798	0.049	0.016	2.05E-03	0.021	0.023	3.71E-01
1.30144874	0.061	0.019	1.52E-03	0.029	0.028	3.08E-01
2.122680695	-0.020	0.007	4.00E-03	-0.019	0.011	7.23E-02
1.576314167	0.076	0.024	1.58E-03	0.037	0.036	3.05E-01
1.30279447	0.060	0.019	1.48E-03	0.028	0.028	3.09E-01
1.302458038	0.060	0.019	1.49E-03	0.028	0.028	3.11E-01
3.242665	0.023	0.009	1.01E-02	0.031	0.013	1.79E-02
5.23771	0.021	0.008	1.15E-02	0.020	0.012	9.55E-02
3.019946	-0.012	0.004	4.28E-03	-0.005	0.006	4.08E-01
4.075672	0.070	0.022	1.51E-03	0.036	0.033	2.71E-01
6.684034	0.026	0.009	3.21E-03	0.022	0.013	9.19E-02
4.082064	0.061	0.019	1.16E-03	0.036	0.028	1.93E-01
2.700671886	-0.023	0.006	3.91E-04	-0.019	0.010	4.32E-02
2.972509	-0.013	0.004	4.30E-03	-0.004	0.007	5.94E-01
3.540744	0.023	0.009	1.52E-02	0.037	0.014	6.78E-03
2.944922	-0.014	0.005	5.61E-03	-0.008	0.007	3.04E-01
1.208593345	0.016	0.006	5.21E-03	0.012	0.008	1.51E-01
3.076131	-0.018	0.006	3.99E-03	-0.014	0.009	1.35E-01
2.436572302	-0.025	0.009	8.02E-03	0.027	0.014	5.52E-02
4.07769	0.070	0.022	1.44E-03	0.044	0.033	1.78E-01
1.303130903	0.059	0.019	1.49E-03	0.028	0.028	3.16E-01
2.31545657	-0.014	0.005	4.71E-03	-0.011	0.007	1.46E-01
2.466514803	-0.026	0.009	4.82E-03	-0.023	0.014	8.52E-02
3.813254	0.019	0.007	8.15E-03	0.030	0.011	5.70E-03
1.603565207	0.035	0.011	1.38E-03	0.013	0.016	4.23E-01
5.593992	0.020	0.007	4.02E-03	-0.009	0.010	3.92E-01
1.301112307	0.061	0.019	1.57E-03	0.029	0.029	3.07E-01
2.956361	-0.013	0.005	4.93E-03	-0.004	0.007	5.37E-01
1.281262784	0.058	0.018	1.41E-03	0.024	0.027	3.82E-01
2.474589185	-0.031	0.010	2.33E-03	-0.006	0.015	7.16E-01
1.362006606	0.062	0.020	1.87E-03	0.036	0.030	2.32E-01
5.192292	0.063	0.020	1.59E-03	0.026	0.030	3.95E-01

1.359651578	0.063	0.020	1.94E-03	0.039	0.030	1.99E-01
3.286401	-0.040	0.014	3.47E-03	-0.032	0.021	1.26E-01
3.076467	-0.018	0.006	3.24E-03	-0.011	0.009	2.42E-01
3.46572	0.026	0.009	4.70E-03	0.034	0.014	1.09E-02
3.010526	-0.013	0.004	2.03E-03	-0.006	0.006	3.41E-01
3.963303	-0.024	0.009	5.04E-03	-0.007	0.013	5.84E-01
1.204219721	0.014	0.005	6.07E-03	0.010	0.008	1.83E-01
2.475598483	-0.032	0.010	2.09E-03	-0.006	0.015	7.18E-01
3.006826	-0.011	0.004	4.52E-03	-0.005	0.006	4.10E-01
1.303467335	0.059	0.018	1.49E-03	0.027	0.028	3.24E-01
2.47391632	-0.030	0.010	2.59E-03	-0.006	0.015	7.05E-01
2.933147	-0.016	0.006	4.58E-03	-0.013	0.008	1.19E-01
3.467738	0.024	0.009	1.05E-02	0.041	0.014	3.12E-03
7.673482	0.023	0.008	4.52E-03	0.001	0.012	9.04E-01
5.179171	0.045	0.014	1.35E-03	0.017	0.021	4.12E-01
1.360324443	0.063	0.020	1.95E-03	0.039	0.030	1.99E-01
6.08552	0.024	0.009	8.37E-03	0.025	0.014	7.05E-02
2.625983851	-0.022	0.008	6.09E-03	-0.008	0.012	5.15E-01
2.231348422	0.076	0.024	1.68E-03	0.039	0.036	2.77E-01
3.850935	0.022	0.008	8.71E-03	0.036	0.012	4.13E-03
3.149809	-0.029	0.010	3.09E-03	-0.017	0.015	2.63E-01
2.236731343	0.073	0.023	1.57E-03	0.033	0.034	3.34E-01
3.009853	-0.012	0.004	2.78E-03	-0.003	0.006	5.65E-01
0.927335699	-0.012	0.004	7.85E-03	-0.026	0.007	8.82E-05
0.570380719	-0.023	0.009	8.22E-03	-0.018	0.013	1.57E-01
7.511322	-0.016	0.007	1.72E-02	-0.014	0.010	1.41E-01
2.231684854	0.075	0.024	1.71E-03	0.038	0.036	2.82E-01
0.881917299	0.062	0.020	1.81E-03	0.037	0.030	2.06E-01
1.208929777	0.016	0.006	5.51E-03	0.012	0.009	1.61E-01
0.922625642	-0.012	0.004	4.00E-03	-0.023	0.006	1.98E-04
0.92363494	-0.012	0.004	5.30E-03	-0.025	0.006	8.85E-05
1.367053095	0.056	0.018	1.71E-03	0.030	0.027	2.55E-01
2.696634695	-0.022	0.006	5.45E-04	-0.017	0.009	6.76E-02
1.300775875	0.061	0.019	1.66E-03	0.029	0.029	3.06E-01
3.730828	0.024	0.009	6.39E-03	0.035	0.013	7.04E-03
1.281599217	0.058	0.018	1.50E-03	0.024	0.027	3.87E-01
3.077476	-0.018	0.006	3.75E-03	-0.007	0.009	4.13E-01
1.367389528	0.055	0.018	1.69E-03	0.029	0.026	2.67E-01
4.082737	0.052	0.016	1.17E-03	0.026	0.024	2.84E-01
3.837814	0.022	0.008	7.20E-03	0.037	0.012	2.73E-03
5.353779	0.057	0.018	1.78E-03	0.025	0.027	3.64E-01
0.92228921	-0.012	0.004	3.75E-03	-0.023	0.006	1.91E-04
3.888615	0.021	0.008	6.99E-03	0.038	0.011	8.51E-04

1.181678737	0.014	0.005	1.62E-03	0.002	0.007	7.60E-01
7.750525	-0.025	0.010	1.13E-02	-0.009	0.015	5.58E-01
1.303803768	0.058	0.018	1.52E-03	0.026	0.027	3.37E-01
3.884915	0.020	0.008	8.04E-03	0.037	0.011	1.01E-03
3.835123	0.022	0.008	7.86E-03	0.038	0.012	1.72E-03
1.306495229	0.055	0.017	1.49E-03	0.023	0.026	3.66E-01
3.867757	-0.013	0.005	1.12E-02	0.001	0.008	9.05E-01
5.665316	0.021	0.008	9.67E-03	0.000	0.012	9.74E-01
3.834786	0.022	0.008	7.92E-03	0.038	0.012	2.10E-03
3.905773	0.022	0.008	8.91E-03	0.038	0.012	2.06E-03
1.300439442	0.060	0.019	1.73E-03	0.029	0.029	3.10E-01
3.870112	-0.013	0.005	7.71E-03	0.000	0.007	9.59E-01
1.370417421	0.051	0.016	1.81E-03	0.029	0.024	2.33E-01
1.306158796	0.055	0.017	1.47E-03	0.023	0.026	3.71E-01
0.697888671	0.047	0.015	1.38E-03	0.006	0.022	7.91E-01
3.230217	0.022	0.008	3.26E-03	0.038	0.011	7.41E-04
2.472234157	-0.024	0.008	4.50E-03	-0.006	0.013	6.54E-01
3.104055	-0.026	0.008	1.76E-03	-0.019	0.012	1.24E-01
5.187918	0.060	0.019	1.60E-03	0.020	0.028	4.71E-01
1.267805481	0.060	0.019	1.83E-03	0.024	0.029	3.98E-01
1.359315146	0.062	0.020	2.13E-03	0.038	0.030	2.06E-01
1.181342305	0.015	0.005	1.82E-03	0.001	0.007	8.85E-01
5.664979	0.021	0.008	9.68E-03	0.002	0.012	8.93E-01
1.575977734	0.075	0.024	1.76E-03	0.035	0.036	3.26E-01
7.873659	0.023	0.008	7.59E-03	-0.001	0.013	9.27E-01
1.203883288	0.014	0.005	6.79E-03	0.010	0.008	1.79E-01
0.923298507	-0.012	0.004	5.50E-03	-0.024	0.006	1.15E-04
1.603901639	0.033	0.011	1.60E-03	0.014	0.016	3.66E-01
3.002788	-0.012	0.004	2.62E-03	0.000	0.006	9.61E-01
3.609376	-0.018	0.007	6.98E-03	-0.003	0.010	7.66E-01
1.304140201	0.058	0.018	1.54E-03	0.025	0.027	3.55E-01
2.625310986	-0.024	0.009	4.65E-03	-0.004	0.013	7.30E-01
1.362343039	0.062	0.020	2.03E-03	0.035	0.030	2.42E-01
2.117970639	-0.021	0.007	3.96E-03	-0.009	0.011	4.16E-01
2.232021287	0.074	0.024	1.84E-03	0.038	0.035	2.79E-01
1.30010301	0.060	0.019	1.80E-03	0.029	0.029	3.11E-01
5.367909	0.031	0.010	2.16E-03	0.015	0.015	3.17E-01
3.145772	-0.058	0.019	2.51E-03	-0.075	0.029	9.05E-03
5.394151	0.025	0.009	8.57E-03	-0.016	0.014	2.67E-01
1.306831661	0.054	0.017	1.62E-03	0.023	0.025	3.67E-01
2.699999021	-0.022	0.006	5.97E-04	-0.019	0.010	4.35E-02
3.449234	0.025	0.009	5.39E-03	0.034	0.013	1.17E-02
1.28193565	0.058	0.018	1.62E-03	0.023	0.027	3.90E-01

1.305822364	0.056	0.018	1.52E-03	0.023	0.026	3.84E-01
2.236394911	0.072	0.023	1.75E-03	0.031	0.034	3.72E-01
2.415040617	-0.023	0.009	8.52E-03	0.005	0.013	6.79E-01
2.38173379	-0.021	0.007	3.75E-03	-0.003	0.011	8.14E-01
3.447889	0.022	0.009	1.03E-02	0.038	0.013	2.54E-03
3.229544	0.021	0.008	8.29E-03	0.029	0.012	1.27E-02
7.229391	-0.030	0.010	4.12E-03	0.010	0.016	5.07E-01
3.421983	0.023	0.009	8.07E-03	0.037	0.013	3.26E-03
3.39877	0.021	0.008	1.27E-02	0.033	0.013	9.82E-03
5.212814	0.018	0.007	5.56E-03	-0.007	0.010	4.77E-01
3.869102	-0.013	0.005	9.46E-03	0.002	0.007	8.20E-01
4.077354	0.070	0.023	1.79E-03	0.041	0.034	2.25E-01
1.299766577	0.060	0.019	1.85E-03	0.029	0.029	3.12E-01
1.305485931	0.056	0.018	1.54E-03	0.023	0.026	3.85E-01
3.43174	0.023	0.009	9.68E-03	0.034	0.013	1.03E-02
5.722509	0.022	0.008	9.09E-03	0.003	0.013	8.02E-01
1.099925618	0.023	0.008	4.48E-03	0.002	0.012	8.67E-01
1.305149498	0.057	0.018	1.54E-03	0.023	0.027	3.84E-01
5.393478	0.026	0.009	5.63E-03	-0.005	0.014	6.94E-01
3.445534	0.026	0.009	5.12E-03	0.034	0.014	1.41E-02
2.972846	-0.012	0.004	6.70E-03	-0.005	0.007	4.16E-01
3.119194	-0.025	0.008	2.21E-03	-0.016	0.012	1.81E-01
1.574968437	0.075	0.024	1.87E-03	0.036	0.036	3.17E-01
1.349895033	0.061	0.020	2.13E-03	0.034	0.030	2.51E-01
1.304476633	0.057	0.018	1.57E-03	0.024	0.027	3.72E-01
0.921279912	-0.012	0.004	4.04E-03	-0.022	0.006	2.61E-04
1.369408123	0.052	0.017	1.92E-03	0.035	0.025	1.57E-01
3.969023	-0.023	0.008	3.79E-03	-0.014	0.012	2.22E-01
0.896720333	0.037	0.012	1.73E-03	0.006	0.018	7.38E-01
1.060899437	0.015	0.006	1.08E-02	0.003	0.009	7.47E-01
3.672289	-0.013	0.005	6.56E-03	0.000	0.007	9.63E-01
1.304813066	0.057	0.018	1.57E-03	0.024	0.027	3.80E-01
1.307168094	0.053	0.017	1.72E-03	0.022	0.025	3.78E-01
1.575641302	0.075	0.024	1.86E-03	0.037	0.036	3.09E-01
1.20926621	0.016	0.006	6.36E-03	0.012	0.009	1.74E-01
2.923727	-0.017	0.006	6.79E-03	-0.007	0.009	4.58E-01
3.829403	0.022	0.008	7.72E-03	0.037	0.012	2.67E-03
5.392805	0.022	0.008	5.52E-03	-0.002	0.012	8.78E-01
1.396322731	0.021	0.006	1.27E-03	0.017	0.010	7.31E-02
2.474925618	-0.030	0.010	3.04E-03	-0.006	0.015	6.77E-01
3.814937	0.020	0.008	7.98E-03	0.034	0.011	2.52E-03
1.350231466	0.061	0.020	2.28E-03	0.033	0.030	2.69E-01
2.947277	-0.013	0.005	8.04E-03	-0.007	0.007	3.47E-01

2.967463	-0.013	0.005	2.89E-03	-0.003	0.007	6.48E-01
3.960948	-0.024	0.008	2.97E-03	-0.015	0.012	2.34E-01
0.881580866	0.060	0.020	2.06E-03	0.037	0.029	2.08E-01
3.002115	-0.012	0.004	2.74E-03	0.000	0.006	9.37E-01
3.100017	-0.041	0.014	4.23E-03	-0.047	0.022	3.00E-02
1.282272082	0.057	0.018	1.74E-03	0.023	0.027	3.94E-01
3.829067	0.021	0.008	8.22E-03	0.036	0.012	2.41E-03
1.299430144	0.060	0.019	1.97E-03	0.029	0.029	3.15E-01
3.671953	-0.013	0.005	7.10E-03	-0.002	0.007	7.80E-01
3.006489	-0.011	0.004	5.98E-03	-0.003	0.006	5.63E-01
2.700335454	-0.022	0.006	5.36E-04	-0.020	0.010	3.71E-02
7.336377	-0.022	0.007	2.59E-03	-0.012	0.011	2.65E-01
1.359988011	0.062	0.020	2.30E-03	0.037	0.030	2.15E-01
3.756734	0.027	0.010	5.87E-03	0.037	0.015	1.12E-02
0.922962075	-0.012	0.004	5.22E-03	-0.024	0.006	1.14E-04
3.396414	0.023	0.009	9.30E-03	0.036	0.013	5.76E-03
5.178834	0.046	0.015	1.79E-03	0.016	0.022	4.75E-01
5.188254	0.060	0.019	1.76E-03	0.021	0.028	4.53E-01
3.672626	-0.013	0.005	6.18E-03	0.001	0.007	9.39E-01
1.604574505	0.032	0.010	1.47E-03	0.012	0.015	4.31E-01
3.493644	0.025	0.010	9.43E-03	0.038	0.014	7.90E-03
1.574632004	0.074	0.024	2.02E-03	0.035	0.036	3.37E-01
1.181005872	0.015	0.005	2.13E-03	0.000	0.007	9.76E-01
3.464037	0.022	0.009	1.17E-02	0.039	0.013	3.02E-03
2.315793002	-0.014	0.005	6.19E-03	-0.015	0.008	4.69E-02
3.010863	-0.013	0.004	2.29E-03	-0.006	0.007	3.19E-01
1.019518229	0.025	0.008	2.32E-03	0.009	0.012	4.64E-01
5.929079	0.020	0.007	6.04E-03	0.011	0.011	2.85E-01
3.396751	0.023	0.009	8.03E-03	0.034	0.013	9.42E-03
1.370080989	0.051	0.016	2.12E-03	0.029	0.025	2.40E-01
2.625647419	-0.022	0.008	7.43E-03	-0.007	0.012	5.59E-01
5.398188	0.024	0.009	8.79E-03	0.005	0.014	7.44E-01
3.811572	0.019	0.007	1.06E-02	0.033	0.011	2.64E-03
1.575304869	0.074	0.024	1.99E-03	0.037	0.036	3.00E-01
1.282608515	0.057	0.018	1.80E-03	0.023	0.027	3.95E-01
1.3495586	0.060	0.020	2.31E-03	0.034	0.030	2.51E-01
0.875188647	0.058	0.019	1.96E-03	0.032	0.028	2.55E-01
1.349222168	0.061	0.020	2.39E-03	0.034	0.030	2.57E-01
0.694524345	0.048	0.016	3.21E-03	0.013	0.024	6.04E-01
2.924063	-0.017	0.006	6.33E-03	-0.008	0.010	4.14E-01
5.191955	0.062	0.020	2.09E-03	0.027	0.030	3.75E-01
7.046372	-0.033	0.011	1.92E-03	-0.014	0.016	3.82E-01
1.307504527	0.052	0.017	1.87E-03	0.021	0.025	3.90E-01

3.907119	0.023	0.008	7.80E-03	0.038	0.013	2.48E-03
1.299093712	0.060	0.019	2.07E-03	0.029	0.029	3.17E-01
3.75707	0.025	0.009	5.92E-03	0.034	0.014	1.19E-02
1.391612674	0.023	0.007	1.61E-03	0.018	0.011	1.00E-01
2.235385613	0.071	0.023	1.96E-03	0.033	0.034	3.35E-01
1.182688035	0.013	0.004	1.85E-03	0.004	0.006	5.04E-01
2.236058478	0.071	0.023	1.96E-03	0.032	0.034	3.56E-01
3.869439	-0.013	0.005	9.64E-03	0.001	0.007	8.79E-01
0.964006851	0.014	0.005	4.12E-03	0.011	0.007	1.18E-01
0.87552508	0.058	0.019	1.98E-03	0.032	0.028	2.56E-01
3.529642	0.025	0.009	5.29E-03	0.036	0.013	6.45E-03
5.673054	0.020	0.007	7.33E-03	0.004	0.011	7.11E-01
3.403143	0.025	0.009	6.85E-03	0.039	0.014	3.69E-03
3.007162	-0.011	0.004	5.56E-03	-0.005	0.006	4.37E-01
1.203210423	0.013	0.005	7.76E-03	0.010	0.007	1.79E-01
2.235722046	0.070	0.023	2.03E-03	0.032	0.034	3.51E-01
3.142744	-0.027	0.009	3.88E-03	-0.027	0.014	5.74E-02
1.358978713	0.061	0.020	2.57E-03	0.037	0.030	2.18E-01
2.966454	-0.013	0.004	4.31E-03	-0.006	0.007	3.79E-01
3.457645	0.022	0.009	1.18E-02	0.040	0.013	2.44E-03
1.18066944	0.015	0.005	2.02E-03	0.000	0.007	9.85E-01
2.234712748	0.070	0.023	2.08E-03	0.034	0.034	3.15E-01
0.7002437	0.034	0.011	2.22E-03	0.016	0.017	3.48E-01
0.875861512	0.058	0.019	1.98E-03	0.032	0.028	2.56E-01
3.242328	0.021	0.009	1.80E-02	0.030	0.013	2.12E-02
2.927764	-0.019	0.007	2.94E-03	-0.020	0.010	4.35E-02
0.874852214	0.057	0.018	2.05E-03	0.031	0.028	2.55E-01
3.960612	-0.025	0.008	2.94E-03	-0.011	0.012	3.71E-01
1.369071691	0.052	0.017	2.18E-03	0.033	0.025	1.91E-01
3.809554	0.020	0.007	8.39E-03	0.026	0.011	1.83E-02
1.357969415	0.061	0.020	2.65E-03	0.036	0.030	2.42E-01
1.18201517	0.014	0.004	1.88E-03	0.003	0.007	6.22E-01
2.93281	-0.017	0.006	4.05E-03	-0.018	0.009	3.88E-02
3.69954	0.021	0.008	6.83E-03	0.029	0.012	1.45E-02
4.074999	0.067	0.022	2.05E-03	0.027	0.032	4.10E-01
3.868429	-0.013	0.005	1.11E-02	0.002	0.008	8.18E-01
1.307840959	0.051	0.016	1.94E-03	0.020	0.025	4.18E-01
2.576191828	-0.028	0.011	1.39E-02	-0.033	0.017	4.97E-02
7.304079	-0.034	0.011	1.23E-03	-0.009	0.016	5.81E-01
3.390022	0.023	0.009	1.34E-02	0.041	0.014	3.31E-03
1.122466601	0.019	0.007	4.81E-03	0.006	0.010	5.29E-01
8.130694	0.024	0.009	7.80E-03	0.008	0.013	5.29E-01
3.909474	0.023	0.009	1.11E-02	0.041	0.014	2.64E-03

2.23235772	0.072	0.023	2.19E-03	0.037	0.035	2.92E-01
1.298757279	0.060	0.019	2.18E-03	0.029	0.029	3.20E-01
2.413694886	-0.023	0.009	1.13E-02	0.020	0.014	1.49E-01
1.203546856	0.013	0.005	8.05E-03	0.010	0.007	1.69E-01
6.683361	0.025	0.009	5.87E-03	0.002	0.013	8.66E-01
1.282944947	0.057	0.018	1.93E-03	0.023	0.027	4.00E-01
3.719726	0.024	0.009	5.52E-03	0.034	0.013	7.29E-03
2.123017127	-0.020	0.007	5.37E-03	-0.015	0.011	1.59E-01
1.605920235	0.030	0.009	1.36E-03	0.016	0.014	2.60E-01
1.368398826	0.053	0.017	2.20E-03	0.032	0.026	2.18E-01
5.189937	0.061	0.020	2.12E-03	0.017	0.030	5.59E-01
3.821329	0.019	0.007	8.62E-03	0.034	0.011	1.87E-03
3.092952	-0.023	0.008	2.93E-03	-0.016	0.012	1.67E-01
1.358305848	0.061	0.020	2.78E-03	0.036	0.030	2.35E-01
2.477953511	-0.026	0.008	1.20E-03	-0.015	0.012	2.21E-01
1.36772596	0.054	0.017	2.20E-03	0.029	0.026	2.74E-01
3.848916	0.023	0.009	7.38E-03	0.035	0.013	6.99E-03
3.011199	-0.014	0.004	2.31E-03	-0.006	0.007	3.36E-01
1.173604355	0.013	0.004	9.50E-04	0.004	0.006	5.09E-01
3.769518	0.022	0.008	9.95E-03	0.028	0.013	2.40E-02
3.716025	0.022	0.008	6.54E-03	0.030	0.012	1.27E-02
3.870448	-0.013	0.005	9.51E-03	0.001	0.007	9.20E-01
1.308177392	0.050	0.016	2.01E-03	0.018	0.024	4.52E-01
0.876197945	0.058	0.019	2.07E-03	0.032	0.028	2.59E-01
1.267469048	0.058	0.019	2.30E-03	0.023	0.028	4.21E-01
5.393815	0.022	0.008	7.57E-03	-0.022	0.013	8.16E-02
1.35864228	0.061	0.020	2.83E-03	0.037	0.030	2.24E-01
7.769029	-0.032	0.011	2.82E-03	-0.028	0.016	8.21E-02
0.695197211	0.051	0.017	2.24E-03	-0.005	0.025	8.26E-01
4.084419	0.043	0.013	1.29E-03	0.025	0.020	2.23E-01
2.944249	-0.014	0.005	6.45E-03	-0.008	0.007	2.57E-01
2.23504918	0.070	0.023	2.16E-03	0.033	0.034	3.39E-01
2.125035723	-0.025	0.008	2.74E-03	0.000	0.012	9.73E-01
3.001106	-0.011	0.004	7.01E-03	0.002	0.006	7.68E-01
2.696298263	-0.022	0.006	6.77E-04	-0.016	0.009	8.85E-02
1.606256667	0.028	0.009	1.79E-03	0.011	0.014	4.08E-01
7.713181	-0.028	0.010	6.59E-03	-0.013	0.015	4.00E-01
2.233367017	0.070	0.023	2.20E-03	0.034	0.034	3.17E-01
3.826039	0.020	0.007	7.42E-03	0.033	0.011	2.30E-03
5.99973	0.021	0.009	2.18E-02	0.005	0.014	7.29E-01
0.874515782	0.056	0.018	2.20E-03	0.031	0.027	2.53E-01
1.616013213	0.020	0.007	2.74E-03	0.005	0.010	5.82E-01
3.259823	0.024	0.009	5.21E-03	0.038	0.013	3.51E-03

4.08341	0.049	0.015	1.37E-03	0.026	0.023	2.60E-01
8.053651	0.022	0.008	9.34E-03	0.006	0.012	6.41E-01
5.852036	0.026	0.009	4.69E-03	0.014	0.014	3.25E-01
5.228626	0.023	0.009	8.60E-03	0.045	0.013	5.72E-04
2.934493	-0.017	0.005	2.16E-03	-0.005	0.008	5.55E-01
1.350567898	0.061	0.020	2.70E-03	0.034	0.030	2.65E-01
2.23370345	0.070	0.023	2.26E-03	0.033	0.034	3.34E-01
3.697858	0.020	0.008	9.47E-03	0.034	0.012	3.03E-03
3.478504	0.025	0.010	1.11E-02	0.040	0.014	4.99E-03
0.919597749	-0.011	0.004	5.64E-03	-0.018	0.006	3.98E-03
1.298420847	0.059	0.019	2.32E-03	0.029	0.029	3.25E-01
2.94324	-0.015	0.005	3.54E-03	-0.006	0.007	4.13E-01
2.699326156	-0.022	0.006	8.20E-04	-0.016	0.010	9.72E-02
1.368062393	0.053	0.017	2.27E-03	0.029	0.026	2.64E-01
1.401032787	0.016	0.005	1.43E-03	0.015	0.007	4.91E-02
2.69596183	-0.021	0.006	7.56E-04	-0.015	0.009	1.16E-01
3.969359	-0.023	0.008	4.40E-03	-0.016	0.012	1.69E-01
3.4166	0.021	0.008	1.09E-02	0.038	0.012	2.23E-03
3.868093	-0.013	0.005	1.32E-02	0.002	0.008	7.84E-01
2.699662589	-0.022	0.006	8.08E-04	-0.018	0.010	6.64E-02
1.28328138	0.056	0.018	2.07E-03	0.023	0.027	4.05E-01
1.209602643	0.016	0.006	7.36E-03	0.012	0.009	1.82E-01
1.574295571	0.073	0.024	2.33E-03	0.034	0.036	3.47E-01
4.076345	0.066	0.022	2.19E-03	0.038	0.032	2.43E-01
3.729146	0.022	0.009	1.32E-02	0.035	0.013	8.67E-03
2.701008319	-0.022	0.006	5.71E-04	-0.020	0.010	3.83E-02
3.473121	0.022	0.009	1.31E-02	0.041	0.013	2.44E-03
1.384211157	0.030	0.010	1.63E-03	0.019	0.014	1.83E-01
0.876534377	0.058	0.019	2.15E-03	0.032	0.028	2.62E-01
3.451589	0.022	0.009	1.23E-02	0.038	0.013	4.52E-03
2.989331	-0.011	0.004	7.15E-03	0.003	0.006	5.91E-01
5.235019	0.022	0.008	9.31E-03	0.043	0.013	7.23E-04
3.505755	0.025	0.009	7.05E-03	0.036	0.014	9.71E-03
1.348885735	0.061	0.020	2.75E-03	0.035	0.030	2.53E-01
2.233030585	0.070	0.023	2.31E-03	0.035	0.034	3.09E-01
3.007835	-0.010	0.004	6.91E-03	-0.003	0.006	6.18E-01
5.354116	0.050	0.016	1.91E-03	0.013	0.024	5.75E-01
3.812918	0.018	0.007	1.31E-02	0.032	0.011	2.89E-03
2.948286	-0.013	0.005	6.19E-03	-0.005	0.007	4.96E-01
2.955351	-0.013	0.005	5.04E-03	-0.007	0.007	3.05E-01
0.881244434	0.059	0.020	2.42E-03	0.036	0.029	2.13E-01
1.368735258	0.051	0.017	2.52E-03	0.031	0.025	2.21E-01
2.232694152	0.071	0.023	2.39E-03	0.035	0.035	3.08E-01

0.696879374	0.045	0.015	2.01E-03	0.014	0.022	5.26E-01
4.077018	0.069	0.022	2.27E-03	0.039	0.034	2.42E-01
0.961988255	0.012	0.004	4.41E-03	0.013	0.006	3.54E-02
2.942903	-0.014	0.005	3.95E-03	-0.005	0.007	4.68E-01
5.213823	0.016	0.006	7.95E-03	-0.003	0.009	7.20E-01
3.009181	-0.011	0.004	3.69E-03	-0.004	0.006	4.67E-01
2.92339	-0.018	0.006	4.86E-03	-0.011	0.009	2.30E-01
3.869775	-0.013	0.005	1.01E-02	0.000	0.007	9.81E-01
1.298084414	0.059	0.019	2.45E-03	0.028	0.029	3.28E-01
5.387086	0.020	0.007	4.10E-03	0.009	0.010	3.61E-01
2.695625398	-0.021	0.006	8.41E-04	-0.013	0.009	1.79E-01
4.075335	0.067	0.022	2.41E-03	0.030	0.033	3.60E-01
2.487373624	-0.039	0.013	2.50E-03	-0.014	0.019	4.71E-01
2.471224859	-0.023	0.008	4.69E-03	-0.010	0.012	4.23E-01
1.390266944	0.024	0.008	1.92E-03	0.016	0.011	1.52E-01
3.001779	-0.012	0.004	4.51E-03	0.002	0.006	8.02E-01
1.173940788	0.013	0.004	1.16E-03	0.004	0.006	4.85E-01
1.202873991	0.013	0.005	8.87E-03	0.010	0.007	1.82E-01
4.076681	0.066	0.022	2.23E-03	0.039	0.032	2.20E-01
5.480951	0.020	0.007	4.85E-03	0.023	0.010	2.33E-02
2.234376315	0.069	0.023	2.36E-03	0.034	0.034	3.19E-01
2.63103034	-0.021	0.008	5.29E-03	-0.014	0.011	2.19E-01
7.884089	-0.028	0.009	1.73E-03	0.000	0.013	9.79E-01
3.672962	-0.012	0.005	7.25E-03	0.001	0.007	8.34E-01
3.01019	-0.012	0.004	3.33E-03	-0.005	0.006	4.54E-01
3.087569	-0.017	0.006	5.57E-03	0.001	0.009	9.34E-01
1.209939075	0.016	0.006	8.00E-03	0.012	0.009	1.83E-01
0.874179349	0.055	0.018	2.46E-03	0.031	0.027	2.56E-01
3.003125	-0.012	0.004	3.33E-03	0.000	0.006	9.79E-01
1.369744556	0.050	0.017	2.42E-03	0.033	0.025	1.77E-01
6.413542	0.023	0.010	1.87E-02	-0.003	0.014	8.19E-01
7.674828	0.024	0.009	9.02E-03	0.020	0.014	1.42E-01
3.40718	0.022	0.009	1.19E-02	0.037	0.013	5.16E-03
1.106317837	0.018	0.007	1.01E-02	-0.001	0.010	9.08E-01
2.695288965	-0.021	0.006	9.43E-04	-0.012	0.009	1.83E-01
3.006153	-0.011	0.004	7.15E-03	-0.003	0.006	5.49E-01
6.739882	-0.035	0.011	1.28E-03	-0.013	0.016	4.19E-01
1.604910937	0.031	0.010	1.72E-03	0.010	0.015	4.91E-01
7.767683	-0.031	0.010	3.38E-03	-0.019	0.016	2.14E-01
1.308513824	0.049	0.016	2.30E-03	0.017	0.024	4.72E-01
1.283617813	0.056	0.018	2.27E-03	0.022	0.027	4.09E-01
1.297747981	0.059	0.019	2.55E-03	0.028	0.029	3.30E-01
1.107327135	0.018	0.006	4.82E-03	0.002	0.009	8.11E-01

1.357632983	0.060	0.020	3.04E-03	0.037	0.030	2.30E-01
5.361517	0.033	0.011	3.33E-03	0.012	0.017	4.56E-01
2.934156	-0.016	0.005	2.99E-03	-0.008	0.008	3.49E-01
7.222326	-0.028	0.010	5.27E-03	-0.015	0.015	3.06E-01
5.190273	0.061	0.020	2.24E-03	0.018	0.030	5.55E-01
3.823347	0.020	0.008	9.83E-03	0.035	0.011	1.89E-03
1.572613408	0.073	0.024	2.56E-03	0.036	0.036	3.16E-01
1.183024468	0.013	0.004	2.49E-03	0.004	0.006	4.95E-01
2.134792268	-0.025	0.010	9.70E-03	0.005	0.014	7.04E-01
2.926418	-0.019	0.006	3.34E-03	-0.024	0.010	1.49E-02
0.702598728	0.028	0.010	3.65E-03	0.016	0.014	2.71E-01
7.299705	-0.028	0.009	2.84E-03	0.009	0.014	5.22E-01
7.674491	0.022	0.009	9.82E-03	0.019	0.013	1.45E-01
3.015236	-0.012	0.004	4.07E-03	-0.005	0.006	4.21E-01
3.423666	0.021	0.008	9.75E-03	0.040	0.012	1.29E-03
3.004807	-0.011	0.004	4.54E-03	-0.003	0.006	5.98E-01
1.573959139	0.073	0.024	2.55E-03	0.035	0.036	3.36E-01
3.969696	-0.022	0.008	4.36E-03	-0.015	0.012	2.08E-01
1.311541718	0.044	0.014	2.17E-03	0.022	0.022	3.17E-01
2.881673	-0.019	0.007	9.53E-03	0.001	0.011	9.63E-01
0.87687081	0.058	0.019	2.36E-03	0.032	0.028	2.66E-01
5.187582	0.054	0.018	2.42E-03	0.014	0.027	6.07E-01
7.995448	0.017	0.008	3.06E-02	0.019	0.012	1.14E-01
2.234039883	0.069	0.023	2.54E-03	0.034	0.034	3.21E-01
5.368246	0.030	0.010	3.09E-03	0.013	0.015	3.77E-01
3.7675	0.030	0.010	2.52E-03	0.038	0.015	1.01E-02
5.187245	0.051	0.017	2.42E-03	0.011	0.025	6.62E-01
3.410881	0.020	0.008	1.18E-02	0.039	0.012	1.22E-03
3.854636	0.020	0.008	1.30E-02	0.034	0.012	4.60E-03
3.883232	0.021	0.008	7.78E-03	0.031	0.012	8.86E-03
3.087233	-0.018	0.007	7.30E-03	0.002	0.010	8.45E-01
3.001443	-0.011	0.004	6.13E-03	0.002	0.006	7.40E-01
1.283954245	0.055	0.018	2.39E-03	0.022	0.027	4.14E-01
2.701344752	-0.022	0.006	6.38E-04	-0.021	0.010	2.94E-02
1.350904331	0.061	0.020	3.11E-03	0.034	0.031	2.70E-01
3.458655	0.024	0.009	8.85E-03	0.035	0.014	1.03E-02
1.57025838	0.073	0.024	2.70E-03	0.033	0.036	3.66E-01
3.539398	0.026	0.009	3.83E-03	0.031	0.013	2.16E-02
1.572276976	0.073	0.024	2.68E-03	0.034	0.036	3.40E-01
3.388004	0.024	0.009	8.30E-03	0.035	0.014	1.06E-02
1.210275508	0.016	0.006	8.64E-03	0.012	0.009	1.71E-01
3.023647	-0.014	0.005	4.16E-03	-0.010	0.007	1.53E-01
1.297411549	0.058	0.019	2.72E-03	0.028	0.029	3.36E-01

3.41189	0.022	0.009	9.78E-03	0.038	0.013	3.12E-03
2.960398	-0.012	0.005	6.07E-03	-0.006	0.007	4.05E-01
5.185563	0.048	0.016	2.76E-03	0.010	0.024	6.70E-01
5.581881	0.025	0.008	2.64E-03	0.006	0.013	6.51E-01
1.60524737	0.031	0.010	1.60E-03	0.011	0.015	4.38E-01
1.571267678	0.073	0.024	2.68E-03	0.034	0.036	3.48E-01
3.866747	-0.012	0.005	1.13E-02	0.002	0.007	8.27E-01
5.364209	0.033	0.011	3.30E-03	0.015	0.017	3.72E-01
5.346378	0.053	0.018	2.94E-03	0.015	0.027	5.84E-01
5.35277	0.056	0.019	3.37E-03	0.029	0.029	3.17E-01
1.573622706	0.072	0.024	2.69E-03	0.034	0.036	3.39E-01
3.146445	-0.062	0.022	4.26E-03	-0.097	0.033	2.85E-03
6.683697	0.020	0.007	6.61E-03	0.003	0.011	7.81E-01
3.093289	-0.023	0.008	3.44E-03	-0.012	0.012	2.90E-01
3.271598	-0.029	0.012	1.53E-02	-0.006	0.018	7.38E-01
3.90611	0.020	0.008	1.33E-02	0.037	0.012	2.15E-03
7.947338	0.018	0.008	1.95E-02	0.006	0.011	6.12E-01
2.486364326	-0.035	0.012	2.32E-03	-0.010	0.017	5.50E-01
2.889411	-0.016	0.006	1.07E-02	-0.005	0.009	6.19E-01
5.350079	0.052	0.018	3.39E-03	0.026	0.026	3.22E-01
2.921035	-0.019	0.007	3.72E-03	-0.011	0.010	2.81E-01
1.311205285	0.044	0.015	2.44E-03	0.021	0.022	3.31E-01
5.379685	0.025	0.009	5.11E-03	0.008	0.013	5.51E-01
1.019181796	0.024	0.009	4.70E-03	0.009	0.013	4.98E-01
1.390603377	0.023	0.008	2.39E-03	0.019	0.011	1.00E-01
3.395069	0.021	0.009	1.29E-02	0.035	0.013	5.51E-03
6.66082	0.024	0.009	7.70E-03	-0.001	0.013	9.41E-01
5.346041	0.053	0.018	3.13E-03	0.011	0.027	6.72E-01
2.970154	-0.012	0.004	8.11E-03	-0.001	0.007	8.24E-01
1.182351603	0.013	0.004	2.52E-03	0.004	0.007	5.54E-01
1.351240763	0.060	0.021	3.33E-03	0.034	0.031	2.72E-01
3.712998	0.021	0.008	9.68E-03	0.039	0.012	1.35E-03
7.497528	0.024	0.009	7.05E-03	0.009	0.013	5.01E-01
1.570594813	0.072	0.024	2.81E-03	0.032	0.036	3.77E-01
3.471439	0.024	0.009	8.73E-03	0.034	0.014	1.29E-02
1.572949841	0.072	0.024	2.82E-03	0.036	0.036	3.17E-01
1.570931245	0.072	0.024	2.81E-03	0.032	0.036	3.71E-01
2.315120137	-0.013	0.005	7.75E-03	-0.007	0.007	3.38E-01
1.060563005	0.016	0.007	1.37E-02	0.005	0.010	5.99E-01
1.308850257	0.048	0.016	2.60E-03	0.016	0.024	4.93E-01
3.851271	0.019	0.008	1.54E-02	0.033	0.012	4.99E-03
1.348549303	0.060	0.020	3.25E-03	0.034	0.030	2.70E-01
1.573286274	0.072	0.024	2.79E-03	0.035	0.036	3.29E-01

2.468869831	-0.026	0.009	2.56E-03	-0.008	0.013	5.30E-01
1.604238072	0.032	0.010	2.16E-03	0.011	0.015	4.91E-01
1.202537558	0.012	0.005	1.03E-02	0.009	0.007	1.98E-01
1.31187815	0.043	0.014	2.26E-03	0.021	0.021	3.13E-01
1.284290678	0.055	0.018	2.58E-03	0.022	0.027	4.19E-01
3.029367	-0.012	0.005	1.21E-02	-0.006	0.007	3.61E-01
0.873842917	0.053	0.018	2.82E-03	0.030	0.027	2.62E-01
1.35729655	0.060	0.020	3.36E-03	0.036	0.031	2.42E-01
3.428712	0.023	0.009	8.20E-03	0.033	0.013	1.05E-02
0.877207242	0.058	0.019	2.58E-03	0.031	0.029	2.71E-01
2.40124688	-0.023	0.009	1.30E-02	0.009	0.014	5.23E-01
7.311144	-0.027	0.009	3.89E-03	0.002	0.014	8.90E-01
6.522883	0.020	0.009	2.10E-02	0.024	0.013	6.85E-02
3.270252	-0.032	0.014	1.93E-02	-0.024	0.021	2.35E-01
3.246029	0.024	0.008	4.70E-03	0.043	0.013	7.22E-04
0.880908001	0.058	0.019	2.87E-03	0.035	0.029	2.23E-01
1.297075116	0.058	0.019	2.91E-03	0.028	0.029	3.41E-01
3.076804	-0.017	0.006	6.57E-03	-0.010	0.009	2.95E-01
1.267132616	0.056	0.019	3.00E-03	0.021	0.028	4.52E-01
3.424002	0.021	0.008	1.01E-02	0.038	0.012	1.72E-03
3.47884	0.024	0.010	1.35E-02	0.040	0.014	6.12E-03
5.546219	0.021	0.008	5.22E-03	-0.005	0.011	6.63E-01
3.094634	-0.024	0.008	2.71E-03	-0.016	0.012	1.87E-01
1.21061194	0.016	0.006	9.44E-03	0.012	0.009	1.81E-01
3.411218	0.020	0.008	1.29E-02	0.039	0.012	1.32E-03
5.344023	0.057	0.020	4.11E-03	0.017	0.030	5.72E-01
3.145436	-0.049	0.017	3.87E-03	-0.059	0.025	2.06E-02
2.973519	-0.012	0.004	6.56E-03	-0.003	0.007	7.02E-01
3.852617	0.020	0.008	1.15E-02	0.032	0.012	6.23E-03
1.180333007	0.016	0.005	2.62E-03	0.000	0.008	9.77E-01
2.105522633	-0.016	0.007	2.20E-02	-0.007	0.011	4.87E-01
3.811909	0.018	0.007	1.57E-02	0.032	0.011	3.45E-03
2.624638121	-0.024	0.010	1.03E-02	-0.007	0.014	6.09E-01
7.781813	-0.025	0.009	7.42E-03	-0.003	0.014	8.36E-01
3.903418	0.022	0.009	9.80E-03	0.035	0.013	7.50E-03
3.423329	0.021	0.008	1.15E-02	0.039	0.012	1.28E-03
4.084083	0.044	0.014	1.84E-03	0.021	0.021	3.17E-01
1.569921948	0.072	0.024	3.01E-03	0.032	0.036	3.77E-01
5.855737	0.031	0.010	3.01E-03	-0.002	0.015	8.74E-01
1.34821287	0.060	0.021	3.40E-03	0.034	0.031	2.70E-01
0.69385148	0.048	0.017	4.32E-03	0.020	0.025	4.35E-01
0.695870076	0.049	0.016	2.38E-03	0.010	0.024	6.84E-01
2.949296	-0.013	0.005	7.11E-03	-0.005	0.007	4.98E-01

7.97358	0.016	0.006	8.53E-03	-0.002	0.009	7.89E-01
1.28462711	0.055	0.018	2.75E-03	0.022	0.027	4.24E-01
1.571604111	0.072	0.024	2.97E-03	0.034	0.036	3.49E-01
5.359499	0.030	0.011	4.14E-03	0.011	0.016	4.88E-01
3.241992	0.018	0.008	2.67E-02	0.029	0.012	1.78E-02
1.210948373	0.016	0.006	9.56E-03	0.012	0.009	1.96E-01
2.923054	-0.018	0.006	4.85E-03	-0.014	0.010	1.56E-01
1.296738684	0.057	0.019	3.08E-03	0.027	0.029	3.44E-01
3.532333	0.021	0.008	7.06E-03	0.031	0.012	6.99E-03
3.525268	0.022	0.009	9.97E-03	0.031	0.013	1.40E-02
1.113382921	0.019	0.007	8.78E-03	-0.005	0.011	6.30E-01
3.83815	0.020	0.008	1.24E-02	0.035	0.012	3.58E-03
2.927091	-0.019	0.007	3.98E-03	-0.018	0.010	7.22E-02
3.007498	-0.010	0.004	7.99E-03	-0.003	0.006	6.08E-01
3.448225	0.021	0.009	1.46E-02	0.040	0.013	1.96E-03
3.468075	0.022	0.009	1.71E-02	0.040	0.014	3.82E-03
3.708287	0.020	0.008	1.13E-02	0.031	0.012	9.84E-03
1.172595057	0.013	0.004	1.77E-03	0.002	0.006	7.31E-01
5.499118	0.014	0.006	1.23E-02	-0.002	0.008	7.74E-01
0.767193785	0.014	0.005	5.94E-03	0.002	0.008	7.86E-01
1.017836066	0.028	0.009	2.74E-03	0.024	0.014	8.43E-02
0.666264008	-0.025	0.009	4.82E-03	-0.018	0.013	1.76E-01
1.842768779	-0.026	0.010	8.56E-03	0.002	0.015	8.78E-01
6.069371	0.024	0.008	4.28E-03	0.014	0.012	2.50E-01
2.25187081	0.030	0.010	2.90E-03	0.015	0.015	3.25E-01
1.571940543	0.072	0.024	3.10E-03	0.034	0.036	3.46E-01
5.360844	0.038	0.013	3.50E-03	0.010	0.019	6.06E-01
1.30918669	0.047	0.016	2.89E-03	0.016	0.024	5.05E-01
3.407517	0.021	0.009	1.47E-02	0.036	0.013	5.16E-03
3.094298	-0.024	0.008	3.27E-03	-0.016	0.012	1.93E-01
3.411554	0.021	0.008	1.26E-02	0.038	0.013	2.15E-03
2.465841938	-0.024	0.009	8.23E-03	-0.024	0.014	7.85E-02
5.354789	0.043	0.015	3.45E-03	0.000	0.022	9.96E-01
3.710643	0.022	0.008	9.39E-03	0.038	0.012	2.20E-03
2.135128701	-0.027	0.010	6.95E-03	-0.001	0.015	9.35E-01
1.310868852	0.044	0.015	2.86E-03	0.021	0.022	3.50E-01
0.929017862	-0.011	0.005	1.54E-02	-0.025	0.007	1.71E-04
3.457982	0.021	0.009	1.49E-02	0.039	0.013	3.16E-03
1.284963543	0.054	0.018	2.91E-03	0.022	0.027	4.28E-01
2.429507218	-0.025	0.009	8.00E-03	-0.012	0.014	3.75E-01
2.400910448	-0.023	0.009	1.53E-02	0.007	0.014	5.95E-01
4.074663	0.063	0.021	2.97E-03	0.029	0.032	3.60E-01
1.605583802	0.030	0.010	1.86E-03	0.011	0.014	4.55E-01

5.480614	0.018	0.007	6.19E-03	0.028	0.010	5.79E-03
3.454954	0.024	0.009	9.05E-03	0.033	0.014	1.92E-02
2.473243455	-0.028	0.010	3.96E-03	-0.003	0.014	8.61E-01
1.356960117	0.059	0.020	3.77E-03	0.035	0.031	2.57E-01
3.906783	0.020	0.008	1.31E-02	0.038	0.012	1.74E-03
2.889074	-0.016	0.006	1.01E-02	-0.007	0.009	4.37E-01
2.119316369	-0.019	0.007	8.00E-03	-0.009	0.011	3.85E-01
0.877543675	0.057	0.019	2.90E-03	0.031	0.029	2.77E-01
5.371947	0.027	0.010	3.99E-03	0.017	0.014	2.36E-01
7.713517	-0.029	0.010	4.04E-03	-0.014	0.015	3.37E-01
2.104849768	-0.016	0.007	1.86E-02	-0.001	0.010	9.29E-01
1.296402251	0.057	0.019	3.29E-03	0.027	0.029	3.53E-01
1.351577196	0.060	0.021	3.87E-03	0.034	0.031	2.76E-01
2.963426	-0.012	0.005	7.70E-03	-0.004	0.007	5.41E-01
1.173267923	0.013	0.004	1.35E-03	0.004	0.006	5.50E-01
1.569585515	0.071	0.024	3.28E-03	0.031	0.036	3.92E-01
2.989667	-0.011	0.004	7.26E-03	0.004	0.006	5.42E-01
5.701314	0.017	0.007	1.33E-02	0.004	0.010	7.36E-01
2.431189381	-0.020	0.008	8.64E-03	-0.016	0.011	1.49E-01
5.351761	0.054	0.019	4.67E-03	0.023	0.029	4.28E-01
1.568576217	0.070	0.024	3.42E-03	0.030	0.036	3.94E-01
5.722173	0.023	0.008	3.86E-03	0.011	0.012	3.59E-01
1.569249082	0.070	0.024	3.36E-03	0.031	0.036	3.88E-01
1.285299976	0.054	0.018	3.02E-03	0.022	0.027	4.28E-01
3.290438	-0.045	0.017	6.34E-03	-0.059	0.025	1.69E-02
7.675164	0.023	0.009	1.17E-02	0.020	0.014	1.57E-01
7.11635	-0.028	0.010	7.59E-03	-0.006	0.015	7.00E-01
0.696206508	0.042	0.014	2.51E-03	0.009	0.021	6.57E-01
5.351424	0.055	0.019	4.13E-03	0.022	0.029	4.51E-01
2.908251	-0.023	0.008	3.62E-03	-0.015	0.012	2.13E-01
8.751076	-0.020	0.007	5.79E-03	-0.004	0.011	7.12E-01
3.42232	0.021	0.008	1.25E-02	0.037	0.012	2.86E-03
5.174797	0.039	0.013	2.88E-03	-0.001	0.020	9.49E-01
1.56891265	0.070	0.024	3.43E-03	0.031	0.036	3.92E-01
1.606929533	0.026	0.009	3.01E-03	0.002	0.013	8.93E-01
2.624974553	-0.024	0.009	9.19E-03	-0.006	0.013	6.78E-01
1.120448006	0.015	0.006	7.19E-03	0.008	0.009	3.73E-01
5.231991	0.018	0.008	2.11E-02	0.024	0.012	4.07E-02
2.958043	-0.012	0.005	7.58E-03	-0.006	0.007	4.03E-01
3.851608	0.018	0.008	1.80E-02	0.031	0.011	5.97E-03
7.173543	-0.021	0.008	1.35E-02	-0.003	0.012	8.13E-01
1.347876437	0.060	0.021	3.81E-03	0.035	0.031	2.62E-01
2.476271348	-0.027	0.010	4.47E-03	-0.004	0.014	7.56E-01

5.380021	0.026	0.009	4.02E-03	0.000	0.014	9.94E-01
5.372956	0.025	0.009	6.25E-03	-0.002	0.014	9.02E-01
0.873506484	0.052	0.018	3.37E-03	0.029	0.026	2.70E-01
0.897056765	0.034	0.011	2.83E-03	0.003	0.017	8.67E-01
0.880571568	0.057	0.019	3.34E-03	0.034	0.029	2.36E-01
3.464374	0.021	0.009	1.79E-02	0.038	0.013	3.51E-03
5.756153	0.024	0.010	1.80E-02	0.007	0.015	6.33E-01
1.296065818	0.057	0.019	3.48E-03	0.026	0.029	3.61E-01
7.883752	-0.028	0.009	1.68E-03	-0.002	0.013	8.87E-01
8.636016	0.019	0.007	1.09E-02	-0.003	0.011	7.54E-01
1.285636408	0.054	0.018	3.18E-03	0.022	0.027	4.28E-01
1.108336433	0.015	0.006	7.02E-03	0.006	0.008	4.70E-01
2.944586	-0.013	0.005	1.02E-02	-0.009	0.007	2.12E-01
7.219634	-0.029	0.010	3.75E-03	-0.028	0.015	6.01E-02
3.490279	0.024	0.009	1.13E-02	0.038	0.014	5.98E-03
1.568239785	0.070	0.024	3.58E-03	0.029	0.036	4.23E-01
3.408862	0.020	0.008	1.38E-02	0.034	0.012	5.32E-03
2.414704184	-0.020	0.009	1.78E-02	0.013	0.013	2.97E-01
1.567230487	0.069	0.024	3.58E-03	0.029	0.035	4.16E-01
2.701681184	-0.021	0.006	8.46E-04	-0.022	0.009	2.28E-02
3.458318	0.022	0.009	1.35E-02	0.038	0.013	4.86E-03
5.186909	0.050	0.017	3.33E-03	0.012	0.026	6.53E-01
2.484009298	-0.032	0.011	3.69E-03	-0.004	0.017	8.32E-01
3.342922	-0.029	0.013	2.75E-02	-0.006	0.020	7.80E-01
3.149473	-0.029	0.010	5.14E-03	-0.023	0.016	1.51E-01
1.356623685	0.059	0.020	4.12E-03	0.035	0.031	2.54E-01
5.343686	0.055	0.020	5.49E-03	0.015	0.029	6.11E-01
2.135801566	-0.029	0.010	3.23E-03	-0.010	0.015	4.99E-01
3.410545	0.020	0.008	1.38E-02	0.036	0.012	2.27E-03
3.885251	0.017	0.007	1.61E-02	0.035	0.011	1.27E-03
3.005143	-0.011	0.004	6.46E-03	-0.004	0.006	4.54E-01
1.356287252	0.059	0.021	4.20E-03	0.035	0.031	2.58E-01
0.877880108	0.057	0.019	3.18E-03	0.031	0.029	2.78E-01
3.52325	0.023	0.009	8.86E-03	0.036	0.013	7.01E-03
2.472907022	-0.027	0.009	3.20E-03	-0.006	0.014	6.70E-01
3.416937	0.020	0.008	1.64E-02	0.039	0.012	2.00E-03
1.309523122	0.046	0.016	3.29E-03	0.016	0.023	5.01E-01
3.422993	0.020	0.008	1.28E-02	0.038	0.012	1.56E-03
3.424338	0.020	0.008	1.14E-02	0.036	0.012	2.75E-03
5.345705	0.056	0.020	4.54E-03	0.013	0.030	6.56E-01
6.086529	0.019	0.009	2.81E-02	0.017	0.013	1.99E-01
2.1051862	-0.016	0.007	2.33E-02	-0.004	0.011	7.23E-01
1.096224859	0.019	0.007	8.54E-03	-0.009	0.011	4.10E-01

3.738903	0.020	0.008	1.77E-02	0.040	0.013	1.50E-03
3.46471	0.021	0.009	1.80E-02	0.039	0.013	3.12E-03
3.229207	0.018	0.008	2.14E-02	0.027	0.012	1.94E-02
1.31053242	0.044	0.015	3.31E-03	0.020	0.023	3.77E-01
2.961744	-0.012	0.005	8.30E-03	-0.003	0.007	6.09E-01
3.409199	0.020	0.008	1.35E-02	0.033	0.012	5.34E-03
1.285972841	0.054	0.018	3.36E-03	0.022	0.027	4.29E-01
3.818301	0.016	0.007	1.52E-02	0.031	0.010	2.10E-03
1.312214583	0.042	0.014	2.72E-03	0.021	0.021	3.19E-01
5.359162	0.035	0.012	4.05E-03	0.015	0.018	3.99E-01
2.87999	-0.018	0.007	1.16E-02	-0.005	0.011	6.19E-01
2.643141913	-0.030	0.010	2.80E-03	0.000	0.015	9.92E-01
3.10069	-0.040	0.015	7.39E-03	-0.045	0.022	4.03E-02
1.56756692	0.069	0.024	3.78E-03	0.029	0.035	4.17E-01
3.49398	0.023	0.010	1.48E-02	0.037	0.014	9.37E-03
3.870784	-0.012	0.005	1.39E-02	0.002	0.007	8.14E-01
3.407853	0.020	0.008	1.78E-02	0.035	0.013	5.62E-03
2.955015	-0.012	0.005	7.63E-03	-0.007	0.007	3.37E-01
3.47514	0.024	0.009	1.07E-02	0.036	0.014	9.71E-03
1.013126009	0.035	0.012	2.79E-03	0.021	0.018	2.28E-01
3.005816	-0.010	0.004	8.27E-03	-0.003	0.006	5.77E-01
2.95737	-0.012	0.005	8.36E-03	-0.007	0.007	3.34E-01
1.266796183	0.053	0.018	3.83E-03	0.019	0.028	4.85E-01
3.100354	-0.040	0.015	7.57E-03	-0.045	0.022	4.11E-02
7.88308	-0.023	0.008	6.20E-03	0.008	0.012	5.10E-01
3.008171	-0.010	0.004	9.77E-03	-0.002	0.006	7.87E-01
1.295729386	0.056	0.019	3.76E-03	0.026	0.029	3.69E-01
2.11561561	-0.017	0.007	1.21E-02	-0.007	0.010	5.18E-01
3.611731	-0.022	0.009	8.90E-03	0.007	0.013	5.84E-01
1.396659163	0.019	0.006	2.26E-03	0.015	0.009	1.16E-01
1.566894054	0.068	0.024	3.85E-03	0.028	0.035	4.24E-01
0.694187913	0.043	0.015	5.34E-03	0.014	0.023	5.50E-01
1.211284805	0.016	0.006	1.13E-02	0.012	0.009	2.10E-01
3.54108	0.020	0.009	3.05E-02	0.036	0.014	8.62E-03
3.909811	0.021	0.009	1.86E-02	0.040	0.013	3.16E-03
1.113719354	0.019	0.007	7.96E-03	-0.004	0.011	7.40E-01
3.408526	0.020	0.008	1.67E-02	0.034	0.012	6.19E-03
1.060226572	0.017	0.007	1.65E-02	0.003	0.011	7.82E-01
1.309859555	0.045	0.015	3.44E-03	0.017	0.023	4.59E-01
6.739545	-0.030	0.010	2.61E-03	-0.008	0.015	5.74E-01
3.775238	0.021	0.008	1.00E-02	0.031	0.012	1.26E-02
2.474252753	-0.029	0.010	4.87E-03	0.000	0.015	9.87E-01
3.756397	0.027	0.010	1.05E-02	0.038	0.016	1.32E-02

3.448898	0.022	0.009	1.26E-02	0.036	0.013	7.82E-03
1.796677514	-0.023	0.010	2.66E-02	-0.004	0.016	7.93E-01
5.352097	0.052	0.018	4.59E-03	0.031	0.027	2.58E-01
5.343013	0.053	0.019	6.16E-03	0.014	0.029	6.36E-01
3.40819	0.020	0.008	1.84E-02	0.034	0.012	5.70E-03
0.69250575	0.040	0.015	6.32E-03	0.014	0.022	5.15E-01
0.87821654	0.056	0.019	3.37E-03	0.031	0.029	2.75E-01
1.351913629	0.059	0.021	4.45E-03	0.033	0.031	2.81E-01
1.347540005	0.059	0.021	4.25E-03	0.036	0.031	2.50E-01
1.286309273	0.054	0.018	3.52E-03	0.021	0.027	4.34E-01
2.489392219	-0.041	0.014	3.39E-03	-0.003	0.021	8.95E-01
1.35595082	0.058	0.021	4.51E-03	0.034	0.031	2.74E-01
3.078822	-0.016	0.006	8.02E-03	-0.007	0.009	4.44E-01
2.702017617	-0.021	0.006	9.31E-04	-0.022	0.009	1.90E-02
0.880235136	0.057	0.019	3.64E-03	0.034	0.029	2.48E-01
2.972173	-0.012	0.004	8.81E-03	-0.002	0.007	7.43E-01
3.731165	0.021	0.009	1.45E-02	0.033	0.013	1.04E-02
1.310195987	0.045	0.015	3.53E-03	0.019	0.023	4.14E-01
3.851944	0.017	0.007	1.85E-02	0.030	0.011	6.91E-03
1.607265965	0.026	0.009	3.20E-03	0.001	0.013	9.29E-01
2.482663567	-0.031	0.010	3.41E-03	-0.005	0.016	7.70E-01
5.349069	0.054	0.019	4.62E-03	0.022	0.029	4.51E-01
6.406477	0.021	0.009	1.66E-02	0.011	0.013	4.11E-01
2.483672865	-0.032	0.011	3.92E-03	-0.002	0.017	9.22E-01
1.179996575	0.016	0.005	3.20E-03	0.000	0.008	9.92E-01
3.812582	0.016	0.007	2.09E-02	0.031	0.011	3.10E-03
2.879318	-0.019	0.008	1.25E-02	-0.001	0.011	9.18E-01
0.933727918	-0.012	0.005	9.32E-03	-0.017	0.007	1.42E-02
5.186236	0.051	0.018	3.63E-03	0.014	0.026	6.01E-01
6.219757	0.020	0.009	2.56E-02	0.008	0.013	5.35E-01
5.344359	0.056	0.020	4.98E-03	0.019	0.030	5.12E-01
3.260159	0.022	0.009	1.02E-02	0.034	0.013	7.46E-03
1.018172498	0.027	0.009	2.79E-03	0.028	0.014	4.22E-02
1.17293149	0.012	0.004	1.92E-03	0.003	0.006	6.73E-01
7.740769	-0.026	0.011	2.28E-02	-0.008	0.017	6.47E-01
3.102372	-0.032	0.012	5.71E-03	-0.038	0.017	2.76E-02
7.239148	-0.021	0.007	5.12E-03	-0.006	0.011	5.60E-01
3.959603	-0.024	0.009	5.69E-03	-0.013	0.013	3.13E-01
3.718044	0.020	0.008	1.16E-02	0.029	0.012	1.42E-02
3.886597	0.018	0.007	1.33E-02	0.034	0.011	1.88E-03
8.487312	0.017	0.007	1.61E-02	0.014	0.010	1.73E-01
1.567903352	0.068	0.024	4.02E-03	0.027	0.035	4.38E-01
2.961407	-0.012	0.005	9.83E-03	-0.002	0.007	7.83E-01

2.962753	-0.012	0.005	9.86E-03	-0.003	0.007	7.01E-01
5.849008	0.021	0.008	8.88E-03	-0.003	0.012	8.32E-01
1.295392953	0.056	0.019	3.98E-03	0.026	0.029	3.75E-01
3.000097	-0.010	0.004	1.24E-02	0.002	0.006	6.82E-01
1.286645706	0.053	0.018	3.69E-03	0.021	0.027	4.35E-01
3.102709	-0.030	0.011	4.85E-03	-0.035	0.016	2.52E-02
3.342585	-0.029	0.013	2.66E-02	0.000	0.019	9.88E-01
3.906446	0.019	0.008	1.72E-02	0.037	0.012	2.03E-03
2.430852948	-0.021	0.008	8.95E-03	-0.014	0.012	2.27E-01
5.186572	0.052	0.018	3.94E-03	0.012	0.027	6.52E-01
3.818637	0.016	0.007	1.61E-02	0.030	0.010	2.56E-03
2.965444	-0.012	0.004	6.31E-03	-0.010	0.007	1.36E-01
2.137147296	-0.026	0.009	4.97E-03	-0.004	0.014	7.83E-01
0.873170051	0.050	0.017	3.96E-03	0.028	0.026	2.79E-01
7.055119	-0.022	0.009	1.25E-02	-0.019	0.013	1.34E-01
5.849345	0.023	0.009	1.35E-02	0.011	0.014	4.27E-01
2.988995	-0.010	0.004	1.48E-02	0.002	0.006	7.01E-01
3.230553	0.020	0.007	6.85E-03	0.038	0.011	5.70E-04
0.897393198	0.032	0.011	3.15E-03	0.002	0.016	9.08E-01
3.103382	-0.027	0.009	3.47E-03	-0.027	0.014	4.46E-02
2.880327	-0.017	0.007	1.46E-02	-0.005	0.011	6.43E-01
3.967677	-0.022	0.008	6.05E-03	-0.005	0.012	6.60E-01
8.698256	0.020	0.008	1.66E-02	-0.017	0.012	1.76E-01
0.693515048	0.040	0.014	5.14E-03	0.015	0.021	4.88E-01
2.702690482	-0.020	0.006	1.08E-03	-0.024	0.009	8.42E-03
3.812245	0.016	0.007	2.15E-02	0.031	0.011	3.56E-03
2.47257059	-0.024	0.009	7.30E-03	-0.007	0.013	5.73E-01
4.083746	0.045	0.015	2.32E-03	0.020	0.022	3.58E-01
7.713854	-0.030	0.010	3.24E-03	-0.021	0.015	1.71E-01
7.673818	0.020	0.008	1.01E-02	0.009	0.012	4.60E-01
5.498445	0.018	0.007	1.30E-02	-0.007	0.011	5.58E-01
7.760618	-0.027	0.009	2.68E-03	-0.032	0.014	1.85E-02
3.083869	-0.016	0.006	7.06E-03	0.003	0.009	7.50E-01
3.101027	-0.039	0.015	8.18E-03	-0.043	0.022	4.91E-02
5.756489	0.026	0.011	1.45E-02	-0.011	0.016	4.98E-01
3.465383	0.022	0.009	1.34E-02	0.034	0.014	1.09E-02
1.105981404	0.018	0.007	8.15E-03	0.000	0.010	9.99E-01
3.959266	-0.023	0.009	6.87E-03	-0.013	0.013	3.06E-01
2.966117	-0.012	0.004	9.47E-03	-0.004	0.007	5.18E-01
0.878552973	0.056	0.019	3.69E-03	0.031	0.029	2.77E-01
5.362527	0.037	0.013	4.33E-03	0.008	0.019	6.60E-01
5.385068	0.025	0.009	6.88E-03	0.014	0.014	3.35E-01
5.856746	0.017	0.007	1.63E-02	0.006	0.011	5.85E-01

1.295056521	0.055	0.019	4.18E-03	0.025	0.029	3.83E-01
1.286982138	0.053	0.018	3.87E-03	0.021	0.027	4.35E-01
3.096989	-0.024	0.008	2.71E-03	-0.032	0.012	7.55E-03
3.078486	-0.015	0.006	9.46E-03	-0.008	0.009	3.83E-01
7.859529	0.018	0.008	1.59E-02	0.012	0.011	2.79E-01
3.716362	0.021	0.008	1.07E-02	0.030	0.012	1.20E-02
8.386719	0.028	0.010	4.86E-03	0.018	0.015	2.12E-01
5.215506	0.015	0.006	8.13E-03	-0.001	0.008	8.99E-01
2.318484463	-0.014	0.006	1.92E-02	-0.008	0.009	3.78E-01
5.746396	0.026	0.011	1.80E-02	-0.005	0.017	7.58E-01
1.347203572	0.059	0.021	4.72E-03	0.035	0.031	2.54E-01
6.289735	0.019	0.008	2.19E-02	0.006	0.012	6.17E-01
5.22829	0.021	0.009	1.52E-02	0.046	0.013	4.09E-04
1.566557622	0.067	0.023	4.41E-03	0.027	0.035	4.43E-01
0.879898703	0.056	0.019	3.91E-03	0.033	0.029	2.58E-01
2.401583313	-0.022	0.009	1.81E-02	0.011	0.014	4.06E-01
3.740249	0.021	0.009	1.47E-02	0.039	0.013	2.53E-03
1.194463176	0.011	0.004	9.81E-03	0.007	0.006	2.46E-01
2.431525814	-0.019	0.008	1.37E-02	-0.019	0.011	9.63E-02
7.263034	-0.030	0.011	5.56E-03	-0.003	0.016	8.30E-01
6.67966	0.018	0.008	2.47E-02	0.026	0.012	3.15E-02
2.400237583	-0.022	0.010	2.57E-02	-0.002	0.015	9.06E-01
2.134455836	-0.020	0.009	2.17E-02	0.011	0.013	4.11E-01
6.984805	-0.022	0.008	9.80E-03	-0.001	0.012	9.47E-01
5.178498	0.044	0.015	3.77E-03	0.011	0.023	6.12E-01
4.06928	0.057	0.020	4.11E-03	0.028	0.030	3.39E-01
7.795607	-0.029	0.010	4.15E-03	-0.001	0.015	9.28E-01
2.954342	-0.012	0.005	9.42E-03	-0.005	0.007	4.33E-01
2.969818	-0.011	0.004	1.35E-02	-0.003	0.007	6.23E-01
8.951253	-0.025	0.010	1.20E-02	-0.017	0.015	2.46E-01
2.46617837	-0.024	0.009	1.13E-02	-0.025	0.014	7.76E-02
5.394824	0.025	0.010	9.04E-03	-0.004	0.014	7.96E-01
2.943576	-0.014	0.005	6.66E-03	-0.006	0.008	3.90E-01
5.344696	0.055	0.020	5.32E-03	0.018	0.030	5.39E-01
1.294720088	0.055	0.019	4.32E-03	0.025	0.029	3.89E-01
1.1833609	0.012	0.004	3.97E-03	0.004	0.006	5.16E-01
0.879562271	0.056	0.019	3.95E-03	0.032	0.029	2.63E-01
1.287318571	0.053	0.018	4.05E-03	0.021	0.028	4.39E-01
1.355614387	0.058	0.021	4.99E-03	0.033	0.031	2.79E-01
5.174461	0.040	0.014	3.38E-03	-0.007	0.020	7.38E-01
3.823684	0.018	0.008	1.61E-02	0.035	0.011	1.84E-03
2.252543675	0.028	0.010	4.42E-03	0.014	0.015	3.44E-01
0.878889405	0.056	0.019	3.91E-03	0.032	0.029	2.73E-01

4.068943	0.057	0.020	3.94E-03	0.026	0.030	3.89E-01
3.671616	-0.011	0.005	1.38E-02	-0.001	0.007	8.95E-01
1.287655004	0.053	0.018	4.14E-03	0.021	0.028	4.42E-01
0.879225838	0.056	0.019	3.98E-03	0.032	0.029	2.68E-01
7.229727	-0.028	0.011	1.06E-02	-0.002	0.016	8.86E-01
3.257468	0.021	0.009	2.02E-02	0.029	0.014	3.61E-02
8.082584	0.025	0.010	9.43E-03	0.021	0.014	1.32E-01
8.078883	0.018	0.007	1.31E-02	0.017	0.011	1.17E-01
4.069616	0.057	0.020	4.17E-03	0.027	0.030	3.69E-01
1.059890139	0.017	0.007	1.78E-02	-0.002	0.011	8.23E-01
1.353932224	0.058	0.021	5.07E-03	0.033	0.031	2.93E-01
2.973182	-0.011	0.004	1.40E-02	-0.003	0.007	7.03E-01
1.108	0.016	0.006	8.51E-03	0.009	0.009	2.84E-01
3.60904	-0.017	0.007	1.57E-02	-0.005	0.010	6.59E-01
5.360508	0.036	0.013	6.26E-03	0.011	0.019	5.75E-01
1.566221189	0.066	0.023	4.82E-03	0.026	0.035	4.48E-01
2.381397357	-0.019	0.007	8.20E-03	-0.001	0.011	8.88E-01
1.211621238	0.015	0.006	1.32E-02	0.011	0.009	2.28E-01
5.185899	0.048	0.017	4.50E-03	0.011	0.025	6.50E-01
5.349406	0.054	0.019	5.55E-03	0.019	0.029	5.03E-01
1.294383655	0.054	0.019	4.49E-03	0.024	0.029	3.97E-01
5.234682	0.020	0.008	1.72E-02	0.044	0.013	5.30E-04
1.352250061	0.058	0.021	5.19E-03	0.033	0.031	2.88E-01
2.226974798	0.064	0.025	1.16E-02	0.042	0.037	2.51E-01
1.34686714	0.058	0.021	5.10E-03	0.035	0.031	2.63E-01
1.848824566	-0.019	0.008	2.27E-02	-0.015	0.012	2.11E-01
5.345032	0.055	0.020	5.98E-03	0.018	0.030	5.53E-01
7.783832	-0.026	0.009	5.37E-03	-0.003	0.014	8.47E-01
5.238046	0.017	0.008	3.01E-02	0.017	0.012	1.48E-01
2.975874	-0.011	0.004	1.35E-02	0.000	0.006	9.55E-01
3.757407	0.022	0.009	1.21E-02	0.032	0.013	1.42E-02
2.401919746	-0.022	0.009	2.12E-02	0.017	0.014	2.28E-01
3.712661	0.019	0.008	1.37E-02	0.037	0.011	1.42E-03
3.269916	-0.030	0.013	2.63E-02	-0.024	0.020	2.34E-01
2.707400538	-0.019	0.006	1.39E-03	-0.024	0.009	5.84E-03
4.273158	0.043	0.015	4.76E-03	0.020	0.023	3.79E-01
2.125372156	-0.024	0.008	4.02E-03	0.002	0.013	8.50E-01
3.410208	0.019	0.008	1.58E-02	0.034	0.012	3.90E-03
5.742359	0.022	0.009	1.39E-02	0.007	0.013	5.70E-01
3.601638	-0.018	0.007	8.36E-03	-0.003	0.010	7.29E-01
3.103045	-0.028	0.010	4.67E-03	-0.029	0.015	4.51E-02
2.316465867	-0.013	0.005	1.02E-02	-0.017	0.008	2.64E-02
1.149381209	0.017	0.006	4.60E-03	-0.003	0.009	7.51E-01

3.422656	0.020	0.008	1.56E-02	0.037	0.012	2.17E-03
5.345368	0.053	0.019	6.03E-03	0.012	0.029	6.87E-01
1.287991436	0.053	0.018	4.34E-03	0.021	0.028	4.43E-01
5.215169	0.014	0.006	1.17E-02	-0.006	0.008	4.98E-01
7.750189	-0.022	0.010	2.83E-02	0.000	0.015	9.94E-01
3.710979	0.020	0.008	1.37E-02	0.037	0.012	1.97E-03
3.838487	0.019	0.008	1.76E-02	0.034	0.012	4.61E-03
3.778938	0.022	0.008	7.18E-03	0.034	0.012	5.89E-03
4.083073	0.047	0.016	2.92E-03	0.025	0.023	2.87E-01
1.294047223	0.054	0.019	4.61E-03	0.024	0.029	4.02E-01
3.428376	0.022	0.009	1.27E-02	0.034	0.013	8.39E-03
6.963609	-0.029	0.010	5.96E-03	-0.004	0.015	7.83E-01
2.962416	-0.012	0.005	1.03E-02	-0.002	0.007	7.32E-01
3.448562	0.021	0.009	1.80E-02	0.038	0.013	3.86E-03
2.488719354	-0.039	0.014	4.88E-03	0.002	0.021	9.06E-01
3.817965	0.016	0.007	1.89E-02	0.032	0.010	1.13E-03
1.352586494	0.058	0.021	5.37E-03	0.032	0.031	2.96E-01
3.004134	-0.011	0.004	5.68E-03	-0.002	0.006	7.42E-01
6.720368	-0.025	0.010	1.04E-02	-0.010	0.014	4.91E-01
3.432076	0.020	0.009	1.98E-02	0.034	0.013	9.99E-03
1.26645975	0.051	0.018	4.85E-03	0.018	0.027	5.19E-01
0.872833619	0.049	0.017	4.68E-03	0.027	0.026	2.91E-01
5.847662	0.024	0.010	1.32E-02	-0.018	0.014	2.09E-01
5.368582	0.028	0.010	6.35E-03	0.005	0.015	7.26E-01
2.967126	-0.012	0.005	6.80E-03	-0.007	0.007	2.95E-01
3.445197	0.023	0.009	1.37E-02	0.033	0.014	1.76E-02
1.354941522	0.058	0.021	5.34E-03	0.031	0.031	3.13E-01
2.960734	-0.012	0.005	1.01E-02	-0.007	0.007	3.30E-01
2.490401517	-0.037	0.014	8.90E-03	-0.009	0.021	6.57E-01
5.362863	0.042	0.015	4.34E-03	0.015	0.022	5.00E-01
3.143081	-0.026	0.010	7.71E-03	-0.028	0.015	5.75E-02
1.095551994	0.018	0.008	1.68E-02	0.004	0.011	7.22E-01
5.848672	0.022	0.009	1.40E-02	0.007	0.013	5.84E-01
5.348733	0.054	0.020	5.97E-03	0.029	0.029	3.15E-01
2.974192	-0.012	0.004	8.44E-03	-0.003	0.007	6.62E-01
2.969145	-0.011	0.004	1.12E-02	-0.002	0.007	7.33E-01
1.354268657	0.058	0.021	5.44E-03	0.033	0.031	2.93E-01
5.393142	0.018	0.007	9.67E-03	-0.011	0.011	3.05E-01
7.780131	-0.025	0.010	8.78E-03	-0.007	0.014	6.42E-01
3.07714	-0.016	0.006	1.05E-02	-0.008	0.009	3.72E-01
1.202201126	0.011	0.005	1.62E-02	0.009	0.007	2.12E-01
5.363199	0.047	0.016	4.09E-03	0.027	0.024	2.69E-01
1.288327869	0.053	0.019	4.52E-03	0.021	0.028	4.41E-01

1.607602398	0.025	0.009	3.89E-03	0.004	0.013	7.82E-01
6.500005	0.028	0.010	6.05E-03	-0.008	0.015	6.13E-01
2.31814803	-0.013	0.006	1.84E-02	-0.006	0.008	4.58E-01
2.473579887	-0.026	0.010	8.38E-03	-0.004	0.015	7.72E-01
3.093625	-0.022	0.008	5.04E-03	-0.014	0.012	2.35E-01
3.451926	0.020	0.009	2.17E-02	0.035	0.013	6.10E-03
3.014563	-0.012	0.005	8.02E-03	-0.004	0.007	5.26E-01
3.602311	-0.018	0.007	9.91E-03	-0.008	0.010	4.21E-01
5.177152	0.044	0.015	4.28E-03	0.001	0.023	9.70E-01
3.852281	0.017	0.007	2.03E-02	0.030	0.011	6.89E-03
3.815273	0.018	0.008	1.82E-02	0.034	0.011	2.61E-03
0.696542941	0.041	0.014	4.22E-03	0.015	0.022	4.95E-01
1.60793883	0.025	0.009	3.42E-03	0.003	0.013	8.19E-01
7.054782	-0.026	0.010	1.27E-02	-0.041	0.015	7.94E-03
2.628338879	-0.020	0.008	1.70E-02	-0.021	0.012	9.89E-02
3.699877	0.019	0.008	1.46E-02	0.028	0.012	1.89E-02
1.787930267	-0.025	0.011	1.80E-02	0.003	0.016	8.45E-01
6.499669	0.025	0.010	1.29E-02	-0.006	0.015	6.94E-01
6.167273	0.018	0.008	2.01E-02	0.008	0.012	4.79E-01
1.29371079	0.054	0.019	4.84E-03	0.023	0.028	4.09E-01
7.06824	-0.023	0.009	8.16E-03	-0.005	0.013	6.88E-01
2.694952532	-0.019	0.006	1.90E-03	-0.011	0.009	2.26E-01
1.288664301	0.053	0.019	4.65E-03	0.021	0.028	4.41E-01
1.355277954	0.057	0.021	5.52E-03	0.032	0.031	3.05E-01
7.766674	-0.024	0.008	3.54E-03	-0.034	0.012	5.01E-03
1.354605089	0.057	0.021	5.66E-03	0.031	0.031	3.09E-01
1.353595792	0.058	0.021	5.50E-03	0.031	0.031	3.10E-01
1.346530707	0.058	0.021	5.48E-03	0.033	0.031	2.81E-01
3.479177	0.022	0.010	2.20E-02	0.039	0.014	6.90E-03
6.738872	-0.029	0.011	7.26E-03	-0.016	0.016	3.16E-01
8.894732	0.019	0.008	1.71E-02	0.009	0.012	4.61E-01
3.102036	-0.033	0.013	8.31E-03	-0.038	0.019	4.25E-02
1.934951309	0.014	0.005	7.98E-03	0.009	0.008	2.58E-01
3.096653	-0.023	0.008	4.11E-03	-0.030	0.012	1.09E-02
2.971164	-0.012	0.004	7.01E-03	0.000	0.007	9.70E-01
3.601302	-0.017	0.007	1.03E-02	-0.004	0.010	7.20E-01
7.376412	-0.019	0.008	2.41E-02	-0.022	0.012	7.19E-02
3.011536	-0.013	0.005	5.64E-03	-0.007	0.007	2.80E-01
1.608275263	0.025	0.008	3.54E-03	0.003	0.013	8.27E-01
2.973855	-0.012	0.004	8.28E-03	-0.004	0.007	5.74E-01
5.71208	0.021	0.009	1.32E-02	0.006	0.013	6.18E-01
2.990004	-0.010	0.004	1.02E-02	0.003	0.006	5.71E-01
5.672717	0.019	0.008	1.22E-02	0.004	0.011	7.15E-01

3.888952	0.017	0.007	1.93E-02	0.035	0.011	1.32E-03
4.069953	0.056	0.020	4.34E-03	0.026	0.029	3.83E-01
3.409535	0.019	0.008	1.79E-02	0.033	0.012	5.93E-03
5.747405	0.025	0.011	1.98E-02	0.011	0.016	4.87E-01
1.194799609	0.010	0.004	1.18E-02	0.007	0.006	2.36E-01
4.273494	0.043	0.015	5.25E-03	0.019	0.023	3.94E-01
1.194126743	0.010	0.004	1.07E-02	0.006	0.006	2.96E-01
3.394732	0.020	0.009	2.18E-02	0.035	0.013	6.05E-03
4.068607	0.056	0.020	4.53E-03	0.023	0.029	4.40E-01
2.622955958	-0.028	0.013	2.62E-02	0.005	0.019	8.07E-01
5.495417	0.020	0.008	1.11E-02	0.009	0.012	4.51E-01
1.390939809	0.021	0.007	4.44E-03	0.016	0.011	1.46E-01
2.483336433	-0.031	0.011	4.64E-03	0.003	0.016	8.70E-01
1.289000734	0.052	0.019	4.83E-03	0.021	0.028	4.44E-01
6.257774	0.019	0.008	1.21E-02	0.004	0.011	7.23E-01
3.34124	-0.025	0.011	2.71E-02	0.006	0.017	7.43E-01
7.324938	-0.018	0.007	1.15E-02	0.000	0.010	9.85E-01
3.508447	0.020	0.009	2.12E-02	0.035	0.013	7.91E-03
0.897729631	0.030	0.011	4.01E-03	0.000	0.016	9.77E-01
2.703363347	-0.019	0.006	1.39E-03	-0.024	0.009	7.41E-03
1.293374358	0.053	0.019	5.03E-03	0.023	0.028	4.18E-01
2.961071	-0.011	0.005	1.24E-02	-0.007	0.007	3.14E-01
5.365891	0.041	0.015	6.36E-03	0.012	0.023	5.82E-01
1.193790311	0.010	0.004	1.13E-02	0.006	0.006	3.00E-01
2.707736971	-0.019	0.006	1.67E-03	-0.023	0.009	1.05E-02
2.104513335	-0.015	0.007	2.45E-02	0.003	0.010	7.62E-01
3.008844	-0.010	0.004	8.14E-03	-0.005	0.006	4.23E-01
1.901644483	-0.015	0.006	8.90E-03	-0.008	0.009	3.84E-01
2.706391241	-0.018	0.006	1.55E-03	-0.026	0.009	2.36E-03
2.3168023	-0.012	0.005	1.64E-02	-0.014	0.008	7.25E-02
5.395161	0.022	0.009	1.16E-02	0.002	0.013	8.72E-01
7.311481	-0.025	0.009	4.92E-03	0.003	0.014	8.53E-01
3.465047	0.021	0.009	2.04E-02	0.037	0.013	6.33E-03
2.108214093	-0.014	0.006	2.39E-02	-0.022	0.009	2.24E-02
1.781538047	-0.024	0.010	1.80E-02	-0.011	0.015	4.66E-01
6.413878	0.017	0.007	1.86E-02	-0.006	0.011	5.57E-01
3.468411	0.020	0.009	2.69E-02	0.038	0.013	4.84E-03
1.289337167	0.052	0.019	4.97E-03	0.021	0.028	4.49E-01
5.863475	0.021	0.009	2.23E-02	-0.003	0.013	8.07E-01
2.879654	-0.018	0.007	1.75E-02	-0.007	0.011	5.08E-01
1.346194275	0.057	0.021	5.83E-03	0.033	0.031	2.89E-01
2.434217274	-0.020	0.008	1.74E-02	-0.026	0.012	3.66E-02
5.21315	0.016	0.006	1.51E-02	-0.008	0.010	4.19E-01

3.409872	0.019	0.008	1.83E-02	0.033	0.012	5.49E-03
4.06827	0.054	0.019	4.93E-03	0.024	0.029	3.95E-01
3.970032	-0.021	0.008	7.24E-03	-0.013	0.012	2.82E-01
1.289673599	0.052	0.019	5.03E-03	0.021	0.028	4.47E-01
2.104176902	-0.014	0.006	2.58E-02	0.006	0.009	5.43E-01
2.920026	-0.018	0.007	6.87E-03	-0.012	0.010	2.42E-01
1.6065931	0.026	0.009	4.32E-03	0.005	0.013	6.94E-01
5.364882	0.036	0.013	5.81E-03	0.023	0.020	2.40E-01
6.406813	0.019	0.007	1.13E-02	0.008	0.011	4.93E-01
5.218197	0.009	0.005	5.79E-02	0.004	0.007	6.17E-01
1.293037925	0.053	0.019	5.21E-03	0.023	0.028	4.24E-01
2.958379	-0.011	0.005	1.45E-02	-0.004	0.007	5.21E-01
4.070625	0.055	0.019	4.61E-03	0.020	0.029	4.78E-01
3.093962	-0.022	0.008	5.16E-03	-0.015	0.012	2.20E-01
3.286065	-0.035	0.013	8.43E-03	-0.023	0.020	2.41E-01
2.707064106	-0.018	0.006	1.59E-03	-0.024	0.009	4.28E-03
1.292701493	0.053	0.019	5.22E-03	0.022	0.028	4.30E-01
1.352922926	0.057	0.021	5.96E-03	0.031	0.031	3.14E-01
3.964313	-0.021	0.008	6.87E-03	0.006	0.012	6.26E-01
5.218533	0.010	0.005	5.21E-02	-0.001	0.007	9.31E-01
3.818974	0.016	0.007	2.04E-02	0.031	0.010	2.57E-03
1.565884757	0.064	0.023	5.66E-03	0.025	0.035	4.71E-01
3.141735	-0.035	0.013	7.10E-03	-0.030	0.020	1.20E-01
3.096317	-0.022	0.008	5.27E-03	-0.025	0.012	3.54E-02
5.387423	0.018	0.007	6.96E-03	0.003	0.010	7.93E-01
5.363872	0.042	0.015	4.83E-03	0.015	0.022	5.10E-01
1.148035478	0.017	0.006	5.85E-03	-0.003	0.009	7.11E-01
2.115279178	-0.016	0.007	2.24E-02	-0.010	0.011	3.28E-01
1.940670663	0.016	0.006	6.57E-03	0.015	0.009	8.77E-02
2.395191094	-0.025	0.010	1.66E-02	-0.017	0.016	2.73E-01
3.0149	-0.012	0.004	7.72E-03	-0.004	0.007	5.35E-01
3.529305	0.022	0.009	1.40E-02	0.039	0.013	3.75E-03
3.871121	-0.011	0.005	1.81E-02	0.003	0.007	6.49E-01
3.825702	0.017	0.007	1.73E-02	0.032	0.011	2.18E-03
5.844634	0.022	0.010	2.67E-02	0.007	0.015	6.20E-01
1.290010032	0.052	0.019	5.19E-03	0.021	0.028	4.47E-01
3.418955	0.022	0.009	1.66E-02	0.037	0.014	8.48E-03
2.948959	-0.013	0.005	1.10E-02	-0.005	0.007	4.73E-01
1.29236506	0.053	0.019	5.29E-03	0.022	0.028	4.33E-01
1.312551015	0.039	0.014	3.79E-03	0.020	0.020	3.32E-01
1.353259359	0.057	0.021	6.06E-03	0.031	0.031	3.15E-01
3.809217	0.017	0.007	1.94E-02	0.024	0.011	2.63E-02
1.399687057	0.015	0.005	3.62E-03	0.014	0.007	5.41E-02

1.930914118	0.012	0.005	1.16E-02	0.011	0.007	9.98E-02
1.195136041	0.010	0.004	1.31E-02	0.008	0.006	1.90E-01
2.703026915	-0.019	0.006	1.50E-03	-0.024	0.009	8.03E-03
2.402256178	-0.022	0.010	2.16E-02	0.018	0.014	2.17E-01
1.211957671	0.015	0.006	1.55E-02	0.011	0.009	2.38E-01
5.358489	0.043	0.015	4.15E-03	0.028	0.022	2.06E-01
8.612129	0.022	0.009	1.69E-02	0.018	0.014	1.81E-01
5.627635	0.015	0.007	2.29E-02	-0.012	0.010	2.51E-01
2.495448006	-0.036	0.014	1.27E-02	-0.008	0.021	7.15E-01
1.292028627	0.052	0.019	5.40E-03	0.022	0.028	4.35E-01
2.21553609	0.063	0.023	5.54E-03	0.018	0.034	6.07E-01
1.291692195	0.052	0.019	5.41E-03	0.022	0.028	4.38E-01
5.708716	0.020	0.009	2.18E-02	-0.001	0.013	9.24E-01
3.084878	-0.016	0.007	1.22E-02	0.003	0.010	7.64E-01
6.432382	0.023	0.009	1.60E-02	0.019	0.014	1.88E-01
3.473458	0.020	0.009	2.71E-02	0.041	0.013	2.36E-03
1.345857842	0.057	0.021	6.15E-03	0.033	0.031	2.86E-01
1.290346464	0.052	0.019	5.38E-03	0.021	0.028	4.48E-01
1.291355762	0.052	0.019	5.43E-03	0.022	0.028	4.41E-01
3.53267	0.020	0.007	9.08E-03	0.030	0.011	7.36E-03
5.83185	0.021	0.009	2.39E-02	0.005	0.014	6.93E-01
3.840842	0.018	0.007	1.55E-02	0.032	0.011	2.85E-03
3.838823	0.018	0.008	2.01E-02	0.033	0.011	4.47E-03
3.023984	-0.013	0.005	8.44E-03	-0.011	0.007	1.41E-01
1.290682897	0.052	0.019	5.42E-03	0.021	0.028	4.45E-01
3.921249	0.029	0.012	1.42E-02	0.055	0.018	2.03E-03
3.697522	0.017	0.007	2.09E-02	0.031	0.011	3.83E-03
3.841178	0.018	0.007	1.54E-02	0.032	0.011	3.82E-03
8.118919	-0.022	0.008	7.96E-03	-0.001	0.012	9.25E-01
1.29101933	0.052	0.019	5.46E-03	0.021	0.028	4.44E-01
3.241655	0.016	0.008	4.20E-02	0.028	0.011	1.55E-02
3.003461	-0.011	0.004	7.10E-03	-0.001	0.006	8.35E-01
1.399350624	0.015	0.005	3.42E-03	0.014	0.007	6.60E-02
1.072338145	0.019	0.008	1.70E-02	0.000	0.012	9.91E-01
3.67128	-0.011	0.005	1.65E-02	0.000	0.007	9.88E-01
2.215872523	0.063	0.023	5.53E-03	0.020	0.034	5.65E-01
2.252880108	0.026	0.010	5.97E-03	0.014	0.014	3.34E-01
2.6946161	-0.019	0.006	2.11E-03	-0.010	0.009	2.72E-01
0.872497186	0.047	0.017	5.70E-03	0.026	0.025	3.05E-01
7.100537	-0.023	0.011	2.79E-02	-0.014	0.016	3.86E-01
2.252207242	0.027	0.010	5.59E-03	0.013	0.015	3.86E-01
4.07399	0.058	0.021	5.18E-03	0.027	0.031	3.82E-01
2.135465133	-0.028	0.010	7.00E-03	-0.005	0.015	7.21E-01

1.877084903	-0.014	0.006	2.38E-02	-0.012	0.009	1.91E-01
2.70504551	-0.018	0.006	1.86E-03	-0.024	0.009	5.30E-03
3.539062	0.023	0.009	8.34E-03	0.030	0.013	2.14E-02
3.720063	0.021	0.008	1.45E-02	0.033	0.013	9.09E-03
7.860202	0.022	0.010	2.26E-02	0.018	0.014	2.15E-01
2.226638366	0.061	0.025	1.44E-02	0.040	0.036	2.68E-01
7.221989	-0.022	0.009	1.11E-02	-0.032	0.013	1.45E-02
2.118307071	-0.019	0.008	1.18E-02	-0.009	0.011	4.56E-01
1.402042085	0.013	0.005	5.44E-03	0.013	0.007	7.02E-02
3.271934	-0.026	0.011	2.22E-02	-0.003	0.017	8.48E-01
5.948592	0.022	0.008	8.40E-03	0.008	0.013	5.28E-01
3.494316	0.022	0.010	2.06E-02	0.036	0.014	1.22E-02
3.060655	-0.017	0.006	7.69E-03	-0.007	0.009	4.55E-01
2.968809	-0.011	0.004	1.29E-02	-0.001	0.007	8.47E-01
0.964343284	0.013	0.005	9.02E-03	0.010	0.007	1.69E-01
5.537471	0.019	0.007	1.01E-02	0.007	0.011	5.36E-01
2.216208955	0.063	0.023	5.75E-03	0.019	0.034	5.73E-01
3.83916	0.018	0.007	1.86E-02	0.032	0.011	4.19E-03
2.216545388	0.063	0.023	5.82E-03	0.017	0.034	6.08E-01
2.988658	-0.010	0.004	1.93E-02	0.003	0.006	5.85E-01
1.012789577	0.033	0.012	4.88E-03	0.019	0.017	2.63E-01
1.147699046	0.017	0.006	5.80E-03	-0.003	0.009	7.57E-01
1.179660142	0.016	0.006	5.18E-03	-0.001	0.009	9.00E-01
2.888738	-0.015	0.006	1.58E-02	-0.004	0.010	6.41E-01
3.145099	-0.040	0.015	7.65E-03	-0.041	0.022	6.55E-02
7.769365	-0.024	0.009	7.04E-03	-0.015	0.013	2.76E-01
1.345521409	0.056	0.021	6.50E-03	0.032	0.031	2.91E-01
1.931250551	0.012	0.005	8.80E-03	0.011	0.007	9.50E-02
3.711315	0.018	0.008	1.73E-02	0.035	0.011	2.21E-03
8.046586	0.017	0.008	2.33E-02	0.013	0.011	2.68E-01
3.469084	0.019	0.009	3.14E-02	0.038	0.013	3.85E-03
2.392499633	-0.019	0.008	1.43E-02	-0.005	0.012	6.85E-01
1.391276242	0.021	0.007	4.57E-03	0.016	0.011	1.49E-01
3.150146	-0.024	0.010	1.12E-02	-0.013	0.014	3.66E-01
7.860539	0.021	0.010	2.95E-02	0.020	0.014	1.67E-01
6.086866	0.018	0.009	3.64E-02	0.021	0.013	1.09E-01
1.565548324	0.064	0.023	6.20E-03	0.024	0.035	4.86E-01
3.417273	0.019	0.008	2.59E-02	0.039	0.013	2.30E-03
0.919261316	-0.010	0.004	1.54E-02	-0.018	0.006	3.53E-03
7.173207	-0.026	0.011	2.23E-02	-0.014	0.017	4.18E-01
3.839496	0.017	0.007	1.76E-02	0.030	0.011	4.95E-03
3.711988	0.018	0.007	1.58E-02	0.035	0.011	1.53E-03
5.34335	0.051	0.020	9.61E-03	0.016	0.030	5.86E-01

4.074326	0.058	0.021	5.53E-03	0.028	0.031	3.75E-01
1.401369219	0.014	0.005	4.99E-03	0.015	0.007	3.59E-02
2.864515	-0.018	0.008	3.46E-02	0.001	0.013	9.50E-01
7.071268	-0.024	0.009	6.95E-03	-0.018	0.013	1.84E-01
2.21419036	0.062	0.023	6.28E-03	0.019	0.034	5.81E-01
1.112037191	0.016	0.007	1.77E-02	0.001	0.010	9.25E-01
2.969482	-0.011	0.004	1.42E-02	-0.001	0.007	9.26E-01
3.008508	-0.010	0.004	1.17E-02	-0.002	0.006	6.70E-01
3.885587	0.015	0.007	2.59E-02	0.033	0.010	1.50E-03
3.84858	0.020	0.008	1.90E-02	0.034	0.012	6.37E-03
2.215199657	0.063	0.023	6.14E-03	0.018	0.034	6.08E-01
7.712845	-0.027	0.010	8.13E-03	-0.021	0.015	1.68E-01
6.613046	0.022	0.010	2.99E-02	-0.004	0.015	7.71E-01
1.639899927	0.015	0.006	1.23E-02	0.008	0.009	3.53E-01
2.10384047	-0.013	0.006	3.29E-02	0.009	0.009	3.14E-01
1.212294103	0.015	0.006	1.67E-02	0.011	0.009	2.48E-01
1.266123318	0.049	0.018	6.30E-03	0.016	0.027	5.61E-01
5.232327	0.015	0.007	3.58E-02	0.024	0.011	2.56E-02
6.9192	-0.023	0.009	1.04E-02	0.005	0.013	7.02E-01
3.712325	0.018	0.007	1.64E-02	0.035	0.011	1.58E-03
3.340903	-0.023	0.011	3.32E-02	0.004	0.016	8.16E-01
1.565211891	0.063	0.023	6.46E-03	0.024	0.035	4.89E-01
3.004471	-0.011	0.004	8.01E-03	-0.002	0.006	7.49E-01
5.401553	0.017	0.007	1.71E-02	-0.006	0.010	5.56E-01
2.213853927	0.062	0.023	6.32E-03	0.019	0.034	5.81E-01
2.486027893	-0.033	0.011	3.60E-03	-0.010	0.017	5.39E-01
2.702354049	-0.020	0.006	1.64E-03	-0.023	0.009	1.55E-02
5.373292	0.024	0.009	9.62E-03	0.001	0.014	9.42E-01
8.809615	0.024	0.010	1.16E-02	0.017	0.014	2.22E-01
3.820992	0.016	0.007	2.27E-02	0.033	0.011	1.89E-03
2.704709078	-0.018	0.006	2.06E-03	-0.023	0.009	9.81E-03
3.003798	-0.011	0.004	8.17E-03	-0.001	0.006	8.60E-01
1.013462442	0.033	0.012	5.49E-03	0.019	0.018	2.78E-01
2.976883	-0.011	0.004	1.19E-02	0.001	0.006	9.07E-01
3.034749	0.022	0.010	3.13E-02	0.026	0.015	9.19E-02
4.271476	0.042	0.016	6.47E-03	0.022	0.023	3.54E-01
1.106990702	0.016	0.006	1.42E-02	-0.002	0.010	8.28E-01
2.213517494	0.062	0.023	6.50E-03	0.019	0.034	5.75E-01
3.854972	0.017	0.008	2.91E-02	0.032	0.012	6.30E-03
2.487037191	-0.034	0.012	5.66E-03	-0.005	0.018	7.69E-01
3.399106	0.018	0.008	3.49E-02	0.032	0.013	9.53E-03
5.389441	0.019	0.008	1.10E-02	0.016	0.011	1.51E-01
7.394243	0.017	0.007	1.65E-02	-0.003	0.010	7.93E-01

3.029703	-0.011	0.005	2.19E-02	-0.005	0.007	4.29E-01
3.673299	-0.011	0.005	1.57E-02	0.002	0.007	8.17E-01
7.827905	-0.026	0.010	7.98E-03	0.008	0.014	5.96E-01
1.564539026	0.063	0.023	6.49E-03	0.023	0.034	5.07E-01
6.615738	0.018	0.008	2.05E-02	0.004	0.012	7.47E-01
6.16761	0.014	0.007	4.02E-02	0.010	0.010	2.86E-01
3.711652	0.018	0.007	1.74E-02	0.034	0.011	1.86E-03
3.260496	0.020	0.008	1.69E-02	0.032	0.012	1.13E-02
1.946053585	0.015	0.006	8.18E-03	0.008	0.009	3.56E-01
5.39617	0.020	0.008	1.39E-02	-0.004	0.012	7.27E-01
2.214863225	0.062	0.023	6.47E-03	0.018	0.034	5.97E-01
1.938315635	0.014	0.005	8.70E-03	0.011	0.008	1.65E-01
1.934614876	0.013	0.005	1.12E-02	0.008	0.008	3.08E-01
1.122803034	0.016	0.006	1.29E-02	0.005	0.010	6.11E-01
2.110232689	-0.014	0.007	4.29E-02	-0.028	0.010	4.59E-03
5.178161	0.044	0.016	4.55E-03	0.009	0.023	6.99E-01
2.214526792	0.062	0.023	6.55E-03	0.020	0.034	5.66E-01
8.12733	0.016	0.008	4.37E-02	0.012	0.011	3.15E-01
5.993001	0.019	0.008	2.51E-02	-0.020	0.012	1.12E-01
4.267438	0.043	0.015	5.72E-03	0.031	0.023	1.75E-01
7.359254	-0.022	0.010	2.42E-02	-0.023	0.014	1.19E-01
5.411646	0.021	0.009	1.38E-02	0.007	0.013	5.78E-01
6.7853	-0.021	0.008	1.14E-02	-0.008	0.012	5.17E-01
5.398861	0.018	0.007	1.05E-02	-0.001	0.011	9.10E-01
2.26095449	0.016	0.006	1.00E-02	0.009	0.009	3.24E-01
2.865524	-0.019	0.008	2.61E-02	0.007	0.013	5.82E-01
3.612068	-0.021	0.009	1.51E-02	0.007	0.013	5.62E-01
5.857082	0.019	0.008	1.27E-02	0.008	0.011	4.76E-01
2.103504037	-0.012	0.006	3.60E-02	0.008	0.009	3.48E-01
3.427703	0.020	0.009	1.87E-02	0.038	0.013	3.55E-03
1.564875459	0.062	0.023	6.78E-03	0.023	0.034	5.03E-01
2.920699	-0.018	0.007	6.38E-03	-0.010	0.010	3.29E-01
3.602648	-0.017	0.007	1.40E-02	-0.009	0.010	3.84E-01
2.979238	-0.010	0.004	1.56E-02	0.008	0.006	2.14E-01
1.564202594	0.062	0.023	6.78E-03	0.021	0.034	5.37E-01
3.428039	0.021	0.009	1.81E-02	0.036	0.013	5.35E-03
1.345184977	0.055	0.021	7.09E-03	0.031	0.031	3.11E-01
5.378002	0.025	0.010	8.70E-03	0.015	0.014	2.90E-01
1.099589185	0.020	0.008	1.02E-02	-0.002	0.012	8.62E-01
7.779795	-0.021	0.008	1.35E-02	-0.005	0.012	6.64E-01
2.706727673	-0.018	0.006	1.90E-03	-0.025	0.009	3.39E-03
2.491074382	-0.042	0.015	5.95E-03	-0.019	0.023	4.06E-01
5.392469	0.018	0.008	1.87E-02	0.003	0.011	8.25E-01

7.640848	-0.022	0.010	3.63E-02	-0.022	0.016	1.60E-01
3.95792	-0.019	0.008	1.95E-02	-0.028	0.012	2.42E-02
3.471103	0.021	0.009	2.17E-02	0.035	0.014	1.11E-02
3.866411	-0.011	0.005	2.14E-02	0.003	0.007	6.59E-01
3.269579	-0.028	0.013	2.99E-02	-0.026	0.019	1.76E-01
2.975201	-0.010	0.004	1.61E-02	-0.003	0.006	6.93E-01
8.103779	0.022	0.009	1.24E-02	0.016	0.013	2.29E-01
3.1017	-0.035	0.013	9.97E-03	-0.039	0.020	5.24E-02
1.175622951	0.017	0.006	3.65E-03	-0.002	0.009	8.38E-01
1.344848544	0.055	0.020	7.23E-03	0.030	0.031	3.26E-01
2.693270369	-0.019	0.006	2.39E-03	-0.009	0.009	3.35E-01
3.767163	0.030	0.010	3.38E-03	0.040	0.015	8.10E-03
3.840506	0.017	0.007	1.86E-02	0.032	0.011	3.16E-03
1.843105212	-0.024	0.010	1.52E-02	0.007	0.015	6.40E-01
2.430516516	-0.021	0.008	1.19E-02	-0.015	0.013	2.32E-01
2.980247	-0.010	0.004	2.38E-02	0.006	0.006	3.46E-01
1.104972107	0.019	0.007	9.55E-03	0.001	0.011	9.53E-01
8.02169	0.027	0.010	5.24E-03	0.005	0.014	7.26E-01
5.213487	0.015	0.006	1.96E-02	-0.004	0.009	6.49E-01
2.213181062	0.061	0.023	6.84E-03	0.019	0.034	5.79E-01
2.316129435	-0.013	0.005	1.14E-02	-0.016	0.008	3.35E-02
7.214252	-0.026	0.010	9.48E-03	-0.010	0.015	5.20E-01
3.101363	-0.036	0.014	1.12E-02	-0.040	0.021	5.65E-02
7.827232	-0.026	0.010	6.58E-03	-0.004	0.014	7.90E-01
1.070655982	0.017	0.007	2.02E-02	0.003	0.011	7.74E-01
2.888065	-0.016	0.007	1.63E-02	-0.003	0.010	7.46E-01
1.013798874	0.031	0.012	7.32E-03	0.023	0.017	1.78E-01
7.164796	-0.022	0.011	3.36E-02	0.000	0.016	9.88E-01
7.498201	0.022	0.011	3.55E-02	0.008	0.016	5.96E-01
5.352434	0.052	0.019	7.07E-03	0.025	0.029	3.83E-01
1.608611696	0.023	0.008	5.20E-03	0.005	0.012	7.01E-01
3.716698	0.019	0.008	1.55E-02	0.029	0.012	1.30E-02
1.344512112	0.055	0.020	7.37E-03	0.030	0.030	3.29E-01
4.059523	0.049	0.018	6.39E-03	0.013	0.027	6.25E-01
5.381367	0.018	0.007	1.21E-02	0.008	0.011	4.60E-01
1.398004894	0.016	0.006	4.61E-03	0.010	0.008	2.27E-01
3.602984	-0.017	0.007	1.63E-02	-0.011	0.011	2.94E-01
8.226577	0.016	0.007	2.63E-02	0.004	0.010	7.02E-01
3.769855	0.018	0.008	2.93E-02	0.025	0.013	4.32E-02
8.389411	0.021	0.009	1.95E-02	0.021	0.013	1.23E-01
8.727525	0.015	0.007	3.67E-02	0.025	0.011	1.72E-02
5.173115	0.038	0.014	7.08E-03	0.004	0.021	8.40E-01
2.494775141	-0.037	0.015	1.59E-02	0.003	0.023	8.85E-01

5.349742	0.051	0.019	7.73E-03	0.023	0.029	4.20E-01
3.479513	0.021	0.010	2.64E-02	0.039	0.014	7.54E-03
3.670943	-0.011	0.005	1.91E-02	0.000	0.007	9.52E-01
5.227954	0.020	0.009	2.47E-02	0.046	0.013	4.52E-04
1.110355028	0.015	0.006	1.09E-02	0.002	0.009	8.46E-01
1.212630536	0.015	0.006	1.87E-02	0.011	0.009	2.52E-01
2.975537	-0.010	0.004	1.74E-02	0.000	0.006	9.74E-01
1.616349645	0.017	0.006	7.59E-03	0.001	0.010	9.00E-01
3.118521	-0.023	0.009	8.15E-03	-0.017	0.013	1.86E-01
5.168741	0.028	0.011	7.11E-03	-0.005	0.016	7.69E-01
3.922931	0.026	0.012	2.47E-02	0.052	0.017	2.55E-03
1.563866161	0.062	0.023	7.21E-03	0.021	0.034	5.44E-01
2.96679	-0.011	0.004	1.21E-02	-0.006	0.007	3.32E-01
3.841515	0.017	0.007	1.81E-02	0.030	0.011	5.95E-03
5.386413	0.019	0.007	7.30E-03	0.009	0.011	3.91E-01
4.070289	0.054	0.020	6.11E-03	0.026	0.029	3.72E-01
3.342249	-0.026	0.013	3.86E-02	0.003	0.019	8.68E-01
1.06123587	0.011	0.006	4.59E-02	-0.005	0.008	5.64E-01
2.392836066	-0.020	0.008	1.25E-02	-0.003	0.012	8.08E-01
1.9389885	0.014	0.006	9.40E-03	0.014	0.008	8.81E-02
2.641796183	-0.041	0.014	2.76E-03	0.003	0.020	8.83E-01
4.271812	0.042	0.016	7.47E-03	0.023	0.023	3.21E-01
3.98248	-0.019	0.009	3.79E-02	-0.018	0.014	1.96E-01
1.938652068	0.014	0.006	9.62E-03	0.013	0.008	1.07E-01
7.760282	-0.028	0.010	4.25E-03	-0.028	0.014	5.28E-02
1.937979202	0.014	0.005	8.86E-03	0.010	0.008	2.07E-01
4.060532	0.046	0.017	6.54E-03	0.010	0.025	6.98E-01
2.212508197	0.060	0.022	7.21E-03	0.017	0.033	6.07E-01
2.110905554	-0.015	0.007	3.34E-02	-0.022	0.010	3.36E-02
2.9715	-0.011	0.004	1.55E-02	-0.001	0.007	9.30E-01
3.11953	-0.021	0.008	7.18E-03	-0.015	0.012	2.05E-01
3.958257	-0.020	0.008	1.78E-02	-0.028	0.013	2.69E-02
5.360172	0.035	0.013	8.20E-03	0.009	0.020	6.59E-01
3.72881	0.018	0.009	3.70E-02	0.032	0.013	1.29E-02
3.982144	-0.020	0.010	4.05E-02	-0.020	0.014	1.60E-01
0.872160754	0.045	0.017	7.04E-03	0.025	0.025	3.21E-01
7.663053	0.022	0.009	1.88E-02	-0.005	0.014	6.92E-01
3.427366	0.020	0.008	1.93E-02	0.037	0.013	2.90E-03
1.193453878	0.010	0.004	1.48E-02	0.006	0.006	3.48E-01
3.426021	0.018	0.008	1.70E-02	0.034	0.012	2.95E-03
3.454617	0.022	0.009	2.19E-02	0.034	0.014	1.45E-02
2.21688182	0.061	0.023	7.08E-03	0.016	0.034	6.45E-01
5.979544	0.019	0.009	2.71E-02	0.004	0.013	7.60E-01

0.929354294	-0.010	0.005	3.15E-02	-0.024	0.007	3.29E-04
3.084205	-0.016	0.006	1.02E-02	0.004	0.009	6.80E-01
2.964435	-0.011	0.005	1.64E-02	-0.009	0.007	1.78E-01
1.183697333	0.012	0.004	6.09E-03	0.005	0.006	4.71E-01
3.468747	0.019	0.009	3.45E-02	0.036	0.013	5.92E-03
2.212844629	0.060	0.022	7.11E-03	0.018	0.033	5.91E-01
6.136994	0.017	0.009	5.50E-02	0.000	0.013	9.94E-01
7.336713	-0.020	0.008	1.30E-02	0.004	0.012	7.35E-01
3.567659	-0.016	0.007	2.07E-02	-0.001	0.010	9.15E-01
2.429843651	-0.022	0.009	1.61E-02	-0.006	0.014	6.36E-01
2.964099	-0.011	0.005	1.54E-02	-0.007	0.007	3.31E-01
1.939324933	0.015	0.006	8.95E-03	0.017	0.008	4.79E-02
2.486700759	-0.034	0.012	4.88E-03	-0.005	0.018	7.74E-01
2.70369978	-0.019	0.006	2.11E-03	-0.026	0.009	4.53E-03
3.068056	-0.014	0.006	1.84E-02	-0.010	0.009	2.91E-01
2.382070223	-0.019	0.007	9.05E-03	-0.002	0.011	8.40E-01
1.94941791	0.015	0.006	8.65E-03	0.012	0.009	1.77E-01
3.717708	0.018	0.008	1.85E-02	0.027	0.011	1.84E-02
3.060991	-0.016	0.006	1.00E-02	-0.005	0.009	6.08E-01
3.567322	-0.019	0.008	1.66E-02	0.002	0.012	8.76E-01
1.344175679	0.054	0.020	7.80E-03	0.029	0.030	3.33E-01
3.707951	0.018	0.008	2.09E-02	0.029	0.011	1.15E-02
3.144426	-0.031	0.012	1.10E-02	-0.034	0.018	6.14E-02
1.343839246	0.054	0.020	7.82E-03	0.029	0.030	3.33E-01
5.234346	0.018	0.008	2.78E-02	0.044	0.012	4.41E-04
1.398341326	0.015	0.005	5.13E-03	0.010	0.008	2.17E-01
5.363536	0.032	0.012	6.77E-03	0.009	0.018	6.25E-01
2.110569122	-0.014	0.007	4.07E-02	-0.025	0.010	1.35E-02
8.728198	0.017	0.007	1.77E-02	-0.012	0.011	2.82E-01
2.913634	-0.020	0.008	1.02E-02	-0.011	0.011	3.57E-01
2.428834353	-0.023	0.009	1.00E-02	-0.003	0.013	8.11E-01
1.10934573	0.015	0.006	1.45E-02	0.009	0.009	3.35E-01
6.353993	-0.018	0.008	2.26E-02	-0.002	0.012	8.78E-01
2.217218253	0.060	0.022	7.25E-03	0.015	0.034	6.45E-01
5.342677	0.050	0.020	1.11E-02	0.005	0.029	8.73E-01
5.593656	0.019	0.008	1.74E-02	-0.011	0.012	3.68E-01
2.112587717	-0.016	0.007	1.89E-02	-0.016	0.010	1.25E-01
4.060196	0.047	0.017	6.78E-03	0.012	0.026	6.37E-01
6.089557	0.017	0.008	3.31E-02	0.003	0.012	8.23E-01
0.766857353	0.013	0.005	1.25E-02	0.002	0.008	7.79E-01
0.76517519	0.013	0.005	1.17E-02	0.001	0.008	9.46E-01
5.173451	0.035	0.013	5.94E-03	0.005	0.019	8.03E-01
5.219206	0.010	0.005	5.61E-02	0.001	0.007	9.22E-01

4.272821	0.040	0.015	8.03E-03	0.019	0.023	4.13E-01
1.942352826	0.015	0.006	9.68E-03	0.013	0.009	1.47E-01
3.88626	0.016	0.007	2.60E-02	0.033	0.010	1.42E-03
6.560563	0.018	0.008	2.77E-02	0.020	0.012	1.09E-01
3.02903	-0.010	0.005	2.59E-02	-0.005	0.007	4.78E-01
2.226301933	0.058	0.025	1.81E-02	0.037	0.036	3.02E-01
5.346714	0.053	0.020	8.14E-03	0.017	0.030	5.68E-01
2.212171764	0.059	0.022	7.81E-03	0.016	0.033	6.17E-01
8.022026	0.023	0.009	8.46E-03	-0.005	0.013	7.12E-01
2.958716	-0.011	0.005	1.66E-02	-0.005	0.007	4.50E-01
3.014227	-0.012	0.005	1.35E-02	-0.005	0.007	4.58E-01
8.744011	0.018	0.007	1.41E-02	-0.008	0.011	4.94E-01
2.970827	-0.011	0.004	1.25E-02	0.000	0.007	9.42E-01
1.195472474	0.010	0.004	1.75E-02	0.008	0.006	2.07E-01
1.402378517	0.013	0.005	6.46E-03	0.011	0.007	1.20E-01
3.601975	-0.017	0.007	1.27E-02	-0.005	0.010	6.23E-01
5.754134	0.024	0.011	2.19E-02	0.000	0.016	9.93E-01
3.00548	-0.010	0.004	1.52E-02	-0.004	0.006	4.54E-01
4.05986	0.048	0.018	7.10E-03	0.015	0.026	5.81E-01
2.704036212	-0.018	0.006	2.39E-03	-0.024	0.009	7.05E-03
8.604055	0.015	0.008	5.19E-02	0.007	0.011	5.70E-01
6.662839	0.021	0.009	1.70E-02	0.016	0.013	2.24E-01
1.212966968	0.015	0.006	2.05E-02	0.011	0.010	2.58E-01
4.085092	0.038	0.013	4.70E-03	0.025	0.020	2.22E-01
1.609620993	0.022	0.008	6.23E-03	0.002	0.012	9.01E-01
3.269243	-0.025	0.012	2.95E-02	-0.022	0.017	1.96E-01
6.59387	-0.021	0.009	2.62E-02	-0.033	0.014	1.86E-02
3.903082	0.019	0.008	2.39E-02	0.033	0.012	7.77E-03
2.622619525	-0.027	0.013	3.16E-02	0.003	0.019	8.66E-01
8.947552	0.020	0.009	3.02E-02	0.012	0.014	3.89E-01
1.644946415	0.013	0.006	2.24E-02	0.003	0.008	7.59E-01
7.802672	-0.022	0.009	1.28E-02	-0.019	0.013	1.58E-01
2.109896256	-0.013	0.007	5.22E-02	-0.029	0.010	3.70E-03
3.073439	-0.016	0.007	1.84E-02	-0.020	0.010	4.84E-02
8.895069	0.020	0.009	2.56E-02	0.008	0.013	5.34E-01
3.343258	-0.026	0.013	5.02E-02	-0.007	0.020	7.30E-01
1.265786885	0.047	0.018	8.07E-03	0.014	0.026	6.03E-01
1.562856863	0.060	0.023	8.29E-03	0.021	0.034	5.35E-01
3.444861	0.021	0.009	2.40E-02	0.034	0.014	1.32E-02
7.425868	-0.028	0.011	9.12E-03	-0.021	0.016	1.85E-01
2.705381943	-0.018	0.006	2.45E-03	-0.024	0.009	5.46E-03
5.173788	0.035	0.013	5.93E-03	-0.003	0.019	8.82E-01
4.269121	0.041	0.015	7.92E-03	0.036	0.023	1.15E-01

2.864851	-0.018	0.008	3.30E-02	0.006	0.013	6.52E-01
7.828241	-0.025	0.010	9.96E-03	0.009	0.015	5.41E-01
7.051082	-0.018	0.008	1.98E-02	-0.019	0.012	1.15E-01
2.86586	-0.017	0.008	3.75E-02	-0.004	0.013	7.49E-01
6.000403	0.020	0.009	3.14E-02	0.011	0.014	4.30E-01
5.464802	0.014	0.007	2.88E-02	0.006	0.010	5.18E-01
3.143754	-0.026	0.010	1.17E-02	-0.034	0.016	3.21E-02
5.999057	0.021	0.009	2.14E-02	-0.006	0.014	6.79E-01
1.193117446	0.010	0.004	1.60E-02	0.005	0.006	4.16E-01
2.96208	-0.011	0.005	1.71E-02	-0.002	0.007	7.65E-01
8.601363	0.018	0.008	2.52E-02	-0.002	0.012	8.54E-01
1.343502814	0.053	0.020	8.44E-03	0.029	0.030	3.44E-01
1.400023489	0.014	0.005	5.63E-03	0.015	0.007	4.89E-02
7.05411	-0.024	0.010	1.34E-02	-0.028	0.015	5.83E-02
3.910147	0.018	0.009	3.51E-02	0.037	0.013	4.17E-03
1.336774162	0.047	0.018	7.65E-03	0.023	0.026	3.77E-01
4.267102	0.041	0.015	7.04E-03	0.031	0.023	1.74E-01
3.525605	0.019	0.008	1.89E-02	0.029	0.012	1.76E-02
1.337110595	0.047	0.018	7.71E-03	0.024	0.027	3.72E-01
3.402807	0.021	0.009	2.33E-02	0.038	0.014	6.09E-03
2.217554686	0.060	0.022	7.75E-03	0.014	0.033	6.73E-01
1.842432346	-0.024	0.010	1.72E-02	-0.007	0.015	6.37E-01
0.93406435	-0.011	0.005	1.90E-02	-0.018	0.007	1.03E-02
2.643478346	-0.026	0.010	7.54E-03	0.002	0.014	8.65E-01
1.337447027	0.048	0.018	7.81E-03	0.024	0.027	3.78E-01
1.110018596	0.014	0.006	1.59E-02	0.006	0.009	5.10E-01
3.119867	-0.021	0.008	5.70E-03	-0.011	0.012	3.39E-01
2.218227551	0.059	0.022	7.98E-03	0.013	0.033	7.05E-01
4.266429	0.041	0.015	7.52E-03	0.032	0.023	1.58E-01
1.949081478	0.015	0.006	9.12E-03	0.013	0.009	1.50E-01
3.120203	-0.021	0.008	5.83E-03	-0.010	0.012	3.69E-01
1.322643993	0.038	0.014	6.76E-03	0.020	0.021	3.40E-01
1.621732567	0.017	0.007	1.00E-02	0.014	0.010	1.53E-01
1.201864693	0.011	0.005	2.37E-02	0.009	0.007	2.16E-01
3.81931	0.015	0.007	2.90E-02	0.030	0.010	3.31E-03
2.211835332	0.058	0.022	8.24E-03	0.015	0.033	6.52E-01
1.019854661	0.021	0.008	9.39E-03	0.008	0.012	4.89E-01
2.218563983	0.059	0.022	7.98E-03	0.014	0.033	6.80E-01
1.147362613	0.016	0.006	7.18E-03	-0.002	0.009	8.55E-01
1.336437729	0.047	0.018	7.79E-03	0.023	0.026	3.81E-01
2.912624	-0.020	0.008	9.41E-03	-0.011	0.012	3.61E-01
1.887850746	-0.016	0.006	1.29E-02	-0.001	0.009	9.52E-01
7.784841	-0.018	0.007	1.00E-02	-0.006	0.011	5.74E-01

7.584327	0.025	0.010	1.23E-02	-0.004	0.015	7.66E-01
1.562520431	0.060	0.023	8.56E-03	0.021	0.034	5.38E-01
8.909535	0.022	0.009	1.67E-02	-0.002	0.014	9.10E-01
5.465811	0.014	0.007	4.23E-02	-0.001	0.010	9.27E-01
5.950274	0.019	0.009	2.59E-02	-0.003	0.013	8.07E-01
4.061542	0.045	0.017	7.37E-03	0.010	0.025	6.81E-01
1.338119892	0.048	0.018	8.18E-03	0.023	0.027	3.94E-01
5.34806	0.050	0.020	1.03E-02	0.019	0.029	5.07E-01
2.694279667	-0.019	0.006	2.86E-03	-0.010	0.009	3.06E-01
2.642132616	-0.038	0.013	3.67E-03	0.001	0.019	9.58E-01
1.33778346	0.048	0.018	8.13E-03	0.023	0.027	3.89E-01
4.070962	0.051	0.019	7.54E-03	0.015	0.028	5.94E-01
3.246366	0.021	0.008	1.38E-02	0.041	0.012	1.06E-03
2.430180083	-0.021	0.009	1.67E-02	-0.010	0.013	4.45E-01
1.941007096	0.015	0.006	9.08E-03	0.014	0.009	1.07E-01
8.340628	0.023	0.009	1.44E-02	-0.002	0.014	8.79E-01
3.958593	-0.021	0.009	1.71E-02	-0.023	0.013	6.94E-02
1.312887448	0.037	0.013	5.41E-03	0.019	0.020	3.47E-01
3.14409	-0.027	0.011	1.36E-02	-0.035	0.017	3.68E-02
7.711835	-0.023	0.011	3.26E-02	0.004	0.016	8.06E-01
3.885924	0.015	0.007	3.15E-02	0.032	0.010	1.55E-03
7.009028	-0.023	0.009	1.32E-02	-0.018	0.014	2.12E-01
8.134395	0.012	0.006	4.12E-02	-0.007	0.009	4.41E-01
8.568056	0.022	0.009	1.96E-02	0.009	0.014	5.12E-01
3.541417	0.017	0.009	5.34E-02	0.032	0.013	1.58E-02
1.563529728	0.060	0.023	8.57E-03	0.020	0.034	5.55E-01
2.49073795	-0.035	0.014	1.10E-02	-0.010	0.020	6.11E-01
6.322032	0.018	0.008	1.52E-02	0.004	0.011	7.11E-01
2.970491	-0.011	0.004	1.47E-02	0.001	0.007	9.05E-01
3.960276	-0.022	0.009	9.76E-03	-0.008	0.013	5.27E-01
2.25321654	0.025	0.009	7.93E-03	0.012	0.014	4.02E-01
2.690242476	-0.016	0.006	3.89E-03	-0.005	0.008	5.68E-01
1.945717152	0.015	0.006	8.97E-03	0.009	0.009	3.16E-01
0.915224125	-0.012	0.005	1.67E-02	-0.029	0.007	1.12E-04
1.014135307	0.030	0.011	7.94E-03	0.024	0.017	1.68E-01
1.796341081	-0.019	0.010	5.07E-02	-0.002	0.014	8.71E-01
2.218900416	0.059	0.022	8.19E-03	0.014	0.033	6.68E-01
1.343166381	0.053	0.020	8.96E-03	0.028	0.030	3.50E-01
5.232664	0.014	0.007	4.66E-02	0.027	0.010	9.49E-03
0.898066063	0.028	0.010	6.44E-03	-0.001	0.015	9.26E-01
2.513278933	-0.029	0.012	1.58E-02	-0.024	0.018	1.82E-01
2.217891118	0.059	0.022	8.40E-03	0.013	0.033	6.89E-01
1.213303401	0.015	0.006	2.23E-02	0.011	0.010	2.58E-01

4.272485	0.040	0.015	8.76E-03	0.021	0.023	3.57E-01
6.068699	0.016	0.007	2.66E-02	0.006	0.011	5.45E-01
5.348396	0.050	0.020	1.06E-02	0.021	0.029	4.71E-01
7.995784	0.015	0.007	4.46E-02	0.016	0.011	1.43E-01
1.338456325	0.049	0.018	8.43E-03	0.023	0.028	3.96E-01
2.964771	-0.011	0.005	2.00E-02	-0.008	0.007	2.30E-01
2.127054319	-0.019	0.007	9.70E-03	-0.001	0.011	9.29E-01
3.454281	0.020	0.009	3.00E-02	0.037	0.014	7.78E-03
0.931709322	-0.009	0.004	3.26E-02	-0.008	0.006	1.97E-01
3.452262	0.017	0.008	3.31E-02	0.035	0.012	4.18E-03
0.871824321	0.043	0.017	8.58E-03	0.023	0.025	3.44E-01
2.314783704	-0.011	0.005	2.74E-02	-0.007	0.007	3.73E-01
5.377666	0.025	0.010	1.25E-02	0.012	0.015	4.13E-01
5.581544	0.023	0.009	1.31E-02	0.012	0.014	4.02E-01
5.745387	0.022	0.012	5.84E-02	0.008	0.017	6.61E-01
2.880663	-0.016	0.007	2.43E-02	-0.004	0.011	7.20E-01
3.073776	-0.016	0.007	1.70E-02	-0.027	0.010	7.56E-03
2.470551994	-0.020	0.008	1.98E-02	-0.011	0.013	3.91E-01
3.05729	-0.011	0.006	4.28E-02	-0.008	0.008	3.54E-01
1.55982897	0.057	0.022	9.07E-03	0.016	0.033	6.29E-01
8.12161	0.016	0.007	2.99E-02	0.000	0.011	9.96E-01
4.060869	0.044	0.017	8.14E-03	0.006	0.025	8.03E-01
2.968472	-0.011	0.004	1.40E-02	0.000	0.007	9.91E-01
3.085214	-0.015	0.007	2.11E-02	0.005	0.010	6.23E-01
2.394854661	-0.023	0.010	1.78E-02	-0.015	0.015	3.21E-01
1.338792758	0.049	0.019	8.60E-03	0.024	0.028	3.89E-01
3.42703	0.019	0.008	2.13E-02	0.036	0.012	3.42E-03
1.562183998	0.059	0.023	9.01E-03	0.020	0.034	5.61E-01
1.339465623	0.050	0.019	8.75E-03	0.024	0.028	3.91E-01
7.288267	-0.022	0.010	2.62E-02	-0.019	0.015	2.03E-01
5.747069	0.022	0.009	2.27E-02	0.005	0.014	7.10E-01
1.944707854	0.015	0.006	9.10E-03	0.011	0.009	1.91E-01
7.960123	-0.016	0.008	4.44E-02	-0.027	0.012	2.40E-02
1.400696354	0.014	0.005	5.72E-03	0.014	0.008	5.57E-02
2.469206264	-0.023	0.009	8.64E-03	-0.006	0.013	6.40E-01
1.563193296	0.060	0.023	9.06E-03	0.020	0.034	5.56E-01
3.260832	0.019	0.008	2.31E-02	0.029	0.012	1.92E-02
3.839833	0.016	0.007	2.30E-02	0.030	0.011	4.64E-03
1.017499633	0.025	0.010	8.87E-03	0.017	0.014	2.29E-01
3.394396	0.018	0.009	3.46E-02	0.037	0.013	4.77E-03
3.717035	0.018	0.008	1.91E-02	0.027	0.011	1.71E-02
1.322307561	0.037	0.014	7.51E-03	0.019	0.021	3.71E-01
1.109682163	0.014	0.006	1.58E-02	0.005	0.009	5.56E-01

1.61702251	0.016	0.006	1.22E-02	0.001	0.010	8.78E-01
2.127390751	-0.019	0.007	1.16E-02	-0.001	0.011	9.02E-01
1.399014191	0.014	0.005	5.70E-03	0.011	0.008	1.34E-01
1.10665427	0.015	0.007	2.27E-02	-0.003	0.010	7.57E-01
3.425348	0.018	0.008	1.96E-02	0.033	0.012	4.16E-03
1.644609983	0.013	0.006	2.24E-02	0.005	0.008	5.11E-01
1.94807218	0.015	0.006	1.10E-02	0.008	0.009	3.82E-01
1.33912919	0.049	0.019	8.75E-03	0.024	0.028	3.92E-01
1.400359922	0.014	0.005	6.81E-03	0.015	0.007	4.30E-02
5.378675	0.025	0.010	1.19E-02	0.016	0.015	2.87E-01
2.876963	-0.017	0.008	3.73E-02	0.001	0.012	9.03E-01
2.211498899	0.057	0.022	8.80E-03	0.014	0.032	6.60E-01
3.840169	0.016	0.007	2.35E-02	0.031	0.011	3.99E-03
2.126717886	-0.019	0.007	8.80E-03	0.002	0.011	8.51E-01
3.340567	-0.022	0.010	3.77E-02	0.005	0.015	7.65E-01
3.08656	-0.016	0.007	1.99E-02	0.002	0.010	8.70E-01
4.064906	0.044	0.017	8.27E-03	0.014	0.025	5.78E-01
2.134119403	-0.017	0.008	3.50E-02	0.011	0.012	3.48E-01
8.216484	0.019	0.009	3.05E-02	0.004	0.013	7.61E-01
7.741105	-0.025	0.011	2.77E-02	-0.014	0.017	3.97E-01
2.692933937	-0.018	0.006	3.33E-03	-0.007	0.009	4.25E-01
5.384058	0.017	0.007	1.54E-02	-0.003	0.011	7.82E-01
1.342829949	0.052	0.020	9.19E-03	0.028	0.030	3.45E-01
3.1488	-0.031	0.012	1.03E-02	-0.029	0.018	1.13E-01
6.089221	0.018	0.008	2.81E-02	-0.001	0.012	9.13E-01
2.955688	-0.011	0.005	1.50E-02	-0.008	0.007	2.19E-01
3.228871	0.015	0.007	4.87E-02	0.026	0.011	1.60E-02
4.268111	0.041	0.015	8.14E-03	0.031	0.023	1.75E-01
4.266766	0.041	0.015	8.27E-03	0.031	0.023	1.77E-01
1.336101297	0.046	0.017	8.39E-03	0.022	0.026	3.94E-01
1.172258625	0.012	0.004	4.63E-03	0.002	0.006	7.55E-01
4.267775	0.041	0.015	8.13E-03	0.031	0.023	1.81E-01
7.759609	-0.029	0.011	5.84E-03	-0.019	0.016	2.39E-01
1.561847565	0.059	0.023	9.34E-03	0.019	0.034	5.81E-01
1.342493516	0.052	0.020	9.23E-03	0.028	0.030	3.45E-01
1.0171632	0.027	0.010	6.59E-03	0.015	0.015	3.15E-01
2.948623	-0.012	0.005	1.70E-02	-0.006	0.007	4.49E-01
1.560165402	0.057	0.022	9.43E-03	0.016	0.033	6.27E-01
2.219573281	0.059	0.022	8.45E-03	0.014	0.033	6.76E-01
1.149717641	0.016	0.006	8.31E-03	-0.002	0.009	8.23E-01
1.622405432	0.018	0.007	8.68E-03	0.008	0.010	4.38E-01
7.761627	-0.020	0.009	1.85E-02	-0.031	0.013	1.48E-02
8.052642	0.020	0.008	1.40E-02	0.015	0.012	2.37E-01

1.340811353	0.050	0.019	9.36E-03	0.026	0.029	3.74E-01
2.219236849	0.058	0.022	8.72E-03	0.013	0.033	7.02E-01
3.41761	0.018	0.009	3.69E-02	0.038	0.013	3.74E-03
5.091362	0.020	0.008	1.55E-02	0.005	0.012	6.60E-01
3.522913	0.021	0.009	2.29E-02	0.038	0.013	4.69E-03
5.892408	0.022	0.011	4.27E-02	0.018	0.016	2.53E-01
3.82402	0.016	0.007	3.09E-02	0.035	0.011	1.62E-03
7.213915	-0.021	0.008	1.19E-02	-0.015	0.012	2.08E-01
5.709725	0.016	0.008	2.89E-02	-0.004	0.011	6.99E-01
1.34047492	0.050	0.019	9.31E-03	0.025	0.029	3.77E-01
1.616686078	0.016	0.006	1.01E-02	-0.002	0.010	8.70E-01
1.559492537	0.057	0.022	9.63E-03	0.016	0.033	6.29E-01
2.99976	-0.009	0.004	1.77E-02	0.002	0.006	6.93E-01
1.342157083	0.051	0.020	9.50E-03	0.027	0.030	3.61E-01
2.051693418	0.016	0.006	9.63E-03	0.008	0.009	3.63E-01
5.586254	0.018	0.007	1.14E-02	-0.005	0.010	6.08E-01
2.63237607	-0.019	0.008	2.17E-02	-0.006	0.012	6.06E-01
5.390114	0.021	0.009	2.00E-02	0.017	0.013	1.94E-01
6.698837	0.023	0.010	2.51E-02	0.032	0.015	3.89E-02
3.731501	0.018	0.009	3.42E-02	0.030	0.013	1.94E-02
3.453944	0.019	0.009	3.41E-02	0.036	0.013	6.45E-03
0.695533643	0.045	0.017	6.85E-03	0.008	0.025	7.36E-01
1.339802055	0.049	0.019	9.32E-03	0.024	0.028	3.90E-01
3.149137	-0.028	0.011	1.22E-02	-0.026	0.017	1.20E-01
2.979575	-0.010	0.004	2.26E-02	0.004	0.006	5.67E-01
2.219909714	0.059	0.022	8.42E-03	0.015	0.033	6.43E-01
1.341484218	0.051	0.020	9.63E-03	0.026	0.029	3.75E-01
8.962019	-0.023	0.010	1.82E-02	-0.012	0.014	3.84E-01
8.114209	0.023	0.009	1.04E-02	0.006	0.014	6.87E-01
3.424675	0.018	0.008	2.34E-02	0.035	0.012	3.31E-03
5.937826	0.023	0.009	1.51E-02	0.004	0.014	7.84E-01
3.390359	0.019	0.009	4.39E-02	0.041	0.014	3.13E-03
1.341147786	0.051	0.019	9.58E-03	0.026	0.029	3.73E-01
0.692842183	0.036	0.014	1.30E-02	0.011	0.021	5.99E-01
3.252085	-0.015	0.007	2.09E-02	-0.002	0.010	8.65E-01
6.686725	-0.017	0.009	6.07E-02	-0.003	0.013	8.23E-01
2.693606802	-0.018	0.006	3.44E-03	-0.008	0.009	3.74E-01
4.272149	0.040	0.015	9.46E-03	0.023	0.023	3.21E-01
2.127727184	-0.018	0.008	1.52E-02	-0.004	0.011	7.52E-01
1.335764864	0.045	0.017	8.66E-03	0.022	0.026	3.95E-01
3.970368	-0.020	0.008	1.09E-02	-0.010	0.012	3.78E-01
2.469879129	-0.020	0.009	1.67E-02	-0.013	0.013	3.00E-01
1.804079031	-0.020	0.010	3.94E-02	-0.006	0.014	6.55E-01

1.340138488	0.050	0.019	9.52E-03	0.025	0.029	3.87E-01
6.523555	0.018	0.009	3.68E-02	0.021	0.013	1.13E-01
2.118979936	-0.018	0.008	1.94E-02	-0.008	0.011	4.63E-01
5.93177	0.015	0.007	2.94E-02	0.006	0.010	5.70E-01
7.766337	-0.021	0.008	1.20E-02	-0.030	0.012	1.48E-02
3.425011	0.018	0.008	2.20E-02	0.033	0.012	5.14E-03
2.431862246	-0.017	0.008	3.38E-02	-0.020	0.012	1.04E-01
3.144763	-0.034	0.013	1.14E-02	-0.033	0.020	1.01E-01
1.610293859	0.020	0.008	9.66E-03	0.007	0.012	5.57E-01
7.79628	-0.020	0.009	2.84E-02	-0.010	0.014	4.61E-01
2.112251285	-0.016	0.007	2.29E-02	-0.017	0.011	1.20E-01
3.251748	-0.015	0.006	1.62E-02	-0.003	0.010	7.76E-01
1.322980426	0.037	0.014	7.60E-03	0.020	0.021	3.36E-01
3.841851	0.016	0.007	2.45E-02	0.027	0.011	1.08E-02
2.988322	-0.009	0.004	2.93E-02	0.007	0.006	2.61E-01
7.861211	0.017	0.009	5.37E-02	0.005	0.013	7.13E-01
1.94033423	0.015	0.006	1.06E-02	0.014	0.009	9.24E-02
5.59231	0.023	0.009	1.68E-02	-0.001	0.014	9.53E-01
1.335428432	0.045	0.017	8.67E-03	0.022	0.026	3.89E-01
0.916233423	-0.012	0.005	2.06E-02	-0.028	0.007	1.79E-04
5.358826	0.039	0.015	8.56E-03	0.029	0.022	1.81E-01
7.760954	-0.023	0.008	6.32E-03	-0.035	0.013	5.38E-03
1.341820651	0.051	0.020	9.95E-03	0.026	0.029	3.72E-01
8.899442	-0.021	0.009	2.42E-02	-0.053	0.014	1.61E-04
3.251412	-0.016	0.006	1.30E-02	-0.002	0.009	8.19E-01
3.717371	0.017	0.008	2.13E-02	0.026	0.011	2.04E-02
3.086897	-0.015	0.007	2.04E-02	-0.002	0.010	8.12E-01
2.704372645	-0.018	0.006	3.10E-03	-0.023	0.009	9.28E-03
2.977219	-0.010	0.004	1.97E-02	0.001	0.006	9.23E-01
3.425684	0.018	0.008	2.16E-02	0.034	0.012	3.11E-03
7.393907	0.016	0.007	2.09E-02	-0.013	0.010	2.19E-01
3.068393	-0.014	0.006	2.28E-02	-0.008	0.009	3.56E-01
2.114942745	-0.015	0.007	4.53E-02	-0.011	0.011	3.36E-01
2.211162466	0.055	0.021	9.51E-03	0.014	0.032	6.69E-01
3.075121	-0.014	0.007	2.61E-02	-0.022	0.010	2.27E-02
1.401705652	0.013	0.005	8.88E-03	0.013	0.007	6.69E-02
6.726761	-0.025	0.010	1.55E-02	-0.009	0.015	5.44E-01
3.508783	0.018	0.009	3.80E-02	0.032	0.013	1.06E-02
5.411982	0.022	0.010	2.31E-02	0.010	0.014	4.98E-01
4.268448	0.041	0.016	8.75E-03	0.034	0.023	1.45E-01
4.268784	0.041	0.015	8.87E-03	0.034	0.023	1.40E-01
2.483	-0.028	0.011	8.10E-03	0.003	0.016	8.48E-01
5.133416	0.017	0.007	1.66E-02	0.004	0.011	6.94E-01

2.689569611	-0.016	0.006	4.50E-03	-0.004	0.008	6.45E-01
1.560501835	0.057	0.022	1.02E-02	0.016	0.033	6.38E-01
2.2259655	0.055	0.024	2.27E-02	0.034	0.035	3.32E-01
1.150054074	0.016	0.006	8.36E-03	-0.001	0.009	8.93E-01
4.067934	0.049	0.019	8.70E-03	0.023	0.028	4.22E-01
7.224008	-0.024	0.009	1.13E-02	0.006	0.014	6.64E-01
2.954678	-0.010	0.005	2.25E-02	-0.005	0.007	4.95E-01
3.148464	-0.034	0.013	9.13E-03	-0.030	0.020	1.26E-01
3.035086	0.019	0.009	3.76E-02	0.016	0.014	2.33E-01
2.690915341	-0.016	0.006	4.40E-03	-0.002	0.009	8.04E-01
3.418619	0.020	0.009	3.29E-02	0.038	0.014	6.91E-03
3.494653	0.021	0.010	3.22E-02	0.034	0.014	1.63E-02
2.706054808	-0.017	0.006	3.22E-03	-0.025	0.009	3.36E-03
5.62528	0.020	0.009	2.42E-02	0.008	0.013	5.23E-01
2.261290922	0.015	0.006	1.71E-02	0.012	0.009	1.97E-01
6.767805	-0.022	0.009	1.55E-02	-0.023	0.013	9.23E-02
3.871457	-0.010	0.005	2.74E-02	0.006	0.007	4.19E-01
6.919537	-0.019	0.008	1.66E-02	0.003	0.012	7.89E-01
1.947735747	0.015	0.006	1.26E-02	0.006	0.009	5.22E-01
5.359835	0.027	0.011	1.01E-02	0.009	0.016	5.76E-01
6.963946	-0.027	0.011	1.20E-02	-0.012	0.016	4.50E-01
8.974803	0.023	0.009	1.70E-02	0.009	0.014	5.45E-01
5.175134	0.039	0.015	8.05E-03	-0.007	0.022	7.56E-01
3.104391	-0.022	0.008	8.74E-03	-0.012	0.012	3.32E-01
1.64427355	0.014	0.006	1.67E-02	0.006	0.008	4.73E-01
3.817628	0.014	0.007	3.66E-02	0.033	0.010	8.49E-04
2.690578909	-0.016	0.006	4.59E-03	-0.004	0.009	6.52E-01
3.756061	0.024	0.011	2.42E-02	0.036	0.016	2.50E-02
3.146782	-0.053	0.022	1.43E-02	-0.089	0.032	5.87E-03
2.911615	-0.019	0.008	1.67E-02	-0.005	0.012	6.92E-01
1.944034989	0.015	0.006	1.07E-02	0.011	0.009	1.94E-01
2.111241987	-0.015	0.007	3.65E-02	-0.018	0.011	8.08E-02
4.063224	0.048	0.018	8.48E-03	0.012	0.027	6.56E-01
1.335091999	0.044	0.017	8.94E-03	0.022	0.025	3.90E-01
5.8517	0.023	0.010	1.78E-02	0.015	0.015	3.18E-01
7.218289	-0.026	0.010	1.41E-02	-0.015	0.016	3.35E-01
8.645436	-0.019	0.007	1.18E-02	0.008	0.011	4.85E-01
1.559156105	0.056	0.022	1.05E-02	0.016	0.032	6.32E-01
3.545118	0.025	0.010	1.31E-02	0.028	0.015	6.74E-02
6.086193	0.020	0.010	3.80E-02	0.014	0.015	3.44E-01
3.654122	-0.011	0.005	1.78E-02	0.006	0.007	4.33E-01
4.065242	0.043	0.016	9.18E-03	0.014	0.025	5.65E-01
3.964649	-0.020	0.008	1.12E-02	0.007	0.012	5.38E-01

1.630143381	0.015	0.006	1.65E-02	0.012	0.009	2.07E-01
1.945380719	0.015	0.006	1.06E-02	0.009	0.009	2.78E-01
2.994378	-0.009	0.004	1.94E-02	0.001	0.006	8.76E-01
1.334419134	0.043	0.017	8.73E-03	0.021	0.025	3.95E-01
2.91397	-0.019	0.008	1.27E-02	-0.009	0.011	4.55E-01
1.560838268	0.057	0.022	1.04E-02	0.017	0.033	6.16E-01
1.334082701	0.043	0.016	8.60E-03	0.021	0.024	3.95E-01
5.97988	0.018	0.009	5.21E-02	0.008	0.014	5.49E-01
5.234009	0.016	0.008	3.93E-02	0.041	0.012	5.13E-04
4.062887	0.047	0.018	9.06E-03	0.014	0.027	6.02E-01
2.887728	-0.015	0.007	2.26E-02	-0.001	0.010	8.88E-01
3.981807	-0.019	0.010	5.80E-02	-0.023	0.015	1.16E-01
1.334755566	0.044	0.017	8.88E-03	0.021	0.025	3.93E-01
1.265450453	0.045	0.017	1.04E-02	0.012	0.026	6.51E-01
2.11292415	-0.015	0.007	2.66E-02	-0.013	0.010	2.01E-01
2.624301688	-0.023	0.010	2.27E-02	0.000	0.015	9.87E-01
3.272271	-0.023	0.011	3.15E-02	0.002	0.016	8.95E-01
2.957706	-0.011	0.005	1.76E-02	-0.007	0.007	3.31E-01
5.413328	0.019	0.008	1.37E-02	0.006	0.011	5.75E-01
5.342341	0.048	0.020	1.47E-02	0.000	0.029	9.96E-01
3.426357	0.018	0.008	2.21E-02	0.036	0.012	2.15E-03
0.699570834	0.033	0.013	1.17E-02	0.012	0.020	5.53E-01
5.176143	0.041	0.016	9.05E-03	-0.003	0.023	8.87E-01
7.574907	0.019	0.009	2.90E-02	0.023	0.013	6.76E-02
2.876626	-0.016	0.008	5.50E-02	-0.006	0.012	6.28E-01
1.901980915	-0.013	0.006	2.00E-02	-0.009	0.009	2.97E-01
7.783495	-0.022	0.009	1.51E-02	0.004	0.014	7.69E-01
6.767469	-0.023	0.010	2.46E-02	-0.019	0.015	2.04E-01
5.13308	0.017	0.008	2.72E-02	0.002	0.011	8.75E-01
5.233	0.014	0.007	5.10E-02	0.030	0.010	3.98E-03
7.858856	0.016	0.007	3.04E-02	0.006	0.011	5.99E-01
5.174124	0.035	0.013	8.15E-03	-0.010	0.020	6.10E-01
1.561511133	0.057	0.022	1.05E-02	0.018	0.034	6.00E-01
5.832186	0.017	0.009	4.67E-02	-0.001	0.013	9.22E-01
1.5611747	0.057	0.022	1.05E-02	0.017	0.033	6.12E-01
6.413206	0.019	0.011	7.39E-02	-0.011	0.016	4.66E-01
2.126381453	-0.020	0.008	9.27E-03	0.006	0.012	5.89E-01
4.258355	0.045	0.017	9.09E-03	0.018	0.026	4.79E-01
7.33604	-0.017	0.007	1.21E-02	-0.009	0.010	3.42E-01
1.954800832	0.013	0.005	1.29E-02	0.005	0.008	5.06E-01
4.273831	0.040	0.015	9.85E-03	0.017	0.023	4.56E-01
4.085428	0.035	0.013	6.59E-03	0.022	0.019	2.65E-01
4.266093	0.040	0.015	9.82E-03	0.031	0.023	1.77E-01

3.074785	-0.014	0.007	3.44E-02	-0.020	0.010	4.53E-02
0.664245412	-0.022	0.010	3.47E-02	-0.045	0.016	4.02E-03
1.213639834	0.014	0.007	2.62E-02	0.010	0.010	2.86E-01
8.946207	0.016	0.009	6.82E-02	0.000	0.013	9.77E-01
3.47985	0.020	0.010	3.80E-02	0.038	0.014	8.78E-03
1.948408613	0.015	0.006	1.27E-02	0.008	0.009	3.56E-01
1.313223881	0.035	0.013	6.48E-03	0.018	0.019	3.48E-01
2.414367751	-0.018	0.009	3.74E-02	0.020	0.013	1.32E-01
3.815609	0.016	0.008	3.56E-02	0.034	0.011	2.44E-03
4.061205	0.043	0.017	9.83E-03	0.007	0.025	7.62E-01
2.965781	-0.010	0.004	1.96E-02	-0.007	0.007	3.06E-01
5.227617	0.018	0.008	3.70E-02	0.043	0.013	5.69E-04
4.059187	0.047	0.018	9.22E-03	0.013	0.027	6.40E-01
8.85066	-0.019	0.008	2.18E-02	-0.025	0.012	4.00E-02
3.148127	-0.038	0.015	8.97E-03	-0.036	0.022	1.01E-01
2.708073403	-0.017	0.006	4.92E-03	-0.020	0.009	2.59E-02
1.558819672	0.055	0.022	1.09E-02	0.014	0.032	6.73E-01
0.701252997	0.030	0.011	7.29E-03	0.026	0.017	1.29E-01
7.780468	-0.023	0.009	1.20E-02	-0.003	0.013	8.02E-01
4.264747	0.039	0.015	1.03E-02	0.033	0.023	1.44E-01
2.882009	-0.017	0.008	3.04E-02	0.002	0.012	8.91E-01
3.432413	0.018	0.009	3.74E-02	0.034	0.013	9.09E-03
5.347724	0.045	0.018	1.28E-02	0.019	0.027	4.86E-01
6.727097	-0.029	0.012	1.83E-02	-0.015	0.018	3.96E-01
5.545546	0.016	0.007	2.51E-02	0.011	0.010	2.78E-01
3.766827	0.029	0.010	3.83E-03	0.041	0.015	7.40E-03
4.073653	0.053	0.021	9.37E-03	0.030	0.031	3.32E-01
1.012116712	0.029	0.011	9.59E-03	0.030	0.017	7.60E-02
5.361181	0.032	0.013	1.15E-02	0.011	0.019	5.57E-01
4.071635	0.049	0.019	8.32E-03	0.020	0.028	4.74E-01
1.55848324	0.055	0.022	1.08E-02	0.013	0.032	6.90E-01
3.700213	0.017	0.008	2.90E-02	0.027	0.012	1.92E-02
1.942689259	0.014	0.006	1.37E-02	0.013	0.009	1.24E-01
4.06457	0.045	0.017	9.18E-03	0.017	0.026	5.04E-01
3.48456	0.018	0.009	3.95E-02	0.029	0.013	2.85E-02
2.888401	-0.015	0.007	2.25E-02	-0.006	0.010	5.44E-01
5.395833	0.020	0.009	2.63E-02	-0.014	0.013	2.97E-01
2.3921632	-0.018	0.008	2.25E-02	0.005	0.012	6.54E-01
3.444524	0.019	0.009	3.44E-02	0.036	0.014	8.89E-03
0.914887693	-0.011	0.005	2.23E-02	-0.029	0.007	1.15E-04
3.592218	-0.012	0.006	5.57E-02	0.010	0.009	2.68E-01
1.321971128	0.036	0.014	9.20E-03	0.017	0.021	4.03E-01
7.358918	-0.022	0.010	3.22E-02	-0.037	0.016	1.82E-02

3.653785	-0.011	0.005	2.17E-02	0.005	0.007	4.71E-01
6.455932	0.018	0.009	5.59E-02	0.003	0.014	8.20E-01
3.528969	0.020	0.009	2.72E-02	0.040	0.013	2.54E-03
6.6948	0.015	0.007	2.45E-02	0.020	0.010	5.06E-02
6.15348	0.016	0.007	1.58E-02	0.022	0.010	2.87E-02
1.179323709	0.016	0.006	9.53E-03	-0.002	0.009	8.06E-01
1.058544409	0.011	0.005	3.70E-02	-0.004	0.008	6.22E-01
3.343595	-0.024	0.013	6.31E-02	-0.008	0.019	6.87E-01
2.317138733	-0.011	0.005	3.01E-02	-0.010	0.008	2.17E-01
2.911952	-0.020	0.008	1.21E-02	-0.005	0.012	6.47E-01
5.440242	-0.019	0.008	1.93E-02	-0.013	0.012	2.76E-01
5.21887	0.009	0.005	6.71E-02	0.003	0.007	6.89E-01
3.387667	0.020	0.009	3.33E-02	0.034	0.014	1.57E-02
2.913297	-0.018	0.008	1.91E-02	-0.013	0.012	2.52E-01
1.944371422	0.015	0.006	1.12E-02	0.011	0.009	2.19E-01
1.058207977	0.011	0.005	3.87E-02	-0.001	0.008	8.53E-01
3.094971	-0.021	0.008	9.50E-03	-0.016	0.012	1.84E-01
3.341912	-0.023	0.012	5.14E-02	0.005	0.018	7.97E-01
3.949173	-0.018	0.008	3.64E-02	0.005	0.013	6.87E-01
7.961468	0.014	0.007	3.39E-02	0.010	0.010	2.91E-01
2.693943235	-0.018	0.006	3.80E-03	-0.009	0.009	3.43E-01
2.415377049	-0.018	0.009	3.18E-02	0.006	0.013	6.28E-01
2.710428432	-0.016	0.006	3.96E-03	-0.019	0.009	2.75E-02
5.712416	0.022	0.010	3.61E-02	0.013	0.016	4.00E-01
6.772852	-0.013	0.007	6.73E-02	0.010	0.011	3.61E-01
2.705718375	-0.017	0.006	3.66E-03	-0.024	0.009	4.97E-03
1.195808906	0.009	0.004	2.45E-02	0.008	0.006	2.29E-01
2.990677	-0.010	0.004	1.65E-02	0.002	0.006	7.95E-01
0.871487888	0.041	0.016	1.09E-02	0.022	0.024	3.72E-01
3.559248	-0.018	0.008	2.59E-02	0.017	0.012	1.46E-01
2.943913	-0.012	0.005	2.04E-02	-0.007	0.007	3.41E-01
2.056067042	0.016	0.006	7.21E-03	0.008	0.009	3.36E-01
0.91589699	-0.011	0.005	2.27E-02	-0.028	0.007	1.34E-04
7.222662	-0.022	0.010	2.26E-02	-0.026	0.015	7.56E-02
1.834357964	-0.022	0.010	1.95E-02	-0.027	0.014	5.64E-02
1.192781013	0.010	0.004	2.17E-02	0.004	0.006	5.26E-01
1.557810374	0.054	0.021	1.14E-02	0.012	0.032	6.96E-01
8.569738	0.020	0.009	3.07E-02	0.017	0.014	2.30E-01
4.258018	0.046	0.018	9.50E-03	0.018	0.026	5.02E-01
7.048727	-0.016	0.008	3.29E-02	-0.023	0.011	4.40E-02
3.062337	-0.014	0.007	3.02E-02	0.000	0.010	9.65E-01
1.016153903	0.028	0.010	7.88E-03	0.018	0.015	2.53E-01
0.915560558	-0.012	0.005	2.05E-02	-0.030	0.007	7.82E-05

7.784168	-0.023	0.009	9.51E-03	0.008	0.013	5.33E-01
1.947399315	0.015	0.006	1.42E-02	0.006	0.009	5.17E-01
3.474803	0.020	0.010	3.34E-02	0.036	0.014	1.04E-02
3.959939	-0.022	0.009	1.25E-02	-0.011	0.013	4.03E-01
3.494989	0.020	0.010	3.84E-02	0.034	0.014	1.98E-02
4.057841	0.047	0.018	1.10E-02	0.015	0.027	5.93E-01
2.51025104	-0.031	0.015	3.58E-02	-0.007	0.022	7.54E-01
5.341668	0.043	0.018	1.67E-02	0.005	0.027	8.38E-01
4.056495	0.046	0.018	1.03E-02	0.010	0.027	7.20E-01
1.558146807	0.054	0.021	1.12E-02	0.013	0.032	6.78E-01
4.063897	0.047	0.018	9.26E-03	0.016	0.027	5.64E-01
3.533006	0.018	0.007	1.40E-02	0.029	0.011	8.39E-03
2.317811598	-0.012	0.005	3.46E-02	-0.005	0.008	5.15E-01
2.209143871	0.051	0.020	1.10E-02	0.006	0.030	8.45E-01
1.949754343	0.015	0.006	1.38E-02	0.008	0.009	3.92E-01
5.177489	0.040	0.015	7.78E-03	0.001	0.023	9.71E-01
5.391123	0.017	0.007	1.80E-02	-0.010	0.011	3.74E-01
1.020527526	0.019	0.007	9.66E-03	0.011	0.011	3.07E-01
0.698561537	0.037	0.014	1.01E-02	0.000	0.021	9.97E-01
1.396995596	0.017	0.006	6.65E-03	0.010	0.009	2.62E-01
3.707615	0.016	0.007	2.50E-02	0.027	0.011	1.22E-02
1.557473942	0.053	0.021	1.20E-02	0.011	0.031	7.33E-01
3.444188	0.019	0.009	3.67E-02	0.036	0.013	6.56E-03
5.380357	0.021	0.008	1.10E-02	-0.008	0.012	5.05E-01
1.333746269	0.041	0.016	9.30E-03	0.020	0.024	3.95E-01
1.397668461	0.015	0.006	7.20E-03	0.011	0.009	2.00E-01
3.670607	-0.010	0.005	2.98E-02	0.001	0.007	8.81E-01
4.265083	0.039	0.015	1.12E-02	0.034	0.023	1.36E-01
8.862098	0.016	0.008	4.85E-02	-0.008	0.012	5.26E-01
1.411462197	0.014	0.005	6.71E-03	0.013	0.008	8.56E-02
1.615003915	0.017	0.007	1.11E-02	0.000	0.010	9.68E-01
6.725079	-0.028	0.012	1.44E-02	-0.041	0.017	1.82E-02
4.057505	0.046	0.018	1.14E-02	0.011	0.027	6.87E-01
1.149044776	0.015	0.006	1.13E-02	0.001	0.009	9.47E-01
3.470766	0.019	0.009	3.93E-02	0.037	0.014	6.84E-03
2.148249572	-0.024	0.009	8.41E-03	0.003	0.013	7.94E-01
2.979911	-0.009	0.004	2.82E-02	0.003	0.006	5.79E-01
3.612404	-0.019	0.009	2.71E-02	0.008	0.013	5.36E-01
2.477617079	-0.022	0.008	9.50E-03	-0.010	0.012	4.42E-01
3.011872	-0.011	0.005	1.62E-02	-0.007	0.007	2.92E-01
3.341576	-0.023	0.012	5.26E-02	0.007	0.017	6.85E-01
5.754471	0.018	0.008	2.22E-02	0.008	0.012	5.25E-01
2.921708	-0.016	0.006	1.00E-02	-0.006	0.009	5.22E-01

2.710091999	-0.017	0.006	3.78E-03	-0.018	0.009	3.52E-02
1.213976266	0.014	0.007	2.83E-02	0.010	0.010	3.02E-01
3.453608	0.018	0.009	4.09E-02	0.035	0.013	7.12E-03
2.210153169	0.052	0.021	1.16E-02	0.009	0.031	7.78E-01
6.499332	0.022	0.009	1.72E-02	0.003	0.014	7.97E-01
2.689906044	-0.016	0.006	5.22E-03	-0.004	0.008	6.61E-01
3.426693	0.018	0.008	2.50E-02	0.035	0.012	2.84E-03
2.210826034	0.054	0.021	1.14E-02	0.012	0.032	7.09E-01
0.934400783	-0.010	0.005	2.85E-02	-0.019	0.007	8.43E-03
2.381060925	-0.017	0.007	1.12E-02	-0.010	0.010	3.30E-01
3.473794	0.018	0.009	4.81E-02	0.040	0.014	3.92E-03
2.057412772	0.014	0.005	1.05E-02	0.011	0.008	1.67E-01
5.420729	0.016	0.007	3.17E-02	0.011	0.011	3.24E-01
2.967799	-0.011	0.004	1.50E-02	-0.002	0.007	7.83E-01
6.131611	0.013	0.007	4.50E-02	0.029	0.010	2.94E-03
2.469542696	-0.021	0.009	1.44E-02	-0.009	0.013	4.82E-01
1.398677759	0.014	0.005	8.27E-03	0.011	0.008	1.65E-01
1.61567678	0.017	0.007	1.10E-02	0.004	0.010	6.96E-01
4.269793	0.040	0.016	1.01E-02	0.029	0.023	2.13E-01
1.950090776	0.014	0.006	1.40E-02	0.005	0.009	5.40E-01
2.125708588	-0.022	0.008	9.24E-03	0.005	0.013	6.62E-01
3.505419	0.020	0.009	3.30E-02	0.036	0.014	1.19E-02
5.465475	0.015	0.008	4.44E-02	0.013	0.011	2.32E-01
5.171769	0.030	0.012	8.66E-03	0.002	0.017	9.02E-01
1.313560313	0.034	0.013	6.94E-03	0.018	0.019	3.51E-01
4.071298	0.048	0.019	1.05E-02	0.015	0.028	5.98E-01
5.746733	0.026	0.011	1.68E-02	0.011	0.016	5.09E-01
5.471531	0.019	0.009	3.17E-02	-0.002	0.013	8.97E-01
7.28894	-0.020	0.010	4.24E-02	0.001	0.015	9.56E-01
0.651797406	-0.025	0.010	1.00E-02	-0.011	0.015	4.35E-01
3.970705	-0.020	0.008	1.29E-02	-0.008	0.012	5.21E-01
8.135068	0.018	0.009	3.46E-02	-0.002	0.013	8.61E-01
7.299369	-0.022	0.009	2.08E-02	0.016	0.014	2.43E-01
5.745723	0.017	0.010	8.82E-02	-0.007	0.015	6.46E-01
5.997711	0.022	0.010	2.95E-02	0.014	0.015	3.77E-01
5.365554	0.027	0.011	1.21E-02	-0.001	0.016	9.35E-01
3.452599	0.016	0.008	4.09E-02	0.035	0.012	3.70E-03
4.065579	0.041	0.016	1.10E-02	0.014	0.024	5.57E-01
3.848243	0.017	0.008	3.43E-02	0.031	0.012	8.20E-03
8.511536	-0.020	0.008	1.75E-02	-0.026	0.013	3.66E-02
3.882896	0.017	0.008	3.01E-02	0.029	0.012	1.29E-02
4.067598	0.047	0.018	1.02E-02	0.017	0.027	5.33E-01
4.065915	0.041	0.016	1.08E-02	0.011	0.024	6.41E-01

5.17076	0.031	0.012	1.17E-02	-0.003	0.018	8.53E-01
1.609957426	0.020	0.008	1.12E-02	0.003	0.012	8.05E-01
3.780621	0.018	0.008	1.89E-02	0.020	0.011	7.34E-02
4.061878	0.044	0.017	1.02E-02	0.011	0.025	6.66E-01
2.114606313	-0.014	0.007	5.96E-02	-0.012	0.011	3.03E-01
3.417946	0.018	0.009	4.53E-02	0.038	0.013	4.94E-03
3.152501	-0.023	0.010	2.52E-02	-0.021	0.015	1.71E-01
5.176816	0.041	0.016	9.76E-03	0.000	0.023	9.85E-01
2.006947884	0.028	0.011	8.36E-03	0.006	0.016	7.25E-01
6.234896	0.019	0.008	2.39E-02	0.015	0.013	2.38E-01
3.034413	0.020	0.011	5.55E-02	0.032	0.016	4.52E-02
5.225935	0.012	0.006	5.89E-02	0.024	0.009	1.01E-02
2.007620749	0.028	0.011	9.04E-03	0.005	0.016	7.44E-01
3.538725	0.020	0.009	1.80E-02	0.029	0.013	2.43E-02
4.067261	0.045	0.018	9.64E-03	0.012	0.026	6.50E-01
3.104727	-0.020	0.008	1.30E-02	-0.012	0.012	3.42E-01
5.233336	0.014	0.007	5.25E-02	0.034	0.011	1.46E-03
2.974528	-0.010	0.004	1.71E-02	-0.004	0.007	5.48E-01
2.051356986	0.016	0.006	1.22E-02	0.008	0.009	4.21E-01
0.693178615	0.037	0.015	1.45E-02	0.016	0.023	4.93E-01
3.030039	-0.010	0.005	3.32E-02	-0.003	0.007	6.18E-01
2.007284316	0.028	0.011	8.91E-03	0.005	0.016	7.45E-01
0.523616589	0.018	0.007	1.55E-02	0.015	0.011	1.65E-01
2.317475165	-0.011	0.005	4.44E-02	-0.007	0.008	3.61E-01
3.443179	0.017	0.008	4.28E-02	0.032	0.012	8.97E-03
7.797626	-0.025	0.010	1.01E-02	-0.023	0.014	1.12E-01
6.193179	0.017	0.008	3.88E-02	0.013	0.012	3.01E-01
3.062	-0.014	0.007	2.88E-02	0.002	0.010	8.51E-01
3.084541	-0.015	0.006	1.71E-02	0.007	0.010	4.74E-01
1.90130805	-0.015	0.006	1.50E-02	-0.005	0.009	5.93E-01
6.222448	0.019	0.009	4.19E-02	-0.013	0.014	3.30E-01
3.982816	-0.018	0.009	4.81E-02	-0.015	0.014	2.80E-01
7.794598	-0.018	0.008	2.20E-02	0.003	0.011	8.16E-01
2.209480303	0.051	0.020	1.19E-02	0.006	0.030	8.37E-01
1.622068999	0.016	0.007	1.57E-02	0.010	0.010	3.36E-01
5.168405	0.026	0.010	1.10E-02	-0.011	0.015	4.61E-01
0.916569856	-0.011	0.005	2.63E-02	-0.028	0.007	1.36E-04
2.490065084	-0.036	0.015	1.58E-02	-0.011	0.022	6.28E-01
3.46942	0.017	0.009	5.25E-02	0.037	0.013	5.05E-03
3.443515	0.017	0.008	4.35E-02	0.032	0.013	1.09E-02
1.105308539	0.017	0.007	1.70E-02	0.006	0.011	5.53E-01
1.125830927	0.016	0.007	2.15E-02	0.002	0.010	8.06E-01
6.000066	0.016	0.008	4.93E-02	0.003	0.012	7.96E-01

2.225629068	0.051	0.023	2.85E-02	0.031	0.034	3.66E-01
5.68752	0.022	0.009	1.84E-02	0.004	0.014	7.89E-01
6.09965	0.019	0.008	2.40E-02	0.021	0.012	9.40E-02
2.476607781	-0.022	0.009	1.64E-02	-0.003	0.014	8.08E-01
3.489943	0.020	0.010	3.69E-02	0.038	0.014	7.84E-03
7.694677	-0.021	0.010	3.87E-02	-0.019	0.015	2.21E-01
2.42849792	-0.021	0.009	1.97E-02	0.001	0.013	9.47E-01
2.395527526	-0.022	0.011	4.31E-02	-0.015	0.016	3.71E-01
2.05707634	0.014	0.005	9.85E-03	0.011	0.008	1.95E-01
2.253552973	0.022	0.009	1.23E-02	0.012	0.013	3.70E-01
2.956024	-0.010	0.005	2.34E-02	-0.005	0.007	4.81E-01
3.819647	0.014	0.007	4.19E-02	0.029	0.010	3.29E-03
3.592555	-0.011	0.006	6.49E-02	0.012	0.009	1.92E-01
4.269457	0.039	0.015	1.10E-02	0.031	0.023	1.74E-01
2.056403474	0.015	0.006	8.80E-03	0.009	0.009	3.04E-01
2.97621	-0.009	0.004	3.38E-02	0.001	0.006	8.91E-01
1.333409836	0.040	0.016	9.85E-03	0.020	0.023	3.88E-01
2.912288	-0.020	0.008	1.10E-02	-0.009	0.012	4.46E-01
2.591667727	-0.033	0.016	4.43E-02	-0.006	0.025	7.96E-01
4.260373	0.039	0.015	1.11E-02	0.020	0.023	3.81E-01
3.057963	-0.012	0.006	3.94E-02	-0.007	0.008	4.32E-01
7.377085	-0.016	0.009	6.96E-02	-0.012	0.014	3.64E-01
3.600966	-0.015	0.007	2.52E-02	-0.002	0.010	8.56E-01
7.919414	-0.024	0.009	8.20E-03	-0.023	0.014	9.65E-02
1.930577685	0.010	0.005	2.56E-02	0.008	0.007	2.12E-01
2.209816736	0.051	0.020	1.23E-02	0.008	0.031	7.95E-01
3.600629	-0.015	0.007	2.56E-02	0.002	0.010	8.22E-01
1.214312699	0.014	0.007	2.98E-02	0.010	0.010	3.11E-01
1.615340347	0.017	0.007	1.21E-02	0.006	0.010	5.62E-01
1.113046489	0.015	0.007	2.89E-02	-0.002	0.011	8.24E-01
5.238383	0.013	0.007	7.21E-02	0.013	0.011	2.27E-01
3.957584	-0.018	0.008	3.23E-02	-0.031	0.012	1.09E-02
4.06356	0.047	0.018	9.96E-03	0.012	0.027	6.49E-01
6.278632	-0.017	0.009	4.90E-02	-0.002	0.013	8.90E-01
1.313896746	0.033	0.012	7.04E-03	0.017	0.018	3.58E-01
5.38675	0.020	0.008	1.33E-02	0.014	0.012	2.46E-01
5.38574	0.019	0.008	1.59E-02	0.006	0.012	6.42E-01
6.290071	0.017	0.008	3.97E-02	0.019	0.013	1.40E-01
3.02432	-0.012	0.005	1.97E-02	-0.010	0.007	1.78E-01
8.642071	0.017	0.008	4.10E-02	-0.016	0.013	2.19E-01
0.775941033	0.010	0.005	3.17E-02	0.009	0.007	2.16E-01
5.176479	0.040	0.015	1.01E-02	-0.005	0.023	8.25E-01
7.761291	-0.019	0.008	1.64E-02	-0.036	0.012	2.69E-03

5.37161	0.020	0.008	1.57E-02	0.009	0.012	4.72E-01
2.692597504	-0.017	0.006	4.84E-03	-0.007	0.009	4.15E-01
7.711499	-0.024	0.011	2.66E-02	-0.003	0.016	8.72E-01
2.210489601	0.052	0.021	1.24E-02	0.009	0.031	7.77E-01
1.397332028	0.016	0.006	6.89E-03	0.010	0.009	2.60E-01
7.759272	-0.026	0.010	1.17E-02	-0.017	0.015	2.59E-01
8.587569	-0.021	0.010	3.10E-02	-0.008	0.015	5.74E-01
8.750403	-0.017	0.009	6.11E-02	-0.003	0.014	8.52E-01
2.007957181	0.028	0.011	9.90E-03	0.005	0.016	7.58E-01
1.20152826	0.010	0.005	3.35E-02	0.009	0.007	2.31E-01
4.270803	0.040	0.016	1.13E-02	0.020	0.023	3.89E-01
3.541753	0.016	0.009	6.65E-02	0.029	0.013	2.48E-02
1.953118669	0.013	0.006	1.82E-02	0.008	0.008	3.51E-01
7.675837	0.018	0.009	3.26E-02	0.012	0.013	3.48E-01
3.825366	0.014	0.007	3.83E-02	0.032	0.010	1.62E-03
3.866074	-0.010	0.005	3.28E-02	0.005	0.007	5.24E-01
2.39990115	-0.020	0.010	4.72E-02	-0.003	0.015	8.31E-01
4.271139	0.040	0.016	1.14E-02	0.022	0.023	3.44E-01
1.26511402	0.043	0.017	1.31E-02	0.010	0.026	6.96E-01
1.645282848	0.012	0.005	2.82E-02	0.006	0.008	4.75E-01
2.260618057	0.014	0.006	1.74E-02	0.008	0.009	3.93E-01
3.820656	0.014	0.007	4.48E-02	0.031	0.010	2.71E-03
7.680211	0.019	0.009	4.13E-02	0.004	0.014	7.45E-01
6.546096	0.023	0.010	2.82E-02	-0.015	0.016	3.20E-01
7.947675	0.019	0.010	5.78E-02	0.011	0.015	4.59E-01
7.674155	0.016	0.008	4.20E-02	0.015	0.012	2.07E-01
2.052029851	0.015	0.006	1.26E-02	0.008	0.009	3.80E-01
4.265756	0.038	0.015	1.28E-02	0.032	0.023	1.58E-01
2.915316	-0.018	0.008	1.53E-02	-0.010	0.011	3.55E-01
5.76961	0.013	0.007	7.08E-02	0.005	0.010	6.30E-01
1.557137509	0.052	0.021	1.34E-02	0.009	0.031	7.77E-01
4.270466	0.040	0.016	1.09E-02	0.024	0.023	3.10E-01
2.305363592	-0.011	0.005	2.05E-02	-0.010	0.007	1.43E-01
1.095215562	0.016	0.008	3.41E-02	0.005	0.011	6.44E-01
1.821573526	-0.023	0.010	1.66E-02	-0.006	0.014	6.88E-01
1.102280646	0.020	0.008	1.28E-02	-0.003	0.012	8.02E-01
2.008293614	0.029	0.011	1.04E-02	0.005	0.017	7.58E-01
7.715873	-0.024	0.010	2.32E-02	-0.010	0.016	5.26E-01
1.148371911	0.015	0.006	1.10E-02	-0.005	0.009	5.95E-01
4.066925	0.044	0.017	9.84E-03	0.012	0.025	6.37E-01
3.779275	0.020	0.008	1.52E-02	0.032	0.012	9.90E-03
1.333073403	0.039	0.015	1.00E-02	0.020	0.022	3.79E-01
3.057627	-0.011	0.006	4.76E-02	-0.003	0.008	7.35E-01

1.787593834	-0.020	0.010	4.68E-02	0.000	0.015	9.77E-01
2.495784438	-0.032	0.014	2.51E-02	-0.012	0.021	5.76E-01
3.290775	-0.037	0.016	2.08E-02	-0.049	0.024	3.77E-02
3.558911	-0.017	0.008	3.22E-02	0.020	0.012	9.55E-02
2.708409836	-0.017	0.006	6.95E-03	-0.017	0.009	7.25E-02
6.874455	0.025	0.011	2.06E-02	0.012	0.016	4.59E-01
8.053314	0.014	0.007	4.80E-02	-0.004	0.011	6.92E-01
2.006611451	0.028	0.011	8.87E-03	0.005	0.016	7.36E-01
2.64145975	-0.038	0.014	5.83E-03	0.007	0.021	7.52E-01
6.804477	0.016	0.008	6.37E-02	0.014	0.013	2.77E-01
3.808881	0.014	0.007	4.10E-02	0.022	0.010	3.11E-02
5.465138	0.014	0.007	3.60E-02	0.014	0.010	1.65E-01
5.824112	0.015	0.007	3.92E-02	0.008	0.011	4.27E-01
4.05885	0.047	0.018	1.10E-02	0.014	0.027	6.02E-01
3.720399	0.018	0.008	3.17E-02	0.031	0.012	1.36E-02
4.066252	0.041	0.016	1.08E-02	0.008	0.024	7.30E-01
7.045026	-0.031	0.013	1.55E-02	-0.020	0.019	2.92E-01
1.639563494	0.013	0.006	2.80E-02	0.004	0.009	6.77E-01
2.051020553	0.016	0.006	1.29E-02	0.007	0.010	4.50E-01
1.321634695	0.035	0.014	1.12E-02	0.016	0.021	4.41E-01
5.172106	0.027	0.011	1.07E-02	0.005	0.016	7.39E-01
2.055730609	0.016	0.006	9.28E-03	0.008	0.009	3.80E-01
4.058514	0.046	0.018	1.22E-02	0.017	0.027	5.43E-01
3.653449	-0.010	0.005	2.97E-02	0.004	0.007	5.43E-01
7.217952	-0.025	0.010	1.75E-02	-0.015	0.015	3.30E-01
5.870876	0.017	0.007	2.28E-02	-0.009	0.011	4.24E-01
5.537808	0.018	0.008	2.16E-02	0.017	0.011	1.31E-01
2.999424	-0.009	0.004	2.45E-02	0.001	0.006	8.25E-01
8.391093	0.014	0.007	4.63E-02	-0.001	0.011	9.30E-01
7.780804	-0.026	0.010	1.05E-02	-0.005	0.015	7.40E-01
5.177825	0.040	0.015	8.30E-03	0.005	0.023	8.21E-01
3.483551	0.018	0.009	5.07E-02	0.031	0.013	2.24E-02
3.442842	0.016	0.008	4.37E-02	0.030	0.012	1.16E-02
1.323316858	0.035	0.014	1.02E-02	0.019	0.020	3.52E-01
8.305302	-0.015	0.006	2.38E-02	-0.011	0.010	2.54E-01
7.882743	-0.020	0.009	2.16E-02	0.004	0.013	7.70E-01
2.009639344	0.030	0.012	1.10E-02	0.006	0.017	7.24E-01
5.798207	0.017	0.009	6.55E-02	0.025	0.014	7.74E-02
8.062735	-0.019	0.008	1.70E-02	0.009	0.012	4.55E-01
7.307443	-0.021	0.010	3.17E-02	-0.016	0.015	2.72E-01
3.028694	-0.010	0.005	3.51E-02	-0.006	0.007	4.13E-01
3.443851	0.017	0.009	4.45E-02	0.033	0.013	9.60E-03
5.216515	0.010	0.005	4.58E-02	0.003	0.008	6.95E-01

7.940273	-0.020	0.009	2.76E-02	-0.015	0.013	2.79E-01
2.691251774	-0.016	0.006	6.87E-03	-0.003	0.009	6.94E-01
7.044353	-0.034	0.014	2.00E-02	-0.011	0.022	6.04E-01
3.855309	0.014	0.007	5.57E-02	0.029	0.011	9.51E-03
3.889288	0.013	0.007	4.62E-02	0.031	0.010	1.68E-03
2.009302912	0.030	0.012	1.10E-02	0.006	0.017	7.40E-01
5.233673	0.015	0.008	5.29E-02	0.038	0.011	7.23E-04
6.917518	-0.021	0.010	2.96E-02	-0.016	0.014	2.74E-01
4.27013	0.040	0.016	1.12E-02	0.028	0.023	2.39E-01
2.009975777	0.030	0.012	1.11E-02	0.006	0.018	7.31E-01
3.418283	0.018	0.009	4.87E-02	0.037	0.014	7.19E-03
8.145833	0.016	0.008	3.96E-02	0.015	0.012	2.06E-01
6.715995	-0.021	0.009	1.79E-02	-0.024	0.013	7.02E-02
7.682229	0.017	0.008	3.30E-02	0.007	0.012	5.41E-01
2.055394177	0.016	0.006	1.03E-02	0.007	0.009	4.21E-01
1.621059701	0.016	0.007	1.35E-02	0.010	0.010	3.19E-01
7.741441	-0.025	0.012	3.77E-02	-0.035	0.018	4.94E-02
1.184033766	0.011	0.004	1.22E-02	0.005	0.006	4.72E-01
0.871151456	0.040	0.016	1.36E-02	0.020	0.024	4.00E-01
4.26542	0.038	0.015	1.38E-02	0.031	0.023	1.72E-01
0.703271593	0.023	0.010	1.85E-02	0.024	0.015	1.02E-01
3.23089	0.016	0.007	2.37E-02	0.034	0.010	1.01E-03
0.961651823	0.010	0.004	1.77E-02	0.014	0.006	3.27E-02
8.727189	0.013	0.007	5.77E-02	0.014	0.010	1.59E-01
1.955137264	0.013	0.005	1.61E-02	0.005	0.008	5.14E-01
2.399564717	-0.020	0.010	3.96E-02	-0.007	0.015	6.27E-01
7.736395	-0.025	0.011	2.85E-02	-0.026	0.017	1.28E-01
2.348763396	0.027	0.009	3.82E-03	0.016	0.014	2.47E-01
0.698225104	0.036	0.015	1.25E-02	-0.003	0.022	8.81E-01
5.226944	0.015	0.008	5.37E-02	0.035	0.011	1.90E-03
1.948745045	0.014	0.006	1.59E-02	0.010	0.009	2.39E-01
5.341331	0.045	0.020	2.04E-02	0.006	0.029	8.49E-01
7.214924	-0.026	0.011	2.45E-02	-0.005	0.017	7.57E-01
3.086224	-0.014	0.007	3.34E-02	0.008	0.010	4.18E-01
5.225598	0.011	0.006	6.87E-02	0.022	0.009	1.62E-02
1.611639589	0.020	0.008	1.10E-02	0.002	0.012	8.80E-01
3.252421	-0.014	0.007	3.29E-02	0.001	0.010	9.36E-01
1.19244458	0.010	0.004	2.54E-02	0.004	0.006	5.82E-01
1.556801077	0.051	0.021	1.43E-02	0.010	0.031	7.51E-01
0.898402496	0.025	0.010	1.03E-02	-0.003	0.015	8.10E-01
5.524687	0.018	0.008	2.62E-02	-0.010	0.012	3.92E-01
2.010648642	0.030	0.012	1.10E-02	0.007	0.017	7.07E-01
4.257682	0.045	0.018	1.23E-02	0.015	0.027	5.70E-01

4.275176	0.037	0.015	1.37E-02	0.017	0.023	4.50E-01
5.948929	0.018	0.007	1.30E-02	0.000	0.011	9.96E-01
2.056739907	0.014	0.006	1.08E-02	0.009	0.008	2.72E-01
3.118858	-0.020	0.008	1.54E-02	-0.019	0.012	1.35E-01
3.95893	-0.019	0.009	2.45E-02	-0.016	0.013	2.17E-01
6.653418	-0.016	0.007	1.79E-02	-0.012	0.010	2.38E-01
6.123537	0.017	0.009	5.56E-02	0.003	0.013	8.35E-01
4.057168	0.045	0.018	1.28E-02	0.007	0.027	8.05E-01
3.013891	-0.011	0.005	2.40E-02	-0.006	0.007	4.19E-01
5.227281	0.016	0.008	4.96E-02	0.039	0.012	1.03E-03
8.398831	0.021	0.010	3.03E-02	0.004	0.014	7.68E-01
6.658465	-0.018	0.009	3.38E-02	-0.005	0.013	6.92E-01
1.946390017	0.014	0.006	1.69E-02	0.006	0.009	5.01E-01
1.40406068	0.012	0.005	1.26E-02	0.012	0.007	9.56E-02
2.008630046	0.029	0.011	1.15E-02	0.005	0.017	7.81E-01
2.010312209	0.030	0.012	1.12E-02	0.006	0.018	7.29E-01
5.583899	0.017	0.007	2.35E-02	0.006	0.011	6.03E-01
5.701651	0.016	0.008	3.57E-02	0.009	0.012	4.33E-01
2.05068412	0.016	0.007	1.38E-02	0.007	0.010	4.80E-01
1.057198679	0.012	0.006	3.49E-02	0.007	0.008	4.05E-01
1.175959383	0.016	0.006	8.82E-03	-0.002	0.009	8.14E-01
3.095307	-0.020	0.008	1.40E-02	-0.019	0.012	1.21E-01
1.935287742	0.013	0.005	1.91E-02	0.012	0.008	1.47E-01
2.399228285	-0.021	0.010	2.81E-02	-0.013	0.014	3.77E-01
2.126045021	-0.021	0.008	1.17E-02	0.005	0.012	6.55E-01
1.556464644	0.050	0.021	1.46E-02	0.010	0.031	7.55E-01
2.118643504	-0.017	0.008	2.64E-02	-0.007	0.012	5.23E-01
5.714099	0.019	0.009	2.39E-02	-0.005	0.013	7.16E-01
4.261719	0.035	0.014	1.41E-02	0.017	0.021	4.24E-01
1.945044287	0.014	0.006	1.38E-02	0.011	0.009	2.21E-01
3.653113	-0.010	0.005	2.93E-02	0.003	0.007	6.35E-01
1.943698556	0.014	0.006	1.57E-02	0.011	0.009	1.91E-01
2.689233178	-0.015	0.005	6.70E-03	-0.004	0.008	6.18E-01
8.569402	0.018	0.009	3.49E-02	-0.001	0.013	9.36E-01
7.289276	-0.020	0.010	4.20E-02	-0.009	0.015	5.63E-01
2.008966479	0.029	0.012	1.16E-02	0.005	0.017	7.68E-01
4.056832	0.045	0.018	1.28E-02	0.006	0.027	8.18E-01
5.852709	0.020	0.009	1.79E-02	0.015	0.013	2.40E-01
2.254225838	0.019	0.008	1.70E-02	0.010	0.012	3.95E-01
1.953455102	0.013	0.006	1.92E-02	0.007	0.008	3.70E-01
2.477280646	-0.021	0.009	1.67E-02	-0.004	0.013	7.40E-01
0.527317348	0.015	0.008	5.24E-02	-0.007	0.012	5.74E-01
2.415713482	-0.017	0.009	4.55E-02	0.005	0.013	6.76E-01

6.661156	0.020	0.008	1.66E-02	0.007	0.012	5.82E-01
2.709755566	-0.017	0.006	4.74E-03	-0.019	0.009	2.72E-02
6.251718	0.023	0.009	1.58E-02	0.009	0.014	5.08E-01
4.264411	0.037	0.015	1.40E-02	0.028	0.022	2.20E-01
7.1833	0.019	0.008	2.32E-02	-0.005	0.012	7.07E-01
2.410666993	-0.027	0.012	2.34E-02	-0.005	0.018	7.78E-01
7.584664	0.018	0.008	2.20E-02	0.007	0.012	5.63E-01
4.26071	0.037	0.015	1.29E-02	0.020	0.022	3.56E-01
3.453272	0.016	0.008	5.01E-02	0.034	0.012	5.96E-03
2.208471006	0.048	0.019	1.39E-02	-0.001	0.029	9.83E-01
1.942016393	0.014	0.006	1.62E-02	0.013	0.009	1.29E-01
5.17547	0.038	0.015	1.24E-02	-0.009	0.023	6.84E-01
3.740585	0.017	0.009	4.98E-02	0.037	0.013	3.75E-03
3.902746	0.016	0.008	4.10E-02	0.031	0.012	6.88E-03
6.131948	0.012	0.006	5.60E-02	0.024	0.009	7.14E-03
3.070075	-0.012	0.006	3.40E-02	-0.007	0.009	4.14E-01
1.952782236	0.013	0.006	1.83E-02	0.008	0.008	3.33E-01
5.42443	0.013	0.006	3.04E-02	-0.006	0.009	5.33E-01
5.175806	0.039	0.015	1.26E-02	-0.005	0.023	8.30E-01
3.957248	-0.015	0.008	6.02E-02	-0.031	0.012	1.22E-02
5.813683	0.016	0.008	4.80E-02	0.008	0.012	4.91E-01
2.208807438	0.048	0.020	1.40E-02	0.002	0.029	9.33E-01
1.41179863	0.013	0.005	1.01E-02	0.007	0.007	3.36E-01
4.073317	0.051	0.020	1.23E-02	0.029	0.030	3.36E-01
5.342004	0.044	0.019	2.02E-02	0.003	0.028	9.10E-01
2.998751	-0.009	0.004	2.62E-02	0.001	0.006	8.67E-01
5.38103	0.019	0.009	3.03E-02	0.001	0.013	9.22E-01
2.055057744	0.016	0.006	1.10E-02	0.008	0.009	3.73E-01
8.827782	0.020	0.009	2.36E-02	-0.001	0.013	9.27E-01
2.600751407	-0.031	0.014	2.55E-02	-0.004	0.021	8.53E-01
2.382406655	-0.018	0.007	1.68E-02	-0.003	0.011	7.64E-01
5.373629	0.022	0.009	1.44E-02	0.010	0.014	4.71E-01
5.769946	0.016	0.008	5.79E-02	0.011	0.013	3.84E-01
5.703333	0.020	0.008	1.65E-02	0.009	0.012	4.68E-01
1.072001713	0.019	0.009	3.97E-02	0.001	0.014	9.30E-01
5.378339	0.024	0.010	1.45E-02	0.019	0.015	2.14E-01
2.010985075	0.029	0.012	1.17E-02	0.006	0.017	7.13E-01
4.257345	0.045	0.018	1.50E-02	0.012	0.027	6.49E-01
6.584786	0.016	0.007	2.90E-02	0.019	0.011	7.21E-02
3.483887	0.017	0.009	5.31E-02	0.029	0.013	3.01E-02
6.58445	0.017	0.008	3.85E-02	0.021	0.012	9.17E-02
2.109559824	-0.012	0.007	8.62E-02	-0.029	0.010	4.10E-03
4.062551	0.044	0.018	1.18E-02	0.011	0.026	6.63E-01

1.214649131	0.014	0.007	3.41E-02	0.010	0.010	3.22E-01
1.332736971	0.037	0.015	1.10E-02	0.019	0.022	3.93E-01
1.020191094	0.018	0.008	1.77E-02	0.003	0.011	7.80E-01
4.25499	0.046	0.019	1.38E-02	0.020	0.028	4.77E-01
2.915652	-0.018	0.008	2.03E-02	-0.011	0.011	3.41E-01
7.324601	-0.016	0.007	1.55E-02	0.004	0.010	7.04E-01
2.052702716	0.015	0.006	1.51E-02	0.008	0.009	3.50E-01
3.824357	0.014	0.007	4.70E-02	0.034	0.011	1.45E-03
2.006275018	0.028	0.011	9.96E-03	0.005	0.016	7.50E-01
5.224253	0.010	0.006	8.09E-02	0.016	0.008	5.75E-02
1.403387815	0.012	0.005	1.06E-02	0.010	0.007	1.31E-01
5.408281	0.019	0.008	2.34E-02	-0.007	0.013	5.69E-01
2.688896746	-0.015	0.005	7.35E-03	-0.004	0.008	6.31E-01
1.137269635	0.015	0.007	2.21E-02	-0.001	0.010	9.39E-01
4.064233	0.044	0.018	1.21E-02	0.017	0.026	5.13E-01
6.781263	-0.018	0.009	5.69E-02	-0.004	0.014	7.89E-01
2.0597678	0.011	0.004	1.32E-02	0.011	0.007	8.67E-02
3.673635	-0.010	0.005	3.11E-02	0.002	0.007	7.85E-01
7.78114	-0.027	0.010	8.97E-03	-0.009	0.015	5.33E-01
3.261169	0.017	0.008	3.54E-02	0.028	0.012	2.26E-02
4.071971	0.048	0.019	1.12E-02	0.022	0.028	4.26E-01
1.314233178	0.032	0.012	7.98E-03	0.015	0.018	3.90E-01
3.757743	0.018	0.009	3.61E-02	0.029	0.013	2.38E-02
3.251076	-0.015	0.006	1.45E-02	0.000	0.009	9.89E-01
2.011321507	0.029	0.012	1.20E-02	0.007	0.017	7.03E-01
0.91455126	-0.011	0.005	2.95E-02	-0.031	0.007	4.56E-05
4.263065	0.036	0.015	1.59E-02	0.021	0.022	3.39E-01
2.882345	-0.017	0.008	3.64E-02	-0.003	0.012	7.91E-01
4.258691	0.042	0.017	1.32E-02	0.020	0.025	4.29E-01
8.828119	0.017	0.008	3.95E-02	0.015	0.013	2.39E-01
2.98092	-0.009	0.004	4.05E-02	0.005	0.006	3.98E-01
3.394059	0.017	0.009	5.30E-02	0.039	0.013	2.88E-03
5.347387	0.046	0.019	1.59E-02	0.017	0.028	5.42E-01
1.196145339	0.009	0.004	3.23E-02	0.008	0.006	2.07E-01
5.928743	0.016	0.007	2.50E-02	0.010	0.011	3.41E-01
2.877299	-0.015	0.008	6.22E-02	0.005	0.012	6.86E-01
2.001228529	0.028	0.011	1.07E-02	0.005	0.017	7.49E-01
3.509119	0.016	0.008	5.54E-02	0.032	0.012	1.09E-02
3.82032	0.013	0.007	5.32E-02	0.030	0.010	2.94E-03
3.654458	-0.011	0.005	2.34E-02	0.006	0.007	4.12E-01
8.992971	0.016	0.008	4.56E-02	0.004	0.012	7.40E-01
2.113260582	-0.014	0.007	4.48E-02	-0.012	0.010	2.39E-01
1.184706631	0.011	0.004	1.68E-02	0.005	0.007	4.56E-01

8.153571	0.019	0.010	6.52E-02	0.008	0.015	6.16E-01
6.521873	0.022	0.009	1.63E-02	0.013	0.013	3.51E-01
1.487832395	-0.010	0.004	2.51E-02	-0.009	0.007	1.53E-01
3.819983	0.013	0.007	5.23E-02	0.030	0.010	2.99E-03
0.929690727	-0.008	0.005	6.44E-02	-0.023	0.007	9.28E-04
2.012330805	0.029	0.011	1.21E-02	0.007	0.017	7.00E-01
3.485906	0.017	0.009	5.39E-02	0.036	0.013	6.01E-03
1.618368241	0.015	0.006	1.55E-02	0.008	0.009	4.23E-01
2.01165794	0.029	0.012	1.21E-02	0.007	0.017	7.06E-01
2.001564962	0.028	0.011	1.08E-02	0.006	0.016	7.31E-01
2.429170785	-0.021	0.009	2.31E-02	-0.010	0.014	4.87E-01
2.994714	-0.009	0.004	2.79E-02	0.002	0.006	7.72E-01
2.395863959	-0.022	0.011	5.51E-02	-0.007	0.017	6.81E-01
3.697185	0.013	0.007	4.61E-02	0.028	0.010	4.91E-03
8.41969	0.018	0.009	5.18E-02	0.006	0.014	6.60E-01
2.48434573	-0.029	0.011	1.19E-02	-0.004	0.017	8.24E-01
7.323592	-0.016	0.006	9.36E-03	-0.009	0.009	3.06E-01
1.016490335	0.026	0.010	1.10E-02	0.006	0.015	6.98E-01
4.263401	0.036	0.015	1.56E-02	0.021	0.022	3.47E-01
2.206788843	0.044	0.018	1.44E-02	-0.002	0.027	9.51E-01
6.686389	-0.014	0.008	8.16E-02	-0.010	0.012	3.94E-01
1.609284561	0.021	0.008	1.15E-02	0.001	0.012	9.26E-01
4.07298	0.049	0.020	1.36E-02	0.025	0.030	4.08E-01
2.682840959	-0.014	0.005	6.76E-03	0.003	0.008	7.04E-01
2.864178	-0.016	0.008	6.10E-02	-0.003	0.012	8.27E-01
8.478565	0.019	0.009	3.16E-02	0.007	0.013	5.90E-01
3.847907	0.015	0.007	4.47E-02	0.028	0.011	1.02E-02
4.066588	0.041	0.016	1.19E-02	0.009	0.024	7.16E-01
3.622834	-0.012	0.006	2.98E-02	-0.006	0.008	5.10E-01
2.059431368	0.011	0.005	1.47E-02	0.011	0.007	1.01E-01
1.112710056	0.015	0.007	3.43E-02	0.003	0.011	7.50E-01
2.012667238	0.029	0.011	1.23E-02	0.006	0.017	7.14E-01
3.654795	-0.011	0.005	2.38E-02	0.005	0.007	4.91E-01
8.063071	-0.020	0.009	2.28E-02	0.001	0.013	9.66E-01
2.963762	-0.010	0.005	3.13E-02	-0.004	0.007	5.42E-01
6.419598	0.015	0.008	4.49E-02	0.006	0.011	5.90E-01
3.074112	-0.013	0.007	4.82E-02	-0.023	0.010	1.87E-02
4.062215	0.043	0.017	1.25E-02	0.010	0.026	6.87E-01
1.332400538	0.035	0.014	1.10E-02	0.018	0.021	3.87E-01
4.072644	0.048	0.020	1.36E-02	0.020	0.029	4.90E-01
2.394518229	-0.022	0.009	2.09E-02	-0.011	0.014	4.30E-01
6.957217	-0.016	0.008	3.08E-02	0.013	0.011	2.64E-01
4.056159	0.044	0.018	1.44E-02	0.004	0.027	8.77E-01

3.780284	0.018	0.008	2.11E-02	0.023	0.012	5.15E-02
6.889258	0.021	0.008	9.75E-03	-0.003	0.012	8.36E-01
1.024564717	0.012	0.005	2.12E-02	0.009	0.008	2.49E-01
3.484896	0.017	0.009	5.35E-02	0.032	0.013	1.69E-02
3.559584	-0.018	0.008	3.20E-02	0.015	0.013	2.33E-01
0.714373868	0.016	0.008	3.94E-02	0.014	0.012	2.49E-01
5.381703	0.019	0.008	2.07E-02	0.002	0.012	8.61E-01
7.215597	-0.024	0.010	2.02E-02	-0.004	0.015	7.98E-01
2.207125275	0.045	0.018	1.53E-02	-0.003	0.028	9.13E-01
6.620112	0.015	0.008	6.39E-02	-0.008	0.012	5.17E-01
8.226241	0.013	0.007	6.56E-02	0.003	0.011	7.84E-01
2.688223881	-0.015	0.005	7.18E-03	-0.004	0.008	5.97E-01
2.01300367	0.028	0.011	1.26E-02	0.006	0.017	7.02E-01
3.485569	0.017	0.009	5.38E-02	0.035	0.013	8.52E-03
4.072308	0.048	0.019	1.24E-02	0.022	0.029	4.38E-01
7.721592	-0.023	0.012	4.61E-02	-0.037	0.017	3.11E-02
5.439906	-0.017	0.008	4.01E-02	-0.018	0.012	1.46E-01
5.382376	0.020	0.009	2.46E-02	-0.010	0.013	4.57E-01
1.556128211	0.049	0.021	1.64E-02	0.010	0.031	7.56E-01
1.861609004	-0.018	0.008	2.38E-02	0.007	0.012	5.54E-01
3.592891	-0.010	0.006	7.84E-02	0.012	0.009	1.91E-01
1.93764277	0.013	0.005	1.96E-02	0.008	0.008	3.14E-01
5.896445	0.020	0.008	9.14E-03	0.011	0.011	3.44E-01
1.403051382	0.012	0.005	1.10E-02	0.008	0.007	2.35E-01
7.311817	-0.022	0.009	1.52E-02	-0.008	0.013	5.55E-01
4.261383	0.035	0.014	1.52E-02	0.018	0.022	3.94E-01
1.611303156	0.019	0.008	1.27E-02	0.001	0.012	9.24E-01
5.361854	0.030	0.013	1.86E-02	0.013	0.019	5.05E-01
2.980584	-0.009	0.004	3.99E-02	0.006	0.006	3.56E-01
2.632712503	-0.019	0.008	2.09E-02	-0.009	0.012	4.72E-01
3.056954	-0.010	0.006	8.17E-02	-0.009	0.008	2.83E-01
3.780957	0.017	0.008	3.02E-02	0.020	0.012	9.19E-02
8.114545	0.020	0.009	3.65E-02	0.019	0.014	1.70E-01
8.698592	0.020	0.009	3.23E-02	-0.008	0.014	5.65E-01
2.011994372	0.029	0.012	1.27E-02	0.007	0.017	7.03E-01
2.005938586	0.028	0.011	1.08E-02	0.005	0.016	7.63E-01
2.208134573	0.047	0.019	1.54E-02	0.000	0.029	9.90E-01
1.264777587	0.040	0.017	1.64E-02	0.008	0.025	7.44E-01
0.609070467	0.019	0.009	4.09E-02	0.016	0.014	2.59E-01
2.013340103	0.028	0.011	1.27E-02	0.006	0.017	6.99E-01
3.774901	0.019	0.009	3.13E-02	0.033	0.013	1.08E-02
2.884028	-0.019	0.008	2.36E-02	-0.005	0.012	6.67E-01
1.64292782	0.012	0.006	3.16E-02	0.025	0.008	3.45E-03

6.896323	0.019	0.009	3.34E-02	-0.007	0.013	6.16E-01
3.5939	-0.012	0.006	5.80E-02	0.007	0.009	4.53E-01
5.481624	0.015	0.007	3.97E-02	0.012	0.011	2.53E-01
1.125494495	0.015	0.007	2.89E-02	-0.002	0.010	8.11E-01
3.864729	-0.011	0.005	2.60E-02	0.004	0.007	5.96E-01
3.593564	-0.011	0.006	6.38E-02	0.008	0.009	3.87E-01
4.058177	0.045	0.018	1.45E-02	0.019	0.027	4.89E-01
1.061908735	0.012	0.006	3.23E-02	-0.005	0.008	5.68E-01
2.001901395	0.028	0.011	1.13E-02	0.006	0.016	7.35E-01
2.623965256	-0.022	0.011	4.28E-02	0.002	0.016	9.07E-01
3.452935	0.016	0.008	5.26E-02	0.033	0.012	5.86E-03
2.003583558	0.027	0.011	1.11E-02	0.005	0.016	7.66E-01
2.642469048	-0.032	0.012	7.05E-03	0.005	0.018	7.73E-01
3.117175	-0.022	0.009	1.43E-02	-0.019	0.014	1.69E-01
4.257009	0.045	0.018	1.53E-02	0.020	0.028	4.59E-01
2.000219232	0.028	0.011	1.22E-02	0.004	0.017	8.12E-01
6.075427	0.016	0.007	2.03E-02	-0.007	0.010	5.08E-01
7.215261	-0.023	0.010	2.06E-02	-0.008	0.015	5.99E-01
1.953791534	0.013	0.006	2.18E-02	0.007	0.008	4.25E-01
2.005265721	0.028	0.011	1.10E-02	0.005	0.016	7.54E-01
1.887514314	-0.015	0.006	2.04E-02	-0.003	0.009	7.65E-01
5.832523	0.016	0.008	3.91E-02	-0.012	0.012	3.03E-01
4.261046	0.036	0.015	1.50E-02	0.020	0.022	3.61E-01
6.135649	0.015	0.008	6.00E-02	-0.005	0.012	6.81E-01
7.641521	-0.019	0.011	7.02E-02	-0.022	0.016	1.67E-01
1.950427208	0.014	0.006	1.74E-02	0.004	0.009	6.17E-01
2.878645	-0.016	0.008	4.28E-02	-0.003	0.012	7.91E-01
2.052366283	0.015	0.006	1.58E-02	0.008	0.009	3.53E-01
6.571329	-0.018	0.009	3.89E-02	-0.039	0.013	2.44E-03
2.133110105	-0.022	0.009	1.73E-02	-0.001	0.014	9.26E-01
3.442506	0.015	0.008	5.25E-02	0.027	0.011	1.88E-02
3.495326	0.018	0.010	5.95E-02	0.031	0.014	3.48E-02
5.085306	0.021	0.011	5.72E-02	-0.011	0.016	4.98E-01
3.068729	-0.013	0.006	3.32E-02	-0.010	0.009	2.73E-01
2.000892097	0.028	0.011	1.19E-02	0.004	0.017	7.89E-01
2.207461708	0.045	0.019	1.61E-02	-0.002	0.028	9.29E-01
2.683177392	-0.014	0.005	7.80E-03	0.002	0.008	7.69E-01
8.231624	0.016	0.008	4.09E-02	0.005	0.012	6.71E-01
3.981471	-0.017	0.010	8.35E-02	-0.020	0.015	1.83E-01
3.141399	-0.030	0.013	1.82E-02	-0.022	0.019	2.38E-01
2.691924639	-0.016	0.006	6.77E-03	-0.007	0.009	4.61E-01
4.263738	0.036	0.015	1.62E-02	0.020	0.022	3.78E-01
2.003247125	0.027	0.011	1.10E-02	0.005	0.016	7.78E-01

2.470215562	-0.018	0.008	3.12E-02	-0.011	0.013	3.88E-01
2.396200391	-0.022	0.012	6.69E-02	-0.009	0.018	5.90E-01
3.399442	0.015	0.008	7.29E-02	0.033	0.012	8.36E-03
5.365218	0.027	0.011	1.48E-02	0.010	0.017	5.47E-01
2.002910692	0.028	0.011	1.10E-02	0.005	0.016	7.68E-01
5.419384	0.013	0.006	3.51E-02	-0.005	0.009	5.83E-01
2.00391999	0.027	0.011	1.14E-02	0.005	0.016	7.57E-01
7.873323	0.017	0.008	3.89E-02	-0.005	0.012	6.68E-01
2.005602153	0.028	0.011	1.12E-02	0.005	0.016	7.69E-01
4.27484	0.036	0.015	1.62E-02	0.019	0.023	4.05E-01
4.277531	0.034	0.014	1.63E-02	0.024	0.021	2.68E-01
5.226608	0.013	0.007	6.74E-02	0.031	0.011	3.25E-03
8.780009	0.021	0.009	2.73E-02	0.012	0.014	3.95E-01
3.522577	0.018	0.009	4.14E-02	0.039	0.013	3.44E-03
0.715383166	0.016	0.008	3.78E-02	0.009	0.012	4.60E-01
2.318820896	-0.013	0.006	4.34E-02	-0.006	0.009	5.44E-01
2.688560313	-0.015	0.005	7.67E-03	-0.005	0.008	5.57E-01
1.621396134	0.016	0.007	1.78E-02	0.015	0.010	1.36E-01
6.069035	0.019	0.008	2.13E-02	0.009	0.012	4.39E-01
2.000555664	0.028	0.011	1.24E-02	0.004	0.017	7.99E-01
3.949846	-0.016	0.009	6.46E-02	0.007	0.013	5.91E-01
3.47043	0.017	0.009	5.96E-02	0.037	0.014	7.05E-03
2.20779814	0.046	0.019	1.63E-02	0.000	0.028	9.91E-01
1.07031955	0.012	0.006	4.64E-02	0.002	0.009	8.14E-01
2.999088	-0.008	0.004	3.26E-02	0.001	0.006	8.83E-01
8.534413	-0.018	0.008	3.19E-02	-0.022	0.013	8.15E-02
4.247925	0.042	0.018	2.01E-02	0.017	0.027	5.23E-01
8.6017	0.017	0.009	5.25E-02	0.000	0.013	9.97E-01
7.616625	-0.027	0.010	9.88E-03	-0.006	0.015	6.82E-01
5.224589	0.010	0.006	8.24E-02	0.017	0.008	4.21E-02
8.809951	0.022	0.010	3.39E-02	-0.007	0.015	6.50E-01
4.274167	0.037	0.015	1.56E-02	0.015	0.023	5.10E-01
2.00257426	0.028	0.011	1.13E-02	0.005	0.016	7.66E-01
2.013676535	0.028	0.011	1.35E-02	0.006	0.017	7.14E-01
5.645466	0.011	0.006	5.69E-02	0.002	0.009	8.08E-01
3.12054	-0.020	0.008	9.18E-03	-0.006	0.012	5.95E-01
3.480186	0.019	0.010	5.61E-02	0.037	0.014	9.58E-03
2.002237827	0.027	0.011	1.18E-02	0.005	0.016	7.57E-01
0.77695033	0.009	0.005	4.48E-02	-0.003	0.007	6.87E-01
6.531293	0.018	0.007	1.50E-02	0.006	0.011	5.65E-01
4.278204	0.033	0.014	1.63E-02	0.020	0.021	3.33E-01
1.321298263	0.033	0.014	1.38E-02	0.014	0.020	4.91E-01
3.140053	-0.026	0.011	2.23E-02	-0.017	0.017	3.08E-01

7.641184	-0.019	0.011	7.91E-02	-0.013	0.016	3.98E-01
5.226271	0.012	0.007	6.96E-02	0.027	0.010	6.64E-03
2.014012968	0.027	0.011	1.34E-02	0.006	0.016	6.96E-01
1.12616736	0.015	0.007	2.56E-02	0.000	0.010	9.99E-01
3.268907	-0.021	0.010	5.01E-02	-0.018	0.016	2.38E-01
5.648831	0.019	0.009	4.79E-02	0.007	0.014	6.28E-01
3.509456	0.015	0.008	6.46E-02	0.033	0.012	8.33E-03
8.624577	0.016	0.008	4.73E-02	-0.003	0.012	8.06E-01
3.94951	-0.016	0.009	5.83E-02	0.006	0.013	6.45E-01
3.484223	0.017	0.009	5.90E-02	0.029	0.013	2.70E-02
2.147913139	-0.024	0.010	1.09E-02	0.004	0.014	7.85E-01
4.256673	0.045	0.019	1.55E-02	0.016	0.028	5.63E-01
6.073072	0.019	0.009	3.22E-02	0.005	0.013	6.81E-01
3.956911	-0.015	0.008	6.29E-02	-0.027	0.012	2.39E-02
3.528632	0.018	0.009	3.98E-02	0.039	0.013	2.42E-03
0.870815023	0.038	0.016	1.69E-02	0.019	0.023	4.31E-01
2.253889405	0.020	0.008	1.90E-02	0.011	0.013	3.84E-01
4.171892	-0.030	0.014	3.38E-02	-0.001	0.021	9.73E-01
2.222601174	0.038	0.018	3.79E-02	0.023	0.027	3.94E-01
5.545882	0.015	0.007	3.56E-02	-0.005	0.010	6.28E-01
5.417701	0.014	0.007	4.00E-02	-0.015	0.010	1.57E-01
2.710764864	-0.016	0.006	6.09E-03	-0.018	0.008	3.78E-02
1.184370198	0.010	0.004	1.66E-02	0.006	0.007	3.80E-01
6.660483	0.021	0.009	2.34E-02	-0.004	0.014	7.74E-01
1.900971617	-0.015	0.006	1.80E-02	-0.002	0.009	8.11E-01
1.115401517	0.017	0.007	2.14E-02	-0.012	0.011	2.70E-01
1.878094201	-0.011	0.006	6.19E-02	-0.006	0.009	4.67E-01
8.687826	0.014	0.008	6.88E-02	0.018	0.012	1.20E-01
2.476944213	-0.021	0.009	2.17E-02	-0.002	0.013	9.08E-01
3.623506	-0.012	0.006	3.50E-02	-0.007	0.009	4.04E-01
3.012208	-0.010	0.005	2.93E-02	-0.007	0.007	3.10E-01
2.433880842	-0.017	0.009	4.63E-02	-0.033	0.013	1.29E-02
5.526369	0.016	0.007	2.77E-02	0.002	0.011	8.80E-01
6.21942	0.016	0.009	6.28E-02	0.016	0.013	2.02E-01
1.148708344	0.015	0.006	1.45E-02	-0.001	0.009	8.85E-01
2.414031319	-0.017	0.009	5.67E-02	0.024	0.013	7.66E-02
3.13938	-0.026	0.011	1.86E-02	-0.023	0.016	1.59E-01
2.402592611	-0.019	0.010	5.15E-02	0.031	0.015	3.83E-02
7.2304	-0.020	0.010	4.03E-02	-0.020	0.015	1.82E-01
4.254654	0.044	0.018	1.61E-02	0.019	0.027	4.86E-01
5.79787	0.021	0.011	7.01E-02	0.016	0.017	3.41E-01
1.999882799	0.028	0.011	1.33E-02	0.004	0.017	8.30E-01
5.507193	0.016	0.008	4.40E-02	0.003	0.012	7.78E-01

6.335153	0.018	0.008	2.59E-02	0.005	0.012	6.62E-01
1.936297039	0.012	0.005	1.98E-02	0.016	0.008	5.10E-02
2.875617	-0.015	0.008	8.01E-02	-0.007	0.012	5.79E-01
1.163511378	0.011	0.004	9.56E-03	0.002	0.006	7.72E-01
7.158404	0.013	0.007	5.98E-02	-0.007	0.010	4.95E-01
3.865065	-0.010	0.005	3.25E-02	0.004	0.007	5.29E-01
3.285728	-0.030	0.013	2.12E-02	-0.020	0.019	2.92E-01
2.014349401	0.027	0.011	1.35E-02	0.007	0.016	6.67E-01
2.993368	-0.008	0.004	3.49E-02	0.002	0.006	6.69E-01
1.608948128	0.021	0.008	1.32E-02	0.004	0.012	7.50E-01
2.004929288	0.027	0.011	1.18E-02	0.005	0.016	7.58E-01
2.882682	-0.017	0.008	4.77E-02	0.000	0.013	9.69E-01
2.377696599	-0.019	0.009	3.24E-02	0.007	0.013	5.74E-01
3.139044	-0.024	0.011	2.53E-02	-0.013	0.016	3.97E-01
3.480523	0.018	0.010	6.01E-02	0.037	0.014	1.09E-02
5.857755	0.016	0.007	3.55E-02	0.011	0.011	3.37E-01
2.971837	-0.009	0.004	4.19E-02	-0.001	0.007	8.59E-01
3.47413	0.017	0.009	6.49E-02	0.039	0.014	5.74E-03
2.512942501	-0.026	0.013	3.86E-02	-0.023	0.019	2.13E-01
1.332064106	0.034	0.013	1.15E-02	0.017	0.020	3.82E-01
2.260281625	0.013	0.006	2.48E-02	0.012	0.009	1.88E-01
3.150482	-0.020	0.009	3.12E-02	-0.013	0.014	3.47E-01
2.148586004	-0.021	0.008	1.39E-02	0.003	0.013	8.13E-01
6.527929	0.011	0.007	1.10E-01	0.002	0.010	8.63E-01
3.566986	-0.018	0.008	2.95E-02	0.008	0.013	5.15E-01
1.214985564	0.014	0.007	3.90E-02	0.009	0.010	3.47E-01
4.277868	0.034	0.014	1.72E-02	0.023	0.021	2.82E-01
1.171922192	0.011	0.004	9.67E-03	0.000	0.006	9.54E-01
4.262728	0.034	0.015	1.83E-02	0.021	0.022	3.36E-01
8.294873	0.017	0.008	2.94E-02	0.005	0.011	6.69E-01
3.600293	-0.014	0.007	3.24E-02	0.004	0.010	6.46E-01
2.11426988	-0.013	0.007	7.69E-02	-0.013	0.011	2.48E-01
4.260037	0.037	0.016	1.62E-02	0.021	0.023	3.73E-01
1.935624174	0.012	0.005	2.21E-02	0.015	0.008	6.39E-02
6.136658	0.020	0.009	3.58E-02	-0.014	0.014	3.20E-01
1.849160998	-0.015	0.008	6.81E-02	-0.003	0.012	7.89E-01
3.343931	-0.020	0.012	9.00E-02	-0.006	0.018	7.46E-01
7.055455	-0.021	0.010	3.49E-02	-0.006	0.015	6.85E-01
6.739209	-0.024	0.010	1.43E-02	0.000	0.015	9.94E-01
2.708746269	-0.017	0.006	8.86E-03	-0.016	0.010	9.27E-02
3.951528	-0.015	0.008	7.23E-02	0.007	0.012	5.59E-01
7.675501	0.018	0.009	4.89E-02	0.014	0.014	3.08E-01
8.975813	0.016	0.009	7.01E-02	0.001	0.013	9.51E-01

2.225292635	0.046	0.022	4.03E-02	0.026	0.033	4.31E-01
2.865187	-0.016	0.008	5.58E-02	0.011	0.013	3.85E-01
2.004256423	0.027	0.011	1.24E-02	0.005	0.016	7.72E-01
2.306709322	-0.011	0.005	2.98E-02	-0.008	0.008	2.88E-01
3.474467	0.018	0.010	6.00E-02	0.037	0.014	8.97E-03
6.104697	0.017	0.009	6.21E-02	0.019	0.013	1.47E-01
3.40247	0.018	0.009	5.23E-02	0.039	0.014	5.81E-03
1.941343528	0.014	0.006	1.96E-02	0.014	0.009	1.14E-01
2.111578419	-0.014	0.007	4.94E-02	-0.015	0.011	1.59E-01
7.357908	-0.023	0.011	3.19E-02	-0.003	0.016	8.61E-01
1.629470516	0.013	0.006	3.00E-02	0.002	0.009	8.62E-01
4.264074	0.036	0.015	1.72E-02	0.023	0.022	2.98E-01
3.152837	-0.021	0.010	3.34E-02	-0.011	0.015	4.44E-01
2.385098116	-0.020	0.008	1.49E-02	0.009	0.012	4.73E-01
3.593228	-0.011	0.006	7.82E-02	0.010	0.009	2.41E-01
5.64513	0.014	0.007	3.94E-02	0.011	0.010	2.60E-01
3.557566	-0.024	0.011	2.46E-02	-0.004	0.016	7.97E-01
3.558575	-0.017	0.008	3.93E-02	0.019	0.012	1.26E-01
2.222264742	0.039	0.019	4.08E-02	0.021	0.028	4.62E-01
8.387056	0.016	0.007	2.59E-02	0.022	0.011	4.72E-02
0.691496452	0.037	0.017	2.65E-02	0.002	0.025	9.24E-01
7.212569	-0.026	0.011	2.07E-02	-0.006	0.017	7.15E-01
2.133446538	-0.019	0.009	2.61E-02	0.006	0.013	6.50E-01
5.863138	0.019	0.010	4.71E-02	-0.015	0.014	3.02E-01
6.592188	-0.017	0.007	1.64E-02	-0.023	0.011	3.53E-02
2.512606068	-0.025	0.013	4.89E-02	-0.020	0.019	2.92E-01
5.370937	0.023	0.010	1.86E-02	0.002	0.015	9.01E-01
3.525941	0.016	0.008	3.12E-02	0.027	0.011	1.97E-02
1.492206019	-0.009	0.004	2.30E-02	-0.011	0.006	8.35E-02
1.102617079	0.018	0.008	1.94E-02	0.000	0.011	9.94E-01
0.914214828	-0.011	0.005	3.32E-02	-0.031	0.007	2.84E-05
7.142928	-0.024	0.010	2.18E-02	-0.018	0.015	2.33E-01
7.574571	0.016	0.008	3.56E-02	0.020	0.011	7.89E-02
3.591882	-0.011	0.006	7.91E-02	0.008	0.009	3.90E-01
1.404733545	0.012	0.005	1.72E-02	0.009	0.007	1.95E-01
7.568179	0.019	0.009	3.18E-02	0.012	0.013	3.55E-01
3.380266	0.022	0.012	7.91E-02	0.037	0.019	4.72E-02
2.432198679	-0.017	0.009	6.12E-02	-0.022	0.014	1.06E-01
3.670271	-0.009	0.005	4.43E-02	0.002	0.007	7.92E-01
0.588211647	-0.016	0.008	5.21E-02	0.005	0.012	6.76E-01
3.469757	0.016	0.009	6.76E-02	0.036	0.013	6.41E-03
2.004592855	0.027	0.011	1.25E-02	0.005	0.016	7.81E-01
7.262698	-0.024	0.011	2.53E-02	-0.002	0.016	9.13E-01

7.660025	0.019	0.008	1.83E-02	-0.001	0.012	9.64E-01
6.68437	0.019	0.009	3.60E-02	0.005	0.013	6.82E-01
6.956881	-0.016	0.008	3.82E-02	0.005	0.011	6.48E-01
1.848488133	-0.017	0.008	4.44E-02	-0.013	0.012	2.88E-01
2.050347688	0.016	0.007	1.76E-02	0.007	0.010	4.95E-01
1.947062882	0.014	0.006	2.02E-02	0.007	0.009	4.42E-01
1.620723269	0.016	0.007	1.39E-02	0.005	0.010	6.13E-01
4.256	0.044	0.019	1.77E-02	0.017	0.028	5.48E-01
5.420057	0.013	0.007	5.81E-02	0.014	0.011	1.94E-01
0.692169317	0.036	0.017	2.88E-02	0.006	0.025	8.01E-01
3.485233	0.016	0.009	6.77E-02	0.034	0.013	1.09E-02
6.802794	0.015	0.008	5.80E-02	-0.015	0.012	2.05E-01
8.223213	0.014	0.008	9.65E-02	-0.016	0.012	1.92E-01
1.046432836	0.011	0.006	7.99E-02	-0.002	0.009	8.12E-01
5.171433	0.032	0.013	1.50E-02	-0.004	0.020	8.28E-01
6.53163	0.017	0.007	2.09E-02	-0.002	0.011	8.92E-01
3.953883	-0.022	0.011	3.68E-02	-0.017	0.016	2.83E-01
1.02490115	0.012	0.005	2.74E-02	0.011	0.008	1.84E-01
3.669934	-0.009	0.004	4.64E-02	0.002	0.007	7.24E-01
2.108550526	-0.012	0.007	6.98E-02	-0.022	0.010	2.87E-02
0.964679716	0.011	0.005	2.21E-02	0.010	0.007	1.70E-01
3.707278	0.015	0.007	3.18E-02	0.026	0.010	1.24E-02
2.976547	-0.009	0.004	3.56E-02	0.001	0.006	8.90E-01
5.216851	0.010	0.005	5.55E-02	0.006	0.008	4.18E-01
1.319952532	0.030	0.012	1.21E-02	0.009	0.018	6.28E-01
7.799308	-0.017	0.009	4.47E-02	0.003	0.013	8.31E-01
7.377421	-0.017	0.009	6.74E-02	-0.008	0.014	5.37E-01
2.877635	-0.015	0.008	6.60E-02	0.010	0.012	4.18E-01
4.052122	0.042	0.018	1.66E-02	0.010	0.026	6.95E-01
3.655131	-0.010	0.005	2.68E-02	0.005	0.007	4.43E-01
2.20510668	0.040	0.017	1.81E-02	-0.003	0.025	9.21E-01
7.219971	-0.019	0.008	1.06E-02	-0.019	0.011	9.16E-02
5.225262	0.010	0.006	8.64E-02	0.021	0.009	1.93E-02
2.20645241	0.043	0.018	1.73E-02	-0.002	0.027	9.30E-01
1.320288965	0.031	0.013	1.30E-02	0.009	0.019	6.30E-01
7.826895	-0.023	0.010	1.79E-02	-0.008	0.015	5.65E-01
1.939661365	0.013	0.006	1.91E-02	0.014	0.008	1.04E-01
8.929721	0.011	0.007	1.16E-01	-0.005	0.011	6.13E-01
2.878308	-0.015	0.008	5.88E-02	0.001	0.012	9.11E-01
2.205443112	0.041	0.017	1.87E-02	-0.003	0.026	8.97E-01
3.140389	-0.027	0.012	2.27E-02	-0.023	0.018	1.94E-01
1.555791779	0.048	0.020	1.92E-02	0.008	0.030	8.02E-01
8.934095	-0.012	0.008	1.14E-01	0.003	0.012	7.85E-01

1.797686812	-0.017	0.009	5.09E-02	0.004	0.013	7.76E-01
3.950519	-0.015	0.009	7.95E-02	0.013	0.013	3.10E-01
4.051785	0.042	0.018	1.62E-02	0.010	0.026	6.94E-01
2.495111573	-0.029	0.014	3.81E-02	-0.001	0.021	9.80E-01
8.133049	0.016	0.008	4.39E-02	-0.010	0.012	4.11E-01
8.805241	0.018	0.008	3.47E-02	-0.002	0.013	8.89E-01
1.8935701	-0.013	0.006	4.27E-02	-0.012	0.009	1.99E-01
1.096561292	0.016	0.007	3.58E-02	-0.007	0.011	5.50E-01
2.137483729	-0.021	0.010	2.60E-02	-0.002	0.014	8.86E-01
1.314569611	0.030	0.011	9.32E-03	0.013	0.017	4.36E-01
3.865402	-0.010	0.005	3.83E-02	0.005	0.007	4.69E-01
3.967341	-0.019	0.008	2.24E-02	-0.004	0.012	7.75E-01
1.955473697	0.012	0.005	2.21E-02	0.004	0.008	5.90E-01
8.46679	-0.014	0.007	5.68E-02	-0.014	0.011	1.92E-01
1.320625398	0.032	0.013	1.41E-02	0.010	0.019	5.97E-01
2.060104233	0.011	0.004	1.65E-02	0.010	0.007	1.13E-01
2.222937607	0.036	0.018	4.18E-02	0.022	0.026	3.86E-01
1.999209934	0.027	0.011	1.49E-02	0.003	0.017	8.47E-01
6.906752	0.018	0.009	3.05E-02	0.012	0.013	3.31E-01
0.54582114	-0.021	0.010	4.27E-02	-0.020	0.015	1.93E-01
3.557902	-0.021	0.009	2.78E-02	0.007	0.014	6.07E-01
3.76649	0.027	0.010	8.16E-03	0.038	0.015	1.19E-02
1.999546367	0.027	0.011	1.48E-02	0.003	0.017	8.42E-01
4.275513	0.036	0.015	1.87E-02	0.018	0.023	4.19E-01
3.608703	-0.014	0.007	4.84E-02	-0.007	0.010	4.82E-01
1.404397113	0.011	0.005	1.92E-02	0.010	0.007	1.42E-01
1.32096183	0.032	0.013	1.50E-02	0.012	0.020	5.47E-01
3.948837	-0.016	0.008	5.46E-02	0.002	0.012	8.97E-01
3.902409	0.014	0.007	5.04E-02	0.030	0.011	5.75E-03
5.979207	0.016	0.008	5.47E-02	0.007	0.012	5.75E-01
3.241319	0.012	0.007	9.38E-02	0.025	0.010	1.78E-02
6.646353	-0.013	0.007	4.00E-02	0.001	0.010	9.56E-01
8.447277	0.017	0.008	3.15E-02	0.020	0.012	8.54E-02
2.254898703	0.018	0.007	1.82E-02	0.007	0.011	5.05E-01
2.014685833	0.026	0.011	1.49E-02	0.006	0.016	7.06E-01
7.217616	-0.025	0.011	1.79E-02	-0.027	0.016	8.64E-02
8.277379	0.019	0.010	5.80E-02	-0.001	0.015	9.27E-01
7.732358	-0.022	0.011	4.41E-02	-0.012	0.016	4.59E-01
3.62317	-0.012	0.006	4.09E-02	-0.007	0.009	4.24E-01
1.952445804	0.013	0.006	2.15E-02	0.007	0.009	4.35E-01
6.388982	-0.014	0.007	6.08E-02	-0.011	0.011	3.24E-01
2.206115977	0.042	0.018	1.83E-02	-0.003	0.026	9.05E-01
5.106838	0.017	0.009	6.83E-02	0.008	0.014	5.81E-01

7.215934	-0.022	0.011	4.27E-02	-0.014	0.016	4.00E-01
3.147791	-0.039	0.016	1.78E-02	-0.046	0.025	6.19E-02
8.241717	-0.017	0.009	6.16E-02	0.010	0.014	4.75E-01
1.900635185	-0.015	0.006	1.90E-02	-0.005	0.010	5.77E-01
0.698897969	0.028	0.012	1.98E-02	-0.002	0.018	9.19E-01
4.253308	0.043	0.018	1.65E-02	0.010	0.026	6.94E-01
3.533343	0.017	0.007	1.86E-02	0.030	0.011	5.04E-03
1.264441155	0.039	0.017	1.99E-02	0.007	0.025	7.87E-01
4.148341	-0.033	0.016	4.10E-02	0.013	0.024	5.91E-01
8.648127	0.019	0.010	5.21E-02	0.006	0.015	7.05E-01
6.785636	-0.019	0.010	5.37E-02	-0.017	0.015	2.48E-01
7.822522	-0.023	0.009	1.47E-02	-0.018	0.014	1.97E-01
8.749057	0.013	0.006	3.35E-02	-0.002	0.009	7.98E-01
1.3196161	0.029	0.012	1.21E-02	0.009	0.017	6.02E-01
2.13109151	-0.025	0.012	2.84E-02	-0.025	0.017	1.41E-01
6.906416	0.018	0.008	3.06E-02	0.016	0.013	1.89E-01
1.614667482	0.016	0.007	1.63E-02	0.002	0.010	8.73E-01
3.728473	0.014	0.008	8.51E-02	0.028	0.012	2.14E-02
5.217861	0.009	0.005	9.10E-02	0.007	0.008	3.86E-01
5.721837	0.021	0.009	1.36E-02	0.005	0.013	7.19E-01
5.170424	0.029	0.012	1.36E-02	-0.005	0.018	7.67E-01
3.910483	0.015	0.008	6.91E-02	0.036	0.012	3.62E-03
1.018845363	0.020	0.009	2.06E-02	0.014	0.013	2.77E-01
4.275849	0.035	0.015	2.01E-02	0.017	0.023	4.50E-01
1.215321997	0.014	0.007	4.29E-02	0.009	0.010	3.66E-01
1.071328848	0.023	0.011	3.39E-02	0.006	0.016	6.99E-01
3.9485	-0.015	0.008	5.23E-02	0.000	0.012	9.76E-01
6.597571	0.016	0.009	6.21E-02	-0.008	0.013	5.39E-01
1.555455346	0.047	0.020	2.04E-02	0.007	0.030	8.07E-01
3.470093	0.016	0.009	7.07E-02	0.037	0.013	5.74E-03
1.554109616	0.046	0.020	2.04E-02	0.005	0.030	8.76E-01
3.847571	0.013	0.007	5.85E-02	0.025	0.010	1.47E-02
5.804263	0.015	0.009	7.42E-02	-0.008	0.013	5.25E-01
0.55355909	0.019	0.009	2.82E-02	0.020	0.013	1.33E-01
1.555118914	0.047	0.020	2.03E-02	0.008	0.030	7.97E-01
1.553773183	0.046	0.020	2.06E-02	0.004	0.029	8.91E-01
3.013554	-0.010	0.005	3.60E-02	-0.006	0.007	3.95E-01
7.289612	-0.018	0.010	7.17E-02	-0.021	0.015	1.75E-01
7.523097	0.015	0.007	4.73E-02	-0.004	0.011	6.97E-01
1.554446048	0.046	0.020	2.02E-02	0.006	0.030	8.38E-01
4.051112	0.041	0.017	1.71E-02	0.008	0.025	7.50E-01
7.510312	-0.017	0.008	5.05E-02	0.001	0.013	9.15E-01
0.934737216	-0.010	0.005	4.76E-02	-0.019	0.007	7.31E-03

7.78215	-0.021	0.010	3.35E-02	0.000	0.015	9.90E-01
2.204770247	0.040	0.017	1.88E-02	-0.002	0.025	9.45E-01
1.176295816	0.017	0.007	1.31E-02	-0.003	0.010	7.74E-01
8.61953	0.014	0.007	4.93E-02	0.001	0.011	9.42E-01
2.111914852	-0.015	0.007	4.44E-02	-0.014	0.011	1.88E-01
1.612985319	0.017	0.007	1.83E-02	0.005	0.011	6.58E-01
1.554782481	0.047	0.020	2.02E-02	0.007	0.030	8.17E-01
3.147118	-0.046	0.020	2.42E-02	-0.075	0.030	1.43E-02
7.798299	-0.022	0.010	2.28E-02	-0.007	0.015	6.40E-01
7.04469	-0.031	0.013	2.00E-02	-0.023	0.020	2.59E-01
2.13378297	-0.017	0.008	4.38E-02	0.011	0.012	3.63E-01
1.94672645	0.013	0.006	2.24E-02	0.005	0.009	5.38E-01
1.860263274	-0.019	0.008	2.22E-02	0.005	0.012	6.58E-01
3.738566	0.015	0.008	8.17E-02	0.041	0.013	9.54E-04
0.630265721	0.014	0.010	1.49E-01	-0.017	0.014	2.48E-01
3.950855	-0.014	0.009	9.25E-02	0.012	0.013	3.36E-01
3.558239	-0.018	0.009	3.63E-02	0.015	0.013	2.49E-01
6.918864	-0.020	0.009	2.89E-02	0.018	0.013	1.70E-01
2.878981	-0.015	0.008	5.67E-02	-0.004	0.011	7.47E-01
4.054477	0.042	0.018	2.13E-02	0.008	0.027	7.68E-01
1.10026205	0.016	0.008	4.90E-02	-0.002	0.012	8.58E-01
5.347051	0.046	0.020	2.18E-02	0.013	0.030	6.72E-01
0.870478591	0.036	0.015	1.99E-02	0.017	0.023	4.63E-01
6.523219	0.015	0.009	1.14E-01	0.007	0.014	6.26E-01
3.140726	-0.027	0.012	2.56E-02	-0.021	0.018	2.31E-01
1.782547345	-0.022	0.011	4.87E-02	0.015	0.017	3.60E-01
0.662563249	-0.018	0.009	4.19E-02	-0.034	0.013	1.04E-02
3.82503	0.013	0.007	5.84E-02	0.033	0.010	1.54E-03
7.288603	-0.020	0.010	5.03E-02	-0.012	0.015	4.28E-01
3.0583	-0.011	0.006	5.13E-02	-0.010	0.008	2.27E-01
0.738933448	-0.015	0.007	2.64E-02	-0.010	0.010	3.02E-01
2.053039149	0.014	0.006	2.01E-02	0.008	0.009	3.66E-01
2.378369464	-0.016	0.008	4.47E-02	0.019	0.012	1.30E-01
6.961591	-0.020	0.009	1.92E-02	-0.012	0.013	3.34E-01
3.779611	0.019	0.008	2.24E-02	0.029	0.012	1.93E-02
7.804691	-0.022	0.009	2.04E-02	-0.004	0.014	7.50E-01
3.495662	0.017	0.010	7.19E-02	0.032	0.014	2.69E-02
3.105064	-0.018	0.008	2.88E-02	-0.010	0.012	3.97E-01
7.197093	0.019	0.008	2.06E-02	0.000	0.012	9.86E-01
1.323653291	0.033	0.014	1.47E-02	0.018	0.020	3.67E-01
1.105644972	0.016	0.007	2.38E-02	0.001	0.011	9.53E-01
1.01581747	0.025	0.011	1.96E-02	0.021	0.016	1.80E-01
3.261505	0.016	0.008	4.55E-02	0.026	0.012	2.92E-02

7.187673	-0.020	0.011	5.87E-02	-0.023	0.016	1.44E-01
1.164520675	0.011	0.004	1.40E-02	0.002	0.007	8.06E-01
1.07166528	0.022	0.011	4.07E-02	0.006	0.016	6.99E-01
2.060440666	0.010	0.004	1.86E-02	0.010	0.006	1.28E-01
7.050409	-0.017	0.009	3.98E-02	-0.022	0.013	8.40E-02
3.847234	0.012	0.007	6.37E-02	0.022	0.010	2.36E-02
5.814019	0.014	0.007	5.37E-02	0.007	0.011	4.92E-01
7.425531	-0.024	0.010	2.16E-02	-0.018	0.016	2.63E-01
6.345919	0.019	0.009	4.10E-02	0.018	0.014	2.08E-01
2.199050893	0.031	0.013	2.21E-02	-0.008	0.020	6.78E-01
7.049063	-0.015	0.008	5.22E-02	-0.021	0.012	8.30E-02
2.378033032	-0.017	0.008	4.62E-02	0.011	0.013	3.66E-01
2.050011255	0.016	0.007	2.01E-02	0.007	0.010	4.78E-01
1.110691461	0.013	0.006	3.32E-02	0.000	0.009	9.72E-01
1.403724248	0.011	0.005	1.61E-02	0.013	0.007	5.86E-02
2.640786885	-0.034	0.013	7.40E-03	0.005	0.019	8.00E-01
2.348426964	0.026	0.009	6.60E-03	0.014	0.014	3.31E-01
5.160667	0.023	0.010	2.79E-02	-0.025	0.016	1.06E-01
3.393723	0.016	0.009	7.34E-02	0.040	0.013	2.06E-03
1.319279667	0.028	0.011	1.20E-02	0.009	0.017	5.83E-01
1.40271495	0.011	0.005	1.43E-02	0.007	0.007	2.84E-01
2.683850257	-0.013	0.005	1.13E-02	0.000	0.008	9.61E-01
5.399871	0.016	0.007	3.06E-02	-0.001	0.011	9.17E-01
7.673146	0.018	0.008	3.24E-02	0.017	0.012	1.58E-01
1.950763641	0.013	0.006	2.19E-02	0.004	0.009	6.17E-01
2.887392	-0.014	0.007	4.29E-02	-0.002	0.010	8.46E-01
3.951192	-0.014	0.008	9.57E-02	0.009	0.013	4.54E-01
1.641918522	0.012	0.006	3.98E-02	0.009	0.008	2.75E-01
0.635648642	0.017	0.010	7.10E-02	0.015	0.014	3.02E-01
3.950183	-0.015	0.009	8.47E-02	0.008	0.013	5.55E-01
2.221928309	0.040	0.020	4.67E-02	0.020	0.029	4.99E-01
7.219298	-0.023	0.010	2.27E-02	0.009	0.015	5.59E-01
2.205779545	0.041	0.017	1.97E-02	-0.004	0.026	8.70E-01
3.815946	0.013	0.007	6.57E-02	0.033	0.011	2.39E-03
1.192108148	0.009	0.004	3.36E-02	0.003	0.007	6.55E-01
3.731838	0.015	0.009	7.54E-02	0.027	0.013	3.78E-02
6.337172	0.021	0.009	1.88E-02	-0.004	0.013	7.88E-01
2.102158307	-0.011	0.006	6.42E-02	0.012	0.009	1.65E-01
6.724742	-0.028	0.012	1.85E-02	-0.042	0.017	1.73E-02
1.610630291	0.019	0.008	1.83E-02	-0.003	0.012	7.82E-01
3.387331	0.017	0.009	6.79E-02	0.035	0.014	1.45E-02
1.178987277	0.016	0.007	1.78E-02	-0.003	0.010	7.61E-01
2.132773673	-0.023	0.010	1.70E-02	-0.009	0.014	5.09E-01

1.115065084	0.016	0.007	2.58E-02	-0.004	0.011	7.43E-01
7.615616	-0.028	0.013	2.71E-02	0.001	0.019	9.38E-01
4.055149	0.042	0.018	2.14E-02	0.010	0.027	7.15E-01
7.17287	-0.018	0.011	8.16E-02	-0.007	0.016	6.71E-01
6.828027	-0.014	0.008	6.40E-02	-0.012	0.011	3.02E-01
1.998200636	0.026	0.011	1.76E-02	0.002	0.016	9.05E-01
0.767530218	0.011	0.005	3.98E-02	-0.002	0.008	8.37E-01
6.772516	-0.017	0.010	8.21E-02	0.010	0.015	5.01E-01
7.71419	-0.023	0.010	2.70E-02	-0.016	0.016	2.88E-01
1.998873501	0.027	0.011	1.68E-02	0.002	0.017	8.86E-01
5.863811	0.015	0.008	6.96E-02	-0.006	0.012	6.06E-01
3.842188	0.013	0.007	5.02E-02	0.024	0.010	1.95E-02
3.779948	0.018	0.008	2.48E-02	0.025	0.012	3.28E-02
2.380724492	-0.015	0.007	2.08E-02	-0.006	0.010	5.54E-01
5.104483	0.019	0.009	2.83E-02	0.008	0.013	5.67E-01
2.015022266	0.025	0.011	1.68E-02	0.005	0.016	7.47E-01
2.203088084	0.036	0.016	2.15E-02	-0.009	0.024	7.18E-01
2.13243724	-0.024	0.010	1.63E-02	-0.017	0.015	2.51E-01
8.790438	0.015	0.007	2.41E-02	-0.005	0.010	6.52E-01
5.216178	0.011	0.005	4.65E-02	-0.003	0.008	7.11E-01
2.683513824	-0.013	0.005	1.08E-02	0.001	0.008	9.29E-01
6.679997	0.017	0.009	5.85E-02	0.007	0.014	5.96E-01
1.185043063	0.010	0.005	2.31E-02	0.005	0.007	4.63E-01
3.442169	0.014	0.007	6.27E-02	0.025	0.011	2.68E-02
2.349099829	0.025	0.009	6.56E-03	0.017	0.014	2.21E-01
2.128063616	-0.017	0.008	3.67E-02	-0.001	0.012	9.03E-01
1.951100073	0.013	0.006	2.50E-02	0.006	0.009	5.12E-01
3.70055	0.014	0.007	5.39E-02	0.026	0.011	1.87E-02
5.899809	0.016	0.008	3.51E-02	0.005	0.012	6.88E-01
8.999363	0.011	0.007	1.14E-01	0.014	0.010	1.66E-01
8.17443	-0.016	0.009	9.32E-02	-0.004	0.014	7.92E-01
4.055822	0.042	0.018	2.09E-02	0.004	0.027	8.77E-01
6.498996	0.015	0.008	4.75E-02	0.003	0.012	7.82E-01
5.548574	0.017	0.009	5.11E-02	-0.015	0.013	2.33E-01
1.877757769	-0.011	0.006	6.70E-02	-0.007	0.009	4.24E-01
3.062673	-0.012	0.007	6.50E-02	0.004	0.010	6.94E-01
3.824693	0.013	0.007	6.43E-02	0.033	0.010	1.92E-03
0.506458527	-0.016	0.010	8.59E-02	0.001	0.014	9.59E-01
2.921372	-0.014	0.007	2.86E-02	-0.009	0.010	3.56E-01
5.172779	0.032	0.013	1.92E-02	0.001	0.020	9.60E-01
3.622497	-0.012	0.006	3.73E-02	-0.004	0.008	6.27E-01
2.885373	-0.015	0.007	3.51E-02	-0.005	0.011	6.26E-01
0.691832885	0.035	0.017	3.57E-02	0.003	0.025	8.93E-01

3.58919	-0.011	0.006	7.22E-02	0.003	0.009	6.96E-01
3.06772	-0.012	0.006	4.96E-02	-0.001	0.009	9.45E-01
1.656721556	0.009	0.004	3.22E-02	0.009	0.006	1.54E-01
3.118185	-0.019	0.009	2.84E-02	-0.017	0.013	1.92E-01
3.291784	-0.028	0.013	3.41E-02	-0.018	0.020	3.75E-01
1.331727673	0.031	0.013	1.35E-02	0.016	0.019	4.03E-01
6.123874	0.015	0.009	1.13E-01	-0.015	0.014	2.92E-01
1.215658429	0.013	0.007	4.74E-02	0.009	0.010	3.82E-01
2.131427942	-0.025	0.011	2.39E-02	-0.026	0.017	1.17E-01
1.998537069	0.026	0.011	1.77E-02	0.002	0.016	8.87E-01
6.430027	0.016	0.008	5.39E-02	-0.008	0.012	5.29E-01
1.410452899	0.013	0.005	1.50E-02	0.017	0.008	3.19E-02
6.056587	0.014	0.008	6.56E-02	-0.006	0.011	5.64E-01
2.398891852	-0.022	0.010	3.22E-02	-0.011	0.015	4.76E-01
1.553436751	0.045	0.020	2.23E-02	0.002	0.029	9.37E-01
4.05414	0.042	0.018	2.26E-02	0.007	0.027	7.97E-01
3.971041	-0.018	0.008	2.18E-02	-0.006	0.012	6.08E-01
2.057749205	0.012	0.005	2.01E-02	0.011	0.008	1.45E-01
5.398525	0.015	0.007	3.44E-02	0.004	0.010	7.32E-01
5.87054	0.017	0.008	3.94E-02	-0.008	0.012	5.10E-01
5.36219	0.030	0.013	2.49E-02	0.009	0.020	6.41E-01
1.126840225	0.016	0.007	2.20E-02	-0.004	0.010	7.32E-01
1.617358943	0.014	0.006	3.25E-02	0.005	0.010	6.31E-01
3.567995	-0.012	0.007	6.14E-02	-0.001	0.010	9.35E-01
0.71067311	0.018	0.008	3.32E-02	0.017	0.013	1.69E-01
1.892897235	-0.011	0.006	6.30E-02	-0.014	0.009	9.88E-02
2.489728652	-0.034	0.014	1.99E-02	-0.019	0.022	3.83E-01
6.351975	0.013	0.008	9.01E-02	-0.012	0.011	2.94E-01
4.262056	0.033	0.014	2.29E-02	0.016	0.021	4.57E-01
1.410116467	0.014	0.005	1.23E-02	0.013	0.008	1.04E-01
2.204433815	0.038	0.017	2.15E-02	-0.003	0.025	9.01E-01
1.072674578	0.013	0.007	5.26E-02	-0.001	0.010	9.35E-01
3.056618	-0.010	0.006	9.63E-02	-0.009	0.009	2.81E-01
4.255663	0.043	0.019	2.10E-02	0.017	0.028	5.40E-01
0.916906288	-0.010	0.005	4.39E-02	-0.027	0.007	1.57E-04
7.043344	-0.025	0.013	5.23E-02	-0.001	0.019	9.71E-01
2.301662833	-0.009	0.004	4.54E-02	-0.004	0.006	5.82E-01
2.687214583	-0.014	0.005	1.07E-02	-0.003	0.008	7.01E-01
0.898738928	0.022	0.009	1.66E-02	-0.005	0.014	7.09E-01
2.692261072	-0.016	0.006	8.85E-03	-0.007	0.009	4.44E-01
2.709419134	-0.016	0.006	9.44E-03	-0.020	0.009	3.08E-02
3.603321	-0.014	0.007	4.50E-02	-0.007	0.011	4.99E-01
8.862435	0.017	0.009	6.68E-02	-0.001	0.014	9.58E-01

4.250617	0.038	0.016	1.86E-02	0.009	0.024	7.05E-01
4.252635	0.041	0.018	2.02E-02	0.006	0.026	8.25E-01
2.691588207	-0.015	0.006	9.69E-03	-0.005	0.009	5.47E-01
7.805027	-0.019	0.008	2.20E-02	-0.008	0.013	5.34E-01
2.060777098	0.010	0.004	2.19E-02	0.009	0.006	1.54E-01
1.553100318	0.044	0.019	2.28E-02	0.002	0.029	9.55E-01
0.662226817	-0.020	0.010	3.52E-02	-0.026	0.014	6.77E-02
4.253645	0.042	0.018	1.94E-02	0.009	0.027	7.38E-01
1.318943235	0.027	0.011	1.24E-02	0.009	0.016	5.63E-01
4.051449	0.041	0.017	1.93E-02	0.009	0.026	7.39E-01
4.054813	0.042	0.018	2.29E-02	0.010	0.027	7.17E-01
3.559921	-0.018	0.009	4.01E-02	0.015	0.013	2.45E-01
5.67339	0.018	0.009	4.42E-02	0.000	0.013	9.82E-01
4.274504	0.035	0.015	2.09E-02	0.015	0.022	5.14E-01
7.461529	0.018	0.009	5.12E-02	-0.007	0.014	5.95E-01
1.834694397	-0.021	0.010	3.53E-02	-0.028	0.015	5.31E-02
2.203424517	0.036	0.016	2.42E-02	-0.007	0.024	7.61E-01
1.631152679	0.013	0.006	3.82E-02	0.012	0.009	2.11E-01
6.792365	-0.011	0.007	1.21E-01	-0.016	0.011	1.50E-01
7.335704	-0.015	0.006	1.50E-02	-0.006	0.009	5.35E-01
8.276033	0.020	0.010	4.25E-02	0.019	0.015	2.01E-01
7.035269	-0.025	0.012	3.01E-02	-0.013	0.017	4.37E-01
1.629134084	0.013	0.006	2.84E-02	0.000	0.009	9.62E-01
2.87629	-0.013	0.008	1.01E-01	-0.011	0.012	3.46E-01
6.364423	-0.020	0.010	3.35E-02	-0.008	0.014	5.51E-01
2.103167605	-0.010	0.006	7.81E-02	0.011	0.008	1.96E-01
5.168069	0.022	0.010	2.55E-02	-0.010	0.015	5.08E-01
6.853259	-0.023	0.010	2.70E-02	-0.007	0.015	6.49E-01
4.249944	0.039	0.017	1.95E-02	0.013	0.025	6.13E-01
7.75759	-0.023	0.011	3.63E-02	-0.011	0.017	5.11E-01
1.997864204	0.025	0.011	1.91E-02	0.002	0.016	9.02E-01
3.855645	0.012	0.007	8.29E-02	0.026	0.010	1.44E-02
2.202751652	0.035	0.016	2.31E-02	-0.007	0.023	7.55E-01
1.147026181	0.014	0.006	1.86E-02	-0.001	0.009	9.07E-01
7.677183	0.015	0.009	8.12E-02	-0.002	0.013	8.81E-01
7.376076	-0.016	0.008	5.29E-02	-0.017	0.012	1.67E-01
7.823867	-0.024	0.009	1.07E-02	-0.010	0.014	4.93E-01
7.500556	0.019	0.008	2.10E-02	0.017	0.012	1.69E-01
2.015358698	0.025	0.010	1.83E-02	0.004	0.016	7.74E-01
2.254562271	0.017	0.008	2.62E-02	0.007	0.011	5.26E-01
1.264104722	0.037	0.016	2.35E-02	0.005	0.024	8.26E-01
0.76652092	0.011	0.005	3.58E-02	0.002	0.008	8.30E-01
7.28793	-0.018	0.010	5.70E-02	-0.017	0.014	2.22E-01

2.221591877	0.040	0.020	5.02E-02	0.018	0.030	5.38E-01
4.256336	0.043	0.019	2.13E-02	0.017	0.028	5.41E-01
1.100934916	0.015	0.008	5.55E-02	-0.005	0.012	6.54E-01
2.981257	-0.008	0.004	5.48E-02	0.004	0.006	5.15E-01
2.063132126	0.009	0.004	1.56E-02	0.010	0.006	8.45E-02
7.745815	-0.022	0.011	3.67E-02	-0.037	0.016	1.77E-02
8.909872	0.019	0.010	4.70E-02	0.001	0.015	9.34E-01
2.059094935	0.011	0.005	2.09E-02	0.011	0.007	1.12E-01
5.804599	0.013	0.008	9.21E-02	-0.002	0.012	8.63E-01
4.248262	0.040	0.018	2.52E-02	0.016	0.027	5.46E-01
1.610966724	0.018	0.008	2.02E-02	-0.003	0.012	7.83E-01
2.204097382	0.037	0.016	2.22E-02	-0.006	0.024	8.19E-01
6.784964	-0.021	0.010	4.41E-02	-0.014	0.015	3.55E-01
3.028021	-0.010	0.005	5.75E-02	-0.005	0.008	4.84E-01
1.058880842	0.011	0.006	6.80E-02	-0.006	0.009	5.09E-01
4.255327	0.043	0.019	2.24E-02	0.021	0.028	4.47E-01
3.147454	-0.042	0.018	2.30E-02	-0.060	0.028	3.14E-02
8.387728	0.014	0.007	5.92E-02	0.001	0.011	8.97E-01
1.954464399	0.012	0.005	2.65E-02	0.006	0.008	4.54E-01
2.494438708	-0.031	0.016	5.57E-02	-0.007	0.024	7.77E-01
5.411309	0.017	0.008	4.14E-02	-0.011	0.012	3.56E-01
8.819371	0.013	0.008	9.13E-02	0.015	0.012	2.07E-01
1.114055787	0.016	0.007	3.15E-02	-0.003	0.011	7.84E-01
1.411125765	0.013	0.005	1.35E-02	0.016	0.008	3.46E-02
7.075641	0.020	0.010	4.10E-02	0.026	0.015	6.88E-02
2.860814	-0.016	0.008	4.60E-02	-0.016	0.012	1.70E-01
3.390695	0.015	0.009	9.43E-02	0.043	0.014	1.98E-03
6.455596	0.015	0.009	9.53E-02	0.013	0.013	3.18E-01
1.629806949	0.013	0.006	3.61E-02	0.007	0.009	4.35E-01
7.910667	-0.023	0.010	2.68E-02	-0.010	0.015	5.22E-01
5.217524	0.009	0.005	8.59E-02	0.006	0.008	4.17E-01
6.528265	0.016	0.009	8.59E-02	0.011	0.014	4.27E-01
8.127666	0.012	0.009	1.64E-01	0.010	0.013	4.59E-01
5.625617	0.016	0.010	1.04E-01	-0.002	0.014	9.14E-01
2.915989	-0.016	0.008	3.49E-02	-0.004	0.011	7.46E-01
1.631825544	0.013	0.006	3.72E-02	0.003	0.009	7.76E-01
1.059553707	0.012	0.007	7.71E-02	-0.006	0.010	5.94E-01
3.480859	0.017	0.010	7.51E-02	0.036	0.014	1.19E-02
6.228168	0.016	0.008	3.92E-02	0.002	0.011	8.30E-01
3.865738	-0.009	0.005	4.81E-02	0.006	0.007	4.27E-01
5.639411	0.014	0.007	5.99E-02	0.000	0.011	9.85E-01
1.552763885	0.043	0.019	2.44E-02	0.003	0.029	9.23E-01
1.893906533	-0.013	0.006	4.44E-02	-0.011	0.010	2.50E-01

2.221255444	0.041	0.021	5.08E-02	0.018	0.030	5.50E-01
2.389808172	-0.018	0.009	4.29E-02	-0.005	0.013	6.92E-01
3.871794	-0.009	0.005	5.27E-02	0.007	0.007	3.23E-01
6.793038	-0.012	0.009	1.71E-01	-0.011	0.013	3.71E-01
1.899289454	-0.014	0.006	2.83E-02	-0.011	0.010	2.48E-01
2.570136041	-0.020	0.010	5.65E-02	0.006	0.015	7.06E-01
6.342555	-0.018	0.009	5.79E-02	-0.004	0.014	7.59E-01
1.070992415	0.019	0.009	4.46E-02	0.001	0.014	9.30E-01
3.528296	0.016	0.008	5.24E-02	0.035	0.012	3.37E-03
7.380786	-0.017	0.009	5.69E-02	-0.024	0.014	7.97E-02
5.660606	0.014	0.008	5.81E-02	-0.016	0.011	1.50E-01
1.941679961	0.013	0.006	2.50E-02	0.013	0.009	1.53E-01
6.774198	-0.020	0.009	3.18E-02	0.005	0.014	7.40E-01
7.796953	-0.020	0.010	3.87E-02	0.001	0.014	9.67E-01
6.964282	-0.024	0.011	2.87E-02	-0.018	0.016	2.67E-01
8.950917	-0.019	0.009	3.16E-02	-0.024	0.013	7.46E-02
3.402134	0.017	0.009	7.38E-02	0.040	0.014	4.06E-03
5.384731	0.022	0.011	3.72E-02	0.009	0.016	5.58E-01
8.585551	0.022	0.010	4.00E-02	0.015	0.016	3.45E-01
3.538389	0.017	0.008	3.72E-02	0.026	0.012	3.36E-02
3.09598	-0.018	0.008	2.72E-02	-0.021	0.012	8.18E-02
7.212233	-0.018	0.009	3.09E-02	-0.009	0.013	4.95E-01
3.393387	0.015	0.009	8.34E-02	0.043	0.013	9.29E-04
5.388768	0.016	0.008	3.41E-02	0.000	0.011	9.86E-01
8.86008	0.013	0.008	1.11E-01	0.009	0.012	4.31E-01
2.403265476	-0.022	0.011	4.66E-02	0.017	0.016	3.02E-01
1.943025691	0.013	0.006	2.60E-02	0.014	0.009	1.14E-01
4.25028	0.038	0.016	2.08E-02	0.009	0.024	7.13E-01
6.439447	0.018	0.009	4.46E-02	0.009	0.013	4.76E-01
3.808544	0.012	0.006	6.43E-02	0.020	0.010	4.09E-02
6.221103	0.014	0.007	4.11E-02	-0.021	0.010	3.61E-02
3.487251	0.016	0.009	8.20E-02	0.039	0.014	3.93E-03
3.030376	-0.009	0.005	6.01E-02	-0.001	0.007	9.17E-01
4.249271	0.039	0.017	2.40E-02	0.014	0.026	5.80E-01
8.180486	-0.023	0.010	2.55E-02	-0.016	0.015	2.99E-01
7.042334	-0.020	0.011	7.98E-02	-0.019	0.017	2.76E-01
7.050745	-0.017	0.008	4.63E-02	-0.019	0.012	1.26E-01
4.050776	0.038	0.017	2.18E-02	0.005	0.025	8.33E-01
7.167151	0.017	0.009	5.13E-02	0.007	0.013	6.03E-01
6.637943	0.015	0.009	8.04E-02	0.005	0.013	7.19E-01
1.622741864	0.016	0.007	1.76E-02	0.004	0.010	7.13E-01
7.947002	0.018	0.009	5.92E-02	0.011	0.014	4.35E-01
6.722387	-0.021	0.012	7.48E-02	-0.016	0.017	3.46E-01

4.1692	-0.026	0.014	5.05E-02	-0.004	0.020	8.61E-01
4.262392	0.032	0.014	2.50E-02	0.018	0.021	3.94E-01
1.939997798	0.013	0.006	2.33E-02	0.014	0.008	9.24E-02
0.870142158	0.034	0.015	2.39E-02	0.016	0.023	4.90E-01
3.250739	-0.014	0.006	1.81E-02	0.001	0.009	8.92E-01
8.439203	0.017	0.010	8.14E-02	0.009	0.014	5.23E-01
5.224926	0.009	0.006	1.09E-01	0.017	0.009	4.47E-02
5.088334	0.016	0.008	5.89E-02	-0.008	0.013	5.04E-01
3.953547	-0.020	0.010	4.08E-02	-0.015	0.015	3.07E-01
1.126503793	0.015	0.007	3.19E-02	-0.005	0.010	6.36E-01
6.565273	0.018	0.010	7.31E-02	-0.005	0.015	7.56E-01
4.168864	-0.026	0.013	5.45E-02	0.010	0.020	6.30E-01
1.933269146	0.010	0.005	4.42E-02	0.007	0.007	3.33E-01
5.545209	0.014	0.007	3.73E-02	0.017	0.010	1.07E-01
1.954127967	0.012	0.005	3.01E-02	0.006	0.008	4.28E-01
2.98193	-0.008	0.004	6.02E-02	0.002	0.006	6.88E-01
1.899625887	-0.015	0.006	2.50E-02	-0.009	0.010	3.34E-01
7.638829	-0.019	0.011	9.10E-02	0.016	0.017	3.41E-01
4.254318	0.042	0.018	2.18E-02	0.013	0.027	6.30E-01
4.248598	0.040	0.018	2.44E-02	0.017	0.026	5.19E-01
1.097234157	0.015	0.008	4.48E-02	0.004	0.011	7.15E-01
6.906079	0.017	0.008	3.37E-02	0.010	0.012	3.92E-01
4.148678	-0.031	0.016	4.60E-02	0.019	0.023	4.13E-01
2.873935	-0.016	0.009	7.41E-02	-0.006	0.013	6.36E-01
2.561052361	-0.022	0.010	2.90E-02	-0.002	0.015	8.74E-01
1.951436506	0.012	0.006	3.09E-02	0.005	0.009	5.64E-01
5.169078	0.024	0.011	2.39E-02	-0.003	0.016	8.69E-01
2.491410815	-0.036	0.016	1.99E-02	-0.015	0.023	5.32E-01
8.750739	-0.016	0.009	8.11E-02	0.003	0.013	8.10E-01
1.633171275	0.014	0.006	2.79E-02	0.009	0.010	3.37E-01
4.252972	0.041	0.018	2.16E-02	0.008	0.026	7.57E-01
5.761199	0.018	0.010	5.82E-02	0.009	0.014	5.44E-01
2.015695131	0.024	0.010	1.96E-02	0.005	0.015	7.43E-01
5.371274	0.020	0.009	2.56E-02	0.009	0.014	4.92E-01
3.246702	0.016	0.008	4.39E-02	0.036	0.012	3.21E-03
1.642591387	0.011	0.006	4.19E-02	0.022	0.008	6.74E-03
4.085765	0.030	0.012	1.43E-02	0.022	0.018	2.38E-01
2.301326401	-0.008	0.004	6.25E-02	0.001	0.006	8.89E-01
3.489606	0.017	0.010	7.57E-02	0.039	0.015	8.20E-03
2.109223391	-0.011	0.007	1.10E-01	-0.027	0.010	8.44E-03
5.900146	0.015	0.007	3.47E-02	0.004	0.011	7.38E-01
1.015144605	0.025	0.011	2.37E-02	0.016	0.017	3.28E-01
5.591974	0.018	0.009	5.93E-02	-0.008	0.014	5.53E-01

6.712967	-0.015	0.009	8.47E-02	-0.001	0.013	9.17E-01
6.648708	-0.018	0.009	4.51E-02	0.000	0.013	9.87E-01
1.129195253	0.017	0.007	2.07E-02	0.000	0.011	9.89E-01
1.618704673	0.014	0.006	2.37E-02	0.006	0.010	5.60E-01
1.876748471	-0.011	0.006	8.59E-02	-0.010	0.009	2.61E-01
3.594237	-0.011	0.006	7.07E-02	0.005	0.009	6.10E-01
3.817292	0.012	0.006	7.52E-02	0.032	0.010	8.55E-04
2.877972	-0.014	0.008	7.44E-02	0.007	0.012	5.70E-01
3.864392	-0.010	0.005	3.39E-02	0.004	0.007	5.98E-01
3.028357	-0.009	0.005	5.93E-02	-0.006	0.007	4.03E-01
1.997527771	0.025	0.011	2.08E-02	0.002	0.016	9.10E-01
2.158006117	-0.021	0.010	3.70E-02	0.000	0.015	9.98E-01
2.992695	-0.008	0.004	3.76E-02	0.002	0.006	6.80E-01
0.699234402	0.025	0.012	3.31E-02	-0.004	0.017	8.07E-01
3.486242	0.015	0.009	8.16E-02	0.037	0.013	5.50E-03
3.981134	-0.017	0.010	1.02E-01	-0.017	0.015	2.57E-01
0.515542207	-0.014	0.007	3.05E-02	0.006	0.010	5.22E-01
3.770191	0.014	0.008	8.90E-02	0.020	0.013	1.08E-01
2.641123318	-0.034	0.014	1.22E-02	0.006	0.020	7.73E-01
0.989575728	0.013	0.007	5.20E-02	0.007	0.010	4.52E-01
3.652776	-0.009	0.005	4.54E-02	0.004	0.007	5.56E-01
1.943362124	0.013	0.006	2.44E-02	0.012	0.009	1.81E-01
7.211897	-0.022	0.010	2.56E-02	-0.012	0.015	4.27E-01
5.148219	0.019	0.009	4.08E-02	-0.003	0.013	8.22E-01
5.494745	0.017	0.010	7.79E-02	-0.002	0.015	9.10E-01
5.220552	0.008	0.005	1.21E-01	0.006	0.007	4.44E-01
7.040316	-0.018	0.010	7.39E-02	0.003	0.015	8.38E-01
2.968136	-0.009	0.004	3.24E-02	-0.001	0.007	8.99E-01
6.089894	0.016	0.009	5.92E-02	0.007	0.013	5.60E-01
7.359927	-0.019	0.010	4.43E-02	-0.009	0.014	5.43E-01
2.05842207	0.011	0.005	2.43E-02	0.011	0.007	1.32E-01
5.340322	0.042	0.019	3.16E-02	0.006	0.029	8.49E-01
4.249608	0.039	0.017	2.27E-02	0.012	0.025	6.44E-01
2.132100807	-0.024	0.010	1.91E-02	-0.022	0.015	1.60E-01
8.970093	-0.019	0.009	2.60E-02	-0.016	0.013	2.24E-01
1.878430634	-0.010	0.006	8.45E-02	-0.007	0.009	4.19E-01
6.73248	-0.021	0.010	3.97E-02	-0.017	0.015	2.73E-01
2.312428676	-0.013	0.007	4.51E-02	-0.002	0.010	8.73E-01
1.552427453	0.042	0.019	2.68E-02	0.001	0.029	9.74E-01
3.024656	-0.010	0.005	4.40E-02	-0.010	0.008	1.75E-01
2.874271	-0.016	0.009	8.29E-02	0.000	0.013	9.86E-01
6.88623	0.020	0.009	2.15E-02	-0.005	0.013	7.13E-01
6.258447	0.011	0.007	1.21E-01	0.003	0.010	8.07E-01

1.876412038	-0.011	0.006	7.48E-02	-0.009	0.009	3.42E-01
1.617695376	0.014	0.006	2.92E-02	0.008	0.010	3.79E-01
2.977556	-0.008	0.004	5.00E-02	0.001	0.006	8.47E-01
1.822246391	-0.016	0.009	8.46E-02	-0.005	0.014	7.01E-01
2.978902	-0.008	0.004	4.49E-02	0.007	0.006	2.42E-01
3.399779	0.014	0.008	9.83E-02	0.034	0.012	6.17E-03
1.201191828	0.009	0.005	6.01E-02	0.008	0.007	2.61E-01
8.387392	0.014	0.008	6.07E-02	0.015	0.011	1.75E-01
3.139716	-0.025	0.011	2.79E-02	-0.019	0.017	2.65E-01
1.150390506	0.014	0.006	2.00E-02	-0.001	0.009	9.26E-01
1.083103988	0.012	0.006	5.11E-02	-0.007	0.009	4.14E-01
3.755724	0.022	0.011	5.22E-02	0.032	0.017	5.04E-02
0.523280157	0.023	0.010	2.74E-02	0.015	0.015	3.20E-01
5.219543	0.007	0.005	1.45E-01	0.003	0.007	7.28E-01
4.278877	0.031	0.014	2.45E-02	0.025	0.020	2.16E-01
2.108886959	-0.012	0.007	9.56E-02	-0.024	0.010	1.79E-02
3.291111	-0.031	0.015	4.13E-02	-0.039	0.022	8.12E-02
6.886566	0.020	0.009	2.42E-02	-0.009	0.013	4.92E-01
4.247589	0.040	0.018	2.93E-02	0.015	0.027	5.82E-01
1.932932713	0.010	0.005	4.61E-02	0.004	0.007	5.56E-01
2.642805481	-0.027	0.011	1.42E-02	0.002	0.016	8.90E-01
1.090169073	0.015	0.007	3.54E-02	0.001	0.011	9.15E-01
1.618031808	0.014	0.006	2.48E-02	0.009	0.010	3.67E-01
1.011443846	0.024	0.011	2.35E-02	0.028	0.016	7.80E-02
0.700916565	0.029	0.013	2.13E-02	0.015	0.019	4.34E-01
4.278541	0.031	0.014	2.42E-02	0.022	0.020	2.92E-01
4.050103	0.036	0.015	2.13E-02	0.007	0.023	7.64E-01
3.432749	0.016	0.009	6.68E-02	0.032	0.013	1.41E-02
2.562734524	-0.017	0.008	4.34E-02	0.013	0.013	2.98E-01
5.172442	0.027	0.012	2.10E-02	0.001	0.018	9.45E-01
8.138095	-0.019	0.009	2.90E-02	-0.036	0.013	7.23E-03
2.063468559	0.009	0.004	1.86E-02	0.009	0.005	1.10E-01
1.804415464	-0.018	0.010	7.64E-02	-0.014	0.015	3.49E-01
3.252758	-0.013	0.007	5.52E-02	0.003	0.010	7.97E-01
2.914307	-0.016	0.008	4.18E-02	-0.008	0.011	5.00E-01
3.846898	0.011	0.006	7.94E-02	0.020	0.009	3.61E-02
6.887575	0.017	0.008	3.55E-02	0.001	0.012	9.37E-01
5.896782	0.024	0.011	2.51E-02	0.015	0.016	3.36E-01
5.681128	0.013	0.007	7.20E-02	-0.003	0.011	7.87E-01
1.114728652	0.016	0.007	3.36E-02	0.000	0.011	9.96E-01
2.382743088	-0.016	0.008	3.25E-02	0.005	0.011	6.60E-01
4.167518	-0.028	0.015	5.29E-02	-0.002	0.022	9.25E-01
8.68749	0.016	0.009	7.62E-02	-0.002	0.013	9.05E-01

5.220216	0.008	0.005	1.01E-01	0.007	0.007	3.48E-01
3.379929	0.020	0.013	1.04E-01	0.032	0.019	8.52E-02
8.803896	0.015	0.007	3.98E-02	0.005	0.011	6.54E-01
4.053804	0.041	0.018	2.60E-02	0.007	0.027	8.01E-01
6.235233	0.017	0.008	4.88E-02	0.003	0.013	8.39E-01
6.69009	-0.018	0.011	9.05E-02	-0.020	0.016	1.97E-01
2.993705	-0.008	0.004	4.88E-02	0.002	0.006	7.41E-01
3.52224	0.016	0.009	6.48E-02	0.037	0.013	3.15E-03
1.612648887	0.017	0.007	1.90E-02	0.001	0.011	9.08E-01
1.997191338	0.024	0.011	2.21E-02	0.002	0.016	9.09E-01
7.376748	-0.015	0.009	1.04E-01	-0.016	0.014	2.27E-01
7.784505	-0.022	0.009	1.75E-02	-0.009	0.014	5.36E-01
3.074448	-0.012	0.007	7.61E-02	-0.019	0.010	5.35E-02
5.547228	0.012	0.006	4.20E-02	0.005	0.009	5.76E-01
2.061113531	0.009	0.004	2.56E-02	0.008	0.006	2.17E-01
5.720827	0.012	0.007	9.98E-02	0.008	0.011	4.60E-01
4.253981	0.041	0.018	2.33E-02	0.010	0.027	7.05E-01
7.0494	-0.016	0.008	6.11E-02	-0.018	0.012	1.48E-01
8.679752	-0.017	0.008	2.59E-02	-0.014	0.012	2.15E-01
7.567842	0.017	0.010	7.38E-02	0.002	0.015	8.94E-01
1.796004649	-0.017	0.010	8.94E-02	0.008	0.014	5.84E-01
7.852801	-0.020	0.010	4.20E-02	0.020	0.014	1.66E-01
1.215994862	0.013	0.007	5.60E-02	0.008	0.010	4.10E-01
1.996854906	0.024	0.010	2.25E-02	0.002	0.016	9.18E-01
7.827568	-0.021	0.010	2.67E-02	0.005	0.014	7.03E-01
1.125158062	0.014	0.007	3.90E-02	-0.007	0.010	4.66E-01
7.64724	-0.017	0.011	1.28E-01	-0.021	0.016	1.92E-01
6.874118	0.019	0.010	6.86E-02	0.013	0.015	3.99E-01
2.131764375	-0.025	0.011	2.27E-02	-0.025	0.016	1.25E-01
2.220919011	0.040	0.021	5.66E-02	0.017	0.031	5.80E-01
8.407578	0.020	0.009	2.68E-02	-0.009	0.014	4.96E-01
2.058758503	0.011	0.005	2.42E-02	0.010	0.007	1.41E-01
3.964986	-0.017	0.008	2.64E-02	0.007	0.012	5.65E-01
2.50890531	-0.023	0.013	6.82E-02	-0.026	0.019	1.71E-01
1.875066308	-0.014	0.007	4.52E-02	0.005	0.011	6.40E-01
7.192047	0.017	0.009	6.55E-02	0.001	0.014	9.23E-01
5.238719	0.010	0.007	1.41E-01	0.012	0.010	2.38E-01
5.681465	0.013	0.008	9.79E-02	0.008	0.011	4.69E-01
3.486578	0.015	0.009	8.46E-02	0.037	0.013	4.88E-03
7.632774	-0.023	0.012	5.37E-02	0.011	0.017	5.30E-01
2.487710056	-0.026	0.012	2.44E-02	-0.009	0.017	5.97E-01
1.012453144	0.026	0.011	2.17E-02	0.022	0.017	1.90E-01
6.078455	0.014	0.007	5.53E-02	0.001	0.011	9.27E-01

4.279214	0.030	0.013	2.59E-02	0.025	0.020	2.10E-01
0.913878395	-0.010	0.005	4.85E-02	-0.030	0.007	6.53E-05
2.203760949	0.036	0.016	2.65E-02	-0.006	0.024	7.93E-01
1.083776853	0.010	0.006	8.73E-02	-0.004	0.009	6.76E-01
1.082431123	0.012	0.006	4.96E-02	0.001	0.009	9.41E-01
3.481195	0.016	0.010	8.91E-02	0.035	0.014	1.50E-02
5.481287	0.014	0.007	5.24E-02	0.015	0.011	1.62E-01
8.803559	0.016	0.008	4.71E-02	-0.001	0.012	9.44E-01
3.486915	0.015	0.009	8.70E-02	0.037	0.013	5.57E-03
3.257131	0.018	0.010	6.53E-02	0.024	0.014	8.68E-02
5.702996	0.014	0.007	2.95E-02	0.007	0.010	4.54E-01
1.844114509	-0.018	0.009	5.53E-02	-0.006	0.014	6.93E-01
1.934278444	0.010	0.005	4.09E-02	0.004	0.008	5.71E-01
3.766154	0.024	0.010	1.67E-02	0.037	0.015	1.40E-02
4.052794	0.040	0.018	2.40E-02	0.007	0.026	7.80E-01
1.26376829	0.035	0.016	2.76E-02	0.004	0.024	8.68E-01
1.551418155	0.041	0.019	2.90E-02	-0.001	0.028	9.72E-01
6.641643	0.016	0.010	1.10E-01	0.003	0.015	8.21E-01
6.769488	-0.017	0.011	1.05E-01	0.006	0.016	7.15E-01
1.956482995	0.011	0.005	3.34E-02	0.004	0.008	5.81E-01
2.062795694	0.009	0.004	1.90E-02	0.010	0.006	8.94E-02
7.143264	-0.020	0.010	4.49E-02	-0.012	0.015	4.23E-01
2.994041	-0.008	0.004	5.13E-02	0.001	0.006	8.21E-01
0.516551505	0.014	0.008	7.89E-02	0.008	0.012	4.81E-01
3.706942	0.014	0.007	3.75E-02	0.025	0.010	1.04E-02
2.049674823	0.015	0.007	2.61E-02	0.008	0.010	4.31E-01
1.314906044	0.027	0.011	1.24E-02	0.011	0.016	5.09E-01
7.616288	-0.029	0.013	1.95E-02	-0.016	0.019	3.91E-01
1.016826768	0.023	0.010	2.14E-02	0.006	0.015	6.93E-01
5.967096	0.017	0.009	6.74E-02	-0.002	0.013	8.62E-01
2.576864693	-0.019	0.013	1.30E-01	0.012	0.019	5.04E-01
3.902073	0.012	0.007	6.72E-02	0.027	0.010	6.16E-03
6.716331	-0.021	0.010	4.00E-02	-0.022	0.015	1.56E-01
1.474711524	-0.009	0.004	4.57E-02	-0.003	0.006	6.25E-01
3.034077	0.017	0.011	1.06E-01	0.035	0.016	2.74E-02
3.400452	0.014	0.008	9.59E-02	0.037	0.013	3.61E-03
1.318606802	0.025	0.010	1.42E-02	0.009	0.015	5.34E-01
2.884364	-0.016	0.008	3.83E-02	-0.006	0.012	6.16E-01
2.062459261	0.009	0.004	2.00E-02	0.009	0.006	1.14E-01
5.854391	0.019	0.009	3.16E-02	-0.013	0.013	3.20E-01
1.491869586	-0.009	0.004	3.63E-02	-0.010	0.006	9.75E-02
2.053375581	0.014	0.006	2.55E-02	0.007	0.009	4.39E-01
2.054721311	0.014	0.006	2.18E-02	0.008	0.009	3.87E-01

6.227831	0.014	0.007	4.56E-02	-0.007	0.011	5.35E-01
4.259028	0.038	0.017	2.32E-02	0.022	0.025	3.66E-01
1.89996232	-0.014	0.007	2.64E-02	-0.009	0.010	3.72E-01
5.710061	0.016	0.008	5.01E-02	-0.008	0.012	5.04E-01
2.88571	-0.014	0.007	4.51E-02	-0.005	0.011	6.49E-01
4.050439	0.036	0.016	2.53E-02	0.006	0.024	8.10E-01
1.632834842	0.014	0.006	3.04E-02	0.006	0.010	5.54E-01
6.67024	0.020	0.010	3.43E-02	0.020	0.014	1.63E-01
2.307045755	-0.011	0.005	4.60E-02	-0.002	0.008	8.39E-01
3.54209	0.013	0.008	1.06E-01	0.025	0.012	3.97E-02
2.199387326	0.030	0.014	2.66E-02	-0.009	0.020	6.56E-01
2.255235136	0.017	0.007	2.63E-02	0.008	0.011	4.58E-01
4.248935	0.039	0.018	2.81E-02	0.015	0.026	5.69E-01
5.171096	0.030	0.013	2.63E-02	-0.005	0.020	8.05E-01
1.069983117	0.011	0.006	5.21E-02	0.001	0.009	9.48E-01
1.551754588	0.041	0.019	2.93E-02	0.000	0.028	9.92E-01
7.641857	-0.019	0.011	7.87E-02	-0.021	0.016	1.78E-01
3.505082	0.017	0.010	8.07E-02	0.037	0.014	1.07E-02
3.956575	-0.013	0.008	9.80E-02	-0.023	0.012	6.03E-02
1.55209102	0.041	0.019	2.99E-02	0.000	0.028	9.88E-01
2.19871446	0.028	0.013	3.27E-02	-0.009	0.020	6.54E-01
7.973243	0.014	0.007	5.37E-02	0.006	0.011	5.61E-01
8.546525	-0.012	0.007	6.30E-02	-0.009	0.010	3.90E-01
3.401797	0.016	0.009	9.00E-02	0.040	0.014	3.11E-03
1.936633472	0.012	0.005	3.16E-02	0.012	0.008	1.27E-01
8.745693	0.012	0.006	6.75E-02	0.000	0.010	9.92E-01
3.344267	-0.017	0.011	1.26E-01	-0.002	0.016	9.05E-01
3.085887	-0.013	0.007	5.82E-02	0.009	0.010	3.75E-01
5.220888	0.007	0.005	1.76E-01	0.004	0.007	5.59E-01
2.224956203	0.039	0.021	6.11E-02	0.019	0.031	5.29E-01
5.695595	0.016	0.009	6.78E-02	0.003	0.013	8.04E-01
6.130602	0.015	0.008	4.39E-02	0.028	0.011	1.43E-02
4.053131	0.040	0.018	2.63E-02	0.005	0.027	8.56E-01
4.252299	0.039	0.017	2.57E-02	0.006	0.026	8.07E-01
1.640236359	0.013	0.006	3.41E-02	0.003	0.009	7.09E-01
1.551081723	0.040	0.019	3.03E-02	-0.001	0.028	9.79E-01
5.133752	0.017	0.008	4.06E-02	-0.001	0.012	9.07E-01
6.597907	0.018	0.010	6.71E-02	-0.009	0.015	5.35E-01
3.293803	-0.021	0.012	9.38E-02	-0.006	0.019	7.54E-01
7.439661	-0.016	0.010	8.81E-02	-0.006	0.014	6.95E-01
2.016031563	0.023	0.010	2.26E-02	0.005	0.015	7.20E-01
1.847815268	-0.017	0.008	4.31E-02	-0.017	0.013	1.81E-01
2.861487	-0.015	0.008	6.36E-02	-0.001	0.012	9.16E-01

1.958838023	0.010	0.005	4.13E-02	0.001	0.007	8.66E-01
3.401461	0.015	0.009	9.32E-02	0.040	0.013	2.56E-03
3.157884	-0.015	0.008	4.85E-02	-0.010	0.011	3.60E-01
1.331391241	0.029	0.012	1.71E-02	0.014	0.018	4.34E-01
5.401216	0.012	0.007	8.41E-02	-0.013	0.010	2.00E-01
7.094145	-0.020	0.009	3.06E-02	-0.023	0.014	9.49E-02
2.319157328	-0.013	0.007	6.45E-02	-0.006	0.010	5.80E-01
1.55074529	0.040	0.018	3.04E-02	0.000	0.028	9.91E-01
0.540101786	0.017	0.008	2.94E-02	0.011	0.012	3.44E-01
8.08292	0.020	0.010	4.41E-02	0.008	0.015	5.76E-01
3.253094	-0.014	0.007	5.54E-02	0.003	0.011	7.54E-01
0.666936873	-0.019	0.009	3.68E-02	-0.017	0.014	2.21E-01
6.670913	0.015	0.007	4.26E-02	0.003	0.011	7.61E-01
3.481532	0.016	0.010	8.66E-02	0.035	0.014	1.51E-02
8.709022	0.017	0.009	4.80E-02	-0.015	0.013	2.31E-01
2.220582579	0.040	0.021	5.95E-02	0.017	0.031	5.93E-01
4.250953	0.037	0.016	2.32E-02	0.011	0.024	6.44E-01
8.789429	0.020	0.009	2.63E-02	-0.021	0.014	1.31E-01
3.655468	-0.010	0.005	3.63E-02	0.005	0.007	4.38E-01
7.640512	-0.016	0.010	1.10E-01	-0.018	0.015	2.17E-01
3.720736	0.015	0.008	6.43E-02	0.027	0.012	2.62E-02
1.011107414	0.023	0.010	2.57E-02	0.027	0.015	8.03E-02
2.711101297	-0.015	0.006	9.63E-03	-0.015	0.008	6.93E-02
2.314447272	-0.009	0.005	6.43E-02	-0.006	0.007	4.46E-01
5.125005	0.013	0.008	8.96E-02	0.002	0.011	8.86E-01
1.996518473	0.023	0.010	2.43E-02	0.001	0.015	9.41E-01
3.400115	0.014	0.008	1.06E-01	0.035	0.013	5.03E-03
3.29683	-0.020	0.011	7.73E-02	0.000	0.017	1.00E+00
0.869805725	0.033	0.015	2.82E-02	0.014	0.022	5.15E-01
7.262361	-0.025	0.011	1.94E-02	0.001	0.016	9.57E-01
1.492878884	-0.009	0.004	3.83E-02	-0.007	0.006	2.39E-01
3.379593	0.021	0.013	9.82E-02	0.030	0.019	1.08E-01
3.116166	-0.021	0.010	3.43E-02	-0.002	0.015	8.93E-01
3.673971	-0.009	0.005	5.46E-02	0.001	0.007	8.75E-01
6.64568	-0.018	0.009	3.65E-02	-0.013	0.013	2.96E-01
2.201405921	0.032	0.015	2.98E-02	-0.014	0.022	5.18E-01
5.221225	0.007	0.005	1.70E-01	0.004	0.007	6.23E-01
2.058085637	0.011	0.005	2.76E-02	0.011	0.008	1.49E-01
2.256244434	0.017	0.008	2.82E-02	0.011	0.011	3.49E-01
7.217279	-0.017	0.008	3.05E-02	-0.014	0.012	2.09E-01
6.091576	0.013	0.007	8.03E-02	0.005	0.011	6.35E-01
1.783893076	-0.017	0.010	1.05E-01	-0.009	0.016	5.67E-01
2.99034	-0.008	0.004	3.79E-02	0.003	0.006	6.26E-01

1.550408857	0.040	0.018	3.09E-02	-0.001	0.027	9.79E-01
5.223916	0.008	0.005	1.41E-01	0.012	0.008	1.34E-01
0.91320553	-0.010	0.005	5.06E-02	-0.027	0.007	3.14E-04
1.17158576	0.010	0.004	1.83E-02	-0.002	0.007	7.98E-01
2.259945192	0.012	0.006	4.41E-02	0.012	0.009	1.86E-01
1.957828725	0.010	0.005	3.71E-02	0.002	0.007	7.47E-01
1.492542452	-0.009	0.004	3.20E-02	-0.008	0.006	2.15E-01
8.152898	0.013	0.009	1.29E-01	0.008	0.013	5.37E-01
7.728657	-0.014	0.009	1.18E-01	-0.011	0.014	4.31E-01
5.507529	0.014	0.008	7.14E-02	-0.002	0.011	8.88E-01
7.287594	-0.017	0.009	6.60E-02	-0.016	0.014	2.61E-01
0.776277465	0.009	0.005	6.26E-02	0.006	0.007	3.85E-01
5.157303	0.018	0.008	2.98E-02	-0.003	0.013	8.30E-01
0.863413506	0.024	0.011	2.84E-02	0.010	0.016	5.45E-01
6.414215	0.018	0.009	5.73E-02	-0.004	0.014	7.71E-01
0.863749939	0.024	0.011	2.81E-02	0.010	0.016	5.45E-01
2.978565	-0.008	0.004	5.29E-02	0.006	0.006	3.66E-01
3.261841	0.015	0.008	5.93E-02	0.026	0.012	2.93E-02
0.863077074	0.023	0.011	2.89E-02	0.010	0.016	5.41E-01
6.648372	-0.018	0.009	5.01E-02	-0.011	0.014	4.16E-01
1.631489112	0.012	0.006	5.28E-02	0.007	0.009	4.45E-01
2.061449963	0.009	0.004	2.55E-02	0.008	0.006	1.78E-01
3.669598	-0.008	0.004	7.33E-02	0.002	0.007	7.26E-01
1.484131637	-0.009	0.005	5.23E-02	-0.002	0.007	7.79E-01
3.864056	-0.010	0.005	3.89E-02	0.004	0.007	6.17E-01
3.5034	0.014	0.009	1.06E-01	0.034	0.013	8.57E-03
6.554844	0.021	0.010	2.46E-02	0.009	0.014	5.16E-01
7.644885	-0.017	0.011	1.13E-01	-0.005	0.016	7.70E-01
7.952048	0.015	0.008	6.28E-02	-0.006	0.012	6.16E-01
8.561328	0.011	0.009	1.99E-01	0.015	0.013	2.68E-01
1.128858821	0.015	0.007	3.33E-02	-0.002	0.011	8.21E-01
7.050072	-0.016	0.009	5.61E-02	-0.016	0.013	2.01E-01
8.583532	0.013	0.007	6.82E-02	0.011	0.011	3.00E-01
1.859926841	-0.017	0.008	4.18E-02	0.008	0.013	5.40E-01
3.095644	-0.017	0.008	3.16E-02	-0.021	0.012	7.45E-02
0.862740641	0.023	0.011	2.98E-02	0.009	0.016	5.50E-01
5.221898	0.007	0.005	1.61E-01	0.006	0.007	4.17E-01
5.380694	0.021	0.011	4.55E-02	-0.020	0.016	2.05E-01
3.816282	0.012	0.007	9.13E-02	0.032	0.010	1.87E-03
5.721164	0.016	0.009	6.72E-02	0.010	0.013	4.26E-01
2.506886714	-0.028	0.014	4.16E-02	-0.020	0.020	3.29E-01
8.867818	0.017	0.009	5.40E-02	0.008	0.013	5.14E-01
2.062122828	0.009	0.004	2.15E-02	0.009	0.006	1.48E-01

7.187337	-0.018	0.011	9.75E-02	-0.030	0.016	5.51E-02
0.742297773	-0.012	0.006	5.32E-02	-0.009	0.009	3.43E-01
3.158893	-0.015	0.007	4.53E-02	-0.013	0.011	2.46E-01
2.687551015	-0.013	0.005	1.36E-02	-0.003	0.008	6.83E-01
3.52796	0.014	0.008	6.10E-02	0.031	0.011	5.76E-03
8.408587	0.018	0.009	4.09E-02	-0.011	0.013	4.02E-01
2.623292391	-0.020	0.012	9.92E-02	0.009	0.018	6.07E-01
0.864086371	0.024	0.011	2.85E-02	0.010	0.017	5.45E-01
5.744041	0.020	0.011	5.89E-02	0.001	0.016	9.72E-01
2.102494739	-0.009	0.006	1.00E-01	0.013	0.008	1.08E-01
1.323989724	0.031	0.013	2.08E-02	0.017	0.020	3.85E-01
3.347632	-0.019	0.012	9.89E-02	-0.014	0.018	4.19E-01
2.202415219	0.033	0.015	3.15E-02	-0.008	0.023	7.20E-01
3.510129	0.014	0.008	8.43E-02	0.029	0.012	1.60E-02
5.648494	0.016	0.008	5.35E-02	0.004	0.012	7.58E-01
1.893233668	-0.011	0.006	7.17E-02	-0.012	0.009	1.84E-01
2.199723758	0.030	0.014	2.88E-02	-0.011	0.021	6.00E-01
2.653571324	-0.023	0.011	3.71E-02	0.006	0.017	7.06E-01
1.952109371	0.012	0.006	3.39E-02	0.006	0.009	4.94E-01
1.153754832	0.012	0.006	2.84E-02	-0.005	0.008	5.25E-01
3.381275	0.021	0.012	7.23E-02	0.032	0.017	6.18E-02
8.743674	0.016	0.008	5.28E-02	0.004	0.012	7.38E-01
3.138707	-0.020	0.010	5.35E-02	-0.007	0.015	6.37E-01
1.900298752	-0.014	0.006	2.57E-02	-0.010	0.010	3.08E-01
5.8554	0.016	0.007	2.65E-02	-0.012	0.011	2.66E-01
5.340995	0.039	0.019	3.97E-02	0.001	0.028	9.72E-01
2.04933839	0.015	0.007	2.92E-02	0.008	0.010	4.28E-01
3.499363	0.013	0.008	9.89E-02	0.035	0.012	4.06E-03
3.487588	0.015	0.009	9.84E-02	0.039	0.014	4.60E-03
2.061786396	0.009	0.004	2.32E-02	0.008	0.006	1.92E-01
1.12751309	0.016	0.007	2.44E-02	-0.001	0.011	9.37E-01
1.61433105	0.016	0.007	2.40E-02	0.004	0.010	7.32E-01
3.385649	0.014	0.009	1.30E-01	0.026	0.014	6.26E-02
3.027684	-0.009	0.005	7.13E-02	-0.005	0.008	5.42E-01
6.965291	-0.021	0.011	4.47E-02	-0.028	0.016	7.47E-02
2.322521654	-0.015	0.008	4.79E-02	-0.003	0.012	7.90E-01
1.898953022	-0.013	0.006	3.61E-02	-0.009	0.009	3.68E-01
2.68418669	-0.013	0.005	1.88E-02	-0.002	0.008	7.83E-01
5.856073	0.021	0.011	5.30E-02	0.001	0.016	9.36E-01
5.893754	0.018	0.009	5.55E-02	-0.024	0.014	8.97E-02
1.957492293	0.010	0.005	4.29E-02	0.004	0.007	6.00E-01
1.083440421	0.010	0.006	8.32E-02	-0.003	0.009	7.76E-01
6.242634	0.019	0.010	4.58E-02	-0.006	0.014	6.62E-01

1.138278933	0.015	0.007	2.95E-02	-0.008	0.010	4.51E-01
2.147576707	-0.024	0.010	1.71E-02	0.003	0.015	8.38E-01
7.955749	0.016	0.009	6.97E-02	-0.002	0.013	8.48E-01
6.920546	-0.023	0.011	3.73E-02	0.006	0.017	7.29E-01
5.170087	0.025	0.012	3.03E-02	-0.007	0.018	7.08E-01
7.615279	-0.021	0.010	3.42E-02	-0.010	0.015	4.93E-01
7.402654	0.016	0.009	6.18E-02	-0.010	0.013	4.32E-01
5.447308	0.014	0.007	5.29E-02	0.004	0.011	7.17E-01
1.937306337	0.011	0.005	3.79E-02	0.009	0.008	2.52E-01
8.997008	0.019	0.010	6.21E-02	0.003	0.015	8.42E-01
5.665989	0.016	0.010	9.77E-02	-0.002	0.015	9.11E-01
5.134762	0.015	0.008	5.31E-02	0.009	0.011	4.04E-01
2.197705163	0.027	0.013	3.24E-02	-0.010	0.019	5.83E-01
7.557749	-0.015	0.008	4.95E-02	-0.007	0.012	5.64E-01
2.623628823	-0.019	0.011	8.77E-02	0.009	0.017	5.76E-01
5.38204	0.015	0.008	4.35E-02	-0.001	0.011	9.07E-01
2.978229	-0.008	0.004	6.99E-02	0.003	0.006	5.92E-01
2.220246146	0.040	0.021	6.33E-02	0.014	0.031	6.45E-01
6.436756	0.016	0.009	8.96E-02	-0.001	0.014	9.62E-01
0.978473452	0.013	0.007	4.92E-02	0.011	0.010	2.79E-01
3.400788	0.014	0.009	1.03E-01	0.037	0.013	3.85E-03
2.991013	-0.008	0.004	4.44E-02	0.002	0.006	7.40E-01
3.069066	-0.011	0.006	6.47E-02	-0.010	0.009	2.85E-01
7.794934	-0.015	0.008	5.63E-02	0.012	0.012	3.08E-01
1.709877906	-0.008	0.004	5.67E-02	0.002	0.007	7.43E-01
2.202078786	0.033	0.015	3.17E-02	-0.008	0.023	7.16E-01
1.167548569	0.011	0.004	1.82E-02	0.002	0.007	7.25E-01
1.630479814	0.012	0.006	5.82E-02	0.013	0.009	1.60E-01
8.539123	0.011	0.006	8.44E-02	0.018	0.009	5.35E-02
0.766184487	0.011	0.005	4.36E-02	0.006	0.008	4.73E-01
1.151736237	0.012	0.006	3.31E-02	-0.006	0.009	4.82E-01
0.862404208	0.022	0.010	3.19E-02	0.009	0.016	5.72E-01
2.681831661	-0.013	0.005	1.53E-02	0.001	0.008	8.63E-01
2.709082701	-0.015	0.006	1.43E-02	-0.017	0.009	7.24E-02
7.045699	-0.029	0.013	2.53E-02	-0.019	0.019	3.19E-01
1.324999021	0.029	0.013	1.99E-02	0.016	0.019	4.04E-01
3.34595	-0.017	0.010	8.35E-02	-0.004	0.015	7.71E-01
1.263431857	0.034	0.016	3.22E-02	0.003	0.024	9.14E-01
1.894242966	-0.013	0.007	5.23E-02	-0.006	0.010	5.51E-01
3.401125	0.014	0.009	1.01E-01	0.039	0.013	3.02E-03
1.010770981	0.022	0.010	2.68E-02	0.028	0.015	5.98E-02
3.386994	0.015	0.009	1.10E-01	0.034	0.014	1.55E-02
1.483795204	-0.009	0.005	6.04E-02	0.001	0.007	9.05E-01

8.464435	-0.014	0.007	4.47E-02	-0.022	0.010	3.27E-02
3.501718	0.014	0.008	9.48E-02	0.036	0.012	3.24E-03
2.887056	-0.013	0.007	5.95E-02	-0.004	0.010	6.98E-01
1.935960607	0.012	0.005	3.00E-02	0.018	0.008	2.54E-02
7.508966	-0.015	0.009	9.44E-02	-0.024	0.014	7.15E-02
7.890481	0.019	0.009	4.05E-02	0.035	0.014	1.18E-02
3.582125	-0.009	0.006	1.25E-01	0.004	0.009	6.42E-01
4.055486	0.039	0.018	3.10E-02	0.006	0.027	8.34E-01
4.052458	0.039	0.018	2.73E-02	0.008	0.026	7.53E-01
1.936969905	0.011	0.005	3.50E-02	0.011	0.008	1.62E-01
2.113933448	-0.011	0.007	1.05E-01	-0.012	0.010	2.49E-01
7.890817	0.017	0.008	4.22E-02	0.028	0.012	2.45E-02
7.824204	-0.023	0.010	1.53E-02	0.000	0.014	9.85E-01
3.347968	-0.018	0.011	1.06E-01	-0.013	0.017	4.31E-01
5.665652	0.014	0.009	1.05E-01	-0.002	0.013	8.92E-01
2.592340592	-0.028	0.017	1.04E-01	-0.016	0.026	5.21E-01
4.049767	0.033	0.015	2.54E-02	0.003	0.022	8.82E-01
8.159627	0.015	0.008	6.42E-02	-0.021	0.012	8.57E-02
0.864422804	0.025	0.011	2.99E-02	0.010	0.017	5.59E-01
3.386322	0.014	0.009	1.31E-01	0.031	0.014	2.71E-02
3.29313	-0.022	0.013	1.00E-01	-0.023	0.020	2.32E-01
1.325335454	0.029	0.012	1.95E-02	0.014	0.018	4.32E-01
1.018508931	0.020	0.009	2.25E-02	0.021	0.013	1.02E-01
2.992359	-0.008	0.004	5.27E-02	0.002	0.006	6.94E-01
8.448959	0.013	0.008	1.26E-01	0.004	0.013	7.71E-01
1.959174456	0.010	0.005	4.71E-02	0.001	0.007	8.64E-01
6.971347	-0.023	0.011	2.79E-02	-0.013	0.016	4.15E-01
4.259701	0.035	0.016	2.75E-02	0.018	0.024	4.47E-01
8.708685	0.018	0.009	5.25E-02	0.005	0.014	7.15E-01
5.776339	0.014	0.008	7.58E-02	-0.010	0.012	3.75E-01
4.277195	0.032	0.015	2.88E-02	0.023	0.022	2.85E-01
3.385985	0.013	0.009	1.44E-01	0.027	0.014	5.24E-02
2.591331294	-0.027	0.017	1.03E-01	-0.020	0.025	4.34E-01
6.769151	-0.016	0.010	8.88E-02	0.009	0.014	5.13E-01
5.412319	0.018	0.009	4.29E-02	0.004	0.014	7.84E-01
3.844879	0.011	0.006	8.07E-02	0.025	0.009	6.13E-03
5.537135	0.017	0.008	3.43E-02	0.009	0.012	4.34E-01
3.39305	0.014	0.009	1.11E-01	0.044	0.013	8.43E-04
3.502054	0.014	0.008	9.20E-02	0.035	0.012	4.02E-03
5.568423	0.016	0.007	3.30E-02	0.006	0.011	5.63E-01
3.88256	0.013	0.007	7.49E-02	0.027	0.011	1.33E-02
3.842524	0.012	0.007	7.24E-02	0.022	0.010	2.70E-02
5.030804	0.017	0.010	7.59E-02	-0.003	0.015	8.13E-01

5.41972	0.009	0.006	1.12E-01	0.009	0.009	2.99E-01
1.493215317	-0.008	0.004	4.97E-02	-0.008	0.006	1.88E-01
1.324662589	0.030	0.013	2.12E-02	0.016	0.019	3.91E-01
1.996182041	0.022	0.010	2.70E-02	0.001	0.015	9.33E-01
5.92706	-0.016	0.008	5.10E-02	-0.010	0.012	3.93E-01
1.196481771	0.008	0.004	6.53E-02	0.009	0.007	1.90E-01
6.882193	0.022	0.011	4.25E-02	0.012	0.016	4.44E-01
2.711774162	-0.014	0.006	1.30E-02	-0.016	0.009	6.57E-02
5.389105	0.018	0.008	3.33E-02	0.013	0.012	2.84E-01
3.347295	-0.019	0.012	1.10E-01	-0.014	0.018	4.44E-01
2.512269635	-0.023	0.013	7.37E-02	-0.010	0.019	5.92E-01
6.99187	0.011	0.007	9.24E-02	0.020	0.010	4.52E-02
8.407241	0.020	0.009	2.66E-02	-0.001	0.013	9.28E-01
2.912961	-0.016	0.008	4.21E-02	-0.010	0.012	3.79E-01
5.924705	-0.016	0.009	7.74E-02	0.000	0.013	9.85E-01
0.869469293	0.031	0.015	3.17E-02	0.014	0.022	5.36E-01
7.116686	-0.021	0.011	5.84E-02	0.001	0.016	9.75E-01
5.046953	0.011	0.006	1.04E-01	0.003	0.010	7.19E-01
3.855981	0.011	0.007	1.04E-01	0.024	0.010	1.49E-02
7.822185	-0.022	0.010	2.95E-02	-0.021	0.015	1.63E-01
8.712722	0.019	0.010	6.40E-02	0.009	0.015	5.45E-01
2.981593	-0.007	0.004	7.83E-02	0.005	0.006	3.78E-01
1.185379496	0.010	0.005	3.45E-02	0.007	0.007	3.19E-01
8.403204	0.016	0.009	7.78E-02	0.007	0.013	5.73E-01
1.011780279	0.024	0.011	2.73E-02	0.025	0.016	1.27E-01
5.526706	0.015	0.008	5.14E-02	-0.009	0.012	4.42E-01
1.642254955	0.011	0.006	5.92E-02	0.015	0.008	6.64E-02
2.404274774	-0.022	0.011	4.21E-02	0.015	0.016	3.63E-01
6.300164	-0.010	0.007	1.34E-01	-0.011	0.010	2.93E-01
2.863505	-0.014	0.008	9.08E-02	-0.002	0.012	8.75E-01
6.832064	0.014	0.008	9.21E-02	0.016	0.013	1.94E-01
7.926479	0.015	0.009	9.05E-02	0.001	0.014	9.40E-01
3.495999	0.015	0.010	1.15E-01	0.031	0.014	2.96E-02
6.991533	0.011	0.007	9.74E-02	0.015	0.010	1.20E-01
8.999026	0.012	0.009	1.63E-01	0.011	0.013	4.08E-01
7.328302	-0.011	0.006	7.14E-02	-0.001	0.009	9.05E-01
1.64158209	0.011	0.006	5.20E-02	0.011	0.009	2.22E-01
5.340658	0.039	0.019	4.09E-02	0.004	0.028	8.92E-01
8.779672	0.013	0.007	5.46E-02	0.015	0.010	1.19E-01
8.71575	0.015	0.009	7.77E-02	0.014	0.013	2.99E-01
3.58549	-0.008	0.006	2.29E-01	0.004	0.010	6.84E-01
0.5185701	0.018	0.009	4.06E-02	0.006	0.013	6.30E-01
6.461652	0.014	0.008	8.41E-02	-0.001	0.012	9.33E-01

6.879165	0.021	0.009	2.07E-02	-0.005	0.014	7.10E-01
5.466484	0.011	0.007	1.19E-01	-0.009	0.010	3.91E-01
5.742023	0.016	0.009	6.76E-02	0.010	0.013	4.16E-01
1.136933203	0.014	0.007	3.55E-02	-0.003	0.010	7.82E-01
4.276522	0.033	0.015	2.95E-02	0.013	0.022	5.50E-01
1.324326156	0.030	0.013	2.23E-02	0.017	0.020	3.86E-01
6.2534	0.018	0.009	4.78E-02	0.006	0.014	6.59E-01
2.201069489	0.031	0.015	3.45E-02	-0.015	0.022	4.99E-01
8.119255	-0.019	0.009	4.24E-02	0.004	0.014	7.51E-01
8.114882	0.012	0.007	8.66E-02	0.019	0.011	7.51E-02
3.503064	0.013	0.009	1.25E-01	0.035	0.013	6.59E-03
1.951772939	0.012	0.006	3.98E-02	0.005	0.009	5.80E-01
1.318270369	0.023	0.010	1.59E-02	0.010	0.014	4.74E-01
2.428161488	-0.017	0.009	5.41E-02	0.005	0.013	6.96E-01
8.279397	0.012	0.009	1.79E-01	0.017	0.014	2.29E-01
1.657057989	0.009	0.004	4.09E-02	0.013	0.007	4.97E-02
0.913541962	-0.010	0.005	5.65E-02	-0.028	0.007	1.91E-04
5.085643	0.018	0.010	8.81E-02	-0.014	0.015	3.60E-01
0.96131539	0.009	0.004	4.73E-02	0.013	0.006	4.42E-02
1.995509175	0.021	0.010	2.74E-02	0.001	0.014	9.28E-01
3.285055	-0.024	0.012	4.15E-02	-0.015	0.018	4.01E-01
1.216331294	0.012	0.007	6.77E-02	0.008	0.010	4.31E-01
1.104635674	0.015	0.007	4.14E-02	-0.003	0.011	7.61E-01
5.169414	0.023	0.011	3.22E-02	-0.003	0.016	8.74E-01
8.780345	0.015	0.007	2.77E-02	0.003	0.011	7.52E-01
3.920913	0.023	0.011	4.66E-02	0.048	0.017	4.42E-03
0.701925862	0.023	0.011	4.05E-02	0.022	0.017	2.04E-01
2.682504527	-0.013	0.005	1.49E-02	0.002	0.008	7.71E-01
6.379899	0.017	0.009	6.24E-02	0.023	0.013	9.12E-02
2.385770981	-0.022	0.010	2.85E-02	0.008	0.015	5.95E-01
6.688744	-0.019	0.010	5.48E-02	-0.014	0.015	3.29E-01
2.511933203	-0.021	0.014	1.20E-01	-0.023	0.020	2.66E-01
2.016367996	0.022	0.010	2.72E-02	0.005	0.015	7.61E-01
3.253431	-0.014	0.008	6.18E-02	0.004	0.011	7.47E-01
5.425439	0.012	0.006	6.72E-02	-0.006	0.009	5.14E-01
3.547136	0.021	0.009	2.34E-02	0.027	0.014	5.36E-02
4.149014	-0.034	0.018	6.16E-02	0.019	0.027	4.95E-01
5.586591	0.016	0.007	3.00E-02	-0.009	0.011	4.27E-01
0.864759237	0.025	0.011	3.15E-02	0.010	0.017	5.69E-01
5.868858	0.015	0.008	5.62E-02	-0.005	0.012	6.57E-01
6.253064	0.019	0.009	4.01E-02	-0.007	0.014	6.24E-01
6.918527	-0.020	0.010	4.94E-02	0.008	0.015	6.06E-01
1.619377539	0.014	0.006	3.14E-02	0.003	0.009	7.63E-01

2.867879	-0.013	0.008	1.11E-01	-0.007	0.012	5.66E-01
3.781293	0.015	0.008	7.08E-02	0.020	0.012	1.01E-01
1.95581013	0.011	0.005	3.58E-02	0.005	0.008	5.32E-01
7.008691	-0.021	0.009	2.78E-02	-0.016	0.014	2.62E-01
1.127176658	0.015	0.007	2.94E-02	-0.003	0.010	8.07E-01
1.611976022	0.017	0.008	2.16E-02	0.003	0.011	8.12E-01
5.167059	0.022	0.011	3.97E-02	-0.013	0.016	4.04E-01
2.59200416	-0.028	0.018	1.21E-01	-0.009	0.027	7.45E-01
6.642653	0.014	0.009	1.19E-01	-0.003	0.013	8.28E-01
8.139105	-0.018	0.008	2.97E-02	-0.013	0.012	2.97E-01
2.769304135	-0.020	0.010	4.15E-02	-0.046	0.015	1.93E-03
5.396843	0.016	0.008	5.25E-02	-0.010	0.013	4.22E-01
2.568790311	-0.017	0.008	4.32E-02	-0.012	0.013	3.31E-01
1.959847321	0.010	0.005	4.51E-02	0.002	0.007	8.15E-01
2.053712014	0.013	0.006	2.95E-02	0.006	0.009	4.81E-01
2.300989968	-0.007	0.004	9.00E-02	-0.002	0.007	7.59E-01
1.791294593	-0.017	0.010	7.01E-02	-0.010	0.014	4.97E-01
2.987985	-0.007	0.004	7.48E-02	0.006	0.006	3.24E-01
5.464129	0.017	0.009	6.85E-02	0.013	0.014	3.44E-01
7.196757	0.019	0.008	2.44E-02	-0.004	0.013	7.33E-01
6.965628	-0.020	0.011	5.65E-02	-0.025	0.016	1.20E-01
3.560257	-0.016	0.009	5.87E-02	0.016	0.013	2.23E-01
4.259364	0.036	0.016	2.77E-02	0.021	0.024	3.96E-01
3.538053	0.015	0.008	5.47E-02	0.024	0.012	4.29E-02
1.643264253	0.011	0.006	5.51E-02	0.017	0.009	5.40E-02
5.169751	0.025	0.012	3.01E-02	-0.007	0.017	6.94E-01
7.380449	-0.018	0.009	6.14E-02	-0.030	0.014	3.07E-02
0.526644483	0.019	0.009	2.89E-02	-0.015	0.013	2.43E-01
1.325671886	0.028	0.012	2.00E-02	0.012	0.018	4.88E-01
3.055945	-0.010	0.006	1.12E-01	0.000	0.009	9.97E-01
3.521904	0.014	0.008	8.30E-02	0.036	0.012	3.11E-03
6.905406	0.015	0.008	6.01E-02	0.012	0.012	2.99E-01
3.152164	-0.020	0.011	6.15E-02	-0.023	0.016	1.44E-01
2.201742354	0.032	0.015	3.38E-02	-0.011	0.022	6.23E-01
2.22327404	0.030	0.017	7.24E-02	0.016	0.025	5.16E-01
5.753798	0.020	0.011	6.32E-02	-0.009	0.016	5.73E-01
1.178650844	0.016	0.008	2.95E-02	-0.005	0.011	6.49E-01
2.101485442	-0.009	0.006	1.35E-01	0.008	0.009	3.99E-01
5.524351	0.016	0.008	4.85E-02	-0.007	0.012	5.58E-01
1.096897724	0.014	0.007	5.96E-02	0.003	0.011	7.64E-01
5.402898	0.018	0.010	7.47E-02	0.009	0.015	5.54E-01
1.484468069	-0.008	0.004	6.02E-02	-0.004	0.007	5.53E-01
0.862067776	0.022	0.010	3.55E-02	0.008	0.015	5.95E-01

6.260802	0.016	0.009	7.64E-02	-0.005	0.013	7.20E-01
5.221561	0.007	0.005	1.91E-01	0.004	0.007	6.14E-01
7.068576	-0.019	0.010	5.46E-02	-0.011	0.015	4.69E-01
3.502727	0.013	0.008	1.15E-01	0.037	0.013	3.46E-03
2.497803034	-0.027	0.014	4.83E-02	-0.004	0.020	8.33E-01
8.842585	0.015	0.008	4.97E-02	-0.001	0.012	9.36E-01
3.557229	-0.026	0.013	4.57E-02	-0.017	0.020	3.86E-01
0.989912161	0.012	0.007	7.81E-02	0.009	0.010	3.62E-01
8.09806	0.014	0.008	8.40E-02	0.002	0.012	8.66E-01
3.272607	-0.019	0.010	6.81E-02	0.004	0.015	7.79E-01
6.722051	-0.017	0.011	1.20E-01	-0.009	0.016	5.60E-01
2.196023	0.025	0.012	3.84E-02	-0.013	0.018	4.56E-01
3.441833	0.012	0.007	8.99E-02	0.025	0.011	2.18E-02
6.960918	-0.020	0.009	2.46E-02	-0.013	0.014	3.27E-01
0.899075361	0.020	0.009	2.57E-02	-0.007	0.013	6.04E-01
3.012545	-0.009	0.005	5.90E-02	-0.006	0.007	4.13E-01
3.889625	0.010	0.006	9.95E-02	0.029	0.009	1.73E-03
2.200396623	0.030	0.014	3.40E-02	-0.009	0.021	6.60E-01
4.251626	0.036	0.017	3.29E-02	0.012	0.025	6.17E-01
5.223243	0.007	0.005	1.82E-01	0.009	0.008	2.70E-01
3.487924	0.015	0.009	1.09E-01	0.039	0.014	5.30E-03
3.501381	0.013	0.008	1.22E-01	0.034	0.012	5.37E-03
1.995845608	0.022	0.010	2.90E-02	0.001	0.015	9.36E-01
8.858061	0.012	0.007	8.62E-02	-0.005	0.011	6.24E-01
7.683239	0.014	0.008	8.43E-02	0.002	0.012	8.55E-01
2.130755077	-0.022	0.012	5.86E-02	-0.022	0.018	2.05E-01
6.608336	-0.019	0.009	3.71E-02	-0.009	0.014	5.22E-01
1.877421336	-0.010	0.006	9.22E-02	-0.008	0.009	3.54E-01
2.64011402	-0.021	0.010	4.31E-02	0.003	0.015	8.30E-01
1.54939956	0.037	0.018	3.80E-02	-0.002	0.026	9.27E-01
1.797350379	-0.016	0.010	1.16E-01	0.012	0.015	4.31E-01
3.069738	-0.010	0.006	8.10E-02	-0.007	0.009	4.02E-01
2.200060191	0.030	0.014	3.50E-02	-0.010	0.021	6.35E-01
8.262575	-0.015	0.008	5.78E-02	-0.016	0.012	1.94E-01
5.222234	0.007	0.005	1.88E-01	0.006	0.007	4.18E-01
8.845613	0.019	0.009	4.26E-02	0.008	0.014	5.89E-01
6.605309	0.019	0.009	4.62E-02	0.011	0.014	4.49E-01
6.52221	0.018	0.009	4.62E-02	0.028	0.013	3.87E-02
6.917854	-0.016	0.009	6.94E-02	-0.017	0.013	2.03E-01
2.198041595	0.027	0.013	3.60E-02	-0.008	0.019	6.61E-01
1.140297529	0.014	0.007	3.17E-02	-0.004	0.010	6.63E-01
2.19736873	0.026	0.013	3.77E-02	-0.014	0.019	4.52E-01
2.488382921	-0.031	0.014	2.40E-02	0.004	0.020	8.36E-01

3.061328	-0.013	0.006	4.48E-02	-0.003	0.010	7.15E-01
5.39146	0.014	0.007	3.99E-02	-0.002	0.010	8.83E-01
5.155621	0.016	0.008	4.60E-02	-0.009	0.012	4.63E-01
3.669261	-0.008	0.004	8.24E-02	0.003	0.006	6.98E-01
3.030712	-0.008	0.005	8.73E-02	0.000	0.007	9.60E-01
1.046096403	0.010	0.006	1.00E-01	-0.003	0.009	7.52E-01
3.706605	0.013	0.006	4.16E-02	0.024	0.009	1.04E-02
0.58215586	-0.016	0.010	1.14E-01	0.001	0.015	9.70E-01
0.700580132	0.027	0.013	3.46E-02	0.005	0.019	8.04E-01
1.549735992	0.037	0.018	3.80E-02	-0.002	0.027	9.31E-01
2.054384879	0.014	0.006	2.71E-02	0.007	0.009	4.37E-01
2.885037	-0.014	0.007	5.58E-02	-0.012	0.011	2.86E-01
2.500158062	-0.019	0.011	7.65E-02	0.001	0.016	9.48E-01
0.86913286	0.030	0.014	3.42E-02	0.013	0.021	5.49E-01
1.879103499	-0.009	0.006	1.24E-01	-0.001	0.009	8.76E-01
8.353749	0.018	0.009	3.32E-02	-0.005	0.013	6.94E-01
5.755816	0.013	0.008	1.15E-01	0.001	0.012	9.50E-01
5.495081	0.011	0.007	1.07E-01	0.005	0.010	6.25E-01
5.029122	0.014	0.008	7.56E-02	-0.003	0.012	7.88E-01
5.433514	0.017	0.011	1.27E-01	0.016	0.017	3.50E-01
2.337324688	0.018	0.007	1.28E-02	0.008	0.011	4.62E-01
6.525574	0.017	0.009	7.15E-02	-0.011	0.014	4.21E-01
5.335276	0.036	0.018	4.95E-02	-0.011	0.027	6.87E-01
2.417732077	-0.015	0.011	1.66E-01	0.008	0.016	6.25E-01
1.16384781	0.010	0.004	2.04E-02	0.001	0.006	8.34E-01
8.636352	0.011	0.007	8.67E-02	0.004	0.010	6.65E-01
3.846561	0.010	0.006	9.75E-02	0.020	0.009	2.88E-02
3.655804	-0.010	0.005	4.11E-02	0.006	0.007	3.66E-01
0.917242721	-0.009	0.005	6.43E-02	-0.025	0.007	3.97E-04
1.845796672	-0.016	0.009	7.46E-02	-0.013	0.013	3.39E-01
8.615157	0.014	0.008	8.59E-02	-0.001	0.013	9.55E-01
4.247253	0.038	0.018	3.84E-02	0.014	0.027	6.04E-01
7.798635	-0.017	0.009	5.69E-02	-0.007	0.013	5.81E-01
5.809982	0.012	0.008	1.19E-01	0.001	0.012	9.11E-01
5.738658	0.015	0.009	9.94E-02	-0.003	0.013	8.44E-01
1.550072425	0.038	0.018	3.78E-02	-0.002	0.027	9.31E-01
7.057474	-0.018	0.010	7.87E-02	-0.039	0.015	1.08E-02
7.660361	0.019	0.009	3.76E-02	0.001	0.014	9.32E-01
1.331054808	0.026	0.011	2.11E-02	0.013	0.017	4.61E-01
0.865095669	0.025	0.012	3.33E-02	0.010	0.017	5.78E-01
1.821237093	-0.020	0.010	4.11E-02	0.003	0.015	8.50E-01
7.181954	0.019	0.009	3.00E-02	-0.003	0.013	7.89E-01
5.105492	0.015	0.008	7.66E-02	-0.001	0.012	9.35E-01

3.585826	-0.008	0.006	2.13E-01	0.004	0.009	6.93E-01
1.487495963	-0.008	0.004	5.82E-02	-0.009	0.007	1.66E-01
3.041478	-0.010	0.006	9.26E-02	0.003	0.009	7.55E-01
7.715536	-0.017	0.009	5.64E-02	-0.009	0.014	5.06E-01
5.338303	0.036	0.019	5.38E-02	-0.003	0.028	9.24E-01
3.48927	0.016	0.010	1.12E-01	0.039	0.015	8.14E-03
1.782883778	-0.015	0.008	7.56E-02	0.008	0.012	4.92E-01
1.931586983	0.010	0.005	3.77E-02	0.008	0.007	2.28E-01
6.565609	0.010	0.007	1.24E-01	-0.002	0.010	8.81E-01
3.346622	-0.018	0.012	1.23E-01	-0.012	0.017	5.01E-01
7.745479	-0.020	0.010	4.96E-02	-0.025	0.015	1.10E-01
6.541723	0.013	0.008	7.80E-02	0.009	0.011	4.48E-01
5.335948	0.037	0.019	4.96E-02	-0.009	0.028	7.53E-01
7.948011	0.011	0.007	1.09E-01	0.017	0.010	7.97E-02
1.12482163	0.013	0.007	5.27E-02	-0.006	0.010	5.59E-01
3.386658	0.014	0.009	1.35E-01	0.033	0.014	1.78E-02
7.639839	-0.014	0.009	1.33E-01	0.026	0.014	5.95E-02
0.757773673	0.010	0.005	6.58E-02	-0.002	0.008	7.73E-01
5.222907	0.007	0.005	1.81E-01	0.006	0.008	4.19E-01
3.816955	0.011	0.006	1.02E-01	0.032	0.010	9.55E-04
5.842952	0.016	0.009	5.49E-02	0.004	0.013	7.60E-01
7.570197	-0.020	0.010	3.83E-02	-0.019	0.015	2.00E-01
1.129531686	0.017	0.007	2.40E-02	0.002	0.011	8.60E-01
7.181281	0.017	0.008	4.11E-02	-0.002	0.012	9.01E-01
8.695901	-0.017	0.009	6.51E-02	-0.003	0.014	8.36E-01
6.804813	0.015	0.009	1.08E-01	-0.002	0.014	9.00E-01
2.322185221	-0.018	0.009	6.12E-02	-0.003	0.014	8.07E-01
6.885893	0.019	0.009	2.81E-02	-0.002	0.013	8.57E-01
1.959510888	0.010	0.005	4.80E-02	0.002	0.007	8.01E-01
5.157639	0.018	0.009	4.91E-02	-0.007	0.013	6.08E-01
2.102831172	-0.009	0.006	1.11E-01	0.012	0.008	1.44E-01
2.113597015	-0.011	0.007	1.10E-01	-0.012	0.010	2.37E-01
7.721928	-0.021	0.011	6.04E-02	-0.048	0.016	3.57E-03
2.866197	-0.013	0.008	1.36E-01	-0.007	0.013	5.75E-01
2.313101541	-0.010	0.005	7.28E-02	0.000	0.008	9.74E-01
8.23196	0.016	0.009	7.09E-02	0.005	0.014	7.03E-01
2.640450453	-0.027	0.012	1.78E-02	0.003	0.017	8.64E-01
6.26753	0.022	0.009	1.62E-02	0.027	0.013	4.37E-02
4.251963	0.036	0.017	3.30E-02	0.009	0.025	7.33E-01
1.810134818	-0.016	0.009	9.04E-02	0.007	0.014	6.15E-01
5.915622	0.012	0.008	1.12E-01	0.004	0.011	7.16E-01
3.808208	0.011	0.006	9.01E-02	0.020	0.009	3.52E-02
3.594573	-0.011	0.006	7.72E-02	0.004	0.009	6.61E-01

7.298696	-0.019	0.010	4.66E-02	0.013	0.014	3.48E-01
2.681495229	-0.012	0.005	2.02E-02	0.003	0.008	7.43E-01
8.404214	0.014	0.009	9.61E-02	-0.002	0.013	8.85E-01
3.072766	-0.012	0.007	6.29E-02	-0.013	0.010	1.90E-01
7.381459	-0.015	0.008	7.80E-02	-0.007	0.013	5.87E-01
1.958165158	0.010	0.005	5.07E-02	0.002	0.007	7.95E-01
1.111700759	0.012	0.007	6.88E-02	-0.001	0.010	8.92E-01
5.336285	0.037	0.019	4.96E-02	-0.008	0.028	7.84E-01
3.04316	-0.007	0.005	1.67E-01	-0.001	0.008	9.27E-01
3.503736	0.014	0.009	1.20E-01	0.036	0.013	7.81E-03
0.737251285	-0.013	0.007	5.33E-02	-0.009	0.010	3.52E-01
8.434829	0.014	0.008	8.74E-02	-0.012	0.012	3.02E-01
2.496457304	-0.026	0.014	6.27E-02	0.011	0.021	5.90E-01
1.995172743	0.021	0.010	3.02E-02	0.001	0.014	9.55E-01
6.918191	-0.017	0.009	5.45E-02	-0.010	0.013	4.41E-01
1.956146562	0.010	0.005	4.25E-02	0.005	0.008	5.01E-01
5.449663	0.016	0.008	4.92E-02	0.010	0.012	4.11E-01
6.384945	-0.018	0.009	4.49E-02	-0.017	0.013	2.01E-01
2.687887448	-0.013	0.005	1.91E-02	-0.004	0.008	6.08E-01
5.339649	0.038	0.019	4.37E-02	-0.001	0.028	9.71E-01
6.905743	0.015	0.008	4.81E-02	0.007	0.012	5.47E-01
2.049001957	0.014	0.007	3.71E-02	0.010	0.010	3.46E-01
3.345613	-0.016	0.009	8.25E-02	0.000	0.014	9.72E-01
7.978963	0.015	0.008	7.81E-02	0.013	0.013	2.85E-01
1.263095425	0.032	0.015	3.81E-02	0.001	0.023	9.59E-01
3.728137	0.012	0.008	1.38E-01	0.026	0.012	2.71E-02
8.021353	0.012	0.007	7.04E-02	0.003	0.010	7.48E-01
1.655375826	0.006	0.004	1.40E-01	0.008	0.006	1.98E-01
3.346286	-0.017	0.011	1.17E-01	-0.008	0.016	6.12E-01
3.863383	-0.010	0.005	4.59E-02	0.003	0.007	6.42E-01
4.245907	0.037	0.018	3.99E-02	0.007	0.027	7.96E-01
8.13103	0.015	0.008	7.17E-02	0.005	0.012	7.01E-01
5.337294	0.037	0.019	5.00E-02	-0.007	0.028	8.04E-01
4.053467	0.038	0.018	3.59E-02	0.005	0.027	8.44E-01
2.563743822	-0.016	0.009	9.49E-02	-0.009	0.014	5.31E-01
2.509241742	-0.024	0.013	6.90E-02	-0.020	0.020	3.11E-01
5.337631	0.037	0.019	5.06E-02	-0.004	0.028	8.85E-01
7.049736	-0.015	0.009	7.37E-02	-0.018	0.013	1.68E-01
0.935073648	-0.009	0.005	8.34E-02	-0.019	0.007	9.96E-03
8.94587	0.013	0.009	1.55E-01	0.005	0.013	6.84E-01
2.156996819	-0.017	0.010	9.73E-02	-0.008	0.015	5.92E-01
1.797013947	-0.016	0.010	1.18E-01	0.008	0.015	6.08E-01
5.334939	0.037	0.019	5.21E-02	-0.012	0.028	6.73E-01

1.164857108	0.010	0.005	2.65E-02	0.002	0.007	8.13E-01
2.883691	-0.016	0.008	5.40E-02	-0.007	0.012	5.91E-01
2.054048446	0.013	0.006	3.02E-02	0.007	0.009	4.57E-01
5.390787	0.022	0.011	4.27E-02	-0.003	0.016	8.50E-01
1.114392219	0.015	0.007	4.47E-02	-0.003	0.011	7.50E-01
6.124883	0.013	0.009	1.20E-01	-0.002	0.013	9.05E-01
7.18229	0.018	0.008	3.61E-02	-0.006	0.013	6.59E-01
2.711437729	-0.014	0.006	1.37E-02	-0.016	0.008	6.55E-02
1.064936628	0.012	0.007	6.10E-02	-0.001	0.010	9.10E-01
1.933605579	0.009	0.005	6.01E-02	0.006	0.007	4.50E-01
1.785238806	-0.015	0.010	1.20E-01	0.015	0.015	3.26E-01
2.157333252	-0.019	0.010	7.26E-02	-0.008	0.015	6.12E-01
1.806097627	-0.020	0.011	6.15E-02	0.012	0.016	4.42E-01
1.645955713	0.011	0.005	4.30E-02	0.012	0.008	1.45E-01
1.155100563	0.011	0.005	3.72E-02	0.000	0.008	9.95E-01
5.339986	0.039	0.019	4.39E-02	0.004	0.029	9.01E-01
0.66660044	-0.021	0.010	3.46E-02	-0.014	0.015	3.57E-01
3.624852	-0.012	0.007	7.32E-02	-0.003	0.010	7.74E-01
3.502391	0.013	0.008	1.10E-01	0.037	0.012	2.75E-03
4.158771	-0.027	0.015	8.18E-02	0.000	0.023	9.97E-01
7.749852	-0.018	0.011	8.64E-02	0.001	0.016	9.38E-01
1.549063127	0.036	0.018	4.07E-02	-0.006	0.026	8.27E-01
4.276186	0.032	0.015	3.40E-02	0.013	0.023	5.56E-01
3.622161	-0.010	0.005	6.85E-02	-0.006	0.008	4.69E-01
2.016704429	0.021	0.010	3.02E-02	0.004	0.015	7.84E-01
6.917182	-0.015	0.008	6.34E-02	-0.008	0.012	5.27E-01
3.923268	0.018	0.011	8.56E-02	0.045	0.016	5.28E-03
7.948684	-0.020	0.009	2.50E-02	-0.002	0.013	8.58E-01
6.641307	0.016	0.009	7.43E-02	0.003	0.013	8.34E-01
2.886046	-0.013	0.007	6.87E-02	-0.005	0.010	6.36E-01
5.392133	0.014	0.007	5.88E-02	-0.007	0.011	5.11E-01
7.285239	-0.021	0.010	2.87E-02	0.007	0.015	6.28E-01
4.24557	0.036	0.018	4.18E-02	0.014	0.027	6.00E-01
5.334603	0.037	0.019	5.17E-02	-0.010	0.028	7.18E-01
5.412991	0.017	0.008	4.37E-02	0.005	0.013	6.83E-01
3.740921	0.012	0.008	1.34E-01	0.033	0.012	8.35E-03
8.013952	-0.017	0.009	6.73E-02	-0.012	0.014	3.95E-01
1.438713237	0.008	0.004	5.36E-02	0.003	0.006	6.42E-01
4.27955	0.027	0.013	3.71E-02	0.021	0.020	2.76E-01
7.228382	-0.019	0.010	7.00E-02	0.021	0.015	1.73E-01
0.97813702	0.012	0.006	6.95E-02	0.009	0.010	3.64E-01
1.326008319	0.026	0.012	2.16E-02	0.011	0.017	5.24E-01
6.236578	0.015	0.008	6.33E-02	0.013	0.012	2.76E-01

6.38259	-0.015	0.009	1.13E-01	-0.004	0.014	7.53E-01
0.865432102	0.025	0.012	3.52E-02	0.009	0.018	5.93E-01
3.696849	0.010	0.006	8.70E-02	0.025	0.009	5.82E-03
5.460092	0.015	0.008	6.91E-02	0.003	0.012	8.15E-01
7.695014	-0.017	0.010	9.22E-02	-0.010	0.015	5.06E-01
5.447644	0.017	0.008	3.62E-02	0.014	0.012	2.54E-01
2.763584781	-0.020	0.010	3.69E-02	-0.044	0.014	2.22E-03
1.15476413	0.012	0.005	3.50E-02	-0.004	0.008	6.00E-01
1.95850159	0.009	0.005	5.37E-02	0.000	0.007	9.72E-01
8.725507	0.013	0.007	7.25E-02	-0.002	0.011	8.21E-01
6.429691	0.012	0.007	8.72E-02	-0.002	0.011	8.69E-01
2.653234891	-0.022	0.012	5.36E-02	0.011	0.017	5.29E-01
8.216821	0.014	0.007	5.17E-02	0.003	0.011	8.06E-01
7.998812	0.014	0.008	6.56E-02	0.006	0.011	6.01E-01
1.961529484	0.009	0.005	6.01E-02	0.002	0.007	8.10E-01
7.034933	-0.025	0.012	3.45E-02	-0.016	0.018	3.68E-01
0.739942745	-0.013	0.006	5.29E-02	0.009	0.010	3.63E-01
1.1534184	0.012	0.006	3.59E-02	-0.004	0.008	6.14E-01
6.201926	0.013	0.008	9.23E-02	-0.010	0.012	3.96E-01
3.10641	-0.018	0.009	4.74E-02	-0.021	0.013	1.16E-01
6.073745	0.017	0.009	4.61E-02	-0.003	0.013	8.05E-01
7.361609	-0.019	0.009	3.68E-02	-0.009	0.014	4.90E-01
3.499026	0.012	0.008	1.41E-01	0.032	0.012	9.52E-03
2.41806851	-0.015	0.011	1.94E-01	0.008	0.017	6.28E-01
6.886903	0.017	0.009	4.25E-02	-0.010	0.013	4.13E-01
7.85852	0.012	0.009	1.49E-01	0.005	0.013	7.21E-01
5.335612	0.037	0.019	5.23E-02	-0.012	0.028	6.77E-01
2.86115	-0.014	0.008	7.74E-02	-0.013	0.012	2.82E-01
7.696023	-0.017	0.010	8.14E-02	-0.019	0.015	2.00E-01
1.090841938	0.014	0.007	4.60E-02	-0.007	0.011	5.17E-01
5.812673	0.015	0.010	1.36E-01	-0.003	0.015	8.64E-01
1.620386836	0.014	0.007	3.14E-02	0.007	0.010	4.92E-01
3.591545	-0.009	0.006	1.33E-01	0.004	0.009	6.28E-01
1.164184243	0.010	0.004	2.52E-02	0.001	0.006	9.25E-01
0.861731343	0.021	0.010	4.06E-02	0.007	0.015	6.30E-01
3.253767	-0.015	0.008	6.91E-02	0.003	0.012	7.84E-01
7.045362	-0.028	0.014	3.81E-02	-0.017	0.020	3.89E-01
8.534077	-0.014	0.008	6.08E-02	-0.024	0.011	3.17E-02
3.262178	0.014	0.008	7.87E-02	0.027	0.012	2.11E-02
3.585153	-0.007	0.007	3.17E-01	0.003	0.010	7.30E-01
6.222785	0.016	0.009	8.68E-02	-0.011	0.014	4.32E-01
1.176632249	0.017	0.008	2.75E-02	-0.004	0.011	7.23E-01
3.013218	-0.009	0.005	6.85E-02	-0.006	0.007	4.41E-01

8.420026	0.013	0.008	1.08E-01	0.011	0.012	3.75E-01
1.433666748	0.010	0.004	2.40E-02	0.005	0.006	4.69E-01
2.769640568	-0.020	0.010	4.79E-02	-0.047	0.015	1.67E-03
3.085551	-0.012	0.007	7.29E-02	0.006	0.010	5.67E-01
1.547380964	0.034	0.017	4.25E-02	-0.005	0.025	8.50E-01
5.840597	-0.017	0.009	6.45E-02	-0.012	0.014	3.83E-01
7.298023	-0.018	0.010	5.66E-02	-0.005	0.014	7.39E-01
8.24138	-0.012	0.008	1.10E-01	-0.004	0.012	7.53E-01
8.26123	-0.020	0.010	5.51E-02	-0.014	0.015	3.58E-01
2.195686567	0.024	0.012	4.15E-02	-0.016	0.018	3.66E-01
1.167885001	0.010	0.004	2.03E-02	0.002	0.007	8.10E-01
3.816619	0.011	0.007	1.10E-01	0.033	0.010	9.85E-04
6.720032	-0.017	0.011	1.11E-01	-0.012	0.016	4.48E-01
5.43957	-0.013	0.008	1.04E-01	-0.013	0.012	2.79E-01
8.28949	-0.012	0.008	1.38E-01	-0.026	0.012	3.03E-02
6.216392	0.018	0.009	5.18E-02	-0.015	0.014	2.72E-01
1.216667727	0.012	0.007	7.69E-02	0.008	0.010	4.49E-01
5.337967	0.036	0.019	5.49E-02	0.000	0.028	9.92E-01
2.68687815	-0.013	0.006	2.04E-02	-0.003	0.008	7.47E-01
8.153908	0.013	0.009	1.29E-01	0.002	0.013	8.87E-01
2.391153903	-0.013	0.007	7.29E-02	-0.001	0.011	9.05E-01
7.181618	0.018	0.009	3.87E-02	-0.006	0.013	6.52E-01
7.513677	0.017	0.009	6.89E-02	-0.002	0.014	9.10E-01
0.554568388	0.013	0.007	8.30E-02	0.013	0.011	2.41E-01
5.806618	0.015	0.008	7.04E-02	-0.014	0.012	2.56E-01
0.575090776	-0.014	0.008	8.41E-02	-0.002	0.012	8.42E-01
3.058636	-0.011	0.006	5.91E-02	-0.009	0.009	3.11E-01
7.494163	-0.015	0.008	7.80E-02	-0.001	0.013	9.15E-01
3.141062	-0.025	0.012	4.28E-02	-0.020	0.018	2.73E-01
7.739086	-0.016	0.011	1.41E-01	-0.007	0.017	6.78E-01
2.433544409	-0.016	0.009	9.05E-02	-0.033	0.014	1.84E-02
3.346959	-0.019	0.012	1.26E-01	-0.014	0.018	4.50E-01
8.894396	0.014	0.009	1.26E-01	0.007	0.014	6.33E-01
4.086101	0.026	0.012	2.46E-02	0.021	0.017	2.24E-01
3.384303	0.013	0.009	1.66E-01	0.020	0.014	1.47E-01
3.510465	0.013	0.008	9.50E-02	0.028	0.012	1.79E-02
5.019029	0.018	0.011	9.40E-02	-0.006	0.016	7.24E-01
8.203363	0.015	0.009	7.47E-02	-0.015	0.013	2.36E-01
0.635985075	0.014	0.010	1.51E-01	0.010	0.015	4.92E-01
3.055608	-0.011	0.007	1.10E-01	0.002	0.010	8.51E-01
5.105156	0.019	0.010	6.20E-02	0.007	0.015	6.60E-01
1.014808172	0.024	0.011	3.52E-02	0.013	0.017	4.36E-01
5.147883	0.016	0.009	7.17E-02	-0.008	0.013	5.69E-01

8.315395	0.014	0.009	1.12E-01	-0.015	0.013	2.49E-01
7.337722	-0.016	0.009	7.10E-02	0.000	0.013	9.81E-01
7.182963	0.016	0.008	5.42E-02	-0.009	0.012	4.56E-01
3.061664	-0.012	0.006	6.01E-02	-0.002	0.010	8.36E-01
0.868796428	0.029	0.014	3.82E-02	0.012	0.021	5.72E-01
6.802458	0.013	0.009	1.42E-01	-0.001	0.013	9.50E-01
7.677519	0.013	0.008	1.15E-01	-0.003	0.012	8.21E-01
7.314508	-0.015	0.007	3.45E-02	0.003	0.011	7.69E-01
3.291448	-0.027	0.014	5.77E-02	-0.026	0.022	2.24E-01
2.320503058	-0.029	0.015	4.56E-02	0.003	0.022	8.80E-01
5.418038	0.011	0.006	6.67E-02	-0.015	0.009	1.14E-01
7.353198	-0.019	0.010	5.62E-02	-0.017	0.015	2.65E-01
8.969084	-0.018	0.010	6.17E-02	0.023	0.014	1.20E-01
5.167732	0.023	0.012	5.11E-02	-0.009	0.018	6.07E-01
2.312765109	-0.010	0.006	7.90E-02	0.002	0.009	8.38E-01
8.40455	0.014	0.010	1.57E-01	-0.016	0.015	2.65E-01
1.99483631	0.020	0.009	3.29E-02	0.000	0.014	9.99E-01
6.922228	-0.016	0.008	4.16E-02	-0.005	0.012	6.44E-01
5.164368	0.017	0.009	5.55E-02	-0.007	0.013	5.82E-01
0.669291901	-0.017	0.009	6.24E-02	-0.028	0.014	4.15E-02
2.763921214	-0.020	0.010	3.87E-02	-0.043	0.014	2.43E-03
6.490921	0.014	0.009	1.02E-01	-0.014	0.013	2.76E-01
2.384761683	-0.016	0.008	4.21E-02	0.006	0.012	6.24E-01
7.822858	-0.021	0.010	2.71E-02	-0.005	0.014	7.41E-01
5.89409	0.015	0.009	7.18E-02	-0.018	0.013	1.51E-01
2.319830193	-0.020	0.010	4.64E-02	0.005	0.015	7.42E-01
8.910208	0.016	0.010	9.93E-02	-0.003	0.015	8.60E-01
5.811328	0.014	0.010	1.60E-01	-0.015	0.015	3.01E-01
6.24869	0.015	0.008	5.15E-02	-0.002	0.012	8.83E-01
8.924338	-0.015	0.009	1.26E-01	0.005	0.014	7.47E-01
8.709358	0.011	0.008	1.58E-01	-0.017	0.012	1.42E-01
5.091025	0.017	0.009	6.16E-02	0.000	0.014	1.00E+00
4.25129	0.035	0.016	3.50E-02	0.012	0.024	6.16E-01
2.561388794	-0.017	0.009	4.56E-02	0.001	0.013	9.22E-01
1.191771715	0.009	0.005	6.04E-02	0.002	0.007	7.28E-01
8.037166	0.015	0.010	1.14E-01	0.023	0.014	1.01E-01
7.643203	-0.013	0.010	1.79E-01	-0.015	0.014	3.02E-01
1.167212136	0.010	0.005	2.81E-02	0.000	0.007	9.95E-01
2.390481037	-0.014	0.008	7.46E-02	0.003	0.012	8.09E-01
3.231226	0.012	0.007	7.14E-02	0.030	0.010	2.55E-03
3.158557	-0.014	0.007	5.05E-02	-0.013	0.011	2.20E-01
1.876075606	-0.011	0.006	7.97E-02	-0.008	0.010	3.89E-01
2.101821874	-0.010	0.006	1.10E-01	0.012	0.009	1.97E-01

6.64669	-0.015	0.009	9.25E-02	0.013	0.013	2.97E-01
3.228535	0.010	0.007	1.48E-01	0.024	0.010	1.85E-02
3.056281	-0.009	0.006	1.27E-01	-0.003	0.009	7.26E-01
5.454373	0.015	0.008	7.81E-02	-0.017	0.013	1.80E-01
0.918924884	-0.007	0.004	8.36E-02	-0.020	0.006	1.75E-03
8.486976	0.010	0.006	8.81E-02	0.004	0.009	6.12E-01
6.669904	0.018	0.009	4.38E-02	0.014	0.013	3.06E-01
3.483214	0.014	0.009	1.29E-01	0.030	0.014	2.96E-02
5.852372	0.017	0.009	4.57E-02	0.013	0.013	2.91E-01
1.09117837	0.013	0.007	6.40E-02	-0.002	0.010	8.65E-01
0.865768534	0.025	0.012	3.75E-02	0.009	0.018	6.01E-01
3.06301	-0.011	0.007	1.20E-01	0.006	0.010	5.55E-01
3.058973	-0.012	0.006	3.69E-02	-0.010	0.009	2.51E-01
6.901706	0.015	0.008	7.03E-02	0.006	0.012	6.20E-01
0.989239295	0.012	0.007	6.96E-02	0.008	0.010	4.19E-01
2.200733056	0.030	0.014	4.05E-02	-0.011	0.022	6.13E-01
5.899473	0.015	0.009	7.77E-02	0.004	0.013	7.61E-01
2.255908001	0.016	0.008	3.40E-02	0.007	0.011	5.23E-01
6.327752	0.012	0.007	9.29E-02	-0.004	0.011	7.00E-01
1.874729875	-0.014	0.007	5.43E-02	0.008	0.011	4.43E-01
7.744133	-0.018	0.011	8.71E-02	-0.035	0.016	2.98E-02
8.046249	0.016	0.009	6.98E-02	0.007	0.013	5.69E-01
1.547044531	0.033	0.016	4.43E-02	-0.005	0.024	8.36E-01
5.840934	-0.014	0.009	1.24E-01	-0.021	0.014	1.20E-01
5.949265	0.018	0.009	3.57E-02	-0.007	0.013	5.86E-01
8.052978	0.015	0.009	1.03E-01	-0.004	0.014	7.86E-01
5.339313	0.037	0.019	5.11E-02	-0.005	0.028	8.69E-01
2.524381209	-0.021	0.012	6.26E-02	-0.006	0.017	7.10E-01
1.80138757	-0.018	0.010	6.57E-02	0.015	0.014	2.94E-01
2.637758992	-0.017	0.009	6.53E-02	-0.004	0.014	7.66E-01
2.567108148	-0.015	0.008	6.16E-02	-0.019	0.012	9.74E-02
7.990738	-0.014	0.009	1.08E-01	-0.001	0.013	9.10E-01
5.157976	0.016	0.009	5.95E-02	-0.008	0.013	5.19E-01
7.874669	0.017	0.009	5.12E-02	0.015	0.013	2.53E-01
6.382254	-0.013	0.008	9.45E-02	-0.001	0.012	9.64E-01
3.488597	0.015	0.010	1.29E-01	0.040	0.014	5.95E-03
3.121213	-0.017	0.008	4.10E-02	-0.001	0.012	9.04E-01
7.769702	-0.023	0.011	3.79E-02	-0.018	0.017	2.78E-01
3.488261	0.015	0.009	1.25E-01	0.039	0.014	5.57E-03
5.33864	0.036	0.019	5.56E-02	-0.006	0.028	8.28E-01
3.043497	-0.006	0.005	2.05E-01	0.000	0.008	9.72E-01
3.380602	0.018	0.012	1.34E-01	0.035	0.018	5.68E-02
2.837936384	-0.013	0.009	1.30E-01	-0.029	0.013	2.66E-02

3.488933	0.015	0.010	1.28E-01	0.040	0.015	6.34E-03
2.017040861	0.021	0.010	3.20E-02	0.004	0.015	7.76E-01
8.857388	0.015	0.008	6.74E-02	0.006	0.012	6.03E-01
1.847478835	-0.016	0.008	5.97E-02	-0.016	0.012	2.11E-01
5.967432	0.013	0.008	9.44E-02	0.005	0.011	6.66E-01
5.849681	0.016	0.010	1.03E-01	0.004	0.014	7.56E-01
6.54576	0.017	0.010	8.07E-02	-0.014	0.014	3.27E-01
6.029	0.018	0.009	4.64E-02	0.007	0.013	6.16E-01
6.227495	0.012	0.007	8.44E-02	-0.003	0.011	7.84E-01
8.827446	0.018	0.009	5.12E-02	0.002	0.014	8.90E-01
1.091514803	0.013	0.007	5.99E-02	0.003	0.011	7.43E-01
4.279886	0.027	0.013	3.58E-02	0.023	0.019	2.28E-01
1.546708099	0.032	0.016	4.58E-02	-0.008	0.024	7.56E-01
3.526277	0.014	0.007	5.57E-02	0.026	0.011	1.68E-02
1.163174945	0.009	0.004	2.52E-02	-0.001	0.006	8.40E-01
6.074081	0.017	0.009	6.14E-02	0.002	0.013	9.03E-01
2.998415	-0.007	0.004	5.72E-02	0.000	0.006	9.60E-01
5.925042	-0.017	0.010	8.03E-02	0.008	0.014	6.01E-01
2.261627355	0.012	0.006	5.24E-02	0.011	0.009	2.35E-01
7.640175	-0.013	0.009	1.76E-01	-0.008	0.014	5.69E-01
2.496120871	-0.024	0.014	8.13E-02	-0.005	0.021	8.10E-01
3.504746	0.015	0.010	1.24E-01	0.037	0.014	9.44E-03
0.703608025	0.021	0.011	4.97E-02	0.002	0.016	9.11E-01
3.379256	0.019	0.013	1.33E-01	0.025	0.019	1.86E-01
1.330718375	0.025	0.011	2.41E-02	0.012	0.016	4.78E-01
2.91969	-0.014	0.007	4.19E-02	-0.011	0.010	2.84E-01
7.162777	0.015	0.010	1.15E-01	0.004	0.015	7.64E-01
7.750862	-0.015	0.010	1.13E-01	0.001	0.014	9.61E-01
3.384639	0.013	0.009	1.61E-01	0.021	0.014	1.33E-01
6.45997	0.012	0.007	1.12E-01	0.000	0.011	9.75E-01
3.765817	0.021	0.010	3.51E-02	0.034	0.015	2.26E-02
7.229055	-0.021	0.010	4.09E-02	-0.003	0.016	8.51E-01
8.567383	0.012	0.008	1.45E-01	0.010	0.013	4.50E-01
6.217065	0.016	0.009	6.06E-02	-0.015	0.013	2.50E-01
7.314845	-0.014	0.007	4.72E-02	0.000	0.011	9.89E-01
0.776613898	0.008	0.005	1.08E-01	0.002	0.007	7.44E-01
3.117512	-0.018	0.009	4.34E-02	-0.017	0.014	2.03E-01
6.733153	-0.020	0.010	4.22E-02	-0.012	0.014	4.07E-01
5.627299	0.009	0.006	1.49E-01	-0.005	0.010	5.67E-01
8.38571	0.018	0.009	3.56E-02	-0.008	0.013	5.53E-01
2.195350135	0.024	0.012	4.46E-02	-0.014	0.018	4.17E-01
6.845521	0.015	0.009	8.18E-02	0.000	0.013	9.94E-01
5.383722	0.017	0.009	4.82E-02	-0.001	0.013	9.31E-01

1.08209469	0.010	0.006	1.14E-01	0.002	0.009	8.30E-01
8.727862	0.011	0.007	9.66E-02	0.005	0.010	6.09E-01
8.999699	0.010	0.007	1.47E-01	0.010	0.010	3.21E-01
5.217188	0.008	0.005	1.34E-01	0.005	0.008	5.21E-01
7.17388	-0.013	0.008	9.63E-02	-0.001	0.012	9.48E-01
3.482878	0.014	0.009	1.19E-01	0.030	0.014	2.83E-02
5.385404	0.019	0.010	4.93E-02	0.005	0.014	7.23E-01
5.501473	0.012	0.007	1.05E-01	0.000	0.011	9.88E-01
3.700886	0.011	0.007	9.38E-02	0.024	0.010	1.76E-02
5.878951	0.012	0.008	1.31E-01	-0.012	0.012	3.38E-01
2.14017519	-0.024	0.012	4.51E-02	0.032	0.018	7.29E-02
5.962722	0.015	0.009	1.03E-01	0.002	0.013	9.06E-01
7.314172	-0.016	0.008	2.96E-02	0.000	0.011	9.75E-01
4.245234	0.035	0.018	4.87E-02	0.011	0.026	6.71E-01
3.845216	0.010	0.006	9.99E-02	0.023	0.009	9.64E-03
8.439539	0.016	0.009	7.99E-02	-0.002	0.014	8.59E-01
8.402195	0.016	0.010	9.74E-02	0.006	0.014	6.79E-01
8.226914	0.011	0.007	1.13E-01	0.002	0.010	8.19E-01
7.586346	0.016	0.009	7.52E-02	0.021	0.013	1.21E-01
2.524717641	-0.023	0.012	5.27E-02	-0.009	0.018	6.25E-01
3.75808	0.014	0.009	9.63E-02	0.027	0.013	3.59E-02
2.018050159	0.020	0.009	3.43E-02	0.003	0.014	8.35E-01
8.449968	-0.015	0.009	1.08E-01	-0.010	0.014	4.58E-01
0.868459995	0.028	0.014	4.06E-02	0.011	0.021	5.88E-01
8.490677	0.012	0.006	6.50E-02	0.005	0.010	5.90E-01
7.851118	-0.018	0.012	1.23E-01	0.010	0.017	5.44E-01
3.582462	-0.008	0.006	1.80E-01	0.003	0.009	7.67E-01
1.961865916	0.009	0.005	6.49E-02	0.001	0.007	8.71E-01
5.892071	0.018	0.011	9.79E-02	0.018	0.016	2.79E-01
4.171555	-0.025	0.015	8.16E-02	-0.004	0.022	8.57E-01
6.068362	0.013	0.008	1.34E-01	0.019	0.012	1.30E-01
3.965322	-0.016	0.008	4.60E-02	0.006	0.012	6.27E-01
0.866104967	0.025	0.012	3.96E-02	0.009	0.018	6.06E-01
8.919628	-0.012	0.007	9.11E-02	-0.003	0.010	7.75E-01
3.012881	-0.009	0.005	7.46E-02	-0.006	0.007	4.00E-01
6.604972	0.017	0.009	5.67E-02	0.009	0.013	5.04E-01
0.588548079	-0.013	0.009	1.46E-01	0.016	0.013	2.18E-01
5.03114	0.016	0.010	1.03E-01	0.000	0.015	9.86E-01
1.21700416	0.012	0.007	8.46E-02	0.007	0.010	4.70E-01
0.917579153	-0.008	0.005	8.19E-02	-0.025	0.007	4.21E-04
0.69116002	0.030	0.016	6.38E-02	0.001	0.025	9.71E-01
2.764257646	-0.020	0.010	4.24E-02	-0.044	0.014	2.25E-03
2.682168094	-0.012	0.005	2.06E-02	0.002	0.008	7.96E-01

1.262758992	0.031	0.015	4.44E-02	0.000	0.023	9.95E-01
7.759945	-0.020	0.009	2.75E-02	-0.018	0.014	1.92E-01
5.161003	0.020	0.011	5.65E-02	-0.021	0.016	1.81E-01
3.383966	0.013	0.010	1.72E-01	0.018	0.014	1.97E-01
2.491747247	-0.035	0.017	4.27E-02	-0.015	0.026	5.62E-01
7.616961	-0.023	0.011	3.54E-02	0.005	0.016	7.53E-01
2.157669684	-0.019	0.010	6.82E-02	-0.003	0.015	8.65E-01
1.051815757	0.013	0.006	4.03E-02	-0.012	0.010	2.17E-01
1.104299241	0.015	0.008	4.08E-02	-0.003	0.011	8.04E-01
6.069708	0.019	0.010	5.78E-02	0.005	0.015	7.13E-01
6.485539	-0.017	0.009	7.47E-02	-0.031	0.014	2.88E-02
6.897332	0.016	0.008	5.83E-02	-0.007	0.012	5.56E-01
3.583471	-0.005	0.006	4.01E-01	0.005	0.009	6.17E-01
7.218625	-0.022	0.010	3.33E-02	0.001	0.016	9.72E-01
0.912869097	-0.009	0.005	7.71E-02	-0.026	0.007	3.82E-04
5.336621	0.036	0.019	5.72E-02	-0.008	0.028	7.67E-01
1.08983264	0.014	0.007	4.74E-02	-0.001	0.011	8.90E-01
1.994499878	0.020	0.009	3.48E-02	0.000	0.014	9.87E-01
8.598672	-0.011	0.007	1.23E-01	0.002	0.011	8.65E-01
2.017713726	0.020	0.009	3.43E-02	0.003	0.014	8.10E-01
5.74505	0.019	0.011	9.30E-02	0.027	0.017	1.06E-01
2.993032	-0.007	0.004	6.79E-02	0.003	0.006	5.61E-01
7.632437	-0.021	0.012	8.71E-02	-0.003	0.018	8.49E-01
6.416906	0.017	0.009	6.64E-02	-0.010	0.014	4.74E-01
2.156660387	-0.016	0.010	1.13E-01	-0.007	0.015	6.34E-01
3.842861	0.011	0.006	9.09E-02	0.020	0.009	3.32E-02
8.447613	0.016	0.010	9.62E-02	0.028	0.014	5.08E-02
8.390083	0.014	0.008	9.50E-02	0.006	0.012	6.17E-01
5.85641	0.019	0.011	8.09E-02	0.026	0.016	1.01E-01
1.056862246	0.009	0.006	1.08E-01	0.006	0.009	4.77E-01
3.059982	-0.013	0.006	3.72E-02	-0.004	0.009	6.78E-01
5.219879	0.007	0.005	1.80E-01	0.007	0.007	3.78E-01
1.412807928	0.010	0.005	2.85E-02	0.014	0.007	4.11E-02
5.814356	0.014	0.008	8.68E-02	0.012	0.012	3.32E-01
8.051632	0.013	0.008	9.95E-02	0.000	0.012	9.71E-01
1.42357377	0.011	0.005	2.60E-02	0.011	0.007	1.31E-01
8.669322	0.018	0.009	4.25E-02	-0.002	0.013	8.51E-01
2.766612674	-0.020	0.010	4.33E-02	-0.047	0.015	1.49E-03
4.246916	0.036	0.018	4.72E-02	0.013	0.027	6.42E-01
6.545424	0.017	0.010	7.51E-02	-0.008	0.014	5.89E-01
1.546371666	0.032	0.016	4.70E-02	-0.007	0.024	7.60E-01
7.761964	-0.017	0.009	7.38E-02	-0.030	0.014	3.07E-02
6.638279	0.016	0.010	1.05E-01	0.011	0.015	4.61E-01

5.722846	0.012	0.009	1.89E-01	-0.010	0.014	4.72E-01
5.408618	0.018	0.009	4.70E-02	-0.001	0.013	9.16E-01
1.933942011	0.009	0.005	6.52E-02	0.005	0.007	5.41E-01
5.781049	0.015	0.009	1.18E-01	-0.004	0.014	7.70E-01
2.396536824	-0.018	0.012	1.44E-01	-0.007	0.018	6.99E-01
5.382713	0.015	0.008	6.96E-02	-0.006	0.012	6.52E-01
0.8664414	0.025	0.012	4.09E-02	0.010	0.019	6.05E-01
5.592646	0.016	0.009	6.96E-02	0.002	0.013	8.69E-01
6.55518	0.020	0.009	2.54E-02	0.003	0.013	8.45E-01
1.547717397	0.033	0.017	4.64E-02	-0.007	0.025	7.87E-01
5.22358	0.007	0.005	2.12E-01	0.010	0.008	2.06E-01
3.070411	-0.010	0.006	8.21E-02	-0.009	0.009	3.11E-01
7.775758	-0.017	0.009	6.99E-02	-0.024	0.014	9.29E-02
5.626626	0.014	0.009	1.18E-01	0.001	0.013	9.65E-01
1.769090042	-0.019	0.010	7.13E-02	-0.015	0.016	3.32E-01
7.717891	-0.016	0.010	1.20E-01	-0.013	0.015	4.09E-01
1.077721067	0.014	0.008	7.26E-02	0.002	0.011	8.86E-01
1.806434059	-0.017	0.010	8.57E-02	0.014	0.015	3.61E-01
1.831666504	-0.020	0.010	4.13E-02	-0.008	0.015	6.03E-01
7.136199	0.020	0.010	4.85E-02	-0.015	0.015	3.14E-01
7.828577	-0.020	0.010	3.89E-02	0.008	0.014	5.70E-01
2.319493761	-0.015	0.008	7.43E-02	-0.004	0.012	7.58E-01
7.852128	-0.019	0.011	8.91E-02	0.017	0.017	2.98E-01
8.403541	0.016	0.009	8.18E-02	0.015	0.014	2.85E-01
3.024993	-0.009	0.005	7.64E-02	-0.010	0.008	1.90E-01
2.622283093	-0.020	0.013	1.11E-01	0.007	0.019	7.07E-01
2.390144605	-0.014	0.008	7.74E-02	0.003	0.012	8.32E-01
3.588854	-0.009	0.006	1.49E-01	0.005	0.009	5.71E-01
5.33393	0.036	0.019	5.99E-02	-0.014	0.029	6.22E-01
3.845552	0.010	0.006	1.12E-01	0.021	0.009	1.88E-02
8.698929	0.016	0.009	7.35E-02	0.006	0.014	6.65E-01
7.276155	-0.019	0.009	3.73E-02	-0.003	0.014	8.51E-01
3.863719	-0.009	0.005	5.40E-02	0.003	0.007	6.52E-01
1.317933937	0.022	0.009	1.97E-02	0.011	0.014	4.43E-01
8.138768	-0.020	0.010	4.07E-02	-0.009	0.015	5.51E-01
7.353535	-0.017	0.010	9.65E-02	-0.029	0.015	5.97E-02
3.504073	0.014	0.009	1.41E-01	0.035	0.014	1.12E-02
6.979085	-0.015	0.008	5.04E-02	-0.008	0.012	5.15E-01
1.548726694	0.034	0.017	4.80E-02	-0.006	0.026	8.27E-01
5.833532	0.014	0.008	9.74E-02	0.007	0.013	5.50E-01
4.150696	-0.037	0.021	7.43E-02	0.011	0.031	7.13E-01
2.763248348	-0.019	0.010	4.44E-02	-0.043	0.014	2.77E-03
3.732174	0.012	0.009	1.52E-01	0.023	0.013	7.56E-02

8.394457	0.015	0.009	8.89E-02	0.009	0.013	4.91E-01
1.956819427	0.010	0.005	5.41E-02	0.004	0.007	5.69E-01
1.154427698	0.011	0.006	4.54E-02	-0.008	0.008	3.56E-01
5.265634	-0.013	0.006	2.31E-02	-0.017	0.009	5.33E-02
5.338976	0.036	0.019	5.64E-02	-0.006	0.028	8.25E-01
3.055272	-0.011	0.007	1.27E-01	0.000	0.011	9.65E-01
8.829128	0.012	0.009	2.00E-01	0.000	0.014	9.77E-01
0.868123563	0.028	0.014	4.28E-02	0.011	0.020	6.00E-01
5.26597	-0.013	0.006	2.36E-02	-0.017	0.009	5.34E-02
5.812337	0.014	0.010	1.37E-01	-0.005	0.014	7.21E-01
7.998139	0.014	0.008	6.55E-02	0.006	0.011	5.79E-01
5.395497	0.011	0.007	9.07E-02	-0.006	0.010	5.16E-01
8.420699	0.016	0.009	6.85E-02	-0.003	0.013	7.96E-01
2.337661121	0.016	0.007	2.06E-02	0.006	0.011	5.55E-01
2.883018	-0.014	0.009	9.60E-02	0.006	0.013	6.46E-01
2.256580866	0.015	0.008	4.38E-02	0.011	0.011	3.53E-01
1.47807585	-0.010	0.006	9.77E-02	0.005	0.009	5.61E-01
0.861394911	0.020	0.010	4.88E-02	0.006	0.015	6.78E-01
3.901736	0.010	0.006	9.90E-02	0.025	0.009	6.39E-03
2.22461977	0.032	0.020	9.78E-02	0.012	0.028	6.67E-01
6.522546	0.013	0.008	1.14E-01	0.037	0.012	2.56E-03
2.633048936	-0.016	0.008	6.15E-02	-0.007	0.013	5.70E-01
6.23927	0.019	0.010	5.25E-02	0.026	0.015	8.02E-02
2.974864	-0.008	0.004	5.90E-02	-0.004	0.007	5.74E-01
3.267561	-0.014	0.008	9.59E-02	-0.013	0.012	2.96E-01
1.861945437	-0.014	0.008	7.56E-02	0.004	0.012	7.23E-01
2.017377294	0.020	0.010	3.53E-02	0.003	0.014	8.11E-01
1.101607781	0.015	0.008	5.13E-02	-0.008	0.012	4.73E-01
7.009364	-0.018	0.009	5.65E-02	-0.029	0.014	3.48E-02
3.254103	-0.015	0.009	8.43E-02	0.003	0.013	7.98E-01
2.432535111	-0.016	0.010	1.21E-01	-0.024	0.015	1.13E-01
7.51334	0.016	0.009	6.93E-02	0.005	0.014	7.17E-01
6.887239	0.016	0.008	5.46E-02	-0.006	0.012	6.34E-01
5.333593	0.036	0.019	6.18E-02	-0.013	0.029	6.59E-01
8.478229	0.019	0.010	5.73E-02	-0.003	0.015	8.46E-01
1.315242476	0.024	0.010	2.07E-02	0.009	0.015	5.76E-01
1.101944213	0.015	0.008	5.86E-02	-0.009	0.012	4.51E-01
7.251932	0.015	0.009	7.26E-02	0.023	0.013	7.42E-02
7.006673	-0.015	0.009	1.10E-01	-0.028	0.014	4.88E-02
8.992634	0.011	0.007	1.30E-01	0.010	0.010	3.57E-01
4.148005	-0.028	0.016	7.58E-02	0.011	0.024	6.41E-01
0.945166626	-0.011	0.005	5.28E-02	-0.002	0.008	7.85E-01
7.393234	0.012	0.007	9.00E-02	-0.024	0.010	1.75E-02

8.222876	0.014	0.009	1.18E-01	-0.014	0.014	2.90E-01
5.050654	0.012	0.007	8.18E-02	0.013	0.010	2.14E-01
2.336988255	0.018	0.007	1.87E-02	0.009	0.011	4.35E-01
8.484285	0.013	0.009	1.56E-01	-0.009	0.014	5.07E-01
0.663572547	-0.017	0.010	8.69E-02	-0.041	0.015	4.87E-03
3.267224	-0.013	0.008	1.00E-01	-0.015	0.012	2.12E-01
2.385434549	-0.019	0.009	3.52E-02	0.006	0.014	6.39E-01
3.952201	-0.013	0.008	1.17E-01	-0.001	0.013	9.56E-01
3.250403	-0.013	0.006	3.01E-02	0.003	0.009	6.99E-01
2.768967702	-0.020	0.010	4.98E-02	-0.046	0.015	2.18E-03
8.553253	-0.017	0.010	7.60E-02	-0.002	0.015	8.81E-01
1.410789332	0.011	0.005	3.81E-02	0.017	0.008	2.93E-02
5.43486	0.014	0.008	7.56E-02	0.002	0.012	8.37E-01
1.892560803	-0.009	0.006	1.24E-01	-0.016	0.009	6.89E-02
7.714527	-0.019	0.010	6.56E-02	-0.020	0.015	1.90E-01
7.104238	-0.017	0.010	7.65E-02	-0.002	0.014	8.70E-01
6.166937	0.010	0.006	1.11E-01	-0.003	0.009	7.28E-01
1.094879129	0.012	0.007	1.02E-01	0.001	0.011	8.96E-01
8.682443	0.015	0.009	7.24E-02	0.014	0.013	2.63E-01
5.916295	0.012	0.007	8.73E-02	0.009	0.010	3.67E-01
0.611425495	0.016	0.009	6.93E-02	0.029	0.013	2.10E-02
5.549247	0.013	0.008	8.37E-02	-0.008	0.012	5.14E-01
8.620203	0.012	0.008	1.17E-01	-0.001	0.012	9.49E-01
7.043007	-0.021	0.013	9.48E-02	0.004	0.019	8.49E-01
7.366319	-0.013	0.008	1.25E-01	-0.003	0.013	8.36E-01
2.39081747	-0.013	0.008	9.50E-02	0.002	0.011	8.84E-01
0.866777832	0.025	0.013	4.37E-02	0.010	0.019	6.13E-01
6.57032	0.016	0.010	1.01E-01	-0.012	0.015	4.24E-01
8.330871	-0.011	0.008	1.46E-01	-0.013	0.012	2.76E-01
2.769977	-0.019	0.010	5.23E-02	-0.048	0.015	1.47E-03
2.766949107	-0.020	0.010	4.73E-02	-0.046	0.015	1.71E-03
6.156507	-0.014	0.009	1.12E-01	0.002	0.013	8.94E-01
2.712447027	-0.013	0.006	1.91E-02	-0.015	0.009	8.14E-02
0.86778713	0.027	0.013	4.40E-02	0.010	0.020	6.12E-01
1.655039393	0.006	0.004	1.47E-01	0.014	0.007	3.72E-02
1.490523856	-0.008	0.004	5.43E-02	-0.007	0.006	2.54E-01
1.710214338	-0.007	0.004	8.99E-02	0.004	0.006	4.83E-01
6.980094	-0.015	0.010	1.22E-01	-0.008	0.015	5.84E-01
1.097907022	0.014	0.008	6.47E-02	0.001	0.012	9.43E-01
2.300653536	-0.008	0.004	9.38E-02	-0.004	0.007	5.71E-01
7.142591	-0.021	0.011	4.42E-02	-0.019	0.016	2.29E-01
1.878767066	-0.009	0.006	1.51E-01	-0.002	0.009	8.14E-01
2.875953	-0.011	0.008	1.72E-01	-0.007	0.012	5.74E-01

3.599956	-0.012	0.006	7.18E-02	0.005	0.010	6.37E-01
1.95715586	0.009	0.005	6.09E-02	0.005	0.007	5.09E-01
3.384976	0.013	0.009	1.77E-01	0.023	0.014	9.63E-02
1.806770492	-0.016	0.010	1.11E-01	0.012	0.015	4.25E-01
2.139838757	-0.023	0.012	5.24E-02	0.029	0.018	9.38E-02
1.612312454	0.016	0.008	2.96E-02	-0.002	0.011	8.43E-01
6.9626	-0.017	0.010	9.88E-02	-0.024	0.015	1.15E-01
6.754012	-0.017	0.011	1.30E-01	0.013	0.017	4.35E-01
1.852861757	-0.011	0.008	1.96E-01	-0.003	0.012	8.35E-01
2.320839491	-0.030	0.016	5.76E-02	0.002	0.024	9.27E-01
4.169537	-0.017	0.010	9.60E-02	0.002	0.015	8.92E-01
1.630816247	0.011	0.006	8.06E-02	0.011	0.009	2.10E-01
0.735905554	-0.012	0.007	7.67E-02	0.003	0.010	7.73E-01
2.063804991	0.008	0.004	3.51E-02	0.009	0.005	1.02E-01
8.295209	0.014	0.008	6.81E-02	-0.007	0.012	5.61E-01
3.285392	-0.024	0.012	5.21E-02	-0.018	0.018	3.26E-01
8.562337	0.010	0.006	1.14E-01	0.015	0.009	1.13E-01
8.541142	-0.013	0.007	7.13E-02	-0.005	0.011	6.38E-01
5.417365	0.014	0.008	7.58E-02	-0.010	0.011	3.73E-01
8.36687	0.013	0.008	1.15E-01	-0.007	0.012	5.70E-01
7.52276	0.010	0.007	1.20E-01	0.006	0.010	5.37E-01
6.094267	0.014	0.009	1.39E-01	-0.007	0.014	6.17E-01
0.867450697	0.026	0.013	4.48E-02	0.010	0.020	6.18E-01
1.326344752	0.024	0.011	2.61E-02	0.010	0.016	5.35E-01
5.850354	0.012	0.008	1.29E-01	0.001	0.012	9.43E-01
7.683575	0.011	0.008	1.50E-01	-0.005	0.011	6.80E-01
3.504409	0.014	0.009	1.43E-01	0.037	0.014	8.52E-03
5.485324	0.018	0.010	7.52E-02	0.000	0.015	9.79E-01
3.385312	0.012	0.009	1.82E-01	0.025	0.014	6.64E-02
1.010434549	0.019	0.010	4.76E-02	0.027	0.014	5.89E-02
6.708593	-0.015	0.009	1.03E-01	-0.020	0.014	1.44E-01
8.841912	0.013	0.007	5.69E-02	-0.013	0.010	1.75E-01
7.943637	0.014	0.010	1.65E-01	-0.004	0.015	7.74E-01
5.336958	0.035	0.019	6.21E-02	-0.010	0.028	7.29E-01
5.719818	0.016	0.010	1.25E-01	0.016	0.015	2.90E-01
1.960183753	0.009	0.005	6.66E-02	0.000	0.007	9.94E-01
1.546035234	0.031	0.016	5.13E-02	-0.008	0.024	7.34E-01
6.585459	0.012	0.010	2.09E-01	0.005	0.015	7.23E-01
7.310808	-0.020	0.009	2.91E-02	0.005	0.014	7.28E-01
0.591575973	-0.015	0.009	1.02E-01	-0.022	0.014	1.13E-01
5.737985	0.016	0.010	1.04E-01	0.002	0.015	9.04E-01
6.720705	-0.017	0.010	8.84E-02	-0.004	0.015	7.67E-01
5.093044	0.015	0.009	8.01E-02	-0.004	0.013	7.75E-01

0.867114265	0.026	0.013	4.49E-02	0.010	0.019	6.17E-01
2.686541718	-0.013	0.006	2.46E-02	-0.002	0.009	7.97E-01
5.446971	0.015	0.008	7.55E-02	0.004	0.012	7.22E-01
1.709541473	-0.008	0.004	6.98E-02	-0.001	0.007	9.03E-01
7.716209	-0.018	0.010	8.19E-02	-0.010	0.015	5.29E-01
3.591209	-0.009	0.006	1.56E-01	0.001	0.009	9.14E-01
6.728779	-0.020	0.011	5.79E-02	-0.025	0.016	1.11E-01
6.228504	0.016	0.008	5.68E-02	0.006	0.013	6.09E-01
3.586499	-0.008	0.006	1.91E-01	0.005	0.009	5.71E-01
8.448623	0.016	0.010	9.59E-02	0.015	0.015	3.16E-01
3.628889	-0.010	0.006	9.92E-02	0.011	0.009	2.06E-01
1.099252753	0.015	0.008	6.72E-02	-0.009	0.012	4.32E-01
0.625219232	0.014	0.007	5.05E-02	-0.006	0.011	5.99E-01
0.94045657	-0.008	0.005	1.32E-01	-0.023	0.008	3.98E-03
3.537716	0.014	0.008	7.74E-02	0.023	0.012	5.31E-02
1.961193051	0.009	0.005	7.03E-02	0.003	0.007	6.57E-01
2.303344996	-0.007	0.004	9.41E-02	-0.006	0.007	3.89E-01
8.265267	-0.016	0.010	1.24E-01	-0.007	0.015	6.36E-01
2.30637289	-0.009	0.005	6.95E-02	-0.008	0.007	3.13E-01
1.262422559	0.029	0.015	4.96E-02	-0.001	0.022	9.57E-01
2.192658674	0.022	0.011	5.21E-02	-0.014	0.017	4.03E-01
0.651460974	-0.020	0.010	3.99E-02	-0.013	0.014	3.47E-01
0.95223171	0.009	0.005	5.93E-02	-0.002	0.007	7.30E-01
7.600476	0.023	0.011	2.96E-02	0.014	0.016	3.85E-01
0.606379007	-0.017	0.009	4.55E-02	-0.002	0.013	8.60E-01
6.594206	-0.014	0.008	9.95E-02	-0.015	0.013	2.20E-01
4.24658	0.036	0.018	5.17E-02	0.013	0.027	6.46E-01
3.037104	-0.014	0.008	9.21E-02	-0.020	0.012	1.16E-01
3.624179	-0.011	0.006	7.72E-02	-0.004	0.009	6.85E-01
7.952385	0.012	0.008	1.39E-01	0.000	0.012	1.00E+00
1.930241253	0.008	0.005	8.07E-02	0.009	0.007	1.95E-01
7.506275	-0.014	0.009	1.05E-01	0.005	0.013	6.91E-01
5.681801	0.012	0.008	1.19E-01	-0.001	0.012	9.43E-01
6.708257	-0.018	0.010	6.94E-02	-0.038	0.015	8.17E-03
3.624516	-0.011	0.006	8.53E-02	-0.002	0.009	8.23E-01
3.071084	-0.010	0.006	8.13E-02	-0.005	0.009	5.95E-01
6.260465	0.012	0.007	9.61E-02	-0.014	0.011	2.12E-01
2.37399584	-0.019	0.010	6.93E-02	0.011	0.015	4.67E-01
2.15834255	-0.018	0.010	6.14E-02	-0.001	0.014	9.27E-01
3.392714	0.012	0.009	1.60E-01	0.044	0.013	9.01E-04
5.858092	0.014	0.008	9.24E-02	-0.001	0.012	9.11E-01
6.965964	-0.020	0.011	5.73E-02	-0.018	0.016	2.66E-01
5.266643	-0.013	0.006	2.49E-02	-0.018	0.009	4.46E-02

7.03897	-0.016	0.011	1.39E-01	0.007	0.017	6.76E-01
5.057382	0.012	0.008	1.31E-01	0.004	0.012	7.44E-01
2.320166626	-0.023	0.012	5.69E-02	0.005	0.018	7.85E-01
2.197032298	0.024	0.012	5.07E-02	-0.014	0.018	4.46E-01
3.951865	-0.013	0.008	1.31E-01	0.003	0.012	8.33E-01
8.366533	0.016	0.009	8.32E-02	-0.011	0.014	4.14E-01
7.758936	-0.024	0.011	3.50E-02	-0.018	0.017	2.74E-01
7.861548	0.012	0.008	1.70E-01	0.002	0.013	8.47E-01
1.887177881	-0.013	0.006	4.89E-02	-0.005	0.010	5.84E-01
1.769426474	-0.018	0.011	8.62E-02	-0.024	0.016	1.32E-01
1.548053829	0.033	0.017	5.15E-02	-0.006	0.025	8.15E-01
2.196695865	0.024	0.012	5.16E-02	-0.010	0.018	5.86E-01
0.952568143	0.010	0.005	5.86E-02	-0.001	0.007	9.09E-01
2.321512356	-0.025	0.014	7.15E-02	0.002	0.021	9.32E-01
3.65244	-0.008	0.005	7.82E-02	0.004	0.007	5.26E-01
8.929385	0.008	0.008	2.69E-01	-0.010	0.011	3.81E-01
6.201589	0.010	0.008	1.99E-01	-0.016	0.012	1.63E-01
3.59491	-0.012	0.007	7.82E-02	0.009	0.010	3.63E-01
5.744378	0.018	0.009	6.30E-02	0.007	0.014	6.12E-01
6.105033	0.015	0.009	1.02E-01	0.003	0.014	8.30E-01
3.509792	0.012	0.008	1.39E-01	0.032	0.012	1.02E-02
2.767958405	-0.019	0.010	5.57E-02	-0.047	0.015	1.46E-03
7.122069	0.017	0.009	7.50E-02	0.001	0.014	9.49E-01
5.029458	0.014	0.008	7.53E-02	-0.008	0.012	4.92E-01
1.139624664	0.013	0.007	4.71E-02	-0.001	0.010	8.86E-01
6.488566	-0.013	0.008	1.02E-01	0.006	0.012	5.82E-01
8.792457	-0.023	0.010	2.02E-02	-0.012	0.015	4.17E-01
2.137820161	-0.018	0.010	7.49E-02	-0.002	0.015	8.96E-01
3.952538	-0.014	0.009	1.07E-01	-0.006	0.013	6.66E-01
8.556954	0.015	0.007	5.21E-02	0.002	0.011	8.56E-01
4.172228	-0.024	0.014	8.14E-02	0.001	0.020	9.78E-01
3.971378	-0.017	0.008	4.54E-02	-0.005	0.012	6.76E-01
7.098519	-0.013	0.008	1.10E-01	-0.012	0.012	3.28E-01
6.364759	-0.018	0.010	7.66E-02	-0.005	0.015	7.59E-01
3.441496	0.011	0.007	1.10E-01	0.026	0.011	1.44E-02
8.274014	0.016	0.009	5.71E-02	0.017	0.013	1.92E-01
3.91082	0.012	0.008	1.43E-01	0.034	0.012	4.59E-03
8.132376	0.014	0.010	1.58E-01	-0.011	0.015	4.54E-01
5.130388	0.017	0.009	5.70E-02	-0.002	0.013	8.70E-01
5.266307	-0.013	0.006	2.51E-02	-0.017	0.009	5.25E-02
1.962202349	0.009	0.005	7.00E-02	0.000	0.007	9.97E-01
7.241166	-0.020	0.010	4.16E-02	-0.010	0.015	4.91E-01
1.429293125	0.009	0.004	4.08E-02	0.008	0.007	2.24E-01

8.304966	-0.010	0.006	9.44E-02	-0.008	0.009	3.82E-01
5.868521	0.011	0.007	1.16E-01	-0.001	0.010	9.23E-01
7.226027	-0.021	0.011	5.04E-02	-0.023	0.016	1.49E-01
5.575152	0.016	0.009	8.76E-02	-0.027	0.014	4.53E-02
2.018386592	0.019	0.009	4.21E-02	0.002	0.014	8.94E-01
1.801724003	-0.019	0.010	6.98E-02	0.008	0.015	6.17E-01
6.496304	0.012	0.007	9.19E-02	0.003	0.011	7.67E-01
1.450151945	0.008	0.004	4.25E-02	0.005	0.006	3.62E-01
2.712110595	-0.013	0.006	2.09E-02	-0.014	0.009	1.02E-01
5.850017	0.012	0.008	1.15E-01	0.000	0.011	9.83E-01
0.563315635	-0.010	0.006	1.05E-01	-0.014	0.009	1.40E-01
0.940793002	-0.008	0.005	1.37E-01	-0.022	0.008	4.71E-03
1.613321752	0.015	0.007	3.48E-02	0.000	0.011	9.78E-01
7.019121	0.010	0.009	2.86E-01	-0.013	0.014	3.43E-01
2.198378028	0.025	0.013	5.53E-02	-0.010	0.019	6.06E-01
2.485691461	-0.024	0.011	2.92E-02	-0.009	0.017	5.92E-01
1.080076095	0.013	0.007	8.01E-02	0.002	0.011	8.58E-01
8.965047	-0.013	0.009	1.56E-01	-0.008	0.014	5.75E-01
0.81193932	0.007	0.005	1.82E-01	0.017	0.007	2.19E-02
5.895099	0.020	0.010	5.42E-02	0.009	0.016	5.66E-01
5.391796	0.014	0.007	5.32E-02	-0.003	0.011	7.93E-01
3.267897	-0.014	0.009	9.72E-02	-0.014	0.013	2.83E-01
6.002758	0.012	0.007	1.01E-01	0.013	0.011	2.39E-01
7.424522	-0.016	0.009	7.30E-02	0.006	0.013	6.58E-01
2.196359432	0.023	0.012	5.61E-02	-0.012	0.018	5.20E-01
7.356563	-0.019	0.010	6.48E-02	-0.026	0.015	8.69E-02
3.067047	-0.011	0.007	1.02E-01	-0.007	0.010	4.86E-01
8.423727	-0.017	0.010	8.00E-02	-0.015	0.015	3.21E-01
0.912532665	-0.008	0.005	9.17E-02	-0.027	0.007	3.54E-04
5.33292	0.035	0.019	6.69E-02	-0.015	0.029	5.96E-01
6.285025	0.022	0.011	4.14E-02	-0.004	0.016	8.24E-01
3.581789	-0.008	0.006	1.54E-01	0.006	0.009	4.90E-01
7.736731	-0.018	0.012	1.30E-01	-0.009	0.018	5.92E-01
2.638095425	-0.016	0.009	6.99E-02	-0.003	0.013	8.04E-01
7.031905	-0.017	0.010	8.55E-02	-0.010	0.015	4.84E-01
0.978809885	0.012	0.007	7.80E-02	0.013	0.010	2.24E-01
5.164031	0.015	0.009	8.96E-02	-0.014	0.014	2.91E-01
6.152807	0.011	0.007	1.04E-01	0.009	0.011	3.95E-01
7.22098	-0.022	0.011	4.90E-02	-0.011	0.016	4.85E-01
5.118276	0.015	0.009	8.79E-02	-0.003	0.013	8.39E-01
5.330229	0.035	0.020	6.88E-02	-0.018	0.029	5.41E-01
7.166815	0.016	0.010	9.27E-02	-0.001	0.014	9.58E-01
8.043558	0.015	0.008	8.45E-02	0.010	0.013	4.28E-01

8.209083	0.014	0.009	9.98E-02	0.013	0.013	3.05E-01
0.777959628	0.008	0.005	9.56E-02	-0.001	0.007	8.69E-01
2.321848789	-0.021	0.011	7.30E-02	0.001	0.017	9.55E-01
5.857419	0.014	0.008	8.36E-02	0.012	0.012	2.99E-01
3.521567	0.012	0.008	1.14E-01	0.033	0.012	4.33E-03
1.102953511	0.014	0.008	6.69E-02	-0.002	0.011	8.26E-01
2.259608759	0.010	0.006	7.46E-02	0.010	0.009	2.57E-01
0.811602887	0.007	0.005	1.83E-01	0.017	0.008	2.52E-02
1.991808417	0.017	0.008	3.78E-02	-0.001	0.012	9.47E-01
8.790102	0.018	0.009	3.67E-02	-0.023	0.013	7.98E-02
5.424767	0.013	0.007	7.08E-02	0.000	0.010	9.94E-01
1.62341473	0.013	0.007	5.37E-02	0.011	0.010	2.60E-01
2.192995106	0.022	0.011	5.72E-02	-0.014	0.017	4.13E-01
1.902317348	-0.010	0.006	7.79E-02	-0.009	0.009	3.14E-01
6.698164	0.015	0.009	1.02E-01	0.017	0.014	2.24E-01
6.901369	0.015	0.008	6.85E-02	0.001	0.012	9.52E-01
3.948164	-0.012	0.008	1.01E-01	0.000	0.011	9.97E-01
5.331911	0.036	0.019	6.75E-02	-0.016	0.029	5.72E-01
3.380939	0.018	0.012	1.22E-01	0.033	0.018	5.77E-02
4.170209	-0.019	0.011	9.55E-02	0.000	0.017	9.82E-01
3.952874	-0.015	0.009	8.82E-02	-0.011	0.013	3.92E-01
7.965842	0.012	0.010	2.26E-01	0.021	0.014	1.43E-01
5.836897	-0.015	0.009	8.24E-02	0.005	0.013	7.20E-01
2.500494495	-0.021	0.013	9.84E-02	-0.002	0.019	9.06E-01
1.040377049	0.012	0.007	9.19E-02	0.010	0.010	3.39E-01
6.971011	-0.023	0.011	3.11E-02	-0.008	0.016	6.24E-01
8.153235	0.015	0.010	1.48E-01	-0.001	0.015	9.65E-01
0.945503058	-0.010	0.005	5.63E-02	-0.003	0.008	7.01E-01
3.26857	-0.015	0.010	1.07E-01	-0.016	0.014	2.56E-01
8.407914	0.018	0.009	3.68E-02	-0.003	0.013	8.26E-01
2.863842	-0.012	0.008	1.54E-01	-0.003	0.012	8.16E-01
6.382927	-0.013	0.008	1.01E-01	-0.020	0.011	8.35E-02
5.922687	-0.011	0.007	1.19E-01	-0.011	0.011	3.10E-01
1.474375092	-0.008	0.004	7.09E-02	-0.002	0.006	8.11E-01
6.586132	0.007	0.006	2.39E-01	-0.002	0.009	8.21E-01
3.138371	-0.019	0.010	6.45E-02	-0.006	0.015	6.72E-01
2.391826768	-0.013	0.008	9.17E-02	0.005	0.011	6.57E-01
8.289154	-0.017	0.010	8.02E-02	-0.032	0.015	2.57E-02
3.348977	-0.015	0.011	1.51E-01	-0.008	0.016	5.98E-01
2.352127722	0.024	0.011	2.39E-02	0.015	0.016	3.42E-01
5.594329	0.014	0.007	6.29E-02	-0.016	0.011	1.32E-01
2.762911916	-0.019	0.009	5.06E-02	-0.043	0.014	2.33E-03
6.742573	-0.018	0.009	4.95E-02	-0.034	0.014	1.58E-02

2.393172498	-0.015	0.008	6.32E-02	-0.002	0.012	8.74E-01
7.090781	-0.015	0.009	1.12E-01	0.001	0.014	9.42E-01
2.305027159	-0.008	0.005	7.14E-02	-0.006	0.007	3.79E-01
5.265298	-0.013	0.006	2.56E-02	-0.017	0.009	5.50E-02
3.533679	0.014	0.007	4.53E-02	0.027	0.011	1.18E-02
5.797534	0.015	0.010	1.39E-01	0.002	0.015	8.93E-01
7.499883	0.017	0.009	4.63E-02	0.016	0.013	2.10E-01
1.99214485	0.017	0.008	3.92E-02	-0.001	0.012	9.40E-01
5.466148	0.010	0.007	1.50E-01	-0.004	0.010	7.20E-01
2.018723024	0.019	0.009	4.58E-02	0.001	0.014	9.32E-01
0.935410081	-0.008	0.005	1.11E-01	-0.019	0.007	1.00E-02
5.712753	0.013	0.009	1.50E-01	0.017	0.013	2.11E-01
5.577171	0.014	0.008	8.04E-02	-0.009	0.012	4.59E-01
3.292793	-0.019	0.013	1.46E-01	-0.022	0.019	2.65E-01
3.037441	-0.014	0.008	9.27E-02	-0.016	0.012	1.98E-01
1.548390262	0.033	0.017	5.46E-02	-0.005	0.026	8.48E-01
3.117848	-0.018	0.009	5.00E-02	-0.024	0.014	7.41E-02
5.446298	0.014	0.008	1.03E-01	0.005	0.013	6.67E-01
3.344604	-0.014	0.010	1.62E-01	0.000	0.015	9.89E-01
6.730461	-0.021	0.011	5.62E-02	-0.017	0.016	2.82E-01
5.407945	0.014	0.008	6.30E-02	-0.011	0.012	3.51E-01
5.071849	0.014	0.008	8.80E-02	0.001	0.012	9.40E-01
8.121947	0.012	0.008	1.48E-01	-0.003	0.012	8.10E-01
7.969206	0.016	0.010	1.17E-01	0.002	0.015	9.08E-01
5.222571	0.006	0.005	2.41E-01	0.005	0.008	5.13E-01
7.740432	-0.016	0.011	1.44E-01	-0.011	0.017	5.04E-01
0.729176902	-0.013	0.007	5.98E-02	-0.019	0.010	7.18E-02
5.484988	0.016	0.009	6.55E-02	0.006	0.013	6.58E-01
1.846469538	-0.013	0.009	1.22E-01	-0.022	0.013	8.51E-02
3.568332	-0.010	0.007	1.30E-01	-0.001	0.010	9.47E-01
1.217340592	0.011	0.007	9.95E-02	0.007	0.010	5.01E-01
2.048665525	0.014	0.007	5.13E-02	0.010	0.010	3.40E-01
7.721255	-0.020	0.011	7.60E-02	-0.030	0.017	7.81E-02
3.292457	-0.024	0.014	9.15E-02	-0.017	0.021	4.32E-01
1.639227061	0.011	0.006	8.13E-02	0.000	0.009	9.74E-01
6.792701	-0.010	0.008	2.05E-01	-0.008	0.011	4.75E-01
1.760342794	-0.016	0.008	5.68E-02	0.000	0.013	9.83E-01
5.723182	0.012	0.009	1.98E-01	-0.005	0.014	7.23E-01
7.223335	-0.022	0.010	3.28E-02	-0.030	0.015	5.39E-02
2.141184487	-0.026	0.014	5.59E-02	0.040	0.020	4.75E-02
1.151399804	0.011	0.006	5.46E-02	-0.006	0.009	5.17E-01
1.076375336	0.014	0.007	6.62E-02	0.006	0.011	5.63E-01
0.609743333	-0.013	0.009	1.70E-01	-0.020	0.014	1.49E-01

5.934126	-0.015	0.009	9.92E-02	-0.024	0.013	7.69E-02
6.471072	0.014	0.008	8.99E-02	0.001	0.012	9.58E-01
1.484804502	-0.008	0.004	8.14E-02	-0.004	0.007	5.65E-01
1.898616589	-0.011	0.006	6.74E-02	-0.008	0.009	4.16E-01
0.810257157	0.007	0.005	1.97E-01	0.017	0.008	2.62E-02
7.221317	-0.020	0.011	7.00E-02	0.000	0.016	9.77E-01
0.812275752	0.007	0.005	1.89E-01	0.017	0.007	2.10E-02
3.391032	0.012	0.009	1.81E-01	0.043	0.014	1.69E-03
1.151063372	0.011	0.006	5.96E-02	-0.002	0.009	8.35E-01
1.020863959	0.015	0.007	4.49E-02	0.011	0.011	3.24E-01
5.547564	0.011	0.007	9.85E-02	0.000	0.010	9.91E-01
0.811266455	0.007	0.005	1.93E-01	0.017	0.008	2.77E-02
2.352464155	0.023	0.010	2.73E-02	0.016	0.015	2.94E-01
8.078547	0.016	0.009	9.08E-02	0.020	0.014	1.46E-01
6.651736	0.020	0.010	4.55E-02	0.006	0.015	6.94E-01
4.04943	0.028	0.014	4.14E-02	-0.002	0.021	9.04E-01
8.384364	0.017	0.009	5.92E-02	0.000	0.013	9.74E-01
1.483458772	-0.008	0.005	9.56E-02	0.002	0.007	7.73E-01
2.349436261	0.021	0.009	1.93E-02	0.018	0.014	1.78E-01
3.676326	-0.007	0.004	1.29E-01	0.005	0.007	4.82E-01
2.653907756	-0.019	0.011	6.70E-02	0.008	0.016	6.15E-01
8.817353	0.016	0.009	7.66E-02	0.008	0.014	5.42E-01
5.933789	-0.015	0.009	1.13E-01	-0.025	0.014	6.98E-02
5.106501	0.011	0.007	1.12E-01	-0.008	0.011	4.83E-01
8.098396	0.015	0.009	1.07E-01	-0.011	0.014	4.25E-01
7.642194	-0.016	0.011	1.27E-01	-0.021	0.016	1.78E-01
3.706269	0.012	0.006	5.40E-02	0.023	0.009	1.10E-02
8.021017	0.011	0.009	2.02E-01	0.008	0.013	5.47E-01
7.979636	0.013	0.008	1.15E-01	-0.006	0.012	6.34E-01
3.031049	-0.007	0.005	1.11E-01	0.000	0.007	9.83E-01
6.530957	0.014	0.007	4.71E-02	0.001	0.010	9.49E-01
3.03374	0.014	0.010	1.64E-01	0.034	0.015	1.76E-02
7.119378	-0.015	0.010	1.24E-01	-0.014	0.015	3.55E-01
7.196421	0.017	0.009	4.67E-02	-0.004	0.013	7.75E-01
2.307382187	-0.009	0.006	9.72E-02	-0.005	0.008	5.65E-01
6.545087	0.015	0.009	9.38E-02	-0.002	0.014	8.87E-01
8.051296	0.015	0.009	8.03E-02	-0.003	0.013	7.98E-01
5.383049	0.014	0.008	7.18E-02	0.000	0.012	9.67E-01
5.333257	0.035	0.019	6.90E-02	-0.013	0.029	6.48E-01
7.585337	0.021	0.010	4.59E-02	0.010	0.015	5.08E-01
7.530162	-0.014	0.008	1.10E-01	-0.010	0.013	4.12E-01
0.944830193	-0.010	0.005	6.61E-02	-0.001	0.008	8.60E-01
1.156446293	0.009	0.005	5.90E-02	0.000	0.007	9.70E-01

5.738322	0.015	0.010	1.38E-01	0.002	0.015	8.94E-01
3.623843	-0.011	0.006	7.51E-02	-0.004	0.009	6.30E-01
5.329556	0.035	0.019	7.05E-02	-0.017	0.029	5.63E-01
8.701284	0.013	0.007	9.02E-02	0.005	0.011	6.28E-01
2.869561	-0.013	0.009	1.42E-01	0.004	0.014	7.70E-01
2.348090531	0.022	0.010	2.52E-02	0.013	0.014	3.58E-01
8.007223	0.012	0.008	1.50E-01	0.006	0.012	6.26E-01
1.262086127	0.028	0.015	5.45E-02	-0.002	0.022	9.26E-01
2.403938341	-0.020	0.011	6.50E-02	0.022	0.016	1.81E-01
3.65614	-0.009	0.005	5.67E-02	0.008	0.007	2.78E-01
6.688071	-0.015	0.009	7.74E-02	-0.009	0.013	4.93E-01
6.342891	-0.014	0.009	1.37E-01	0.007	0.014	6.03E-01
1.01447174	0.023	0.011	4.11E-02	0.013	0.017	4.29E-01
5.239056	0.007	0.006	2.46E-01	0.011	0.009	2.25E-01
3.060318	-0.013	0.006	4.54E-02	-0.004	0.009	6.46E-01
6.732816	-0.015	0.008	5.86E-02	-0.005	0.012	6.77E-01
4.246243	0.035	0.018	5.61E-02	0.007	0.027	7.95E-01
3.105737	-0.015	0.008	7.97E-02	-0.014	0.013	2.67E-01
7.142255	-0.021	0.011	5.49E-02	-0.014	0.016	3.79E-01
0.690823587	0.029	0.016	7.35E-02	0.003	0.024	9.09E-01
2.393845363	-0.016	0.009	6.34E-02	-0.003	0.013	8.06E-01
2.305700024	-0.008	0.005	7.40E-02	-0.010	0.007	1.69E-01
6.958226	-0.016	0.009	7.02E-02	0.001	0.013	9.49E-01
1.960520186	0.009	0.005	7.75E-02	0.001	0.007	9.03E-01
6.853596	-0.020	0.010	5.26E-02	-0.006	0.015	7.05E-01
1.405069978	0.010	0.005	3.79E-02	0.006	0.007	3.94E-01
5.754807	0.014	0.008	9.96E-02	0.006	0.012	6.29E-01
7.061175	-0.014	0.009	1.31E-01	-0.024	0.014	7.77E-02
5.440579	-0.017	0.009	5.38E-02	-0.010	0.014	4.68E-01
7.136536	0.019	0.010	4.95E-02	-0.007	0.015	6.15E-01
1.992481282	0.017	0.009	4.25E-02	-0.002	0.013	8.81E-01
3.381611	0.017	0.011	1.26E-01	0.029	0.016	7.42E-02
3.500036	0.012	0.008	1.24E-01	0.036	0.012	2.97E-03
1.428956692	0.009	0.004	4.71E-02	0.006	0.007	3.32E-01
5.624944	0.015	0.009	8.61E-02	0.018	0.013	1.78E-01
3.676663	-0.007	0.004	1.29E-01	0.004	0.007	5.24E-01
1.894579398	-0.012	0.007	8.04E-02	-0.005	0.010	6.47E-01
0.861058478	0.019	0.010	5.85E-02	0.005	0.015	7.28E-01
2.76728554	-0.019	0.010	5.79E-02	-0.046	0.015	1.69E-03
8.076192	-0.014	0.009	1.21E-01	-0.006	0.014	6.58E-01
7.213242	-0.011	0.007	1.32E-01	-0.001	0.011	9.34E-01
6.722724	-0.017	0.011	1.16E-01	-0.016	0.016	3.03E-01
7.060838	-0.016	0.009	8.81E-02	-0.021	0.014	1.42E-01

3.582798	-0.007	0.006	2.74E-01	0.001	0.009	9.56E-01
6.929966	0.014	0.009	9.79E-02	0.001	0.013	9.27E-01
3.481868	0.014	0.010	1.43E-01	0.038	0.014	7.02E-03
6.554507	0.022	0.011	4.10E-02	0.006	0.016	7.30E-01
2.097111818	-0.010	0.008	1.87E-01	-0.007	0.012	5.56E-01
3.625189	-0.011	0.007	1.07E-01	0.000	0.010	9.99E-01
3.956238	-0.012	0.008	1.36E-01	-0.019	0.012	1.23E-01
7.214588	-0.015	0.010	1.05E-01	-0.006	0.014	6.90E-01
7.803009	-0.017	0.009	6.56E-02	-0.015	0.014	2.86E-01
2.982266	-0.007	0.004	1.08E-01	0.001	0.006	9.10E-01
2.762239051	-0.018	0.009	5.83E-02	-0.042	0.014	3.00E-03
6.242971	0.013	0.008	1.24E-01	-0.008	0.013	5.31E-01
6.255082	0.015	0.009	9.93E-02	0.007	0.014	6.08E-01
2.860141	-0.012	0.008	1.13E-01	-0.010	0.012	3.86E-01
1.994163445	0.019	0.009	4.28E-02	0.000	0.014	9.78E-01
2.886719	-0.011	0.007	9.18E-02	-0.008	0.010	4.37E-01
6.091912	0.011	0.007	1.38E-01	0.016	0.011	1.60E-01
6.130939	0.014	0.008	9.27E-02	0.028	0.012	2.19E-02
2.766276242	-0.019	0.010	5.65E-02	-0.047	0.015	1.36E-03
2.553314412	-0.018	0.011	9.31E-02	-0.008	0.016	6.30E-01
1.330381943	0.022	0.010	3.06E-02	0.011	0.015	4.94E-01
6.243644	0.012	0.008	1.45E-01	0.011	0.012	3.74E-01
2.193331539	0.021	0.011	6.37E-02	-0.014	0.017	4.27E-01
7.165132	-0.017	0.011	1.22E-01	0.011	0.016	4.82E-01
6.556526	-0.012	0.009	1.60E-01	-0.003	0.013	8.01E-01
2.41033056	-0.022	0.012	6.97E-02	-0.002	0.018	8.98E-01
0.810930022	0.006	0.005	2.05E-01	0.016	0.008	3.07E-02
3.67599	-0.007	0.004	1.31E-01	0.005	0.007	4.70E-01
3.29212	-0.026	0.014	7.39E-02	-0.018	0.021	4.10E-01
6.045485	0.010	0.007	1.17E-01	0.013	0.010	1.90E-01
3.349987	-0.016	0.010	1.11E-01	-0.024	0.015	1.03E-01
5.798543	0.011	0.009	2.13E-01	0.017	0.013	1.78E-01
8.096714	0.013	0.009	1.30E-01	0.004	0.013	7.56E-01
8.302947	0.012	0.007	7.25E-02	0.010	0.010	3.35E-01
6.462325	0.012	0.007	6.78E-02	0.006	0.010	5.65E-01
3.846225	0.009	0.006	1.31E-01	0.019	0.009	4.29E-02
6.615402	0.012	0.008	1.39E-01	0.010	0.012	3.67E-01
2.916325	-0.013	0.008	8.60E-02	0.001	0.011	8.99E-01
7.642867	-0.013	0.011	2.29E-01	-0.011	0.016	4.75E-01
7.912013	-0.012	0.008	1.21E-01	-0.005	0.011	6.37E-01
7.48407	-0.014	0.009	1.46E-01	-0.009	0.014	5.16E-01
2.764594079	-0.019	0.010	5.45E-02	-0.043	0.014	2.64E-03
0.624882799	0.011	0.006	5.10E-02	-0.011	0.009	1.89E-01

1.783556643	-0.014	0.010	1.65E-01	-0.009	0.015	5.57E-01
7.041325	-0.020	0.013	1.10E-01	-0.033	0.019	8.56E-02
5.143845	0.012	0.007	1.23E-01	-0.009	0.011	4.27E-01
5.32922	0.035	0.019	7.32E-02	-0.014	0.029	6.16E-01
7.367665	-0.015	0.009	7.66E-02	0.006	0.013	6.31E-01
2.393508931	-0.016	0.008	5.81E-02	-0.004	0.012	7.35E-01
1.991471984	0.017	0.008	4.07E-02	-0.001	0.012	9.43E-01
3.674308	-0.007	0.005	1.08E-01	0.002	0.007	8.20E-01
2.192322241	0.021	0.011	6.02E-02	-0.015	0.017	3.80E-01
8.497069	-0.017	0.009	4.72E-02	-0.024	0.013	6.39E-02
5.707034	0.014	0.009	1.37E-01	0.008	0.014	5.84E-01
3.863046	-0.009	0.005	6.91E-02	0.003	0.007	6.81E-01
2.770986298	-0.019	0.010	6.38E-02	-0.049	0.015	1.17E-03
5.166723	0.019	0.010	6.05E-02	-0.015	0.015	3.30E-01
7.999149	0.014	0.009	1.08E-01	0.008	0.013	5.52E-01
4.140267	-0.031	0.019	9.93E-02	0.014	0.028	6.11E-01
5.945564	0.012	0.007	8.49E-02	0.000	0.011	9.80E-01
7.191711	0.015	0.009	1.03E-01	-0.007	0.014	6.23E-01
0.809920724	0.006	0.005	2.03E-01	0.017	0.008	2.80E-02
5.26698	-0.013	0.006	2.88E-02	-0.017	0.009	4.93E-02
5.842279	0.016	0.009	7.73E-02	0.006	0.013	6.57E-01
1.545025936	0.029	0.015	5.88E-02	-0.012	0.023	6.10E-01
5.267316	-0.013	0.006	2.85E-02	-0.018	0.009	4.44E-02
3.95422	-0.018	0.011	1.09E-01	-0.018	0.016	2.70E-01
7.015756	0.010	0.007	1.32E-01	-0.010	0.010	3.34E-01
8.626932	-0.019	0.010	5.94E-02	-0.002	0.015	8.70E-01
6.532303	0.012	0.008	1.64E-01	0.013	0.012	3.11E-01
3.036768	-0.013	0.008	1.04E-01	-0.019	0.012	1.09E-01
3.656813	-0.009	0.005	6.64E-02	0.009	0.007	2.17E-01
0.810593589	0.006	0.005	2.07E-01	0.017	0.008	2.89E-02
6.816252	0.016	0.010	1.29E-01	0.007	0.015	6.55E-01
1.408770736	0.011	0.006	4.90E-02	0.017	0.009	4.79E-02
0.51722437	-0.016	0.010	1.13E-01	-0.012	0.015	4.40E-01
5.516613	0.015	0.009	1.12E-01	0.000	0.014	9.83E-01
8.894059	0.013	0.009	1.47E-01	0.000	0.014	9.76E-01
7.919078	-0.019	0.010	5.54E-02	-0.023	0.015	1.14E-01
5.071512	0.014	0.008	9.01E-02	0.005	0.012	6.86E-01
2.770313433	-0.019	0.010	6.22E-02	-0.048	0.015	1.24E-03
2.195013702	0.022	0.012	6.29E-02	-0.014	0.017	4.27E-01
3.035422	0.012	0.008	1.21E-01	0.005	0.012	6.72E-01
3.87213	-0.008	0.004	9.40E-02	0.008	0.007	2.32E-01
6.688407	-0.017	0.009	5.38E-02	-0.009	0.013	4.72E-01
3.527287	0.012	0.007	8.33E-02	0.025	0.010	1.46E-02

2.28955126	-0.007	0.005	1.49E-01	-0.010	0.007	1.35E-01
5.334266	0.034	0.019	7.15E-02	-0.013	0.028	6.54E-01
1.545698801	0.029	0.016	6.01E-02	-0.011	0.023	6.53E-01
1.992817715	0.017	0.009	4.46E-02	-0.002	0.013	8.93E-01
5.950947	0.010	0.006	1.19E-01	0.005	0.009	6.31E-01
3.110447	-0.020	0.010	4.61E-02	-0.018	0.015	2.23E-01
1.477739418	-0.009	0.005	1.17E-01	0.005	0.008	5.70E-01
1.43904967	0.007	0.004	7.19E-02	0.004	0.006	4.89E-01
0.569371422	0.019	0.009	4.45E-02	0.014	0.014	3.11E-01
3.027348	-0.009	0.005	1.13E-01	-0.005	0.008	5.50E-01
3.527623	0.012	0.007	9.34E-02	0.028	0.011	9.34E-03
3.566649	-0.014	0.008	8.51E-02	0.015	0.012	2.18E-01
8.746029	0.013	0.007	6.67E-02	0.002	0.011	8.82E-01
3.045515	-0.009	0.006	1.20E-01	-0.009	0.009	3.13E-01
3.675317	-0.007	0.005	1.17E-01	0.004	0.007	5.40E-01
2.577201126	-0.015	0.012	1.97E-01	0.015	0.017	3.83E-01
5.527379	0.012	0.007	7.85E-02	-0.002	0.010	8.20E-01
5.896109	0.018	0.008	2.96E-02	0.008	0.012	5.06E-01
3.983153	-0.014	0.009	1.33E-01	-0.011	0.013	4.11E-01
2.767621972	-0.019	0.010	6.16E-02	-0.047	0.015	1.49E-03
0.553222657	0.014	0.008	6.77E-02	0.007	0.011	5.31E-01
5.451345	0.014	0.008	7.72E-02	-0.003	0.011	7.79E-01
3.586162	-0.007	0.006	2.52E-01	0.005	0.009	5.90E-01
6.462661	0.015	0.008	6.80E-02	0.015	0.012	2.42E-01
5.329893	0.035	0.019	7.34E-02	-0.019	0.029	5.05E-01
8.905498	-0.014	0.009	1.17E-01	-0.012	0.013	3.58E-01
8.787747	0.012	0.008	1.21E-01	0.012	0.012	3.12E-01
1.643937118	0.010	0.006	7.07E-02	0.006	0.009	4.80E-01
2.770649865	-0.019	0.010	6.57E-02	-0.050	0.015	9.49E-04
8.818026	0.015	0.009	9.61E-02	0.005	0.013	7.01E-01
1.545362368	0.029	0.015	5.98E-02	-0.011	0.023	6.24E-01
3.589527	-0.009	0.006	1.37E-01	0.001	0.009	9.16E-01
7.710153	-0.017	0.010	8.42E-02	-0.017	0.014	2.48E-01
1.859590409	-0.014	0.009	9.69E-02	0.006	0.013	6.24E-01
3.1054	-0.015	0.008	7.68E-02	-0.015	0.012	2.33E-01
6.963273	-0.019	0.010	6.61E-02	-0.002	0.015	9.02E-01
3.214404	-0.031	0.016	5.98E-02	-0.017	0.024	4.76E-01
7.337386	-0.014	0.009	1.01E-01	0.011	0.013	3.93E-01
4.167182	-0.022	0.013	9.07E-02	-0.001	0.020	9.42E-01
8.562	0.011	0.009	2.01E-01	0.014	0.013	2.76E-01
2.402929043	-0.017	0.010	1.09E-01	0.033	0.016	3.41E-02
8.744347	0.016	0.009	7.94E-02	-0.016	0.014	2.59E-01
2.140848055	-0.025	0.013	5.51E-02	0.038	0.019	4.85E-02

8.840567	0.016	0.009	8.06E-02	0.013	0.013	3.21E-01
7.462539	0.014	0.010	1.43E-01	-0.006	0.014	6.67E-01
2.556678737	-0.025	0.012	4.03E-02	-0.016	0.019	3.81E-01
0.669628334	-0.012	0.008	1.34E-01	-0.023	0.011	4.43E-02
7.515359	0.014	0.009	1.19E-01	-0.009	0.013	4.80E-01
3.510802	0.012	0.008	1.28E-01	0.028	0.012	1.90E-02
1.63249841	0.012	0.006	6.37E-02	0.006	0.010	5.54E-01
8.744683	0.013	0.007	6.35E-02	-0.008	0.010	4.14E-01
6.181067	0.012	0.007	1.04E-01	-0.002	0.011	8.40E-01
7.659688	0.015	0.008	6.21E-02	-0.003	0.012	8.23E-01
0.899411793	0.018	0.009	4.14E-02	-0.009	0.013	4.72E-01
1.200855395	0.008	0.005	1.18E-01	0.008	0.008	3.13E-01
8.836866	0.012	0.008	1.05E-01	0.001	0.011	8.95E-01
3.214741	-0.030	0.016	6.14E-02	-0.017	0.024	4.81E-01
6.321696	0.013	0.007	6.57E-02	0.008	0.011	4.78E-01
6.186786	-0.016	0.009	7.06E-02	-0.020	0.013	1.47E-01
6.712631	-0.015	0.010	1.42E-01	-0.002	0.015	8.79E-01
2.313437974	-0.008	0.005	1.15E-01	-0.002	0.008	8.02E-01
7.852464	-0.016	0.011	1.39E-01	0.027	0.016	8.64E-02
5.711744	0.015	0.009	1.09E-01	0.000	0.014	9.85E-01
1.082767556	0.010	0.006	9.27E-02	-0.006	0.009	4.86E-01
6.334817	0.019	0.010	7.36E-02	0.007	0.015	6.48E-01
5.386077	0.013	0.007	6.25E-02	0.006	0.010	5.78E-01
3.584817	-0.005	0.007	4.86E-01	0.004	0.010	6.54E-01
5.330565	0.034	0.020	7.80E-02	-0.016	0.029	5.80E-01
1.491533154	-0.008	0.004	6.68E-02	-0.006	0.006	2.98E-01
5.66397	0.012	0.008	1.40E-01	0.007	0.012	5.52E-01
5.50181	0.010	0.007	1.34E-01	-0.001	0.010	9.46E-01
5.254532	-0.012	0.005	2.30E-02	-0.010	0.008	2.11E-01
3.054935	-0.011	0.008	1.52E-01	0.005	0.012	7.05E-01
1.619713971	0.013	0.006	4.91E-02	0.004	0.010	6.73E-01
1.914092488	-0.011	0.006	5.29E-02	-0.007	0.009	3.92E-01
1.155436995	0.010	0.005	5.84E-02	-0.002	0.008	8.29E-01
5.714435	0.012	0.008	1.56E-01	-0.015	0.013	2.31E-01
6.385282	-0.013	0.008	1.26E-01	-0.004	0.013	7.25E-01
7.92547	0.015	0.009	1.10E-01	-0.006	0.014	6.69E-01
2.764930511	-0.019	0.010	5.76E-02	-0.044	0.015	2.48E-03
7.574234	0.013	0.008	1.25E-01	0.014	0.013	2.73E-01
2.71278346	-0.013	0.006	2.69E-02	-0.016	0.009	6.55E-02
5.081942	0.013	0.009	1.40E-01	-0.011	0.013	4.01E-01
5.018692	0.015	0.010	1.32E-01	-0.006	0.015	6.62E-01
6.900696	0.016	0.009	6.32E-02	-0.006	0.013	6.69E-01
6.045821	0.009	0.006	1.27E-01	0.008	0.009	3.85E-01

1.490187424	-0.008	0.004	6.31E-02	-0.010	0.006	1.02E-01
6.284352	-0.013	0.009	1.55E-01	-0.016	0.014	2.39E-01
0.582828725	-0.010	0.009	2.48E-01	-0.005	0.013	7.08E-01
6.90036	0.017	0.009	5.47E-02	-0.010	0.013	4.39E-01
1.879439931	-0.009	0.006	1.34E-01	-0.004	0.009	6.48E-01
6.674614	0.017	0.010	8.74E-02	-0.015	0.015	3.17E-01
0.944493761	-0.009	0.005	8.71E-02	-0.001	0.007	8.84E-01
1.123139467	0.012	0.006	6.94E-02	0.000	0.010	9.92E-01
7.823531	-0.020	0.009	2.83E-02	-0.011	0.014	4.36E-01
2.762575483	-0.018	0.009	6.02E-02	-0.042	0.014	2.94E-03
2.322858087	-0.011	0.007	9.06E-02	-0.005	0.010	5.90E-01
2.140511622	-0.023	0.012	5.82E-02	0.035	0.018	5.51E-02
0.953241008	0.010	0.006	6.38E-02	0.001	0.008	9.18E-01
5.853045	0.013	0.007	7.44E-02	0.010	0.011	3.96E-01
2.633385368	-0.014	0.008	8.78E-02	-0.022	0.013	8.04E-02
0.765848055	0.009	0.005	9.21E-02	0.007	0.008	3.69E-01
3.214068	-0.031	0.016	6.07E-02	-0.017	0.024	4.75E-01
1.16250208	0.009	0.004	2.68E-02	-0.003	0.006	6.05E-01
5.268998	-0.013	0.006	3.15E-02	-0.018	0.009	4.64E-02
5.332584	0.035	0.019	7.37E-02	-0.015	0.029	6.04E-01
2.158678982	-0.017	0.009	7.13E-02	-0.007	0.014	6.04E-01
2.869897	-0.013	0.009	1.53E-01	-0.002	0.014	9.00E-01
3.521231	0.011	0.008	1.38E-01	0.031	0.011	5.63E-03
7.483734	-0.011	0.008	1.66E-01	-0.017	0.012	1.45E-01
3.041142	-0.009	0.006	1.23E-01	0.004	0.009	6.70E-01
1.544353071	0.028	0.015	6.26E-02	-0.010	0.022	6.38E-01
0.812612185	0.006	0.005	2.05E-01	0.017	0.007	2.02E-02
5.742695	0.014	0.009	1.04E-01	0.012	0.013	3.55E-01
0.965016149	0.009	0.005	6.53E-02	0.013	0.007	9.24E-02
3.067383	-0.010	0.006	1.09E-01	-0.002	0.010	8.70E-01
1.847142403	-0.015	0.009	7.86E-02	-0.013	0.013	3.22E-01
6.887912	0.014	0.008	7.62E-02	0.004	0.012	7.67E-01
7.04368	-0.022	0.012	7.77E-02	0.004	0.019	8.38E-01
8.619194	0.014	0.009	1.34E-01	0.001	0.014	9.59E-01
7.639166	-0.014	0.012	2.36E-01	0.029	0.017	1.01E-01
1.677580377	-0.005	0.004	1.80E-01	-0.001	0.006	8.61E-01
3.727801	0.010	0.008	1.86E-01	0.025	0.012	3.02E-02
3.625525	-0.011	0.007	1.24E-01	0.002	0.010	8.35E-01
3.844543	0.009	0.006	1.28E-01	0.023	0.009	9.79E-03
0.606715439	-0.015	0.008	7.54E-02	0.012	0.012	3.27E-01
1.643600685	0.010	0.006	7.52E-02	0.010	0.009	2.21E-01
2.761902618	-0.017	0.009	6.72E-02	-0.040	0.014	4.54E-03
8.670668	-0.012	0.009	1.48E-01	-0.025	0.013	4.99E-02

5.548237	0.010	0.008	1.98E-01	-0.006	0.012	6.35E-01
8.36115	-0.015	0.009	1.10E-01	0.013	0.014	3.42E-01
2.321175924	-0.028	0.016	7.29E-02	0.003	0.023	8.91E-01
7.16446	-0.015	0.011	1.62E-01	0.001	0.016	9.48E-01
3.549155	0.022	0.014	1.00E-01	0.047	0.020	2.15E-02
3.247038	0.013	0.008	1.05E-01	0.031	0.012	7.07E-03
1.488168828	-0.008	0.004	7.34E-02	-0.009	0.007	1.96E-01
6.202262	0.015	0.009	9.09E-02	-0.012	0.013	3.65E-01
1.261749694	0.027	0.014	6.04E-02	-0.003	0.022	8.97E-01
5.583563	0.012	0.006	6.54E-02	0.002	0.010	8.21E-01
1.993827012	0.018	0.009	4.79E-02	-0.001	0.014	9.57E-01
1.78322021	-0.013	0.008	1.29E-01	0.005	0.012	6.64E-01
8.384028	0.014	0.007	5.13E-02	0.002	0.011	8.17E-01
3.120876	-0.016	0.008	3.99E-02	-0.002	0.012	8.64E-01
5.647821	0.013	0.008	8.73E-02	-0.016	0.012	1.70E-01
1.646292146	0.010	0.005	7.06E-02	0.010	0.008	2.36E-01
7.033924	-0.024	0.010	2.27E-02	-0.021	0.016	1.70E-01
1.040040617	0.011	0.007	1.10E-01	0.006	0.010	5.63E-01
6.073409	0.014	0.007	5.27E-02	-0.001	0.011	8.92E-01
2.484682163	-0.027	0.011	1.89E-02	-0.009	0.017	6.14E-01
2.534474186	-0.022	0.011	4.62E-02	-0.009	0.016	5.99E-01
0.66390898	-0.015	0.009	1.18E-01	-0.043	0.014	2.02E-03
5.384395	0.016	0.009	7.56E-02	0.004	0.014	7.80E-01
7.804354	-0.016	0.009	6.61E-02	-0.011	0.013	4.02E-01
6.530621	0.013	0.007	5.01E-02	-0.001	0.010	9.12E-01
7.964496	0.010	0.008	2.18E-01	0.014	0.013	2.51E-01
1.028601908	0.011	0.007	1.00E-01	0.009	0.010	3.70E-01
2.130418644	-0.020	0.012	1.04E-01	-0.018	0.018	3.03E-01
1.619041106	0.012	0.006	5.06E-02	0.001	0.010	9.11E-01
7.299033	-0.016	0.010	9.42E-02	0.016	0.014	2.71E-01
3.150819	-0.015	0.009	8.94E-02	-0.012	0.014	3.68E-01
3.52695	0.012	0.007	8.24E-02	0.024	0.010	1.95E-02
8.768234	0.014	0.009	1.14E-01	-0.001	0.013	9.68E-01
7.161432	0.015	0.009	9.55E-02	0.001	0.013	9.32E-01
6.085184	0.013	0.008	1.16E-01	0.024	0.012	5.89E-02
3.980798	-0.014	0.010	1.77E-01	-0.014	0.016	3.63E-01
7.054446	-0.015	0.009	1.08E-01	-0.025	0.014	7.32E-02
3.657486	-0.008	0.005	8.68E-02	0.009	0.007	2.20E-01
8.108489	-0.012	0.008	1.47E-01	0.001	0.013	9.48E-01
3.675654	-0.007	0.005	1.34E-01	0.005	0.007	4.82E-01
6.725415	-0.021	0.013	9.37E-02	-0.055	0.019	4.18E-03
1.84680597	-0.014	0.009	1.07E-01	-0.008	0.013	5.54E-01
2.352800587	0.021	0.010	3.43E-02	0.014	0.015	3.43E-01

6.920882	-0.018	0.010	7.01E-02	0.013	0.015	3.55E-01
2.255571568	0.015	0.008	5.07E-02	0.007	0.011	5.32E-01
7.13519	0.014	0.009	1.04E-01	-0.019	0.013	1.50E-01
0.728504037	-0.013	0.007	6.01E-02	-0.007	0.011	5.28E-01
7.037624	-0.023	0.011	3.25E-02	-0.018	0.016	2.80E-01
3.345277	-0.014	0.009	1.30E-01	0.002	0.014	8.58E-01
5.840261	-0.014	0.009	1.14E-01	-0.002	0.013	8.61E-01
6.462998	0.013	0.008	9.73E-02	0.009	0.012	4.68E-01
2.76863127	-0.018	0.010	6.65E-02	-0.045	0.015	2.57E-03
5.921678	-0.017	0.008	4.41E-02	-0.012	0.012	3.25E-01
1.162838512	0.009	0.004	2.80E-02	-0.002	0.006	7.21E-01
6.991197	0.011	0.007	1.24E-01	0.007	0.010	4.85E-01
2.190303646	0.021	0.011	6.21E-02	-0.013	0.017	4.25E-01
5.328883	0.034	0.019	8.09E-02	-0.016	0.029	5.72E-01
0.76786665	0.009	0.005	8.49E-02	-0.002	0.008	7.81E-01
2.374332273	-0.022	0.013	7.63E-02	0.012	0.019	5.17E-01
2.156323954	-0.014	0.009	1.44E-01	-0.011	0.014	4.21E-01
7.140236	-0.018	0.011	8.27E-02	-0.008	0.016	6.27E-01
6.477128	0.013	0.008	1.26E-01	-0.009	0.013	4.85E-01
7.665408	0.017	0.011	1.47E-01	0.004	0.017	8.36E-01
6.573684	0.011	0.008	2.04E-01	0.016	0.012	1.88E-01
6.756703	-0.013	0.008	9.99E-02	-0.007	0.011	5.19E-01
2.49208368	-0.031	0.017	6.58E-02	-0.006	0.025	8.15E-01
8.085948	0.010	0.009	2.48E-01	0.011	0.014	4.35E-01
5.268662	-0.013	0.006	3.22E-02	-0.018	0.009	4.63E-02
7.52747	-0.015	0.010	1.42E-01	-0.016	0.015	2.86E-01
1.09757059	0.013	0.008	1.09E-01	-0.004	0.012	7.12E-01
6.980431	-0.015	0.010	1.24E-01	-0.016	0.015	2.67E-01
0.735569122	-0.011	0.007	9.47E-02	-0.006	0.010	5.64E-01
5.635373	-0.017	0.010	8.48E-02	-0.025	0.015	9.19E-02
6.731134	-0.023	0.011	2.70E-02	-0.039	0.016	1.38E-02
1.166875703	0.009	0.005	4.88E-02	0.000	0.007	9.86E-01
3.482541	0.013	0.009	1.58E-01	0.030	0.014	2.92E-02
6.585123	0.014	0.009	1.46E-01	0.007	0.014	6.06E-01
6.327415	0.014	0.009	9.72E-02	-0.007	0.013	5.66E-01
6.740891	-0.023	0.011	3.75E-02	-0.014	0.017	3.94E-01
1.993154147	0.017	0.009	5.03E-02	-0.001	0.013	9.29E-01
8.775972	0.014	0.009	1.17E-01	-0.008	0.013	5.53E-01
1.090505505	0.013	0.007	6.44E-02	-0.003	0.011	7.43E-01
6.931985	0.009	0.008	2.79E-01	0.023	0.012	6.65E-02
1.99349058	0.018	0.009	5.03E-02	-0.001	0.013	9.52E-01
5.970124	0.011	0.009	2.53E-01	0.014	0.014	3.03E-01
0.562979202	-0.010	0.007	1.51E-01	-0.007	0.010	5.17E-01

2.768294837	-0.018	0.010	6.96E-02	-0.044	0.015	2.83E-03
5.332248	0.034	0.019	7.80E-02	-0.015	0.029	6.13E-01
6.700855	0.010	0.008	2.10E-01	-0.011	0.012	3.25E-01
0.941129435	-0.007	0.005	1.62E-01	-0.022	0.008	4.78E-03
2.771322731	-0.018	0.010	7.22E-02	-0.047	0.015	2.25E-03
1.62476046	0.012	0.006	6.85E-02	0.008	0.009	4.10E-01
5.593319	0.013	0.008	1.07E-01	-0.010	0.012	3.85E-01
0.939447272	-0.008	0.005	1.55E-01	-0.022	0.008	4.86E-03
4.276859	0.029	0.015	5.13E-02	0.018	0.022	4.27E-01
2.609162222	-0.021	0.013	1.09E-01	0.025	0.019	2.00E-01
6.724069	-0.022	0.012	6.89E-02	-0.022	0.018	2.12E-01
3.063346	-0.010	0.007	1.67E-01	0.008	0.011	4.41E-01
1.040713482	0.011	0.007	1.15E-01	0.015	0.010	1.56E-01
8.067781	0.013	0.008	9.71E-02	0.014	0.012	2.47E-01
5.253859	-0.012	0.005	2.26E-02	-0.011	0.008	1.54E-01
1.154091265	0.011	0.006	5.46E-02	-0.007	0.008	3.82E-01
3.656477	-0.009	0.005	6.93E-02	0.008	0.007	2.44E-01
8.02068	0.010	0.008	2.18E-01	-0.007	0.012	5.77E-01
8.014288	-0.012	0.009	1.68E-01	-0.002	0.013	8.67E-01
1.084449719	0.011	0.007	1.16E-01	0.004	0.010	6.80E-01
3.65715	-0.008	0.005	8.03E-02	0.009	0.007	2.09E-01
3.721072	0.012	0.008	1.21E-01	0.023	0.012	4.93E-02
3.882223	0.010	0.007	1.30E-01	0.026	0.010	1.26E-02
3.154519	-0.016	0.009	7.66E-02	-0.003	0.013	8.52E-01
7.180945	0.015	0.008	7.13E-02	0.000	0.012	9.89E-01
2.856777	-0.010	0.007	1.69E-01	-0.010	0.011	3.75E-01
0.765511622	0.009	0.005	7.41E-02	0.000	0.008	9.53E-01
1.064600196	0.009	0.007	1.55E-01	-0.009	0.010	3.47E-01
1.544016638	0.027	0.015	6.78E-02	-0.011	0.022	6.13E-01
3.755388	0.018	0.011	1.04E-01	0.025	0.016	1.24E-01
5.267653	-0.013	0.006	3.19E-02	-0.018	0.009	4.31E-02
5.331575	0.034	0.020	7.79E-02	-0.018	0.029	5.46E-01
3.974742	-0.014	0.009	1.50E-01	0.007	0.014	6.29E-01
2.191985809	0.020	0.011	7.73E-02	-0.017	0.017	3.23E-01
1.858581111	-0.014	0.009	1.31E-01	0.000	0.014	9.76E-01
5.93749	0.013	0.008	9.58E-02	0.007	0.011	5.20E-01
1.544689503	0.028	0.015	6.67E-02	-0.012	0.023	5.93E-01
2.765939809	-0.018	0.010	6.39E-02	-0.045	0.015	1.94E-03
8.085612	0.014	0.011	1.95E-01	0.013	0.016	4.27E-01
0.939783704	-0.008	0.005	1.47E-01	-0.023	0.008	3.75E-03
2.681158796	-0.011	0.005	3.83E-02	0.005	0.008	5.36E-01
5.264961	-0.013	0.006	2.90E-02	-0.016	0.009	5.92E-02
1.931923416	0.008	0.005	7.36E-02	0.006	0.007	3.75E-01

6.978749	-0.016	0.010	9.45E-02	-0.009	0.014	5.44E-01
7.736058	-0.017	0.012	1.35E-01	-0.018	0.017	3.11E-01
6.656446	-0.016	0.009	8.00E-02	0.009	0.014	5.09E-01
2.765266944	-0.018	0.010	6.25E-02	-0.045	0.015	2.04E-03
7.747834	-0.013	0.010	1.92E-01	0.011	0.015	4.41E-01
8.221867	0.018	0.010	7.40E-02	-0.010	0.015	5.17E-01
5.550256	0.012	0.007	1.06E-01	0.003	0.011	7.85E-01
6.597234	0.011	0.008	1.52E-01	-0.009	0.012	4.40E-01
2.87528	-0.011	0.009	1.92E-01	0.000	0.013	9.91E-01
1.153081967	0.011	0.006	5.81E-02	-0.003	0.008	7.31E-01
8.138432	-0.015	0.009	8.41E-02	-0.018	0.013	1.61E-01
0.953577441	0.010	0.006	6.56E-02	0.002	0.008	8.57E-01
1.057535111	0.010	0.006	7.53E-02	0.009	0.008	2.69E-01
7.719237	-0.019	0.011	8.06E-02	-0.027	0.017	1.07E-01
5.251504	-0.012	0.005	2.15E-02	-0.009	0.008	2.15E-01
0.518906533	0.014	0.009	1.26E-01	0.000	0.014	1.00E+00
0.809584292	0.006	0.005	2.23E-01	0.017	0.008	3.03E-02
2.870234	-0.014	0.009	1.40E-01	-0.002	0.014	8.69E-01
2.771659163	-0.018	0.010	7.66E-02	-0.046	0.015	2.68E-03
3.971714	-0.016	0.009	5.99E-02	-0.007	0.013	5.91E-01
5.950611	0.010	0.008	1.94E-01	-0.013	0.011	2.36E-01
5.090016	0.012	0.009	1.74E-01	-0.006	0.013	6.50E-01
3.392377	0.012	0.009	1.92E-01	0.044	0.013	9.79E-04
1.162165647	0.009	0.004	3.21E-02	-0.004	0.006	5.17E-01
3.845888	0.009	0.006	1.43E-01	0.020	0.009	2.60E-02
0.952904575	0.010	0.005	7.23E-02	0.000	0.008	9.89E-01
5.833196	0.014	0.008	7.91E-02	0.011	0.012	3.72E-01
8.229942	0.009	0.007	2.11E-01	-0.011	0.010	2.88E-01
1.428620259	0.008	0.004	7.01E-02	0.006	0.007	3.57E-01
8.376626	0.014	0.009	9.94E-02	0.011	0.013	4.14E-01
7.033587	-0.023	0.011	2.66E-02	-0.017	0.016	2.88E-01
6.222112	0.013	0.009	1.27E-01	-0.005	0.013	7.04E-01
1.217677025	0.011	0.007	1.18E-01	0.006	0.010	5.35E-01
8.383691	0.018	0.010	6.55E-02	-0.009	0.015	5.53E-01
1.137942501	0.013	0.007	6.02E-02	-0.004	0.010	6.73E-01
8.233306	0.010	0.008	1.73E-01	0.017	0.011	1.43E-01
2.914643	-0.014	0.008	7.07E-02	-0.009	0.011	4.04E-01
5.126351	0.013	0.008	1.11E-01	-0.001	0.012	9.19E-01
2.868215	-0.012	0.009	1.61E-01	0.003	0.013	8.05E-01
3.856318	0.009	0.006	1.62E-01	0.021	0.009	2.46E-02
6.534321	-0.012	0.008	1.32E-01	0.004	0.012	7.46E-01
5.127024	0.011	0.007	1.24E-01	0.002	0.011	8.67E-01
3.501045	0.011	0.008	1.77E-01	0.033	0.012	6.79E-03

3.584144	-0.003	0.007	6.52E-01	0.005	0.010	6.05E-01
5.399534	0.011	0.006	7.16E-02	-0.006	0.009	5.21E-01
1.478412283	-0.010	0.006	1.30E-01	0.005	0.009	5.83E-01
5.425776	0.009	0.007	1.62E-01	0.003	0.010	7.43E-01
2.525726939	-0.017	0.010	7.84E-02	0.007	0.015	6.44E-01
5.039215	0.012	0.008	1.24E-01	0.003	0.012	8.16E-01
2.599405677	-0.026	0.017	1.14E-01	0.011	0.025	6.52E-01
7.011383	0.016	0.010	1.10E-01	-0.003	0.015	8.61E-01
6.578057	0.013	0.010	2.21E-01	0.000	0.015	9.77E-01
8.484621	0.011	0.008	1.58E-01	-0.002	0.012	8.41E-01
1.178314412	0.017	0.009	5.24E-02	-0.008	0.013	5.15E-01
8.086285	0.009	0.007	1.88E-01	0.018	0.010	6.92E-02
1.005724492	0.013	0.008	9.95E-02	0.012	0.012	3.12E-01
2.433207977	-0.015	0.010	1.39E-01	-0.031	0.015	3.93E-02
5.854727	0.014	0.009	1.09E-01	0.001	0.013	9.46E-01
5.820748	0.010	0.008	2.20E-01	0.009	0.013	4.71E-01
2.39855542	-0.020	0.011	7.58E-02	-0.011	0.016	5.11E-01
5.434523	0.013	0.008	1.16E-01	0.012	0.013	3.24E-01
7.063866	-0.013	0.010	1.72E-01	-0.026	0.014	7.31E-02
6.225813	0.014	0.008	6.88E-02	0.006	0.011	5.84E-01
1.196818204	0.007	0.005	1.16E-01	0.010	0.007	1.74E-01
1.842095914	-0.016	0.009	8.25E-02	-0.009	0.014	5.39E-01
7.614943	-0.019	0.010	7.12E-02	-0.018	0.016	2.35E-01
1.801051138	-0.017	0.010	1.00E-01	0.007	0.015	6.32E-01
6.709939	-0.016	0.009	7.52E-02	-0.030	0.013	2.36E-02
8.619867	0.009	0.007	1.56E-01	-0.002	0.010	8.72E-01
5.566405	0.014	0.009	1.21E-01	-0.001	0.014	9.53E-01
6.475109	0.010	0.007	1.47E-01	-0.007	0.011	5.34E-01
6.224467	0.013	0.009	1.46E-01	0.002	0.013	8.67E-01
3.618124	-0.009	0.006	1.65E-01	-0.001	0.009	9.09E-01
7.21627	-0.017	0.011	1.13E-01	-0.028	0.016	7.82E-02
0.545484708	-0.016	0.011	1.31E-01	-0.028	0.016	6.99E-02
3.047534	-0.013	0.007	6.33E-02	-0.015	0.010	1.45E-01
6.125556	0.012	0.008	1.28E-01	-0.001	0.012	9.18E-01
7.592738	0.013	0.009	1.21E-01	-0.001	0.013	9.54E-01
1.028265476	0.011	0.007	1.16E-01	0.006	0.010	5.69E-01
2.684523122	-0.012	0.006	4.08E-02	-0.004	0.009	6.08E-01
3.213732	-0.030	0.016	6.69E-02	-0.017	0.024	4.83E-01
2.873598	-0.013	0.009	1.47E-01	-0.005	0.014	6.94E-01
2.351791289	0.023	0.011	2.81E-02	0.013	0.016	4.04E-01
6.474773	0.011	0.007	1.16E-01	-0.008	0.011	4.58E-01
7.528816	-0.016	0.009	6.39E-02	0.000	0.013	9.92E-01
1.693729141	-0.006	0.004	1.44E-01	0.000	0.006	9.73E-01

7.402317	0.017	0.009	6.13E-02	-0.021	0.014	1.18E-01
8.427091	-0.015	0.010	1.31E-01	-0.005	0.014	7.26E-01
2.914979	-0.014	0.008	6.67E-02	-0.011	0.011	3.40E-01
6.297473	-0.013	0.008	8.60E-02	-0.007	0.011	5.25E-01
5.264625	-0.013	0.006	3.03E-02	-0.016	0.009	6.54E-02
3.534015	0.014	0.007	5.37E-02	0.027	0.011	1.20E-02
4.151033	-0.035	0.021	9.61E-02	0.009	0.032	7.70E-01
1.694402006	-0.007	0.004	9.22E-02	-0.004	0.006	5.45E-01
2.886383	-0.011	0.007	1.14E-01	-0.006	0.010	5.74E-01
3.25444	-0.014	0.009	1.18E-01	0.003	0.014	8.48E-01
5.091698	0.016	0.009	9.26E-02	0.006	0.014	6.80E-01
3.151155	-0.015	0.009	8.57E-02	-0.013	0.013	3.27E-01
5.667671	0.014	0.008	8.02E-02	0.019	0.012	1.22E-01
3.215077	-0.028	0.016	7.28E-02	-0.016	0.023	5.05E-01
3.129287	-0.016	0.009	7.78E-02	-0.010	0.014	4.87E-01
4.157089	-0.023	0.016	1.46E-01	0.007	0.023	7.71E-01
3.025329	-0.008	0.005	1.24E-01	-0.009	0.008	2.45E-01
1.848151701	-0.015	0.008	8.11E-02	-0.018	0.013	1.42E-01
3.526614	0.012	0.007	8.18E-02	0.025	0.010	1.71E-02
5.695931	0.011	0.009	2.13E-01	-0.002	0.013	8.72E-01
7.328639	-0.010	0.006	1.15E-01	0.002	0.009	8.51E-01
2.391490335	-0.012	0.007	1.05E-01	0.002	0.011	8.56E-01
1.146689748	0.012	0.006	4.67E-02	-0.001	0.009	9.35E-01
2.686205285	-0.013	0.006	3.34E-02	-0.002	0.009	8.37E-01
3.066711	-0.010	0.007	1.33E-01	-0.005	0.010	6.31E-01
0.860722046	0.018	0.010	7.22E-02	0.004	0.015	7.89E-01
6.730798	-0.024	0.012	4.27E-02	-0.030	0.018	9.16E-02
5.268325	-0.013	0.006	3.36E-02	-0.018	0.009	3.88E-02
3.116503	-0.018	0.010	5.96E-02	-0.005	0.014	7.25E-01
5.581208	0.014	0.008	9.81E-02	-0.004	0.013	7.35E-01
1.152072669	0.011	0.006	5.55E-02	-0.007	0.009	4.45E-01
8.464771	-0.011	0.007	1.45E-01	-0.021	0.011	6.18E-02
7.243521	-0.015	0.010	1.20E-01	-0.007	0.015	6.51E-01
8.837875	0.018	0.009	4.69E-02	0.002	0.014	8.65E-01
2.019059457	0.018	0.009	5.82E-02	0.001	0.014	9.63E-01
1.991135552	0.016	0.008	4.93E-02	-0.001	0.012	9.29E-01
5.399198	0.011	0.006	7.84E-02	-0.011	0.010	2.67E-01
1.261413262	0.026	0.014	6.75E-02	-0.004	0.021	8.66E-01
8.754776	-0.012	0.009	1.75E-01	0.006	0.014	6.56E-01
7.618307	-0.022	0.011	5.07E-02	-0.011	0.017	5.25E-01
1.932596281	0.008	0.005	9.04E-02	0.004	0.007	5.61E-01
0.930027159	-0.006	0.005	2.15E-01	-0.021	0.007	2.16E-03
2.386107414	-0.022	0.011	6.06E-02	0.012	0.017	4.76E-01

5.708043	0.013	0.009	1.42E-01	-0.010	0.013	4.40E-01
8.713059	0.014	0.008	9.38E-02	0.009	0.013	4.82E-01
5.639747	0.011	0.007	1.18E-01	-0.005	0.010	6.10E-01
6.530284	0.013	0.007	6.22E-02	0.004	0.011	7.15E-01
8.132713	0.012	0.009	1.65E-01	-0.015	0.013	2.63E-01
8.183514	0.013	0.009	1.47E-01	-0.014	0.013	3.04E-01
6.596225	-0.018	0.010	5.65E-02	0.000	0.014	9.90E-01
8.819035	0.012	0.008	1.32E-01	0.012	0.012	3.23E-01
0.812948618	0.006	0.005	2.25E-01	0.017	0.007	2.14E-02
1.412471495	0.009	0.005	4.94E-02	0.010	0.007	1.60E-01
0.940120137	-0.007	0.005	1.58E-01	-0.023	0.008	4.00E-03
3.07243	-0.011	0.006	1.03E-01	-0.011	0.010	2.43E-01
3.542426	0.010	0.008	1.80E-01	0.021	0.012	7.49E-02
3.073103	-0.011	0.007	1.05E-01	-0.011	0.010	2.61E-01
6.354666	-0.014	0.009	1.16E-01	0.006	0.013	6.39E-01
3.625862	-0.010	0.007	1.44E-01	0.006	0.011	5.92E-01
3.293466	-0.018	0.013	1.56E-01	-0.008	0.019	6.63E-01
6.307902	0.013	0.007	7.13E-02	-0.010	0.011	3.37E-01
8.474528	-0.014	0.010	1.49E-01	-0.004	0.014	7.70E-01
2.527072669	-0.019	0.011	7.95E-02	0.001	0.016	9.70E-01
6.216729	0.015	0.009	8.41E-02	-0.024	0.013	7.12E-02
6.078792	0.012	0.008	1.18E-01	0.010	0.012	3.99E-01
1.447124052	0.006	0.004	1.09E-01	0.005	0.006	3.98E-01
8.103443	0.014	0.008	8.30E-02	0.026	0.012	2.85E-02
5.508202	0.012	0.007	9.43E-02	-0.009	0.010	3.63E-01
5.105828	0.012	0.008	1.45E-01	-0.004	0.012	7.47E-01
5.499455	0.012	0.008	1.35E-01	-0.004	0.012	7.58E-01
3.967004	-0.015	0.008	7.36E-02	0.003	0.013	8.04E-01
2.048329092	0.013	0.007	6.50E-02	0.010	0.010	3.58E-01
0.81328505	0.006	0.005	2.23E-01	0.017	0.008	2.31E-02
8.986578	0.009	0.006	1.32E-01	-0.002	0.009	7.85E-01
5.267989	-0.013	0.006	3.43E-02	-0.018	0.009	4.41E-02
3.106073	-0.015	0.009	8.52E-02	-0.021	0.013	1.03E-01
5.254195	-0.011	0.005	2.61E-02	-0.010	0.008	2.04E-01
2.507223147	-0.022	0.013	9.89E-02	-0.017	0.019	3.92E-01
8.593289	-0.011	0.009	2.24E-01	0.015	0.014	2.59E-01
1.171249327	0.009	0.005	4.80E-02	-0.006	0.007	4.02E-01
2.256917299	0.014	0.008	6.01E-02	0.010	0.011	3.94E-01
2.26230022	0.011	0.006	8.52E-02	0.014	0.010	1.29E-01
3.376565	0.019	0.013	1.38E-01	0.003	0.019	8.69E-01
1.081758258	0.010	0.006	1.30E-01	-0.001	0.010	8.87E-01
7.158067	0.010	0.007	1.58E-01	-0.009	0.011	4.19E-01
5.854055	0.017	0.010	6.83E-02	0.006	0.014	6.99E-01

3.27328	-0.017	0.011	1.05E-01	0.010	0.016	5.32E-01
5.104146	0.012	0.007	8.25E-02	0.006	0.011	5.83E-01
7.071604	-0.017	0.010	8.52E-02	-0.017	0.014	2.33E-01
3.166631	-0.011	0.007	9.59E-02	0.014	0.010	1.62E-01
7.227036	-0.018	0.011	9.11E-02	-0.002	0.016	9.08E-01
5.811664	0.014	0.010	1.72E-01	-0.012	0.015	4.06E-01
7.926143	0.014	0.009	1.28E-01	0.005	0.014	7.42E-01
5.471194	0.009	0.006	1.29E-01	-0.009	0.009	3.44E-01
1.641245657	0.009	0.006	1.09E-01	0.006	0.009	5.07E-01
5.932107	0.009	0.006	1.41E-01	-0.008	0.009	3.79E-01
3.663878	-0.008	0.005	1.08E-01	0.009	0.008	2.44E-01
8.818362	0.012	0.009	1.98E-01	0.026	0.014	5.52E-02
1.633507707	0.013	0.007	5.63E-02	0.014	0.010	1.69E-01
8.867145	0.013	0.009	1.43E-01	0.008	0.013	5.51E-01
2.639104722	-0.016	0.008	6.48E-02	-0.001	0.013	9.36E-01
7.993766	0.014	0.008	7.98E-02	0.012	0.012	3.17E-01
4.420179	-0.023	0.015	1.17E-01	0.006	0.022	7.80E-01
1.92990482	0.007	0.004	9.73E-02	0.008	0.007	2.36E-01
2.223610472	0.024	0.016	1.41E-01	0.004	0.024	8.66E-01
6.414888	0.015	0.010	1.26E-01	0.003	0.015	8.17E-01
4.138921	-0.028	0.018	1.05E-01	0.011	0.026	6.89E-01
3.807872	0.009	0.006	1.41E-01	0.020	0.009	2.99E-02
8.161982	0.015	0.009	7.30E-02	0.024	0.013	5.63E-02
7.227709	-0.013	0.009	1.57E-01	-0.007	0.014	6.20E-01
1.655712258	0.006	0.004	1.45E-01	0.003	0.006	6.31E-01
6.604636	0.018	0.010	6.76E-02	0.009	0.015	5.57E-01
3.15923	-0.013	0.007	7.64E-02	-0.009	0.011	4.34E-01
0.809247859	0.006	0.005	2.40E-01	0.016	0.008	3.24E-02
7.612924	-0.029	0.016	8.25E-02	-0.043	0.025	7.85E-02
0.808911426	0.006	0.005	2.42E-01	0.016	0.008	3.56E-02
5.090353	0.012	0.009	1.66E-01	-0.004	0.013	7.62E-01
2.713119892	-0.012	0.006	3.25E-02	-0.016	0.009	7.25E-02
4.149351	-0.032	0.019	9.82E-02	0.014	0.028	6.30E-01
1.903326645	-0.010	0.006	1.02E-01	-0.004	0.009	6.45E-01
2.312092244	-0.012	0.008	1.06E-01	-0.005	0.011	6.62E-01
1.156782726	0.009	0.005	6.53E-02	-0.001	0.007	8.72E-01
0.808574994	0.006	0.005	2.52E-01	0.016	0.008	4.01E-02
0.635312209	0.012	0.009	1.85E-01	-0.002	0.013	8.53E-01
1.11573795	0.012	0.007	8.57E-02	-0.012	0.011	2.78E-01
7.884425	-0.010	0.007	1.23E-01	0.018	0.010	6.90E-02
5.525023	0.013	0.008	1.23E-01	-0.003	0.012	8.24E-01
0.594603866	-0.011	0.007	1.32E-01	0.005	0.011	6.53E-01
7.980981	-0.011	0.007	1.16E-01	-0.013	0.010	2.06E-01

1.802060436	-0.014	0.009	1.42E-01	0.007	0.014	6.08E-01
7.835979	-0.014	0.010	1.63E-01	0.011	0.015	4.58E-01
5.498109	0.013	0.008	1.09E-01	-0.008	0.012	4.82E-01
3.38363	0.012	0.010	2.27E-01	0.017	0.014	2.33E-01
4.149687	-0.033	0.019	8.99E-02	0.007	0.029	7.97E-01
3.588517	-0.007	0.006	2.15E-01	0.007	0.009	4.57E-01
7.326284	-0.012	0.007	8.82E-02	-0.003	0.010	7.98E-01
1.128522388	0.013	0.007	6.51E-02	-0.003	0.011	8.11E-01
6.831728	0.013	0.009	1.39E-01	0.016	0.013	2.25E-01
7.378767	-0.016	0.010	1.11E-01	-0.011	0.015	4.64E-01
5.868185	0.010	0.007	1.24E-01	-0.011	0.010	2.69E-01
8.386383	0.016	0.009	6.28E-02	0.009	0.013	5.15E-01
1.33004551	0.021	0.010	3.74E-02	0.010	0.015	4.85E-01
8.836529	0.008	0.007	2.68E-01	-0.008	0.011	4.46E-01
8.115218	0.015	0.010	1.32E-01	0.019	0.015	1.88E-01
8.497405	-0.017	0.009	6.68E-02	-0.012	0.014	3.93E-01
8.893723	0.014	0.009	1.48E-01	-0.006	0.014	6.62E-01
2.379715195	-0.012	0.007	9.16E-02	0.007	0.010	4.81E-01
2.765603377	-0.018	0.010	6.83E-02	-0.046	0.015	1.64E-03
1.960856619	0.008	0.005	9.63E-02	0.003	0.007	6.84E-01
5.25184	-0.012	0.005	2.36E-02	-0.010	0.008	1.88E-01
7.064203	-0.017	0.011	1.22E-01	-0.025	0.016	1.17E-01
1.015481037	0.019	0.011	7.13E-02	0.011	0.016	4.84E-01
6.780926	-0.008	0.007	2.45E-01	-0.002	0.011	8.50E-01
6.727434	-0.020	0.012	9.67E-02	-0.013	0.018	4.57E-01
7.617971	-0.027	0.015	6.55E-02	-0.007	0.022	7.43E-01
7.755908	-0.016	0.011	1.34E-01	-0.007	0.016	6.66E-01
7.830932	-0.015	0.010	1.44E-01	-0.009	0.015	5.30E-01
8.741992	0.014	0.009	1.06E-01	-0.013	0.013	3.20E-01
0.945839491	-0.009	0.005	7.99E-02	-0.004	0.008	5.77E-01
1.16519354	0.010	0.005	4.47E-02	0.001	0.007	8.53E-01
5.16134	0.017	0.010	8.05E-02	-0.016	0.015	2.92E-01
1.13659677	0.012	0.007	6.29E-02	-0.002	0.010	8.34E-01
3.268234	-0.014	0.009	1.22E-01	-0.013	0.013	3.41E-01
5.252177	-0.011	0.005	2.55E-02	-0.011	0.008	1.62E-01
5.495754	0.014	0.009	1.03E-01	0.001	0.013	9.16E-01
3.059309	-0.011	0.006	6.23E-02	-0.007	0.009	4.28E-01
7.213579	-0.012	0.008	1.28E-01	-0.015	0.012	2.27E-01
3.282027	-0.013	0.008	1.15E-01	-0.027	0.012	2.54E-02
6.523892	0.013	0.009	1.65E-01	0.006	0.014	6.72E-01
6.723733	-0.021	0.011	6.72E-02	-0.005	0.017	7.52E-01
0.690487154	0.027	0.016	9.38E-02	-0.001	0.024	9.74E-01
3.663542	-0.008	0.005	1.03E-01	0.008	0.008	3.11E-01

1.218013457	0.011	0.007	1.27E-01	0.006	0.010	5.80E-01
5.816038	0.012	0.007	1.03E-01	-0.004	0.011	7.50E-01
2.146567409	-0.024	0.012	5.80E-02	-0.006	0.019	7.30E-01
5.330902	0.033	0.020	9.09E-02	-0.017	0.029	5.69E-01
2.397546122	-0.022	0.014	1.16E-01	-0.005	0.021	8.07E-01
1.490860289	-0.007	0.004	8.03E-02	-0.005	0.006	4.31E-01
1.412135062	0.010	0.005	4.61E-02	0.007	0.007	3.39E-01
3.223488	-0.016	0.009	8.60E-02	0.000	0.014	9.90E-01
3.151828	-0.013	0.009	1.21E-01	-0.012	0.013	3.47E-01
5.253522	-0.012	0.005	2.49E-02	-0.011	0.008	1.44E-01
0.70158943	0.021	0.012	6.36E-02	0.025	0.017	1.46E-01
2.531782726	-0.016	0.012	1.88E-01	-0.014	0.018	4.26E-01
5.017347	0.009	0.006	1.31E-01	-0.004	0.009	6.84E-01
5.089343	0.014	0.009	1.37E-01	-0.001	0.014	9.44E-01
7.799644	-0.011	0.007	1.28E-01	0.005	0.011	6.59E-01
5.109193	0.012	0.009	1.64E-01	-0.006	0.013	6.65E-01
1.176968681	0.016	0.009	5.56E-02	-0.005	0.013	6.94E-01
1.139961096	0.013	0.007	6.17E-02	-0.007	0.010	5.12E-01
5.791478	0.012	0.008	1.28E-01	0.010	0.012	4.23E-01
3.549491	0.022	0.015	1.36E-01	0.054	0.022	1.34E-02
1.781201615	-0.016	0.012	1.56E-01	0.015	0.017	3.93E-01
6.088885	0.014	0.009	1.42E-01	-0.011	0.014	4.38E-01
4.163144	-0.021	0.014	1.47E-01	0.000	0.021	9.87E-01
8.703975	0.009	0.006	1.50E-01	0.016	0.009	8.82E-02
7.366992	-0.012	0.008	1.56E-01	0.004	0.013	7.34E-01
5.54891	0.012	0.008	1.11E-01	-0.014	0.012	2.17E-01
1.678589675	-0.005	0.004	1.74E-01	0.001	0.006	8.73E-01
0.960978958	0.008	0.004	8.85E-02	0.013	0.007	5.26E-02
3.069402	-0.009	0.006	1.35E-01	-0.007	0.009	4.29E-01
5.383385	0.015	0.008	6.73E-02	0.004	0.012	7.50E-01
2.300317103	-0.007	0.005	1.09E-01	-0.005	0.007	4.62E-01
3.657823	-0.008	0.005	1.09E-01	0.009	0.007	2.26E-01
4.280223	0.024	0.013	5.94E-02	0.022	0.019	2.44E-01
1.63518987	0.013	0.007	6.49E-02	0.002	0.010	8.13E-01
8.478902	0.009	0.007	1.67E-01	0.004	0.010	7.16E-01
3.041815	-0.008	0.006	1.70E-01	0.001	0.009	8.93E-01
8.07922	0.014	0.010	1.74E-01	0.010	0.015	5.37E-01
3.433086	0.013	0.009	1.43E-01	0.029	0.013	2.57E-02
2.193667972	0.020	0.011	7.47E-02	-0.014	0.017	3.98E-01
3.53738	0.013	0.008	1.12E-01	0.021	0.012	7.39E-02
6.50808	0.017	0.009	6.25E-02	0.049	0.014	4.01E-04
4.169873	-0.014	0.009	1.14E-01	0.001	0.014	9.42E-01
5.507865	0.010	0.007	1.54E-01	-0.006	0.011	5.71E-01

2.496793736	-0.022	0.014	1.16E-01	0.003	0.021	8.87E-01
4.161462	-0.023	0.014	1.07E-01	-0.028	0.021	1.94E-01
2.567781013	-0.017	0.010	8.01E-02	-0.010	0.015	5.17E-01
2.771995596	-0.018	0.010	8.37E-02	-0.046	0.015	2.67E-03
3.151492	-0.014	0.009	1.02E-01	-0.011	0.013	4.04E-01
6.768478	-0.009	0.006	1.41E-01	0.004	0.009	6.82E-01
8.113872	0.016	0.009	9.38E-02	0.007	0.014	6.39E-01
8.37528	-0.013	0.009	1.56E-01	0.014	0.014	3.18E-01
7.709817	-0.018	0.011	9.99E-02	-0.030	0.016	6.17E-02
1.423910203	0.010	0.005	4.25E-02	0.011	0.007	1.15E-01
3.586835	-0.007	0.006	2.37E-01	0.007	0.009	4.37E-01
7.611242	-0.028	0.017	1.01E-01	-0.038	0.025	1.33E-01
1.613658184	0.014	0.007	4.56E-02	-0.003	0.011	8.04E-01
5.454709	0.012	0.008	1.12E-01	-0.021	0.012	6.65E-02
2.608825789	-0.021	0.014	1.29E-01	0.029	0.020	1.52E-01
3.628553	-0.009	0.006	1.36E-01	0.012	0.009	1.81E-01
1.821909958	-0.014	0.009	1.33E-01	-0.004	0.014	7.65E-01
0.807902129	0.006	0.005	2.58E-01	0.015	0.008	4.90E-02
5.453363	0.013	0.010	1.88E-01	-0.030	0.015	4.12E-02
7.926816	0.013	0.010	2.05E-01	-0.015	0.015	3.22E-01
5.861456	0.017	0.010	9.18E-02	-0.004	0.015	7.87E-01
3.042824	-0.006	0.005	2.69E-01	-0.001	0.008	8.90E-01
0.553895522	0.012	0.008	1.12E-01	0.010	0.011	3.84E-01
6.585795	0.008	0.009	3.70E-01	-0.010	0.013	4.54E-01
7.017102	0.009	0.010	3.74E-01	0.022	0.014	1.25E-01
8.767561	0.013	0.009	1.58E-01	0.008	0.014	5.53E-01
1.317597504	0.020	0.009	2.80E-02	0.009	0.014	5.11E-01
1.858917543	-0.013	0.009	1.57E-01	0.004	0.013	7.75E-01
8.174767	-0.015	0.009	1.06E-01	0.016	0.014	2.38E-01
8.041876	0.016	0.009	6.65E-02	-0.002	0.013	8.89E-01
1.138615366	0.013	0.007	5.81E-02	-0.005	0.010	6.02E-01
5.413664	0.012	0.007	1.12E-01	0.006	0.011	5.80E-01
6.679324	0.010	0.009	2.66E-01	0.032	0.013	1.33E-02
2.147240274	-0.022	0.011	4.38E-02	0.003	0.016	8.60E-01
8.913236	0.010	0.008	2.30E-01	0.009	0.012	4.57E-01
7.325274	-0.012	0.007	1.05E-01	-0.001	0.011	8.97E-01
6.548451	-0.011	0.010	2.34E-01	0.007	0.014	6.05E-01
5.104819	0.020	0.013	1.06E-01	0.006	0.019	7.66E-01
5.760863	0.010	0.008	1.74E-01	0.004	0.011	7.22E-01
5.664643	0.010	0.008	1.97E-01	-0.006	0.012	6.27E-01
8.166356	0.013	0.009	1.52E-01	-0.008	0.014	5.47E-01
3.741258	0.009	0.008	2.55E-01	0.030	0.012	1.46E-02
2.404947639	-0.018	0.011	9.16E-02	0.002	0.016	9.20E-01

8.832492	0.014	0.009	1.46E-01	0.010	0.014	4.84E-01
7.500219	0.016	0.009	6.47E-02	0.031	0.013	1.87E-02
2.394181796	-0.016	0.009	7.23E-02	-0.002	0.013	8.85E-01
6.882529	0.017	0.010	6.99E-02	0.014	0.014	3.42E-01
5.269335	-0.012	0.006	3.92E-02	-0.018	0.009	4.05E-02
1.491196721	-0.007	0.004	8.72E-02	-0.006	0.006	3.44E-01
5.780712	0.013	0.008	1.19E-01	-0.010	0.013	4.41E-01
8.804232	0.012	0.007	7.78E-02	0.004	0.010	7.08E-01
7.107266	-0.014	0.011	2.11E-01	0.031	0.017	5.85E-02
6.699173	0.016	0.010	1.09E-01	0.032	0.015	2.89E-02
1.434676046	0.007	0.004	7.42E-02	0.010	0.006	1.06E-01
1.409780034	0.011	0.005	4.56E-02	0.014	0.008	9.45E-02
6.725751	-0.019	0.012	1.13E-01	-0.035	0.018	4.30E-02
7.325947	-0.013	0.007	7.04E-02	-0.006	0.011	5.84E-01
3.34494	-0.013	0.009	1.68E-01	0.002	0.014	8.61E-01
5.331238	0.033	0.020	8.95E-02	-0.019	0.029	5.16E-01
0.808238561	0.006	0.005	2.61E-01	0.015	0.008	4.96E-02
8.583869	0.009	0.007	1.65E-01	0.006	0.010	5.46E-01
3.668925	-0.006	0.004	1.47E-01	0.004	0.006	5.46E-01
8.832829	0.011	0.007	1.23E-01	-0.003	0.010	7.46E-01
3.042151	-0.007	0.006	1.93E-01	0.002	0.008	8.21E-01
2.194677269	0.020	0.012	8.43E-02	-0.009	0.017	5.98E-01
6.586468	0.012	0.009	1.77E-01	-0.007	0.013	5.75E-01
8.263585	-0.016	0.010	1.09E-01	-0.025	0.015	8.76E-02
3.674981	-0.007	0.005	1.40E-01	0.002	0.007	7.18E-01
2.671738684	-0.010	0.005	8.12E-02	0.007	0.008	4.11E-01
2.680485931	-0.011	0.005	4.37E-02	0.003	0.008	6.71E-01
8.64409	-0.012	0.008	1.33E-01	-0.018	0.012	1.35E-01
7.027868	-0.019	0.009	3.79E-02	-0.018	0.014	1.86E-01
0.846255444	-0.014	0.008	6.71E-02	-0.016	0.011	1.48E-01
8.082248	0.012	0.008	1.04E-01	0.015	0.011	1.95E-01
2.194004404	0.020	0.011	7.90E-02	-0.013	0.017	4.56E-01
8.382009	0.013	0.008	1.28E-01	0.022	0.013	7.39E-02
3.037777	-0.013	0.008	1.12E-01	-0.010	0.012	3.93E-01
0.706972351	0.013	0.009	1.14E-01	0.026	0.013	3.84E-02
8.464099	-0.012	0.007	8.97E-02	-0.013	0.011	2.27E-01
6.063652	0.009	0.007	1.76E-01	-0.007	0.010	4.82E-01
8.412624	-0.016	0.011	1.45E-01	-0.007	0.016	6.59E-01
5.547901	0.010	0.008	1.88E-01	-0.006	0.012	5.81E-01
0.813621483	0.006	0.005	2.32E-01	0.017	0.008	2.44E-02
1.156109861	0.009	0.005	8.19E-02	-0.003	0.007	7.11E-01
0.846928309	-0.014	0.007	6.70E-02	-0.016	0.011	1.44E-01
6.364086	-0.012	0.008	1.31E-01	-0.020	0.012	9.31E-02

8.924675	-0.013	0.009	1.66E-01	0.000	0.014	9.86E-01
7.573898	0.013	0.008	1.07E-01	0.001	0.012	9.21E-01
7.498537	0.012	0.010	2.07E-01	0.006	0.014	6.73E-01
1.261076829	0.025	0.014	7.52E-02	-0.004	0.021	8.30E-01
8.859743	0.013	0.009	1.63E-01	0.017	0.014	2.31E-01
2.55701517	-0.020	0.012	8.85E-02	-0.010	0.018	5.63E-01
7.644549	-0.014	0.011	1.87E-01	-0.014	0.016	3.73E-01
1.077384634	0.011	0.008	1.53E-01	0.007	0.011	5.58E-01
5.13678	0.011	0.008	1.41E-01	-0.003	0.011	7.82E-01
3.792732	0.012	0.006	4.58E-02	0.014	0.009	1.14E-01
2.35313702	0.019	0.010	4.33E-02	0.015	0.014	2.83E-01
2.561725226	-0.018	0.010	8.49E-02	-0.001	0.016	9.43E-01
5.462783	0.011	0.008	1.72E-01	-0.005	0.012	6.86E-01
6.896659	0.013	0.009	1.37E-01	-0.009	0.013	4.71E-01
5.926724	-0.014	0.008	7.06E-02	-0.001	0.012	9.42E-01
0.777286763	0.007	0.005	1.31E-01	0.000	0.007	9.62E-01
0.935746513	-0.007	0.005	1.57E-01	-0.019	0.008	1.05E-02
2.224283337	0.026	0.018	1.53E-01	0.004	0.026	8.88E-01
2.607143626	-0.017	0.012	1.40E-01	0.018	0.017	3.06E-01
5.01802	0.012	0.007	9.81E-02	-0.008	0.011	4.78E-01
6.264839	0.013	0.007	7.05E-02	-0.018	0.011	9.60E-02
1.844450942	-0.014	0.009	1.23E-01	-0.001	0.014	9.15E-01
1.433330316	0.009	0.004	3.75E-02	0.002	0.007	7.47E-01
3.157547	-0.013	0.008	9.73E-02	-0.008	0.011	5.11E-01
7.82454	-0.020	0.010	3.41E-02	0.009	0.014	5.27E-01
6.020925	0.011	0.008	1.70E-01	-0.011	0.012	3.70E-01
2.680822364	-0.011	0.005	4.37E-02	0.004	0.008	5.76E-01
3.482205	0.013	0.009	1.77E-01	0.036	0.014	1.09E-02
1.074020308	0.009	0.006	1.70E-01	0.000	0.009	9.95E-01
5.761536	0.014	0.010	1.72E-01	0.002	0.016	9.10E-01
3.382284	0.013	0.010	1.68E-01	0.020	0.014	1.71E-01
7.516032	-0.018	0.011	9.17E-02	-0.015	0.016	3.58E-01
8.206728	0.009	0.007	1.74E-01	0.003	0.010	7.38E-01
3.765481	0.018	0.010	7.18E-02	0.030	0.015	3.98E-02
2.38307952	-0.013	0.008	8.34E-02	0.008	0.011	4.98E-01
5.134425	0.014	0.008	9.06E-02	0.003	0.013	8.40E-01
6.885557	0.016	0.008	5.27E-02	0.001	0.012	9.28E-01
1.543680206	0.025	0.015	8.14E-02	-0.012	0.022	5.94E-01
7.717555	-0.016	0.010	1.35E-01	-0.024	0.016	1.22E-01
0.846591877	-0.014	0.007	6.82E-02	-0.016	0.011	1.46E-01
1.990799119	0.015	0.008	5.63E-02	-0.002	0.012	9.00E-01
1.809798385	-0.016	0.011	1.27E-01	-0.002	0.016	9.03E-01
8.487649	0.010	0.006	1.05E-01	0.014	0.010	1.44E-01

1.028938341	0.011	0.007	1.25E-01	0.014	0.010	1.78E-01
5.126687	0.012	0.008	1.33E-01	0.005	0.011	6.84E-01
5.921341	-0.017	0.009	7.00E-02	-0.006	0.014	6.81E-01
2.29998067	-0.007	0.005	1.14E-01	-0.007	0.007	3.04E-01
2.992023	-0.006	0.004	1.05E-01	0.005	0.006	3.78E-01
1.423237338	0.010	0.005	3.88E-02	0.011	0.007	1.22E-01
3.160912	-0.012	0.007	8.67E-02	-0.001	0.011	9.13E-01
3.223152	-0.017	0.010	9.01E-02	-0.001	0.015	9.66E-01
4.244897	0.030	0.017	8.61E-02	0.008	0.026	7.46E-01
6.292426	0.011	0.008	1.71E-01	0.014	0.012	2.43E-01
6.805822	0.014	0.010	1.44E-01	-0.007	0.014	6.36E-01
2.506550281	-0.023	0.013	8.99E-02	-0.024	0.020	2.25E-01
2.374668706	-0.026	0.015	8.85E-02	0.005	0.023	8.34E-01
7.216607	-0.019	0.011	7.94E-02	-0.020	0.016	2.25E-01
6.398739	0.011	0.009	2.06E-01	-0.008	0.013	5.18E-01
6.166264	0.011	0.007	1.38E-01	0.000	0.011	9.68E-01
2.713456325	-0.012	0.006	3.58E-02	-0.017	0.009	5.21E-02
5.844298	0.013	0.010	1.89E-01	0.011	0.014	4.52E-01
1.679598972	-0.006	0.004	1.23E-01	-0.001	0.006	8.37E-01
3.843197	0.009	0.006	1.48E-01	0.018	0.009	5.31E-02
2.378705897	-0.011	0.008	1.50E-01	0.018	0.012	1.32E-01
6.414551	0.016	0.010	9.70E-02	-0.001	0.014	9.57E-01
0.813957915	0.006	0.005	2.34E-01	0.017	0.008	2.46E-02
1.990126254	0.015	0.008	5.84E-02	-0.002	0.012	8.55E-01
7.327629	-0.011	0.006	8.62E-02	-0.006	0.010	5.58E-01
7.756244	-0.015	0.010	1.38E-01	-0.013	0.015	4.11E-01
3.770528	0.010	0.008	2.17E-01	0.014	0.012	2.45E-01
3.262514	0.011	0.008	1.35E-01	0.025	0.011	2.72E-02
2.977892	-0.006	0.004	1.39E-01	0.003	0.006	6.37E-01
3.326773	0.015	0.009	8.87E-02	0.017	0.013	2.07E-01
1.326681184	0.021	0.010	4.07E-02	0.010	0.016	5.35E-01
6.46367	0.013	0.009	1.39E-01	0.002	0.013	8.94E-01
2.643814779	-0.017	0.009	6.89E-02	0.010	0.014	4.95E-01
3.025666	-0.008	0.006	1.56E-01	-0.010	0.008	2.44E-01
2.654580621	-0.016	0.010	9.46E-02	0.006	0.015	6.66E-01
7.48037	0.012	0.009	1.59E-01	-0.009	0.013	4.86E-01
5.666325	0.012	0.009	1.89E-01	-0.004	0.014	7.78E-01
0.912196232	-0.008	0.005	1.33E-01	-0.025	0.007	1.02E-03
0.610079765	-0.011	0.008	1.89E-01	-0.003	0.013	8.04E-01
7.338395	-0.018	0.009	5.57E-02	-0.032	0.014	2.21E-02
7.327966	-0.009	0.006	1.50E-01	0.001	0.010	8.94E-01
3.560594	-0.013	0.009	1.19E-01	0.019	0.013	1.44E-01
2.509914607	-0.019	0.013	1.53E-01	-0.001	0.020	9.42E-01

6.249026	0.013	0.007	7.60E-02	0.004	0.011	7.10E-01
2.63876829	-0.015	0.009	7.19E-02	-0.005	0.013	7.11E-01
6.075091	0.016	0.010	1.00E-01	-0.009	0.015	5.50E-01
8.155926	0.012	0.008	1.61E-01	0.000	0.012	9.85E-01
0.847264742	-0.013	0.007	6.95E-02	-0.016	0.011	1.46E-01
8.703302	0.011	0.010	2.43E-01	0.011	0.014	4.64E-01
3.026002	-0.008	0.006	1.61E-01	-0.008	0.008	3.73E-01
3.58448	-0.003	0.007	6.47E-01	0.008	0.010	4.02E-01
7.946329	0.015	0.010	1.32E-01	0.028	0.014	4.80E-02
2.560379496	-0.015	0.009	9.42E-02	-0.004	0.013	7.80E-01
2.417395645	-0.012	0.010	2.42E-01	0.012	0.015	4.21E-01
3.283037	-0.013	0.009	1.29E-01	-0.011	0.013	3.99E-01
3.583135	-0.005	0.006	4.37E-01	0.001	0.009	9.16E-01
0.84827404	-0.013	0.007	7.16E-02	-0.016	0.011	1.46E-01
7.375739	-0.012	0.008	1.37E-01	-0.016	0.012	2.08E-01
3.44116	0.011	0.007	1.35E-01	0.025	0.011	2.00E-02
6.363077	0.011	0.008	1.64E-01	0.021	0.012	7.87E-02
7.980645	-0.013	0.008	8.36E-02	-0.017	0.011	1.40E-01
2.432871544	-0.014	0.010	1.66E-01	-0.028	0.016	7.12E-02
8.364178	0.016	0.009	8.12E-02	0.000	0.013	9.80E-01
8.570075	0.015	0.009	1.18E-01	0.026	0.014	6.14E-02
1.54166161	0.024	0.014	8.32E-02	-0.013	0.020	5.25E-01
7.168833	-0.014	0.011	1.98E-01	-0.031	0.017	6.28E-02
5.546555	0.014	0.008	9.36E-02	-0.009	0.012	4.53E-01
3.619133	-0.009	0.006	1.71E-01	-0.003	0.009	7.69E-01
2.425470027	-0.012	0.009	1.81E-01	0.018	0.013	1.66E-01
5.978871	0.013	0.008	1.10E-01	0.003	0.012	8.16E-01
7.613261	-0.026	0.015	7.29E-02	-0.026	0.022	2.24E-01
6.232205	0.012	0.009	1.91E-01	0.006	0.013	6.32E-01
1.078057499	0.012	0.008	1.14E-01	0.001	0.011	9.62E-01
8.692536	-0.015	0.011	1.68E-01	-0.025	0.016	1.10E-01
2.379378762	-0.011	0.007	1.17E-01	0.010	0.011	3.51E-01
0.847937607	-0.013	0.007	7.14E-02	-0.016	0.011	1.45E-01
4.445411	-0.023	0.016	1.47E-01	0.020	0.024	3.95E-01
1.624424027	0.012	0.006	7.31E-02	0.014	0.010	1.52E-01
5.087998	0.012	0.009	1.69E-01	-0.007	0.013	5.78E-01
7.392897	0.011	0.007	1.10E-01	-0.015	0.010	1.45E-01
5.891735	0.014	0.010	1.79E-01	0.011	0.015	4.68E-01
3.37892	0.016	0.013	2.03E-01	0.019	0.019	3.19E-01
3.045179	-0.008	0.006	1.77E-01	-0.007	0.008	4.02E-01
1.21834989	0.010	0.007	1.37E-01	0.005	0.011	6.07E-01
2.575855395	-0.012	0.010	2.22E-01	-0.009	0.015	5.41E-01
0.899748226	0.016	0.008	5.77E-02	-0.010	0.012	4.19E-01

2.592677025	-0.022	0.017	1.88E-01	0.011	0.025	6.64E-01
2.148922437	-0.015	0.008	5.97E-02	0.009	0.012	4.55E-01
3.774565	0.015	0.009	1.12E-01	0.036	0.014	1.05E-02
3.213395	-0.029	0.016	7.80E-02	-0.016	0.024	5.06E-01
6.895986	0.015	0.009	1.12E-01	-0.008	0.014	5.91E-01
8.109162	0.016	0.009	6.50E-02	0.016	0.013	2.42E-01
8.568393	0.013	0.008	1.01E-01	-0.005	0.012	6.77E-01
3.86271	-0.008	0.005	9.41E-02	0.003	0.008	6.47E-01
0.845919011	-0.014	0.008	7.06E-02	-0.016	0.011	1.58E-01
6.31093	0.018	0.010	8.01E-02	-0.009	0.015	5.41E-01
3.95321	-0.015	0.009	1.12E-01	-0.011	0.014	4.15E-01
5.264288	-0.012	0.006	3.83E-02	-0.015	0.009	7.33E-02
1.452506973	0.006	0.004	9.03E-02	-0.001	0.006	7.99E-01
7.518723	-0.018	0.009	6.15E-02	-0.009	0.014	5.01E-01
1.76236139	-0.016	0.009	7.57E-02	0.012	0.013	3.44E-01
1.477402985	-0.008	0.005	1.37E-01	0.003	0.008	6.92E-01
6.131275	0.010	0.007	1.62E-01	0.020	0.011	5.73E-02
8.150207	0.014	0.009	1.06E-01	0.022	0.013	9.80E-02
4.310838	-0.023	0.014	1.04E-01	0.023	0.021	2.76E-01
1.120111573	0.010	0.006	1.01E-01	0.003	0.009	7.25E-01
0.750372156	-0.009	0.005	1.09E-01	-0.015	0.008	5.82E-02
8.479911	-0.011	0.009	2.27E-01	-0.015	0.013	2.54E-01
8.954954	-0.013	0.009	1.33E-01	0.013	0.013	3.25E-01
5.254868	-0.011	0.005	2.96E-02	-0.009	0.008	2.25E-01
0.707981649	0.011	0.008	1.93E-01	0.005	0.012	6.63E-01
2.347754098	0.020	0.010	4.45E-02	0.013	0.014	3.77E-01
1.137606068	0.012	0.007	8.49E-02	-0.004	0.010	6.80E-01
7.785178	-0.010	0.006	8.73E-02	-0.003	0.009	7.71E-01
8.908862	0.013	0.008	1.02E-01	0.008	0.012	5.03E-01
8.325152	-0.014	0.009	1.34E-01	-0.024	0.014	8.53E-02
8.201681	0.012	0.008	1.66E-01	0.001	0.013	9.10E-01
2.096775385	-0.010	0.008	2.17E-01	-0.006	0.012	6.09E-01
7.559095	0.014	0.009	1.11E-01	0.015	0.014	2.54E-01
1.686327624	-0.006	0.004	9.92E-02	0.005	0.006	4.04E-01
1.472692929	-0.006	0.004	1.47E-01	0.000	0.006	9.56E-01
5.102128	0.014	0.009	1.39E-01	0.012	0.014	3.72E-01
3.223825	-0.015	0.009	9.72E-02	0.001	0.013	9.24E-01
0.704953756	0.018	0.010	7.98E-02	-0.001	0.015	9.32E-01
4.15036	-0.031	0.019	1.13E-01	0.013	0.029	6.42E-01
2.155987521	-0.012	0.009	1.65E-01	-0.010	0.013	4.29E-01
7.461193	0.016	0.011	1.28E-01	-0.015	0.016	3.46E-01
3.348305	-0.014	0.011	2.13E-01	-0.010	0.017	5.44E-01
6.159872	0.011	0.008	1.64E-01	0.019	0.012	1.19E-01

7.337049	-0.015	0.008	8.11E-02	0.006	0.012	6.42E-01
1.540988745	0.023	0.013	8.91E-02	-0.014	0.020	4.80E-01
3.224834	-0.013	0.008	1.07E-01	0.004	0.012	7.10E-01
6.529611	0.013	0.008	1.05E-01	0.010	0.012	3.72E-01
3.16293	-0.012	0.007	7.72E-02	-0.004	0.010	6.86E-01
7.060502	-0.014	0.011	1.78E-01	-0.017	0.016	2.80E-01
2.404611206	-0.018	0.011	9.10E-02	0.008	0.016	6.33E-01
0.542120382	-0.011	0.009	2.22E-01	0.009	0.013	4.79E-01
3.974069	-0.014	0.010	1.40E-01	0.005	0.014	7.13E-01
8.01227	-0.014	0.009	1.15E-01	-0.021	0.013	1.23E-01
1.486823098	-0.006	0.004	1.50E-01	-0.004	0.007	5.91E-01
7.529825	-0.015	0.009	8.69E-02	-0.002	0.013	8.75E-01
3.658159	-0.008	0.005	1.31E-01	0.009	0.007	2.07E-01
1.73511035	-0.008	0.005	1.07E-01	-0.006	0.008	4.68E-01
1.140633961	0.012	0.007	7.26E-02	-0.005	0.010	6.37E-01
6.900023	0.015	0.008	7.04E-02	-0.013	0.012	2.90E-01
8.742328	0.012	0.008	1.23E-01	0.003	0.012	8.17E-01
1.485140935	-0.007	0.004	1.14E-01	-0.003	0.007	6.03E-01
7.104575	-0.016	0.010	1.04E-01	0.002	0.014	8.84E-01
3.973396	-0.016	0.010	1.11E-01	-0.001	0.015	9.29E-01
7.183636	0.013	0.008	1.03E-01	-0.007	0.012	5.71E-01
2.883355	-0.013	0.009	1.17E-01	-0.001	0.013	9.56E-01
3.047197	-0.011	0.007	9.25E-02	-0.012	0.010	2.37E-01
5.853718	0.016	0.009	9.15E-02	0.016	0.014	2.45E-01
3.026675	-0.008	0.006	1.59E-01	-0.006	0.009	4.64E-01
5.255541	-0.011	0.005	3.10E-02	-0.009	0.008	2.20E-01
0.664581845	-0.014	0.009	1.24E-01	-0.037	0.014	6.64E-03
5.52536	0.013	0.008	1.16E-01	-0.002	0.012	8.44E-01
6.153143	0.012	0.008	1.46E-01	0.012	0.012	2.98E-01
4.419842	-0.021	0.014	1.36E-01	0.009	0.021	6.57E-01
6.463334	0.014	0.010	1.43E-01	0.022	0.014	1.13E-01
1.645619281	0.009	0.006	9.70E-02	0.009	0.008	2.66E-01
5.626963	0.010	0.009	2.82E-01	-0.005	0.014	7.25E-01
5.097418	0.014	0.009	1.20E-01	0.007	0.013	5.97E-01
2.563070957	-0.012	0.007	1.21E-01	-0.001	0.011	9.36E-01
1.861272572	-0.014	0.008	9.14E-02	0.011	0.012	3.55E-01
7.656324	0.012	0.008	1.15E-01	-0.003	0.011	8.18E-01
8.399167	0.017	0.010	8.62E-02	0.005	0.015	7.41E-01
5.00288	0.012	0.008	1.25E-01	0.002	0.012	8.30E-01
3.166967	-0.010	0.006	1.05E-01	0.013	0.010	1.71E-01
7.818821	-0.016	0.010	1.02E-01	-0.008	0.015	5.99E-01
5.743705	0.017	0.011	1.43E-01	0.001	0.017	9.47E-01
1.541998043	0.024	0.014	8.37E-02	-0.012	0.021	5.64E-01

0.917915586	-0.007	0.005	1.33E-01	-0.025	0.007	4.33E-04
8.354085	0.015	0.009	9.89E-02	-0.001	0.013	9.15E-01
2.128400049	-0.013	0.009	1.22E-01	0.001	0.013	9.35E-01
1.541325177	0.023	0.014	8.88E-02	-0.014	0.020	4.99E-01
2.644151211	-0.016	0.009	8.17E-02	0.015	0.014	2.76E-01
3.04787	-0.013	0.007	6.41E-02	-0.014	0.011	1.85E-01
1.694738439	-0.006	0.004	1.42E-01	0.000	0.006	9.98E-01
4.281569	0.020	0.012	7.78E-02	0.019	0.017	2.69E-01
5.252513	-0.011	0.005	2.90E-02	-0.011	0.008	1.55E-01
6.749975	-0.012	0.011	2.71E-01	0.016	0.016	3.01E-01
3.040805	-0.008	0.006	1.58E-01	0.004	0.009	6.06E-01
5.134089	0.013	0.008	1.17E-01	-0.003	0.012	8.21E-01
1.835030829	-0.013	0.009	1.46E-01	-0.028	0.013	3.82E-02
5.823776	0.012	0.007	9.86E-02	0.010	0.011	3.59E-01
1.990462687	0.015	0.008	6.06E-02	-0.002	0.012	8.60E-01
0.847601174	-0.013	0.007	7.36E-02	-0.016	0.011	1.50E-01
3.116839	-0.017	0.009	6.88E-02	-0.010	0.014	4.62E-01
0.689814289	0.027	0.016	9.30E-02	0.001	0.024	9.55E-01
2.998078	-0.006	0.004	1.07E-01	0.001	0.006	8.46E-01
5.148555	0.013	0.009	1.37E-01	-0.011	0.013	4.03E-01
1.720980181	-0.010	0.006	7.64E-02	-0.001	0.008	9.04E-01
8.271323	0.010	0.009	2.52E-01	0.013	0.013	3.12E-01
4.150023	-0.031	0.019	1.11E-01	0.013	0.029	6.39E-01
8.646782	0.009	0.006	1.27E-01	0.009	0.009	3.03E-01
3.548818	0.020	0.013	1.06E-01	0.037	0.019	4.81E-02
1.542670908	0.024	0.014	8.42E-02	-0.010	0.021	6.25E-01
3.224497	-0.013	0.008	1.06E-01	0.004	0.012	7.51E-01
4.128492	-0.019	0.012	1.31E-01	0.012	0.019	5.19E-01
3.587845	-0.007	0.006	2.48E-01	0.005	0.009	5.46E-01
1.542334475	0.024	0.014	8.15E-02	-0.010	0.021	6.19E-01
1.694065574	-0.006	0.004	1.45E-01	-0.004	0.006	4.72E-01
0.951895278	0.008	0.005	9.22E-02	-0.003	0.007	6.82E-01
1.422900905	0.010	0.005	3.81E-02	0.011	0.007	1.16E-01
3.382621	0.012	0.010	2.06E-01	0.014	0.014	3.17E-01
2.99135	-0.007	0.004	9.18E-02	0.002	0.006	7.58E-01
1.260740396	0.024	0.014	8.19E-02	-0.005	0.020	8.00E-01
0.75743724	0.009	0.005	9.55E-02	0.000	0.008	9.51E-01
5.253186	-0.011	0.005	2.89E-02	-0.012	0.008	1.17E-01
2.761566185	-0.016	0.009	8.63E-02	-0.039	0.014	4.63E-03
7.755235	-0.015	0.011	2.00E-01	-0.020	0.017	2.47E-01
7.358245	-0.016	0.010	1.16E-01	-0.025	0.016	1.04E-01
6.874791	0.017	0.010	9.26E-02	0.001	0.015	9.32E-01
2.606470761	-0.020	0.014	1.50E-01	0.002	0.020	9.18E-01

1.70920504	-0.007	0.005	1.06E-01	-0.002	0.007	7.18E-01
6.154825	0.011	0.008	1.71E-01	-0.020	0.012	1.02E-01
3.391368	0.011	0.009	2.41E-01	0.043	0.014	1.69E-03
1.894915831	-0.012	0.007	9.30E-02	-0.008	0.010	4.59E-01
5.155957	0.015	0.009	8.60E-02	-0.001	0.013	9.68E-01
6.800776	0.015	0.009	9.81E-02	0.001	0.013	9.52E-01
3.284719	-0.018	0.011	1.07E-01	-0.010	0.017	5.67E-01
3.296494	-0.016	0.011	1.57E-01	-0.007	0.016	6.68E-01
7.494836	-0.012	0.009	2.00E-01	-0.013	0.014	3.73E-01
1.024228285	0.008	0.005	1.14E-01	0.009	0.008	2.42E-01
3.240983	0.008	0.007	2.43E-01	0.021	0.010	2.85E-02
7.039643	-0.017	0.011	1.31E-01	0.007	0.017	6.65E-01
2.194340837	0.020	0.012	8.48E-02	-0.011	0.017	5.32E-01
0.848610472	-0.013	0.007	7.64E-02	-0.015	0.011	1.50E-01
7.182627	0.015	0.008	7.93E-02	-0.008	0.012	5.39E-01
2.982602	-0.006	0.004	1.61E-01	0.001	0.006	8.83E-01
6.78059	-0.009	0.008	2.46E-01	-0.004	0.012	7.21E-01
7.412747	-0.018	0.010	8.75E-02	0.001	0.015	9.38E-01
3.500372	0.011	0.008	1.63E-01	0.037	0.012	2.13E-03
5.08968	0.012	0.008	1.49E-01	0.000	0.012	9.96E-01
2.019395889	0.017	0.009	7.23E-02	0.001	0.014	9.71E-01
1.888187179	-0.010	0.006	1.09E-01	0.001	0.009	9.27E-01
8.133722	0.011	0.009	2.32E-01	-0.034	0.014	1.28E-02
6.962936	-0.015	0.010	1.28E-01	-0.007	0.015	6.44E-01
3.392041	0.011	0.009	2.38E-01	0.045	0.013	9.26E-04
5.152256	0.013	0.009	1.43E-01	-0.007	0.013	6.17E-01
3.027011	-0.008	0.006	1.57E-01	-0.006	0.008	4.89E-01
1.0625816	0.009	0.006	1.24E-01	-0.003	0.009	7.15E-01
6.742237	-0.019	0.012	1.24E-01	-0.041	0.018	2.21E-02
2.57652826	-0.011	0.010	2.51E-01	-0.008	0.015	5.85E-01
8.426082	0.013	0.008	1.18E-01	0.006	0.013	6.18E-01
4.139258	-0.029	0.019	1.29E-01	0.015	0.028	5.98E-01
5.445962	0.015	0.009	1.00E-01	0.000	0.013	9.94E-01
1.054170785	0.009	0.006	1.30E-01	-0.007	0.009	4.53E-01
3.337875	-0.014	0.009	1.32E-01	0.006	0.014	6.55E-01
5.925378	-0.013	0.010	1.76E-01	-0.005	0.014	7.40E-01
8.988597	0.012	0.009	1.80E-01	-0.011	0.013	3.96E-01
7.19743	0.014	0.008	9.99E-02	0.002	0.012	8.81E-01
3.973733	-0.015	0.010	1.31E-01	0.003	0.015	8.63E-01
7.212906	-0.014	0.009	1.19E-01	-0.001	0.014	9.53E-01
1.435685344	0.007	0.004	1.05E-01	0.004	0.006	4.85E-01
3.215414	-0.025	0.015	9.31E-02	-0.014	0.023	5.45E-01
0.538756056	0.011	0.007	1.20E-01	0.018	0.011	9.60E-02

2.04799266	0.012	0.007	7.93E-02	0.010	0.010	3.37E-01
7.73875	-0.014	0.011	2.03E-01	-0.005	0.017	7.52E-01
3.696512	0.008	0.006	1.47E-01	0.022	0.008	8.21E-03
1.54300734	0.024	0.014	8.75E-02	-0.011	0.021	6.07E-01
0.860385613	0.017	0.010	9.07E-02	0.003	0.015	8.62E-01
6.312612	0.010	0.007	1.54E-01	0.004	0.011	7.20E-01
0.582492293	-0.009	0.010	3.85E-01	-0.001	0.015	9.62E-01
0.845582579	-0.014	0.008	7.50E-02	-0.016	0.012	1.67E-01
2.373659408	-0.014	0.009	1.17E-01	0.012	0.013	3.51E-01
6.197889	0.014	0.008	8.70E-02	0.012	0.012	3.21E-01
5.108184	0.011	0.008	1.78E-01	-0.017	0.012	1.38E-01
8.484957	0.013	0.009	1.45E-01	-0.016	0.013	2.28E-01
8.425745	0.011	0.008	2.11E-01	0.009	0.013	4.64E-01
5.328547	0.031	0.019	1.04E-01	-0.017	0.029	5.43E-01
6.702201	-0.016	0.010	9.59E-02	-0.039	0.014	6.59E-03
1.913083191	-0.014	0.008	6.56E-02	-0.025	0.011	2.68E-02
6.191496	0.014	0.009	1.33E-01	0.000	0.014	9.76E-01
5.790132	0.009	0.007	2.17E-01	0.002	0.011	8.69E-01
6.310594	0.017	0.010	7.85E-02	-0.001	0.014	9.33E-01
5.81301	0.011	0.009	2.25E-01	0.004	0.013	7.57E-01
8.687153	0.009	0.007	1.87E-01	0.003	0.010	7.50E-01
7.283893	-0.016	0.010	1.08E-01	0.006	0.015	6.99E-01
7.7189	-0.016	0.010	1.21E-01	-0.023	0.015	1.30E-01
1.760679227	-0.015	0.008	6.65E-02	-0.013	0.012	3.00E-01
8.134058	0.009	0.008	2.59E-01	-0.022	0.011	5.13E-02
7.455137	-0.010	0.009	2.45E-01	-0.017	0.013	1.78E-01
0.990248593	0.009	0.007	1.85E-01	0.012	0.010	2.38E-01
6.653082	-0.011	0.007	1.44E-01	-0.014	0.011	2.22E-01
1.158128456	0.009	0.005	6.40E-02	-0.003	0.007	6.24E-01
8.388065	0.016	0.009	8.34E-02	0.004	0.014	7.57E-01
1.696084169	-0.006	0.004	1.76E-01	0.001	0.007	8.32E-01
3.680364	-0.006	0.005	2.02E-01	0.011	0.007	1.01E-01
8.790775	0.015	0.009	8.82E-02	-0.011	0.013	4.14E-01
1.613994617	0.013	0.007	6.39E-02	0.002	0.011	8.65E-01
2.654244189	-0.017	0.010	1.05E-01	0.010	0.015	5.15E-01
7.315518	-0.009	0.007	1.55E-01	-0.007	0.010	5.10E-01
5.664307	0.009	0.007	2.23E-01	0.002	0.011	8.59E-01
3.664215	-0.008	0.005	1.40E-01	0.010	0.008	1.86E-01
5.873904	0.011	0.008	1.57E-01	0.002	0.012	8.69E-01
7.357235	-0.015	0.010	1.44E-01	-0.007	0.015	6.51E-01
3.663206	-0.008	0.005	1.10E-01	0.008	0.007	2.60E-01
2.418404943	-0.012	0.012	3.07E-01	0.012	0.018	4.93E-01
7.172534	-0.015	0.011	1.71E-01	-0.041	0.017	1.53E-02

0.783006117	0.006	0.004	1.72E-01	0.006	0.007	3.46E-01
5.776002	0.012	0.008	1.35E-01	0.001	0.012	9.33E-01
2.311755811	-0.014	0.009	1.14E-01	-0.004	0.014	7.55E-01
6.921219	-0.014	0.008	1.02E-01	0.011	0.012	3.69E-01
7.032241	-0.014	0.008	8.98E-02	-0.010	0.012	4.36E-01
7.044017	-0.020	0.013	1.18E-01	0.014	0.019	4.49E-01
6.711958	-0.016	0.009	8.01E-02	-0.007	0.013	5.90E-01
3.129623	-0.016	0.009	8.16E-02	-0.013	0.014	3.61E-01
3.61846	-0.008	0.006	2.03E-01	0.001	0.009	9.38E-01
8.25181	0.011	0.008	1.63E-01	0.015	0.012	2.14E-01
6.536676	0.012	0.008	1.46E-01	0.003	0.012	8.09E-01
5.660269	0.011	0.008	1.67E-01	-0.006	0.012	5.94E-01
3.222815	-0.017	0.010	9.68E-02	-0.001	0.015	9.49E-01
7.860875	0.010	0.009	2.64E-01	0.003	0.013	7.92E-01
5.163358	0.018	0.011	9.67E-02	-0.020	0.016	2.29E-01
1.116410815	0.012	0.007	8.44E-02	-0.015	0.011	1.43E-01
5.772638	0.012	0.009	1.64E-01	-0.007	0.013	5.70E-01
8.042212	0.014	0.009	1.08E-01	-0.022	0.013	9.01E-02
3.026339	-0.008	0.006	1.67E-01	-0.006	0.009	4.68E-01
5.694922	0.013	0.008	1.14E-01	-0.014	0.012	2.65E-01
1.488505261	-0.007	0.004	9.07E-02	-0.010	0.007	1.15E-01
1.669505995	0.005	0.004	2.11E-01	0.003	0.006	5.59E-01
8.643754	-0.009	0.006	1.28E-01	-0.021	0.009	1.55E-02
8.836193	0.011	0.008	1.90E-01	0.007	0.012	5.45E-01
7.341423	0.012	0.009	1.44E-01	-0.003	0.013	8.44E-01
0.613444091	-0.012	0.009	1.72E-01	-0.020	0.013	1.28E-01
3.065701	-0.010	0.008	1.77E-01	-0.003	0.011	8.25E-01
8.498751	-0.016	0.010	9.46E-02	-0.014	0.015	3.27E-01
5.569769	0.012	0.009	1.77E-01	0.006	0.013	6.46E-01
0.603687546	0.012	0.009	1.80E-01	-0.015	0.013	2.52E-01
1.475047957	-0.007	0.004	1.23E-01	-0.002	0.006	7.21E-01
7.239484	-0.015	0.009	8.94E-02	-0.018	0.013	1.70E-01
3.348641	-0.014	0.011	2.14E-01	-0.009	0.016	6.02E-01
5.62629	0.013	0.010	1.76E-01	0.001	0.015	9.25E-01
5.728565	0.011	0.009	1.94E-01	-0.018	0.013	1.60E-01
8.699938	0.013	0.008	1.14E-01	0.015	0.012	2.24E-01
0.807565696	0.006	0.005	2.86E-01	0.015	0.008	5.73E-02
8.658893	0.014	0.009	1.26E-01	-0.020	0.013	1.40E-01
8.402531	0.012	0.009	1.89E-01	0.005	0.014	7.25E-01
3.975079	-0.012	0.009	2.03E-01	0.007	0.014	6.20E-01
6.80414	0.009	0.009	2.77E-01	0.006	0.013	6.42E-01
1.81047125	-0.012	0.009	1.86E-01	0.003	0.014	8.10E-01
7.394579	0.012	0.007	1.08E-01	-0.005	0.011	6.64E-01

8.579159	0.010	0.007	1.61E-01	-0.001	0.010	8.89E-01
6.129256	0.007	0.008	3.43E-01	-0.007	0.012	5.50E-01
1.693392709	-0.006	0.004	1.54E-01	0.003	0.006	5.89E-01
3.110783	-0.019	0.010	6.51E-02	-0.010	0.015	5.20E-01
2.760556888	-0.016	0.009	8.44E-02	-0.037	0.014	6.24E-03
0.612098361	0.016	0.010	1.12E-01	0.028	0.015	5.24E-02
3.674644	-0.006	0.005	1.64E-01	0.002	0.007	7.92E-01
0.937092244	-0.007	0.005	1.81E-01	-0.020	0.008	8.86E-03
8.041539	0.012	0.008	1.56E-01	0.003	0.012	8.40E-01
0.626564962	0.012	0.007	9.66E-02	0.005	0.011	6.46E-01
3.587172	-0.007	0.006	2.56E-01	0.006	0.009	5.02E-01
1.540652312	0.022	0.013	9.64E-02	-0.015	0.020	4.61E-01
4.281232	0.021	0.012	7.96E-02	0.015	0.018	4.10E-01
2.270374602	0.010	0.006	9.70E-02	0.009	0.009	3.34E-01
4.326987	-0.020	0.014	1.51E-01	0.015	0.021	4.89E-01
4.166845	-0.021	0.014	1.22E-01	-0.005	0.021	8.13E-01
2.772332028	-0.017	0.010	9.70E-02	-0.047	0.015	2.62E-03
3.9014	0.008	0.006	1.52E-01	0.025	0.009	5.33E-03
1.777164424	-0.015	0.011	1.64E-01	-0.005	0.016	7.77E-01
6.691099	-0.011	0.010	2.54E-01	-0.007	0.014	6.19E-01
7.312826	-0.015	0.009	8.41E-02	-0.011	0.013	3.82E-01
3.383294	0.011	0.009	2.49E-01	0.017	0.014	2.28E-01
3.589863	-0.007	0.006	1.97E-01	-0.002	0.009	8.37E-01
2.874944	-0.010	0.009	2.28E-01	0.004	0.013	7.43E-01
5.085979	0.013	0.009	1.61E-01	-0.011	0.014	4.46E-01
2.655926352	-0.014	0.009	1.22E-01	0.017	0.013	1.91E-01
7.828914	-0.016	0.010	1.04E-01	0.008	0.015	5.62E-01
7.744469	-0.015	0.011	1.70E-01	-0.027	0.016	8.16E-02
0.729849768	-0.012	0.007	8.97E-02	-0.023	0.011	3.15E-02
3.106746	-0.016	0.009	8.02E-02	-0.022	0.014	1.15E-01
5.591301	0.011	0.008	1.79E-01	0.000	0.012	9.91E-01
1.96489381	0.007	0.005	1.19E-01	0.000	0.007	9.95E-01
5.163022	0.017	0.010	9.73E-02	-0.013	0.015	3.93E-01
8.551235	0.015	0.010	1.31E-01	0.013	0.015	3.90E-01
3.112129	-0.018	0.010	7.91E-02	-0.002	0.015	9.05E-01
2.301999266	-0.006	0.004	1.63E-01	-0.003	0.006	6.30E-01
2.639441155	-0.015	0.009	9.22E-02	0.000	0.013	9.69E-01
3.660514	-0.008	0.005	8.51E-02	0.004	0.007	5.12E-01
8.593625	-0.011	0.008	1.57E-01	0.001	0.012	9.63E-01
5.149901	0.011	0.007	1.05E-01	-0.009	0.010	3.75E-01
3.059645	-0.011	0.006	7.86E-02	-0.005	0.009	6.13E-01
5.997038	0.018	0.010	8.07E-02	0.005	0.015	7.61E-01
7.16917	-0.014	0.009	1.25E-01	-0.023	0.014	1.09E-01

2.842310007	-0.012	0.008	1.36E-01	-0.007	0.012	5.45E-01
7.184309	0.013	0.008	1.34E-01	-0.013	0.013	2.90E-01
3.320717	0.016	0.012	1.66E-01	0.012	0.018	4.88E-01
3.549828	0.020	0.015	1.79E-01	0.058	0.023	1.01E-02
5.469512	-0.012	0.008	1.20E-01	-0.015	0.012	2.12E-01
8.9166	-0.008	0.007	2.45E-01	-0.008	0.010	4.40E-01
8.439875	0.013	0.009	1.23E-01	-0.009	0.013	4.83E-01
3.349314	-0.013	0.010	2.10E-01	-0.012	0.015	4.43E-01
3.132651	-0.016	0.009	7.89E-02	-0.019	0.013	1.55E-01
1.782210913	-0.013	0.009	1.56E-01	-0.005	0.013	7.10E-01
2.044291901	0.011	0.006	8.44E-02	0.006	0.009	4.94E-01
7.571207	0.016	0.009	7.05E-02	0.000	0.013	9.77E-01
1.654030095	0.006	0.005	1.96E-01	0.014	0.007	3.43E-02
6.241625	0.011	0.009	2.02E-01	-0.009	0.013	5.07E-01
7.535545	-0.017	0.010	1.04E-01	-0.019	0.016	2.21E-01
1.720643748	-0.010	0.006	8.45E-02	-0.002	0.009	8.49E-01
8.700947	0.010	0.008	2.02E-01	0.005	0.011	6.54E-01
8.914918	0.013	0.008	1.12E-01	0.015	0.012	2.35E-01
1.735446782	-0.009	0.005	9.06E-02	-0.001	0.008	8.71E-01
7.891154	0.016	0.010	1.09E-01	0.020	0.015	1.81E-01
3.78163	0.012	0.009	1.79E-01	0.018	0.013	1.71E-01
5.915958	0.011	0.008	1.56E-01	0.010	0.011	4.06E-01
2.371640812	-0.011	0.007	1.23E-01	0.006	0.010	5.98E-01
0.845246146	-0.014	0.008	7.88E-02	-0.016	0.012	1.81E-01
5.0557	0.013	0.009	1.41E-01	-0.003	0.013	8.30E-01
3.499699	0.011	0.008	1.83E-01	0.034	0.012	4.21E-03
0.938437974	-0.007	0.005	2.10E-01	-0.021	0.008	8.39E-03
7.381122	-0.011	0.009	1.86E-01	-0.001	0.013	9.17E-01
6.352311	0.009	0.008	2.33E-01	-0.013	0.011	2.69E-01
2.761229753	-0.016	0.009	9.26E-02	-0.040	0.014	4.05E-03
7.716882	-0.015	0.010	1.31E-01	0.002	0.015	9.05E-01
1.218686322	0.010	0.007	1.51E-01	0.005	0.011	6.29E-01
4.167854	-0.019	0.014	1.57E-01	0.009	0.020	6.74E-01
7.840353	-0.011	0.007	1.39E-01	0.010	0.011	3.43E-01
8.683453	-0.009	0.008	2.59E-01	0.004	0.012	7.64E-01
6.342218	-0.015	0.009	9.69E-02	-0.014	0.013	2.90E-01
3.676999	-0.006	0.004	1.80E-01	0.004	0.007	5.23E-01
2.306036457	-0.008	0.005	1.13E-01	-0.007	0.007	3.48E-01
1.824937852	-0.016	0.010	1.26E-01	-0.004	0.015	8.02E-01
8.768907	-0.017	0.010	9.51E-02	-0.017	0.015	2.78E-01
2.604452165	-0.021	0.013	1.14E-01	0.014	0.019	4.83E-01
6.519518	-0.012	0.009	1.62E-01	-0.027	0.013	3.49E-02
5.249485	-0.011	0.005	2.93E-02	-0.009	0.007	2.19E-01

7.732021	-0.014	0.011	2.09E-01	0.003	0.017	8.78E-01
7.955413	0.011	0.007	9.93E-02	0.001	0.010	9.27E-01
3.974406	-0.013	0.010	1.78E-01	0.006	0.014	6.78E-01
5.625953	0.008	0.009	3.34E-01	-0.003	0.013	8.20E-01
6.961254	-0.015	0.008	6.44E-02	-0.013	0.012	2.91E-01
7.835643	-0.013	0.010	1.83E-01	0.022	0.015	1.35E-01
6.801112	0.013	0.010	1.72E-01	-0.010	0.014	4.81E-01
1.260403964	0.023	0.013	8.90E-02	-0.006	0.020	7.78E-01
8.408251	0.016	0.009	8.50E-02	-0.016	0.014	2.48E-01
5.5008	0.010	0.008	1.73E-01	-0.009	0.011	4.32E-01
0.690150722	0.026	0.016	1.04E-01	-0.006	0.024	8.18E-01
0.937428676	-0.007	0.005	1.93E-01	-0.020	0.008	1.08E-02
5.732939	0.010	0.009	2.55E-01	-0.013	0.013	3.24E-01
5.471867	0.009	0.008	2.59E-01	-0.010	0.012	3.98E-01
5.028785	0.012	0.009	1.70E-01	-0.009	0.013	5.19E-01
7.56145	0.013	0.009	1.58E-01	0.020	0.013	1.35E-01
0.661890384	-0.013	0.010	1.81E-01	-0.028	0.014	5.25E-02
7.162104	0.014	0.009	1.22E-01	-0.010	0.014	4.90E-01
8.886322	0.012	0.009	1.58E-01	-0.001	0.013	9.23E-01
1.168557866	0.009	0.004	4.52E-02	0.000	0.007	9.52E-01
3.537043	0.012	0.008	1.33E-01	0.020	0.012	8.09E-02
5.25285	-0.011	0.005	3.15E-02	-0.011	0.008	1.50E-01
8.126993	0.009	0.010	3.83E-01	0.003	0.015	8.53E-01
2.416049914	-0.011	0.009	1.91E-01	0.002	0.013	8.53E-01
3.727464	0.009	0.008	2.42E-01	0.024	0.012	4.07E-02
5.651522	0.013	0.008	1.19E-01	-0.003	0.012	8.34E-01
1.92855909	0.007	0.004	1.08E-01	0.004	0.006	5.69E-01
3.630235	-0.008	0.006	1.78E-01	0.013	0.009	1.39E-01
1.543343773	0.024	0.014	9.43E-02	-0.012	0.021	5.92E-01
0.848946905	-0.012	0.007	8.44E-02	-0.015	0.011	1.59E-01
7.741778	-0.017	0.012	1.73E-01	-0.026	0.018	1.59E-01
5.269671	-0.012	0.006	4.74E-02	-0.019	0.009	3.95E-02
8.234652	0.015	0.009	1.06E-01	-0.002	0.013	9.09E-01
3.131978	-0.016	0.009	5.99E-02	-0.023	0.013	7.33E-02
1.191435283	0.007	0.005	1.19E-01	0.002	0.007	7.89E-01
3.049552	-0.015	0.008	5.75E-02	-0.001	0.012	9.45E-01
2.652898459	-0.018	0.012	1.31E-01	0.017	0.018	3.30E-01
2.773341326	-0.017	0.010	1.05E-01	-0.048	0.016	2.02E-03
7.324265	-0.012	0.006	5.47E-02	0.009	0.010	3.47E-01
7.338059	-0.015	0.009	1.09E-01	-0.034	0.014	1.45E-02
6.521537	0.014	0.009	1.11E-01	0.014	0.014	3.14E-01
6.250709	0.010	0.007	1.24E-01	0.003	0.010	7.93E-01
5.566741	0.012	0.007	1.01E-01	0.006	0.011	6.05E-01

2.338333986	0.015	0.008	6.02E-02	0.011	0.012	3.66E-01
6.608	-0.016	0.008	5.60E-02	-0.013	0.012	2.89E-01
3.544781	0.016	0.010	1.14E-01	0.019	0.015	1.99E-01
1.185715929	0.008	0.005	9.70E-02	0.006	0.007	3.94E-01
6.144059	-0.012	0.010	1.93E-01	-0.021	0.014	1.36E-01
8.202354	0.012	0.008	1.38E-01	-0.022	0.012	6.92E-02
8.951589	-0.010	0.008	1.74E-01	-0.014	0.011	2.24E-01
8.161646	0.012	0.009	1.87E-01	0.027	0.014	4.67E-02
2.351454857	0.021	0.011	4.38E-02	0.013	0.016	3.97E-01
3.511138	0.011	0.008	1.78E-01	0.026	0.012	2.70E-02
8.117237	-0.013	0.009	1.70E-01	0.027	0.014	5.59E-02
8.2037	0.010	0.007	1.66E-01	-0.010	0.011	3.48E-01
8.794139	0.012	0.008	1.42E-01	-0.009	0.012	4.87E-01
7.191374	0.015	0.010	1.24E-01	-0.007	0.015	6.42E-01
4.281905	0.019	0.011	8.71E-02	0.016	0.017	3.34E-01
2.525390506	-0.018	0.011	9.69E-02	-0.005	0.016	7.59E-01
5.866503	0.012	0.007	8.44E-02	0.005	0.010	6.46E-01
1.623078297	0.012	0.007	8.05E-02	0.007	0.010	4.82E-01
4.161126	-0.023	0.015	1.20E-01	-0.021	0.022	3.45E-01
1.989789821	0.015	0.008	7.00E-02	-0.002	0.012	8.61E-01
7.605186	-0.010	0.009	2.90E-01	-0.007	0.014	5.98E-01
1.435012479	0.006	0.004	1.14E-01	0.011	0.006	6.61E-02
3.588181	-0.007	0.006	2.77E-01	0.007	0.009	4.61E-01
2.379042329	-0.011	0.008	1.62E-01	0.012	0.011	2.93E-01
1.843778077	-0.013	0.010	1.71E-01	0.011	0.014	4.59E-01
8.609101	0.010	0.009	2.52E-01	0.001	0.014	9.13E-01
0.807229264	0.006	0.005	2.92E-01	0.014	0.008	8.04E-02
3.045852	-0.009	0.006	1.43E-01	-0.010	0.009	2.76E-01
7.260006	-0.015	0.009	1.06E-01	-0.005	0.014	7.30E-01
7.472295	0.013	0.008	1.06E-01	0.000	0.012	9.81E-01
1.962875214	0.008	0.005	1.12E-01	0.001	0.007	8.73E-01
5.431159	0.010	0.007	1.77E-01	-0.009	0.011	3.88E-01
2.76089332	-0.016	0.009	9.09E-02	-0.038	0.014	6.06E-03
2.167089797	0.018	0.011	8.98E-02	-0.007	0.016	6.45E-01
1.139288231	0.012	0.007	7.64E-02	0.002	0.010	8.56E-01
3.224161	-0.014	0.009	1.13E-01	0.003	0.013	8.19E-01
3.975751	-0.011	0.009	2.23E-01	0.008	0.013	5.51E-01
6.803131	0.014	0.010	1.72E-01	-0.019	0.016	2.15E-01
5.687184	0.012	0.008	1.25E-01	0.009	0.012	4.42E-01
3.377574	0.015	0.013	2.22E-01	0.005	0.019	8.01E-01
5.42914	0.011	0.007	1.23E-01	-0.004	0.011	7.21E-01
2.267346709	0.011	0.007	1.34E-01	0.011	0.011	3.36E-01
7.361945	-0.017	0.009	6.43E-02	-0.009	0.013	5.11E-01

1.87540274	-0.010	0.007	1.34E-01	0.001	0.010	9.32E-01
2.259272327	0.009	0.006	1.25E-01	0.009	0.009	3.20E-01
3.313316	-0.022	0.012	7.45E-02	0.004	0.019	8.20E-01
4.147668	-0.020	0.013	1.27E-01	0.007	0.020	7.08E-01
3.066374	-0.010	0.007	1.73E-01	-0.005	0.011	6.36E-01
6.99086	0.010	0.007	1.60E-01	0.010	0.010	3.39E-01
1.422564473	0.009	0.005	4.85E-02	0.005	0.007	4.54E-01
7.779458	-0.014	0.009	1.19E-01	-0.013	0.013	3.47E-01
1.963211647	0.008	0.005	1.11E-01	0.002	0.007	7.43E-01
5.16605	0.016	0.010	1.04E-01	-0.010	0.015	4.96E-01
7.039307	-0.015	0.011	1.71E-01	0.008	0.016	6.00E-01
6.524901	0.009	0.007	2.34E-01	-0.006	0.011	6.11E-01
3.856654	0.007	0.006	2.12E-01	0.020	0.009	2.40E-02
2.884701	-0.012	0.008	1.09E-01	-0.009	0.011	4.33E-01
2.868552	-0.012	0.009	1.75E-01	0.008	0.013	5.60E-01
6.822308	-0.019	0.010	6.00E-02	0.006	0.015	7.04E-01
1.879776364	-0.009	0.006	1.78E-01	-0.008	0.009	4.12E-01
5.250831	-0.011	0.005	2.99E-02	-0.009	0.008	2.13E-01
0.665927575	-0.015	0.009	1.00E-01	-0.024	0.014	9.04E-02
6.160208	0.010	0.007	1.72E-01	0.010	0.011	3.47E-01
7.228045	-0.014	0.011	2.10E-01	0.007	0.016	6.56E-01
8.720797	-0.011	0.009	2.18E-01	-0.005	0.013	7.22E-01
8.521629	0.009	0.008	2.55E-01	0.002	0.012	8.63E-01
2.506213849	-0.023	0.013	8.95E-02	-0.019	0.020	3.38E-01
2.097448251	-0.008	0.008	2.89E-01	-0.006	0.011	6.25E-01
6.985141	-0.012	0.008	1.11E-01	-0.001	0.011	9.42E-01
2.043955469	0.010	0.006	8.71E-02	0.006	0.009	4.92E-01
2.261963788	0.010	0.006	1.01E-01	0.014	0.009	1.31E-01
7.545301	0.014	0.010	1.55E-01	0.010	0.014	4.96E-01
1.476393687	-0.006	0.004	1.59E-01	0.001	0.007	9.19E-01
8.029091	0.015	0.009	8.87E-02	-0.003	0.013	8.45E-01
6.442139	-0.010	0.010	3.18E-01	-0.020	0.015	1.81E-01
2.303681429	-0.006	0.004	1.63E-01	-0.008	0.007	2.35E-01
8.14617	0.014	0.009	1.22E-01	0.012	0.014	3.88E-01
7.355217	-0.013	0.010	1.98E-01	-0.023	0.015	1.30E-01
2.223946905	0.022	0.017	1.83E-01	-0.001	0.024	9.59E-01
1.989453389	0.015	0.008	7.43E-02	-0.003	0.012	8.33E-01
2.397209689	-0.018	0.014	1.91E-01	-0.001	0.021	9.53E-01
6.959236	-0.013	0.007	8.76E-02	-0.001	0.011	9.40E-01
1.42222804	0.010	0.005	4.40E-02	0.008	0.007	2.53E-01
3.621824	-0.008	0.006	1.24E-01	-0.006	0.008	4.80E-01
2.498139467	-0.024	0.014	7.76E-02	0.001	0.020	9.59E-01
2.257590164	0.012	0.007	9.26E-02	0.010	0.011	3.40E-01

8.748721	0.014	0.008	8.41E-02	-0.005	0.012	6.56E-01
2.494102276	-0.021	0.015	1.57E-01	-0.002	0.022	9.27E-01
7.119714	-0.015	0.011	1.49E-01	-0.019	0.016	2.33E-01
0.844909714	-0.014	0.008	8.27E-02	-0.016	0.012	1.89E-01
0.740279178	-0.010	0.006	1.23E-01	0.001	0.010	8.90E-01
3.701222	0.009	0.006	1.61E-01	0.021	0.009	2.30E-02
5.930761	0.016	0.010	1.06E-01	-0.002	0.015	9.01E-01
5.97046	0.011	0.009	2.44E-01	0.025	0.014	7.07E-02
2.336651823	0.016	0.008	4.50E-02	0.007	0.012	5.45E-01
2.369958649	-0.009	0.007	1.88E-01	0.015	0.010	1.35E-01
3.22517	-0.012	0.008	1.24E-01	0.005	0.011	6.61E-01
5.431832	0.011	0.008	1.32E-01	-0.007	0.011	5.48E-01
1.485477367	-0.006	0.004	1.46E-01	-0.005	0.007	4.72E-01
6.896996	0.012	0.008	1.47E-01	-0.013	0.012	2.81E-01
5.751106	0.013	0.011	2.20E-01	0.002	0.016	9.05E-01
5.795179	-0.011	0.009	2.21E-01	-0.007	0.013	5.79E-01
8.735263	-0.014	0.008	1.09E-01	-0.022	0.013	8.42E-02
3.889961	0.007	0.006	2.09E-01	0.026	0.008	2.08E-03
6.331116	0.015	0.010	1.30E-01	-0.005	0.015	7.13E-01
8.154244	0.010	0.008	2.02E-01	0.001	0.012	9.33E-01
3.066038	-0.010	0.007	1.69E-01	-0.005	0.011	6.28E-01
6.438774	0.014	0.010	1.61E-01	0.013	0.015	4.01E-01
6.57402	0.008	0.007	2.32E-01	0.009	0.010	3.95E-01
8.858734	0.009	0.007	2.04E-01	-0.005	0.011	6.33E-01
1.075366039	0.012	0.007	9.65E-02	-0.004	0.011	7.26E-01
0.977800587	0.008	0.006	1.86E-01	0.008	0.010	3.89E-01
2.488046489	-0.025	0.013	6.08E-02	0.001	0.020	9.48E-01
1.150726939	0.010	0.006	8.20E-02	-0.002	0.009	8.27E-01
7.107602	-0.010	0.009	2.89E-01	0.020	0.014	1.37E-01
0.764838757	0.008	0.005	1.24E-01	0.000	0.008	9.88E-01
5.713762	0.012	0.008	1.23E-01	0.004	0.012	7.46E-01
1.421891608	0.009	0.005	5.07E-02	0.008	0.007	2.89E-01
6.137667	0.012	0.010	2.56E-01	-0.032	0.015	3.54E-02
6.261138	0.010	0.008	1.80E-01	-0.003	0.011	8.11E-01
5.139472	0.010	0.007	1.33E-01	-0.010	0.010	3.40E-01
5.728902	0.010	0.008	2.43E-01	-0.024	0.013	5.30E-02
8.436511	-0.012	0.009	1.71E-01	-0.033	0.013	9.79E-03
2.590994862	-0.021	0.018	2.48E-01	-0.022	0.027	4.04E-01
8.852342	-0.013	0.009	1.50E-01	0.000	0.013	9.99E-01
6.379562	0.010	0.008	1.92E-01	0.017	0.011	1.31E-01
6.261474	0.008	0.007	2.50E-01	-0.002	0.010	8.37E-01
0.814294348	0.006	0.005	2.66E-01	0.017	0.008	2.70E-02
7.093472	-0.015	0.011	1.59E-01	0.011	0.016	5.05E-01

0.714710301	0.011	0.008	1.45E-01	0.017	0.012	1.36E-01
7.795271	-0.013	0.008	1.27E-01	0.005	0.012	6.77E-01
5.527042	0.012	0.007	1.09E-01	-0.004	0.011	7.04E-01
1.057871544	0.008	0.006	1.34E-01	0.009	0.008	2.72E-01
3.109101	-0.017	0.010	7.52E-02	-0.014	0.015	3.34E-01
8.734927	-0.015	0.008	5.36E-02	-0.011	0.011	3.17E-01
0.561969905	0.012	0.008	1.23E-01	-0.001	0.012	9.64E-01
1.129868118	0.014	0.007	6.82E-02	0.002	0.011	8.92E-01
7.478015	0.012	0.008	1.21E-01	-0.010	0.012	4.13E-01
6.953853	-0.011	0.008	1.94E-01	-0.005	0.012	6.81E-01
1.962538782	0.008	0.005	1.14E-01	0.000	0.007	9.71E-01
2.188957915	0.018	0.011	1.11E-01	-0.014	0.017	3.99E-01
6.450213	0.013	0.009	1.41E-01	-0.005	0.013	6.67E-01
5.166386	0.016	0.010	9.64E-02	-0.013	0.015	3.96E-01
5.855064	0.015	0.009	1.22E-01	-0.021	0.014	1.30E-01
1.477066552	-0.007	0.005	1.56E-01	0.002	0.007	7.95E-01
3.11011	-0.018	0.010	7.19E-02	-0.017	0.015	2.42E-01
1.006060925	0.012	0.008	1.24E-01	0.013	0.012	2.70E-01
3.375892	0.017	0.013	1.84E-01	-0.004	0.020	8.41E-01
7.954403	0.013	0.010	1.99E-01	-0.011	0.015	4.81E-01
7.216943	-0.016	0.009	8.25E-02	-0.012	0.013	3.81E-01
1.406752141	0.009	0.005	8.65E-02	0.010	0.008	2.33E-01
7.797962	-0.016	0.010	1.01E-01	0.001	0.014	9.52E-01
7.734713	-0.014	0.011	2.01E-01	-0.008	0.016	6.14E-01
8.273678	0.012	0.008	1.28E-01	0.011	0.012	3.53E-01
7.461866	0.014	0.010	1.63E-01	-0.001	0.015	9.23E-01
7.664062	0.015	0.010	1.22E-01	-0.009	0.014	5.52E-01
3.376901	0.016	0.012	1.87E-01	0.007	0.018	7.07E-01
1.913419623	-0.012	0.007	7.31E-02	-0.019	0.010	5.68E-02
2.190640078	0.019	0.011	9.76E-02	-0.012	0.017	4.89E-01
4.316558	-0.021	0.015	1.50E-01	0.022	0.022	3.22E-01
1.146016883	0.011	0.006	8.10E-02	-0.002	0.009	7.89E-01
6.614056	0.009	0.010	3.47E-01	0.003	0.014	8.46E-01
3.15822	-0.012	0.008	1.16E-01	-0.013	0.011	2.47E-01
0.688468559	0.025	0.015	1.03E-01	-0.001	0.023	9.78E-01
1.874393443	-0.012	0.007	1.12E-01	0.011	0.011	3.27E-01
2.019732322	0.016	0.009	8.36E-02	0.000	0.014	9.98E-01
5.410973	0.012	0.008	1.46E-01	-0.014	0.013	2.48E-01
6.125219	0.013	0.010	1.90E-01	-0.019	0.015	1.94E-01
5.590964	0.012	0.008	1.36E-01	-0.003	0.012	7.95E-01
3.73823	0.009	0.008	2.88E-01	0.042	0.013	8.62E-04
7.677856	0.009	0.008	2.60E-01	-0.001	0.012	9.21E-01
5.5442	0.012	0.009	1.80E-01	0.005	0.013	6.96E-01

8.436848	-0.011	0.009	2.13E-01	-0.028	0.014	3.95E-02
0.849283337	-0.012	0.007	9.02E-02	-0.015	0.010	1.62E-01
0.715046734	0.011	0.008	1.54E-01	0.015	0.012	2.08E-01
5.929752	0.012	0.007	1.08E-01	-0.011	0.011	3.02E-01
3.109774	-0.017	0.010	8.37E-02	-0.015	0.015	2.90E-01
8.852005	-0.013	0.009	1.38E-01	-0.020	0.014	1.50E-01
6.188132	-0.014	0.010	1.67E-01	-0.025	0.015	9.69E-02
3.154856	-0.014	0.009	1.15E-01	-0.005	0.013	6.87E-01
6.916845	-0.014	0.009	1.12E-01	-0.014	0.013	2.98E-01
2.262636653	0.010	0.006	1.17E-01	0.012	0.010	2.21E-01
8.050959	0.012	0.009	1.97E-01	0.003	0.014	8.03E-01
2.680149498	-0.010	0.006	6.18E-02	0.003	0.008	7.07E-01
7.791906	-0.017	0.011	1.15E-01	-0.011	0.016	5.02E-01
4.138585	-0.024	0.016	1.24E-01	0.011	0.023	6.33E-01
2.383415953	-0.013	0.008	9.10E-02	0.006	0.011	5.99E-01
3.213059	-0.027	0.016	9.30E-02	-0.015	0.024	5.40E-01
1.260067531	0.022	0.013	9.56E-02	-0.006	0.020	7.52E-01
6.901033	0.014	0.009	1.03E-01	-0.007	0.013	5.94E-01
0.939110839	-0.006	0.005	2.19E-01	-0.022	0.008	6.04E-03
8.587233	-0.009	0.007	1.56E-01	0.002	0.010	8.16E-01
8.385373	0.010	0.007	1.24E-01	-0.006	0.010	5.17E-01
5.747742	0.014	0.011	2.03E-01	0.014	0.016	3.95E-01
6.369469	0.012	0.009	1.94E-01	-0.014	0.014	3.07E-01
2.146230976	-0.023	0.013	8.70E-02	-0.010	0.020	6.16E-01
3.618796	-0.008	0.006	2.10E-01	-0.001	0.009	9.28E-01
3.660851	-0.008	0.005	8.94E-02	0.005	0.007	4.60E-01
8.664612	0.013	0.009	1.76E-01	-0.005	0.014	7.11E-01
6.724406	-0.019	0.011	8.24E-02	-0.039	0.016	1.48E-02
3.072093	-0.010	0.006	1.24E-01	-0.009	0.009	3.58E-01
7.610906	-0.022	0.015	1.25E-01	-0.007	0.022	7.37E-01
8.782027	0.013	0.008	8.88E-02	0.010	0.012	3.63E-01
3.62788	-0.009	0.006	1.47E-01	0.012	0.009	2.01E-01
7.530835	-0.009	0.009	3.04E-01	-0.006	0.013	6.61E-01
6.057596	0.009	0.008	2.78E-01	0.006	0.012	6.33E-01
1.905681674	-0.010	0.006	1.10E-01	0.001	0.009	8.99E-01
4.128828	-0.019	0.013	1.50E-01	0.016	0.020	4.30E-01
8.291845	0.012	0.008	1.08E-01	-0.011	0.012	3.52E-01
1.084113286	0.008	0.006	1.86E-01	-0.001	0.009	9.38E-01
7.7152	-0.016	0.010	1.11E-01	-0.017	0.015	2.65E-01
5.020375	0.011	0.008	1.80E-01	-0.004	0.012	7.61E-01
3.556893	-0.026	0.017	1.19E-01	-0.026	0.025	2.96E-01
5.255205	-0.011	0.005	3.61E-02	-0.009	0.008	2.41E-01
1.315578909	0.020	0.010	4.26E-02	0.007	0.014	6.16E-01

1.47673012	-0.006	0.005	1.61E-01	0.002	0.007	7.42E-01
2.047319794	0.012	0.007	9.52E-02	0.010	0.010	3.50E-01
5.164704	0.015	0.009	1.12E-01	-0.010	0.014	4.59E-01
1.738138243	-0.006	0.006	2.96E-01	0.006	0.008	4.89E-01
1.927549792	0.006	0.004	1.86E-01	0.004	0.006	5.48E-01
5.048299	0.016	0.011	1.54E-01	-0.017	0.016	3.02E-01
1.860936139	-0.014	0.008	8.63E-02	0.006	0.012	6.45E-01
6.52591	0.013	0.009	1.34E-01	-0.011	0.013	4.17E-01
2.87057	-0.013	0.010	1.80E-01	-0.005	0.015	7.20E-01
1.489178126	-0.008	0.004	7.83E-02	-0.007	0.006	2.67E-01
1.077048202	0.011	0.007	1.57E-01	0.002	0.011	8.32E-01
2.337997553	0.014	0.007	5.72E-02	0.007	0.011	5.20E-01
1.329709078	0.019	0.010	4.86E-02	0.010	0.014	4.62E-01
7.659352	0.012	0.008	1.37E-01	0.000	0.012	9.72E-01
3.608367	-0.010	0.007	1.64E-01	-0.009	0.010	4.01E-01
1.538633717	0.020	0.012	1.07E-01	-0.016	0.019	3.84E-01
8.643417	-0.012	0.007	1.20E-01	-0.018	0.011	9.67E-02
6.029336	0.016	0.009	6.79E-02	0.005	0.013	6.86E-01
4.128155	-0.016	0.011	1.61E-01	0.011	0.017	5.06E-01
3.222479	-0.018	0.011	1.06E-01	-0.002	0.016	9.00E-01
2.639777587	-0.015	0.009	1.07E-01	-0.002	0.014	8.62E-01
8.165683	0.012	0.009	1.49E-01	-0.007	0.013	5.95E-01
2.409994128	-0.019	0.012	1.10E-01	0.005	0.018	7.64E-01
3.272944	-0.015	0.010	1.59E-01	0.009	0.015	5.70E-01
7.65767	0.016	0.010	1.22E-01	-0.006	0.015	7.18E-01
1.927213359	0.006	0.004	1.69E-01	0.006	0.006	3.73E-01
4.244561	0.027	0.017	1.11E-01	0.005	0.025	8.37E-01
6.931648	0.008	0.008	3.18E-01	0.008	0.012	5.02E-01
4.129165	-0.019	0.014	1.57E-01	0.011	0.020	5.85E-01
4.139931	-0.025	0.018	1.61E-01	0.016	0.027	5.58E-01
5.864148	0.013	0.009	1.34E-01	0.000	0.013	9.87E-01
6.605981	0.013	0.008	8.19E-02	0.017	0.011	1.26E-01
2.146903841	-0.021	0.011	6.57E-02	0.002	0.017	9.27E-01
2.046983362	0.012	0.007	9.72E-02	0.010	0.010	3.57E-01
6.039429	0.012	0.008	1.51E-01	-0.005	0.013	6.66E-01
7.183973	0.013	0.008	1.21E-01	-0.005	0.012	6.71E-01
7.902929	-0.014	0.009	9.72E-02	-0.036	0.013	5.36E-03
7.231073	-0.017	0.010	1.05E-01	-0.004	0.015	8.01E-01
1.858244678	-0.010	0.008	1.93E-01	0.001	0.012	9.51E-01
5.928406	0.013	0.007	8.71E-02	0.007	0.011	5.21E-01
1.913756056	-0.011	0.006	7.86E-02	-0.012	0.009	1.77E-01
3.603657	-0.011	0.007	1.49E-01	-0.005	0.011	6.31E-01
2.599069244	-0.025	0.017	1.40E-01	0.014	0.026	5.86E-01

8.216148	0.011	0.008	1.38E-01	0.006	0.011	6.29E-01
8.180822	-0.017	0.010	8.13E-02	0.000	0.015	9.95E-01
7.588028	-0.016	0.010	1.01E-01	-0.024	0.015	1.03E-01
3.972051	-0.016	0.009	8.74E-02	-0.008	0.014	5.68E-01
4.160116	-0.020	0.013	1.28E-01	0.002	0.020	9.20E-01
5.250158	-0.011	0.005	3.32E-02	-0.010	0.008	1.89E-01
3.496335	0.011	0.010	2.68E-01	0.029	0.014	4.20E-02
2.045301199	0.011	0.007	9.74E-02	0.007	0.010	5.07E-01
7.563469	0.012	0.009	1.77E-01	0.013	0.014	3.53E-01
4.406722	-0.021	0.015	1.75E-01	0.005	0.023	8.18E-01
3.629226	-0.008	0.006	1.73E-01	0.011	0.009	2.13E-01
5.585245	0.015	0.009	8.67E-02	-0.020	0.013	1.31E-01
6.928284	-0.018	0.011	1.10E-01	-0.003	0.017	8.61E-01
1.128185955	0.012	0.007	8.91E-02	-0.004	0.010	7.04E-01
5.800562	-0.015	0.010	1.29E-01	-0.014	0.014	3.15E-01
6.099987	0.010	0.007	1.44E-01	0.017	0.011	1.19E-01
0.72884047	-0.012	0.007	8.65E-02	-0.015	0.011	1.47E-01
7.725293	-0.014	0.010	1.61E-01	-0.019	0.014	1.85E-01
2.377023734	-0.016	0.011	1.29E-01	0.010	0.016	5.27E-01
3.534352	0.013	0.007	8.32E-02	0.027	0.011	1.52E-02
5.630663	0.011	0.008	1.54E-01	-0.015	0.011	1.81E-01
5.095399	0.014	0.009	1.23E-01	-0.008	0.014	5.63E-01
7.098855	-0.013	0.010	1.89E-01	-0.007	0.014	6.18E-01
2.620937362	-0.018	0.012	1.50E-01	0.013	0.018	4.74E-01
2.045637632	0.011	0.007	9.83E-02	0.007	0.010	4.77E-01
2.759211157	-0.013	0.008	9.33E-02	-0.029	0.012	1.56E-02
0.511168583	0.009	0.009	3.12E-01	-0.012	0.014	3.78E-01
4.427244	-0.020	0.015	1.76E-01	0.010	0.022	6.42E-01
8.833502	0.011	0.009	2.24E-01	-0.023	0.013	8.25E-02
5.251167	-0.011	0.005	3.30E-02	-0.009	0.008	2.48E-01
6.870081	0.013	0.008	9.27E-02	0.000	0.011	9.99E-01
5.038878	0.013	0.009	1.64E-01	-0.002	0.013	9.06E-01
6.064998	-0.014	0.008	9.22E-02	-0.030	0.012	1.41E-02
5.782058	0.008	0.007	2.58E-01	-0.002	0.011	8.59E-01
8.604391	0.010	0.009	2.67E-01	0.014	0.013	2.87E-01
2.159015415	-0.014	0.009	1.22E-01	-0.012	0.013	3.52E-01
5.713089	0.012	0.009	2.10E-01	0.010	0.014	4.63E-01
1.928222657	0.007	0.004	1.20E-01	0.004	0.006	5.74E-01
7.821849	-0.014	0.009	1.06E-01	-0.018	0.013	1.72E-01
7.086071	0.010	0.006	1.08E-01	0.016	0.009	7.58E-02
8.667304	0.011	0.007	1.21E-01	0.023	0.010	2.78E-02
5.412655	0.013	0.008	1.03E-01	-0.005	0.012	6.61E-01
7.09179	-0.015	0.011	1.57E-01	0.001	0.016	9.66E-01

2.178528505	0.018	0.011	1.08E-01	-0.012	0.017	4.87E-01
2.044628334	0.011	0.006	9.70E-02	0.007	0.010	4.61E-01
2.258935894	0.009	0.006	1.28E-01	0.010	0.009	2.33E-01
2.606134328	-0.019	0.013	1.55E-01	0.012	0.020	5.53E-01
8.037502	0.011	0.007	1.41E-01	0.008	0.011	4.89E-01
0.782669684	0.006	0.004	1.60E-01	0.005	0.007	4.80E-01
2.581574749	-0.010	0.010	3.36E-01	0.010	0.016	5.30E-01
7.515022	0.012	0.009	2.03E-01	-0.010	0.014	4.76E-01
4.314875	-0.022	0.015	1.35E-01	0.011	0.022	6.27E-01
3.222142	-0.018	0.011	1.06E-01	-0.003	0.017	8.57E-01
8.39042	0.011	0.008	1.69E-01	0.007	0.012	5.74E-01
4.159107	-0.018	0.012	1.57E-01	-0.001	0.019	9.53E-01
3.111456	-0.018	0.010	8.57E-02	-0.007	0.016	6.62E-01
7.940609	-0.017	0.010	8.37E-02	-0.012	0.015	4.22E-01
2.608489356	-0.019	0.013	1.57E-01	0.032	0.020	1.05E-01
2.587630536	0.013	0.008	8.63E-02	0.013	0.012	2.83E-01
1.219022755	0.010	0.007	1.65E-01	0.005	0.011	6.71E-01
1.790285295	-0.013	0.012	2.52E-01	-0.014	0.018	4.40E-01
1.860599706	-0.014	0.008	9.64E-02	0.007	0.012	5.88E-01
7.361273	-0.014	0.009	1.16E-01	-0.003	0.014	8.36E-01
6.056923	0.011	0.007	1.49E-01	-0.004	0.011	7.21E-01
3.862374	-0.008	0.005	1.18E-01	0.005	0.008	5.07E-01
5.644793	0.010	0.007	1.19E-01	0.010	0.010	3.20E-01
0.84961977	-0.012	0.007	9.49E-02	-0.014	0.010	1.66E-01
6.22514	0.011	0.007	1.21E-01	-0.001	0.011	9.58E-01
0.844573281	-0.014	0.008	8.80E-02	-0.015	0.012	1.97E-01
1.451161243	0.007	0.004	8.29E-02	0.002	0.006	7.65E-01
5.48196	0.010	0.007	1.61E-01	0.007	0.011	5.26E-01
1.721316614	-0.009	0.005	1.04E-01	0.000	0.008	9.54E-01
3.49869	0.010	0.009	2.62E-01	0.029	0.013	2.04E-02
4.320595	-0.019	0.013	1.64E-01	0.023	0.020	2.46E-01
7.038297	-0.021	0.011	6.67E-02	0.017	0.017	3.13E-01
6.223458	0.012	0.008	1.28E-01	0.001	0.012	9.34E-01
6.710276	-0.012	0.009	1.83E-01	-0.019	0.013	1.48E-01
1.647637876	0.009	0.005	8.64E-02	0.010	0.008	2.10E-01
1.825610717	-0.014	0.009	1.08E-01	-0.008	0.013	5.41E-01
5.038206	0.012	0.009	1.86E-01	-0.017	0.013	2.11E-01
8.892714	0.011	0.008	1.97E-01	-0.005	0.012	7.15E-01
5.014655	0.010	0.007	1.21E-01	0.000	0.010	9.62E-01
6.907761	0.012	0.007	1.03E-01	0.010	0.011	3.59E-01
6.246335	-0.011	0.007	1.48E-01	-0.004	0.011	7.52E-01
5.831514	0.012	0.010	2.56E-01	0.002	0.015	9.19E-01
7.740096	-0.013	0.011	2.50E-01	-0.018	0.016	2.75E-01

1.075029606	0.011	0.007	1.23E-01	0.002	0.010	8.38E-01
2.405956937	-0.020	0.013	1.29E-01	-0.016	0.020	4.20E-01
0.708318082	0.010	0.008	1.73E-01	0.000	0.011	9.68E-01
7.352189	-0.012	0.010	2.19E-01	-0.017	0.015	2.40E-01
8.223549	0.008	0.007	2.70E-01	-0.018	0.011	9.03E-02
7.109957	-0.011	0.009	1.95E-01	-0.010	0.013	4.40E-01
0.706635919	0.012	0.010	2.02E-01	0.025	0.014	8.06E-02
1.808789087	-0.016	0.011	1.33E-01	-0.005	0.016	7.57E-01
6.477801	0.009	0.008	2.87E-01	-0.004	0.012	7.67E-01
1.708868608	-0.007	0.005	1.20E-01	-0.001	0.007	8.82E-01
2.047656227	0.012	0.007	9.48E-02	0.010	0.010	3.44E-01
7.74346	-0.016	0.010	9.43E-02	-0.023	0.014	9.95E-02
5.031813	0.013	0.010	1.61E-01	-0.007	0.014	6.14E-01
8.024718	-0.013	0.008	1.27E-01	-0.009	0.013	4.63E-01
3.162594	-0.011	0.007	1.05E-01	0.000	0.010	9.67E-01
5.115249	0.014	0.009	1.28E-01	0.010	0.014	4.83E-01
2.14152092	-0.022	0.014	1.02E-01	0.035	0.020	8.91E-02
8.940824	-0.009	0.007	2.33E-01	-0.015	0.011	1.66E-01
5.11794	0.012	0.009	1.54E-01	-0.005	0.013	6.82E-01
1.788266699	-0.012	0.009	1.76E-01	0.015	0.013	2.54E-01
3.972387	-0.016	0.010	9.50E-02	-0.007	0.014	6.14E-01
5.097081	0.013	0.009	1.61E-01	0.004	0.014	7.78E-01
6.477464	0.011	0.010	2.73E-01	-0.014	0.016	3.80E-01
1.489850991	-0.007	0.004	8.45E-02	-0.009	0.006	1.60E-01
1.127849523	0.012	0.007	8.44E-02	-0.004	0.011	7.21E-01
0.74061561	-0.009	0.006	1.51E-01	-0.006	0.010	4.96E-01
8.006887	0.008	0.007	2.82E-01	-0.008	0.011	4.85E-01
7.257651	-0.017	0.011	1.31E-01	-0.019	0.016	2.33E-01
2.554323709	-0.017	0.011	1.33E-01	0.002	0.017	8.90E-01
0.777623195	0.006	0.005	1.60E-01	0.000	0.007	9.83E-01
8.469145	-0.010	0.009	2.48E-01	-0.023	0.013	8.16E-02
6.738536	-0.011	0.006	8.31E-02	-0.008	0.010	4.24E-01
1.924858331	0.005	0.004	1.73E-01	0.005	0.006	3.55E-01
8.843931	0.012	0.008	1.35E-01	0.014	0.012	2.26E-01
5.801908	0.011	0.008	1.75E-01	-0.001	0.012	9.24E-01
1.489514558	-0.007	0.004	7.95E-02	-0.007	0.006	2.49E-01
3.500709	0.010	0.008	2.21E-01	0.036	0.012	2.87E-03
7.50863	-0.011	0.009	2.56E-01	-0.029	0.014	3.60E-02
0.938101541	-0.006	0.005	2.19E-01	-0.020	0.008	9.09E-03
6.322369	0.011	0.009	1.91E-01	0.010	0.013	4.32E-01
7.428896	0.009	0.009	3.22E-01	0.003	0.013	8.26E-01
4.160789	-0.025	0.016	1.34E-01	-0.013	0.024	5.91E-01
8.10748	0.009	0.007	2.11E-01	-0.004	0.010	6.82E-01

1.90265378	-0.009	0.006	1.34E-01	-0.007	0.009	4.40E-01
8.61112	-0.012	0.008	1.20E-01	-0.001	0.012	9.46E-01
8.704311	0.007	0.007	2.57E-01	0.019	0.010	4.85E-02
5.804935	0.010	0.009	2.40E-01	0.005	0.013	7.23E-01
1.168221434	0.009	0.004	4.93E-02	0.000	0.007	9.80E-01
1.735783215	-0.009	0.005	9.51E-02	0.004	0.008	6.25E-01
0.654488867	-0.018	0.011	9.36E-02	-0.032	0.016	4.62E-02
8.425409	0.010	0.008	2.05E-01	0.000	0.012	9.80E-01
3.881887	0.008	0.006	1.90E-01	0.024	0.010	1.39E-02
1.045423538	0.007	0.005	1.84E-01	0.012	0.008	1.45E-01
2.130082212	-0.016	0.012	1.70E-01	-0.014	0.018	4.41E-01
1.914428921	-0.010	0.006	7.62E-02	-0.003	0.008	6.78E-01
1.695747737	-0.006	0.004	1.66E-01	0.001	0.006	8.35E-01
3.97306	-0.015	0.010	1.18E-01	-0.003	0.015	8.44E-01
5.160331	0.017	0.010	1.15E-01	-0.029	0.016	6.69E-02
5.79888	0.008	0.007	2.77E-01	0.005	0.011	6.61E-01
3.972724	-0.016	0.010	1.05E-01	-0.005	0.015	7.43E-01
7.788206	-0.011	0.008	1.70E-01	0.005	0.012	6.72E-01
6.961927	-0.015	0.009	1.09E-01	-0.016	0.014	2.31E-01
6.125892	0.015	0.010	1.23E-01	0.005	0.015	7.23E-01
3.382957	0.011	0.009	2.53E-01	0.015	0.014	2.89E-01
1.426601664	0.008	0.004	7.05E-02	0.009	0.007	2.00E-01
0.86004918	0.015	0.010	1.10E-01	0.001	0.014	9.29E-01
6.08081	0.015	0.009	9.03E-02	0.020	0.014	1.45E-01
8.310349	0.010	0.010	2.95E-01	0.021	0.015	1.49E-01
4.380143	-0.016	0.012	1.61E-01	0.000	0.017	9.88E-01
5.097754	0.012	0.008	1.58E-01	-0.001	0.012	9.39E-01
5.32821	0.029	0.019	1.26E-01	-0.018	0.029	5.24E-01
8.916937	-0.012	0.009	1.63E-01	-0.017	0.013	1.79E-01
4.147332	-0.021	0.015	1.59E-01	0.005	0.022	8.29E-01
6.553834	0.014	0.008	6.47E-02	-0.003	0.011	8.07E-01
1.259731099	0.021	0.013	1.02E-01	-0.007	0.019	7.33E-01
6.735508	-0.015	0.009	8.18E-02	0.004	0.013	7.76E-01
5.250494	-0.011	0.005	3.36E-02	-0.010	0.008	2.03E-01
7.506611	-0.009	0.008	2.68E-01	0.009	0.012	4.23E-01
2.773004894	-0.017	0.010	1.11E-01	-0.047	0.016	2.47E-03
1.835367262	-0.012	0.010	2.04E-01	-0.003	0.014	8.26E-01
2.045974064	0.011	0.007	1.03E-01	0.008	0.010	4.36E-01
8.623904	-0.014	0.009	1.31E-01	-0.006	0.014	6.34E-01
1.067628089	0.008	0.007	2.01E-01	-0.008	0.010	4.28E-01
5.028449	0.011	0.008	1.79E-01	-0.016	0.012	1.85E-01
7.743796	-0.017	0.010	1.00E-01	-0.020	0.015	1.96E-01
6.968656	-0.015	0.009	1.07E-01	-0.005	0.014	7.22E-01

2.409657695	-0.019	0.012	1.15E-01	0.010	0.018	5.98E-01
6.839129	-0.015	0.009	1.08E-01	0.025	0.014	7.64E-02
0.844236849	-0.014	0.008	8.98E-02	-0.015	0.012	2.00E-01
7.9396	-0.009	0.008	2.49E-01	-0.007	0.012	5.59E-01
3.165958	-0.010	0.007	1.51E-01	0.007	0.010	4.82E-01
5.67911	0.009	0.007	1.90E-01	-0.002	0.010	8.64E-01
8.450305	-0.012	0.010	2.23E-01	-0.007	0.015	6.33E-01
1.488841693	-0.007	0.004	8.50E-02	-0.010	0.006	1.29E-01
8.845277	0.013	0.009	1.31E-01	0.012	0.013	3.38E-01
5.591637	0.012	0.010	1.95E-01	-0.018	0.014	1.99E-01
2.176846342	0.018	0.011	1.18E-01	-0.011	0.017	5.22E-01
3.627544	-0.009	0.006	1.57E-01	0.009	0.009	3.17E-01
3.221806	-0.019	0.012	1.11E-01	-0.004	0.018	8.24E-01
2.772668461	-0.017	0.010	1.11E-01	-0.046	0.015	2.67E-03
5.539154	0.012	0.007	8.81E-02	-0.006	0.011	5.58E-01
1.707522877	-0.007	0.005	1.58E-01	-0.003	0.007	6.52E-01
5.997375	0.016	0.010	9.35E-02	0.016	0.015	2.67E-01
5.526033	0.013	0.008	1.02E-01	0.007	0.012	5.77E-01
6.341882	-0.015	0.009	8.74E-02	-0.020	0.013	1.28E-01
3.321054	0.016	0.011	1.70E-01	0.023	0.017	1.71E-01
8.406232	-0.013	0.008	1.13E-01	-0.021	0.012	8.03E-02
7.509976	-0.011	0.008	1.63E-01	0.015	0.012	2.20E-01
7.979299	0.011	0.009	1.95E-01	-0.004	0.013	7.82E-01
2.713792758	-0.012	0.006	4.75E-02	-0.016	0.009	6.43E-02
8.673023	0.010	0.008	2.27E-01	0.021	0.012	9.55E-02
0.528999511	0.010	0.007	1.53E-01	-0.001	0.011	9.41E-01
7.88678	-0.010	0.009	2.94E-01	0.018	0.014	1.86E-01
3.758416	0.011	0.009	2.02E-01	0.025	0.013	5.77E-02
4.086438	0.020	0.011	6.53E-02	0.019	0.016	2.53E-01
0.936419379	-0.007	0.005	2.01E-01	-0.019	0.008	1.36E-02
2.604788598	-0.021	0.013	1.22E-01	0.013	0.020	5.08E-01
2.427825055	-0.013	0.008	1.27E-01	0.009	0.013	4.56E-01
0.806892831	0.005	0.005	3.17E-01	0.013	0.008	9.13E-02
5.150238	0.011	0.008	1.74E-01	-0.010	0.013	4.06E-01
8.627268	-0.013	0.009	1.62E-01	-0.010	0.013	4.50E-01
2.716147786	-0.012	0.006	5.71E-02	-0.018	0.009	5.42E-02
6.439111	0.015	0.010	1.52E-01	0.030	0.016	5.75E-02
6.528602	0.012	0.008	1.43E-01	0.007	0.012	5.50E-01
3.945809	-0.011	0.008	1.58E-01	-0.004	0.012	7.33E-01
1.640572792	0.010	0.006	1.12E-01	0.003	0.009	7.70E-01
3.375556	0.018	0.014	1.87E-01	-0.014	0.020	4.89E-01
3.112465	-0.020	0.011	7.20E-02	-0.003	0.017	8.47E-01
6.732144	-0.014	0.009	9.11E-02	-0.016	0.013	2.18E-01

5.262943	-0.011	0.006	4.75E-02	-0.014	0.008	8.63E-02
2.020068755	0.016	0.010	9.33E-02	0.000	0.014	9.80E-01
8.380663	0.011	0.009	1.83E-01	0.010	0.013	4.19E-01
5.453027	0.009	0.007	2.53E-01	-0.018	0.011	1.02E-01
6.471408	0.011	0.008	1.44E-01	0.012	0.011	2.74E-01
6.180731	0.010	0.008	2.16E-01	-0.024	0.012	4.27E-02
5.431495	0.010	0.007	1.82E-01	-0.010	0.011	3.74E-01
3.628217	-0.009	0.006	1.69E-01	0.013	0.009	1.70E-01
3.049889	-0.014	0.008	7.26E-02	0.002	0.012	8.73E-01
1.062245168	0.009	0.006	1.20E-01	-0.003	0.008	6.93E-01
8.418344	-0.012	0.010	2.21E-01	0.005	0.015	7.60E-01
0.539092488	0.009	0.007	1.88E-01	0.020	0.011	5.73E-02
8.466454	-0.013	0.010	1.74E-01	-0.021	0.014	1.49E-01
1.902990213	-0.009	0.006	1.33E-01	-0.006	0.009	5.28E-01
2.57518253	0.015	0.009	9.43E-02	0.013	0.013	3.50E-01
7.646904	-0.013	0.011	2.39E-01	-0.017	0.017	3.24E-01
5.249149	-0.010	0.005	3.63E-02	-0.010	0.007	2.00E-01
8.102434	0.012	0.010	2.31E-01	0.020	0.015	1.73E-01
7.8057	-0.014	0.008	1.02E-01	-0.029	0.012	1.79E-02
4.446421	-0.020	0.016	2.08E-01	0.018	0.024	4.41E-01
1.076711769	0.012	0.007	1.17E-01	0.010	0.011	3.77E-01
3.976088	-0.010	0.009	2.31E-01	0.004	0.013	7.65E-01
8.414979	-0.010	0.008	1.81E-01	-0.002	0.012	8.56E-01
2.064141424	0.006	0.004	7.20E-02	0.009	0.005	1.03E-01
0.550531196	0.014	0.010	1.52E-01	0.043	0.014	2.53E-03
6.461988	0.011	0.008	1.61E-01	-0.008	0.012	5.07E-01
0.900084659	0.014	0.008	7.73E-02	-0.011	0.012	3.62E-01
6.557535	0.010	0.008	2.21E-01	0.009	0.012	4.24E-01
8.240035	-0.010	0.008	1.89E-01	-0.022	0.011	4.89E-02
3.705932	0.010	0.006	9.22E-02	0.023	0.009	9.07E-03
3.221133	-0.020	0.013	1.16E-01	-0.006	0.019	7.71E-01
7.463548	0.011	0.009	2.24E-01	-0.001	0.014	9.39E-01
3.652103	-0.007	0.005	1.48E-01	0.005	0.007	5.01E-01
8.892377	0.011	0.007	1.56E-01	0.000	0.011	9.66E-01
0.936082946	-0.007	0.005	2.01E-01	-0.019	0.008	1.24E-02
5.018356	0.015	0.011	1.52E-01	-0.010	0.016	5.21E-01
2.189967213	0.018	0.011	1.09E-01	-0.015	0.017	3.77E-01
4.130174	-0.020	0.015	1.62E-01	0.007	0.022	7.44E-01
8.145497	0.012	0.008	1.25E-01	0.007	0.012	5.32E-01
8.789765	0.016	0.008	5.81E-02	-0.009	0.013	4.59E-01
7.104911	-0.012	0.008	1.40E-01	0.000	0.012	1.00E+00
6.003767	0.012	0.008	1.53E-01	0.010	0.012	4.11E-01
7.006336	-0.009	0.006	1.58E-01	-0.008	0.009	3.71E-01

0.629929288	0.011	0.010	2.87E-01	0.005	0.015	7.67E-01
5.656569	0.008	0.007	2.97E-01	-0.012	0.011	2.88E-01
5.624608	0.011	0.009	2.26E-01	0.006	0.014	6.55E-01
1.48715953	-0.006	0.004	1.43E-01	-0.007	0.007	2.98E-01
6.464007	0.011	0.008	1.52E-01	0.001	0.012	9.14E-01
8.50077	0.009	0.009	2.98E-01	-0.002	0.013	8.57E-01
1.620050404	0.011	0.006	8.39E-02	0.002	0.010	8.05E-01
7.226363	-0.016	0.010	1.10E-01	-0.022	0.015	1.40E-01
7.679538	0.013	0.009	1.77E-01	-0.001	0.014	9.52E-01
3.159902	-0.012	0.007	9.47E-02	-0.005	0.011	6.69E-01
3.945472	-0.012	0.009	1.69E-01	-0.002	0.013	8.64E-01
0.611761928	0.011	0.007	1.43E-01	0.025	0.011	2.46E-02
3.256795	0.014	0.010	1.70E-01	0.019	0.015	1.94E-01
8.007896	0.012	0.008	1.57E-01	0.027	0.012	2.90E-02
0.953913873	0.009	0.005	1.06E-01	0.003	0.008	7.26E-01
1.54031588	0.021	0.013	1.14E-01	-0.015	0.020	4.53E-01
6.873782	0.016	0.009	9.18E-02	0.014	0.014	3.16E-01
6.028663	0.014	0.009	1.33E-01	0.009	0.013	4.91E-01
5.805272	0.010	0.008	1.97E-01	0.008	0.012	4.89E-01
3.520558	0.010	0.007	1.85E-01	0.031	0.011	3.74E-03
8.976822	0.016	0.010	1.00E-01	0.017	0.015	2.55E-01
3.038114	-0.012	0.008	1.35E-01	-0.004	0.011	7.10E-01
5.270008	-0.012	0.006	5.55E-02	-0.018	0.009	4.29E-02
5.020711	0.014	0.010	1.85E-01	-0.003	0.015	8.58E-01
8.469818	-0.011	0.010	2.38E-01	-0.026	0.015	7.33E-02
1.925531196	0.004	0.004	2.59E-01	0.005	0.006	3.65E-01
3.048207	-0.013	0.007	7.09E-02	-0.013	0.011	2.33E-01
5.851363	0.016	0.010	1.08E-01	0.015	0.014	3.11E-01
8.314386	0.011	0.009	2.29E-01	-0.007	0.013	5.91E-01
6.67495	0.015	0.010	1.36E-01	-0.003	0.015	8.34E-01
1.710550771	-0.006	0.004	1.65E-01	0.005	0.006	4.45E-01
2.271047468	0.009	0.006	1.35E-01	-0.003	0.009	7.73E-01
4.170546	-0.018	0.013	1.55E-01	0.001	0.019	9.61E-01
6.6985	0.011	0.010	2.40E-01	0.014	0.014	3.22E-01
5.255877	-0.010	0.005	4.36E-02	-0.009	0.008	2.26E-01
8.638371	0.011	0.007	1.20E-01	0.002	0.011	8.70E-01
1.886168583	-0.013	0.007	8.40E-02	-0.013	0.011	2.24E-01
4.326651	-0.019	0.014	1.78E-01	0.015	0.021	4.56E-01
7.739759	-0.012	0.011	2.57E-01	-0.025	0.016	1.14E-01
7.075305	0.012	0.008	1.51E-01	0.020	0.013	1.10E-01
3.629899	-0.008	0.006	2.02E-01	0.013	0.009	1.51E-01
2.377360166	-0.015	0.010	1.25E-01	0.009	0.014	5.37E-01
3.262851	0.011	0.008	1.55E-01	0.026	0.011	2.16E-02

5.853382	0.015	0.010	1.47E-01	0.007	0.016	6.57E-01
2.299307805	-0.008	0.005	1.18E-01	-0.007	0.007	3.25E-01
5.472876	0.010	0.009	2.78E-01	-0.012	0.014	3.88E-01
1.868674089	-0.012	0.009	1.98E-01	-0.017	0.013	2.02E-01
1.538297284	0.019	0.012	1.22E-01	-0.015	0.018	4.01E-01
6.571665	-0.013	0.009	1.44E-01	-0.026	0.013	5.12E-02
4.432963	-0.019	0.014	1.92E-01	0.009	0.021	6.69E-01
3.954556	-0.014	0.011	2.06E-01	-0.017	0.017	3.07E-01
7.64354	-0.010	0.010	3.13E-01	-0.016	0.014	2.70E-01
8.968075	0.014	0.009	9.55E-02	0.023	0.013	7.29E-02
6.517163	-0.015	0.011	1.69E-01	-0.033	0.016	4.27E-02
1.538970149	0.020	0.013	1.16E-01	-0.015	0.019	4.31E-01
5.983918	0.011	0.009	2.12E-01	0.004	0.013	7.44E-01
7.171861	-0.012	0.009	1.59E-01	-0.017	0.013	2.00E-01
1.632161977	0.010	0.006	1.26E-01	0.005	0.009	6.09E-01
0.73926988	-0.011	0.006	9.16E-02	-0.002	0.010	8.63E-01
2.257253731	0.012	0.007	8.78E-02	0.010	0.011	3.39E-01
2.145894544	-0.023	0.014	1.04E-01	-0.009	0.021	6.74E-01
3.791386	0.012	0.006	3.09E-02	0.012	0.008	1.54E-01
1.535605823	0.017	0.011	1.23E-01	-0.018	0.017	2.77E-01
1.677916809	-0.004	0.004	3.09E-01	0.003	0.006	5.55E-01
2.351118424	0.019	0.010	5.72E-02	0.013	0.015	3.87E-01
3.22147	-0.019	0.012	1.16E-01	-0.005	0.018	8.04E-01
1.833685099	-0.014	0.010	1.40E-01	-0.015	0.015	2.97E-01
7.946665	0.013	0.010	1.70E-01	0.012	0.014	4.13E-01
5.762545	0.011	0.009	1.99E-01	-0.007	0.013	5.98E-01
3.732511	0.009	0.008	2.92E-01	0.018	0.012	1.51E-01
3.250066	-0.011	0.006	6.55E-02	0.004	0.009	6.59E-01
2.672075116	-0.010	0.006	9.16E-02	0.005	0.009	5.49E-01
8.686817	0.008	0.007	2.24E-01	-0.001	0.010	9.03E-01
8.509853	0.010	0.008	1.94E-01	-0.011	0.011	3.24E-01
8.121274	0.012	0.009	1.91E-01	0.000	0.013	9.94E-01
3.587508	-0.006	0.006	2.96E-01	0.006	0.009	5.13E-01
7.031232	-0.013	0.010	1.93E-01	-0.020	0.015	1.80E-01
3.520222	0.009	0.007	1.82E-01	0.030	0.011	4.71E-03
1.989116956	0.014	0.008	8.80E-02	-0.003	0.012	7.99E-01
2.138156594	-0.015	0.010	1.46E-01	0.003	0.015	8.65E-01
2.760220455	-0.015	0.009	9.96E-02	-0.037	0.014	6.28E-03
1.259394666	0.020	0.013	1.06E-01	-0.007	0.019	7.20E-01
1.219359188	0.010	0.007	1.73E-01	0.004	0.011	6.83E-01
5.466821	0.008	0.006	2.20E-01	-0.009	0.009	3.12E-01
6.35433	-0.009	0.007	1.87E-01	-0.002	0.011	8.12E-01
6.705902	-0.009	0.007	2.12E-01	0.015	0.011	1.74E-01

7.38415	0.008	0.008	3.10E-01	0.019	0.012	1.02E-01
0.849956203	-0.011	0.007	1.03E-01	-0.014	0.010	1.74E-01
5.518968	0.009	0.009	2.78E-01	-0.021	0.013	1.08E-01
7.41241	-0.015	0.010	1.09E-01	-0.006	0.014	6.96E-01
2.383752386	-0.012	0.007	1.17E-01	-0.002	0.011	8.47E-01
2.685868852	-0.012	0.006	5.78E-02	-0.004	0.009	6.62E-01
4.351883	-0.021	0.014	1.47E-01	0.014	0.021	5.15E-01
8.310685	0.009	0.007	2.24E-01	0.012	0.011	2.71E-01
1.851852459	-0.009	0.008	2.59E-01	-0.002	0.012	8.77E-01
0.937765109	-0.007	0.005	2.12E-01	-0.019	0.008	1.34E-02
5.302305	0.023	0.015	1.21E-01	-0.016	0.022	4.79E-01
8.986915	0.009	0.007	2.18E-01	0.014	0.010	1.73E-01
2.349772694	0.018	0.009	4.75E-02	0.022	0.013	1.04E-01
5.302642	0.023	0.015	1.20E-01	-0.016	0.022	4.76E-01
8.148188	0.009	0.008	2.98E-01	0.013	0.012	3.07E-01
3.954893	-0.013	0.011	2.17E-01	-0.017	0.016	2.99E-01
6.582431	0.010	0.007	1.89E-01	0.008	0.011	4.92E-01
6.164918	-0.014	0.009	1.31E-01	-0.008	0.014	5.72E-01
5.263952	-0.011	0.006	4.95E-02	-0.014	0.009	9.18E-02
1.683972596	-0.005	0.004	2.17E-01	0.005	0.006	3.59E-01
1.539643014	0.020	0.013	1.19E-01	-0.015	0.019	4.43E-01
2.178192072	0.019	0.011	9.65E-02	-0.013	0.017	4.52E-01
8.128002	0.009	0.009	3.25E-01	-0.005	0.014	6.94E-01
5.677427	0.015	0.009	1.00E-01	-0.017	0.013	2.00E-01
5.302978	0.023	0.015	1.22E-01	-0.017	0.022	4.38E-01
5.435532	0.014	0.009	1.20E-01	-0.025	0.014	6.89E-02
8.866808	0.009	0.007	2.30E-01	0.004	0.011	7.21E-01
1.073347443	0.008	0.006	1.69E-01	0.003	0.009	7.44E-01
0.938774407	-0.006	0.005	2.38E-01	-0.021	0.008	7.50E-03
1.446787619	0.006	0.004	1.40E-01	0.006	0.006	3.33E-01
8.746702	-0.011	0.010	2.72E-01	-0.005	0.014	7.11E-01
8.098733	0.012	0.009	1.76E-01	-0.017	0.013	2.10E-01
8.635343	0.016	0.010	1.21E-01	-0.019	0.015	1.96E-01
6.45055	0.010	0.007	1.54E-01	0.000	0.011	9.84E-01
5.047962	0.014	0.010	1.76E-01	-0.009	0.015	5.66E-01
8.399504	0.011	0.008	1.60E-01	0.005	0.012	6.49E-01
8.261566	-0.010	0.008	2.06E-01	0.002	0.012	8.85E-01
0.780651089	0.006	0.004	1.69E-01	0.006	0.007	3.60E-01
8.135404	0.009	0.008	2.51E-01	-0.012	0.012	3.20E-01
2.758874725	-0.013	0.008	9.72E-02	-0.029	0.012	1.19E-02
5.107174	0.009	0.009	2.88E-01	0.006	0.013	6.46E-01
4.432627	-0.019	0.015	2.08E-01	0.011	0.022	6.34E-01
3.807535	0.008	0.006	1.96E-01	0.019	0.009	3.28E-02

5.669353	0.012	0.008	1.54E-01	0.003	0.012	8.03E-01
5.327538	0.028	0.019	1.39E-01	-0.016	0.028	5.81E-01
1.80912552	-0.017	0.011	1.22E-01	-0.004	0.017	8.14E-01
6.951498	-0.012	0.009	1.70E-01	0.005	0.013	6.86E-01
2.044964766	0.011	0.007	1.06E-01	0.007	0.010	4.81E-01
4.311175	-0.020	0.013	1.26E-01	0.022	0.020	2.63E-01
5.038542	0.013	0.010	1.85E-01	-0.004	0.014	7.71E-01
7.615952	-0.020	0.013	1.18E-01	-0.014	0.019	4.75E-01
0.591912405	-0.015	0.010	1.16E-01	-0.013	0.014	3.45E-01
5.140817	0.011	0.007	1.47E-01	0.005	0.011	6.27E-01
8.601027	0.014	0.010	1.36E-01	0.006	0.014	6.87E-01
5.037869	0.011	0.008	1.68E-01	-0.021	0.012	9.57E-02
8.84023	0.016	0.009	8.51E-02	-0.006	0.014	6.73E-01
0.584174456	0.015	0.008	5.78E-02	0.021	0.012	7.68E-02
2.75820186	-0.013	0.008	9.28E-02	-0.027	0.011	1.89E-02
3.337539	-0.015	0.010	1.25E-01	0.012	0.015	4.25E-01
3.542763	0.009	0.008	2.27E-01	0.023	0.012	5.05E-02
7.161768	0.016	0.010	1.04E-01	-0.004	0.015	7.95E-01
3.34965	-0.012	0.010	2.14E-01	-0.016	0.015	2.76E-01
8.202018	0.011	0.008	1.78E-01	-0.013	0.013	2.97E-01
3.661187	-0.008	0.005	1.05E-01	0.006	0.007	3.91E-01
7.960459	-0.009	0.007	1.91E-01	-0.014	0.011	1.96E-01
6.095277	0.013	0.010	1.78E-01	-0.005	0.014	7.17E-01
5.866839	0.016	0.009	8.68E-02	0.008	0.014	5.65E-01
0.843900416	-0.014	0.008	9.61E-02	-0.015	0.012	2.19E-01
0.911859799	-0.007	0.005	1.81E-01	-0.024	0.008	1.65E-03
3.626198	-0.009	0.007	1.89E-01	0.007	0.011	5.19E-01
2.593686322	-0.021	0.016	1.92E-01	0.001	0.024	9.80E-01
6.734499	-0.019	0.011	7.71E-02	-0.017	0.016	3.11E-01
2.191649376	0.017	0.011	1.23E-01	-0.017	0.017	3.02E-01
1.155773428	0.008	0.005	1.10E-01	-0.005	0.008	5.31E-01
3.945136	-0.012	0.009	1.75E-01	0.001	0.014	9.46E-01
7.649259	-0.011	0.011	3.19E-01	-0.018	0.016	2.59E-01
3.844206	0.008	0.006	2.04E-01	0.022	0.009	1.62E-02
1.158464889	0.008	0.005	8.16E-02	-0.006	0.007	4.06E-01
1.166202838	0.009	0.005	7.59E-02	0.000	0.008	9.92E-01
5.478932	0.011	0.008	1.38E-01	-0.029	0.012	1.16E-02
8.452323	0.017	0.013	1.98E-01	0.024	0.020	2.29E-01
1.259058233	0.020	0.012	1.09E-01	-0.007	0.018	7.08E-01
8.130021	0.011	0.008	1.89E-01	-0.012	0.012	3.38E-01
0.988902863	0.010	0.007	1.54E-01	0.010	0.011	3.67E-01
8.485294	0.013	0.009	1.26E-01	-0.001	0.013	9.58E-01
1.721653046	-0.008	0.005	1.38E-01	-0.002	0.008	8.01E-01

6.210337	0.010	0.008	2.23E-01	-0.013	0.012	2.58E-01
3.680027	-0.005	0.005	2.41E-01	0.012	0.007	7.77E-02
5.303314	0.023	0.015	1.25E-01	-0.018	0.022	4.23E-01
5.541509	0.010	0.008	2.09E-01	0.011	0.012	3.52E-01
1.69877563	-0.005	0.004	2.21E-01	0.003	0.006	5.79E-01
1.473365794	-0.006	0.004	1.89E-01	-0.001	0.006	8.29E-01
0.570717152	-0.011	0.009	2.27E-01	-0.004	0.014	7.55E-01
8.011933	-0.011	0.009	2.24E-01	-0.003	0.013	8.47E-01
6.960581	-0.017	0.010	8.99E-02	-0.016	0.015	2.69E-01
8.420362	0.011	0.008	1.29E-01	-0.008	0.011	4.55E-01
8.13675	0.016	0.010	8.72E-02	-0.009	0.014	5.21E-01
8.780682	0.014	0.008	9.80E-02	0.005	0.012	7.01E-01
1.473702227	-0.006	0.004	1.78E-01	-0.002	0.006	7.55E-01
6.587814	0.010	0.009	2.73E-01	-0.004	0.013	7.76E-01
6.773861	-0.012	0.008	1.59E-01	-0.004	0.012	7.67E-01
2.800928799	-0.020	0.014	1.62E-01	-0.073	0.021	6.82E-04
1.539979447	0.020	0.013	1.23E-01	-0.015	0.019	4.40E-01
6.135985	0.011	0.008	1.89E-01	-0.011	0.012	3.90E-01
2.292915586	-0.005	0.004	2.57E-01	-0.011	0.007	8.60E-02
0.850292635	-0.011	0.007	1.09E-01	-0.014	0.010	1.77E-01
2.338670418	0.015	0.008	6.51E-02	0.012	0.012	3.24E-01
1.449815513	0.006	0.004	1.29E-01	0.005	0.006	3.88E-01
7.246213	-0.018	0.011	8.19E-02	-0.021	0.016	1.73E-01
1.834021532	-0.014	0.010	1.39E-01	-0.023	0.014	1.07E-01
1.963548079	0.007	0.005	1.41E-01	0.002	0.007	7.25E-01
8.947216	0.009	0.009	3.09E-01	-0.006	0.013	6.65E-01
1.166539271	0.009	0.005	7.57E-02	0.000	0.007	9.88E-01
0.814630781	0.005	0.005	2.97E-01	0.016	0.008	3.19E-02
7.238811	-0.012	0.008	1.16E-01	-0.002	0.011	8.53E-01
3.046861	-0.010	0.007	1.35E-01	-0.014	0.010	1.62E-01
1.073683876	0.008	0.006	2.02E-01	0.003	0.009	7.43E-01
3.220797	-0.021	0.013	1.25E-01	-0.007	0.020	7.18E-01
7.830596	-0.013	0.010	1.81E-01	0.001	0.015	9.41E-01
1.909046	-0.010	0.006	8.96E-02	-0.020	0.009	2.62E-02
2.717157083	-0.012	0.006	6.81E-02	-0.018	0.010	5.64E-02
3.327109	0.015	0.009	1.12E-01	0.014	0.014	3.03E-01
7.586682	0.011	0.008	1.91E-01	0.019	0.012	1.29E-01
1.777500856	-0.013	0.010	1.98E-01	-0.007	0.015	6.19E-01
1.698439197	-0.005	0.004	2.16E-01	0.006	0.006	3.37E-01
5.135098	0.011	0.008	1.53E-01	0.009	0.011	4.36E-01
5.026767	0.011	0.009	1.95E-01	-0.015	0.013	2.40E-01
2.600414974	-0.016	0.012	1.90E-01	0.013	0.018	4.66E-01
5.592983	0.012	0.010	2.04E-01	-0.008	0.014	5.91E-01

8.850323	-0.011	0.009	2.05E-01	-0.021	0.013	1.15E-01
1.445441889	0.006	0.004	1.36E-01	0.002	0.006	7.18E-01
3.254776	-0.013	0.009	1.82E-01	0.002	0.014	8.62E-01
2.257926597	0.010	0.007	1.21E-01	0.009	0.010	3.79E-01
2.758538292	-0.013	0.008	9.70E-02	-0.028	0.012	1.70E-02
7.041662	-0.012	0.011	2.79E-01	-0.024	0.017	1.65E-01
3.597601	0.011	0.007	1.18E-01	0.019	0.011	7.33E-02
3.036432	-0.010	0.008	1.75E-01	-0.017	0.012	1.39E-01
3.391704	0.010	0.009	2.88E-01	0.043	0.014	1.44E-03
3.063683	-0.009	0.008	2.51E-01	0.005	0.011	6.52E-01
3.581452	-0.007	0.006	2.30E-01	0.007	0.009	4.51E-01
1.00673379	0.011	0.007	1.55E-01	0.014	0.011	2.18E-01
6.09494	0.010	0.007	1.78E-01	0.002	0.011	8.26E-01
6.842494	0.011	0.008	1.64E-01	0.015	0.012	2.22E-01
5.159658	0.013	0.009	1.70E-01	-0.019	0.014	1.61E-01
7.342769	0.014	0.011	1.84E-01	0.023	0.016	1.44E-01
4.434982	-0.022	0.017	1.95E-01	0.017	0.025	4.87E-01
6.779244	0.013	0.008	1.19E-01	0.020	0.013	1.13E-01
1.820900661	-0.013	0.010	1.69E-01	-0.003	0.014	8.35E-01
1.927886225	0.006	0.004	1.58E-01	0.003	0.006	6.55E-01
7.315181	-0.010	0.007	1.40E-01	-0.001	0.010	9.23E-01
6.460306	0.009	0.007	1.81E-01	-0.001	0.010	8.90E-01
5.096745	0.012	0.009	1.71E-01	0.009	0.014	5.13E-01
1.116074382	0.011	0.007	1.13E-01	-0.010	0.011	3.66E-01
4.242879	0.022	0.015	1.30E-01	-0.004	0.022	8.41E-01
3.225507	-0.011	0.007	1.43E-01	0.005	0.011	6.25E-01
7.093809	-0.013	0.010	1.78E-01	0.002	0.015	8.82E-01
6.832401	0.014	0.010	1.83E-01	0.015	0.016	3.33E-01
5.39045	0.014	0.009	1.08E-01	0.001	0.013	9.41E-01
5.706361	0.007	0.007	2.80E-01	-0.012	0.010	2.56E-01
6.000739	0.010	0.008	2.34E-01	0.011	0.012	3.67E-01
1.044750673	0.006	0.005	2.44E-01	0.013	0.008	9.93E-02
1.07940323	0.011	0.008	1.33E-01	0.005	0.011	6.64E-01
1.258721801	0.019	0.012	1.12E-01	-0.007	0.018	6.97E-01
1.474038659	-0.006	0.004	1.50E-01	-0.001	0.006	8.36E-01
6.525238	0.009	0.007	2.11E-01	-0.012	0.011	2.67E-01
4.243215	0.023	0.015	1.32E-01	-0.003	0.023	8.92E-01
6.740218	-0.014	0.008	6.66E-02	-0.011	0.011	3.17E-01
5.496763	0.012	0.008	1.24E-01	0.012	0.012	3.22E-01
7.451437	0.008	0.008	2.79E-01	0.004	0.012	7.60E-01
5.501137	0.011	0.008	1.86E-01	-0.002	0.012	8.74E-01
8.050623	0.011	0.009	2.46E-01	0.007	0.014	6.17E-01
6.132284	0.010	0.007	1.82E-01	0.016	0.011	1.43E-01

3.84387	0.008	0.006	2.02E-01	0.020	0.009	2.85E-02
7.188683	-0.015	0.012	1.92E-01	-0.004	0.017	8.02E-01
5.595001	0.011	0.007	1.28E-01	-0.002	0.011	8.66E-01
1.708195743	-0.007	0.005	1.60E-01	0.000	0.007	9.83E-01
6.726424	-0.016	0.010	1.27E-01	-0.005	0.015	7.28E-01
1.906018106	-0.009	0.006	1.35E-01	0.000	0.009	9.92E-01
5.156966	0.014	0.009	1.18E-01	-0.004	0.013	7.39E-01
6.460643	0.011	0.008	2.08E-01	-0.005	0.013	7.09E-01
3.568668	-0.008	0.007	2.33E-01	0.001	0.011	9.18E-01
1.762697822	-0.014	0.009	1.31E-01	0.008	0.014	5.49E-01
8.948898	-0.011	0.008	2.01E-01	-0.015	0.013	2.27E-01
1.454525569	0.005	0.004	1.53E-01	0.000	0.005	9.87E-01
2.043619036	0.010	0.006	1.03E-01	0.006	0.009	5.29E-01
5.249822	-0.011	0.005	3.63E-02	-0.009	0.007	2.23E-01
1.416845119	0.007	0.004	9.54E-02	0.008	0.007	2.25E-01
1.535942256	0.017	0.011	1.29E-01	-0.017	0.017	3.23E-01
3.765145	0.015	0.010	1.08E-01	0.026	0.014	6.48E-02
1.45082481	0.007	0.004	7.53E-02	0.002	0.006	7.21E-01
3.440824	0.010	0.007	1.46E-01	0.022	0.011	3.67E-02
2.872589	-0.011	0.009	2.31E-01	-0.002	0.014	9.03E-01
8.197308	0.011	0.006	8.68E-02	0.001	0.010	8.77E-01
3.21575	-0.022	0.015	1.28E-01	-0.012	0.022	5.96E-01
2.046646929	0.011	0.007	1.13E-01	0.009	0.010	3.96E-01
7.075978	0.013	0.010	1.76E-01	0.013	0.014	3.50E-01
3.520895	0.009	0.007	2.08E-01	0.032	0.011	3.69E-03
1.118092978	0.011	0.007	1.20E-01	-0.004	0.010	7.08E-01
2.620264497	-0.016	0.012	1.95E-01	0.016	0.019	3.95E-01
1.252329582	0.014	0.009	1.25E-01	-0.008	0.013	5.47E-01
8.326161	-0.009	0.008	2.88E-01	-0.017	0.012	1.78E-01
3.043833	-0.004	0.005	3.77E-01	-0.001	0.008	8.64E-01
7.267744	-0.015	0.010	1.27E-01	0.008	0.015	6.15E-01
0.843563983	-0.014	0.008	9.97E-02	-0.015	0.012	2.36E-01
5.048971	0.013	0.010	1.82E-01	-0.002	0.015	8.99E-01
1.025237583	0.008	0.005	1.43E-01	0.010	0.008	2.36E-01
6.53634	0.008	0.007	2.31E-01	0.013	0.010	2.05E-01
2.046310497	0.011	0.007	1.12E-01	0.009	0.010	4.10E-01
2.166753364	0.017	0.011	1.06E-01	-0.007	0.016	6.58E-01
0.668282603	-0.014	0.010	1.81E-01	-0.037	0.015	1.34E-02
1.140970394	0.011	0.007	8.70E-02	-0.006	0.010	5.47E-01
0.651124541	-0.014	0.009	1.02E-01	-0.010	0.013	4.27E-01
1.146353315	0.010	0.006	8.96E-02	-0.003	0.009	7.18E-01
4.402348	-0.016	0.012	1.86E-01	0.005	0.018	7.67E-01
8.533404	0.009	0.006	1.16E-01	-0.005	0.008	5.55E-01

7.404	-0.013	0.010	2.00E-01	0.013	0.015	4.05E-01
5.301969	0.022	0.015	1.29E-01	-0.016	0.022	4.71E-01
1.021536824	0.012	0.007	7.76E-02	0.007	0.010	5.22E-01
1.537960851	0.018	0.012	1.29E-01	-0.014	0.018	4.43E-01
1.251320284	0.013	0.009	1.29E-01	-0.008	0.013	5.42E-01
1.101271348	0.012	0.008	1.15E-01	-0.003	0.012	7.72E-01
7.358581	-0.014	0.011	2.00E-01	-0.032	0.016	4.15E-02
6.016215	0.011	0.008	1.68E-01	0.008	0.012	5.20E-01
2.405284071	-0.016	0.011	1.33E-01	0.004	0.016	8.17E-01
8.511199	-0.010	0.008	1.77E-01	-0.016	0.011	1.54E-01
1.258385368	0.019	0.012	1.14E-01	-0.007	0.018	6.93E-01
6.129593	0.009	0.009	2.88E-01	0.000	0.013	9.95E-01
1.04441424	0.006	0.005	3.03E-01	0.019	0.008	1.56E-02
2.716484218	-0.012	0.006	6.17E-02	-0.018	0.009	5.97E-02
3.042487	-0.005	0.005	3.16E-01	0.001	0.008	8.63E-01
5.86953	0.012	0.008	1.45E-01	-0.005	0.012	7.00E-01
7.143601	-0.010	0.008	1.87E-01	-0.002	0.012	8.35E-01
3.050225	-0.015	0.008	8.02E-02	0.007	0.013	5.72E-01
8.269304	0.008	0.008	2.81E-01	-0.009	0.011	4.25E-01
1.251993149	0.014	0.009	1.27E-01	-0.008	0.013	5.46E-01
3.988199	-0.011	0.007	1.21E-01	0.005	0.010	6.24E-01
5.101791	0.011	0.009	2.26E-01	0.008	0.013	5.53E-01
6.15247	0.014	0.010	1.55E-01	0.009	0.015	5.51E-01
2.294934182	-0.008	0.006	1.88E-01	-0.002	0.009	8.27E-01
1.988780524	0.014	0.008	9.59E-02	-0.003	0.012	7.91E-01
7.956085	0.007	0.006	2.36E-01	-0.005	0.009	5.76E-01
2.178864938	0.018	0.011	1.18E-01	-0.014	0.017	4.01E-01
7.399289	0.014	0.008	8.18E-02	-0.010	0.012	4.04E-01
1.800714705	-0.013	0.010	2.09E-01	0.000	0.015	9.85E-01
5.167396	0.019	0.012	1.07E-01	-0.012	0.018	4.81E-01
3.983489	-0.011	0.009	2.02E-01	-0.009	0.013	4.90E-01
2.096438953	-0.009	0.008	2.58E-01	-0.008	0.012	5.12E-01
7.582982	0.015	0.010	1.52E-01	0.027	0.016	8.52E-02
3.872467	-0.006	0.005	1.57E-01	0.009	0.007	2.03E-01
0.706299486	0.009	0.008	2.37E-01	0.020	0.012	8.73E-02
1.406079276	0.009	0.005	9.02E-02	0.012	0.008	1.43E-01
3.843533	0.008	0.006	2.00E-01	0.017	0.009	5.95E-02
4.426908	-0.020	0.015	1.78E-01	0.013	0.022	5.41E-01
1.251656716	0.013	0.009	1.28E-01	-0.008	0.013	5.40E-01
6.736854	-0.015	0.008	6.31E-02	-0.023	0.012	5.40E-02
5.419047	0.011	0.008	1.53E-01	-0.010	0.012	3.76E-01
8.717432	-0.016	0.010	9.07E-02	-0.031	0.014	2.83E-02
7.659015	0.009	0.007	1.84E-01	0.004	0.010	7.25E-01

8.234315	0.012	0.009	1.59E-01	-0.006	0.013	6.29E-01
5.301632	0.022	0.015	1.30E-01	-0.015	0.022	4.84E-01
7.186664	-0.011	0.009	2.47E-01	-0.024	0.014	8.49E-02
3.378584	0.014	0.013	2.80E-01	0.016	0.019	3.94E-01
1.11842941	0.011	0.007	1.26E-01	-0.004	0.010	6.79E-01
8.057688	0.009	0.007	1.88E-01	-0.012	0.011	2.80E-01
4.144641	-0.016	0.013	1.97E-01	0.016	0.019	3.97E-01
5.262606	-0.011	0.006	5.18E-02	-0.014	0.008	9.39E-02
0.936755811	-0.006	0.005	2.23E-01	-0.019	0.008	1.17E-02
6.927611	-0.017	0.011	1.29E-01	0.020	0.016	2.14E-01
1.006397357	0.011	0.008	1.53E-01	0.014	0.011	2.31E-01
5.092708	0.013	0.010	1.86E-01	-0.001	0.014	9.16E-01
7.805364	-0.013	0.008	8.43E-02	-0.021	0.011	5.79E-02
8.552244	-0.011	0.009	2.43E-01	0.015	0.014	2.77E-01
6.81928	0.008	0.009	3.50E-01	-0.002	0.013	8.69E-01
7.064539	-0.012	0.010	2.38E-01	-0.020	0.016	2.03E-01
5.303651	0.022	0.015	1.31E-01	-0.017	0.022	4.26E-01
8.838548	0.014	0.010	1.66E-01	-0.014	0.015	3.51E-01
8.096041	0.012	0.008	1.49E-01	-0.014	0.012	2.67E-01
1.124485197	0.010	0.007	1.38E-01	-0.009	0.010	3.61E-01
2.514624664	-0.020	0.014	1.56E-01	-0.024	0.021	2.48E-01
7.96887	0.009	0.008	2.24E-01	0.009	0.011	4.31E-01
7.697369	-0.015	0.011	1.72E-01	-0.001	0.016	9.55E-01
1.763034255	-0.014	0.009	1.36E-01	0.008	0.014	5.86E-01
8.050287	0.012	0.009	1.93E-01	0.012	0.013	3.65E-01
1.964557377	0.007	0.005	1.47E-01	0.001	0.007	9.34E-01
1.803742598	-0.012	0.010	2.17E-01	0.003	0.015	8.43E-01
4.378798	-0.018	0.013	1.76E-01	0.008	0.020	6.90E-01
7.559431	0.011	0.009	2.52E-01	0.006	0.014	6.80E-01
5.725874	0.010	0.010	2.95E-01	0.015	0.015	2.97E-01
2.513615366	-0.017	0.012	1.62E-01	-0.014	0.018	4.33E-01
3.377238	0.015	0.012	2.25E-01	0.008	0.019	6.63E-01
7.35959	-0.013	0.009	1.64E-01	-0.016	0.014	2.67E-01
3.231563	0.008	0.006	1.85E-01	0.025	0.009	6.49E-03
7.360263	-0.015	0.010	1.31E-01	0.017	0.015	2.62E-01
1.252666014	0.014	0.009	1.27E-01	-0.008	0.014	5.44E-01
2.313774407	-0.007	0.005	1.58E-01	-0.005	0.008	4.84E-01
4.437	-0.020	0.016	1.91E-01	0.021	0.023	3.64E-01
1.253002447	0.014	0.009	1.26E-01	-0.008	0.014	5.53E-01
4.397301	-0.019	0.015	2.03E-01	0.013	0.022	5.36E-01
5.768264	0.009	0.009	2.93E-01	-0.005	0.013	6.89E-01
7.470277	0.010	0.008	2.51E-01	-0.023	0.012	6.68E-02
1.486486665	-0.005	0.004	2.37E-01	-0.001	0.007	8.89E-01

3.111793	-0.017	0.010	9.50E-02	-0.004	0.015	7.74E-01
6.257101	-0.013	0.009	1.54E-01	-0.019	0.014	1.71E-01
2.396873257	-0.015	0.013	2.58E-01	0.001	0.020	9.75E-01
3.660178	-0.007	0.005	1.24E-01	0.005	0.007	4.37E-01
8.496733	-0.016	0.009	7.11E-02	-0.016	0.014	2.51E-01
3.378247	0.014	0.013	2.70E-01	0.016	0.019	4.12E-01
3.11583	-0.016	0.010	1.11E-01	0.003	0.015	8.54E-01
6.591851	-0.009	0.006	1.44E-01	-0.014	0.009	1.21E-01
1.409107169	0.009	0.006	9.88E-02	0.016	0.009	6.32E-02
0.737587717	-0.009	0.007	1.54E-01	-0.005	0.010	6.22E-01
7.859193	0.007	0.006	2.62E-01	0.005	0.009	5.49E-01
6.798757	0.014	0.009	1.43E-01	0.018	0.014	2.04E-01
5.56876	0.011	0.007	1.33E-01	-0.006	0.011	5.97E-01
4.145313	-0.016	0.012	1.86E-01	0.013	0.018	4.86E-01
8.375617	-0.010	0.008	2.26E-01	0.022	0.012	6.67E-02
1.988444091	0.013	0.008	1.01E-01	-0.003	0.012	7.85E-01
3.612741	-0.011	0.008	2.06E-01	0.011	0.013	3.93E-01
5.843625	0.012	0.009	1.77E-01	0.012	0.013	3.57E-01
1.253338879	0.014	0.009	1.26E-01	-0.008	0.014	5.55E-01
8.019335	0.010	0.008	2.50E-01	-0.028	0.013	2.70E-02
1.695074872	-0.005	0.004	1.95E-01	0.002	0.006	7.12E-01
3.56093	-0.012	0.008	1.60E-01	0.022	0.012	8.43E-02
8.172075	-0.009	0.007	1.95E-01	-0.005	0.010	6.49E-01
2.299644238	-0.007	0.005	1.32E-01	-0.007	0.007	3.60E-01
5.706697	0.009	0.009	2.98E-01	-0.005	0.013	6.94E-01
2.62194666	-0.016	0.013	2.09E-01	0.011	0.019	5.65E-01
8.658557	0.013	0.009	1.63E-01	-0.004	0.014	7.92E-01
4.320931	-0.018	0.013	1.87E-01	0.015	0.020	4.45E-01
6.080137	0.011	0.007	1.18E-01	0.010	0.010	3.12E-01
1.795331784	-0.009	0.007	2.38E-01	0.018	0.011	1.03E-01
3.266888	-0.009	0.008	2.27E-01	-0.010	0.011	3.65E-01
0.536401028	0.012	0.009	1.66E-01	0.016	0.013	2.29E-01
1.100598483	0.010	0.008	2.23E-01	-0.008	0.012	4.97E-01
0.519579398	0.009	0.009	2.95E-01	-0.016	0.013	2.23E-01
5.895772	0.018	0.010	7.14E-02	0.013	0.015	3.75E-01
6.366105	0.014	0.010	1.56E-01	0.008	0.015	5.80E-01
3.790377	0.011	0.006	6.10E-02	0.014	0.009	9.96E-02
8.405559	0.012	0.010	2.39E-01	0.024	0.015	1.23E-01
8.924002	-0.011	0.009	2.13E-01	-0.002	0.013	8.58E-01
5.634028	-0.011	0.008	1.75E-01	-0.011	0.013	3.90E-01
1.434003181	0.007	0.004	8.56E-02	0.004	0.006	5.14E-01
1.736119648	-0.009	0.005	8.53E-02	0.006	0.008	4.57E-01
3.212722	-0.025	0.016	1.12E-01	-0.013	0.024	5.75E-01

5.436205	0.012	0.009	1.62E-01	-0.003	0.013	8.08E-01
5.472204	0.010	0.008	2.53E-01	-0.004	0.013	7.64E-01
8.822736	-0.013	0.008	1.22E-01	0.002	0.012	9.03E-01
1.249638121	0.012	0.008	1.36E-01	-0.008	0.012	5.18E-01
3.975415	-0.010	0.009	2.70E-01	0.008	0.014	5.49E-01
8.129685	0.009	0.007	2.09E-01	-0.006	0.011	5.90E-01
8.828792	0.009	0.009	3.03E-01	0.017	0.013	1.92E-01
4.280896	0.020	0.012	1.05E-01	0.018	0.018	3.35E-01
5.924369	-0.010	0.008	2.38E-01	0.004	0.013	7.61E-01
6.816588	0.011	0.009	2.46E-01	0.016	0.014	2.42E-01
1.435348911	0.006	0.004	1.48E-01	0.008	0.006	2.08E-01
3.22046	-0.021	0.014	1.35E-01	-0.008	0.021	6.88E-01
3.381948	0.013	0.010	2.17E-01	0.025	0.015	9.98E-02
1.406415708	0.009	0.005	1.05E-01	0.011	0.008	1.63E-01
1.250983851	0.013	0.009	1.33E-01	-0.008	0.013	5.18E-01
5.313071	0.024	0.017	1.47E-01	-0.021	0.025	4.03E-01
7.757254	-0.014	0.010	1.75E-01	-0.023	0.016	1.49E-01
2.191312943	0.017	0.011	1.34E-01	-0.019	0.017	2.71E-01
1.249301688	0.012	0.008	1.37E-01	-0.008	0.012	5.16E-01
6.137331	0.008	0.008	3.46E-01	-0.001	0.013	9.37E-01
6.560227	0.009	0.010	3.39E-01	0.016	0.015	2.85E-01
0.951558845	0.007	0.005	1.31E-01	-0.001	0.007	8.27E-01
1.246273795	0.011	0.008	1.49E-01	-0.008	0.012	4.84E-01
4.144977	-0.018	0.014	1.97E-01	0.016	0.021	4.45E-01
2.837599951	-0.010	0.009	2.89E-01	-0.027	0.014	4.83E-02
5.130052	0.013	0.009	1.37E-01	-0.003	0.013	7.99E-01
2.863169	-0.010	0.008	1.94E-01	-0.006	0.012	5.94E-01
5.70737	0.013	0.011	2.33E-01	-0.006	0.017	7.29E-01
1.039704184	0.009	0.007	2.08E-01	0.004	0.010	7.03E-01
4.311511	-0.019	0.013	1.36E-01	0.017	0.019	3.60E-01
4.310502	-0.022	0.015	1.54E-01	0.028	0.023	2.33E-01
8.37629	0.009	0.008	2.32E-01	0.012	0.011	2.73E-01
1.628797651	0.010	0.006	1.16E-01	-0.001	0.009	9.27E-01
1.988107658	0.013	0.008	1.03E-01	-0.004	0.012	7.64E-01
7.691649	0.013	0.009	1.32E-01	0.011	0.013	4.14E-01
7.023831	-0.015	0.009	1.21E-01	-0.028	0.014	5.04E-02
1.248965256	0.012	0.008	1.39E-01	-0.008	0.012	5.19E-01
0.843227551	-0.014	0.008	1.03E-01	-0.014	0.012	2.44E-01
7.569861	-0.014	0.010	1.36E-01	-0.016	0.014	2.71E-01
7.645222	-0.010	0.011	3.26E-01	0.007	0.016	6.45E-01
0.778296061	0.006	0.005	1.80E-01	-0.002	0.007	7.19E-01
5.494408	0.011	0.009	2.04E-01	-0.019	0.013	1.35E-01
1.053161488	0.010	0.006	1.23E-01	-0.004	0.009	6.55E-01

4.434645	-0.021	0.017	2.09E-01	0.015	0.025	5.45E-01
2.99505	-0.006	0.004	1.56E-01	0.003	0.006	5.93E-01
1.246610228	0.011	0.008	1.49E-01	-0.008	0.012	4.87E-01
7.644212	-0.011	0.011	3.20E-01	-0.011	0.016	4.72E-01
1.250647419	0.013	0.008	1.35E-01	-0.008	0.013	5.18E-01
1.027929043	0.009	0.007	2.12E-01	0.005	0.010	6.22E-01
8.497742	-0.016	0.010	1.03E-01	0.016	0.015	2.75E-01
3.496671	0.010	0.010	2.86E-01	0.033	0.014	2.32E-02
7.735049	-0.017	0.011	1.39E-01	-0.021	0.017	2.17E-01
1.247955958	0.012	0.008	1.44E-01	-0.008	0.012	4.93E-01
7.496855	0.007	0.006	2.41E-01	0.021	0.009	2.67E-02
2.09610252	-0.009	0.008	2.66E-01	-0.007	0.012	5.64E-01
6.417916	0.009	0.007	2.18E-01	-0.005	0.011	6.26E-01
3.166295	-0.009	0.007	1.60E-01	0.011	0.010	2.43E-01
6.843503	0.009	0.008	2.55E-01	0.006	0.012	6.04E-01
1.258048936	0.018	0.012	1.20E-01	-0.007	0.017	6.82E-01
7.7623	-0.013	0.010	1.89E-01	-0.028	0.015	6.29E-02
2.29089699	-0.006	0.005	2.15E-01	-0.008	0.007	2.23E-01
5.444953	-0.010	0.008	2.29E-01	-0.008	0.012	4.93E-01
0.806556398	0.005	0.005	3.51E-01	0.013	0.008	9.95E-02
6.224803	0.008	0.007	2.69E-01	-0.008	0.011	4.48E-01
2.715811353	-0.011	0.006	6.36E-02	-0.017	0.009	6.77E-02
2.375005138	-0.026	0.018	1.37E-01	-0.001	0.026	9.81E-01
5.462447	0.009	0.008	2.67E-01	0.001	0.012	9.10E-01
4.049094	0.022	0.013	9.01E-02	-0.006	0.019	7.62E-01
5.566068	0.011	0.008	2.04E-01	0.002	0.013	8.85E-01
0.564324933	-0.013	0.010	1.74E-01	-0.006	0.015	6.85E-01
2.534810619	-0.019	0.012	1.14E-01	-0.005	0.018	7.95E-01
1.447460484	0.006	0.004	1.41E-01	0.003	0.006	5.72E-01
2.289887693	-0.005	0.005	2.39E-01	-0.011	0.007	1.02E-01
2.868888	-0.012	0.009	1.97E-01	0.010	0.013	4.43E-01
8.207064	0.009	0.006	1.80E-01	0.002	0.010	8.59E-01
2.311419379	-0.016	0.011	1.54E-01	-0.004	0.016	8.19E-01
3.677336	-0.005	0.004	2.26E-01	0.004	0.007	5.44E-01
1.248628823	0.012	0.008	1.42E-01	-0.008	0.012	5.12E-01
1.963884512	0.007	0.005	1.51E-01	0.002	0.007	8.02E-01
1.253675312	0.014	0.009	1.28E-01	-0.008	0.014	5.48E-01
4.326314	-0.018	0.014	1.94E-01	0.017	0.021	4.26E-01
8.726852	0.010	0.008	1.96E-01	0.003	0.012	8.28E-01
3.065365	-0.010	0.008	2.13E-01	0.000	0.012	9.92E-01
7.660698	0.012	0.009	1.65E-01	0.004	0.013	7.73E-01
3.679018	-0.005	0.005	2.45E-01	0.007	0.007	3.20E-01
1.250310986	0.013	0.008	1.36E-01	-0.008	0.013	5.19E-01

8.156263	0.008	0.008	3.13E-01	-0.008	0.012	5.24E-01
7.50392	-0.013	0.010	1.84E-01	-0.018	0.015	2.31E-01
2.371977245	-0.010	0.007	1.57E-01	0.004	0.011	6.82E-01
1.634517005	0.011	0.007	1.13E-01	0.003	0.010	7.39E-01
0.540438219	0.012	0.008	1.34E-01	0.006	0.012	5.88E-01
1.245264497	0.011	0.008	1.55E-01	-0.008	0.011	4.95E-01
2.671402251	-0.008	0.005	1.42E-01	0.010	0.008	1.94E-01
0.944157328	-0.006	0.005	2.14E-01	-0.001	0.007	8.31E-01
2.874608	-0.010	0.009	2.73E-01	0.004	0.013	7.67E-01
5.263279	-0.011	0.006	5.52E-02	-0.014	0.008	9.23E-02
1.248292391	0.012	0.008	1.44E-01	-0.008	0.012	4.98E-01
1.244591632	0.011	0.008	1.58E-01	-0.008	0.011	5.10E-01
1.24560093	0.011	0.008	1.54E-01	-0.008	0.012	4.89E-01
6.949479	-0.013	0.009	1.26E-01	-0.006	0.013	6.59E-01
1.249974553	0.012	0.008	1.38E-01	-0.008	0.012	5.16E-01
8.463089	-0.010	0.008	1.98E-01	-0.023	0.012	4.46E-02
1.828302178	-0.018	0.011	9.27E-02	-0.005	0.016	7.70E-01
2.358183509	0.012	0.008	1.13E-01	0.013	0.012	2.65E-01
1.24694666	0.011	0.008	1.50E-01	-0.008	0.012	4.96E-01
3.156202	-0.012	0.008	1.63E-01	0.001	0.013	9.14E-01
7.47465	0.011	0.008	1.77E-01	-0.007	0.012	5.92E-01
2.425133594	-0.010	0.009	2.38E-01	0.023	0.013	6.97E-02
6.825672	0.009	0.009	2.78E-01	0.001	0.013	9.63E-01
5.916631	0.013	0.010	1.92E-01	-0.006	0.014	6.99E-01
1.407761439	0.010	0.006	8.58E-02	0.018	0.009	3.54E-02
7.994775	0.011	0.008	1.89E-01	0.007	0.013	5.90E-01
1.254011745	0.015	0.010	1.28E-01	-0.009	0.014	5.50E-01
1.25737607	0.017	0.011	1.22E-01	-0.007	0.017	6.63E-01
8.699265	0.009	0.008	2.71E-01	-0.003	0.012	8.13E-01
1.244928065	0.011	0.008	1.57E-01	-0.008	0.011	5.05E-01
1.4858138	-0.005	0.004	2.15E-01	-0.003	0.007	6.43E-01
1.329372645	0.018	0.009	5.46E-02	0.010	0.014	4.71E-01
5.577507	0.013	0.009	1.69E-01	-0.012	0.014	4.07E-01
4.446757	-0.019	0.016	2.42E-01	0.020	0.024	4.12E-01
1.157119158	0.008	0.005	9.76E-02	-0.002	0.007	7.49E-01
5.271017	-0.011	0.006	6.65E-02	-0.018	0.009	4.52E-02
7.037961	-0.022	0.012	6.52E-02	-0.002	0.018	8.97E-01
1.241563739	0.010	0.007	1.77E-01	-0.007	0.011	5.18E-01
5.970797	0.009	0.008	2.87E-01	0.011	0.012	3.76E-01
1.245937362	0.011	0.008	1.53E-01	-0.008	0.012	4.88E-01
8.125984	-0.010	0.010	3.03E-01	0.018	0.014	2.10E-01
8.603718	0.007	0.007	3.06E-01	-0.002	0.010	8.37E-01
3.651767	-0.006	0.005	1.80E-01	0.006	0.007	4.10E-01

5.312735	0.024	0.017	1.49E-01	-0.021	0.025	4.09E-01
1.247283093	0.011	0.008	1.49E-01	-0.008	0.012	4.87E-01
4.157425	-0.020	0.016	2.12E-01	0.007	0.023	7.74E-01
2.716820651	-0.011	0.006	6.92E-02	-0.017	0.009	7.32E-02
1.25603034	0.016	0.010	1.24E-01	-0.008	0.016	6.22E-01
3.433422	0.012	0.009	2.12E-01	0.028	0.014	4.30E-02
5.499791	0.015	0.012	2.05E-01	0.003	0.018	8.50E-01
4.313866	-0.020	0.014	1.71E-01	0.008	0.021	7.08E-01
3.629562	-0.007	0.006	2.14E-01	0.013	0.009	1.50E-01
6.735844	-0.010	0.006	1.06E-01	-0.006	0.009	5.19E-01
1.247619525	0.011	0.008	1.48E-01	-0.008	0.012	4.92E-01
1.21969562	0.009	0.007	1.90E-01	0.004	0.011	6.94E-01
2.606807194	-0.017	0.012	1.80E-01	0.009	0.018	6.40E-01
1.256366773	0.016	0.011	1.25E-01	-0.008	0.016	6.28E-01
6.418252	0.009	0.007	2.02E-01	0.008	0.011	4.63E-01
1.537624419	0.018	0.012	1.33E-01	-0.015	0.018	4.04E-01
6.773525	-0.009	0.008	2.38E-01	-0.004	0.012	7.57E-01
1.257039638	0.017	0.011	1.24E-01	-0.008	0.016	6.48E-01
0.633293614	0.012	0.008	1.74E-01	-0.003	0.013	8.04E-01
7.766001	-0.013	0.009	1.51E-01	-0.026	0.013	4.99E-02
1.417181551	0.007	0.005	1.04E-01	0.006	0.007	3.85E-01
7.722938	-0.011	0.008	1.68E-01	-0.014	0.011	2.22E-01
5.301296	0.022	0.015	1.39E-01	-0.016	0.022	4.73E-01
2.545240029	-0.042	0.028	1.33E-01	-0.042	0.041	3.15E-01
5.162013	0.014	0.010	1.53E-01	-0.020	0.015	1.66E-01
7.714863	-0.016	0.010	1.15E-01	-0.013	0.015	3.77E-01
5.124669	0.009	0.007	2.11E-01	-0.006	0.011	6.00E-01
5.312398	0.024	0.017	1.49E-01	-0.020	0.025	4.19E-01
1.539306582	0.019	0.013	1.33E-01	-0.014	0.019	4.54E-01
2.497130169	-0.019	0.014	1.73E-01	-0.011	0.021	5.98E-01
2.38947174	-0.015	0.010	1.48E-01	-0.003	0.015	8.37E-01
7.876687	0.010	0.009	2.91E-01	0.002	0.014	9.14E-01
8.567047	0.007	0.008	3.54E-01	0.002	0.011	8.85E-01
8.758141	0.010	0.009	2.87E-01	-0.027	0.014	5.22E-02
8.092341	-0.010	0.009	2.72E-01	-0.011	0.014	4.15E-01
7.728993	-0.010	0.010	3.54E-01	-0.021	0.015	1.81E-01
3.033404	0.010	0.009	2.57E-01	0.031	0.013	1.84E-02
1.257712503	0.018	0.011	1.24E-01	-0.007	0.017	6.69E-01
2.303008564	-0.006	0.004	1.61E-01	-0.006	0.006	3.82E-01
1.824264987	-0.011	0.009	2.64E-01	-0.002	0.014	8.61E-01
5.327874	0.027	0.019	1.49E-01	-0.016	0.028	5.63E-01
8.363169	0.010	0.009	2.57E-01	-0.002	0.013	9.00E-01
1.244255199	0.011	0.008	1.62E-01	-0.008	0.011	5.09E-01

1.473029361	-0.005	0.004	2.14E-01	0.000	0.006	9.51E-01
1.256703205	0.017	0.011	1.25E-01	-0.008	0.016	6.34E-01
8.268968	0.006	0.007	3.72E-01	-0.004	0.011	7.23E-01
5.313407	0.024	0.017	1.51E-01	-0.020	0.025	4.14E-01
2.616563739	-0.018	0.013	1.75E-01	0.019	0.019	3.41E-01
1.656048691	0.006	0.004	1.90E-01	0.001	0.006	8.26E-01
5.4971	0.012	0.009	1.74E-01	0.006	0.013	6.31E-01
2.308055053	-0.009	0.006	1.58E-01	-0.003	0.010	7.58E-01
1.255693908	0.016	0.010	1.27E-01	-0.008	0.015	6.04E-01
0.979146318	0.009	0.007	1.90E-01	0.013	0.011	2.16E-01
5.108856	0.011	0.010	2.47E-01	-0.005	0.014	7.06E-01
1.241227306	0.010	0.007	1.80E-01	-0.006	0.011	5.60E-01
5.131734	0.012	0.009	1.68E-01	-0.010	0.013	4.58E-01
7.794261	-0.015	0.010	1.21E-01	0.005	0.014	7.06E-01
7.245876	-0.017	0.010	9.90E-02	-0.021	0.015	1.68E-01
1.254348177	0.015	0.010	1.30E-01	-0.009	0.014	5.50E-01
4.282241	0.018	0.011	1.12E-01	0.011	0.016	4.96E-01
2.020405187	0.015	0.010	1.13E-01	-0.001	0.014	9.71E-01
5.670362	0.010	0.008	2.29E-01	0.019	0.012	1.17E-01
3.163267	-0.010	0.007	1.32E-01	0.004	0.010	7.20E-01
1.880112797	-0.008	0.006	2.09E-01	-0.004	0.010	6.96E-01
7.039979	-0.011	0.010	2.48E-01	0.004	0.014	7.92E-01
0.806219966	0.005	0.005	3.62E-01	0.013	0.008	1.09E-01
3.727128	0.008	0.008	2.95E-01	0.022	0.012	5.58E-02
4.405712	-0.016	0.013	2.07E-01	0.010	0.019	6.13E-01
5.27068	-0.011	0.006	6.58E-02	-0.018	0.009	4.37E-02
6.658128	-0.011	0.009	2.20E-01	-0.017	0.013	1.92E-01
7.038634	-0.018	0.011	1.26E-01	0.004	0.017	8.35E-01
7.749516	-0.011	0.011	2.86E-01	-0.004	0.016	8.26E-01
1.152745535	0.009	0.006	1.05E-01	-0.005	0.009	5.57E-01
2.347417666	0.017	0.010	7.24E-02	0.012	0.014	4.00E-01
5.015328	0.010	0.008	2.26E-01	-0.008	0.013	5.48E-01
6.805149	0.009	0.008	2.94E-01	-0.009	0.012	4.61E-01
5.263615	-0.011	0.006	5.52E-02	-0.014	0.009	9.35E-02
7.696359	-0.013	0.010	1.81E-01	-0.014	0.015	3.45E-01
7.273127	-0.017	0.011	1.13E-01	0.002	0.016	8.94E-01
7.392561	0.009	0.007	1.72E-01	0.005	0.010	5.77E-01
8.015634	-0.009	0.009	2.82E-01	-0.008	0.013	5.24E-01
7.562459	0.013	0.009	1.55E-01	-0.007	0.014	6.17E-01
6.646017	-0.012	0.009	1.59E-01	-0.015	0.013	2.48E-01
7.618644	-0.015	0.008	7.20E-02	-0.013	0.012	2.79E-01
8.631978	0.007	0.008	3.73E-01	-0.001	0.012	9.18E-01
5.822766	0.011	0.009	1.98E-01	0.011	0.013	4.06E-01

0.770221678	0.007	0.005	1.61E-01	-0.001	0.008	9.48E-01
5.531416	0.012	0.009	2.01E-01	0.016	0.014	2.46E-01
7.823195	-0.018	0.010	7.04E-02	-0.004	0.015	7.61E-01
0.850629068	-0.011	0.007	1.24E-01	-0.014	0.010	1.85E-01
1.243918767	0.011	0.008	1.65E-01	-0.008	0.011	5.04E-01
6.556862	-0.010	0.009	2.31E-01	0.008	0.013	5.47E-01
3.940762	-0.010	0.008	1.96E-01	0.013	0.012	2.69E-01
3.791723	0.011	0.006	4.27E-02	0.010	0.008	2.48E-01
1.243582334	0.010	0.008	1.67E-01	-0.008	0.011	5.04E-01
6.781599	-0.011	0.010	2.47E-01	-0.002	0.015	8.65E-01
1.439386102	0.006	0.004	1.42E-01	0.004	0.006	5.27E-01
1.534260093	0.016	0.011	1.35E-01	-0.016	0.016	3.14E-01
4.373751	-0.017	0.014	2.17E-01	0.004	0.021	8.31E-01
6.049185	0.007	0.007	3.23E-01	-0.016	0.011	1.32E-01
4.406049	-0.017	0.013	2.00E-01	0.011	0.020	5.89E-01
3.980461	-0.012	0.011	2.59E-01	-0.013	0.016	4.34E-01
8.144488	0.011	0.008	1.63E-01	0.006	0.012	6.33E-01
1.069646685	0.007	0.006	2.44E-01	-0.001	0.009	9.32E-01
0.842891118	-0.013	0.008	1.07E-01	-0.014	0.012	2.48E-01
1.656385124	0.006	0.004	1.68E-01	0.004	0.006	5.23E-01
3.748996	0.011	0.009	2.04E-01	0.040	0.013	1.95E-03
4.423543	-0.020	0.015	1.93E-01	0.013	0.023	5.78E-01
2.327904575	-0.005	0.005	2.98E-01	-0.010	0.008	1.99E-01
5.922014	-0.014	0.008	9.99E-02	-0.025	0.012	4.13E-02
7.252268	0.013	0.011	2.07E-01	0.027	0.016	9.02E-02
3.071421	-0.008	0.006	1.71E-01	-0.006	0.009	4.96E-01
7.080351	0.008	0.009	3.86E-01	0.001	0.013	9.54E-01
7.308453	-0.015	0.010	1.38E-01	-0.001	0.015	9.74E-01
2.289214828	-0.005	0.005	3.00E-01	-0.010	0.007	1.47E-01
1.493551749	-0.006	0.004	1.27E-01	-0.011	0.006	9.67E-02
3.873139	-0.006	0.005	1.80E-01	0.009	0.007	1.63E-01
3.705596	0.010	0.006	1.07E-01	0.025	0.009	5.33E-03
8.505816	-0.012	0.009	1.94E-01	-0.004	0.013	7.54E-01
5.463793	0.011	0.008	1.69E-01	-0.001	0.012	9.05E-01
6.001748	0.011	0.008	1.61E-01	0.003	0.011	8.01E-01
2.258263029	0.009	0.006	1.49E-01	0.011	0.010	2.67E-01
5.09338	0.010	0.007	1.66E-01	0.002	0.011	8.36E-01
6.123201	0.010	0.007	1.88E-01	-0.001	0.011	9.63E-01
3.792059	0.011	0.006	5.35E-02	0.011	0.008	2.05E-01
7.301724	-0.017	0.010	9.00E-02	-0.020	0.015	1.86E-01
8.452996	0.026	0.016	1.08E-01	0.021	0.024	3.77E-01
5.44798	0.012	0.007	8.85E-02	0.023	0.011	3.39E-02
3.34023	-0.013	0.010	1.93E-01	0.012	0.015	4.20E-01

1.255357475	0.015	0.010	1.31E-01	-0.008	0.015	5.87E-01
6.099314	0.014	0.009	1.41E-01	0.010	0.014	4.70E-01
7.576926	0.010	0.008	2.30E-01	0.002	0.013	8.56E-01
1.065273061	0.009	0.007	1.72E-01	0.003	0.010	7.28E-01
6.622803	-0.013	0.009	1.32E-01	-0.008	0.013	5.25E-01
1.898280157	-0.008	0.006	1.71E-01	-0.006	0.009	5.14E-01
0.951222413	0.007	0.005	1.37E-01	-0.001	0.007	9.16E-01
1.846133105	-0.011	0.009	2.02E-01	-0.020	0.013	1.21E-01
6.355003	-0.013	0.009	1.56E-01	-0.005	0.014	6.91E-01
0.622191338	0.010	0.009	2.74E-01	0.009	0.013	5.15E-01
1.478748715	-0.008	0.007	2.25E-01	0.004	0.010	6.72E-01
5.4103	0.011	0.008	1.96E-01	-0.006	0.012	6.14E-01
8.870846	-0.009	0.008	2.73E-01	-0.001	0.012	9.06E-01
3.049216	-0.014	0.008	7.70E-02	-0.005	0.012	6.86E-01
7.478351	0.011	0.008	1.59E-01	0.006	0.011	5.73E-01
5.313744	0.024	0.017	1.54E-01	-0.023	0.025	3.67E-01
5.300959	0.021	0.014	1.44E-01	-0.016	0.022	4.67E-01
5.628981	0.010	0.008	2.17E-01	-0.012	0.012	3.04E-01
2.773677759	-0.015	0.011	1.44E-01	-0.049	0.016	2.09E-03
1.255021042	0.015	0.010	1.32E-01	-0.008	0.015	5.72E-01
0.859712748	0.014	0.009	1.36E-01	0.000	0.014	9.92E-01
3.536707	0.011	0.008	1.79E-01	0.020	0.012	8.45E-02
6.664184	0.016	0.010	1.01E-01	0.006	0.015	7.03E-01
4.433973	-0.019	0.016	2.31E-01	0.021	0.024	3.85E-01
1.25468461	0.015	0.010	1.33E-01	-0.009	0.015	5.59E-01
1.987771226	0.013	0.008	1.10E-01	-0.005	0.012	6.95E-01
3.070748	-0.008	0.006	1.73E-01	-0.008	0.009	3.74E-01
2.311082946	-0.019	0.013	1.50E-01	-0.001	0.020	9.44E-01
2.403601908	-0.016	0.011	1.40E-01	0.021	0.016	2.11E-01
6.262147	0.009	0.009	3.21E-01	0.006	0.013	6.75E-01
7.538909	-0.010	0.008	2.23E-01	-0.029	0.013	2.36E-02
2.757865427	-0.012	0.008	1.06E-01	-0.026	0.011	2.19E-02
6.362741	0.013	0.011	2.44E-01	0.018	0.017	2.76E-01
6.689753	-0.011	0.009	2.06E-01	-0.020	0.013	1.36E-01
8.906507	-0.009	0.009	3.05E-01	-0.017	0.013	1.72E-01
7.990402	-0.016	0.009	8.62E-02	-0.013	0.014	3.65E-01
2.14925887	-0.013	0.008	9.89E-02	0.005	0.011	6.52E-01
0.689477857	0.025	0.016	1.12E-01	0.010	0.024	6.82E-01
5.098763	0.011	0.008	1.68E-01	0.000	0.012	9.95E-01
7.113322	-0.014	0.010	1.55E-01	-0.002	0.015	9.02E-01
8.056006	0.011	0.009	2.25E-01	-0.008	0.014	5.69E-01
5.87525	0.012	0.009	1.87E-01	0.003	0.013	8.21E-01
2.384425251	-0.011	0.008	1.34E-01	0.001	0.011	9.33E-01

5.003217	0.015	0.012	1.95E-01	0.006	0.017	7.46E-01
2.514288231	-0.020	0.014	1.46E-01	-0.024	0.021	2.36E-01
1.908709567	-0.010	0.006	9.20E-02	-0.018	0.009	3.70E-02
3.70526	0.009	0.006	1.10E-01	0.025	0.009	4.43E-03
8.581514	0.007	0.007	3.24E-01	0.008	0.010	4.70E-01
0.60166895	-0.010	0.009	2.87E-01	-0.011	0.013	4.26E-01
0.711009542	0.012	0.009	1.74E-01	0.007	0.013	6.05E-01
8.402868	0.009	0.008	2.52E-01	0.017	0.012	1.62E-01
8.640726	0.012	0.009	1.82E-01	-0.026	0.013	4.06E-02
3.581116	-0.007	0.006	2.51E-01	0.006	0.009	4.85E-01
6.586805	0.013	0.011	2.25E-01	0.016	0.016	3.06E-01
7.030896	-0.012	0.009	1.86E-01	-0.016	0.014	2.41E-01
1.696757034	-0.005	0.004	2.60E-01	0.006	0.006	3.35E-01
3.440487	0.010	0.007	1.48E-01	0.020	0.011	6.03E-02
7.431923	0.010	0.009	2.76E-01	-0.004	0.014	8.01E-01
7.323256	-0.011	0.006	7.41E-02	-0.006	0.009	5.07E-01
5.888371	0.010	0.007	1.49E-01	-0.002	0.011	8.59E-01
3.107419	-0.016	0.010	1.10E-01	-0.013	0.015	3.88E-01
6.919873	-0.011	0.007	1.46E-01	0.006	0.011	6.13E-01
5.668344	0.013	0.010	1.84E-01	0.023	0.015	1.17E-01
5.814692	0.011	0.009	2.53E-01	-0.001	0.014	9.23E-01
8.798513	0.008	0.008	3.34E-01	0.002	0.012	8.86E-01
4.163481	-0.017	0.013	2.06E-01	0.006	0.020	7.49E-01
6.192506	0.009	0.008	2.50E-01	0.013	0.012	2.90E-01
1.932259848	0.007	0.005	1.45E-01	0.006	0.007	4.30E-01
4.402012	-0.016	0.012	1.97E-01	0.000	0.018	9.80E-01
1.079066797	0.011	0.008	1.37E-01	0.006	0.011	6.19E-01
8.821054	-0.012	0.009	2.13E-01	-0.022	0.014	1.11E-01
1.177305114	0.016	0.009	9.79E-02	-0.008	0.014	5.58E-01
6.10436	0.011	0.009	2.20E-01	0.013	0.013	3.06E-01
1.243245902	0.010	0.008	1.73E-01	-0.008	0.011	4.83E-01
3.220124	-0.021	0.014	1.49E-01	-0.009	0.021	6.72E-01
5.271353	-0.011	0.006	7.28E-02	-0.019	0.009	3.80E-02
3.620142	-0.008	0.006	1.95E-01	-0.013	0.009	1.46E-01
3.668588	-0.005	0.004	2.26E-01	0.005	0.006	4.46E-01
7.875678	0.012	0.009	1.99E-01	0.008	0.014	5.39E-01
8.905835	-0.007	0.006	2.51E-01	-0.015	0.010	1.15E-01
4.155406	-0.020	0.015	1.82E-01	0.001	0.022	9.81E-01
6.300837	-0.009	0.007	2.17E-01	-0.002	0.011	8.61E-01
6.088548	0.012	0.010	2.45E-01	-0.012	0.015	4.31E-01
1.14568045	0.010	0.006	9.80E-02	-0.007	0.009	4.21E-01
2.525054074	-0.014	0.010	1.35E-01	-0.003	0.014	8.53E-01
1.640909224	0.008	0.006	1.72E-01	0.003	0.009	7.40E-01

7.041998	-0.014	0.013	2.67E-01	-0.022	0.019	2.42E-01
5.700305	0.010	0.007	1.84E-01	-0.022	0.011	4.15E-02
3.571359	-0.007	0.006	2.54E-01	0.009	0.009	3.14E-01
2.588976266	-0.014	0.016	3.79E-01	0.017	0.023	4.79E-01
5.312062	0.024	0.017	1.57E-01	-0.021	0.025	4.04E-01
1.679935405	-0.006	0.004	1.64E-01	-0.003	0.006	5.70E-01
7.131489	-0.019	0.011	1.00E-01	-0.042	0.017	1.26E-02
7.285575	-0.016	0.010	9.41E-02	-0.023	0.014	1.13E-01
8.162319	0.011	0.007	1.43E-01	0.008	0.011	4.43E-01
8.635679	0.014	0.010	1.66E-01	-0.014	0.015	3.70E-01
6.313621	-0.010	0.009	2.67E-01	-0.027	0.013	3.95E-02
1.023218987	0.008	0.006	1.38E-01	0.004	0.009	6.22E-01
0.780987521	0.005	0.004	2.08E-01	0.003	0.006	6.48E-01
7.583991	0.015	0.011	1.72E-01	-0.014	0.016	3.97E-01
5.995693	0.008	0.008	3.65E-01	-0.004	0.013	7.34E-01
6.220093	0.011	0.009	2.30E-01	-0.020	0.014	1.32E-01
5.101118	0.011	0.008	1.97E-01	0.000	0.012	9.82E-01
2.633721801	-0.012	0.008	1.61E-01	-0.022	0.013	8.70E-02
1.241900171	0.010	0.007	1.86E-01	-0.008	0.011	4.69E-01
1.041049914	0.008	0.007	2.37E-01	0.018	0.011	8.09E-02
7.999822	0.012	0.009	1.72E-01	0.016	0.013	2.21E-01
7.924461	-0.007	0.008	3.76E-01	-0.015	0.012	2.06E-01
7.131153	-0.015	0.010	1.49E-01	-0.021	0.015	1.71E-01
5.327201	0.026	0.019	1.61E-01	-0.020	0.028	4.72E-01
7.757927	-0.016	0.011	1.60E-01	0.007	0.017	6.68E-01
1.424583068	0.008	0.005	8.09E-02	0.010	0.007	1.60E-01
8.693546	0.011	0.008	1.98E-01	-0.010	0.012	4.24E-01
1.177977979	0.015	0.009	1.04E-01	-0.009	0.014	5.17E-01
7.02013	0.006	0.008	4.62E-01	-0.005	0.012	6.84E-01
8.403877	0.011	0.009	1.88E-01	-0.001	0.013	9.47E-01
7.027195	-0.019	0.009	4.08E-02	-0.035	0.014	1.22E-02
8.05466	0.010	0.010	3.04E-01	0.008	0.014	6.00E-01
3.658495	-0.006	0.005	2.19E-01	0.010	0.008	2.15E-01
3.225843	-0.010	0.007	1.57E-01	0.006	0.011	5.82E-01
2.302335699	-0.006	0.004	1.97E-01	-0.002	0.006	7.53E-01
6.888921	0.013	0.008	1.19E-01	-0.003	0.012	7.85E-01
7.05781	-0.011	0.010	2.74E-01	-0.020	0.015	1.96E-01
7.707798	-0.013	0.010	2.08E-01	-0.020	0.016	1.99E-01
6.701865	-0.015	0.009	9.28E-02	-0.026	0.013	4.39E-02
5.446635	0.010	0.008	1.87E-01	0.002	0.011	8.82E-01
3.031385	-0.006	0.005	2.29E-01	0.002	0.007	8.00E-01
5.451008	0.012	0.008	1.30E-01	-0.001	0.012	9.36E-01
5.037533	0.012	0.008	1.48E-01	-0.019	0.012	1.29E-01

6.870417	0.015	0.010	1.28E-01	0.011	0.015	4.40E-01
1.242909469	0.010	0.007	1.79E-01	-0.008	0.011	4.67E-01
2.655589919	-0.012	0.009	1.70E-01	0.016	0.014	2.23E-01
0.841545388	-0.014	0.009	1.09E-01	-0.014	0.013	2.79E-01
0.911523367	-0.006	0.005	2.11E-01	-0.025	0.008	1.24E-03
4.430945	-0.018	0.015	2.14E-01	0.014	0.022	5.16E-01
3.606685	0.009	0.009	2.68E-01	-0.018	0.013	1.50E-01
1.699448495	-0.004	0.004	3.08E-01	0.005	0.006	4.00E-01
1.70785931	-0.006	0.005	1.92E-01	-0.002	0.007	8.25E-01
3.040469	-0.007	0.006	1.93E-01	0.007	0.008	4.15E-01
6.212019	0.010	0.008	1.90E-01	-0.004	0.011	7.11E-01
7.162441	0.013	0.010	2.25E-01	-0.013	0.015	4.02E-01
1.918466112	-0.007	0.005	1.37E-01	-0.007	0.007	3.25E-01
8.369897	0.009	0.007	1.69E-01	-0.004	0.010	6.75E-01
6.946115	0.010	0.008	2.51E-01	0.007	0.012	5.95E-01
2.370631515	-0.008	0.007	2.48E-01	0.007	0.010	4.99E-01
3.5902	-0.006	0.006	2.93E-01	-0.001	0.009	8.72E-01
7.75288	-0.012	0.011	2.90E-01	-0.001	0.016	9.41E-01
3.965658	-0.012	0.008	1.41E-01	0.007	0.012	5.74E-01
7.851455	-0.014	0.011	2.20E-01	0.019	0.017	2.45E-01
2.340352581	0.012	0.007	7.95E-02	0.007	0.010	4.92E-01
5.270344	-0.011	0.006	6.87E-02	-0.018	0.009	4.38E-02
1.534596526	0.016	0.011	1.46E-01	-0.016	0.016	3.32E-01
5.422748	0.012	0.008	1.52E-01	-0.004	0.012	7.55E-01
5.699968	0.011	0.009	2.08E-01	-0.043	0.013	1.16E-03
5.577843	0.014	0.009	1.28E-01	-0.017	0.013	2.12E-01
6.959908	-0.012	0.009	1.94E-01	-0.005	0.013	7.27E-01
0.814967213	0.005	0.005	3.27E-01	0.016	0.008	3.46E-02
5.826467	0.010	0.007	1.36E-01	0.015	0.010	1.24E-01
5.710398	0.011	0.007	1.48E-01	-0.005	0.011	6.67E-01
2.861823	-0.010	0.008	2.21E-01	-0.009	0.012	4.60E-01
5.815701	0.009	0.006	1.86E-01	0.002	0.010	8.39E-01
2.598059946	-0.022	0.018	2.20E-01	0.009	0.027	7.27E-01
2.02074162	0.015	0.010	1.20E-01	0.000	0.014	9.93E-01
8.446941	0.011	0.008	1.87E-01	0.012	0.012	3.14E-01
0.93137289	-0.004	0.004	3.57E-01	-0.010	0.006	1.11E-01
7.702415	-0.014	0.010	1.85E-01	0.013	0.015	3.92E-01
6.845858	0.013	0.010	2.05E-01	-0.002	0.015	9.10E-01
3.911156	0.009	0.008	2.72E-01	0.030	0.012	1.04E-02
3.793069	0.010	0.006	9.70E-02	0.016	0.009	7.29E-02
8.131367	0.011	0.007	1.34E-01	-0.003	0.011	7.88E-01
0.581819427	-0.011	0.008	1.92E-01	0.012	0.012	3.26E-01
7.725629	-0.012	0.010	2.16E-01	-0.024	0.015	1.01E-01

8.885985	0.010	0.009	2.69E-01	-0.010	0.013	4.36E-01
8.103106	0.009	0.008	2.25E-01	0.028	0.011	1.32E-02
8.624913	0.009	0.006	1.77E-01	-0.007	0.010	4.54E-01
8.643081	-0.010	0.007	1.55E-01	-0.008	0.011	4.60E-01
1.636535601	0.009	0.006	1.51E-01	-0.001	0.009	9.40E-01
1.405406411	0.008	0.005	9.60E-02	0.005	0.008	4.78E-01
2.656262784	-0.012	0.009	1.54E-01	0.021	0.013	1.10E-01
8.750066	-0.010	0.010	3.14E-01	0.000	0.015	9.82E-01
7.530498	-0.009	0.009	3.40E-01	-0.016	0.014	2.31E-01
5.396506	0.010	0.008	1.90E-01	-0.017	0.011	1.26E-01
5.256214	-0.010	0.005	5.62E-02	-0.010	0.008	2.13E-01
3.940426	-0.009	0.008	2.23E-01	0.009	0.012	4.28E-01
2.095766088	-0.009	0.008	2.81E-01	-0.006	0.012	6.18E-01
5.574816	0.011	0.009	2.21E-01	-0.017	0.013	1.92E-01
7.310471	-0.016	0.009	7.88E-02	0.006	0.014	6.57E-01
6.252054	0.018	0.010	7.87E-02	0.011	0.016	4.90E-01
3.440151	0.010	0.007	1.64E-01	0.022	0.011	4.72E-02
1.197154637	0.007	0.005	2.09E-01	0.011	0.008	1.75E-01
4.243888	0.023	0.016	1.49E-01	-0.003	0.024	9.02E-01
0.84188182	-0.014	0.009	1.11E-01	-0.014	0.013	2.68E-01
2.188621483	0.016	0.011	1.49E-01	-0.013	0.017	4.26E-01
4.445075	-0.021	0.017	2.29E-01	0.027	0.026	2.98E-01
1.84546024	-0.011	0.009	2.03E-01	0.002	0.013	8.75E-01
1.886841448	-0.011	0.007	1.10E-01	-0.009	0.010	3.86E-01
5.303987	0.021	0.015	1.46E-01	-0.018	0.022	4.06E-01
2.49982163	-0.018	0.013	1.58E-01	0.000	0.019	9.84E-01
7.284902	-0.018	0.009	4.92E-02	0.022	0.014	1.15E-01
8.690854	0.009	0.007	1.96E-01	0.009	0.011	4.20E-01
5.51123	0.008	0.008	2.94E-01	0.002	0.012	8.52E-01
5.996702	0.010	0.008	2.05E-01	0.002	0.012	8.35E-01
4.15507	-0.018	0.013	1.61E-01	0.009	0.019	6.49E-01
0.842554686	-0.013	0.008	1.12E-01	-0.014	0.013	2.58E-01
7.945992	0.012	0.009	1.89E-01	0.014	0.013	2.79E-01
2.290560558	-0.005	0.005	2.44E-01	-0.007	0.007	3.27E-01
8.1169	-0.007	0.008	4.15E-01	0.003	0.012	7.84E-01
0.841208955	-0.014	0.009	1.11E-01	-0.014	0.013	2.81E-01
3.659841	-0.007	0.005	1.66E-01	0.006	0.007	3.60E-01
8.62054	0.011	0.008	1.55E-01	-0.001	0.012	9.43E-01
6.753675	-0.013	0.011	2.42E-01	-0.016	0.017	3.33E-01
6.766123	0.011	0.008	1.63E-01	-0.015	0.012	2.25E-01
8.783373	0.011	0.008	2.01E-01	-0.005	0.012	6.81E-01
2.71412919	-0.011	0.006	6.97E-02	-0.016	0.009	7.11E-02
7.309462	-0.017	0.010	9.89E-02	-0.007	0.015	6.53E-01

1.912746758	-0.015	0.009	1.04E-01	-0.031	0.013	1.93E-02
5.155284	0.013	0.010	1.70E-01	-0.024	0.015	1.04E-01
3.155529	-0.013	0.009	1.43E-01	-0.001	0.013	9.08E-01
7.826559	-0.015	0.010	1.19E-01	0.002	0.014	9.14E-01
5.630327	0.008	0.007	2.31E-01	-0.002	0.010	8.06E-01
7.272791	-0.019	0.011	7.87E-02	-0.003	0.016	8.37E-01
7.563805	0.011	0.009	2.58E-01	0.011	0.014	4.48E-01
5.086652	0.012	0.009	1.73E-01	0.001	0.013	9.63E-01
7.911676	-0.009	0.008	2.29E-01	-0.003	0.012	8.18E-01
3.741594	0.006	0.008	4.19E-01	0.026	0.012	3.31E-02
0.842218253	-0.013	0.008	1.13E-01	-0.014	0.013	2.57E-01
5.150574	0.011	0.009	1.99E-01	-0.008	0.013	5.31E-01
8.653174	0.009	0.007	1.82E-01	-0.004	0.010	7.20E-01
7.391215	0.009	0.006	1.11E-01	-0.002	0.008	7.84E-01
5.926051	-0.013	0.010	2.10E-01	-0.018	0.015	2.25E-01
3.9448	-0.012	0.009	2.05E-01	0.000	0.014	9.74E-01
1.242236604	0.010	0.007	1.89E-01	-0.008	0.011	4.47E-01
1.476057255	-0.005	0.004	2.14E-01	-0.001	0.006	9.29E-01
6.652746	-0.008	0.008	3.35E-01	-0.008	0.013	5.29E-01
7.063193	-0.011	0.008	1.95E-01	-0.009	0.013	4.61E-01
7.82925	-0.010	0.007	1.69E-01	0.013	0.011	2.28E-01
3.755052	0.015	0.011	1.84E-01	0.020	0.016	2.18E-01
1.079739662	0.010	0.007	1.86E-01	0.007	0.011	5.38E-01
1.426265231	0.008	0.005	8.60E-02	0.009	0.007	1.63E-01
6.694463	0.010	0.009	2.24E-01	0.013	0.013	3.21E-01
6.71835	-0.010	0.008	1.95E-01	0.008	0.012	5.17E-01
6.550806	-0.013	0.008	9.83E-02	-0.034	0.012	4.14E-03
8.466117	-0.009	0.008	2.61E-01	-0.018	0.012	1.20E-01
3.626534	-0.009	0.007	2.14E-01	0.007	0.010	4.73E-01
3.764808	0.014	0.009	1.30E-01	0.024	0.014	9.45E-02
5.096072	0.011	0.009	2.24E-01	-0.003	0.013	8.03E-01
0.941465867	-0.006	0.005	2.85E-01	-0.021	0.008	6.95E-03
1.426938096	0.007	0.004	1.11E-01	0.005	0.007	4.87E-01
8.49606	-0.011	0.006	8.16E-02	-0.008	0.009	3.83E-01
3.128951	-0.013	0.009	1.53E-01	-0.013	0.014	3.63E-01
6.807168	0.008	0.008	3.30E-01	0.009	0.012	4.82E-01
8.662257	0.010	0.007	1.43E-01	0.012	0.010	2.53E-01
7.61898	-0.014	0.008	7.43E-02	-0.015	0.012	2.28E-01
0.502757769	0.012	0.009	1.97E-01	0.010	0.013	4.72E-01
7.383814	0.005	0.008	5.33E-01	0.011	0.012	3.36E-01
5.004226	0.012	0.010	2.29E-01	0.002	0.015	9.15E-01
8.383355	0.014	0.009	1.32E-01	-0.018	0.014	1.98E-01
2.665682897	-0.008	0.006	1.73E-01	-0.002	0.009	8.19E-01

5.904519	0.010	0.008	2.15E-01	-0.002	0.011	8.65E-01
4.104605	-0.011	0.011	3.07E-01	0.006	0.017	7.04E-01
3.662869	-0.007	0.005	1.49E-01	0.009	0.007	2.29E-01
3.238628	-0.009	0.006	1.16E-01	0.007	0.009	4.41E-01
8.547197	-0.013	0.009	1.45E-01	-0.029	0.013	2.66E-02
2.987649	-0.006	0.004	1.66E-01	0.006	0.006	3.23E-01
7.453119	-0.014	0.009	1.27E-01	0.008	0.013	5.50E-01
5.27169	-0.011	0.006	7.65E-02	-0.020	0.009	3.29E-02
7.851791	-0.015	0.011	1.78E-01	0.029	0.017	8.76E-02
1.145007585	0.010	0.006	9.45E-02	-0.005	0.009	5.72E-01
2.544903597	-0.042	0.027	1.25E-01	-0.038	0.041	3.46E-01
3.296158	-0.016	0.013	2.18E-01	-0.010	0.019	6.01E-01
8.058024	0.010	0.008	2.05E-01	-0.002	0.012	8.30E-01
5.126014	0.009	0.007	1.86E-01	-0.005	0.010	6.20E-01
6.846194	0.008	0.008	3.20E-01	0.018	0.012	1.41E-01
8.645099	-0.011	0.007	1.21E-01	0.011	0.011	2.98E-01
6.302856	0.010	0.008	2.06E-01	0.017	0.012	1.58E-01
1.497588941	-0.007	0.005	1.60E-01	-0.011	0.007	1.03E-01
2.608152924	-0.019	0.014	1.88E-01	0.030	0.021	1.50E-01
4.282914	0.017	0.011	1.24E-01	0.008	0.016	6.34E-01
1.111364326	0.008	0.006	2.08E-01	0.000	0.010	9.82E-01
2.145558111	-0.021	0.014	1.34E-01	-0.005	0.021	8.10E-01
1.987434793	0.012	0.008	1.21E-01	-0.005	0.012	6.46E-01
4.351547	-0.018	0.014	1.87E-01	0.006	0.021	7.81E-01
7.662716	0.014	0.010	1.77E-01	-0.002	0.015	9.00E-01
5.500127	0.013	0.011	2.26E-01	0.000	0.016	9.88E-01
1.695411304	-0.005	0.004	2.23E-01	0.003	0.006	6.38E-01
7.619316	-0.018	0.010	7.93E-02	-0.007	0.015	6.46E-01
3.160239	-0.011	0.007	1.23E-01	0.000	0.011	9.74E-01
2.618582334	-0.018	0.013	1.84E-01	0.029	0.020	1.41E-01
3.901063	0.007	0.006	2.05E-01	0.024	0.008	3.61E-03
1.475720822	-0.005	0.004	2.19E-01	0.000	0.006	9.69E-01
1.242573036	0.010	0.007	1.89E-01	-0.008	0.011	4.60E-01
5.055364	0.013	0.010	1.86E-01	0.000	0.014	9.91E-01
2.341361879	0.011	0.006	1.03E-01	0.001	0.010	9.24E-01
5.557994	0.010	0.007	1.79E-01	0.006	0.011	5.97E-01
7.782486	-0.013	0.009	1.51E-01	0.001	0.014	9.39E-01
6.039092	0.013	0.010	1.88E-01	0.012	0.014	4.08E-01
8.621213	0.008	0.009	3.66E-01	0.013	0.014	3.19E-01
2.095429655	-0.008	0.008	2.96E-01	-0.005	0.012	6.53E-01
4.396965	-0.016	0.013	2.25E-01	0.011	0.020	5.82E-01
3.607021	0.009	0.008	2.89E-01	-0.016	0.012	2.05E-01
1.220032053	0.009	0.007	2.08E-01	0.003	0.011	7.47E-01

5.072185	0.010	0.008	2.09E-01	-0.011	0.012	3.23E-01
2.155651089	-0.010	0.008	2.33E-01	-0.009	0.013	4.77E-01
2.1506046	-0.010	0.007	1.86E-01	0.000	0.011	1.00E+00
6.602617	0.010	0.008	1.87E-01	0.010	0.012	3.86E-01
7.932535	-0.010	0.010	3.06E-01	-0.016	0.014	2.75E-01
2.919353	-0.011	0.007	1.26E-01	-0.012	0.011	2.80E-01
5.401889	0.010	0.007	1.75E-01	-0.008	0.011	4.38E-01
5.49609	0.011	0.009	2.13E-01	0.006	0.013	6.20E-01
1.19109885	0.007	0.005	1.75E-01	0.001	0.008	8.85E-01
4.243552	0.023	0.016	1.49E-01	-0.003	0.023	9.09E-01
6.254409	0.012	0.010	1.95E-01	0.012	0.014	4.15E-01
1.450488378	0.006	0.004	9.37E-02	0.003	0.006	6.24E-01
1.130204551	0.012	0.007	1.06E-01	0.003	0.011	8.00E-01
4.160453	-0.019	0.014	1.80E-01	-0.007	0.022	7.45E-01
2.872253	-0.011	0.009	2.65E-01	-0.005	0.014	7.05E-01
6.565946	0.009	0.008	2.61E-01	-0.009	0.012	4.55E-01
5.529397	0.012	0.009	1.95E-01	0.005	0.014	7.13E-01
5.806954	0.012	0.009	1.92E-01	-0.019	0.014	1.59E-01
0.768203083	0.007	0.005	2.03E-01	-0.005	0.008	5.68E-01
1.427947394	0.007	0.004	1.13E-01	0.008	0.007	2.48E-01
5.839924	-0.013	0.009	1.68E-01	-0.017	0.014	2.34E-01
6.417243	0.010	0.007	1.61E-01	-0.006	0.010	5.44E-01
7.467585	0.009	0.009	2.92E-01	-0.002	0.013	8.69E-01
0.758110105	0.006	0.005	2.16E-01	-0.002	0.008	8.23E-01
8.071482	-0.011	0.009	2.21E-01	-0.013	0.014	3.47E-01
3.679354	-0.005	0.005	2.68E-01	0.008	0.007	2.41E-01
5.982235	0.011	0.008	1.92E-01	0.000	0.012	9.92E-01
8.995326	0.012	0.009	1.47E-01	-0.001	0.013	9.41E-01
5.26227	-0.010	0.006	6.10E-02	-0.014	0.008	9.13E-02
7.755572	-0.013	0.011	2.36E-01	-0.011	0.016	4.81E-01
4.127819	-0.013	0.010	2.22E-01	0.015	0.015	3.42E-01
1.697093467	-0.005	0.004	2.76E-01	0.007	0.007	2.64E-01
7.833287	-0.012	0.011	2.68E-01	0.006	0.016	7.08E-01
0.840872523	-0.014	0.009	1.14E-01	-0.014	0.013	2.82E-01
3.273617	-0.015	0.011	1.64E-01	0.014	0.016	3.81E-01
7.720246	-0.015	0.011	1.61E-01	-0.015	0.016	3.43E-01
5.248812	-0.010	0.005	5.04E-02	-0.008	0.007	2.70E-01
8.867481	0.008	0.007	2.62E-01	0.017	0.011	1.23E-01
5.429813	0.012	0.009	1.63E-01	-0.007	0.013	5.78E-01
7.527134	-0.013	0.010	1.94E-01	-0.013	0.015	3.93E-01
1.240890874	0.010	0.007	1.97E-01	-0.006	0.011	5.70E-01
4.445748	-0.019	0.016	2.33E-01	0.017	0.024	4.70E-01
7.391552	0.009	0.006	1.36E-01	0.009	0.009	3.38E-01

2.798237338	-0.018	0.013	1.84E-01	-0.071	0.020	3.20E-04
8.979177	0.008	0.010	3.77E-01	0.003	0.014	8.25E-01
0.840199657	-0.014	0.009	1.14E-01	-0.013	0.013	3.01E-01
7.752544	-0.011	0.010	2.97E-01	-0.002	0.015	8.81E-01
1.327017617	0.018	0.010	7.13E-02	0.010	0.015	5.16E-01
5.061419	0.010	0.009	2.67E-01	-0.003	0.013	8.16E-01
4.442047	-0.018	0.015	2.30E-01	0.018	0.022	4.10E-01
5.6744	0.014	0.011	1.73E-01	0.014	0.016	3.75E-01
1.537287986	0.017	0.012	1.46E-01	-0.015	0.018	3.96E-01
6.332798	0.016	0.010	1.20E-01	-0.010	0.015	5.31E-01
5.960367	0.013	0.011	2.18E-01	-0.007	0.016	6.66E-01
5.572797	0.010	0.007	1.58E-01	-0.005	0.011	6.17E-01
8.175103	-0.018	0.009	4.97E-02	0.007	0.013	6.04E-01
1.841086616	-0.013	0.010	2.00E-01	-0.003	0.015	8.40E-01
6.529948	0.011	0.008	1.42E-01	0.012	0.011	2.81E-01
7.187	-0.012	0.010	2.43E-01	-0.025	0.015	9.91E-02
3.59962	-0.009	0.007	1.52E-01	0.006	0.010	5.24E-01
0.839863225	-0.014	0.009	1.14E-01	-0.013	0.013	3.06E-01
7.252941	0.009	0.009	2.89E-01	0.000	0.013	9.93E-01
2.801601664	-0.019	0.015	2.08E-01	-0.067	0.022	2.34E-03
2.583593345	-0.008	0.011	4.63E-01	0.005	0.016	7.30E-01
6.010832	-0.010	0.010	3.21E-01	0.009	0.015	5.58E-01
1.53392366	0.016	0.011	1.51E-01	-0.017	0.016	3.03E-01
6.619775	0.010	0.009	2.89E-01	-0.002	0.014	8.66E-01
0.574754343	-0.012	0.009	1.56E-01	0.013	0.013	3.22E-01
6.888248	0.011	0.008	1.74E-01	0.003	0.012	8.19E-01
1.407088574	0.009	0.006	1.19E-01	0.012	0.008	1.53E-01
6.560899	0.010	0.008	2.05E-01	0.000	0.012	1.00E+00
7.918405	-0.012	0.011	2.72E-01	-0.041	0.016	9.81E-03
3.976424	-0.010	0.009	2.57E-01	-0.002	0.013	8.95E-01
1.062918033	0.007	0.006	2.48E-01	-0.010	0.009	2.76E-01
7.796616	-0.011	0.009	1.96E-01	-0.005	0.013	7.19E-01
5.135435	0.010	0.007	1.75E-01	-0.001	0.011	9.59E-01
4.086774	0.018	0.010	9.27E-02	0.014	0.016	3.81E-01
1.520802789	0.012	0.009	1.76E-01	-0.014	0.013	3.12E-01
3.133324	-0.013	0.009	1.60E-01	-0.015	0.014	2.67E-01
8.455688	0.011	0.009	2.37E-01	0.019	0.014	1.70E-01
5.239392	0.004	0.005	4.66E-01	0.010	0.008	2.12E-01
8.101424	-0.007	0.007	3.15E-01	-0.015	0.010	1.33E-01
0.50679496	-0.009	0.010	3.67E-01	0.023	0.015	1.28E-01
5.159321	0.014	0.010	1.71E-01	-0.030	0.015	4.83E-02
8.318423	0.011	0.009	2.27E-01	0.012	0.014	3.99E-01
5.143509	0.010	0.008	2.39E-01	-0.012	0.012	3.40E-01

3.132988	-0.013	0.009	1.35E-01	-0.012	0.013	3.55E-01
6.289398	0.008	0.006	2.26E-01	-0.004	0.009	6.88E-01
6.618093	-0.013	0.009	1.30E-01	-0.025	0.013	5.19E-02
6.506061	0.009	0.008	2.55E-01	-0.008	0.012	4.99E-01
3.726791	0.008	0.008	3.19E-01	0.020	0.012	8.56E-02
7.832615	-0.013	0.011	2.27E-01	0.006	0.016	7.24E-01
5.973825	-0.007	0.007	3.63E-01	-0.008	0.011	4.70E-01
2.655253487	-0.013	0.009	1.61E-01	0.012	0.014	3.75E-01
5.492053	0.008	0.007	2.11E-01	-0.012	0.010	2.24E-01
5.980217	0.010	0.009	2.47E-01	0.018	0.013	1.70E-01
4.38048	-0.016	0.012	1.99E-01	0.010	0.018	5.96E-01
4.170882	-0.017	0.013	2.08E-01	0.007	0.020	7.35E-01
3.626871	-0.008	0.007	2.18E-01	0.009	0.010	3.71E-01
6.300501	-0.006	0.006	3.11E-01	-0.006	0.009	5.29E-01
0.84053609	-0.014	0.009	1.16E-01	-0.014	0.013	2.93E-01
4.43229	-0.017	0.015	2.50E-01	0.012	0.023	5.81E-01
8.79683	-0.008	0.009	3.95E-01	-0.005	0.013	7.29E-01
2.801265231	-0.019	0.015	1.94E-01	-0.074	0.022	7.08E-04
7.921096	0.010	0.009	2.55E-01	-0.015	0.013	2.34E-01
6.169292	-0.014	0.009	1.10E-01	-0.005	0.013	7.04E-01
6.637606	0.010	0.009	2.47E-01	0.013	0.014	3.30E-01
6.370479	0.012	0.009	2.10E-01	0.023	0.014	1.09E-01
6.040102	0.011	0.010	2.45E-01	0.008	0.015	5.85E-01
2.554660142	-0.015	0.011	1.63E-01	-0.005	0.016	7.46E-01
2.128736482	-0.012	0.010	2.03E-01	-0.001	0.014	9.50E-01
7.070595	-0.009	0.009	3.12E-01	-0.021	0.014	1.28E-01
1.144671152	0.010	0.006	1.08E-01	-0.003	0.009	7.32E-01
5.071176	0.010	0.008	2.16E-01	0.006	0.012	6.27E-01
8.325825	-0.009	0.009	3.67E-01	-0.012	0.014	3.95E-01
5.314417	0.023	0.017	1.67E-01	-0.022	0.025	3.86E-01
7.196084	0.013	0.009	1.21E-01	-0.004	0.013	7.64E-01
6.214037	-0.013	0.010	1.89E-01	-0.031	0.015	3.21E-02
7.266735	-0.013	0.011	2.59E-01	0.000	0.017	9.91E-01
1.451497676	0.006	0.004	1.06E-01	0.001	0.006	8.38E-01
2.155314656	-0.010	0.008	2.27E-01	-0.015	0.012	2.19E-01
8.889349	0.010	0.007	1.59E-01	0.001	0.010	8.97E-01
6.618429	-0.016	0.009	7.75E-02	-0.054	0.014	7.00E-05
8.248109	-0.009	0.008	2.49E-01	-0.005	0.012	6.58E-01
6.795057	0.017	0.009	6.93E-02	-0.001	0.014	9.53E-01
6.072399	0.013	0.010	2.06E-01	-0.006	0.015	7.09E-01
4.282578	0.016	0.011	1.34E-01	0.009	0.016	5.91E-01
6.005449	0.014	0.009	1.12E-01	0.014	0.013	2.73E-01
5.769274	0.007	0.008	3.72E-01	0.004	0.013	7.34E-01

6.809187	0.008	0.009	3.35E-01	-0.024	0.013	5.80E-02
5.140481	0.010	0.007	1.84E-01	-0.001	0.011	9.62E-01
7.809401	-0.010	0.010	2.80E-01	-0.021	0.014	1.41E-01
0.946175924	-0.007	0.005	1.59E-01	-0.005	0.008	5.69E-01
7.693332	-0.013	0.010	2.10E-01	-0.010	0.015	4.89E-01
2.398218987	-0.018	0.012	1.41E-01	-0.006	0.018	7.34E-01
5.585918	0.008	0.006	1.66E-01	-0.011	0.009	2.26E-01
5.687857	0.012	0.008	1.23E-01	0.008	0.012	4.91E-01
0.613107658	-0.010	0.009	2.91E-01	-0.013	0.014	3.46E-01
2.302672131	-0.006	0.004	1.93E-01	-0.003	0.006	6.52E-01
5.921005	-0.013	0.008	1.13E-01	-0.015	0.012	2.35E-01
2.405620504	-0.016	0.012	1.66E-01	-0.006	0.018	7.24E-01
8.049277	0.011	0.008	1.73E-01	0.006	0.012	6.42E-01
1.421555175	0.008	0.005	8.41E-02	0.005	0.007	4.96E-01
5.761872	0.008	0.008	2.96E-01	-0.009	0.012	4.27E-01
6.922564	-0.010	0.007	1.76E-01	-0.014	0.011	2.01E-01
7.484743	-0.010	0.008	2.44E-01	-0.004	0.012	7.39E-01
4.434309	-0.018	0.016	2.39E-01	0.012	0.023	6.15E-01
2.509578175	-0.016	0.013	2.19E-01	-0.013	0.020	5.19E-01
7.850446	-0.014	0.012	2.30E-01	0.012	0.017	4.91E-01
1.85454392	-0.009	0.009	2.92E-01	-0.007	0.013	6.22E-01
3.297167	-0.013	0.011	2.40E-01	-0.001	0.016	9.35E-01
2.293924884	-0.005	0.005	2.82E-01	-0.010	0.007	1.68E-01
7.495846	0.006	0.006	3.16E-01	0.015	0.009	8.70E-02
5.824448	0.010	0.008	2.14E-01	0.005	0.012	6.82E-01
6.828363	-0.012	0.010	2.22E-01	0.014	0.015	3.37E-01
5.791142	0.008	0.007	2.84E-01	-0.006	0.011	6.05E-01
6.632223	0.009	0.010	3.71E-01	0.008	0.015	6.04E-01
7.390879	0.009	0.006	1.05E-01	-0.007	0.008	3.89E-01
2.714465623	-0.010	0.006	8.14E-02	-0.016	0.009	7.55E-02
0.8509655	-0.010	0.007	1.41E-01	-0.013	0.010	1.89E-01
5.74606	0.010	0.011	3.91E-01	-0.016	0.017	3.37E-01
0.7756046	0.007	0.005	1.58E-01	0.006	0.007	4.09E-01
2.166416932	0.016	0.011	1.36E-01	-0.002	0.016	8.80E-01
8.150543	0.011	0.008	1.40E-01	0.015	0.011	1.85E-01
3.122895	-0.014	0.008	1.02E-01	-0.003	0.012	8.31E-01
6.591515	-0.009	0.007	1.88E-01	-0.008	0.010	4.42E-01
1.527531441	0.013	0.009	1.72E-01	-0.013	0.014	3.48E-01
5.516949	0.012	0.009	1.83E-01	0.007	0.014	6.20E-01
8.657884	0.009	0.007	2.18E-01	-0.013	0.011	2.23E-01
1.00538806	0.011	0.008	1.75E-01	0.009	0.013	4.67E-01
7.243858	-0.010	0.009	2.33E-01	-0.010	0.013	4.60E-01
4.168191	-0.016	0.013	2.26E-01	0.005	0.020	8.18E-01

8.318087	0.011	0.008	1.90E-01	0.026	0.012	3.72E-02
2.644487644	-0.013	0.009	1.47E-01	0.017	0.013	2.01E-01
3.281691	-0.011	0.008	1.71E-01	-0.025	0.012	3.97E-02
3.583807	-0.001	0.006	8.61E-01	0.005	0.010	5.95E-01
1.486150232	-0.005	0.004	2.80E-01	-0.002	0.007	7.84E-01
2.064477857	0.006	0.004	9.65E-02	0.010	0.005	6.30E-02
4.328333	-0.019	0.016	2.50E-01	0.021	0.024	3.81E-01
5.161676	0.015	0.011	1.49E-01	-0.016	0.016	3.21E-01
1.918802545	-0.007	0.005	1.49E-01	-0.009	0.007	2.08E-01
2.717493516	-0.011	0.006	8.86E-02	-0.018	0.010	6.82E-02
5.435869	0.010	0.008	1.98E-01	-0.011	0.011	3.38E-01
0.805883533	0.005	0.005	4.09E-01	0.013	0.008	1.19E-01
3.107755	-0.016	0.010	1.12E-01	-0.015	0.015	3.18E-01
7.872987	0.011	0.008	1.89E-01	0.001	0.012	9.46E-01
0.537746758	0.012	0.010	2.10E-01	0.029	0.014	4.47E-02
5.907884	0.011	0.009	2.29E-01	-0.016	0.014	2.64E-01
3.497008	0.010	0.010	3.22E-01	0.034	0.014	1.72E-02
1.49489748	-0.006	0.004	1.52E-01	-0.010	0.006	1.05E-01
7.360936	-0.015	0.010	1.11E-01	0.007	0.014	6.44E-01
5.31408	0.023	0.017	1.70E-01	-0.023	0.025	3.64E-01
3.988536	-0.010	0.007	1.67E-01	0.007	0.010	5.02E-01
0.530681674	0.013	0.009	1.51E-01	0.005	0.013	7.28E-01
3.212386	-0.023	0.016	1.33E-01	-0.012	0.023	6.18E-01
2.638431857	-0.013	0.009	1.49E-01	-0.004	0.013	7.86E-01
4.436328	-0.018	0.015	2.35E-01	0.023	0.022	3.06E-01
6.197552	0.010	0.008	2.33E-01	0.009	0.012	4.62E-01
8.612465	0.011	0.009	2.26E-01	0.017	0.013	1.86E-01
1.165866406	0.009	0.005	1.00E-01	-0.001	0.008	8.82E-01
7.102219	-0.015	0.010	1.41E-01	-0.013	0.015	4.00E-01
8.232969	0.008	0.007	2.22E-01	0.014	0.010	1.54E-01
7.500892	0.014	0.010	1.62E-01	0.026	0.014	7.29E-02
8.647791	0.012	0.011	2.63E-01	-0.008	0.016	6.35E-01
8.699601	0.008	0.007	2.17E-01	-0.004	0.010	6.58E-01
5.874241	0.012	0.009	2.09E-01	-0.006	0.014	6.86E-01
1.734773917	-0.007	0.005	1.88E-01	-0.006	0.008	4.10E-01
7.580627	0.013	0.010	2.26E-01	-0.008	0.015	6.20E-01
5.272026	-0.011	0.006	8.04E-02	-0.020	0.009	3.26E-02
3.219787	-0.020	0.015	1.68E-01	-0.009	0.022	6.64E-01
1.536278689	0.016	0.011	1.51E-01	-0.017	0.017	3.17E-01
0.546494005	0.015	0.009	1.11E-01	0.022	0.014	1.14E-01
1.763370688	-0.012	0.009	1.84E-01	0.005	0.014	7.35E-01
1.817872767	-0.010	0.008	2.11E-01	-0.008	0.012	4.71E-01
6.223121	0.010	0.007	2.00E-01	-0.002	0.011	8.83E-01

6.508416	0.014	0.009	1.18E-01	0.022	0.013	9.76E-02
3.238964	-0.009	0.006	1.23E-01	0.007	0.008	3.89E-01
1.793649621	-0.012	0.011	2.65E-01	0.003	0.016	8.41E-01
7.896873	-0.010	0.009	2.95E-01	0.005	0.014	7.04E-01
8.492695	0.007	0.009	4.49E-01	-0.008	0.013	5.20E-01
2.590321997	-0.016	0.016	3.04E-01	0.006	0.024	8.10E-01
8.804905	0.011	0.008	1.91E-01	-0.019	0.013	1.25E-01
5.54992	0.009	0.008	2.40E-01	-0.001	0.011	9.32E-01
1.925194764	0.004	0.004	2.64E-01	0.006	0.006	3.20E-01
1.483122339	-0.006	0.005	2.24E-01	0.003	0.008	6.72E-01
8.770925	0.008	0.006	2.27E-01	0.000	0.010	9.60E-01
7.210551	-0.013	0.010	2.11E-01	-0.016	0.015	2.78E-01
0.839526792	-0.014	0.009	1.19E-01	-0.013	0.013	3.14E-01
1.646628578	0.008	0.005	1.35E-01	0.008	0.008	3.08E-01
8.208073	0.006	0.006	3.10E-01	0.004	0.009	6.39E-01
4.426571	-0.017	0.015	2.30E-01	0.011	0.022	6.07E-01
3.661523	-0.007	0.005	1.33E-01	0.006	0.007	3.74E-01
3.94009	-0.009	0.008	2.40E-01	0.006	0.011	5.78E-01
5.866166	0.008	0.007	2.14E-01	-0.006	0.010	5.48E-01
3.873476	-0.006	0.005	2.05E-01	0.010	0.007	1.38E-01
2.021078052	0.014	0.010	1.32E-01	0.000	0.014	9.83E-01
7.569524	-0.012	0.009	2.16E-01	-0.016	0.014	2.63E-01
5.082278	0.013	0.011	2.22E-01	-0.007	0.016	6.63E-01
8.692873	-0.009	0.009	2.86E-01	-0.026	0.013	4.83E-02
1.784229508	-0.009	0.009	3.37E-01	-0.006	0.014	6.60E-01
6.636933	0.007	0.008	3.58E-01	0.021	0.011	6.38E-02
0.652133839	-0.015	0.010	1.23E-01	-0.017	0.015	2.63E-01
5.311725	0.022	0.017	1.75E-01	-0.021	0.025	3.95E-01
2.594022755	-0.023	0.019	2.23E-01	-0.002	0.028	9.31E-01
5.004562	0.012	0.010	2.41E-01	-0.001	0.015	9.23E-01
0.626228529	0.013	0.009	1.59E-01	0.002	0.014	8.82E-01
2.79857377	-0.018	0.013	1.89E-01	-0.066	0.020	9.49E-04
0.537410325	0.010	0.009	2.59E-01	0.030	0.013	2.38E-02
1.424919501	0.007	0.005	1.10E-01	0.006	0.007	3.76E-01
2.859468	-0.011	0.008	1.60E-01	-0.017	0.012	1.55E-01
1.536951554	0.017	0.012	1.52E-01	-0.016	0.017	3.54E-01
1.527867874	0.012	0.010	1.92E-01	-0.014	0.014	3.12E-01
2.560715929	-0.013	0.009	1.42E-01	-0.007	0.013	6.10E-01
4.314539	-0.019	0.014	1.82E-01	0.009	0.021	6.59E-01
3.133661	-0.013	0.010	1.86E-01	-0.013	0.014	3.76E-01
2.775023489	-0.016	0.011	1.54E-01	-0.051	0.016	1.88E-03
4.138248	-0.020	0.014	1.61E-01	0.008	0.022	6.96E-01
1.708532175	-0.006	0.005	1.79E-01	0.001	0.007	8.76E-01

8.051969	0.009	0.008	2.18E-01	0.011	0.011	3.51E-01
5.300623	0.020	0.014	1.63E-01	-0.016	0.021	4.42E-01
4.14094	-0.023	0.017	1.92E-01	0.020	0.026	4.46E-01
2.38038806	-0.009	0.007	1.82E-01	0.004	0.010	6.91E-01
6.527256	0.007	0.007	3.08E-01	0.000	0.010	9.86E-01
1.056525814	0.007	0.006	2.28E-01	0.008	0.009	3.66E-01
4.436664	-0.018	0.015	2.44E-01	0.025	0.023	2.65E-01
2.376014436	-0.023	0.017	1.71E-01	-0.003	0.025	9.13E-01
1.696420602	-0.004	0.004	3.22E-01	0.006	0.006	3.78E-01
4.316221	-0.018	0.014	2.00E-01	0.016	0.020	4.46E-01
5.248476	-0.010	0.005	5.15E-02	-0.009	0.007	2.25E-01
4.117053	-0.012	0.010	2.41E-01	0.016	0.015	3.00E-01
1.699784928	-0.004	0.004	3.08E-01	0.007	0.006	2.65E-01
8.225904	0.008	0.007	2.61E-01	0.003	0.011	7.85E-01
1.029274774	0.008	0.007	2.41E-01	0.018	0.010	8.54E-02
3.966668	-0.012	0.008	1.48E-01	0.007	0.012	5.99E-01
1.987098361	0.012	0.008	1.33E-01	-0.005	0.012	6.40E-01
2.557688035	0.006	0.009	4.99E-01	-0.003	0.013	7.96E-01
1.795668216	-0.010	0.010	3.25E-01	0.022	0.015	1.41E-01
0.838853927	-0.014	0.009	1.19E-01	-0.013	0.013	3.33E-01
2.409321263	-0.017	0.012	1.64E-01	0.011	0.018	5.38E-01
5.66969	0.009	0.008	2.37E-01	0.013	0.012	2.48E-01
2.798910203	-0.018	0.014	1.97E-01	-0.068	0.020	9.15E-04
6.713303	-0.011	0.010	2.85E-01	0.011	0.015	4.55E-01
6.993215	0.010	0.007	1.73E-01	0.006	0.011	5.57E-01
0.751381453	-0.006	0.005	2.36E-01	-0.011	0.008	1.63E-01
6.865707	-0.006	0.008	3.97E-01	0.006	0.011	6.07E-01
2.258599462	0.008	0.006	1.67E-01	0.010	0.009	2.59E-01
3.22618	-0.010	0.007	1.67E-01	0.007	0.010	5.02E-01
5.927733	0.013	0.010	1.84E-01	-0.008	0.015	5.94E-01
7.329311	-0.009	0.006	1.53E-01	-0.001	0.009	8.94E-01
8.040866	-0.011	0.008	1.69E-01	-0.023	0.012	4.51E-02
0.83919036	-0.014	0.009	1.20E-01	-0.013	0.013	3.24E-01
8.043894	0.008	0.007	2.56E-01	0.010	0.010	3.13E-01
6.741227	-0.015	0.011	1.82E-01	-0.010	0.017	5.58E-01
5.924033	-0.012	0.010	2.35E-01	-0.006	0.015	6.89E-01
5.304324	0.021	0.015	1.59E-01	-0.020	0.022	3.69E-01
4.368032	-0.018	0.016	2.70E-01	0.006	0.024	7.96E-01
1.929568388	0.006	0.004	1.83E-01	0.007	0.007	3.17E-01
5.129379	0.011	0.008	1.74E-01	-0.004	0.012	7.12E-01
6.241961	0.011	0.009	2.12E-01	0.001	0.013	9.36E-01
2.56744458	-0.011	0.008	1.64E-01	-0.014	0.012	2.56E-01
7.307107	-0.015	0.011	1.65E-01	-0.010	0.016	5.34E-01

6.718013	-0.015	0.010	1.26E-01	0.007	0.015	6.24E-01
4.420515	-0.017	0.014	2.25E-01	0.015	0.021	4.93E-01
1.168894299	0.008	0.005	7.37E-02	-0.002	0.007	8.16E-01
3.156538	-0.011	0.008	1.95E-01	-0.005	0.012	7.05E-01
5.589282	-0.015	0.010	1.49E-01	0.004	0.015	7.80E-01
6.843839	0.008	0.008	3.36E-01	0.006	0.013	6.33E-01
3.872803	-0.006	0.005	1.98E-01	0.009	0.007	1.95E-01
0.838181062	-0.014	0.009	1.19E-01	-0.012	0.013	3.58E-01
4.443393	-0.018	0.015	2.36E-01	0.018	0.023	4.37E-01
1.103962809	0.011	0.008	1.47E-01	-0.002	0.011	8.84E-01
5.154275	0.012	0.009	1.74E-01	-0.016	0.013	2.20E-01
5.409964	0.009	0.007	2.02E-01	-0.002	0.011	8.73E-01
5.314753	0.023	0.017	1.74E-01	-0.022	0.025	3.72E-01
6.238933	0.013	0.010	1.65E-01	0.004	0.014	7.88E-01
7.169506	-0.014	0.010	1.28E-01	-0.015	0.014	2.90E-01
3.725446	0.007	0.008	3.97E-01	0.010	0.012	3.93E-01
8.641735	0.008	0.007	2.81E-01	-0.021	0.011	4.27E-02
8.440885	-0.013	0.009	1.76E-01	-0.015	0.014	2.94E-01
1.152409102	0.009	0.006	1.29E-01	-0.007	0.009	4.41E-01
0.815303646	0.005	0.005	3.58E-01	0.016	0.008	3.51E-02
6.649718	0.010	0.010	2.97E-01	-0.013	0.015	3.57E-01
1.087814045	0.010	0.007	1.90E-01	0.012	0.011	2.67E-01
5.257896	-0.010	0.005	5.91E-02	-0.010	0.008	1.78E-01
7.739423	-0.010	0.011	3.48E-01	-0.020	0.016	2.26E-01
6.333135	0.011	0.009	2.08E-01	0.007	0.014	6.08E-01
8.097724	0.009	0.009	2.93E-01	0.002	0.013	8.93E-01
2.505877416	-0.020	0.014	1.47E-01	-0.019	0.020	3.47E-01
4.379134	-0.018	0.014	2.16E-01	0.005	0.021	8.23E-01
7.02484	0.007	0.009	4.30E-01	-0.008	0.014	5.85E-01
1.161829215	0.008	0.005	8.08E-02	-0.006	0.007	3.61E-01
1.408434304	0.009	0.006	1.06E-01	0.014	0.009	9.72E-02
6.680333	0.008	0.007	2.15E-01	-0.001	0.010	9.09E-01
4.129838	-0.018	0.014	2.14E-01	0.012	0.021	5.74E-01
8.330535	-0.007	0.007	3.42E-01	-0.008	0.011	4.87E-01
6.729116	-0.017	0.012	1.39E-01	-0.023	0.017	1.82E-01
0.838517494	-0.014	0.009	1.20E-01	-0.012	0.013	3.42E-01
4.414796	-0.016	0.014	2.27E-01	0.007	0.020	7.33E-01
1.840750184	-0.012	0.010	2.36E-01	0.000	0.015	9.84E-01
4.430608	-0.017	0.014	2.29E-01	0.007	0.021	7.32E-01
5.916967	0.010	0.008	2.16E-01	-0.007	0.012	5.82E-01
1.859253976	-0.010	0.009	2.46E-01	0.013	0.013	3.26E-01
7.490799	-0.012	0.010	2.25E-01	0.009	0.015	5.38E-01
7.111976	-0.008	0.007	2.66E-01	-0.017	0.010	9.86E-02

8.221194	0.012	0.008	1.53E-01	0.001	0.012	9.12E-01
5.015665	0.008	0.008	2.98E-01	-0.002	0.012	8.64E-01
8.760832	0.012	0.008	1.59E-01	-0.004	0.013	7.53E-01
1.634853438	0.010	0.007	1.27E-01	0.000	0.010	9.66E-01
5.468839	-0.012	0.008	1.23E-01	0.009	0.012	4.69E-01
5.02643	0.011	0.009	2.49E-01	-0.013	0.014	3.24E-01
1.678253242	-0.004	0.004	2.67E-01	0.003	0.006	5.56E-01
4.158434	-0.019	0.016	2.37E-01	0.006	0.024	8.15E-01
0.75878297	0.007	0.005	2.03E-01	-0.001	0.008	8.53E-01
7.366655	-0.010	0.008	2.50E-01	0.006	0.013	6.60E-01
3.247375	0.009	0.007	2.32E-01	0.026	0.011	1.70E-02
1.091851236	0.010	0.007	1.76E-01	0.003	0.011	7.44E-01
5.940518	0.008	0.007	2.27E-01	0.008	0.010	3.97E-01
2.380051627	-0.009	0.007	1.68E-01	0.005	0.010	6.00E-01
0.751717886	-0.007	0.005	1.78E-01	-0.017	0.008	3.09E-02
1.531905065	0.014	0.010	1.70E-01	-0.021	0.015	1.76E-01
4.368368	-0.019	0.017	2.57E-01	0.003	0.025	9.09E-01
6.599253	-0.015	0.010	1.43E-01	-0.028	0.015	6.42E-02
3.124913	-0.014	0.009	1.00E-01	0.001	0.013	9.61E-01
7.282211	-0.013	0.010	1.67E-01	0.012	0.014	4.07E-01
7.561786	0.011	0.009	2.24E-01	-0.006	0.013	6.40E-01
2.340016149	0.014	0.007	6.35E-02	0.011	0.011	3.11E-01
5.050317	0.008	0.006	1.92E-01	0.006	0.010	5.51E-01
7.70948	-0.012	0.010	2.50E-01	-0.027	0.016	8.47E-02
7.018111	0.005	0.009	6.06E-01	-0.003	0.014	8.46E-01
0.61849058	0.014	0.009	1.28E-01	0.028	0.014	4.62E-02
8.585887	0.012	0.010	2.44E-01	0.010	0.016	5.18E-01
6.882865	0.017	0.010	7.44E-02	0.025	0.014	7.96E-02
6.715322	-0.012	0.009	1.81E-01	-0.038	0.014	6.86E-03
8.70162	0.009	0.007	1.70E-01	0.000	0.010	9.64E-01
1.43131172	0.007	0.004	1.17E-01	0.005	0.006	4.76E-01
0.837844629	-0.014	0.009	1.21E-01	-0.012	0.013	3.72E-01
8.528021	0.012	0.009	2.01E-01	0.000	0.013	9.74E-01
2.867542	-0.009	0.008	2.92E-01	-0.004	0.012	7.33E-01
7.742451	-0.011	0.010	2.80E-01	-0.021	0.015	1.73E-01
5.622925	0.008	0.009	3.51E-01	-0.005	0.013	7.28E-01
7.122405	0.012	0.009	1.92E-01	-0.003	0.014	8.19E-01
4.378461	-0.015	0.012	2.08E-01	0.012	0.018	4.98E-01
3.071757	-0.008	0.006	1.92E-01	-0.006	0.009	4.95E-01
4.242542	0.020	0.014	1.72E-01	-0.006	0.021	7.71E-01
1.138951798	0.011	0.007	1.14E-01	-0.006	0.010	5.69E-01
1.646965011	0.008	0.005	1.38E-01	0.012	0.008	1.31E-01
1.010098116	0.013	0.009	1.46E-01	0.023	0.014	9.16E-02

2.043282603	0.009	0.006	1.31E-01	0.005	0.008	5.39E-01
6.769824	-0.006	0.008	4.30E-01	-0.005	0.012	6.64E-01
1.220368485	0.009	0.007	2.24E-01	0.003	0.011	8.08E-01
0.735232689	-0.009	0.007	1.93E-01	-0.004	0.010	7.09E-01
3.792396	0.010	0.006	7.88E-02	0.012	0.009	1.66E-01
2.42580646	-0.011	0.009	2.19E-01	0.013	0.013	3.30E-01
0.837508197	-0.013	0.009	1.21E-01	-0.011	0.013	3.84E-01
6.1666	0.008	0.006	2.24E-01	-0.004	0.010	6.76E-01
4.415132	-0.015	0.013	2.34E-01	0.007	0.019	7.12E-01
1.533250795	0.015	0.011	1.70E-01	-0.017	0.016	2.70E-01
3.790041	0.011	0.006	9.16E-02	0.016	0.009	9.29E-02
7.34075	0.013	0.010	1.77E-01	0.020	0.014	1.59E-01
8.270986	0.008	0.010	4.07E-01	0.022	0.014	1.23E-01
4.159444	-0.015	0.012	2.09E-01	0.005	0.017	7.82E-01
1.007070223	0.009	0.007	2.12E-01	0.015	0.011	1.83E-01
4.280559	0.019	0.012	1.24E-01	0.020	0.019	2.72E-01
0.570044287	-0.007	0.007	2.83E-01	-0.003	0.010	7.32E-01
4.154734	-0.016	0.012	1.81E-01	0.013	0.017	4.59E-01
4.244225	0.023	0.017	1.62E-01	0.001	0.025	9.79E-01
6.417579	0.010	0.008	2.29E-01	-0.005	0.012	6.54E-01
4.382835	-0.015	0.012	2.04E-01	0.005	0.017	7.95E-01
8.937796	-0.010	0.008	2.28E-01	0.003	0.012	7.80E-01
2.297625642	-0.010	0.007	1.83E-01	-0.008	0.011	4.45E-01
7.725966	-0.011	0.010	2.68E-01	-0.015	0.014	2.96E-01
1.964220944	0.006	0.005	1.86E-01	0.001	0.007	9.40E-01
2.602097137	-0.019	0.015	2.14E-01	0.014	0.023	5.38E-01
5.136444	0.008	0.007	2.79E-01	-0.004	0.011	7.30E-01
1.094206264	0.009	0.007	2.43E-01	-0.001	0.011	9.35E-01
0.751045021	-0.006	0.005	2.38E-01	-0.014	0.008	7.70E-02
3.312979	-0.019	0.012	1.29E-01	0.011	0.018	5.47E-01
1.87405701	-0.010	0.008	2.00E-01	0.010	0.011	3.59E-01
2.526063372	-0.017	0.011	1.32E-01	0.002	0.017	8.92E-01
1.78052875	-0.012	0.011	2.70E-01	0.008	0.016	6.32E-01
7.829587	-0.011	0.008	1.71E-01	0.013	0.011	2.66E-01
1.080412528	0.009	0.007	2.06E-01	-0.001	0.011	9.09E-01
5.715781	0.012	0.010	2.22E-01	0.014	0.015	3.58E-01
3.955902	-0.009	0.008	2.77E-01	-0.010	0.013	4.24E-01
2.98664	-0.005	0.004	1.91E-01	0.003	0.006	6.43E-01
7.665744	0.013	0.010	1.97E-01	-0.004	0.015	7.81E-01
3.874149	-0.006	0.005	2.17E-01	0.010	0.007	1.33E-01
4.334052	-0.017	0.014	2.19E-01	0.020	0.020	3.32E-01
4.334725	-0.016	0.013	2.47E-01	0.014	0.020	4.80E-01
7.220644	-0.016	0.009	7.82E-02	-0.015	0.014	2.87E-01

8.800868	0.010	0.008	1.97E-01	-0.006	0.012	6.12E-01
0.759455836	0.006	0.005	2.04E-01	-0.005	0.008	5.35E-01
1.157792023	0.008	0.005	1.13E-01	-0.003	0.007	6.56E-01
6.819616	0.008	0.008	3.04E-01	0.006	0.012	6.10E-01
2.327568143	-0.006	0.005	2.92E-01	-0.011	0.008	1.75E-01
3.79105	0.011	0.005	4.82E-02	0.014	0.008	8.21E-02
2.424797162	-0.009	0.008	3.01E-01	0.025	0.013	4.42E-02
7.060165	-0.009	0.009	3.24E-01	-0.001	0.014	9.52E-01
6.432046	0.012	0.009	1.82E-01	0.022	0.013	9.14E-02
6.655773	-0.009	0.008	2.58E-01	0.005	0.012	6.63E-01
6.971684	-0.014	0.009	1.44E-01	-0.005	0.014	7.17E-01
8.648464	0.008	0.008	3.24E-01	0.008	0.012	4.98E-01
6.072736	0.010	0.010	2.96E-01	-0.004	0.015	7.89E-01
1.240554441	0.009	0.007	2.14E-01	-0.006	0.011	5.89E-01
1.023891852	0.007	0.005	2.05E-01	0.014	0.008	7.26E-02
8.442903	0.013	0.010	1.99E-01	0.004	0.015	7.79E-01
2.995387	-0.005	0.004	2.12E-01	0.003	0.006	6.29E-01
2.310746513	-0.021	0.015	1.75E-01	-0.001	0.023	9.58E-01
3.571696	-0.006	0.006	2.93E-01	0.009	0.008	2.76E-01
6.397057	0.011	0.010	2.86E-01	-0.006	0.015	6.83E-01
1.840413751	-0.011	0.010	2.66E-01	-0.001	0.015	9.38E-01
8.296555	0.012	0.009	1.61E-01	-0.015	0.013	2.41E-01
3.6807	-0.005	0.005	3.06E-01	0.011	0.007	1.05E-01
8.598335	-0.009	0.008	2.49E-01	-0.015	0.012	2.17E-01
1.905345241	-0.008	0.006	1.94E-01	-0.002	0.009	8.28E-01
5.033159	0.013	0.011	2.23E-01	0.002	0.016	9.09E-01
0.772913139	0.007	0.005	1.64E-01	0.002	0.007	7.83E-01
8.436175	-0.009	0.009	3.33E-01	-0.020	0.014	1.52E-01
7.848427	0.006	0.008	4.36E-01	0.004	0.012	7.13E-01
8.814661	-0.011	0.008	1.69E-01	-0.005	0.012	6.67E-01
6.510098	0.010	0.009	2.73E-01	-0.001	0.013	9.47E-01
2.021750918	0.014	0.010	1.43E-01	0.001	0.014	9.68E-01
0.577782236	-0.010	0.009	2.68E-01	0.007	0.014	6.27E-01
2.618245902	-0.017	0.013	2.09E-01	0.036	0.020	6.66E-02
7.975598	0.009	0.008	3.09E-01	0.013	0.012	2.85E-01
3.590873	-0.006	0.006	3.20E-01	0.001	0.009	9.44E-01
6.777562	0.014	0.009	1.39E-01	0.014	0.014	3.09E-01
3.199601	0.006	0.005	2.40E-01	0.018	0.008	2.47E-02
2.66601933	-0.008	0.006	1.61E-01	0.000	0.009	9.90E-01
5.993338	0.010	0.008	2.43E-01	-0.015	0.013	2.42E-01
7.969543	0.011	0.009	2.43E-01	-0.011	0.013	4.03E-01
5.425103	0.011	0.008	1.71E-01	-0.003	0.012	8.23E-01
5.85069	0.010	0.008	2.44E-01	-0.005	0.012	6.99E-01

5.10852	0.010	0.009	2.73E-01	-0.016	0.013	2.32E-01
2.145221678	-0.020	0.014	1.59E-01	0.000	0.021	9.98E-01
1.098243455	0.011	0.008	1.49E-01	0.005	0.012	6.81E-01
6.798421	0.013	0.010	1.70E-01	0.021	0.014	1.49E-01
8.418007	-0.010	0.010	3.25E-01	-0.001	0.014	9.21E-01
4.437673	-0.019	0.015	2.21E-01	0.021	0.023	3.71E-01
2.757192562	-0.011	0.007	1.22E-01	-0.024	0.011	2.79E-02
6.969329	-0.011	0.008	1.86E-01	-0.001	0.012	9.45E-01
1.495233912	-0.006	0.004	1.52E-01	-0.009	0.007	1.67E-01
3.131642	-0.014	0.009	1.05E-01	-0.025	0.013	5.19E-02
5.749088	0.011	0.010	2.75E-01	0.010	0.015	5.09E-01
7.646567	-0.009	0.010	3.42E-01	-0.008	0.015	5.85E-01
5.645803	0.008	0.008	3.07E-01	-0.016	0.012	1.71E-01
8.489331	-0.011	0.010	2.65E-01	-0.019	0.014	1.87E-01
3.04417	-0.004	0.005	4.11E-01	-0.003	0.008	7.04E-01
2.370967947	-0.008	0.007	2.52E-01	0.008	0.010	4.53E-01
5.583226	0.011	0.008	1.64E-01	0.009	0.012	4.49E-01
7.062857	0.009	0.009	3.11E-01	0.004	0.013	7.66E-01
1.914765354	-0.009	0.006	1.25E-01	-0.001	0.008	9.17E-01
5.30466	0.020	0.015	1.66E-01	-0.020	0.022	3.57E-01
4.431281	-0.018	0.015	2.33E-01	0.021	0.023	3.70E-01
7.986028	0.010	0.008	1.77E-01	0.008	0.011	4.54E-01
7.519396	-0.012	0.010	2.26E-01	-0.009	0.015	5.53E-01
3.153174	-0.013	0.010	1.94E-01	-0.007	0.015	6.24E-01
1.534932958	0.015	0.011	1.69E-01	-0.016	0.017	3.27E-01
8.666967	0.009	0.007	1.74E-01	0.011	0.010	2.62E-01
8.32347	-0.008	0.006	2.27E-01	-0.012	0.010	2.17E-01
5.523341	0.008	0.007	2.59E-01	-0.019	0.011	9.09E-02
2.179874235	0.016	0.011	1.51E-01	-0.009	0.017	5.97E-01
4.423207	-0.016	0.013	2.22E-01	0.013	0.020	5.13E-01
5.165041	0.013	0.010	1.63E-01	-0.014	0.014	3.12E-01
1.72602667	-0.006	0.005	1.84E-01	0.007	0.007	3.24E-01
1.497252508	-0.006	0.005	1.61E-01	-0.014	0.007	4.78E-02
2.190976511	0.016	0.011	1.60E-01	-0.015	0.017	3.75E-01
0.950549547	0.006	0.005	1.76E-01	0.000	0.007	9.92E-01
7.658679	0.009	0.007	1.85E-01	0.002	0.010	8.82E-01
8.324816	-0.013	0.009	1.73E-01	-0.032	0.014	2.15E-02
6.72306	-0.014	0.012	2.14E-01	-0.014	0.017	4.12E-01
3.159566	-0.011	0.007	1.41E-01	-0.007	0.011	5.16E-01
5.280437	-0.011	0.007	1.17E-01	-0.025	0.011	1.86E-02
6.690426	-0.006	0.006	3.53E-01	-0.012	0.010	2.26E-01
5.102464	0.010	0.009	2.53E-01	0.014	0.013	2.90E-01
6.650054	0.009	0.009	3.26E-01	-0.010	0.014	4.60E-01

7.77744	-0.012	0.010	2.46E-01	-0.013	0.015	3.96E-01
2.797900905	-0.017	0.013	2.03E-01	-0.072	0.019	2.21E-04
2.605125031	-0.018	0.013	1.75E-01	0.015	0.020	4.45E-01
5.948256	0.010	0.008	1.85E-01	0.013	0.012	2.60E-01
3.334511	0.013	0.009	1.59E-01	0.016	0.014	2.50E-01
8.149871	0.009	0.008	2.87E-01	0.020	0.013	1.06E-01
8.10277	0.008	0.007	2.65E-01	0.015	0.011	1.66E-01
7.116013	-0.014	0.010	1.90E-01	-0.006	0.015	6.97E-01
3.048543	-0.013	0.007	9.03E-02	-0.008	0.011	4.97E-01
1.521139222	0.012	0.009	1.94E-01	-0.015	0.013	2.73E-01
8.607419	0.007	0.009	4.54E-01	-0.011	0.014	4.40E-01
6.73719	-0.015	0.010	1.46E-01	-0.017	0.015	2.81E-01
5.402562	0.010	0.008	2.45E-01	-0.001	0.012	9.42E-01
5.158312	0.012	0.009	2.06E-01	-0.012	0.014	3.72E-01
4.307138	-0.016	0.012	1.82E-01	0.010	0.018	5.80E-01
4.171219	-0.016	0.013	2.48E-01	-0.002	0.020	9.39E-01
4.327996	-0.017	0.014	2.38E-01	0.026	0.021	2.30E-01
7.454801	-0.009	0.008	2.57E-01	-0.015	0.011	1.80E-01
2.801938096	-0.018	0.015	2.25E-01	-0.068	0.022	2.22E-03
5.090689	0.013	0.010	1.81E-01	-0.001	0.014	9.25E-01
6.728443	-0.013	0.009	1.30E-01	-0.010	0.013	4.10E-01
1.42996599	0.006	0.004	1.67E-01	0.001	0.007	9.19E-01
3.198592	0.006	0.005	2.49E-01	0.018	0.008	2.82E-02
7.915377	0.015	0.009	1.02E-01	-0.028	0.014	5.19E-02
7.799981	-0.012	0.009	1.77E-01	0.008	0.013	5.48E-01
5.904856	0.009	0.008	2.68E-01	-0.005	0.012	6.94E-01
2.510587472	-0.014	0.014	3.28E-01	-0.001	0.021	9.62E-01
5.517286	0.015	0.010	1.21E-01	-0.012	0.015	4.32E-01
2.684859555	-0.010	0.006	9.38E-02	-0.002	0.009	7.84E-01
2.323194519	-0.008	0.006	2.10E-01	-0.008	0.009	3.81E-01
3.721408	0.009	0.008	2.39E-01	0.018	0.011	9.99E-02
8.465781	-0.008	0.007	2.26E-01	-0.016	0.010	1.10E-01
1.158801321	0.007	0.005	1.18E-01	-0.003	0.007	6.22E-01
8.474864	-0.009	0.009	3.48E-01	0.008	0.014	5.48E-01
2.021414485	0.014	0.010	1.44E-01	0.000	0.014	9.99E-01
3.03845	-0.010	0.007	1.72E-01	0.002	0.011	8.34E-01
3.664551	-0.006	0.005	2.39E-01	0.012	0.008	1.33E-01
2.025788109	0.011	0.008	1.47E-01	0.003	0.012	7.74E-01
4.166509	-0.019	0.015	2.06E-01	-0.007	0.022	7.40E-01
2.028479569	0.010	0.007	1.43E-01	0.005	0.010	5.97E-01
2.025451676	0.012	0.008	1.48E-01	0.003	0.012	7.90E-01
0.851301933	-0.010	0.007	1.56E-01	-0.013	0.010	1.90E-01
1.536615121	0.016	0.012	1.59E-01	-0.018	0.017	2.92E-01

2.598396379	-0.022	0.018	2.11E-01	0.006	0.026	8.15E-01
5.048635	0.012	0.010	2.29E-01	-0.015	0.014	2.94E-01
7.137208	0.013	0.010	1.80E-01	-0.014	0.014	3.41E-01
6.05154	0.008	0.007	2.31E-01	-0.005	0.010	6.31E-01
3.107082	-0.015	0.010	1.20E-01	-0.018	0.014	2.13E-01
4.330015	-0.017	0.013	2.17E-01	0.014	0.020	4.76E-01
8.560991	0.007	0.008	3.86E-01	0.002	0.013	8.73E-01
5.062092	0.010	0.009	2.74E-01	-0.014	0.014	3.23E-01
3.339894	-0.011	0.009	2.48E-01	0.016	0.014	2.31E-01
7.363964	0.010	0.008	2.04E-01	-0.001	0.012	9.22E-01
5.129042	0.010	0.008	2.07E-01	-0.003	0.012	7.86E-01
3.662533	-0.007	0.005	1.51E-01	0.008	0.007	2.34E-01
7.463885	0.009	0.007	2.38E-01	0.001	0.011	9.25E-01
3.947828	-0.009	0.007	2.31E-01	0.002	0.011	8.39E-01
5.870203	0.010	0.007	1.45E-01	-0.010	0.011	3.34E-01
7.355553	-0.010	0.010	3.20E-01	-0.014	0.015	3.48E-01
0.705963054	0.010	0.010	3.19E-01	0.004	0.015	7.70E-01
7.585	0.015	0.010	1.37E-01	0.016	0.015	2.88E-01
1.96758527	0.006	0.004	2.00E-01	0.000	0.007	9.85E-01
2.33631539	0.014	0.009	1.03E-01	0.008	0.013	5.37E-01
7.511994	-0.012	0.009	1.82E-01	-0.019	0.014	1.68E-01
2.028143137	0.010	0.007	1.47E-01	0.005	0.011	6.40E-01
2.562398092	-0.015	0.012	2.14E-01	0.009	0.018	5.94E-01
8.728535	0.011	0.009	2.21E-01	-0.019	0.014	1.82E-01
1.908373134	-0.009	0.006	9.96E-02	-0.015	0.009	7.59E-02
3.155192	-0.012	0.009	1.75E-01	-0.008	0.013	5.35E-01
6.951161	-0.009	0.007	2.23E-01	0.001	0.011	9.07E-01
2.492420113	-0.026	0.018	1.54E-01	0.003	0.027	9.03E-01
6.786309	0.013	0.009	1.62E-01	0.017	0.014	2.33E-01
2.645833374	-0.014	0.010	1.96E-01	0.017	0.016	2.81E-01
5.708379	0.008	0.009	3.62E-01	-0.007	0.014	5.95E-01
6.248354	0.009	0.006	1.65E-01	-0.002	0.009	8.31E-01
1.093869831	0.010	0.007	1.93E-01	0.003	0.011	7.60E-01
7.415438	-0.013	0.010	1.96E-01	-0.024	0.015	1.16E-01
7.64253	-0.010	0.011	3.64E-01	-0.014	0.016	3.91E-01
6.531966	0.009	0.008	2.14E-01	0.000	0.011	9.71E-01
5.256887	-0.010	0.005	5.85E-02	-0.009	0.008	2.21E-01
6.757376	-0.007	0.006	2.33E-01	-0.002	0.009	8.31E-01
3.725782	0.007	0.008	4.00E-01	0.015	0.012	2.22E-01
1.088150477	0.010	0.007	1.86E-01	0.004	0.011	6.87E-01
7.777776	-0.012	0.010	2.33E-01	-0.015	0.015	3.23E-01
1.186052361	0.007	0.005	1.68E-01	0.005	0.008	5.22E-01
5.756826	0.011	0.010	2.38E-01	-0.014	0.014	3.22E-01

3.748659	0.010	0.009	2.58E-01	0.037	0.013	4.19E-03
7.042671	-0.013	0.013	3.13E-01	-0.012	0.019	5.27E-01
1.769762907	-0.013	0.010	1.95E-01	-0.026	0.015	8.75E-02
4.157761	-0.018	0.015	2.37E-01	0.015	0.022	4.98E-01
6.068026	0.007	0.008	3.56E-01	0.013	0.011	2.33E-01
2.757528994	-0.011	0.007	1.26E-01	-0.025	0.011	2.76E-02
5.544873	0.009	0.007	2.16E-01	0.014	0.011	1.73E-01
4.435991	-0.018	0.016	2.55E-01	0.021	0.023	3.77E-01
3.613414	-0.009	0.008	2.60E-01	0.011	0.012	3.52E-01
3.124577	-0.013	0.008	1.13E-01	-0.001	0.013	9.48E-01
5.923023	-0.011	0.008	1.84E-01	-0.007	0.013	5.79E-01
6.316313	0.006	0.008	4.37E-01	-0.018	0.012	1.36E-01
1.965230242	0.006	0.005	2.02E-01	-0.002	0.007	7.62E-01
7.598458	0.016	0.010	1.24E-01	0.024	0.016	1.23E-01
6.678314	-0.010	0.007	1.84E-01	0.012	0.011	2.61E-01
1.445105456	0.005	0.004	1.87E-01	0.006	0.006	3.08E-01
3.377911	0.012	0.013	3.48E-01	0.005	0.019	7.73E-01
7.395589	0.008	0.007	2.52E-01	0.000	0.011	9.78E-01
3.198929	0.006	0.005	2.50E-01	0.018	0.008	2.70E-02
7.184645	0.011	0.008	1.83E-01	-0.008	0.013	5.07E-01
4.001657	0.011	0.009	2.32E-01	0.017	0.014	2.35E-01
5.311389	0.022	0.016	1.87E-01	-0.021	0.025	3.88E-01
1.093533399	0.010	0.007	1.78E-01	-0.004	0.011	7.28E-01
3.238291	-0.009	0.006	1.44E-01	0.005	0.009	5.35E-01
1.886505016	-0.011	0.007	1.34E-01	-0.010	0.011	3.42E-01
1.794322486	-0.012	0.010	2.38E-01	0.015	0.016	3.34E-01
4.438346	-0.019	0.016	2.41E-01	0.024	0.024	3.08E-01
0.815640078	0.005	0.005	3.77E-01	0.016	0.008	3.82E-02
3.132315	-0.014	0.009	1.04E-01	-0.018	0.013	1.62E-01
6.094604	0.010	0.009	2.78E-01	0.004	0.013	7.61E-01
3.219451	-0.019	0.015	1.88E-01	-0.010	0.022	6.50E-01
0.636321507	0.007	0.008	3.74E-01	0.009	0.011	4.10E-01
8.414643	-0.009	0.008	2.20E-01	-0.012	0.011	2.87E-01
2.424460729	-0.007	0.009	4.11E-01	0.026	0.013	4.74E-02
8.416325	0.009	0.008	2.66E-01	-0.009	0.012	4.68E-01
3.138034	-0.014	0.010	1.65E-01	-0.008	0.015	5.79E-01
5.459756	0.009	0.008	2.66E-01	-0.015	0.012	1.98E-01
5.975507	0.011	0.010	2.77E-01	-0.011	0.015	4.45E-01
2.042946171	0.008	0.006	1.39E-01	0.005	0.008	5.52E-01
8.741655	0.011	0.009	2.31E-01	-0.009	0.013	5.06E-01
1.145344018	0.009	0.006	1.26E-01	-0.008	0.009	4.06E-01
2.141857353	-0.018	0.013	1.82E-01	0.031	0.020	1.24E-01
7.942292	0.008	0.008	3.40E-01	-0.001	0.012	9.35E-01

3.990218	-0.009	0.008	2.53E-01	0.004	0.012	7.57E-01
4.223702	0.011	0.011	3.09E-01	0.011	0.017	5.00E-01
2.368949352	-0.007	0.007	3.36E-01	0.021	0.011	4.87E-02
5.480278	0.009	0.006	1.67E-01	0.017	0.009	7.01E-02
0.960642525	0.006	0.005	1.74E-01	0.013	0.007	5.45E-02
6.814906	0.009	0.008	2.56E-01	-0.009	0.011	4.25E-01
2.042609738	0.008	0.005	1.41E-01	0.005	0.008	5.05E-01
0.837171764	-0.013	0.009	1.28E-01	-0.011	0.013	3.93E-01
2.71547492	-0.010	0.006	8.66E-02	-0.016	0.009	7.81E-02
5.877605	0.014	0.011	2.09E-01	-0.008	0.016	6.20E-01
5.668007	0.011	0.009	2.02E-01	0.023	0.013	7.27E-02
8.266276	0.013	0.008	1.15E-01	-0.011	0.012	3.38E-01
5.046616	0.009	0.008	2.93E-01	0.000	0.012	9.72E-01
4.316894	-0.017	0.014	2.37E-01	0.024	0.022	2.74E-01
7.590383	-0.011	0.009	2.22E-01	-0.023	0.013	7.85E-02
1.165529973	0.008	0.005	1.06E-01	-0.001	0.008	9.27E-01
7.58601	0.012	0.009	1.90E-01	0.024	0.014	7.83E-02
3.725109	0.006	0.008	4.29E-01	0.006	0.012	6.31E-01
4.418833	-0.017	0.014	2.42E-01	0.005	0.021	8.18E-01
8.166692	0.010	0.008	2.47E-01	0.004	0.012	7.54E-01
6.718686	-0.013	0.010	2.04E-01	-0.005	0.015	7.46E-01
5.257223	-0.010	0.005	6.00E-02	-0.009	0.008	2.33E-01
1.917120382	-0.008	0.005	1.36E-01	-0.001	0.008	9.46E-01
8.48664	0.009	0.007	2.01E-01	-0.005	0.011	6.71E-01
7.3606	-0.013	0.010	1.65E-01	0.014	0.014	3.25E-01
7.248231	-0.014	0.009	1.52E-01	0.004	0.014	7.91E-01
6.753339	-0.011	0.010	2.60E-01	-0.029	0.014	3.85E-02
6.360386	-0.008	0.009	3.90E-01	-0.032	0.013	1.87E-02
3.11112	-0.017	0.010	1.08E-01	-0.011	0.015	4.69E-01
0.759119403	0.006	0.005	2.04E-01	-0.004	0.008	6.05E-01
2.294597749	-0.006	0.005	2.71E-01	-0.007	0.008	3.42E-01
5.273035	-0.011	0.006	9.60E-02	-0.020	0.009	3.30E-02
8.576131	-0.010	0.010	2.87E-01	-0.014	0.015	3.45E-01
5.006244	0.013	0.012	2.44E-01	-0.007	0.017	6.70E-01
8.860416	0.008	0.007	2.41E-01	0.002	0.010	8.46E-01
7.365983	-0.008	0.008	3.04E-01	-0.005	0.012	6.66E-01
0.521934426	0.012	0.010	2.14E-01	0.004	0.014	7.76E-01
0.918252019	-0.006	0.005	2.35E-01	-0.023	0.007	9.05E-04
8.262912	-0.008	0.008	2.60E-01	-0.017	0.011	1.35E-01
6.687735	-0.011	0.009	2.55E-01	-0.011	0.014	4.07E-01
6.140695	0.014	0.011	1.81E-01	-0.002	0.016	8.79E-01
5.917304	0.009	0.008	2.78E-01	-0.009	0.012	4.46E-01
6.835092	0.010	0.009	2.96E-01	-0.015	0.014	2.74E-01

4.402684	-0.014	0.012	2.28E-01	0.002	0.018	9.18E-01
1.419536579	0.008	0.005	1.11E-01	0.005	0.007	4.73E-01
7.745142	-0.013	0.010	1.72E-01	-0.017	0.015	2.49E-01
8.97043	-0.013	0.009	1.35E-01	-0.022	0.013	9.77E-02
7.228718	-0.017	0.011	1.30E-01	-0.003	0.016	8.49E-01
8.032792	0.009	0.008	3.10E-01	0.003	0.013	8.07E-01
3.216087	-0.019	0.014	1.81E-01	-0.010	0.021	6.52E-01
7.087753	0.008	0.007	2.56E-01	0.005	0.011	6.55E-01
0.662899682	-0.009	0.008	2.90E-01	-0.026	0.012	3.37E-02
3.88155	0.007	0.006	2.68E-01	0.022	0.009	1.37E-02
0.859376315	0.013	0.009	1.71E-01	-0.001	0.014	9.42E-01
8.337264	0.011	0.008	1.42E-01	-0.012	0.011	2.95E-01
0.769548813	0.007	0.005	1.66E-01	0.004	0.008	6.06E-01
7.140573	-0.013	0.011	2.09E-01	-0.012	0.016	4.60E-01
5.576834	0.009	0.008	2.33E-01	-0.006	0.011	5.90E-01
5.717799	0.007	0.008	3.23E-01	0.001	0.011	9.24E-01
8.729544	-0.012	0.009	1.68E-01	-0.029	0.013	3.18E-02
4.013768	-0.014	0.012	2.46E-01	0.004	0.018	8.28E-01
8.747038	-0.012	0.010	2.15E-01	-0.027	0.014	6.10E-02
7.657333	0.013	0.010	1.94E-01	-0.004	0.015	7.72E-01
8.197644	0.009	0.007	2.01E-01	-0.001	0.011	9.09E-01
6.051877	0.009	0.007	2.08E-01	-0.003	0.011	7.84E-01
5.735294	0.012	0.010	2.24E-01	0.004	0.015	8.08E-01
8.316741	0.009	0.008	3.01E-01	0.002	0.013	8.84E-01
3.199265	0.006	0.005	2.50E-01	0.018	0.008	2.45E-02
2.042273306	0.008	0.005	1.45E-01	0.006	0.008	4.67E-01
6.313285	-0.006	0.006	3.56E-01	-0.006	0.009	5.16E-01
7.379103	-0.012	0.010	2.34E-01	-0.003	0.015	8.62E-01
2.416386347	-0.009	0.009	3.19E-01	0.010	0.013	4.31E-01
1.624087595	0.010	0.007	1.46E-01	0.015	0.010	1.31E-01
8.803223	0.010	0.008	1.88E-01	-0.008	0.012	4.73E-01
3.200611	0.007	0.006	2.42E-01	0.019	0.008	2.52E-02
1.409443602	0.009	0.006	1.02E-01	0.018	0.008	3.40E-02
6.220766	0.010	0.007	1.61E-01	-0.027	0.011	1.42E-02
2.179537803	0.016	0.011	1.44E-01	-0.009	0.017	5.90E-01
8.296892	0.011	0.008	1.54E-01	-0.004	0.012	7.57E-01
7.300378	-0.010	0.007	1.81E-01	-0.010	0.011	3.87E-01
5.969787	0.006	0.007	4.38E-01	0.000	0.011	9.88E-01
5.25756	-0.010	0.005	6.34E-02	-0.009	0.008	2.25E-01
8.797167	-0.006	0.008	4.34E-01	-0.006	0.011	5.69E-01
5.699632	0.013	0.011	2.25E-01	-0.029	0.016	6.82E-02
2.02208735	0.014	0.009	1.51E-01	0.001	0.014	9.60E-01
2.714802055	-0.010	0.006	9.06E-02	-0.015	0.009	8.89E-02

1.531568632	0.014	0.010	1.77E-01	-0.018	0.015	2.25E-01
7.057138	-0.009	0.008	2.50E-01	-0.021	0.012	6.68E-02
6.489576	0.007	0.006	2.74E-01	-0.009	0.009	3.45E-01
3.923604	0.011	0.010	2.46E-01	0.036	0.015	1.51E-02
1.240218008	0.009	0.007	2.26E-01	-0.006	0.011	5.90E-01
2.168099095	0.014	0.011	1.90E-01	-0.009	0.016	5.60E-01
6.849559	-0.012	0.009	2.03E-01	-0.019	0.014	1.58E-01
5.109529	0.010	0.008	2.26E-01	-0.007	0.012	5.38E-01
5.585581	0.009	0.006	1.59E-01	-0.012	0.010	2.19E-01
7.267408	-0.016	0.012	1.58E-01	0.008	0.017	6.54E-01
6.719023	-0.012	0.009	1.97E-01	-0.015	0.013	2.73E-01
6.438438	0.011	0.010	2.60E-01	-0.008	0.015	5.93E-01
1.021200391	0.011	0.007	1.11E-01	0.008	0.011	4.26E-01
8.596989	-0.013	0.010	2.16E-01	-0.001	0.015	9.46E-01
4.440028	-0.017	0.015	2.65E-01	0.015	0.023	5.14E-01
7.979972	0.012	0.010	2.14E-01	0.007	0.014	6.15E-01
1.472356496	-0.005	0.004	2.57E-01	-0.001	0.007	9.02E-01
6.33078	0.012	0.009	1.64E-01	-0.005	0.013	6.71E-01
6.704893	-0.010	0.009	2.23E-01	-0.014	0.013	2.60E-01
2.314110839	-0.006	0.005	2.09E-01	-0.008	0.007	3.08E-01
4.168527	-0.016	0.014	2.39E-01	0.010	0.021	6.24E-01
3.04888	-0.013	0.008	9.78E-02	-0.008	0.011	4.92E-01
3.334174	0.012	0.009	1.76E-01	0.016	0.013	2.45E-01
2.62060093	-0.015	0.013	2.24E-01	0.017	0.019	3.57E-01
4.443056	-0.018	0.016	2.47E-01	0.016	0.023	4.79E-01
1.909382432	-0.011	0.007	1.15E-01	-0.021	0.010	3.14E-02
6.862007	0.014	0.011	1.97E-01	0.018	0.016	2.43E-01
7.718228	-0.011	0.010	2.93E-01	-0.007	0.015	6.31E-01
5.066802	0.010	0.008	1.99E-01	-0.007	0.012	5.57E-01
5.02744	0.012	0.009	1.99E-01	-0.007	0.014	5.87E-01
2.101149009	-0.006	0.007	3.81E-01	0.007	0.010	5.16E-01
5.049981	0.011	0.009	1.99E-01	0.003	0.013	8.40E-01
6.015542	0.010	0.009	2.72E-01	0.013	0.013	3.36E-01
7.619653	-0.020	0.013	1.13E-01	-0.002	0.019	9.35E-01
1.220704918	0.009	0.007	2.42E-01	0.002	0.011	8.57E-01
2.026460974	0.011	0.008	1.58E-01	0.004	0.011	7.49E-01
7.310135	-0.016	0.010	9.33E-02	-0.008	0.014	5.90E-01
5.300287	0.019	0.014	1.79E-01	-0.017	0.021	4.19E-01
7.323929	-0.011	0.006	8.39E-02	0.003	0.009	7.61E-01
3.122558	-0.013	0.008	1.33E-01	-0.001	0.012	9.43E-01
4.116717	-0.011	0.010	2.68E-01	0.019	0.015	1.94E-01
2.565762417	-0.011	0.010	2.88E-01	0.002	0.015	9.17E-01
8.340291	0.011	0.009	2.11E-01	0.002	0.013	8.84E-01

2.856104	-0.007	0.007	3.31E-01	-0.007	0.011	5.03E-01
3.764472	0.013	0.009	1.67E-01	0.022	0.014	1.16E-01
8.01597	-0.009	0.008	2.70E-01	-0.010	0.012	4.24E-01
5.433177	0.010	0.010	2.95E-01	0.007	0.015	6.51E-01
1.482449474	-0.006	0.006	3.16E-01	0.001	0.009	9.06E-01
6.143387	-0.011	0.010	2.73E-01	-0.022	0.015	1.24E-01
5.92807	0.016	0.010	1.21E-01	0.000	0.015	9.78E-01
8.533067	0.008	0.006	1.85E-01	-0.005	0.009	6.29E-01
8.992298	0.010	0.009	2.50E-01	0.025	0.013	6.25E-02
6.848549	-0.010	0.009	2.51E-01	0.009	0.013	5.16E-01
8.063407	-0.013	0.009	1.67E-01	0.004	0.014	7.68E-01
3.595246	-0.009	0.007	1.86E-01	0.013	0.010	1.89E-01
0.661217519	-0.012	0.009	1.95E-01	-0.009	0.014	5.18E-01
3.696176	0.007	0.005	2.22E-01	0.021	0.008	9.50E-03
7.428559	0.011	0.010	2.64E-01	0.013	0.014	3.57E-01
7.185318	0.013	0.008	1.35E-01	-0.003	0.013	8.10E-01
4.145986	-0.013	0.012	2.94E-01	0.004	0.018	8.39E-01
6.268876	0.012	0.010	2.33E-01	-0.018	0.015	2.16E-01
8.725843	0.006	0.006	3.00E-01	0.005	0.009	5.46E-01
2.68553242	-0.011	0.006	9.45E-02	-0.004	0.010	6.82E-01
1.905008808	-0.008	0.006	1.94E-01	-0.004	0.009	6.90E-01
6.657456	-0.010	0.009	2.49E-01	-0.013	0.014	3.50E-01
1.532914363	0.014	0.010	1.73E-01	-0.019	0.016	2.34E-01
6.697828	0.008	0.007	2.51E-01	0.014	0.010	1.68E-01
5.26126	-0.010	0.005	7.59E-02	-0.011	0.008	1.58E-01
3.197919	0.006	0.005	2.70E-01	0.017	0.008	3.43E-02
0.95088598	0.006	0.005	1.77E-01	0.000	0.007	9.55E-01
2.376687301	-0.016	0.012	1.95E-01	0.008	0.018	6.77E-01
1.895588696	-0.009	0.006	1.52E-01	-0.004	0.009	6.39E-01
8.747375	-0.012	0.010	2.00E-01	-0.012	0.014	3.91E-01
7.568515	0.012	0.009	1.59E-01	-0.004	0.013	7.73E-01
8.289827	-0.009	0.008	2.81E-01	-0.025	0.012	4.65E-02
8.137759	-0.011	0.009	1.80E-01	-0.026	0.013	4.07E-02
2.150268167	-0.009	0.007	2.01E-01	0.002	0.011	8.87E-01
5.154611	0.014	0.011	2.08E-01	-0.017	0.016	2.86E-01
7.682566	0.010	0.009	2.41E-01	0.004	0.013	7.40E-01
7.570534	-0.010	0.009	2.67E-01	-0.017	0.014	2.24E-01
4.225384	0.012	0.011	2.60E-01	0.017	0.016	3.05E-01
7.793252	-0.011	0.010	2.94E-01	0.016	0.016	3.03E-01
8.066772	0.011	0.007	1.29E-01	0.019	0.011	9.54E-02
7.056465	-0.010	0.011	3.80E-01	-0.013	0.016	4.07E-01
7.268081	-0.017	0.011	1.10E-01	0.000	0.016	9.85E-01
8.349375	-0.008	0.008	3.44E-01	0.007	0.013	5.57E-01

3.498354	0.008	0.009	3.68E-01	0.032	0.013	1.61E-02
5.28111	-0.011	0.007	1.27E-01	-0.025	0.011	1.80E-02
1.535269391	0.015	0.011	1.84E-01	-0.020	0.017	2.22E-01
1.825274284	-0.014	0.010	1.79E-01	-0.005	0.016	7.44E-01
7.344115	-0.011	0.010	2.88E-01	0.007	0.016	6.72E-01
0.667273306	-0.009	0.008	2.76E-01	-0.020	0.013	1.09E-01
6.162563	0.013	0.010	1.96E-01	-0.008	0.015	5.91E-01
4.13051	-0.015	0.014	2.63E-01	0.015	0.021	4.62E-01
0.607388304	-0.009	0.010	3.81E-01	0.004	0.015	8.07E-01
7.30307	-0.015	0.010	1.31E-01	-0.002	0.015	8.96E-01
7.501229	0.012	0.010	2.18E-01	0.014	0.015	3.36E-01
5.627972	0.009	0.007	1.86E-01	-0.020	0.010	3.72E-02
2.526399804	-0.016	0.011	1.41E-01	-0.001	0.016	9.67E-01
8.53946	0.006	0.006	2.98E-01	0.007	0.009	4.53E-01
3.658832	-0.006	0.005	2.84E-01	0.009	0.008	2.57E-01
0.594940299	-0.011	0.009	2.21E-01	-0.010	0.013	4.41E-01
8.508844	0.011	0.010	2.45E-01	0.009	0.014	5.14E-01
2.267010276	0.009	0.007	2.18E-01	0.010	0.011	3.81E-01
4.139594	-0.023	0.019	2.25E-01	0.014	0.028	6.13E-01
6.21101	0.007	0.007	3.41E-01	0.004	0.010	7.14E-01
2.564080254	-0.009	0.009	2.73E-01	0.006	0.013	6.23E-01
5.073195	0.009	0.008	2.24E-01	-0.013	0.012	2.52E-01
5.272699	-0.011	0.006	9.39E-02	-0.020	0.009	3.30E-02
7.576589	0.009	0.009	3.41E-01	-0.030	0.014	3.23E-02
5.08867	0.009	0.007	2.28E-01	-0.010	0.011	3.57E-01
1.986761928	0.011	0.008	1.52E-01	-0.006	0.011	6.26E-01
6.598916	-0.013	0.009	1.49E-01	-0.025	0.014	6.66E-02
3.109437	-0.015	0.010	1.28E-01	-0.013	0.015	3.92E-01
5.281446	-0.011	0.007	1.27E-01	-0.025	0.011	1.98E-02
7.237129	-0.019	0.011	9.16E-02	-0.017	0.017	3.32E-01
0.911186934	-0.006	0.005	2.60E-01	-0.026	0.008	9.66E-04
2.026797406	0.011	0.008	1.60E-01	0.004	0.011	7.24E-01
1.521475655	0.012	0.009	2.07E-01	-0.015	0.014	2.62E-01
2.310410081	-0.022	0.017	1.81E-01	0.001	0.025	9.73E-01
7.839343	-0.009	0.008	2.85E-01	0.007	0.013	5.84E-01
5.931098	0.010	0.008	2.39E-01	-0.009	0.012	4.69E-01
2.616900171	-0.016	0.013	2.19E-01	0.022	0.019	2.61E-01
7.785514	-0.008	0.006	2.27E-01	-0.004	0.009	6.32E-01
3.793741	0.009	0.006	1.41E-01	0.013	0.009	1.63E-01
4.325978	-0.016	0.013	2.37E-01	0.016	0.020	4.15E-01
5.280101	-0.011	0.007	1.23E-01	-0.024	0.010	1.95E-02
5.25655	-0.009	0.005	6.70E-02	-0.009	0.008	2.55E-01
1.843441644	-0.011	0.010	2.89E-01	0.015	0.015	3.02E-01

1.200518963	0.006	0.006	2.72E-01	0.008	0.008	3.64E-01
3.944463	-0.011	0.009	2.44E-01	-0.001	0.013	9.68E-01
6.719359	-0.011	0.010	2.93E-01	-0.007	0.015	6.47E-01
0.744989234	-0.009	0.006	1.40E-01	-0.007	0.009	4.37E-01
5.686848	0.010	0.008	2.08E-01	0.018	0.011	1.19E-01
5.512912	0.008	0.010	3.81E-01	-0.007	0.014	6.47E-01
1.833348667	-0.013	0.010	1.81E-01	0.004	0.015	7.66E-01
1.5312322	0.014	0.010	1.76E-01	-0.016	0.015	2.90E-01
2.983275	-0.005	0.004	2.25E-01	-0.001	0.006	8.43E-01
1.638890629	0.008	0.006	2.00E-01	-0.001	0.009	9.34E-01
5.873568	0.011	0.008	1.88E-01	-0.009	0.012	4.73E-01
5.864484	0.011	0.008	1.94E-01	0.003	0.013	7.84E-01
5.014992	0.012	0.009	2.04E-01	-0.007	0.014	6.14E-01
5.538144	0.010	0.008	2.09E-01	0.012	0.012	2.97E-01
5.508538	0.009	0.008	2.46E-01	-0.007	0.012	5.38E-01
1.239881576	0.009	0.007	2.32E-01	-0.006	0.011	5.65E-01
1.647974309	0.007	0.005	1.44E-01	0.004	0.008	5.64E-01
8.868154	0.011	0.009	2.33E-01	0.006	0.014	6.66E-01
5.476241	0.010	0.007	1.41E-01	-0.004	0.010	6.66E-01
1.805761194	-0.012	0.011	2.40E-01	0.010	0.016	5.25E-01
2.797564473	-0.016	0.013	2.06E-01	-0.068	0.019	3.79E-04
2.397882554	-0.018	0.014	1.78E-01	-0.007	0.020	7.30E-01
3.198256	0.006	0.005	2.67E-01	0.017	0.008	3.01E-02
8.104452	0.007	0.007	2.86E-01	0.001	0.010	9.33E-01
7.83968	-0.008	0.009	3.33E-01	0.008	0.013	5.13E-01
5.101455	0.008	0.008	3.06E-01	0.001	0.012	9.38E-01
5.792151	-0.011	0.009	2.01E-01	-0.003	0.013	8.31E-01
3.701559	0.007	0.006	2.37E-01	0.020	0.009	2.01E-02
6.921892	-0.013	0.010	2.12E-01	-0.002	0.015	9.07E-01
5.387759	0.008	0.006	1.65E-01	0.002	0.009	8.52E-01
7.269426	-0.013	0.010	2.01E-01	-0.006	0.015	6.77E-01
1.986425495	0.011	0.007	1.53E-01	-0.005	0.011	6.35E-01
6.167946	0.007	0.008	3.78E-01	0.008	0.012	4.68E-01
4.224039	0.011	0.011	3.05E-01	0.011	0.016	5.04E-01
8.96572	-0.012	0.009	1.86E-01	0.004	0.014	7.64E-01
8.119592	-0.013	0.009	1.44E-01	0.006	0.013	6.33E-01
5.272363	-0.010	0.006	9.59E-02	-0.019	0.009	3.95E-02
6.68908	-0.009	0.007	2.24E-01	-0.011	0.011	3.10E-01
1.022546122	0.010	0.006	1.11E-01	-0.002	0.009	8.08E-01
1.662777343	0.005	0.004	2.18E-01	0.004	0.006	5.43E-01
2.027806704	0.010	0.007	1.59E-01	0.004	0.011	6.83E-01
4.378125	-0.014	0.012	2.25E-01	0.011	0.018	5.41E-01
4.437337	-0.018	0.015	2.50E-01	0.021	0.023	3.54E-01

5.649504	0.009	0.008	2.36E-01	-0.001	0.012	9.24E-01
2.552977979	-0.013	0.011	2.27E-01	-0.006	0.016	7.32E-01
0.726821874	0.006	0.007	4.00E-01	0.010	0.011	3.68E-01
6.472081	-0.011	0.009	2.42E-01	0.004	0.014	7.62E-01
0.769885246	0.007	0.005	1.65E-01	-0.002	0.008	8.40E-01
1.527195009	0.012	0.009	1.90E-01	-0.016	0.014	2.49E-01
3.226516	-0.009	0.007	1.83E-01	0.008	0.010	4.35E-01
7.271782	-0.016	0.011	1.39E-01	0.000	0.016	9.94E-01
6.14776	0.009	0.008	2.50E-01	0.004	0.012	7.58E-01
1.747221923	-0.008	0.007	3.07E-01	0.001	0.011	9.15E-01
5.304997	0.020	0.015	1.82E-01	-0.021	0.022	3.38E-01
3.200274	0.006	0.006	2.56E-01	0.018	0.008	2.65E-02
8.255174	0.012	0.008	1.33E-01	0.007	0.012	5.70E-01
7.579617	-0.012	0.010	2.32E-01	-0.013	0.015	3.76E-01
7.700733	-0.011	0.009	2.28E-01	0.012	0.014	3.87E-01
0.805547101	0.004	0.005	4.60E-01	0.013	0.008	1.26E-01
2.373322975	-0.010	0.008	2.20E-01	0.014	0.012	2.35E-01
5.506856	0.009	0.008	2.94E-01	0.001	0.012	9.63E-01
2.026124541	0.011	0.008	1.60E-01	0.003	0.011	7.66E-01
2.835917788	-0.008	0.010	4.25E-01	-0.030	0.016	5.52E-02
7.821512	-0.014	0.010	1.79E-01	-0.004	0.015	8.02E-01
5.106165	0.009	0.008	2.58E-01	-0.013	0.011	2.67E-01
2.672411549	-0.010	0.007	1.29E-01	0.007	0.010	5.14E-01
8.705657	0.009	0.009	2.82E-01	0.018	0.013	1.57E-01
5.278755	-0.011	0.007	1.19E-01	-0.025	0.010	1.61E-02
0.607051872	-0.007	0.007	3.50E-01	0.006	0.011	5.63E-01
6.608673	-0.012	0.010	2.14E-01	-0.004	0.015	8.07E-01
5.277409	-0.011	0.007	1.18E-01	-0.023	0.010	2.32E-02
5.095736	0.010	0.009	2.69E-01	-0.008	0.013	5.32E-01
5.163695	0.013	0.011	2.31E-01	-0.020	0.016	2.18E-01
5.260924	-0.010	0.005	7.42E-02	-0.011	0.008	1.69E-01
5.144182	0.008	0.008	2.88E-01	-0.008	0.012	4.77E-01
7.439998	-0.012	0.010	2.00E-01	-0.014	0.014	3.22E-01
2.497466601	-0.017	0.014	2.09E-01	-0.011	0.021	6.10E-01
5.644457	0.008	0.007	2.08E-01	0.000	0.010	9.77E-01
3.200947	0.007	0.006	2.50E-01	0.019	0.009	2.53E-02
7.362282	-0.014	0.009	1.28E-01	-0.010	0.014	4.75E-01
4.401002	-0.018	0.015	2.42E-01	0.016	0.023	4.69E-01
2.095093222	-0.007	0.008	3.35E-01	-0.003	0.012	7.65E-01
7.218962	-0.016	0.010	1.09E-01	0.012	0.015	4.07E-01
6.600935	-0.010	0.008	2.59E-01	-0.022	0.013	8.05E-02
5.682138	0.009	0.009	3.19E-01	-0.001	0.013	9.37E-01
8.895405	0.007	0.007	2.79E-01	-0.001	0.010	9.01E-01

3.199938	0.006	0.006	2.57E-01	0.018	0.008	2.58E-02
5.261933	-0.010	0.006	7.62E-02	-0.013	0.008	1.06E-01
8.329189	-0.009	0.008	2.72E-01	-0.026	0.012	2.77E-02
5.609468	-0.013	0.010	2.00E-01	-0.017	0.015	2.57E-01
7.735386	-0.014	0.011	2.22E-01	-0.014	0.017	4.09E-01
8.280743	-0.012	0.008	1.74E-01	-0.013	0.013	2.89E-01
8.18385	0.008	0.009	3.83E-01	-0.008	0.013	5.34E-01
6.140359	0.013	0.010	1.99E-01	-0.009	0.015	5.62E-01
2.086345975	-0.007	0.006	2.25E-01	0.006	0.008	4.80E-01
5.777348	0.010	0.009	2.49E-01	0.001	0.013	9.49E-01
4.306465	-0.015	0.012	1.99E-01	0.012	0.018	4.95E-01
8.294537	0.013	0.010	1.89E-01	-0.004	0.015	7.87E-01
1.432993883	0.008	0.005	9.07E-02	0.001	0.007	8.55E-01
3.044842	-0.005	0.005	3.25E-01	-0.006	0.008	4.82E-01
2.350781992	0.016	0.010	9.18E-02	0.018	0.014	2.20E-01
1.889869342	0.011	0.007	1.44E-01	0.005	0.011	6.55E-01
4.330688	-0.019	0.016	2.33E-01	0.019	0.023	4.10E-01
5.500464	0.009	0.008	2.63E-01	-0.019	0.012	1.10E-01
3.046188	-0.008	0.006	2.23E-01	-0.009	0.009	3.16E-01
0.711345975	0.008	0.008	3.23E-01	0.001	0.012	9.61E-01
2.027133839	0.011	0.008	1.63E-01	0.004	0.011	7.08E-01
2.406293369	-0.019	0.015	2.11E-01	-0.015	0.023	5.12E-01
3.161585	-0.010	0.007	1.52E-01	-0.002	0.011	8.52E-01
3.862037	-0.007	0.005	1.82E-01	0.006	0.008	4.18E-01
5.31509	0.021	0.017	2.01E-01	-0.024	0.025	3.26E-01
1.47538439	-0.005	0.004	2.37E-01	0.001	0.006	8.80E-01
5.755143	0.009	0.008	2.35E-01	-0.005	0.012	6.83E-01
5.734621	0.011	0.010	2.88E-01	0.000	0.015	9.89E-01
5.833869	0.010	0.009	2.67E-01	0.002	0.014	8.60E-01
8.771934	0.009	0.007	2.01E-01	0.007	0.010	4.86E-01
0.509822853	0.011	0.010	2.38E-01	-0.005	0.014	7.24E-01
6.092249	0.007	0.007	3.30E-01	0.002	0.010	8.47E-01
6.24398	0.008	0.009	3.90E-01	0.003	0.013	8.44E-01
3.873812	-0.005	0.005	2.40E-01	0.010	0.007	1.43E-01
1.221041351	0.008	0.007	2.53E-01	0.002	0.011	8.86E-01
2.570808906	-0.009	0.010	3.83E-01	-0.002	0.015	8.72E-01
5.278418	-0.011	0.007	1.20E-01	-0.024	0.010	1.82E-02
0.663236115	-0.010	0.010	2.89E-01	-0.023	0.015	1.10E-01
5.275054	-0.011	0.007	1.07E-01	-0.022	0.010	2.66E-02
2.417059212	-0.008	0.010	3.80E-01	0.011	0.014	4.43E-01
7.665071	0.009	0.009	2.98E-01	0.001	0.013	9.33E-01
0.671646929	-0.012	0.010	2.26E-01	-0.001	0.014	9.59E-01
0.836835332	-0.013	0.009	1.38E-01	-0.011	0.013	4.13E-01

7.821176	-0.012	0.010	2.49E-01	0.001	0.015	9.41E-01
5.280773	-0.011	0.007	1.31E-01	-0.025	0.011	1.98E-02
5.468166	0.007	0.007	3.34E-01	0.001	0.010	9.56E-01
7.100874	-0.009	0.010	3.87E-01	0.008	0.015	6.08E-01
5.639074	0.008	0.007	2.25E-01	0.002	0.010	8.33E-01
1.885832151	-0.012	0.008	1.20E-01	-0.012	0.011	2.75E-01
1.824601419	-0.010	0.008	2.00E-01	0.005	0.012	6.79E-01
0.729513335	-0.010	0.007	1.59E-01	-0.021	0.010	4.02E-02
5.576161	0.008	0.008	3.01E-01	-0.009	0.012	4.80E-01
2.578883288	-0.008	0.010	4.10E-01	-0.002	0.015	8.97E-01
5.439233	-0.008	0.008	2.79E-01	-0.015	0.012	2.06E-01
2.866533	-0.008	0.008	3.55E-01	-0.001	0.013	9.31E-01
3.543436	-0.017	0.012	1.69E-01	-0.025	0.018	1.63E-01
6.898341	0.010	0.008	2.46E-01	0.000	0.012	9.69E-01
8.597326	-0.012	0.008	1.41E-01	-0.005	0.012	6.72E-01
2.376350869	-0.019	0.014	1.91E-01	0.004	0.021	8.38E-01
4.151369	-0.027	0.021	2.05E-01	0.011	0.032	7.34E-01
2.75954759	-0.012	0.009	1.69E-01	-0.035	0.013	7.50E-03
0.592248838	-0.014	0.010	1.47E-01	-0.014	0.014	3.28E-01
5.132743	0.012	0.010	2.31E-01	-0.003	0.015	8.62E-01
7.191038	0.011	0.010	2.97E-01	-0.007	0.015	6.45E-01
2.353473452	0.015	0.009	1.07E-01	0.016	0.014	2.54E-01
7.068913	-0.015	0.010	1.53E-01	-0.008	0.016	6.01E-01
2.524044776	-0.015	0.013	2.47E-01	-0.001	0.019	9.60E-01
7.95844	0.008	0.008	2.95E-01	-0.009	0.012	4.73E-01
7.722601	-0.012	0.011	2.51E-01	-0.017	0.016	2.67E-01
2.759884023	-0.013	0.009	1.59E-01	-0.035	0.013	8.24E-03
7.717218	-0.012	0.010	2.58E-01	-0.019	0.015	2.12E-01
1.53257793	0.014	0.010	1.78E-01	-0.020	0.015	2.04E-01
1.699112063	-0.004	0.004	3.61E-01	0.004	0.006	5.14E-01
2.838272816	-0.009	0.009	3.43E-01	-0.015	0.014	2.66E-01
0.900421091	0.011	0.008	1.30E-01	-0.013	0.011	2.53E-01
1.421218742	0.007	0.005	1.25E-01	0.002	0.007	8.09E-01
7.481379	-0.012	0.009	1.94E-01	-0.015	0.013	2.65E-01
4.307474	-0.015	0.012	1.99E-01	0.004	0.017	8.13E-01
1.533587228	0.014	0.011	1.85E-01	-0.017	0.016	2.91E-01
5.613505	0.008	0.007	2.47E-01	-0.013	0.011	2.17E-01
2.139502324	-0.016	0.012	1.79E-01	0.036	0.017	3.65E-02
7.014411	0.011	0.009	2.50E-01	0.001	0.014	9.21E-01
3.590536	-0.005	0.006	3.66E-01	0.001	0.009	8.97E-01
5.765909	0.009	0.009	2.90E-01	-0.019	0.013	1.40E-01
5.9893	0.007	0.008	3.74E-01	0.001	0.012	9.46E-01
3.219115	-0.019	0.015	2.05E-01	-0.010	0.022	6.58E-01

7.369011	0.012	0.009	2.08E-01	0.018	0.014	2.04E-01
2.270711035	0.008	0.006	1.76E-01	0.004	0.009	6.34E-01
5.276736	-0.010	0.007	1.18E-01	-0.022	0.010	2.84E-02
7.395925	0.008	0.007	2.80E-01	0.005	0.011	6.32E-01
3.726455	0.007	0.008	3.76E-01	0.019	0.012	1.12E-01
7.445717	0.012	0.009	1.76E-01	-0.004	0.013	7.88E-01
3.294139	-0.010	0.012	4.11E-01	0.009	0.018	6.28E-01
6.556189	-0.009	0.009	3.21E-01	-0.017	0.013	2.17E-01
8.314723	0.010	0.009	2.84E-01	-0.002	0.014	8.68E-01
3.376229	0.014	0.013	2.77E-01	-0.005	0.019	7.95E-01
5.277746	-0.011	0.007	1.19E-01	-0.023	0.010	2.04E-02
7.426204	-0.011	0.008	1.83E-01	-0.007	0.012	5.56E-01
8.228596	-0.013	0.010	1.83E-01	-0.018	0.014	2.11E-01
5.315426	0.021	0.017	2.06E-01	-0.024	0.025	3.24E-01
4.404703	-0.015	0.013	2.57E-01	0.006	0.019	7.39E-01
3.046525	-0.008	0.006	2.05E-01	-0.013	0.010	1.73E-01
2.339006851	0.015	0.008	8.42E-02	0.014	0.013	2.75E-01
5.326192	0.024	0.019	2.05E-01	-0.026	0.028	3.49E-01
2.796891608	-0.016	0.013	2.14E-01	-0.066	0.019	4.78E-04
4.405039	-0.015	0.013	2.52E-01	0.005	0.020	8.09E-01
6.787991	-0.008	0.010	4.50E-01	0.002	0.015	9.00E-01
5.326528	0.024	0.019	2.05E-01	-0.024	0.028	3.87E-01
7.545638	0.010	0.010	3.08E-01	0.009	0.015	5.33E-01
8.137086	0.012	0.009	1.70E-01	-0.014	0.013	3.02E-01
2.756856129	-0.011	0.007	1.33E-01	-0.025	0.011	2.08E-02
6.337508	0.009	0.007	1.87E-01	0.002	0.010	8.51E-01
5.649167	0.010	0.009	2.84E-01	0.001	0.014	9.60E-01
2.149595302	-0.011	0.007	1.55E-01	-0.001	0.011	9.09E-01
2.161370443	-0.012	0.010	2.31E-01	-0.014	0.014	3.33E-01
8.884303	0.010	0.008	1.91E-01	0.011	0.012	3.31E-01
1.427610962	0.007	0.005	1.21E-01	0.009	0.007	1.94E-01
1.239545143	0.009	0.007	2.40E-01	-0.006	0.011	5.68E-01
5.685838	0.010	0.008	2.20E-01	-0.006	0.012	6.30E-01
4.161799	-0.020	0.017	2.27E-01	-0.025	0.025	3.17E-01
3.659505	-0.006	0.005	2.52E-01	0.007	0.007	3.20E-01
8.663603	0.011	0.008	1.80E-01	0.010	0.013	4.10E-01
8.43853	0.009	0.007	2.15E-01	-0.012	0.011	2.90E-01
1.159137754	0.007	0.005	1.25E-01	-0.002	0.007	7.49E-01
6.304538	0.005	0.006	3.71E-01	-0.003	0.009	7.49E-01
7.309126	-0.015	0.010	1.35E-01	0.006	0.015	7.15E-01
7.020466	0.007	0.009	4.83E-01	0.001	0.014	9.68E-01
8.709694	0.007	0.008	4.01E-01	-0.013	0.012	2.76E-01
6.79472	0.014	0.010	1.41E-01	-0.006	0.014	6.67E-01

3.174033	-0.007	0.006	1.90E-01	0.004	0.009	6.31E-01
5.525696	0.011	0.008	1.87E-01	0.000	0.013	9.96E-01
3.627207	-0.008	0.006	2.38E-01	0.010	0.010	2.93E-01
1.528204306	0.012	0.010	2.26E-01	-0.015	0.014	2.84E-01
5.871213	0.013	0.010	1.87E-01	-0.010	0.015	4.75E-01
2.774687057	-0.015	0.011	1.81E-01	-0.049	0.016	2.44E-03
1.434339613	0.006	0.004	1.20E-01	0.006	0.006	3.17E-01
8.054324	0.010	0.010	3.49E-01	0.007	0.015	6.56E-01
5.770283	0.007	0.008	3.40E-01	0.008	0.012	4.82E-01
4.225048	0.011	0.010	2.78E-01	0.012	0.015	4.53E-01
2.027470272	0.010	0.007	1.65E-01	0.004	0.011	7.07E-01
6.33448	0.010	0.009	2.78E-01	-0.009	0.013	5.06E-01
7.749179	-0.009	0.011	4.15E-01	-0.012	0.016	4.53E-01
2.531109861	-0.012	0.011	2.69E-01	-0.004	0.017	8.27E-01
8.516246	0.006	0.007	3.41E-01	-0.003	0.010	7.93E-01
7.977954	0.008	0.010	4.20E-01	0.031	0.015	3.71E-02
8.851669	-0.011	0.009	1.86E-01	-0.023	0.013	7.10E-02
2.991686	-0.005	0.004	1.84E-01	0.005	0.006	4.27E-01
1.413817225	0.007	0.004	1.17E-01	0.010	0.007	1.18E-01
2.028816002	0.009	0.007	1.56E-01	0.006	0.010	5.56E-01
4.310165	-0.017	0.014	2.02E-01	0.019	0.020	3.53E-01
6.581758	0.006	0.008	4.35E-01	0.003	0.012	7.88E-01
4.311848	-0.017	0.013	1.88E-01	0.016	0.020	4.07E-01
7.679874	0.010	0.009	2.62E-01	-0.002	0.014	9.08E-01
8.195962	-0.011	0.009	2.03E-01	-0.011	0.013	3.90E-01
6.063316	0.008	0.007	2.51E-01	-0.006	0.010	5.49E-01
1.069310252	0.007	0.006	3.11E-01	-0.001	0.010	9.23E-01
6.020589	0.011	0.009	2.32E-01	-0.018	0.014	1.73E-01
2.168435527	0.013	0.011	2.07E-01	-0.012	0.016	4.38E-01
4.173237	-0.017	0.014	2.23E-01	-0.002	0.021	9.33E-01
1.710887203	-0.005	0.004	2.51E-01	0.003	0.006	6.54E-01
2.985967	-0.005	0.004	2.33E-01	0.002	0.006	7.57E-01
5.686175	0.009	0.008	2.62E-01	-0.013	0.013	2.99E-01
1.800378273	-0.009	0.010	3.87E-01	-0.007	0.015	6.30E-01
8.723488	0.008	0.010	4.27E-01	-0.023	0.015	1.24E-01
5.017683	0.007	0.006	2.12E-01	-0.006	0.008	4.95E-01
2.869225	-0.010	0.009	2.73E-01	0.006	0.013	6.82E-01
2.609498654	-0.015	0.013	2.49E-01	0.018	0.019	3.56E-01
0.851638366	-0.009	0.007	1.78E-01	-0.013	0.010	1.98E-01
3.571023	-0.006	0.006	3.15E-01	0.010	0.009	2.73E-01
1.136260338	0.010	0.007	1.25E-01	-0.005	0.010	6.12E-01
8.390756	0.008	0.009	3.30E-01	0.000	0.013	9.76E-01
8.11219	-0.009	0.007	2.21E-01	-0.013	0.011	2.31E-01

8.56772	0.009	0.008	2.85E-01	0.022	0.012	7.84E-02
2.022423783	0.013	0.009	1.64E-01	0.001	0.014	9.62E-01
0.7301862	-0.010	0.007	1.80E-01	-0.014	0.011	2.07E-01
8.915591	-0.007	0.009	4.38E-01	-0.027	0.013	3.81E-02
2.637422559	-0.011	0.009	2.32E-01	-0.004	0.013	7.57E-01
2.080626621	-0.004	0.004	3.71E-01	0.006	0.006	3.33E-01
3.606348	0.009	0.009	3.12E-01	-0.021	0.014	1.19E-01
6.432719	0.011	0.009	1.93E-01	0.005	0.013	6.88E-01
8.849314	0.007	0.008	3.65E-01	-0.002	0.011	8.76E-01
6.984468	-0.011	0.010	2.71E-01	0.007	0.015	6.42E-01
3.704923	0.008	0.006	1.50E-01	0.024	0.009	5.58E-03
3.161921	-0.010	0.007	1.65E-01	0.000	0.011	9.88E-01
5.582217	0.012	0.008	1.11E-01	0.011	0.011	3.43E-01
6.76545	0.010	0.008	2.14E-01	-0.009	0.012	4.62E-01
7.316527	-0.008	0.006	1.88E-01	0.000	0.009	9.56E-01
4.4333	-0.016	0.016	3.00E-01	0.012	0.023	6.10E-01
7.806036	-0.012	0.009	1.76E-01	-0.034	0.013	9.91E-03
5.049308	0.012	0.010	2.35E-01	0.004	0.015	7.83E-01
7.778785	-0.009	0.008	2.78E-01	-0.001	0.012	9.62E-01
7.691986	0.011	0.008	1.49E-01	0.012	0.012	2.86E-01
5.842616	0.010	0.008	1.90E-01	0.009	0.012	4.30E-01
3.201284	0.007	0.006	2.58E-01	0.019	0.009	2.63E-02
5.005908	0.010	0.009	2.52E-01	-0.001	0.013	9.13E-01
5.281783	-0.011	0.007	1.39E-01	-0.025	0.011	2.32E-02
3.550164	0.015	0.016	3.29E-01	0.059	0.023	1.09E-02
4.409077	-0.014	0.012	2.52E-01	0.014	0.018	4.49E-01
7.39357	0.008	0.007	2.50E-01	-0.018	0.010	6.63E-02
7.134853	0.010	0.009	2.66E-01	-0.022	0.013	1.02E-01
8.080902	0.010	0.009	2.58E-01	0.006	0.013	6.31E-01
2.601760705	-0.019	0.015	2.06E-01	0.000	0.022	9.93E-01
7.719573	-0.013	0.011	2.44E-01	-0.019	0.016	2.32E-01
1.908036702	-0.008	0.006	1.40E-01	-0.010	0.008	2.31E-01
7.707462	-0.013	0.011	2.57E-01	-0.013	0.017	4.37E-01
4.339435	-0.016	0.014	2.67E-01	0.004	0.021	8.50E-01
3.597938	0.009	0.007	1.97E-01	0.012	0.011	2.68E-01
4.315548	-0.016	0.012	1.87E-01	0.009	0.018	6.11E-01
6.55047	-0.012	0.008	1.56E-01	-0.022	0.012	7.17E-02
1.482785907	-0.006	0.005	2.97E-01	0.003	0.008	7.22E-01
3.548482	0.016	0.012	1.72E-01	0.027	0.017	1.18E-01
1.059217274	0.006	0.006	3.54E-01	-0.007	0.010	4.35E-01
6.763768	0.014	0.009	1.43E-01	-0.011	0.014	4.15E-01
6.236915	0.009	0.007	1.95E-01	0.008	0.011	4.79E-01
6.682352	0.008	0.008	3.23E-01	-0.007	0.012	5.55E-01

3.66186	-0.007	0.005	1.59E-01	0.007	0.007	3.30E-01
3.570687	-0.007	0.006	2.97E-01	0.010	0.010	3.18E-01
8.479575	-0.008	0.007	2.74E-01	0.002	0.011	8.76E-01
2.610171519	-0.018	0.014	1.83E-01	0.009	0.020	6.62E-01
5.502146	0.009	0.009	2.86E-01	0.000	0.013	9.70E-01
2.49342941	-0.022	0.017	1.95E-01	0.014	0.026	5.83E-01
7.325611	-0.010	0.007	1.50E-01	-0.008	0.011	4.77E-01
1.966575973	0.005	0.004	2.25E-01	-0.001	0.007	8.33E-01
6.507743	0.011	0.010	2.69E-01	0.033	0.015	2.33E-02
6.735172	-0.015	0.009	1.12E-01	0.010	0.014	4.75E-01
8.690181	0.005	0.006	3.68E-01	0.012	0.008	1.53E-01
5.806281	0.010	0.008	2.17E-01	-0.006	0.012	6.30E-01
6.225476	0.012	0.009	1.82E-01	0.012	0.014	3.90E-01
4.448776	-0.018	0.017	2.84E-01	0.022	0.025	3.73E-01
3.988872	-0.009	0.007	2.19E-01	0.008	0.010	4.45E-01
1.177641546	0.015	0.010	1.37E-01	-0.010	0.015	4.80E-01
6.255419	0.012	0.009	1.79E-01	-0.001	0.014	9.50E-01
0.974099829	0.005	0.006	3.32E-01	0.019	0.008	2.29E-02
6.375189	0.013	0.009	1.58E-01	0.000	0.014	9.78E-01
3.163603	-0.010	0.007	1.64E-01	0.007	0.010	5.13E-01
5.75548	0.008	0.008	3.54E-01	0.004	0.012	7.54E-01
8.781691	0.013	0.008	1.32E-01	0.010	0.012	4.26E-01
5.311052	0.021	0.016	2.07E-01	-0.022	0.024	3.69E-01
1.929231955	0.006	0.004	2.02E-01	0.003	0.007	6.43E-01
0.632957181	0.010	0.009	2.50E-01	0.003	0.013	8.10E-01
5.549583	0.008	0.008	2.90E-01	0.002	0.012	8.96E-01
2.485018596	-0.021	0.011	6.69E-02	-0.008	0.017	6.54E-01
4.320258	-0.016	0.014	2.57E-01	0.021	0.021	3.14E-01
5.273372	-0.010	0.006	1.11E-01	-0.020	0.010	3.30E-02
8.593962	-0.011	0.009	2.34E-01	-0.002	0.014	8.93E-01
6.968319	-0.014	0.009	1.43E-01	-0.003	0.014	8.29E-01
6.459633	0.010	0.009	2.61E-01	0.002	0.013	8.65E-01
2.796555175	-0.015	0.012	2.23E-01	-0.064	0.018	5.41E-04
7.483398	-0.009	0.008	2.54E-01	-0.024	0.012	3.82E-02
7.284566	-0.018	0.010	6.56E-02	0.017	0.014	2.36E-01
5.743368	0.010	0.010	3.19E-01	-0.005	0.016	7.30E-01
3.856991	0.005	0.006	3.43E-01	0.017	0.009	4.91E-02
5.91394	-0.010	0.008	2.23E-01	-0.010	0.012	4.10E-01
5.279091	-0.011	0.007	1.28E-01	-0.024	0.010	1.74E-02
2.025115243	0.011	0.008	1.67E-01	0.003	0.012	8.16E-01
4.425898	-0.016	0.015	2.80E-01	0.006	0.022	7.75E-01
2.30771862	-0.008	0.006	2.04E-01	-0.002	0.009	7.87E-01
5.900482	0.011	0.008	2.10E-01	-0.003	0.013	8.41E-01

8.79212	-0.011	0.009	2.07E-01	-0.006	0.013	6.67E-01
6.990524	0.008	0.007	2.36E-01	0.009	0.010	3.61E-01
5.706024	0.009	0.008	2.72E-01	-0.007	0.012	5.53E-01
0.518233668	0.014	0.010	1.45E-01	-0.002	0.014	8.76E-01
2.144885246	-0.018	0.014	1.92E-01	-0.002	0.021	9.24E-01
0.918588451	-0.005	0.005	2.59E-01	-0.022	0.007	1.32E-03
7.019457	0.005	0.009	5.59E-01	-0.023	0.013	7.49E-02
3.439814	0.009	0.007	1.97E-01	0.023	0.011	3.52E-02
5.030468	0.011	0.010	2.53E-01	-0.003	0.014	8.20E-01
7.352862	-0.014	0.010	1.54E-01	-0.014	0.015	3.36E-01
4.426235	-0.016	0.015	2.67E-01	0.007	0.022	7.52E-01
6.028327	0.011	0.008	1.51E-01	0.006	0.012	6.44E-01
5.064784	0.008	0.008	2.92E-01	0.001	0.012	9.37E-01
5.40088	0.007	0.006	2.66E-01	-0.025	0.009	8.17E-03
8.842922	0.011	0.008	1.51E-01	-0.002	0.012	8.94E-01
3.535025	0.011	0.008	1.77E-01	0.025	0.012	4.12E-02
5.989637	0.010	0.010	3.36E-01	0.004	0.015	8.01E-01
0.546157573	-0.008	0.009	3.68E-01	0.001	0.013	9.16E-01
4.446084	-0.018	0.016	2.85E-01	0.020	0.025	4.12E-01
3.662196	-0.006	0.005	1.68E-01	0.009	0.007	2.11E-01
1.779855885	-0.012	0.010	1.95E-01	0.011	0.014	4.34E-01
8.075855	-0.009	0.010	3.62E-01	0.000	0.014	9.82E-01
3.983826	-0.010	0.009	2.54E-01	-0.005	0.013	6.81E-01
6.308239	0.013	0.010	1.70E-01	-0.012	0.014	3.95E-01
3.989209	-0.008	0.007	2.35E-01	0.007	0.011	5.16E-01
5.315762	0.021	0.017	2.13E-01	-0.026	0.025	3.00E-01
2.610507952	-0.018	0.014	1.82E-01	0.012	0.020	5.47E-01
8.949907	0.005	0.006	4.00E-01	-0.015	0.009	1.05E-01
5.282119	-0.011	0.007	1.45E-01	-0.025	0.011	2.29E-02
5.283128	-0.011	0.008	1.50E-01	-0.025	0.011	2.46E-02
2.339343284	0.015	0.008	7.32E-02	0.014	0.012	2.57E-01
2.578546856	-0.008	0.010	3.89E-01	0.005	0.015	7.41E-01
2.17920137	0.016	0.011	1.70E-01	-0.010	0.017	5.49E-01
8.795485	-0.007	0.009	4.32E-01	-0.010	0.014	4.44E-01
7.865585	-0.013	0.011	2.22E-01	-0.026	0.016	1.11E-01
5.509548	0.006	0.007	3.78E-01	-0.001	0.011	9.32E-01
5.278082	-0.010	0.007	1.25E-01	-0.024	0.010	1.82E-02
5.153602	0.010	0.008	2.18E-01	-0.009	0.012	4.57E-01
2.982939	-0.005	0.004	2.42E-01	-0.001	0.006	8.66E-01
1.032975532	0.006	0.005	2.44E-01	0.012	0.008	1.25E-01
5.260587	-0.010	0.005	7.57E-02	-0.011	0.008	1.77E-01
6.024626	0.012	0.010	2.12E-01	-0.013	0.015	3.58E-01
4.181985	-0.012	0.012	2.99E-01	-0.001	0.017	9.37E-01

6.512117	0.008	0.011	4.64E-01	0.018	0.016	2.53E-01
4.346164	-0.016	0.015	2.88E-01	0.023	0.022	2.85E-01
7.711162	-0.011	0.010	2.29E-01	-0.009	0.014	5.30E-01
1.895252263	-0.009	0.007	1.74E-01	-0.006	0.010	5.77E-01
4.225721	0.013	0.012	2.90E-01	0.024	0.018	1.84E-01
5.894763	0.013	0.010	2.09E-01	-0.008	0.015	6.17E-01
5.107847	0.009	0.008	2.53E-01	-0.016	0.012	1.82E-01
1.073011011	0.007	0.006	2.36E-01	0.000	0.009	9.71E-01
7.014074	0.010	0.010	3.02E-01	-0.006	0.014	6.57E-01
4.41917	-0.016	0.014	2.60E-01	0.007	0.022	7.57E-01
6.263493	0.010	0.007	1.67E-01	0.010	0.011	3.69E-01
3.875158	-0.005	0.004	2.40E-01	0.012	0.007	8.24E-02
0.956605334	-0.009	0.006	1.40E-01	0.009	0.009	3.09E-01
5.867512	0.010	0.009	2.29E-01	0.017	0.013	1.91E-01
2.604115733	-0.018	0.014	2.07E-01	0.023	0.021	2.70E-01
3.58078	-0.006	0.006	3.30E-01	0.007	0.009	4.37E-01
5.584236	0.009	0.008	2.56E-01	0.014	0.012	2.66E-01
2.555333007	-0.013	0.011	2.60E-01	-0.009	0.017	5.77E-01
0.632620749	0.007	0.008	3.40E-01	0.004	0.012	7.54E-01
1.005051627	0.010	0.009	2.21E-01	0.008	0.013	5.09E-01
7.529489	-0.012	0.009	1.78E-01	-0.007	0.013	5.84E-01
8.256183	-0.008	0.010	3.94E-01	-0.023	0.014	1.17E-01
1.221377783	0.008	0.007	2.67E-01	0.001	0.011	9.15E-01
7.559768	0.008	0.010	3.96E-01	0.014	0.014	3.43E-01
5.47254	0.008	0.008	2.96E-01	-0.003	0.012	8.11E-01
8.203027	0.009	0.007	2.20E-01	-0.013	0.011	2.32E-01
7.016093	0.007	0.009	4.14E-01	0.007	0.013	6.02E-01
3.054599	-0.009	0.009	3.03E-01	0.010	0.013	4.54E-01
2.654917054	-0.012	0.010	1.99E-01	0.007	0.014	6.26E-01
2.418741375	-0.010	0.014	4.59E-01	0.008	0.021	6.87E-01
8.971775	0.010	0.009	2.91E-01	-0.017	0.013	1.96E-01
8.275696	0.007	0.007	2.99E-01	0.002	0.011	8.47E-01
8.013615	-0.012	0.009	1.74E-01	-0.007	0.013	5.94E-01
5.283465	-0.011	0.008	1.51E-01	-0.026	0.011	2.32E-02
1.967248838	0.005	0.004	2.22E-01	-0.001	0.007	8.48E-01
1.725690237	-0.006	0.005	1.91E-01	0.009	0.007	2.23E-01
6.889594	0.013	0.008	1.08E-01	-0.003	0.013	8.39E-01
4.172564	-0.016	0.014	2.33E-01	0.006	0.020	7.58E-01
8.31876	0.010	0.009	2.65E-01	-0.002	0.014	8.99E-01
3.266552	-0.008	0.007	2.73E-01	-0.008	0.011	4.80E-01
2.79722804	-0.016	0.013	2.20E-01	-0.068	0.019	3.22E-04
5.940181	0.006	0.007	3.86E-01	0.008	0.011	4.81E-01
7.738414	-0.010	0.011	3.44E-01	0.009	0.017	5.89E-01

8.48563	0.012	0.010	1.99E-01	0.002	0.014	9.14E-01
3.212049	-0.021	0.015	1.67E-01	-0.009	0.023	6.94E-01
1.706177147	-0.005	0.005	2.63E-01	-0.003	0.007	7.09E-01
4.430272	-0.018	0.015	2.34E-01	0.008	0.023	7.30E-01
5.129715	0.010	0.008	2.21E-01	-0.006	0.013	6.60E-01
7.612588	-0.019	0.013	1.66E-01	-0.046	0.020	2.11E-02
4.129501	-0.015	0.014	2.77E-01	0.015	0.021	4.80E-01
4.395283	-0.017	0.014	2.37E-01	0.007	0.021	7.35E-01
3.677672	-0.005	0.005	3.07E-01	0.005	0.007	4.89E-01
8.240371	-0.006	0.006	3.06E-01	-0.005	0.009	5.98E-01
8.968411	0.009	0.007	2.20E-01	0.023	0.011	3.34E-02
1.909718865	-0.011	0.008	1.38E-01	-0.027	0.011	1.61E-02
6.421616	-0.011	0.008	1.68E-01	-0.024	0.012	4.55E-02
1.840077318	-0.011	0.010	2.72E-01	-0.008	0.015	5.75E-01
3.790713	0.010	0.006	7.39E-02	0.013	0.008	1.07E-01
5.428804	0.008	0.006	2.04E-01	-0.001	0.009	8.76E-01
0.910514069	-0.006	0.005	2.79E-01	-0.026	0.008	1.41E-03
6.103015	0.013	0.009	1.62E-01	-0.007	0.013	6.10E-01
4.383171	-0.013	0.012	2.56E-01	0.004	0.018	8.19E-01
1.852525324	-0.006	0.008	4.46E-01	0.002	0.012	8.44E-01
7.903265	-0.013	0.010	1.75E-01	-0.040	0.015	5.92E-03
7.071941	-0.010	0.008	1.78E-01	-0.013	0.011	2.65E-01
6.423299	-0.012	0.009	1.77E-01	-0.019	0.014	1.63E-01
5.082951	0.015	0.012	2.07E-01	-0.006	0.018	7.51E-01
5.405926	0.007	0.007	3.20E-01	0.005	0.010	6.21E-01
5.485661	0.010	0.010	2.79E-01	0.009	0.014	5.43E-01
6.695136	0.010	0.009	2.83E-01	0.019	0.013	1.60E-01
1.697766332	-0.004	0.004	3.26E-01	0.003	0.006	5.97E-01
8.775635	0.005	0.009	5.44E-01	-0.016	0.013	2.21E-01
1.916447517	-0.008	0.006	1.82E-01	0.002	0.008	8.21E-01
6.655437	-0.008	0.008	3.13E-01	0.010	0.012	4.18E-01
6.011169	-0.010	0.011	3.56E-01	0.002	0.016	8.92E-01
6.767133	-0.009	0.009	3.28E-01	0.000	0.014	9.91E-01
5.248139	-0.009	0.005	6.11E-02	-0.010	0.007	1.94E-01
5.960704	0.012	0.009	1.95E-01	0.006	0.014	6.50E-01
7.578944	-0.010	0.009	2.59E-01	-0.021	0.013	1.11E-01
3.050562	-0.013	0.009	1.53E-01	0.012	0.013	3.65E-01
7.835306	-0.011	0.010	2.76E-01	0.014	0.015	3.49E-01
4.001993	0.010	0.009	2.85E-01	0.015	0.014	2.67E-01
1.812489846	-0.010	0.010	3.34E-01	0.005	0.015	7.61E-01
5.310716	0.020	0.016	2.13E-01	-0.021	0.024	3.86E-01
8.038175	0.009	0.007	1.97E-01	0.016	0.011	1.33E-01
2.308391485	-0.009	0.007	2.07E-01	0.001	0.011	9.02E-01

7.758599	-0.018	0.011	1.00E-01	-0.009	0.017	5.85E-01
8.555608	0.008	0.007	2.61E-01	-0.006	0.010	5.73E-01
1.634180573	0.010	0.007	1.40E-01	0.009	0.010	3.76E-01
7.005663	-0.010	0.010	3.21E-01	-0.021	0.014	1.41E-01
6.663175	0.009	0.009	2.98E-01	0.011	0.013	4.27E-01
6.645344	-0.008	0.007	2.25E-01	-0.011	0.010	3.04E-01
6.381917	-0.012	0.010	2.41E-01	0.003	0.015	8.27E-01
5.82243	0.007	0.010	4.72E-01	-0.006	0.015	6.64E-01
7.97829	0.009	0.009	3.11E-01	0.036	0.014	8.11E-03
6.394365	0.008	0.008	3.31E-01	0.006	0.013	6.11E-01
6.957553	-0.011	0.010	2.82E-01	0.013	0.015	3.62E-01
8.901461	-0.010	0.009	3.03E-01	-0.028	0.014	5.11E-02
4.315212	-0.017	0.014	2.23E-01	0.014	0.021	5.24E-01
5.527715	0.010	0.008	2.20E-01	0.002	0.012	8.88E-01
6.394029	0.009	0.008	2.98E-01	0.006	0.012	6.13E-01
6.539368	0.009	0.010	3.31E-01	-0.002	0.014	8.65E-01
3.976761	-0.009	0.009	3.08E-01	-0.004	0.013	7.33E-01
5.277073	-0.010	0.007	1.28E-01	-0.022	0.010	2.69E-02
8.463426	-0.010	0.008	2.26E-01	-0.025	0.012	4.60E-02
5.282792	-0.011	0.007	1.53E-01	-0.025	0.011	2.49E-02
8.296219	0.011	0.009	1.96E-01	-0.001	0.013	9.09E-01
5.326865	0.023	0.019	2.19E-01	-0.024	0.028	3.98E-01
5.529061	0.009	0.007	2.36E-01	0.009	0.011	4.18E-01
8.081238	0.009	0.008	2.83E-01	-0.006	0.013	6.15E-01
7.580963	0.008	0.007	2.45E-01	0.002	0.010	8.70E-01
6.312276	0.008	0.008	2.88E-01	-0.006	0.012	6.05E-01
8.902807	0.008	0.008	2.80E-01	0.004	0.011	7.50E-01
1.446451187	0.005	0.004	1.81E-01	0.006	0.006	3.25E-01
4.309493	-0.015	0.013	2.45E-01	0.013	0.020	5.11E-01
1.654702961	0.005	0.004	3.03E-01	0.017	0.007	1.18E-02
4.407058	-0.016	0.015	2.80E-01	0.009	0.022	6.94E-01
5.869194	0.010	0.008	2.09E-01	0.002	0.012	8.63E-01
6.949143	-0.012	0.009	1.81E-01	-0.006	0.013	6.43E-01
7.942965	0.009	0.008	2.91E-01	0.003	0.012	7.75E-01
6.470399	0.008	0.008	2.93E-01	-0.002	0.011	8.81E-01
7.199112	-0.013	0.010	2.07E-01	0.010	0.015	5.03E-01
3.197583	0.006	0.005	2.95E-01	0.017	0.008	3.71E-02
0.742970639	-0.008	0.006	2.07E-01	-0.012	0.009	1.97E-01
8.61482	0.008	0.007	2.77E-01	-0.013	0.011	2.36E-01
1.170912895	0.008	0.005	1.22E-01	-0.011	0.007	1.31E-01
5.092035	0.010	0.009	2.42E-01	0.004	0.013	7.38E-01
2.358519941	0.011	0.008	1.79E-01	0.014	0.012	2.42E-01
8.606746	0.008	0.009	3.75E-01	-0.005	0.013	6.87E-01

1.329036212	0.016	0.009	7.61E-02	0.007	0.014	5.98E-01
7.525115	-0.007	0.006	2.80E-01	-0.021	0.009	2.32E-02
7.89149	0.012	0.010	1.91E-01	0.019	0.014	1.82E-01
5.945228	0.010	0.008	1.98E-01	-0.011	0.011	3.37E-01
6.179385	-0.010	0.007	1.54E-01	-0.030	0.010	3.76E-03
4.14565	-0.012	0.012	3.11E-01	0.011	0.018	5.41E-01
3.606012	0.010	0.010	3.11E-01	-0.024	0.015	1.12E-01
5.955994	0.009	0.007	1.98E-01	0.004	0.011	7.00E-01
5.165713	0.013	0.010	1.82E-01	-0.012	0.015	4.10E-01
4.335734	-0.012	0.012	3.02E-01	0.008	0.018	6.63E-01
5.29995	0.018	0.014	2.03E-01	-0.018	0.021	4.01E-01
8.857725	0.012	0.010	2.60E-01	0.004	0.015	7.83E-01
2.774350624	-0.014	0.011	1.96E-01	-0.048	0.016	2.68E-03
5.282456	-0.011	0.007	1.52E-01	-0.025	0.011	2.38E-02
6.231868	0.008	0.008	3.35E-01	-0.006	0.013	6.47E-01
3.726118	0.007	0.008	4.17E-01	0.017	0.012	1.45E-01
5.435196	0.010	0.008	2.16E-01	-0.008	0.012	5.20E-01
0.549858331	-0.008	0.009	3.52E-01	-0.018	0.014	1.90E-01
5.554293	0.010	0.008	2.37E-01	-0.018	0.012	1.36E-01
3.237955	-0.008	0.006	1.93E-01	0.005	0.009	6.17E-01
1.532241497	0.013	0.010	2.02E-01	-0.021	0.015	1.67E-01
1.720307316	-0.009	0.006	1.42E-01	-0.003	0.009	6.90E-01
5.139135	0.008	0.008	2.87E-01	-0.010	0.011	3.56E-01
7.581299	0.012	0.009	1.91E-01	0.010	0.014	4.80E-01
1.520466357	0.011	0.009	2.26E-01	-0.013	0.013	3.20E-01
7.303406	-0.015	0.009	1.09E-01	0.000	0.014	9.84E-01
1.530895767	0.013	0.010	1.95E-01	-0.017	0.015	2.49E-01
4.43801	-0.017	0.016	2.69E-01	0.021	0.024	3.64E-01
4.379471	-0.015	0.013	2.57E-01	0.004	0.020	8.53E-01
6.155162	0.007	0.008	3.44E-01	-0.012	0.011	3.02E-01
5.713426	0.008	0.008	3.42E-01	0.018	0.013	1.59E-01
7.008355	-0.014	0.010	1.34E-01	-0.020	0.014	1.55E-01
6.773188	-0.005	0.007	4.64E-01	0.005	0.010	6.24E-01
5.931434	0.007	0.007	3.35E-01	0.003	0.011	8.03E-01
5.279428	-0.010	0.007	1.36E-01	-0.025	0.010	1.66E-02
6.881856	0.012	0.010	2.26E-01	0.006	0.015	6.77E-01
6.64198	0.008	0.010	4.38E-01	-0.011	0.015	4.64E-01
3.793405	0.009	0.006	1.44E-01	0.014	0.009	1.31E-01
0.836498899	-0.012	0.009	1.50E-01	-0.010	0.013	4.40E-01
1.429629557	0.006	0.004	1.60E-01	0.002	0.007	7.53E-01
1.875739173	-0.008	0.007	2.34E-01	-0.004	0.010	6.86E-01
5.695258	0.009	0.008	2.73E-01	-0.006	0.012	6.24E-01
6.723396	-0.013	0.010	2.07E-01	0.004	0.015	7.77E-01

5.529734	0.008	0.008	3.44E-01	-0.003	0.012	7.91E-01
7.984346	-0.010	0.009	3.04E-01	-0.009	0.014	5.24E-01
8.465444	-0.006	0.005	2.83E-01	-0.010	0.008	2.17E-01
2.570472474	-0.008	0.010	4.35E-01	-0.001	0.015	9.59E-01
6.001076	0.010	0.009	2.47E-01	0.009	0.013	4.86E-01
5.812	0.010	0.010	3.29E-01	-0.008	0.016	6.29E-01
1.986089063	0.010	0.007	1.72E-01	-0.006	0.011	5.96E-01
7.03426	-0.017	0.010	9.82E-02	-0.024	0.015	1.18E-01
6.902042	0.009	0.008	2.44E-01	0.010	0.012	4.10E-01
5.823103	0.011	0.009	2.24E-01	0.026	0.014	6.05E-02
7.382131	-0.009	0.009	2.86E-01	-0.009	0.013	4.66E-01
5.275391	-0.010	0.007	1.19E-01	-0.022	0.010	2.33E-02
0.910850502	-0.006	0.005	2.91E-01	-0.026	0.008	8.67E-04
7.190701	0.011	0.010	3.06E-01	-0.012	0.016	4.28E-01
1.239208711	0.008	0.007	2.56E-01	-0.006	0.011	5.71E-01
1.157455591	0.007	0.005	1.50E-01	-0.004	0.007	5.93E-01
2.18828505	0.014	0.011	1.98E-01	-0.020	0.017	2.31E-01
2.293252019	-0.004	0.005	3.74E-01	-0.012	0.007	7.27E-02
6.575366	0.011	0.009	2.16E-01	-0.009	0.013	5.16E-01
1.906354539	-0.008	0.006	1.90E-01	-0.002	0.009	8.08E-01
4.384853	-0.016	0.014	2.53E-01	0.003	0.020	8.69E-01
8.833165	0.009	0.008	2.58E-01	-0.023	0.012	5.35E-02
3.040132	-0.007	0.006	2.36E-01	0.009	0.009	3.09E-01
2.064814289	0.006	0.004	1.22E-01	0.010	0.005	7.16E-02
5.462111	0.011	0.010	3.00E-01	0.012	0.016	4.46E-01
0.712691705	0.007	0.008	3.20E-01	0.017	0.011	1.23E-01
5.12736	0.008	0.007	3.10E-01	-0.002	0.011	8.38E-01
8.670332	-0.008	0.009	3.46E-01	-0.016	0.013	2.33E-01
0.717401762	0.008	0.008	2.65E-01	0.016	0.011	1.51E-01
5.771965	0.007	0.009	3.99E-01	-0.001	0.013	9.19E-01
5.449326	0.010	0.008	2.11E-01	0.007	0.012	5.48E-01
0.626901395	0.010	0.008	2.00E-01	0.011	0.012	3.42E-01
6.932321	0.005	0.008	4.86E-01	0.013	0.012	2.74E-01
2.553650844	-0.012	0.011	2.50E-01	-0.002	0.016	9.08E-01
7.185655	0.013	0.009	1.41E-01	-0.006	0.013	6.25E-01
2.024778811	0.011	0.008	1.73E-01	0.002	0.013	8.58E-01
1.045087105	0.005	0.005	3.38E-01	0.010	0.008	2.01E-01
5.246794	-0.009	0.005	6.29E-02	-0.005	0.007	5.11E-01
1.654366528	0.005	0.005	3.14E-01	0.019	0.007	6.34E-03
0.704617323	0.016	0.010	1.21E-01	0.003	0.015	8.30E-01
2.268019574	0.008	0.007	2.49E-01	0.012	0.010	2.49E-01
4.35121	-0.016	0.014	2.53E-01	0.010	0.021	6.33E-01
4.325641	-0.015	0.012	2.28E-01	0.012	0.018	5.06E-01

5.63571	-0.011	0.009	2.30E-01	-0.031	0.014	2.55E-02
3.597265	0.009	0.007	1.81E-01	0.022	0.010	3.08E-02
7.357572	-0.011	0.010	2.87E-01	-0.002	0.015	9.18E-01
4.315885	-0.017	0.013	2.09E-01	0.011	0.020	5.92E-01
4.41816	-0.015	0.014	2.85E-01	0.010	0.021	6.26E-01
6.564937	0.009	0.008	2.62E-01	0.000	0.012	9.78E-01
6.596561	-0.012	0.010	2.26E-01	0.017	0.015	2.67E-01
1.169230732	0.008	0.005	8.83E-02	-0.003	0.007	7.09E-01
6.385618	-0.005	0.008	5.49E-01	0.003	0.012	8.19E-01
2.144212381	-0.017	0.013	2.05E-01	0.003	0.020	8.66E-01
2.527409102	-0.012	0.010	2.38E-01	0.004	0.016	8.15E-01
7.950366	-0.009	0.010	3.43E-01	-0.018	0.014	2.13E-01
7.801999	-0.009	0.009	2.76E-01	-0.018	0.013	1.48E-01
1.141643259	0.010	0.006	1.13E-01	-0.005	0.010	5.74E-01
2.154978224	-0.008	0.008	3.01E-01	-0.016	0.012	1.79E-01
2.180210668	0.015	0.011	1.87E-01	-0.014	0.016	3.96E-01
2.774014191	-0.014	0.011	1.96E-01	-0.048	0.016	2.44E-03
6.95789	-0.010	0.009	2.74E-01	0.006	0.013	6.47E-01
2.350109126	0.016	0.009	8.71E-02	0.025	0.014	6.61E-02
1.917793247	-0.007	0.005	1.74E-01	-0.003	0.007	6.76E-01
8.956636	-0.010	0.009	2.58E-01	-0.015	0.014	2.58E-01
8.031446	0.007	0.008	4.00E-01	-0.001	0.012	9.33E-01
7.210214	-0.009	0.007	1.82E-01	-0.006	0.010	5.53E-01
8.363842	0.010	0.008	1.71E-01	0.002	0.011	8.52E-01
6.015879	0.010	0.009	2.66E-01	0.001	0.013	9.30E-01
7.850782	-0.009	0.010	3.63E-01	0.013	0.015	3.96E-01
8.837539	0.013	0.009	1.67E-01	0.011	0.014	4.16E-01
1.965566675	0.005	0.005	2.38E-01	-0.002	0.007	7.33E-01
4.335061	-0.015	0.014	2.89E-01	0.015	0.021	4.76E-01
6.598243	0.011	0.009	2.46E-01	-0.016	0.014	2.36E-01
3.561266	-0.009	0.008	2.50E-01	0.024	0.012	5.01E-02
2.40898483	-0.017	0.013	1.88E-01	0.011	0.019	5.58E-01
8.19798	0.010	0.008	1.70E-01	0.005	0.011	6.43E-01
6.141032	0.012	0.009	1.83E-01	-0.004	0.013	7.59E-01
7.276492	-0.014	0.009	1.27E-01	0.001	0.014	9.60E-01
7.753889	-0.009	0.010	4.12E-01	-0.008	0.015	5.84E-01
5.279764	-0.010	0.007	1.39E-01	-0.024	0.010	1.93E-02
2.871243	-0.009	0.010	3.76E-01	-0.002	0.015	8.67E-01
4.32766	-0.016	0.015	2.63E-01	0.030	0.022	1.67E-01
5.956667	-0.007	0.009	4.40E-01	-0.008	0.013	5.08E-01
7.709144	-0.010	0.010	3.00E-01	-0.019	0.015	2.18E-01
7.958777	0.011	0.008	1.79E-01	0.002	0.012	8.36E-01
1.007406655	0.008	0.007	2.52E-01	0.013	0.010	2.14E-01

5.660942	0.008	0.007	2.65E-01	-0.014	0.010	1.84E-01
1.46630071	-0.006	0.007	3.30E-01	0.006	0.010	5.49E-01
4.396629	-0.014	0.013	2.80E-01	0.010	0.019	5.87E-01
2.142193785	-0.015	0.013	2.58E-01	0.027	0.019	1.60E-01
6.347938	-0.008	0.008	3.20E-01	-0.017	0.013	1.67E-01
0.815976511	0.004	0.005	4.24E-01	0.016	0.008	4.37E-02
3.121549	-0.011	0.008	1.74E-01	-0.005	0.012	6.76E-01
8.280406	-0.013	0.009	1.86E-01	-0.005	0.014	7.01E-01
7.605523	-0.007	0.012	5.51E-01	0.002	0.018	9.20E-01
1.424246636	0.007	0.005	1.26E-01	0.011	0.007	1.03E-01
1.98575263	0.010	0.007	1.79E-01	-0.006	0.011	5.90E-01
5.273708	-0.010	0.006	1.20E-01	-0.021	0.010	2.96E-02
8.30934	0.006	0.010	5.04E-01	0.009	0.014	5.41E-01
3.900727	0.006	0.005	2.72E-01	0.025	0.008	2.38E-03
5.274718	-0.010	0.007	1.20E-01	-0.021	0.010	3.10E-02
2.607816491	-0.016	0.013	2.27E-01	0.017	0.019	3.82E-01
1.651002202	0.006	0.005	2.12E-01	0.002	0.007	8.12E-01
2.294261316	-0.005	0.005	3.11E-01	-0.008	0.007	2.63E-01
2.514961096	-0.016	0.014	2.69E-01	-0.025	0.021	2.45E-01
8.329526	-0.008	0.008	2.96E-01	-0.023	0.012	4.82E-02
5.008599	0.010	0.009	2.70E-01	-0.005	0.013	7.11E-01
6.214374	-0.010	0.010	3.00E-01	-0.012	0.014	3.89E-01
6.760068	0.014	0.010	1.84E-01	0.000	0.016	9.88E-01
1.117756545	0.009	0.007	2.01E-01	0.000	0.010	9.80E-01
3.874485	-0.005	0.005	2.57E-01	0.012	0.007	8.16E-02
3.19691	0.006	0.005	3.02E-01	0.017	0.008	4.27E-02
1.730063861	-0.008	0.005	1.37E-01	0.000	0.008	9.68E-01
5.001198	0.011	0.010	2.61E-01	0.002	0.015	9.16E-01
5.102801	0.010	0.009	2.93E-01	0.011	0.014	4.28E-01
3.890298	0.005	0.005	3.36E-01	0.024	0.008	2.43E-03
2.656599217	-0.011	0.008	2.03E-01	0.022	0.013	8.10E-02
5.821084	0.010	0.010	3.31E-01	0.012	0.015	4.21E-01
6.422962	-0.017	0.009	5.42E-02	-0.021	0.014	1.15E-01
6.675287	0.011	0.011	2.95E-01	0.008	0.016	6.25E-01
5.086315	0.009	0.008	2.49E-01	0.003	0.012	8.10E-01
7.536554	-0.010	0.009	2.49E-01	-0.021	0.013	1.25E-01
8.113536	0.010	0.010	2.83E-01	0.005	0.014	7.50E-01
3.619806	-0.006	0.006	2.97E-01	-0.009	0.009	3.11E-01
6.960245	-0.013	0.010	1.97E-01	-0.013	0.015	3.61E-01
6.555517	0.010	0.007	1.62E-01	-0.001	0.011	9.00E-01
5.125342	0.009	0.008	2.94E-01	-0.002	0.013	8.88E-01
7.137881	0.013	0.010	1.97E-01	-0.015	0.015	3.06E-01
3.678681	-0.004	0.005	3.34E-01	0.006	0.007	3.49E-01

3.122222	-0.012	0.008	1.67E-01	-0.005	0.013	6.77E-01
8.898097	0.009	0.008	2.18E-01	0.000	0.011	9.99E-01
4.418497	-0.015	0.014	2.98E-01	0.008	0.021	6.90E-01
4.383508	-0.014	0.013	2.77E-01	0.000	0.019	9.84E-01
5.04628	0.009	0.009	2.98E-01	-0.004	0.013	7.83E-01
5.809645	0.006	0.008	4.30E-01	0.000	0.012	9.77E-01
3.749332	0.010	0.009	2.83E-01	0.042	0.013	1.68E-03
8.340964	0.013	0.010	1.68E-01	0.006	0.014	6.62E-01
2.717829949	-0.010	0.006	1.18E-01	-0.017	0.010	8.63E-02
4.140603	-0.021	0.018	2.45E-01	0.018	0.027	5.11E-01
2.176509909	0.014	0.011	1.97E-01	-0.012	0.017	4.78E-01
6.238261	0.013	0.010	2.00E-01	-0.019	0.015	1.98E-01
5.810991	0.008	0.008	3.17E-01	-0.011	0.011	3.20E-01
1.730400294	-0.008	0.005	1.29E-01	-0.002	0.008	8.36E-01
1.625433325	0.008	0.006	1.84E-01	0.002	0.009	8.39E-01
5.789796	0.008	0.009	4.04E-01	-0.005	0.014	7.20E-01
5.587936	-0.008	0.009	3.36E-01	-0.020	0.013	1.26E-01
8.889686	0.011	0.008	1.65E-01	-0.001	0.012	9.39E-01
5.531752	0.009	0.008	2.53E-01	0.015	0.011	1.85E-01
4.329678	-0.015	0.012	2.40E-01	0.008	0.019	6.71E-01
1.707186445	-0.005	0.005	2.66E-01	-0.003	0.007	6.90E-01
4.300072	-0.014	0.014	3.02E-01	0.003	0.021	8.69E-01
5.310379	0.020	0.016	2.20E-01	-0.022	0.024	3.65E-01
1.035666993	0.006	0.005	2.22E-01	0.007	0.007	3.23E-01
3.277654	-0.014	0.012	2.31E-01	0.006	0.017	7.44E-01
7.205841	-0.007	0.006	2.68E-01	-0.011	0.009	2.55E-01
1.067964522	0.008	0.007	2.32E-01	-0.005	0.010	6.27E-01
2.535147052	-0.014	0.011	2.32E-01	0.000	0.017	9.78E-01
7.015083	0.007	0.010	4.51E-01	-0.003	0.014	8.60E-01
2.602770002	-0.016	0.013	2.12E-01	0.008	0.019	6.75E-01
0.688804991	0.021	0.016	1.69E-01	-0.002	0.023	9.21E-01
8.917946	-0.010	0.009	2.38E-01	-0.012	0.013	3.55E-01
6.557872	0.006	0.007	3.58E-01	-0.002	0.010	8.63E-01
2.041936873	0.007	0.005	1.74E-01	0.006	0.008	4.49E-01
1.317261072	0.016	0.009	6.98E-02	0.004	0.014	7.57E-01
5.027103	0.009	0.008	2.68E-01	-0.008	0.012	4.79E-01
8.596317	0.011	0.009	2.14E-01	0.014	0.014	3.10E-01
7.935899	-0.007	0.010	4.66E-01	0.003	0.015	8.34E-01
8.987588	0.005	0.007	4.90E-01	-0.003	0.010	7.70E-01
2.841973575	-0.009	0.008	2.62E-01	-0.008	0.012	5.13E-01
5.258232	-0.009	0.005	8.16E-02	-0.011	0.008	1.60E-01
2.16742623	0.014	0.011	1.94E-01	-0.015	0.016	3.34E-01
5.261597	-0.009	0.005	9.10E-02	-0.012	0.008	1.59E-01

7.732694	-0.011	0.011	3.28E-01	-0.001	0.017	9.55E-01
5.316099	0.020	0.017	2.25E-01	-0.027	0.025	2.79E-01
4.412777	-0.014	0.014	3.01E-01	0.017	0.020	3.99E-01
4.40874	-0.016	0.015	2.75E-01	0.013	0.022	5.51E-01
5.305333	0.019	0.015	2.06E-01	-0.021	0.022	3.28E-01
1.452170541	0.005	0.004	2.14E-01	-0.001	0.006	8.38E-01
2.793527282	-0.015	0.012	2.28E-01	-0.063	0.018	5.57E-04
5.438224	0.009	0.007	2.16E-01	0.015	0.010	1.45E-01
7.28322	-0.011	0.010	2.76E-01	0.002	0.016	9.19E-01
4.412441	-0.015	0.014	2.89E-01	0.018	0.021	3.95E-01
1.872038414	-0.009	0.007	2.51E-01	0.000	0.011	9.76E-01
7.367328	-0.010	0.008	2.32E-01	0.002	0.013	8.88E-01
5.247803	-0.009	0.005	6.25E-02	-0.009	0.007	2.03E-01
0.770558111	0.007	0.005	2.04E-01	-0.001	0.008	8.95E-01
3.108765	-0.016	0.010	1.13E-01	-0.009	0.015	5.53E-01
1.966912405	0.005	0.004	2.46E-01	-0.001	0.007	8.22E-01
6.952171	-0.009	0.009	3.05E-01	-0.003	0.013	8.37E-01
8.257529	-0.009	0.009	3.16E-01	-0.011	0.013	4.03E-01
8.769243	-0.007	0.009	4.06E-01	-0.004	0.013	7.62E-01
8.029428	0.010	0.008	2.08E-01	-0.002	0.012	8.74E-01
5.622589	0.010	0.009	2.89E-01	0.004	0.013	7.41E-01
5.455718	0.008	0.008	3.26E-01	0.004	0.012	7.24E-01
6.956544	-0.008	0.008	2.80E-01	0.002	0.012	8.62E-01
7.634792	-0.008	0.012	4.69E-01	0.019	0.017	2.76E-01
5.983581	0.011	0.009	2.65E-01	-0.016	0.014	2.43E-01
6.866044	-0.007	0.009	4.46E-01	0.012	0.014	3.91E-01
6.051204	0.008	0.007	2.64E-01	-0.001	0.011	9.47E-01
4.156752	-0.015	0.016	3.27E-01	0.012	0.023	6.05E-01
5.421402	0.008	0.008	3.19E-01	0.002	0.011	8.40E-01
5.993674	0.010	0.009	2.81E-01	-0.002	0.013	8.70E-01
4.443729	-0.016	0.015	2.92E-01	0.019	0.023	4.05E-01
5.657914	0.009	0.008	2.84E-01	-0.006	0.013	6.32E-01
2.685195987	-0.010	0.006	1.17E-01	-0.002	0.009	8.25E-01
1.094542696	0.007	0.007	3.26E-01	0.001	0.011	8.95E-01
5.46783	0.006	0.007	3.56E-01	-0.012	0.010	2.20E-01
6.527593	0.008	0.009	3.82E-01	-0.003	0.013	7.96E-01
0.773249572	0.006	0.005	2.14E-01	0.001	0.007	9.18E-01
8.842249	0.011	0.008	1.82E-01	-0.009	0.012	4.62E-01
0.736914852	-0.007	0.007	2.88E-01	-0.009	0.010	3.55E-01
7.348152	-0.013	0.011	2.41E-01	-0.019	0.017	2.77E-01
6.843166	0.007	0.007	2.98E-01	-0.010	0.011	3.36E-01
4.420852	-0.015	0.014	2.75E-01	0.010	0.020	6.37E-01
3.20162	0.006	0.006	2.80E-01	0.019	0.009	2.99E-02

1.70651358	-0.005	0.005	2.87E-01	-0.003	0.007	6.39E-01
2.603106435	-0.018	0.014	1.95E-01	0.011	0.021	6.17E-01
2.799583068	-0.016	0.014	2.53E-01	-0.066	0.021	1.49E-03
1.021873257	0.010	0.007	1.27E-01	0.003	0.010	7.46E-01
3.566313	-0.008	0.007	2.54E-01	0.020	0.011	6.75E-02
8.664276	0.007	0.008	3.53E-01	-0.008	0.011	4.66E-01
2.679813066	-0.009	0.006	1.06E-01	0.000	0.008	9.73E-01
3.197246	0.006	0.005	3.06E-01	0.017	0.008	3.99E-02
1.419873012	0.007	0.005	1.33E-01	0.003	0.007	6.83E-01
4.42287	-0.016	0.014	2.71E-01	0.013	0.021	5.43E-01
1.744866895	-0.007	0.006	2.50E-01	0.005	0.009	5.91E-01
2.022760215	0.013	0.009	1.80E-01	0.001	0.014	9.59E-01
3.156874	-0.010	0.008	2.26E-01	-0.008	0.012	5.09E-01
2.544567164	-0.039	0.028	1.53E-01	-0.033	0.041	4.20E-01
5.033495	0.009	0.009	3.32E-01	-0.002	0.013	8.70E-01
7.091117	-0.015	0.010	1.38E-01	-0.015	0.015	3.29E-01
7.884762	-0.008	0.009	3.55E-01	0.030	0.013	2.01E-02
7.959786	-0.010	0.010	3.41E-01	-0.031	0.015	4.29E-02
4.394946	-0.017	0.015	2.47E-01	0.015	0.022	4.88E-01
7.801663	-0.009	0.009	2.88E-01	-0.021	0.013	1.05E-01
2.845337901	-0.007	0.009	4.17E-01	0.000	0.013	9.78E-01
4.312184	-0.016	0.013	2.17E-01	0.018	0.019	3.39E-01
5.075886	0.009	0.008	2.56E-01	0.004	0.012	7.42E-01
2.59334989	-0.015	0.014	2.77E-01	0.006	0.021	7.86E-01
4.152042	-0.020	0.017	2.32E-01	0.015	0.025	5.54E-01
6.898005	0.010	0.008	2.25E-01	-0.004	0.012	7.44E-01
4.242206	0.017	0.014	2.20E-01	-0.007	0.021	7.27E-01
1.625096893	0.008	0.006	1.88E-01	0.003	0.009	7.68E-01
8.690518	0.007	0.007	2.78E-01	0.006	0.010	5.15E-01
2.029152435	0.009	0.006	1.68E-01	0.006	0.009	5.43E-01
3.874822	-0.005	0.004	2.63E-01	0.011	0.007	8.99E-02
5.274381	-0.010	0.006	1.23E-01	-0.021	0.010	3.05E-02
2.986976	-0.005	0.004	2.43E-01	0.004	0.006	4.82E-01
8.90247	0.011	0.010	2.59E-01	-0.002	0.014	8.77E-01
6.94376	0.010	0.008	2.49E-01	0.021	0.013	9.93E-02
5.672381	0.009	0.009	2.89E-01	0.006	0.013	6.62E-01
1.773463665	-0.010	0.009	2.72E-01	0.008	0.013	5.56E-01
5.766919	-0.010	0.009	2.76E-01	-0.005	0.013	7.10E-01
2.51159677	-0.009	0.014	4.89E-01	-0.019	0.020	3.57E-01
6.80986	0.005	0.011	6.67E-01	-0.008	0.017	6.21E-01
3.044506	-0.004	0.005	4.23E-01	-0.006	0.008	4.51E-01
5.519304	0.008	0.008	3.18E-01	-0.018	0.012	1.35E-01
1.627788353	0.006	0.006	2.57E-01	-0.002	0.009	7.74E-01

2.498475899	-0.019	0.013	1.57E-01	0.002	0.020	9.18E-01
5.574479	0.009	0.009	3.09E-01	-0.014	0.014	3.11E-01
2.2713839	0.008	0.006	2.22E-01	0.002	0.009	8.34E-01
6.964619	-0.014	0.010	1.71E-01	-0.011	0.015	4.86E-01
7.031569	-0.010	0.010	2.88E-01	-0.017	0.014	2.44E-01
0.965352581	0.007	0.005	1.98E-01	0.016	0.008	4.93E-02
4.429599	-0.016	0.014	2.65E-01	0.017	0.022	4.29E-01
4.419506	-0.016	0.015	2.77E-01	0.010	0.022	6.58E-01
8.413634	-0.008	0.008	3.20E-01	-0.010	0.012	3.85E-01
6.943423	0.010	0.009	2.58E-01	0.016	0.013	2.19E-01
5.888034	0.008	0.008	2.85E-01	-0.003	0.011	7.66E-01
2.545576462	-0.034	0.028	2.23E-01	-0.041	0.042	3.22E-01
5.65455	0.006	0.007	3.86E-01	0.018	0.011	8.54E-02
6.441802	-0.006	0.008	4.62E-01	-0.007	0.012	5.58E-01
8.175439	-0.013	0.008	8.44E-02	-0.011	0.011	3.19E-01
7.2267	-0.013	0.009	1.59E-01	-0.007	0.014	6.04E-01
8.906844	-0.010	0.008	2.11E-01	-0.010	0.012	4.13E-01
3.297503	-0.011	0.010	3.03E-01	-0.006	0.015	6.77E-01
3.313652	-0.017	0.012	1.37E-01	-0.005	0.017	7.91E-01
7.487435	0.011	0.010	2.63E-01	-0.016	0.015	2.66E-01
8.39513	-0.010	0.010	2.95E-01	-0.012	0.014	3.85E-01
4.224375	0.011	0.011	3.24E-01	0.010	0.017	5.61E-01
0.836162466	-0.012	0.009	1.58E-01	-0.009	0.013	4.66E-01
0.900757524	0.010	0.007	1.55E-01	-0.014	0.011	2.11E-01
1.407425006	0.008	0.006	1.44E-01	0.015	0.008	7.18E-02
5.003553	0.011	0.010	2.99E-01	0.006	0.015	6.93E-01
7.756581	-0.012	0.011	2.60E-01	-0.025	0.016	1.22E-01
8.537104	-0.008	0.007	2.77E-01	-0.005	0.011	6.38E-01
4.158098	-0.016	0.016	3.00E-01	0.010	0.023	6.73E-01
6.825335	0.009	0.009	2.99E-01	0.017	0.013	2.02E-01
7.070258	-0.008	0.009	3.77E-01	-0.018	0.013	1.80E-01
6.251045	0.008	0.007	2.60E-01	0.011	0.011	2.83E-01
8.937123	0.010	0.008	2.14E-01	0.014	0.012	2.61E-01
0.741961341	-0.007	0.006	2.50E-01	-0.012	0.009	2.08E-01
6.968992	-0.011	0.010	2.58E-01	-0.002	0.014	8.81E-01
6.973702	-0.011	0.009	1.98E-01	-0.010	0.013	4.56E-01
1.776491559	-0.009	0.011	4.05E-01	0.015	0.017	3.61E-01
4.104269	-0.009	0.012	4.36E-01	0.008	0.017	6.50E-01
8.827109	0.008	0.007	2.90E-01	0.003	0.011	7.90E-01
1.092860533	0.008	0.007	2.49E-01	-0.006	0.011	5.75E-01
7.499546	0.014	0.010	1.58E-01	0.009	0.015	5.33E-01
5.388432	0.010	0.008	2.05E-01	-0.008	0.012	4.90E-01
1.96623954	0.005	0.005	2.43E-01	-0.001	0.007	8.55E-01

8.829464	0.003	0.009	7.03E-01	0.001	0.013	9.46E-01
2.267683142	0.008	0.007	2.58E-01	0.009	0.011	4.18E-01
6.294781	-0.008	0.007	2.58E-01	-0.013	0.011	2.27E-01
3.519885	0.008	0.007	2.82E-01	0.028	0.010	7.58E-03
8.546188	-0.006	0.005	2.83E-01	-0.002	0.008	7.81E-01
6.202599	0.008	0.008	2.71E-01	0.002	0.011	8.85E-01
2.857786	-0.009	0.008	2.42E-01	-0.018	0.011	1.19E-01
4.329006	-0.015	0.013	2.51E-01	0.016	0.020	4.18E-01
7.26909	-0.013	0.011	2.23E-01	-0.023	0.016	1.40E-01
7.57087	0.013	0.010	1.86E-01	-0.011	0.015	4.52E-01
7.917396	-0.008	0.010	4.29E-01	-0.029	0.015	5.09E-02
8.444586	0.012	0.007	8.61E-02	0.001	0.010	9.57E-01
8.916264	-0.006	0.008	4.68E-01	-0.013	0.012	2.93E-01
7.788878	-0.011	0.009	2.38E-01	0.002	0.014	8.95E-01
2.775359922	-0.014	0.011	2.08E-01	-0.049	0.017	3.11E-03
5.74135	0.010	0.010	3.32E-01	0.012	0.015	4.39E-01
2.987312	-0.005	0.004	2.05E-01	0.003	0.006	5.71E-01
5.009272	0.010	0.010	2.76E-01	-0.002	0.014	8.66E-01
6.33549	0.011	0.009	2.30E-01	-0.001	0.013	9.26E-01
5.539827	0.010	0.008	2.16E-01	-0.011	0.013	3.68E-01
1.528877172	0.012	0.010	2.28E-01	-0.015	0.014	3.14E-01
3.599283	-0.008	0.007	2.15E-01	0.007	0.010	4.62E-01
6.407823	-0.007	0.007	3.60E-01	-0.009	0.011	3.91E-01
1.238872278	0.008	0.007	2.71E-01	-0.006	0.011	5.72E-01
5.274045	-0.010	0.006	1.25E-01	-0.021	0.010	2.99E-02
5.644121	0.009	0.007	2.01E-01	-0.009	0.010	3.55E-01
8.747711	-0.009	0.008	2.52E-01	-0.007	0.012	5.93E-01
7.258661	-0.012	0.011	2.73E-01	-0.004	0.017	7.95E-01
5.029795	0.009	0.008	2.55E-01	-0.011	0.012	3.38E-01
6.76646	0.014	0.010	1.32E-01	-0.003	0.014	8.15E-01
4.413787	-0.015	0.014	2.90E-01	0.012	0.021	5.68E-01
5.544537	0.007	0.007	3.17E-01	0.012	0.010	2.30E-01
8.414307	-0.009	0.009	3.04E-01	-0.004	0.013	7.57E-01
7.776094	-0.009	0.010	3.54E-01	-0.029	0.014	3.99E-02
7.865249	-0.010	0.009	2.59E-01	-0.013	0.013	3.06E-01
5.113903	0.010	0.009	2.62E-01	-0.001	0.014	9.48E-01
8.812306	0.011	0.009	2.13E-01	0.010	0.014	4.77E-01
8.899106	-0.009	0.007	2.31E-01	-0.025	0.011	2.30E-02
5.737312	0.010	0.010	3.20E-01	-0.025	0.015	1.08E-01
7.672136	0.010	0.009	2.56E-01	-0.005	0.013	6.76E-01
3.511811	0.009	0.008	2.44E-01	0.028	0.011	1.37E-02
3.619469	-0.006	0.006	3.38E-01	-0.004	0.009	6.99E-01
2.182229264	0.013	0.011	2.42E-01	-0.020	0.016	2.17E-01

7.539245	-0.009	0.009	3.36E-01	-0.025	0.014	6.44E-02
1.022882554	0.009	0.006	1.44E-01	0.003	0.009	7.24E-01
6.00444	0.010	0.008	2.19E-01	0.010	0.012	3.98E-01
5.679446	0.007	0.009	4.46E-01	-0.001	0.013	9.19E-01
6.332462	0.011	0.008	1.77E-01	-0.006	0.012	6.25E-01
2.144548813	-0.017	0.013	2.14E-01	-0.001	0.020	9.43E-01
0.650788109	-0.015	0.011	1.55E-01	-0.012	0.016	4.31E-01
1.652684365	0.005	0.005	3.21E-01	0.002	0.007	7.54E-01
1.89222437	-0.006	0.006	3.68E-01	-0.017	0.010	7.57E-02
2.189294348	0.014	0.011	2.19E-01	-0.018	0.017	2.78E-01
2.860477	-0.008	0.008	3.06E-01	-0.005	0.012	6.52E-01
2.029488867	0.008	0.006	1.68E-01	0.006	0.009	5.35E-01
3.53637	0.009	0.008	2.54E-01	0.020	0.012	8.34E-02
8.133385	0.008	0.009	3.52E-01	-0.011	0.014	4.29E-01
1.5157563	0.009	0.008	2.55E-01	-0.015	0.012	2.30E-01
1.405742843	0.007	0.005	1.56E-01	0.009	0.008	2.31E-01
2.18693932	0.014	0.011	2.06E-01	-0.018	0.016	2.78E-01
1.871701982	-0.009	0.008	2.45E-01	0.000	0.011	9.88E-01
2.097784683	-0.005	0.008	5.02E-01	-0.004	0.012	7.09E-01
6.786982	0.014	0.009	1.46E-01	0.000	0.014	9.99E-01
8.772944	-0.008	0.009	3.49E-01	-0.021	0.013	1.09E-01
2.181556398	0.014	0.011	2.04E-01	-0.014	0.016	4.01E-01
8.222204	0.011	0.011	3.11E-01	0.004	0.016	8.11E-01
6.589496	0.007	0.007	3.78E-01	0.001	0.011	9.10E-01
4.38519	-0.015	0.013	2.72E-01	0.007	0.020	7.32E-01
4.374088	-0.016	0.015	2.88E-01	0.014	0.023	5.58E-01
5.039551	0.008	0.008	3.00E-01	-0.002	0.012	8.47E-01
7.929171	0.007	0.007	2.66E-01	-0.002	0.010	8.64E-01
5.310043	0.019	0.016	2.30E-01	-0.022	0.024	3.52E-01
7.787869	-0.008	0.007	2.89E-01	-0.001	0.011	9.49E-01
5.24713	-0.009	0.005	6.53E-02	-0.006	0.007	4.34E-01
7.692659	0.007	0.007	2.88E-01	0.010	0.010	3.05E-01
8.232297	0.011	0.010	2.51E-01	0.010	0.015	5.16E-01
6.815915	0.007	0.009	4.28E-01	0.005	0.013	7.07E-01
7.801326	-0.010	0.008	2.57E-01	-0.025	0.013	4.98E-02
5.6781	0.009	0.008	2.44E-01	-0.014	0.012	2.48E-01
7.694341	-0.012	0.010	2.42E-01	-0.010	0.016	5.38E-01
5.609805	-0.012	0.010	2.57E-01	-0.017	0.015	2.78E-01
6.90507	0.009	0.008	2.93E-01	0.016	0.012	2.02E-01
8.186205	0.011	0.010	2.82E-01	0.010	0.015	5.16E-01
8.884639	0.010	0.008	2.23E-01	0.013	0.012	3.03E-01
1.430302422	0.006	0.005	1.82E-01	0.001	0.007	8.41E-01
2.6037793	-0.017	0.015	2.64E-01	0.027	0.022	2.15E-01

3.547473	0.015	0.009	1.24E-01	0.022	0.014	1.22E-01
4.408067	-0.016	0.015	2.85E-01	0.013	0.023	5.58E-01
1.111027893	0.007	0.006	2.55E-01	0.000	0.009	9.91E-01
7.24554	-0.013	0.010	2.03E-01	-0.015	0.015	3.22E-01
4.349192	-0.018	0.015	2.34E-01	0.014	0.022	5.44E-01
8.791784	-0.011	0.010	2.41E-01	-0.022	0.014	1.33E-01
8.823409	-0.007	0.007	3.05E-01	0.004	0.010	6.52E-01
1.853198189	-0.006	0.008	5.02E-01	0.001	0.012	9.47E-01
2.558024468	0.009	0.011	4.18E-01	0.015	0.016	3.58E-01
8.748048	-0.008	0.008	3.45E-01	-0.026	0.012	3.13E-02
7.207186	0.005	0.007	4.68E-01	0.004	0.011	7.41E-01
2.34539907	0.010	0.007	1.59E-01	0.009	0.010	3.80E-01
7.560104	0.008	0.009	3.98E-01	0.020	0.014	1.46E-01
3.511474	0.009	0.008	2.54E-01	0.024	0.012	3.92E-02
6.05726	0.008	0.009	3.92E-01	-0.002	0.013	8.53E-01
6.513799	-0.008	0.009	3.68E-01	-0.018	0.013	1.75E-01
6.296463	-0.008	0.009	3.99E-01	0.001	0.014	9.18E-01
7.300715	-0.012	0.009	1.85E-01	-0.015	0.014	2.74E-01
6.927947	-0.010	0.008	1.91E-01	0.005	0.012	6.89E-01
7.486425	0.012	0.010	2.40E-01	-0.002	0.015	8.82E-01
1.004715195	0.010	0.009	2.41E-01	0.007	0.013	5.58E-01
6.584113	0.009	0.008	2.69E-01	0.006	0.012	5.78E-01
7.085398	0.007	0.006	2.25E-01	-0.003	0.008	6.93E-01
2.040927575	0.006	0.005	1.84E-01	0.008	0.007	2.70E-01
4.401339	-0.014	0.013	2.95E-01	0.011	0.020	5.67E-01
5.729238	0.006	0.008	4.37E-01	-0.013	0.011	2.55E-01
0.746334965	-0.008	0.006	1.53E-01	0.004	0.009	6.78E-01
2.715138488	-0.010	0.006	1.12E-01	-0.015	0.009	9.55E-02
7.965169	0.010	0.009	2.85E-01	-0.003	0.014	8.27E-01
7.833624	-0.007	0.010	4.69E-01	0.002	0.015	8.85E-01
6.007131	-0.011	0.010	2.44E-01	-0.008	0.014	5.58E-01
4.152379	-0.016	0.014	2.62E-01	0.005	0.021	8.03E-01
5.647485	0.010	0.008	1.96E-01	-0.009	0.011	4.31E-01
4.384181	-0.014	0.013	2.87E-01	0.007	0.019	7.29E-01
7.520405	0.008	0.007	2.40E-01	-0.004	0.011	7.40E-01
1.08074896	0.008	0.007	2.37E-01	0.000	0.010	9.83E-01
3.123231	-0.012	0.008	1.54E-01	-0.006	0.012	6.33E-01
6.278969	-0.006	0.009	4.75E-01	-0.019	0.013	1.52E-01
7.080688	0.008	0.008	3.70E-01	-0.003	0.013	8.34E-01
1.221714216	0.008	0.007	2.96E-01	0.001	0.011	9.36E-01
5.118613	0.010	0.009	2.74E-01	-0.005	0.013	7.12E-01
5.260251	-0.009	0.005	8.37E-02	-0.011	0.008	1.65E-01
6.446849	0.008	0.009	3.31E-01	-0.018	0.013	1.65E-01

7.753217	-0.009	0.010	3.76E-01	0.000	0.015	9.98E-01
7.23141	-0.013	0.009	1.66E-01	-0.002	0.014	8.70E-01
7.066558	0.008	0.008	3.14E-01	-0.007	0.012	5.56E-01
6.683024	0.007	0.007	3.03E-01	0.004	0.011	7.11E-01
5.770619	0.006	0.008	4.31E-01	-0.004	0.011	7.24E-01
7.243185	-0.012	0.008	1.32E-01	-0.013	0.012	2.95E-01
4.341454	-0.015	0.014	3.07E-01	0.014	0.021	5.19E-01
2.657272082	-0.010	0.008	2.36E-01	0.019	0.012	1.21E-01
7.431587	0.010	0.012	3.77E-01	0.000	0.018	9.94E-01
7.102892	-0.010	0.009	2.78E-01	0.002	0.014	9.01E-01
4.421188	-0.014	0.013	2.91E-01	0.006	0.020	7.63E-01
8.890695	-0.007	0.006	2.96E-01	0.000	0.009	9.78E-01
1.793986053	-0.010	0.010	3.18E-01	0.016	0.016	3.11E-01
6.360722	-0.007	0.009	4.20E-01	-0.026	0.013	4.99E-02
6.242298	0.013	0.010	1.89E-01	-0.001	0.015	9.44E-01
4.381489	-0.015	0.014	3.07E-01	0.009	0.021	6.68E-01
6.738199	-0.011	0.008	1.84E-01	-0.023	0.012	5.98E-02
7.798971	-0.007	0.007	3.14E-01	0.007	0.011	5.40E-01
0.974436261	0.005	0.006	3.92E-01	0.018	0.009	4.13E-02
6.237251	0.008	0.008	2.78E-01	0.008	0.011	4.78E-01
7.511658	-0.007	0.010	4.50E-01	-0.023	0.014	1.08E-01
4.395956	-0.015	0.014	2.89E-01	0.011	0.021	6.12E-01
1.967921703	0.005	0.004	2.59E-01	-0.001	0.007	8.97E-01
3.689784	-0.005	0.004	2.60E-01	0.004	0.006	5.35E-01
5.259578	-0.009	0.005	8.80E-02	-0.011	0.008	1.56E-01
3.137361	-0.012	0.010	2.33E-01	-0.013	0.014	3.48E-01
5.283801	-0.010	0.008	1.71E-01	-0.026	0.011	2.51E-02
5.081605	0.010	0.010	3.16E-01	-0.012	0.015	3.96E-01
1.912410325	-0.015	0.011	1.60E-01	-0.035	0.016	2.78E-02
1.880785662	-0.007	0.007	2.95E-01	-0.007	0.010	4.74E-01
5.723519	0.008	0.010	4.30E-01	-0.009	0.014	5.25E-01
6.636597	0.006	0.007	4.18E-01	0.011	0.010	2.82E-01
0.816312943	0.004	0.005	4.45E-01	0.015	0.008	4.97E-02
2.607480059	-0.014	0.013	2.68E-01	0.015	0.019	4.25E-01
2.799246636	-0.016	0.014	2.61E-01	-0.069	0.021	7.77E-04
6.22413	0.008	0.008	3.59E-01	-0.001	0.012	9.60E-01
1.928895522	0.005	0.004	2.33E-01	0.002	0.006	7.48E-01
6.262484	0.007	0.007	3.15E-01	0.000	0.010	9.63E-01
5.771629	0.005	0.007	4.19E-01	-0.005	0.010	5.96E-01
0.851974798	-0.009	0.007	2.09E-01	-0.013	0.010	2.07E-01
2.79588231	-0.014	0.012	2.27E-01	-0.062	0.018	5.45E-04
7.614606	-0.011	0.009	2.20E-01	-0.014	0.014	3.01E-01
6.61742	0.007	0.007	3.52E-01	-0.006	0.011	5.70E-01

1.721989479	-0.006	0.005	2.36E-01	-0.005	0.008	4.90E-01
1.528540739	0.011	0.010	2.52E-01	-0.015	0.014	2.85E-01
1.118765843	0.009	0.007	2.06E-01	-0.001	0.010	9.61E-01
1.706850012	-0.005	0.005	2.95E-01	-0.004	0.007	5.42E-01
8.068117	0.009	0.009	3.45E-01	0.011	0.014	4.25E-01
3.16394	-0.008	0.007	2.19E-01	0.008	0.010	4.44E-01
4.381826	-0.015	0.015	2.93E-01	0.012	0.022	5.75E-01
7.676173	0.008	0.009	3.35E-01	0.007	0.013	5.55E-01
0.708654514	0.011	0.009	2.33E-01	-0.005	0.013	7.11E-01
0.634975777	0.008	0.009	3.46E-01	0.002	0.013	8.93E-01
2.189630781	0.014	0.011	2.06E-01	-0.017	0.017	3.12E-01
1.315915341	0.015	0.009	9.55E-02	0.006	0.014	6.70E-01
7.610569	-0.016	0.014	2.66E-01	-0.015	0.021	4.66E-01
1.798023244	-0.008	0.009	3.74E-01	-0.009	0.014	5.05E-01
7.368001	-0.010	0.009	2.61E-01	-0.002	0.013	8.54E-01
2.340689014	0.010	0.007	1.42E-01	0.004	0.010	7.07E-01
4.448439	-0.017	0.017	3.14E-01	0.019	0.025	4.52E-01
4.31723	-0.015	0.014	2.74E-01	0.025	0.021	2.31E-01
2.041264008	0.006	0.005	1.92E-01	0.007	0.007	3.66E-01
7.329648	-0.008	0.006	2.02E-01	-0.007	0.009	4.39E-01
1.910055297	-0.013	0.009	1.53E-01	-0.034	0.014	1.17E-02
8.961682	-0.010	0.008	2.23E-01	0.012	0.012	3.17E-01
3.758752	0.008	0.009	3.38E-01	0.021	0.013	1.04E-01
0.689141424	0.022	0.016	1.73E-01	0.010	0.024	6.57E-01
5.325855	0.022	0.018	2.38E-01	-0.027	0.028	3.28E-01
7.989056	0.010	0.011	3.59E-01	-0.032	0.017	5.26E-02
3.861701	-0.006	0.005	2.11E-01	0.007	0.008	3.81E-01
2.176173477	0.014	0.011	2.07E-01	-0.011	0.016	5.01E-01
6.726088	-0.013	0.010	2.24E-01	-0.011	0.016	4.61E-01
8.756122	0.007	0.006	2.57E-01	-0.006	0.009	4.84E-01
2.504195253	-0.019	0.014	1.70E-01	-0.021	0.021	3.25E-01
5.2764	-0.010	0.007	1.38E-01	-0.022	0.010	3.04E-02
6.786646	0.014	0.010	1.57E-01	0.008	0.015	5.65E-01
6.293099	0.008	0.007	2.85E-01	0.020	0.011	6.30E-02
7.622008	-0.016	0.010	1.24E-01	0.020	0.015	2.05E-01
2.339679716	0.014	0.008	7.66E-02	0.014	0.012	2.34E-01
4.441711	-0.015	0.015	3.28E-01	0.021	0.023	3.66E-01
0.745998532	-0.009	0.006	1.30E-01	0.002	0.009	8.14E-01
1.698102765	-0.004	0.004	3.46E-01	0.007	0.006	2.80E-01
2.85644	-0.006	0.007	3.96E-01	-0.011	0.011	3.21E-01
1.766735013	-0.009	0.010	4.17E-01	0.011	0.016	4.68E-01
5.115585	0.012	0.010	2.09E-01	0.008	0.015	5.87E-01
7.180608	0.011	0.008	2.01E-01	-0.004	0.012	7.46E-01

5.309707	0.019	0.016	2.35E-01	-0.022	0.024	3.57E-01
1.985416198	0.009	0.007	1.95E-01	-0.006	0.011	5.89E-01
2.375678003	-0.022	0.018	2.22E-01	-0.003	0.027	9.19E-01
5.165377	0.013	0.010	1.82E-01	-0.008	0.015	5.94E-01
3.569004	-0.007	0.007	3.34E-01	0.002	0.011	8.86E-01
4.409749	-0.013	0.013	3.04E-01	0.008	0.019	6.84E-01
8.527684	0.007	0.009	3.84E-01	0.007	0.013	5.80E-01
5.247467	-0.009	0.005	6.38E-02	-0.007	0.007	3.10E-01
6.370815	0.007	0.006	2.90E-01	0.014	0.009	1.36E-01
6.468044	0.011	0.008	1.78E-01	-0.020	0.012	9.47E-02
0.956941767	-0.008	0.006	1.67E-01	0.009	0.008	3.16E-01
7.562123	0.010	0.009	2.72E-01	-0.016	0.014	2.67E-01
1.123475899	0.009	0.006	1.69E-01	-0.001	0.010	9.44E-01
6.519855	-0.009	0.008	2.42E-01	-0.015	0.012	2.26E-01
4.234805	0.012	0.013	3.49E-01	0.017	0.020	3.83E-01
5.589619	-0.014	0.010	1.62E-01	0.010	0.015	5.02E-01
8.578486	-0.011	0.008	1.70E-01	-0.014	0.012	2.12E-01
3.038787	-0.009	0.007	2.07E-01	0.007	0.010	4.74E-01
2.310073648	-0.020	0.016	2.17E-01	0.003	0.024	8.95E-01
3.137025	-0.011	0.010	2.56E-01	-0.019	0.015	1.90E-01
3.754715	0.012	0.011	2.81E-01	0.016	0.016	3.35E-01
3.039796	-0.007	0.006	2.41E-01	0.010	0.009	2.47E-01
4.127483	-0.010	0.009	3.17E-01	0.017	0.014	2.41E-01
8.057015	0.011	0.008	1.58E-01	0.001	0.011	9.20E-01
0.704280891	0.014	0.010	1.64E-01	-0.005	0.015	7.30E-01
8.320778	-0.010	0.008	2.37E-01	-0.006	0.012	6.39E-01
1.007743088	0.008	0.007	2.57E-01	0.014	0.010	1.45E-01
5.937153	0.011	0.010	2.49E-01	-0.001	0.014	9.42E-01
8.25652	-0.007	0.009	4.25E-01	-0.001	0.013	9.36E-01
5.449999	0.011	0.010	2.34E-01	-0.004	0.014	7.89E-01
4.235141	0.012	0.013	3.41E-01	0.019	0.020	3.30E-01
7.61427	-0.013	0.011	2.49E-01	-0.028	0.017	9.60E-02
8.037839	0.007	0.007	2.64E-01	0.011	0.010	2.48E-01
2.287532665	-0.007	0.006	2.73E-01	0.002	0.009	7.92E-01
3.645711	0.007	0.004	1.37E-01	0.018	0.007	6.36E-03
8.131703	0.009	0.009	3.06E-01	-0.004	0.014	7.68E-01
2.554996575	-0.012	0.011	2.76E-01	-0.012	0.017	4.78E-01
3.161248	-0.010	0.007	1.66E-01	-0.002	0.011	8.42E-01
8.532058	-0.010	0.009	2.70E-01	-0.019	0.014	1.76E-01
6.973366	-0.013	0.010	1.82E-01	-0.001	0.015	9.69E-01
5.098427	0.009	0.009	2.66E-01	-0.005	0.013	6.98E-01
5.813346	0.008	0.008	3.15E-01	-0.001	0.012	9.62E-01
6.815579	0.008	0.009	3.65E-01	0.005	0.013	6.87E-01

0.688132126	0.018	0.015	2.42E-01	-0.003	0.023	8.78E-01
7.968197	0.007	0.009	4.34E-01	-0.006	0.013	6.48E-01
1.871029117	-0.009	0.008	2.68E-01	0.003	0.012	8.20E-01
6.437092	0.008	0.010	3.88E-01	0.011	0.014	4.22E-01
3.620479	-0.006	0.006	2.76E-01	-0.012	0.009	1.77E-01
2.531446293	-0.012	0.012	3.52E-01	-0.003	0.019	8.54E-01
5.120968	0.009	0.007	2.39E-01	-0.007	0.011	4.99E-01
3.064019	-0.006	0.008	4.21E-01	0.007	0.012	5.73E-01
7.756917	-0.011	0.011	2.87E-01	-0.019	0.016	2.27E-01
3.572032	-0.005	0.006	3.78E-01	0.008	0.008	3.32E-01
0.835826034	-0.012	0.009	1.67E-01	-0.009	0.013	4.89E-01
8.311695	0.009	0.008	2.78E-01	0.011	0.012	3.62E-01
2.143875948	-0.015	0.013	2.22E-01	0.009	0.019	6.26E-01
5.259915	-0.009	0.005	8.75E-02	-0.011	0.008	1.48E-01
8.969757	-0.011	0.008	1.43E-01	0.000	0.012	9.84E-01
3.201956	0.006	0.006	2.95E-01	0.019	0.009	3.37E-02
0.742634206	-0.007	0.006	2.32E-01	-0.010	0.009	2.66E-01
6.314967	0.008	0.008	3.36E-01	-0.024	0.012	4.39E-02
2.086009542	-0.006	0.006	2.55E-01	0.006	0.008	4.62E-01
7.180272	0.011	0.008	1.97E-01	-0.002	0.012	8.67E-01
8.379318	0.007	0.008	3.39E-01	-0.009	0.011	4.32E-01
5.733275	0.006	0.009	4.90E-01	-0.012	0.013	3.31E-01
1.14433472	0.009	0.006	1.58E-01	-0.004	0.009	6.82E-01
5.781385	0.009	0.010	3.59E-01	-0.010	0.014	4.69E-01
1.768753609	-0.011	0.010	2.90E-01	-0.017	0.016	2.87E-01
5.116258	0.009	0.008	2.91E-01	-0.002	0.013	8.52E-01
4.224712	0.011	0.011	3.33E-01	0.007	0.016	6.89E-01
3.689447	-0.005	0.004	2.72E-01	0.003	0.006	6.74E-01
5.158985	0.010	0.009	2.65E-01	-0.020	0.013	1.35E-01
2.024442378	0.011	0.009	1.89E-01	0.001	0.013	9.12E-01
2.858459	-0.006	0.008	4.05E-01	-0.009	0.012	4.19E-01
8.519946	-0.009	0.010	3.47E-01	-0.038	0.015	1.13E-02
8.384701	0.010	0.010	2.97E-01	-0.011	0.014	4.52E-01
8.838212	0.011	0.008	1.78E-01	-0.006	0.012	6.27E-01
3.990891	-0.008	0.008	3.69E-01	0.004	0.013	7.64E-01
7.356226	-0.012	0.010	2.34E-01	-0.015	0.015	3.14E-01
7.772393	-0.013	0.012	2.78E-01	-0.009	0.018	6.05E-01
8.914582	0.011	0.008	1.70E-01	0.004	0.012	7.03E-01
8.485967	0.010	0.009	2.75E-01	0.000	0.014	9.78E-01
1.776827991	-0.010	0.011	3.70E-01	0.014	0.016	4.01E-01
6.397393	0.009	0.009	3.29E-01	-0.002	0.014	8.78E-01
1.238535845	0.008	0.007	2.85E-01	-0.006	0.011	5.62E-01
0.509149988	-0.009	0.008	2.87E-01	-0.007	0.013	5.67E-01

4.448103	-0.016	0.017	3.23E-01	0.019	0.025	4.52E-01
2.637086127	-0.010	0.009	2.66E-01	-0.005	0.013	7.32E-01
3.136352	-0.012	0.010	2.33E-01	-0.017	0.016	2.65E-01
7.282884	-0.012	0.010	2.31E-01	0.004	0.015	7.97E-01
1.516092733	0.009	0.008	2.54E-01	-0.019	0.012	1.24E-01
8.118582	-0.009	0.007	2.17E-01	-0.006	0.010	5.89E-01
6.879501	0.013	0.009	1.61E-01	-0.003	0.014	8.35E-01
2.335978958	0.012	0.009	1.66E-01	0.009	0.013	4.98E-01
3.770864	0.006	0.008	4.63E-01	0.010	0.012	4.17E-01
7.788542	-0.011	0.009	2.40E-01	0.003	0.014	8.49E-01
8.707676	0.006	0.009	4.60E-01	-0.033	0.013	9.68E-03
2.671065818	-0.006	0.005	2.46E-01	0.009	0.007	2.02E-01
4.156416	-0.014	0.015	3.29E-01	0.015	0.022	4.91E-01
0.717738194	0.008	0.008	3.01E-01	0.014	0.011	2.20E-01
5.858428	0.010	0.008	1.96E-01	-0.014	0.012	2.23E-01
1.190762417	0.006	0.006	2.54E-01	0.001	0.008	8.63E-01
6.484866	-0.009	0.008	2.69E-01	-0.017	0.012	1.43E-01
7.832951	-0.011	0.011	3.08E-01	0.014	0.017	3.94E-01
1.635526303	0.008	0.007	2.09E-01	0.004	0.010	6.76E-01
7.914031	0.011	0.010	2.66E-01	-0.029	0.015	5.64E-02
7.313836	-0.012	0.008	1.26E-01	0.011	0.012	3.30E-01
7.839007	-0.010	0.009	2.74E-01	0.011	0.013	3.83E-01
1.452843406	0.005	0.004	2.13E-01	0.002	0.006	7.64E-01
5.014319	0.008	0.007	2.54E-01	-0.002	0.010	8.69E-01
7.613597	-0.018	0.013	1.82E-01	-0.026	0.020	1.98E-01
7.997803	0.009	0.009	2.97E-01	0.007	0.013	5.62E-01
6.740554	-0.013	0.009	1.55E-01	-0.003	0.014	8.21E-01
8.097051	0.011	0.008	1.87E-01	-0.015	0.012	2.20E-01
6.025635	-0.009	0.009	3.30E-01	-0.016	0.014	2.25E-01
4.447093	-0.016	0.017	3.59E-01	0.023	0.026	3.79E-01
6.442812	0.011	0.007	1.41E-01	-0.009	0.011	3.94E-01
3.218778	-0.017	0.015	2.45E-01	-0.009	0.022	6.83E-01
2.408648397	-0.017	0.014	2.19E-01	0.009	0.020	6.42E-01
1.802733301	-0.010	0.010	3.38E-01	0.005	0.015	7.65E-01
4.321268	-0.016	0.015	2.91E-01	0.018	0.023	4.27E-01
7.029886	-0.010	0.010	3.44E-01	-0.015	0.015	3.37E-01
3.57035	-0.007	0.007	3.11E-01	0.010	0.010	3.10E-01
5.732602	0.009	0.009	3.39E-01	-0.011	0.014	4.22E-01
3.659168	-0.005	0.005	3.32E-01	0.009	0.008	2.60E-01
0.859039883	0.011	0.009	2.20E-01	-0.002	0.014	8.64E-01
8.467126	-0.009	0.009	3.17E-01	-0.023	0.014	1.08E-01
2.30906435	-0.013	0.010	1.94E-01	0.006	0.015	6.94E-01
5.733948	0.010	0.011	3.63E-01	0.001	0.016	9.63E-01

2.664000734	-0.016	0.012	1.88E-01	-0.008	0.018	6.36E-01
6.721714	-0.011	0.011	2.93E-01	-0.004	0.016	7.85E-01
8.287471	-0.008	0.009	3.86E-01	-0.011	0.013	4.21E-01
4.130847	-0.012	0.013	3.35E-01	0.022	0.019	2.44E-01
8.252819	0.009	0.008	2.70E-01	0.006	0.012	5.87E-01
6.461315	0.007	0.009	4.41E-01	-0.007	0.014	6.13E-01
8.872864	-0.012	0.010	2.14E-01	0.028	0.015	5.33E-02
5.109866	0.009	0.008	2.43E-01	-0.012	0.012	3.04E-01
5.049644	0.009	0.008	2.69E-01	0.005	0.012	6.96E-01
6.062979	0.008	0.008	3.13E-01	-0.001	0.012	9.48E-01
0.725812576	0.006	0.007	4.46E-01	0.004	0.011	6.99E-01
3.513829	0.008	0.008	3.13E-01	0.026	0.012	3.32E-02
6.582095	0.005	0.007	4.60E-01	0.006	0.011	5.68E-01
0.531018106	0.011	0.009	2.25E-01	0.002	0.014	8.83E-01
5.810318	0.007	0.008	3.85E-01	-0.007	0.012	5.56E-01
7.30778	-0.011	0.011	2.89E-01	-0.017	0.016	2.78E-01
8.257865	-0.010	0.008	2.42E-01	-0.004	0.013	7.30E-01
7.877024	0.009	0.009	3.29E-01	0.001	0.014	9.28E-01
6.079464	0.010	0.007	1.84E-01	0.007	0.011	5.57E-01
6.023953	0.008	0.007	2.44E-01	0.003	0.011	7.74E-01
6.264502	0.012	0.009	1.74E-01	-0.008	0.013	5.35E-01
8.326498	-0.008	0.009	3.36E-01	-0.012	0.013	3.75E-01
0.649442378	-0.015	0.010	1.55E-01	0.012	0.016	4.32E-01
8.46208	0.005	0.007	4.57E-01	0.011	0.010	2.85E-01
2.369285784	-0.007	0.007	3.16E-01	0.017	0.010	1.03E-01
2.37130438	-0.007	0.007	3.39E-01	0.010	0.010	3.26E-01
2.601087839	-0.018	0.016	2.57E-01	0.006	0.024	8.13E-01
2.597723514	-0.019	0.018	3.08E-01	-0.003	0.027	9.21E-01
4.104942	-0.012	0.013	3.55E-01	0.008	0.019	6.79E-01
6.004103	0.011	0.009	2.50E-01	0.013	0.014	3.60E-01
8.503125	0.009	0.007	2.14E-01	0.019	0.010	6.60E-02
5.933453	-0.005	0.007	4.36E-01	-0.012	0.010	2.40E-01
2.040591143	0.006	0.004	1.86E-01	0.008	0.007	2.14E-01
5.987618	0.008	0.008	3.14E-01	0.019	0.012	9.41E-02
8.962692	-0.007	0.008	3.69E-01	-0.018	0.012	1.43E-01
1.428283827	0.006	0.005	1.69E-01	0.003	0.007	6.94E-01
7.864912	-0.010	0.008	2.10E-01	-0.012	0.012	3.06E-01
7.237465	-0.016	0.011	1.26E-01	-0.001	0.016	9.73E-01
5.914276	-0.014	0.010	1.90E-01	-0.015	0.015	3.39E-01
1.074693173	0.008	0.007	2.67E-01	-0.001	0.010	9.48E-01
6.542059	0.007	0.008	3.79E-01	0.001	0.012	9.18E-01
6.446512	0.010	0.010	2.77E-01	-0.019	0.014	1.84E-01
8.672687	0.006	0.007	3.60E-01	0.007	0.010	4.65E-01

5.445289	-0.007	0.008	3.65E-01	0.001	0.012	9.55E-01
7.035606	-0.018	0.012	1.30E-01	-0.025	0.018	1.74E-01
3.965995	-0.010	0.008	2.08E-01	0.008	0.012	4.93E-01
7.733367	-0.011	0.012	3.57E-01	0.009	0.018	6.19E-01
5.259242	-0.009	0.005	9.10E-02	-0.011	0.008	1.81E-01
6.424644	0.008	0.007	2.76E-01	0.008	0.010	4.59E-01
5.246457	-0.009	0.005	6.80E-02	-0.005	0.007	4.90E-01
5.096408	0.009	0.008	2.87E-01	0.005	0.013	6.99E-01
8.36216	0.008	0.007	2.25E-01	0.001	0.010	9.36E-01
8.095368	0.009	0.007	2.07E-01	0.007	0.011	5.34E-01
6.883875	0.015	0.011	1.68E-01	0.016	0.016	2.96E-01
1.465964277	-0.006	0.006	3.65E-01	0.007	0.009	4.26E-01
7.447399	0.009	0.007	1.65E-01	-0.014	0.010	1.74E-01
6.49765	0.014	0.010	1.73E-01	-0.021	0.015	1.50E-01
2.023096648	0.012	0.009	1.94E-01	0.001	0.014	9.52E-01
2.023769513	0.012	0.009	1.94E-01	0.001	0.013	9.38E-01
7.639502	-0.010	0.012	4.08E-01	0.047	0.018	1.05E-02
1.454862001	0.004	0.004	2.61E-01	0.000	0.005	9.39E-01
7.462202	0.009	0.009	3.56E-01	-0.005	0.014	7.44E-01
2.17785564	0.016	0.012	1.62E-01	-0.012	0.017	4.80E-01
0.910177636	-0.005	0.005	3.16E-01	-0.026	0.008	1.57E-03
5.716117	0.008	0.009	3.82E-01	-0.007	0.013	5.75E-01
1.141306827	0.010	0.006	1.26E-01	-0.004	0.010	6.86E-01
2.548604355	-0.016	0.013	2.11E-01	-0.024	0.019	2.11E-01
2.04160044	0.007	0.005	1.94E-01	0.006	0.007	4.16E-01
2.065150722	0.006	0.004	1.27E-01	0.010	0.005	5.70E-02
5.652868	-0.009	0.010	3.78E-01	-0.019	0.015	1.98E-01
3.338212	-0.008	0.009	3.49E-01	0.007	0.013	6.14E-01
5.92235	-0.009	0.008	2.59E-01	-0.018	0.012	1.32E-01
1.529213604	0.012	0.010	2.35E-01	-0.014	0.015	3.35E-01
6.99658	0.009	0.008	2.46E-01	0.002	0.012	8.73E-01
5.996366	0.007	0.007	3.10E-01	0.009	0.011	4.30E-01
1.525176413	0.011	0.009	2.27E-01	-0.014	0.014	3.21E-01
7.267071	-0.014	0.011	2.12E-01	0.008	0.016	6.02E-01
2.024105946	0.011	0.009	1.94E-01	0.002	0.013	9.03E-01
6.064661	-0.011	0.008	1.80E-01	-0.023	0.013	7.00E-02
4.185012	-0.012	0.014	3.84E-01	0.011	0.021	5.92E-01
1.414153658	0.006	0.004	1.39E-01	0.011	0.006	7.41E-02
6.977403	-0.009	0.011	4.04E-01	0.000	0.016	9.88E-01
8.882284	-0.013	0.009	1.78E-01	-0.012	0.014	4.12E-01
8.611456	-0.007	0.006	2.64E-01	0.005	0.009	6.23E-01
8.151889	-0.013	0.011	2.40E-01	-0.003	0.016	8.72E-01
1.530222902	0.012	0.010	2.37E-01	-0.019	0.015	2.08E-01

4.412104	-0.015	0.015	3.04E-01	0.019	0.022	4.05E-01
3.989882	-0.008	0.007	2.90E-01	0.003	0.011	7.82E-01
6.127911	-0.007	0.007	3.02E-01	-0.015	0.011	1.56E-01
6.662502	0.007	0.008	4.07E-01	0.014	0.012	2.34E-01
8.715414	0.007	0.008	3.51E-01	0.020	0.011	8.48E-02
3.605676	0.010	0.011	3.31E-01	-0.020	0.016	2.02E-01
2.997742	-0.005	0.004	2.38E-01	0.003	0.006	5.82E-01
4.408404	-0.016	0.016	3.01E-01	0.020	0.024	4.06E-01
0.595949596	-0.008	0.008	3.66E-01	-0.021	0.013	9.91E-02
6.161554	0.008	0.006	2.32E-01	0.004	0.010	6.46E-01
5.707706	0.010	0.011	3.55E-01	-0.012	0.017	4.73E-01
6.92963	0.009	0.010	3.65E-01	0.002	0.015	8.79E-01
2.796218742	-0.014	0.012	2.50E-01	-0.063	0.018	4.80E-04
0.773586004	0.007	0.005	1.79E-01	0.003	0.007	6.60E-01
0.805210668	0.004	0.006	5.25E-01	0.012	0.008	1.59E-01
5.598702	0.010	0.009	2.37E-01	-0.010	0.013	4.68E-01
2.0298253	0.008	0.006	1.76E-01	0.006	0.009	5.25E-01
5.299614	0.017	0.014	2.34E-01	-0.019	0.021	3.69E-01
1.693056276	-0.004	0.004	2.63E-01	0.005	0.006	3.88E-01
2.149931735	-0.009	0.007	2.09E-01	0.000	0.011	9.64E-01
5.628645	0.010	0.009	3.06E-01	-0.024	0.014	8.23E-02
2.02343308	0.012	0.009	1.97E-01	0.001	0.014	9.51E-01
8.028418	-0.008	0.008	3.37E-01	-0.008	0.012	5.21E-01
1.628124786	0.007	0.006	2.57E-01	0.002	0.009	8.06E-01
2.552305114	-0.014	0.011	1.96E-01	0.000	0.017	9.98E-01
7.257315	-0.012	0.009	1.76E-01	-0.022	0.014	1.13E-01
1.881122094	-0.007	0.007	2.97E-01	-0.006	0.010	5.76E-01
6.916509	-0.011	0.009	2.13E-01	-0.016	0.013	2.20E-01
1.725353805	-0.006	0.005	2.23E-01	0.008	0.007	2.45E-01
8.199326	0.007	0.008	4.00E-01	0.000	0.012	9.96E-01
4.406385	-0.016	0.016	3.21E-01	0.014	0.023	5.40E-01
4.442383	-0.015	0.015	3.22E-01	0.019	0.022	3.88E-01
6.241288	0.006	0.007	3.99E-01	-0.005	0.011	6.79E-01
8.661921	0.010	0.008	2.10E-01	0.017	0.012	1.51E-01
1.425928799	0.007	0.005	1.35E-01	0.010	0.007	1.42E-01
2.375341571	-0.023	0.019	2.30E-01	0.000	0.028	9.94E-01
6.37317	0.008	0.010	4.44E-01	0.002	0.015	8.75E-01
7.230737	-0.013	0.011	2.35E-01	0.004	0.016	7.97E-01
8.85907	0.008	0.009	3.89E-01	-0.010	0.014	4.68E-01
4.306801	-0.014	0.012	2.42E-01	0.014	0.017	4.32E-01
8.968747	-0.010	0.009	2.97E-01	0.025	0.014	6.87E-02
8.711713	-0.010	0.010	3.15E-01	-0.003	0.014	8.29E-01
2.535483484	-0.012	0.011	2.75E-01	0.010	0.016	5.21E-01

8.900452	-0.007	0.009	4.60E-01	-0.009	0.014	4.93E-01
4.384517	-0.014	0.013	2.84E-01	0.009	0.020	6.56E-01
2.181892831	0.013	0.011	2.17E-01	-0.014	0.016	3.98E-01
5.514931	0.006	0.007	4.18E-01	-0.012	0.011	2.63E-01
8.617848	-0.014	0.009	1.08E-01	-0.010	0.013	4.71E-01
5.305669	0.018	0.015	2.28E-01	-0.022	0.022	3.05E-01
8.943179	-0.010	0.009	2.70E-01	-0.010	0.013	4.58E-01
7.999485	0.008	0.008	2.99E-01	0.014	0.012	2.37E-01
2.657608515	-0.009	0.008	2.55E-01	0.015	0.012	2.01E-01
1.852188892	-0.007	0.008	3.92E-01	0.003	0.012	8.19E-01
4.44272	-0.016	0.016	3.23E-01	0.018	0.024	4.55E-01
5.861793	0.014	0.011	1.95E-01	-0.004	0.016	8.13E-01
7.112312	-0.009	0.009	3.19E-01	-0.018	0.013	1.76E-01
1.809461953	-0.012	0.010	2.39E-01	-0.010	0.015	5.22E-01
5.83656	-0.008	0.008	3.24E-01	0.012	0.013	3.51E-01
2.870907	-0.010	0.010	3.28E-01	-0.005	0.015	7.14E-01
3.955229	-0.008	0.010	3.94E-01	-0.014	0.015	3.56E-01
5.982572	0.013	0.010	1.79E-01	-0.005	0.015	7.28E-01
1.238199413	0.008	0.007	2.95E-01	-0.006	0.011	5.79E-01
7.779122	-0.009	0.009	3.10E-01	-0.009	0.014	5.08E-01
5.574143	0.009	0.009	3.13E-01	-0.005	0.013	7.22E-01
8.904825	-0.011	0.009	2.12E-01	-0.003	0.013	8.48E-01
6.831391	0.010	0.009	2.56E-01	0.018	0.014	1.84E-01
3.211713	-0.019	0.015	1.98E-01	-0.007	0.022	7.48E-01
1.525849278	0.011	0.009	2.47E-01	-0.014	0.014	3.13E-01
1.135923905	0.008	0.007	2.06E-01	-0.009	0.010	3.45E-01
3.668252	-0.004	0.004	3.34E-01	0.006	0.006	3.44E-01
8.470491	-0.006	0.009	4.94E-01	0.000	0.013	9.82E-01
7.617298	-0.018	0.014	1.80E-01	0.001	0.020	9.49E-01
6.179048	-0.012	0.009	1.91E-01	-0.029	0.013	3.24E-02
1.408097871	0.009	0.006	1.41E-01	0.015	0.009	7.68E-02
5.496427	0.010	0.009	2.53E-01	0.011	0.013	3.99E-01
7.831605	-0.007	0.008	3.51E-01	0.014	0.011	2.12E-01
6.650391	0.007	0.009	4.48E-01	-0.016	0.013	2.22E-01
3.196574	0.005	0.005	3.37E-01	0.016	0.008	4.72E-02
5.325519	0.021	0.018	2.55E-01	-0.027	0.027	3.16E-01
6.211346	0.007	0.007	3.13E-01	0.005	0.011	6.71E-01
0.654152435	-0.012	0.009	2.03E-01	-0.019	0.014	1.55E-01
0.682412772	0.013	0.011	2.37E-01	-0.003	0.016	8.32E-01
7.907976	0.009	0.009	2.80E-01	-0.014	0.013	2.88E-01
8.413297	-0.008	0.009	3.50E-01	-0.004	0.013	7.40E-01
8.198317	0.010	0.008	1.81E-01	-0.007	0.012	5.75E-01
7.341087	0.010	0.009	2.67E-01	-0.003	0.013	8.36E-01

7.594084	-0.011	0.009	1.95E-01	-0.002	0.013	8.96E-01
2.634058233	-0.009	0.009	3.01E-01	-0.018	0.013	1.52E-01
8.938469	-0.009	0.008	2.86E-01	-0.009	0.012	4.67E-01
2.177182775	0.014	0.011	2.04E-01	-0.013	0.017	4.37E-01
4.449785	-0.016	0.016	3.15E-01	0.021	0.024	3.71E-01
8.812979	0.010	0.009	2.45E-01	-0.011	0.013	4.02E-01
7.009701	-0.013	0.009	1.60E-01	-0.038	0.013	5.12E-03
4.327323	-0.015	0.014	2.86E-01	0.024	0.021	2.52E-01
5.954648	0.010	0.008	2.36E-01	-0.022	0.012	7.52E-02
7.826222	-0.013	0.009	1.69E-01	0.000	0.014	9.81E-01
0.676020553	0.006	0.010	5.73E-01	-0.015	0.015	3.16E-01
5.316435	0.019	0.017	2.49E-01	-0.028	0.025	2.53E-01
8.351394	-0.009	0.010	3.58E-01	-0.001	0.014	9.38E-01
5.490035	0.008	0.009	3.70E-01	-0.013	0.013	3.19E-01
2.408311965	-0.018	0.015	2.28E-01	0.005	0.023	8.09E-01
1.445778322	0.005	0.004	2.08E-01	0.001	0.006	8.76E-01
1.119775141	0.008	0.007	2.38E-01	0.001	0.010	8.94E-01
8.935104	0.011	0.010	2.51E-01	-0.007	0.014	6.21E-01
4.131856	-0.012	0.013	3.53E-01	0.007	0.019	7.26E-01
7.562796	0.013	0.010	1.99E-01	-0.009	0.015	5.53E-01
2.65693565	-0.009	0.008	2.65E-01	0.023	0.012	6.82E-02
1.222050648	0.008	0.007	3.16E-01	0.000	0.011	9.67E-01
5.57818	0.012	0.009	1.95E-01	-0.029	0.013	3.05E-02
4.331361	-0.015	0.014	2.83E-01	0.007	0.021	7.52E-01
8.335918	0.013	0.009	1.85E-01	0.001	0.014	9.21E-01
5.862465	0.012	0.009	1.63E-01	0.021	0.013	1.04E-01
3.136016	-0.013	0.011	2.32E-01	-0.016	0.016	3.16E-01
7.003645	-0.007	0.008	3.90E-01	-0.018	0.012	1.30E-01
6.304201	0.006	0.008	4.65E-01	-0.005	0.011	6.73E-01
3.03946	-0.008	0.006	2.27E-01	0.010	0.009	2.59E-01
8.176112	-0.013	0.009	1.28E-01	-0.009	0.013	5.02E-01
6.897668	0.010	0.008	2.18E-01	-0.005	0.012	6.61E-01
5.258905	-0.009	0.005	9.65E-02	-0.010	0.008	2.04E-01
3.24973	-0.009	0.006	1.48E-01	0.003	0.009	7.11E-01
2.100812576	-0.005	0.007	4.90E-01	0.003	0.011	7.53E-01
6.966637	-0.012	0.010	2.29E-01	-0.005	0.015	7.63E-01
1.022209689	0.009	0.006	1.37E-01	0.000	0.009	9.88E-01
2.646169807	-0.012	0.011	2.85E-01	0.018	0.016	2.60E-01
1.662104478	0.004	0.004	3.20E-01	0.008	0.006	1.93E-01
6.323042	-0.010	0.008	2.57E-01	0.018	0.013	1.59E-01
8.92703	-0.011	0.009	2.35E-01	-0.011	0.013	3.94E-01
7.373721	-0.010	0.010	2.84E-01	-0.007	0.014	6.15E-01
4.146996	-0.014	0.014	3.24E-01	0.013	0.021	5.56E-01

0.780314656	0.005	0.004	2.86E-01	0.006	0.007	3.55E-01
8.967738	0.014	0.010	1.57E-01	0.002	0.015	9.02E-01
4.41345	-0.013	0.013	3.14E-01	0.008	0.019	6.83E-01
8.946543	0.008	0.009	4.10E-01	-0.012	0.014	3.88E-01
0.772576707	0.007	0.005	1.69E-01	0.001	0.007	9.22E-01
4.172901	-0.015	0.014	2.67E-01	0.005	0.021	7.93E-01
6.187796	-0.011	0.010	2.68E-01	-0.010	0.015	4.97E-01
6.604299	0.010	0.008	2.58E-01	0.005	0.013	7.12E-01
6.271231	-0.011	0.010	2.78E-01	-0.012	0.015	4.33E-01
7.889808	0.012	0.010	2.44E-01	0.028	0.015	6.38E-02
7.676846	0.007	0.008	4.15E-01	0.001	0.012	9.36E-01
5.07555	0.011	0.010	2.69E-01	0.001	0.015	9.67E-01
3.861028	-0.006	0.005	2.30E-01	0.007	0.008	3.94E-01
8.004532	0.009	0.007	1.97E-01	0.003	0.010	7.89E-01
2.347081233	0.014	0.009	1.31E-01	0.014	0.014	3.01E-01
1.965903107	0.005	0.005	2.66E-01	-0.001	0.007	8.43E-01
2.187275752	0.014	0.011	2.06E-01	-0.018	0.016	2.74E-01
4.318576	-0.015	0.014	2.81E-01	0.022	0.021	2.89E-01
6.267194	0.013	0.009	1.21E-01	0.009	0.013	4.71E-01
1.761015659	-0.012	0.008	1.55E-01	-0.015	0.012	2.35E-01
3.61375	-0.007	0.008	3.60E-01	0.011	0.011	3.37E-01
8.993307	0.006	0.008	4.58E-01	-0.003	0.011	8.05E-01
5.023739	0.008	0.008	3.13E-01	-0.009	0.012	4.57E-01
6.754348	-0.009	0.009	3.34E-01	0.006	0.014	6.82E-01
6.030009	0.012	0.009	2.18E-01	-0.002	0.014	9.05E-01
4.164154	-0.010	0.010	3.11E-01	0.001	0.015	9.49E-01
3.679691	-0.004	0.005	3.84E-01	0.010	0.007	1.31E-01
4.014105	-0.013	0.012	2.73E-01	0.009	0.017	5.92E-01
7.889472	0.012	0.009	1.74E-01	0.030	0.014	2.64E-02
0.731868363	-0.009	0.007	2.01E-01	0.013	0.010	2.19E-01
8.325488	-0.005	0.008	4.96E-01	-0.022	0.012	6.94E-02
7.222999	-0.009	0.008	2.74E-01	-0.013	0.012	2.88E-01
2.297962075	-0.007	0.006	2.77E-01	-0.004	0.009	6.79E-01
8.483948	0.008	0.009	3.64E-01	-0.006	0.013	6.67E-01
2.618918767	-0.016	0.013	2.37E-01	0.024	0.020	2.28E-01
8.689508	-0.007	0.007	3.28E-01	0.008	0.010	4.22E-01
6.774871	-0.010	0.010	2.80E-01	0.001	0.014	9.31E-01
2.29728921	-0.010	0.009	2.62E-01	-0.005	0.013	7.15E-01
3.196237	0.005	0.006	3.46E-01	0.016	0.008	5.22E-02
1.719298018	-0.008	0.006	1.78E-01	-0.009	0.008	2.78E-01
1.985079765	0.009	0.007	2.11E-01	-0.005	0.010	6.18E-01
4.411768	-0.016	0.017	3.23E-01	0.022	0.025	3.79E-01
5.936817	0.010	0.008	2.51E-01	-0.005	0.012	7.06E-01

4.048757	0.017	0.012	1.68E-01	-0.008	0.018	6.69E-01
7.983	0.010	0.009	2.46E-01	-0.013	0.013	3.39E-01
7.98771	0.005	0.007	4.56E-01	-0.011	0.011	3.24E-01
7.668099	0.010	0.010	3.06E-01	-0.006	0.015	6.77E-01
4.435655	-0.016	0.016	3.14E-01	0.013	0.024	5.73E-01
4.404367	-0.013	0.013	3.11E-01	0.006	0.019	7.51E-01
3.283373	-0.010	0.009	2.96E-01	0.003	0.014	8.47E-01
8.431465	0.007	0.008	3.55E-01	0.020	0.012	9.73E-02
1.752604845	-0.008	0.007	2.26E-01	0.001	0.010	9.34E-01
5.816374	0.012	0.009	1.93E-01	0.001	0.014	9.50E-01
7.177244	0.013	0.011	2.42E-01	0.018	0.016	2.81E-01
5.944555	0.011	0.009	2.14E-01	-0.001	0.014	9.68E-01
4.411432	-0.015	0.015	3.11E-01	0.019	0.022	3.76E-01
5.599039	0.009	0.008	2.65E-01	-0.001	0.012	9.37E-01
5.30937	0.018	0.016	2.50E-01	-0.023	0.023	3.34E-01
1.831330071	-0.014	0.010	1.81E-01	-0.003	0.016	8.46E-01
7.522424	0.005	0.006	4.21E-01	0.008	0.010	4.06E-01
2.563407389	-0.009	0.009	3.17E-01	0.007	0.014	6.03E-01
4.431954	-0.016	0.016	3.25E-01	0.012	0.025	6.22E-01
6.311266	0.012	0.010	2.30E-01	-0.014	0.015	3.44E-01
8.071145	-0.008	0.009	3.63E-01	0.002	0.013	8.66E-01
3.303896	-0.013	0.010	1.93E-01	0.000	0.015	9.99E-01
6.946451	0.008	0.008	3.53E-01	0.004	0.013	7.72E-01
3.327446	0.011	0.009	2.16E-01	0.016	0.014	2.40E-01
4.435318	-0.017	0.016	3.12E-01	0.016	0.024	5.23E-01
7.846408	0.011	0.010	2.85E-01	0.003	0.015	8.45E-01
3.281355	-0.010	0.008	2.07E-01	-0.024	0.012	4.63E-02
6.243307	0.007	0.008	3.68E-01	-0.007	0.012	5.39E-01
0.576100073	0.012	0.010	2.38E-01	0.023	0.016	1.36E-01
7.100201	-0.010	0.010	3.06E-01	-0.016	0.015	2.81E-01
0.750708588	-0.006	0.005	2.96E-01	-0.010	0.008	2.14E-01
5.061756	0.009	0.009	3.32E-01	-0.010	0.014	4.68E-01
3.114484	-0.011	0.010	2.53E-01	0.014	0.015	3.42E-01
6.221775	0.008	0.007	2.66E-01	-0.012	0.011	2.88E-01
2.660636408	-0.014	0.012	2.66E-01	-0.004	0.018	8.35E-01
1.722325911	-0.006	0.005	2.41E-01	-0.007	0.007	3.70E-01
5.716454	0.005	0.008	5.36E-01	-0.025	0.013	4.86E-02
6.979422	-0.010	0.008	2.17E-01	-0.019	0.012	1.23E-01
7.890145	0.009	0.008	2.34E-01	0.021	0.011	6.46E-02
2.086682408	-0.006	0.006	2.81E-01	0.008	0.009	3.74E-01
8.657211	0.007	0.009	4.33E-01	0.010	0.014	4.73E-01
8.798176	0.008	0.009	3.58E-01	-0.007	0.013	5.71E-01
2.800592366	-0.014	0.014	3.12E-01	-0.066	0.021	1.91E-03

5.044598	0.007	0.007	3.11E-01	0.001	0.010	9.53E-01
5.610814	0.009	0.009	3.28E-01	-0.013	0.013	3.35E-01
7.066894	0.006	0.006	3.44E-01	0.003	0.009	7.66E-01
5.316772	0.019	0.017	2.55E-01	-0.030	0.025	2.23E-01
8.438866	0.007	0.007	3.23E-01	0.004	0.010	6.67E-01
0.576436506	0.010	0.008	2.33E-01	0.019	0.012	1.23E-01
0.615126254	0.008	0.008	3.10E-01	0.003	0.012	7.69E-01
7.758263	-0.015	0.011	1.65E-01	0.008	0.017	6.29E-01
3.334847	0.011	0.009	2.25E-01	0.016	0.014	2.49E-01
6.907089	0.010	0.008	2.17E-01	0.009	0.011	4.16E-01
6.118827	-0.009	0.008	2.94E-01	0.014	0.012	2.74E-01
5.258569	-0.009	0.005	9.70E-02	-0.010	0.008	2.06E-01
6.638615	0.007	0.009	4.23E-01	0.010	0.013	4.47E-01
3.941099	-0.008	0.008	3.17E-01	0.015	0.012	2.08E-01
4.344818	-0.012	0.013	3.52E-01	0.014	0.019	4.66E-01
8.143142	0.014	0.010	1.75E-01	0.017	0.016	2.72E-01
1.427274529	0.006	0.005	1.71E-01	0.006	0.007	3.74E-01
5.662961	0.008	0.008	2.69E-01	0.010	0.011	3.64E-01
6.056251	0.008	0.008	3.65E-01	-0.020	0.013	1.03E-01
4.154397	-0.014	0.013	2.82E-01	0.007	0.020	7.18E-01
2.553987277	-0.013	0.012	2.89E-01	-0.007	0.018	6.84E-01
4.152715	-0.016	0.017	3.54E-01	0.012	0.026	6.38E-01
6.805486	0.005	0.009	5.47E-01	-0.005	0.013	7.22E-01
8.828455	0.007	0.009	4.67E-01	0.014	0.014	3.26E-01
3.228198	0.005	0.006	4.46E-01	0.021	0.009	2.47E-02
7.775421	-0.011	0.009	2.63E-01	-0.015	0.014	2.78E-01
8.20269	0.008	0.009	3.45E-01	-0.029	0.013	2.41E-02
5.862802	0.011	0.009	2.25E-01	-0.002	0.014	8.67E-01
5.51022	0.006	0.008	4.70E-01	-0.014	0.013	2.71E-01
7.396262	0.006	0.008	4.28E-01	0.001	0.011	9.61E-01
7.487098	0.013	0.009	1.68E-01	-0.001	0.014	9.59E-01
0.816649376	0.004	0.005	4.74E-01	0.015	0.008	5.34E-02
2.87158	-0.008	0.010	4.12E-01	0.001	0.014	9.25E-01
5.044934	0.007	0.007	3.10E-01	0.004	0.010	6.98E-01
7.556067	0.011	0.008	1.87E-01	0.002	0.012	8.79E-01
3.881214	0.005	0.006	3.41E-01	0.021	0.009	1.20E-02
1.726363103	-0.005	0.005	2.49E-01	0.008	0.007	2.69E-01
4.343809	-0.014	0.014	3.19E-01	0.013	0.021	5.28E-01
6.002085	0.012	0.010	2.24E-01	0.010	0.015	4.98E-01
7.557413	-0.008	0.008	3.23E-01	-0.005	0.011	6.62E-01
8.264594	0.009	0.009	3.01E-01	0.001	0.013	9.14E-01
4.17391	-0.013	0.013	2.99E-01	-0.001	0.019	9.75E-01
1.525512846	0.011	0.009	2.51E-01	-0.013	0.014	3.42E-01

6.741564	-0.011	0.011	3.44E-01	-0.017	0.017	3.01E-01
6.609346	0.009	0.009	3.32E-01	0.003	0.013	8.31E-01
7.968533	0.008	0.010	4.04E-01	-0.001	0.015	9.63E-01
3.323745	0.010	0.010	3.02E-01	0.025	0.015	9.04E-02
2.794873012	-0.014	0.012	2.52E-01	-0.058	0.018	9.39E-04
5.119622	0.008	0.006	2.27E-01	-0.005	0.010	5.92E-01
2.756519697	-0.010	0.007	1.62E-01	-0.024	0.011	2.59E-02
2.139165892	-0.013	0.011	2.46E-01	0.032	0.017	5.67E-02
5.546892	0.008	0.008	3.15E-01	-0.002	0.013	8.72E-01
4.433636	-0.015	0.017	3.66E-01	0.020	0.025	4.20E-01
3.875494	-0.005	0.004	2.74E-01	0.012	0.007	7.66E-02
5.572124	-0.008	0.008	3.12E-01	-0.028	0.012	1.45E-02
7.737741	-0.007	0.011	5.08E-01	0.000	0.017	9.91E-01
6.626167	0.008	0.010	4.18E-01	-0.005	0.014	7.08E-01
7.199448	-0.011	0.009	2.06E-01	-0.001	0.013	9.46E-01
1.520129924	0.010	0.009	2.66E-01	-0.012	0.013	3.43E-01
5.656905	0.007	0.008	3.63E-01	-0.013	0.012	2.67E-01
7.29264	-0.014	0.011	2.05E-01	-0.010	0.017	5.33E-01
8.564356	-0.012	0.009	1.82E-01	-0.007	0.013	5.96E-01
1.494224615	-0.005	0.004	2.15E-01	-0.010	0.006	1.31E-01
1.686664057	-0.004	0.004	2.77E-01	0.006	0.006	3.34E-01
6.01857	0.007	0.009	4.70E-01	-0.016	0.014	2.46E-01
2.298298507	-0.006	0.006	2.86E-01	-0.006	0.008	5.05E-01
3.789704	0.011	0.007	1.30E-01	0.018	0.011	9.57E-02
1.773127233	-0.010	0.010	3.15E-01	0.016	0.015	2.80E-01
0.603014681	0.008	0.008	3.42E-01	-0.029	0.013	2.06E-02
5.10381	0.008	0.007	2.37E-01	0.000	0.011	9.79E-01
5.517622	0.012	0.010	2.44E-01	-0.015	0.015	3.19E-01
0.725476144	0.008	0.007	2.55E-01	0.013	0.011	2.38E-01
2.358856374	0.010	0.008	2.36E-01	0.015	0.013	2.39E-01
7.486762	0.011	0.009	2.41E-01	0.002	0.014	8.88E-01
7.339068	-0.013	0.011	2.30E-01	-0.004	0.017	8.23E-01
6.649045	-0.010	0.009	2.49E-01	-0.001	0.013	9.43E-01
8.358122	0.006	0.007	3.97E-01	0.005	0.011	6.42E-01
6.567628	-0.008	0.008	3.54E-01	-0.025	0.012	4.77E-02
1.724344507	-0.005	0.005	2.55E-01	0.001	0.007	8.71E-01
6.383936	0.008	0.008	3.18E-01	0.015	0.011	1.78E-01
7.519059	-0.010	0.010	3.33E-01	-0.018	0.015	2.38E-01
7.912349	-0.007	0.007	3.37E-01	-0.007	0.011	4.89E-01
0.98856643	0.008	0.007	2.88E-01	0.011	0.011	3.02E-01
7.647577	-0.007	0.011	5.32E-01	-0.012	0.016	4.65E-01
7.74817	-0.008	0.010	4.34E-01	0.004	0.015	7.64E-01
4.373415	-0.013	0.014	3.36E-01	0.011	0.020	6.01E-01

3.155865	-0.010	0.009	2.51E-01	0.007	0.013	6.07E-01
6.165591	0.006	0.007	3.72E-01	-0.006	0.010	5.68E-01
3.202293	0.006	0.006	3.14E-01	0.019	0.009	3.96E-02
3.065028	-0.007	0.008	3.60E-01	0.004	0.012	7.51E-01
4.15978	-0.014	0.013	2.76E-01	0.008	0.019	6.62E-01
8.247772	-0.006	0.008	4.18E-01	0.003	0.011	8.05E-01
4.334389	-0.014	0.014	3.25E-01	0.023	0.021	2.59E-01
8.880602	0.008	0.008	3.21E-01	0.013	0.012	2.62E-01
8.052305	0.008	0.006	1.98E-01	0.012	0.009	1.66E-01
6.904734	0.008	0.009	3.29E-01	0.016	0.013	2.21E-01
5.725537	0.007	0.009	4.77E-01	-0.001	0.014	9.47E-01
6.346255	0.006	0.006	3.04E-01	0.002	0.009	8.39E-01
1.23786298	0.008	0.007	3.08E-01	-0.006	0.011	5.69E-01
6.62684	0.009	0.008	2.99E-01	-0.014	0.012	2.69E-01
6.326069	0.008	0.008	2.98E-01	-0.006	0.012	5.85E-01
6.518845	0.008	0.007	2.55E-01	-0.008	0.011	4.58E-01
8.097387	0.011	0.008	1.49E-01	-0.014	0.012	2.36E-01
8.957982	0.010	0.008	2.33E-01	-0.017	0.012	1.66E-01
1.431648153	0.006	0.004	1.47E-01	0.008	0.007	2.21E-01
7.4945	-0.008	0.009	3.62E-01	-0.009	0.014	5.12E-01
6.730125	-0.013	0.010	1.75E-01	-0.017	0.015	2.54E-01
4.444738	-0.015	0.017	3.53E-01	0.030	0.025	2.31E-01
8.075183	0.009	0.008	2.53E-01	-0.001	0.012	9.65E-01
7.977617	0.006	0.009	4.98E-01	-0.002	0.013	8.69E-01
3.572369	-0.005	0.006	4.11E-01	0.009	0.008	2.80E-01
8.876565	0.008	0.007	3.01E-01	0.004	0.011	6.93E-01
0.519915831	0.009	0.009	3.31E-01	-0.009	0.013	5.00E-01
0.740952043	-0.006	0.006	3.23E-01	0.000	0.010	9.94E-01
0.774595302	0.006	0.005	2.24E-01	-0.001	0.007	8.81E-01
7.37944	-0.010	0.011	3.41E-01	-0.018	0.016	2.59E-01
7.995112	0.007	0.009	4.69E-01	0.005	0.014	7.09E-01
3.695839	0.006	0.005	2.76E-01	0.020	0.008	1.17E-02
8.697919	0.005	0.007	4.29E-01	-0.022	0.010	3.69E-02
8.193607	0.009	0.008	2.78E-01	0.012	0.013	3.43E-01
6.951834	-0.009	0.009	3.36E-01	-0.005	0.013	7.18E-01
1.626442623	0.006	0.006	2.75E-01	-0.006	0.009	4.75E-01
3.175715	-0.006	0.006	2.67E-01	0.004	0.008	6.62E-01
7.583318	0.013	0.010	2.01E-01	0.018	0.015	2.14E-01
6.010496	-0.010	0.010	3.10E-01	0.004	0.014	7.95E-01
5.469176	-0.008	0.008	2.70E-01	-0.007	0.011	5.39E-01
7.553376	0.011	0.010	2.59E-01	0.007	0.015	6.36E-01
3.226852	-0.008	0.006	2.34E-01	0.010	0.010	3.14E-01
6.709603	-0.013	0.010	2.19E-01	-0.036	0.016	2.11E-02

1.685991192	-0.005	0.004	2.20E-01	0.005	0.006	3.81E-01
0.770894544	0.006	0.005	2.44E-01	-0.004	0.008	6.17E-01
5.744714	0.010	0.010	3.08E-01	0.011	0.015	4.55E-01
8.923329	-0.008	0.009	3.71E-01	0.002	0.013	8.72E-01
6.323714	0.006	0.007	4.27E-01	0.013	0.011	2.42E-01
8.874546	0.009	0.009	3.18E-01	-0.008	0.014	5.52E-01
2.177519207	0.015	0.011	1.90E-01	-0.013	0.017	4.48E-01
6.226149	0.010	0.007	1.96E-01	0.003	0.011	7.76E-01
2.847020064	-0.005	0.008	4.89E-01	-0.007	0.012	5.63E-01
0.835489601	-0.011	0.009	1.84E-01	-0.008	0.013	5.17E-01
4.31252	-0.013	0.011	2.46E-01	0.015	0.017	3.69E-01
7.280529	-0.012	0.009	2.08E-01	-0.017	0.014	2.26E-01
5.550592	0.009	0.008	3.00E-01	-0.001	0.012	9.58E-01
7.388187	-0.008	0.008	2.98E-01	-0.019	0.012	1.16E-01
1.968594568	0.005	0.004	2.94E-01	-0.001	0.007	8.47E-01
6.539704	0.008	0.009	3.73E-01	0.019	0.014	1.73E-01
0.575427208	-0.008	0.008	3.68E-01	0.002	0.013	8.87E-01
2.41672278	-0.007	0.009	4.62E-01	0.011	0.014	4.33E-01
7.130143	-0.012	0.011	2.79E-01	-0.027	0.016	9.88E-02
6.802122	0.009	0.010	3.62E-01	0.010	0.015	5.13E-01
7.716545	-0.012	0.010	2.40E-01	0.004	0.015	7.85E-01
5.867175	0.014	0.010	1.70E-01	0.021	0.015	1.61E-01
7.961805	0.008	0.008	3.29E-01	0.017	0.012	1.71E-01
7.152684	0.006	0.007	4.31E-01	-0.005	0.011	6.66E-01
7.762637	-0.010	0.011	3.61E-01	-0.023	0.016	1.54E-01
5.15663	0.011	0.009	2.18E-01	-0.002	0.013	8.91E-01
1.124148764	0.008	0.007	2.28E-01	-0.006	0.010	5.25E-01
5.410636	0.009	0.008	2.75E-01	-0.017	0.012	1.63E-01
5.503155	0.008	0.009	3.47E-01	-0.009	0.013	4.83E-01
1.729727428	-0.007	0.005	2.12E-01	0.002	0.008	8.08E-01
5.602739	0.012	0.010	2.40E-01	0.006	0.015	6.95E-01
0.625892097	0.011	0.008	1.79E-01	-0.002	0.012	8.57E-01
3.236273	-0.006	0.006	3.46E-01	0.006	0.009	4.99E-01
3.2827	-0.009	0.008	3.01E-01	-0.018	0.013	1.50E-01
3.064356	-0.006	0.008	4.60E-01	0.008	0.012	4.99E-01
1.52483998	0.011	0.009	2.29E-01	-0.016	0.014	2.65E-01
6.420607	0.007	0.009	4.42E-01	-0.004	0.013	7.55E-01
8.111181	0.007	0.009	4.02E-01	-0.019	0.013	1.48E-01
3.326437	0.010	0.009	2.24E-01	0.019	0.013	1.44E-01
6.915499	-0.012	0.009	2.15E-01	-0.029	0.014	4.00E-02
7.439325	-0.010	0.010	3.21E-01	-0.001	0.014	9.43E-01
7.812429	0.006	0.008	4.47E-01	0.006	0.012	6.18E-01
1.864636897	-0.007	0.009	4.24E-01	-0.006	0.013	6.23E-01

7.28423	-0.015	0.010	1.34E-01	0.008	0.015	6.06E-01
8.550225	0.008	0.007	3.10E-01	0.003	0.011	7.55E-01
8.942169	0.009	0.009	3.15E-01	-0.024	0.014	8.31E-02
8.070472	-0.007	0.008	4.03E-01	0.003	0.012	7.75E-01
0.630602153	0.006	0.010	5.59E-01	-0.004	0.014	7.71E-01
5.686511	0.007	0.007	2.94E-01	0.000	0.010	9.91E-01
0.718074627	0.007	0.008	3.51E-01	0.014	0.011	2.12E-01
0.901093956	0.010	0.007	1.85E-01	-0.015	0.011	1.65E-01
8.676388	0.011	0.009	2.04E-01	-0.021	0.013	1.01E-01
5.070167	0.006	0.007	3.59E-01	-0.006	0.010	5.84E-01
3.162257	-0.009	0.007	2.12E-01	0.002	0.011	8.25E-01
5.068484	0.008	0.008	3.12E-01	-0.008	0.012	5.10E-01
7.092463	-0.009	0.011	4.20E-01	-0.002	0.016	9.11E-01
6.815242	0.009	0.009	3.36E-01	-0.005	0.014	7.07E-01
8.865126	0.007	0.009	4.52E-01	0.009	0.014	5.06E-01
7.754226	-0.008	0.010	4.40E-01	-0.012	0.015	4.16E-01
7.708471	-0.009	0.010	3.62E-01	-0.012	0.015	4.08E-01
7.518387	-0.012	0.009	1.71E-01	-0.004	0.013	7.87E-01
5.561695	-0.008	0.009	3.69E-01	-0.019	0.014	1.54E-01
5.478596	0.007	0.007	2.99E-01	-0.022	0.010	3.38E-02
1.051479325	0.009	0.006	1.74E-01	-0.008	0.009	3.81E-01
7.474987	0.010	0.008	1.78E-01	-0.013	0.011	2.37E-01
6.005786	0.010	0.009	2.58E-01	0.007	0.013	6.16E-01
5.634364	-0.010	0.009	2.97E-01	-0.008	0.014	5.49E-01
5.131397	0.009	0.008	2.71E-01	-0.006	0.013	6.39E-01
0.502421336	0.008	0.008	2.80E-01	-0.009	0.012	4.20E-01
7.69535	-0.009	0.010	3.49E-01	-0.013	0.015	3.76E-01
1.767744311	-0.008	0.009	3.88E-01	0.014	0.014	3.32E-01
8.615493	0.008	0.009	3.73E-01	0.014	0.013	2.64E-01
1.907363837	-0.008	0.006	1.97E-01	-0.008	0.009	3.66E-01
1.91039173	-0.014	0.011	1.95E-01	-0.036	0.016	2.59E-02
5.325183	0.020	0.018	2.68E-01	-0.026	0.027	3.38E-01
3.235936	-0.006	0.006	3.34E-01	0.007	0.009	4.89E-01
4.109315	-0.008	0.009	4.01E-01	0.005	0.014	7.42E-01
5.479269	0.008	0.007	2.56E-01	-0.021	0.010	4.48E-02
6.647026	-0.009	0.008	2.86E-01	0.001	0.012	9.17E-01
3.595583	-0.008	0.007	2.28E-01	0.015	0.010	1.47E-01
4.42758	-0.014	0.015	3.37E-01	0.007	0.022	7.65E-01
2.858122	-0.007	0.008	3.91E-01	-0.017	0.011	1.44E-01
1.786920969	0.010	0.008	2.19E-01	0.007	0.012	5.58E-01
1.870692684	-0.009	0.008	2.98E-01	0.003	0.012	8.35E-01
6.937031	-0.009	0.009	3.51E-01	0.012	0.014	3.75E-01
7.48508	-0.011	0.011	2.92E-01	-0.016	0.016	3.35E-01

7.275146	-0.015	0.010	1.45E-01	0.003	0.015	8.50E-01
2.646506239	-0.013	0.012	2.78E-01	0.026	0.017	1.35E-01
8.764196	0.008	0.009	3.63E-01	0.016	0.014	2.52E-01
8.341637	-0.009	0.008	2.44E-01	-0.012	0.012	3.06E-01
3.616105	-0.005	0.006	4.19E-01	0.004	0.009	6.20E-01
8.456024	0.008	0.009	3.62E-01	0.003	0.013	8.25E-01
8.185869	0.010	0.010	3.19E-01	-0.003	0.015	8.41E-01
3.121885	-0.010	0.008	2.12E-01	-0.002	0.013	8.45E-01
4.438683	-0.016	0.016	3.08E-01	0.026	0.023	2.66E-01
1.116747247	0.010	0.007	1.74E-01	-0.017	0.010	1.08E-01
0.618154147	0.008	0.008	3.33E-01	0.014	0.012	2.68E-01
5.726547	0.009	0.010	3.65E-01	0.012	0.015	4.21E-01
5.284138	-0.010	0.008	1.97E-01	-0.025	0.012	2.83E-02
7.133844	0.011	0.010	2.70E-01	-0.024	0.014	8.79E-02
2.129745779	-0.011	0.012	3.37E-01	-0.008	0.017	6.41E-01
8.846286	0.009	0.009	2.85E-01	0.002	0.013	9.02E-01
3.2356	-0.006	0.006	3.24E-01	0.007	0.009	4.34E-01
7.260679	-0.013	0.009	1.54E-01	-0.020	0.014	1.57E-01
1.680271838	-0.005	0.004	2.28E-01	0.000	0.006	9.93E-01
2.356164913	-0.004	0.007	5.27E-01	0.026	0.010	1.12E-02
2.615218008	-0.014	0.014	3.18E-01	0.024	0.020	2.44E-01
6.2968	-0.009	0.008	3.08E-01	-0.001	0.013	9.31E-01
2.983612	-0.004	0.004	3.24E-01	-0.001	0.006	8.41E-01
3.741931	0.004	0.008	6.36E-01	0.021	0.012	8.55E-02
5.124332	0.007	0.008	3.49E-01	-0.010	0.012	3.82E-01
4.329342	-0.014	0.013	2.88E-01	0.014	0.020	4.93E-01
5.780039	0.005	0.007	4.64E-01	0.020	0.011	7.50E-02
8.481593	0.010	0.008	2.34E-01	0.008	0.013	4.97E-01
0.619499878	-0.007	0.009	4.46E-01	0.017	0.013	1.88E-01
2.838945681	-0.006	0.009	4.89E-01	-0.015	0.013	2.36E-01
1.054507218	0.007	0.006	2.74E-01	-0.005	0.009	6.01E-01
6.898678	0.008	0.008	3.02E-01	-0.001	0.012	9.18E-01
3.124241	-0.011	0.008	1.84E-01	0.005	0.012	6.76E-01
5.002544	0.009	0.008	2.72E-01	0.001	0.012	9.66E-01
8.441894	-0.008	0.009	3.46E-01	-0.018	0.013	1.57E-01
5.057719	0.009	0.011	3.92E-01	0.000	0.016	9.87E-01
8.857052	0.012	0.009	1.73E-01	0.007	0.013	5.97E-01
2.030161732	0.007	0.006	1.88E-01	0.006	0.008	4.93E-01
8.781018	0.007	0.006	2.41E-01	0.006	0.009	5.26E-01
5.734957	0.009	0.010	3.50E-01	0.001	0.015	9.23E-01
7.085734	0.008	0.006	2.02E-01	0.006	0.009	5.25E-01
4.107633	-0.010	0.012	3.79E-01	0.010	0.017	5.72E-01
3.039123	-0.008	0.006	2.27E-01	0.010	0.010	3.06E-01

5.422412	0.012	0.009	1.84E-01	-0.005	0.013	7.24E-01
8.815334	-0.007	0.008	4.08E-01	-0.016	0.012	1.97E-01
8.899779	-0.008	0.010	4.21E-01	-0.024	0.014	9.89E-02
8.092004	-0.009	0.010	3.79E-01	-0.008	0.015	5.98E-01
5.113566	0.010	0.009	3.02E-01	0.002	0.014	8.97E-01
5.940854	0.006	0.006	3.34E-01	0.006	0.009	5.23E-01
7.949357	0.008	0.010	3.97E-01	-0.007	0.014	6.48E-01
7.211224	-0.011	0.010	2.81E-01	-0.036	0.015	1.77E-02
1.880449229	-0.006	0.007	3.37E-01	-0.003	0.010	7.81E-01
5.72621	0.011	0.010	2.82E-01	0.016	0.015	2.61E-01
6.833073	0.008	0.008	3.57E-01	0.016	0.012	1.85E-01
3.255113	-0.010	0.010	3.27E-01	0.004	0.015	7.79E-01
8.933086	-0.007	0.007	3.27E-01	-0.001	0.011	9.55E-01
3.216423	-0.016	0.014	2.64E-01	-0.007	0.021	7.24E-01
5.807963	0.006	0.008	4.21E-01	-0.018	0.012	1.18E-01
8.573103	-0.009	0.009	3.10E-01	0.003	0.014	8.53E-01
7.407027	-0.010	0.009	2.67E-01	-0.019	0.013	1.52E-01
6.970674	-0.015	0.010	1.61E-01	-0.013	0.016	4.10E-01
5.600384	0.011	0.008	1.81E-01	-0.008	0.012	5.22E-01
3.990554	-0.008	0.008	3.56E-01	0.005	0.012	6.82E-01
7.123415	0.009	0.008	2.41E-01	0.002	0.012	8.93E-01
8.45266	0.018	0.014	2.15E-01	0.025	0.021	2.41E-01
7.829923	-0.012	0.010	2.30E-01	0.010	0.015	5.18E-01
3.617787	-0.005	0.006	3.77E-01	-0.002	0.009	8.00E-01
5.147546	0.008	0.008	2.93E-01	-0.006	0.012	6.03E-01
2.493765843	-0.021	0.018	2.44E-01	0.003	0.027	9.09E-01
8.144151	0.005	0.007	4.72E-01	-0.001	0.010	9.12E-01
1.86497333	-0.008	0.009	3.62E-01	-0.009	0.013	4.73E-01
6.075764	0.009	0.008	2.27E-01	-0.018	0.011	1.18E-01
1.530559334	0.012	0.010	2.46E-01	-0.017	0.015	2.64E-01
3.764135	0.011	0.009	2.42E-01	0.019	0.014	1.61E-01
5.047626	0.008	0.009	3.72E-01	-0.001	0.013	9.66E-01
7.028204	-0.015	0.010	1.47E-01	-0.016	0.015	2.95E-01
7.943301	0.008	0.009	3.70E-01	-0.003	0.014	8.26E-01
8.284107	-0.005	0.008	4.90E-01	-0.007	0.012	5.75E-01
0.628583558	0.007	0.008	3.83E-01	-0.005	0.011	6.76E-01
4.439692	-0.015	0.015	3.29E-01	0.017	0.022	4.35E-01
6.405804	-0.012	0.009	1.75E-01	-0.008	0.013	5.24E-01
2.17011769	0.012	0.010	2.43E-01	-0.014	0.015	3.49E-01
2.143539516	-0.014	0.012	2.53E-01	0.012	0.018	5.07E-01
8.4281	0.011	0.010	2.64E-01	0.014	0.014	3.34E-01
0.732877661	-0.009	0.007	1.99E-01	0.000	0.010	9.65E-01
6.97202	-0.011	0.009	2.06E-01	-0.008	0.013	5.36E-01

3.745295	-0.014	0.011	1.86E-01	-0.015	0.016	3.26E-01
7.452109	0.008	0.010	4.46E-01	0.014	0.015	3.62E-01
5.32451	0.020	0.018	2.75E-01	-0.026	0.027	3.31E-01
3.688774	-0.005	0.004	2.77E-01	0.002	0.007	8.16E-01
7.401644	-0.009	0.010	3.93E-01	-0.027	0.015	7.84E-02
8.740646	-0.008	0.006	1.92E-01	-0.010	0.010	2.86E-01
7.241839	-0.012	0.010	2.35E-01	-0.023	0.015	1.21E-01
1.009761683	0.011	0.009	2.07E-01	0.025	0.013	5.33E-02
5.082615	0.014	0.012	2.56E-01	-0.010	0.018	5.76E-01
7.256642	-0.014	0.011	1.83E-01	-0.009	0.016	5.64E-01
2.621273795	-0.011	0.012	3.57E-01	0.020	0.018	2.77E-01
4.413114	-0.012	0.012	3.27E-01	0.009	0.018	6.21E-01
6.410851	-0.015	0.009	1.10E-01	-0.031	0.014	2.27E-02
5.489025	0.009	0.008	2.75E-01	-0.019	0.012	9.76E-02
3.934707	-0.004	0.006	5.34E-01	0.014	0.009	1.28E-01
1.465627844	-0.005	0.006	3.99E-01	0.008	0.009	3.63E-01
1.237526548	0.007	0.008	3.18E-01	-0.007	0.011	5.61E-01
5.245448	-0.008	0.005	8.84E-02	-0.005	0.007	4.84E-01
6.789674	0.012	0.009	1.77E-01	0.009	0.014	5.13E-01
5.119286	0.008	0.007	2.36E-01	-0.003	0.010	7.44E-01
7.931189	0.012	0.010	2.63E-01	-0.002	0.016	8.77E-01
6.750311	-0.007	0.013	5.85E-01	0.030	0.019	1.12E-01
8.053987	0.010	0.010	3.11E-01	0.014	0.015	3.54E-01
8.602036	0.009	0.009	3.21E-01	-0.024	0.014	8.23E-02
1.983397602	0.008	0.006	2.35E-01	-0.005	0.009	6.17E-01
2.386443846	-0.017	0.013	1.82E-01	0.008	0.019	6.95E-01
8.539796	0.007	0.009	4.32E-01	0.018	0.013	1.73E-01
2.916662	-0.008	0.008	2.92E-01	-0.001	0.012	9.50E-01
6.962264	-0.009	0.009	3.07E-01	-0.010	0.013	4.26E-01
2.652562026	-0.013	0.012	2.91E-01	0.015	0.018	4.04E-01
2.357847076	0.010	0.008	1.74E-01	0.012	0.011	2.93E-01
6.796739	0.013	0.011	2.54E-01	0.035	0.016	3.32E-02
7.575917	0.010	0.007	1.50E-01	-0.002	0.010	8.42E-01
7.392224	0.008	0.008	2.81E-01	0.003	0.011	8.00E-01
7.93018	0.015	0.011	1.82E-01	0.022	0.016	1.73E-01
1.081421825	0.008	0.007	2.33E-01	0.003	0.010	7.69E-01
6.148433	0.010	0.009	2.93E-01	-0.003	0.014	8.08E-01
1.968258136	0.005	0.004	2.95E-01	-0.001	0.007	8.78E-01
5.971806	0.013	0.009	1.42E-01	-0.022	0.013	9.25E-02
6.199571	0.012	0.009	1.93E-01	0.018	0.014	1.99E-01
4.2994	-0.013	0.014	3.47E-01	0.008	0.021	7.09E-01
3.807199	0.005	0.006	3.58E-01	0.017	0.009	5.00E-02
3.939753	-0.007	0.007	3.57E-01	0.006	0.011	6.13E-01

8.503461	0.009	0.007	2.21E-01	0.008	0.011	4.53E-01
3.280682	-0.013	0.009	1.57E-01	-0.011	0.013	4.03E-01
5.553957	0.008	0.007	2.59E-01	-0.012	0.011	2.83E-01
0.956268901	-0.009	0.006	1.74E-01	0.011	0.010	2.64E-01
8.607755	0.006	0.007	4.63E-01	-0.005	0.011	6.26E-01
5.538817	0.008	0.008	3.40E-01	0.006	0.012	6.06E-01
3.438805	0.008	0.008	2.91E-01	0.023	0.012	5.00E-02
2.844665035	-0.009	0.008	3.15E-01	-0.010	0.013	4.50E-01
2.295607047	-0.009	0.008	2.93E-01	0.004	0.012	7.63E-01
0.946512356	-0.006	0.005	2.64E-01	-0.005	0.008	4.94E-01
3.556556	-0.022	0.020	2.77E-01	-0.029	0.030	3.28E-01
3.17706	-0.006	0.006	2.99E-01	0.008	0.008	3.28E-01
5.057046	0.006	0.007	3.68E-01	0.002	0.011	8.47E-01
7.415102	-0.009	0.008	2.90E-01	-0.033	0.012	8.48E-03
3.613077	-0.008	0.008	3.45E-01	0.011	0.012	3.60E-01
6.147424	0.007	0.006	2.92E-01	0.004	0.009	6.67E-01
5.832859	0.008	0.008	3.09E-01	-0.012	0.012	3.20E-01
8.404886	0.008	0.009	3.53E-01	-0.009	0.013	5.13E-01
8.253155	0.011	0.009	2.34E-01	0.015	0.014	2.68E-01
5.152593	0.008	0.008	3.14E-01	-0.013	0.012	2.77E-01
0.852311231	-0.008	0.007	2.43E-01	-0.013	0.010	2.12E-01
6.515481	0.012	0.009	2.18E-01	-0.025	0.014	7.85E-02
2.775696354	-0.013	0.011	2.47E-01	-0.048	0.017	3.66E-03
3.621488	-0.006	0.006	2.55E-01	-0.007	0.008	3.90E-01
1.516429166	0.009	0.008	2.80E-01	-0.019	0.012	1.15E-01
5.284474	-0.010	0.008	2.01E-01	-0.026	0.012	2.90E-02
6.952843	-0.009	0.009	3.28E-01	-0.015	0.013	2.70E-01
1.788603132	-0.008	0.008	3.07E-01	0.003	0.012	8.14E-01
1.838058723	-0.007	0.010	4.92E-01	-0.016	0.015	2.97E-01
4.396292	-0.014	0.014	3.12E-01	0.014	0.020	5.02E-01
3.646047	0.007	0.005	1.33E-01	0.017	0.007	1.91E-02
7.201467	0.013	0.011	2.32E-01	0.009	0.017	5.96E-01
1.519120626	0.009	0.009	2.89E-01	-0.010	0.013	4.47E-01
2.295270614	-0.007	0.007	3.05E-01	0.001	0.010	8.88E-01
1.519793492	0.009	0.009	2.87E-01	-0.014	0.013	2.75E-01
4.429263	-0.014	0.015	3.40E-01	0.020	0.022	3.69E-01
2.335642525	0.011	0.008	1.98E-01	0.008	0.012	4.95E-01
1.906690971	-0.008	0.006	2.09E-01	-0.004	0.009	6.42E-01
2.498812332	-0.016	0.013	2.20E-01	-0.003	0.020	8.97E-01
2.621610228	-0.010	0.012	4.02E-01	0.021	0.019	2.70E-01
0.957278199	-0.007	0.005	2.10E-01	0.008	0.008	3.02E-01
3.2393	-0.007	0.006	2.00E-01	0.008	0.008	3.52E-01
7.688958	-0.011	0.008	1.60E-01	-0.001	0.012	9.48E-01

5.306006	0.017	0.015	2.49E-01	-0.023	0.022	2.84E-01
4.13152	-0.013	0.014	3.60E-01	0.017	0.020	3.97E-01
3.370173	0.021	0.013	1.10E-01	0.021	0.019	2.82E-01
1.222387081	0.007	0.008	3.37E-01	0.000	0.011	9.84E-01
3.174369	-0.007	0.006	2.48E-01	0.002	0.009	7.85E-01
7.074968	0.009	0.009	3.38E-01	0.008	0.014	5.43E-01
8.158618	-0.010	0.009	2.79E-01	-0.005	0.013	6.75E-01
4.234468	0.012	0.013	3.61E-01	0.015	0.020	4.57E-01
7.747497	-0.007	0.010	4.37E-01	-0.003	0.014	8.60E-01
6.135312	0.006	0.011	5.80E-01	-0.020	0.016	2.02E-01
8.169384	0.010	0.008	2.43E-01	-0.018	0.013	1.50E-01
8.637361	-0.006	0.007	3.76E-01	0.006	0.011	6.13E-01
2.167762662	0.012	0.011	2.53E-01	-0.011	0.016	4.96E-01
6.212355	0.007	0.007	2.87E-01	-0.002	0.010	8.76E-01
1.79095816	-0.007	0.009	4.52E-01	0.008	0.013	5.44E-01
3.284046	-0.010	0.010	3.34E-01	-0.004	0.015	8.01E-01
7.941955	0.009	0.009	3.13E-01	0.004	0.013	7.71E-01
2.605461463	-0.014	0.013	2.70E-01	0.015	0.019	4.28E-01
5.531079	0.005	0.007	4.89E-01	0.008	0.011	4.49E-01
8.522638	0.005	0.007	4.99E-01	0.003	0.011	7.79E-01
8.702629	0.005	0.007	5.06E-01	0.011	0.011	3.13E-01
2.995723	-0.004	0.004	3.23E-01	0.002	0.006	7.80E-01
8.244072	0.006	0.008	4.76E-01	0.003	0.012	8.13E-01
8.521965	0.006	0.009	4.94E-01	-0.009	0.014	5.08E-01
5.569096	0.008	0.007	2.74E-01	-0.011	0.010	3.05E-01
6.288053	0.006	0.009	4.90E-01	-0.004	0.013	7.87E-01
6.687062	-0.009	0.010	4.06E-01	-0.013	0.015	4.15E-01
4.429935	-0.014	0.014	3.05E-01	0.013	0.021	5.16E-01
2.605797896	-0.014	0.013	2.87E-01	0.018	0.019	3.43E-01
6.236242	0.009	0.008	2.39E-01	0.004	0.011	7.22E-01
6.852923	-0.012	0.010	2.40E-01	-0.012	0.016	4.59E-01
5.068148	0.008	0.008	3.29E-01	-0.010	0.012	4.15E-01
3.234927	-0.006	0.006	3.32E-01	0.008	0.009	4.04E-01
8.125647	-0.008	0.009	3.75E-01	0.016	0.014	2.57E-01
0.804874235	0.003	0.006	5.65E-01	0.011	0.008	1.90E-01
5.275727	-0.009	0.007	1.55E-01	-0.022	0.010	2.33E-02
6.265175	0.010	0.009	2.42E-01	-0.033	0.013	1.11E-02
2.344389772	0.009	0.006	1.64E-01	0.005	0.009	6.22E-01
5.036523	0.008	0.009	3.71E-01	-0.014	0.014	3.05E-01
4.409413	-0.011	0.012	3.36E-01	0.014	0.018	4.20E-01
2.290224125	-0.004	0.005	3.80E-01	-0.008	0.007	2.32E-01
6.92862	-0.014	0.011	2.13E-01	-0.013	0.017	4.48E-01
1.414490091	0.006	0.004	1.77E-01	0.009	0.006	1.55E-01

6.715658	-0.010	0.008	2.08E-01	-0.015	0.012	1.85E-01
8.563683	0.007	0.008	4.15E-01	-0.013	0.012	2.86E-01
5.081269	0.010	0.009	3.08E-01	-0.005	0.014	7.48E-01
3.681036	-0.004	0.005	4.17E-01	0.013	0.007	5.42E-02
7.648922	-0.008	0.011	4.58E-01	-0.006	0.016	7.15E-01
5.324173	0.019	0.018	2.83E-01	-0.027	0.027	3.15E-01
6.566282	0.009	0.008	2.91E-01	-0.011	0.012	3.83E-01
1.036003425	0.006	0.005	2.40E-01	0.004	0.007	5.55E-01
6.310257	0.009	0.008	2.62E-01	0.003	0.012	8.27E-01
5.508875	0.007	0.008	3.39E-01	-0.014	0.011	2.02E-01
8.199663	0.007	0.008	3.83E-01	-0.002	0.012	8.71E-01
5.099773	0.011	0.009	2.38E-01	-0.002	0.013	9.10E-01
5.905192	0.007	0.008	3.57E-01	-0.014	0.011	2.34E-01
1.916783949	-0.006	0.005	2.34E-01	0.004	0.008	6.15E-01
5.021384	0.009	0.010	3.86E-01	-0.008	0.015	6.17E-01
7.656997	0.010	0.008	2.49E-01	-0.003	0.013	7.90E-01
8.329862	-0.006	0.008	4.11E-01	-0.018	0.012	1.33E-01
0.671310497	-0.012	0.010	2.13E-01	-0.004	0.015	7.66E-01
6.910789	0.010	0.008	2.15E-01	0.001	0.012	9.56E-01
2.283831906	-0.005	0.005	3.34E-01	-0.010	0.008	1.76E-01
6.50236	-0.009	0.009	3.42E-01	-0.006	0.014	6.45E-01
0.975109126	0.004	0.006	5.04E-01	0.020	0.009	2.67E-02
5.276063	-0.009	0.007	1.57E-01	-0.022	0.010	2.78E-02
5.670026	0.008	0.010	4.09E-01	0.014	0.015	3.43E-01
6.964955	-0.012	0.010	2.28E-01	-0.017	0.014	2.26E-01
8.800195	0.007	0.006	2.48E-01	0.010	0.009	2.73E-01
5.780376	0.008	0.009	3.87E-01	0.023	0.014	8.89E-02
7.60014	0.014	0.010	1.70E-01	0.000	0.016	9.75E-01
1.496916075	-0.006	0.005	2.11E-01	-0.014	0.007	3.25E-02
7.876351	0.006	0.009	5.10E-01	-0.006	0.014	6.56E-01
3.064692	-0.007	0.008	4.07E-01	0.008	0.012	5.00E-01
7.63311	-0.012	0.011	3.11E-01	0.013	0.017	4.47E-01
8.889013	0.008	0.008	3.08E-01	0.001	0.012	9.62E-01
5.05099	0.006	0.007	3.49E-01	0.009	0.010	3.95E-01
5.877941	0.009	0.010	3.50E-01	-0.005	0.014	7.07E-01
5.743032	0.008	0.010	4.33E-01	0.012	0.015	4.23E-01
7.301051	-0.013	0.009	1.77E-01	-0.014	0.014	3.29E-01
2.500830927	-0.012	0.013	3.44E-01	-0.008	0.019	6.93E-01
3.237618	-0.007	0.006	2.82E-01	0.005	0.009	6.05E-01
1.73376462	-0.007	0.005	1.92E-01	-0.001	0.008	9.23E-01
4.332034	-0.014	0.014	2.99E-01	0.016	0.020	4.25E-01
7.613933	-0.017	0.015	2.52E-01	-0.041	0.022	6.43E-02
2.286859799	-0.010	0.009	2.67E-01	-0.001	0.013	9.36E-01

6.915163	-0.011	0.009	2.57E-01	-0.021	0.014	1.42E-01
8.669995	-0.005	0.010	6.04E-01	-0.017	0.014	2.33E-01
8.991961	-0.009	0.009	3.28E-01	0.020	0.014	1.50E-01
8.409933	0.009	0.008	2.87E-01	0.011	0.012	3.64E-01
4.342799	-0.012	0.013	3.39E-01	0.015	0.019	4.48E-01
7.663389	0.009	0.008	2.81E-01	-0.001	0.012	9.13E-01
7.812765	0.006	0.008	4.29E-01	-0.002	0.012	8.76E-01
6.878828	0.013	0.009	1.70E-01	-0.012	0.014	3.81E-01
1.455198434	0.004	0.004	2.97E-01	0.001	0.005	8.11E-01
6.616747	-0.009	0.008	2.80E-01	-0.009	0.013	4.72E-01
0.835153169	-0.011	0.008	1.96E-01	-0.008	0.013	5.45E-01
4.223366	0.010	0.012	3.96E-01	0.019	0.018	2.95E-01
6.480828	-0.008	0.007	2.74E-01	-0.017	0.010	1.00E-01
6.994225	0.007	0.007	3.41E-01	0.005	0.011	6.35E-01
1.830657206	-0.011	0.010	2.75E-01	-0.013	0.015	4.09E-01
0.816985809	0.004	0.005	5.01E-01	0.015	0.008	5.96E-02
7.942628	0.007	0.009	4.15E-01	-0.008	0.013	5.42E-01
7.141919	-0.014	0.011	2.22E-01	-0.017	0.017	3.11E-01
0.595613164	-0.007	0.009	4.01E-01	-0.033	0.013	1.15E-02
6.092585	0.007	0.008	3.49E-01	0.003	0.011	7.74E-01
5.941863	-0.007	0.007	3.57E-01	-0.022	0.011	4.77E-02
1.123812332	0.008	0.006	2.34E-01	-0.004	0.010	6.97E-01
0.990585026	0.005	0.007	4.60E-01	0.015	0.010	1.63E-01
1.855216785	-0.010	0.009	2.73E-01	-0.012	0.014	4.01E-01
0.68342207	0.015	0.013	2.59E-01	-0.010	0.019	6.08E-01
4.345154	-0.012	0.014	3.98E-01	0.016	0.021	4.66E-01
8.97514	0.011	0.010	2.41E-01	-0.018	0.014	2.02E-01
7.458502	0.008	0.009	3.55E-01	0.008	0.013	5.44E-01
0.630938586	0.011	0.011	3.01E-01	0.005	0.016	7.54E-01
5.942873	0.009	0.009	2.93E-01	0.002	0.013	8.71E-01
2.799919501	-0.014	0.014	3.19E-01	-0.069	0.021	9.80E-04
4.319922	-0.014	0.014	3.07E-01	0.019	0.021	3.61E-01
8.767897	0.007	0.009	4.43E-01	-0.002	0.013	9.04E-01
8.374944	-0.010	0.009	2.84E-01	0.008	0.014	5.48E-01
2.295943479	-0.011	0.010	2.94E-01	0.007	0.015	6.72E-01
8.906171	-0.005	0.008	5.07E-01	-0.019	0.012	1.01E-01
2.795209445	-0.013	0.012	2.71E-01	-0.056	0.017	1.43E-03
8.971439	0.009	0.008	2.89E-01	-0.018	0.012	1.51E-01
2.030498165	0.007	0.005	1.95E-01	0.006	0.008	4.56E-01
1.917456814	-0.007	0.005	1.87E-01	0.000	0.008	9.79E-01
8.569066	0.009	0.009	3.44E-01	-0.008	0.014	5.46E-01
8.80255	-0.006	0.006	3.62E-01	0.002	0.010	8.01E-01
7.119041	-0.008	0.010	3.90E-01	-0.008	0.014	5.56E-01

5.651859	0.009	0.010	3.34E-01	-0.011	0.015	4.44E-01
4.414123	-0.015	0.015	3.15E-01	0.017	0.022	4.26E-01
8.930731	0.008	0.009	3.42E-01	0.003	0.013	8.43E-01
5.818729	0.006	0.008	4.26E-01	0.001	0.012	9.62E-01
8.938805	-0.006	0.008	4.41E-01	0.005	0.013	6.78E-01
4.00233	0.009	0.010	3.40E-01	0.019	0.014	1.87E-01
8.517591	-0.008	0.009	3.89E-01	0.000	0.014	9.93E-01
6.334144	0.007	0.006	2.59E-01	-0.005	0.009	5.65E-01
4.444066	-0.015	0.016	3.59E-01	0.023	0.024	3.44E-01
8.822063	0.006	0.007	3.93E-01	0.004	0.011	6.96E-01
1.00807952	0.007	0.006	2.68E-01	0.016	0.010	8.95E-02
4.425562	-0.013	0.014	3.46E-01	0.007	0.021	7.43E-01
6.96731	-0.010	0.008	2.25E-01	-0.002	0.013	8.53E-01
5.964741	-0.005	0.006	3.68E-01	-0.006	0.009	4.83E-01
2.800255933	-0.014	0.014	3.31E-01	-0.065	0.021	2.02E-03
2.296279912	-0.013	0.012	2.82E-01	0.004	0.018	8.07E-01
1.497925373	-0.005	0.005	2.48E-01	-0.010	0.007	1.59E-01
6.219084	0.007	0.008	3.57E-01	0.005	0.012	6.54E-01
2.282486176	-0.004	0.005	3.94E-01	-0.005	0.007	4.55E-01
0.738260582	-0.007	0.007	2.63E-01	-0.011	0.010	2.51E-01
4.331024	-0.015	0.015	3.19E-01	0.014	0.022	5.30E-01
3.137698	-0.011	0.010	2.59E-01	-0.012	0.014	4.01E-01
5.437215	0.007	0.007	2.80E-01	0.008	0.010	4.21E-01
4.428926	-0.012	0.014	3.73E-01	0.012	0.020	5.43E-01
5.076222	0.007	0.007	3.50E-01	0.003	0.011	7.95E-01
3.439141	0.008	0.008	3.07E-01	0.024	0.011	3.65E-02
6.008141	0.009	0.008	2.78E-01	0.007	0.012	5.58E-01
8.55258	-0.009	0.010	3.61E-01	0.024	0.015	9.69E-02
5.475904	0.007	0.006	2.72E-01	-0.010	0.009	2.64E-01
4.120417	-0.009	0.012	4.41E-01	0.015	0.018	4.11E-01
7.81983	-0.010	0.010	3.27E-01	0.001	0.015	9.59E-01
1.494561047	-0.005	0.004	2.27E-01	-0.009	0.006	1.61E-01
5.154948	0.011	0.011	3.06E-01	-0.025	0.016	1.16E-01
0.549521899	-0.007	0.009	4.27E-01	-0.016	0.013	2.21E-01
2.169781258	0.011	0.010	2.84E-01	-0.012	0.016	4.31E-01
6.975721	-0.012	0.011	2.88E-01	0.001	0.016	9.41E-01
4.137912	-0.015	0.013	2.60E-01	0.010	0.020	6.06E-01
2.499485197	-0.016	0.013	2.23E-01	-0.007	0.020	7.27E-01
4.151706	-0.020	0.019	2.80E-01	0.016	0.028	5.59E-01
2.503185955	-0.016	0.013	2.29E-01	-0.016	0.020	4.12E-01
0.568362124	0.009	0.008	2.24E-01	0.016	0.011	1.68E-01
1.984743333	0.008	0.007	2.33E-01	-0.005	0.010	6.19E-01
5.430149	0.009	0.007	2.43E-01	-0.009	0.011	4.26E-01

2.598732811	-0.018	0.017	3.02E-01	0.003	0.026	8.94E-01
5.638401	0.009	0.008	2.95E-01	0.010	0.013	4.41E-01
7.013738	0.009	0.010	3.64E-01	-0.007	0.015	6.30E-01
7.619989	-0.018	0.014	1.86E-01	0.005	0.021	8.01E-01
8.286462	-0.006	0.008	4.78E-01	-0.020	0.012	1.13E-01
6.146078	0.013	0.010	2.06E-01	-0.004	0.015	7.90E-01
4.431618	-0.015	0.016	3.48E-01	0.018	0.024	4.45E-01
1.41314436	0.007	0.005	1.54E-01	0.014	0.007	4.71E-02
5.973488	-0.004	0.007	6.00E-01	-0.007	0.011	5.24E-01
1.237190115	0.007	0.008	3.31E-01	-0.006	0.011	5.66E-01
3.280345	-0.012	0.009	1.84E-01	-0.001	0.014	9.48E-01
2.660299976	-0.012	0.011	2.95E-01	0.001	0.017	9.73E-01
8.509517	0.006	0.008	4.08E-01	-0.014	0.012	2.22E-01
5.766246	0.010	0.009	2.65E-01	-0.011	0.013	4.33E-01
7.064875	-0.008	0.010	4.02E-01	-0.028	0.014	4.89E-02
1.008752386	0.009	0.007	2.13E-01	0.022	0.011	4.49E-02
1.62610619	0.007	0.006	2.51E-01	-0.005	0.009	5.33E-01
5.093717	0.008	0.008	3.30E-01	-0.008	0.012	5.02E-01
1.436021776	0.006	0.004	1.84E-01	0.000	0.006	9.98E-01
4.350874	-0.014	0.014	3.05E-01	0.018	0.021	3.93E-01
7.825886	-0.012	0.009	2.08E-01	-0.005	0.014	7.19E-01
4.395619	-0.013	0.014	3.41E-01	0.006	0.021	7.76E-01
1.637208466	0.006	0.006	2.74E-01	-0.005	0.009	6.08E-01
1.027592611	0.006	0.007	3.95E-01	0.008	0.011	4.80E-01
2.587294103	0.011	0.009	2.42E-01	0.012	0.014	3.96E-01
1.983734035	0.008	0.006	2.42E-01	-0.005	0.010	6.15E-01
5.03215	0.007	0.008	3.55E-01	-0.010	0.011	3.80E-01
7.197766	0.009	0.008	2.97E-01	0.004	0.013	7.35E-01
8.91189	0.007	0.009	4.29E-01	0.000	0.013	9.72E-01
4.299736	-0.013	0.014	3.45E-01	0.003	0.021	8.79E-01
0.975445559	0.004	0.006	4.78E-01	0.019	0.008	2.31E-02
2.030834598	0.007	0.005	1.97E-01	0.006	0.008	4.40E-01
7.137545	0.011	0.010	2.59E-01	-0.002	0.015	8.72E-01
6.251381	0.008	0.008	3.45E-01	0.010	0.012	3.99E-01
1.081085393	0.008	0.007	2.29E-01	0.004	0.010	7.12E-01
5.404244	0.007	0.007	3.29E-01	-0.002	0.010	8.68E-01
8.702966	0.008	0.009	4.30E-01	0.016	0.014	2.57E-01
2.617909469	-0.015	0.013	2.49E-01	0.025	0.020	2.15E-01
1.72468094	-0.005	0.005	2.84E-01	0.002	0.007	7.74E-01
3.167304	-0.007	0.006	3.05E-01	0.011	0.010	2.43E-01
2.356501346	-0.003	0.007	6.65E-01	0.022	0.010	3.66E-02
8.00756	0.006	0.007	3.29E-01	0.017	0.010	8.64E-02
8.367542	0.008	0.008	3.12E-01	0.003	0.012	7.92E-01

8.186542	0.011	0.010	2.71E-01	0.008	0.014	5.91E-01
6.052213	0.007	0.007	2.88E-01	-0.001	0.010	9.46E-01
7.850109	-0.013	0.012	2.77E-01	0.000	0.017	9.79E-01
3.944127	-0.008	0.009	3.31E-01	0.001	0.013	9.25E-01
2.350445559	0.014	0.009	1.33E-01	0.022	0.014	1.15E-01
1.103289944	0.010	0.008	2.14E-01	-0.004	0.011	6.94E-01
1.98306117	0.007	0.006	2.41E-01	-0.005	0.009	6.16E-01
8.795148	-0.004	0.010	6.40E-01	-0.017	0.014	2.26E-01
0.984529239	0.008	0.006	1.57E-01	0.011	0.008	2.02E-01
8.839221	0.010	0.010	2.86E-01	-0.001	0.014	9.31E-01
7.925134	0.012	0.010	2.08E-01	-0.005	0.014	7.20E-01
0.660881086	-0.013	0.009	1.63E-01	-0.004	0.014	7.94E-01
4.347173	-0.014	0.015	3.68E-01	0.014	0.023	5.37E-01
1.895925128	-0.007	0.006	2.43E-01	-0.003	0.009	7.22E-01
4.333716	-0.014	0.015	3.25E-01	0.026	0.022	2.41E-01
7.493827	-0.011	0.010	2.53E-01	0.004	0.014	7.77E-01
3.980125	-0.010	0.012	4.09E-01	-0.007	0.018	6.79E-01
1.524503548	0.011	0.009	2.54E-01	-0.015	0.014	2.88E-01
5.750433	0.007	0.009	4.68E-01	-0.006	0.014	6.70E-01
5.158648	0.008	0.008	3.37E-01	-0.011	0.012	3.65E-01
7.702079	-0.011	0.010	2.87E-01	0.026	0.015	8.06E-02
8.771598	0.009	0.009	2.93E-01	0.001	0.013	9.28E-01
8.858398	0.006	0.008	4.21E-01	-0.009	0.012	4.42E-01
5.308361	0.017	0.015	2.70E-01	-0.024	0.023	3.02E-01
3.794078	0.008	0.006	2.06E-01	0.011	0.009	2.14E-01
4.417824	-0.012	0.012	3.19E-01	0.009	0.018	6.09E-01
4.103596	-0.007	0.011	5.35E-01	0.006	0.016	6.90E-01
6.466698	-0.005	0.008	5.73E-01	-0.019	0.012	1.15E-01
8.809278	0.009	0.008	3.03E-01	0.003	0.012	7.82E-01
8.714404	-0.009	0.009	2.92E-01	-0.015	0.013	2.38E-01
7.327293	-0.008	0.007	2.00E-01	-0.008	0.010	4.10E-01
6.770833	0.013	0.011	2.63E-01	-0.014	0.017	4.09E-01
7.960795	-0.009	0.009	2.87E-01	-0.011	0.013	3.74E-01
5.284811	-0.010	0.008	2.16E-01	-0.026	0.012	2.72E-02
2.182565696	0.011	0.011	2.95E-01	-0.020	0.016	2.19E-01
1.455871299	0.003	0.004	3.83E-01	-0.001	0.005	9.18E-01
3.32139	0.012	0.012	3.22E-01	0.039	0.018	2.78E-02
5.734285	0.010	0.011	3.44E-01	-0.005	0.016	7.61E-01
8.931067	0.009	0.009	2.81E-01	-0.005	0.013	7.17E-01
0.725139711	0.008	0.007	2.92E-01	0.012	0.011	2.80E-01
1.529550037	0.011	0.010	2.72E-01	-0.018	0.015	2.08E-01
6.481165	-0.009	0.008	2.54E-01	0.002	0.011	8.55E-01
8.207401	0.007	0.007	2.98E-01	0.002	0.010	8.68E-01

3.955565	-0.007	0.009	4.37E-01	-0.007	0.013	6.05E-01
6.239606	0.012	0.011	2.70E-01	0.031	0.016	5.60E-02
3.160575	-0.009	0.007	2.19E-01	0.002	0.011	8.39E-01
7.970552	0.009	0.009	2.91E-01	-0.003	0.013	8.38E-01
5.308697	0.017	0.015	2.74E-01	-0.023	0.023	3.13E-01
2.871916	-0.007	0.010	4.38E-01	-0.001	0.014	9.46E-01
2.296616345	-0.013	0.012	2.84E-01	-0.001	0.018	9.48E-01
4.398311	-0.013	0.015	3.74E-01	0.018	0.022	4.06E-01
6.498659	0.007	0.007	3.30E-01	-0.007	0.011	5.16E-01
2.597387081	-0.018	0.019	3.43E-01	-0.006	0.028	8.20E-01
7.728321	-0.005	0.008	5.42E-01	-0.010	0.012	4.28E-01
7.20685	0.008	0.007	2.62E-01	-0.004	0.011	7.19E-01
1.972631759	0.005	0.005	3.28E-01	-0.003	0.007	7.13E-01
3.690793	-0.005	0.004	3.14E-01	0.008	0.007	2.37E-01
5.677091	0.011	0.010	2.56E-01	-0.013	0.015	3.88E-01
5.299277	0.015	0.014	2.70E-01	-0.019	0.021	3.43E-01
2.718166381	-0.009	0.007	1.64E-01	-0.016	0.010	9.25E-02
5.065793	0.007	0.007	3.27E-01	0.003	0.011	7.53E-01
1.418527282	0.007	0.005	1.55E-01	0.003	0.007	6.93E-01
6.997253	0.009	0.008	2.27E-01	0.004	0.011	7.45E-01
1.466637142	-0.006	0.007	4.07E-01	0.005	0.010	6.01E-01
7.400972	-0.007	0.008	3.98E-01	0.007	0.012	5.55E-01
0.676356986	0.004	0.008	5.62E-01	-0.020	0.012	7.65E-02
5.977862	0.011	0.010	3.02E-01	-0.013	0.015	4.09E-01
5.862129	0.010	0.008	2.14E-01	0.004	0.012	7.02E-01
3.175042	-0.006	0.006	2.64E-01	0.006	0.008	4.92E-01
7.370693	0.012	0.009	2.02E-01	0.010	0.014	4.43E-01
7.723274	-0.009	0.010	3.54E-01	-0.014	0.015	3.67E-01
2.793863714	-0.013	0.012	2.78E-01	-0.060	0.018	8.19E-04
3.920576	0.014	0.011	2.11E-01	0.038	0.017	1.99E-02
0.730522633	-0.008	0.007	2.91E-01	-0.001	0.011	8.92E-01
8.933759	-0.004	0.006	5.54E-01	-0.004	0.009	6.92E-01
4.407731	-0.014	0.015	3.50E-01	0.017	0.023	4.50E-01
8.494714	0.005	0.006	3.98E-01	0.002	0.010	8.67E-01
7.593747	-0.009	0.009	3.24E-01	-0.001	0.013	9.24E-01
6.904397	0.009	0.010	3.82E-01	0.018	0.015	2.33E-01
6.466362	-0.009	0.008	2.65E-01	-0.016	0.012	1.89E-01
2.080290188	-0.003	0.004	4.93E-01	0.007	0.006	2.28E-01
6.554171	0.013	0.009	1.75E-01	-0.009	0.014	5.32E-01
4.31353	-0.013	0.012	2.91E-01	0.008	0.018	6.65E-01
4.103932	-0.007	0.011	5.38E-01	0.006	0.016	7.03E-01
4.321604	-0.013	0.014	3.43E-01	0.014	0.020	4.81E-01
8.851333	-0.006	0.008	4.16E-01	-0.026	0.012	2.57E-02

0.531354539	0.010	0.009	2.79E-01	0.014	0.014	3.04E-01
3.914857	-0.008	0.009	3.54E-01	0.028	0.013	3.39E-02
7.638157	-0.008	0.010	3.99E-01	0.013	0.015	3.64E-01
4.309829	-0.014	0.013	3.02E-01	0.020	0.020	3.10E-01
6.102678	0.011	0.009	2.27E-01	0.003	0.013	8.16E-01
7.373384	-0.009	0.010	3.30E-01	-0.008	0.014	5.66E-01
3.202629	0.006	0.007	3.44E-01	0.019	0.010	5.15E-02
1.526185711	0.011	0.009	2.59E-01	-0.015	0.014	2.88E-01
6.078119	0.006	0.007	4.08E-01	-0.004	0.010	7.18E-01
1.66109518	0.004	0.004	3.62E-01	0.001	0.006	8.23E-01
4.399993	-0.012	0.013	3.27E-01	0.018	0.019	3.45E-01
5.089007	0.007	0.007	3.41E-01	-0.007	0.011	4.83E-01
5.309034	0.017	0.016	2.79E-01	-0.024	0.023	3.10E-01
2.09475679	-0.006	0.007	4.23E-01	-0.003	0.011	7.84E-01
1.970613164	0.004	0.004	3.16E-01	-0.003	0.007	6.53E-01
8.856715	0.009	0.008	2.75E-01	-0.001	0.012	9.61E-01
5.889044	0.011	0.009	2.22E-01	-0.003	0.014	8.41E-01
8.258202	-0.009	0.010	3.51E-01	-0.017	0.015	2.70E-01
6.095613	0.008	0.009	3.59E-01	0.006	0.014	6.35E-01
7.12476	-0.008	0.008	3.27E-01	-0.017	0.012	1.71E-01
8.755113	-0.008	0.010	4.19E-01	0.012	0.014	4.26E-01
6.381581	-0.007	0.007	3.02E-01	-0.006	0.011	5.83E-01
1.518447761	0.008	0.009	3.35E-01	-0.011	0.013	3.83E-01
8.700274	0.008	0.009	3.60E-01	0.001	0.013	9.52E-01
1.526858576	0.010	0.009	2.66E-01	-0.017	0.014	2.35E-01
2.08567311	-0.005	0.006	3.28E-01	0.006	0.008	4.42E-01
4.407394	-0.015	0.016	3.51E-01	0.013	0.023	5.68E-01
6.326406	0.007	0.008	3.90E-01	-0.016	0.012	1.87E-01
6.697491	0.006	0.006	3.21E-01	0.017	0.009	7.30E-02
6.756367	-0.007	0.007	3.40E-01	0.004	0.011	6.90E-01
2.675775875	-0.010	0.007	1.84E-01	0.003	0.011	7.80E-01
7.667763	0.011	0.011	3.13E-01	0.009	0.016	5.88E-01
5.051326	0.006	0.007	3.77E-01	0.012	0.010	2.27E-01
3.65143	-0.004	0.004	3.39E-01	0.007	0.007	2.90E-01
6.112771	-0.009	0.007	2.03E-01	-0.003	0.011	7.71E-01
8.005205	0.009	0.010	3.49E-01	0.005	0.015	7.38E-01
8.600017	0.009	0.007	2.25E-01	0.013	0.011	2.42E-01
0.620172743	-0.007	0.009	4.46E-01	-0.019	0.013	1.48E-01
1.970949596	0.004	0.005	3.28E-01	-0.003	0.007	6.24E-01
2.263982383	0.007	0.007	3.13E-01	0.016	0.010	1.15E-01
6.736181	-0.010	0.008	2.11E-01	-0.001	0.012	9.26E-01
1.623751162	0.008	0.007	2.44E-01	0.012	0.010	2.10E-01
7.737404	-0.007	0.011	5.30E-01	0.005	0.017	7.74E-01

3.512147	0.007	0.008	3.31E-01	0.028	0.011	1.60E-02
5.114239	0.010	0.009	2.72E-01	-0.001	0.014	9.64E-01
2.644824076	-0.012	0.009	2.03E-01	0.020	0.014	1.51E-01
8.546861	-0.008	0.009	3.53E-01	-0.013	0.013	3.26E-01
2.341025447	0.008	0.006	2.00E-01	0.003	0.010	7.68E-01
5.600048	0.012	0.010	2.01E-01	-0.017	0.014	2.31E-01
1.904672376	-0.007	0.006	2.95E-01	-0.004	0.010	7.07E-01
8.95529	-0.007	0.007	3.17E-01	0.012	0.011	2.48E-01
8.472173	-0.008	0.010	4.44E-01	-0.029	0.015	5.17E-02
1.719970883	-0.008	0.006	1.85E-01	-0.005	0.009	5.42E-01
7.720919	-0.011	0.010	2.60E-01	-0.007	0.015	6.40E-01
6.936022	0.010	0.009	2.89E-01	0.011	0.014	4.24E-01
8.247436	-0.005	0.008	4.83E-01	-0.005	0.011	6.76E-01
1.515419868	0.008	0.008	3.08E-01	-0.013	0.012	2.74E-01
8.486303	0.007	0.008	3.41E-01	0.000	0.011	9.75E-01
6.607327	0.006	0.008	4.56E-01	0.006	0.012	5.94E-01
7.820503	-0.009	0.010	3.60E-01	-0.007	0.015	6.51E-01
7.527807	-0.007	0.011	5.15E-01	-0.002	0.016	8.93E-01
1.730736726	-0.007	0.005	1.91E-01	-0.001	0.008	9.21E-01
5.540163	0.009	0.008	2.71E-01	-0.002	0.012	8.44E-01
6.707248	-0.009	0.010	3.44E-01	-0.020	0.015	1.86E-01
1.719634451	-0.008	0.006	1.81E-01	-0.006	0.009	4.78E-01
1.526522143	0.010	0.009	2.65E-01	-0.015	0.014	2.76E-01
8.54888	-0.008	0.007	2.86E-01	-0.024	0.011	2.28E-02
6.130266	0.008	0.009	3.51E-01	0.024	0.013	5.40E-02
2.369622217	-0.007	0.007	3.24E-01	0.017	0.010	9.33E-02
2.359192806	0.010	0.009	2.50E-01	0.020	0.013	1.30E-01
8.551908	-0.007	0.009	4.21E-01	0.030	0.014	3.24E-02
5.963059	0.009	0.010	3.57E-01	0.004	0.015	7.83E-01
1.637544898	0.007	0.006	2.66E-01	0.000	0.009	9.76E-01
5.923696	-0.009	0.009	3.05E-01	0.002	0.013	8.76E-01
8.808269	0.009	0.009	3.34E-01	-0.031	0.013	1.81E-02
1.653693663	0.004	0.005	4.05E-01	0.009	0.007	1.94E-01
3.17807	-0.005	0.005	3.23E-01	0.013	0.008	1.17E-01
5.034841	0.009	0.009	3.46E-01	0.004	0.014	7.47E-01
5.631	0.008	0.008	3.12E-01	-0.010	0.012	3.75E-01
6.491594	0.007	0.008	3.72E-01	-0.003	0.012	7.81E-01
8.022363	0.010	0.008	2.29E-01	-0.007	0.012	5.51E-01
1.033311965	0.005	0.005	3.05E-01	0.006	0.008	4.38E-01
6.84283	0.006	0.007	4.10E-01	-0.004	0.010	7.01E-01
8.06509	0.011	0.009	2.03E-01	0.023	0.013	8.42E-02
5.828149	0.010	0.008	2.23E-01	0.030	0.012	1.52E-02
0.613780524	-0.011	0.010	2.80E-01	-0.020	0.015	1.82E-01

6.102005	-0.006	0.007	3.54E-01	-0.001	0.010	9.13E-01
6.94746	-0.007	0.009	4.57E-01	0.005	0.013	7.32E-01
7.066221	0.007	0.006	2.78E-01	-0.002	0.009	8.47E-01
2.292579153	-0.003	0.004	4.69E-01	-0.010	0.007	1.47E-01
1.823928554	-0.009	0.010	3.69E-01	-0.002	0.014	8.97E-01
8.680088	-0.007	0.007	3.46E-01	-0.017	0.011	1.01E-01
5.737649	0.010	0.011	3.62E-01	-0.021	0.016	1.75E-01
1.496579643	-0.006	0.004	1.87E-01	-0.011	0.007	8.61E-02
5.324846	0.019	0.018	2.94E-01	-0.025	0.027	3.53E-01
0.781660387	0.004	0.004	3.92E-01	0.000	0.007	9.82E-01
5.536462	0.006	0.006	2.95E-01	-0.001	0.009	8.89E-01
6.959572	-0.009	0.009	2.67E-01	0.002	0.013	8.48E-01
3.94379	-0.008	0.009	3.41E-01	-0.001	0.013	9.60E-01
6.358031	-0.010	0.009	2.88E-01	0.013	0.014	3.42E-01
2.756183264	-0.010	0.007	1.75E-01	-0.023	0.011	3.33E-02
5.306342	0.016	0.015	2.67E-01	-0.024	0.022	2.81E-01
8.143478	0.006	0.008	4.54E-01	0.000	0.011	9.77E-01
1.734101052	-0.006	0.005	2.25E-01	-0.001	0.007	8.54E-01
8.073837	0.006	0.009	4.98E-01	0.004	0.013	7.47E-01
1.465291412	-0.004	0.005	4.17E-01	0.006	0.008	4.33E-01
7.667426	0.010	0.009	2.89E-01	0.008	0.014	5.76E-01
6.45526	0.006	0.007	4.50E-01	0.001	0.011	9.37E-01
2.510923905	-0.010	0.015	5.09E-01	-0.011	0.022	6.12E-01
5.403908	0.007	0.007	3.14E-01	-0.005	0.011	6.34E-01
5.016001	0.008	0.009	3.80E-01	0.009	0.014	5.24E-01
3.732847	0.005	0.008	5.39E-01	0.013	0.012	2.79E-01
7.121733	0.009	0.009	2.98E-01	-0.009	0.013	4.99E-01
3.774228	0.010	0.010	2.96E-01	0.034	0.015	1.95E-02
3.308606	-0.014	0.012	2.52E-01	0.006	0.018	7.51E-01
0.653816002	-0.012	0.008	1.69E-01	-0.014	0.013	2.63E-01
5.953302	0.010	0.009	2.50E-01	0.006	0.013	6.73E-01
7.746151	-0.008	0.011	4.55E-01	-0.022	0.016	1.67E-01
5.483306	0.009	0.009	3.14E-01	0.009	0.013	4.98E-01
5.826131	0.008	0.007	2.55E-01	0.005	0.010	5.99E-01
8.124638	0.008	0.008	3.35E-01	0.012	0.012	2.94E-01
7.754899	-0.008	0.011	4.64E-01	-0.014	0.017	3.87E-01
7.697032	-0.011	0.011	3.08E-01	0.003	0.016	8.38E-01
5.416356	-0.008	0.009	3.88E-01	0.008	0.014	5.84E-01
5.308024	0.016	0.015	2.78E-01	-0.023	0.023	3.12E-01
5.317108	0.017	0.016	2.89E-01	-0.030	0.025	2.17E-01
5.454036	0.007	0.009	4.17E-01	-0.015	0.013	2.33E-01
8.823072	-0.011	0.010	2.42E-01	0.001	0.014	9.28E-01
3.433759	0.009	0.010	3.56E-01	0.028	0.015	6.87E-02

8.408924	0.007	0.006	2.74E-01	-0.001	0.009	8.72E-01
4.444402	-0.015	0.017	3.88E-01	0.028	0.025	2.61E-01
2.594359188	-0.018	0.020	3.51E-01	0.006	0.030	8.39E-01
2.718502814	-0.009	0.007	1.76E-01	-0.016	0.010	1.14E-01
5.132407	0.010	0.010	3.14E-01	0.000	0.015	9.94E-01
6.765787	0.008	0.008	2.89E-01	-0.011	0.012	3.51E-01
3.054263	-0.008	0.009	3.97E-01	0.011	0.013	4.01E-01
8.057352	0.009	0.008	2.47E-01	-0.011	0.011	3.06E-01
6.616411	-0.008	0.009	3.79E-01	-0.003	0.014	8.11E-01
2.755846831	-0.010	0.007	1.78E-01	-0.022	0.011	4.31E-02
2.548940788	-0.014	0.012	2.27E-01	-0.017	0.018	3.45E-01
0.670974064	-0.012	0.010	1.98E-01	-0.012	0.014	4.09E-01
5.09439	0.009	0.008	2.76E-01	-0.008	0.012	5.14E-01
6.88051	0.015	0.009	1.06E-01	-0.004	0.013	7.83E-01
3.497344	0.006	0.010	4.98E-01	0.034	0.014	1.66E-02
4.336744	-0.014	0.015	3.75E-01	0.016	0.023	4.89E-01
2.569126743	-0.009	0.007	1.90E-01	-0.006	0.011	5.81E-01
3.91553	-0.009	0.009	3.21E-01	0.024	0.013	6.95E-02
8.503798	0.011	0.009	2.32E-01	0.006	0.013	6.32E-01
6.51279	-0.008	0.008	2.96E-01	0.007	0.012	5.36E-01
3.236609	-0.006	0.006	3.85E-01	0.006	0.010	5.19E-01
7.643876	-0.006	0.010	5.45E-01	-0.014	0.016	3.70E-01
3.173696	-0.006	0.006	2.70E-01	0.005	0.009	5.42E-01
5.565732	0.007	0.007	3.00E-01	-0.010	0.010	3.16E-01
6.261811	0.005	0.008	5.46E-01	0.000	0.012	9.94E-01
5.954311	0.007	0.008	3.63E-01	-0.003	0.012	7.77E-01
1.495906778	-0.005	0.004	2.12E-01	-0.011	0.007	1.06E-01
7.101883	-0.012	0.009	1.94E-01	-0.004	0.013	7.88E-01
4.379807	-0.014	0.014	3.29E-01	0.002	0.021	9.10E-01
3.646384	0.008	0.005	1.22E-01	0.015	0.008	5.62E-02
7.011719	0.010	0.010	2.78E-01	-0.008	0.014	5.61E-01
7.951712	0.008	0.007	2.46E-01	-0.014	0.011	1.94E-01
8.876229	0.010	0.010	3.08E-01	0.008	0.015	5.96E-01
6.899687	0.010	0.008	2.25E-01	-0.010	0.012	3.98E-01
4.33708	-0.013	0.015	3.91E-01	0.003	0.023	8.90E-01
8.74031	-0.009	0.008	2.59E-01	0.005	0.012	6.75E-01
5.491717	0.010	0.009	3.15E-01	-0.023	0.014	1.05E-01
3.692812	-0.004	0.005	4.48E-01	0.008	0.007	2.44E-01
6.024289	0.009	0.008	2.55E-01	-0.004	0.012	7.68E-01
8.572766	-0.008	0.009	3.95E-01	0.000	0.013	9.92E-01
3.235263	-0.006	0.006	3.48E-01	0.008	0.009	3.84E-01
8.695564	-0.010	0.009	3.09E-01	0.007	0.014	6.44E-01
2.29863494	-0.006	0.005	2.62E-01	-0.006	0.008	4.67E-01

5.429477	0.008	0.007	2.60E-01	0.003	0.011	8.09E-01
1.925867629	0.003	0.004	4.23E-01	0.007	0.006	2.38E-01
6.329434	0.008	0.008	3.05E-01	-0.006	0.012	6.05E-01
5.588946	-0.012	0.009	2.05E-01	-0.010	0.014	4.75E-01
1.823592121	-0.009	0.010	3.43E-01	0.004	0.014	7.92E-01
2.187612185	0.013	0.011	2.33E-01	-0.017	0.016	3.12E-01
7.731685	-0.009	0.012	4.48E-01	-0.002	0.017	9.06E-01
2.16877196	0.012	0.011	2.72E-01	-0.015	0.016	3.40E-01
5.08497	0.009	0.010	3.80E-01	-0.017	0.016	2.80E-01
8.646445	0.007	0.006	2.64E-01	0.006	0.009	5.14E-01
8.279061	0.006	0.009	5.44E-01	0.011	0.014	4.06E-01
3.693148	-0.004	0.005	4.31E-01	0.010	0.007	1.61E-01
7.638493	-0.011	0.012	3.91E-01	0.026	0.018	1.65E-01
5.0991	0.009	0.007	2.44E-01	0.000	0.011	9.99E-01
2.67644874	-0.009	0.007	1.73E-01	0.006	0.010	5.47E-01
6.99557	0.010	0.007	1.61E-01	-0.005	0.011	6.49E-01
5.400207	0.008	0.007	2.65E-01	-0.009	0.011	4.15E-01
7.167487	0.009	0.010	3.33E-01	-0.006	0.014	6.85E-01
4.398647	-0.014	0.016	3.77E-01	0.012	0.024	6.22E-01
6.118154	-0.011	0.010	2.63E-01	0.011	0.015	4.41E-01
3.175378	-0.006	0.006	2.84E-01	0.005	0.008	5.58E-01
0.974772694	0.004	0.006	5.05E-01	0.018	0.009	4.60E-02
2.614208711	-0.013	0.013	2.96E-01	0.040	0.019	3.38E-02
2.150941033	-0.008	0.007	2.59E-01	-0.007	0.011	5.15E-01
7.620999	-0.015	0.011	1.88E-01	0.007	0.017	6.75E-01
4.382162	-0.012	0.013	3.25E-01	0.015	0.019	4.35E-01
1.780865182	-0.009	0.011	3.94E-01	0.017	0.016	2.85E-01
6.582768	0.006	0.008	4.46E-01	-0.010	0.012	3.91E-01
5.114912	0.009	0.010	3.27E-01	-0.001	0.014	9.42E-01
2.166080499	0.012	0.011	2.51E-01	-0.007	0.016	6.70E-01
7.235447	0.004	0.009	6.33E-01	0.014	0.014	2.98E-01
8.947889	0.007	0.009	4.50E-01	0.012	0.013	3.52E-01
1.529886469	0.011	0.010	2.80E-01	-0.019	0.015	1.96E-01
8.94116	-0.009	0.009	3.15E-01	-0.024	0.013	6.05E-02
4.44743	-0.013	0.016	4.11E-01	0.021	0.024	3.73E-01
8.789092	0.012	0.010	2.44E-01	-0.015	0.015	3.00E-01
1.968931001	0.004	0.004	3.23E-01	-0.002	0.007	8.13E-01
1.984070467	0.008	0.007	2.49E-01	-0.005	0.010	5.95E-01
5.834541	0.009	0.009	2.99E-01	-0.001	0.013	9.09E-01
0.538419623	0.009	0.009	2.80E-01	0.013	0.013	3.03E-01
4.31824	-0.014	0.014	3.11E-01	0.023	0.020	2.55E-01
4.141276	-0.018	0.018	3.10E-01	0.015	0.027	5.76E-01
0.973763396	0.004	0.005	4.29E-01	0.019	0.008	1.44E-02

0.625555664	0.009	0.008	2.74E-01	-0.005	0.012	7.07E-01
7.004654	-0.006	0.006	3.18E-01	-0.011	0.010	2.40E-01
0.771230976	0.006	0.005	2.77E-01	-0.005	0.008	4.89E-01
6.681006	0.006	0.007	3.68E-01	0.001	0.010	8.97E-01
0.643386592	0.009	0.009	3.10E-01	0.004	0.014	7.88E-01
2.56475312	-0.007	0.007	3.21E-01	-0.002	0.011	8.73E-01
3.914521	-0.008	0.009	3.69E-01	0.027	0.013	3.87E-02
5.821757	0.006	0.009	4.85E-01	0.010	0.013	4.44E-01
8.837202	0.008	0.007	2.78E-01	0.019	0.011	9.44E-02
4.182321	-0.011	0.013	3.90E-01	-0.005	0.019	8.03E-01
6.121855	-0.008	0.009	3.48E-01	-0.016	0.013	2.33E-01
1.732755322	-0.006	0.005	2.19E-01	0.002	0.008	7.65E-01
3.639319	0.008	0.005	1.31E-01	0.012	0.008	1.25E-01
8.419353	0.007	0.010	4.50E-01	-0.002	0.015	8.80E-01
5.092371	0.009	0.009	3.35E-01	0.001	0.014	9.16E-01
5.139808	0.007	0.007	2.81E-01	-0.013	0.010	1.91E-01
6.436419	0.009	0.009	3.35E-01	-0.014	0.014	3.06E-01
8.666295	-0.009	0.008	2.83E-01	-0.013	0.012	2.92E-01
4.345827	-0.013	0.015	3.83E-01	0.024	0.023	2.92E-01
7.791233	-0.012	0.010	2.27E-01	0.012	0.015	4.38E-01
1.9844069	0.008	0.007	2.47E-01	-0.005	0.010	5.90E-01
8.184523	-0.013	0.010	1.87E-01	-0.045	0.015	2.94E-03
7.962814	0.010	0.009	2.62E-01	0.014	0.014	3.20E-01
1.008415953	0.008	0.007	2.35E-01	0.020	0.010	5.01E-02
8.563346	0.007	0.008	4.03E-01	-0.011	0.012	3.34E-01
1.849497431	-0.006	0.008	4.61E-01	0.001	0.012	9.42E-01
1.197491069	0.006	0.006	3.73E-01	0.012	0.009	1.95E-01
8.265603	-0.008	0.009	4.15E-01	-0.011	0.014	4.32E-01
2.613872278	-0.014	0.014	2.91E-01	0.042	0.020	3.70E-02
2.562061659	-0.011	0.010	2.87E-01	0.004	0.016	7.96E-01
3.876167	-0.004	0.004	3.14E-01	0.012	0.007	7.44E-02
3.11482	-0.011	0.009	2.61E-01	0.006	0.014	6.62E-01
3.218442	-0.015	0.015	2.97E-01	-0.008	0.022	7.13E-01
5.758508	0.009	0.010	4.11E-01	-0.009	0.016	5.65E-01
2.309400783	-0.014	0.012	2.46E-01	0.002	0.018	9.21E-01
1.667487399	-0.005	0.004	2.33E-01	0.007	0.006	2.28E-01
6.370142	0.009	0.009	2.98E-01	0.006	0.013	6.43E-01
6.071726	0.009	0.010	4.14E-01	-0.016	0.016	2.95E-01
6.495968	0.007	0.009	4.33E-01	-0.006	0.013	6.44E-01
3.237282	-0.006	0.006	3.41E-01	0.005	0.010	5.67E-01
7.424858	-0.010	0.009	2.84E-01	0.003	0.014	8.35E-01
3.915194	-0.008	0.009	3.40E-01	0.026	0.013	4.78E-02
1.056189381	0.006	0.006	2.75E-01	-0.002	0.009	8.09E-01

5.751443	0.008	0.010	4.11E-01	-0.009	0.014	5.32E-01
5.49239	0.007	0.007	3.05E-01	-0.005	0.010	6.59E-01
6.888585	0.009	0.008	2.98E-01	0.003	0.012	7.84E-01
4.383844	-0.012	0.013	3.67E-01	0.000	0.020	9.97E-01
6.019579	0.006	0.008	4.51E-01	0.006	0.012	6.28E-01
6.626504	0.006	0.009	4.95E-01	-0.006	0.014	6.62E-01
5.555975	0.008	0.010	4.17E-01	-0.029	0.015	5.81E-02
7.106593	0.015	0.010	1.34E-01	0.016	0.015	2.96E-01
6.85225	-0.008	0.009	3.37E-01	-0.016	0.013	1.95E-01
5.045271	0.007	0.007	3.32E-01	-0.002	0.010	8.84E-01
7.007682	-0.011	0.009	2.30E-01	-0.028	0.014	4.51E-02
1.776155126	-0.009	0.010	3.83E-01	-0.006	0.015	6.96E-01
3.977097	-0.008	0.009	3.89E-01	-0.005	0.013	7.05E-01
4.392928	-0.011	0.012	3.61E-01	0.012	0.018	5.13E-01
5.032823	0.007	0.008	4.07E-01	0.002	0.013	8.56E-01
7.998476	0.008	0.008	3.31E-01	-0.009	0.013	4.69E-01
7.198776	0.009	0.010	3.34E-01	0.012	0.014	3.89E-01
2.508568877	-0.011	0.012	3.96E-01	-0.019	0.018	2.96E-01
1.780192317	-0.009	0.010	3.70E-01	0.013	0.015	3.85E-01
6.143723	-0.008	0.009	3.68E-01	-0.031	0.013	1.69E-02
5.087325	0.007	0.007	3.18E-01	-0.008	0.010	4.10E-01
7.103902	-0.009	0.010	3.66E-01	0.003	0.014	8.18E-01
8.324143	-0.007	0.008	3.72E-01	-0.011	0.011	3.30E-01
8.831483	0.010	0.010	3.59E-01	-0.010	0.016	5.05E-01
3.678345	-0.004	0.005	3.88E-01	0.007	0.007	3.27E-01
4.405376	-0.013	0.014	3.50E-01	0.008	0.021	7.15E-01
0.769212381	0.006	0.005	2.48E-01	0.005	0.008	5.46E-01
4.328669	-0.014	0.015	3.49E-01	0.020	0.023	3.77E-01
1.907027404	-0.007	0.006	2.13E-01	-0.002	0.009	7.97E-01
3.157211	-0.009	0.008	2.74E-01	-0.005	0.012	6.53E-01
0.960306092	0.005	0.005	2.86E-01	0.013	0.007	7.12E-02
8.435838	-0.004	0.008	5.97E-01	-0.018	0.012	1.30E-01
0.85870345	0.010	0.009	2.71E-01	-0.003	0.013	8.01E-01
6.336835	0.009	0.008	2.20E-01	-0.008	0.012	4.76E-01
7.831942	-0.006	0.009	4.82E-01	0.010	0.013	4.31E-01
4.416478	-0.013	0.014	3.46E-01	0.003	0.021	8.95E-01
1.820564228	-0.009	0.009	3.19E-01	0.002	0.013	8.94E-01
3.543772	-0.013	0.011	2.54E-01	-0.018	0.017	2.84E-01
4.312857	-0.012	0.012	3.08E-01	0.013	0.018	4.55E-01
2.143203083	-0.012	0.012	3.17E-01	0.014	0.018	4.42E-01
6.250372	0.007	0.007	3.61E-01	0.001	0.011	8.99E-01
5.64984	0.007	0.008	3.99E-01	-0.002	0.012	8.70E-01
1.761352092	-0.010	0.008	2.30E-01	-0.005	0.013	6.97E-01

7.861884	0.006	0.008	4.77E-01	0.003	0.012	7.82E-01
4.105278	-0.010	0.012	4.15E-01	0.013	0.018	4.71E-01
1.749576951	-0.010	0.007	1.90E-01	0.005	0.011	6.61E-01
8.940151	0.008	0.007	2.07E-01	0.003	0.010	7.33E-01
3.236945	-0.006	0.006	3.82E-01	0.006	0.010	5.39E-01
6.050868	0.009	0.008	2.76E-01	-0.005	0.012	7.10E-01
1.425255933	0.006	0.005	2.20E-01	0.006	0.007	3.50E-01
6.289062	0.007	0.008	3.33E-01	0.002	0.011	8.61E-01
2.266673844	0.007	0.007	3.47E-01	0.014	0.011	1.97E-01
7.484407	-0.006	0.007	4.05E-01	0.006	0.011	5.98E-01
8.8695	-0.007	0.009	4.12E-01	-0.016	0.013	2.32E-01
3.781966	0.007	0.010	4.39E-01	0.015	0.014	2.96E-01
3.12525	-0.012	0.009	1.84E-01	0.005	0.013	7.08E-01
2.159351847	-0.008	0.009	3.18E-01	-0.015	0.013	2.29E-01
4.234132	0.011	0.013	3.90E-01	0.018	0.020	3.65E-01
8.346684	0.008	0.009	3.79E-01	-0.007	0.014	6.34E-01
5.888707	0.010	0.009	2.75E-01	0.000	0.014	9.85E-01
5.936144	0.007	0.007	3.29E-01	-0.006	0.011	5.68E-01
5.514594	0.006	0.007	4.38E-01	-0.001	0.011	9.01E-01
6.250036	0.006	0.007	3.71E-01	0.006	0.010	5.92E-01
2.279458282	-0.007	0.006	2.49E-01	-0.011	0.009	2.05E-01
7.398617	0.012	0.008	1.51E-01	0.000	0.012	9.82E-01
8.994316	-0.009	0.009	3.12E-01	-0.017	0.013	1.81E-01
6.192169	0.009	0.010	3.40E-01	-0.010	0.014	4.86E-01
1.678926107	-0.003	0.004	3.95E-01	0.000	0.006	9.42E-01
1.454189136	0.004	0.004	3.13E-01	0.001	0.005	8.86E-01
2.663664301	-0.017	0.014	2.29E-01	-0.006	0.022	7.74E-01
1.70012136	-0.003	0.004	4.10E-01	0.007	0.006	2.21E-01
2.04025471	0.005	0.004	2.21E-01	0.009	0.007	1.87E-01
2.262973085	0.007	0.006	2.97E-01	0.012	0.010	2.27E-01
0.975781992	0.005	0.006	4.04E-01	0.018	0.008	2.60E-02
6.82769	-0.007	0.008	3.97E-01	-0.009	0.013	4.92E-01
5.44327	0.006	0.007	4.40E-01	-0.019	0.011	9.38E-02
1.628461218	0.007	0.006	2.71E-01	0.000	0.009	9.75E-01
7.017438	0.004	0.009	6.82E-01	0.018	0.014	1.84E-01
5.039888	0.007	0.008	3.68E-01	-0.001	0.012	9.39E-01
2.859805	-0.008	0.008	3.15E-01	-0.016	0.012	1.72E-01
2.424124297	-0.004	0.009	6.48E-01	0.020	0.014	1.43E-01
8.788083	0.008	0.009	3.50E-01	-0.002	0.013	8.89E-01
7.820167	-0.009	0.010	3.74E-01	-0.009	0.015	5.53E-01
6.465016	0.010	0.009	2.77E-01	0.005	0.014	7.30E-01
1.327354049	0.014	0.010	1.43E-01	0.008	0.014	5.61E-01
7.630755	-0.012	0.012	3.33E-01	0.017	0.018	3.45E-01

1.236853682	0.007	0.008	3.54E-01	-0.006	0.011	5.66E-01
8.187215	0.008	0.011	4.67E-01	-0.001	0.016	9.69E-01
6.245662	-0.007	0.008	4.15E-01	-0.007	0.012	5.37E-01
5.808973	0.006	0.008	4.08E-01	-0.011	0.011	3.21E-01
7.080015	0.006	0.009	5.16E-01	0.009	0.013	5.05E-01
0.530008808	0.009	0.009	3.21E-01	-0.004	0.014	7.52E-01
3.860691	-0.006	0.005	2.72E-01	0.007	0.008	4.07E-01
7.688621	-0.012	0.010	2.13E-01	-0.005	0.014	7.23E-01
6.671922	0.008	0.010	3.96E-01	-0.004	0.014	7.84E-01
4.382498	-0.011	0.012	3.36E-01	0.011	0.018	5.34E-01
7.765665	-0.010	0.009	3.15E-01	-0.024	0.014	8.84E-02
3.281018	-0.011	0.008	1.81E-01	-0.020	0.013	1.10E-01
5.323837	0.018	0.018	3.03E-01	-0.028	0.026	2.99E-01
7.876014	0.007	0.008	3.75E-01	0.005	0.011	6.62E-01
7.491472	0.005	0.009	5.82E-01	-0.011	0.013	4.08E-01
0.986211402	0.006	0.005	2.75E-01	0.008	0.008	2.98E-01
6.273586	0.007	0.009	4.27E-01	-0.006	0.013	6.22E-01
2.03117103	0.006	0.005	2.07E-01	0.006	0.008	4.01E-01
2.568117446	-0.009	0.008	2.69E-01	-0.004	0.013	7.81E-01
3.64672	0.009	0.006	1.21E-01	0.014	0.008	9.71E-02
5.613842	0.007	0.008	3.45E-01	-0.009	0.012	4.38E-01
6.478137	0.006	0.009	4.71E-01	-0.015	0.013	2.50E-01
3.517194	0.008	0.007	2.86E-01	0.019	0.011	6.59E-02
5.892744	0.008	0.010	4.47E-01	-0.006	0.015	6.88E-01
8.528694	0.009	0.008	2.37E-01	0.018	0.011	1.20E-01
6.292763	0.008	0.008	3.15E-01	0.013	0.011	2.40E-01
7.79157	-0.010	0.009	2.67E-01	-0.003	0.014	8.05E-01
7.5379	0.005	0.009	5.83E-01	-0.019	0.014	1.64E-01
6.453914	-0.008	0.008	3.32E-01	-0.008	0.012	5.20E-01
7.42755	0.007	0.009	4.32E-01	-0.008	0.013	5.60E-01
7.71049	-0.010	0.009	2.62E-01	-0.006	0.013	6.39E-01
5.736976	0.008	0.011	4.78E-01	-0.022	0.017	1.98E-01
7.633447	-0.012	0.012	3.32E-01	0.002	0.019	9.32E-01
6.552489	0.009	0.008	2.89E-01	-0.003	0.012	8.14E-01
1.6974299	-0.003	0.004	4.41E-01	0.004	0.007	5.52E-01
1.044077808	0.003	0.005	5.49E-01	0.012	0.008	1.29E-01
1.222723514	0.007	0.008	3.70E-01	-0.001	0.011	9.32E-01
6.88522	0.012	0.008	1.35E-01	0.005	0.012	6.84E-01
5.972142	0.011	0.010	2.43E-01	-0.022	0.014	1.23E-01
6.504715	-0.012	0.009	1.87E-01	-0.013	0.013	3.51E-01
2.565089552	-0.007	0.007	3.75E-01	0.003	0.011	7.70E-01
3.211377	-0.017	0.015	2.41E-01	-0.005	0.022	8.22E-01
4.143968	-0.012	0.014	3.79E-01	0.018	0.021	4.00E-01

5.786768	-0.012	0.010	2.23E-01	-0.024	0.015	1.05E-01
5.611823	-0.006	0.008	4.53E-01	-0.029	0.013	2.13E-02
5.80258	0.009	0.010	3.63E-01	-0.008	0.015	6.02E-01
1.076038904	0.010	0.007	1.83E-01	0.003	0.011	8.02E-01
1.718961586	-0.007	0.006	2.10E-01	-0.010	0.008	2.34E-01
3.678009	-0.004	0.005	4.05E-01	0.006	0.007	3.82E-01
6.6514	0.009	0.009	2.93E-01	0.003	0.013	8.49E-01
5.621243	0.005	0.008	4.87E-01	0.002	0.011	8.79E-01
6.165928	0.007	0.008	3.68E-01	-0.015	0.012	1.88E-01
6.734835	-0.009	0.008	2.64E-01	-0.002	0.013	8.98E-01
8.783037	0.008	0.010	4.33E-01	0.002	0.015	8.95E-01
1.464954979	-0.004	0.005	4.36E-01	0.004	0.008	5.71E-01
0.986547835	0.006	0.005	2.89E-01	0.011	0.008	1.66E-01
7.831269	-0.008	0.010	4.22E-01	0.012	0.015	4.24E-01
8.651155	0.009	0.008	2.61E-01	-0.002	0.012	8.41E-01
3.534688	0.009	0.008	2.29E-01	0.024	0.012	4.12E-02
7.971898	0.010	0.009	2.62E-01	0.006	0.013	6.53E-01
7.600813	0.016	0.011	1.58E-01	0.006	0.017	7.25E-01
5.064111	0.006	0.007	3.85E-01	-0.014	0.010	1.60E-01
1.438376805	0.005	0.004	2.12E-01	0.000	0.006	9.93E-01
3.23459	-0.006	0.006	3.53E-01	0.008	0.009	3.80E-01
6.543405	-0.008	0.010	4.58E-01	-0.028	0.015	6.41E-02
3.28371	-0.009	0.010	3.81E-01	0.004	0.015	8.06E-01
7.094482	-0.010	0.009	2.59E-01	-0.025	0.013	5.56E-02
0.834816736	-0.010	0.008	2.18E-01	-0.007	0.013	5.90E-01
7.751198	-0.007	0.010	4.61E-01	-0.008	0.014	5.79E-01
6.223794	0.007	0.008	3.40E-01	-0.005	0.011	6.43E-01
6.208655	-0.007	0.008	4.18E-01	0.001	0.012	9.66E-01
2.842982873	-0.006	0.008	4.14E-01	-0.013	0.012	2.49E-01
0.627910692	0.007	0.007	3.30E-01	0.001	0.011	9.07E-01
5.696268	0.006	0.009	5.24E-01	-0.022	0.014	1.05E-01
5.058055	0.009	0.012	4.51E-01	0.001	0.018	9.59E-01
1.482113041	-0.004	0.006	4.85E-01	-0.001	0.009	9.30E-01
5.573806	0.009	0.008	2.95E-01	-0.008	0.013	5.47E-01
2.169444825	0.011	0.011	2.90E-01	-0.014	0.016	3.80E-01
8.367879	0.011	0.009	2.54E-01	0.016	0.014	2.54E-01
8.882621	-0.010	0.008	2.34E-01	-0.007	0.012	5.92E-01
1.890205774	0.010	0.008	2.12E-01	0.004	0.011	7.36E-01
1.825947149	-0.012	0.011	2.74E-01	-0.001	0.016	9.74E-01
4.24187	0.014	0.014	2.93E-01	-0.011	0.020	5.84E-01
3.946145	-0.007	0.008	3.24E-01	-0.002	0.011	8.75E-01
1.130540984	0.010	0.007	1.91E-01	0.001	0.011	9.58E-01
6.501687	-0.007	0.009	4.15E-01	-0.004	0.013	7.40E-01

7.731012	-0.008	0.011	4.44E-01	0.013	0.016	4.17E-01
8.831147	0.011	0.010	2.74E-01	-0.001	0.015	9.22E-01
8.310012	0.008	0.010	4.11E-01	0.012	0.014	4.19E-01
3.748323	0.007	0.009	4.04E-01	0.033	0.013	1.04E-02
8.380327	0.009	0.008	2.70E-01	0.014	0.013	2.60E-01
5.493062	0.007	0.009	4.68E-01	-0.011	0.014	4.43E-01
6.117818	-0.012	0.010	2.40E-01	0.017	0.016	2.67E-01
2.665010032	-0.009	0.007	2.37E-01	-0.003	0.011	7.98E-01
5.285147	-0.009	0.008	2.40E-01	-0.026	0.012	3.14E-02
8.020344	0.007	0.009	4.57E-01	-0.008	0.014	5.56E-01
4.416815	-0.012	0.013	3.41E-01	0.005	0.019	8.08E-01
0.930363592	-0.003	0.005	5.88E-01	-0.019	0.007	8.31E-03
7.617634	-0.017	0.014	2.47E-01	-0.009	0.021	6.75E-01
3.202966	0.006	0.007	3.63E-01	0.019	0.010	6.06E-02
6.108061	-0.008	0.009	3.56E-01	-0.002	0.013	8.46E-01
4.403021	-0.013	0.014	3.43E-01	0.003	0.021	8.82E-01
1.972295327	0.004	0.005	3.43E-01	-0.003	0.007	7.12E-01
6.464343	0.012	0.009	2.08E-01	-0.007	0.014	5.91E-01
0.817322241	0.003	0.005	5.37E-01	0.015	0.008	6.46E-02
7.67651	0.007	0.008	4.38E-01	0.009	0.013	4.89E-01
4.336407	-0.012	0.014	4.02E-01	0.025	0.021	2.35E-01
7.939937	-0.004	0.007	5.46E-01	0.000	0.011	9.88E-01
6.378217	0.012	0.009	1.75E-01	-0.003	0.013	8.20E-01
5.239729	0.002	0.005	7.08E-01	0.008	0.008	2.82E-01
5.565395	0.009	0.009	3.13E-01	-0.027	0.014	5.73E-02
5.957003	-0.006	0.009	5.10E-01	-0.009	0.013	4.76E-01
3.370509	0.019	0.013	1.26E-01	0.016	0.019	3.85E-01
2.645496942	-0.012	0.010	2.57E-01	0.010	0.015	5.29E-01
5.717463	0.006	0.008	4.46E-01	-0.008	0.013	5.37E-01
0.717065329	0.007	0.008	3.40E-01	0.010	0.011	3.85E-01
4.416142	-0.013	0.014	3.75E-01	0.006	0.022	7.71E-01
7.185991	0.011	0.009	1.99E-01	-0.012	0.013	3.72E-01
5.307688	0.016	0.015	2.90E-01	-0.023	0.022	3.06E-01
0.671983362	-0.010	0.009	2.76E-01	-0.018	0.014	1.81E-01
5.119959	0.009	0.008	2.53E-01	-0.008	0.011	4.94E-01
8.463762	-0.006	0.006	3.59E-01	-0.008	0.009	3.42E-01
0.994958649	0.005	0.006	3.95E-01	0.013	0.009	1.44E-01
1.969940299	0.004	0.004	3.16E-01	-0.002	0.007	7.96E-01
0.605033276	-0.010	0.009	2.58E-01	-0.002	0.014	8.91E-01
7.571543	0.012	0.009	2.16E-01	-0.006	0.014	6.79E-01
2.599742109	-0.015	0.017	3.68E-01	0.013	0.025	6.00E-01
7.322919	-0.008	0.006	2.02E-01	0.000	0.009	9.91E-01
7.774412	-0.007	0.013	5.90E-01	-0.017	0.020	3.94E-01

8.667977	-0.006	0.007	3.54E-01	-0.007	0.010	4.88E-01
5.009609	0.010	0.010	3.35E-01	0.002	0.015	8.92E-01
8.014625	-0.009	0.009	3.43E-01	-0.007	0.014	5.94E-01
7.129807	-0.009	0.011	4.26E-01	-0.011	0.017	5.25E-01
6.653755	-0.009	0.007	2.46E-01	-0.008	0.011	4.45E-01
8.361823	0.007	0.006	2.59E-01	-0.008	0.009	4.05E-01
3.375219	0.011	0.013	4.02E-01	-0.024	0.020	2.22E-01
7.737068	-0.008	0.011	4.91E-01	0.002	0.017	9.21E-01
7.227372	-0.011	0.011	3.15E-01	-0.003	0.016	8.65E-01
7.877697	0.007	0.009	4.41E-01	-0.001	0.014	9.71E-01
3.280009	-0.012	0.010	2.34E-01	0.006	0.015	6.75E-01
7.269763	-0.012	0.010	2.35E-01	-0.004	0.014	7.95E-01
1.089496207	0.007	0.007	2.93E-01	-0.002	0.011	8.32E-01
1.705840714	-0.004	0.005	3.64E-01	-0.002	0.007	7.51E-01
5.59702	-0.009	0.009	3.44E-01	-0.014	0.014	2.92E-01
8.58185	0.005	0.008	5.16E-01	0.014	0.012	2.46E-01
6.217402	0.012	0.010	2.39E-01	-0.011	0.015	4.83E-01
7.611578	-0.015	0.015	3.33E-01	-0.053	0.023	2.15E-02
0.781323954	0.004	0.004	4.05E-01	0.001	0.007	8.59E-01
7.248568	-0.013	0.010	1.91E-01	-0.001	0.015	9.42E-01
5.307352	0.016	0.015	2.90E-01	-0.023	0.022	3.07E-01
8.944861	0.008	0.008	3.27E-01	-0.006	0.012	5.92E-01
4.440365	-0.014	0.017	3.92E-01	0.020	0.025	4.29E-01
3.744959	-0.014	0.011	1.91E-01	-0.018	0.016	2.51E-01
3.497681	0.006	0.009	5.05E-01	0.032	0.014	2.25E-02
8.454342	0.010	0.013	4.41E-01	-0.002	0.019	9.11E-01
6.922901	-0.009	0.009	2.96E-01	-0.002	0.013	8.69E-01
2.384088818	-0.008	0.007	3.01E-01	0.002	0.011	8.33E-01
5.606777	-0.009	0.010	3.58E-01	0.002	0.015	9.08E-01
8.870173	-0.007	0.009	4.43E-01	-0.010	0.013	4.38E-01
7.040989	-0.011	0.010	2.88E-01	-0.003	0.015	8.19E-01
7.792579	-0.011	0.010	3.15E-01	-0.014	0.016	3.60E-01
8.423054	-0.008	0.008	3.07E-01	-0.022	0.012	6.94E-02
1.444096159	0.005	0.004	2.33E-01	0.007	0.006	2.46E-01
6.700519	0.005	0.011	6.62E-01	-0.005	0.017	7.59E-01
6.048176	0.005	0.009	5.43E-01	-0.006	0.013	6.56E-01
1.511382677	0.007	0.007	3.59E-01	-0.009	0.011	4.33E-01
8.819708	0.008	0.010	4.41E-01	0.010	0.015	4.83E-01
1.911064595	-0.017	0.015	2.61E-01	-0.054	0.022	1.44E-02
4.33237	-0.015	0.015	3.26E-01	0.023	0.023	3.17E-01
1.459572058	0.003	0.004	3.31E-01	0.001	0.005	8.02E-01
0.741288476	-0.006	0.006	3.59E-01	-0.001	0.010	9.52E-01
4.306128	-0.013	0.013	3.12E-01	0.012	0.019	5.26E-01

8.469482	-0.008	0.010	4.07E-01	-0.017	0.014	2.42E-01
6.365769	0.008	0.009	3.42E-01	0.014	0.013	2.80E-01
0.970735503	-0.005	0.005	3.75E-01	0.010	0.008	1.90E-01
8.018662	0.006	0.008	4.08E-01	-0.011	0.011	3.40E-01
7.521414	0.008	0.008	3.16E-01	-0.011	0.012	3.68E-01
7.555394	-0.009	0.009	3.47E-01	0.012	0.014	3.66E-01
7.241503	-0.012	0.010	2.06E-01	-0.020	0.014	1.58E-01
0.738597015	-0.007	0.007	2.71E-01	-0.009	0.010	3.55E-01
6.642989	0.006	0.008	4.67E-01	-0.013	0.011	2.54E-01
5.618888	-0.004	0.007	5.36E-01	-0.011	0.010	2.74E-01
8.738628	0.008	0.007	2.98E-01	0.007	0.011	5.43E-01
5.617542	0.008	0.009	4.09E-01	0.015	0.014	2.64E-01
1.119102276	0.008	0.007	2.81E-01	0.003	0.010	7.82E-01
0.712355273	0.006	0.008	4.50E-01	0.018	0.012	1.56E-01
0.639349401	-0.009	0.009	3.13E-01	-0.013	0.014	3.21E-01
8.394121	0.005	0.007	4.23E-01	0.003	0.010	7.74E-01
2.672747981	-0.010	0.008	2.00E-01	0.005	0.011	6.58E-01
6.894641	-0.011	0.011	3.33E-01	-0.006	0.017	7.11E-01
0.648096648	-0.010	0.011	3.53E-01	-0.016	0.016	3.07E-01
6.022944	0.009	0.006	1.48E-01	-0.009	0.009	3.42E-01
8.995662	0.009	0.008	2.48E-01	0.005	0.011	6.26E-01
4.336071	-0.011	0.014	4.34E-01	0.020	0.020	3.38E-01
7.460184	0.011	0.010	2.69E-01	-0.004	0.015	8.00E-01
2.138493027	-0.011	0.011	3.13E-01	0.013	0.016	4.20E-01
6.593533	-0.006	0.008	4.93E-01	-0.023	0.012	6.08E-02
4.344482	-0.010	0.012	3.99E-01	0.014	0.017	4.09E-01
5.897118	0.011	0.009	2.20E-01	0.019	0.014	1.77E-01
5.306679	0.016	0.015	2.86E-01	-0.023	0.022	2.90E-01
4.447766	-0.014	0.017	4.15E-01	0.018	0.025	4.66E-01
6.88152	0.009	0.008	2.71E-01	0.009	0.012	4.70E-01
6.606318	0.010	0.008	2.14E-01	0.020	0.011	7.68E-02
8.417671	-0.006	0.009	4.78E-01	-0.011	0.013	4.17E-01
7.447736	0.009	0.007	2.02E-01	0.003	0.010	7.68E-01
2.345735503	0.009	0.007	2.03E-01	0.010	0.010	3.48E-01
5.521659	0.006	0.009	4.89E-01	-0.012	0.013	3.51E-01
5.137117	0.007	0.007	3.46E-01	-0.015	0.011	1.80E-01
8.190242	-0.012	0.010	2.44E-01	-0.001	0.015	9.35E-01
7.32662	-0.008	0.007	2.20E-01	-0.003	0.010	7.91E-01
6.81356	-0.009	0.010	3.83E-01	-0.013	0.015	4.14E-01
4.410759	-0.012	0.014	3.75E-01	0.014	0.021	4.99E-01
7.309798	-0.014	0.010	1.60E-01	-0.009	0.015	5.55E-01
8.157945	-0.011	0.010	2.62E-01	-0.011	0.015	4.65E-01
2.793190849	-0.012	0.012	3.18E-01	-0.061	0.018	9.06E-04

6.330107	0.008	0.007	2.46E-01	0.001	0.010	9.16E-01
6.817598	0.001	0.010	8.99E-01	0.009	0.015	5.15E-01
6.883202	0.015	0.011	1.63E-01	0.026	0.016	1.04E-01
6.330443	0.007	0.007	2.91E-01	-0.003	0.010	7.49E-01
1.924521899	0.004	0.004	3.71E-01	0.006	0.006	3.17E-01
6.629532	0.010	0.009	2.89E-01	-0.014	0.014	3.26E-01
7.171525	-0.010	0.010	3.11E-01	-0.008	0.015	5.84E-01
6.605645	0.009	0.010	3.43E-01	0.019	0.015	1.96E-01
7.190365	0.010	0.010	2.97E-01	-0.022	0.015	1.33E-01
4.348855	-0.014	0.014	3.11E-01	0.011	0.020	5.75E-01
6.157517	0.010	0.007	1.75E-01	0.022	0.011	3.26E-02
6.025972	-0.009	0.010	3.80E-01	-0.017	0.015	2.62E-01
5.307015	0.016	0.015	2.88E-01	-0.022	0.022	3.12E-01
4.403694	-0.012	0.013	3.55E-01	0.005	0.020	8.17E-01
1.041386347	0.005	0.007	4.69E-01	0.021	0.011	5.68E-02
2.493092978	-0.024	0.018	1.92E-01	0.004	0.027	8.90E-01
4.362985	-0.012	0.013	3.41E-01	0.013	0.019	4.90E-01
3.987863	-0.008	0.007	2.67E-01	0.009	0.011	3.81E-01
4.449448	-0.015	0.017	3.81E-01	0.023	0.025	3.53E-01
1.731409591	-0.007	0.005	1.79E-01	0.000	0.008	9.58E-01
8.738291	0.011	0.010	2.80E-01	0.021	0.015	1.45E-01
3.984162	-0.009	0.009	3.08E-01	-0.001	0.013	9.69E-01
6.49294	-0.009	0.008	2.68E-01	-0.020	0.012	9.80E-02
6.489912	0.010	0.010	3.18E-01	-0.007	0.015	6.70E-01
4.318913	-0.012	0.013	3.49E-01	0.021	0.020	2.98E-01
8.880939	0.009	0.010	3.74E-01	0.014	0.015	3.35E-01
1.816190604	0.014	0.011	2.21E-01	0.012	0.017	4.87E-01
5.245784	-0.008	0.005	1.04E-01	-0.006	0.007	4.22E-01
2.614881576	-0.012	0.014	3.80E-01	0.034	0.021	1.02E-01
7.257988	-0.011	0.010	2.86E-01	-0.021	0.015	1.65E-01
6.956208	-0.006	0.006	3.26E-01	-0.007	0.010	4.40E-01
6.302519	0.007	0.007	2.96E-01	0.012	0.010	2.51E-01
5.054354	0.007	0.008	3.94E-01	-0.002	0.012	8.53E-01
1.493888182	-0.005	0.004	2.72E-01	-0.010	0.006	1.15E-01
4.154061	-0.011	0.013	3.64E-01	0.008	0.019	6.79E-01
4.352219	-0.014	0.014	3.10E-01	0.015	0.021	4.65E-01
8.26022	0.008	0.007	2.78E-01	0.001	0.011	9.09E-01
0.57441791	-0.009	0.010	3.69E-01	0.018	0.014	2.19E-01
0.74835356	-0.006	0.006	2.71E-01	-0.010	0.008	2.15E-01
1.636199168	0.007	0.006	2.61E-01	-0.001	0.010	9.53E-01
8.817689	0.010	0.010	3.05E-01	0.002	0.015	8.77E-01
3.263187	0.007	0.008	3.31E-01	0.024	0.011	2.97E-02
6.79943	0.009	0.009	3.51E-01	0.014	0.014	3.27E-01

8.2471	-0.005	0.007	4.89E-01	0.007	0.010	5.02E-01
8.589252	0.006	0.008	4.38E-01	-0.005	0.011	6.89E-01
4.41446	-0.013	0.014	3.50E-01	0.014	0.020	4.86E-01
2.085336677	-0.005	0.006	3.67E-01	0.006	0.008	4.50E-01
8.062062	-0.006	0.008	4.28E-01	-0.003	0.012	7.90E-01
5.827813	0.010	0.009	2.78E-01	0.020	0.014	1.48E-01
8.201345	0.006	0.008	4.84E-01	-0.009	0.012	4.56E-01
6.731807	-0.011	0.008	1.87E-01	-0.010	0.012	4.01E-01
7.29836	-0.011	0.009	2.17E-01	0.005	0.014	6.95E-01
1.039367751	0.006	0.007	4.38E-01	0.005	0.011	6.18E-01
1.186388794	0.006	0.006	2.87E-01	0.005	0.009	5.59E-01
7.857847	0.004	0.008	5.88E-01	0.012	0.011	3.02E-01
4.339099	-0.014	0.015	3.51E-01	0.012	0.022	5.85E-01
3.989545	-0.007	0.007	3.34E-01	0.005	0.011	6.43E-01
4.387881	-0.012	0.013	3.89E-01	0.015	0.020	4.52E-01
1.67926254	-0.004	0.004	3.60E-01	-0.001	0.006	9.05E-01
8.170393	0.011	0.010	2.65E-01	0.010	0.014	5.03E-01
5.323164	0.017	0.018	3.19E-01	-0.029	0.026	2.74E-01
3.195901	0.005	0.006	4.10E-01	0.016	0.008	5.66E-02
5.053009	0.008	0.008	3.36E-01	-0.005	0.012	6.97E-01
6.22985	-0.007	0.007	3.17E-01	-0.030	0.010	3.24E-03
6.244989	-0.008	0.010	4.45E-01	-0.019	0.015	2.08E-01
8.263248	-0.007	0.007	3.21E-01	-0.008	0.011	4.57E-01
5.667334	0.012	0.011	3.01E-01	0.007	0.017	6.63E-01
4.368705	-0.013	0.016	4.22E-01	0.007	0.023	7.62E-01
5.246121	-0.008	0.005	9.95E-02	-0.005	0.007	4.79E-01
6.04313	0.010	0.009	2.54E-01	0.000	0.014	9.86E-01
5.820411	0.006	0.009	5.20E-01	-0.002	0.013	8.97E-01
5.162686	0.011	0.010	2.70E-01	-0.015	0.015	3.08E-01
6.645008	-0.010	0.009	2.57E-01	-0.012	0.014	3.66E-01
6.226822	0.008	0.010	4.24E-01	0.002	0.015	9.07E-01
2.263309518	0.006	0.006	3.26E-01	0.012	0.010	2.00E-01
2.755510399	-0.009	0.007	1.96E-01	-0.020	0.011	5.54E-02
4.37106	-0.011	0.012	3.66E-01	0.011	0.018	5.35E-01
8.276369	0.009	0.010	3.89E-01	0.042	0.015	5.32E-03
2.676112307	-0.009	0.007	1.98E-01	0.003	0.010	7.47E-01
6.434737	0.007	0.009	3.92E-01	0.002	0.013	8.99E-01
4.427917	-0.012	0.014	3.90E-01	0.008	0.021	7.22E-01
6.244653	-0.006	0.009	5.52E-01	-0.020	0.014	1.47E-01
4.345491	-0.012	0.015	4.33E-01	0.018	0.023	4.22E-01
7.370356	0.010	0.009	2.67E-01	0.008	0.013	5.50E-01
1.871365549	-0.008	0.008	3.11E-01	0.003	0.012	8.14E-01
7.378094	-0.008	0.009	4.03E-01	-0.012	0.014	3.75E-01

7.813438	0.006	0.008	4.36E-01	0.012	0.012	3.12E-01
7.524442	0.007	0.008	3.86E-01	0.001	0.013	9.53E-01
6.003431	0.008	0.008	3.19E-01	-0.001	0.013	9.25E-01
1.514074138	0.008	0.008	3.00E-01	-0.013	0.012	2.60E-01
3.803161	0.007	0.006	2.17E-01	0.013	0.008	1.18E-01
7.918069	-0.007	0.010	4.96E-01	-0.045	0.015	2.40E-03
8.169047	0.008	0.010	3.88E-01	-0.016	0.015	2.60E-01
4.162135	-0.017	0.018	3.44E-01	-0.019	0.027	4.87E-01
2.485355028	-0.016	0.011	1.48E-01	0.002	0.017	8.85E-01
6.506397	0.008	0.010	4.52E-01	-0.024	0.016	1.32E-01
6.401094	0.008	0.006	1.95E-01	0.007	0.010	4.56E-01
1.648310741	0.006	0.005	2.67E-01	0.006	0.007	4.24E-01
1.798359677	-0.006	0.009	4.89E-01	-0.005	0.013	7.16E-01
5.80729	0.009	0.010	3.46E-01	-0.003	0.015	8.37E-01
5.075213	0.009	0.010	3.29E-01	-0.003	0.014	8.39E-01
8.406569	-0.006	0.008	4.77E-01	-0.020	0.012	9.89E-02
5.633691	-0.008	0.009	3.68E-01	-0.008	0.013	5.49E-01
4.415469	-0.012	0.013	3.74E-01	0.007	0.020	7.32E-01
6.061297	0.006	0.008	4.40E-01	-0.002	0.012	8.80E-01
1.078393932	0.009	0.008	2.52E-01	-0.002	0.011	8.66E-01
7.117695	-0.007	0.008	4.27E-01	-0.007	0.013	5.64E-01
3.176051	-0.006	0.006	3.14E-01	0.005	0.008	5.74E-01
6.534658	-0.010	0.009	2.85E-01	-0.005	0.014	7.18E-01
4.108979	-0.007	0.009	4.51E-01	0.006	0.014	6.65E-01
4.40403	-0.012	0.013	3.74E-01	0.011	0.019	5.90E-01
7.742114	-0.010	0.011	3.67E-01	-0.003	0.016	8.33E-01
3.265542	-0.007	0.007	3.24E-01	-0.002	0.011	8.44E-01
4.223029	0.010	0.013	4.16E-01	0.024	0.019	2.10E-01
5.036187	0.007	0.008	4.08E-01	-0.010	0.012	4.14E-01
8.718442	-0.004	0.008	5.86E-01	0.000	0.012	9.93E-01
8.17914	-0.009	0.007	1.87E-01	-0.010	0.011	3.40E-01
1.87271128	-0.007	0.007	3.71E-01	0.010	0.011	3.72E-01
4.344145	-0.011	0.013	3.88E-01	0.014	0.019	4.53E-01
7.462875	0.009	0.010	3.89E-01	-0.008	0.015	5.83E-01
6.573347	0.004	0.007	6.02E-01	-0.001	0.011	9.20E-01
4.338089	-0.012	0.014	3.95E-01	0.010	0.020	6.16E-01
5.045943	0.006	0.008	4.29E-01	0.002	0.012	8.96E-01
3.580443	-0.005	0.006	4.74E-01	0.009	0.009	3.31E-01
5.073531	0.009	0.009	2.98E-01	-0.006	0.013	6.50E-01
1.738474676	-0.003	0.006	5.46E-01	0.006	0.009	4.62E-01
8.435165	0.005	0.007	4.45E-01	-0.008	0.010	4.16E-01
8.575794	-0.008	0.010	4.16E-01	-0.018	0.015	2.06E-01
5.149228	0.008	0.007	2.76E-01	-0.007	0.010	4.70E-01

0.504103499	-0.008	0.008	3.29E-01	-0.008	0.012	5.01E-01
8.960673	-0.010	0.009	2.67E-01	-0.007	0.013	5.92E-01
7.464894	0.006	0.009	5.21E-01	0.002	0.014	8.96E-01
7.02955	-0.009	0.010	3.89E-01	-0.016	0.015	2.78E-01
1.971286029	0.004	0.005	3.59E-01	-0.002	0.007	7.32E-01
4.352892	-0.014	0.014	3.18E-01	0.015	0.022	4.84E-01
8.194953	-0.009	0.008	2.72E-01	-0.015	0.013	2.26E-01
8.456697	0.006	0.007	4.23E-01	0.005	0.011	6.56E-01
6.551479	-0.010	0.010	3.05E-01	-0.014	0.015	3.41E-01
1.733091754	-0.006	0.005	2.38E-01	0.000	0.008	9.89E-01
7.589374	-0.005	0.009	5.56E-01	-0.020	0.014	1.37E-01
2.283495473	-0.004	0.005	3.97E-01	-0.014	0.007	6.05E-02
5.570105	0.007	0.009	4.00E-01	-0.006	0.013	6.63E-01
0.628247125	0.007	0.006	2.69E-01	0.008	0.009	3.70E-01
6.004776	0.008	0.008	3.01E-01	0.013	0.012	2.71E-01
6.088212	0.008	0.008	3.47E-01	-0.017	0.012	1.55E-01
8.24609	-0.003	0.007	6.17E-01	0.009	0.010	3.83E-01
6.731471	-0.019	0.011	9.41E-02	-0.022	0.017	1.77E-01
3.745632	-0.012	0.010	2.42E-01	-0.010	0.015	5.11E-01
0.9872207	0.005	0.006	3.53E-01	0.012	0.008	1.51E-01
5.7215	0.010	0.009	2.62E-01	-0.002	0.013	8.86E-01
7.564814	0.005	0.007	5.16E-01	0.001	0.010	8.91E-01
5.536799	0.009	0.008	2.92E-01	-0.003	0.012	8.08E-01
7.792243	-0.010	0.009	3.02E-01	-0.009	0.014	5.39E-01
2.676785172	-0.009	0.007	1.78E-01	0.008	0.010	4.00E-01
7.814111	-0.011	0.010	2.46E-01	0.021	0.014	1.50E-01
0.985874969	0.006	0.005	2.50E-01	0.008	0.007	2.74E-01
8.08864	-0.009	0.008	3.07E-01	-0.007	0.012	5.55E-01
7.734376	-0.006	0.011	5.71E-01	-0.018	0.016	2.74E-01
4.317903	-0.013	0.014	3.54E-01	0.027	0.021	1.97E-01
6.325397	-0.006	0.010	5.52E-01	-0.018	0.015	2.34E-01
1.73645608	-0.007	0.005	1.98E-01	0.008	0.008	3.32E-01
6.083502	0.011	0.010	2.60E-01	-0.001	0.014	9.63E-01
5.676418	0.007	0.010	4.67E-01	-0.002	0.015	8.75E-01
8.900788	-0.004	0.009	6.36E-01	-0.003	0.013	7.98E-01
7.693668	-0.010	0.010	3.01E-01	0.005	0.015	7.38E-01
1.786248104	-0.007	0.009	4.86E-01	0.006	0.014	6.76E-01
1.737801811	-0.003	0.006	5.83E-01	0.009	0.008	2.64E-01
1.734437485	-0.005	0.005	2.78E-01	-0.005	0.007	5.14E-01
7.220307	-0.010	0.007	1.87E-01	-0.007	0.011	5.05E-01
5.957676	0.006	0.007	3.92E-01	0.007	0.010	5.06E-01
7.554385	-0.009	0.009	3.48E-01	0.024	0.014	8.12E-02
6.949816	-0.008	0.009	3.99E-01	-0.011	0.014	4.48E-01

2.175837044	0.011	0.011	2.96E-01	-0.015	0.016	3.65E-01
6.985477	-0.006	0.006	3.05E-01	-0.005	0.009	5.84E-01
5.440915	-0.010	0.009	2.99E-01	-0.015	0.014	3.03E-01
4.173574	-0.011	0.012	3.51E-01	-0.004	0.018	8.14E-01
6.416233	0.004	0.011	6.99E-01	-0.016	0.016	3.14E-01
0.768875948	0.006	0.005	2.76E-01	0.005	0.008	5.60E-01
5.143172	0.009	0.009	3.42E-01	-0.020	0.014	1.51E-01
6.550134	-0.008	0.008	3.27E-01	-0.012	0.012	3.22E-01
3.282364	-0.008	0.008	3.24E-01	-0.020	0.012	1.01E-01
1.982724737	0.007	0.006	2.68E-01	-0.005	0.009	5.94E-01
3.550501	0.011	0.015	4.90E-01	0.060	0.023	9.07E-03
6.12892	0.006	0.010	5.57E-01	-0.019	0.015	1.94E-01
7.629746	-0.010	0.013	4.36E-01	0.019	0.020	3.28E-01
1.687000489	-0.004	0.004	3.28E-01	0.004	0.006	5.45E-01
5.298941	0.014	0.014	3.04E-01	-0.021	0.020	3.10E-01
7.163787	0.012	0.011	2.71E-01	-0.002	0.016	8.95E-01
8.196298	-0.006	0.009	5.11E-01	-0.016	0.014	2.43E-01
0.649105946	-0.013	0.011	2.37E-01	-0.002	0.016	8.97E-01
6.468717	0.008	0.011	4.53E-01	-0.012	0.016	4.68E-01
1.637881331	0.006	0.006	2.89E-01	-0.002	0.009	7.82E-01
6.338181	0.007	0.008	3.67E-01	0.008	0.012	4.94E-01
6.966301	-0.012	0.010	2.56E-01	-0.006	0.015	6.86E-01
6.210673	0.006	0.008	4.93E-01	-0.005	0.012	6.84E-01
1.23651725	0.007	0.008	3.75E-01	-0.006	0.011	5.74E-01
2.309737216	-0.015	0.014	2.84E-01	0.001	0.021	9.65E-01
7.31249	-0.010	0.009	2.27E-01	-0.021	0.013	1.09E-01
2.776032787	-0.012	0.011	2.73E-01	-0.046	0.017	5.64E-03
2.574846097	0.013	0.010	1.97E-01	0.015	0.015	3.38E-01
6.551143	-0.010	0.009	2.53E-01	-0.020	0.013	1.37E-01
6.206636	0.007	0.007	3.32E-01	-0.007	0.011	4.82E-01
5.998048	0.008	0.010	4.04E-01	0.009	0.015	5.27E-01
7.351852	-0.007	0.010	4.80E-01	-0.010	0.015	5.02E-01
8.495387	-0.008	0.007	2.94E-01	-0.016	0.011	1.40E-01
5.025421	0.009	0.010	3.58E-01	-0.009	0.014	5.26E-01
5.605094	-0.007	0.009	4.17E-01	-0.017	0.013	1.97E-01
4.323959	-0.012	0.013	3.63E-01	0.013	0.020	5.23E-01
6.658801	-0.008	0.009	3.76E-01	-0.016	0.014	2.45E-01
5.162349	0.010	0.009	2.94E-01	-0.020	0.014	1.45E-01
5.317445	0.016	0.016	3.17E-01	-0.030	0.025	2.24E-01
4.153724	-0.010	0.012	4.01E-01	0.010	0.018	5.84E-01
5.059401	0.006	0.008	3.97E-01	-0.005	0.011	6.56E-01
1.045759971	0.005	0.006	3.69E-01	0.005	0.008	5.14E-01
3.26386	0.007	0.008	3.67E-01	0.020	0.011	7.85E-02

5.059064	0.008	0.009	3.86E-01	-0.012	0.014	3.85E-01
2.287196232	-0.007	0.007	3.33E-01	-0.002	0.011	8.88E-01
8.164001	0.006	0.006	3.63E-01	0.007	0.010	4.47E-01
5.785759	0.006	0.009	4.92E-01	0.008	0.014	5.51E-01
8.459725	0.004	0.006	5.65E-01	-0.014	0.009	1.37E-01
1.420209445	0.006	0.005	1.97E-01	0.002	0.007	7.56E-01
8.901125	-0.008	0.009	3.93E-01	-0.022	0.013	9.44E-02
5.930088	0.010	0.009	2.45E-01	-0.009	0.013	4.95E-01
4.325305	-0.013	0.014	3.37E-01	0.013	0.020	5.16E-01
7.089435	-0.006	0.010	5.05E-01	-0.003	0.014	8.30E-01
5.730584	0.008	0.009	4.19E-01	-0.002	0.014	9.06E-01
6.952507	-0.008	0.010	3.88E-01	-0.001	0.014	9.19E-01
0.852647663	-0.007	0.007	2.93E-01	-0.013	0.010	2.19E-01
0.579800832	0.006	0.007	3.57E-01	-0.009	0.010	3.84E-01
0.718411059	0.006	0.008	4.17E-01	0.012	0.011	2.76E-01
3.935043	-0.003	0.006	6.43E-01	0.019	0.009	4.37E-02
1.907700269	-0.007	0.006	2.41E-01	-0.009	0.009	3.14E-01
5.801235	0.004	0.009	6.91E-01	-0.016	0.014	2.37E-01
8.363505	0.009	0.008	2.90E-01	0.004	0.013	7.80E-01
2.665346464	-0.008	0.007	2.27E-01	-0.005	0.010	6.31E-01
5.448317	0.009	0.007	2.08E-01	0.010	0.011	3.63E-01
5.084297	0.010	0.010	2.95E-01	-0.004	0.014	8.04E-01
5.532425	0.006	0.007	3.84E-01	-0.001	0.010	8.84E-01
1.903663078	-0.006	0.006	3.15E-01	0.000	0.010	9.84E-01
0.804537803	0.003	0.006	6.32E-01	0.010	0.008	2.21E-01
1.029611206	0.005	0.007	4.61E-01	0.021	0.011	5.06E-02
8.691527	0.006	0.008	4.27E-01	-0.008	0.011	4.60E-01
3.603993	-0.007	0.008	3.54E-01	-0.004	0.011	7.16E-01
5.054691	0.006	0.007	3.84E-01	0.001	0.011	9.12E-01
1.439722535	0.005	0.004	2.43E-01	0.005	0.006	4.25E-01
3.876504	-0.004	0.004	3.38E-01	0.013	0.007	5.45E-02
2.085000245	-0.005	0.006	3.81E-01	0.008	0.008	3.70E-01
7.875342	0.008	0.009	3.67E-01	0.005	0.013	6.81E-01
5.624271	0.007	0.009	4.42E-01	-0.007	0.013	5.84E-01
1.034994128	0.004	0.005	3.95E-01	-0.001	0.007	8.44E-01
1.495570345	-0.005	0.004	2.26E-01	-0.009	0.007	1.73E-01
8.099069	0.007	0.009	4.00E-01	-0.035	0.013	6.94E-03
7.073286	-0.009	0.009	3.05E-01	-0.013	0.013	3.25E-01
1.683636163	-0.003	0.004	5.11E-01	0.008	0.006	1.83E-01
2.549950086	-0.013	0.012	2.76E-01	-0.015	0.018	4.12E-01
1.918129679	-0.005	0.005	2.85E-01	-0.003	0.007	6.25E-01
7.40299	0.009	0.010	3.71E-01	0.009	0.015	5.58E-01
6.528938	0.007	0.007	3.29E-01	0.007	0.011	5.03E-01

0.644395889	0.006	0.009	4.75E-01	-0.007	0.013	5.91E-01
6.502697	-0.009	0.010	3.96E-01	0.001	0.015	9.66E-01
6.595888	-0.009	0.007	2.35E-01	-0.004	0.011	7.16E-01
0.578791534	-0.005	0.009	5.61E-01	0.020	0.013	1.26E-01
5.148892	0.007	0.008	3.56E-01	-0.011	0.011	3.45E-01
8.283771	-0.005	0.008	5.16E-01	-0.003	0.012	7.82E-01
6.518172	0.008	0.008	2.88E-01	0.001	0.011	9.08E-01
5.407609	0.009	0.008	2.55E-01	-0.019	0.012	1.12E-01
8.791111	0.008	0.009	3.70E-01	-0.006	0.013	6.60E-01
4.314203	-0.013	0.013	3.23E-01	0.009	0.020	6.60E-01
0.603351113	0.008	0.010	4.33E-01	-0.027	0.015	6.08E-02
8.962355	-0.009	0.009	3.44E-01	-0.010	0.014	4.48E-01
5.403571	0.008	0.008	3.16E-01	-0.002	0.011	8.26E-01
2.3622207	0.010	0.007	1.69E-01	0.014	0.010	1.77E-01
3.783648	-0.012	0.010	2.31E-01	0.007	0.016	6.59E-01
4.23615	0.012	0.013	3.63E-01	0.020	0.020	3.21E-01
7.572216	-0.005	0.009	5.87E-01	0.008	0.014	5.85E-01
2.180547101	0.012	0.011	2.57E-01	-0.013	0.016	4.22E-01
1.521812087	0.009	0.009	3.22E-01	-0.019	0.014	1.71E-01
1.511046244	0.007	0.007	3.53E-01	-0.010	0.011	3.56E-01
6.496977	0.011	0.010	2.56E-01	-0.009	0.015	5.59E-01
8.189233	-0.010	0.008	2.19E-01	0.004	0.012	7.50E-01
1.472020064	-0.004	0.004	4.12E-01	0.000	0.007	9.75E-01
7.811083	-0.010	0.009	2.73E-01	-0.012	0.014	3.85E-01
4.3465	-0.012	0.015	4.25E-01	0.033	0.022	1.48E-01
0.78334255	0.003	0.004	4.33E-01	0.008	0.006	2.25E-01
6.454923	0.009	0.009	3.44E-01	-0.023	0.013	9.01E-02
6.581422	0.005	0.008	5.53E-01	0.010	0.012	4.11E-01
5.703669	0.011	0.009	2.42E-01	-0.021	0.014	1.16E-01
1.636872033	0.006	0.006	2.94E-01	-0.002	0.009	8.29E-01
7.58029	0.010	0.010	3.39E-01	0.003	0.015	8.38E-01
1.912073893	-0.015	0.012	2.30E-01	-0.042	0.018	2.33E-02
2.610844385	-0.015	0.014	2.87E-01	0.020	0.021	3.22E-01
0.97948275	0.006	0.007	3.99E-01	0.013	0.011	2.18E-01
0.624209934	-0.009	0.010	3.81E-01	0.004	0.015	7.81E-01
7.542946	-0.015	0.011	1.72E-01	0.002	0.016	9.19E-01
6.953516	-0.007	0.008	4.22E-01	-0.008	0.012	5.36E-01
5.803926	0.005	0.008	4.86E-01	-0.009	0.012	4.49E-01
8.453333	0.017	0.016	2.85E-01	0.011	0.023	6.46E-01
3.265879	-0.007	0.007	3.07E-01	-0.005	0.011	6.30E-01
6.39302	0.007	0.010	4.52E-01	-0.005	0.014	7.08E-01
1.65335723	0.003	0.005	5.22E-01	0.011	0.007	1.19E-01
7.351516	-0.010	0.010	3.15E-01	-0.004	0.015	7.79E-01

5.73664	0.007	0.010	5.01E-01	-0.004	0.015	7.76E-01
5.114576	0.012	0.011	2.88E-01	0.001	0.017	9.40E-01
8.373935	0.008	0.009	3.86E-01	0.001	0.013	9.29E-01
8.470154	-0.004	0.008	6.12E-01	-0.006	0.012	6.27E-01
1.44947908	0.004	0.004	3.64E-01	0.005	0.006	3.73E-01
5.1425	0.006	0.007	3.66E-01	-0.014	0.010	1.68E-01
5.560685	0.006	0.008	4.58E-01	-0.009	0.011	4.49E-01
1.762024957	-0.010	0.009	2.35E-01	0.018	0.013	1.68E-01
2.362557132	0.008	0.007	2.57E-01	0.016	0.011	1.33E-01
8.221531	0.010	0.008	2.36E-01	-0.011	0.013	3.72E-01
6.629868	0.009	0.008	3.00E-01	0.011	0.012	3.64E-01
7.471959	0.011	0.009	2.19E-01	-0.001	0.013	9.62E-01
5.515267	0.005	0.008	5.26E-01	-0.012	0.012	3.08E-01
1.869346954	-0.008	0.009	3.97E-01	-0.004	0.013	7.74E-01
5.889716	0.008	0.009	4.01E-01	-0.002	0.014	9.14E-01
1.970276731	0.004	0.004	3.35E-01	-0.003	0.007	6.85E-01
3.704587	0.007	0.006	2.42E-01	0.023	0.008	5.74E-03
7.255633	0.007	0.011	5.37E-01	0.020	0.016	2.11E-01
0.9418023	-0.003	0.005	5.15E-01	-0.019	0.008	1.40E-02
4.32295	-0.012	0.013	3.35E-01	0.018	0.019	3.45E-01
7.389196	0.008	0.008	2.95E-01	0.009	0.012	4.71E-01
0.745325667	-0.007	0.006	2.60E-01	-0.009	0.009	3.16E-01
7.06252	0.009	0.010	3.71E-01	-0.011	0.016	4.90E-01
7.012392	0.008	0.009	3.92E-01	-0.016	0.014	2.44E-01
7.012728	0.010	0.010	3.33E-01	-0.008	0.016	6.17E-01
0.650451676	-0.011	0.011	3.05E-01	-0.009	0.016	5.74E-01
2.284168339	-0.005	0.005	3.92E-01	-0.007	0.008	3.47E-01
7.819494	-0.008	0.008	3.26E-01	0.006	0.012	6.04E-01
3.966331	-0.009	0.008	2.63E-01	0.008	0.012	5.12E-01
5.694249	0.006	0.008	4.44E-01	-0.015	0.011	1.92E-01
5.3235	0.018	0.018	3.21E-01	-0.028	0.026	2.88E-01
7.30206	-0.013	0.010	2.04E-01	0.003	0.015	8.34E-01
0.834480303	-0.010	0.008	2.36E-01	-0.006	0.013	6.36E-01
7.782823	-0.008	0.008	3.09E-01	0.008	0.012	5.27E-01
5.010954	0.008	0.009	3.60E-01	-0.009	0.014	5.02E-01
8.679415	-0.010	0.009	2.47E-01	-0.009	0.013	4.80E-01
8.080565	0.008	0.008	3.68E-01	-0.003	0.012	7.90E-01
5.540499	0.009	0.008	2.96E-01	0.005	0.013	6.68E-01
4.331697	-0.013	0.014	3.44E-01	0.012	0.021	5.47E-01
0.778968926	0.003	0.005	4.41E-01	-0.004	0.007	5.86E-01
2.285850502	-0.011	0.011	3.31E-01	0.004	0.016	7.95E-01
5.510893	0.007	0.009	4.48E-01	0.000	0.013	9.83E-01
7.249241	-0.009	0.008	2.93E-01	-0.014	0.013	2.67E-01

7.964833	0.006	0.007	4.12E-01	-0.005	0.011	6.65E-01
7.672809	0.008	0.009	3.82E-01	0.012	0.013	3.50E-01
8.321115	-0.008	0.008	3.19E-01	-0.011	0.013	3.60E-01
2.559370198	0.004	0.008	5.97E-01	0.000	0.012	9.88E-01
1.515083435	0.008	0.008	3.33E-01	-0.014	0.012	2.44E-01
4.399656	-0.012	0.015	4.15E-01	0.017	0.023	4.45E-01
5.883997	0.007	0.007	3.61E-01	0.011	0.011	3.26E-01
2.296952777	-0.011	0.011	3.17E-01	0.000	0.016	9.98E-01
6.924583	0.007	0.011	5.04E-01	-0.006	0.017	7.35E-01
4.283251	0.012	0.011	2.59E-01	0.006	0.016	7.21E-01
2.181219966	0.012	0.011	2.76E-01	-0.015	0.016	3.58E-01
2.65828138	-0.007	0.008	3.61E-01	0.014	0.012	2.45E-01
7.381795	-0.005	0.006	4.40E-01	-0.005	0.009	6.08E-01
7.248904	-0.010	0.010	2.87E-01	-0.007	0.014	6.00E-01
8.255847	-0.010	0.009	2.95E-01	-0.020	0.014	1.64E-01
5.072522	0.007	0.008	3.76E-01	-0.016	0.012	1.80E-01
7.244194	-0.007	0.009	4.68E-01	-0.006	0.014	6.53E-01
6.260129	0.005	0.007	4.41E-01	-0.012	0.011	2.41E-01
1.432321018	0.006	0.004	1.98E-01	0.005	0.006	4.78E-01
5.028113	0.009	0.010	3.86E-01	-0.021	0.015	1.62E-01
6.996916	0.009	0.008	2.72E-01	-0.001	0.012	9.48E-01
4.321941	-0.011	0.013	3.71E-01	0.019	0.019	3.16E-01
5.041233	0.008	0.008	3.57E-01	0.005	0.013	7.04E-01
0.506122094	-0.007	0.010	5.05E-01	0.002	0.015	8.75E-01
8.13204	0.008	0.009	3.77E-01	-0.001	0.013	9.11E-01
6.014533	0.008	0.008	3.16E-01	0.004	0.011	7.08E-01
5.115921	0.009	0.009	3.25E-01	0.000	0.013	9.76E-01
7.174216	-0.006	0.009	4.74E-01	-0.005	0.013	6.83E-01
2.182902129	0.011	0.011	2.98E-01	-0.020	0.016	2.27E-01
2.427488622	-0.008	0.008	3.19E-01	0.014	0.012	2.52E-01
1.731073159	-0.007	0.005	2.06E-01	-0.001	0.008	9.03E-01
4.162471	-0.014	0.015	3.38E-01	-0.014	0.022	5.38E-01
5.894426	0.009	0.010	3.54E-01	-0.027	0.015	7.40E-02
3.304232	-0.012	0.010	2.45E-01	-0.008	0.015	6.01E-01
1.519457059	0.008	0.009	3.38E-01	-0.014	0.013	2.69E-01
8.37999	0.010	0.009	2.27E-01	0.008	0.013	5.28E-01
1.775145828	-0.011	0.011	3.44E-01	-0.021	0.017	2.22E-01
6.801785	0.008	0.011	4.54E-01	-0.002	0.016	8.96E-01
5.634701	-0.008	0.011	4.51E-01	-0.011	0.016	4.89E-01
5.528724	0.006	0.007	3.32E-01	0.005	0.010	5.93E-01
2.370295082	-0.005	0.007	4.53E-01	0.013	0.010	2.10E-01
2.593013457	-0.014	0.019	4.56E-01	0.026	0.028	3.62E-01
3.875831	-0.004	0.004	3.40E-01	0.012	0.007	6.04E-02

7.46523	0.006	0.010	5.36E-01	-0.004	0.014	7.86E-01
6.774534	-0.010	0.009	2.48E-01	0.020	0.013	1.12E-01
8.128339	0.005	0.008	5.01E-01	0.000	0.012	9.86E-01
1.518111329	0.008	0.009	3.76E-01	-0.015	0.013	2.50E-01
3.667916	-0.004	0.004	4.08E-01	0.006	0.006	3.63E-01
4.155743	-0.012	0.014	3.81E-01	0.014	0.021	5.16E-01
3.178406	-0.005	0.005	3.86E-01	0.014	0.008	8.66E-02
8.738964	0.008	0.008	3.12E-01	0.008	0.011	4.86E-01
2.187948618	0.013	0.011	2.56E-01	-0.017	0.017	2.94E-01
7.391888	0.008	0.008	3.18E-01	0.006	0.011	5.72E-01
1.971622461	0.004	0.005	3.76E-01	-0.002	0.007	7.82E-01
0.909841204	-0.005	0.005	4.06E-01	-0.024	0.008	2.96E-03
5.006917	0.010	0.010	3.51E-01	0.000	0.016	9.81E-01
6.502024	-0.007	0.009	4.32E-01	0.002	0.013	8.85E-01
8.176449	-0.014	0.011	1.83E-01	-0.005	0.016	7.55E-01
8.520619	0.007	0.009	4.25E-01	-0.005	0.013	7.04E-01
3.857327	0.004	0.006	4.83E-01	0.015	0.008	8.66E-02
2.839282114	-0.005	0.008	5.06E-01	-0.015	0.012	2.22E-01
6.947797	-0.006	0.009	4.78E-01	0.003	0.013	7.90E-01
5.074877	0.010	0.010	3.04E-01	-0.008	0.015	6.13E-01
8.522974	0.005	0.009	6.12E-01	0.006	0.014	6.83E-01
6.303192	0.007	0.009	4.56E-01	-0.011	0.013	4.08E-01
5.424094	0.006	0.007	3.66E-01	-0.011	0.010	2.52E-01
7.731348	-0.008	0.012	5.19E-01	0.017	0.017	3.20E-01
6.695809	-0.007	0.009	4.14E-01	-0.013	0.013	3.18E-01
3.979789	-0.009	0.013	4.60E-01	-0.002	0.019	9.31E-01
4.309156	-0.012	0.013	3.58E-01	0.008	0.019	6.56E-01
5.86583	0.004	0.007	5.04E-01	-0.005	0.010	6.40E-01
2.098121116	-0.004	0.008	6.25E-01	-0.006	0.012	6.15E-01
4.42859	-0.012	0.014	4.18E-01	0.007	0.021	7.41E-01
3.639655	0.007	0.005	1.52E-01	0.012	0.008	1.17E-01
8.575121	-0.004	0.008	6.25E-01	0.007	0.011	5.48E-01
1.453516271	0.004	0.004	2.50E-01	0.003	0.005	6.29E-01
1.517102031	0.008	0.008	3.37E-01	-0.013	0.012	3.08E-01
1.657730854	0.005	0.005	3.25E-01	0.011	0.007	1.22E-01
2.755173966	-0.009	0.007	2.09E-01	-0.020	0.011	6.51E-02
5.918313	0.006	0.008	4.94E-01	0.004	0.012	7.36E-01
1.669169562	0.002	0.004	5.25E-01	0.004	0.006	4.99E-01
1.910728162	-0.015	0.013	2.67E-01	-0.044	0.020	2.27E-02
8.434493	0.008	0.010	3.97E-01	-0.007	0.015	6.43E-01
0.97039907	-0.004	0.005	3.91E-01	0.009	0.008	2.70E-01
1.692046978	-0.004	0.004	2.77E-01	0.006	0.006	2.83E-01
8.005541	0.011	0.010	2.63E-01	-0.010	0.015	5.17E-01

5.001534	0.010	0.011	3.59E-01	0.005	0.017	7.66E-01
4.401675	-0.011	0.013	3.98E-01	0.005	0.019	7.73E-01
5.869867	0.007	0.008	3.34E-01	-0.010	0.011	3.58E-01
7.610233	-0.012	0.013	3.62E-01	-0.007	0.020	7.17E-01
7.786187	-0.006	0.006	3.49E-01	-0.001	0.009	8.76E-01
2.595704918	-0.016	0.019	3.88E-01	0.010	0.028	7.26E-01
7.204495	-0.007	0.007	3.45E-01	-0.003	0.011	7.70E-01
5.992665	0.006	0.008	4.80E-01	-0.014	0.012	2.41E-01
4.391246	-0.012	0.013	3.38E-01	0.004	0.019	8.30E-01
0.631275018	0.010	0.010	2.97E-01	0.007	0.015	6.45E-01
8.550562	0.007	0.008	4.15E-01	0.006	0.012	6.44E-01
3.136689	-0.010	0.010	3.14E-01	-0.018	0.015	2.17E-01
1.52214852	0.009	0.009	3.25E-01	-0.021	0.014	1.37E-01
2.293588451	-0.003	0.005	4.66E-01	-0.012	0.007	8.95E-02
8.192598	0.008	0.009	3.66E-01	0.003	0.014	8.44E-01
5.533771	0.007	0.008	3.63E-01	-0.002	0.012	8.88E-01
5.019365	0.008	0.011	4.67E-01	-0.009	0.017	5.93E-01
6.391001	0.008	0.009	3.58E-01	0.008	0.013	5.59E-01
1.036339858	0.005	0.005	3.23E-01	0.007	0.007	3.20E-01
6.039765	0.009	0.010	3.88E-01	0.006	0.015	6.99E-01
1.862954735	-0.006	0.008	4.25E-01	0.009	0.012	4.50E-01
8.537777	0.004	0.007	5.71E-01	0.012	0.011	2.87E-01
7.783159	-0.011	0.009	2.51E-01	0.008	0.014	5.55E-01
8.487985	0.006	0.006	3.35E-01	0.007	0.009	4.71E-01
2.84264644	-0.007	0.008	3.91E-01	-0.015	0.012	1.95E-01
5.117267	0.008	0.009	3.56E-01	-0.008	0.013	5.27E-01
2.291233423	-0.004	0.005	4.22E-01	-0.010	0.007	1.44E-01
6.36375	-0.006	0.007	4.34E-01	-0.005	0.011	6.63E-01
8.233979	0.006	0.009	4.80E-01	-0.015	0.013	2.36E-01
6.09023	0.009	0.010	3.59E-01	-0.003	0.015	8.11E-01
3.991227	-0.006	0.009	4.63E-01	0.003	0.013	7.90E-01
0.601332518	-0.008	0.010	4.29E-01	0.000	0.015	9.82E-01
6.999271	-0.006	0.008	4.27E-01	-0.005	0.012	6.42E-01
8.143815	0.003	0.008	7.38E-01	0.002	0.013	8.88E-01
0.648769513	-0.008	0.008	3.00E-01	-0.012	0.012	2.92E-01
7.678192	0.006	0.009	5.08E-01	-0.009	0.014	5.31E-01
3.519549	0.006	0.007	4.17E-01	0.027	0.010	9.70E-03
8.268295	0.001	0.007	8.68E-01	-0.007	0.011	5.24E-01
2.031507463	0.006	0.005	2.25E-01	0.007	0.007	3.56E-01
8.663267	0.010	0.009	3.01E-01	0.001	0.014	9.20E-01
1.982388304	0.006	0.006	2.84E-01	-0.005	0.009	6.05E-01
7.006	-0.006	0.007	3.94E-01	-0.010	0.011	3.57E-01
6.373506	0.008	0.008	3.02E-01	0.001	0.012	9.03E-01

4.441374	-0.012	0.016	4.31E-01	0.019	0.023	4.07E-01
1.518784194	0.008	0.009	3.75E-01	-0.012	0.013	3.67E-01
3.93437	-0.004	0.006	5.66E-01	0.012	0.009	1.84E-01
6.848213	-0.008	0.009	3.91E-01	-0.002	0.013	8.64E-01
7.778449	-0.009	0.011	4.06E-01	-0.005	0.016	7.68E-01
8.250127	-0.007	0.009	4.01E-01	-0.018	0.013	1.77E-01
0.752054319	-0.006	0.005	2.73E-01	-0.010	0.008	2.01E-01
5.657578	0.007	0.008	4.13E-01	-0.016	0.012	1.97E-01
5.682474	0.006	0.007	3.94E-01	0.004	0.011	6.94E-01
5.600721	0.008	0.007	2.99E-01	-0.002	0.011	8.58E-01
1.052488622	-0.005	0.006	4.57E-01	-0.022	0.009	2.15E-02
6.288389	0.006	0.007	4.41E-01	0.003	0.011	8.15E-01
2.266337411	0.006	0.007	3.92E-01	0.017	0.011	1.27E-01
1.684645461	-0.004	0.004	3.09E-01	0.004	0.006	4.56E-01
7.974589	0.006	0.007	4.02E-01	-0.011	0.011	3.34E-01
8.427764	0.009	0.009	2.98E-01	0.005	0.013	6.74E-01
8.444249	0.012	0.008	1.37E-01	0.009	0.012	4.69E-01
7.15874	0.006	0.007	3.85E-01	0.003	0.010	7.77E-01
2.264318816	0.007	0.007	3.39E-01	0.017	0.010	9.69E-02
4.439356	-0.014	0.017	3.92E-01	0.018	0.025	4.75E-01
5.891062	0.007	0.009	4.13E-01	0.009	0.013	4.71E-01
0.977464155	0.004	0.007	5.15E-01	0.009	0.010	3.69E-01
7.016766	0.003	0.011	7.47E-01	0.008	0.016	6.28E-01
7.014747	0.005	0.008	5.46E-01	0.001	0.011	9.57E-01
5.540836	0.009	0.009	3.15E-01	0.008	0.014	5.72E-01
6.553498	0.012	0.010	1.92E-01	0.002	0.014	8.67E-01
7.254623	-0.012	0.011	2.76E-01	-0.003	0.016	8.42E-01
5.451681	0.007	0.008	3.98E-01	-0.013	0.012	2.93E-01
7.62369	-0.015	0.013	2.35E-01	-0.005	0.019	7.89E-01
0.588884512	-0.003	0.010	7.93E-01	0.018	0.015	2.47E-01
1.625769758	0.007	0.006	2.62E-01	-0.002	0.009	8.53E-01
4.003002	0.007	0.009	4.34E-01	0.013	0.013	3.29E-01
7.304752	-0.009	0.012	4.46E-01	-0.044	0.017	9.93E-03
1.509027649	0.006	0.007	3.53E-01	-0.010	0.010	3.29E-01
4.380816	-0.011	0.012	3.80E-01	0.014	0.018	4.42E-01
4.299063	-0.011	0.014	4.16E-01	0.011	0.020	5.74E-01
6.86638	-0.007	0.009	4.60E-01	0.006	0.014	6.56E-01
2.718839246	-0.008	0.007	2.09E-01	-0.016	0.010	1.15E-01
6.822644	-0.014	0.010	1.86E-01	-0.002	0.016	8.96E-01
8.724834	0.011	0.009	2.37E-01	-0.007	0.013	6.06E-01
5.519641	-0.008	0.010	4.03E-01	-0.027	0.015	7.60E-02
5.778694	0.008	0.009	3.49E-01	-0.001	0.013	9.09E-01
7.978626	0.007	0.009	3.96E-01	0.008	0.013	5.41E-01

7.802336	-0.007	0.008	4.30E-01	-0.008	0.012	4.96E-01
8.477892	0.013	0.010	2.16E-01	0.006	0.016	7.09E-01
2.666692195	-0.006	0.005	2.38E-01	0.011	0.008	1.64E-01
7.722265	-0.010	0.010	3.16E-01	-0.033	0.015	3.00E-02
7.701406	-0.009	0.010	3.83E-01	0.006	0.015	6.68E-01
7.018448	0.002	0.009	7.89E-01	-0.006	0.013	6.45E-01
8.84494	0.009	0.008	2.84E-01	0.003	0.012	8.01E-01
0.643050159	0.007	0.009	4.43E-01	-0.002	0.014	8.69E-01
8.753767	0.007	0.009	4.48E-01	0.007	0.013	5.82E-01
7.246549	-0.010	0.009	2.97E-01	-0.031	0.014	2.64E-02
1.660758747	0.003	0.004	4.16E-01	0.003	0.006	6.57E-01
5.031477	0.007	0.010	4.53E-01	-0.006	0.014	6.72E-01
2.609835087	-0.012	0.013	3.53E-01	0.004	0.019	8.32E-01
1.142316124	0.009	0.007	1.97E-01	-0.004	0.010	6.69E-01
6.318332	0.006	0.010	5.44E-01	-0.018	0.015	2.34E-01
6.043803	0.007	0.007	3.09E-01	0.010	0.011	3.49E-01
8.825764	0.007	0.008	3.39E-01	0.010	0.012	4.06E-01
8.913909	0.006	0.008	4.97E-01	0.004	0.013	7.58E-01
7.989392	0.007	0.009	4.29E-01	-0.022	0.013	1.07E-01
5.450335	0.009	0.009	3.21E-01	-0.011	0.014	4.37E-01
2.50116736	-0.011	0.013	4.05E-01	-0.008	0.019	6.88E-01
7.256978	-0.014	0.011	1.96E-01	-0.016	0.016	3.17E-01
0.950213115	0.005	0.005	3.00E-01	0.000	0.007	9.55E-01
1.074356741	0.005	0.007	4.85E-01	0.000	0.010	9.75E-01
1.103626376	0.009	0.008	2.46E-01	-0.004	0.011	7.40E-01
7.986364	0.010	0.010	3.10E-01	0.005	0.015	7.45E-01
7.24453	-0.010	0.010	2.83E-01	0.001	0.014	9.50E-01
7.21156	-0.012	0.010	2.47E-01	-0.015	0.015	3.25E-01
2.183574994	0.011	0.011	2.89E-01	-0.019	0.016	2.44E-01
5.91764	0.005	0.008	5.07E-01	-0.007	0.012	5.35E-01
0.565670663	0.008	0.007	2.59E-01	0.016	0.011	1.34E-01
5.920332	-0.010	0.010	3.19E-01	-0.009	0.015	5.24E-01
4.087111	0.013	0.010	1.88E-01	0.010	0.015	5.17E-01
0.80420137	0.003	0.006	6.56E-01	0.010	0.008	2.44E-01
7.329984	-0.007	0.006	2.80E-01	-0.006	0.009	5.07E-01
3.203302	0.006	0.007	3.97E-01	0.019	0.010	7.13E-02
3.273953	-0.010	0.011	3.43E-01	0.024	0.016	1.36E-01
4.397974	-0.011	0.014	4.22E-01	0.018	0.020	3.77E-01
5.981563	-0.011	0.009	2.14E-01	0.002	0.013	8.95E-01
6.620448	0.005	0.007	4.92E-01	-0.013	0.011	2.58E-01
8.971103	0.007	0.008	3.78E-01	-0.004	0.012	7.66E-01
6.054905	0.008	0.009	3.68E-01	-0.013	0.013	3.44E-01
5.118949	0.008	0.009	3.61E-01	-0.006	0.014	6.63E-01

8.817016	0.008	0.008	3.09E-01	-0.009	0.012	4.56E-01
6.32506	-0.008	0.010	4.40E-01	-0.024	0.015	1.00E-01
4.131183	-0.011	0.014	4.36E-01	0.026	0.021	2.05E-01
8.515909	0.009	0.009	3.00E-01	-0.006	0.013	6.39E-01
2.16103401	-0.009	0.009	3.26E-01	-0.010	0.014	4.52E-01
8.850996	-0.007	0.009	4.50E-01	-0.025	0.013	5.60E-02
2.659963543	-0.009	0.010	3.62E-01	0.002	0.015	9.20E-01
7.678865	0.006	0.009	4.55E-01	0.010	0.013	4.42E-01
2.916998	-0.007	0.008	3.86E-01	-0.003	0.012	8.08E-01
2.268356007	0.006	0.007	3.64E-01	0.006	0.010	5.09E-01
1.479085148	-0.005	0.007	4.77E-01	0.001	0.010	8.85E-01
2.180883533	0.012	0.011	2.74E-01	-0.011	0.016	4.91E-01
6.054568	0.008	0.009	3.69E-01	-0.004	0.013	7.80E-01
8.415989	0.008	0.009	3.31E-01	-0.008	0.013	5.38E-01
3.115493	-0.012	0.010	2.51E-01	0.007	0.015	6.32E-01
0.768539516	0.005	0.005	3.74E-01	-0.003	0.008	6.92E-01
7.395252	0.007	0.007	3.43E-01	-0.005	0.011	6.60E-01
2.549613653	-0.012	0.012	3.35E-01	-0.017	0.018	3.62E-01
1.767407879	-0.007	0.009	4.38E-01	0.017	0.013	2.15E-01
8.354422	0.010	0.010	2.74E-01	-0.012	0.014	4.07E-01
1.223059946	0.006	0.008	4.09E-01	-0.001	0.011	9.05E-01
6.63256	0.007	0.008	3.82E-01	0.009	0.012	4.81E-01
2.152959628	-0.008	0.008	3.09E-01	-0.005	0.012	6.79E-01
8.323806	-0.005	0.007	4.22E-01	-0.013	0.010	1.89E-01
6.98615	-0.008	0.009	3.52E-01	-0.022	0.013	9.54E-02
7.475323	-0.005	0.008	5.56E-01	-0.022	0.012	5.59E-02
4.341117	-0.012	0.016	4.26E-01	0.015	0.023	5.10E-01
8.81197	0.008	0.009	4.06E-01	0.006	0.014	6.46E-01
5.730247	0.008	0.010	4.29E-01	0.013	0.015	3.96E-01
0.667946171	-0.007	0.010	4.57E-01	-0.027	0.014	5.87E-02
5.714771	0.005	0.008	5.15E-01	-0.011	0.012	3.58E-01
4.428253	-0.011	0.014	4.32E-01	0.006	0.021	7.63E-01
6.765114	-0.006	0.010	5.31E-01	-0.004	0.015	7.99E-01
5.605767	-0.011	0.010	2.71E-01	-0.010	0.015	4.85E-01
1.42088231	0.006	0.005	1.92E-01	0.002	0.007	7.99E-01
4.176602	-0.011	0.012	3.39E-01	0.005	0.018	7.86E-01
4.141949	-0.014	0.016	3.91E-01	0.013	0.024	6.00E-01
7.264716	-0.009	0.011	3.74E-01	-0.008	0.016	6.11E-01
8.79784	0.007	0.010	4.94E-01	-0.011	0.014	4.41E-01
8.360477	0.007	0.009	4.59E-01	-0.004	0.014	7.87E-01
4.354575	-0.012	0.014	4.14E-01	0.014	0.021	4.91E-01
8.875219	0.011	0.009	2.59E-01	-0.013	0.014	3.45E-01
7.210887	-0.010	0.011	3.49E-01	-0.028	0.016	8.63E-02

8.800531	0.008	0.008	3.14E-01	0.003	0.011	7.81E-01
1.718625153	-0.006	0.005	2.54E-01	-0.008	0.008	3.37E-01
4.142286	-0.013	0.015	3.89E-01	0.009	0.023	7.04E-01
7.478688	0.007	0.009	4.36E-01	0.011	0.013	4.17E-01
3.69012	-0.004	0.004	3.85E-01	0.006	0.007	3.53E-01
8.312031	0.008	0.008	3.53E-01	0.011	0.012	3.81E-01
5.733612	0.005	0.010	5.96E-01	-0.001	0.014	9.17E-01
5.245112	-0.007	0.005	1.14E-01	-0.004	0.007	5.29E-01
0.954250306	0.005	0.005	2.80E-01	0.005	0.007	4.68E-01
8.224559	-0.010	0.008	2.17E-01	-0.018	0.012	1.59E-01
0.984192806	0.008	0.006	1.98E-01	0.012	0.009	1.88E-01
6.445167	-0.004	0.007	5.23E-01	-0.020	0.010	5.88E-02
1.08848691	0.007	0.007	3.17E-01	0.007	0.011	5.44E-01
5.66868	0.009	0.011	4.13E-01	0.014	0.016	3.85E-01
4.343472	-0.011	0.014	4.03E-01	0.020	0.020	3.25E-01
7.60485	-0.005	0.010	6.01E-01	-0.013	0.014	3.75E-01
2.361547835	0.012	0.007	1.03E-01	0.015	0.011	1.59E-01
6.293436	0.010	0.010	2.96E-01	0.021	0.014	1.48E-01
7.89721	-0.007	0.009	4.61E-01	-0.008	0.014	5.80E-01
2.328241008	-0.003	0.005	5.60E-01	-0.008	0.008	2.86E-01
2.675439442	-0.009	0.008	2.29E-01	0.003	0.011	7.69E-01
1.802396868	-0.006	0.011	5.56E-01	0.007	0.016	6.68E-01
3.943454	-0.007	0.008	3.86E-01	0.000	0.013	9.72E-01
7.35017	-0.010	0.010	3.02E-01	0.000	0.015	9.92E-01
5.034505	0.012	0.013	3.85E-01	0.015	0.020	4.58E-01
5.555639	0.008	0.010	4.01E-01	-0.011	0.015	4.40E-01
6.821298	0.008	0.010	4.43E-01	0.012	0.015	4.29E-01
5.317781	0.016	0.016	3.41E-01	-0.030	0.025	2.18E-01
2.58292048	-0.004	0.012	7.32E-01	-0.010	0.018	5.88E-01
8.756459	0.008	0.008	3.01E-01	-0.006	0.011	5.72E-01
5.61216	-0.005	0.007	4.09E-01	-0.021	0.010	3.58E-02
7.090108	-0.009	0.009	3.09E-01	0.015	0.014	2.76E-01
5.322828	0.017	0.017	3.37E-01	-0.030	0.026	2.55E-01
5.423421	0.005	0.006	4.07E-01	-0.003	0.009	7.23E-01
4.350537	-0.014	0.014	3.27E-01	0.021	0.021	3.20E-01
6.290408	0.008	0.010	4.22E-01	0.013	0.014	3.58E-01
0.834143871	-0.010	0.008	2.45E-01	-0.005	0.013	6.70E-01
1.064263763	0.005	0.006	4.01E-01	-0.005	0.010	6.15E-01
8.492359	0.006	0.009	5.37E-01	-0.028	0.014	4.16E-02
0.773922437	0.006	0.005	2.41E-01	-0.002	0.007	8.05E-01
6.789337	0.013	0.011	2.25E-01	0.025	0.016	1.21E-01
6.415897	0.006	0.010	5.49E-01	0.004	0.014	8.04E-01
6.126229	0.009	0.009	3.08E-01	0.009	0.013	4.70E-01

7.174552	-0.005	0.010	6.12E-01	-0.024	0.014	8.91E-02
6.572675	0.007	0.008	3.29E-01	0.000	0.011	9.98E-01
4.354911	-0.010	0.013	4.20E-01	0.009	0.019	6.44E-01
8.985233	-0.006	0.008	4.34E-01	-0.011	0.011	3.42E-01
8.762178	-0.008	0.009	3.80E-01	0.005	0.014	7.35E-01
5.509884	0.006	0.008	4.73E-01	-0.002	0.013	8.93E-01
7.202476	-0.005	0.011	6.19E-01	-0.009	0.016	5.70E-01
8.522301	0.004	0.007	5.88E-01	-0.001	0.010	9.36E-01
1.522821385	0.009	0.009	3.28E-01	-0.020	0.014	1.57E-01
5.035514	0.008	0.010	4.21E-01	-0.007	0.015	6.44E-01
4.044384	0.016	0.013	2.09E-01	-0.001	0.019	9.41E-01
0.955932469	-0.008	0.007	2.21E-01	0.012	0.010	2.35E-01
3.692475	-0.003	0.005	5.03E-01	0.009	0.007	2.30E-01
7.855156	0.007	0.007	3.26E-01	0.008	0.010	4.52E-01
3.216759	-0.013	0.014	3.44E-01	-0.006	0.021	7.67E-01
6.29108	-0.011	0.010	2.92E-01	0.006	0.015	7.03E-01
1.911401028	-0.016	0.015	2.79E-01	-0.056	0.022	1.23E-02
7.555731	0.010	0.008	2.22E-01	0.009	0.013	4.76E-01
0.986884267	0.005	0.005	3.27E-01	0.012	0.008	1.26E-01
5.532089	0.008	0.009	3.28E-01	0.001	0.013	9.61E-01
6.807505	0.005	0.009	5.87E-01	0.015	0.013	2.49E-01
7.092126	-0.006	0.008	4.11E-01	0.003	0.011	7.99E-01
1.507345486	0.005	0.006	3.89E-01	-0.008	0.009	4.19E-01
3.690457	-0.004	0.004	3.92E-01	0.007	0.007	3.05E-01
8.959327	0.005	0.006	3.97E-01	0.000	0.009	9.59E-01
6.297136	-0.007	0.007	3.42E-01	-0.004	0.011	7.09E-01
4.109652	-0.006	0.009	5.05E-01	0.005	0.014	7.04E-01
1.805424761	-0.008	0.011	4.45E-01	0.013	0.016	4.16E-01
7.160759	0.009	0.009	3.55E-01	0.009	0.014	5.10E-01
2.578210423	-0.007	0.013	5.76E-01	0.011	0.019	5.53E-01
8.951926	-0.005	0.007	4.19E-01	-0.024	0.010	1.82E-02
8.336254	0.007	0.009	4.30E-01	0.020	0.013	1.26E-01
2.406629802	-0.017	0.018	3.40E-01	-0.003	0.027	9.05E-01
1.236180817	0.006	0.008	4.01E-01	-0.006	0.011	5.68E-01
7.147638	0.005	0.007	5.07E-01	0.000	0.011	9.85E-01
3.126596	-0.010	0.009	2.78E-01	0.012	0.014	4.08E-01
5.560349	0.006	0.007	4.18E-01	0.002	0.011	8.51E-01
8.396812	0.009	0.008	2.49E-01	-0.006	0.012	6.37E-01
7.250923	-0.011	0.009	2.13E-01	-0.002	0.013	9.01E-01
8.460061	0.005	0.006	4.56E-01	-0.007	0.009	4.68E-01
6.226485	0.007	0.007	3.04E-01	0.002	0.010	8.57E-01
4.195105	-0.007	0.009	4.52E-01	0.017	0.014	2.21E-01
7.430578	0.008	0.010	4.41E-01	-0.013	0.015	3.63E-01

5.638738	0.008	0.008	3.42E-01	0.000	0.012	9.70E-01
6.944096	0.007	0.008	4.22E-01	0.025	0.012	4.92E-02
5.867848	0.008	0.008	3.07E-01	-0.003	0.012	7.84E-01
3.930333	0.008	0.008	2.82E-01	0.024	0.011	3.82E-02
3.647057	0.008	0.005	1.57E-01	0.014	0.008	7.92E-02
1.684309029	-0.003	0.004	3.96E-01	0.003	0.006	6.18E-01
6.40143	0.008	0.007	2.65E-01	0.014	0.010	1.61E-01
1.635862736	0.007	0.007	2.78E-01	-0.003	0.010	7.38E-01
6.690762	-0.006	0.010	5.71E-01	-0.010	0.015	4.92E-01
5.318117	0.015	0.016	3.48E-01	-0.031	0.025	2.03E-01
8.077201	0.008	0.010	3.99E-01	0.023	0.014	1.10E-01
3.977434	-0.007	0.009	4.53E-01	-0.006	0.014	6.89E-01
8.588915	0.005	0.006	4.51E-01	0.002	0.009	8.59E-01
6.791019	-0.008	0.010	3.99E-01	0.005	0.015	7.27E-01
3.316344	-0.012	0.013	3.41E-01	0.004	0.019	8.29E-01
2.505540984	-0.014	0.014	3.14E-01	-0.024	0.020	2.44E-01
7.509303	-0.006	0.009	4.93E-01	0.000	0.014	9.81E-01
3.240646	0.004	0.006	5.60E-01	0.017	0.009	6.24E-02
4.397638	-0.011	0.013	4.08E-01	0.018	0.020	3.53E-01
5.715444	0.008	0.009	3.63E-01	-0.002	0.014	9.11E-01
2.572491069	-0.007	0.011	5.07E-01	0.000	0.016	9.97E-01
8.802886	-0.005	0.007	5.16E-01	-0.005	0.011	6.31E-01
6.079128	0.007	0.008	3.77E-01	0.009	0.013	4.96E-01
8.345338	0.007	0.009	4.39E-01	0.002	0.013	8.78E-01
7.520742	0.007	0.006	3.01E-01	-0.006	0.009	5.10E-01
7.792916	-0.009	0.009	3.25E-01	0.007	0.013	5.86E-01
8.838885	0.008	0.010	4.19E-01	-0.020	0.014	1.55E-01
8.168711	0.006	0.008	4.80E-01	-0.013	0.012	2.99E-01
2.583256912	-0.005	0.011	6.72E-01	-0.004	0.017	7.95E-01
6.389319	-0.006	0.008	4.26E-01	0.003	0.012	8.23E-01
2.795545877	-0.012	0.012	3.11E-01	-0.054	0.018	1.93E-03
7.925806	0.009	0.009	3.18E-01	-0.013	0.014	3.39E-01
8.105798	0.007	0.008	3.63E-01	-0.006	0.012	6.03E-01
2.986303	-0.004	0.004	3.74E-01	0.004	0.006	4.79E-01
6.619439	0.005	0.008	5.36E-01	-0.009	0.012	4.70E-01
3.664888	-0.004	0.005	4.21E-01	0.013	0.008	8.69E-02
7.390542	0.006	0.006	3.11E-01	-0.012	0.009	1.90E-01
3.703577	0.006	0.005	3.01E-01	0.021	0.008	7.17E-03
7.764992	-0.009	0.011	4.18E-01	-0.019	0.017	2.45E-01
8.468809	-0.006	0.010	5.65E-01	-0.022	0.015	1.25E-01
8.723152	0.005	0.011	6.68E-01	-0.027	0.016	8.17E-02
3.701895	0.005	0.005	3.75E-01	0.019	0.008	1.80E-02
6.633905	-0.005	0.009	5.72E-01	-0.017	0.014	2.04E-01

1.418863714	0.006	0.005	2.15E-01	0.003	0.007	7.24E-01
7.982664	0.009	0.010	3.78E-01	0.010	0.015	4.90E-01
6.361731	0.007	0.007	3.45E-01	0.008	0.011	4.93E-01
1.873720577	-0.007	0.008	3.38E-01	0.008	0.011	4.52E-01
6.101669	-0.004	0.007	5.33E-01	-0.020	0.011	6.34E-02
4.196788	-0.009	0.012	4.43E-01	0.016	0.017	3.41E-01
1.413480793	0.006	0.004	2.04E-01	0.012	0.007	7.59E-02
4.195442	-0.007	0.009	4.50E-01	0.018	0.014	1.82E-01
5.062429	0.006	0.007	3.67E-01	-0.003	0.010	7.45E-01
2.142530218	-0.010	0.012	4.22E-01	0.025	0.019	1.84E-01
4.375433	-0.013	0.016	4.12E-01	0.015	0.023	5.27E-01
8.958655	-0.008	0.008	3.29E-01	-0.024	0.012	3.79E-02
3.577079	0.011	0.008	1.55E-01	0.020	0.012	8.65E-02
8.251137	0.008	0.009	3.51E-01	0.014	0.014	3.07E-01
6.212692	0.009	0.009	2.99E-01	-0.001	0.013	9.09E-01
3.174705	-0.006	0.006	3.13E-01	0.001	0.008	9.16E-01
7.535208	-0.008	0.008	3.27E-01	-0.012	0.013	3.55E-01
7.411737	-0.010	0.008	2.40E-01	-0.008	0.013	5.16E-01
7.647913	-0.006	0.011	5.72E-01	-0.011	0.016	5.00E-01
4.313193	-0.011	0.012	3.64E-01	0.014	0.018	4.41E-01
4.3899	-0.013	0.014	3.74E-01	0.012	0.021	5.68E-01
5.711407	0.009	0.009	3.51E-01	-0.022	0.014	1.15E-01
7.017775	0.003	0.009	7.10E-01	0.013	0.014	3.34E-01
1.11708368	0.008	0.007	2.34E-01	-0.013	0.010	2.08E-01
4.11638	-0.007	0.010	4.74E-01	0.017	0.014	2.25E-01
7.271445	-0.011	0.011	3.00E-01	0.000	0.016	9.94E-01
7.52848	-0.009	0.009	3.22E-01	-0.008	0.014	5.59E-01
3.154183	-0.009	0.009	3.27E-01	0.002	0.014	8.82E-01
5.504165	-0.013	0.009	1.84E-01	-0.037	0.014	9.23E-03
8.312368	0.009	0.009	3.04E-01	0.001	0.013	9.20E-01
2.098793981	-0.004	0.009	6.36E-01	-0.006	0.013	6.35E-01
8.592616	0.009	0.007	2.15E-01	0.012	0.011	2.74E-01
2.263645951	0.006	0.007	3.54E-01	0.017	0.010	8.51E-02
6.942078	-0.007	0.008	4.14E-01	0.006	0.012	6.48E-01
7.368674	0.007	0.009	4.42E-01	0.005	0.014	7.29E-01
6.081819	0.008	0.008	3.08E-01	0.007	0.012	5.42E-01
8.550898	0.009	0.010	3.73E-01	0.002	0.015	8.98E-01
3.312306	-0.013	0.012	2.90E-01	0.011	0.018	5.57E-01
0.817658674	0.003	0.005	5.88E-01	0.014	0.008	7.48E-02
6.850231	0.007	0.007	3.44E-01	-0.004	0.011	7.16E-01
5.023402	0.007	0.008	3.80E-01	-0.009	0.012	4.69E-01
7.411401	-0.010	0.008	2.22E-01	-0.008	0.012	4.73E-01
1.811144115	-0.007	0.010	4.69E-01	-0.004	0.015	7.72E-01

4.324296	-0.013	0.015	3.93E-01	0.021	0.023	3.62E-01
0.778632493	0.004	0.005	4.11E-01	-0.005	0.007	4.30E-01
6.448195	-0.007	0.009	4.58E-01	-0.020	0.013	1.27E-01
7.82555	-0.012	0.009	1.91E-01	-0.001	0.013	9.44E-01
3.759089	0.007	0.009	4.49E-01	0.020	0.013	1.24E-01
5.125678	0.007	0.007	3.43E-01	-0.007	0.011	4.97E-01
7.975935	0.005	0.008	5.48E-01	0.015	0.011	1.87E-01
8.167029	0.007	0.007	2.85E-01	0.003	0.010	7.47E-01
5.009945	0.008	0.010	4.07E-01	-0.003	0.015	8.44E-01
8.392102	0.005	0.009	5.50E-01	0.010	0.013	4.43E-01
2.984285	-0.004	0.004	3.47E-01	-0.001	0.006	9.30E-01
4.363322	-0.011	0.012	3.72E-01	0.017	0.018	3.43E-01
1.854880352	-0.009	0.009	3.24E-01	-0.005	0.014	7.23E-01
7.872314	0.007	0.008	3.98E-01	-0.012	0.012	3.20E-01
1.522484952	0.009	0.009	3.30E-01	-0.019	0.014	1.73E-01
8.716087	0.008	0.009	4.15E-01	-0.012	0.014	3.69E-01
8.352739	-0.009	0.009	3.24E-01	0.005	0.014	7.24E-01
4.235814	0.011	0.013	4.06E-01	0.018	0.020	3.69E-01
8.251473	0.007	0.008	4.16E-01	0.018	0.012	1.39E-01
2.186602887	0.011	0.011	3.05E-01	-0.022	0.016	1.82E-01
4.410422	-0.011	0.013	4.04E-01	0.011	0.019	5.84E-01
2.919017	-0.008	0.008	3.29E-01	-0.006	0.012	6.32E-01
8.909199	0.008	0.007	2.45E-01	-0.003	0.011	7.75E-01
7.375066	-0.009	0.008	2.92E-01	-0.005	0.013	7.02E-01
6.681679	0.006	0.010	5.64E-01	0.008	0.015	5.98E-01
4.305119	-0.013	0.014	3.76E-01	0.015	0.021	4.68E-01
7.378431	-0.008	0.009	3.70E-01	-0.005	0.013	6.90E-01
8.281416	-0.007	0.009	4.56E-01	-0.021	0.014	1.42E-01
3.16764	-0.006	0.006	3.54E-01	0.007	0.009	4.81E-01
1.170576462	0.007	0.005	1.78E-01	-0.013	0.008	8.72E-02
6.943087	0.008	0.008	3.71E-01	0.010	0.013	4.17E-01
6.187123	-0.006	0.008	4.33E-01	-0.006	0.012	6.40E-01
8.174094	-0.007	0.009	4.03E-01	0.004	0.013	7.61E-01
6.955871	-0.006	0.006	3.22E-01	-0.007	0.010	4.87E-01
3.548146	0.012	0.011	2.47E-01	0.020	0.016	2.09E-01
0.744652802	-0.007	0.006	2.52E-01	-0.008	0.009	3.72E-01
6.376871	-0.005	0.009	5.54E-01	0.009	0.013	4.69E-01
7.878033	0.004	0.008	5.99E-01	-0.001	0.012	9.46E-01
7.443362	-0.008	0.009	3.97E-01	0.010	0.014	4.64E-01
4.181648	-0.011	0.014	4.14E-01	0.009	0.021	6.57E-01
1.854207487	-0.004	0.009	6.24E-01	-0.014	0.013	2.82E-01
1.736792513	-0.005	0.005	3.02E-01	0.009	0.008	2.53E-01
2.170454123	0.010	0.010	3.19E-01	-0.009	0.015	5.33E-01

5.033832	0.007	0.010	5.04E-01	0.002	0.015	8.74E-01
7.372375	0.006	0.010	5.35E-01	0.009	0.014	5.49E-01
2.368276486	-0.005	0.008	5.25E-01	0.023	0.012	5.06E-02
8.853351	-0.010	0.009	2.80E-01	0.006	0.013	6.28E-01
7.261689	-0.011	0.010	2.64E-01	-0.005	0.015	7.52E-01
5.063774	0.006	0.007	3.81E-01	-0.012	0.011	2.56E-01
3.647393	0.007	0.005	1.93E-01	0.015	0.008	4.36E-02
6.48823	-0.007	0.008	3.98E-01	0.007	0.011	5.49E-01
2.619255199	-0.014	0.013	3.05E-01	0.028	0.020	1.57E-01
8.298574	0.006	0.008	4.24E-01	-0.013	0.011	2.42E-01
2.515633961	-0.013	0.015	3.79E-01	-0.016	0.022	4.79E-01
6.216056	0.008	0.008	3.31E-01	-0.026	0.013	4.02E-02
0.657180328	0.008	0.011	4.99E-01	0.000	0.017	9.82E-01
5.584908	0.009	0.009	3.30E-01	-0.006	0.013	6.27E-01
5.99502	0.001	0.009	8.72E-01	-0.005	0.013	6.83E-01
7.695687	-0.008	0.010	4.13E-01	-0.020	0.015	1.79E-01
1.982051872	0.006	0.006	3.10E-01	-0.005	0.009	6.03E-01
3.51753	0.007	0.007	3.33E-01	0.023	0.011	3.20E-02
6.966974	-0.012	0.010	2.45E-01	-0.006	0.015	7.08E-01
0.557259848	0.010	0.008	2.43E-01	0.016	0.012	1.94E-01
7.624026	-0.013	0.013	3.10E-01	-0.012	0.020	5.39E-01
1.4403954	0.004	0.004	3.04E-01	0.007	0.006	2.12E-01
5.890053	0.007	0.009	4.39E-01	-0.005	0.013	7.19E-01
1.969603866	0.004	0.004	3.71E-01	-0.001	0.007	8.20E-01
7.489117	0.010	0.009	2.57E-01	-0.004	0.014	7.70E-01
5.073867	0.009	0.009	3.41E-01	-0.009	0.014	5.00E-01
8.66293	-0.007	0.008	4.26E-01	-0.002	0.013	8.82E-01
1.657394421	0.004	0.005	3.34E-01	0.008	0.007	2.18E-01
6.520191	-0.007	0.007	3.35E-01	0.004	0.011	7.39E-01
3.247711	0.006	0.007	4.27E-01	0.022	0.010	3.74E-02
8.117573	-0.007	0.009	4.26E-01	0.013	0.014	3.62E-01
8.816344	-0.008	0.009	3.71E-01	0.005	0.014	6.96E-01
8.219176	-0.006	0.007	4.21E-01	-0.008	0.010	4.13E-01
6.853932	-0.007	0.008	3.60E-01	-0.009	0.012	4.51E-01
6.428345	0.007	0.006	2.77E-01	-0.006	0.009	4.88E-01
8.511872	-0.009	0.008	2.86E-01	-0.023	0.012	5.37E-02
0.623200636	-0.006	0.007	4.41E-01	0.002	0.011	8.31E-01
5.47725	0.007	0.007	3.40E-01	-0.005	0.011	6.33E-01
7.249913	-0.007	0.006	3.03E-01	-0.001	0.010	9.47E-01
6.817934	0.002	0.011	8.37E-01	0.000	0.016	9.75E-01
2.326558845	-0.003	0.006	5.49E-01	-0.015	0.009	8.20E-02
6.768142	-0.006	0.007	3.79E-01	-0.001	0.011	8.90E-01
8.095705	0.009	0.009	2.95E-01	-0.005	0.013	7.11E-01

8.159964	0.006	0.007	3.65E-01	-0.008	0.010	4.20E-01
6.580413	0.005	0.007	5.05E-01	-0.009	0.010	3.76E-01
6.503033	-0.008	0.010	4.29E-01	0.008	0.016	5.86E-01
3.724773	0.003	0.008	6.72E-01	0.002	0.012	8.66E-01
0.774931735	0.005	0.005	3.02E-01	0.002	0.007	7.68E-01
8.574448	0.008	0.008	3.37E-01	0.010	0.012	4.17E-01
8.391429	0.006	0.008	4.30E-01	0.007	0.012	5.45E-01
1.971958894	0.004	0.005	3.96E-01	-0.002	0.007	7.72E-01
2.326895278	-0.003	0.006	5.98E-01	-0.013	0.008	1.24E-01
6.155498	0.006	0.008	4.71E-01	-0.003	0.012	8.28E-01
7.20214	-0.007	0.011	5.07E-01	-0.001	0.016	9.32E-01
2.538511378	-0.013	0.013	3.26E-01	0.005	0.020	8.13E-01
8.870509	-0.007	0.009	4.71E-01	0.005	0.014	7.06E-01
1.916111084	-0.006	0.006	2.99E-01	0.002	0.009	8.56E-01
8.229605	0.007	0.008	4.14E-01	-0.015	0.012	2.23E-01
8.481257	0.007	0.009	4.20E-01	0.010	0.013	4.44E-01
4.146323	-0.009	0.012	4.56E-01	0.013	0.018	4.70E-01
3.861364	-0.005	0.005	3.09E-01	0.007	0.008	3.54E-01
7.870295	0.008	0.008	3.09E-01	-0.007	0.012	5.39E-01
7.308789	-0.010	0.010	3.20E-01	0.000	0.015	9.77E-01
5.528388	0.005	0.007	4.80E-01	-0.004	0.011	7.28E-01
5.567078	0.007	0.007	3.24E-01	0.000	0.011	9.83E-01
0.583165158	-0.006	0.008	4.24E-01	0.009	0.012	4.18E-01
7.15403	0.005	0.007	4.73E-01	-0.014	0.010	1.64E-01
2.662655004	-0.023	0.021	2.72E-01	-0.011	0.031	7.31E-01
7.06353	-0.008	0.011	4.54E-01	-0.032	0.016	4.56E-02
8.589588	0.008	0.008	3.15E-01	0.003	0.011	8.15E-01
3.519212	0.006	0.007	4.04E-01	0.025	0.010	1.46E-02
5.879287	0.006	0.009	4.59E-01	-0.006	0.013	6.34E-01
5.047289	0.005	0.008	5.60E-01	0.000	0.012	9.95E-01
5.782394	0.005	0.009	5.56E-01	-0.004	0.014	7.70E-01
6.018907	0.007	0.011	5.26E-01	-0.003	0.016	8.48E-01
5.100109	0.009	0.009	3.22E-01	-0.009	0.014	5.15E-01
6.456942	0.005	0.010	6.31E-01	-0.011	0.014	4.30E-01
6.869745	0.011	0.010	2.51E-01	-0.011	0.014	4.47E-01
1.075702471	0.008	0.007	2.45E-01	-0.005	0.011	6.13E-01
1.78187448	-0.007	0.008	3.63E-01	-0.008	0.012	4.87E-01
4.381153	-0.010	0.012	4.06E-01	0.009	0.018	6.29E-01
6.82298	0.005	0.010	6.16E-01	0.015	0.015	3.26E-01
7.803345	-0.008	0.010	4.11E-01	-0.015	0.015	3.06E-01
5.883661	0.007	0.007	3.14E-01	0.010	0.011	3.43E-01
5.741686	0.009	0.009	3.39E-01	0.006	0.014	6.88E-01
0.833807438	-0.010	0.008	2.54E-01	-0.005	0.012	7.04E-01

3.737894	0.004	0.009	6.83E-01	0.041	0.013	1.53E-03
6.701192	0.007	0.009	4.62E-01	-0.007	0.014	6.17E-01
5.086988	0.008	0.008	3.33E-01	-0.011	0.012	3.42E-01
8.793803	0.003	0.006	5.97E-01	-0.011	0.009	2.43E-01
3.195564	0.004	0.006	4.60E-01	0.015	0.008	7.12E-02
4.343136	-0.011	0.014	4.04E-01	0.026	0.020	1.92E-01
5.578516	0.007	0.008	3.51E-01	-0.021	0.012	7.59E-02
2.835244923	-0.006	0.010	5.74E-01	-0.022	0.015	1.45E-01
6.563591	0.010	0.009	3.03E-01	0.002	0.014	8.90E-01
5.285483	-0.009	0.008	2.84E-01	-0.026	0.012	3.33E-02
7.848763	0.004	0.009	6.08E-01	0.007	0.013	6.03E-01
8.879929	0.009	0.009	3.33E-01	0.017	0.014	2.12E-01
0.909168339	-0.004	0.006	4.23E-01	-0.024	0.008	4.24E-03
6.768815	-0.006	0.008	4.58E-01	0.009	0.012	4.54E-01
2.794536579	-0.012	0.012	3.18E-01	-0.057	0.018	1.34E-03
2.646842672	-0.012	0.013	3.48E-01	0.024	0.019	2.13E-01
6.784627	-0.007	0.010	4.48E-01	-0.012	0.014	4.11E-01
6.106043	-0.012	0.009	1.87E-01	-0.014	0.014	3.00E-01
0.530345241	0.009	0.010	3.27E-01	0.001	0.014	9.48E-01
6.040438	0.006	0.008	4.54E-01	0.005	0.012	6.51E-01
6.133294	0.006	0.009	5.11E-01	-0.004	0.014	7.53E-01
6.398066	-0.008	0.008	3.02E-01	-0.018	0.011	1.05E-01
6.496641	0.010	0.009	3.04E-01	-0.001	0.014	9.29E-01
6.820625	-0.013	0.010	2.16E-01	0.002	0.015	8.86E-01
1.830993638	-0.011	0.010	2.93E-01	-0.011	0.015	4.85E-01
2.615554441	-0.011	0.014	4.29E-01	0.028	0.020	1.74E-01
8.918283	-0.007	0.007	3.72E-01	-0.010	0.011	3.83E-01
1.676571079	0.005	0.004	2.61E-01	0.004	0.006	5.24E-01
0.988229998	0.006	0.007	3.78E-01	0.013	0.010	2.00E-01
2.138829459	-0.010	0.011	3.48E-01	0.023	0.016	1.64E-01
1.808452655	-0.011	0.012	3.66E-01	-0.014	0.017	4.25E-01
6.093931	0.007	0.011	5.22E-01	-0.011	0.016	4.95E-01
5.458746	-0.008	0.008	3.40E-01	-0.014	0.012	2.38E-01
2.580901884	0.016	0.012	1.61E-01	0.030	0.017	8.63E-02
6.75132	-0.007	0.009	4.82E-01	-0.016	0.014	2.51E-01
6.58815	0.006	0.009	4.80E-01	-0.012	0.014	3.66E-01
6.231195	0.007	0.010	4.66E-01	-0.017	0.015	2.58E-01
2.100476144	-0.004	0.008	6.16E-01	0.002	0.011	8.60E-01
4.190732	-0.009	0.014	5.20E-01	0.010	0.020	6.07E-01
2.617236604	-0.012	0.013	3.46E-01	0.018	0.019	3.44E-01
6.11008	-0.010	0.011	3.29E-01	-0.004	0.016	8.07E-01
0.534382432	0.010	0.010	2.88E-01	-0.001	0.014	9.18E-01
0.634302912	0.008	0.010	4.29E-01	0.004	0.015	7.92E-01

7.394916	0.007	0.007	3.56E-01	-0.011	0.011	3.43E-01
4.348182	-0.011	0.012	3.69E-01	0.007	0.018	7.15E-01
7.033251	-0.014	0.010	1.60E-01	-0.011	0.015	4.71E-01
1.810807683	-0.007	0.009	4.40E-01	0.001	0.014	9.58E-01
6.008477	0.010	0.010	3.28E-01	0.030	0.015	3.89E-02
2.304690727	-0.004	0.005	3.47E-01	-0.001	0.007	8.31E-01
1.223396379	0.006	0.008	4.29E-01	-0.002	0.011	8.79E-01
1.733428187	-0.006	0.005	2.47E-01	0.000	0.008	9.77E-01
1.169567164	0.007	0.005	1.54E-01	-0.005	0.007	4.98E-01
4.421861	-0.011	0.014	4.30E-01	0.019	0.020	3.45E-01
1.235844385	0.006	0.008	4.19E-01	-0.007	0.011	5.38E-01
6.630541	0.005	0.009	5.48E-01	0.010	0.013	4.35E-01
1.869683386	-0.008	0.009	3.48E-01	0.004	0.013	7.83E-01
5.298604	0.013	0.013	3.41E-01	-0.021	0.020	2.88E-01
8.751412	-0.006	0.007	4.13E-01	0.004	0.011	7.29E-01
3.127605	-0.012	0.010	1.94E-01	0.002	0.014	9.07E-01
3.747314	0.006	0.008	4.35E-01	0.024	0.012	5.81E-02
2.165744067	0.010	0.010	3.16E-01	-0.006	0.015	6.98E-01
8.705994	0.006	0.009	5.08E-01	0.005	0.013	6.92E-01
3.218105	-0.013	0.014	3.60E-01	-0.007	0.022	7.58E-01
8.688163	0.004	0.006	4.88E-01	0.017	0.009	6.88E-02
3.929997	0.008	0.008	3.12E-01	0.024	0.011	3.32E-02
7.58971	-0.007	0.010	4.82E-01	0.006	0.015	6.93E-01
0.54447541	-0.006	0.008	4.44E-01	0.012	0.012	3.05E-01
7.733031	-0.008	0.011	4.57E-01	0.005	0.016	7.62E-01
6.003094	0.007	0.009	4.42E-01	-0.001	0.013	9.37E-01
5.719482	0.007	0.010	4.95E-01	0.013	0.015	3.99E-01
0.670637632	-0.010	0.009	3.11E-01	-0.004	0.014	7.95E-01
8.980186	0.012	0.010	2.38E-01	-0.001	0.015	9.27E-01
4.233795	0.010	0.013	4.49E-01	0.017	0.020	3.89E-01
5.781722	0.007	0.009	4.65E-01	-0.005	0.014	7.02E-01
8.237007	0.006	0.010	5.27E-01	-0.018	0.015	2.06E-01
5.509211	0.004	0.007	6.15E-01	-0.015	0.011	1.77E-01
2.492756545	-0.018	0.017	2.85E-01	0.000	0.026	9.94E-01
8.072491	-0.009	0.009	3.16E-01	-0.003	0.013	8.00E-01
5.718136	0.005	0.010	6.39E-01	0.007	0.015	6.10E-01
1.981042574	0.005	0.006	3.24E-01	-0.005	0.008	5.38E-01
0.983856374	0.007	0.006	2.27E-01	0.013	0.009	1.53E-01
5.579189	0.008	0.010	4.28E-01	-0.023	0.014	1.12E-01
1.513737705	0.007	0.008	3.36E-01	-0.012	0.012	2.98E-01
8.849987	0.006	0.007	4.08E-01	0.002	0.010	8.77E-01
7.6718	0.006	0.009	4.57E-01	-0.005	0.013	7.10E-01
3.279672	-0.010	0.011	3.40E-01	0.007	0.016	6.77E-01

7.020803	0.004	0.010	6.44E-01	-0.006	0.014	6.76E-01
1.430975287	0.005	0.004	2.58E-01	0.005	0.007	4.39E-01
3.577415	0.011	0.008	1.66E-01	0.018	0.011	1.14E-01
0.6847678	0.012	0.013	3.65E-01	0.003	0.019	8.87E-01
1.49624321	-0.005	0.004	2.54E-01	-0.009	0.007	1.79E-01
5.783067	0.009	0.009	3.29E-01	-0.015	0.014	2.63E-01
6.053223	0.005	0.008	4.86E-01	0.012	0.012	3.17E-01
6.443821	0.012	0.010	2.30E-01	-0.008	0.015	5.76E-01
1.97397749	0.004	0.005	3.92E-01	-0.004	0.007	5.87E-01
1.915101786	-0.006	0.006	2.74E-01	-0.002	0.008	8.52E-01
3.135679	-0.011	0.011	3.22E-01	-0.013	0.017	4.35E-01
4.144304	-0.009	0.011	4.06E-01	0.015	0.017	3.73E-01
3.234254	-0.005	0.006	4.01E-01	0.009	0.009	3.29E-01
4.359957	-0.009	0.012	4.24E-01	0.007	0.018	6.80E-01
5.098091	0.007	0.008	3.74E-01	-0.010	0.012	4.27E-01
1.785575239	-0.008	0.010	4.58E-01	0.000	0.015	9.85E-01
5.442934	0.006	0.007	3.91E-01	-0.007	0.011	4.97E-01
5.599712	0.012	0.010	2.14E-01	-0.013	0.014	3.78E-01
6.07139	0.008	0.011	4.50E-01	-0.023	0.016	1.44E-01
6.544078	0.007	0.008	3.82E-01	-0.020	0.012	1.09E-01
1.416508686	0.006	0.004	1.93E-01	0.004	0.007	5.48E-01
0.987893565	0.005	0.006	3.93E-01	0.014	0.009	1.26E-01
6.848886	-0.007	0.009	4.27E-01	0.004	0.013	7.40E-01
7.264044	-0.009	0.011	4.04E-01	0.019	0.016	2.35E-01
3.231899	0.005	0.006	4.31E-01	0.022	0.009	1.28E-02
3.638982	0.007	0.005	1.79E-01	0.013	0.007	8.36E-02
7.172197	-0.008	0.009	3.94E-01	-0.017	0.014	2.10E-01
3.695503	0.005	0.005	3.57E-01	0.019	0.008	1.33E-02
6.907425	0.007	0.006	2.56E-01	0.009	0.009	3.32E-01
3.620815	-0.005	0.006	3.77E-01	-0.008	0.009	3.31E-01
0.703944458	0.013	0.010	2.30E-01	0.005	0.016	7.53E-01
4.410086	-0.010	0.012	3.99E-01	0.009	0.018	6.23E-01
3.754379	0.008	0.011	4.49E-01	0.014	0.016	3.99E-01
7.513004	0.010	0.010	3.38E-01	0.019	0.015	2.17E-01
2.129072914	-0.008	0.010	4.24E-01	-0.001	0.015	9.57E-01
5.153265	0.007	0.008	3.50E-01	-0.009	0.011	4.00E-01
5.741013	0.006	0.009	5.06E-01	0.009	0.014	5.36E-01
3.721745	0.006	0.007	4.24E-01	0.014	0.011	1.99E-01
4.425225	-0.012	0.015	4.14E-01	0.006	0.023	7.82E-01
5.805608	0.009	0.009	3.31E-01	0.000	0.014	9.75E-01
1.647301444	0.006	0.005	2.35E-01	0.011	0.008	1.46E-01
1.974313922	0.004	0.005	3.90E-01	-0.003	0.007	6.63E-01
4.374424	-0.012	0.015	4.31E-01	0.020	0.022	3.75E-01

0.858367017	0.009	0.009	3.30E-01	-0.004	0.013	7.35E-01
7.654978	0.008	0.008	3.59E-01	-0.019	0.013	1.31E-01
5.729911	0.007	0.010	4.75E-01	0.004	0.014	7.59E-01
2.031843895	0.006	0.005	2.43E-01	0.007	0.007	3.22E-01
7.147301	0.005	0.007	4.72E-01	0.002	0.011	8.83E-01
6.680669	0.006	0.009	4.90E-01	-0.004	0.014	7.63E-01
1.025574015	0.005	0.006	3.49E-01	0.010	0.009	2.64E-01
8.671005	-0.006	0.009	5.14E-01	-0.011	0.013	3.68E-01
7.525788	0.010	0.009	2.82E-01	-0.045	0.014	1.20E-03
7.266062	-0.011	0.011	3.12E-01	-0.024	0.016	1.37E-01
4.208563	-0.009	0.013	5.08E-01	0.012	0.020	5.51E-01
6.408159	-0.006	0.009	5.19E-01	-0.014	0.013	2.84E-01
1.747558356	-0.006	0.007	4.41E-01	0.001	0.011	9.36E-01
3.64201	-0.003	0.004	4.53E-01	0.007	0.005	2.08E-01
0.994622217	0.004	0.006	4.43E-01	0.011	0.009	2.00E-01
7.954067	0.007	0.009	4.25E-01	-0.004	0.013	7.92E-01
7.287257	-0.009	0.009	3.67E-01	0.000	0.014	9.76E-01
6.602954	0.008	0.009	3.86E-01	0.005	0.014	6.89E-01
1.078730365	0.007	0.008	3.25E-01	0.003	0.011	7.61E-01
2.368612919	-0.005	0.007	5.17E-01	0.023	0.011	3.76E-02
2.579556154	-0.004	0.011	7.18E-01	0.018	0.016	2.40E-01
8.51019	0.006	0.007	3.65E-01	0.001	0.010	9.52E-01
0.641367996	0.009	0.010	3.96E-01	-0.019	0.015	2.16E-01
2.286186934	-0.011	0.011	3.51E-01	0.003	0.017	8.73E-01
8.776308	0.008	0.007	2.53E-01	0.008	0.011	4.70E-01
2.719512112	-0.008	0.007	2.38E-01	-0.014	0.010	1.90E-01
6.203944	0.003	0.008	7.24E-01	-0.021	0.012	7.64E-02
7.563132	0.007	0.009	4.65E-01	0.002	0.014	8.67E-01
7.189356	-0.012	0.011	2.59E-01	-0.010	0.016	5.42E-01
4.235477	0.010	0.013	4.43E-01	0.016	0.020	4.07E-01
6.254073	0.007	0.008	3.89E-01	-0.009	0.012	4.62E-01
2.08701884	-0.005	0.006	3.90E-01	0.009	0.009	3.29E-01
1.471347198	-0.003	0.005	5.13E-01	-0.001	0.007	8.47E-01
2.279794715	-0.006	0.006	3.28E-01	-0.009	0.009	2.85E-01
7.985691	0.007	0.008	4.02E-01	0.005	0.012	6.96E-01
6.428009	0.009	0.007	2.24E-01	0.003	0.011	7.79E-01
3.915866	-0.008	0.009	3.55E-01	0.027	0.013	4.18E-02
7.668436	0.007	0.009	4.34E-01	-0.011	0.014	4.29E-01
8.492023	0.009	0.010	3.53E-01	-0.018	0.014	2.12E-01
3.605339	0.008	0.010	4.45E-01	-0.012	0.015	4.28E-01
2.657944947	-0.007	0.008	3.96E-01	0.014	0.012	2.45E-01
5.054018	0.006	0.008	4.27E-01	-0.004	0.012	7.64E-01
1.626779056	0.005	0.006	4.14E-01	-0.006	0.008	4.45E-01

6.827354	-0.006	0.008	4.52E-01	0.006	0.012	5.78E-01
5.835551	0.008	0.008	3.33E-01	0.007	0.012	5.66E-01
2.15968828	-0.007	0.008	3.90E-01	-0.016	0.013	2.04E-01
8.902134	-0.008	0.008	3.40E-01	0.004	0.012	7.33E-01
5.69324	0.008	0.009	3.81E-01	0.001	0.013	9.44E-01
7.987374	0.006	0.009	5.06E-01	-0.016	0.013	2.11E-01
4.103259	-0.005	0.010	6.45E-01	0.006	0.016	6.88E-01
7.635129	-0.007	0.012	5.51E-01	0.021	0.018	2.36E-01
6.399412	-0.009	0.010	3.62E-01	-0.024	0.015	1.03E-01
8.700611	0.006	0.007	4.27E-01	-0.003	0.011	8.14E-01
7.253278	0.005	0.010	6.34E-01	-0.009	0.014	5.27E-01
2.14286665	-0.010	0.012	4.11E-01	0.018	0.018	3.07E-01
2.754837534	-0.009	0.007	2.28E-01	-0.018	0.011	8.24E-02
8.354758	0.011	0.010	2.80E-01	-0.019	0.015	1.98E-01
7.17758	0.009	0.011	3.75E-01	0.024	0.016	1.30E-01
1.915774651	-0.006	0.006	2.97E-01	0.002	0.009	8.41E-01
7.156385	-0.005	0.007	4.85E-01	-0.004	0.010	6.66E-01
8.936786	0.007	0.009	4.19E-01	0.004	0.013	7.69E-01
5.060747	0.005	0.008	5.13E-01	-0.006	0.012	6.32E-01
8.062398	-0.006	0.007	4.29E-01	0.006	0.011	5.71E-01
8.954281	0.007	0.009	4.50E-01	0.010	0.013	4.55E-01
6.849222	-0.008	0.009	3.89E-01	-0.012	0.013	3.49E-01
3.900391	0.004	0.005	4.31E-01	0.022	0.008	5.77E-03
6.534994	-0.010	0.008	2.47E-01	-0.004	0.013	7.52E-01
1.417517984	0.005	0.005	2.90E-01	0.003	0.007	6.68E-01
3.518203	0.006	0.007	3.53E-01	0.021	0.010	3.94E-02
6.745601	-0.009	0.009	3.23E-01	-0.018	0.014	1.85E-01
8.248445	-0.006	0.008	4.79E-01	-0.011	0.013	3.68E-01
1.046769268	0.004	0.007	5.90E-01	-0.010	0.010	3.16E-01
7.541264	0.006	0.009	5.33E-01	0.005	0.013	6.98E-01
8.06408	0.009	0.009	3.28E-01	0.003	0.014	8.29E-01
6.152134	0.010	0.009	2.72E-01	0.011	0.013	4.15E-01
3.21104	-0.015	0.014	2.93E-01	-0.003	0.021	9.06E-01
0.51587864	-0.010	0.008	2.36E-01	0.009	0.012	4.70E-01
7.83396	-0.006	0.011	5.69E-01	0.001	0.016	9.38E-01
2.343044042	0.009	0.006	1.51E-01	-0.002	0.009	8.14E-01
2.581911182	-0.001	0.013	9.33E-01	-0.017	0.019	3.70E-01
3.783312	-0.013	0.011	2.62E-01	-0.001	0.017	9.42E-01
5.758844	0.007	0.009	4.60E-01	0.001	0.014	9.45E-01
6.112098	0.006	0.008	5.09E-01	-0.012	0.013	3.36E-01
1.66244091	0.004	0.004	3.85E-01	0.005	0.006	3.97E-01
6.779581	0.009	0.008	2.58E-01	0.008	0.012	5.14E-01
4.108642	-0.006	0.010	5.03E-01	0.006	0.014	6.97E-01

4.207553	-0.008	0.011	5.06E-01	0.009	0.017	6.11E-01
8.437184	-0.004	0.006	4.55E-01	-0.022	0.009	1.16E-02
5.578853	0.008	0.008	3.56E-01	-0.018	0.013	1.53E-01
8.277042	0.006	0.009	5.05E-01	0.018	0.014	1.80E-01
2.721530707	-0.009	0.008	2.56E-01	-0.007	0.012	5.31E-01
8.09335	0.009	0.009	3.14E-01	-0.004	0.013	7.62E-01
7.919751	-0.009	0.009	3.08E-01	-0.019	0.013	1.59E-01
5.320809	0.015	0.017	3.74E-01	-0.034	0.025	1.80E-01
2.836254221	-0.005	0.010	6.01E-01	-0.036	0.015	1.79E-02
7.71991	-0.012	0.013	3.30E-01	-0.019	0.019	3.17E-01
1.755296305	0.009	0.008	2.97E-01	0.012	0.012	3.25E-01
5.597356	-0.008	0.009	3.97E-01	-0.016	0.014	2.42E-01
5.693913	0.005	0.008	5.51E-01	-0.009	0.012	4.57E-01
2.721194275	-0.009	0.008	2.54E-01	-0.008	0.011	4.94E-01
2.603442868	-0.014	0.015	3.44E-01	0.022	0.022	3.10E-01
8.395466	0.006	0.009	4.77E-01	-0.003	0.013	8.15E-01
2.407302667	-0.019	0.020	3.39E-01	0.008	0.029	7.75E-01
2.596041351	-0.014	0.019	4.40E-01	0.005	0.028	8.51E-01
4.385863	-0.012	0.014	3.91E-01	0.010	0.020	6.36E-01
1.855553218	-0.009	0.009	3.06E-01	-0.019	0.014	1.64E-01
5.942536	0.008	0.010	4.04E-01	-0.007	0.014	6.29E-01
6.320014	0.007	0.008	3.51E-01	0.005	0.011	6.39E-01
4.175592	-0.013	0.015	3.81E-01	0.032	0.022	1.46E-01
3.339557	-0.007	0.009	4.23E-01	0.015	0.013	2.32E-01
5.476577	0.007	0.007	3.54E-01	0.002	0.011	8.43E-01
4.324632	-0.013	0.016	4.27E-01	0.022	0.024	3.51E-01
3.803498	0.006	0.006	2.68E-01	0.012	0.008	1.48E-01
2.597050648	-0.015	0.018	4.02E-01	0.006	0.027	8.39E-01
8.386046	0.011	0.008	2.07E-01	-0.003	0.013	8.28E-01
0.668955469	-0.010	0.010	3.15E-01	-0.028	0.015	5.49E-02
6.087539	0.011	0.011	3.15E-01	0.012	0.016	4.33E-01
7.221653	-0.009	0.009	2.81E-01	-0.009	0.013	4.67E-01
5.680455	0.005	0.008	5.55E-01	-0.001	0.012	9.63E-01
2.719848544	-0.008	0.007	2.49E-01	-0.012	0.011	2.56E-01
6.671586	0.007	0.008	3.78E-01	-0.004	0.012	7.24E-01
4.207217	-0.009	0.012	4.71E-01	0.001	0.018	9.52E-01
8.923666	-0.006	0.009	4.57E-01	-0.010	0.013	4.32E-01
6.871763	-0.005	0.007	4.44E-01	-0.007	0.011	5.09E-01
8.067108	0.009	0.008	2.49E-01	0.028	0.011	1.29E-02
6.992206	0.004	0.007	5.51E-01	0.004	0.010	7.28E-01
6.107388	0.007	0.011	5.17E-01	-0.011	0.017	5.35E-01
8.703639	0.004	0.008	6.10E-01	0.012	0.012	3.11E-01
1.235507952	0.006	0.008	4.30E-01	-0.007	0.011	5.26E-01

7.743124	-0.009	0.010	3.90E-01	-0.029	0.015	5.34E-02
8.459389	0.004	0.007	5.58E-01	-0.010	0.011	3.53E-01
7.008018	-0.011	0.010	2.65E-01	-0.022	0.014	1.19E-01
5.528051	0.006	0.008	4.82E-01	0.000	0.012	9.74E-01
1.004378762	0.008	0.009	3.54E-01	0.002	0.013	8.59E-01
7.56616	-0.008	0.007	2.72E-01	0.007	0.011	4.91E-01
3.735202	-0.012	0.009	1.62E-01	0.004	0.013	7.89E-01
0.587875214	-0.006	0.007	3.93E-01	-0.009	0.010	3.66E-01
7.993429	0.008	0.009	3.27E-01	0.018	0.013	1.49E-01
0.970062638	-0.004	0.005	3.96E-01	0.008	0.008	2.82E-01
3.735539	-0.013	0.009	1.49E-01	0.006	0.014	6.55E-01
3.324081	0.008	0.010	4.14E-01	0.021	0.015	1.45E-01
2.271720333	0.006	0.006	3.49E-01	0.001	0.010	8.79E-01
6.538022	0.004	0.009	6.72E-01	0.014	0.014	3.17E-01
0.586529484	-0.007	0.010	4.58E-01	-0.036	0.015	1.45E-02
0.521597994	0.008	0.010	4.06E-01	-0.009	0.014	5.06E-01
8.715077	0.006	0.009	5.15E-01	0.012	0.013	3.49E-01
2.039918277	0.005	0.004	2.71E-01	0.009	0.007	1.69E-01
5.520313	-0.009	0.008	3.08E-01	-0.004	0.012	7.48E-01
3.256459	0.008	0.010	4.15E-01	0.016	0.015	3.03E-01
8.22725	0.004	0.008	6.06E-01	-0.004	0.012	7.28E-01
4.00132	0.008	0.009	3.92E-01	0.018	0.014	1.88E-01
1.524167115	0.009	0.009	3.32E-01	-0.014	0.014	3.30E-01
3.279336	-0.011	0.011	3.23E-01	0.008	0.016	6.45E-01
8.975476	0.005	0.007	4.82E-01	-0.009	0.011	4.12E-01
0.758446538	0.005	0.005	3.62E-01	-0.001	0.008	8.70E-01
4.386199	-0.011	0.013	3.97E-01	0.004	0.019	8.33E-01
8.820717	-0.007	0.009	4.12E-01	-0.017	0.013	1.97E-01
7.590047	-0.009	0.010	3.70E-01	-0.006	0.015	7.14E-01
0.743643504	-0.006	0.006	3.38E-01	-0.011	0.009	2.19E-01
8.551571	0.009	0.009	3.25E-01	0.021	0.013	1.12E-01
8.977158	0.011	0.010	2.51E-01	0.021	0.014	1.42E-01
1.471010766	-0.003	0.005	5.26E-01	0.000	0.008	9.66E-01
1.451834108	0.004	0.004	2.82E-01	-0.002	0.006	7.28E-01
2.984621	-0.003	0.004	4.36E-01	0.004	0.006	5.69E-01
6.331452	0.008	0.010	3.97E-01	0.009	0.015	5.61E-01
6.307566	0.007	0.007	3.48E-01	-0.007	0.011	5.26E-01
4.449112	-0.013	0.018	4.45E-01	0.028	0.026	2.87E-01
5.4537	0.006	0.008	4.49E-01	-0.022	0.013	7.58E-02
2.579219721	-0.004	0.009	6.67E-01	0.008	0.014	5.66E-01
1.505999755	0.005	0.006	4.17E-01	-0.011	0.009	2.07E-01
7.150329	-0.007	0.007	3.20E-01	-0.001	0.010	9.59E-01
5.640083	0.009	0.008	2.84E-01	-0.012	0.012	3.26E-01

8.367206	0.005	0.008	5.25E-01	-0.012	0.012	3.26E-01
0.710336677	0.008	0.008	3.03E-01	0.004	0.012	7.15E-01
7.08708	0.008	0.006	1.97E-01	0.001	0.009	8.80E-01
8.214129	0.005	0.006	4.24E-01	-0.001	0.009	9.21E-01
3.108092	-0.012	0.010	2.18E-01	-0.010	0.015	4.97E-01
4.386536	-0.011	0.013	4.07E-01	0.009	0.019	6.28E-01
7.778113	-0.008	0.009	3.92E-01	-0.004	0.014	7.62E-01
6.624485	-0.011	0.010	2.67E-01	-0.013	0.015	3.88E-01
5.404917	0.005	0.006	4.49E-01	-0.003	0.009	7.70E-01
8.109499	0.012	0.010	2.18E-01	0.023	0.014	1.10E-01
7.027531	-0.015	0.010	1.20E-01	-0.035	0.014	1.70E-02
4.105614	-0.008	0.011	4.76E-01	0.016	0.017	3.56E-01
1.440058967	0.004	0.004	2.87E-01	0.006	0.006	3.26E-01
5.067812	0.007	0.008	3.77E-01	-0.008	0.012	4.98E-01
1.732418889	-0.006	0.005	2.55E-01	0.003	0.008	7.28E-01
0.546830438	0.011	0.008	1.89E-01	0.029	0.012	1.40E-02
3.691129	-0.004	0.004	3.75E-01	0.009	0.007	1.89E-01
8.273341	0.008	0.009	3.83E-01	0.003	0.013	8.34E-01
3.744622	-0.013	0.010	2.11E-01	-0.018	0.015	2.45E-01
0.833471006	-0.009	0.008	2.66E-01	-0.004	0.012	7.19E-01
8.223886	0.004	0.009	6.45E-01	-0.005	0.013	7.17E-01
8.446268	0.005	0.008	4.82E-01	0.005	0.011	6.89E-01
6.470063	0.005	0.006	4.22E-01	-0.003	0.008	7.11E-01
0.578455102	-0.004	0.008	6.32E-01	0.020	0.011	8.17E-02
2.666355762	-0.006	0.006	2.59E-01	0.003	0.009	6.86E-01
7.434278	0.004	0.010	6.80E-01	-0.016	0.015	2.82E-01
1.517774896	0.007	0.008	4.12E-01	-0.015	0.013	2.30E-01
8.765879	-0.007	0.009	4.11E-01	-0.018	0.013	1.73E-01
7.534199	0.005	0.009	5.41E-01	0.011	0.013	4.15E-01
5.996029	0.006	0.008	4.75E-01	0.000	0.012	9.71E-01
6.920209	-0.009	0.009	3.14E-01	0.014	0.013	2.67E-01
1.223732811	0.006	0.008	4.46E-01	-0.002	0.011	8.44E-01
5.670699	0.006	0.008	5.06E-01	0.007	0.013	5.76E-01
5.821421	0.007	0.009	4.71E-01	0.020	0.014	1.51E-01
5.430486	0.007	0.007	3.56E-01	-0.006	0.011	5.78E-01
2.660972841	-0.014	0.014	3.33E-01	-0.007	0.021	7.50E-01
7.430241	0.008	0.010	4.53E-01	0.001	0.015	9.25E-01
6.295791	0.006	0.010	5.32E-01	0.005	0.014	7.21E-01
2.287869097	-0.005	0.005	3.95E-01	0.002	0.008	7.69E-01
1.812826278	-0.008	0.011	4.20E-01	0.010	0.016	5.13E-01
6.238597	0.009	0.009	3.06E-01	-0.011	0.013	4.22E-01
5.815365	0.006	0.007	4.22E-01	0.010	0.010	3.38E-01
8.922656	-0.005	0.007	4.24E-01	0.006	0.010	5.33E-01

4.370723	-0.010	0.013	4.28E-01	0.014	0.019	4.59E-01
6.32977	0.008	0.008	3.25E-01	-0.013	0.012	2.74E-01
8.675378	0.005	0.007	4.24E-01	0.003	0.010	7.78E-01
2.098457548	-0.004	0.008	6.69E-01	-0.006	0.013	6.07E-01
7.701742	-0.007	0.008	3.95E-01	0.013	0.013	3.14E-01
1.453852704	0.003	0.004	3.73E-01	0.000	0.005	9.76E-01
5.913603	-0.006	0.008	4.35E-01	0.003	0.012	7.72E-01
5.8083	0.004	0.009	6.68E-01	-0.023	0.013	8.87E-02
5.522668	-0.007	0.007	3.22E-01	-0.009	0.011	4.36E-01
4.226057	0.010	0.013	4.43E-01	0.027	0.020	1.84E-01
1.888523611	-0.005	0.006	4.06E-01	0.000	0.010	9.78E-01
8.656874	0.006	0.007	4.49E-01	0.003	0.011	7.61E-01
6.661493	0.008	0.009	3.81E-01	0.005	0.014	7.34E-01
8.841576	0.008	0.009	3.56E-01	-0.015	0.014	2.85E-01
2.129409347	-0.008	0.011	4.45E-01	-0.003	0.017	8.72E-01
6.72777	-0.010	0.011	3.83E-01	-0.009	0.017	6.13E-01
8.180149	-0.010	0.010	3.41E-01	-0.022	0.016	1.64E-01
6.788664	0.011	0.010	2.61E-01	0.012	0.015	4.21E-01
8.829801	0.001	0.010	8.82E-01	0.004	0.015	7.84E-01
4.367695	-0.011	0.015	4.87E-01	0.009	0.023	7.06E-01
8.29992	-0.006	0.007	3.93E-01	0.001	0.011	9.49E-01
7.940946	-0.008	0.009	3.68E-01	0.000	0.013	9.94E-01
7.880052	-0.007	0.008	4.11E-01	0.005	0.012	6.65E-01
5.987955	0.007	0.008	4.24E-01	0.016	0.012	1.80E-01
1.862281869	-0.006	0.008	4.32E-01	0.012	0.012	3.13E-01
7.18801	-0.010	0.011	3.95E-01	-0.006	0.017	7.36E-01
1.980369709	0.005	0.005	3.24E-01	-0.004	0.008	6.17E-01
7.875005	0.007	0.008	3.46E-01	0.004	0.012	7.47E-01
3.773219	0.007	0.011	4.97E-01	0.030	0.016	6.60E-02
5.032486	0.005	0.008	5.29E-01	-0.003	0.012	8.23E-01
1.897943724	-0.005	0.006	4.01E-01	-0.005	0.009	6.05E-01
5.732266	0.006	0.009	4.75E-01	-0.004	0.013	7.30E-01
7.406018	-0.012	0.011	2.74E-01	-0.009	0.016	5.87E-01
5.650176	0.007	0.010	4.80E-01	-0.006	0.014	6.89E-01
8.42339	-0.007	0.009	4.72E-01	-0.018	0.014	1.91E-01
3.203639	0.006	0.007	4.37E-01	0.018	0.011	8.72E-02
3.304568	-0.010	0.010	3.04E-01	-0.008	0.015	6.06E-01
8.101761	-0.005	0.009	5.32E-01	-0.021	0.013	1.11E-01
3.796769	0.006	0.006	3.05E-01	0.017	0.008	4.63E-02
2.501503793	-0.010	0.013	4.15E-01	-0.008	0.019	6.58E-01
4.39932	-0.010	0.014	4.86E-01	0.008	0.021	6.93E-01
3.165622	-0.006	0.007	4.16E-01	0.007	0.010	5.15E-01
0.563652068	-0.007	0.007	3.57E-01	-0.006	0.011	5.81E-01

7.589037	-0.005	0.010	6.11E-01	-0.011	0.014	4.51E-01
6.324387	0.006	0.007	4.18E-01	0.001	0.010	8.86E-01
0.658862491	-0.011	0.009	2.38E-01	-0.010	0.014	4.90E-01
6.26282	0.008	0.008	2.94E-01	-0.004	0.012	7.12E-01
8.431128	0.004	0.007	5.43E-01	0.030	0.011	4.92E-03
5.030131	0.007	0.009	4.36E-01	-0.012	0.014	3.69E-01
6.412869	0.003	0.008	6.59E-01	-0.008	0.011	4.77E-01
8.0698	0.007	0.009	4.65E-01	-0.008	0.013	5.54E-01
6.381244	-0.007	0.008	3.35E-01	-0.028	0.011	1.35E-02
7.857511	0.005	0.007	4.87E-01	0.010	0.011	3.44E-01
7.312153	-0.010	0.009	2.59E-01	-0.010	0.013	4.22E-01
3.536034	0.007	0.008	3.77E-01	0.018	0.012	1.39E-01
6.798084	0.010	0.009	2.86E-01	0.017	0.014	2.25E-01
8.777654	-0.009	0.009	3.21E-01	-0.008	0.013	5.70E-01
6.045148	0.007	0.008	3.62E-01	0.005	0.011	6.69E-01
8.471164	0.006	0.006	3.39E-01	-0.009	0.009	3.55E-01
5.240065	0.001	0.005	8.47E-01	0.006	0.007	4.16E-01
5.603076	0.007	0.008	3.73E-01	0.006	0.012	6.23E-01
6.128247	-0.007	0.010	4.78E-01	-0.026	0.015	7.82E-02
2.588639834	-0.005	0.010	6.40E-01	-0.005	0.015	7.49E-01
3.621151	-0.005	0.006	4.08E-01	-0.008	0.008	3.34E-01
3.176724	-0.005	0.006	4.07E-01	0.007	0.008	3.95E-01
1.063590898	-0.007	0.006	3.00E-01	-0.009	0.009	3.59E-01
2.560043063	-0.009	0.010	3.77E-01	0.006	0.015	7.06E-01
6.372161	0.004	0.006	4.82E-01	-0.005	0.009	5.70E-01
0.589557377	0.006	0.007	3.72E-01	0.008	0.010	4.41E-01
1.904335943	-0.006	0.007	3.57E-01	-0.004	0.010	7.13E-01
1.159474186	0.006	0.005	2.23E-01	-0.004	0.007	5.31E-01
7.102556	-0.009	0.009	3.33E-01	-0.008	0.014	5.66E-01
7.533862	0.008	0.008	3.48E-01	0.020	0.013	1.18E-01
6.79001	0.010	0.009	2.73E-01	0.007	0.013	6.12E-01
5.724192	0.005	0.011	6.26E-01	-0.009	0.016	5.87E-01
1.689355517	-0.004	0.004	3.24E-01	0.001	0.005	8.44E-01
5.623262	0.004	0.009	6.57E-01	-0.009	0.013	5.04E-01
7.840689	-0.006	0.008	4.50E-01	0.016	0.012	1.91E-01
7.974926	0.005	0.008	5.06E-01	-0.008	0.012	4.85E-01
7.36531	-0.006	0.008	4.08E-01	-0.003	0.012	8.14E-01
4.185349	-0.009	0.014	5.01E-01	0.013	0.021	5.43E-01
6.930302	0.006	0.009	4.82E-01	0.001	0.014	9.44E-01
3.177397	-0.005	0.006	4.06E-01	0.010	0.008	2.48E-01
6.761413	-0.005	0.009	6.00E-01	-0.016	0.014	2.71E-01
5.107511	0.006	0.009	4.71E-01	-0.008	0.013	5.48E-01
8.588242	0.005	0.008	5.32E-01	0.005	0.011	6.44E-01

8.054997	0.004	0.008	6.56E-01	0.009	0.012	4.77E-01
7.967524	0.005	0.006	3.87E-01	-0.002	0.009	8.09E-01
7.809064	-0.006	0.008	4.82E-01	-0.019	0.012	1.25E-01
7.194738	0.008	0.008	3.05E-01	0.002	0.012	8.90E-01
4.184676	-0.009	0.013	5.00E-01	0.003	0.019	8.79E-01
6.908098	0.007	0.007	3.21E-01	0.011	0.011	3.35E-01
7.195075	0.009	0.008	2.81E-01	-0.001	0.012	9.29E-01
6.048513	0.005	0.008	5.49E-01	-0.002	0.012	8.78E-01
8.814325	-0.008	0.009	4.05E-01	0.005	0.014	7.39E-01
5.056036	0.006	0.008	4.59E-01	-0.008	0.012	4.97E-01
8.188224	-0.011	0.010	2.80E-01	-0.009	0.015	5.19E-01
7.115677	-0.006	0.009	5.12E-01	0.003	0.014	8.39E-01
4.335398	-0.009	0.012	4.69E-01	0.016	0.018	3.88E-01
5.322155	0.015	0.017	3.76E-01	-0.031	0.026	2.32E-01
8.68278	0.008	0.009	3.55E-01	-0.013	0.014	3.27E-01
8.685808	0.007	0.008	3.98E-01	0.002	0.012	8.85E-01
1.873384145	-0.007	0.008	3.45E-01	0.003	0.011	7.73E-01
4.29839	-0.010	0.013	4.41E-01	0.009	0.020	6.31E-01
4.337753	-0.010	0.014	4.80E-01	0.014	0.021	4.97E-01
8.608428	-0.009	0.010	3.33E-01	0.008	0.014	5.54E-01
7.261352	-0.010	0.010	2.87E-01	-0.026	0.014	6.63E-02
4.025207	-0.008	0.013	5.51E-01	0.008	0.019	6.69E-01
5.646812	0.009	0.008	2.74E-01	-0.007	0.012	5.26E-01
8.376963	0.007	0.007	3.00E-01	-0.008	0.010	3.89E-01
7.280865	-0.011	0.011	2.93E-01	-0.017	0.016	2.79E-01
7.549338	-0.009	0.009	3.38E-01	-0.001	0.013	9.52E-01
6.40614	0.004	0.006	5.37E-01	-0.002	0.010	8.72E-01
3.05258	-0.006	0.009	4.58E-01	0.012	0.013	3.31E-01
6.606991	0.007	0.008	3.79E-01	0.009	0.012	4.22E-01
6.444157	0.008	0.008	3.35E-01	-0.001	0.012	9.15E-01
5.959022	0.005	0.008	5.05E-01	-0.005	0.012	7.11E-01
8.165346	0.007	0.009	3.85E-01	-0.002	0.013	8.79E-01
8.306648	-0.010	0.008	2.27E-01	-0.009	0.012	4.57E-01
2.186266455	0.011	0.011	3.05E-01	-0.024	0.016	1.48E-01
3.127268	-0.013	0.010	1.77E-01	0.007	0.014	6.37E-01
1.97532322	0.004	0.005	4.07E-01	-0.004	0.007	5.73E-01
5.021047	0.007	0.010	4.70E-01	-0.011	0.014	4.54E-01
1.969267433	0.004	0.004	4.14E-01	-0.002	0.007	7.89E-01
8.201008	0.005	0.008	5.26E-01	-0.013	0.012	3.00E-01
6.671249	0.009	0.009	3.49E-01	-0.007	0.014	5.99E-01
0.901430389	0.008	0.007	2.74E-01	-0.016	0.010	1.29E-01
5.906538	-0.008	0.009	3.50E-01	-0.010	0.013	4.36E-01
7.692322	0.007	0.007	3.06E-01	0.009	0.010	3.97E-01

7.544628	-0.006	0.010	5.00E-01	-0.013	0.014	3.45E-01
4.04472	0.015	0.012	2.14E-01	-0.002	0.018	9.28E-01
4.422197	-0.011	0.015	4.52E-01	0.022	0.022	3.08E-01
5.007254	0.008	0.010	4.06E-01	0.001	0.015	9.51E-01
5.613169	0.004	0.007	5.27E-01	-0.005	0.010	6.35E-01
7.724956	-0.007	0.009	4.43E-01	-0.010	0.014	4.56E-01
1.765052851	-0.008	0.010	4.07E-01	0.010	0.014	4.78E-01
5.414337	0.009	0.009	3.35E-01	0.004	0.014	7.96E-01
7.844053	0.007	0.008	3.97E-01	0.001	0.012	9.58E-01
1.65066577	0.004	0.005	3.45E-01	0.001	0.007	8.72E-01
1.190425985	0.006	0.006	3.87E-01	0.001	0.010	9.05E-01
1.974650355	0.004	0.005	4.14E-01	-0.003	0.007	6.90E-01
1.692719843	-0.004	0.004	3.48E-01	0.007	0.006	2.41E-01
0.585856619	0.010	0.010	3.17E-01	0.007	0.014	6.36E-01
5.84127	-0.006	0.008	4.80E-01	-0.022	0.013	8.28E-02
7.059829	-0.007	0.009	4.67E-01	0.007	0.014	5.95E-01
1.514747003	0.007	0.008	3.60E-01	-0.013	0.012	2.58E-01
8.360141	0.005	0.008	4.86E-01	-0.012	0.012	2.91E-01
3.570014	-0.005	0.007	4.24E-01	0.010	0.010	3.15E-01
0.612771226	-0.005	0.008	5.25E-01	0.000	0.012	9.95E-01
0.949203817	0.005	0.005	3.47E-01	-0.001	0.008	8.68E-01
3.87684	-0.004	0.004	3.81E-01	0.014	0.007	3.94E-02
6.433728	-0.006	0.007	4.04E-01	0.011	0.011	3.23E-01
6.221439	0.007	0.008	4.10E-01	-0.017	0.012	1.75E-01
2.160697578	-0.008	0.009	3.67E-01	-0.006	0.013	6.19E-01
8.477556	-0.007	0.011	5.07E-01	-0.019	0.016	2.35E-01
0.646078052	-0.006	0.008	4.86E-01	0.003	0.013	8.14E-01
7.718564	-0.008	0.010	4.44E-01	-0.013	0.015	3.92E-01
6.129929	0.004	0.008	6.03E-01	0.004	0.012	7.20E-01
8.291509	0.010	0.009	2.79E-01	-0.006	0.013	6.39E-01
1.456207732	0.002	0.004	5.11E-01	-0.002	0.005	7.74E-01
5.595674	0.007	0.010	4.76E-01	-0.011	0.015	4.62E-01
1.468655738	0.010	0.007	1.72E-01	-0.006	0.011	6.01E-01
5.043588	0.005	0.009	5.61E-01	-0.010	0.013	4.44E-01
8.353076	-0.006	0.008	4.08E-01	0.000	0.011	9.96E-01
6.409841	-0.009	0.009	3.34E-01	-0.013	0.013	3.30E-01
5.86482	0.006	0.009	5.39E-01	-0.013	0.014	3.49E-01
2.272393198	0.006	0.007	3.99E-01	-0.003	0.010	7.79E-01
1.470674333	-0.003	0.005	5.57E-01	0.000	0.008	9.84E-01
2.589312699	-0.007	0.017	6.64E-01	0.028	0.025	2.71E-01
4.229758	-0.008	0.013	5.18E-01	0.015	0.019	4.25E-01
6.524228	0.004	0.007	5.85E-01	-0.006	0.011	5.78E-01
8.056342	0.005	0.007	4.96E-01	0.006	0.010	5.31E-01

5.519977	-0.008	0.010	4.43E-01	-0.013	0.015	3.80E-01
3.74765	0.006	0.008	4.77E-01	0.026	0.012	3.75E-02
8.603045	0.006	0.009	5.52E-01	-0.025	0.014	7.86E-02
6.551816	-0.006	0.006	3.48E-01	-0.020	0.009	2.57E-02
4.163817	-0.008	0.010	4.35E-01	0.005	0.015	7.48E-01
8.339955	0.004	0.006	4.89E-01	0.004	0.009	6.85E-01
4.11739	-0.007	0.011	5.09E-01	0.013	0.016	4.17E-01
5.720491	0.005	0.007	5.03E-01	0.002	0.011	8.78E-01
8.49135	-0.008	0.008	3.17E-01	-0.019	0.012	1.25E-01
0.614116956	-0.007	0.009	4.22E-01	-0.017	0.013	1.94E-01
1.141979692	0.008	0.007	2.16E-01	-0.006	0.010	5.37E-01
5.120295	0.008	0.009	3.79E-01	-0.002	0.013	8.74E-01
8.530376	-0.007	0.010	5.15E-01	0.002	0.015	8.75E-01
6.218411	0.005	0.008	5.48E-01	-0.028	0.013	2.36E-02
0.852984096	-0.006	0.007	3.54E-01	-0.012	0.010	2.36E-01
7.689631	-0.008	0.007	2.72E-01	0.006	0.011	6.11E-01
8.694218	-0.005	0.010	5.85E-01	-0.009	0.015	5.56E-01
6.483856	-0.006	0.007	4.43E-01	-0.003	0.011	7.70E-01
2.154641791	-0.006	0.008	4.76E-01	-0.013	0.012	2.87E-01
2.372986543	-0.006	0.007	4.37E-01	0.012	0.011	2.66E-01
1.468319305	0.010	0.008	1.77E-01	-0.007	0.011	5.29E-01
1.896261561	-0.006	0.006	2.93E-01	0.000	0.009	9.61E-01
3.645375	0.005	0.004	2.56E-01	0.016	0.006	8.11E-03
4.37577	-0.011	0.016	4.61E-01	0.009	0.023	6.97E-01
5.322491	0.015	0.017	3.76E-01	-0.031	0.026	2.32E-01
8.196971	0.009	0.007	1.95E-01	-0.007	0.011	5.17E-01
0.633630046	0.006	0.009	5.12E-01	0.008	0.014	5.82E-01
4.162808	-0.010	0.013	4.13E-01	-0.002	0.019	9.34E-01
0.995295082	0.004	0.006	4.95E-01	0.010	0.010	2.89E-01
4.339771	-0.010	0.014	4.60E-01	0.009	0.020	6.45E-01
1.766398581	-0.007	0.011	5.32E-01	0.009	0.016	5.79E-01
2.065487154	0.005	0.004	2.02E-01	0.011	0.005	4.45E-02
2.183238561	0.010	0.011	3.37E-01	-0.022	0.016	1.77E-01
5.01701	0.005	0.008	4.77E-01	-0.012	0.011	3.15E-01
1.980706141	0.005	0.005	3.40E-01	-0.005	0.008	5.27E-01
2.614545143	-0.010	0.012	4.15E-01	0.037	0.018	3.48E-02
0.817995106	0.003	0.005	6.30E-01	0.014	0.008	8.36E-02
4.141613	-0.012	0.014	3.95E-01	0.014	0.020	5.02E-01
0.682749205	0.012	0.013	3.57E-01	-0.012	0.019	5.29E-01
4.417487	-0.011	0.013	3.86E-01	0.013	0.019	4.86E-01
0.757100807	0.006	0.005	2.74E-01	0.005	0.008	5.12E-01
2.98563	-0.004	0.004	3.68E-01	0.005	0.006	4.58E-01
7.260343	-0.012	0.009	1.91E-01	-0.019	0.013	1.53E-01

7.11164	-0.005	0.007	4.42E-01	-0.019	0.010	5.83E-02
1.836712992	-0.005	0.009	5.83E-01	-0.014	0.013	2.96E-01
5.037196	0.007	0.008	3.69E-01	-0.022	0.012	7.12E-02
5.822093	0.003	0.008	6.64E-01	-0.005	0.012	6.73E-01
7.018784	0.003	0.009	7.70E-01	-0.008	0.014	5.64E-01
2.308727918	-0.008	0.008	3.28E-01	0.008	0.012	5.13E-01
7.040652	-0.010	0.011	3.98E-01	0.006	0.017	7.15E-01
2.544230732	-0.031	0.029	2.79E-01	-0.021	0.043	6.20E-01
6.618766	-0.011	0.010	2.62E-01	-0.041	0.015	4.61E-03
7.651614	0.010	0.008	2.08E-01	0.004	0.012	7.27E-01
5.061083	0.004	0.008	6.04E-01	-0.002	0.011	8.38E-01
7.201804	0.011	0.011	3.49E-01	0.007	0.017	6.76E-01
8.859407	0.005	0.009	5.88E-01	0.004	0.014	7.87E-01
6.389992	-0.008	0.010	3.93E-01	-0.027	0.015	6.40E-02
2.545912895	-0.025	0.028	3.73E-01	-0.041	0.041	3.16E-01
1.856898948	-0.006	0.008	4.48E-01	-0.003	0.012	7.73E-01
7.671463	0.007	0.009	4.44E-01	-0.008	0.013	5.32E-01
3.941435	-0.006	0.008	4.44E-01	0.015	0.012	2.18E-01
1.509364081	0.006	0.007	4.17E-01	-0.012	0.010	2.51E-01
3.249393	-0.007	0.006	2.70E-01	0.005	0.009	5.79E-01
6.4741	-0.007	0.009	4.57E-01	-0.020	0.014	1.51E-01
7.95474	0.008	0.009	3.96E-01	-0.016	0.014	2.34E-01
2.4076391	-0.018	0.019	3.35E-01	0.009	0.029	7.54E-01
5.78946	0.007	0.009	4.20E-01	-0.011	0.013	4.09E-01
2.535819917	-0.010	0.011	3.45E-01	0.001	0.017	9.36E-01
6.606654	0.007	0.008	3.78E-01	0.023	0.011	3.80E-02
7.138218	0.006	0.009	4.91E-01	-0.020	0.014	1.34E-01
8.617175	0.004	0.007	5.25E-01	0.000	0.010	9.99E-01
1.443759726	0.004	0.004	3.09E-01	0.008	0.006	1.50E-01
5.013646	0.007	0.008	4.01E-01	0.001	0.012	9.49E-01
5.904183	0.005	0.008	5.05E-01	-0.008	0.011	5.03E-01
7.572889	-0.006	0.007	4.10E-01	0.002	0.011	8.52E-01
5.524014	0.006	0.007	3.83E-01	-0.009	0.011	4.29E-01
6.799094	0.008	0.010	4.03E-01	0.019	0.015	1.83E-01
5.541172	0.007	0.009	4.42E-01	0.013	0.014	3.43E-01
6.265848	0.007	0.007	3.20E-01	-0.005	0.010	6.01E-01
8.074846	0.008	0.010	3.93E-01	0.000	0.014	9.73E-01
4.241533	0.012	0.013	3.72E-01	-0.013	0.020	5.06E-01
8.122283	0.005	0.008	5.42E-01	-0.009	0.012	4.69E-01
6.70893	-0.005	0.008	5.65E-01	-0.013	0.012	2.68E-01
1.756642036	-0.004	0.008	6.56E-01	0.018	0.012	1.29E-01
5.432841	0.007	0.010	4.73E-01	0.002	0.014	8.99E-01
5.676082	0.004	0.009	6.55E-01	-0.001	0.013	9.31E-01

7.837661	-0.008	0.009	3.90E-01	0.011	0.014	4.23E-01
8.422045	0.006	0.007	3.71E-01	-0.025	0.010	1.40E-02
5.573133	0.009	0.009	3.06E-01	-0.011	0.013	4.06E-01
3.547809	0.012	0.010	2.37E-01	0.019	0.015	2.12E-01
2.285514069	-0.008	0.009	3.73E-01	0.002	0.013	8.73E-01
6.492604	-0.009	0.010	3.43E-01	-0.018	0.015	2.37E-01
6.974712	-0.010	0.009	2.58E-01	0.003	0.013	8.18E-01
6.915836	-0.009	0.009	3.03E-01	-0.020	0.013	1.20E-01
8.228259	-0.009	0.009	3.37E-01	-0.021	0.013	1.16E-01
1.20018253	0.004	0.006	4.85E-01	0.007	0.010	4.44E-01
6.426999	0.005	0.006	3.79E-01	0.003	0.009	7.07E-01
3.772883	0.006	0.010	5.88E-01	0.030	0.016	5.91E-02
1.224069244	0.006	0.008	4.60E-01	-0.002	0.011	8.32E-01
2.565425985	-0.006	0.008	4.50E-01	0.011	0.012	3.42E-01
3.616441	-0.004	0.006	5.47E-01	0.003	0.009	7.63E-01
3.689111	-0.004	0.004	3.94E-01	0.003	0.006	6.67E-01
6.151797	0.008	0.010	4.01E-01	0.007	0.015	6.15E-01
6.696818	-0.008	0.009	3.43E-01	0.009	0.013	5.05E-01
6.812887	0.005	0.010	6.06E-01	0.014	0.016	3.78E-01
7.682902	0.006	0.008	4.75E-01	-0.001	0.013	9.45E-01
5.881642	-0.007	0.007	3.10E-01	-0.005	0.011	6.22E-01
6.17232	0.006	0.007	4.00E-01	-0.001	0.011	9.00E-01
2.86687	-0.005	0.008	5.72E-01	0.005	0.013	7.03E-01
7.163114	0.007	0.010	4.94E-01	-0.011	0.015	4.43E-01
8.087967	-0.009	0.009	3.41E-01	-0.007	0.014	6.12E-01
3.569341	-0.005	0.007	4.59E-01	0.004	0.011	6.98E-01
1.972968192	0.004	0.005	4.38E-01	-0.004	0.007	5.61E-01
4.09283	0.014	0.013	2.79E-01	0.020	0.020	3.04E-01
1.841759481	-0.009	0.009	3.56E-01	-0.004	0.014	7.88E-01
3.227189	-0.006	0.006	3.37E-01	0.012	0.009	2.02E-01
1.869010521	-0.006	0.009	4.72E-01	-0.013	0.013	3.41E-01
3.127941	-0.012	0.010	1.93E-01	-0.003	0.014	8.51E-01
7.522087	0.004	0.008	6.51E-01	-0.006	0.012	6.33E-01
1.981379007	0.005	0.006	3.53E-01	-0.004	0.009	6.11E-01
1.498261806	-0.005	0.005	3.10E-01	-0.009	0.007	1.98E-01
4.127146	-0.006	0.009	4.95E-01	0.014	0.013	2.83E-01
6.510771	0.005	0.007	4.62E-01	-0.006	0.011	6.00E-01
5.865157	0.005	0.008	4.85E-01	0.003	0.011	7.73E-01
6.355339	-0.009	0.008	2.72E-01	-0.017	0.012	1.83E-01
5.930425	0.011	0.011	3.13E-01	0.001	0.017	9.44E-01
7.514686	0.005	0.007	4.87E-01	0.001	0.011	9.00E-01
3.92966	0.007	0.008	3.61E-01	0.027	0.012	2.13E-02
4.441038	-0.011	0.016	4.94E-01	0.020	0.024	4.03E-01

8.943851	0.008	0.008	2.96E-01	-0.005	0.011	6.26E-01
8.729207	-0.007	0.008	3.95E-01	-0.019	0.012	9.95E-02
8.98927	-0.007	0.008	3.68E-01	-0.003	0.012	7.88E-01
5.818393	0.007	0.009	4.69E-01	0.004	0.014	7.60E-01
0.6548253	-0.013	0.010	2.03E-01	-0.024	0.015	9.58E-02
0.764502324	0.005	0.005	3.79E-01	0.000	0.008	9.99E-01
6.633569	-0.006	0.009	4.72E-01	-0.023	0.013	9.14E-02
5.053345	0.005	0.007	4.43E-01	-0.005	0.011	6.58E-01
7.569188	-0.005	0.009	5.54E-01	-0.021	0.014	1.23E-01
8.432138	0.006	0.008	4.65E-01	0.003	0.012	8.15E-01
5.403235	0.006	0.007	3.61E-01	0.003	0.010	7.56E-01
3.880877	0.004	0.005	4.66E-01	0.020	0.008	1.17E-02
3.703241	0.005	0.005	3.71E-01	0.022	0.008	6.10E-03
6.391674	-0.008	0.010	4.03E-01	-0.021	0.015	1.56E-01
6.617084	0.005	0.007	4.82E-01	-0.008	0.011	4.97E-01
8.295882	0.007	0.008	3.48E-01	0.003	0.011	7.65E-01
0.500066308	0.006	0.009	5.11E-01	-0.009	0.013	4.75E-01
7.526797	-0.009	0.011	4.04E-01	-0.026	0.016	1.00E-01
1.829647908	-0.009	0.011	3.95E-01	-0.013	0.016	4.31E-01
4.002666	0.007	0.010	4.69E-01	0.014	0.015	3.50E-01
3.735875	-0.013	0.009	1.48E-01	0.011	0.014	4.20E-01
0.833134573	-0.009	0.008	2.79E-01	-0.004	0.012	7.33E-01
8.401859	0.007	0.009	4.49E-01	0.006	0.013	6.32E-01
0.94954025	0.005	0.005	3.46E-01	0.000	0.008	9.78E-01
8.456361	0.004	0.008	5.62E-01	-0.003	0.011	7.72E-01
2.28820553	-0.004	0.005	4.49E-01	0.000	0.008	9.99E-01
3.787686	0.011	0.009	1.93E-01	0.017	0.013	2.01E-01
1.009425251	0.009	0.008	3.10E-01	0.026	0.013	4.03E-02
4.332706	-0.012	0.015	4.26E-01	0.019	0.022	3.84E-01
7.83026	-0.008	0.010	4.51E-01	0.012	0.015	4.38E-01
4.319249	-0.010	0.014	4.64E-01	0.024	0.021	2.58E-01
7.800654	-0.007	0.008	3.75E-01	-0.013	0.012	2.47E-01
6.993888	0.005	0.007	4.75E-01	0.005	0.011	6.28E-01
2.77771495	-0.011	0.012	3.58E-01	-0.047	0.017	6.83E-03
0.5639885	-0.006	0.008	4.36E-01	-0.007	0.012	5.33E-01
6.654764	0.003	0.008	7.35E-01	0.013	0.011	2.40E-01
0.554231955	0.005	0.007	5.21E-01	0.009	0.011	4.07E-01
5.599375	0.008	0.008	3.15E-01	-0.006	0.012	6.01E-01
7.245203	-0.008	0.009	3.70E-01	0.006	0.014	6.50E-01
2.084663812	-0.004	0.006	4.71E-01	0.008	0.009	3.44E-01
3.615432	-0.003	0.006	6.57E-01	0.012	0.009	1.81E-01
3.703914	0.006	0.005	3.11E-01	0.022	0.008	7.39E-03
7.442016	0.007	0.008	3.94E-01	0.011	0.013	3.74E-01

7.554048	0.007	0.009	4.29E-01	0.025	0.013	5.99E-02
2.343380475	0.009	0.006	1.30E-01	-0.003	0.009	7.75E-01
6.467035	-0.004	0.009	6.97E-01	-0.022	0.014	1.13E-01
6.403113	0.008	0.009	3.95E-01	0.002	0.013	8.61E-01
2.568453878	-0.008	0.009	3.48E-01	-0.002	0.014	8.64E-01
1.872374847	-0.006	0.007	3.78E-01	0.011	0.011	2.99E-01
6.40244	-0.007	0.010	4.88E-01	-0.025	0.015	9.75E-02
3.328455	0.009	0.010	3.58E-01	0.023	0.014	1.17E-01
5.580871	0.006	0.007	4.26E-01	-0.012	0.011	2.77E-01
8.774626	-0.009	0.009	2.95E-01	-0.001	0.013	9.45E-01
6.299491	0.007	0.009	4.63E-01	-0.012	0.013	3.68E-01
6.313958	-0.007	0.009	3.97E-01	-0.027	0.013	3.78E-02
0.522943724	0.006	0.009	5.21E-01	-0.002	0.013	8.52E-01
6.611028	0.005	0.008	5.03E-01	-0.008	0.012	4.99E-01
1.507681918	0.005	0.006	4.51E-01	-0.007	0.010	4.52E-01
7.988046	0.006	0.010	5.79E-01	-0.027	0.015	7.23E-02
6.538358	0.006	0.010	5.30E-01	0.016	0.015	2.89E-01
0.638340103	-0.009	0.011	3.90E-01	0.009	0.016	5.82E-01
8.8261	0.005	0.007	4.63E-01	0.006	0.010	5.14E-01
8.580504	-0.005	0.009	5.69E-01	-0.012	0.013	3.76E-01
8.980859	0.006	0.008	4.94E-01	-0.006	0.012	6.32E-01
8.457034	0.005	0.007	4.77E-01	0.008	0.011	4.31E-01
1.455534867	0.003	0.004	4.25E-01	0.002	0.005	6.83E-01
8.18856	-0.008	0.009	3.69E-01	0.000	0.013	9.91E-01
4.227067	0.008	0.010	4.34E-01	0.013	0.015	3.88E-01
2.678130903	-0.008	0.006	2.04E-01	0.009	0.009	3.48E-01
5.15091	0.006	0.007	4.23E-01	-0.012	0.010	2.61E-01
8.252483	0.009	0.009	3.13E-01	0.009	0.013	4.76E-01
8.070136	0.008	0.008	3.10E-01	0.002	0.012	8.78E-01
8.618521	-0.007	0.007	2.91E-01	-0.014	0.010	1.41E-01
1.653020798	0.003	0.005	5.65E-01	0.006	0.007	3.66E-01
8.73459	-0.007	0.007	2.96E-01	-0.003	0.011	7.68E-01
7.206177	-0.007	0.007	3.46E-01	-0.005	0.011	6.46E-01
7.661034	0.007	0.008	3.78E-01	0.000	0.012	9.99E-01
6.395038	-0.007	0.008	4.18E-01	-0.012	0.012	3.38E-01
8.295546	0.006	0.008	4.31E-01	-0.007	0.012	5.44E-01
4.322613	-0.011	0.013	3.80E-01	0.016	0.019	3.90E-01
4.415805	-0.010	0.014	4.65E-01	0.005	0.021	7.96E-01
5.298268	0.012	0.013	3.77E-01	-0.022	0.020	2.67E-01
6.913481	-0.006	0.009	4.89E-01	-0.001	0.014	9.47E-01
1.09891632	0.008	0.008	3.20E-01	-0.013	0.012	2.81E-01
0.673329092	0.007	0.010	4.77E-01	0.007	0.015	6.27E-01
3.747987	0.006	0.008	5.01E-01	0.029	0.012	2.22E-02

7.516704	0.004	0.008	6.52E-01	-0.013	0.013	3.10E-01
5.650513	0.007	0.008	3.76E-01	-0.006	0.012	6.49E-01
2.835581356	-0.005	0.011	6.66E-01	-0.032	0.016	4.63E-02
1.119438708	0.006	0.007	3.70E-01	0.001	0.010	9.49E-01
5.617206	0.007	0.010	4.95E-01	0.010	0.014	5.01E-01
6.322705	0.007	0.009	4.55E-01	0.013	0.014	3.63E-01
2.160024713	-0.007	0.008	3.85E-01	-0.011	0.012	3.95E-01
2.515297529	-0.012	0.014	3.97E-01	-0.016	0.021	4.42E-01
1.235171519	0.006	0.008	4.57E-01	-0.007	0.011	5.26E-01
1.705504282	-0.003	0.005	4.54E-01	0.000	0.007	9.59E-01
8.683116	-0.007	0.009	4.37E-01	0.003	0.014	8.11E-01
8.919292	-0.004	0.007	5.14E-01	-0.003	0.010	7.54E-01
6.411187	-0.009	0.008	2.50E-01	-0.022	0.011	5.48E-02
4.417151	-0.011	0.014	4.07E-01	0.010	0.021	6.25E-01
8.034811	0.007	0.007	2.99E-01	0.010	0.010	3.21E-01
1.51441057	0.007	0.008	3.50E-01	-0.013	0.012	2.75E-01
7.282547	-0.010	0.010	3.30E-01	0.014	0.015	3.29E-01
2.530773428	-0.009	0.013	4.63E-01	0.002	0.019	9.36E-01
6.096959	-0.008	0.010	4.25E-01	-0.011	0.015	4.57E-01
7.931862	0.007	0.008	3.97E-01	-0.003	0.012	7.92E-01
6.823317	-0.010	0.011	3.67E-01	0.014	0.016	3.94E-01
3.303559	-0.011	0.013	3.95E-01	0.009	0.019	6.27E-01
8.922993	-0.005	0.008	5.50E-01	0.007	0.012	5.96E-01
1.415499388	0.005	0.004	2.37E-01	0.005	0.006	4.35E-01
7.917732	-0.005	0.009	5.95E-01	-0.032	0.014	1.90E-02
2.522699046	-0.008	0.012	5.08E-01	0.000	0.018	9.83E-01
4.22673	0.009	0.011	4.49E-01	0.017	0.017	3.04E-01
7.003981	-0.005	0.008	5.24E-01	-0.018	0.012	1.38E-01
7.523433	0.007	0.008	3.99E-01	-0.007	0.012	5.23E-01
7.192383	0.009	0.009	3.19E-01	0.006	0.013	6.48E-01
8.085276	0.005	0.008	5.20E-01	0.014	0.013	2.85E-01
5.321145	0.014	0.017	4.03E-01	-0.033	0.025	1.91E-01
7.099528	-0.008	0.010	4.36E-01	-0.012	0.014	4.08E-01
1.471683631	-0.003	0.005	5.22E-01	-0.001	0.007	9.01E-01
8.358459	0.004	0.009	6.69E-01	-0.004	0.013	7.45E-01
5.318454	0.014	0.016	3.98E-01	-0.032	0.025	1.87E-01
2.185593589	0.010	0.011	3.46E-01	-0.023	0.016	1.53E-01
3.217096	-0.012	0.014	3.98E-01	-0.005	0.021	7.97E-01
4.319586	-0.010	0.013	4.40E-01	0.016	0.019	3.99E-01
6.317995	-0.008	0.009	3.84E-01	-0.021	0.014	1.23E-01
6.309248	0.004	0.007	6.17E-01	-0.014	0.011	1.94E-01
3.97777	-0.007	0.010	5.02E-01	-0.007	0.015	6.30E-01
8.124975	0.006	0.009	4.84E-01	0.007	0.013	6.16E-01

6.33919	0.007	0.008	4.30E-01	0.013	0.012	3.15E-01
8.429782	0.005	0.009	5.62E-01	0.023	0.014	9.98E-02
8.65822	0.006	0.007	3.62E-01	-0.004	0.010	7.00E-01
6.425654	0.007	0.008	3.68E-01	0.012	0.012	2.97E-01
8.177122	0.007	0.009	4.36E-01	0.005	0.014	6.90E-01
5.321818	0.014	0.017	3.97E-01	-0.032	0.025	2.05E-01
2.66871079	-0.005	0.005	2.44E-01	0.009	0.007	1.87E-01
5.617879	0.007	0.010	4.58E-01	-0.012	0.014	3.84E-01
0.58081013	-0.007	0.008	3.90E-01	-0.012	0.012	2.87E-01
8.030773	-0.009	0.008	2.69E-01	0.001	0.012	9.12E-01
7.23982	-0.009	0.010	3.54E-01	-0.019	0.014	1.84E-01
5.723855	0.005	0.009	5.99E-01	-0.006	0.013	6.43E-01
5.776675	0.005	0.007	4.98E-01	-0.019	0.011	7.93E-02
7.683911	0.004	0.008	6.48E-01	-0.001	0.013	9.41E-01
2.601424272	-0.014	0.016	3.79E-01	0.005	0.023	8.21E-01
0.550867629	0.010	0.010	3.10E-01	0.030	0.014	3.75E-02
2.552641546	-0.009	0.009	3.27E-01	0.000	0.013	9.88E-01
8.191925	0.008	0.011	4.76E-01	0.026	0.016	9.23E-02
5.605431	-0.008	0.009	3.79E-01	-0.029	0.014	3.79E-02
3.596928	0.006	0.006	3.57E-01	0.021	0.010	2.53E-02
8.7356	-0.007	0.008	3.46E-01	-0.023	0.012	5.06E-02
5.885006	-0.005	0.007	4.86E-01	0.007	0.011	5.17E-01
8.948225	0.006	0.009	5.23E-01	0.002	0.013	8.89E-01
6.664521	0.013	0.011	2.32E-01	0.009	0.017	6.07E-01
3.561939	-0.006	0.007	3.96E-01	0.022	0.011	4.48E-02
7.687949	0.007	0.010	4.86E-01	0.016	0.015	2.75E-01
5.28582	-0.008	0.008	3.18E-01	-0.026	0.012	3.49E-02
6.481501	-0.009	0.008	2.43E-01	0.007	0.012	5.25E-01
8.029764	0.007	0.009	4.74E-01	0.005	0.014	7.44E-01
5.052672	0.007	0.008	4.15E-01	-0.005	0.013	6.89E-01
2.032180328	0.005	0.005	2.71E-01	0.007	0.007	2.77E-01
5.121977	0.008	0.010	3.91E-01	-0.007	0.015	6.57E-01
1.870356252	-0.007	0.009	4.41E-01	0.010	0.013	4.52E-01
8.702293	0.005	0.008	5.58E-01	0.001	0.012	9.36E-01
8.061052	-0.006	0.011	5.48E-01	-0.015	0.016	3.35E-01
6.315304	0.005	0.009	5.87E-01	-0.029	0.013	2.46E-02
7.132162	-0.007	0.010	4.55E-01	-0.017	0.015	2.68E-01
7.881734	0.009	0.009	3.10E-01	0.032	0.014	2.05E-02
2.652225593	-0.010	0.013	4.28E-01	0.016	0.019	4.00E-01
4.330351	-0.011	0.015	4.44E-01	0.021	0.022	3.53E-01
4.361976	-0.011	0.014	4.19E-01	0.006	0.021	7.68E-01
5.31879	0.014	0.016	4.00E-01	-0.033	0.025	1.87E-01
1.807106924	-0.008	0.010	4.44E-01	-0.003	0.015	8.15E-01

3.051908	-0.007	0.009	4.53E-01	0.015	0.014	2.71E-01
8.259211	-0.005	0.007	4.59E-01	-0.011	0.010	2.89E-01
3.327782	0.009	0.009	3.57E-01	0.020	0.014	1.58E-01
6.567292	-0.006	0.009	4.65E-01	-0.032	0.013	1.40E-02
8.183177	0.003	0.010	8.02E-01	-0.005	0.015	7.65E-01
1.130877416	0.009	0.007	2.23E-01	-0.006	0.011	6.01E-01
0.782333252	0.003	0.004	4.45E-01	0.004	0.007	5.44E-01
7.422503	-0.007	0.007	3.14E-01	-0.017	0.011	1.31E-01
8.129012	-0.007	0.010	5.00E-01	-0.012	0.014	4.27E-01
8.79313	-0.007	0.009	4.30E-01	-0.003	0.013	8.02E-01
8.74502	0.008	0.008	3.31E-01	-0.009	0.012	4.43E-01
0.505785662	-0.006	0.010	5.58E-01	0.003	0.015	8.27E-01
3.742267	0.002	0.008	8.51E-01	0.015	0.013	2.20E-01
4.439019	-0.012	0.016	4.45E-01	0.021	0.023	3.60E-01
6.838793	-0.009	0.009	3.19E-01	0.006	0.014	6.41E-01
5.128706	0.006	0.007	4.20E-01	-0.004	0.010	7.10E-01
7.849773	-0.015	0.011	1.82E-01	0.000	0.017	9.82E-01
5.757162	0.006	0.009	4.70E-01	0.002	0.013	8.98E-01
7.086407	0.006	0.006	3.58E-01	0.017	0.009	6.46E-02
0.656843895	0.007	0.011	5.32E-01	-0.002	0.016	8.80E-01
8.099406	0.007	0.011	4.81E-01	-0.033	0.016	3.71E-02
6.104024	0.007	0.010	4.50E-01	0.012	0.014	4.20E-01
6.873109	-0.007	0.010	4.52E-01	-0.020	0.014	1.55E-01
6.914827	-0.008	0.009	3.56E-01	-0.016	0.013	2.10E-01
1.91173746	-0.014	0.014	3.02E-01	-0.048	0.021	2.09E-02
7.540928	0.007	0.009	4.41E-01	0.015	0.013	2.62E-01
5.035178	0.008	0.011	4.76E-01	0.003	0.016	8.70E-01
7.11063	0.007	0.008	3.51E-01	-0.002	0.011	8.90E-01
4.137575	-0.011	0.013	3.93E-01	0.015	0.020	4.47E-01
2.523035478	-0.008	0.011	4.76E-01	0.006	0.017	7.18E-01
1.512728407	0.007	0.008	3.74E-01	-0.014	0.011	2.18E-01
7.984009	-0.006	0.009	4.98E-01	0.013	0.014	3.29E-01
8.773953	-0.009	0.009	2.88E-01	0.016	0.013	2.15E-01
3.7712	0.003	0.008	7.03E-01	0.008	0.013	5.18E-01
5.794842	-0.007	0.009	4.54E-01	0.004	0.013	7.54E-01
7.815793	0.005	0.008	5.25E-01	-0.001	0.011	9.64E-01
8.624241	0.006	0.008	4.34E-01	-0.003	0.012	8.22E-01
3.277317	-0.013	0.012	2.82E-01	0.011	0.018	5.43E-01
4.045057	0.015	0.012	2.17E-01	-0.002	0.018	9.01E-01
2.720184977	-0.008	0.007	2.75E-01	-0.011	0.011	3.10E-01
6.193515	0.007	0.009	4.02E-01	-0.007	0.013	6.22E-01
3.217769	-0.012	0.014	4.03E-01	-0.006	0.021	7.89E-01
4.440701	-0.011	0.016	5.00E-01	0.024	0.025	3.29E-01

4.347509	-0.010	0.014	4.55E-01	0.014	0.020	4.91E-01
3.176388	-0.005	0.006	3.94E-01	0.008	0.008	3.56E-01
8.474192	-0.007	0.009	4.45E-01	-0.015	0.014	2.78E-01
1.981715439	0.005	0.006	3.64E-01	-0.004	0.009	6.48E-01
7.424185	-0.009	0.008	2.93E-01	0.007	0.012	5.79E-01
7.072613	-0.006	0.009	4.79E-01	-0.023	0.013	7.89E-02
0.955596036	-0.007	0.007	2.78E-01	0.012	0.010	2.13E-01
5.511903	-0.008	0.008	3.35E-01	-0.025	0.012	3.67E-02
5.52065	-0.005	0.007	4.24E-01	-0.002	0.010	8.11E-01
5.459419	0.005	0.007	4.87E-01	-0.010	0.011	3.78E-01
0.557932713	-0.005	0.009	5.53E-01	0.009	0.014	5.26E-01
3.911493	0.005	0.008	5.51E-01	0.028	0.011	1.47E-02
7.237802	-0.012	0.010	2.38E-01	-0.011	0.015	4.57E-01
2.65962711	-0.007	0.009	4.58E-01	0.008	0.014	5.82E-01
7.930517	0.011	0.010	2.62E-01	0.034	0.014	1.86E-02
8.381336	-0.008	0.009	3.30E-01	0.019	0.013	1.49E-01
8.730553	0.009	0.007	2.19E-01	-0.014	0.011	1.96E-01
6.431709	0.006	0.009	5.08E-01	0.017	0.014	2.34E-01
7.679201	0.006	0.009	4.94E-01	0.008	0.013	5.39E-01
5.987282	0.010	0.010	3.22E-01	0.004	0.015	8.02E-01
1.054843651	0.006	0.006	3.44E-01	-0.011	0.009	2.29E-01
1.768080744	-0.009	0.011	3.83E-01	-0.001	0.016	9.67E-01
6.201253	0.003	0.008	7.36E-01	-0.012	0.012	2.99E-01
6.37788	0.009	0.009	3.04E-01	0.008	0.013	5.37E-01
0.957614632	-0.005	0.005	3.21E-01	0.008	0.008	2.86E-01
8.911218	0.003	0.006	6.72E-01	-0.008	0.009	3.80E-01
0.512177881	-0.009	0.008	2.95E-01	0.002	0.013	8.59E-01
7.321237	-0.007	0.006	2.36E-01	0.000	0.009	9.73E-01
2.872925	-0.006	0.009	5.45E-01	0.002	0.014	9.04E-01
0.566343528	-0.004	0.010	6.61E-01	-0.013	0.015	3.67E-01
1.737128945	-0.004	0.005	4.55E-01	0.013	0.008	9.15E-02
5.990983	0.005	0.007	4.47E-01	-0.008	0.011	4.44E-01
7.143937	-0.006	0.008	4.66E-01	-0.004	0.012	7.16E-01
4.376106	-0.012	0.017	4.63E-01	0.014	0.025	5.82E-01
6.717004	-0.008	0.010	4.22E-01	-0.004	0.014	7.73E-01
6.090903	0.006	0.007	3.79E-01	-0.002	0.011	8.43E-01
6.211682	0.007	0.010	4.61E-01	0.008	0.015	5.81E-01
1.830320773	-0.009	0.010	3.75E-01	-0.008	0.016	6.30E-01
6.851914	-0.006	0.008	4.40E-01	-0.006	0.012	6.11E-01
6.059951	-0.006	0.008	4.28E-01	-0.002	0.012	8.78E-01
5.965077	-0.005	0.006	4.21E-01	-0.012	0.009	1.99E-01
1.737465378	-0.003	0.005	5.62E-01	0.012	0.008	1.31E-01
1.430638855	0.004	0.004	3.42E-01	0.005	0.007	4.08E-01

6.46939	-0.006	0.007	4.29E-01	-0.012	0.010	2.38E-01
6.840138	-0.007	0.009	4.42E-01	0.013	0.014	3.41E-01
3.941772	-0.006	0.008	4.61E-01	0.011	0.012	3.84E-01
7.770038	-0.014	0.013	2.69E-01	-0.023	0.019	2.34E-01
1.714587962	-0.004	0.005	4.19E-01	0.007	0.007	3.50E-01
5.797197	0.004	0.009	6.28E-01	-0.004	0.013	7.62E-01
0.634639344	0.005	0.009	5.20E-01	0.007	0.013	5.58E-01
8.87892	-0.005	0.009	5.85E-01	0.017	0.013	1.91E-01
5.003889	0.007	0.010	4.87E-01	0.002	0.015	8.92E-01
6.185441	0.006	0.010	5.53E-01	-0.002	0.014	9.07E-01
0.909504771	-0.004	0.006	4.71E-01	-0.024	0.008	3.80E-03
1.510036946	0.005	0.007	4.51E-01	-0.009	0.010	3.95E-01
7.11534	-0.006	0.010	5.58E-01	0.015	0.014	2.98E-01
6.71364	-0.007	0.010	4.58E-01	0.009	0.015	5.62E-01
2.720857842	-0.008	0.007	2.75E-01	-0.009	0.011	4.26E-01
7.133171	-0.007	0.009	4.33E-01	0.001	0.013	9.26E-01
8.273005	0.009	0.010	3.33E-01	-0.002	0.014	8.82E-01
3.803834	0.006	0.006	3.07E-01	0.010	0.008	2.18E-01
7.47566	-0.006	0.009	5.41E-01	-0.021	0.014	1.40E-01
8.795821	-0.005	0.008	5.03E-01	-0.007	0.012	5.75E-01
6.20058	0.006	0.008	4.77E-01	0.011	0.012	3.69E-01
2.513951798	-0.012	0.014	3.80E-01	-0.018	0.020	3.86E-01
4.003339	0.006	0.010	5.31E-01	0.017	0.014	2.21E-01
5.824785	0.009	0.010	3.80E-01	0.009	0.015	5.53E-01
8.0735	0.006	0.010	5.65E-01	0.014	0.015	3.45E-01
4.37476	-0.010	0.014	4.71E-01	0.019	0.021	3.57E-01
0.58989381	0.003	0.008	7.20E-01	0.013	0.011	2.56E-01
1.979696844	0.005	0.005	3.44E-01	-0.004	0.008	5.76E-01
7.928834	0.007	0.009	4.02E-01	0.001	0.013	9.24E-01
6.743246	-0.009	0.011	3.95E-01	-0.042	0.016	8.27E-03
1.508691216	0.005	0.007	4.30E-01	-0.007	0.010	4.55E-01
3.033067	0.005	0.008	4.99E-01	0.023	0.012	4.56E-02
1.44174113	0.004	0.004	3.09E-01	0.008	0.006	1.83E-01
6.026644	-0.007	0.008	4.15E-01	-0.018	0.012	1.34E-01
8.987251	0.003	0.007	6.36E-01	0.011	0.011	2.89E-01
5.04157	0.006	0.008	4.52E-01	0.004	0.012	7.48E-01
5.699296	0.007	0.009	4.01E-01	-0.016	0.013	2.05E-01
7.129134	0.011	0.011	2.98E-01	-0.006	0.016	6.94E-01
8.401186	0.005	0.008	5.27E-01	0.002	0.012	8.62E-01
5.881306	-0.007	0.007	3.10E-01	-0.012	0.010	2.55E-01
2.521353315	-0.012	0.013	3.71E-01	0.004	0.020	8.53E-01
7.302733	-0.011	0.011	3.22E-01	0.011	0.016	4.82E-01
2.165407634	0.009	0.010	3.64E-01	-0.014	0.015	3.73E-01

1.448806215	0.003	0.004	4.33E-01	0.001	0.006	8.70E-01
1.63384414	0.007	0.007	2.95E-01	0.012	0.010	2.47E-01
5.97618	0.007	0.008	4.10E-01	-0.015	0.012	2.44E-01
8.834174	-0.006	0.008	3.97E-01	-0.009	0.011	4.17E-01
3.70425	0.006	0.006	3.00E-01	0.023	0.008	5.46E-03
7.646231	-0.004	0.011	6.77E-01	-0.028	0.016	7.65E-02
7.418466	-0.007	0.009	4.43E-01	-0.006	0.013	6.36E-01
7.12106	-0.010	0.010	3.21E-01	-0.020	0.015	1.89E-01
3.203975	0.005	0.008	4.69E-01	0.018	0.011	1.06E-01
7.59543	0.008	0.009	3.35E-01	0.011	0.013	4.08E-01
1.511719109	0.006	0.007	4.32E-01	-0.010	0.011	3.57E-01
3.275635	-0.011	0.012	3.67E-01	0.013	0.017	4.49E-01
6.046494	0.006	0.009	5.17E-01	0.002	0.014	8.79E-01
3.195228	0.004	0.006	5.04E-01	0.015	0.008	7.61E-02
6.29209	0.007	0.010	4.86E-01	0.007	0.015	6.69E-01
2.832889895	0.011	0.011	3.47E-01	-0.012	0.017	5.00E-01
1.02355542	0.005	0.005	4.00E-01	0.006	0.008	4.38E-01
6.621457	-0.006	0.010	5.54E-01	0.002	0.014	9.05E-01
1.704831417	-0.003	0.004	4.62E-01	-0.002	0.007	8.18E-01
7.914704	-0.007	0.010	5.14E-01	-0.023	0.015	1.28E-01
6.395711	0.006	0.008	4.87E-01	0.000	0.012	9.83E-01
7.272454	-0.013	0.011	2.14E-01	0.005	0.016	7.47E-01
0.646750918	0.008	0.010	4.32E-01	-0.023	0.015	1.21E-01
4.010404	0.008	0.011	4.57E-01	0.019	0.017	2.45E-01
7.263371	-0.009	0.010	3.70E-01	0.014	0.014	3.24E-01
5.025085	0.007	0.010	4.72E-01	-0.014	0.015	3.55E-01
7.65195	0.008	0.007	2.39E-01	0.011	0.011	2.79E-01
6.13363	0.003	0.008	6.96E-01	-0.001	0.012	9.04E-01
5.117604	0.007	0.008	4.01E-01	-0.004	0.012	7.68E-01
3.693484	-0.003	0.005	5.35E-01	0.013	0.007	7.23E-02
5.240402	0.000	0.005	9.56E-01	0.005	0.007	4.91E-01
7.476333	-0.006	0.008	4.91E-01	-0.012	0.012	3.07E-01
4.010068	0.008	0.011	4.61E-01	0.017	0.017	3.27E-01
3.125586	-0.010	0.009	2.70E-01	0.005	0.014	7.18E-01
5.601057	0.007	0.008	4.34E-01	-0.005	0.012	6.78E-01
1.664459506	-0.003	0.004	5.02E-01	0.004	0.006	5.43E-01
4.393264	-0.009	0.013	4.81E-01	0.012	0.019	5.16E-01
5.907547	0.006	0.009	4.70E-01	-0.013	0.013	3.33E-01
1.692383411	-0.004	0.004	3.48E-01	0.005	0.006	3.57E-01
8.227923	-0.011	0.009	2.63E-01	-0.025	0.014	7.75E-02
7.146965	0.004	0.006	5.34E-01	0.002	0.009	8.42E-01
8.111517	0.005	0.010	5.80E-01	-0.014	0.014	3.25E-01
2.852739418	0.008	0.009	3.79E-01	-0.002	0.013	8.79E-01

2.719175679	-0.008	0.007	2.62E-01	-0.014	0.010	1.70E-01
7.452446	0.004	0.010	6.67E-01	0.014	0.014	3.14E-01
2.094083925	-0.005	0.007	4.90E-01	0.000	0.010	9.79E-01
6.544414	0.008	0.009	3.32E-01	-0.019	0.013	1.43E-01
8.210092	0.008	0.010	4.30E-01	-0.002	0.014	8.81E-01
7.505266	0.007	0.007	3.40E-01	-0.003	0.011	7.65E-01
5.97517	0.005	0.008	5.45E-01	-0.002	0.012	9.03E-01
8.494041	0.002	0.007	7.98E-01	0.004	0.011	7.26E-01
6.043466	0.006	0.007	4.21E-01	0.006	0.010	5.83E-01
7.800317	-0.007	0.008	4.15E-01	0.001	0.012	9.10E-01
8.473855	0.005	0.008	5.27E-01	-0.012	0.012	3.29E-01
2.100139711	-0.003	0.008	6.93E-01	0.000	0.012	9.95E-01
0.514196477	0.005	0.008	5.48E-01	-0.004	0.012	7.46E-01
8.213793	0.004	0.007	5.23E-01	0.010	0.010	3.32E-01
1.829311475	-0.010	0.011	3.44E-01	0.000	0.016	9.77E-01
6.540713	0.010	0.010	3.00E-01	0.025	0.015	1.00E-01
2.170790555	0.009	0.010	3.69E-01	-0.009	0.015	5.25E-01
2.039581845	0.005	0.004	3.01E-01	0.009	0.007	1.64E-01
8.353412	0.006	0.007	3.49E-01	-0.006	0.010	5.69E-01
1.093196966	0.007	0.007	3.47E-01	-0.006	0.011	5.54E-01
8.415652	0.007	0.008	4.26E-01	-0.005	0.013	6.98E-01
2.343716907	0.008	0.006	1.80E-01	0.004	0.009	6.64E-01
0.707645217	0.007	0.009	4.54E-01	0.008	0.013	5.68E-01
7.189019	-0.010	0.012	3.73E-01	-0.009	0.017	5.91E-01
5.739331	-0.007	0.010	4.91E-01	-0.007	0.015	6.44E-01
8.00184	0.007	0.008	4.05E-01	-0.007	0.012	5.32E-01
4.390236	-0.011	0.013	4.12E-01	0.015	0.020	4.48E-01
6.016888	0.004	0.007	5.49E-01	0.008	0.011	4.34E-01
5.594665	0.009	0.009	3.07E-01	-0.018	0.013	1.75E-01
5.042916	0.006	0.007	4.54E-01	-0.009	0.011	4.10E-01
2.282149743	-0.003	0.005	5.35E-01	-0.004	0.007	6.03E-01
6.141368	0.009	0.010	3.64E-01	-0.012	0.015	4.39E-01
2.526736237	-0.012	0.011	2.78E-01	0.000	0.016	9.88E-01
3.773555	0.008	0.011	4.63E-01	0.030	0.016	6.12E-02
6.237588	0.008	0.010	4.42E-01	-0.006	0.015	7.04E-01
7.328975	-0.005	0.006	4.43E-01	0.001	0.009	8.81E-01
5.007927	0.007	0.010	4.66E-01	-0.001	0.014	9.37E-01
6.880174	0.012	0.009	2.09E-01	-0.002	0.014	8.87E-01
3.978106	-0.007	0.011	5.23E-01	-0.008	0.016	6.08E-01
5.964404	-0.005	0.007	4.99E-01	-0.002	0.011	8.69E-01
4.411095	-0.011	0.015	4.55E-01	0.019	0.023	4.15E-01
7.655988	0.007	0.009	4.30E-01	-0.001	0.013	9.40E-01
5.698623	0.007	0.009	4.30E-01	-0.024	0.013	7.18E-02

7.596775	-0.004	0.010	6.99E-01	0.008	0.014	5.59E-01
5.095063	0.008	0.008	3.51E-01	-0.008	0.013	5.34E-01
5.479605	0.007	0.007	3.15E-01	-0.005	0.010	6.01E-01
4.305792	-0.011	0.014	4.28E-01	0.015	0.021	4.72E-01
4.176938	-0.007	0.009	4.30E-01	0.005	0.014	7.00E-01
5.074204	0.008	0.009	3.45E-01	-0.011	0.013	3.97E-01
5.753461	0.006	0.010	5.56E-01	-0.023	0.016	1.47E-01
7.275482	-0.011	0.009	2.24E-01	0.005	0.013	6.77E-01
6.750984	-0.005	0.011	6.23E-01	-0.010	0.016	5.30E-01
7.72361	-0.007	0.009	4.68E-01	0.001	0.014	9.19E-01
5.536126	0.006	0.007	4.37E-01	0.003	0.011	7.77E-01
8.710031	0.002	0.008	8.28E-01	-0.019	0.012	1.11E-01
4.398984	-0.010	0.015	5.04E-01	0.005	0.022	8.22E-01
1.853534622	-0.003	0.008	7.46E-01	0.006	0.012	6.16E-01
5.75077	0.004	0.010	6.95E-01	0.006	0.015	6.88E-01
3.788022	0.013	0.010	1.81E-01	0.022	0.015	1.42E-01
5.647149	0.008	0.008	2.76E-01	-0.004	0.012	7.27E-01
7.708135	-0.006	0.011	5.70E-01	-0.008	0.016	6.17E-01
3.372191	-0.006	0.011	5.80E-01	-0.004	0.017	8.00E-01
8.76386	0.008	0.010	4.50E-01	0.009	0.015	5.47E-01
8.385037	0.008	0.009	3.89E-01	-0.018	0.013	1.67E-01
2.662991436	-0.020	0.019	3.06E-01	-0.013	0.029	6.61E-01
6.060961	0.006	0.008	4.96E-01	-0.020	0.012	1.14E-01
4.102923	-0.004	0.010	7.11E-01	0.007	0.016	6.31E-01
7.550684	0.007	0.009	4.33E-01	0.007	0.014	6.26E-01
2.361211402	0.011	0.007	1.36E-01	0.015	0.011	1.82E-01
3.050898	-0.009	0.009	3.23E-01	0.014	0.014	3.11E-01
1.464618547	-0.002	0.005	6.09E-01	0.004	0.007	5.98E-01
5.785422	0.005	0.011	6.75E-01	0.004	0.016	8.02E-01
8.063744	0.007	0.009	4.66E-01	0.019	0.014	1.54E-01
0.818331539	0.002	0.006	6.60E-01	0.014	0.008	9.38E-02
5.636719	0.007	0.009	4.08E-01	-0.008	0.013	5.13E-01
3.801479	0.005	0.006	3.58E-01	0.010	0.008	2.33E-01
5.883324	0.005	0.007	4.61E-01	0.015	0.010	1.59E-01
2.917671	-0.006	0.009	5.24E-01	-0.002	0.014	8.88E-01
8.59127	-0.006	0.008	4.47E-01	0.022	0.013	7.75E-02
5.657241	0.006	0.009	4.92E-01	-0.025	0.014	6.95E-02
8.735936	0.005	0.009	5.81E-01	0.003	0.013	7.89E-01
8.475201	-0.006	0.009	5.30E-01	0.004	0.014	7.61E-01
5.244439	-0.007	0.005	1.23E-01	-0.005	0.007	5.17E-01
8.84595	0.008	0.010	4.25E-01	-0.005	0.015	7.17E-01
7.814447	-0.008	0.010	4.04E-01	0.010	0.014	4.71E-01
2.679140201	-0.008	0.006	1.99E-01	0.005	0.009	5.41E-01

5.441588	0.007	0.008	4.19E-01	-0.007	0.012	5.70E-01
7.708807	-0.005	0.010	6.04E-01	-0.006	0.014	6.95E-01
7.636811	-0.004	0.009	6.35E-01	0.017	0.014	2.22E-01
8.686144	0.004	0.006	4.64E-01	0.007	0.008	3.89E-01
4.107969	-0.007	0.011	5.35E-01	0.007	0.016	6.60E-01
1.523157817	0.008	0.009	3.90E-01	-0.018	0.014	1.88E-01
0.604023978	0.003	0.006	6.86E-01	-0.023	0.010	1.71E-02
1.499943969	-0.004	0.005	3.83E-01	-0.009	0.007	2.02E-01
7.398953	0.011	0.009	2.54E-01	-0.006	0.014	6.72E-01
7.598121	0.011	0.010	2.75E-01	0.007	0.014	6.07E-01
2.677458038	-0.008	0.006	2.28E-01	0.014	0.010	1.38E-01
7.444035	0.007	0.008	4.03E-01	0.015	0.012	2.06E-01
4.370387	-0.009	0.012	4.44E-01	0.016	0.018	3.79E-01
2.185257157	0.010	0.011	3.39E-01	-0.028	0.016	8.28E-02
7.588701	-0.003	0.009	7.42E-01	0.004	0.014	7.42E-01
8.415316	-0.004	0.008	6.35E-01	0.010	0.013	4.49E-01
8.410606	-0.005	0.009	5.30E-01	0.002	0.013	9.05E-01
5.633018	-0.008	0.010	4.50E-01	-0.018	0.016	2.61E-01
7.577935	-0.005	0.009	5.66E-01	-0.004	0.014	7.64E-01
4.134884	-0.011	0.015	4.48E-01	0.008	0.022	7.29E-01
6.745264	-0.007	0.007	3.40E-01	-0.009	0.011	4.13E-01
7.386169	0.006	0.009	5.20E-01	-0.011	0.013	3.86E-01
7.113658	-0.008	0.010	4.01E-01	0.010	0.014	4.90E-01
5.905529	0.007	0.008	3.81E-01	-0.012	0.011	2.80E-01
7.762973	-0.007	0.012	5.28E-01	-0.021	0.017	2.27E-01
5.679782	0.001	0.010	8.94E-01	-0.010	0.015	5.28E-01
6.378889	-0.005	0.010	5.97E-01	-0.007	0.015	6.45E-01
1.087477612	0.006	0.007	4.00E-01	0.008	0.011	4.55E-01
7.377758	-0.005	0.009	5.68E-01	-0.009	0.014	5.44E-01
5.244102	-0.007	0.005	1.21E-01	-0.003	0.007	6.37E-01
7.46624	0.006	0.007	4.32E-01	-0.009	0.011	4.33E-01
7.549675	-0.006	0.007	4.17E-01	0.007	0.011	5.41E-01
5.535789	0.008	0.009	3.53E-01	-0.013	0.013	3.21E-01
7.726302	-0.007	0.009	4.59E-01	-0.011	0.014	4.06E-01
5.013982	0.009	0.012	4.46E-01	-0.010	0.018	5.64E-01
5.901828	-0.008	0.007	2.86E-01	-0.020	0.011	5.76E-02
6.631887	0.000	0.009	9.69E-01	0.002	0.014	9.12E-01
3.178743	-0.004	0.005	4.51E-01	0.015	0.008	5.09E-02
7.787196	-0.006	0.010	5.65E-01	-0.013	0.015	3.82E-01
3.335184	0.009	0.009	3.46E-01	0.014	0.014	2.95E-01
8.178804	-0.007	0.007	3.68E-01	-0.014	0.011	2.22E-01
6.532639	0.004	0.008	6.05E-01	0.014	0.012	2.61E-01
8.426755	-0.007	0.009	4.65E-01	0.001	0.014	9.67E-01

7.787533	-0.004	0.007	5.86E-01	-0.012	0.010	2.55E-01
0.685104233	0.011	0.013	4.06E-01	0.000	0.020	9.98E-01
6.712294	-0.005	0.008	5.05E-01	0.001	0.012	9.34E-01
6.287043	-0.003	0.009	7.16E-01	-0.034	0.013	1.16E-02
3.314325	-0.009	0.010	3.95E-01	0.009	0.015	5.37E-01
2.60243357	-0.012	0.015	4.16E-01	0.013	0.022	5.77E-01
5.066129	0.006	0.007	4.26E-01	0.000	0.011	9.77E-01
6.074418	0.009	0.009	2.78E-01	-0.001	0.013	9.43E-01
4.091484	0.014	0.012	2.59E-01	0.016	0.018	3.83E-01
6.005113	0.007	0.007	3.30E-01	0.006	0.010	5.52E-01
1.794995351	-0.006	0.009	5.08E-01	0.016	0.014	2.47E-01
6.86167	0.009	0.011	3.85E-01	0.007	0.016	6.62E-01
6.14305	-0.006	0.009	5.00E-01	-0.006	0.013	6.64E-01
7.837325	-0.008	0.009	3.86E-01	0.017	0.013	2.13E-01
8.188897	-0.007	0.008	3.90E-01	0.010	0.012	3.94E-01
2.595032053	0.017	0.020	3.75E-01	-0.004	0.029	8.82E-01
5.954984	0.006	0.007	4.47E-01	-0.023	0.011	4.24E-02
3.946482	-0.006	0.007	4.30E-01	0.002	0.011	8.34E-01
4.19275	-0.008	0.010	4.36E-01	0.008	0.015	6.12E-01
8.440212	0.007	0.010	4.95E-01	-0.008	0.015	5.99E-01
6.369133	0.007	0.009	4.16E-01	-0.019	0.013	1.59E-01
1.980033276	0.005	0.005	3.54E-01	-0.004	0.008	6.16E-01
6.841148	0.007	0.009	4.12E-01	0.019	0.013	1.45E-01
8.596653	0.007	0.010	4.43E-01	0.015	0.014	2.85E-01
8.398158	0.007	0.009	4.49E-01	-0.001	0.014	9.31E-01
3.745968	-0.009	0.010	3.48E-01	-0.002	0.015	8.70E-01
8.033128	0.006	0.007	3.71E-01	0.002	0.011	8.44E-01
8.454006	0.009	0.014	5.15E-01	0.000	0.021	9.89E-01
3.517867	0.006	0.007	4.03E-01	0.022	0.010	2.95E-02
4.190059	-0.007	0.013	5.86E-01	0.008	0.020	6.84E-01
4.39461	-0.011	0.014	4.07E-01	0.015	0.020	4.51E-01
3.217432	-0.011	0.014	4.20E-01	-0.005	0.021	8.05E-01
2.286523367	-0.009	0.010	4.00E-01	0.002	0.016	8.99E-01
1.669842427	0.002	0.004	5.70E-01	0.002	0.006	6.81E-01
6.182413	-0.006	0.010	5.63E-01	-0.008	0.015	5.98E-01
8.818699	0.002	0.008	7.54E-01	0.014	0.011	2.22E-01
7.83497	-0.006	0.010	5.23E-01	0.016	0.015	2.92E-01
5.614515	0.003	0.008	7.09E-01	-0.004	0.011	7.13E-01
4.387208	-0.009	0.013	4.97E-01	0.013	0.019	5.17E-01
5.116594	0.007	0.008	3.88E-01	-0.001	0.012	9.26E-01
4.333379	-0.011	0.014	4.35E-01	0.021	0.021	3.15E-01
7.858184	0.003	0.009	7.19E-01	0.004	0.013	7.55E-01
3.630572	-0.003	0.004	4.59E-01	0.007	0.006	2.63E-01

1.234835087	0.005	0.008	4.82E-01	-0.007	0.011	5.21E-01
8.096378	0.005	0.009	5.74E-01	-0.018	0.014	1.84E-01
1.517438463	0.007	0.008	4.33E-01	-0.015	0.013	2.38E-01
3.051571	-0.008	0.010	3.89E-01	0.015	0.014	2.84E-01
7.117023	-0.007	0.010	4.75E-01	0.000	0.015	9.94E-01
2.794200147	-0.010	0.012	3.87E-01	-0.057	0.018	1.53E-03
8.502788	-0.006	0.008	4.11E-01	0.023	0.012	4.48E-02
4.348519	-0.011	0.013	4.03E-01	0.010	0.020	6.21E-01
0.664918277	-0.006	0.008	4.86E-01	-0.029	0.012	1.62E-02
7.46994	0.006	0.009	4.88E-01	-0.011	0.013	4.07E-01
7.612251	-0.011	0.011	3.43E-01	-0.030	0.017	7.03E-02
6.692781	-0.008	0.008	2.94E-01	0.001	0.012	9.33E-01
5.77432	0.006	0.007	4.25E-01	0.003	0.011	7.57E-01
2.34405334	0.007	0.006	2.54E-01	0.010	0.009	2.55E-01
0.771567409	0.005	0.005	3.25E-01	-0.008	0.007	2.92E-01
8.319769	-0.004	0.009	6.32E-01	-0.015	0.014	2.70E-01
7.401308	-0.006	0.010	5.32E-01	-0.012	0.014	4.06E-01
7.486089	0.009	0.009	3.33E-01	-0.007	0.014	6.02E-01
7.896537	-0.006	0.009	5.10E-01	0.003	0.014	8.42E-01
3.736211	-0.013	0.009	1.55E-01	0.017	0.014	2.29E-01
4.044047	0.014	0.013	2.82E-01	-0.002	0.019	9.19E-01
6.543069	-0.008	0.009	4.24E-01	-0.024	0.014	9.33E-02
1.881458527	-0.005	0.007	4.91E-01	-0.004	0.011	7.37E-01
7.030223	-0.006	0.009	4.73E-01	-0.014	0.013	2.75E-01
7.469604	0.009	0.009	3.56E-01	0.003	0.014	8.12E-01
8.406905	0.009	0.010	3.57E-01	-0.003	0.015	8.58E-01
5.691894	0.009	0.010	4.03E-01	-0.005	0.015	7.61E-01
7.362955	-0.007	0.009	4.43E-01	-0.006	0.014	6.69E-01
6.810869	-0.013	0.011	2.68E-01	0.013	0.017	4.49E-01
1.685318326	-0.004	0.004	3.17E-01	0.008	0.006	1.55E-01
1.025910448	0.004	0.006	4.77E-01	0.012	0.009	2.00E-01
2.790162956	-0.009	0.012	4.42E-01	-0.052	0.018	3.16E-03
1.415835821	0.005	0.004	2.48E-01	0.005	0.006	4.27E-01
6.002421	0.006	0.009	4.64E-01	0.002	0.013	8.58E-01
3.55622	-0.019	0.024	4.49E-01	-0.035	0.036	3.36E-01
5.768601	0.004	0.009	6.38E-01	-0.004	0.014	7.54E-01
8.501106	0.004	0.008	6.08E-01	-0.015	0.012	2.11E-01
6.306556	0.008	0.009	3.98E-01	0.012	0.014	3.82E-01
2.357510644	0.009	0.007	2.17E-01	0.013	0.011	2.26E-01
6.447522	0.009	0.010	3.93E-01	-0.029	0.015	5.48E-02
2.838609249	-0.004	0.009	6.23E-01	-0.012	0.013	3.74E-01
8.888677	-0.006	0.009	4.89E-01	-0.008	0.013	5.35E-01
2.341698312	0.008	0.007	2.36E-01	-0.004	0.010	7.10E-01

5.648158	0.005	0.006	3.77E-01	-0.009	0.009	3.52E-01
4.305455	-0.011	0.014	4.34E-01	0.013	0.021	5.29E-01
2.789153658	-0.009	0.011	4.39E-01	-0.048	0.017	4.92E-03
1.009088818	0.008	0.008	3.27E-01	0.024	0.012	4.37E-02
5.034168	0.007	0.011	5.28E-01	0.011	0.017	5.19E-01
7.37473	-0.006	0.009	4.97E-01	-0.001	0.013	9.12E-01
5.601394	0.010	0.012	3.73E-01	-0.004	0.017	8.03E-01
0.644059457	0.004	0.008	5.81E-01	0.000	0.012	9.80E-01
8.66865	-0.004	0.006	5.15E-01	0.003	0.010	7.83E-01
5.138126	-0.007	0.008	3.69E-01	-0.002	0.011	8.43E-01
8.681434	-0.005	0.006	4.27E-01	0.004	0.009	6.48E-01
0.52630805	0.011	0.009	2.17E-01	-0.026	0.013	4.45E-02
8.976149	0.008	0.010	4.38E-01	-0.004	0.015	7.70E-01
8.938132	-0.007	0.008	3.84E-01	-0.004	0.012	7.65E-01
8.067445	0.008	0.008	3.04E-01	0.014	0.012	2.31E-01
1.864300465	-0.004	0.008	6.00E-01	0.000	0.013	9.72E-01
0.987557132	0.004	0.006	4.73E-01	0.011	0.009	1.84E-01
7.592402	0.005	0.009	5.84E-01	0.005	0.013	7.11E-01
6.686052	-0.005	0.010	5.97E-01	-0.006	0.015	6.92E-01
7.303743	-0.009	0.008	2.74E-01	-0.002	0.012	8.79E-01
8.955963	0.007	0.008	3.57E-01	0.001	0.011	9.65E-01
6.288725	0.005	0.008	5.61E-01	0.003	0.012	8.21E-01
8.536768	-0.005	0.008	5.63E-01	-0.003	0.013	7.82E-01
8.225568	0.006	0.007	3.81E-01	-0.001	0.011	9.46E-01
7.544292	-0.006	0.010	5.32E-01	0.007	0.015	6.58E-01
2.720521409	-0.008	0.007	2.84E-01	-0.009	0.011	3.92E-01
0.705626621	0.007	0.009	4.88E-01	-0.003	0.014	8.13E-01
8.853015	-0.011	0.009	2.29E-01	-0.002	0.013	8.92E-01
3.184798	0.006	0.006	3.13E-01	0.018	0.009	4.62E-02
2.173482016	0.009	0.010	3.79E-01	-0.015	0.015	2.94E-01
7.930853	0.008	0.009	3.75E-01	-0.007	0.014	5.86E-01
1.508354783	0.005	0.007	4.55E-01	-0.005	0.010	5.80E-01
1.44813335	0.003	0.004	4.18E-01	0.005	0.006	4.42E-01
7.549002	-0.007	0.010	4.82E-01	-0.007	0.014	6.53E-01
7.46052	0.010	0.011	3.51E-01	-0.009	0.016	5.53E-01
8.805914	-0.005	0.009	6.19E-01	-0.002	0.014	8.76E-01
8.04053	-0.005	0.007	4.46E-01	-0.016	0.011	1.25E-01
3.688438	-0.004	0.004	4.24E-01	0.003	0.007	6.01E-01
6.62213	-0.006	0.009	4.88E-01	0.002	0.013	8.92E-01
7.344451	-0.007	0.010	4.73E-01	0.006	0.015	6.88E-01
7.906966	0.006	0.008	4.02E-01	-0.004	0.011	7.09E-01
5.120632	0.006	0.007	4.04E-01	-0.005	0.011	6.84E-01
3.919231	-0.004	0.010	7.21E-01	0.026	0.016	8.89E-02

8.778663	-0.004	0.007	4.90E-01	-0.007	0.010	4.89E-01
5.700978	0.008	0.010	4.10E-01	-0.004	0.014	7.71E-01
3.702905	0.004	0.005	4.24E-01	0.021	0.008	6.97E-03
5.488689	0.006	0.008	4.57E-01	-0.020	0.012	1.15E-01
1.456880597	0.002	0.004	5.77E-01	0.003	0.005	5.73E-01
6.950152	-0.006	0.009	4.63E-01	-0.004	0.013	7.37E-01
8.333563	-0.004	0.009	6.29E-01	-0.003	0.013	8.45E-01
4.153388	-0.008	0.013	5.26E-01	0.017	0.019	3.73E-01
8.594971	-0.004	0.007	5.74E-01	0.003	0.010	7.61E-01
1.832002936	-0.012	0.010	2.57E-01	-0.013	0.015	4.05E-01
5.652531	-0.007	0.010	4.71E-01	-0.019	0.015	2.11E-01
5.319127	0.013	0.017	4.16E-01	-0.033	0.025	1.76E-01
7.252605	0.007	0.009	4.40E-01	0.018	0.014	1.89E-01
5.063438	0.008	0.010	4.20E-01	-0.009	0.015	5.27E-01
7.789215	-0.010	0.010	3.25E-01	-0.006	0.016	7.14E-01
5.020038	0.006	0.011	5.53E-01	-0.012	0.016	4.59E-01
6.28738	-0.005	0.009	5.67E-01	-0.015	0.014	2.70E-01
8.518264	0.010	0.008	2.16E-01	0.010	0.012	4.09E-01
0.55490482	0.007	0.009	4.28E-01	-0.008	0.013	5.52E-01
4.13623	-0.008	0.012	5.02E-01	0.014	0.019	4.56E-01
0.585520186	0.008	0.009	3.63E-01	-0.009	0.013	4.81E-01
0.517560803	-0.005	0.008	4.70E-01	-0.001	0.011	9.60E-01
3.595919	-0.007	0.007	3.49E-01	0.017	0.011	1.04E-01
5.724528	0.005	0.010	5.91E-01	-0.012	0.015	4.33E-01
2.67779447	-0.008	0.006	2.16E-01	0.010	0.009	2.94E-01
1.512391975	0.006	0.008	3.95E-01	-0.013	0.011	2.50E-01
2.093747492	-0.005	0.006	4.52E-01	0.001	0.009	9.33E-01
6.184431	0.004	0.007	5.85E-01	0.011	0.010	2.82E-01
1.224405677	0.005	0.008	4.96E-01	-0.003	0.012	8.06E-01
7.262025	-0.015	0.010	1.65E-01	0.010	0.016	5.13E-01
7.272118	-0.013	0.011	2.40E-01	0.014	0.016	3.94E-01
8.562673	0.004	0.006	5.13E-01	0.016	0.009	8.45E-02
4.403357	-0.011	0.015	4.69E-01	-0.002	0.022	9.42E-01
5.427794	0.007	0.007	3.28E-01	-0.012	0.010	2.46E-01
7.136872	0.011	0.010	2.81E-01	-0.017	0.015	2.34E-01
1.45990849	0.003	0.004	4.82E-01	0.002	0.005	7.08E-01
6.319677	0.006	0.008	4.76E-01	0.007	0.012	5.66E-01
4.014441	-0.010	0.012	4.07E-01	0.011	0.018	5.53E-01
8.395803	0.005	0.010	5.78E-01	-0.009	0.014	5.17E-01
7.313499	-0.010	0.008	1.99E-01	0.016	0.012	1.72E-01
8.964037	-0.006	0.008	4.83E-01	-0.002	0.012	8.49E-01
6.070044	0.008	0.009	4.01E-01	0.012	0.014	4.06E-01
4.362312	-0.011	0.014	4.38E-01	0.010	0.021	6.18E-01

5.880969	-0.006	0.007	4.30E-01	-0.014	0.011	2.05E-01
1.974986787	0.004	0.005	4.54E-01	-0.004	0.007	6.14E-01
0.647760215	-0.006	0.010	5.80E-01	-0.012	0.015	4.04E-01
5.963395	0.006	0.010	5.27E-01	-0.012	0.015	4.31E-01
7.900238	-0.005	0.008	5.62E-01	0.000	0.013	9.99E-01
4.360294	-0.009	0.013	4.69E-01	0.004	0.019	8.43E-01
8.585214	0.007	0.010	5.11E-01	0.016	0.015	2.85E-01
7.985355	0.005	0.008	5.31E-01	0.018	0.012	1.31E-01
8.449632	-0.007	0.009	4.28E-01	-0.009	0.013	4.93E-01
3.266215	-0.006	0.007	4.28E-01	-0.005	0.011	6.12E-01
5.144518	0.006	0.009	4.93E-01	-0.014	0.014	3.00E-01
1.52349425	0.008	0.009	3.89E-01	-0.018	0.014	1.96E-01
5.03686	0.008	0.010	4.60E-01	-0.020	0.015	1.84E-01
2.611853682	-0.011	0.013	3.89E-01	0.035	0.020	7.70E-02
1.43265745	0.005	0.004	2.46E-01	0.001	0.007	9.32E-01
2.873262	-0.006	0.009	5.39E-01	0.002	0.014	8.66E-01
6.668558	0.006	0.006	3.19E-01	-0.013	0.010	1.87E-01
4.213609	0.010	0.013	4.24E-01	0.015	0.019	4.38E-01
6.116808	-0.006	0.009	4.85E-01	0.011	0.014	4.29E-01
0.537073893	0.007	0.009	4.31E-01	0.028	0.013	3.35E-02
5.920668	-0.007	0.008	3.45E-01	-0.010	0.012	4.05E-01
8.39984	0.004	0.007	5.86E-01	0.001	0.011	9.25E-01
7.300042	-0.008	0.008	3.48E-01	0.009	0.012	4.28E-01
1.649320039	0.005	0.005	3.32E-01	0.014	0.007	6.79E-02
0.803192072	0.002	0.006	7.64E-01	0.007	0.009	4.10E-01
8.746366	0.008	0.007	2.55E-01	0.003	0.010	7.37E-01
7.910331	-0.010	0.010	3.31E-01	-0.009	0.015	5.31E-01
7.2738	-0.010	0.009	2.79E-01	-0.003	0.014	8.23E-01
0.600659653	0.007	0.007	3.51E-01	0.003	0.011	7.95E-01
8.292518	0.009	0.009	3.48E-01	-0.017	0.014	2.08E-01
0.576772939	0.007	0.010	4.47E-01	0.027	0.014	5.68E-02
7.920424	-0.007	0.009	4.16E-01	-0.006	0.013	6.35E-01
1.685654759	-0.004	0.004	3.52E-01	0.007	0.006	2.56E-01
6.775543	0.008	0.010	4.29E-01	0.007	0.015	6.20E-01
3.126932	-0.011	0.010	2.49E-01	0.014	0.014	3.30E-01
5.000862	0.008	0.011	4.63E-01	-0.006	0.016	7.18E-01
5.919659	-0.008	0.008	3.07E-01	0.008	0.012	5.32E-01
1.02624688	0.004	0.007	5.33E-01	0.014	0.010	1.75E-01
7.458838	0.007	0.009	4.50E-01	0.010	0.013	4.64E-01
1.711223636	-0.003	0.004	4.46E-01	0.004	0.006	4.73E-01
3.185135	0.006	0.006	3.32E-01	0.018	0.009	5.83E-02
5.770956	0.004	0.008	6.31E-01	-0.013	0.011	2.58E-01
6.301846	0.006	0.007	3.57E-01	-0.006	0.010	5.25E-01

1.717615855	-0.004	0.005	4.36E-01	-0.002	0.008	7.55E-01
3.263524	0.006	0.008	4.27E-01	0.023	0.011	3.99E-02
6.993552	0.005	0.007	4.96E-01	0.005	0.011	6.69E-01
7.194402	0.007	0.008	3.82E-01	0.000	0.012	9.96E-01
6.65611	-0.007	0.009	4.25E-01	0.010	0.013	4.59E-01
7.356899	-0.010	0.010	3.23E-01	-0.011	0.015	4.94E-01
6.753002	-0.007	0.010	4.93E-01	-0.016	0.015	2.84E-01
7.595766	0.009	0.010	3.74E-01	0.011	0.015	4.40E-01
7.467922	0.005	0.009	5.41E-01	-0.003	0.013	8.24E-01
7.295332	-0.004	0.010	6.84E-01	0.007	0.016	6.64E-01
6.642316	0.006	0.009	5.37E-01	-0.004	0.014	7.76E-01
8.3376	0.006	0.007	4.21E-01	-0.008	0.010	4.13E-01
8.701956	0.004	0.007	5.63E-01	-0.005	0.011	6.55E-01
2.406966234	-0.016	0.020	4.11E-01	0.008	0.029	7.85E-01
7.598794	0.009	0.009	3.33E-01	0.013	0.014	3.26E-01
7.389869	0.006	0.008	4.17E-01	-0.004	0.012	7.33E-01
6.989851	0.006	0.007	3.63E-01	0.004	0.010	7.26E-01
6.63626	0.004	0.007	5.43E-01	-0.006	0.011	5.68E-01
3.572705	-0.003	0.006	6.06E-01	0.010	0.008	2.49E-01
7.628064	0.004	0.008	6.21E-01	0.013	0.011	2.32E-01
6.816925	0.005	0.010	6.18E-01	0.010	0.015	4.73E-01
5.297931	0.011	0.013	4.13E-01	-0.022	0.019	2.53E-01
7.583655	0.009	0.011	4.06E-01	-0.006	0.016	7.24E-01
1.728381698	-0.003	0.005	4.74E-01	0.004	0.007	6.19E-01
0.83279814	-0.008	0.008	3.06E-01	-0.003	0.012	7.79E-01
3.914184	-0.006	0.008	5.02E-01	0.025	0.013	4.62E-02
4.108306	-0.006	0.010	5.67E-01	0.005	0.015	7.39E-01
1.856226083	-0.005	0.008	5.36E-01	-0.017	0.012	1.59E-01
2.58426621	-0.001	0.011	9.00E-01	0.006	0.017	7.17E-01
4.308147	-0.012	0.015	4.06E-01	0.006	0.022	8.00E-01
4.424889	-0.010	0.014	4.54E-01	0.008	0.021	6.99E-01
0.520925128	-0.009	0.009	2.94E-01	0.004	0.013	7.59E-01
8.559645	-0.005	0.008	5.43E-01	-0.007	0.012	5.63E-01
4.342463	-0.009	0.012	4.87E-01	0.013	0.018	4.96E-01
6.172993	-0.003	0.007	6.45E-01	-0.018	0.010	6.27E-02
8.686481	0.004	0.005	4.71E-01	0.004	0.008	6.57E-01
0.983519941	0.006	0.006	3.07E-01	0.010	0.009	2.68E-01
1.513401272	0.007	0.008	3.79E-01	-0.012	0.011	3.12E-01
5.156293	0.008	0.008	3.38E-01	-0.008	0.012	5.29E-01
7.209205	0.004	0.007	6.03E-01	0.004	0.010	7.18E-01
6.707584	-0.008	0.009	3.64E-01	-0.010	0.014	4.80E-01
2.53884781	-0.012	0.014	3.79E-01	0.007	0.020	7.17E-01
7.985019	0.007	0.008	3.83E-01	-0.001	0.012	9.43E-01

7.840016	-0.004	0.007	5.08E-01	0.009	0.010	3.48E-01
0.753063616	-0.005	0.005	4.01E-01	-0.005	0.008	5.63E-01
8.677397	0.007	0.009	4.24E-01	0.007	0.014	6.03E-01
5.02172	0.007	0.011	5.46E-01	0.001	0.016	9.74E-01
4.166172	-0.010	0.014	4.73E-01	0.003	0.021	8.99E-01
5.94691	0.006	0.009	4.55E-01	-0.008	0.013	5.26E-01
8.564692	-0.012	0.009	1.97E-01	-0.014	0.013	3.04E-01
2.616227306	-0.010	0.014	4.66E-01	0.025	0.020	2.22E-01
6.625831	0.005	0.009	5.74E-01	-0.014	0.013	2.96E-01
8.000494	-0.008	0.010	4.28E-01	0.001	0.014	9.48E-01
2.667028627	-0.006	0.005	2.75E-01	0.014	0.008	7.39E-02
0.639685833	-0.008	0.009	4.04E-01	-0.009	0.014	5.11E-01
2.507896012	-0.010	0.013	4.48E-01	-0.016	0.019	3.96E-01
1.725017372	-0.004	0.005	4.22E-01	0.007	0.007	3.38E-01
3.535698	0.007	0.008	3.88E-01	0.018	0.012	1.50E-01
8.025391	0.005	0.009	5.67E-01	-0.012	0.013	3.42E-01
5.975843	0.006	0.009	4.91E-01	-0.029	0.014	3.01E-02
8.228932	-0.006	0.008	4.49E-01	-0.017	0.012	1.44E-01
6.274259	-0.004	0.007	5.82E-01	-0.013	0.010	2.22E-01
7.77273	-0.009	0.012	4.53E-01	-0.009	0.017	5.94E-01
8.890022	0.010	0.010	3.44E-01	0.010	0.015	5.13E-01
2.667701493	-0.005	0.005	2.86E-01	0.013	0.007	7.80E-02
2.777378517	-0.010	0.012	3.94E-01	-0.045	0.017	8.36E-03
7.095154	0.007	0.010	4.82E-01	-0.021	0.015	1.68E-01
5.872558	0.008	0.008	3.27E-01	-0.022	0.012	7.29E-02
1.726699535	-0.003	0.005	4.98E-01	0.006	0.007	4.03E-01
8.421372	0.005	0.007	4.65E-01	0.001	0.011	9.57E-01
8.910881	0.002	0.006	7.68E-01	-0.003	0.009	7.79E-01
2.42715219	-0.007	0.008	4.27E-01	0.016	0.012	1.97E-01
1.758997064	-0.005	0.008	5.89E-01	0.015	0.013	2.26E-01
0.641704429	0.008	0.010	4.44E-01	-0.004	0.015	7.72E-01
3.264196	0.005	0.008	4.90E-01	0.015	0.011	1.87E-01
8.766552	0.006	0.009	5.14E-01	0.007	0.013	5.82E-01
7.389533	0.007	0.007	3.33E-01	-0.001	0.010	9.26E-01
2.518325422	0.021	0.029	4.56E-01	0.002	0.043	9.56E-01
8.762851	0.003	0.009	7.57E-01	0.002	0.014	8.76E-01
8.18486	-0.010	0.010	3.15E-01	-0.039	0.015	8.15E-03
8.882957	0.009	0.010	3.74E-01	0.006	0.015	6.78E-01
5.320472	0.013	0.017	4.28E-01	-0.032	0.025	1.96E-01
1.857908246	-0.005	0.008	5.53E-01	0.007	0.012	5.60E-01
6.183422	0.003	0.008	6.88E-01	-0.007	0.012	5.56E-01
5.441925	0.006	0.007	3.79E-01	-0.010	0.010	3.37E-01
6.285361	0.009	0.010	4.09E-01	0.003	0.015	8.24E-01

4.424216	-0.010	0.015	4.98E-01	0.008	0.022	7.27E-01
6.89565	0.007	0.010	4.86E-01	-0.009	0.015	5.69E-01
8.41397	-0.008	0.009	4.09E-01	-0.019	0.014	1.83E-01
3.434095	0.008	0.011	4.99E-01	0.031	0.017	6.55E-02
6.249363	0.009	0.009	3.45E-01	0.006	0.014	6.86E-01
2.099130414	-0.003	0.009	7.31E-01	-0.005	0.013	6.98E-01
0.61714485	-0.006	0.008	4.78E-01	0.020	0.013	1.17E-01
8.381673	0.005	0.008	5.04E-01	0.020	0.011	7.78E-02
5.067475	0.006	0.008	4.68E-01	-0.010	0.012	4.06E-01
1.234498654	0.005	0.008	5.00E-01	-0.008	0.011	5.11E-01
8.806587	0.008	0.008	2.93E-01	-0.007	0.011	5.16E-01
1.499607536	-0.004	0.005	3.81E-01	-0.010	0.007	1.70E-01
0.949876682	0.005	0.005	3.72E-01	-0.001	0.008	9.41E-01
6.001412	0.008	0.009	3.64E-01	0.002	0.013	8.79E-01
6.623812	-0.007	0.010	5.03E-01	-0.009	0.015	5.43E-01
8.524656	0.004	0.007	6.27E-01	0.004	0.011	7.26E-01
8.95058	-0.008	0.008	3.71E-01	-0.028	0.013	2.75E-02
3.639992	0.006	0.005	2.43E-01	0.012	0.007	9.71E-02
5.571788	-0.004	0.008	6.12E-01	-0.014	0.011	2.06E-01
7.195748	0.009	0.008	2.89E-01	-0.007	0.013	5.87E-01
7.752207	-0.005	0.010	6.15E-01	-0.001	0.014	9.40E-01
6.936358	0.006	0.010	5.05E-01	0.006	0.014	6.91E-01
1.731746024	-0.006	0.005	2.70E-01	0.002	0.008	8.02E-01
6.763095	0.009	0.010	3.61E-01	0.002	0.015	8.83E-01
3.681373	-0.002	0.005	6.16E-01	0.014	0.007	4.32E-02
4.387545	-0.010	0.014	5.03E-01	0.015	0.021	4.71E-01
3.310624	-0.014	0.014	3.17E-01	0.017	0.021	4.17E-01
4.153051	-0.009	0.014	5.40E-01	0.019	0.021	3.68E-01
2.284841204	-0.005	0.006	4.53E-01	-0.004	0.009	6.35E-01
7.932872	-0.003	0.008	6.84E-01	-0.022	0.013	8.12E-02
5.483642	0.007	0.008	3.78E-01	0.014	0.012	2.62E-01
1.741502569	-0.006	0.006	3.09E-01	0.010	0.009	2.75E-01
2.917334	-0.006	0.009	4.87E-01	-0.003	0.013	8.20E-01
3.233918	-0.004	0.006	4.60E-01	0.009	0.009	3.07E-01
8.19899	0.006	0.008	4.63E-01	0.001	0.011	9.63E-01
5.094726	0.008	0.009	3.60E-01	-0.009	0.013	4.87E-01
0.973426964	0.003	0.005	5.72E-01	0.019	0.007	1.20E-02
8.759486	0.005	0.009	5.84E-01	-0.003	0.013	8.43E-01
6.230859	0.004	0.010	6.57E-01	-0.010	0.015	4.85E-01
5.576498	0.005	0.007	5.20E-01	-0.004	0.011	7.05E-01
3.860355	-0.005	0.005	3.69E-01	0.007	0.008	3.55E-01
5.911585	0.005	0.009	5.46E-01	-0.011	0.013	4.15E-01
1.117420113	0.006	0.007	3.47E-01	-0.005	0.010	5.97E-01

5.027776	0.007	0.010	4.67E-01	-0.011	0.014	4.51E-01
2.859132	-0.006	0.008	4.57E-01	-0.011	0.012	3.71E-01
1.745203328	-0.005	0.006	4.55E-01	0.003	0.009	7.66E-01
6.064325	-0.008	0.008	3.53E-01	-0.025	0.012	4.23E-02
2.590658429	-0.007	0.017	6.74E-01	-0.004	0.026	8.84E-01
1.444769024	0.003	0.004	3.98E-01	0.004	0.006	4.45E-01
0.908831906	-0.004	0.006	5.14E-01	-0.023	0.008	6.68E-03
6.390328	-0.007	0.009	4.70E-01	-0.003	0.014	8.02E-01
5.153938	0.006	0.008	4.47E-01	-0.016	0.013	1.97E-01
1.722662344	-0.004	0.005	3.91E-01	-0.004	0.007	5.77E-01
7.542273	0.001	0.010	8.94E-01	0.000	0.015	9.92E-01
4.369041	-0.009	0.015	5.50E-01	0.016	0.022	4.60E-01
7.460857	0.009	0.011	3.94E-01	-0.017	0.016	3.00E-01
1.717952288	-0.004	0.005	3.93E-01	-0.004	0.008	6.55E-01
4.241197	0.011	0.013	4.06E-01	-0.010	0.020	5.95E-01
6.661829	0.008	0.011	4.51E-01	-0.010	0.016	5.55E-01
7.099864	-0.007	0.010	4.83E-01	-0.013	0.014	3.62E-01
3.164612	-0.004	0.007	5.11E-01	0.006	0.010	5.43E-01
8.400513	0.005	0.008	5.59E-01	0.018	0.012	1.37E-01
6.510435	0.006	0.010	5.48E-01	-0.003	0.015	8.30E-01
5.623935	0.006	0.008	4.85E-01	0.005	0.013	7.12E-01
8.668313	-0.005	0.007	4.37E-01	-0.003	0.010	7.76E-01
0.514532909	0.004	0.008	6.37E-01	0.004	0.013	7.67E-01
5.400543	0.004	0.006	4.69E-01	-0.020	0.009	2.39E-02
5.782731	0.007	0.009	4.39E-01	-0.017	0.014	2.12E-01
1.790621727	-0.003	0.011	7.43E-01	0.012	0.016	4.60E-01
8.561664	0.000	0.009	9.87E-01	0.017	0.013	1.83E-01
3.373537	0.009	0.012	4.61E-01	-0.010	0.017	5.54E-01
8.996671	0.007	0.009	4.33E-01	0.009	0.013	5.08E-01
7.816129	0.007	0.008	4.07E-01	0.007	0.012	5.62E-01
8.093686	-0.006	0.009	4.86E-01	-0.016	0.013	2.24E-01
7.689967	-0.007	0.008	3.84E-01	0.013	0.012	2.48E-01
6.841484	0.006	0.009	4.95E-01	0.022	0.013	8.68E-02
6.423635	-0.007	0.009	4.19E-01	-0.003	0.014	8.26E-01
2.678467335	-0.008	0.006	2.23E-01	0.008	0.009	3.73E-01
2.288541962	-0.003	0.005	5.87E-01	-0.003	0.007	6.87E-01
8.000831	-0.005	0.009	5.72E-01	0.004	0.014	7.75E-01
5.772974	0.004	0.008	5.96E-01	-0.010	0.011	4.05E-01
8.932749	-0.003	0.007	6.59E-01	-0.004	0.011	7.12E-01
7.305088	-0.009	0.012	4.62E-01	-0.013	0.018	4.43E-01
7.509639	-0.006	0.008	4.61E-01	0.011	0.011	3.41E-01
1.975659653	0.004	0.005	4.61E-01	-0.004	0.007	5.83E-01
1.135587472	0.007	0.007	3.06E-01	-0.018	0.010	7.16E-02

8.910545	0.004	0.008	5.96E-01	0.005	0.012	6.79E-01
7.918741	-0.006	0.009	4.84E-01	-0.022	0.013	9.85E-02
6.682015	0.005	0.009	5.98E-01	-0.021	0.014	1.39E-01
6.568301	0.004	0.008	6.31E-01	-0.004	0.012	7.66E-01
4.190395	-0.008	0.014	5.65E-01	0.015	0.021	4.90E-01
7.409719	-0.008	0.008	3.10E-01	-0.013	0.011	2.51E-01
7.123078	0.006	0.007	4.23E-01	-0.001	0.011	9.61E-01
8.798849	0.003	0.007	6.38E-01	0.005	0.010	6.54E-01
4.400329	-0.009	0.012	4.46E-01	0.019	0.018	2.89E-01
5.406263	0.005	0.007	5.09E-01	-0.003	0.010	7.33E-01
5.026094	0.006	0.009	4.86E-01	-0.007	0.013	5.72E-01
2.754501101	-0.008	0.007	2.77E-01	-0.019	0.011	7.77E-02
7.135863	0.008	0.009	4.12E-01	-0.014	0.014	3.26E-01
1.449142647	0.003	0.004	4.54E-01	0.002	0.006	7.65E-01
6.423971	-0.006	0.008	4.55E-01	0.002	0.011	8.54E-01
8.997344	0.006	0.009	4.66E-01	0.002	0.013	8.83E-01
6.806495	0.005	0.008	5.26E-01	-0.014	0.012	2.55E-01
5.810655	0.005	0.008	4.90E-01	-0.012	0.012	2.94E-01
6.643662	0.004	0.009	6.14E-01	-0.003	0.013	8.00E-01
5.06983	0.004	0.007	5.14E-01	-0.008	0.010	4.50E-01
7.405009	-0.008	0.009	3.55E-01	0.006	0.013	6.38E-01
1.506336188	0.004	0.006	4.83E-01	-0.011	0.009	2.30E-01
5.911248	0.004	0.007	5.16E-01	-0.010	0.010	3.45E-01
8.100079	0.004	0.008	6.02E-01	-0.008	0.012	4.91E-01
6.995234	0.008	0.008	3.17E-01	-0.002	0.011	8.32E-01
5.636383	0.007	0.009	4.32E-01	-0.010	0.013	4.60E-01
6.987496	-0.005	0.006	4.64E-01	0.002	0.009	8.54E-01
2.668037925	-0.005	0.005	2.71E-01	0.010	0.007	1.43E-01
5.067139	0.006	0.008	4.14E-01	-0.008	0.012	4.97E-01
3.374883	0.009	0.013	4.81E-01	-0.013	0.019	4.94E-01
7.412074	-0.008	0.009	4.01E-01	-0.005	0.013	7.05E-01
6.562918	-0.003	0.009	7.39E-01	-0.014	0.014	3.08E-01
8.094359	0.008	0.009	3.46E-01	-0.028	0.013	3.27E-02
0.705290188	0.007	0.009	4.31E-01	-0.002	0.013	9.06E-01
3.930669	0.007	0.008	3.53E-01	0.022	0.011	4.99E-02
3.210704	-0.013	0.014	3.55E-01	-0.001	0.021	9.78E-01
2.039245412	0.004	0.004	3.24E-01	0.009	0.007	1.59E-01
7.088089	0.007	0.010	5.14E-01	0.002	0.015	9.01E-01
6.239943	0.006	0.007	3.43E-01	0.016	0.010	9.60E-02
8.14146	-0.004	0.008	6.35E-01	0.005	0.012	7.08E-01
5.974497	-0.005	0.010	6.29E-01	0.003	0.014	8.07E-01
8.541478	-0.007	0.009	4.61E-01	0.011	0.014	4.01E-01
1.417854416	0.005	0.005	3.11E-01	0.002	0.007	8.00E-01

7.326956	-0.007	0.007	3.26E-01	0.000	0.010	9.69E-01
2.664673599	-0.008	0.009	3.43E-01	-0.010	0.013	4.17E-01
2.792854416	-0.010	0.012	4.29E-01	-0.060	0.018	1.18E-03
8.137423	0.007	0.009	4.35E-01	-0.014	0.014	3.26E-01
6.465353	0.007	0.010	4.95E-01	-0.008	0.015	5.76E-01
8.791448	-0.008	0.010	4.14E-01	-0.012	0.014	3.96E-01
5.244775	-0.007	0.005	1.47E-01	-0.005	0.007	4.57E-01
8.261903	0.006	0.007	3.57E-01	0.013	0.010	1.88E-01
4.009731	0.007	0.011	5.22E-01	0.012	0.016	4.67E-01
6.979758	-0.009	0.010	3.91E-01	-0.018	0.015	2.35E-01
5.809309	0.003	0.007	6.52E-01	-0.004	0.011	7.11E-01
4.215628	-0.008	0.013	5.03E-01	-0.002	0.019	8.97E-01
3.789368	0.011	0.009	2.13E-01	0.020	0.013	1.36E-01
4.317567	-0.010	0.014	4.64E-01	0.028	0.021	1.88E-01
4.338426	-0.008	0.013	5.11E-01	0.008	0.019	6.78E-01
4.34179	-0.009	0.013	5.00E-01	0.015	0.019	4.31E-01
8.210765	-0.008	0.010	4.41E-01	-0.014	0.015	3.70E-01
1.234162222	0.005	0.008	5.08E-01	-0.007	0.012	5.22E-01
2.668374358	-0.005	0.005	2.61E-01	0.011	0.007	1.16E-01
8.724497	-0.006	0.010	5.83E-01	-0.012	0.016	4.41E-01
2.776369219	-0.010	0.011	3.77E-01	-0.045	0.017	8.37E-03
0.532700269	-0.009	0.009	2.96E-01	-0.004	0.013	7.71E-01
6.594879	0.004	0.007	4.99E-01	-0.011	0.010	2.58E-01
3.702232	0.004	0.005	4.46E-01	0.019	0.008	1.65E-02
8.230278	0.004	0.009	6.77E-01	0.001	0.013	9.59E-01
4.346837	-0.010	0.015	5.22E-01	0.024	0.022	2.82E-01
8.531049	-0.005	0.010	6.41E-01	-0.004	0.015	8.12E-01
1.667823832	-0.004	0.004	2.90E-01	0.003	0.006	6.56E-01
3.692139	-0.003	0.005	5.82E-01	0.009	0.007	1.96E-01
2.508232444	-0.010	0.014	4.81E-01	-0.021	0.021	3.19E-01
5.787441	0.007	0.009	3.97E-01	0.010	0.013	4.14E-01
7.079678	0.003	0.009	7.02E-01	0.013	0.013	3.38E-01
4.30882	-0.011	0.014	4.36E-01	0.011	0.021	6.01E-01
8.036493	-0.009	0.010	3.65E-01	0.022	0.015	1.31E-01
6.894304	-0.006	0.010	5.59E-01	-0.004	0.014	7.91E-01
6.56931	-0.009	0.009	3.38E-01	0.003	0.014	8.19E-01
0.818667972	0.002	0.006	6.91E-01	0.013	0.008	1.05E-01
0.677702716	0.007	0.011	5.11E-01	-0.007	0.016	6.63E-01
5.575825	0.007	0.009	4.76E-01	-0.028	0.014	4.41E-02
4.020161	-0.009	0.013	4.81E-01	0.007	0.019	6.98E-01
6.266521	0.009	0.009	3.13E-01	-0.005	0.014	7.26E-01
6.263157	0.007	0.007	3.19E-01	0.006	0.010	5.46E-01
6.676969	0.008	0.009	3.74E-01	0.024	0.013	5.74E-02

5.243766	-0.007	0.005	1.40E-01	-0.002	0.007	7.32E-01
0.655498165	-0.009	0.011	4.36E-01	-0.013	0.017	4.54E-01
3.651094	-0.003	0.004	4.92E-01	0.009	0.007	1.67E-01
1.523830683	0.008	0.009	3.92E-01	-0.016	0.014	2.54E-01
1.741839002	-0.006	0.006	3.05E-01	0.004	0.009	6.46E-01
8.241044	-0.005	0.010	5.76E-01	-0.005	0.014	7.35E-01
8.100415	-0.006	0.009	5.13E-01	-0.011	0.013	4.18E-01
1.975996085	0.004	0.005	4.61E-01	-0.004	0.007	6.24E-01
6.445839	0.008	0.010	3.92E-01	-0.022	0.014	1.20E-01
6.976057	-0.007	0.010	4.86E-01	-0.004	0.015	7.87E-01
4.298727	-0.009	0.013	5.05E-01	0.012	0.020	5.42E-01
4.353565	-0.009	0.015	5.27E-01	0.000	0.022	9.96E-01
5.768937	0.004	0.010	6.81E-01	0.010	0.015	5.06E-01
7.204159	-0.004	0.007	5.07E-01	0.005	0.010	5.87E-01
7.853137	-0.009	0.010	3.85E-01	0.019	0.015	1.98E-01
5.728229	0.007	0.011	5.42E-01	-0.020	0.016	2.24E-01
3.123904	-0.008	0.008	2.99E-01	0.001	0.012	9.24E-01
5.116931	0.007	0.008	4.08E-01	-0.012	0.013	3.59E-01
8.297228	0.006	0.007	4.41E-01	-0.003	0.011	7.99E-01
0.853320529	-0.006	0.007	4.13E-01	-0.012	0.010	2.55E-01
0.775268167	0.004	0.005	3.38E-01	0.001	0.007	8.68E-01
2.569463176	-0.009	0.009	3.28E-01	0.005	0.013	7.19E-01
2.600078542	-0.010	0.016	5.41E-01	0.019	0.024	4.34E-01
0.906476878	0.005	0.006	4.49E-01	-0.009	0.009	3.15E-01
1.775482261	-0.007	0.011	5.05E-01	-0.016	0.017	3.40E-01
5.966087	-0.006	0.010	5.56E-01	-0.002	0.014	8.81E-01
7.588365	-0.004	0.010	6.74E-01	-0.005	0.015	7.35E-01
8.629623	-0.006	0.006	3.33E-01	0.007	0.009	4.37E-01
6.783954	-0.004	0.006	5.13E-01	-0.015	0.010	1.26E-01
0.832461708	-0.008	0.008	3.16E-01	-0.003	0.012	8.04E-01
7.834297	-0.005	0.011	6.10E-01	0.010	0.016	5.38E-01
3.995264	-0.007	0.009	4.20E-01	0.012	0.013	3.70E-01
8.480247	-0.005	0.008	5.96E-01	0.003	0.013	7.91E-01
5.906202	-0.007	0.009	4.56E-01	-0.015	0.014	2.60E-01
2.542212136	0.027	0.036	4.54E-01	0.020	0.053	7.05E-01
1.915438219	-0.005	0.006	3.61E-01	-0.003	0.009	7.47E-01
7.385496	-0.007	0.008	3.91E-01	-0.002	0.012	8.51E-01
2.361884267	0.010	0.007	1.72E-01	0.016	0.011	1.28E-01
7.832278	-0.005	0.010	6.18E-01	0.017	0.015	2.49E-01
1.170240029	0.006	0.005	2.03E-01	-0.011	0.008	1.34E-01
1.055852948	0.005	0.006	3.79E-01	-0.007	0.009	4.19E-01
1.446114754	0.004	0.004	3.34E-01	0.002	0.006	7.09E-01
5.760526	0.005	0.007	5.35E-01	-0.001	0.011	9.55E-01

8.274687	0.005	0.006	4.00E-01	0.006	0.009	5.32E-01
5.512239	-0.008	0.008	3.48E-01	-0.025	0.012	4.53E-02
3.031722	-0.003	0.005	5.27E-01	0.005	0.008	5.39E-01
7.936236	-0.004	0.009	6.39E-01	-0.001	0.013	9.63E-01
6.380235	0.008	0.009	4.03E-01	0.012	0.013	3.68E-01
7.387514	0.005	0.008	5.26E-01	-0.005	0.012	6.63E-01
5.91865	0.004	0.010	6.89E-01	-0.003	0.015	8.20E-01
0.803864938	0.002	0.006	7.86E-01	0.009	0.008	3.08E-01
2.596714216	-0.013	0.019	4.82E-01	0.001	0.028	9.83E-01
6.695472	0.008	0.010	4.17E-01	0.001	0.015	9.41E-01
4.300409	-0.010	0.014	5.02E-01	0.015	0.022	4.93E-01
7.405345	-0.010	0.010	3.47E-01	0.001	0.015	9.29E-01
8.382682	0.011	0.009	2.28E-01	-0.004	0.013	7.82E-01
6.080474	0.010	0.010	3.23E-01	0.014	0.015	3.41E-01
2.283159041	-0.003	0.005	5.11E-01	-0.014	0.007	6.45E-02
5.579526	0.008	0.010	4.38E-01	-0.027	0.014	5.97E-02
6.345583	0.006	0.009	4.81E-01	0.022	0.013	9.58E-02
8.072828	-0.007	0.011	4.97E-01	-0.001	0.016	9.70E-01
1.316251774	0.012	0.009	2.00E-01	0.003	0.014	8.28E-01
7.886444	-0.004	0.009	6.57E-01	0.021	0.014	1.12E-01
1.224742109	0.005	0.008	5.18E-01	-0.003	0.012	7.72E-01
2.587966968	0.009	0.008	2.49E-01	0.011	0.012	3.76E-01
7.184982	0.009	0.008	2.69E-01	-0.006	0.012	6.59E-01
0.791416932	-0.005	0.005	2.96E-01	0.002	0.007	7.47E-01
4.091821	0.013	0.012	2.93E-01	0.023	0.018	2.09E-01
8.322797	-0.008	0.009	3.79E-01	0.011	0.014	4.13E-01
7.931526	0.006	0.009	4.66E-01	0.003	0.013	7.99E-01
3.167977	-0.005	0.006	4.24E-01	0.006	0.009	5.34E-01
7.540255	0.010	0.009	2.62E-01	-0.013	0.013	3.15E-01
8.807933	0.004	0.007	6.18E-01	-0.022	0.011	3.61E-02
6.985814	-0.006	0.008	4.69E-01	-0.010	0.012	3.73E-01
8.032119	-0.007	0.009	3.90E-01	-0.019	0.013	1.41E-01
2.788817225	-0.009	0.012	4.58E-01	-0.046	0.017	7.91E-03
7.70006	-0.006	0.007	3.76E-01	0.013	0.011	2.33E-01
8.656202	0.004	0.007	5.39E-01	-0.012	0.011	2.77E-01
2.346744801	0.010	0.008	2.37E-01	0.017	0.012	1.76E-01
0.95996966	0.004	0.005	4.28E-01	0.012	0.007	1.11E-01
6.402776	0.007	0.009	4.68E-01	-0.015	0.013	2.73E-01
8.44223	-0.007	0.010	4.64E-01	-0.009	0.015	5.27E-01
2.574173232	-0.004	0.009	6.12E-01	-0.001	0.013	9.23E-01
8.315059	0.004	0.008	6.39E-01	-0.026	0.012	3.99E-02
6.084847	0.005	0.007	4.59E-01	0.010	0.011	3.74E-01
5.801571	0.005	0.010	5.74E-01	-0.005	0.014	7.47E-01

1.784902373	-0.006	0.011	6.04E-01	0.017	0.016	2.87E-01
0.63665794	0.004	0.008	5.92E-01	0.000	0.011	9.96E-01
7.577262	0.007	0.009	4.85E-01	-0.003	0.014	8.06E-01
7.956422	0.004	0.009	6.84E-01	0.003	0.013	8.22E-01
6.3402	0.007	0.009	4.00E-01	0.007	0.013	5.60E-01
8.495723	-0.005	0.006	3.39E-01	-0.018	0.009	3.46E-02
0.832125275	-0.008	0.008	3.17E-01	-0.003	0.012	8.26E-01
1.979360411	0.004	0.005	3.76E-01	-0.005	0.007	5.35E-01
6.980767	-0.007	0.010	5.00E-01	-0.007	0.015	6.14E-01
5.659933	0.005	0.009	5.91E-01	-0.008	0.013	5.26E-01
8.225231	0.006	0.008	4.17E-01	-0.011	0.012	3.68E-01
5.320136	0.013	0.017	4.41E-01	-0.031	0.025	2.14E-01
8.973794	0.002	0.007	7.69E-01	0.016	0.011	1.58E-01
3.801816	0.005	0.006	3.58E-01	0.010	0.008	2.34E-01
2.559706631	0.004	0.008	6.30E-01	0.003	0.012	8.00E-01
1.68060827	-0.003	0.004	3.94E-01	0.004	0.006	4.56E-01
8.154581	0.007	0.009	4.46E-01	-0.013	0.013	3.29E-01
6.396047	0.003	0.009	7.43E-01	-0.007	0.014	6.30E-01
8.995999	0.011	0.010	2.48E-01	0.031	0.014	2.98E-02
3.564294	0.005	0.006	4.16E-01	0.017	0.009	6.53E-02
7.122742	0.006	0.009	4.98E-01	-0.004	0.013	7.89E-01
3.177733	-0.004	0.006	4.71E-01	0.012	0.008	1.47E-01
1.973641057	0.003	0.005	4.82E-01	-0.005	0.007	4.90E-01
6.079801	0.008	0.008	3.44E-01	0.005	0.012	7.01E-01
8.813316	0.007	0.010	4.84E-01	-0.011	0.014	4.57E-01
0.715719599	0.005	0.008	4.85E-01	-0.011	0.012	3.50E-01
6.245999	-0.005	0.010	6.17E-01	-0.011	0.015	4.72E-01
0.906140445	0.005	0.006	4.47E-01	-0.008	0.009	3.71E-01
8.070809	-0.005	0.008	5.14E-01	0.011	0.012	3.60E-01
1.092524101	0.006	0.007	4.01E-01	-0.007	0.011	5.25E-01
5.321482	0.013	0.017	4.53E-01	-0.033	0.025	1.91E-01
5.068821	0.006	0.007	4.11E-01	-0.006	0.011	5.86E-01
3.284382	-0.007	0.010	5.11E-01	-0.003	0.016	8.64E-01
8.913573	0.002	0.008	8.47E-01	0.005	0.012	6.98E-01
1.74553976	-0.004	0.006	5.08E-01	0.007	0.009	4.48E-01
7.458165	0.005	0.009	5.51E-01	0.009	0.013	4.96E-01
2.86216	-0.006	0.008	4.53E-01	-0.007	0.012	5.46E-01
0.529335943	0.008	0.009	4.17E-01	0.004	0.014	7.66E-01
7.576253	0.006	0.007	4.19E-01	-0.018	0.011	9.69E-02
2.173145584	0.007	0.010	4.38E-01	-0.016	0.014	2.65E-01
6.012514	0.007	0.009	4.27E-01	0.022	0.013	8.06E-02
8.911554	0.003	0.010	7.40E-01	-0.005	0.014	7.11E-01
8.610783	-0.008	0.008	3.41E-01	-0.007	0.012	5.74E-01

3.569677	-0.005	0.007	4.81E-01	0.008	0.011	4.76E-01
0.995967947	0.004	0.006	5.23E-01	0.010	0.009	2.85E-01
6.139686	0.007	0.009	4.83E-01	-0.001	0.014	9.24E-01
6.169965	-0.010	0.011	3.73E-01	0.000	0.016	9.79E-01
5.523678	0.005	0.007	4.50E-01	-0.016	0.010	1.01E-01
2.328577441	-0.002	0.005	6.60E-01	-0.012	0.008	1.04E-01
3.734866	-0.010	0.009	2.28E-01	0.001	0.013	9.12E-01
6.280315	-0.004	0.008	6.56E-01	-0.007	0.012	5.41E-01
8.298237	0.005	0.007	4.69E-01	-0.005	0.010	6.17E-01
6.716668	-0.007	0.009	4.47E-01	-0.011	0.013	3.77E-01
5.024075	0.006	0.008	4.72E-01	-0.014	0.012	2.71E-01
7.421158	0.004	0.007	5.46E-01	-0.011	0.011	2.78E-01
5.658251	0.005	0.009	5.53E-01	-0.012	0.013	3.38E-01
8.269641	0.005	0.011	6.32E-01	-0.036	0.016	2.47E-02
4.175929	-0.008	0.010	4.02E-01	0.015	0.014	2.83E-01
1.509700514	0.005	0.007	4.72E-01	-0.012	0.010	2.54E-01
6.990187	0.007	0.007	3.32E-01	0.010	0.010	3.34E-01
5.790469	0.005	0.008	5.69E-01	-0.009	0.012	4.46E-01
2.72186714	-0.008	0.008	3.27E-01	-0.006	0.012	6.42E-01
1.873047712	-0.006	0.007	4.51E-01	0.005	0.011	6.26E-01
1.13525104	0.007	0.007	2.86E-01	-0.019	0.010	5.31E-02
5.556648	-0.005	0.009	5.88E-01	-0.025	0.014	6.17E-02
7.621335	-0.011	0.010	2.45E-01	0.008	0.014	5.81E-01
5.436878	0.006	0.009	4.67E-01	-0.004	0.013	7.74E-01
5.100782	0.006	0.008	5.01E-01	-0.003	0.012	8.39E-01
5.414001	0.005	0.007	4.90E-01	-0.001	0.010	8.88E-01
4.337416	-0.009	0.016	5.77E-01	0.011	0.023	6.35E-01
5.762208	0.005	0.008	5.17E-01	-0.018	0.013	1.62E-01
5.673727	0.006	0.008	4.56E-01	-0.015	0.012	2.07E-01
0.762820161	-0.002	0.005	6.64E-01	-0.013	0.008	9.80E-02
7.819157	-0.007	0.010	4.85E-01	0.005	0.015	7.51E-01
4.352556	-0.011	0.014	4.36E-01	0.023	0.021	2.60E-01
4.156079	-0.010	0.015	5.13E-01	0.019	0.023	4.08E-01
6.749638	-0.008	0.012	5.30E-01	0.016	0.018	3.60E-01
6.372497	0.004	0.007	5.70E-01	0.001	0.011	9.33E-01
4.391582	-0.009	0.013	4.77E-01	0.005	0.019	7.88E-01
5.011291	0.006	0.008	4.85E-01	-0.007	0.012	5.88E-01
6.399075	0.004	0.008	5.94E-01	-0.011	0.012	3.85E-01
4.347846	-0.009	0.013	5.04E-01	0.008	0.020	7.01E-01
2.065823587	0.004	0.004	2.39E-01	0.012	0.006	3.64E-02
1.068973819	0.004	0.007	5.56E-01	-0.005	0.010	6.30E-01
1.979023978	0.004	0.005	3.85E-01	-0.004	0.007	6.07E-01
0.716728897	-0.006	0.008	4.13E-01	-0.005	0.011	6.48E-01

8.499088	-0.005	0.007	4.55E-01	0.000	0.010	9.90E-01
1.977341816	0.004	0.005	4.46E-01	-0.003	0.007	6.62E-01
8.35173	-0.004	0.007	5.61E-01	-0.005	0.010	6.37E-01
8.57243	-0.005	0.010	5.96E-01	0.025	0.015	9.67E-02
1.507009053	0.004	0.006	4.92E-01	-0.010	0.009	3.00E-01
7.652287	0.008	0.007	2.68E-01	0.013	0.011	2.45E-01
8.233642	0.004	0.010	6.67E-01	-0.020	0.015	1.73E-01
1.437031074	0.004	0.004	3.23E-01	0.004	0.006	5.54E-01
8.144824	0.007	0.009	4.09E-01	0.016	0.013	2.31E-01
5.094053	0.006	0.008	4.64E-01	-0.010	0.012	3.93E-01
7.091454	-0.009	0.010	3.90E-01	-0.004	0.015	8.07E-01
2.802274529	-0.009	0.013	4.97E-01	-0.064	0.020	1.48E-03
5.901492	-0.009	0.008	2.60E-01	-0.026	0.012	2.50E-02
6.694127	0.005	0.008	4.81E-01	0.002	0.011	8.60E-01
1.466973575	-0.004	0.007	6.16E-01	0.003	0.011	7.57E-01
6.685716	0.005	0.008	5.15E-01	-0.004	0.012	7.11E-01
2.669383655	-0.005	0.005	2.78E-01	0.008	0.007	2.79E-01
3.773892	0.008	0.010	4.61E-01	0.031	0.015	4.66E-02
5.795515	-0.007	0.008	3.71E-01	-0.006	0.012	6.14E-01
3.564631	0.005	0.006	4.07E-01	0.018	0.009	5.21E-02
2.778051382	-0.009	0.012	4.23E-01	-0.045	0.017	9.67E-03
7.195411	0.008	0.008	3.28E-01	-0.008	0.012	5.04E-01
8.293527	-0.008	0.007	2.86E-01	-0.014	0.011	2.11E-01
8.862771	0.007	0.009	4.52E-01	0.001	0.014	9.17E-01
7.171188	-0.007	0.009	4.56E-01	0.013	0.014	3.52E-01
6.305547	0.004	0.007	5.52E-01	-0.006	0.010	5.63E-01
2.61151725	-0.011	0.013	4.22E-01	0.032	0.020	1.09E-01
4.323286	-0.010	0.014	4.62E-01	0.020	0.021	3.47E-01
7.87265	0.006	0.008	4.63E-01	-0.010	0.012	3.92E-01
6.709266	-0.006	0.010	5.22E-01	-0.023	0.015	1.11E-01
7.836315	-0.006	0.009	5.36E-01	0.016	0.013	2.28E-01
4.388218	-0.008	0.012	5.33E-01	0.020	0.019	2.92E-01
0.948867384	0.004	0.005	4.15E-01	-0.002	0.008	8.29E-01
6.770161	-0.002	0.008	8.40E-01	-0.004	0.012	7.61E-01
7.537563	0.004	0.008	6.47E-01	-0.009	0.013	4.56E-01
3.115157	-0.009	0.009	3.27E-01	0.008	0.014	5.39E-01
6.270895	-0.005	0.008	5.42E-01	-0.002	0.011	8.72E-01
8.978504	0.005	0.009	6.09E-01	-0.008	0.014	5.43E-01
7.479697	0.007	0.009	4.59E-01	-0.009	0.013	5.01E-01
2.335306092	0.008	0.008	3.16E-01	0.007	0.011	5.20E-01
8.166019	0.007	0.008	4.11E-01	-0.007	0.012	5.82E-01
0.858030585	0.007	0.009	4.12E-01	-0.005	0.013	6.72E-01
3.498017	0.004	0.009	6.95E-01	0.033	0.013	1.47E-02

6.119163	-0.006	0.008	4.74E-01	0.008	0.012	5.37E-01
6.284015	-0.002	0.009	8.01E-01	0.002	0.014	9.15E-01
0.995631515	0.004	0.006	5.61E-01	0.010	0.010	2.77E-01
5.319463	0.013	0.017	4.46E-01	-0.033	0.025	1.85E-01
7.471286	0.004	0.006	4.67E-01	-0.004	0.009	6.31E-01
3.27799	-0.008	0.011	4.61E-01	0.015	0.017	3.60E-01
2.647179104	-0.012	0.015	4.40E-01	0.028	0.022	2.13E-01
2.855767	-0.003	0.007	6.40E-01	-0.012	0.011	2.62E-01
2.830534867	0.011	0.012	3.73E-01	-0.033	0.018	6.25E-02
8.267622	-0.005	0.007	4.15E-01	-0.001	0.010	8.80E-01
0.946848789	-0.004	0.005	4.48E-01	-0.006	0.008	4.61E-01
5.070839	0.004	0.007	5.85E-01	-0.004	0.010	7.23E-01
4.342126	-0.007	0.012	5.42E-01	0.018	0.018	3.30E-01
3.642347	-0.002	0.004	5.22E-01	0.008	0.005	1.51E-01
2.67510301	-0.008	0.008	3.09E-01	0.001	0.012	9.31E-01
8.88363	0.007	0.009	4.07E-01	-0.005	0.013	6.92E-01
5.62158	0.004	0.008	5.95E-01	0.004	0.012	7.42E-01
1.813835576	-0.005	0.010	6.40E-01	-0.004	0.016	7.81E-01
8.718778	-0.005	0.009	5.82E-01	0.015	0.014	2.84E-01
2.185930022	0.009	0.011	3.95E-01	-0.024	0.016	1.52E-01
7.987037	0.005	0.010	5.82E-01	-0.018	0.014	2.06E-01
2.356837778	0.000	0.007	9.82E-01	0.015	0.011	1.52E-01
3.204311	0.005	0.008	5.18E-01	0.018	0.012	1.27E-01
3.934034	-0.003	0.006	6.63E-01	0.012	0.009	2.20E-01
6.424308	0.006	0.008	4.85E-01	0.009	0.012	4.68E-01
5.513248	0.003	0.009	7.21E-01	-0.006	0.013	6.34E-01
6.346592	0.007	0.007	3.33E-01	0.001	0.010	9.44E-01
7.206514	0.004	0.007	5.84E-01	-0.004	0.011	7.10E-01
6.926938	-0.012	0.012	3.31E-01	0.009	0.018	6.30E-01
2.636749694	-0.006	0.009	5.25E-01	-0.007	0.013	6.13E-01
8.976485	0.007	0.009	4.06E-01	-0.006	0.013	6.47E-01
0.50814069	-0.007	0.009	4.61E-01	-0.009	0.014	5.00E-01
8.491013	0.005	0.007	5.27E-01	-0.001	0.011	9.34E-01
7.10121	0.007	0.007	3.08E-01	0.008	0.010	4.45E-01
7.785851	-0.004	0.006	5.13E-01	0.003	0.010	7.66E-01
8.332217	-0.004	0.007	5.74E-01	-0.008	0.010	4.39E-01
4.109988	-0.004	0.010	6.47E-01	0.005	0.014	7.39E-01
6.711621	-0.007	0.008	3.27E-01	0.003	0.011	8.04E-01
5.669017	0.004	0.007	5.24E-01	-0.002	0.010	8.44E-01
5.623598	0.006	0.009	5.29E-01	-0.015	0.014	3.01E-01
8.548543	-0.007	0.009	4.02E-01	-0.024	0.013	6.63E-02
8.126657	0.004	0.009	6.43E-01	0.005	0.013	6.79E-01
6.271567	-0.007	0.009	4.18E-01	-0.013	0.013	3.17E-01

5.73193	0.006	0.009	4.89E-01	0.007	0.014	6.03E-01
7.923115	-0.004	0.008	6.37E-01	-0.010	0.012	4.03E-01
8.754103	0.006	0.009	4.88E-01	0.008	0.013	5.48E-01
6.410178	-0.009	0.009	3.14E-01	-0.003	0.014	8.22E-01
5.595338	0.007	0.010	4.79E-01	0.002	0.016	9.22E-01
4.400666	-0.011	0.015	4.83E-01	0.025	0.022	2.71E-01
2.547258625	-0.012	0.018	5.04E-01	-0.022	0.027	4.03E-01
3.783985	-0.009	0.010	3.51E-01	0.014	0.015	3.35E-01
1.233825789	0.005	0.008	5.26E-01	-0.007	0.012	5.19E-01
3.702568	0.004	0.005	4.56E-01	0.020	0.008	9.64E-03
7.456483	-0.005	0.008	5.60E-01	-0.014	0.013	2.84E-01
3.052244	-0.006	0.009	5.13E-01	0.015	0.013	2.46E-01
5.978535	0.007	0.007	3.37E-01	0.001	0.011	9.26E-01
5.515603	0.004	0.007	5.88E-01	-0.022	0.010	3.58E-02
7.929507	0.004	0.007	6.29E-01	0.010	0.011	3.47E-01
8.004868	0.005	0.007	4.93E-01	-0.005	0.011	6.19E-01
5.680792	0.004	0.009	6.79E-01	-0.006	0.013	6.18E-01
1.033648397	0.004	0.005	4.67E-01	0.005	0.008	5.25E-01
6.087875	0.009	0.010	3.52E-01	0.014	0.014	3.30E-01
4.36164	-0.009	0.013	4.78E-01	0.006	0.020	7.72E-01
7.061511	-0.003	0.009	7.20E-01	-0.020	0.014	1.56E-01
7.202813	-0.003	0.010	7.66E-01	-0.014	0.015	3.32E-01
0.753400049	-0.004	0.005	4.70E-01	-0.007	0.008	4.04E-01
7.419139	0.004	0.008	5.63E-01	-0.004	0.011	7.55E-01
3.607358	0.004	0.008	6.26E-01	-0.017	0.012	1.48E-01
8.257193	-0.004	0.010	6.48E-01	-0.012	0.014	4.06E-01
8.453669	0.010	0.014	4.72E-01	-0.002	0.021	9.28E-01
1.453179839	0.003	0.004	3.96E-01	0.002	0.005	7.02E-01
2.298971373	-0.004	0.005	4.08E-01	-0.007	0.008	3.79E-01
2.354819183	0.007	0.007	2.98E-01	0.012	0.011	2.52E-01
7.435624	0.007	0.009	4.74E-01	-0.014	0.014	3.28E-01
8.786737	0.005	0.008	5.14E-01	-0.017	0.012	1.54E-01
8.458379	0.007	0.009	4.38E-01	0.007	0.013	5.86E-01
6.302183	0.004	0.006	4.45E-01	-0.002	0.009	7.74E-01
0.536064595	0.007	0.008	3.83E-01	0.020	0.012	9.53E-02
7.621671	-0.015	0.012	2.31E-01	0.022	0.018	2.26E-01
6.821971	-0.008	0.010	3.86E-01	0.008	0.014	5.65E-01
5.486334	-0.005	0.009	5.86E-01	0.001	0.013	9.09E-01
2.169108392	0.009	0.011	4.02E-01	-0.018	0.016	2.64E-01
8.515236	0.005	0.009	6.18E-01	-0.011	0.014	4.48E-01
8.379654	0.005	0.006	4.58E-01	-0.003	0.009	7.56E-01
8.72147	0.005	0.010	6.30E-01	-0.008	0.014	5.78E-01
7.07766	0.006	0.009	4.93E-01	0.009	0.013	5.01E-01

0.831788843	-0.008	0.008	3.26E-01	-0.002	0.012	8.61E-01
3.913848	-0.006	0.008	5.00E-01	0.027	0.012	3.17E-02
0.819004404	0.002	0.006	7.06E-01	0.013	0.008	1.20E-01
3.939417	-0.004	0.007	6.07E-01	0.008	0.011	4.25E-01
0.985538537	0.005	0.005	2.76E-01	0.009	0.007	2.26E-01
8.626596	-0.004	0.009	6.21E-01	0.011	0.013	4.02E-01
8.907853	-0.005	0.008	4.95E-01	0.006	0.011	5.76E-01
0.721775385	-0.004	0.007	5.73E-01	0.002	0.011	8.79E-01
2.661309273	-0.014	0.016	3.95E-01	-0.011	0.024	6.32E-01
8.405223	0.008	0.010	3.92E-01	0.012	0.015	3.96E-01
1.714251529	-0.003	0.005	4.85E-01	0.008	0.007	2.63E-01
3.108428	-0.012	0.010	2.22E-01	-0.010	0.015	4.97E-01
0.984865672	0.006	0.005	2.79E-01	0.010	0.008	2.24E-01
5.678437	0.008	0.010	4.50E-01	-0.035	0.015	2.46E-02
7.994439	0.006	0.007	3.54E-01	-0.002	0.010	8.66E-01
5.416692	-0.004	0.008	5.80E-01	-0.006	0.012	6.00E-01
7.070931	-0.006	0.009	5.03E-01	-0.006	0.014	6.86E-01
2.360874969	0.010	0.008	1.73E-01	0.016	0.011	1.52E-01
0.62757426	0.007	0.008	4.05E-01	-0.002	0.012	8.93E-01
0.943820896	-0.002	0.004	6.57E-01	-0.003	0.007	6.25E-01
6.46838	0.006	0.009	4.75E-01	-0.019	0.013	1.44E-01
7.348488	-0.008	0.011	4.44E-01	-0.014	0.016	3.69E-01
7.694004	-0.006	0.008	4.14E-01	-0.004	0.012	7.67E-01
2.837263518	-0.003	0.010	7.26E-01	-0.015	0.015	3.19E-01
0.678712014	0.008	0.011	4.80E-01	-0.010	0.017	5.54E-01
5.925715	-0.004	0.008	5.95E-01	-0.012	0.012	3.22E-01
6.430364	0.005	0.009	5.99E-01	-0.011	0.013	4.18E-01
8.684126	0.005	0.007	4.82E-01	0.000	0.011	9.71E-01
1.677243944	-0.002	0.004	5.75E-01	0.001	0.006	8.70E-01
7.316863	-0.005	0.006	3.61E-01	-0.008	0.009	3.56E-01
5.130724	0.005	0.007	4.25E-01	-0.010	0.010	3.09E-01
5.008936	0.006	0.010	5.41E-01	-0.005	0.014	7.36E-01
7.512331	-0.008	0.009	3.62E-01	-0.013	0.013	3.18E-01
5.692567	-0.006	0.010	5.19E-01	0.003	0.014	8.18E-01
7.540591	0.007	0.008	3.61E-01	0.014	0.012	2.40E-01
6.916172	-0.009	0.009	3.42E-01	-0.019	0.014	1.61E-01
6.659474	-0.005	0.008	5.51E-01	-0.001	0.011	9.17E-01
7.753553	-0.003	0.011	7.49E-01	-0.004	0.016	8.16E-01
8.641062	0.005	0.008	4.80E-01	-0.018	0.011	1.16E-01
7.429905	-0.007	0.011	4.97E-01	0.000	0.016	9.77E-01
5.142836	0.007	0.008	4.36E-01	-0.027	0.012	3.10E-02
5.835887	0.006	0.010	5.39E-01	0.000	0.015	9.75E-01
3.194891	0.003	0.006	5.50E-01	0.015	0.009	8.70E-02

1.705167849	-0.003	0.004	5.25E-01	0.000	0.007	9.52E-01
5.835214	0.006	0.008	4.55E-01	0.014	0.012	2.69E-01
6.486884	0.005	0.008	5.31E-01	-0.002	0.011	8.74E-01
2.288878395	-0.002	0.005	7.18E-01	-0.003	0.007	6.37E-01
2.165071201	0.008	0.010	4.09E-01	-0.015	0.015	3.38E-01
5.588273	-0.007	0.009	4.52E-01	-0.021	0.014	1.41E-01
7.451773	0.002	0.010	8.75E-01	0.003	0.015	8.19E-01
7.232419	-0.007	0.009	4.49E-01	-0.002	0.014	8.84E-01
3.749669	0.006	0.009	5.33E-01	0.042	0.014	2.31E-03
8.813652	0.007	0.008	4.06E-01	-0.010	0.012	3.85E-01
6.265512	0.006	0.007	4.13E-01	-0.014	0.010	1.83E-01
4.367023	-0.009	0.013	4.85E-01	0.008	0.019	6.64E-01
0.80285564	0.001	0.006	8.26E-01	0.007	0.009	4.48E-01
4.087447	0.011	0.010	2.70E-01	0.008	0.015	5.69E-01
7.10693	0.007	0.008	4.14E-01	0.026	0.012	3.06E-02
3.630908	-0.003	0.004	5.00E-01	0.005	0.006	3.79E-01
1.804751896	-0.006	0.010	5.64E-01	0.006	0.015	7.05E-01
8.96942	-0.006	0.008	4.43E-01	0.006	0.012	6.42E-01
8.193943	0.008	0.010	4.22E-01	0.012	0.014	4.08E-01
8.187551	-0.009	0.010	3.59E-01	0.007	0.014	6.27E-01
7.566833	0.004	0.007	5.44E-01	0.003	0.010	7.47E-01
3.994255	-0.008	0.010	4.40E-01	0.007	0.015	6.37E-01
2.093411059	-0.004	0.006	4.69E-01	0.003	0.009	7.17E-01
0.687795694	0.011	0.015	4.68E-01	-0.015	0.023	5.00E-01
3.298176	0.010	0.011	3.94E-01	0.017	0.017	3.31E-01
2.611180817	-0.012	0.014	3.98E-01	0.026	0.020	2.08E-01
5.040224	0.005	0.010	5.70E-01	-0.008	0.014	5.69E-01
8.895742	0.003	0.006	6.42E-01	-0.003	0.009	7.28E-01
8.451987	0.003	0.011	7.83E-01	0.012	0.016	4.59E-01
0.541783949	-0.002	0.007	8.07E-01	0.010	0.011	3.58E-01
6.297809	-0.008	0.010	3.91E-01	0.000	0.015	9.99E-01
8.253492	-0.002	0.008	7.79E-01	0.019	0.012	1.33E-01
1.225078542	0.005	0.008	5.37E-01	-0.004	0.012	7.33E-01
4.110324	-0.005	0.010	6.33E-01	0.003	0.015	8.30E-01
4.118735	-0.007	0.011	5.64E-01	0.015	0.017	3.89E-01
6.162227	0.009	0.010	3.65E-01	-0.003	0.016	8.67E-01
6.893631	0.008	0.009	3.57E-01	-0.005	0.013	7.00E-01
3.439478	0.006	0.007	4.49E-01	0.021	0.011	5.91E-02
6.623139	-0.006	0.010	5.32E-01	-0.007	0.015	6.58E-01
7.151002	-0.005	0.007	4.53E-01	-0.011	0.010	3.02E-01
2.66736506	-0.005	0.005	2.97E-01	0.013	0.007	7.57E-02
0.901766822	0.007	0.007	3.40E-01	-0.016	0.010	1.11E-01
7.853473	-0.008	0.010	4.39E-01	0.029	0.015	5.93E-02

8.227586	-0.012	0.009	1.96E-01	-0.015	0.014	2.80E-01
7.772057	-0.009	0.012	4.64E-01	-0.005	0.018	7.65E-01
8.618185	-0.010	0.007	1.77E-01	-0.012	0.011	2.84E-01
2.499148764	-0.011	0.012	3.79E-01	-0.007	0.018	7.04E-01
0.985202104	0.006	0.005	2.73E-01	0.010	0.008	1.93E-01
8.989606	-0.005	0.007	4.18E-01	0.006	0.010	5.42E-01
8.157272	-0.005	0.008	4.89E-01	0.009	0.011	4.10E-01
8.525666	0.002	0.007	7.60E-01	0.012	0.011	2.47E-01
6.794384	0.007	0.007	3.24E-01	-0.003	0.010	7.81E-01
2.679476633	-0.007	0.006	2.17E-01	0.003	0.009	7.24E-01
6.780253	-0.006	0.008	4.67E-01	-0.005	0.012	6.62E-01
6.493613	0.003	0.008	7.27E-01	-0.011	0.012	3.53E-01
7.887117	-0.004	0.008	5.97E-01	0.020	0.013	1.13E-01
5.79417	0.005	0.008	5.52E-01	0.001	0.012	9.30E-01
1.431984585	0.004	0.004	3.11E-01	0.006	0.006	3.68E-01
8.825091	0.005	0.007	4.52E-01	0.004	0.010	6.88E-01
5.006581	0.007	0.011	5.12E-01	-0.010	0.016	5.57E-01
1.82863861	-0.009	0.010	3.56E-01	-0.003	0.015	8.51E-01
6.376198	0.003	0.007	6.37E-01	-0.018	0.011	1.01E-01
8.60641	0.003	0.009	7.21E-01	0.003	0.013	8.50E-01
0.573745045	0.007	0.009	4.30E-01	0.019	0.013	1.45E-01
5.646139	0.004	0.008	6.40E-01	-0.017	0.011	1.36E-01
3.036095	-0.005	0.007	4.90E-01	-0.012	0.011	2.77E-01
7.889135	0.010	0.010	2.94E-01	0.031	0.015	4.01E-02
8.084603	-0.006	0.008	5.00E-01	0.014	0.013	2.61E-01
6.259792	-0.005	0.007	4.38E-01	-0.017	0.010	1.05E-01
8.448286	0.010	0.010	3.30E-01	0.005	0.015	7.54E-01
1.652347932	0.003	0.005	5.28E-01	0.002	0.007	8.18E-01
5.736303	0.006	0.011	5.71E-01	0.005	0.017	7.75E-01
2.577537558	-0.002	0.009	8.22E-01	0.014	0.013	2.93E-01
4.240524	0.010	0.013	4.33E-01	-0.009	0.019	6.13E-01
4.355247	-0.008	0.014	5.52E-01	0.010	0.021	6.38E-01
2.03251676	0.004	0.004	3.27E-01	0.008	0.007	2.50E-01
8.22254	0.003	0.007	6.34E-01	0.001	0.011	9.55E-01
1.04172278	0.003	0.007	6.74E-01	0.022	0.011	4.35E-02
6.570992	-0.006	0.008	4.52E-01	-0.029	0.013	1.91E-02
2.776705652	-0.009	0.011	4.20E-01	-0.043	0.017	1.06E-02
7.128798	0.008	0.009	4.11E-01	-0.012	0.014	3.95E-01
3.763799	0.008	0.009	4.11E-01	0.016	0.014	2.48E-01
1.744530462	-0.005	0.006	4.26E-01	0.010	0.009	2.73E-01
5.541845	0.005	0.009	5.67E-01	0.000	0.013	9.77E-01
8.398494	0.006	0.009	5.06E-01	0.013	0.014	3.62E-01
4.353229	-0.010	0.015	5.08E-01	0.008	0.022	7.03E-01

6.27224	0.001	0.007	8.36E-01	-0.003	0.010	7.58E-01
5.286156	-0.007	0.008	3.75E-01	-0.026	0.012	3.73E-02
6.989178	-0.003	0.007	6.55E-01	-0.011	0.010	2.92E-01
0.6094069	0.008	0.009	3.63E-01	0.001	0.014	9.50E-01
8.637698	-0.006	0.007	4.38E-01	-0.001	0.011	9.11E-01
0.721438953	-0.004	0.007	5.92E-01	0.013	0.011	2.47E-01
0.649778811	-0.008	0.011	4.23E-01	0.003	0.016	8.55E-01
5.417029	0.007	0.008	3.91E-01	-0.013	0.012	2.74E-01
0.803528505	0.001	0.006	8.03E-01	0.008	0.008	3.37E-01
8.391766	0.006	0.008	4.99E-01	0.009	0.012	4.72E-01
8.784046	-0.003	0.008	6.68E-01	0.004	0.012	7.54E-01
7.410055	-0.008	0.007	3.08E-01	-0.018	0.011	1.03E-01
6.762086	0.006	0.007	4.45E-01	-0.003	0.011	7.53E-01
6.127574	-0.005	0.007	5.23E-01	-0.011	0.011	3.24E-01
8.309003	0.006	0.010	5.18E-01	0.005	0.014	7.50E-01
6.42128	-0.007	0.009	4.61E-01	-0.014	0.014	3.01E-01
5.874913	0.006	0.009	5.08E-01	-0.008	0.013	5.31E-01
1.687336922	-0.003	0.004	4.54E-01	0.002	0.006	7.28E-01
6.792029	-0.003	0.008	6.84E-01	-0.011	0.012	3.54E-01
6.23456	0.005	0.007	5.09E-01	0.000	0.011	9.70E-01
8.958991	0.004	0.007	5.51E-01	-0.009	0.011	4.34E-01
1.978351113	0.004	0.005	4.06E-01	-0.002	0.007	7.54E-01
5.50652	0.007	0.011	5.15E-01	-0.004	0.016	7.86E-01
1.037685588	0.003	0.006	5.93E-01	0.005	0.009	5.55E-01
4.300745	-0.009	0.015	5.46E-01	0.020	0.023	3.84E-01
7.491136	0.004	0.009	6.99E-01	0.009	0.014	4.99E-01
1.51306484	0.006	0.008	4.35E-01	-0.014	0.011	2.36E-01
3.890634	0.003	0.005	5.69E-01	0.022	0.007	2.70E-03
2.662318571	-0.018	0.021	3.80E-01	-0.017	0.031	5.77E-01
0.83145241	-0.008	0.008	3.33E-01	-0.002	0.012	8.86E-01
2.534137754	-0.007	0.011	5.59E-01	-0.005	0.017	7.86E-01
1.717279423	-0.004	0.005	4.88E-01	-0.003	0.008	6.79E-01
8.623568	-0.008	0.009	3.53E-01	0.020	0.013	1.20E-01
7.481715	-0.006	0.009	4.59E-01	0.002	0.013	8.58E-01
6.316649	0.004	0.008	5.84E-01	-0.012	0.012	3.38E-01
1.143998287	0.006	0.006	2.98E-01	-0.002	0.009	8.13E-01
6.587141	0.004	0.007	6.02E-01	0.009	0.011	4.16E-01
7.888462	-0.005	0.009	5.98E-01	0.023	0.014	9.82E-02
7.825213	-0.012	0.009	1.81E-01	0.016	0.014	2.54E-01
0.647423783	-0.006	0.010	5.80E-01	-0.022	0.015	1.60E-01
3.991564	-0.005	0.009	5.56E-01	0.004	0.013	7.85E-01
7.976944	0.002	0.008	7.90E-01	0.013	0.012	3.01E-01
3.880541	0.003	0.005	5.37E-01	0.020	0.008	1.02E-02

3.918894	-0.004	0.010	6.69E-01	0.026	0.015	8.99E-02
8.610447	-0.006	0.007	3.90E-01	-0.007	0.011	5.44E-01
6.295118	-0.010	0.009	2.52E-01	-0.005	0.013	6.87E-01
1.004042329	0.007	0.009	4.25E-01	0.002	0.013	8.94E-01
8.652501	-0.003	0.009	7.11E-01	-0.013	0.014	3.47E-01
4.009058	0.009	0.013	5.11E-01	0.018	0.020	3.69E-01
1.978687546	0.004	0.005	4.00E-01	-0.003	0.007	7.03E-01
8.279734	0.004	0.008	5.89E-01	0.016	0.012	1.84E-01
8.625923	-0.005	0.008	5.51E-01	-0.007	0.012	5.47E-01
7.916386	0.010	0.010	3.15E-01	-0.020	0.015	1.93E-01
8.372589	0.006	0.009	5.31E-01	0.018	0.013	1.67E-01
1.027256178	0.004	0.008	6.09E-01	0.011	0.011	3.38E-01
0.969726205	-0.004	0.005	4.31E-01	0.009	0.008	2.69E-01
0.71201884	0.005	0.008	5.29E-01	0.015	0.012	2.31E-01
8.369561	0.006	0.008	4.47E-01	0.000	0.011	9.87E-01
7.103229	-0.006	0.011	5.69E-01	0.013	0.016	4.14E-01
6.230523	0.005	0.009	6.04E-01	-0.010	0.013	4.47E-01
6.460979	0.003	0.008	7.43E-01	-0.005	0.012	7.02E-01
2.507559579	-0.009	0.012	4.53E-01	-0.015	0.018	4.06E-01
6.431036	0.006	0.009	5.13E-01	0.014	0.013	2.83E-01
3.303223	-0.007	0.011	5.55E-01	0.005	0.017	7.78E-01
5.46312	0.003	0.007	6.40E-01	-0.005	0.010	5.94E-01
4.353902	-0.008	0.015	5.81E-01	0.011	0.022	6.14E-01
2.407975532	-0.014	0.018	4.10E-01	0.008	0.026	7.54E-01
7.079342	0.002	0.009	7.86E-01	0.007	0.013	5.74E-01
4.206881	-0.006	0.012	6.30E-01	0.012	0.019	5.29E-01
3.797106	0.006	0.006	3.39E-01	0.016	0.009	5.85E-02
1.826956447	-0.010	0.011	3.46E-01	-0.005	0.016	7.59E-01
6.743919	-0.007	0.010	4.36E-01	-0.018	0.014	2.10E-01
0.655161732	-0.011	0.011	3.16E-01	-0.025	0.017	1.29E-01
6.117481	-0.004	0.010	6.63E-01	0.032	0.015	2.69E-02
8.446604	0.005	0.007	5.27E-01	0.014	0.011	1.87E-01
6.240952	0.007	0.008	4.29E-01	0.008	0.012	5.21E-01
7.996457	0.003	0.007	6.84E-01	0.000	0.011	9.80E-01
0.584510888	0.008	0.008	2.99E-01	0.007	0.012	5.73E-01
3.561603	-0.006	0.008	4.67E-01	0.028	0.012	1.58E-02
4.421525	-0.009	0.014	5.30E-01	0.017	0.022	4.41E-01
6.516827	-0.007	0.008	3.71E-01	-0.015	0.012	2.24E-01
8.758477	0.004	0.006	4.53E-01	0.001	0.009	8.79E-01
8.565028	0.003	0.008	7.59E-01	-0.017	0.012	1.67E-01
5.407272	0.008	0.009	3.97E-01	-0.019	0.014	1.79E-01
4.117726	-0.006	0.011	6.03E-01	0.016	0.016	3.13E-01
7.437306	0.008	0.010	4.05E-01	0.011	0.014	4.37E-01

2.583929777	-0.001	0.012	9.31E-01	0.013	0.018	4.55E-01
2.619928065	-0.008	0.013	5.28E-01	0.025	0.019	1.83E-01
3.667579	-0.003	0.004	5.50E-01	0.006	0.007	3.39E-01
5.099436	0.007	0.007	3.67E-01	-0.003	0.011	7.88E-01
7.07295	-0.007	0.009	4.22E-01	-0.013	0.013	3.46E-01
8.53845	-0.006	0.007	3.90E-01	0.003	0.011	8.16E-01
2.792517984	-0.009	0.012	4.62E-01	-0.061	0.019	1.05E-03
1.977005383	0.004	0.005	4.67E-01	-0.004	0.007	5.80E-01
8.443913	0.009	0.009	3.00E-01	0.018	0.013	1.54E-01
7.118705	-0.005	0.008	5.82E-01	-0.024	0.013	5.64E-02
3.276981	-0.012	0.012	3.23E-01	0.013	0.018	4.79E-01
7.608551	-0.007	0.011	5.13E-01	-0.006	0.017	7.27E-01
7.895191	0.007	0.009	4.14E-01	0.018	0.013	1.67E-01
4.390909	-0.010	0.013	4.47E-01	0.009	0.019	6.55E-01
7.148311	0.004	0.007	6.31E-01	0.007	0.011	5.16E-01
1.505663323	0.004	0.006	5.28E-01	-0.010	0.009	2.40E-01
2.50251309	-0.010	0.013	4.41E-01	0.003	0.019	8.73E-01
4.097204	-0.003	0.009	6.98E-01	0.005	0.013	6.98E-01
8.444922	0.007	0.007	3.26E-01	-0.008	0.011	4.23E-01
5.005572	0.006	0.009	5.15E-01	-0.007	0.013	6.01E-01
1.065609494	0.004	0.007	5.22E-01	0.002	0.010	8.71E-01
7.611915	-0.010	0.014	4.91E-01	-0.041	0.021	5.50E-02
4.301082	-0.009	0.015	5.20E-01	0.018	0.022	3.99E-01
8.377972	0.005	0.007	5.00E-01	-0.003	0.010	7.90E-01
6.749302	-0.007	0.012	5.20E-01	0.012	0.017	4.82E-01
6.937368	-0.007	0.009	4.48E-01	0.013	0.014	3.53E-01
6.576712	0.001	0.009	8.68E-01	0.006	0.013	6.35E-01
7.374393	-0.006	0.008	4.98E-01	0.008	0.013	5.39E-01
0.669964766	-0.006	0.009	4.91E-01	-0.017	0.013	1.96E-01
6.291417	-0.009	0.009	3.39E-01	0.019	0.014	1.84E-01
4.392255	-0.008	0.013	5.41E-01	0.005	0.019	7.95E-01
4.385526	-0.010	0.014	4.87E-01	0.010	0.022	6.49E-01
3.311297	-0.011	0.011	3.49E-01	0.007	0.017	6.97E-01
0.759792268	0.003	0.005	5.05E-01	-0.002	0.008	8.12E-01
2.084327379	-0.003	0.006	5.78E-01	0.009	0.009	3.43E-01
6.176693	0.007	0.008	3.80E-01	-0.013	0.012	2.77E-01
8.181495	-0.010	0.009	2.72E-01	-0.001	0.013	9.32E-01
5.887698	0.004	0.009	6.26E-01	-0.001	0.013	9.52E-01
7.448745	0.002	0.006	7.18E-01	0.000	0.009	9.59E-01
7.604513	-0.002	0.011	8.24E-01	-0.011	0.016	5.03E-01
5.58289	0.008	0.009	3.74E-01	0.011	0.013	3.95E-01
4.194433	-0.006	0.011	5.80E-01	0.013	0.016	4.06E-01
1.32869978	0.012	0.009	1.83E-01	0.002	0.014	9.01E-01

7.157731	0.004	0.006	4.89E-01	-0.010	0.010	3.08E-01
8.290163	-0.008	0.010	4.29E-01	-0.022	0.015	1.33E-01
0.724466846	0.004	0.007	5.49E-01	0.018	0.011	9.16E-02
7.293986	-0.008	0.010	4.30E-01	0.001	0.015	9.60E-01
1.001350869	0.006	0.008	4.47E-01	-0.002	0.012	8.74E-01
2.673084414	-0.009	0.009	3.26E-01	0.004	0.013	7.39E-01
0.657853193	0.008	0.011	4.77E-01	0.002	0.017	8.93E-01
7.417793	-0.004	0.009	6.49E-01	-0.002	0.013	8.98E-01
5.3198	0.012	0.017	4.63E-01	-0.032	0.025	1.97E-01
7.591392	0.008	0.011	4.29E-01	-0.015	0.016	3.59E-01
2.615890874	-0.008	0.012	4.95E-01	0.031	0.018	8.79E-02
2.331605334	0.004	0.006	4.71E-01	-0.003	0.008	7.30E-01
1.169903597	0.006	0.005	2.10E-01	-0.007	0.007	3.56E-01
7.663725	0.008	0.011	4.57E-01	-0.001	0.017	9.37E-01
2.85307585	0.007	0.009	4.60E-01	-0.005	0.014	7.09E-01
3.617451	-0.003	0.006	5.68E-01	-0.002	0.009	7.87E-01
7.01542	0.004	0.009	6.22E-01	-0.004	0.014	7.85E-01
0.50948642	0.006	0.008	4.70E-01	0.000	0.012	9.97E-01
7.793925	-0.007	0.010	4.74E-01	0.008	0.014	5.76E-01
1.508018351	0.004	0.006	4.87E-01	-0.007	0.010	4.75E-01
4.373078	-0.009	0.015	5.53E-01	0.017	0.022	4.35E-01
1.676234646	0.004	0.004	3.07E-01	0.002	0.006	7.85E-01
1.754959873	0.006	0.008	4.55E-01	0.011	0.012	3.35E-01
6.899351	0.006	0.008	4.33E-01	-0.009	0.012	4.69E-01
8.016307	-0.008	0.010	4.32E-01	-0.014	0.015	3.59E-01
8.416662	0.003	0.008	6.90E-01	-0.011	0.012	3.67E-01
6.95318	-0.004	0.008	5.91E-01	-0.007	0.012	5.43E-01
5.543864	0.006	0.010	5.10E-01	0.010	0.014	4.70E-01
7.488781	0.008	0.010	4.37E-01	0.002	0.015	8.83E-01
6.421953	0.002	0.008	8.42E-01	-0.009	0.012	4.51E-01
7.082706	-0.002	0.008	7.96E-01	-0.001	0.012	9.20E-01
4.349528	-0.012	0.015	4.49E-01	0.016	0.023	4.79E-01
0.976118424	0.004	0.006	5.30E-01	0.019	0.009	3.30E-02
8.274351	0.006	0.007	3.45E-01	-0.001	0.010	9.21E-01
4.120081	-0.005	0.011	6.23E-01	0.018	0.016	2.52E-01
7.261016	-0.009	0.009	3.28E-01	-0.025	0.014	6.55E-02
6.017561	0.007	0.009	4.78E-01	0.009	0.014	5.20E-01
5.947246	0.006	0.009	4.70E-01	-0.008	0.013	5.16E-01
6.624149	-0.010	0.010	3.30E-01	-0.015	0.015	3.23E-01
7.35488	-0.004	0.010	6.76E-01	-0.025	0.015	1.03E-01
7.941619	-0.005	0.009	5.79E-01	-0.014	0.014	3.26E-01
6.559554	-0.007	0.010	4.74E-01	-0.010	0.015	4.89E-01
6.426663	-0.005	0.008	5.31E-01	0.006	0.012	6.44E-01

4.393601	-0.009	0.014	5.43E-01	0.014	0.021	5.06E-01
8.269977	0.004	0.010	7.06E-01	-0.019	0.015	2.10E-01
0.831115977	-0.008	0.008	3.39E-01	-0.001	0.012	9.06E-01
1.085459016	0.006	0.008	4.57E-01	0.002	0.012	8.31E-01
7.538573	-0.005	0.010	6.17E-01	-0.009	0.015	5.30E-01
8.651492	0.006	0.009	4.91E-01	-0.010	0.013	4.35E-01
5.802244	0.006	0.010	5.17E-01	-0.008	0.014	5.98E-01
3.929324	0.006	0.008	4.21E-01	0.025	0.012	3.12E-02
5.621916	-0.009	0.011	3.94E-01	0.006	0.016	6.96E-01
1.670851725	-0.002	0.004	5.25E-01	0.003	0.006	5.85E-01
6.893295	0.008	0.009	4.17E-01	0.008	0.014	5.41E-01
0.727158307	0.004	0.007	5.86E-01	-0.002	0.011	8.46E-01
7.865921	-0.008	0.011	4.59E-01	-0.022	0.016	1.69E-01
4.392591	-0.008	0.012	5.41E-01	0.011	0.018	5.63E-01
4.323623	-0.010	0.014	4.86E-01	0.013	0.021	5.21E-01
1.437367507	0.004	0.004	3.09E-01	0.003	0.006	6.85E-01
7.124424	-0.005	0.008	5.69E-01	-0.011	0.012	3.64E-01
1.851516026	-0.004	0.008	6.52E-01	0.004	0.012	7.12E-01
1.480430878	0.009	0.008	2.31E-01	-0.008	0.011	4.74E-01
8.189906	-0.007	0.009	4.32E-01	-0.002	0.014	8.89E-01
5.138799	0.006	0.008	4.70E-01	-0.008	0.012	5.08E-01
8.2508	0.005	0.008	4.74E-01	0.003	0.011	7.91E-01
2.087355273	-0.004	0.006	5.13E-01	0.009	0.009	3.18E-01
8.322124	-0.007	0.009	4.57E-01	0.009	0.014	5.14E-01
8.654183	0.006	0.009	4.89E-01	-0.025	0.013	6.50E-02
3.68541	-0.003	0.004	5.13E-01	0.006	0.007	3.29E-01
5.297595	0.010	0.013	4.59E-01	-0.023	0.019	2.35E-01
6.577048	0.003	0.010	7.53E-01	-0.004	0.015	8.04E-01
7.25967	-0.011	0.011	3.41E-01	0.013	0.017	4.45E-01
3.051235	-0.008	0.010	4.13E-01	0.015	0.014	2.87E-01
7.281874	-0.008	0.010	4.50E-01	0.017	0.015	2.75E-01
4.236487	0.009	0.013	5.09E-01	0.021	0.019	2.83E-01
6.543741	-0.004	0.009	6.81E-01	-0.035	0.014	1.35E-02
7.109621	-0.006	0.010	5.23E-01	-0.010	0.015	4.79E-01
0.581146562	0.004	0.009	6.30E-01	-0.005	0.013	7.12E-01
5.474895	-0.005	0.007	5.12E-01	-0.017	0.011	1.12E-01
4.226394	0.007	0.012	5.38E-01	0.021	0.018	2.48E-01
1.512055542	0.005	0.007	4.63E-01	-0.013	0.011	2.60E-01
1.976332518	0.004	0.005	4.77E-01	-0.003	0.007	6.51E-01
7.37002	0.009	0.010	3.48E-01	0.010	0.014	4.81E-01
7.490463	-0.004	0.009	6.47E-01	0.014	0.014	3.23E-01
5.953639	0.005	0.009	5.53E-01	-0.003	0.013	8.16E-01
6.493276	0.003	0.010	7.18E-01	-0.009	0.014	5.39E-01

6.081483	0.007	0.008	3.74E-01	0.008	0.012	4.66E-01
2.663327869	-0.016	0.017	3.49E-01	-0.011	0.025	6.49E-01
8.980523	0.007	0.010	4.73E-01	-0.003	0.014	8.30E-01
7.54261	0.003	0.010	7.86E-01	0.007	0.015	6.58E-01
8.731563	0.005	0.009	5.58E-01	0.008	0.013	5.52E-01
8.534749	-0.004	0.008	5.68E-01	-0.014	0.012	2.34E-01
8.785392	0.003	0.010	7.21E-01	0.015	0.014	2.91E-01
3.598274	0.005	0.007	4.74E-01	0.004	0.010	6.60E-01
2.677121605	-0.007	0.007	2.64E-01	0.012	0.010	2.23E-01
8.218503	-0.004	0.006	5.17E-01	-0.003	0.009	7.43E-01
4.142622	-0.010	0.013	4.59E-01	0.011	0.020	5.63E-01
5.719145	0.003	0.008	6.70E-01	-0.002	0.012	8.80E-01
1.81686347	0.008	0.010	4.06E-01	-0.001	0.014	9.56E-01
2.569799609	-0.007	0.008	4.36E-01	0.008	0.013	5.06E-01
6.83812	-0.008	0.008	3.68E-01	-0.002	0.013	8.69E-01
8.647118	0.004	0.007	5.77E-01	0.007	0.010	4.97E-01
4.208899	-0.006	0.015	6.91E-01	0.018	0.022	4.18E-01
8.708349	0.005	0.009	6.19E-01	0.004	0.014	7.94E-01
5.103473	0.006	0.007	4.23E-01	-0.009	0.011	4.20E-01
1.447796917	0.003	0.004	4.45E-01	0.004	0.006	5.43E-01
6.418925	-0.007	0.008	3.49E-01	-0.008	0.012	4.64E-01
5.076559	0.005	0.007	5.26E-01	-0.002	0.011	8.18E-01
7.207859	-0.005	0.007	4.83E-01	-0.008	0.011	4.53E-01
0.659871789	-0.009	0.010	3.45E-01	-0.009	0.014	5.28E-01
5.74976	-0.008	0.010	4.39E-01	0.003	0.015	8.65E-01
6.821635	0.007	0.010	5.05E-01	0.013	0.015	3.97E-01
7.224345	-0.008	0.010	4.29E-01	0.018	0.014	2.10E-01
6.363414	0.003	0.006	5.96E-01	0.010	0.009	2.50E-01
1.868001223	-0.005	0.009	6.15E-01	-0.012	0.013	3.80E-01
0.593258136	0.010	0.010	3.63E-01	-0.008	0.016	5.91E-01
6.21471	-0.004	0.008	6.60E-01	0.011	0.012	3.76E-01
4.000311	0.005	0.010	5.98E-01	0.009	0.015	5.24E-01
2.518661855	0.021	0.030	4.94E-01	0.001	0.045	9.83E-01
8.145161	0.007	0.009	4.27E-01	-0.002	0.013	8.82E-01
3.373201	0.009	0.011	4.42E-01	-0.006	0.017	7.17E-01
6.913817	-0.006	0.009	5.52E-01	-0.005	0.014	7.14E-01
2.669720088	-0.005	0.005	3.27E-01	0.009	0.007	2.11E-01
0.56264277	-0.004	0.007	5.99E-01	-0.002	0.010	8.04E-01
4.107297	-0.006	0.011	6.13E-01	0.017	0.017	3.31E-01
8.878584	-0.003	0.008	7.05E-01	0.000	0.012	9.76E-01
8.302275	-0.005	0.007	4.34E-01	-0.003	0.010	7.80E-01
1.043741375	0.001	0.006	8.60E-01	0.012	0.008	1.69E-01
7.939264	-0.004	0.006	5.53E-01	-0.001	0.009	8.70E-01

5.055027	0.005	0.008	5.16E-01	0.000	0.011	9.80E-01
6.04784	-0.009	0.009	3.24E-01	-0.010	0.013	4.57E-01
6.574693	0.004	0.008	6.49E-01	0.008	0.012	5.22E-01
6.021598	0.008	0.010	4.30E-01	-0.009	0.015	5.36E-01
8.038511	0.006	0.007	4.21E-01	0.002	0.011	8.41E-01
2.542885001	0.020	0.033	5.42E-01	0.009	0.050	8.51E-01
7.032578	-0.010	0.010	3.09E-01	-0.002	0.015	9.03E-01
0.714037436	0.006	0.008	4.49E-01	0.009	0.012	4.70E-01
5.06512	0.004	0.008	5.76E-01	-0.007	0.012	5.38E-01
2.536829215	0.007	0.012	5.48E-01	0.014	0.019	4.67E-01
3.184462	0.005	0.006	3.55E-01	0.018	0.009	4.44E-02
8.422381	0.004	0.009	6.40E-01	-0.030	0.013	2.33E-02
3.249057	-0.006	0.006	3.80E-01	0.007	0.009	4.49E-01
5.596011	0.004	0.008	5.91E-01	-0.010	0.011	3.60E-01
4.105951	-0.006	0.012	6.13E-01	0.025	0.018	1.75E-01
4.369378	-0.008	0.014	5.80E-01	0.023	0.021	2.69E-01
5.685502	0.007	0.008	3.96E-01	0.006	0.013	6.41E-01
1.092187668	0.006	0.007	4.25E-01	-0.004	0.010	6.90E-01
3.337202	-0.008	0.010	4.45E-01	0.009	0.015	5.58E-01
3.564967	0.005	0.006	4.07E-01	0.019	0.009	4.27E-02
6.833746	0.006	0.009	4.94E-01	0.010	0.013	4.47E-01
5.492726	0.005	0.008	5.69E-01	-0.007	0.012	5.58E-01
7.273464	-0.009	0.009	3.29E-01	-0.001	0.014	9.14E-01
6.077782	0.004	0.007	5.84E-01	-0.005	0.010	5.92E-01
6.583777	0.004	0.009	6.51E-01	0.002	0.013	8.66E-01
2.342707609	0.007	0.006	2.56E-01	-0.005	0.009	5.83E-01
6.384609	-0.007	0.009	4.53E-01	0.002	0.013	8.99E-01
6.491258	0.004	0.007	5.96E-01	-0.005	0.011	6.66E-01
8.074173	0.007	0.009	4.19E-01	-0.007	0.013	5.69E-01
6.669567	0.008	0.009	3.42E-01	-0.003	0.013	8.02E-01
8.263921	-0.007	0.010	4.89E-01	-0.033	0.015	2.64E-02
5.523005	0.005	0.009	5.95E-01	-0.016	0.013	2.17E-01
4.175256	-0.009	0.014	5.18E-01	0.029	0.021	1.64E-01
8.88834	-0.006	0.009	4.97E-01	-0.027	0.014	5.42E-02
6.410514	-0.009	0.009	3.26E-01	-0.017	0.014	2.36E-01
5.504501	-0.008	0.009	3.43E-01	-0.022	0.013	8.31E-02
6.572002	-0.004	0.007	5.78E-01	-0.003	0.011	7.71E-01
8.006214	-0.004	0.010	6.81E-01	-0.019	0.015	2.16E-01
7.295668	-0.006	0.010	5.70E-01	0.004	0.016	7.98E-01
8.356104	0.005	0.007	4.91E-01	0.004	0.011	6.92E-01
6.451895	0.007	0.009	4.32E-01	-0.016	0.013	2.09E-01
8.110844	0.004	0.008	6.42E-01	-0.008	0.012	4.74E-01
2.523708344	-0.006	0.012	6.20E-01	0.007	0.018	7.13E-01

3.802152	0.005	0.006	3.64E-01	0.011	0.008	1.97E-01
3.185471	0.005	0.006	3.98E-01	0.017	0.009	7.04E-02
7.904611	0.003	0.010	7.37E-01	-0.009	0.015	5.78E-01
0.578118669	-0.004	0.007	6.06E-01	0.017	0.010	1.05E-01
2.678803768	-0.007	0.006	2.56E-01	0.007	0.009	4.15E-01
2.658617813	-0.005	0.008	5.52E-01	0.017	0.012	1.67E-01
3.857664	0.003	0.006	6.54E-01	0.013	0.009	1.12E-01
8.001167	-0.005	0.010	6.13E-01	0.017	0.014	2.29E-01
4.229422	-0.007	0.013	5.91E-01	0.020	0.020	3.14E-01
2.580229019	0.011	0.010	2.75E-01	0.030	0.015	5.16E-02
3.312643	-0.011	0.013	3.85E-01	0.014	0.019	4.72E-01
3.64773	0.004	0.005	3.36E-01	0.014	0.007	4.37E-02
7.636474	-0.005	0.011	6.47E-01	0.028	0.016	8.03E-02
1.510373379	0.004	0.007	5.31E-01	-0.010	0.011	3.32E-01
6.63727	0.004	0.010	6.39E-01	0.019	0.014	1.83E-01
6.379226	-0.004	0.007	5.34E-01	0.002	0.010	8.72E-01
3.759425	0.005	0.009	5.69E-01	0.020	0.013	1.15E-01
1.233489356	0.005	0.008	5.59E-01	-0.008	0.012	5.13E-01
3.170332	-0.005	0.006	4.00E-01	0.004	0.009	6.35E-01
8.445258	0.008	0.008	3.19E-01	-0.003	0.012	7.83E-01
6.479483	0.003	0.010	7.44E-01	-0.012	0.015	4.17E-01
2.03890898	0.004	0.004	3.60E-01	0.009	0.007	1.59E-01
5.945901	0.005	0.008	5.00E-01	0.003	0.012	8.28E-01
8.705321	0.004	0.007	6.16E-01	0.005	0.011	6.38E-01
6.62785	0.008	0.008	3.06E-01	-0.010	0.011	3.94E-01
0.90681331	0.004	0.006	4.95E-01	-0.011	0.009	2.03E-01
8.638707	0.005	0.007	4.43E-01	0.003	0.010	7.34E-01
1.043404943	0.002	0.006	7.74E-01	0.014	0.009	1.24E-01
2.503522388	-0.012	0.013	3.43E-01	-0.013	0.019	4.97E-01
3.128614	-0.008	0.009	3.69E-01	-0.009	0.014	5.25E-01
6.27325	0.004	0.007	5.80E-01	-0.022	0.010	2.62E-02
1.760006362	-0.006	0.008	4.67E-01	0.017	0.013	1.83E-01
1.787257402	-0.005	0.011	6.62E-01	0.008	0.016	6.11E-01
5.951956	-0.005	0.008	5.13E-01	-0.008	0.012	5.30E-01
5.843962	0.005	0.008	5.32E-01	0.003	0.012	8.23E-01
3.615769	-0.001	0.006	8.47E-01	0.011	0.009	2.17E-01
7.175225	-0.005	0.009	5.61E-01	-0.015	0.013	2.57E-01
7.14932	-0.007	0.007	2.90E-01	0.004	0.010	6.83E-01
3.550837	0.006	0.015	7.01E-01	0.058	0.022	7.77E-03
8.974467	0.006	0.008	4.78E-01	0.008	0.012	4.73E-01
6.184095	0.004	0.008	6.41E-01	0.008	0.012	4.79E-01
3.744286	-0.012	0.010	2.53E-01	-0.016	0.015	2.78E-01
2.789826523	-0.008	0.012	5.00E-01	-0.050	0.017	4.48E-03

7.579954	-0.006	0.009	5.30E-01	-0.008	0.014	5.61E-01
0.908495473	-0.003	0.006	5.66E-01	-0.022	0.008	1.03E-02
8.507162	-0.007	0.009	4.08E-01	0.009	0.013	4.70E-01
8.005877	0.010	0.010	3.45E-01	-0.007	0.015	6.64E-01
6.596898	0.005	0.010	6.51E-01	0.006	0.015	7.12E-01
6.121518	-0.005	0.008	5.28E-01	-0.003	0.012	8.06E-01
6.729789	-0.010	0.011	3.26E-01	-0.023	0.016	1.41E-01
3.112802	-0.012	0.012	3.06E-01	0.007	0.017	6.69E-01
2.722203572	-0.007	0.008	3.90E-01	-0.003	0.013	8.09E-01
6.755694	-0.003	0.009	7.35E-01	-0.004	0.013	7.64E-01
1.510709812	0.005	0.007	5.00E-01	-0.012	0.011	2.78E-01
1.683299731	-0.001	0.004	8.27E-01	0.007	0.006	2.28E-01
6.505724	0.004	0.008	6.49E-01	-0.008	0.012	4.97E-01
0.59123954	-0.008	0.010	3.88E-01	-0.026	0.014	6.26E-02
4.177275	-0.007	0.011	5.20E-01	0.007	0.017	6.75E-01
1.039031319	0.004	0.008	6.38E-01	0.008	0.012	5.05E-01
8.847632	0.003	0.007	6.35E-01	0.001	0.011	9.39E-01
3.802825	0.005	0.006	3.41E-01	0.013	0.008	1.11E-01
8.200335	0.004	0.008	6.29E-01	-0.003	0.012	7.73E-01
5.684829	0.005	0.008	4.85E-01	0.006	0.011	5.69E-01
5.140145	0.005	0.007	4.95E-01	-0.008	0.010	4.23E-01
5.141154	0.004	0.009	5.99E-01	-0.006	0.013	6.30E-01
6.374516	0.006	0.007	4.03E-01	-0.014	0.011	2.13E-01
6.713976	-0.007	0.010	4.90E-01	-0.005	0.015	7.52E-01
7.579281	-0.007	0.010	4.47E-01	-0.007	0.015	6.09E-01
8.61011	-0.005	0.008	4.74E-01	-0.010	0.011	3.57E-01
6.895313	0.006	0.011	5.81E-01	0.012	0.016	4.50E-01
5.023066	0.006	0.008	4.65E-01	-0.014	0.012	2.37E-01
7.435961	-0.010	0.010	3.42E-01	-0.020	0.015	1.96E-01
4.18434	-0.008	0.015	6.00E-01	0.001	0.022	9.61E-01
5.620907	0.002	0.006	7.64E-01	0.001	0.009	9.33E-01
6.577385	0.005	0.010	6.44E-01	-0.007	0.015	6.53E-01
0.853656961	-0.005	0.007	4.62E-01	-0.012	0.010	2.62E-01
3.276644	-0.010	0.012	3.76E-01	0.012	0.018	4.93E-01
3.987527	-0.006	0.007	4.15E-01	0.009	0.011	3.79E-01
8.018998	0.004	0.007	5.73E-01	-0.012	0.010	2.36E-01
3.994592	-0.007	0.009	4.39E-01	0.006	0.014	6.70E-01
7.501565	0.004	0.007	5.54E-01	0.006	0.010	5.79E-01
2.419077808	-0.005	0.015	7.51E-01	-0.002	0.023	9.22E-01
7.751534	-0.004	0.009	6.69E-01	-0.008	0.014	5.70E-01
6.09595	0.006	0.009	4.77E-01	0.004	0.013	7.54E-01
3.12996	-0.009	0.009	3.46E-01	-0.016	0.014	2.34E-01
6.126565	0.005	0.007	4.33E-01	0.007	0.010	5.19E-01

7.365646	-0.005	0.008	5.68E-01	0.005	0.012	6.84E-01
3.255449	-0.006	0.010	5.37E-01	0.006	0.015	6.97E-01
7.630419	-0.008	0.013	5.53E-01	0.036	0.019	6.03E-02
0.830779545	-0.008	0.008	3.48E-01	-0.001	0.012	9.33E-01
8.214466	0.004	0.007	5.60E-01	-0.006	0.010	5.72E-01
3.577752	0.008	0.007	2.74E-01	0.015	0.011	1.79E-01
8.540132	0.002	0.007	8.14E-01	0.020	0.011	6.74E-02
3.686419	-0.002	0.005	5.82E-01	0.008	0.007	2.50E-01
8.609774	0.003	0.007	6.70E-01	-0.008	0.011	4.40E-01
7.200121	-0.010	0.011	3.91E-01	-0.020	0.017	2.38E-01
6.044139	0.005	0.008	5.03E-01	0.002	0.012	8.51E-01
7.820839	-0.006	0.010	5.66E-01	-0.006	0.015	6.73E-01
8.29891	0.005	0.007	5.20E-01	-0.018	0.011	1.09E-01
6.884548	0.010	0.009	2.63E-01	0.018	0.014	2.03E-01
5.55463	0.006	0.008	4.42E-01	-0.005	0.012	6.69E-01
1.516765598	0.006	0.008	4.84E-01	-0.016	0.012	1.98E-01
4.298054	-0.009	0.013	5.01E-01	0.010	0.020	6.14E-01
7.790897	-0.010	0.010	3.18E-01	0.021	0.015	1.56E-01
6.57503	0.004	0.007	5.13E-01	-0.002	0.010	8.57E-01
6.764105	0.009	0.011	4.28E-01	-0.022	0.016	1.81E-01
0.516215072	0.002	0.008	7.71E-01	0.007	0.013	5.72E-01
6.561572	-0.005	0.009	5.76E-01	0.006	0.013	6.23E-01
4.375097	-0.009	0.015	5.58E-01	0.023	0.023	3.21E-01
0.568025691	0.007	0.008	3.49E-01	0.015	0.012	1.96E-01
7.609223	0.010	0.012	4.10E-01	0.008	0.018	6.52E-01
8.393448	-0.005	0.008	5.62E-01	-0.022	0.012	7.36E-02
8.6922	-0.002	0.008	7.54E-01	0.002	0.011	8.29E-01
2.175500612	0.008	0.011	4.50E-01	-0.018	0.016	2.68E-01
8.892041	0.007	0.009	3.92E-01	-0.016	0.013	2.17E-01
0.633966479	0.005	0.010	6.21E-01	0.004	0.014	7.88E-01
7.735722	-0.006	0.011	5.49E-01	-0.004	0.016	7.84E-01
4.146659	-0.008	0.014	5.92E-01	0.016	0.022	4.53E-01
7.8491	0.002	0.011	8.49E-01	0.024	0.017	1.55E-01
3.736548	-0.012	0.009	1.92E-01	0.023	0.014	1.10E-01
6.22884	0.007	0.009	4.11E-01	-0.006	0.013	6.22E-01
1.752268412	-0.004	0.007	5.22E-01	0.002	0.010	8.49E-01
1.032302667	0.003	0.005	6.10E-01	0.015	0.008	5.92E-02
1.856562515	-0.004	0.008	6.47E-01	-0.008	0.012	5.00E-01
6.018234	0.005	0.008	5.57E-01	-0.002	0.013	8.86E-01
3.763126	0.007	0.009	4.66E-01	0.013	0.013	3.50E-01
4.194769	-0.005	0.010	6.32E-01	0.015	0.015	3.11E-01
1.084786151	0.005	0.007	4.87E-01	-0.004	0.011	7.12E-01
7.565487	-0.005	0.006	3.97E-01	-0.017	0.010	6.96E-02

7.986701	0.004	0.007	5.58E-01	-0.002	0.011	8.30E-01
2.792181551	-0.010	0.013	4.47E-01	-0.060	0.019	1.39E-03
7.553712	0.006	0.008	4.11E-01	0.008	0.011	4.66E-01
6.994561	0.006	0.008	4.67E-01	0.006	0.011	6.12E-01
8.664949	0.003	0.008	7.10E-01	-0.005	0.012	6.39E-01
8.319096	0.006	0.009	4.76E-01	0.015	0.013	2.48E-01
2.515970394	-0.010	0.016	5.11E-01	-0.010	0.023	6.69E-01
3.194555	0.003	0.006	5.75E-01	0.014	0.009	1.02E-01
6.958563	-0.010	0.010	3.09E-01	0.010	0.014	4.95E-01
2.664337167	-0.009	0.010	3.91E-01	-0.009	0.015	5.34E-01
4.092494	0.012	0.013	3.44E-01	0.027	0.019	1.59E-01
7.002635	-0.005	0.008	5.73E-01	-0.017	0.012	1.65E-01
8.087294	-0.005	0.007	5.13E-01	0.015	0.011	1.92E-01
4.283587	0.009	0.011	3.90E-01	0.006	0.016	7.23E-01
2.517988989	0.017	0.026	5.13E-01	0.002	0.038	9.61E-01
6.678987	0.005	0.010	6.04E-01	0.031	0.014	3.00E-02
6.676296	-0.005	0.008	5.17E-01	0.003	0.011	7.59E-01
2.596377783	-0.012	0.019	5.29E-01	0.006	0.028	8.45E-01
1.977678248	0.004	0.005	4.73E-01	-0.003	0.007	7.06E-01
8.865463	-0.006	0.008	4.81E-01	0.010	0.012	4.00E-01
4.367359	-0.008	0.013	5.25E-01	0.007	0.019	6.94E-01
4.338762	-0.008	0.014	5.58E-01	0.007	0.021	7.35E-01
6.856287	-0.006	0.010	5.60E-01	0.008	0.015	5.68E-01
7.65666	0.006	0.008	4.25E-01	-0.007	0.011	5.60E-01
5.895436	0.009	0.010	3.51E-01	0.006	0.015	6.96E-01
0.819340837	0.002	0.006	7.43E-01	0.013	0.008	1.32E-01
3.239637	-0.005	0.006	3.78E-01	0.009	0.009	2.84E-01
1.415162956	0.004	0.004	3.00E-01	0.002	0.006	7.17E-01
1.712569366	-0.003	0.004	5.62E-01	0.008	0.006	2.31E-01
3.576742	0.009	0.008	2.60E-01	0.019	0.012	1.04E-01
2.272056765	0.005	0.007	4.38E-01	-0.002	0.010	8.04E-01
3.801143	0.005	0.006	3.91E-01	0.010	0.009	2.38E-01
1.480767311	0.009	0.007	2.25E-01	-0.006	0.011	5.84E-01
4.362649	-0.009	0.013	5.11E-01	0.008	0.020	6.84E-01
8.158281	-0.007	0.010	4.77E-01	-0.012	0.014	3.86E-01
2.789490091	-0.008	0.012	5.10E-01	-0.048	0.017	4.99E-03
6.673604	-0.005	0.009	5.49E-01	-0.014	0.013	2.79E-01
5.615524	-0.007	0.009	4.51E-01	-0.018	0.013	1.67E-01
6.429354	0.005	0.008	5.04E-01	0.001	0.012	9.52E-01
7.477678	0.008	0.009	3.47E-01	-0.031	0.013	1.71E-02
8.573776	0.006	0.009	5.05E-01	-0.013	0.014	3.56E-01
7.354207	-0.008	0.010	4.23E-01	-0.025	0.016	1.05E-01
1.815854172	0.011	0.011	3.06E-01	0.005	0.017	7.48E-01

7.607878	-0.005	0.013	6.85E-01	-0.009	0.020	6.53E-01
7.696696	-0.007	0.010	4.81E-01	-0.007	0.014	6.33E-01
2.389135307	-0.008	0.012	5.05E-01	-0.003	0.017	8.82E-01
0.596286029	-0.003	0.007	5.96E-01	-0.002	0.010	8.52E-01
7.885435	-0.005	0.008	5.09E-01	0.016	0.012	1.99E-01
7.086744	0.006	0.006	2.97E-01	0.008	0.009	4.07E-01
0.53673746	0.006	0.009	4.69E-01	0.019	0.013	1.47E-01
0.642040861	0.003	0.008	6.71E-01	-0.009	0.012	4.64E-01
0.56533423	0.005	0.008	5.05E-01	0.022	0.012	5.70E-02
6.470736	0.006	0.008	4.97E-01	-0.004	0.012	7.28E-01
5.044261	0.004	0.008	5.96E-01	-0.001	0.011	9.52E-01
1.766062148	-0.005	0.011	6.24E-01	0.004	0.016	8.12E-01
3.900054	0.003	0.005	5.53E-01	0.021	0.008	6.71E-03
6.278296	-0.006	0.009	5.32E-01	0.001	0.013	9.67E-01
6.113781	-0.008	0.009	3.76E-01	0.002	0.013	8.76E-01
2.551968681	-0.009	0.011	3.95E-01	-0.005	0.016	7.72E-01
6.752666	-0.005	0.010	6.56E-01	-0.020	0.015	1.82E-01
1.225414974	0.004	0.008	5.73E-01	-0.004	0.012	7.08E-01
8.033465	-0.003	0.007	6.37E-01	-0.006	0.011	5.80E-01
8.907517	-0.006	0.009	4.65E-01	0.013	0.013	3.24E-01
0.584847321	0.007	0.010	4.54E-01	-0.005	0.014	7.19E-01
8.642744	-0.006	0.009	5.17E-01	-0.025	0.014	6.89E-02
6.994897	0.006	0.008	4.60E-01	-0.001	0.011	9.35E-01
3.800806	0.006	0.006	3.67E-01	0.010	0.009	2.86E-01
5.484652	0.007	0.009	4.21E-01	0.003	0.013	8.42E-01
8.97884	0.006	0.010	5.78E-01	-0.002	0.015	9.09E-01
5.955321	0.004	0.007	5.53E-01	-0.009	0.011	4.24E-01
4.17492	-0.008	0.012	5.34E-01	0.016	0.018	3.78E-01
5.008263	0.005	0.008	5.84E-01	-0.004	0.012	7.73E-01
7.349834	-0.008	0.011	4.43E-01	-0.010	0.016	5.14E-01
8.346011	-0.005	0.009	5.28E-01	0.002	0.013	9.05E-01
5.456391	0.006	0.009	4.76E-01	-0.004	0.013	7.84E-01
3.641674	-0.002	0.004	5.87E-01	0.007	0.005	1.64E-01
7.811419	-0.009	0.009	3.54E-01	-0.001	0.014	9.25E-01
7.929844	0.006	0.009	4.78E-01	0.029	0.013	2.38E-02
8.535086	-0.005	0.008	5.53E-01	-0.007	0.011	5.70E-01
6.497987	0.010	0.010	3.00E-01	-0.015	0.015	3.22E-01
6.532976	0.004	0.010	6.56E-01	0.011	0.014	4.53E-01
2.334296795	0.006	0.006	3.45E-01	0.005	0.010	6.00E-01
0.979819183	0.004	0.007	5.83E-01	0.014	0.010	1.92E-01
2.777042085	-0.008	0.011	4.59E-01	-0.044	0.017	9.96E-03
4.182657	-0.007	0.013	6.01E-01	-0.002	0.020	9.35E-01
8.594298	-0.004	0.007	6.35E-01	-0.008	0.011	4.61E-01

5.971133	0.005	0.007	4.43E-01	-0.002	0.011	8.54E-01
3.543099	-0.009	0.011	3.82E-01	-0.005	0.016	7.61E-01
6.362404	-0.007	0.009	4.46E-01	0.006	0.014	6.43E-01
7.895864	-0.005	0.009	5.53E-01	0.009	0.013	4.93E-01
5.00759	0.008	0.011	4.86E-01	-0.003	0.017	8.39E-01
5.136107	0.005	0.009	6.06E-01	-0.010	0.013	4.54E-01
3.684737	-0.003	0.004	5.08E-01	0.007	0.007	2.86E-01
6.440793	0.003	0.010	7.45E-01	0.013	0.015	3.85E-01
5.563713	0.006	0.009	4.96E-01	-0.018	0.013	1.67E-01
1.03533056	0.003	0.005	5.12E-01	0.006	0.007	4.15E-01
6.855951	-0.005	0.008	5.07E-01	0.013	0.012	2.60E-01
1.71828872	-0.004	0.005	4.02E-01	-0.006	0.008	4.76E-01
1.658740152	0.004	0.004	4.01E-01	0.008	0.007	2.25E-01
6.108398	-0.005	0.008	5.11E-01	-0.007	0.012	5.83E-01
8.773617	-0.007	0.009	4.43E-01	0.012	0.014	3.93E-01
0.996640812	0.004	0.006	4.47E-01	0.014	0.009	1.08E-01
8.66394	0.004	0.008	6.27E-01	-0.009	0.012	4.79E-01
6.495632	-0.007	0.010	4.51E-01	-0.008	0.014	5.72E-01
7.373048	-0.007	0.010	4.81E-01	-0.016	0.015	2.69E-01
2.094420357	-0.003	0.007	6.25E-01	0.000	0.011	9.69E-01
5.405253	0.003	0.007	6.20E-01	-0.004	0.010	7.31E-01
7.179935	0.007	0.009	4.20E-01	-0.008	0.013	5.17E-01
3.573042	-0.002	0.006	6.70E-01	0.010	0.008	2.19E-01
2.833226327	0.008	0.011	4.63E-01	-0.017	0.017	3.28E-01
2.670729386	-0.003	0.005	4.99E-01	0.009	0.007	2.21E-01
1.757651333	0.007	0.008	3.74E-01	0.004	0.012	7.25E-01
6.338854	0.005	0.008	5.11E-01	0.012	0.012	3.02E-01
5.690548	0.004	0.007	5.60E-01	-0.019	0.010	5.81E-02
7.95373	0.005	0.008	4.96E-01	-0.009	0.012	4.71E-01
5.460428	0.005	0.007	4.25E-01	0.013	0.010	1.99E-01
0.791080499	-0.005	0.005	3.20E-01	-0.002	0.007	8.24E-01
2.282822608	-0.003	0.005	5.71E-01	-0.010	0.007	1.81E-01
7.233092	-0.007	0.011	5.19E-01	-0.005	0.017	7.72E-01
7.289949	-0.005	0.011	6.32E-01	-0.010	0.016	5.15E-01
2.093074627	-0.004	0.006	4.89E-01	0.004	0.009	6.57E-01
5.151583	0.004	0.007	5.51E-01	-0.012	0.011	2.93E-01
8.93174	0.003	0.008	6.56E-01	-0.018	0.011	1.10E-01
7.59072	-0.006	0.009	4.75E-01	-0.018	0.013	1.68E-01
4.003675	0.004	0.010	7.09E-01	0.018	0.015	2.22E-01
0.599650355	-0.007	0.009	4.45E-01	-0.009	0.013	4.79E-01
8.008569	-0.006	0.008	4.45E-01	0.018	0.012	1.13E-01
2.541875703	0.023	0.034	5.00E-01	0.023	0.050	6.39E-01
4.045393	0.012	0.011	2.76E-01	-0.003	0.017	8.64E-01

6.311603	0.009	0.011	4.21E-01	-0.005	0.016	7.55E-01
1.71694299	-0.003	0.005	5.51E-01	-0.003	0.007	6.93E-01
0.785361145	-0.004	0.004	2.99E-01	-0.003	0.006	6.47E-01
6.04683	0.003	0.007	6.80E-01	0.005	0.011	6.44E-01
8.528357	0.005	0.007	4.57E-01	0.004	0.011	7.24E-01
7.882407	-0.007	0.009	4.53E-01	0.007	0.013	5.75E-01
1.764043553	-0.006	0.009	5.57E-01	0.003	0.014	8.45E-01
8.787074	0.001	0.008	8.93E-01	-0.010	0.012	3.94E-01
1.785911671	-0.005	0.009	5.56E-01	-0.004	0.013	7.55E-01
0.549185466	-0.003	0.008	6.81E-01	-0.015	0.013	2.24E-01
1.665805236	0.003	0.004	5.31E-01	0.012	0.006	4.20E-02
5.473213	0.004	0.008	6.64E-01	-0.004	0.012	7.64E-01
4.371396	-0.007	0.011	5.36E-01	0.012	0.016	4.81E-01
7.96315	0.004	0.009	6.38E-01	-0.002	0.013	8.74E-01
3.942445	-0.006	0.009	5.23E-01	0.007	0.013	5.61E-01
4.304782	-0.009	0.014	5.23E-01	0.010	0.021	6.35E-01
8.7827	0.004	0.008	6.09E-01	0.010	0.012	4.32E-01
5.64042	0.007	0.009	4.28E-01	-0.006	0.013	6.50E-01
0.631611451	0.007	0.010	4.88E-01	0.011	0.015	4.55E-01
6.862343	0.010	0.010	3.40E-01	0.018	0.015	2.21E-01
5.542182	0.004	0.008	5.60E-01	-0.015	0.011	2.02E-01
8.435502	0.006	0.008	4.29E-01	-0.017	0.012	1.61E-01
0.830443112	-0.007	0.008	3.55E-01	-0.001	0.012	9.58E-01
8.832156	0.007	0.009	4.80E-01	-0.001	0.014	9.69E-01
8.465108	-0.004	0.008	6.20E-01	-0.016	0.011	1.56E-01
2.670392953	-0.004	0.005	4.33E-01	0.008	0.007	2.32E-01
8.544842	0.006	0.007	4.38E-01	-0.001	0.011	8.99E-01
2.670056521	-0.004	0.005	3.68E-01	0.009	0.007	1.89E-01
7.519732	-0.006	0.009	5.47E-01	-0.001	0.014	9.65E-01
6.059278	0.004	0.007	6.10E-01	0.002	0.011	8.53E-01
6.378553	0.008	0.008	3.11E-01	-0.010	0.011	3.78E-01
7.622344	-0.010	0.010	3.44E-01	0.025	0.015	1.04E-01
8.48193	0.006	0.006	3.44E-01	0.000	0.010	9.61E-01
4.322277	-0.008	0.013	5.02E-01	0.023	0.019	2.22E-01
1.771108637	-0.006	0.010	5.78E-01	0.010	0.015	4.96E-01
8.678406	0.003	0.009	7.71E-01	0.002	0.014	9.01E-01
3.320381	0.008	0.011	4.91E-01	0.007	0.017	6.69E-01
5.056373	0.004	0.009	6.26E-01	-0.008	0.013	5.35E-01
8.901797	-0.006	0.008	4.21E-01	0.002	0.011	8.92E-01
8.076528	-0.006	0.009	4.87E-01	0.005	0.013	7.00E-01
7.059156	-0.006	0.010	5.80E-01	-0.007	0.015	6.35E-01
3.434431	0.007	0.013	5.51E-01	0.030	0.019	1.10E-01
5.976516	0.006	0.008	4.65E-01	0.002	0.011	8.87E-01

7.922442	0.006	0.010	5.41E-01	-0.004	0.015	8.02E-01
0.556586983	-0.006	0.009	5.19E-01	0.008	0.013	5.16E-01
8.934431	-0.003	0.009	6.98E-01	-0.003	0.013	8.38E-01
6.459297	0.005	0.007	4.96E-01	0.008	0.011	4.51E-01
7.629409	-0.005	0.007	4.94E-01	0.008	0.010	4.34E-01
5.784413	-0.008	0.009	4.07E-01	-0.016	0.014	2.46E-01
0.652470272	-0.011	0.011	3.37E-01	-0.020	0.016	2.18E-01
5.461774	0.004	0.009	6.37E-01	0.005	0.014	7.03E-01
6.517836	0.008	0.010	4.55E-01	-0.003	0.016	8.46E-01
5.825121	0.008	0.010	4.32E-01	0.005	0.015	7.20E-01
8.164674	-0.009	0.009	3.05E-01	-0.007	0.013	6.10E-01
0.905804013	0.004	0.006	4.92E-01	-0.010	0.009	2.82E-01
6.114453	0.006	0.009	4.60E-01	0.006	0.013	6.60E-01
4.202843	-0.005	0.011	6.55E-01	0.009	0.016	5.61E-01
5.497436	0.006	0.009	5.14E-01	-0.004	0.013	7.41E-01
1.799032542	0.008	0.011	4.37E-01	0.000	0.016	9.99E-01
1.700457793	-0.002	0.004	5.72E-01	0.007	0.006	2.31E-01
1.716606557	-0.003	0.005	5.41E-01	-0.001	0.007	8.51E-01
5.641093	-0.005	0.008	5.37E-01	-0.006	0.013	6.48E-01
7.363628	0.007	0.008	4.15E-01	-0.001	0.012	9.04E-01
7.338732	-0.011	0.010	2.74E-01	-0.017	0.015	2.63E-01
5.685165	0.005	0.008	4.75E-01	0.006	0.011	6.15E-01
7.476669	-0.005	0.008	5.43E-01	-0.015	0.013	2.32E-01
8.810288	0.006	0.009	5.18E-01	-0.016	0.014	2.39E-01
2.752818938	-0.007	0.007	3.64E-01	-0.016	0.011	1.36E-01
5.561022	0.006	0.009	5.60E-01	-0.009	0.014	5.37E-01
5.404581	0.004	0.007	5.30E-01	-0.005	0.010	6.42E-01
2.754164668	-0.007	0.007	3.28E-01	-0.019	0.011	7.78E-02
1.826620015	-0.009	0.011	3.95E-01	0.001	0.016	9.54E-01
2.753155371	-0.007	0.007	3.55E-01	-0.017	0.011	1.09E-01
6.59858	0.004	0.008	6.27E-01	-0.027	0.012	2.30E-02
6.938377	0.008	0.010	4.20E-01	0.016	0.014	2.62E-01
6.58916	0.003	0.008	6.93E-01	0.002	0.012	8.81E-01
7.087416	0.005	0.007	4.67E-01	-0.003	0.010	7.91E-01
7.529152	-0.007	0.009	4.38E-01	0.006	0.013	6.58E-01
4.389563	-0.008	0.015	5.80E-01	0.011	0.022	6.03E-01
4.390573	-0.010	0.014	4.93E-01	0.013	0.021	5.19E-01
6.621121	-0.006	0.008	4.58E-01	0.002	0.012	8.54E-01
7.684248	0.003	0.006	6.63E-01	0.002	0.010	7.94E-01
8.54417	-0.005	0.010	5.79E-01	-0.011	0.015	4.63E-01
7.894855	0.008	0.009	3.57E-01	-0.007	0.013	6.11E-01
7.146629	0.003	0.006	6.76E-01	0.002	0.010	8.58E-01
8.212111	-0.006	0.008	4.42E-01	0.003	0.013	8.10E-01

1.97666895	0.003	0.005	4.95E-01	-0.003	0.007	6.61E-01
8.540469	0.002	0.007	7.57E-01	0.011	0.011	3.15E-01
5.767255	-0.008	0.009	3.61E-01	0.003	0.013	8.41E-01
3.996274	-0.006	0.009	4.96E-01	0.021	0.013	1.14E-01
6.677642	0.004	0.009	6.68E-01	0.013	0.013	3.28E-01
7.654642	0.006	0.008	4.73E-01	-0.010	0.013	4.27E-01
5.88467	-0.004	0.007	5.45E-01	0.003	0.011	7.71E-01
7.765328	-0.006	0.010	5.58E-01	-0.020	0.016	1.87E-01
4.120754	-0.005	0.012	6.75E-01	0.016	0.017	3.61E-01
7.959113	0.006	0.008	4.80E-01	-0.021	0.013	1.02E-01
7.160086	0.006	0.010	5.24E-01	0.015	0.015	3.10E-01
1.233152924	0.004	0.008	5.79E-01	-0.008	0.012	5.17E-01
6.982786	-0.005	0.010	6.33E-01	-0.006	0.015	6.79E-01
3.9919	-0.005	0.009	5.38E-01	0.002	0.013	8.52E-01
1.068637387	0.003	0.007	6.50E-01	-0.009	0.010	3.58E-01
6.252727	0.009	0.009	3.21E-01	-0.016	0.014	2.58E-01
4.115371	-0.003	0.009	7.09E-01	0.016	0.014	2.38E-01
2.651889161	-0.007	0.013	5.73E-01	0.022	0.019	2.44E-01
0.559278444	0.008	0.009	3.58E-01	0.005	0.014	7.35E-01
4.28426	0.009	0.011	4.12E-01	0.012	0.016	4.27E-01
5.598366	0.005	0.008	5.12E-01	-0.004	0.012	7.21E-01
8.337936	-0.007	0.008	4.26E-01	-0.004	0.012	7.50E-01
3.204648	0.005	0.008	5.68E-01	0.017	0.012	1.51E-01
6.163236	0.007	0.010	4.35E-01	0.001	0.014	9.24E-01
8.983551	0.003	0.010	7.33E-01	-0.010	0.015	4.89E-01
5.516276	0.005	0.009	5.67E-01	-0.017	0.013	2.07E-01
6.284688	0.008	0.011	4.32E-01	-0.005	0.016	7.41E-01
6.881183	0.008	0.009	3.75E-01	0.030	0.013	2.32E-02
8.685471	0.006	0.008	4.47E-01	0.001	0.012	9.38E-01
1.142652557	0.007	0.007	3.24E-01	-0.003	0.010	7.35E-01
1.704494984	-0.003	0.004	5.42E-01	-0.001	0.007	9.27E-01
8.046922	0.004	0.009	6.78E-01	-0.016	0.014	2.54E-01
7.118368	-0.005	0.010	6.24E-01	-0.005	0.015	7.06E-01
6.372834	0.002	0.008	7.61E-01	0.003	0.012	7.89E-01
8.378308	0.006	0.008	3.92E-01	-0.011	0.011	3.35E-01
8.774289	-0.009	0.009	3.32E-01	0.012	0.013	3.67E-01
1.870019819	-0.005	0.009	5.35E-01	0.006	0.013	6.25E-01
2.56609885	-0.006	0.011	5.58E-01	0.002	0.016	9.12E-01
4.118399	-0.006	0.012	5.87E-01	0.020	0.018	2.62E-01
0.503767066	-0.006	0.009	5.04E-01	-0.012	0.014	3.59E-01
1.648983607	0.005	0.005	3.47E-01	0.010	0.007	1.59E-01
7.744806	-0.007	0.009	4.50E-01	-0.021	0.014	1.28E-01
6.563254	-0.003	0.009	6.94E-01	-0.021	0.013	1.05E-01

0.752390751	-0.003	0.005	5.14E-01	-0.001	0.008	8.54E-01
6.903724	0.006	0.010	5.49E-01	0.023	0.015	1.37E-01
7.387851	-0.006	0.008	4.66E-01	-0.016	0.012	1.66E-01
5.497772	0.005	0.008	4.95E-01	-0.010	0.011	3.92E-01
8.655529	0.003	0.009	7.52E-01	-0.020	0.014	1.42E-01
1.838395155	-0.003	0.010	7.35E-01	-0.006	0.015	7.05E-01
0.687459261	0.012	0.015	4.41E-01	-0.019	0.022	4.03E-01
6.933667	-0.008	0.009	3.52E-01	-0.005	0.013	7.14E-01
5.831177	0.004	0.010	6.58E-01	-0.002	0.014	9.06E-01
5.072858	0.005	0.008	5.32E-01	-0.020	0.012	8.24E-02
8.595307	-0.007	0.008	3.89E-01	0.001	0.012	9.65E-01
7.988719	0.005	0.010	5.91E-01	-0.028	0.014	4.86E-02
7.115004	-0.005	0.009	6.29E-01	0.002	0.014	9.10E-01
0.642713726	0.004	0.010	6.70E-01	-0.010	0.014	4.94E-01
5.696604	-0.008	0.009	4.01E-01	-0.039	0.014	4.91E-03
7.923788	-0.002	0.008	7.91E-01	-0.006	0.012	6.28E-01
7.3643	0.004	0.008	5.84E-01	-0.004	0.011	7.34E-01
6.280651	-0.004	0.007	6.10E-01	-0.004	0.010	7.36E-01
3.598947	-0.005	0.007	4.79E-01	0.007	0.010	4.80E-01
8.627941	-0.006	0.008	4.17E-01	-0.010	0.012	4.20E-01
3.434768	0.008	0.014	5.64E-01	0.028	0.020	1.67E-01
8.649137	0.002	0.008	7.65E-01	0.016	0.012	1.62E-01
5.240738	-0.001	0.004	9.00E-01	0.004	0.007	5.39E-01
0.741624908	-0.004	0.006	4.90E-01	-0.009	0.009	3.59E-01
7.59913	0.007	0.011	5.19E-01	0.026	0.016	1.06E-01
8.355431	-0.007	0.009	4.68E-01	-0.010	0.014	4.60E-01
1.865982628	0.007	0.009	4.58E-01	-0.005	0.013	7.26E-01
6.315977	0.003	0.008	6.68E-01	-0.020	0.011	7.34E-02
0.642377294	0.003	0.011	7.55E-01	-0.022	0.016	1.73E-01
6.340536	0.005	0.007	5.24E-01	0.005	0.011	6.70E-01
4.301418	-0.009	0.014	5.21E-01	0.013	0.021	5.36E-01
6.643998	0.004	0.008	6.00E-01	-0.008	0.012	4.78E-01
7.081697	-0.004	0.007	5.71E-01	0.000	0.010	9.62E-01
4.391919	-0.008	0.013	5.66E-01	0.006	0.020	7.53E-01
5.893081	0.004	0.007	5.70E-01	-0.011	0.011	3.29E-01
5.788114	0.007	0.009	4.20E-01	0.005	0.014	6.95E-01
7.090444	-0.005	0.010	5.73E-01	0.019	0.014	1.96E-01
6.674277	0.005	0.010	6.13E-01	-0.017	0.015	2.70E-01
4.215964	-0.006	0.011	5.74E-01	0.003	0.016	8.62E-01
7.023494	-0.007	0.009	4.35E-01	-0.024	0.014	8.39E-02
8.091668	-0.004	0.008	6.47E-01	-0.004	0.012	7.54E-01
7.036952	-0.012	0.012	3.12E-01	-0.001	0.017	9.47E-01
7.634456	-0.005	0.010	6.16E-01	0.008	0.015	6.09E-01

6.505388	0.005	0.007	5.17E-01	-0.015	0.011	1.51E-01
7.945656	0.007	0.009	4.56E-01	0.013	0.014	3.56E-01
7.957095	-0.007	0.010	4.63E-01	0.012	0.015	4.37E-01
5.74034	-0.007	0.011	5.11E-01	0.003	0.016	8.50E-01
8.177794	0.003	0.008	7.33E-01	0.002	0.012	8.96E-01
7.483061	-0.009	0.010	3.65E-01	-0.020	0.015	1.80E-01
3.126259	-0.007	0.009	4.51E-01	0.010	0.014	4.55E-01
3.99661	-0.006	0.009	4.72E-01	0.018	0.013	1.66E-01
7.33133	-0.006	0.006	3.66E-01	-0.001	0.009	8.93E-01
6.263829	0.009	0.008	2.94E-01	-0.006	0.013	6.11E-01
3.265206	-0.006	0.008	4.45E-01	-0.001	0.011	9.47E-01
5.087661	0.005	0.008	5.41E-01	-0.010	0.011	3.91E-01
2.511260338	-0.005	0.014	7.38E-01	-0.016	0.021	4.53E-01
3.210367	-0.011	0.014	4.20E-01	0.001	0.020	9.44E-01
7.285912	-0.011	0.009	2.47E-01	-0.020	0.014	1.50E-01
1.047778566	-0.011	0.013	3.79E-01	-0.021	0.019	2.74E-01
5.717126	0.002	0.009	8.01E-01	-0.017	0.013	1.81E-01
7.38886	0.005	0.008	5.47E-01	-0.004	0.012	7.46E-01
6.649381	-0.007	0.010	4.99E-01	-0.011	0.015	4.48E-01
7.967861	0.003	0.009	7.61E-01	-0.004	0.014	7.77E-01
4.165836	-0.007	0.012	5.71E-01	-0.004	0.018	8.15E-01
7.161095	0.006	0.009	5.35E-01	0.000	0.014	9.99E-01
7.067903	-0.005	0.006	3.78E-01	0.001	0.009	9.34E-01
4.096867	-0.003	0.009	7.20E-01	0.007	0.013	6.19E-01
2.753491803	-0.007	0.007	3.40E-01	-0.018	0.011	9.86E-02
7.666417	0.008	0.011	4.63E-01	-0.001	0.016	9.41E-01
2.37265011	-0.003	0.007	6.54E-01	0.012	0.011	2.70E-01
6.118491	-0.005	0.009	5.86E-01	0.014	0.013	3.06E-01
6.45896	0.005	0.007	4.53E-01	0.012	0.010	2.67E-01
8.566038	-0.009	0.009	3.38E-01	-0.001	0.014	9.64E-01
6.15718	0.008	0.008	3.29E-01	0.023	0.012	6.31E-02
6.692108	0.006	0.009	4.61E-01	0.023	0.013	7.19E-02
8.66764	0.004	0.007	5.38E-01	0.010	0.010	3.37E-01
8.506489	-0.005	0.007	4.56E-01	-0.018	0.010	8.77E-02
6.16189	0.007	0.008	3.84E-01	0.005	0.012	6.85E-01
6.63155	-0.010	0.009	2.47E-01	0.009	0.013	5.11E-01
7.297687	-0.006	0.010	5.01E-01	0.022	0.014	1.22E-01
6.490249	0.005	0.008	5.29E-01	-0.018	0.013	1.45E-01
1.050470027	0.004	0.006	4.92E-01	-0.008	0.010	3.85E-01
2.83490849	-0.005	0.011	6.74E-01	-0.027	0.016	9.93E-02
6.388646	-0.003	0.009	7.37E-01	0.000	0.014	9.86E-01
1.225751407	0.004	0.008	5.91E-01	-0.005	0.012	6.81E-01
5.767928	0.002	0.009	8.60E-01	0.008	0.014	5.34E-01

1.049797162	-0.005	0.007	4.79E-01	-0.016	0.011	1.50E-01
0.743307071	-0.005	0.006	4.01E-01	-0.014	0.009	1.39E-01
4.366686	-0.009	0.015	5.47E-01	0.010	0.022	6.37E-01
5.476914	0.004	0.007	5.94E-01	0.001	0.011	9.29E-01
1.458899193	0.003	0.004	4.30E-01	0.001	0.005	9.10E-01
4.195778	-0.005	0.011	6.46E-01	0.026	0.016	1.09E-01
5.876596	0.008	0.010	4.42E-01	0.011	0.015	4.82E-01
0.659535356	-0.008	0.010	4.16E-01	-0.013	0.016	4.01E-01
7.845063	-0.006	0.010	5.33E-01	0.001	0.015	9.23E-01
8.87421	0.005	0.010	6.26E-01	0.001	0.015	9.53E-01
8.856043	0.003	0.008	6.70E-01	-0.009	0.012	4.85E-01
8.090322	-0.010	0.009	2.74E-01	-0.025	0.014	7.13E-02
2.854758	-0.004	0.008	6.58E-01	-0.007	0.012	5.87E-01
6.860997	0.007	0.010	5.09E-01	0.006	0.015	7.01E-01
4.178284	-0.008	0.014	5.89E-01	0.019	0.021	3.59E-01
7.981991	-0.005	0.008	5.80E-01	-0.003	0.012	8.36E-01
7.036279	0.001	0.012	9.29E-01	-0.009	0.018	6.20E-01
3.746977	0.005	0.009	5.80E-01	0.020	0.013	1.16E-01
2.918007	-0.005	0.009	6.22E-01	-0.005	0.014	7.38E-01
8.599008	-0.005	0.007	5.04E-01	0.015	0.010	1.45E-01
8.331544	0.008	0.010	4.12E-01	-0.005	0.015	7.13E-01
6.518509	0.008	0.011	4.33E-01	0.009	0.016	5.76E-01
7.247558	-0.008	0.010	3.99E-01	-0.014	0.015	3.58E-01
3.684401	-0.003	0.004	5.38E-01	0.008	0.007	2.03E-01
4.350201	-0.010	0.013	4.53E-01	0.016	0.019	4.04E-01
8.536095	0.008	0.008	3.21E-01	-0.003	0.012	8.17E-01
8.095032	0.006	0.009	4.89E-01	-0.002	0.013	9.01E-01
6.178039	-0.004	0.008	6.22E-01	-0.015	0.011	1.84E-01
5.012637	0.006	0.010	5.50E-01	-0.001	0.016	9.26E-01
1.805088329	-0.005	0.011	6.30E-01	0.013	0.016	4.12E-01
0.96905334	-0.003	0.005	5.11E-01	0.011	0.008	1.54E-01
4.217983	-0.006	0.011	5.81E-01	0.021	0.017	2.13E-01
8.428437	0.009	0.010	3.93E-01	0.018	0.015	2.42E-01
2.345062638	0.006	0.007	3.56E-01	0.007	0.010	4.68E-01
7.330321	-0.005	0.006	4.61E-01	-0.007	0.009	4.70E-01
6.011505	0.008	0.010	4.04E-01	0.004	0.015	7.92E-01
6.622467	-0.008	0.010	4.20E-01	0.000	0.015	9.75E-01
8.317414	0.005	0.008	5.58E-01	0.013	0.012	3.01E-01
5.02273	0.005	0.008	5.12E-01	-0.015	0.012	2.19E-01
2.080963054	-0.002	0.005	6.76E-01	0.006	0.007	4.17E-01
8.291172	-0.003	0.008	7.48E-01	-0.012	0.012	3.36E-01
3.512484	0.005	0.008	5.27E-01	0.024	0.012	4.09E-02
6.782272	-0.005	0.009	6.08E-01	-0.001	0.014	9.70E-01

8.856379	0.005	0.008	5.17E-01	0.000	0.012	9.73E-01
7.016429	0.001	0.008	9.33E-01	0.009	0.012	4.55E-01
3.763462	0.006	0.009	4.84E-01	0.012	0.013	3.79E-01
7.354544	-0.005	0.010	6.28E-01	-0.025	0.016	1.09E-01
8.520956	0.005	0.010	6.30E-01	0.002	0.015	9.12E-01
8.504134	0.008	0.009	3.82E-01	0.003	0.013	8.34E-01
8.107816	0.004	0.008	6.17E-01	-0.002	0.011	8.81E-01
0.77425887	0.004	0.005	4.21E-01	-0.002	0.007	7.39E-01
4.333043	-0.009	0.015	5.31E-01	0.019	0.022	3.83E-01
8.86479	0.003	0.009	7.36E-01	0.003	0.013	8.27E-01
3.754042	0.005	0.011	6.36E-01	0.011	0.016	5.17E-01
7.707125	-0.008	0.011	4.83E-01	-0.005	0.016	7.80E-01
8.504471	-0.003	0.009	7.76E-01	-0.004	0.014	7.67E-01
6.232541	0.004	0.006	5.76E-01	0.008	0.010	3.89E-01
6.555853	-0.004	0.008	6.03E-01	-0.012	0.011	2.66E-01
6.365432	0.004	0.009	6.80E-01	0.000	0.013	9.75E-01
5.286493	-0.007	0.008	4.24E-01	-0.026	0.013	4.17E-02
3.562276	-0.004	0.007	5.36E-01	0.023	0.010	2.62E-02
1.773800098	-0.006	0.010	5.09E-01	0.012	0.014	4.22E-01
6.714986	-0.005	0.009	5.87E-01	-0.038	0.013	2.95E-03
3.333838	0.006	0.009	5.24E-01	0.013	0.013	3.10E-01
8.684462	0.006	0.009	5.15E-01	0.005	0.013	6.89E-01
4.192078	-0.008	0.013	5.22E-01	0.013	0.019	4.99E-01
6.619102	-0.008	0.009	3.88E-01	-0.027	0.014	5.34E-02
6.191833	0.008	0.009	3.90E-01	-0.010	0.014	4.78E-01
7.844726	-0.007	0.010	4.90E-01	-0.004	0.014	7.85E-01
7.994102	0.007	0.008	4.05E-01	0.000	0.012	9.84E-01
8.217494	-0.004	0.006	5.70E-01	-0.009	0.009	3.61E-01
2.822460484	-0.010	0.015	5.03E-01	-0.069	0.023	2.33E-03
5.983245	0.007	0.009	4.70E-01	-0.008	0.014	5.62E-01
1.715933692	-0.003	0.005	5.46E-01	0.003	0.007	6.34E-01
6.705565	-0.005	0.008	5.38E-01	0.015	0.013	2.21E-01
5.452018	0.004	0.008	5.83E-01	-0.009	0.012	4.31E-01
7.936572	-0.003	0.010	7.68E-01	-0.008	0.015	5.88E-01
1.038022021	0.003	0.007	6.39E-01	0.008	0.010	4.10E-01
6.422289	-0.001	0.008	9.17E-01	-0.002	0.011	8.29E-01
5.903847	-0.001	0.006	8.80E-01	-0.014	0.009	1.16E-01
7.78686	-0.006	0.008	4.81E-01	-0.003	0.012	7.83E-01
3.992237	-0.006	0.010	5.16E-01	0.003	0.014	8.58E-01
1.456544164	0.002	0.004	6.63E-01	-0.001	0.005	8.37E-01
3.722081	0.004	0.007	5.75E-01	0.013	0.011	2.36E-01
0.971744801	-0.003	0.005	6.13E-01	0.012	0.008	1.09E-01
4.285606	0.008	0.011	4.29E-01	0.020	0.016	2.13E-01

2.090046734	-0.003	0.006	6.04E-01	0.016	0.008	6.26E-02
5.662288	-0.004	0.007	5.97E-01	0.002	0.011	8.29E-01
3.911829	0.004	0.008	6.43E-01	0.028	0.011	1.35E-02
7.058147	-0.005	0.009	5.54E-01	-0.017	0.014	2.10E-01
1.71559726	-0.003	0.005	5.49E-01	0.003	0.007	6.81E-01
8.305639	-0.007	0.008	3.84E-01	-0.008	0.012	5.21E-01
2.862832	-0.005	0.008	5.36E-01	-0.005	0.012	6.63E-01
8.579495	0.007	0.009	4.46E-01	-0.005	0.014	7.10E-01
2.089710301	-0.003	0.006	5.90E-01	0.017	0.009	4.28E-02
6.783618	-0.006	0.007	4.35E-01	-0.006	0.011	6.04E-01
8.637025	-0.004	0.006	5.12E-01	-0.008	0.009	3.33E-01
7.60956	0.013	0.017	4.36E-01	0.003	0.025	9.11E-01
2.547595057	-0.010	0.016	5.42E-01	-0.015	0.024	5.26E-01
4.01074	0.008	0.013	5.22E-01	0.018	0.019	3.44E-01
4.020833	-0.005	0.010	5.91E-01	0.007	0.015	6.50E-01
7.01071	-0.009	0.010	3.79E-01	-0.034	0.015	1.82E-02
1.714924394	-0.003	0.005	5.18E-01	0.005	0.007	4.45E-01
7.83867	-0.005	0.007	4.93E-01	0.015	0.010	1.20E-01
7.813774	0.005	0.010	6.37E-01	0.025	0.015	8.76E-02
8.288817	-0.007	0.009	4.07E-01	-0.030	0.013	2.52E-02
1.799368975	0.009	0.011	4.38E-01	0.010	0.016	5.27E-01
2.674766577	-0.008	0.009	3.68E-01	0.000	0.014	9.92E-01
8.898433	0.005	0.009	5.71E-01	0.004	0.014	7.57E-01
0.577445804	-0.003	0.009	6.91E-01	0.012	0.013	3.45E-01
6.589833	0.001	0.007	9.20E-01	-0.002	0.010	8.57E-01
8.426418	0.006	0.008	4.76E-01	0.009	0.012	4.68E-01
1.065945926	0.004	0.007	5.73E-01	-0.012	0.010	2.45E-01
8.012606	-0.008	0.010	4.21E-01	-0.034	0.015	1.97E-02
2.346071935	0.008	0.007	2.93E-01	0.018	0.011	1.08E-01
0.83010668	-0.007	0.008	3.68E-01	0.000	0.012	9.97E-01
5.552611	0.005	0.008	5.57E-01	-0.019	0.013	1.28E-01
6.485875	-0.009	0.010	3.29E-01	-0.022	0.014	1.22E-01
2.153968926	-0.004	0.009	6.06E-01	-0.006	0.013	6.28E-01
6.721041	-0.005	0.011	6.70E-01	-0.006	0.016	7.00E-01
6.859652	-0.006	0.008	4.66E-01	-0.004	0.012	7.58E-01
8.396476	0.007	0.008	3.85E-01	-0.011	0.012	3.81E-01
8.993644	0.003	0.009	7.30E-01	-0.003	0.013	8.13E-01
3.113475	-0.011	0.012	3.47E-01	0.017	0.018	3.51E-01
3.76279	0.006	0.009	5.07E-01	0.016	0.013	2.38E-01
2.542548569	0.021	0.035	5.53E-01	0.012	0.052	8.21E-01
5.043252	0.004	0.007	6.21E-01	-0.009	0.011	4.36E-01
8.565365	0.002	0.008	7.76E-01	-0.022	0.012	7.60E-02
7.343105	0.006	0.009	5.49E-01	0.011	0.014	4.42E-01

8.661248	0.006	0.008	4.81E-01	-0.007	0.012	5.87E-01
7.275819	-0.010	0.010	2.84E-01	0.003	0.014	8.22E-01
7.658006	0.006	0.010	5.19E-01	-0.013	0.015	3.70E-01
1.743857597	0.002	0.006	6.82E-01	0.007	0.009	4.53E-01
6.801449	0.005	0.010	6.31E-01	-0.018	0.015	2.29E-01
4.174583	-0.006	0.010	5.76E-01	0.004	0.015	7.78E-01
7.174889	-0.003	0.008	7.42E-01	-0.015	0.012	2.17E-01
2.342371177	0.007	0.006	2.73E-01	-0.005	0.010	5.90E-01
7.464221	0.006	0.008	4.99E-01	-0.007	0.012	5.70E-01
8.608765	0.002	0.006	7.92E-01	-0.003	0.010	7.59E-01
4.013432	-0.006	0.012	5.94E-01	-0.007	0.018	6.81E-01
8.997681	-0.004	0.007	6.07E-01	0.007	0.011	5.23E-01
6.347601	-0.003	0.008	7.34E-01	-0.018	0.011	1.06E-01
8.731226	0.007	0.010	5.22E-01	-0.001	0.015	9.39E-01
8.242053	-0.004	0.009	6.74E-01	0.007	0.014	6.03E-01
1.727035968	-0.002	0.005	6.29E-01	0.006	0.007	4.06E-01
7.551021	-0.004	0.009	6.23E-01	0.019	0.013	1.35E-01
2.539184243	-0.009	0.015	5.42E-01	-0.002	0.022	9.44E-01
7.747161	-0.005	0.010	6.34E-01	0.005	0.015	7.31E-01
6.18645	-0.007	0.010	4.53E-01	-0.006	0.014	6.82E-01
6.703547	-0.005	0.007	5.09E-01	-0.016	0.011	1.63E-01
7.650605	-0.001	0.006	8.13E-01	-0.005	0.009	5.62E-01
7.456819	-0.005	0.008	5.82E-01	-0.018	0.013	1.56E-01
7.322583	-0.005	0.006	3.84E-01	-0.004	0.009	6.57E-01
0.590566675	0.004	0.008	5.84E-01	0.015	0.012	2.00E-01
4.014778	-0.009	0.013	4.67E-01	0.019	0.019	2.97E-01
6.034382	0.004	0.010	6.85E-01	-0.001	0.014	9.21E-01
6.574357	0.004	0.008	6.41E-01	-0.008	0.012	5.10E-01
7.738077	-0.004	0.011	6.96E-01	0.006	0.016	7.23E-01
8.755786	0.006	0.010	5.10E-01	-0.001	0.014	9.50E-01
6.198898	-0.005	0.007	5.16E-01	0.007	0.010	5.29E-01
8.556618	0.004	0.008	5.84E-01	0.004	0.012	7.71E-01
8.489667	-0.004	0.008	5.83E-01	-0.017	0.012	1.45E-01
5.677764	0.008	0.009	3.87E-01	-0.023	0.014	9.65E-02
1.649656472	0.004	0.005	4.70E-01	0.003	0.007	6.58E-01
8.278388	0.003	0.007	6.91E-01	-0.015	0.011	1.75E-01
5.692903	0.005	0.008	5.32E-01	-0.002	0.012	8.81E-01
5.127697	0.004	0.008	5.61E-01	-0.003	0.011	7.64E-01
1.470337901	-0.002	0.006	7.33E-01	0.001	0.009	9.05E-01
1.416172253	0.005	0.004	2.83E-01	0.004	0.007	5.30E-01
0.683085637	0.009	0.012	4.91E-01	-0.016	0.019	3.89E-01
2.7245586	-0.007	0.009	4.00E-01	-0.006	0.013	6.70E-01
6.778908	0.007	0.008	3.85E-01	0.009	0.012	4.62E-01

6.954862	-0.003	0.007	7.03E-01	-0.006	0.010	5.51E-01
8.008232	0.004	0.008	6.71E-01	0.017	0.012	1.76E-01
2.636076829	-0.003	0.009	7.21E-01	-0.005	0.013	7.13E-01
1.978014681	0.004	0.005	4.77E-01	-0.002	0.007	7.53E-01
3.305241	-0.007	0.009	4.90E-01	0.002	0.014	9.14E-01
5.514258	0.002	0.008	8.37E-01	-0.006	0.012	6.09E-01
6.376534	-0.004	0.007	5.39E-01	0.002	0.010	8.63E-01
3.631244	-0.003	0.004	5.28E-01	0.004	0.006	4.54E-01
7.495509	0.004	0.008	6.46E-01	0.019	0.011	9.38E-02
6.872772	-0.005	0.009	5.95E-01	-0.012	0.013	3.54E-01
1.791967458	-0.005	0.009	5.72E-01	0.008	0.014	5.74E-01
3.935379	-0.001	0.006	9.21E-01	0.023	0.009	1.42E-02
4.30781	-0.011	0.016	4.93E-01	0.006	0.024	8.03E-01
0.752727184	-0.003	0.005	5.43E-01	0.002	0.008	7.87E-01
8.552917	-0.005	0.007	5.14E-01	0.001	0.011	8.97E-01
5.554966	0.005	0.008	4.84E-01	-0.010	0.011	3.77E-01
4.199479	-0.005	0.012	6.64E-01	0.026	0.018	1.37E-01
3.734529	-0.009	0.008	2.98E-01	0.001	0.013	9.36E-01
3.760771	0.005	0.008	5.49E-01	0.029	0.012	1.34E-02
5.059737	0.003	0.008	6.84E-01	-0.011	0.011	3.19E-01
0.658526058	-0.009	0.010	3.76E-01	-0.016	0.015	2.98E-01
8.896078	0.004	0.008	6.65E-01	0.003	0.013	7.84E-01
6.185777	0.007	0.010	5.27E-01	0.004	0.016	7.98E-01
6.139013	0.004	0.009	6.08E-01	-0.007	0.013	5.65E-01
8.2942	0.006	0.009	5.34E-01	-0.013	0.014	3.38E-01
1.748567654	-0.006	0.007	4.28E-01	0.010	0.011	3.45E-01
1.414826523	0.004	0.004	3.46E-01	0.003	0.006	5.87E-01
5.297259	0.008	0.013	5.04E-01	-0.023	0.019	2.13E-01
8.65923	0.003	0.009	7.38E-01	-0.031	0.014	2.44E-02
3.580107	-0.002	0.006	7.20E-01	0.009	0.009	3.46E-01
2.538174945	-0.009	0.014	4.97E-01	0.011	0.020	5.81E-01
7.253614	0.004	0.010	7.07E-01	-0.007	0.015	6.40E-01
6.488903	-0.004	0.008	6.11E-01	-0.001	0.012	9.08E-01
6.809523	-0.001	0.010	9.44E-01	-0.023	0.015	1.24E-01
7.152348	0.002	0.007	7.35E-01	-0.008	0.011	4.80E-01
1.440731833	0.004	0.004	3.55E-01	0.011	0.006	6.51E-02
5.562031	-0.005	0.010	6.28E-01	-0.020	0.015	1.71E-01
6.850568	-0.006	0.008	4.59E-01	-0.019	0.012	1.27E-01
7.474314	0.007	0.008	3.99E-01	-0.001	0.012	9.33E-01
7.66709	-0.007	0.010	4.66E-01	0.016	0.015	2.68E-01
5.792487	-0.008	0.010	4.45E-01	0.005	0.015	7.14E-01
5.823439	0.005	0.007	4.82E-01	0.014	0.011	2.05E-01
2.089373868	-0.003	0.006	5.59E-01	0.017	0.009	5.07E-02

1.80777979	-0.007	0.011	5.03E-01	-0.007	0.016	6.60E-01
1.844787375	-0.005	0.009	5.95E-01	0.007	0.014	6.06E-01
1.691710546	-0.003	0.004	4.54E-01	0.005	0.006	3.33E-01
7.90192	-0.004	0.009	6.26E-01	-0.008	0.013	5.63E-01
1.316924639	0.011	0.009	2.13E-01	-0.001	0.013	9.59E-01
8.949571	-0.007	0.009	4.28E-01	-0.024	0.013	7.29E-02
8.591943	0.005	0.008	5.46E-01	0.003	0.012	8.11E-01
8.863781	0.004	0.009	6.59E-01	-0.010	0.013	4.36E-01
6.616074	0.007	0.008	4.22E-01	0.005	0.012	6.62E-01
8.998017	-0.006	0.010	5.50E-01	-0.012	0.015	3.92E-01
6.193851	0.005	0.007	4.71E-01	-0.008	0.011	4.71E-01
3.302886	-0.005	0.012	6.94E-01	0.008	0.018	6.61E-01
6.627177	0.005	0.009	6.10E-01	-0.017	0.014	2.23E-01
3.691802	-0.002	0.005	6.33E-01	0.010	0.007	1.45E-01
8.341974	-0.005	0.010	5.86E-01	-0.006	0.015	6.59E-01
1.506672621	0.004	0.006	5.68E-01	-0.010	0.009	2.70E-01
3.518876	0.004	0.007	5.22E-01	0.026	0.010	1.07E-02
7.567506	0.002	0.007	7.57E-01	-0.001	0.010	8.81E-01
8.108153	-0.005	0.007	5.05E-01	-0.001	0.010	9.33E-01
4.37005	-0.008	0.014	5.49E-01	0.023	0.021	2.63E-01
7.106257	0.008	0.008	3.34E-01	0.004	0.012	7.25E-01
1.728718131	-0.003	0.005	5.35E-01	0.005	0.007	4.86E-01
6.777226	0.007	0.008	3.86E-01	0.001	0.012	9.57E-01
3.113138	-0.011	0.012	3.49E-01	0.010	0.018	5.77E-01
1.746212625	-0.003	0.007	7.08E-01	0.006	0.010	5.83E-01
1.232816491	0.004	0.008	6.02E-01	-0.008	0.012	4.99E-01
7.927489	-0.007	0.009	4.22E-01	-0.010	0.013	4.66E-01
2.153296061	-0.005	0.008	5.43E-01	-0.001	0.013	9.21E-01
0.99630438	0.004	0.006	5.37E-01	0.011	0.009	2.21E-01
6.084511	0.005	0.008	5.04E-01	-0.003	0.012	7.83E-01
7.81512	-0.007	0.010	4.78E-01	0.011	0.015	4.60E-01
5.99031	0.005	0.007	4.08E-01	-0.001	0.010	9.48E-01
5.479942	0.005	0.006	4.66E-01	0.004	0.010	6.45E-01
8.368215	0.008	0.008	3.53E-01	0.006	0.012	6.30E-01
7.281538	-0.007	0.010	5.07E-01	0.003	0.016	8.45E-01
7.824877	-0.012	0.009	2.17E-01	0.020	0.014	1.42E-01
3.233581	-0.004	0.006	5.35E-01	0.010	0.009	2.57E-01
6.81827	0.001	0.010	9.09E-01	0.008	0.015	5.84E-01
6.006122	0.004	0.006	4.85E-01	0.014	0.009	1.22E-01
3.183453	0.005	0.005	3.93E-01	0.016	0.008	4.33E-02
3.370846	0.014	0.012	2.47E-01	0.021	0.018	2.54E-01
1.066282359	0.003	0.007	6.28E-01	-0.008	0.010	4.27E-01
8.31775	0.006	0.009	5.31E-01	0.012	0.013	3.74E-01

6.797412	-0.004	0.010	7.28E-01	0.001	0.015	9.56E-01
6.338517	0.003	0.008	7.13E-01	0.013	0.012	2.93E-01
4.389227	-0.007	0.013	5.76E-01	0.015	0.020	4.56E-01
3.800134	0.005	0.006	4.02E-01	0.008	0.009	3.80E-01
1.973304624	0.003	0.005	5.60E-01	-0.005	0.007	5.01E-01
4.227403	0.006	0.010	5.63E-01	0.018	0.015	2.45E-01
3.153847	-0.008	0.009	4.24E-01	0.006	0.014	6.75E-01
8.013279	-0.008	0.009	3.67E-01	0.000	0.013	9.76E-01
5.141827	-0.005	0.008	5.43E-01	-0.019	0.011	9.68E-02
6.705229	-0.004	0.007	6.19E-01	-0.004	0.011	7.11E-01
2.619591632	-0.010	0.013	4.77E-01	0.023	0.020	2.50E-01
1.814844874	-0.005	0.010	6.31E-01	-0.004	0.016	7.91E-01
0.501075606	-0.006	0.010	5.67E-01	-0.034	0.015	2.30E-02
8.326834	-0.004	0.010	6.63E-01	-0.017	0.014	2.40E-01
7.593075	0.006	0.009	4.58E-01	0.002	0.013	8.75E-01
7.880724	0.005	0.009	5.73E-01	0.012	0.014	3.84E-01
7.10592	0.007	0.010	4.44E-01	0.009	0.014	5.51E-01
5.152929	0.005	0.008	5.09E-01	-0.017	0.012	1.64E-01
2.613535845	-0.009	0.013	4.94E-01	0.048	0.020	1.43E-02
3.295821	-0.006	0.011	5.82E-01	-0.013	0.016	4.09E-01
8.863108	0.005	0.007	5.11E-01	0.006	0.011	6.02E-01
1.467982873	0.009	0.008	2.73E-01	-0.008	0.012	5.12E-01
1.716270125	-0.003	0.005	5.58E-01	0.001	0.007	8.43E-01
7.543955	-0.005	0.010	5.99E-01	0.000	0.015	9.85E-01
6.309584	0.002	0.007	7.57E-01	-0.007	0.010	4.78E-01
7.317873	-0.006	0.006	3.06E-01	-0.006	0.009	4.87E-01
5.671372	0.003	0.007	6.95E-01	-0.012	0.011	2.98E-01
8.945197	0.004	0.009	6.48E-01	-0.018	0.013	1.83E-01
5.286829	-0.007	0.009	4.41E-01	-0.026	0.013	4.44E-02
8.491686	-0.007	0.010	4.83E-01	-0.034	0.014	1.74E-02
1.827629312	-0.009	0.010	3.69E-01	0.003	0.014	8.58E-01
2.089037436	-0.003	0.006	5.72E-01	0.014	0.009	1.15E-01
6.479819	0.001	0.007	8.66E-01	-0.004	0.011	7.38E-01
6.449877	0.004	0.007	5.87E-01	0.005	0.010	6.20E-01
8.40926	0.004	0.007	5.76E-01	0.007	0.010	4.72E-01
7.037288	-0.011	0.011	3.15E-01	0.006	0.017	7.24E-01
7.508294	-0.003	0.009	7.56E-01	-0.016	0.013	2.21E-01
5.966423	-0.004	0.009	6.48E-01	-0.010	0.013	4.45E-01
6.793374	-0.002	0.010	8.26E-01	-0.010	0.015	4.97E-01
3.760435	0.004	0.008	5.76E-01	0.027	0.012	2.37E-02
8.645772	-0.006	0.011	5.97E-01	0.014	0.016	3.84E-01
7.076314	0.006	0.009	4.62E-01	0.009	0.013	4.75E-01
8.045913	0.004	0.008	5.95E-01	0.004	0.011	7.19E-01

1.75462344	0.004	0.007	6.07E-01	0.013	0.011	2.41E-01
7.878706	-0.005	0.008	5.60E-01	-0.002	0.013	8.97E-01
2.645160509	-0.008	0.010	4.09E-01	0.020	0.015	1.65E-01
4.308483	-0.010	0.014	4.80E-01	0.010	0.021	6.15E-01
7.464557	0.003	0.008	7.06E-01	0.008	0.013	5.46E-01
0.661553952	-0.004	0.010	6.41E-01	-0.007	0.014	6.36E-01
5.07454	0.007	0.009	4.08E-01	-0.011	0.013	3.78E-01
7.126106	0.007	0.008	3.95E-01	0.001	0.012	9.24E-01
2.151277465	-0.005	0.007	4.81E-01	-0.007	0.011	5.17E-01
3.772546	0.002	0.010	8.23E-01	0.024	0.014	1.03E-01
0.556923416	-0.004	0.008	6.31E-01	0.012	0.011	2.75E-01
6.719696	-0.004	0.008	6.05E-01	-0.002	0.012	8.73E-01
1.448469782	0.002	0.004	5.41E-01	0.005	0.006	4.03E-01
0.520588696	-0.009	0.010	3.54E-01	-0.012	0.015	4.28E-01
1.757314901	0.006	0.008	4.42E-01	0.015	0.012	2.22E-01
3.339221	-0.004	0.008	6.16E-01	0.012	0.013	3.28E-01
5.926388	-0.006	0.008	4.39E-01	-0.005	0.012	6.89E-01
5.482297	0.005	0.008	5.43E-01	0.007	0.012	5.65E-01
3.733184	0.002	0.008	8.38E-01	0.009	0.012	4.74E-01
6.259119	-0.005	0.007	4.63E-01	-0.010	0.010	3.30E-01
1.000005138	0.004	0.007	5.18E-01	0.006	0.010	5.60E-01
5.149565	0.005	0.007	4.92E-01	-0.011	0.010	2.58E-01
8.229269	-0.005	0.008	4.99E-01	-0.023	0.012	5.11E-02
0.972081233	-0.002	0.005	6.34E-01	0.013	0.007	7.71E-02
7.593411	-0.004	0.009	6.92E-01	0.008	0.014	5.72E-01
2.788480793	-0.007	0.012	5.40E-01	-0.048	0.017	5.43E-03
1.750249816	-0.005	0.007	4.22E-01	0.004	0.010	7.03E-01
6.150788	0.006	0.010	5.65E-01	0.005	0.015	7.20E-01
2.867206	-0.003	0.008	7.03E-01	-0.001	0.013	9.56E-01
7.030559	-0.006	0.010	5.61E-01	0.001	0.015	9.34E-01
7.179599	0.006	0.009	4.91E-01	-0.007	0.013	5.85E-01
8.963701	-0.004	0.007	5.76E-01	0.005	0.011	6.21E-01
5.955657	0.005	0.008	5.31E-01	0.005	0.012	7.15E-01
2.330596036	0.004	0.006	4.59E-01	-0.012	0.008	1.50E-01
7.626718	-0.009	0.012	4.70E-01	-0.004	0.019	8.33E-01
8.665622	0.004	0.007	6.31E-01	0.005	0.011	6.67E-01
7.363291	0.007	0.009	4.30E-01	-0.004	0.013	7.75E-01
5.019702	0.005	0.011	6.74E-01	-0.014	0.016	3.86E-01
7.294995	-0.002	0.011	8.24E-01	-0.008	0.016	6.03E-01
7.258997	-0.007	0.012	5.17E-01	0.004	0.017	8.34E-01
8.285116	-0.007	0.009	4.65E-01	-0.003	0.014	8.18E-01
6.77117	0.007	0.010	4.84E-01	-0.019	0.014	1.78E-01
7.578272	-0.005	0.010	5.93E-01	-0.001	0.015	9.33E-01

6.311939	0.007	0.010	4.83E-01	-0.005	0.015	7.24E-01
1.131213849	0.007	0.007	3.61E-01	-0.010	0.011	3.64E-01
0.513523611	-0.004	0.007	5.05E-01	-0.009	0.010	3.57E-01
2.778387815	-0.008	0.012	4.83E-01	-0.044	0.017	1.10E-02
5.002207	0.007	0.010	4.90E-01	-0.005	0.014	7.04E-01
2.342034744	0.007	0.007	2.92E-01	-0.008	0.010	4.21E-01
5.064447	0.003	0.007	6.43E-01	-0.008	0.011	4.79E-01
4.394274	-0.009	0.014	5.25E-01	0.013	0.021	5.30E-01
5.408954	0.006	0.008	4.25E-01	-0.007	0.012	5.52E-01
2.658954245	-0.004	0.008	6.29E-01	0.012	0.013	3.38E-01
2.753828236	-0.007	0.007	3.51E-01	-0.018	0.011	9.31E-02
7.375403	-0.005	0.008	4.90E-01	-0.003	0.011	7.81E-01
7.242848	0.002	0.008	8.23E-01	-0.009	0.012	4.34E-01
1.197827502	0.004	0.008	6.20E-01	0.013	0.011	2.37E-01
7.66238	0.005	0.009	5.80E-01	0.000	0.014	9.96E-01
2.647515537	-0.012	0.018	5.06E-01	0.022	0.026	4.00E-01
4.00603	0.004	0.010	6.71E-01	0.022	0.016	1.63E-01
6.996243	0.007	0.008	3.93E-01	-0.005	0.011	6.56E-01
1.749240519	-0.006	0.007	4.12E-01	0.006	0.011	5.98E-01
8.470827	-0.003	0.008	7.52E-01	0.004	0.013	7.63E-01
8.733918	0.001	0.008	9.00E-01	-0.012	0.012	3.20E-01
3.123568	-0.007	0.008	3.68E-01	0.000	0.012	9.84E-01
3.562612	-0.003	0.007	6.00E-01	0.021	0.010	3.75E-02
0.802519207	0.001	0.006	9.10E-01	0.007	0.009	4.49E-01
5.819066	0.002	0.008	8.15E-01	0.003	0.012	8.13E-01
5.919322	-0.006	0.008	4.60E-01	-0.003	0.012	7.89E-01
8.91761	-0.005	0.007	4.88E-01	-0.012	0.011	2.56E-01
2.839954979	-0.002	0.009	8.66E-01	0.000	0.014	9.83E-01
8.249118	0.003	0.008	6.95E-01	0.011	0.011	3.51E-01
3.782976	-0.010	0.012	3.96E-01	-0.003	0.017	8.75E-01
0.739606313	-0.005	0.007	4.68E-01	0.008	0.010	4.16E-01
4.176265	-0.008	0.011	4.81E-01	0.010	0.016	5.41E-01
7.200458	0.007	0.009	4.67E-01	-0.008	0.014	5.77E-01
5.55362	0.006	0.008	4.40E-01	-0.012	0.012	3.22E-01
5.704342	-0.003	0.009	7.26E-01	-0.024	0.013	6.55E-02
8.12632	-0.004	0.011	7.08E-01	0.004	0.016	8.03E-01
5.488352	0.004	0.008	6.11E-01	-0.022	0.012	5.32E-02
7.12005	-0.007	0.010	4.91E-01	-0.011	0.015	4.53E-01
2.659290678	-0.005	0.009	6.09E-01	0.009	0.013	5.07E-01
1.793313188	-0.005	0.010	6.16E-01	-0.006	0.015	6.81E-01
8.146506	0.004	0.007	5.43E-01	-0.001	0.010	9.27E-01
0.734896256	-0.004	0.007	5.42E-01	-0.006	0.010	5.45E-01
8.634333	0.003	0.006	6.42E-01	-0.012	0.009	1.95E-01

6.852586	-0.005	0.009	5.33E-01	-0.015	0.013	2.37E-01
8.003186	-0.002	0.006	7.06E-01	-0.012	0.009	1.50E-01
2.090719599	-0.003	0.005	5.96E-01	0.012	0.008	1.35E-01
3.797442	0.006	0.006	3.57E-01	0.014	0.009	1.09E-01
4.303773	-0.008	0.014	5.56E-01	0.013	0.021	5.47E-01
3.650758	-0.002	0.004	5.93E-01	0.009	0.007	1.54E-01
8.364515	0.007	0.010	4.55E-01	-0.003	0.014	8.36E-01
5.080932	0.006	0.009	4.82E-01	-0.009	0.013	4.94E-01
3.194218	0.003	0.006	6.16E-01	0.013	0.009	1.32E-01
2.088701003	-0.003	0.006	5.73E-01	0.013	0.009	1.42E-01
3.802489	0.005	0.006	3.86E-01	0.011	0.008	1.71E-01
1.745876193	-0.003	0.007	6.81E-01	0.001	0.010	9.01E-01
4.296035	-0.006	0.012	6.26E-01	0.004	0.019	8.27E-01
0.857694152	0.006	0.008	4.88E-01	-0.006	0.012	6.19E-01
3.276308	-0.009	0.012	4.53E-01	0.012	0.018	4.92E-01
6.958899	-0.007	0.008	4.09E-01	0.000	0.012	9.74E-01
5.596347	0.003	0.008	7.45E-01	-0.008	0.013	5.46E-01
0.675011255	0.006	0.010	5.84E-01	-0.008	0.015	5.76E-01
5.070503	0.003	0.007	6.80E-01	-0.003	0.010	7.95E-01
6.196543	-0.004	0.008	6.25E-01	-0.010	0.011	3.99E-01
5.040897	0.005	0.009	5.67E-01	0.001	0.013	9.59E-01
5.641766	0.003	0.007	6.28E-01	-0.003	0.010	8.02E-01
7.099192	-0.005	0.009	5.51E-01	-0.010	0.013	4.46E-01
7.457829	0.004	0.008	6.14E-01	0.018	0.012	1.28E-01
8.641399	0.002	0.007	8.03E-01	-0.026	0.011	1.73E-02
7.198103	0.005	0.009	5.94E-01	-0.003	0.013	8.09E-01
6.096286	0.006	0.010	5.23E-01	0.013	0.014	3.55E-01
1.066618791	0.002	0.007	7.48E-01	-0.002	0.010	8.12E-01
8.869163	-0.005	0.008	5.53E-01	0.011	0.012	3.61E-01
2.099466846	-0.002	0.009	8.43E-01	-0.002	0.013	9.04E-01
4.174247	-0.006	0.011	5.63E-01	0.000	0.017	9.98E-01
6.520864	0.006	0.009	4.91E-01	0.013	0.014	3.40E-01
8.732572	0.004	0.009	6.52E-01	-0.001	0.013	9.37E-01
2.722540005	-0.006	0.009	4.77E-01	-0.001	0.013	9.65E-01
7.524106	0.006	0.008	5.06E-01	-0.019	0.012	1.32E-01
3.321726	0.006	0.011	6.18E-01	0.032	0.017	5.44E-02
4.136566	-0.007	0.013	5.83E-01	0.015	0.020	4.43E-01
7.134517	0.005	0.009	5.78E-01	-0.033	0.013	1.11E-02
2.752146073	-0.006	0.007	3.99E-01	-0.015	0.011	1.68E-01
4.006367	0.004	0.011	6.92E-01	0.022	0.017	1.88E-01
3.310961	-0.010	0.012	4.03E-01	0.006	0.018	7.23E-01
6.797075	0.008	0.010	4.25E-01	0.013	0.014	3.56E-01
2.850720822	-0.003	0.008	6.91E-01	0.002	0.011	8.88E-01

8.724161	-0.006	0.009	5.26E-01	-0.013	0.013	3.39E-01
2.083654514	-0.003	0.007	6.28E-01	0.010	0.010	3.14E-01
3.877177	-0.003	0.004	5.19E-01	0.014	0.007	3.51E-02
8.361487	-0.003	0.009	7.15E-01	-0.003	0.014	8.09E-01
4.194096	-0.005	0.011	6.09E-01	0.020	0.016	2.10E-01
7.233428	-0.008	0.011	4.71E-01	-0.021	0.016	1.94E-01
6.176357	0.006	0.007	4.43E-01	-0.014	0.011	1.96E-01
0.561297039	-0.005	0.008	4.98E-01	0.000	0.012	9.93E-01
4.132865	-0.007	0.013	5.52E-01	0.010	0.019	5.93E-01
3.301877	-0.005	0.011	6.73E-01	0.011	0.017	5.31E-01
7.459174	0.008	0.009	3.94E-01	0.007	0.014	6.12E-01
3.642683	-0.002	0.004	6.08E-01	0.009	0.006	1.18E-01
8.676051	0.006	0.009	5.05E-01	-0.026	0.013	5.83E-02
6.267867	0.011	0.010	2.36E-01	0.025	0.014	8.26E-02
6.422626	-0.001	0.008	9.21E-01	-0.003	0.011	8.01E-01
4.191068	-0.006	0.014	6.68E-01	0.011	0.021	5.83E-01
8.089649	0.002	0.008	7.77E-01	-0.006	0.012	6.20E-01
8.047259	0.003	0.008	7.17E-01	-0.004	0.012	7.76E-01
3.979452	-0.006	0.013	6.54E-01	0.001	0.020	9.47E-01
6.70792	-0.009	0.010	3.88E-01	-0.027	0.015	7.90E-02
0.971071935	-0.003	0.005	6.06E-01	0.010	0.008	1.81E-01
1.067291656	0.003	0.006	6.67E-01	-0.010	0.010	3.03E-01
1.88179496	-0.004	0.007	6.38E-01	-0.002	0.011	8.85E-01
2.612190115	-0.009	0.013	4.56E-01	0.036	0.019	5.17E-02
1.327690482	0.010	0.009	2.82E-01	0.005	0.014	7.12E-01
2.079953756	-0.001	0.004	8.47E-01	0.008	0.006	1.88E-01
6.02328	0.007	0.009	4.20E-01	-0.001	0.013	9.34E-01
5.586927	0.006	0.008	4.66E-01	-0.022	0.012	6.84E-02
8.796494	-0.002	0.009	8.26E-01	0.005	0.013	7.34E-01
7.59442	-0.005	0.009	5.83E-01	0.000	0.013	9.72E-01
4.000647	0.005	0.010	5.91E-01	0.007	0.014	6.07E-01
6.612374	0.005	0.008	5.92E-01	-0.003	0.013	7.87E-01
3.31197	-0.008	0.010	3.91E-01	0.009	0.014	5.17E-01
6.160545	0.005	0.008	5.50E-01	0.006	0.012	6.36E-01
6.405468	-0.008	0.011	4.80E-01	-0.001	0.016	9.54E-01
7.339741	0.004	0.011	7.35E-01	-0.024	0.016	1.32E-01
0.583838023	0.007	0.008	4.03E-01	0.014	0.012	2.41E-01
1.142988989	0.006	0.007	3.46E-01	-0.002	0.010	8.65E-01
0.791753364	-0.004	0.005	3.74E-01	0.002	0.007	7.73E-01
8.461744	0.002	0.008	8.00E-01	-0.007	0.012	5.46E-01
6.44012	0.004	0.007	5.90E-01	0.011	0.010	2.96E-01
4.424552	-0.008	0.014	5.76E-01	0.010	0.021	6.25E-01
4.237496	0.008	0.012	5.38E-01	0.002	0.018	9.20E-01

3.555884	-0.014	0.028	6.06E-01	-0.035	0.042	3.98E-01
1.732082457	-0.005	0.005	3.62E-01	0.002	0.008	7.97E-01
6.94275	0.005	0.009	5.31E-01	0.010	0.013	4.33E-01
8.734254	-0.006	0.007	4.37E-01	-0.002	0.011	8.31E-01
7.431251	0.005	0.011	6.50E-01	-0.003	0.016	8.36E-01
6.934676	-0.005	0.008	5.94E-01	0.004	0.013	7.48E-01
0.829770247	-0.007	0.008	3.83E-01	0.001	0.012	9.63E-01
5.450672	0.006	0.009	4.64E-01	-0.003	0.013	8.11E-01
6.954189	-0.003	0.007	6.89E-01	-0.005	0.010	6.22E-01
3.999302	0.005	0.010	6.33E-01	0.015	0.015	3.24E-01
2.160361145	-0.005	0.008	5.41E-01	-0.006	0.013	6.59E-01
7.404336	-0.004	0.010	7.14E-01	0.036	0.016	2.21E-02
3.33081	-0.006	0.010	5.72E-01	0.014	0.015	3.58E-01
6.630877	0.005	0.010	6.42E-01	0.017	0.015	2.57E-01
6.548788	-0.004	0.010	6.82E-01	0.016	0.015	2.76E-01
1.753614142	-0.005	0.007	5.19E-01	0.015	0.011	1.65E-01
8.866136	0.004	0.009	6.32E-01	0.001	0.014	9.30E-01
3.64302	-0.002	0.004	6.37E-01	0.009	0.006	1.19E-01
4.180302	-0.007	0.013	6.07E-01	0.015	0.020	4.56E-01
8.170729	0.006	0.009	5.26E-01	0.019	0.013	1.47E-01
0.722111818	0.005	0.007	4.80E-01	-0.001	0.011	9.13E-01
4.393937	-0.008	0.014	5.63E-01	0.014	0.021	5.05E-01
0.990921458	0.002	0.007	8.06E-01	0.015	0.011	1.69E-01
2.818759726	-0.010	0.016	5.29E-01	-0.078	0.024	1.18E-03
8.27536	0.004	0.008	6.16E-01	-0.001	0.012	9.14E-01
7.463212	0.005	0.010	6.35E-01	-0.018	0.015	2.42E-01
4.377788	-0.008	0.015	5.89E-01	0.021	0.022	3.32E-01
6.017897	0.004	0.008	6.30E-01	0.001	0.012	9.29E-01
6.035392	0.005	0.009	5.32E-01	-0.020	0.013	1.28E-01
4.208226	-0.005	0.013	7.22E-01	0.009	0.019	6.31E-01
6.763432	0.009	0.011	4.06E-01	-0.003	0.016	8.51E-01
6.361395	0.007	0.008	4.40E-01	0.008	0.013	5.51E-01
5.582553	0.007	0.008	3.83E-01	0.015	0.012	2.05E-01
2.661645706	-0.013	0.018	4.60E-01	-0.017	0.026	5.08E-01
6.351638	0.004	0.008	6.48E-01	-0.005	0.012	6.86E-01
2.323530952	-0.003	0.006	5.89E-01	-0.009	0.009	3.42E-01
0.57172645	-0.003	0.008	7.14E-01	0.017	0.013	1.71E-01
2.362893565	0.005	0.008	5.01E-01	0.019	0.011	9.85E-02
5.943546	0.006	0.008	4.61E-01	0.014	0.012	2.56E-01
6.681342	0.006	0.010	5.56E-01	0.009	0.015	5.46E-01
8.421035	0.003	0.008	7.18E-01	0.007	0.011	5.61E-01
1.713915097	-0.003	0.005	5.82E-01	0.007	0.007	3.10E-01
6.737863	0.004	0.010	6.84E-01	-0.008	0.014	5.90E-01

7.904948	0.003	0.010	7.91E-01	-0.005	0.015	7.55E-01
1.143325422	0.006	0.007	3.43E-01	-0.005	0.010	6.31E-01
3.15351	-0.008	0.010	4.33E-01	0.002	0.014	8.85E-01
6.486211	0.001	0.010	9.45E-01	-0.001	0.015	9.19E-01
1.767071446	-0.003	0.010	7.57E-01	0.008	0.015	6.06E-01
5.663297	0.005	0.009	5.93E-01	0.004	0.013	7.54E-01
4.182994	-0.006	0.014	6.54E-01	0.001	0.021	9.60E-01
5.000525	0.005	0.009	6.01E-01	-0.003	0.013	8.30E-01
7.773066	-0.008	0.012	4.99E-01	-0.012	0.018	5.16E-01
0.520252263	0.006	0.010	5.64E-01	-0.001	0.015	9.45E-01
1.226087839	0.004	0.008	6.21E-01	-0.005	0.012	6.54E-01
8.00655	0.004	0.008	6.43E-01	-0.015	0.013	2.29E-01
8.508171	-0.005	0.009	5.72E-01	0.014	0.014	3.08E-01
8.728871	0.005	0.009	5.71E-01	-0.023	0.014	9.81E-02
6.266184	0.007	0.009	4.67E-01	-0.005	0.013	6.99E-01
7.250586	-0.008	0.010	4.14E-01	-0.003	0.015	8.64E-01
7.188346	-0.005	0.010	6.43E-01	0.000	0.015	9.84E-01
8.977831	-0.004	0.009	6.48E-01	-0.007	0.013	5.90E-01
8.875556	0.006	0.007	3.89E-01	0.009	0.011	4.34E-01
5.989973	0.006	0.010	5.31E-01	-0.004	0.014	7.72E-01
2.328913873	-0.001	0.005	7.67E-01	-0.013	0.008	8.61E-02
8.218839	-0.004	0.007	5.21E-01	-0.001	0.010	9.54E-01
7.911003	-0.006	0.008	4.80E-01	0.000	0.012	9.75E-01
8.597999	0.004	0.007	6.07E-01	-0.007	0.011	4.97E-01
3.80047	0.005	0.006	4.00E-01	0.009	0.009	3.24E-01
8.616503	-0.007	0.008	3.89E-01	-0.005	0.011	6.74E-01
2.669047223	-0.005	0.005	3.23E-01	0.009	0.007	1.77E-01
4.187704	-0.008	0.015	6.00E-01	0.015	0.023	5.17E-01
6.702874	-0.004	0.008	6.00E-01	-0.015	0.012	2.15E-01
8.417334	0.003	0.006	5.63E-01	-0.006	0.009	5.36E-01
6.588487	0.002	0.007	8.22E-01	-0.004	0.011	7.09E-01
4.24086	0.009	0.013	4.93E-01	-0.010	0.019	6.18E-01
6.61271	0.003	0.007	6.64E-01	-0.009	0.011	4.33E-01
1.789948862	-0.003	0.012	7.85E-01	-0.014	0.017	4.30E-01
0.547839736	0.007	0.011	5.24E-01	0.012	0.016	4.52E-01
2.353809885	0.009	0.009	3.27E-01	0.015	0.013	2.35E-01
6.795393	0.010	0.009	2.59E-01	0.003	0.013	8.34E-01
3.053926	-0.004	0.009	6.49E-01	0.009	0.014	5.17E-01
5.943209	0.007	0.009	4.20E-01	0.007	0.013	5.96E-01
4.102586	-0.002	0.011	8.58E-01	0.009	0.016	5.85E-01
1.459235625	0.002	0.003	4.84E-01	0.000	0.005	9.80E-01
4.043711	0.011	0.013	3.93E-01	-0.004	0.020	8.22E-01
8.719787	0.004	0.007	5.99E-01	0.004	0.011	6.92E-01

6.664857	-0.002	0.010	8.71E-01	0.008	0.015	6.13E-01
1.143661855	0.006	0.006	3.49E-01	-0.004	0.009	6.59E-01
8.630969	0.005	0.009	6.04E-01	0.003	0.013	8.22E-01
5.638065	0.006	0.009	4.96E-01	-0.001	0.013	9.42E-01
6.1195	0.003	0.006	6.39E-01	0.008	0.009	3.50E-01
1.232480059	0.004	0.008	6.25E-01	-0.008	0.012	5.02E-01
8.207737	0.003	0.006	6.47E-01	0.005	0.009	5.60E-01
8.055669	0.002	0.008	7.81E-01	-0.005	0.012	6.73E-01
8.194616	-0.006	0.008	4.67E-01	-0.004	0.013	7.79E-01
3.638646	0.004	0.004	3.28E-01	0.012	0.007	6.56E-02
7.47936	0.005	0.009	5.47E-01	-0.005	0.014	7.04E-01
4.183667	-0.007	0.015	6.34E-01	0.007	0.023	7.51E-01
5.287166	-0.006	0.009	4.58E-01	-0.026	0.013	4.20E-02
1.029947639	0.002	0.007	7.43E-01	0.021	0.011	5.02E-02
0.969389772	-0.003	0.005	5.19E-01	0.011	0.008	1.68E-01
0.599313922	-0.006	0.008	4.73E-01	-0.013	0.012	2.92E-01
7.286585	-0.008	0.010	3.96E-01	0.008	0.015	5.71E-01
4.110661	-0.004	0.010	6.73E-01	0.002	0.015	8.70E-01
7.923451	-0.003	0.007	7.11E-01	-0.002	0.010	8.36E-01
7.623354	-0.012	0.013	3.32E-01	0.009	0.019	6.32E-01
3.880205	0.003	0.005	6.12E-01	0.020	0.007	7.78E-03
1.833012234	-0.009	0.009	3.51E-01	0.006	0.014	6.48E-01
0.779305358	0.002	0.004	6.72E-01	-0.001	0.007	9.35E-01
1.715260827	-0.003	0.005	5.67E-01	0.005	0.007	4.86E-01
7.558759	0.005	0.009	5.83E-01	0.002	0.013	9.00E-01
4.23716	0.007	0.013	5.64E-01	0.010	0.019	5.84E-01
5.808636	0.002	0.009	8.26E-01	-0.023	0.013	8.65E-02
7.207523	0.000	0.007	9.96E-01	-0.003	0.011	7.94E-01
0.533373134	-0.005	0.007	5.04E-01	-0.016	0.011	1.26E-01
8.192261	0.006	0.009	5.05E-01	0.009	0.014	5.38E-01
6.235569	0.005	0.007	4.25E-01	-0.002	0.010	8.21E-01
8.581177	0.004	0.009	6.03E-01	0.004	0.013	7.79E-01
4.135893	-0.005	0.012	6.48E-01	0.010	0.017	5.76E-01
3.179752	-0.002	0.005	6.78E-01	0.011	0.008	1.45E-01
6.827018	-0.003	0.007	6.63E-01	0.004	0.010	7.18E-01
8.807596	-0.006	0.007	3.89E-01	-0.026	0.011	1.61E-02
0.590230242	0.002	0.007	7.92E-01	0.008	0.010	4.09E-01
1.891887937	-0.002	0.007	7.27E-01	-0.016	0.011	1.34E-01
0.819677269	0.001	0.006	8.01E-01	0.012	0.009	1.49E-01
7.456147	-0.004	0.008	6.16E-01	-0.006	0.012	6.44E-01
8.494378	0.002	0.008	7.62E-01	0.003	0.011	8.00E-01
4.240187	0.008	0.012	5.10E-01	-0.010	0.018	5.83E-01
7.952721	0.005	0.008	5.41E-01	-0.008	0.012	5.14E-01

6.074754	0.006	0.009	4.91E-01	-0.007	0.014	6.01E-01
5.110202	0.006	0.009	4.97E-01	-0.014	0.013	2.92E-01
6.893968	-0.003	0.012	7.83E-01	-0.006	0.017	7.38E-01
8.942506	0.005	0.009	5.60E-01	-0.016	0.014	2.29E-01
4.048421	0.010	0.011	3.90E-01	-0.013	0.017	4.57E-01
8.30362	-0.001	0.008	8.59E-01	-0.010	0.012	4.14E-01
6.839802	-0.006	0.009	5.41E-01	0.026	0.014	6.50E-02
4.115035	-0.003	0.009	7.71E-01	0.016	0.014	2.37E-01
6.010159	-0.006	0.009	5.38E-01	-0.002	0.014	8.76E-01
2.636413262	-0.004	0.009	6.57E-01	-0.005	0.013	7.00E-01
6.610019	-0.005	0.008	5.44E-01	0.003	0.012	8.17E-01
6.541386	0.004	0.009	6.82E-01	-0.001	0.013	9.68E-01
0.965689014	0.004	0.006	5.52E-01	0.017	0.009	6.41E-02
7.149993	-0.006	0.007	4.06E-01	0.000	0.011	9.90E-01
7.76331	-0.005	0.012	6.50E-01	-0.022	0.018	2.26E-01
8.688499	0.002	0.007	7.62E-01	0.011	0.010	2.63E-01
6.438102	0.007	0.010	4.53E-01	-0.017	0.014	2.33E-01
8.743001	-0.008	0.009	3.69E-01	-0.005	0.014	7.11E-01
2.724895033	-0.007	0.009	4.25E-01	-0.007	0.013	5.80E-01
6.259456	-0.004	0.007	5.37E-01	-0.012	0.011	2.51E-01
1.92082114	-0.004	0.006	4.89E-01	-0.012	0.009	2.07E-01
0.545148275	-0.003	0.010	7.35E-01	-0.011	0.015	4.67E-01
5.456055	0.005	0.008	5.26E-01	-0.006	0.012	6.36E-01
6.578394	0.002	0.009	8.47E-01	-0.007	0.014	6.03E-01
7.167824	0.008	0.009	3.95E-01	-0.002	0.014	9.06E-01
6.927275	-0.010	0.012	4.19E-01	0.021	0.018	2.34E-01
3.553865	-0.014	0.020	4.78E-01	0.041	0.029	1.62E-01
7.581972	-0.003	0.010	7.43E-01	0.014	0.014	3.16E-01
6.561236	0.004	0.008	5.78E-01	0.001	0.012	9.34E-01
6.1629	0.006	0.009	5.02E-01	-0.007	0.014	6.30E-01
8.343319	0.003	0.008	7.18E-01	0.023	0.011	4.65E-02
8.9129	-0.006	0.010	5.37E-01	0.012	0.015	4.32E-01
4.297045	-0.006	0.013	6.43E-01	0.009	0.019	6.56E-01
1.085122584	0.004	0.008	5.93E-01	0.003	0.011	7.63E-01
7.406355	-0.007	0.010	4.57E-01	-0.026	0.014	7.45E-02
8.077874	-0.005	0.009	5.90E-01	0.011	0.013	3.76E-01
7.703761	-0.004	0.010	6.88E-01	0.010	0.015	5.21E-01
7.921433	0.004	0.010	7.12E-01	0.008	0.016	6.05E-01
6.624822	-0.008	0.010	3.98E-01	-0.013	0.015	3.59E-01
0.764165892	0.003	0.005	5.51E-01	-0.001	0.008	9.04E-01
2.723885735	-0.007	0.009	4.36E-01	-0.001	0.013	9.11E-01
2.543221434	0.015	0.032	6.32E-01	0.006	0.048	9.00E-01
1.827965745	-0.009	0.011	3.73E-01	0.007	0.016	6.61E-01

1.713242231	-0.002	0.005	6.18E-01	0.009	0.007	2.03E-01
4.188713	-0.006	0.013	6.16E-01	0.011	0.019	5.75E-01
5.475568	0.005	0.008	5.05E-01	-0.009	0.012	4.61E-01
5.640756	0.005	0.009	5.42E-01	-0.004	0.013	7.76E-01
5.426112	0.003	0.007	7.04E-01	-0.009	0.011	3.80E-01
7.863903	0.004	0.009	6.64E-01	0.014	0.013	2.88E-01
1.751259114	0.005	0.007	4.89E-01	0.005	0.010	6.36E-01
6.44483	-0.004	0.008	6.38E-01	-0.025	0.012	3.39E-02
5.671708	0.005	0.009	5.45E-01	-0.010	0.013	4.57E-01
5.807627	0.005	0.009	5.89E-01	-0.018	0.014	2.12E-01
8.988261	-0.005	0.010	6.30E-01	-0.025	0.015	9.19E-02
5.035851	0.005	0.009	5.50E-01	-0.016	0.013	2.16E-01
6.472418	0.002	0.010	8.16E-01	0.003	0.014	8.49E-01
7.132835	-0.003	0.009	7.13E-01	-0.004	0.014	7.82E-01
3.685747	-0.002	0.004	6.16E-01	0.006	0.007	3.52E-01
6.030345	0.004	0.008	6.11E-01	-0.016	0.013	2.12E-01
0.585183753	0.004	0.009	6.40E-01	-0.014	0.014	2.96E-01
6.115799	0.005	0.010	6.32E-01	0.032	0.015	2.93E-02
0.605369709	-0.008	0.008	3.63E-01	-0.001	0.012	9.15E-01
2.822124052	-0.010	0.016	5.23E-01	-0.071	0.023	2.03E-03
8.048941	-0.005	0.008	5.19E-01	-0.006	0.013	6.48E-01
3.574724	-0.002	0.006	6.76E-01	0.009	0.009	3.03E-01
2.673420847	-0.008	0.010	3.90E-01	0.003	0.015	8.49E-01
2.791845119	-0.008	0.012	5.18E-01	-0.062	0.019	7.75E-04
7.200794	0.008	0.011	4.68E-01	-0.010	0.017	5.41E-01
6.194188	0.007	0.008	4.13E-01	-0.001	0.012	9.18E-01
6.231532	0.003	0.007	6.58E-01	-0.011	0.010	2.94E-01
3.759762	0.004	0.008	6.16E-01	0.021	0.012	9.54E-02
7.950702	-0.005	0.008	5.09E-01	0.003	0.012	8.10E-01
7.069249	-0.004	0.008	6.04E-01	-0.002	0.012	8.46E-01
0.955259604	-0.005	0.006	4.42E-01	0.012	0.009	1.96E-01
6.845185	0.005	0.010	6.08E-01	-0.002	0.016	8.84E-01
7.658343	0.004	0.007	5.73E-01	-0.014	0.010	1.70E-01
6.198562	-0.005	0.009	5.67E-01	0.000	0.013	9.94E-01
6.575702	0.005	0.008	5.52E-01	0.000	0.012	9.75E-01
0.829433815	-0.007	0.008	3.88E-01	0.001	0.012	9.38E-01
2.661982138	-0.015	0.019	4.52E-01	-0.018	0.029	5.45E-01
6.5646	-0.007	0.009	4.62E-01	-0.006	0.013	6.47E-01
2.502849523	-0.010	0.014	4.51E-01	-0.005	0.020	7.87E-01
2.585948373	0.011	0.013	4.03E-01	0.004	0.019	8.34E-01
4.000984	0.005	0.009	5.92E-01	0.011	0.014	4.28E-01
5.629318	0.004	0.006	5.78E-01	-0.011	0.009	2.56E-01
7.885771	-0.003	0.009	7.35E-01	0.000	0.013	9.91E-01

2.996396	-0.003	0.004	5.00E-01	0.007	0.006	2.47E-01
8.853688	0.003	0.009	7.78E-01	0.013	0.014	3.59E-01
2.355828481	-0.001	0.007	8.77E-01	0.024	0.010	1.63E-02
0.853993394	-0.004	0.007	5.31E-01	-0.011	0.010	2.77E-01
7.653632	-0.003	0.010	7.71E-01	-0.004	0.015	7.72E-01
6.742909	-0.006	0.010	5.38E-01	-0.033	0.015	2.45E-02
8.482266	0.009	0.009	3.29E-01	-0.005	0.014	7.10E-01
0.754409347	-0.003	0.005	6.26E-01	-0.006	0.008	4.34E-01
2.06616002	0.004	0.004	3.24E-01	0.012	0.006	3.50E-02
5.629654	-0.004	0.007	5.74E-01	-0.007	0.010	4.57E-01
3.786003	-0.006	0.012	6.46E-01	0.025	0.018	1.75E-01
2.32723171	-0.002	0.005	7.46E-01	-0.011	0.008	1.89E-01
8.59598	0.007	0.008	4.29E-01	0.006	0.013	6.21E-01
4.043374	0.010	0.013	4.37E-01	-0.008	0.020	6.92E-01
6.030682	0.004	0.007	5.41E-01	-0.001	0.011	9.14E-01
7.53319	-0.005	0.010	6.16E-01	0.012	0.015	4.37E-01
8.96471	-0.001	0.009	8.82E-01	-0.012	0.013	3.61E-01
0.675347688	0.005	0.010	6.25E-01	-0.002	0.014	8.99E-01
4.369714	-0.007	0.014	6.12E-01	0.026	0.020	1.93E-01
8.542151	-0.006	0.009	5.21E-01	0.014	0.013	2.62E-01
8.538114	0.003	0.007	7.00E-01	0.002	0.011	8.57E-01
0.556250551	0.006	0.010	5.16E-01	-0.007	0.014	6.27E-01
3.37421	0.007	0.013	5.89E-01	-0.017	0.019	3.76E-01
7.164123	0.008	0.010	4.52E-01	0.014	0.015	3.43E-01
5.799552	-0.005	0.009	5.38E-01	-0.006	0.013	6.15E-01
8.234988	0.006	0.008	4.33E-01	-0.014	0.012	2.28E-01
8.081911	0.004	0.009	6.44E-01	0.009	0.013	4.78E-01
2.787807928	-0.007	0.012	5.62E-01	-0.054	0.018	2.44E-03
7.208869	0.002	0.007	7.38E-01	0.000	0.010	9.92E-01
8.67336	0.003	0.008	6.76E-01	0.021	0.012	9.65E-02
7.746824	-0.005	0.011	6.56E-01	0.003	0.016	8.49E-01
2.099803279	-0.002	0.008	8.54E-01	0.001	0.012	9.53E-01
4.119745	-0.005	0.012	6.61E-01	0.019	0.018	2.76E-01
5.748078	0.002	0.010	8.51E-01	0.007	0.014	6.03E-01
7.120723	-0.009	0.010	3.55E-01	-0.016	0.015	3.06E-01
7.924124	-0.002	0.008	7.83E-01	-0.016	0.012	1.64E-01
1.418190849	0.004	0.005	3.55E-01	0.005	0.007	5.19E-01
7.723947	-0.005	0.010	6.03E-01	0.003	0.015	8.43E-01
1.047442134	-0.009	0.011	4.19E-01	-0.021	0.016	1.84E-01
8.881275	0.006	0.010	5.59E-01	0.010	0.015	5.14E-01
7.429232	0.001	0.010	9.38E-01	0.000	0.014	9.95E-01
5.535453	0.004	0.009	6.31E-01	-0.021	0.014	1.30E-01
8.956972	-0.004	0.009	6.66E-01	-0.012	0.014	3.81E-01

6.108734	0.007	0.009	3.98E-01	0.007	0.013	6.09E-01
8.239698	-0.004	0.008	6.55E-01	-0.025	0.013	5.01E-02
8.632315	0.000	0.008	9.71E-01	0.001	0.013	9.67E-01
6.975048	-0.009	0.010	3.96E-01	0.008	0.015	6.11E-01
8.763187	0.004	0.009	6.49E-01	-0.007	0.014	5.89E-01
5.772301	0.004	0.009	6.61E-01	0.000	0.013	9.96E-01
3.933697	-0.002	0.006	7.35E-01	0.010	0.009	3.10E-01
1.888860044	-0.003	0.007	6.03E-01	-0.003	0.010	7.49E-01
0.667609738	-0.004	0.010	6.60E-01	-0.016	0.014	2.68E-01
2.038572547	0.004	0.004	4.12E-01	0.009	0.007	1.49E-01
6.274932	0.007	0.010	4.56E-01	0.004	0.015	7.74E-01
3.133997	-0.006	0.010	5.65E-01	-0.007	0.015	6.22E-01
7.710826	-0.005	0.009	5.31E-01	-0.003	0.013	8.26E-01
7.560777	-0.006	0.007	4.01E-01	0.006	0.010	5.65E-01
7.90663	0.005	0.007	4.99E-01	-0.002	0.011	8.32E-01
8.69691	-0.005	0.010	6.37E-01	-0.016	0.015	2.91E-01
1.867664791	-0.003	0.009	7.52E-01	-0.007	0.013	6.08E-01
6.540041	0.004	0.008	5.90E-01	0.012	0.011	2.75E-01
8.441221	-0.005	0.009	5.33E-01	-0.027	0.013	4.18E-02
5.564386	0.005	0.010	6.37E-01	-0.020	0.015	1.88E-01
7.073959	0.004	0.008	5.64E-01	0.002	0.011	8.91E-01
7.545974	0.002	0.010	7.98E-01	-0.001	0.014	9.24E-01
5.111884	0.006	0.009	4.99E-01	0.009	0.014	5.01E-01
3.204984	0.004	0.008	6.20E-01	0.017	0.013	1.81E-01
8.674369	0.007	0.010	5.15E-01	-0.018	0.016	2.60E-01
2.521016883	-0.010	0.014	4.63E-01	-0.009	0.021	6.68E-01
6.721378	-0.005	0.011	6.54E-01	-0.007	0.017	6.83E-01
3.17336	-0.004	0.006	5.08E-01	0.006	0.009	4.79E-01
6.187459	-0.004	0.011	7.19E-01	0.004	0.016	8.08E-01
2.825488378	-0.007	0.013	5.94E-01	-0.041	0.020	3.63E-02
0.748689993	-0.003	0.006	5.92E-01	-0.007	0.008	4.05E-01
8.375953	-0.003	0.007	6.70E-01	0.016	0.010	1.33E-01
8.868827	-0.006	0.009	5.18E-01	0.012	0.014	3.79E-01
8.575458	-0.003	0.009	7.73E-01	0.000	0.013	9.87E-01
5.877268	0.007	0.011	5.46E-01	-0.022	0.017	2.07E-01
6.489239	0.002	0.006	7.19E-01	-0.011	0.010	2.52E-01
5.490371	0.004	0.008	6.70E-01	-0.009	0.012	4.54E-01
5.561358	0.005	0.008	4.85E-01	-0.005	0.011	6.42E-01
8.232633	0.008	0.010	4.11E-01	0.015	0.015	3.27E-01
4.13522	-0.007	0.013	5.85E-01	0.009	0.019	6.24E-01
2.266000979	0.004	0.007	5.74E-01	0.019	0.011	9.14E-02
3.665224	-0.002	0.005	6.58E-01	0.015	0.008	5.62E-02
0.829097382	-0.007	0.008	3.93E-01	0.001	0.012	9.24E-01

3.984499	-0.006	0.009	4.72E-01	0.001	0.013	9.45E-01
7.004318	-0.004	0.008	5.90E-01	-0.015	0.012	2.26E-01
2.580565451	0.011	0.012	3.32E-01	0.023	0.017	1.94E-01
3.686756	-0.002	0.005	6.18E-01	0.007	0.007	2.75E-01
8.900115	-0.002	0.008	8.40E-01	-0.010	0.012	4.12E-01
8.713395	0.004	0.007	5.65E-01	0.000	0.011	9.72E-01
1.651338635	0.003	0.005	5.07E-01	0.004	0.007	5.71E-01
6.602281	0.004	0.008	6.54E-01	0.010	0.012	4.19E-01
2.161706875	-0.006	0.010	5.79E-01	-0.013	0.015	3.76E-01
7.864576	-0.005	0.010	6.58E-01	-0.013	0.015	3.93E-01
7.85381	-0.005	0.008	5.17E-01	0.019	0.012	1.13E-01
7.573562	0.004	0.008	6.13E-01	-0.007	0.011	5.31E-01
7.134181	0.006	0.009	5.29E-01	-0.031	0.013	2.07E-02
0.64843308	-0.008	0.010	4.51E-01	-0.019	0.015	2.06E-01
4.187368	-0.006	0.012	6.03E-01	0.015	0.018	3.99E-01
4.218992	-0.006	0.013	6.60E-01	0.015	0.019	4.26E-01
5.010618	0.006	0.009	5.03E-01	-0.013	0.014	3.64E-01
1.822582824	-0.002	0.009	8.44E-01	0.013	0.014	3.43E-01
1.903999511	-0.004	0.007	5.04E-01	-0.002	0.010	8.63E-01
4.285269	0.008	0.010	4.67E-01	0.016	0.016	3.07E-01
5.663634	0.004	0.008	6.30E-01	0.001	0.012	9.53E-01
1.232143626	0.004	0.008	6.38E-01	-0.008	0.012	5.06E-01
7.475996	-0.005	0.009	5.60E-01	-0.012	0.013	3.79E-01
7.934217	-0.003	0.008	7.22E-01	0.013	0.013	2.88E-01
2.501840225	-0.009	0.013	4.60E-01	-0.008	0.019	6.86E-01
4.386872	-0.007	0.013	6.09E-01	0.019	0.020	3.42E-01
5.76019	0.003	0.008	7.26E-01	-0.005	0.012	6.83E-01
8.771262	0.004	0.007	5.58E-01	0.001	0.011	9.20E-01
6.121182	-0.004	0.008	6.44E-01	-0.005	0.012	6.48E-01
8.467463	-0.008	0.010	4.28E-01	-0.015	0.015	2.90E-01
7.582645	0.005	0.009	6.14E-01	0.015	0.014	2.60E-01
5.159994	0.004	0.009	6.28E-01	-0.018	0.013	1.62E-01
2.366257891	0.013	0.012	2.83E-01	0.044	0.018	1.57E-02
6.22043	0.006	0.009	5.07E-01	-0.032	0.013	1.47E-02
5.503492	0.004	0.009	6.05E-01	-0.012	0.013	3.55E-01
5.752115	0.001	0.010	9.40E-01	-0.005	0.016	7.52E-01
7.80099	-0.005	0.009	5.67E-01	-0.021	0.013	1.08E-01
1.186725226	0.004	0.007	5.31E-01	0.006	0.010	5.52E-01
7.49921	0.005	0.009	5.64E-01	0.005	0.013	6.99E-01
7.330994	-0.005	0.006	4.23E-01	-0.004	0.009	7.02E-01
5.489362	0.006	0.009	5.38E-01	-0.009	0.014	5.25E-01
7.078669	0.004	0.009	6.44E-01	0.013	0.013	3.25E-01
5.243429	-0.006	0.005	1.92E-01	-0.002	0.007	7.48E-01

6.564264	-0.006	0.009	5.30E-01	-0.004	0.014	7.49E-01
5.914612	-0.009	0.010	3.96E-01	-0.021	0.015	1.75E-01
1.660422315	0.002	0.004	6.55E-01	0.003	0.006	6.82E-01
2.533801321	-0.005	0.011	6.51E-01	-0.006	0.016	6.97E-01
2.724222168	-0.007	0.009	4.26E-01	-0.003	0.013	8.07E-01
4.191741	-0.007	0.012	5.85E-01	0.009	0.018	6.14E-01
7.334358	-0.004	0.006	4.66E-01	-0.007	0.009	4.47E-01
7.406691	-0.007	0.010	4.71E-01	-0.027	0.014	5.36E-02
6.620784	0.003	0.009	7.30E-01	-0.008	0.014	5.92E-01
8.717769	-0.006	0.009	5.09E-01	-0.011	0.014	4.41E-01
7.345124	0.004	0.010	7.13E-01	-0.015	0.015	3.20E-01
2.426815757	-0.005	0.009	5.53E-01	0.017	0.013	1.94E-01
4.238842	0.008	0.012	5.07E-01	-0.007	0.017	6.93E-01
7.403327	-0.004	0.008	6.49E-01	0.006	0.012	6.51E-01
3.164276	-0.004	0.007	6.06E-01	0.011	0.010	3.00E-01
8.661585	0.006	0.008	4.79E-01	0.006	0.012	6.21E-01
3.979116	-0.006	0.013	6.59E-01	0.000	0.019	9.86E-01
6.050195	0.005	0.009	5.53E-01	-0.005	0.013	6.83E-01
8.21783	-0.003	0.006	6.07E-01	-0.004	0.009	6.41E-01
8.758814	0.003	0.006	6.16E-01	-0.001	0.009	8.93E-01
6.206972	-0.003	0.008	6.87E-01	-0.013	0.011	2.50E-01
5.455046	0.005	0.007	4.92E-01	-0.013	0.011	2.02E-01
4.029244	-0.007	0.014	6.26E-01	-0.015	0.020	4.45E-01
3.298513	0.009	0.011	4.20E-01	0.015	0.017	3.66E-01
2.360538537	0.008	0.008	2.83E-01	0.016	0.012	1.58E-01
7.353871	-0.009	0.010	3.85E-01	-0.028	0.016	7.05E-02
1.0326391	0.003	0.005	5.74E-01	0.011	0.008	1.48E-01
7.139227	0.004	0.010	6.84E-01	-0.012	0.015	4.35E-01
7.864239	0.002	0.008	8.16E-01	0.005	0.012	6.51E-01
8.632988	-0.006	0.009	4.60E-01	-0.001	0.013	9.54E-01
8.1603	-0.003	0.007	7.06E-01	0.000	0.011	9.86E-01
5.787777	0.004	0.007	5.39E-01	0.016	0.011	1.30E-01
8.19327	-0.008	0.010	4.52E-01	-0.005	0.015	7.65E-01
8.957309	-0.006	0.010	5.50E-01	-0.014	0.014	3.33E-01
8.324479	-0.007	0.009	3.93E-01	-0.017	0.013	2.04E-01
1.841423049	-0.006	0.010	5.61E-01	0.007	0.015	6.29E-01
3.946818	-0.004	0.007	5.55E-01	0.006	0.010	5.93E-01
0.902103254	0.005	0.007	4.27E-01	-0.018	0.010	7.66E-02
2.848365794	-0.004	0.009	6.88E-01	-0.008	0.013	5.62E-01
1.001687301	0.005	0.008	5.44E-01	-0.007	0.012	5.75E-01
0.621854906	-0.005	0.009	5.96E-01	0.007	0.014	6.00E-01
8.141796	-0.003	0.010	7.38E-01	0.005	0.015	7.57E-01
8.37158	0.004	0.010	6.52E-01	0.012	0.014	3.98E-01

5.762881	0.004	0.011	7.36E-01	-0.011	0.016	5.14E-01
3.544445	0.005	0.010	5.77E-01	0.008	0.015	5.92E-01
7.535881	0.004	0.009	6.90E-01	-0.017	0.013	2.08E-01
7.506948	-0.001	0.008	8.77E-01	-0.004	0.011	7.37E-01
6.880847	0.007	0.007	3.56E-01	0.005	0.011	6.53E-01
8.97985	0.006	0.009	5.40E-01	-0.003	0.013	8.37E-01
5.941527	-0.003	0.009	6.94E-01	-0.021	0.013	1.10E-01
7.488108	-0.007	0.009	4.52E-01	-0.022	0.013	9.47E-02
3.794414	0.005	0.006	4.24E-01	0.007	0.009	4.44E-01
6.675623	0.004	0.010	7.22E-01	0.011	0.016	4.78E-01
8.998354	-0.004	0.008	5.95E-01	-0.013	0.012	2.82E-01
5.934462	-0.004	0.010	6.91E-01	-0.021	0.015	1.47E-01
7.141246	-0.007	0.011	5.04E-01	-0.020	0.016	2.08E-01
6.992542	0.004	0.007	5.91E-01	-0.003	0.011	7.60E-01
8.639716	0.004	0.007	6.08E-01	-0.012	0.011	2.96E-01
0.658189626	-0.006	0.010	5.74E-01	-0.018	0.015	2.29E-01
5.777012	0.003	0.008	6.71E-01	-0.005	0.012	6.76E-01
8.873874	0.003	0.009	7.12E-01	-0.013	0.013	3.47E-01
5.805945	0.005	0.007	5.02E-01	-0.010	0.011	3.59E-01
2.852066552	-0.005	0.008	4.99E-01	-0.018	0.012	1.18E-01
7.169842	-0.007	0.009	4.64E-01	-0.019	0.014	1.63E-01
3.179079	-0.003	0.005	6.22E-01	0.015	0.008	4.92E-02
3.686083	-0.002	0.004	6.73E-01	0.009	0.007	1.66E-01
5.502483	0.003	0.007	6.78E-01	0.002	0.011	8.30E-01
7.504593	0.005	0.009	5.53E-01	-0.019	0.013	1.56E-01
4.116044	-0.003	0.009	7.29E-01	0.018	0.014	1.87E-01
1.664795938	-0.002	0.004	5.76E-01	0.003	0.006	5.96E-01
2.997405	-0.003	0.004	4.76E-01	0.004	0.006	4.78E-01
7.515695	-0.004	0.007	5.53E-01	-0.001	0.010	9.14E-01
4.219329	-0.005	0.012	6.67E-01	0.009	0.018	6.06E-01
1.055180083	0.004	0.006	4.71E-01	-0.011	0.009	1.99E-01
8.693882	0.006	0.009	5.14E-01	-0.015	0.014	2.98E-01
8.475874	-0.006	0.010	5.52E-01	-0.028	0.015	6.27E-02
7.416448	0.005	0.010	6.22E-01	-0.008	0.015	5.92E-01
3.184126	0.005	0.006	4.24E-01	0.016	0.008	5.34E-02
1.420545877	0.005	0.005	3.00E-01	0.003	0.007	6.73E-01
8.198653	0.004	0.008	5.72E-01	-0.001	0.011	9.31E-01
7.648586	-0.004	0.011	7.20E-01	0.000	0.016	9.82E-01
8.245417	-0.002	0.007	7.86E-01	-0.003	0.011	7.93E-01
1.661768045	0.002	0.004	5.58E-01	0.004	0.006	5.09E-01
7.480706	0.005	0.010	5.84E-01	-0.015	0.014	3.03E-01
5.123996	0.003	0.008	6.82E-01	-0.015	0.012	2.27E-01
4.363658	-0.007	0.012	5.83E-01	0.016	0.018	3.99E-01

8.461071	-0.003	0.009	7.12E-01	0.002	0.013	9.02E-01
6.189814	-0.003	0.008	6.74E-01	-0.016	0.012	1.68E-01
1.712905799	-0.002	0.004	6.32E-01	0.007	0.007	3.21E-01
6.305884	0.005	0.009	5.69E-01	0.006	0.013	6.69E-01
0.577109371	0.006	0.010	5.14E-01	0.025	0.014	7.28E-02
3.943117	-0.005	0.009	5.93E-01	0.003	0.013	8.12E-01
8.254838	0.007	0.009	4.33E-01	0.010	0.014	4.54E-01
5.68853	0.004	0.008	6.15E-01	-0.023	0.012	6.15E-02
7.426877	-0.006	0.009	4.79E-01	0.016	0.014	2.48E-01
8.118246	-0.004	0.007	5.02E-01	-0.002	0.010	8.52E-01
5.022393	0.005	0.009	5.69E-01	-0.010	0.014	4.81E-01
3.313988	-0.008	0.011	4.71E-01	0.004	0.016	7.96E-01
5.14721	0.005	0.008	5.66E-01	-0.014	0.013	2.78E-01
7.344787	0.005	0.012	6.49E-01	0.011	0.018	5.24E-01
7.971561	0.005	0.009	5.58E-01	-0.005	0.014	7.44E-01
7.973916	0.004	0.009	6.71E-01	-0.021	0.013	1.09E-01
2.334633227	0.005	0.007	4.16E-01	0.008	0.010	4.16E-01
0.50040274	0.004	0.009	6.14E-01	-0.001	0.013	9.43E-01
2.285177636	-0.004	0.007	5.40E-01	-0.001	0.011	9.59E-01
8.120264	-0.007	0.009	4.38E-01	-0.001	0.014	9.39E-01
4.301755	-0.008	0.014	5.66E-01	0.012	0.020	5.68E-01
5.838579	-0.004	0.007	5.33E-01	-0.003	0.010	7.49E-01
7.371702	-0.006	0.010	5.34E-01	0.000	0.014	9.73E-01
8.348029	-0.007	0.010	4.93E-01	-0.005	0.015	7.30E-01
8.939478	-0.002	0.010	8.13E-01	0.006	0.015	6.97E-01
4.096531	-0.002	0.009	7.92E-01	0.011	0.014	4.37E-01
1.922166871	0.002	0.005	7.06E-01	-0.002	0.007	7.58E-01
7.561114	-0.007	0.010	4.93E-01	0.009	0.014	5.50E-01
3.183789	0.004	0.005	4.22E-01	0.016	0.008	4.37E-02
1.026919746	0.003	0.008	6.80E-01	0.013	0.011	2.65E-01
3.374546	0.007	0.013	5.80E-01	-0.017	0.020	3.98E-01
5.052336	0.004	0.007	6.01E-01	-0.001	0.011	9.51E-01
5.483979	-0.003	0.007	6.57E-01	-0.002	0.010	8.52E-01
4.220674	-0.008	0.013	5.55E-01	0.006	0.019	7.75E-01
7.54732	0.007	0.009	4.52E-01	-0.012	0.014	4.09E-01
4.359621	-0.006	0.013	6.36E-01	0.010	0.020	6.34E-01
8.303284	0.006	0.007	4.50E-01	0.002	0.011	8.61E-01
6.227158	0.002	0.009	8.71E-01	-0.012	0.014	3.97E-01
5.241411	-0.001	0.004	8.07E-01	0.002	0.006	7.78E-01
8.44795	0.007	0.010	5.10E-01	0.004	0.015	8.05E-01
1.026583313	0.003	0.007	6.61E-01	0.014	0.011	1.80E-01
2.99606	-0.003	0.004	5.23E-01	0.005	0.006	4.01E-01
2.27912185	-0.004	0.006	4.71E-01	-0.006	0.009	4.75E-01

7.3172	-0.006	0.006	3.29E-01	-0.012	0.009	1.76E-01
7.637484	-0.002	0.009	8.50E-01	0.000	0.013	9.71E-01
7.256306	-0.010	0.011	3.83E-01	0.002	0.017	8.84E-01
6.367114	-0.003	0.008	6.87E-01	0.009	0.012	4.31E-01
8.589924	0.007	0.007	3.09E-01	0.003	0.010	7.81E-01
6.563927	0.005	0.009	6.08E-01	0.010	0.013	4.38E-01
4.42388	-0.007	0.014	6.01E-01	0.012	0.021	5.73E-01
5.025758	0.005	0.010	6.50E-01	-0.006	0.015	7.00E-01
0.608734035	0.004	0.008	6.23E-01	0.015	0.012	2.16E-01
0.611089063	0.005	0.009	6.14E-01	0.024	0.014	7.98E-02
0.828424517	-0.007	0.008	3.99E-01	0.002	0.012	8.88E-01
4.098886	-0.004	0.010	7.25E-01	0.004	0.015	7.87E-01
3.579434	-0.001	0.006	8.58E-01	0.010	0.009	2.72E-01
1.7727908	-0.006	0.009	5.35E-01	0.040	0.014	4.61E-03
4.210581	-0.004	0.012	7.51E-01	0.023	0.018	1.83E-01
8.332553	-0.001	0.007	8.72E-01	-0.006	0.010	5.27E-01
7.565824	-0.006	0.006	3.40E-01	0.000	0.009	9.73E-01
1.087141179	0.004	0.007	6.23E-01	0.001	0.011	9.42E-01
8.371916	0.005	0.008	5.57E-01	0.004	0.012	7.34E-01
7.633783	-0.006	0.012	6.11E-01	-0.003	0.018	8.50E-01
2.032853193	0.004	0.004	4.16E-01	0.008	0.007	2.42E-01
6.440457	0.002	0.010	7.96E-01	0.022	0.014	1.24E-01
3.994928	-0.006	0.009	5.08E-01	0.013	0.013	3.30E-01
6.939386	-0.004	0.010	6.51E-01	0.028	0.014	5.56E-02
5.944218	0.007	0.009	4.29E-01	0.005	0.013	7.09E-01
0.732541228	-0.005	0.007	4.57E-01	-0.001	0.010	9.58E-01
0.552549792	0.001	0.009	9.03E-01	0.010	0.014	4.73E-01
1.924185466	0.002	0.004	6.67E-01	0.009	0.006	1.11E-01
6.10537	0.002	0.008	8.46E-01	-0.005	0.012	6.70E-01
0.555914118	0.007	0.008	4.27E-01	-0.008	0.012	4.94E-01
8.327843	0.004	0.006	5.16E-01	-0.006	0.010	5.53E-01
6.743582	-0.008	0.010	4.57E-01	-0.018	0.015	2.31E-01
6.009486	0.004	0.009	6.43E-01	-0.008	0.013	5.31E-01
5.683483	0.004	0.009	6.13E-01	-0.002	0.013	8.55E-01
3.435104	0.007	0.015	6.18E-01	0.029	0.022	1.87E-01
7.029214	-0.006	0.010	5.17E-01	-0.014	0.014	3.36E-01
7.413083	-0.007	0.008	4.26E-01	0.000	0.012	9.97E-01
6.400085	-0.006	0.008	4.52E-01	-0.020	0.012	9.97E-02
4.006703	0.004	0.011	7.30E-01	0.024	0.017	1.60E-01
6.834756	0.003	0.009	7.66E-01	-0.006	0.013	6.29E-01
3.760098	0.004	0.008	6.19E-01	0.023	0.012	5.23E-02
6.691435	0.000	0.008	9.72E-01	0.006	0.013	6.40E-01
6.576039	0.004	0.008	6.42E-01	-0.002	0.012	8.46E-01

1.684981894	-0.003	0.004	4.73E-01	0.009	0.006	1.30E-01
8.347693	-0.005	0.010	5.73E-01	0.000	0.014	9.74E-01
8.122956	0.004	0.008	6.69E-01	0.008	0.012	5.31E-01
8.307994	0.002	0.009	7.81E-01	0.007	0.013	6.15E-01
1.746885491	-0.003	0.007	6.98E-01	0.003	0.011	8.03E-01
7.371029	0.006	0.010	5.39E-01	0.014	0.015	3.51E-01
2.536492782	0.003	0.011	8.00E-01	0.002	0.016	8.82E-01
4.087783	0.009	0.010	3.63E-01	0.008	0.015	5.92E-01
6.037074	-0.008	0.010	4.06E-01	-0.007	0.015	6.29E-01
5.978198	0.005	0.007	5.11E-01	0.001	0.011	9.52E-01
8.038848	0.006	0.008	4.89E-01	-0.010	0.012	3.95E-01
5.830168	0.006	0.008	4.26E-01	0.008	0.011	4.96E-01
0.763156594	-0.001	0.005	8.08E-01	-0.014	0.008	7.31E-02
6.400421	0.003	0.007	7.03E-01	-0.013	0.010	2.00E-01
7.84338	0.005	0.009	5.93E-01	-0.002	0.013	8.91E-01
5.122314	0.004	0.009	6.31E-01	0.000	0.013	9.76E-01
7.292304	-0.008	0.011	4.66E-01	-0.003	0.017	8.63E-01
1.226424272	0.004	0.008	6.45E-01	-0.005	0.012	6.47E-01
1.161492782	0.005	0.005	3.32E-01	-0.011	0.007	1.39E-01
7.384486	0.003	0.008	7.40E-01	0.003	0.012	7.85E-01
8.618858	0.002	0.007	7.56E-01	-0.001	0.010	9.60E-01
5.91293	0.004	0.007	5.98E-01	0.008	0.011	4.55E-01
8.442567	0.005	0.010	6.22E-01	0.008	0.014	5.58E-01
8.199999	0.003	0.008	6.86E-01	-0.005	0.012	6.77E-01
0.828760949	-0.007	0.008	4.02E-01	0.001	0.012	9.06E-01
5.287502	-0.006	0.009	4.85E-01	-0.027	0.013	4.26E-02
4.098549	-0.003	0.010	7.45E-01	0.004	0.014	7.90E-01
8.823745	-0.002	0.008	7.95E-01	0.005	0.012	6.59E-01
2.332278199	0.004	0.006	5.40E-01	0.005	0.009	5.66E-01
4.185685	-0.006	0.014	6.62E-01	0.015	0.020	4.66E-01
5.95633	0.005	0.006	4.36E-01	0.000	0.010	9.63E-01
7.380113	0.006	0.008	4.61E-01	-0.006	0.012	5.98E-01
7.26438	-0.006	0.011	5.83E-01	-0.005	0.016	7.35E-01
5.111211	0.006	0.009	5.22E-01	-0.006	0.014	6.80E-01
6.281997	-0.006	0.010	5.65E-01	0.009	0.015	5.67E-01
3.328119	0.007	0.010	4.58E-01	0.023	0.014	1.13E-01
3.278663	-0.007	0.011	5.13E-01	0.008	0.016	6.37E-01
7.764319	-0.005	0.012	6.67E-01	-0.026	0.018	1.46E-01
3.691466	-0.002	0.005	6.15E-01	0.011	0.007	1.11E-01
8.066435	0.007	0.009	4.36E-01	0.019	0.013	1.44E-01
3.185808	0.004	0.006	4.98E-01	0.016	0.009	8.64E-02
7.385832	-0.006	0.009	5.31E-01	-0.002	0.013	8.72E-01
6.972693	-0.008	0.010	4.08E-01	0.001	0.014	9.62E-01

0.516887937	-0.004	0.008	6.49E-01	0.000	0.012	9.99E-01
8.287808	0.006	0.009	4.58E-01	0.001	0.013	9.22E-01
5.010282	0.006	0.010	5.66E-01	-0.009	0.015	5.28E-01
3.685074	-0.002	0.004	6.03E-01	0.008	0.007	2.38E-01
8.209756	0.005	0.010	6.08E-01	-0.004	0.015	7.65E-01
8.108826	0.004	0.007	4.98E-01	0.009	0.010	3.83E-01
3.942781	-0.005	0.009	5.84E-01	0.006	0.013	6.37E-01
4.296372	-0.006	0.012	6.27E-01	0.008	0.018	6.66E-01
8.745356	0.003	0.006	6.11E-01	-0.006	0.010	5.03E-01
6.924247	0.003	0.011	8.21E-01	0.000	0.017	9.93E-01
6.677978	-0.005	0.010	5.73E-01	0.004	0.014	7.87E-01
1.190089552	0.004	0.008	5.77E-01	0.000	0.011	9.98E-01
3.928987	0.006	0.008	4.72E-01	0.022	0.012	6.75E-02
4.10225	-0.001	0.011	9.05E-01	0.011	0.016	5.00E-01
2.292242721	-0.001	0.004	7.47E-01	-0.009	0.006	1.44E-01
8.292182	0.007	0.009	4.28E-01	-0.025	0.014	7.04E-02
1.638554196	0.004	0.006	5.34E-01	-0.008	0.009	3.74E-01
5.296922	0.007	0.012	5.48E-01	-0.024	0.019	2.02E-01
6.358703	0.007	0.009	4.73E-01	0.014	0.014	3.29E-01
8.67706	0.004	0.009	6.15E-01	-0.010	0.013	4.46E-01
3.164949	-0.004	0.007	5.76E-01	0.007	0.010	5.15E-01
6.75233	-0.005	0.011	6.78E-01	-0.023	0.017	1.70E-01
3.925959	-0.002	0.009	8.06E-01	0.017	0.013	2.08E-01
5.633355	-0.004	0.010	6.48E-01	-0.005	0.014	7.52E-01
7.117359	-0.004	0.008	6.37E-01	-0.013	0.011	2.56E-01
8.801204	0.007	0.008	3.91E-01	-0.020	0.012	9.57E-02
6.925256	-0.007	0.011	5.58E-01	0.006	0.017	7.44E-01
7.343442	0.006	0.011	5.71E-01	0.013	0.016	4.26E-01
5.08396	0.004	0.007	5.41E-01	-0.003	0.011	7.65E-01
8.372253	0.005	0.008	5.34E-01	-0.001	0.013	9.54E-01
7.023158	0.004	0.009	6.54E-01	-0.029	0.014	3.35E-02
7.442353	0.007	0.011	5.36E-01	0.027	0.016	9.78E-02
8.366197	0.004	0.009	6.40E-01	-0.006	0.013	6.40E-01
3.998965	0.003	0.010	7.57E-01	0.017	0.014	2.22E-01
8.049614	0.006	0.009	5.15E-01	0.006	0.014	6.58E-01
1.702139956	-0.002	0.004	6.23E-01	0.005	0.006	4.47E-01
4.358275	-0.008	0.015	5.77E-01	0.010	0.022	6.43E-01
6.415561	0.004	0.008	6.50E-01	0.007	0.012	5.84E-01
7.141582	-0.008	0.011	4.52E-01	-0.026	0.016	1.04E-01
5.506183	0.004	0.011	7.05E-01	-0.013	0.016	4.13E-01
8.986242	0.004	0.009	6.39E-01	-0.010	0.013	4.53E-01
8.614148	0.002	0.008	7.74E-01	-0.009	0.011	4.33E-01
8.981868	0.003	0.010	7.62E-01	-0.002	0.015	8.79E-01

8.636689	0.002	0.006	7.87E-01	-0.002	0.009	8.58E-01
6.429018	0.004	0.007	6.18E-01	0.000	0.011	9.71E-01
3.573378	-0.002	0.006	7.30E-01	0.012	0.009	1.74E-01
1.768417176	-0.005	0.010	6.04E-01	-0.008	0.016	6.07E-01
8.852678	-0.008	0.010	4.37E-01	-0.006	0.015	6.91E-01
7.27851	-0.007	0.009	4.09E-01	-0.004	0.013	7.32E-01
2.323867384	-0.003	0.007	6.46E-01	-0.007	0.010	4.85E-01
7.203149	-0.002	0.008	7.99E-01	-0.004	0.011	7.00E-01
5.13207	0.005	0.009	5.75E-01	-0.009	0.014	5.24E-01
2.083990947	-0.003	0.007	6.81E-01	0.010	0.010	3.14E-01
6.132957	0.004	0.008	5.79E-01	0.000	0.012	9.92E-01
0.828088084	-0.006	0.008	4.02E-01	0.002	0.011	8.72E-01
7.159749	0.003	0.008	7.48E-01	0.008	0.012	4.77E-01
2.504531686	-0.010	0.013	4.79E-01	-0.010	0.020	6.14E-01
8.094696	0.006	0.008	4.56E-01	-0.010	0.012	3.82E-01
5.958685	0.003	0.008	6.83E-01	-0.015	0.012	2.28E-01
6.830718	0.004	0.008	6.50E-01	0.009	0.012	4.74E-01
8.978168	-0.006	0.010	5.38E-01	-0.001	0.015	9.49E-01
1.682963298	0.000	0.004	9.53E-01	0.007	0.006	2.28E-01
1.037349156	0.003	0.006	6.60E-01	0.004	0.009	6.24E-01
2.634394666	-0.003	0.009	6.88E-01	-0.015	0.013	2.45E-01
7.564141	0.005	0.010	5.91E-01	0.015	0.015	3.16E-01
6.335826	0.005	0.008	5.23E-01	-0.008	0.012	4.75E-01
8.529703	0.006	0.009	4.60E-01	0.022	0.013	8.74E-02
0.68207634	0.008	0.012	4.76E-01	-0.008	0.018	6.51E-01
6.353321	0.003	0.007	6.81E-01	-0.002	0.011	8.24E-01
7.471622	0.006	0.007	4.32E-01	0.000	0.011	9.87E-01
7.425195	-0.007	0.010	5.07E-01	0.001	0.015	9.71E-01
6.006459	-0.003	0.008	6.69E-01	0.011	0.012	3.50E-01
3.60433	-0.005	0.008	5.46E-01	-0.004	0.012	7.63E-01
4.371733	-0.006	0.011	6.01E-01	0.010	0.016	5.19E-01
6.77588	0.006	0.011	5.42E-01	0.001	0.016	9.34E-01
6.854942	-0.005	0.009	5.70E-01	0.005	0.014	7.41E-01
8.785055	0.002	0.008	7.84E-01	0.002	0.012	8.63E-01
8.830474	0.004	0.009	6.44E-01	-0.008	0.013	5.41E-01
8.136077	-0.004	0.006	5.10E-01	-0.006	0.009	4.87E-01
6.921555	-0.005	0.010	5.89E-01	0.016	0.015	2.57E-01
6.341209	0.004	0.008	6.50E-01	0.004	0.012	7.35E-01
6.950825	-0.004	0.008	5.86E-01	0.005	0.012	6.58E-01
2.184920724	0.008	0.011	4.82E-01	-0.032	0.016	5.19E-02
5.752452	0.003	0.012	7.75E-01	0.009	0.017	6.05E-01
6.940059	-0.003	0.009	7.44E-01	0.027	0.014	5.60E-02
3.535361	0.006	0.009	4.87E-01	0.017	0.013	1.80E-01

7.904275	0.004	0.008	5.88E-01	-0.002	0.011	8.70E-01
5.122987	0.004	0.009	6.52E-01	-0.014	0.013	2.81E-01
3.308269	-0.007	0.011	5.08E-01	0.011	0.017	5.00E-01
6.207982	-0.007	0.009	4.60E-01	0.013	0.013	3.20E-01
5.426449	0.004	0.006	4.85E-01	-0.011	0.009	2.13E-01
1.85588965	-0.005	0.008	5.64E-01	-0.021	0.012	7.30E-02
5.886689	-0.005	0.009	5.86E-01	-0.010	0.013	4.46E-01
7.493491	-0.010	0.010	3.25E-01	-0.001	0.015	9.60E-01
2.344726205	0.006	0.007	3.56E-01	0.005	0.010	6.24E-01
8.89305	0.002	0.008	8.19E-01	-0.013	0.012	2.63E-01
6.973029	-0.008	0.010	4.54E-01	0.000	0.015	1.00E+00
6.733489	-0.007	0.009	4.43E-01	-0.001	0.014	9.45E-01
8.926021	0.003	0.006	6.52E-01	0.006	0.010	5.38E-01
6.103688	0.005	0.009	5.45E-01	-0.007	0.013	5.77E-01
8.603382	0.002	0.007	8.25E-01	-0.015	0.010	1.58E-01
6.34861	0.004	0.009	6.78E-01	-0.018	0.014	2.03E-01
6.022271	-0.002	0.008	8.08E-01	-0.005	0.012	6.80E-01
8.320442	-0.004	0.009	6.12E-01	0.002	0.013	8.81E-01
7.19272	0.006	0.009	4.77E-01	0.003	0.013	8.25E-01
8.594634	-0.003	0.007	7.03E-01	0.002	0.010	8.80E-01
6.552825	0.006	0.009	4.93E-01	-0.012	0.014	3.59E-01
0.711682408	0.002	0.008	8.18E-01	0.012	0.012	3.57E-01
1.82729288	-0.009	0.011	4.38E-01	-0.002	0.017	9.14E-01
7.127115	-0.003	0.008	7.33E-01	0.000	0.011	9.73E-01
3.125923	-0.007	0.009	4.32E-01	0.007	0.014	6.11E-01
5.551265	0.002	0.009	8.16E-01	-0.010	0.013	4.47E-01
7.409046	0.002	0.008	7.62E-01	-0.006	0.011	5.67E-01
4.349864	-0.009	0.015	5.47E-01	0.018	0.022	4.08E-01
6.552152	0.002	0.006	6.85E-01	-0.012	0.009	1.83E-01
6.012851	0.005	0.007	4.52E-01	0.012	0.011	2.39E-01
7.729666	-0.001	0.011	9.16E-01	0.007	0.016	6.56E-01
7.30677	-0.009	0.011	3.94E-01	0.001	0.017	9.37E-01
6.75704	-0.006	0.010	5.45E-01	-0.015	0.015	3.05E-01
7.423513	0.002	0.007	7.86E-01	0.004	0.011	6.90E-01
8.206391	0.003	0.007	6.56E-01	-0.006	0.010	5.89E-01
7.742787	-0.005	0.010	6.01E-01	-0.022	0.015	1.33E-01
7.150666	-0.005	0.007	5.15E-01	-0.005	0.010	6.19E-01
8.990952	-0.008	0.008	3.06E-01	-0.004	0.012	7.48E-01
7.330657	-0.004	0.006	4.84E-01	-0.003	0.009	7.10E-01
4.024871	-0.004	0.012	7.37E-01	0.017	0.018	3.55E-01
2.365921458	0.014	0.013	2.91E-01	0.043	0.019	2.62E-02
6.434064	-0.005	0.008	5.52E-01	0.015	0.012	2.15E-01
4.137239	-0.007	0.012	5.83E-01	0.013	0.018	4.67E-01

8.451314	0.002	0.008	7.83E-01	-0.003	0.012	8.15E-01
7.808391	0.005	0.009	6.14E-01	-0.018	0.014	1.79E-01
2.72321287	-0.006	0.009	4.96E-01	0.003	0.014	8.31E-01
8.254165	0.008	0.010	4.59E-01	0.000	0.015	9.95E-01
8.434156	0.002	0.009	8.65E-01	-0.003	0.014	8.22E-01
1.499271103	-0.003	0.005	5.11E-01	-0.011	0.007	1.49E-01
2.505204551	-0.009	0.012	4.68E-01	-0.019	0.018	2.78E-01
3.695167	0.003	0.005	5.53E-01	0.018	0.007	1.68E-02
6.572338	0.005	0.007	4.61E-01	0.000	0.011	9.64E-01
5.643448	0.003	0.007	7.27E-01	-0.008	0.011	4.33E-01
3.573714	-0.002	0.006	7.18E-01	0.011	0.009	1.97E-01
5.984254	-0.006	0.009	4.69E-01	0.010	0.013	4.23E-01
8.888004	-0.006	0.009	5.38E-01	-0.032	0.014	2.30E-02
3.574387	-0.002	0.006	7.06E-01	0.010	0.009	2.62E-01
5.619561	0.004	0.009	6.13E-01	-0.010	0.013	4.57E-01
7.396598	0.003	0.010	7.43E-01	-0.007	0.014	6.24E-01
4.031599	0.008	0.013	5.64E-01	0.005	0.019	7.86E-01
3.806862	0.003	0.006	6.60E-01	0.016	0.009	7.19E-02
4.372742	-0.006	0.014	6.47E-01	0.016	0.020	4.29E-01
1.794658919	-0.005	0.010	6.03E-01	0.018	0.015	2.27E-01
6.787655	-0.001	0.008	9.18E-01	0.008	0.012	5.15E-01
6.600598	-0.004	0.008	5.73E-01	-0.014	0.011	2.17E-01
2.830871299	0.008	0.012	5.00E-01	-0.033	0.018	6.85E-02
1.086468314	0.005	0.008	5.21E-01	-0.014	0.011	2.12E-01
6.185104	0.004	0.009	6.53E-01	0.002	0.014	8.98E-01
1.46899217	0.007	0.007	3.00E-01	-0.003	0.011	7.70E-01
8.592279	0.005	0.007	4.47E-01	0.011	0.010	2.60E-01
3.796433	0.004	0.005	4.72E-01	0.015	0.008	6.33E-02
1.837385858	-0.004	0.010	7.14E-01	-0.007	0.015	6.13E-01
8.60069	0.006	0.009	4.95E-01	-0.007	0.013	5.94E-01
1.738811108	-0.001	0.006	8.72E-01	0.010	0.009	2.47E-01
8.571084	-0.005	0.006	3.91E-01	0.010	0.009	2.59E-01
2.539857108	-0.009	0.017	6.10E-01	-0.001	0.025	9.72E-01
2.423787864	0.001	0.010	9.47E-01	0.013	0.015	3.61E-01
7.915713	0.008	0.008	3.56E-01	-0.016	0.012	2.08E-01
6.539031	0.006	0.011	5.98E-01	0.006	0.016	7.20E-01
3.942108	-0.004	0.008	6.02E-01	0.009	0.013	4.80E-01
8.707003	0.004	0.007	5.52E-01	-0.007	0.010	4.89E-01
6.067353	-0.006	0.010	5.68E-01	-0.002	0.015	9.09E-01
4.115707	-0.003	0.009	7.66E-01	0.018	0.014	2.03E-01
0.736241987	-0.003	0.007	6.16E-01	-0.003	0.010	7.37E-01
8.45737	0.003	0.007	6.82E-01	0.002	0.010	8.52E-01
2.556005872	0.001	0.010	9.39E-01	0.000	0.015	9.86E-01

7.575244	0.006	0.008	4.65E-01	0.034	0.012	5.86E-03
8.06879	0.003	0.008	7.48E-01	-0.002	0.012	8.81E-01
3.681709	-0.001	0.005	7.43E-01	0.014	0.007	3.98E-02
6.200917	0.003	0.008	6.86E-01	-0.014	0.012	2.48E-01
2.264991681	0.004	0.007	5.48E-01	0.018	0.011	9.20E-02
6.122528	0.004	0.010	7.11E-01	0.010	0.016	5.30E-01
6.098305	-0.003	0.010	7.63E-01	-0.002	0.014	9.15E-01
0.907149743	0.003	0.006	5.76E-01	-0.014	0.009	1.05E-01
8.831819	0.004	0.009	6.33E-01	0.007	0.014	6.11E-01
6.45425	-0.007	0.009	4.16E-01	-0.020	0.013	1.24E-01
2.154305358	-0.004	0.009	6.33E-01	-0.010	0.013	4.25E-01
6.812215	0.003	0.009	7.54E-01	0.012	0.013	3.46E-01
1.231807194	0.003	0.008	6.63E-01	-0.008	0.012	5.05E-01
5.402226	0.005	0.008	5.76E-01	-0.011	0.012	3.76E-01
7.560441	0.001	0.008	8.54E-01	0.020	0.012	8.38E-02
6.483184	-0.005	0.008	5.39E-01	-0.006	0.012	6.41E-01
7.599803	0.006	0.010	5.31E-01	0.005	0.015	7.52E-01
8.028082	-0.003	0.008	7.01E-01	0.004	0.012	7.58E-01
3.114148	-0.008	0.011	4.87E-01	0.022	0.017	2.07E-01
6.652409	-0.001	0.009	9.04E-01	0.008	0.013	5.52E-01
1.226760705	0.003	0.008	6.58E-01	-0.005	0.012	6.38E-01
8.517255	-0.004	0.009	6.32E-01	-0.001	0.014	9.16E-01
5.631336	0.006	0.009	4.51E-01	-0.017	0.013	1.72E-01
6.682688	0.002	0.007	7.67E-01	0.006	0.011	5.93E-01
6.989515	-0.002	0.007	7.72E-01	-0.014	0.010	1.71E-01
6.409168	0.006	0.007	4.13E-01	0.005	0.011	6.63E-01
1.863964032	-0.002	0.008	7.71E-01	0.001	0.013	9.67E-01
5.443607	0.001	0.009	8.87E-01	-0.008	0.014	5.44E-01
7.550011	0.006	0.008	4.79E-01	0.005	0.012	6.52E-01
2.840291412	-0.001	0.010	9.23E-01	-0.005	0.015	7.59E-01
3.742604	-0.001	0.009	9.13E-01	0.009	0.013	4.70E-01
6.791356	-0.005	0.009	5.94E-01	0.010	0.014	4.75E-01
6.253736	0.006	0.010	5.41E-01	-0.011	0.014	4.25E-01
8.635006	0.006	0.008	4.80E-01	-0.008	0.012	5.19E-01
4.324968	-0.007	0.014	6.19E-01	0.017	0.021	4.14E-01
3.92024	0.010	0.011	3.80E-01	0.032	0.016	4.57E-02
7.193729	-0.005	0.008	5.72E-01	-0.004	0.013	7.64E-01
8.821726	0.004	0.008	6.22E-01	0.000	0.012	9.88E-01
4.357602	-0.006	0.012	6.12E-01	0.011	0.018	5.64E-01
7.866594	-0.004	0.008	6.02E-01	-0.013	0.012	2.86E-01
6.301173	-0.002	0.008	8.12E-01	-0.002	0.012	8.91E-01
5.678773	0.004	0.008	6.77E-01	-0.021	0.013	1.01E-01
4.237832	0.007	0.012	5.66E-01	0.001	0.018	9.69E-01

8.058361	0.005	0.008	5.02E-01	-0.011	0.012	3.41E-01
1.051142892	0.005	0.006	4.34E-01	-0.008	0.009	3.98E-01
8.675042	0.004	0.008	6.23E-01	0.008	0.012	5.12E-01
7.842708	0.004	0.008	6.19E-01	0.011	0.012	3.38E-01
2.752482506	-0.006	0.007	4.43E-01	-0.015	0.011	1.67E-01
1.481776609	-0.001	0.006	8.43E-01	-0.001	0.010	8.93E-01
0.678375581	0.004	0.011	7.28E-01	-0.012	0.017	4.71E-01
5.121304	0.003	0.008	6.51E-01	-0.004	0.011	7.15E-01
4.210245	-0.004	0.015	7.67E-01	0.025	0.022	2.46E-01
8.513218	0.006	0.008	4.83E-01	0.006	0.012	5.92E-01
2.284504771	-0.003	0.006	6.48E-01	-0.005	0.008	5.49E-01
6.173329	0.007	0.010	4.69E-01	0.014	0.015	3.24E-01
3.130969	-0.008	0.009	3.81E-01	-0.008	0.013	5.44E-01
5.60644	-0.006	0.008	4.85E-01	0.003	0.012	8.08E-01
5.938836	0.005	0.010	6.36E-01	-0.018	0.015	2.16E-01
3.130633	-0.008	0.009	3.56E-01	-0.013	0.013	3.37E-01
3.330137	-0.005	0.010	5.98E-01	0.009	0.014	5.27E-01
7.902593	-0.009	0.010	3.96E-01	-0.041	0.015	8.44E-03
3.168313	-0.004	0.006	5.62E-01	0.005	0.009	5.76E-01
7.291295	-0.004	0.010	6.58E-01	0.000	0.015	9.83E-01
3.294475	-0.002	0.011	8.90E-01	0.015	0.017	3.59E-01
5.943882	0.005	0.008	5.13E-01	0.004	0.012	7.49E-01
5.477586	0.006	0.008	4.33E-01	-0.007	0.012	5.46E-01
0.745662099	-0.004	0.006	4.69E-01	0.000	0.009	9.64E-01
3.608031	-0.004	0.007	5.83E-01	-0.010	0.011	3.47E-01
6.256428	0.005	0.008	5.11E-01	0.000	0.012	9.77E-01
1.771781502	-0.003	0.009	7.53E-01	0.027	0.014	5.36E-02
5.56775	-0.002	0.008	8.18E-01	-0.001	0.012	9.60E-01
8.90718	-0.008	0.009	3.86E-01	0.000	0.013	9.82E-01
8.25551	0.005	0.010	5.95E-01	-0.003	0.014	8.14E-01
7.592065	0.006	0.009	5.44E-01	-0.001	0.014	9.15E-01
5.467494	0.003	0.007	6.64E-01	-0.016	0.010	1.10E-01
5.898127	-0.005	0.010	6.05E-01	0.002	0.015	9.12E-01
6.434401	0.004	0.009	6.40E-01	0.020	0.014	1.42E-01
1.04306851	0.002	0.006	8.10E-01	0.017	0.009	6.93E-02
7.06723	0.003	0.007	6.62E-01	0.003	0.011	7.62E-01
5.740004	-0.005	0.011	6.48E-01	0.008	0.017	6.28E-01
8.191588	0.003	0.009	7.11E-01	0.018	0.013	1.59E-01
4.239178	0.007	0.012	5.22E-01	-0.009	0.017	6.19E-01
6.677305	0.003	0.009	7.75E-01	0.019	0.014	1.80E-01
0.90546758	0.004	0.006	5.42E-01	-0.011	0.009	2.36E-01
3.683728	-0.002	0.004	6.26E-01	0.010	0.007	1.28E-01
6.175011	0.007	0.009	4.44E-01	0.002	0.013	8.72E-01

8.496396	-0.010	0.009	2.52E-01	0.002	0.013	8.62E-01
6.126901	0.004	0.008	6.23E-01	0.002	0.011	8.29E-01
2.723549303	-0.007	0.009	4.76E-01	0.001	0.014	9.20E-01
2.847692929	-0.002	0.009	8.04E-01	0.000	0.013	9.90E-01
5.982908	0.005	0.008	5.70E-01	0.004	0.012	7.17E-01
0.750035723	-0.004	0.005	5.00E-01	-0.011	0.008	1.88E-01
6.717341	-0.005	0.009	5.60E-01	-0.013	0.013	3.44E-01
5.470521	0.004	0.008	5.98E-01	-0.012	0.012	3.32E-01
5.715108	0.005	0.009	5.97E-01	-0.010	0.013	4.56E-01
8.718105	-0.003	0.008	7.24E-01	0.004	0.012	7.24E-01
8.748384	0.008	0.009	3.65E-01	-0.010	0.013	4.49E-01
6.457951	-0.003	0.009	7.54E-01	0.028	0.014	4.72E-02
0.967707609	-0.004	0.006	4.69E-01	0.014	0.008	1.09E-01
8.668986	0.004	0.006	5.37E-01	0.006	0.009	5.33E-01
5.040561	0.005	0.009	6.15E-01	-0.005	0.014	7.02E-01
3.57977	-0.001	0.006	8.15E-01	0.009	0.009	3.49E-01
7.058483	-0.004	0.008	6.33E-01	-0.012	0.012	3.35E-01
8.651828	0.003	0.007	6.61E-01	0.005	0.011	6.62E-01
8.482602	0.008	0.010	3.98E-01	-0.024	0.015	1.02E-01
8.120937	0.002	0.008	7.96E-01	0.003	0.012	8.12E-01
3.554201	-0.013	0.020	5.30E-01	0.030	0.030	3.29E-01
0.564997798	-0.006	0.007	4.01E-01	0.004	0.010	7.08E-01
8.49505	-0.004	0.007	5.79E-01	-0.001	0.011	8.97E-01
8.396139	0.004	0.009	6.90E-01	-0.021	0.013	1.09E-01
8.85537	0.004	0.008	5.70E-01	-0.007	0.011	5.09E-01
0.996977245	0.004	0.006	4.87E-01	0.014	0.009	1.07E-01
6.295454	0.001	0.008	9.34E-01	-0.001	0.012	9.41E-01
3.210031	-0.009	0.013	4.92E-01	0.003	0.020	8.62E-01
8.784382	0.006	0.008	4.30E-01	-0.003	0.012	7.83E-01
8.162655	0.006	0.008	4.83E-01	0.000	0.012	9.79E-01
8.30463	-0.002	0.008	8.29E-01	-0.009	0.011	4.32E-01
5.15192	0.004	0.009	6.73E-01	-0.008	0.013	5.37E-01
8.224222	0.000	0.009	9.81E-01	0.003	0.013	8.41E-01
2.780069978	-0.008	0.012	5.23E-01	-0.045	0.018	1.11E-02
4.238505	0.007	0.012	5.32E-01	-0.002	0.018	9.26E-01
7.446054	0.006	0.007	4.02E-01	0.005	0.011	6.37E-01
5.53478	0.004	0.009	6.31E-01	-0.009	0.013	4.95E-01
6.190824	-0.004	0.010	6.56E-01	-0.008	0.015	5.67E-01
8.01126	0.003	0.009	7.74E-01	0.005	0.013	7.14E-01
7.24083	-0.010	0.009	2.87E-01	-0.021	0.014	1.24E-01
3.931006	0.005	0.007	4.86E-01	0.021	0.011	6.58E-02
6.581085	0.002	0.009	7.95E-01	0.006	0.013	6.55E-01
7.22569	-0.007	0.010	4.83E-01	-0.005	0.015	7.24E-01

8.822399	0.002	0.008	7.82E-01	0.004	0.012	7.18E-01
7.597785	0.008	0.009	4.06E-01	-0.004	0.014	7.59E-01
6.837783	-0.004	0.009	6.76E-01	0.000	0.014	9.93E-01
5.40559	0.002	0.007	7.53E-01	-0.001	0.011	9.34E-01
8.119928	-0.006	0.009	5.36E-01	-0.011	0.014	4.22E-01
1.227097137	0.003	0.008	6.66E-01	-0.006	0.012	6.27E-01
7.645558	-0.001	0.011	8.98E-01	0.010	0.016	5.47E-01
8.927366	0.005	0.008	5.54E-01	0.002	0.012	8.73E-01
0.602005383	-0.003	0.007	6.50E-01	-0.013	0.011	2.16E-01
2.58561194	0.009	0.013	4.54E-01	0.008	0.019	6.71E-01
8.512545	0.006	0.009	5.13E-01	-0.005	0.014	7.01E-01
8.915255	0.007	0.007	3.26E-01	0.010	0.010	3.12E-01
0.827751652	-0.006	0.008	4.13E-01	0.002	0.011	8.64E-01
6.972356	-0.008	0.009	3.88E-01	-0.002	0.013	8.86E-01
0.510495718	-0.007	0.010	5.04E-01	-0.016	0.015	2.75E-01
7.996121	0.002	0.007	8.00E-01	-0.005	0.011	6.60E-01
5.137453	0.003	0.007	6.35E-01	-0.005	0.011	6.06E-01
5.521996	0.001	0.008	9.28E-01	-0.022	0.012	7.29E-02
4.296708	-0.005	0.013	6.64E-01	0.008	0.019	6.54E-01
3.135343	-0.007	0.012	5.40E-01	-0.008	0.017	6.62E-01
4.098213	-0.002	0.009	7.95E-01	0.004	0.014	7.96E-01
3.932015	-0.003	0.007	6.90E-01	0.014	0.010	1.85E-01
8.693209	-0.005	0.010	6.57E-01	-0.040	0.016	1.03E-02
7.082034	-0.002	0.007	7.91E-01	0.003	0.011	7.98E-01
6.948806	-0.005	0.009	5.57E-01	-0.003	0.013	8.42E-01
7.944983	0.003	0.009	7.52E-01	0.012	0.013	3.85E-01
8.125311	-0.005	0.009	5.84E-01	0.010	0.014	4.55E-01
5.083287	0.005	0.009	6.05E-01	-0.002	0.014	8.94E-01
8.176785	-0.007	0.009	4.41E-01	-0.006	0.014	6.45E-01
8.123965	-0.007	0.009	4.50E-01	0.029	0.014	3.97E-02
4.213273	0.006	0.010	5.65E-01	0.008	0.015	5.63E-01
7.748506	-0.004	0.010	7.20E-01	-0.013	0.015	3.91E-01
3.683391	-0.002	0.004	6.37E-01	0.010	0.007	1.23E-01
8.62996	0.004	0.009	6.41E-01	0.026	0.014	5.46E-02
7.420821	0.001	0.007	8.44E-01	-0.011	0.010	2.88E-01
0.596958894	-0.004	0.010	6.61E-01	0.001	0.014	9.47E-01
7.266399	-0.004	0.011	7.29E-01	-0.017	0.017	3.16E-01
0.629592855	0.005	0.010	6.62E-01	-0.003	0.016	8.31E-01
6.484193	-0.004	0.009	6.82E-01	-0.018	0.013	1.69E-01
7.955076	0.007	0.009	4.29E-01	-0.002	0.013	8.48E-01
8.777317	-0.009	0.009	3.41E-01	-0.010	0.013	4.54E-01
8.147516	-0.004	0.006	5.16E-01	0.016	0.009	8.40E-02
1.688009787	-0.002	0.004	5.50E-01	0.006	0.006	3.23E-01

0.785697578	-0.004	0.004	3.97E-01	0.000	0.006	9.99E-01
8.30833	0.003	0.008	7.19E-01	-0.007	0.013	5.60E-01
6.759731	0.008	0.011	4.37E-01	0.001	0.016	9.47E-01
1.890542207	0.007	0.008	4.10E-01	0.000	0.012	9.83E-01
4.12681	-0.003	0.008	7.32E-01	0.013	0.013	2.89E-01
8.299247	0.007	0.008	4.07E-01	0.005	0.012	6.89E-01
1.22743357	0.003	0.008	6.69E-01	-0.006	0.012	6.10E-01
8.321451	-0.003	0.008	6.62E-01	-0.009	0.011	4.12E-01
5.787104	-0.006	0.010	5.38E-01	-0.018	0.016	2.37E-01
1.480094446	0.008	0.008	3.27E-01	-0.008	0.012	4.64E-01
5.474222	0.005	0.008	5.27E-01	-0.008	0.012	4.92E-01
7.507284	0.006	0.008	4.29E-01	-0.010	0.012	3.79E-01
8.243735	0.003	0.007	6.33E-01	0.007	0.011	5.35E-01
5.878278	0.004	0.009	7.01E-01	-0.008	0.014	5.82E-01
6.81457	0.004	0.009	6.05E-01	-0.006	0.013	6.34E-01
0.957951064	-0.003	0.005	5.04E-01	0.009	0.007	2.41E-01
1.053834353	0.003	0.006	6.49E-01	-0.004	0.009	6.58E-01
2.5738368	-0.004	0.011	6.91E-01	-0.011	0.017	4.94E-01
7.944647	-0.006	0.009	4.94E-01	0.000	0.013	9.98E-01
2.853748715	-0.003	0.009	7.23E-01	-0.011	0.014	4.49E-01
7.671127	0.003	0.008	6.78E-01	-0.011	0.012	3.65E-01
8.89776	0.004	0.007	5.51E-01	-0.001	0.011	9.53E-01
8.757468	-0.003	0.007	6.59E-01	-0.006	0.011	5.66E-01
6.90978	0.007	0.009	4.36E-01	0.000	0.013	9.91E-01
2.518998287	0.015	0.029	6.06E-01	-0.001	0.044	9.77E-01
2.359529239	0.007	0.009	4.61E-01	0.019	0.013	1.44E-01
6.651063	0.005	0.008	5.39E-01	-0.007	0.012	5.56E-01
6.084174	0.006	0.009	5.23E-01	-0.010	0.013	4.52E-01
8.925011	-0.004	0.008	6.61E-01	0.011	0.012	3.88E-01
8.959664	-0.003	0.008	7.14E-01	-0.010	0.011	3.92E-01
2.090383166	-0.002	0.006	6.77E-01	0.014	0.008	1.00E-01
7.123751	0.006	0.008	4.58E-01	-0.008	0.012	5.12E-01
2.559033766	0.001	0.008	9.43E-01	0.008	0.012	5.07E-01
7.505602	-0.003	0.009	7.66E-01	-0.009	0.014	5.23E-01
3.762453	0.005	0.009	5.75E-01	0.019	0.013	1.45E-01
2.153632493	-0.004	0.008	6.43E-01	-0.006	0.013	6.31E-01
1.742511867	-0.006	0.006	3.23E-01	-0.002	0.009	8.31E-01
4.295699	-0.005	0.012	6.86E-01	0.002	0.018	8.93E-01
4.024198	0.009	0.012	4.52E-01	0.017	0.018	3.35E-01
3.205321	0.004	0.009	6.61E-01	0.016	0.013	2.08E-01
0.600996085	-0.003	0.009	7.22E-01	0.001	0.013	9.16E-01
4.283924	0.007	0.011	4.82E-01	0.007	0.016	6.75E-01
2.77906068	-0.007	0.012	5.44E-01	-0.045	0.018	9.94E-03

8.015298	-0.002	0.008	7.70E-01	0.004	0.013	7.23E-01
7.809737	-0.005	0.010	6.43E-01	-0.013	0.015	3.73E-01
6.476118	-0.006	0.010	5.34E-01	-0.012	0.015	4.46E-01
0.802182775	0.000	0.006	9.65E-01	0.006	0.009	4.57E-01
0.65751676	0.004	0.011	6.97E-01	0.007	0.016	6.48E-01
4.181312	-0.007	0.015	6.36E-01	0.010	0.022	6.53E-01
1.896597994	-0.004	0.006	4.94E-01	0.002	0.008	7.86E-01
7.730676	-0.003	0.011	7.73E-01	0.006	0.017	6.96E-01
5.603749	0.003	0.010	7.67E-01	-0.004	0.014	7.60E-01
2.673757279	-0.008	0.011	4.38E-01	0.002	0.016	8.97E-01
5.684156	-0.005	0.009	6.12E-01	-0.019	0.013	1.51E-01
6.76074	-0.004	0.009	6.65E-01	-0.014	0.014	2.89E-01
3.978443	-0.005	0.012	6.90E-01	-0.005	0.017	7.62E-01
7.793588	-0.003	0.010	8.01E-01	0.023	0.015	1.35E-01
7.013401	0.005	0.010	6.38E-01	-0.014	0.015	3.54E-01
8.323133	-0.005	0.009	5.88E-01	0.003	0.013	8.18E-01
2.367603621	-0.001	0.009	9.32E-01	0.028	0.014	3.95E-02
8.944188	0.006	0.007	3.94E-01	-0.003	0.010	7.96E-01
2.077598728	0.000	0.004	9.31E-01	0.009	0.005	9.65E-02
7.764655	-0.005	0.012	6.96E-01	-0.019	0.018	2.68E-01
4.422534	-0.007	0.014	6.37E-01	0.022	0.022	2.98E-01
5.816711	0.006	0.009	4.72E-01	-0.010	0.013	4.32E-01
2.589649131	0.013	0.017	4.20E-01	0.026	0.025	2.97E-01
6.39672	0.003	0.009	7.70E-01	0.003	0.013	8.32E-01
8.246763	-0.002	0.007	7.83E-01	0.012	0.010	2.56E-01
2.634731099	-0.003	0.009	7.34E-01	-0.012	0.013	3.40E-01
8.559982	-0.002	0.008	7.97E-01	0.002	0.012	8.52E-01
4.045729	0.010	0.011	3.45E-01	-0.005	0.016	7.65E-01
8.948562	-0.004	0.009	6.88E-01	-0.015	0.013	2.48E-01
7.620326	-0.012	0.014	4.01E-01	0.005	0.020	8.06E-01
6.407486	-0.004	0.008	5.85E-01	-0.001	0.012	9.42E-01
8.259548	-0.005	0.009	5.65E-01	0.006	0.014	6.41E-01
2.264655248	0.004	0.007	5.56E-01	0.016	0.011	1.18E-01
6.469053	-0.006	0.008	4.92E-01	-0.014	0.012	2.41E-01
6.016552	0.003	0.007	6.40E-01	0.003	0.011	7.50E-01
0.624546367	-0.003	0.010	7.54E-01	-0.018	0.015	2.23E-01
5.637056	0.006	0.009	5.23E-01	-0.008	0.014	5.42E-01
1.658067287	0.003	0.005	5.11E-01	0.012	0.007	9.89E-02
6.117145	-0.002	0.009	7.79E-01	0.023	0.013	8.02E-02
7.175562	-0.007	0.011	5.12E-01	-0.022	0.016	1.61E-01
2.802610962	-0.007	0.014	6.00E-01	-0.066	0.020	1.26E-03
0.997986543	0.003	0.006	5.64E-01	0.014	0.009	1.30E-01
5.917977	0.002	0.008	7.84E-01	-0.008	0.012	5.21E-01

3.722418	0.003	0.007	6.57E-01	0.012	0.011	2.80E-01
6.218747	0.005	0.009	5.72E-01	-0.016	0.013	2.01E-01
1.811480548	-0.004	0.010	7.13E-01	-0.017	0.015	2.81E-01
5.837906	0.004	0.009	6.43E-01	-0.005	0.013	6.99E-01
7.909658	0.004	0.011	7.14E-01	-0.006	0.016	7.06E-01
5.73563	0.005	0.008	5.33E-01	-0.003	0.012	8.28E-01
7.472632	0.006	0.008	4.30E-01	-0.006	0.012	6.37E-01
5.981226	-0.009	0.011	4.09E-01	0.014	0.016	3.84E-01
5.434187	0.002	0.008	8.08E-01	0.001	0.012	9.55E-01
7.88207	0.005	0.008	5.74E-01	0.018	0.012	1.52E-01
2.330932469	0.004	0.006	4.93E-01	-0.004	0.008	5.95E-01
6.433391	-0.006	0.009	5.08E-01	0.000	0.013	9.90E-01
6.107052	0.004	0.009	6.17E-01	-0.005	0.013	7.12E-01
2.804629557	-0.007	0.014	6.18E-01	-0.067	0.021	1.69E-03
0.78468828	-0.003	0.004	4.33E-01	-0.003	0.006	6.48E-01
7.685257	0.008	0.010	3.83E-01	-0.005	0.014	7.47E-01
7.690977	0.004	0.010	7.09E-01	0.001	0.015	9.51E-01
5.575488	0.005	0.008	4.90E-01	-0.039	0.012	8.84E-04
1.835703695	-0.003	0.010	7.43E-01	0.002	0.014	8.69E-01
2.532119158	-0.004	0.012	7.26E-01	-0.010	0.017	5.53E-01
4.359285	-0.005	0.013	6.88E-01	0.006	0.019	7.44E-01
2.674430144	-0.008	0.010	4.38E-01	0.001	0.015	9.68E-01
7.29365	-0.007	0.010	5.26E-01	-0.013	0.016	3.92E-01
8.502115	0.003	0.008	6.93E-01	0.014	0.011	2.20E-01
2.617573036	-0.008	0.013	5.21E-01	0.023	0.020	2.48E-01
8.301602	0.005	0.007	4.88E-01	0.007	0.011	5.32E-01
6.717677	-0.008	0.011	4.75E-01	0.008	0.016	6.23E-01
1.048451431	0.003	0.013	7.97E-01	-0.010	0.019	5.93E-01
7.276828	-0.010	0.009	2.86E-01	-0.007	0.014	6.29E-01
1.919811842	0.002	0.005	7.02E-01	-0.011	0.008	1.85E-01
0.971408368	-0.002	0.005	6.60E-01	0.011	0.008	1.47E-01
6.145069	-0.002	0.008	7.87E-01	-0.004	0.012	7.70E-01
5.803253	-0.008	0.009	3.80E-01	-0.013	0.013	3.40E-01
7.730003	-0.002	0.011	8.29E-01	0.009	0.016	5.73E-01
8.35644	0.005	0.009	6.10E-01	0.004	0.014	7.48E-01
0.820013702	0.001	0.006	8.60E-01	0.012	0.009	1.64E-01
6.156171	-0.006	0.008	5.04E-01	0.001	0.012	9.37E-01
5.488016	0.003	0.008	6.96E-01	-0.006	0.012	5.96E-01
6.519182	-0.004	0.009	6.33E-01	-0.025	0.013	5.70E-02
5.056709	0.004	0.009	6.73E-01	-0.005	0.013	7.24E-01
6.859988	-0.004	0.008	6.12E-01	-0.004	0.012	7.38E-01
6.76175	-0.001	0.008	8.75E-01	-0.013	0.011	2.51E-01
6.617757	-0.006	0.009	5.11E-01	-0.009	0.014	4.99E-01

0.748017127	-0.003	0.006	5.99E-01	-0.011	0.008	1.94E-01
3.998629	0.002	0.009	8.10E-01	0.009	0.014	5.34E-01
6.457615	-0.002	0.008	8.38E-01	0.023	0.012	5.08E-02
8.824418	0.009	0.009	3.31E-01	0.011	0.014	4.25E-01
0.560960607	-0.006	0.008	4.93E-01	-0.003	0.013	8.18E-01
8.53374	0.002	0.006	7.57E-01	-0.005	0.009	5.82E-01
2.532792023	-0.006	0.012	6.06E-01	-0.003	0.018	8.76E-01
8.123629	-0.003	0.007	6.94E-01	0.005	0.010	6.51E-01
1.711896501	-0.002	0.004	5.95E-01	0.007	0.006	2.84E-01
4.236823	0.006	0.013	6.28E-01	0.016	0.019	3.99E-01
2.790499388	-0.007	0.012	5.85E-01	-0.055	0.018	2.33E-03
5.61687	0.003	0.011	7.79E-01	0.010	0.016	5.22E-01
3.371855	0.000	0.011	9.95E-01	0.006	0.017	7.04E-01
8.804568	0.005	0.007	5.05E-01	-0.017	0.011	1.12E-01
3.295485	-0.004	0.011	7.49E-01	-0.005	0.017	7.84E-01
2.722876437	-0.006	0.009	5.48E-01	0.002	0.014	8.86E-01
6.337845	-0.003	0.008	6.63E-01	0.008	0.011	4.69E-01
6.057933	0.001	0.008	9.27E-01	0.003	0.012	8.04E-01
8.321788	0.004	0.008	5.93E-01	-0.008	0.013	5.32E-01
7.350843	-0.006	0.010	5.33E-01	-0.007	0.015	6.47E-01
8.526002	0.001	0.009	8.84E-01	0.010	0.014	4.88E-01
8.720124	0.005	0.009	5.98E-01	-0.007	0.014	6.09E-01
6.628859	0.003	0.008	7.41E-01	-0.007	0.011	5.57E-01
5.409627	0.004	0.007	5.50E-01	-0.003	0.010	7.90E-01
5.612496	-0.005	0.008	5.33E-01	-0.010	0.012	4.02E-01
5.887025	-0.005	0.009	5.64E-01	-0.007	0.013	6.00E-01
4.284597	0.007	0.010	4.99E-01	0.014	0.016	3.80E-01
7.803681	-0.005	0.009	5.53E-01	-0.013	0.014	3.38E-01
6.427672	0.005	0.007	4.13E-01	0.003	0.010	7.58E-01
6.042457	-0.003	0.010	7.83E-01	0.034	0.015	2.34E-02
7.130816	-0.011	0.012	3.76E-01	-0.018	0.018	3.12E-01
6.870754	0.006	0.010	5.43E-01	0.003	0.014	8.59E-01
7.496182	0.001	0.008	8.88E-01	0.021	0.012	9.62E-02
1.086804747	0.004	0.008	6.04E-01	-0.014	0.011	2.11E-01
7.076651	0.004	0.008	5.96E-01	0.015	0.012	2.37E-01
7.88779	0.004	0.008	6.12E-01	0.021	0.012	8.79E-02
0.594267433	-0.003	0.007	6.56E-01	-0.004	0.011	7.18E-01
7.422167	-0.005	0.007	4.76E-01	-0.012	0.011	2.76E-01
5.903174	-0.010	0.009	2.67E-01	-0.001	0.013	9.17E-01
8.979513	0.004	0.009	6.47E-01	-0.005	0.013	6.86E-01
5.618552	0.005	0.007	5.20E-01	-0.016	0.011	1.50E-01
7.517041	0.001	0.010	9.21E-01	-0.026	0.015	8.57E-02
7.011046	0.003	0.010	7.28E-01	-0.017	0.015	2.50E-01

6.283343	-0.005	0.010	6.17E-01	-0.008	0.014	6.00E-01
5.241074	-0.001	0.004	7.66E-01	0.004	0.006	5.87E-01
8.717096	-0.007	0.009	4.26E-01	-0.021	0.013	1.04E-01
5.753125	-0.012	0.011	2.63E-01	-0.019	0.016	2.45E-01
7.482725	-0.006	0.009	4.73E-01	0.000	0.013	9.81E-01
7.340414	0.004	0.010	6.91E-01	0.017	0.015	2.69E-01
6.665866	0.005	0.008	5.88E-01	-0.004	0.012	7.32E-01
3.724436	0.001	0.008	9.35E-01	-0.002	0.012	9.00E-01
3.919567	0.000	0.011	9.67E-01	0.027	0.016	9.39E-02
5.069157	0.003	0.007	6.38E-01	-0.012	0.010	2.44E-01
6.80885	0.001	0.009	8.85E-01	-0.017	0.014	2.37E-01
5.100446	0.005	0.009	5.72E-01	-0.010	0.014	4.83E-01
6.100323	0.004	0.008	6.43E-01	0.002	0.011	8.71E-01
1.231470761	0.003	0.008	6.87E-01	-0.008	0.012	4.99E-01
2.502176658	-0.008	0.013	5.13E-01	-0.003	0.019	8.72E-01
8.438193	0.002	0.007	7.62E-01	-0.014	0.011	2.22E-01
2.331941767	0.004	0.006	5.26E-01	0.000	0.009	9.78E-01
1.419200147	0.004	0.005	3.83E-01	0.005	0.007	5.35E-01
8.307657	0.002	0.010	8.01E-01	0.002	0.015	8.79E-01
3.325091	-0.006	0.010	5.32E-01	0.011	0.015	4.65E-01
4.297717	-0.006	0.013	6.26E-01	0.012	0.019	5.33E-01
8.543833	-0.004	0.008	5.66E-01	0.008	0.011	4.61E-01
8.488322	0.004	0.007	6.31E-01	-0.001	0.011	9.17E-01
5.042579	0.003	0.007	6.70E-01	-0.002	0.011	8.52E-01
8.920301	0.002	0.006	7.00E-01	0.000	0.009	9.72E-01
6.978412	-0.004	0.010	6.93E-01	0.009	0.015	5.21E-01
7.331667	-0.005	0.006	4.10E-01	0.000	0.009	9.93E-01
2.529427698	0.008	0.013	5.07E-01	0.005	0.019	7.79E-01
6.252391	0.009	0.010	3.74E-01	-0.001	0.015	9.28E-01
6.836774	-0.002	0.008	8.31E-01	0.006	0.012	5.93E-01
2.778724248	-0.007	0.012	5.42E-01	-0.044	0.018	1.29E-02
8.893387	0.003	0.008	6.79E-01	-0.004	0.012	7.57E-01
3.035759	0.003	0.007	6.44E-01	-0.004	0.011	6.91E-01
0.534718865	0.006	0.008	5.00E-01	-0.011	0.012	3.67E-01
8.595644	0.004	0.008	6.55E-01	0.007	0.012	5.40E-01
3.279	-0.007	0.010	5.13E-01	0.004	0.015	7.83E-01
3.804171	0.004	0.006	4.80E-01	0.009	0.008	2.81E-01
0.637330805	-0.003	0.010	7.66E-01	-0.006	0.015	6.90E-01
6.392347	-0.005	0.010	5.81E-01	-0.031	0.014	2.90E-02
4.177947	-0.006	0.014	6.88E-01	0.026	0.021	2.21E-01
1.749913384	-0.007	0.007	3.59E-01	0.007	0.011	5.22E-01
3.173023	-0.003	0.006	5.91E-01	0.005	0.009	5.38E-01
4.377115	-0.007	0.015	6.20E-01	0.023	0.022	2.95E-01

8.088976	-0.004	0.008	6.35E-01	0.002	0.013	8.81E-01
6.11479	0.007	0.010	4.79E-01	0.004	0.014	7.60E-01
6.692445	-0.003	0.008	6.81E-01	0.006	0.012	6.07E-01
4.372405	-0.005	0.012	6.74E-01	0.010	0.018	5.61E-01
2.546249327	-0.013	0.026	6.17E-01	-0.036	0.039	3.51E-01
4.286615	0.007	0.011	5.38E-01	0.016	0.016	3.19E-01
0.707308784	0.004	0.009	6.64E-01	0.005	0.014	7.32E-01
2.171126988	0.006	0.010	5.12E-01	-0.007	0.014	6.44E-01
6.308575	-0.003	0.008	6.54E-01	-0.007	0.012	5.27E-01
8.950244	0.002	0.008	7.71E-01	-0.022	0.012	6.88E-02
2.577873991	0.000	0.008	9.68E-01	0.011	0.013	3.62E-01
5.045607	0.002	0.007	7.31E-01	-0.001	0.010	9.39E-01
8.308667	0.002	0.007	7.39E-01	0.000	0.011	9.64E-01
7.678529	0.003	0.009	7.34E-01	0.004	0.014	7.83E-01
5.909902	0.002	0.007	8.02E-01	-0.007	0.011	5.45E-01
3.687092	-0.002	0.005	6.72E-01	0.008	0.007	2.46E-01
6.194861	-0.003	0.008	7.00E-01	-0.009	0.013	4.91E-01
6.693454	0.004	0.007	6.07E-01	0.013	0.011	2.21E-01
8.071818	-0.007	0.009	4.33E-01	-0.009	0.013	5.05E-01
7.321574	-0.006	0.006	3.18E-01	-0.002	0.009	8.57E-01
4.110997	-0.004	0.010	7.11E-01	0.000	0.015	9.82E-01
3.771537	0.001	0.008	9.50E-01	0.010	0.013	4.23E-01
2.780406411	-0.007	0.012	5.67E-01	-0.044	0.018	1.37E-02
8.373262	0.004	0.010	7.00E-01	0.020	0.015	1.76E-01
0.548512601	0.005	0.010	6.20E-01	-0.012	0.014	4.18E-01
5.55833	0.003	0.007	6.25E-01	0.007	0.011	5.33E-01
6.136322	0.003	0.009	7.20E-01	-0.023	0.014	9.81E-02
2.551632249	-0.008	0.012	4.92E-01	-0.013	0.018	4.65E-01
4.218319	-0.004	0.011	7.17E-01	0.029	0.017	8.43E-02
0.668619036	-0.006	0.010	5.65E-01	-0.026	0.015	7.85E-02
7.107939	-0.001	0.010	9.42E-01	0.024	0.014	9.80E-02
6.513462	-0.004	0.009	6.41E-01	-0.006	0.014	6.70E-01
8.925348	0.006	0.008	4.76E-01	0.018	0.012	1.49E-01
0.756427942	-0.002	0.005	7.63E-01	0.001	0.008	9.45E-01
0.948530952	0.003	0.005	5.60E-01	-0.002	0.008	8.46E-01
1.047105701	0.002	0.008	7.89E-01	-0.015	0.012	2.17E-01
5.986609	0.004	0.009	6.29E-01	-0.027	0.013	3.60E-02
0.772240274	0.004	0.005	3.78E-01	0.001	0.007	9.02E-01
5.900819	-0.006	0.009	5.15E-01	-0.018	0.014	1.90E-01
6.569983	0.001	0.009	9.10E-01	-0.020	0.014	1.57E-01
5.767591	0.001	0.009	9.36E-01	0.004	0.014	7.50E-01
6.891613	-0.001	0.010	9.52E-01	-0.006	0.014	6.74E-01
8.824754	0.000	0.009	9.95E-01	0.000	0.014	9.84E-01

6.320687	0.007	0.009	4.75E-01	0.005	0.014	7.25E-01
6.149442	0.007	0.009	4.67E-01	-0.007	0.014	6.35E-01
4.358612	-0.006	0.013	6.42E-01	0.008	0.019	6.67E-01
3.614086	-0.003	0.007	7.13E-01	0.010	0.011	3.58E-01
7.036615	-0.011	0.012	3.65E-01	-0.024	0.018	1.84E-01
2.184247859	0.007	0.011	5.17E-01	-0.021	0.016	1.84E-01
8.498415	-0.007	0.010	4.70E-01	0.009	0.014	5.46E-01
8.069127	0.002	0.008	7.92E-01	0.009	0.013	4.61E-01
4.286279	0.007	0.011	5.37E-01	0.017	0.016	2.95E-01
6.148097	0.003	0.007	7.18E-01	-0.015	0.010	1.55E-01
0.571390017	-0.001	0.009	9.36E-01	0.005	0.014	7.06E-01
5.534444	0.005	0.010	6.03E-01	0.001	0.016	9.35E-01
3.993919	-0.007	0.010	5.10E-01	0.003	0.015	8.23E-01
8.548207	-0.005	0.008	5.68E-01	-0.010	0.012	4.06E-01
6.019243	0.003	0.009	7.10E-01	0.003	0.014	8.28E-01
5.729575	0.001	0.009	8.83E-01	-0.011	0.014	4.48E-01
7.291967	-0.007	0.010	5.05E-01	-0.006	0.015	6.90E-01
7.140909	-0.006	0.010	5.90E-01	-0.012	0.016	4.51E-01
8.77328	-0.005	0.010	5.96E-01	-0.013	0.014	3.74E-01
0.827415219	-0.006	0.008	4.28E-01	0.002	0.011	8.41E-01
2.779397113	-0.007	0.012	5.53E-01	-0.046	0.018	8.73E-03
8.532394	0.003	0.007	7.02E-01	-0.009	0.011	4.17E-01
7.571879	-0.001	0.009	9.15E-01	-0.001	0.013	9.23E-01
8.116227	0.006	0.009	4.65E-01	-0.018	0.013	1.66E-01
7.231746	-0.008	0.011	4.88E-01	0.007	0.016	6.68E-01
5.932443	0.003	0.006	6.09E-01	-0.003	0.010	7.66E-01
0.792089797	-0.004	0.005	4.41E-01	0.000	0.008	9.53E-01
8.833838	0.004	0.009	6.79E-01	-0.010	0.014	4.74E-01
2.522362613	-0.005	0.013	6.81E-01	-0.005	0.019	7.75E-01
6.89094	0.006	0.010	5.12E-01	0.002	0.014	8.98E-01
7.242175	-0.004	0.008	5.75E-01	-0.010	0.012	3.92E-01
1.748231221	-0.004	0.007	5.75E-01	0.007	0.011	5.47E-01
8.236334	-0.003	0.009	7.26E-01	0.006	0.013	6.29E-01
8.424736	-0.005	0.009	5.39E-01	-0.015	0.013	2.32E-01
7.546983	0.005	0.010	5.97E-01	-0.024	0.014	9.31E-02
0.733214093	-0.006	0.007	4.09E-01	0.005	0.010	6.26E-01
6.668221	0.003	0.007	6.44E-01	-0.017	0.010	1.03E-01
0.535055297	-0.005	0.009	5.86E-01	-0.033	0.014	1.47E-02
4.184003	-0.005	0.014	7.02E-01	0.003	0.021	8.82E-01
6.762423	0.005	0.008	5.31E-01	0.007	0.012	5.66E-01
0.902439687	0.005	0.007	4.79E-01	-0.019	0.010	6.11E-02
4.372069	-0.005	0.011	6.63E-01	0.008	0.017	6.21E-01
2.083318082	-0.003	0.007	7.13E-01	0.011	0.011	3.07E-01

6.931312	0.000	0.008	9.53E-01	0.006	0.012	6.10E-01
5.773984	0.004	0.008	5.89E-01	-0.003	0.012	7.72E-01
6.390665	0.005	0.009	5.63E-01	-0.003	0.013	8.39E-01
7.789551	-0.007	0.011	5.33E-01	-0.001	0.016	9.62E-01
0.567352826	-0.004	0.007	5.96E-01	-0.016	0.010	1.22E-01
8.794475	0.004	0.007	5.64E-01	-0.010	0.011	3.58E-01
7.8962	-0.004	0.009	6.54E-01	0.000	0.013	9.81E-01
3.565977	-0.003	0.007	6.81E-01	0.022	0.010	2.53E-02
7.408037	0.003	0.007	7.12E-01	-0.010	0.011	3.54E-01
1.042732077	0.001	0.007	8.36E-01	0.021	0.010	3.85E-02
2.503858821	-0.011	0.013	4.02E-01	-0.025	0.019	1.99E-01
3.761107	0.004	0.008	6.01E-01	0.027	0.012	2.48E-02
6.69379	0.004	0.008	5.87E-01	0.000	0.012	9.69E-01
7.000953	0.007	0.011	5.19E-01	-0.010	0.016	5.27E-01
1.689019085	-0.002	0.004	6.14E-01	0.003	0.005	6.32E-01
6.447858	-0.001	0.010	8.95E-01	-0.037	0.015	1.57E-02
4.028571	-0.005	0.012	6.82E-01	0.000	0.017	9.94E-01
7.332676	-0.004	0.006	4.56E-01	-0.006	0.009	4.76E-01
6.40715	0.003	0.007	6.80E-01	0.007	0.010	5.22E-01
5.688866	0.004	0.007	5.90E-01	-0.010	0.011	3.47E-01
6.579403	-0.006	0.008	4.12E-01	-0.019	0.012	1.07E-01
6.120173	-0.004	0.007	5.46E-01	0.000	0.010	9.98E-01
5.829831	0.005	0.010	6.12E-01	0.014	0.015	3.35E-01
6.621794	-0.003	0.009	7.51E-01	0.007	0.014	6.15E-01
1.728045265	-0.002	0.005	6.60E-01	0.004	0.007	5.65E-01
0.686449963	0.007	0.015	6.39E-01	-0.021	0.022	3.39E-01
6.70321	-0.004	0.008	6.31E-01	-0.012	0.012	3.19E-01
6.034046	-0.004	0.007	5.47E-01	0.003	0.011	7.77E-01
8.253828	0.006	0.007	4.31E-01	0.003	0.011	7.56E-01
1.086131882	0.005	0.008	5.39E-01	-0.012	0.012	3.07E-01
6.639625	-0.004	0.008	6.66E-01	-0.015	0.012	2.12E-01
1.034657695	0.002	0.005	6.26E-01	-0.003	0.007	6.56E-01
2.087691705	-0.003	0.006	6.64E-01	0.013	0.009	1.51E-01
0.558605579	-0.001	0.007	9.36E-01	-0.004	0.010	7.06E-01
8.921983	-0.004	0.007	5.63E-01	0.010	0.010	3.55E-01
6.27695	-0.003	0.009	7.66E-01	-0.014	0.013	2.86E-01
5.467157	0.003	0.007	6.49E-01	-0.004	0.010	6.89E-01
6.803467	0.003	0.010	7.31E-01	-0.025	0.015	1.02E-01
7.841362	0.004	0.008	6.69E-01	-0.018	0.013	1.52E-01
5.834878	0.005	0.009	5.82E-01	0.009	0.014	5.18E-01
4.101914	0.000	0.011	9.83E-01	0.010	0.016	5.25E-01
4.091148	0.011	0.012	3.68E-01	0.013	0.017	4.70E-01
8.570748	-0.006	0.008	4.71E-01	-0.003	0.012	8.02E-01

3.563958	0.004	0.006	5.65E-01	0.016	0.009	8.99E-02
8.212783	-0.006	0.007	3.63E-01	0.010	0.010	3.23E-01
7.514013	0.003	0.007	6.19E-01	0.004	0.010	7.18E-01
0.801846342	0.000	0.006	9.57E-01	0.006	0.009	4.87E-01
8.739637	0.003	0.009	7.77E-01	0.009	0.014	5.09E-01
1.74419403	0.002	0.006	7.12E-01	0.012	0.009	1.90E-01
6.307229	0.005	0.009	5.81E-01	0.013	0.013	3.47E-01
0.645068755	-0.005	0.010	5.96E-01	-0.002	0.015	9.09E-01
1.826283582	-0.006	0.009	5.52E-01	0.009	0.014	5.33E-01
1.227770002	0.003	0.008	6.89E-01	-0.006	0.012	5.93E-01
7.430914	0.002	0.010	8.18E-01	0.002	0.015	8.76E-01
7.078333	0.003	0.008	6.81E-01	0.027	0.013	3.57E-02
4.388891	-0.006	0.013	6.60E-01	0.023	0.020	2.45E-01
7.286921	0.003	0.010	7.81E-01	0.004	0.015	7.65E-01
8.518601	0.002	0.011	8.77E-01	-0.007	0.016	6.57E-01
8.549216	-0.005	0.008	5.35E-01	-0.013	0.012	2.64E-01
8.393111	-0.003	0.009	7.03E-01	-0.022	0.014	1.04E-01
1.712232934	-0.002	0.004	6.47E-01	0.009	0.006	1.72E-01
8.260893	-0.005	0.008	5.13E-01	-0.016	0.012	1.78E-01
7.43697	0.006	0.011	6.02E-01	0.001	0.016	9.57E-01
8.21312	0.001	0.006	8.64E-01	0.011	0.009	1.91E-01
7.096837	-0.005	0.010	6.39E-01	0.000	0.015	9.85E-01
8.743338	-0.005	0.008	5.41E-01	-0.004	0.012	7.26E-01
6.144732	0.003	0.008	6.47E-01	0.003	0.011	8.12E-01
8.281079	-0.002	0.007	7.71E-01	-0.006	0.011	5.86E-01
7.504256	-0.003	0.009	7.45E-01	-0.011	0.013	4.15E-01
0.789061904	-0.004	0.005	3.67E-01	-0.003	0.007	6.77E-01
7.437979	0.004	0.009	6.76E-01	0.008	0.014	5.63E-01
8.825427	0.003	0.007	6.58E-01	0.004	0.010	7.02E-01
0.562306337	0.007	0.009	4.21E-01	-0.006	0.013	6.66E-01
3.926969	-0.001	0.009	8.79E-01	0.015	0.013	2.57E-01
6.159199	0.001	0.006	8.51E-01	0.012	0.009	1.98E-01
6.435074	0.003	0.009	7.28E-01	-0.001	0.013	9.31E-01
5.110875	0.005	0.009	5.74E-01	-0.017	0.013	2.06E-01
7.754562	-0.003	0.010	7.93E-01	-0.006	0.015	6.87E-01
3.325427	-0.006	0.010	5.70E-01	0.011	0.015	4.30E-01
6.266857	0.010	0.009	2.49E-01	-0.004	0.014	7.95E-01
3.274289	-0.006	0.011	5.81E-01	0.027	0.017	1.11E-01
3.190181	-0.003	0.006	6.48E-01	0.012	0.010	1.94E-01
1.761688525	-0.006	0.009	4.80E-01	0.014	0.013	2.87E-01
7.193056	0.005	0.009	5.98E-01	-0.004	0.013	7.63E-01
7.010373	-0.007	0.009	4.73E-01	-0.021	0.014	1.29E-01
5.43385	0.003	0.009	7.70E-01	0.005	0.013	6.79E-01

8.009242	-0.004	0.008	6.22E-01	0.017	0.012	1.69E-01
6.831055	0.004	0.010	6.38E-01	0.014	0.014	3.32E-01
4.135557	-0.005	0.013	6.73E-01	0.016	0.019	3.91E-01
6.343228	-0.002	0.007	7.60E-01	0.001	0.011	9.47E-01
2.388798874	-0.006	0.014	6.53E-01	-0.008	0.020	7.08E-01
5.893417	0.007	0.010	5.11E-01	-0.019	0.015	2.07E-01
8.186878	0.006	0.010	5.57E-01	0.001	0.014	9.37E-01
7.487771	0.003	0.010	7.60E-01	-0.018	0.014	2.12E-01
2.332614632	0.003	0.006	5.49E-01	0.002	0.008	8.15E-01
5.910912	-0.005	0.009	5.97E-01	-0.012	0.013	3.52E-01
8.647454	0.003	0.011	7.66E-01	-0.005	0.016	7.49E-01
7.444708	0.007	0.009	3.82E-01	0.017	0.013	1.91E-01
2.674093712	-0.008	0.011	4.52E-01	0.002	0.016	9.23E-01
5.656232	0.000	0.011	9.69E-01	-0.033	0.016	3.70E-02
3.190518	-0.003	0.006	6.71E-01	0.014	0.010	1.57E-01
0.902776119	0.005	0.007	4.84E-01	-0.019	0.010	5.52E-02
5.837233	-0.005	0.011	6.60E-01	-0.007	0.016	6.60E-01
8.359132	0.003	0.009	7.57E-01	0.009	0.013	4.99E-01
5.287839	-0.006	0.009	5.34E-01	-0.026	0.013	5.01E-02
5.518295	-0.005	0.008	5.77E-01	-0.013	0.012	2.98E-01
5.45841	-0.005	0.009	5.91E-01	-0.009	0.013	4.86E-01
6.26854	0.007	0.009	4.54E-01	-0.005	0.013	7.06E-01
8.952935	0.005	0.007	5.20E-01	-0.023	0.011	3.65E-02
0.646414485	-0.005	0.009	6.00E-01	-0.005	0.014	6.96E-01
8.839894	0.007	0.008	3.66E-01	-0.023	0.012	6.57E-02
6.600262	0.001	0.007	8.37E-01	0.000	0.010	9.74E-01
5.720154	0.004	0.010	6.90E-01	0.012	0.015	4.16E-01
6.27796	-0.003	0.008	7.31E-01	-0.008	0.012	5.13E-01
8.159291	0.004	0.008	6.40E-01	-0.027	0.012	2.69E-02
5.06041	0.003	0.007	6.91E-01	-0.008	0.010	4.58E-01
7.526461	-0.004	0.010	7.01E-01	-0.026	0.014	6.46E-02
8.779336	0.003	0.006	6.64E-01	0.004	0.009	6.43E-01
7.022149	-0.010	0.010	3.10E-01	-0.021	0.014	1.39E-01
6.869072	-0.003	0.010	7.32E-01	-0.002	0.015	8.89E-01
1.462263518	-0.001	0.004	6.92E-01	0.002	0.006	7.41E-01
8.912563	-0.005	0.008	5.18E-01	0.003	0.012	7.91E-01
6.864362	-0.004	0.009	6.77E-01	-0.004	0.013	7.54E-01
7.382468	-0.005	0.009	5.82E-01	-0.018	0.013	1.74E-01
6.610355	-0.004	0.010	6.91E-01	-0.009	0.014	5.39E-01
3.736884	-0.010	0.009	2.70E-01	0.029	0.014	3.96E-02
7.53689	-0.004	0.011	7.21E-01	-0.012	0.016	4.32E-01
0.994285784	0.002	0.006	6.73E-01	0.009	0.008	2.85E-01
8.099742	-0.006	0.008	4.85E-01	-0.030	0.013	1.86E-02

1.050133594	-0.003	0.007	6.26E-01	-0.017	0.010	9.00E-02
2.78814436	-0.006	0.012	6.13E-01	-0.049	0.018	5.13E-03
6.969665	-0.005	0.008	4.74E-01	-0.003	0.011	7.78E-01
8.68177	-0.003	0.007	6.28E-01	0.002	0.010	8.79E-01
6.656783	-0.005	0.009	6.19E-01	0.017	0.014	2.23E-01
7.25025	-0.005	0.007	4.44E-01	0.001	0.011	9.52E-01
7.594757	-0.003	0.010	7.21E-01	-0.002	0.014	8.83E-01
5.296586	0.006	0.012	5.96E-01	-0.024	0.018	1.92E-01
3.314661	-0.006	0.010	5.63E-01	0.004	0.015	7.98E-01
2.265328114	0.004	0.007	5.83E-01	0.021	0.011	5.57E-02
7.449081	-0.005	0.007	4.39E-01	0.005	0.011	6.15E-01
8.020008	0.001	0.009	9.01E-01	-0.014	0.013	3.13E-01
3.233245	-0.003	0.006	6.06E-01	0.012	0.009	1.85E-01
1.131550281	0.005	0.007	5.23E-01	-0.012	0.011	2.92E-01
4.007713	-0.008	0.011	5.00E-01	0.008	0.017	6.59E-01
3.331819	0.004	0.010	6.95E-01	0.017	0.015	2.74E-01
0.529672376	0.005	0.008	5.75E-01	0.006	0.012	6.20E-01
6.909107	-0.001	0.008	9.28E-01	0.002	0.013	8.80E-01
8.292854	0.003	0.007	7.33E-01	-0.006	0.011	5.90E-01
2.566771715	-0.005	0.007	4.99E-01	-0.003	0.011	7.68E-01
6.431373	0.003	0.008	7.31E-01	0.025	0.013	4.34E-02
7.490126	0.004	0.006	5.04E-01	0.006	0.010	5.42E-01
8.069463	0.004	0.010	6.85E-01	-0.005	0.015	7.43E-01
7.270099	-0.007	0.009	3.93E-01	-0.004	0.013	7.46E-01
4.212936	0.007	0.011	5.55E-01	0.006	0.017	7.03E-01
8.769579	-0.001	0.010	9.26E-01	-0.017	0.015	2.48E-01
8.72517	0.006	0.010	5.36E-01	-0.026	0.015	9.29E-02
7.416111	0.003	0.008	6.69E-01	0.007	0.012	5.52E-01
7.94532	0.003	0.008	6.76E-01	0.014	0.012	2.33E-01
7.771047	-0.007	0.011	5.04E-01	-0.028	0.016	7.76E-02
0.579464399	0.004	0.008	6.30E-01	0.009	0.012	4.36E-01
6.503369	-0.005	0.010	6.40E-01	-0.008	0.015	5.81E-01
8.767224	0.002	0.006	7.27E-01	0.014	0.010	1.50E-01
8.272668	0.005	0.009	5.77E-01	0.008	0.014	5.62E-01
8.092677	-0.003	0.009	7.58E-01	0.003	0.014	8.34E-01
6.629195	0.005	0.009	5.79E-01	-0.026	0.014	5.95E-02
7.098182	-0.004	0.009	6.57E-01	-0.015	0.013	2.60E-01
7.43226	0.004	0.010	6.66E-01	-0.010	0.015	5.21E-01
8.521292	0.004	0.009	6.80E-01	-0.001	0.014	9.44E-01
6.437765	0.005	0.009	5.97E-01	-0.006	0.014	6.59E-01
7.002299	-0.002	0.007	7.47E-01	-0.009	0.010	3.60E-01
8.493032	-0.005	0.010	5.80E-01	-0.010	0.015	4.81E-01
1.921157573	-0.003	0.006	5.98E-01	-0.006	0.009	4.69E-01

6.122864	0.004	0.007	5.80E-01	-0.010	0.010	3.06E-01
0.854329826	-0.004	0.007	6.09E-01	-0.011	0.011	2.94E-01
8.476883	0.004	0.007	5.62E-01	-0.019	0.011	9.23E-02
8.154917	0.004	0.010	6.69E-01	-0.034	0.015	2.24E-02
7.083379	0.004	0.008	6.21E-01	-0.007	0.012	5.81E-01
6.580749	0.003	0.009	7.03E-01	-0.011	0.013	4.07E-01
8.24239	0.007	0.008	4.17E-01	-0.003	0.013	8.01E-01
6.093595	0.004	0.010	7.06E-01	0.003	0.015	8.39E-01
3.113811	-0.009	0.012	4.47E-01	0.021	0.018	2.40E-01
7.118032	-0.004	0.009	6.43E-01	0.012	0.013	3.58E-01
3.925623	-0.001	0.009	8.66E-01	0.018	0.013	1.61E-01
3.682719	-0.002	0.004	7.34E-01	0.013	0.007	4.64E-02
2.521689748	-0.006	0.012	6.05E-01	0.003	0.018	8.52E-01
7.564478	0.005	0.010	6.32E-01	-0.009	0.015	5.59E-01
7.649595	0.000	0.009	9.90E-01	-0.015	0.014	2.72E-01
7.419475	0.003	0.007	7.11E-01	-0.001	0.011	9.36E-01
8.953608	-0.003	0.010	7.56E-01	-0.003	0.015	8.65E-01
8.13574	0.002	0.006	7.53E-01	-0.003	0.010	7.26E-01
7.189692	-0.005	0.009	5.73E-01	-0.009	0.014	5.18E-01
7.103565	-0.004	0.009	6.29E-01	0.006	0.013	6.67E-01
1.316588207	0.009	0.009	2.95E-01	-0.001	0.013	9.64E-01
8.977495	-0.003	0.009	7.57E-01	0.005	0.014	7.35E-01
2.650206998	-0.008	0.017	6.44E-01	0.014	0.025	5.81E-01
5.55934	0.004	0.008	6.73E-01	-0.008	0.012	5.07E-01
5.674736	0.005	0.009	5.91E-01	-0.018	0.014	1.90E-01
6.346928	0.008	0.009	3.52E-01	-0.013	0.013	3.10E-01
6.138677	0.004	0.008	6.69E-01	-0.004	0.012	7.24E-01
7.691313	0.004	0.010	6.68E-01	0.008	0.015	5.93E-01
8.719115	-0.003	0.009	7.35E-01	0.017	0.013	1.84E-01
6.568974	-0.005	0.008	5.67E-01	-0.003	0.013	8.25E-01
6.549797	-0.005	0.010	6.41E-01	0.007	0.015	6.27E-01
7.606532	-0.002	0.012	8.87E-01	0.006	0.018	7.48E-01
1.231134328	0.003	0.008	7.08E-01	-0.008	0.012	4.88E-01
4.099222	-0.003	0.010	7.64E-01	0.003	0.015	8.66E-01
8.765542	0.003	0.007	7.08E-01	-0.003	0.011	8.12E-01
3.896353	0.002	0.005	6.63E-01	0.019	0.007	6.20E-03
2.033189626	0.003	0.004	4.72E-01	0.008	0.006	2.37E-01
3.330474	-0.005	0.010	6.27E-01	0.011	0.015	4.51E-01
2.651552728	-0.005	0.013	7.13E-01	0.021	0.019	2.68E-01
2.172809151	0.005	0.010	5.98E-01	-0.019	0.014	1.87E-01
1.228106435	0.003	0.008	7.01E-01	-0.007	0.012	5.70E-01
2.750800343	-0.005	0.008	5.12E-01	-0.016	0.012	1.80E-01
2.269365305	0.003	0.006	5.59E-01	0.015	0.009	8.84E-02

2.423451431	0.001	0.011	9.07E-01	0.008	0.016	6.17E-01
3.645038	0.003	0.004	4.70E-01	0.015	0.006	1.17E-02
5.421066	0.002	0.006	6.95E-01	-0.002	0.009	8.53E-01
3.789031	0.011	0.011	3.17E-01	0.027	0.016	1.04E-01
7.623017	-0.014	0.013	3.10E-01	0.009	0.020	6.50E-01
1.199846097	0.003	0.008	7.27E-01	0.008	0.011	4.96E-01
1.228442868	0.003	0.008	7.02E-01	-0.007	0.012	5.61E-01
6.380908	0.003	0.008	7.00E-01	-0.007	0.012	5.36E-01
3.784321	-0.006	0.010	5.51E-01	0.020	0.014	1.67E-01
6.903388	0.005	0.010	6.20E-01	0.019	0.014	1.88E-01
8.215475	-0.003	0.008	7.38E-01	-0.006	0.012	6.40E-01
8.425072	0.003	0.009	7.37E-01	-0.015	0.013	2.49E-01
3.183116	0.004	0.005	4.76E-01	0.015	0.008	5.44E-02
6.579067	-0.006	0.008	4.79E-01	-0.013	0.012	2.82E-01
0.771903841	0.003	0.005	4.97E-01	-0.003	0.007	6.64E-01
2.75180964	-0.006	0.008	4.59E-01	-0.015	0.011	1.94E-01
2.780742843	-0.006	0.012	5.99E-01	-0.046	0.018	1.08E-02
5.557657	0.004	0.007	5.20E-01	-0.001	0.010	9.40E-01
6.029672	0.009	0.009	3.30E-01	-0.002	0.014	8.96E-01
8.284444	0.003	0.008	7.31E-01	0.001	0.012	9.13E-01
3.248721	-0.004	0.006	5.34E-01	0.008	0.010	3.84E-01
2.574509665	0.006	0.010	5.64E-01	0.007	0.015	6.45E-01
4.012423	-0.005	0.012	7.06E-01	0.005	0.018	7.91E-01
2.386780279	-0.012	0.015	4.18E-01	-0.001	0.022	9.77E-01
4.119072	-0.003	0.011	7.80E-01	0.021	0.017	2.12E-01
0.522270859	0.003	0.008	7.28E-01	-0.003	0.012	7.72E-01
0.525971617	0.006	0.009	4.96E-01	-0.014	0.014	3.26E-01
1.089159775	0.004	0.007	6.17E-01	-0.005	0.011	6.20E-01
6.368124	0.001	0.008	9.19E-01	0.005	0.012	6.96E-01
8.578149	-0.006	0.009	5.03E-01	-0.010	0.013	4.30E-01
6.350293	-0.004	0.008	6.49E-01	-0.015	0.012	2.31E-01
6.425317	0.004	0.007	5.77E-01	0.001	0.010	9.39E-01
4.025543	-0.004	0.013	7.68E-01	0.005	0.020	8.09E-01
1.2287793	0.003	0.008	7.07E-01	-0.007	0.012	5.56E-01
7.631764	0.005	0.012	6.80E-01	0.000	0.019	9.81E-01
3.13467	-0.004	0.011	7.43E-01	-0.003	0.017	8.71E-01
2.173818449	0.006	0.010	5.27E-01	-0.014	0.015	3.46E-01
8.455351	0.003	0.012	7.74E-01	0.002	0.017	9.00E-01
5.437887	0.003	0.008	6.98E-01	0.007	0.012	5.26E-01
7.427213	0.003	0.009	7.26E-01	0.003	0.014	8.03E-01
3.553529	-0.011	0.019	5.44E-01	0.051	0.028	6.79E-02
8.786401	0.004	0.008	6.56E-01	-0.001	0.012	9.59E-01
0.85735772	0.005	0.008	5.77E-01	-0.007	0.012	5.61E-01

1.764716418	-0.004	0.010	7.11E-01	0.015	0.015	3.25E-01
2.846683631	-0.002	0.009	8.21E-01	-0.011	0.013	4.26E-01
6.181403	0.003	0.007	7.21E-01	0.012	0.011	2.84E-01
8.328853	-0.002	0.007	7.77E-01	-0.028	0.011	1.19E-02
6.171983	0.003	0.007	6.78E-01	-0.004	0.010	6.53E-01
8.761841	-0.004	0.009	6.51E-01	-0.004	0.013	7.49E-01
8.355767	-0.005	0.009	5.92E-01	-0.020	0.013	1.21E-01
6.590169	0.000	0.007	9.68E-01	-0.004	0.011	7.19E-01
8.270313	0.001	0.008	8.79E-01	-0.016	0.012	2.00E-01
2.91868	-0.004	0.008	6.51E-01	-0.003	0.013	8.36E-01
0.827078786	-0.006	0.008	4.40E-01	0.003	0.011	8.04E-01
6.818943	0.002	0.010	8.30E-01	0.010	0.014	4.96E-01
7.420148	0.002	0.007	7.82E-01	-0.009	0.011	3.88E-01
3.309951	0.006	0.011	5.84E-01	0.005	0.017	7.47E-01
3.323409	0.004	0.010	6.68E-01	0.020	0.015	1.81E-01
6.814233	0.007	0.010	4.95E-01	-0.008	0.015	5.60E-01
6.453241	-0.004	0.007	5.49E-01	-0.022	0.010	2.83E-02
8.844267	0.004	0.008	5.61E-01	0.001	0.011	8.95E-01
4.30411	-0.008	0.014	5.89E-01	0.013	0.021	5.41E-01
0.680394177	0.005	0.012	6.65E-01	-0.019	0.017	2.77E-01
8.12262	0.003	0.008	7.02E-01	-0.005	0.011	6.78E-01
1.748904086	-0.005	0.007	4.84E-01	0.010	0.011	3.64E-01
0.507131392	-0.003	0.010	7.46E-01	0.016	0.015	2.95E-01
1.680944703	-0.002	0.004	6.49E-01	0.002	0.006	7.20E-01
4.219665	-0.005	0.013	6.83E-01	0.013	0.020	5.21E-01
2.372313678	-0.003	0.007	6.44E-01	0.008	0.011	4.29E-01
8.890359	-0.002	0.007	7.47E-01	-0.005	0.011	6.44E-01
8.671678	-0.003	0.007	6.14E-01	-0.014	0.010	1.86E-01
5.674063	0.004	0.008	6.70E-01	-0.001	0.013	9.48E-01
4.19847	-0.004	0.012	7.66E-01	0.018	0.018	3.15E-01
7.403663	-0.006	0.010	5.65E-01	-0.006	0.015	6.92E-01
7.253951	-0.007	0.012	5.48E-01	-0.002	0.017	8.91E-01
0.616135552	-0.006	0.010	5.75E-01	0.010	0.015	5.12E-01
6.428681	0.003	0.008	6.57E-01	-0.010	0.012	3.78E-01
1.425592366	0.004	0.005	3.92E-01	0.008	0.007	2.53E-01
2.183911426	0.007	0.011	5.30E-01	-0.020	0.016	2.12E-01
7.881397	0.006	0.009	5.16E-01	0.027	0.014	4.55E-02
3.858	0.001	0.006	8.16E-01	0.013	0.008	1.24E-01
8.988933	-0.008	0.010	4.10E-01	-0.010	0.014	4.73E-01
5.610477	0.004	0.008	6.10E-01	-0.013	0.012	2.79E-01
3.232235	0.002	0.006	7.03E-01	0.019	0.009	3.34E-02
6.986487	-0.003	0.008	7.02E-01	-0.012	0.012	3.08E-01
2.55566944	-0.007	0.011	5.09E-01	-0.006	0.016	7.01E-01

3.986517	-0.005	0.007	4.83E-01	0.005	0.011	6.63E-01
6.736517	-0.007	0.007	3.68E-01	-0.015	0.011	1.68E-01
5.437551	0.004	0.008	6.16E-01	0.007	0.011	5.29E-01
7.088762	0.006	0.008	4.28E-01	-0.004	0.011	7.26E-01
0.535728162	-0.003	0.008	6.64E-01	0.008	0.011	4.60E-01
8.282425	0.002	0.010	8.20E-01	0.014	0.014	3.26E-01
6.332125	-0.002	0.007	7.93E-01	-0.013	0.011	2.37E-01
8.110508	-0.002	0.010	8.12E-01	-0.012	0.014	3.96E-01
0.931036457	0.000	0.005	9.92E-01	-0.012	0.007	8.45E-02
2.33496966	0.006	0.007	4.25E-01	0.006	0.011	5.87E-01
8.861089	-0.002	0.009	8.71E-01	-0.002	0.014	8.75E-01
6.371824	0.004	0.006	4.72E-01	0.000	0.009	9.91E-01
6.471745	0.005	0.009	5.75E-01	0.006	0.013	6.41E-01
2.997069	-0.003	0.004	5.28E-01	0.005	0.006	4.17E-01
8.285789	-0.002	0.008	8.16E-01	-0.013	0.012	2.88E-01
5.879623	0.003	0.008	6.86E-01	-0.003	0.012	7.69E-01
6.06668	0.001	0.006	8.16E-01	-0.005	0.009	5.60E-01
2.272729631	0.003	0.007	6.94E-01	-0.001	0.011	9.09E-01
5.658587	0.003	0.009	7.41E-01	-0.015	0.014	2.68E-01
6.592524	-0.005	0.007	5.45E-01	-0.011	0.011	3.06E-01
8.55359	-0.004	0.007	5.66E-01	-0.003	0.010	7.95E-01
7.491808	-0.005	0.008	5.70E-01	-0.011	0.012	3.62E-01
2.828179839	0.007	0.013	5.59E-01	-0.044	0.019	2.03E-02
6.03371	0.003	0.008	7.00E-01	0.021	0.012	8.35E-02
6.751993	-0.004	0.009	6.62E-01	-0.022	0.014	1.20E-01
2.650879863	-0.005	0.014	7.25E-01	0.024	0.021	2.38E-01
4.233459	0.005	0.013	6.90E-01	0.012	0.020	5.37E-01
7.498874	0.001	0.009	8.86E-01	-0.002	0.013	8.54E-01
7.166478	0.003	0.008	6.87E-01	0.000	0.012	9.79E-01
8.99869	0.001	0.008	9.01E-01	-0.009	0.012	4.52E-01
0.676693418	0.001	0.008	8.79E-01	-0.018	0.011	1.19E-01
7.899565	-0.003	0.009	7.04E-01	-0.001	0.013	9.61E-01
1.68834622	-0.002	0.004	6.67E-01	0.006	0.006	3.07E-01
3.338548	-0.002	0.008	7.76E-01	0.012	0.013	3.57E-01
3.193882	0.002	0.006	7.02E-01	0.014	0.009	1.36E-01
6.930639	-0.009	0.010	3.44E-01	-0.003	0.015	8.55E-01
0.665591143	0.002	0.009	8.27E-01	-0.024	0.014	8.05E-02
3.918558	-0.003	0.010	7.37E-01	0.023	0.015	1.23E-01
2.038236115	0.003	0.004	4.75E-01	0.010	0.007	1.44E-01
5.066466	0.003	0.008	6.78E-01	-0.001	0.012	9.45E-01
5.570778	0.004	0.007	5.75E-01	-0.005	0.011	6.22E-01
8.249791	-0.003	0.008	7.25E-01	-0.003	0.012	8.20E-01
8.083257	-0.004	0.009	6.70E-01	-0.008	0.014	5.93E-01

8.44324	0.005	0.010	5.76E-01	0.002	0.014	8.72E-01
8.89877	-0.002	0.008	7.60E-01	0.007	0.012	5.89E-01
8.445931	-0.006	0.009	5.23E-01	-0.004	0.014	7.90E-01
1.713578664	-0.002	0.005	7.06E-01	0.008	0.007	2.22E-01
1.443086861	0.003	0.004	5.08E-01	0.008	0.006	1.93E-01
2.779733545	-0.007	0.012	5.57E-01	-0.045	0.018	1.08E-02
6.090567	0.004	0.009	6.20E-01	-0.006	0.013	6.36E-01
8.508508	0.004	0.009	6.39E-01	0.006	0.014	6.84E-01
6.188469	-0.007	0.009	4.31E-01	-0.016	0.013	2.19E-01
6.247344	-0.005	0.008	5.50E-01	-0.005	0.012	7.14E-01
5.726883	0.003	0.011	7.51E-01	-0.012	0.016	4.61E-01
5.004899	0.003	0.009	7.50E-01	-0.003	0.014	8.39E-01
8.27065	0.001	0.009	9.09E-01	0.004	0.014	7.90E-01
1.711560069	-0.002	0.004	6.36E-01	0.006	0.006	3.41E-01
6.936695	-0.004	0.009	6.90E-01	0.010	0.014	4.78E-01
8.691191	0.002	0.006	7.52E-01	0.001	0.010	8.97E-01
8.090995	0.002	0.007	7.99E-01	0.005	0.011	6.51E-01
5.559676	0.005	0.009	5.62E-01	0.003	0.014	8.29E-01
2.273066063	0.003	0.008	6.69E-01	-0.003	0.011	7.93E-01
4.022852	0.008	0.013	5.30E-01	0.010	0.019	6.04E-01
8.765206	-0.005	0.009	5.86E-01	0.005	0.014	7.15E-01
1.668160264	-0.004	0.004	3.99E-01	0.002	0.006	7.01E-01
7.87736	0.003	0.009	7.63E-01	0.001	0.014	9.60E-01
4.028908	-0.005	0.012	6.90E-01	-0.005	0.018	7.96E-01
7.836988	-0.005	0.009	5.94E-01	0.018	0.014	2.08E-01
8.268631	-0.002	0.007	8.05E-01	-0.015	0.011	1.79E-01
8.764869	-0.007	0.010	4.90E-01	0.029	0.015	5.37E-02
1.729390996	-0.003	0.005	5.48E-01	0.006	0.008	4.37E-01
2.834235625	0.008	0.011	4.90E-01	-0.031	0.017	6.63E-02
7.208196	-0.004	0.007	5.47E-01	-0.008	0.010	4.59E-01
5.14149	-0.006	0.009	5.30E-01	-0.020	0.013	1.34E-01
8.209419	0.005	0.009	5.99E-01	-0.002	0.014	9.06E-01
7.887453	-0.003	0.008	7.27E-01	0.023	0.013	6.61E-02
0.543802545	0.007	0.008	3.80E-01	0.021	0.012	8.31E-02
1.042059212	0.001	0.007	8.54E-01	0.022	0.011	4.38E-02
6.296127	-0.003	0.007	6.33E-01	0.004	0.010	7.16E-01
4.09754	-0.001	0.009	9.06E-01	0.003	0.013	7.93E-01
3.338885	-0.002	0.008	7.76E-01	0.012	0.013	3.22E-01
8.388401	0.008	0.010	4.33E-01	0.004	0.015	7.87E-01
1.444432591	0.003	0.004	4.81E-01	0.004	0.006	4.97E-01
0.505449229	-0.003	0.009	7.32E-01	-0.002	0.013	9.04E-01
4.377452	-0.007	0.016	6.63E-01	0.025	0.023	2.74E-01
5.415346	-0.003	0.009	7.70E-01	-0.022	0.013	9.43E-02

5.487343	0.002	0.007	7.58E-01	-0.004	0.011	6.99E-01
6.164582	-0.003	0.009	7.71E-01	-0.012	0.014	3.77E-01
3.135006	-0.005	0.011	6.65E-01	-0.004	0.017	8.02E-01
8.526675	-0.003	0.009	7.71E-01	-0.020	0.014	1.47E-01
7.135526	0.005	0.009	5.57E-01	-0.013	0.013	3.08E-01
0.755755077	0.003	0.005	5.71E-01	0.000	0.008	9.68E-01
5.024748	0.004	0.009	6.45E-01	-0.013	0.013	3.12E-01
8.740983	-0.005	0.008	5.03E-01	-0.029	0.012	1.37E-02
8.656538	0.004	0.009	6.06E-01	-0.008	0.013	5.21E-01
3.323072	0.005	0.011	6.68E-01	0.015	0.016	3.39E-01
6.778235	-0.002	0.010	8.49E-01	-0.006	0.015	7.15E-01
3.787349	0.008	0.009	3.82E-01	0.020	0.014	1.45E-01
4.297381	-0.005	0.013	6.95E-01	0.011	0.019	5.82E-01
6.615065	0.002	0.008	7.61E-01	0.006	0.012	5.93E-01
3.324418	0.004	0.010	7.22E-01	0.024	0.015	1.09E-01
3.373874	0.004	0.012	7.29E-01	-0.015	0.018	4.19E-01
7.454128	0.005	0.009	5.60E-01	0.012	0.014	4.05E-01
7.856501	-0.002	0.006	7.30E-01	-0.005	0.009	5.92E-01
0.636994372	-0.004	0.009	6.05E-01	-0.011	0.013	3.74E-01
0.528326645	0.002	0.008	8.15E-01	-0.015	0.012	2.05E-01
6.254746	0.003	0.009	7.66E-01	0.005	0.014	7.38E-01
6.835765	-0.005	0.010	6.43E-01	-0.016	0.016	2.94E-01
8.443576	0.008	0.010	4.43E-01	0.011	0.015	4.72E-01
8.526339	0.004	0.009	6.69E-01	-0.012	0.014	3.68E-01
8.477219	-0.003	0.009	7.81E-01	-0.023	0.014	8.70E-02
8.524993	0.002	0.007	7.81E-01	0.004	0.010	7.07E-01
2.54927722	-0.007	0.010	5.03E-01	-0.002	0.015	9.19E-01
0.99765011	0.003	0.006	5.86E-01	0.016	0.009	9.02E-02
5.947919	0.006	0.010	5.65E-01	0.011	0.016	4.71E-01
6.00915	0.003	0.009	7.24E-01	-0.019	0.014	1.60E-01
7.51805	-0.002	0.008	7.96E-01	-0.003	0.012	8.30E-01
5.562704	0.004	0.010	6.92E-01	-0.024	0.015	9.73E-02
5.103137	0.004	0.008	6.63E-01	0.003	0.012	8.13E-01
3.205657	0.003	0.009	7.07E-01	0.016	0.013	2.38E-01
6.038756	0.004	0.008	6.14E-01	0.014	0.012	2.19E-01
3.190854	-0.003	0.007	7.01E-01	0.015	0.010	1.20E-01
8.286126	-0.001	0.008	8.74E-01	-0.018	0.012	1.53E-01
6.06197	-0.004	0.008	5.76E-01	0.002	0.012	8.68E-01
1.229115733	0.003	0.008	7.23E-01	-0.007	0.012	5.37E-01
4.189723	-0.003	0.013	8.16E-01	0.016	0.019	4.19E-01
4.36534	-0.006	0.014	6.75E-01	0.009	0.022	6.79E-01
7.440671	0.003	0.008	7.13E-01	-0.005	0.012	6.76E-01
1.815181307	-0.001	0.007	8.66E-01	-0.007	0.010	5.03E-01

8.128675	-0.004	0.008	5.83E-01	-0.005	0.012	6.67E-01
8.657547	0.001	0.008	8.69E-01	-0.009	0.012	4.76E-01
6.249699	0.006	0.010	5.28E-01	0.002	0.015	9.02E-01
8.187887	-0.007	0.008	4.29E-01	0.001	0.012	9.24E-01
6.806159	0.003	0.008	6.47E-01	-0.007	0.011	5.64E-01
2.808666748	-0.007	0.015	6.34E-01	-0.065	0.022	2.63E-03
8.335245	-0.004	0.009	6.89E-01	-0.008	0.014	5.55E-01
7.155376	0.003	0.007	6.65E-01	-0.001	0.010	9.56E-01
7.199785	-0.008	0.011	4.39E-01	-0.012	0.016	4.57E-01
5.81772	0.004	0.009	6.49E-01	-0.008	0.013	5.28E-01
6.305211	0.002	0.007	7.31E-01	-0.003	0.011	7.90E-01
7.4077	0.004	0.008	5.90E-01	-0.006	0.011	5.95E-01
8.116564	0.001	0.008	8.60E-01	-0.009	0.012	4.57E-01
4.365004	-0.006	0.015	6.95E-01	0.008	0.022	7.34E-01
5.912594	0.002	0.007	8.01E-01	-0.003	0.010	7.64E-01
8.147179	-0.003	0.007	6.69E-01	0.016	0.010	1.34E-01
7.993093	0.004	0.008	5.96E-01	-0.010	0.012	4.07E-01
6.547442	-0.004	0.010	6.76E-01	-0.003	0.015	8.62E-01
4.222693	0.004	0.012	7.42E-01	0.018	0.018	3.29E-01
2.586957671	0.006	0.010	5.32E-01	0.006	0.015	6.79E-01
3.916203	-0.005	0.009	5.80E-01	0.027	0.013	3.75E-02
8.926357	0.002	0.008	7.82E-01	-0.008	0.012	5.44E-01
4.114698	-0.001	0.009	8.94E-01	0.014	0.014	3.14E-01
1.462599951	-0.001	0.004	7.18E-01	0.001	0.006	8.52E-01
5.815028	0.004	0.009	6.32E-01	0.001	0.014	9.56E-01
6.930975	-0.009	0.010	3.47E-01	-0.005	0.014	7.36E-01
8.741319	0.003	0.008	7.23E-01	-0.013	0.013	2.87E-01
8.157609	-0.003	0.006	6.97E-01	0.000	0.010	9.70E-01
2.803283827	-0.006	0.014	6.69E-01	-0.067	0.021	1.19E-03
5.643111	0.003	0.007	6.53E-01	-0.008	0.010	4.13E-01
1.661431612	0.002	0.004	7.05E-01	0.001	0.006	8.24E-01
6.851241	0.002	0.008	8.28E-01	-0.006	0.012	6.34E-01
7.125097	-0.003	0.008	6.81E-01	-0.021	0.012	8.36E-02
5.589955	-0.003	0.008	7.29E-01	0.000	0.012	9.88E-01
1.461254221	-0.001	0.004	7.43E-01	-0.001	0.006	9.23E-01
5.711071	0.005	0.010	5.93E-01	-0.012	0.014	3.93E-01
6.196207	-0.001	0.008	8.66E-01	-0.015	0.012	2.41E-01
5.11323	0.003	0.007	7.08E-01	0.000	0.011	9.94E-01
8.723825	0.003	0.008	7.34E-01	-0.002	0.012	8.55E-01
5.058391	0.002	0.009	8.44E-01	-0.011	0.013	4.10E-01
5.427458	0.004	0.007	5.42E-01	-0.008	0.011	4.85E-01
7.848091	0.001	0.011	8.90E-01	0.004	0.016	7.93E-01
3.926632	-0.002	0.009	8.65E-01	0.015	0.013	2.46E-01

7.420485	0.002	0.007	7.69E-01	-0.006	0.010	5.69E-01
8.220185	0.002	0.005	6.96E-01	0.005	0.008	5.03E-01
3.641337	-0.001	0.004	7.51E-01	0.008	0.005	1.47E-01
1.739147541	-0.001	0.006	8.87E-01	0.012	0.009	1.89E-01
1.001014436	0.003	0.007	6.82E-01	0.002	0.011	8.64E-01
8.033801	-0.002	0.008	8.10E-01	-0.009	0.013	4.58E-01
6.034719	0.003	0.007	6.67E-01	-0.008	0.011	4.58E-01
4.220338	-0.006	0.012	6.00E-01	0.006	0.018	7.49E-01
3.992573	-0.005	0.010	6.28E-01	0.003	0.015	8.65E-01
6.771506	-0.002	0.009	8.46E-01	-0.018	0.014	1.94E-01
0.947185221	-0.002	0.005	6.56E-01	-0.005	0.008	4.93E-01
5.693576	0.003	0.008	7.18E-01	-0.003	0.012	7.83E-01
8.04524	0.003	0.009	7.50E-01	-0.007	0.013	5.98E-01
5.88938	0.003	0.008	7.13E-01	0.007	0.011	5.29E-01
7.492818	-0.004	0.008	6.07E-01	-0.003	0.012	8.09E-01
0.942811598	0.004	0.006	4.40E-01	-0.012	0.008	1.45E-01
4.009395	0.004	0.012	7.13E-01	0.010	0.018	5.70E-01
5.774993	0.003	0.007	7.14E-01	-0.003	0.011	7.56E-01
3.165285	-0.003	0.007	6.58E-01	0.006	0.010	5.52E-01
8.712049	-0.003	0.009	7.17E-01	0.004	0.013	7.79E-01
5.474559	-0.003	0.009	7.25E-01	-0.018	0.013	1.60E-01
1.085795449	0.004	0.008	6.06E-01	-0.006	0.011	6.08E-01
7.808728	0.003	0.008	6.76E-01	-0.021	0.012	7.69E-02
8.75444	-0.002	0.008	7.99E-01	-0.001	0.013	9.29E-01
8.058697	0.004	0.008	6.28E-01	-0.012	0.012	3.40E-01
7.550348	0.004	0.008	6.10E-01	-0.003	0.012	8.28E-01
6.869408	0.006	0.010	5.44E-01	-0.001	0.015	9.43E-01
7.234774	0.002	0.011	8.61E-01	0.023	0.016	1.38E-01
3.57506	-0.002	0.006	7.76E-01	0.010	0.009	2.71E-01
5.655559	-0.005	0.006	4.04E-01	-0.006	0.009	5.07E-01
5.909566	-0.005	0.009	5.75E-01	-0.014	0.014	2.98E-01
6.811542	-0.006	0.010	5.26E-01	-0.007	0.014	6.34E-01
5.151247	0.003	0.007	6.77E-01	-0.019	0.010	4.97E-02
7.278174	-0.007	0.009	4.30E-01	-0.007	0.014	6.31E-01
0.820350135	0.001	0.006	9.01E-01	0.012	0.009	1.77E-01
8.559309	-0.004	0.007	6.00E-01	-0.009	0.011	4.18E-01
6.711285	-0.002	0.009	8.41E-01	0.002	0.013	9.09E-01
6.544751	0.004	0.008	6.12E-01	-0.011	0.012	3.36E-01
7.608214	-0.004	0.011	7.09E-01	-0.004	0.016	7.89E-01
2.52068045	-0.009	0.015	5.74E-01	-0.019	0.023	4.13E-01
7.637147	-0.001	0.011	9.10E-01	0.009	0.017	6.10E-01
2.802947394	-0.007	0.014	6.33E-01	-0.068	0.020	9.51E-04
2.421769268	0.008	0.012	5.12E-01	-0.007	0.019	6.94E-01

6.855614	-0.004	0.008	6.05E-01	0.014	0.012	2.20E-01
3.693821	-0.002	0.005	7.36E-01	0.015	0.007	4.25E-02
8.072155	0.003	0.008	7.31E-01	0.006	0.011	6.01E-01
3.189845	-0.003	0.006	6.65E-01	0.012	0.009	2.02E-01
0.94314803	0.004	0.005	4.33E-01	-0.009	0.008	2.27E-01
5.426785	0.003	0.006	5.99E-01	-0.009	0.009	3.25E-01
1.77413653	-0.002	0.011	8.26E-01	0.001	0.016	9.26E-01
6.349283	-0.003	0.008	7.24E-01	-0.007	0.012	5.91E-01
3.308942	-0.007	0.013	5.98E-01	-0.003	0.019	8.88E-01
2.171799853	0.005	0.009	5.52E-01	-0.009	0.014	5.32E-01
2.172136286	0.006	0.009	5.39E-01	-0.021	0.014	1.33E-01
6.077446	-0.005	0.009	5.56E-01	-0.018	0.014	1.82E-01
0.564661365	-0.007	0.009	4.77E-01	0.004	0.014	7.81E-01
7.280192	-0.008	0.010	3.90E-01	-0.017	0.014	2.35E-01
6.362068	-0.005	0.008	5.45E-01	-0.010	0.011	3.69E-01
1.230797896	0.003	0.008	7.30E-01	-0.008	0.012	4.80E-01
8.504807	-0.002	0.009	8.02E-01	-0.006	0.014	6.40E-01
8.087631	-0.002	0.007	7.19E-01	0.004	0.010	6.74E-01
5.568087	0.006	0.008	4.61E-01	0.000	0.012	9.96E-01
7.466912	-0.004	0.009	6.29E-01	-0.023	0.014	9.39E-02
7.899901	0.005	0.009	5.84E-01	0.004	0.013	7.44E-01
3.513493	0.003	0.008	7.16E-01	0.023	0.012	5.48E-02
6.558881	-0.003	0.008	6.72E-01	0.002	0.011	8.92E-01
6.655101	0.001	0.008	8.72E-01	0.022	0.012	6.75E-02
8.244745	0.004	0.010	6.55E-01	0.018	0.014	2.26E-01
8.03111	-0.006	0.007	3.65E-01	-0.009	0.010	3.67E-01
7.441344	0.005	0.010	6.09E-01	0.001	0.014	9.72E-01
1.740829704	-0.004	0.007	5.57E-01	0.018	0.010	6.75E-02
8.445595	-0.002	0.009	8.01E-01	-0.002	0.014	9.07E-01
8.760496	0.002	0.009	8.24E-01	-0.004	0.013	7.71E-01
2.291569856	-0.001	0.004	7.68E-01	-0.012	0.007	6.12E-02
7.989729	-0.004	0.010	6.70E-01	-0.016	0.015	2.78E-01
8.973121	0.005	0.009	5.85E-01	0.010	0.014	4.63E-01
6.839466	-0.006	0.009	5.38E-01	0.033	0.014	1.68E-02
7.209878	-0.004	0.007	5.27E-01	0.000	0.010	9.70E-01
4.36063	-0.005	0.012	6.69E-01	0.003	0.018	8.75E-01
8.883966	0.006	0.009	5.46E-01	-0.006	0.014	6.71E-01
8.884976	0.003	0.007	6.25E-01	0.002	0.011	8.86E-01
6.800439	0.004	0.007	6.00E-01	0.010	0.010	3.25E-01
4.133875	-0.007	0.015	6.34E-01	0.014	0.022	5.31E-01
8.139778	-0.005	0.009	5.35E-01	-0.006	0.013	6.09E-01
5.694586	0.003	0.009	7.67E-01	-0.015	0.013	2.53E-01
3.923941	0.004	0.009	6.77E-01	0.028	0.014	3.73E-02

1.441404698	0.003	0.004	4.68E-01	0.007	0.006	2.79E-01
6.402103	-0.001	0.010	8.87E-01	-0.018	0.015	2.21E-01
8.312704	0.005	0.009	6.19E-01	0.004	0.014	7.56E-01
3.332156	0.003	0.010	7.60E-01	0.007	0.015	6.36E-01
1.926876927	0.001	0.004	7.40E-01	0.005	0.006	4.50E-01
0.73792415	-0.004	0.007	5.80E-01	-0.003	0.010	7.72E-01
4.193087	-0.005	0.010	6.43E-01	0.009	0.015	5.43E-01
8.282089	0.003	0.008	7.13E-01	0.005	0.013	6.76E-01
0.614453389	-0.006	0.008	5.11E-01	-0.005	0.013	7.13E-01
5.041906	0.003	0.009	7.32E-01	-0.001	0.013	9.25E-01
8.472509	0.005	0.008	5.87E-01	-0.015	0.013	2.24E-01
2.847356496	-0.001	0.009	9.00E-01	-0.011	0.013	4.06E-01
7.557076	-0.003	0.008	7.03E-01	-0.005	0.012	6.81E-01
0.660208221	-0.008	0.010	4.23E-01	-0.012	0.015	4.30E-01
6.910116	0.006	0.009	4.99E-01	0.004	0.013	7.43E-01
5.543191	-0.005	0.011	6.67E-01	-0.042	0.016	1.10E-02
5.619225	-0.003	0.008	7.45E-01	-0.013	0.012	2.68E-01
6.314294	-0.003	0.007	6.28E-01	-0.021	0.010	3.72E-02
6.702538	-0.004	0.008	6.40E-01	-0.027	0.012	2.74E-02
0.524625887	0.005	0.010	6.15E-01	0.008	0.015	5.91E-01
2.365585026	0.012	0.014	3.64E-01	0.040	0.020	5.01E-02
6.415224	0.002	0.008	7.48E-01	0.000	0.011	9.84E-01
0.502084903	0.003	0.008	7.21E-01	-0.011	0.012	3.90E-01
0.744316369	-0.004	0.006	5.50E-01	-0.006	0.009	5.12E-01
8.928376	-0.005	0.008	4.84E-01	0.008	0.012	4.76E-01
0.977127722	0.001	0.007	8.68E-01	0.011	0.010	3.04E-01
2.75046391	-0.005	0.008	5.21E-01	-0.017	0.012	1.54E-01
6.610691	-0.003	0.009	7.39E-01	-0.012	0.014	3.73E-01
4.042701	0.009	0.014	5.04E-01	-0.015	0.021	4.68E-01
8.024045	-0.004	0.008	6.18E-01	-0.023	0.012	5.75E-02
8.004195	-0.001	0.009	9.31E-01	-0.008	0.014	5.72E-01
3.179415	-0.002	0.005	7.37E-01	0.013	0.008	8.70E-02
6.134976	0.005	0.009	5.99E-01	-0.020	0.013	1.26E-01
6.325733	0.005	0.007	4.81E-01	-0.006	0.011	6.03E-01
7.291631	-0.005	0.010	6.47E-01	-0.006	0.015	6.87E-01
0.930700024	0.000	0.005	9.97E-01	-0.015	0.007	3.74E-02
8.220521	0.004	0.006	5.49E-01	0.005	0.009	5.40E-01
3.180088	-0.002	0.005	7.68E-01	0.010	0.008	2.01E-01
8.736273	-0.006	0.009	5.41E-01	0.011	0.014	4.37E-01
2.530436995	-0.005	0.013	7.09E-01	0.002	0.020	9.24E-01
7.934554	-0.002	0.009	7.77E-01	0.012	0.013	3.74E-01
6.509089	-0.002	0.008	7.59E-01	0.003	0.012	7.96E-01
6.276277	0.005	0.008	4.89E-01	-0.009	0.011	4.05E-01

6.439784	0.004	0.009	6.58E-01	0.001	0.013	9.67E-01
7.003308	-0.003	0.008	7.21E-01	-0.008	0.012	5.24E-01
3.761444	0.004	0.008	6.21E-01	0.025	0.012	3.93E-02
4.364667	-0.006	0.014	6.66E-01	0.007	0.021	7.23E-01
7.684921	-0.002	0.007	7.95E-01	-0.004	0.010	6.99E-01
8.814998	-0.004	0.008	5.83E-01	-0.014	0.011	2.15E-01
1.229452165	0.003	0.008	7.34E-01	-0.007	0.012	5.28E-01
0.63800367	0.004	0.009	6.87E-01	0.025	0.013	6.01E-02
7.992084	0.004	0.008	5.62E-01	-0.021	0.011	5.92E-02
1.002023734	0.004	0.008	6.18E-01	-0.005	0.012	6.60E-01
6.823653	-0.004	0.009	6.93E-01	0.014	0.014	3.15E-01
5.827476	0.005	0.009	5.78E-01	0.005	0.014	7.29E-01
6.879838	0.007	0.010	4.85E-01	0.014	0.016	3.55E-01
2.088364571	-0.002	0.006	6.98E-01	0.016	0.009	7.95E-02
0.674674823	0.005	0.009	5.40E-01	-0.011	0.013	4.12E-01
3.879868	0.002	0.005	7.05E-01	0.019	0.007	8.64E-03
8.887667	-0.006	0.009	5.15E-01	-0.013	0.013	3.37E-01
7.814784	-0.005	0.009	5.67E-01	0.011	0.014	4.16E-01
6.312949	0.004	0.007	5.89E-01	0.001	0.010	9.27E-01
5.676755	0.004	0.010	6.99E-01	0.000	0.015	9.79E-01
3.181434	0.004	0.005	4.82E-01	0.013	0.008	9.42E-02
1.670515292	-0.002	0.004	5.69E-01	0.004	0.006	4.68E-01
1.896934426	-0.003	0.006	5.41E-01	0.000	0.008	9.99E-01
8.238016	0.000	0.008	9.82E-01	-0.006	0.011	6.09E-01
5.899137	0.005	0.010	5.84E-01	-0.010	0.014	4.85E-01
2.092738194	-0.003	0.006	6.31E-01	0.004	0.008	6.10E-01
4.201498	-0.003	0.012	8.17E-01	0.020	0.018	2.69E-01
0.959633227	0.003	0.005	6.19E-01	0.011	0.008	1.37E-01
6.820962	-0.009	0.010	4.08E-01	0.007	0.016	6.37E-01
6.643325	0.002	0.009	8.27E-01	-0.009	0.014	5.23E-01
8.566374	-0.008	0.008	3.41E-01	-0.008	0.012	5.14E-01
3.932352	-0.002	0.007	7.32E-01	0.012	0.010	2.58E-01
7.154703	-0.003	0.007	6.80E-01	-0.021	0.010	3.96E-02
6.875127	0.004	0.011	7.12E-01	0.002	0.016	8.83E-01
5.8988	0.003	0.007	6.90E-01	-0.006	0.011	6.01E-01
6.910453	0.007	0.009	4.28E-01	-0.001	0.014	9.52E-01
2.803620259	-0.007	0.014	6.39E-01	-0.064	0.021	2.15E-03
4.106287	-0.004	0.014	7.59E-01	0.020	0.021	3.28E-01
3.052917	-0.003	0.008	7.32E-01	0.012	0.012	3.54E-01
7.905957	-0.002	0.008	8.41E-01	0.005	0.011	6.65E-01
6.213028	0.006	0.009	5.51E-01	-0.012	0.014	3.78E-01
1.817199902	0.006	0.010	5.25E-01	-0.002	0.014	8.83E-01
0.826742354	-0.005	0.007	4.60E-01	0.003	0.011	7.77E-01

5.441252	0.004	0.009	6.92E-01	-0.001	0.014	9.28E-01
0.785024713	-0.003	0.004	4.46E-01	-0.002	0.006	7.06E-01
6.892622	0.006	0.009	4.86E-01	0.000	0.014	9.75E-01
4.096194	-0.002	0.009	8.62E-01	0.009	0.014	5.37E-01
1.702476389	-0.002	0.004	6.62E-01	0.007	0.007	2.98E-01
3.553192	-0.010	0.017	5.84E-01	0.057	0.026	2.72E-02
2.832553462	0.007	0.012	5.27E-01	-0.014	0.018	4.17E-01
6.635924	0.004	0.007	6.05E-01	-0.007	0.011	5.36E-01
6.46468	0.007	0.009	4.63E-01	-0.012	0.014	3.79E-01
8.475537	-0.004	0.010	7.22E-01	-0.009	0.015	5.51E-01
3.899718	0.002	0.005	7.18E-01	0.020	0.008	7.28E-03
8.178467	0.003	0.009	7.79E-01	0.007	0.013	6.27E-01
3.134333	-0.003	0.010	7.42E-01	-0.004	0.016	7.77E-01
4.011077	0.006	0.013	6.44E-01	0.009	0.019	6.29E-01
6.015206	0.003	0.008	7.01E-01	0.019	0.012	1.22E-01
6.046158	0.003	0.009	7.15E-01	0.004	0.013	7.35E-01
7.815457	-0.007	0.010	5.33E-01	-0.004	0.016	7.99E-01
7.249577	-0.005	0.008	5.65E-01	-0.010	0.012	3.92E-01
2.739361634	0.003	0.011	7.59E-01	-0.019	0.016	2.55E-01
5.571115	0.005	0.008	5.64E-01	0.004	0.012	7.34E-01
8.055333	0.001	0.009	9.46E-01	0.002	0.013	8.74E-01
8.027073	0.006	0.010	5.65E-01	-0.010	0.015	4.73E-01
7.68593	0.004	0.007	5.87E-01	0.002	0.010	8.50E-01
6.838456	-0.005	0.009	5.49E-01	-0.001	0.013	9.62E-01
8.680425	0.004	0.009	6.41E-01	0.000	0.013	9.72E-01
7.274137	-0.008	0.010	4.19E-01	-0.003	0.015	8.57E-01
7.296005	-0.004	0.010	6.92E-01	0.000	0.015	9.99E-01
4.363995	-0.007	0.014	6.23E-01	0.015	0.021	4.60E-01
7.846745	0.002	0.010	8.08E-01	0.004	0.015	8.15E-01
2.41941424	-0.002	0.017	8.81E-01	-0.010	0.025	6.76E-01
1.667150966	-0.003	0.004	4.92E-01	0.008	0.006	1.64E-01
2.265664546	0.004	0.007	6.28E-01	0.020	0.011	6.59E-02
0.670301199	-0.005	0.008	5.21E-01	0.001	0.012	9.23E-01
6.599589	-0.004	0.009	6.18E-01	-0.010	0.013	4.42E-01
7.457156	-0.003	0.009	7.29E-01	-0.014	0.013	2.61E-01
8.99398	-0.005	0.008	5.46E-01	-0.013	0.012	2.76E-01
2.791508686	-0.006	0.012	6.39E-01	-0.060	0.019	1.27E-03
6.860661	0.004	0.009	6.85E-01	0.004	0.014	7.45E-01
7.42654	-0.008	0.010	4.44E-01	0.013	0.015	3.73E-01
0.779978224	0.002	0.004	6.98E-01	0.003	0.007	5.93E-01
6.369806	0.002	0.009	7.93E-01	-0.010	0.014	4.43E-01
1.498598238	-0.003	0.005	5.20E-01	-0.012	0.007	8.56E-02
8.710367	0.002	0.009	8.10E-01	0.005	0.014	7.13E-01

2.091392464	-0.002	0.005	7.51E-01	0.014	0.008	8.85E-02
6.981777	-0.003	0.010	7.90E-01	-0.021	0.015	1.70E-01
5.062765	0.004	0.007	5.75E-01	-0.001	0.011	9.58E-01
6.693117	0.002	0.008	7.94E-01	0.005	0.012	6.88E-01
8.78741	-0.001	0.009	9.37E-01	0.001	0.013	9.31E-01
2.28181331	-0.001	0.005	8.81E-01	-0.006	0.007	3.80E-01
8.944524	0.005	0.007	4.89E-01	-0.004	0.010	7.10E-01
8.147852	0.000	0.009	9.63E-01	0.018	0.013	1.70E-01
3.947491	-0.003	0.007	6.70E-01	0.004	0.011	7.25E-01
6.986823	-0.002	0.008	7.73E-01	0.001	0.012	9.13E-01
7.907639	0.004	0.009	6.32E-01	-0.002	0.013	9.01E-01
1.687673355	-0.002	0.004	5.96E-01	0.004	0.006	4.85E-01
8.357786	0.002	0.007	7.23E-01	0.006	0.010	5.37E-01
5.123659	0.002	0.006	7.42E-01	-0.009	0.010	3.43E-01
4.340108	-0.004	0.013	7.24E-01	0.013	0.019	4.86E-01
6.157853	0.007	0.009	4.55E-01	0.016	0.014	2.43E-01
0.548849034	0.003	0.010	7.17E-01	-0.017	0.014	2.43E-01
7.604177	0.009	0.010	4.00E-01	0.002	0.015	8.76E-01
3.797779	0.005	0.006	4.27E-01	0.013	0.009	1.44E-01
0.99899584	-0.002	0.006	7.40E-01	0.008	0.009	4.01E-01
2.174154881	0.005	0.010	6.05E-01	-0.013	0.015	3.97E-01
4.017469	-0.007	0.013	5.96E-01	0.012	0.020	5.52E-01
7.120387	-0.007	0.011	4.95E-01	-0.019	0.016	2.52E-01
6.500342	0.007	0.009	4.30E-01	-0.003	0.013	8.36E-01
6.232878	-0.005	0.008	5.61E-01	-0.009	0.012	4.47E-01
6.286034	-0.001	0.008	8.86E-01	0.012	0.012	3.54E-01
5.790805	0.002	0.008	8.26E-01	-0.010	0.011	3.75E-01
7.634119	-0.003	0.011	7.58E-01	0.002	0.016	9.12E-01
7.005327	-0.003	0.010	7.56E-01	-0.019	0.015	1.84E-01
4.093166	0.010	0.013	4.74E-01	0.014	0.020	4.94E-01
8.064417	0.004	0.008	6.07E-01	0.002	0.011	8.68E-01
2.852402985	-0.004	0.008	6.25E-01	-0.012	0.012	3.28E-01
6.788328	-0.001	0.010	8.84E-01	0.020	0.015	1.77E-01
7.021139	0.000	0.010	9.84E-01	-0.012	0.015	4.21E-01
5.296249	0.006	0.012	6.35E-01	-0.025	0.018	1.71E-01
4.043038	0.009	0.014	5.22E-01	-0.011	0.020	5.76E-01
8.394793	0.004	0.008	5.98E-01	-0.001	0.011	9.43E-01
6.84754	-0.004	0.009	6.58E-01	0.005	0.013	7.28E-01
4.18905	-0.005	0.012	7.08E-01	0.014	0.018	4.49E-01
7.453455	-0.001	0.008	8.57E-01	-0.001	0.012	9.05E-01
6.487894	-0.003	0.008	6.85E-01	0.008	0.012	4.83E-01
4.221347	-0.006	0.014	6.53E-01	0.007	0.020	7.12E-01
7.531171	-0.001	0.010	9.01E-01	0.006	0.014	6.99E-01

3.788358	0.012	0.012	3.05E-01	0.029	0.018	9.52E-02
2.184584292	0.007	0.011	5.43E-01	-0.026	0.016	1.09E-01
1.671861023	0.002	0.004	6.90E-01	0.003	0.006	5.78E-01
5.919995	-0.007	0.010	4.76E-01	0.007	0.014	6.10E-01
5.416019	-0.005	0.010	6.24E-01	0.002	0.015	9.01E-01
4.023188	0.008	0.013	5.48E-01	0.014	0.019	4.83E-01
6.149106	0.008	0.009	3.90E-01	0.006	0.014	6.82E-01
5.759853	0.004	0.009	6.89E-01	-0.009	0.014	4.98E-01
8.370234	0.003	0.008	7.11E-01	0.003	0.011	7.81E-01
4.08812	0.008	0.010	4.48E-01	0.007	0.015	6.59E-01
6.209664	-0.006	0.010	5.66E-01	0.008	0.014	5.60E-01
5.891399	0.004	0.009	6.28E-01	0.000	0.014	9.84E-01
7.983673	-0.002	0.009	7.96E-01	0.008	0.014	5.63E-01
8.507498	-0.004	0.010	6.91E-01	0.025	0.015	8.96E-02
0.788052606	0.000	0.004	9.77E-01	0.001	0.007	8.44E-01
8.506826	-0.003	0.007	6.52E-01	-0.003	0.011	7.65E-01
5.084633	0.005	0.010	6.07E-01	-0.010	0.015	4.94E-01
8.173421	-0.001	0.009	8.87E-01	-0.012	0.014	4.03E-01
5.534107	0.004	0.008	6.01E-01	-0.001	0.012	9.66E-01
1.666478101	0.001	0.004	8.13E-01	0.012	0.006	3.88E-02
8.937459	-0.002	0.007	7.49E-01	0.009	0.011	4.25E-01
7.836652	-0.003	0.007	6.77E-01	0.016	0.011	1.48E-01
8.49034	0.004	0.007	5.07E-01	-0.005	0.010	6.18E-01
6.35904	0.005	0.009	5.44E-01	0.009	0.013	5.02E-01
4.123109	-0.002	0.009	8.22E-01	0.001	0.014	9.40E-01
0.942138733	-0.001	0.005	8.93E-01	-0.017	0.008	3.44E-02
2.572827502	-0.001	0.010	8.93E-01	-0.003	0.015	8.56E-01
2.825151945	-0.005	0.013	7.07E-01	-0.043	0.020	2.87E-02
6.634578	0.007	0.009	4.37E-01	-0.015	0.014	2.95E-01
8.195289	-0.005	0.008	5.11E-01	-0.014	0.011	2.11E-01
1.889532909	0.004	0.007	5.86E-01	0.003	0.010	7.74E-01
6.420943	0.002	0.009	8.48E-01	-0.013	0.013	3.51E-01
8.161309	0.003	0.008	7.25E-01	0.013	0.012	2.86E-01
8.008905	-0.004	0.008	6.37E-01	0.008	0.012	4.94E-01
3.248048	0.002	0.007	7.37E-01	0.017	0.010	9.75E-02
7.44639	-0.001	0.008	8.95E-01	0.007	0.012	5.72E-01
6.748965	-0.002	0.012	8.58E-01	0.021	0.018	2.36E-01
8.086958	-0.003	0.008	7.31E-01	0.022	0.012	6.63E-02
3.209694	-0.008	0.013	5.60E-01	0.005	0.020	7.88E-01
2.751473208	-0.005	0.008	4.84E-01	-0.015	0.011	1.79E-01
8.65351	-0.001	0.009	9.20E-01	-0.006	0.013	6.33E-01
6.634915	0.005	0.009	5.55E-01	-0.014	0.013	2.94E-01
7.66137	0.006	0.009	4.87E-01	-0.004	0.014	7.71E-01

3.806526	0.002	0.006	7.58E-01	0.014	0.009	9.09E-02
7.492145	-0.003	0.008	6.97E-01	0.000	0.011	9.91E-01
4.35693	-0.005	0.013	7.20E-01	0.021	0.019	2.64E-01
3.3261	0.004	0.009	6.15E-01	0.020	0.013	1.11E-01
4.199816	-0.002	0.012	8.40E-01	0.023	0.018	2.00E-01
0.619163445	-0.002	0.008	8.03E-01	0.013	0.012	2.75E-01
0.794108392	-0.004	0.005	4.04E-01	0.001	0.008	8.65E-01
1.229788598	0.003	0.008	7.44E-01	-0.008	0.012	5.03E-01
8.816007	0.003	0.009	7.36E-01	-0.002	0.014	8.75E-01
3.896017	0.002	0.005	6.80E-01	0.018	0.007	8.79E-03
7.081024	0.002	0.007	8.24E-01	-0.011	0.011	3.28E-01
7.953394	0.003	0.009	7.58E-01	-0.012	0.014	4.02E-01
3.643356	-0.001	0.004	7.64E-01	0.009	0.006	1.05E-01
3.435441	0.006	0.015	7.16E-01	0.022	0.023	3.22E-01
6.451559	0.004	0.008	6.05E-01	-0.004	0.012	7.66E-01
0.608397602	0.006	0.010	5.38E-01	0.012	0.015	4.18E-01
6.503706	0.004	0.008	6.48E-01	-0.010	0.013	4.37E-01
6.533648	-0.002	0.007	7.42E-01	0.011	0.011	3.20E-01
6.967983	-0.007	0.009	4.60E-01	-0.001	0.014	9.61E-01
6.317659	-0.002	0.009	8.08E-01	-0.025	0.014	8.36E-02
6.945778	0.001	0.008	8.63E-01	0.015	0.012	2.21E-01
4.388554	-0.004	0.013	7.39E-01	0.026	0.019	1.71E-01
8.946879	0.002	0.008	8.39E-01	-0.006	0.012	6.22E-01
7.626045	-0.006	0.013	6.70E-01	0.004	0.020	8.20E-01
7.572552	0.005	0.009	5.56E-01	0.011	0.013	4.17E-01
4.007376	-0.006	0.011	5.78E-01	0.004	0.016	8.12E-01
2.571481771	0.004	0.010	6.79E-01	0.000	0.016	9.92E-01
5.750097	0.002	0.008	7.71E-01	-0.005	0.012	6.88E-01
6.87681	0.007	0.009	3.84E-01	0.018	0.013	1.53E-01
6.684707	0.003	0.010	7.55E-01	-0.031	0.015	3.57E-02
6.672259	0.005	0.009	5.81E-01	0.004	0.014	7.96E-01
7.558086	-0.005	0.009	5.90E-01	-0.005	0.013	6.96E-01
1.230125031	0.003	0.008	7.48E-01	-0.008	0.012	4.95E-01
8.010587	-0.005	0.007	4.87E-01	0.001	0.011	9.26E-01
1.668833129	0.000	0.004	9.88E-01	0.004	0.006	5.40E-01
3.805517	0.002	0.006	7.12E-01	0.010	0.008	2.32E-01
2.850047957	-0.004	0.008	5.99E-01	0.003	0.012	8.29E-01
8.303957	0.000	0.008	9.65E-01	-0.006	0.012	6.09E-01
5.696941	-0.004	0.007	6.23E-01	-0.023	0.011	3.51E-02
2.360202104	0.006	0.008	4.46E-01	0.015	0.012	2.17E-01
7.871641	-0.005	0.010	6.36E-01	-0.025	0.015	9.88E-02
8.060043	-0.001	0.008	8.61E-01	0.000	0.011	9.73E-01
3.516857	0.003	0.007	6.54E-01	0.016	0.011	1.44E-01

3.926296	-0.001	0.009	8.86E-01	0.017	0.013	2.05E-01
5.77903	0.004	0.009	6.77E-01	-0.005	0.013	6.93E-01
5.470185	0.005	0.009	5.69E-01	-0.022	0.014	1.04E-01
6.515817	0.004	0.010	6.84E-01	-0.016	0.015	2.73E-01
2.725231466	-0.006	0.009	5.22E-01	-0.007	0.013	5.79E-01
8.696237	0.004	0.009	6.35E-01	0.010	0.013	4.64E-01
1.230461463	0.003	0.008	7.50E-01	-0.008	0.012	4.87E-01
3.648066	0.003	0.004	5.23E-01	0.012	0.006	5.65E-02
6.570656	0.003	0.010	7.68E-01	-0.029	0.014	4.16E-02
8.801877	-0.003	0.008	7.27E-01	0.000	0.012	9.71E-01
4.228412	0.005	0.011	6.56E-01	0.020	0.017	2.36E-01
3.999975	0.003	0.010	7.53E-01	0.012	0.015	4.34E-01
5.859101	0.004	0.008	6.33E-01	-0.019	0.012	1.20E-01
8.799858	0.004	0.007	5.69E-01	0.006	0.011	5.66E-01
5.786432	0.001	0.009	9.04E-01	-0.013	0.013	3.07E-01
0.680730609	0.005	0.012	6.76E-01	-0.013	0.017	4.42E-01
6.576375	0.001	0.009	9.15E-01	0.008	0.014	5.43E-01
6.377207	-0.002	0.010	8.67E-01	0.016	0.014	2.52E-01
0.60806117	-0.001	0.009	8.82E-01	-0.004	0.014	7.76E-01
2.82582481	-0.005	0.013	6.99E-01	-0.043	0.019	2.47E-02
6.09124	0.003	0.007	6.94E-01	0.002	0.010	8.23E-01
2.584939075	0.010	0.013	4.18E-01	0.017	0.019	3.59E-01
4.239851	0.006	0.012	6.10E-01	-0.010	0.018	5.89E-01
4.238169	0.006	0.012	6.15E-01	-0.002	0.018	8.96E-01
8.358795	0.003	0.008	7.49E-01	0.003	0.013	7.88E-01
5.784749	-0.006	0.010	5.44E-01	-0.027	0.015	6.74E-02
7.00499	-0.003	0.010	7.64E-01	-0.021	0.015	1.58E-01
5.718472	0.001	0.010	9.51E-01	0.004	0.015	7.83E-01
6.482174	0.005	0.009	5.84E-01	-0.007	0.013	6.09E-01
6.10066	-0.004	0.008	6.22E-01	-0.009	0.012	4.57E-01
4.047075	-0.001	0.009	8.98E-01	-0.017	0.014	2.15E-01
8.525329	0.000	0.008	9.68E-01	0.008	0.011	4.79E-01
2.422105701	0.009	0.012	4.75E-01	-0.004	0.018	8.27E-01
7.315854	0.002	0.006	7.70E-01	0.004	0.010	6.54E-01
8.934768	0.007	0.011	5.32E-01	-0.002	0.016	8.81E-01
0.727494739	0.001	0.007	8.90E-01	-0.012	0.011	2.56E-01
0.679048446	0.005	0.011	6.68E-01	-0.021	0.017	2.16E-01
6.481838	0.002	0.009	7.87E-01	-0.006	0.014	6.49E-01
8.630296	-0.004	0.009	6.64E-01	0.005	0.013	7.15E-01
7.528143	-0.003	0.009	7.34E-01	-0.006	0.013	6.61E-01
4.008049	-0.007	0.010	4.91E-01	0.009	0.015	5.31E-01
7.789888	-0.005	0.009	5.65E-01	0.007	0.013	5.89E-01
7.386841	0.002	0.009	8.61E-01	-0.008	0.013	5.24E-01

7.963487	0.001	0.008	9.51E-01	-0.005	0.012	6.67E-01
0.743979936	-0.003	0.006	5.62E-01	-0.007	0.009	4.57E-01
4.303437	-0.006	0.014	6.62E-01	0.021	0.021	3.22E-01
3.554538	-0.010	0.022	6.63E-01	0.017	0.033	6.10E-01
4.364331	-0.005	0.014	6.94E-01	0.013	0.021	5.20E-01
4.218656	-0.003	0.011	7.68E-01	0.017	0.017	3.08E-01
8.212447	-0.006	0.008	4.29E-01	-0.002	0.011	8.58E-01
8.142805	0.006	0.010	5.56E-01	0.009	0.014	5.53E-01
1.038358454	0.002	0.007	7.91E-01	0.010	0.011	3.77E-01
0.511841448	-0.005	0.009	5.91E-01	0.000	0.014	9.85E-01
7.388524	0.004	0.008	6.61E-01	-0.001	0.012	9.24E-01
5.558667	0.002	0.007	7.54E-01	-0.004	0.010	6.78E-01
8.245754	-0.001	0.007	8.98E-01	0.003	0.011	7.70E-01
6.811878	-0.007	0.009	4.46E-01	0.004	0.013	7.80E-01
8.536432	-0.003	0.009	7.41E-01	0.006	0.013	6.34E-01
4.365677	-0.006	0.015	6.89E-01	0.007	0.022	7.54E-01
6.635251	-0.002	0.007	8.00E-01	-0.010	0.010	3.59E-01
2.739025202	0.003	0.011	7.68E-01	-0.019	0.016	2.35E-01
1.627451921	0.002	0.006	6.71E-01	-0.001	0.008	9.45E-01
0.820686567	0.001	0.006	9.23E-01	0.012	0.009	1.93E-01
6.995907	0.006	0.008	4.52E-01	-0.010	0.011	4.00E-01
7.317536	-0.005	0.006	3.85E-01	-0.007	0.009	4.31E-01
3.98719	-0.004	0.007	5.72E-01	0.012	0.011	2.68E-01
1.498934671	-0.003	0.005	5.48E-01	-0.013	0.007	6.65E-02
1.920148275	0.002	0.006	7.21E-01	-0.010	0.009	2.25E-01
7.002972	-0.003	0.008	6.79E-01	-0.005	0.012	6.72E-01
3.722754	0.003	0.008	7.38E-01	0.009	0.011	4.16E-01
1.775818693	-0.004	0.011	7.13E-01	-0.006	0.016	7.15E-01
6.755021	0.000	0.011	9.89E-01	0.013	0.016	4.33E-01
7.410392	-0.004	0.007	6.17E-01	-0.014	0.011	2.15E-01
5.882988	0.002	0.007	7.43E-01	0.004	0.010	7.02E-01
4.198133	-0.003	0.012	7.98E-01	0.016	0.018	3.86E-01
8.611793	0.003	0.010	7.68E-01	0.009	0.015	5.23E-01
3.640328	0.003	0.004	4.67E-01	0.011	0.006	9.30E-02
7.369683	-0.002	0.010	8.08E-01	0.007	0.014	6.22E-01
7.906293	0.004	0.007	6.11E-01	0.007	0.011	5.22E-01
3.805853	0.002	0.006	7.46E-01	0.011	0.008	2.09E-01
6.934003	-0.004	0.008	5.78E-01	-0.010	0.012	4.00E-01
6.795729	0.000	0.008	9.97E-01	0.005	0.012	6.91E-01
0.680057744	-0.007	0.012	5.69E-01	-0.030	0.017	8.54E-02
6.067689	0.003	0.010	7.79E-01	0.013	0.014	3.54E-01
8.450641	-0.006	0.010	5.42E-01	0.018	0.014	2.18E-01
7.949693	0.004	0.007	5.67E-01	-0.004	0.010	7.04E-01

3.552519	-0.008	0.015	5.90E-01	0.062	0.022	5.75E-03
0.650115243	-0.005	0.010	6.04E-01	-0.010	0.015	4.93E-01
7.251596	0.004	0.009	6.24E-01	0.010	0.014	4.80E-01
8.382345	0.009	0.010	3.84E-01	0.017	0.015	2.52E-01
5.759181	0.004	0.010	7.00E-01	-0.001	0.014	9.31E-01
6.546433	0.006	0.010	5.63E-01	-0.015	0.015	3.10E-01
3.746304	-0.005	0.010	5.95E-01	0.005	0.014	7.02E-01
5.860447	0.007	0.009	4.57E-01	0.008	0.013	5.29E-01
8.513891	0.003	0.007	6.93E-01	0.008	0.010	4.61E-01
4.20318	-0.003	0.011	8.08E-01	0.016	0.017	3.46E-01
7.704097	-0.004	0.011	6.85E-01	0.005	0.016	7.74E-01
5.655896	-0.008	0.008	3.27E-01	-0.020	0.012	9.60E-02
1.742175434	-0.005	0.006	4.15E-01	-0.002	0.009	8.54E-01
8.182841	-0.003	0.010	7.83E-01	-0.001	0.015	9.43E-01
3.306587	0.001	0.008	8.71E-01	0.008	0.013	5.46E-01
8.344665	0.003	0.009	7.01E-01	0.016	0.013	2.34E-01
2.422442134	0.010	0.012	4.24E-01	0.002	0.018	8.91E-01
8.881611	0.004	0.009	6.84E-01	0.012	0.013	3.78E-01
8.736609	-0.005	0.007	4.71E-01	0.007	0.011	5.01E-01
8.03582	0.002	0.009	8.59E-01	0.001	0.013	9.49E-01
1.088823342	0.004	0.007	6.36E-01	-0.001	0.011	8.93E-01
1.788939564	-0.003	0.010	7.46E-01	0.007	0.015	6.14E-01
6.837111	0.004	0.008	6.04E-01	0.011	0.012	3.40E-01
3.51282	0.003	0.008	7.06E-01	0.028	0.012	1.84E-02
8.461407	-0.004	0.006	5.20E-01	-0.003	0.009	7.79E-01
8.313713	-0.004	0.010	6.80E-01	0.009	0.015	5.48E-01
0.801509909	0.000	0.006	9.90E-01	0.007	0.009	4.39E-01
4.357266	-0.004	0.012	7.27E-01	0.019	0.019	3.20E-01
7.521751	0.000	0.010	9.84E-01	-0.017	0.015	2.41E-01
6.796402	-0.002	0.010	8.56E-01	0.004	0.015	8.01E-01
5.952293	0.004	0.008	6.10E-01	0.005	0.012	6.95E-01
1.068300954	0.003	0.007	6.13E-01	0.001	0.010	9.49E-01
6.747956	-0.005	0.011	6.84E-01	-0.017	0.017	3.14E-01
8.246427	0.004	0.007	5.74E-01	0.011	0.010	2.61E-01
1.030284071	0.001	0.007	8.96E-01	0.021	0.011	5.05E-02
6.174338	0.005	0.008	5.39E-01	-0.004	0.011	7.15E-01
8.450978	0.002	0.009	8.37E-01	0.016	0.013	2.33E-01
5.477923	0.005	0.008	5.25E-01	-0.008	0.011	4.62E-01
3.667243	-0.001	0.004	7.71E-01	0.009	0.007	1.87E-01
8.928712	-0.006	0.008	4.58E-01	0.010	0.012	3.77E-01
5.834205	-0.004	0.009	6.54E-01	-0.010	0.013	4.49E-01
8.722142	0.003	0.009	7.12E-01	-0.005	0.013	6.82E-01
2.751136775	-0.005	0.008	5.33E-01	-0.016	0.012	1.75E-01

0.580473697	-0.003	0.009	7.17E-01	0.006	0.013	6.64E-01
6.549461	0.005	0.010	6.06E-01	0.003	0.014	8.45E-01
6.308911	-0.004	0.010	6.90E-01	-0.010	0.015	4.96E-01
1.462936384	-0.001	0.004	7.77E-01	-0.002	0.006	7.75E-01
6.672595	-0.003	0.010	7.75E-01	-0.017	0.015	2.60E-01
1.470001468	-0.001	0.006	8.96E-01	0.001	0.009	9.24E-01
3.860019	-0.003	0.005	5.44E-01	0.008	0.008	2.90E-01
7.587019	-0.005	0.007	5.29E-01	0.000	0.011	9.65E-01
8.001504	0.004	0.008	5.97E-01	0.004	0.012	7.19E-01
7.41813	-0.002	0.009	8.33E-01	-0.009	0.014	5.07E-01
8.177458	0.003	0.008	7.44E-01	0.000	0.012	9.88E-01
3.785667	-0.004	0.012	7.22E-01	0.025	0.018	1.66E-01
4.197797	-0.003	0.012	7.64E-01	0.020	0.017	2.56E-01
0.67433839	0.006	0.010	5.11E-01	0.002	0.014	8.62E-01
6.729452	-0.008	0.011	4.49E-01	-0.015	0.016	3.42E-01
8.604727	0.002	0.009	7.95E-01	0.023	0.013	8.36E-02
2.7400345	0.003	0.011	7.84E-01	-0.017	0.016	3.07E-01
8.983214	-0.004	0.008	6.30E-01	0.003	0.012	7.81E-01
5.473886	0.003	0.008	7.20E-01	0.003	0.013	8.29E-01
6.865035	-0.001	0.010	9.51E-01	0.012	0.015	4.20E-01
8.106134	0.006	0.009	5.08E-01	-0.011	0.013	3.86E-01
8.530712	-0.002	0.006	7.59E-01	0.002	0.008	8.44E-01
4.356593	-0.004	0.012	7.22E-01	0.021	0.018	2.46E-01
6.871427	-0.002	0.007	7.40E-01	-0.003	0.011	7.61E-01
4.113689	0.000	0.010	9.91E-01	0.014	0.015	3.57E-01
6.3873	0.000	0.008	9.78E-01	-0.011	0.011	3.30E-01
8.433147	-0.005	0.008	5.31E-01	0.001	0.012	9.37E-01
5.828822	-0.002	0.008	8.18E-01	0.015	0.011	1.85E-01
2.037899682	0.003	0.004	5.11E-01	0.010	0.007	1.32E-01
7.094818	0.004	0.009	6.75E-01	-0.014	0.013	2.82E-01
6.867726	0.005	0.009	5.77E-01	-0.006	0.014	6.75E-01
0.826405921	-0.005	0.007	4.76E-01	0.004	0.011	7.39E-01
7.208532	-0.004	0.007	5.45E-01	-0.010	0.010	3.21E-01
1.098579887	0.005	0.008	5.67E-01	-0.009	0.012	4.55E-01
7.455474	-0.002	0.008	8.49E-01	0.003	0.012	8.22E-01
3.743949	-0.009	0.010	3.41E-01	-0.012	0.015	4.07E-01
2.84399217	-0.002	0.008	8.37E-01	-0.017	0.012	1.63E-01
2.091728897	-0.001	0.005	7.85E-01	0.010	0.008	1.86E-01
8.558973	-0.004	0.008	6.05E-01	-0.016	0.011	1.73E-01
4.035973	0.008	0.015	5.90E-01	-0.005	0.022	8.09E-01
8.467799	-0.004	0.009	6.22E-01	-0.013	0.013	3.29E-01
7.362618	-0.005	0.009	5.81E-01	0.001	0.013	9.48E-01
1.722998777	-0.002	0.005	6.09E-01	-0.003	0.007	6.58E-01

4.16449	-0.005	0.011	6.75E-01	0.002	0.016	9.24E-01
1.442077563	0.003	0.004	5.17E-01	0.006	0.006	3.29E-01
5.288175	-0.005	0.009	5.91E-01	-0.025	0.013	6.02E-02
0.539765354	0.005	0.008	4.99E-01	0.020	0.011	7.90E-02
8.018325	0.002	0.008	8.03E-01	-0.004	0.011	7.46E-01
8.560655	0.000	0.008	9.74E-01	-0.009	0.011	4.19E-01
6.007804	0.003	0.007	6.90E-01	-0.009	0.010	3.70E-01
3.186144	0.003	0.006	6.12E-01	0.015	0.009	1.08E-01
7.35118	-0.005	0.010	6.20E-01	0.003	0.015	8.28E-01
5.69795	0.006	0.008	4.28E-01	-0.003	0.012	7.93E-01
8.6488	0.001	0.009	9.07E-01	0.008	0.013	5.23E-01
6.779917	0.004	0.008	6.24E-01	-0.007	0.012	5.42E-01
8.060379	0.006	0.007	4.48E-01	-0.006	0.011	5.54E-01
2.984957	-0.001	0.004	7.39E-01	0.006	0.006	3.35E-01
6.02799	0.004	0.009	6.07E-01	-0.014	0.013	2.63E-01
7.534872	-0.005	0.007	4.82E-01	-0.011	0.011	3.25E-01
8.622558	-0.006	0.009	5.24E-01	0.018	0.013	1.64E-01
8.010251	-0.002	0.008	8.17E-01	-0.003	0.012	7.89E-01
6.689417	-0.002	0.008	7.93E-01	-0.018	0.012	1.22E-01
1.741166137	-0.004	0.006	5.28E-01	0.015	0.010	1.26E-01
4.005694	0.003	0.010	7.45E-01	0.020	0.014	1.64E-01
5.604085	0.001	0.009	8.92E-01	0.005	0.013	7.01E-01
2.556342305	-0.013	0.013	2.92E-01	-0.002	0.019	9.36E-01
6.447185	0.004	0.009	6.46E-01	-0.030	0.014	3.11E-02
1.500280401	-0.003	0.005	5.96E-01	-0.008	0.007	3.09E-01
5.935135	-0.004	0.009	6.15E-01	-0.019	0.013	1.52E-01
2.750127477	-0.005	0.008	5.55E-01	-0.015	0.012	2.07E-01
6.52692	0.002	0.007	8.29E-01	0.006	0.011	6.04E-01
6.826008	0.002	0.008	7.57E-01	0.004	0.012	7.52E-01
3.515175	0.002	0.008	8.36E-01	0.020	0.011	7.93E-02
0.620509175	-0.001	0.008	8.79E-01	-0.019	0.012	1.29E-01
5.704006	0.000	0.010	9.92E-01	-0.039	0.014	5.89E-03
1.650329337	0.003	0.005	5.87E-01	-0.001	0.007	8.81E-01
8.33289	0.005	0.007	4.78E-01	0.003	0.010	7.65E-01
6.812551	0.001	0.008	9.37E-01	0.005	0.012	6.83E-01
2.983948	-0.002	0.004	6.51E-01	-0.001	0.006	8.13E-01
6.395375	-0.003	0.007	7.15E-01	-0.004	0.011	7.31E-01
6.5212	0.003	0.008	6.49E-01	0.004	0.011	7.34E-01
7.991074	0.000	0.009	9.81E-01	0.002	0.013	8.83E-01
8.786065	0.001	0.007	8.73E-01	0.003	0.011	7.92E-01
3.191191	-0.002	0.007	7.51E-01	0.017	0.010	9.46E-02
8.652164	-0.003	0.010	7.94E-01	-0.004	0.015	7.81E-01
8.925684	0.005	0.010	6.16E-01	0.024	0.015	1.20E-01

5.826804	0.005	0.007	4.40E-01	0.005	0.010	6.32E-01
6.355676	-0.004	0.008	5.74E-01	-0.002	0.012	8.48E-01
2.83221703	0.007	0.012	5.74E-01	-0.023	0.018	2.02E-01
7.263707	-0.005	0.010	6.17E-01	0.020	0.015	1.89E-01
8.966056	-0.003	0.009	7.80E-01	-0.009	0.014	5.35E-01
6.625158	0.000	0.010	9.95E-01	-0.013	0.015	3.81E-01
8.757131	-0.003	0.010	7.67E-01	-0.010	0.015	4.84E-01
8.570411	0.004	0.009	6.30E-01	0.014	0.013	2.91E-01
7.886107	-0.002	0.009	8.59E-01	0.016	0.013	2.30E-01
3.032058	-0.002	0.006	7.15E-01	0.008	0.009	3.44E-01
0.593594568	0.003	0.010	7.32E-01	-0.006	0.015	6.94E-01
6.195534	-0.001	0.010	9.48E-01	-0.020	0.015	1.82E-01
8.609437	0.000	0.008	9.78E-01	-0.003	0.012	8.07E-01
2.85038439	-0.002	0.008	8.15E-01	-0.001	0.011	9.36E-01
5.884333	-0.004	0.007	6.19E-01	0.008	0.011	4.71E-01
0.794444825	-0.004	0.005	4.16E-01	0.002	0.008	8.49E-01
2.088028138	-0.002	0.006	7.19E-01	0.016	0.009	7.29E-02
3.302213	-0.002	0.011	8.25E-01	0.011	0.016	4.92E-01
8.300929	-0.002	0.007	7.47E-01	-0.002	0.010	8.30E-01
7.25496	-0.008	0.012	4.93E-01	0.011	0.017	5.30E-01
3.913511	-0.004	0.008	6.54E-01	0.027	0.012	2.52E-02
6.82399	-0.002	0.010	8.62E-01	0.004	0.015	7.77E-01
5.947583	0.005	0.009	5.68E-01	-0.003	0.013	8.34E-01
4.3031	-0.006	0.014	6.68E-01	0.020	0.021	3.46E-01
3.513157	0.003	0.008	7.45E-01	0.023	0.012	4.99E-02
0.552886225	0.008	0.009	3.75E-01	0.016	0.014	2.56E-01
6.508752	0.004	0.007	5.62E-01	0.003	0.010	7.44E-01
6.05255	0.003	0.007	6.26E-01	-0.008	0.010	4.18E-01
7.956758	0.003	0.010	7.54E-01	0.015	0.014	3.01E-01
8.368888	-0.004	0.009	6.70E-01	0.006	0.014	6.93E-01
8.378645	0.005	0.008	4.93E-01	-0.021	0.012	6.66E-02
1.820227795	-0.004	0.010	7.01E-01	-0.002	0.015	9.19E-01
2.749791045	-0.005	0.008	5.55E-01	-0.014	0.012	2.25E-01
8.840903	0.003	0.009	7.64E-01	0.008	0.013	5.49E-01
5.901155	0.000	0.010	9.98E-01	-0.015	0.015	2.90E-01
8.614484	0.001	0.007	8.78E-01	-0.011	0.011	3.05E-01
8.283098	0.005	0.007	5.21E-01	-0.012	0.011	2.76E-01
7.870968	0.006	0.010	5.70E-01	-0.016	0.015	2.72E-01
8.117909	0.003	0.009	7.12E-01	-0.006	0.013	6.28E-01
4.097876	-0.001	0.009	9.30E-01	0.004	0.014	7.67E-01
2.918344	-0.003	0.009	7.70E-01	-0.004	0.013	7.49E-01
7.526125	0.005	0.010	6.31E-01	-0.025	0.015	8.10E-02
7.415775	-0.005	0.011	6.89E-01	0.005	0.017	7.62E-01

5.470858	0.003	0.007	7.17E-01	-0.007	0.010	5.06E-01
2.541539271	0.011	0.030	7.10E-01	0.024	0.045	5.89E-01
1.038694886	0.002	0.008	8.15E-01	0.010	0.011	3.73E-01
6.142714	-0.001	0.009	9.23E-01	-0.013	0.013	3.24E-01
0.606042574	-0.005	0.010	6.00E-01	-0.004	0.015	7.82E-01
7.124088	-0.002	0.008	7.85E-01	-0.013	0.011	2.40E-01
5.482969	0.003	0.007	6.60E-01	0.003	0.011	8.14E-01
1.770099339	-0.004	0.010	6.96E-01	-0.001	0.015	9.68E-01
6.021262	0.004	0.010	6.93E-01	-0.020	0.015	1.68E-01
8.136413	0.003	0.007	6.86E-01	-0.005	0.010	6.30E-01
1.703149254	-0.002	0.004	6.54E-01	0.004	0.006	4.91E-01
7.074296	0.002	0.007	7.38E-01	0.003	0.011	7.52E-01
4.1258	-0.001	0.008	9.04E-01	0.011	0.013	3.80E-01
8.967065	-0.003	0.008	7.11E-01	-0.021	0.012	8.07E-02
4.302427	-0.006	0.014	6.72E-01	0.016	0.022	4.48E-01
3.30726	0.002	0.009	8.23E-01	0.003	0.013	8.36E-01
8.802213	-0.004	0.008	6.45E-01	-0.005	0.012	6.69E-01
6.583104	0.001	0.009	9.00E-01	-0.021	0.013	1.11E-01
8.574112	0.005	0.009	5.59E-01	-0.002	0.013	8.78E-01
6.891276	-0.003	0.010	7.95E-01	0.000	0.015	9.87E-01
7.201131	0.007	0.012	5.55E-01	-0.012	0.017	4.93E-01
0.716056031	0.002	0.008	8.24E-01	-0.005	0.012	6.89E-01
8.315732	0.003	0.009	7.12E-01	-0.007	0.014	6.07E-01
5.710734	0.005	0.008	5.41E-01	0.000	0.012	9.90E-01
7.941282	-0.002	0.007	7.44E-01	-0.003	0.011	7.71E-01
6.872436	-0.002	0.008	8.09E-01	-0.004	0.011	7.44E-01
1.441068265	0.003	0.004	4.81E-01	0.007	0.006	2.34E-01
7.034597	-0.008	0.011	4.83E-01	-0.010	0.016	5.27E-01
6.443484	0.006	0.006	3.65E-01	0.007	0.010	4.85E-01
8.994989	0.003	0.007	6.49E-01	0.016	0.010	1.22E-01
4.203853	0.006	0.013	6.50E-01	0.018	0.019	3.51E-01
5.135771	0.003	0.007	6.90E-01	-0.011	0.010	2.77E-01
8.579831	0.005	0.009	5.66E-01	-0.006	0.013	6.49E-01
8.288481	0.003	0.009	7.81E-01	-0.016	0.014	2.43E-01
7.626381	-0.006	0.012	5.88E-01	0.000	0.018	9.78E-01
6.359376	-0.002	0.010	8.28E-01	-0.006	0.014	6.67E-01
5.388095	0.004	0.008	6.38E-01	-0.004	0.013	7.57E-01
3.565304	0.004	0.006	5.70E-01	0.020	0.009	3.45E-02
3.552856	-0.009	0.016	5.94E-01	0.062	0.024	1.04E-02
3.294812	-0.001	0.011	9.26E-01	0.007	0.016	6.50E-01
1.885495718	-0.007	0.008	3.97E-01	-0.010	0.012	4.07E-01
8.34231	-0.003	0.010	7.45E-01	-0.007	0.015	6.39E-01
4.024534	0.007	0.013	5.68E-01	0.019	0.019	3.23E-01

8.640053	0.006	0.010	5.78E-01	-0.038	0.015	1.25E-02
3.264869	-0.005	0.008	5.31E-01	-0.001	0.012	9.55E-01
6.142041	-0.002	0.008	8.02E-01	-0.007	0.012	5.78E-01
6.424981	0.004	0.007	5.58E-01	-0.001	0.011	9.49E-01
6.366778	-0.001	0.007	8.49E-01	0.013	0.011	2.17E-01
5.858765	0.004	0.008	6.64E-01	-0.021	0.012	8.60E-02
2.781079276	-0.005	0.012	6.69E-01	-0.048	0.018	8.33E-03
2.739698067	0.003	0.011	7.91E-01	-0.018	0.016	2.69E-01
7.414429	0.004	0.010	6.88E-01	-0.015	0.014	3.00E-01
2.546922192	-0.008	0.020	6.95E-01	-0.025	0.030	4.02E-01
3.278327	-0.006	0.012	6.21E-01	0.019	0.018	2.73E-01
7.368338	-0.004	0.009	6.82E-01	0.000	0.014	9.83E-01
8.285453	-0.002	0.007	7.30E-01	0.004	0.010	7.23E-01
6.558208	0.002	0.007	7.59E-01	0.011	0.010	3.07E-01
1.784565941	-0.002	0.010	8.64E-01	0.010	0.016	5.35E-01
7.349161	0.004	0.011	7.07E-01	-0.015	0.016	3.73E-01
2.426142892	-0.004	0.009	6.80E-01	0.013	0.013	3.22E-01
7.190028	0.005	0.011	6.60E-01	-0.023	0.017	1.70E-01
8.397485	-0.001	0.008	8.61E-01	-0.014	0.012	2.63E-01
8.259884	-0.002	0.009	8.39E-01	0.014	0.014	3.05E-01
5.607113	0.005	0.008	5.86E-01	-0.009	0.012	4.74E-01
8.479238	0.003	0.007	6.68E-01	0.003	0.010	7.66E-01
8.035483	-0.003	0.010	7.27E-01	0.000	0.014	9.81E-01
7.448072	0.003	0.006	6.76E-01	0.004	0.009	6.68E-01
8.876901	0.003	0.008	7.14E-01	0.005	0.012	6.55E-01
7.72933	0.001	0.010	9.03E-01	-0.009	0.015	5.28E-01
8.912227	-0.001	0.009	9.19E-01	-0.002	0.013	9.09E-01
2.354146318	0.007	0.008	4.20E-01	0.017	0.012	1.75E-01
6.082492	0.005	0.010	6.33E-01	0.021	0.015	1.59E-01
8.249455	0.003	0.007	7.28E-01	0.015	0.011	1.88E-01
5.442261	0.003	0.007	6.83E-01	-0.012	0.010	2.44E-01
2.740370932	0.003	0.011	8.00E-01	-0.016	0.016	3.30E-01
3.643692	-0.001	0.004	8.20E-01	0.010	0.006	7.37E-02
1.7273724	-0.001	0.005	7.73E-01	0.008	0.007	2.57E-01
3.80518	0.002	0.006	6.72E-01	0.010	0.008	2.28E-01
7.748843	-0.004	0.010	7.25E-01	-0.011	0.015	4.56E-01
7.94431	-0.006	0.011	5.94E-01	0.005	0.016	7.59E-01
3.205994	0.003	0.009	7.57E-01	0.016	0.014	2.67E-01
7.653296	-0.001	0.009	8.86E-01	0.001	0.014	9.30E-01
2.996733	-0.002	0.004	6.01E-01	0.007	0.006	2.62E-01
4.18333	-0.005	0.014	7.42E-01	0.004	0.021	8.55E-01
1.740493271	-0.004	0.007	5.54E-01	0.015	0.010	1.39E-01
6.868399	0.006	0.011	6.08E-01	-0.025	0.016	1.17E-01

4.092157	0.010	0.013	4.77E-01	0.027	0.020	1.78E-01
6.258783	0.001	0.008	8.91E-01	-0.003	0.012	8.17E-01
6.706238	0.000	0.007	9.98E-01	0.004	0.011	7.06E-01
6.941741	-0.003	0.009	7.69E-01	0.017	0.013	2.19E-01
1.865646195	0.004	0.009	6.46E-01	-0.002	0.013	8.82E-01
2.803956692	-0.007	0.014	6.40E-01	-0.064	0.021	2.39E-03
6.862679	0.005	0.010	6.34E-01	0.011	0.015	4.81E-01
6.516154	-0.006	0.009	4.85E-01	-0.025	0.013	5.58E-02
6.096622	-0.006	0.010	5.68E-01	0.000	0.015	9.85E-01
5.735967	0.005	0.009	6.26E-01	-0.001	0.014	9.60E-01
6.399748	-0.005	0.008	5.27E-01	-0.017	0.013	1.83E-01
1.048787864	0.003	0.011	8.01E-01	-0.021	0.017	2.18E-01
4.17862	-0.004	0.013	7.67E-01	0.018	0.019	3.29E-01
8.864453	-0.004	0.007	5.34E-01	0.012	0.010	2.60E-01
7.652623	0.006	0.008	4.54E-01	0.021	0.012	8.26E-02
8.628614	-0.006	0.009	4.93E-01	-0.003	0.014	8.14E-01
7.436297	-0.005	0.010	6.50E-01	-0.023	0.015	1.35E-01
6.401767	-0.002	0.009	8.40E-01	0.001	0.013	9.27E-01
5.986945	0.002	0.009	8.03E-01	-0.014	0.014	3.21E-01
4.029581	-0.004	0.012	7.49E-01	-0.008	0.018	6.58E-01
5.958012	0.006	0.009	5.41E-01	-0.003	0.014	8.47E-01
0.796799853	-0.004	0.006	4.74E-01	0.002	0.009	8.47E-01
4.123782	0.000	0.009	9.66E-01	-0.005	0.013	7.36E-01
0.513187179	-0.003	0.008	7.38E-01	-0.012	0.012	2.98E-01
7.670454	0.002	0.008	8.50E-01	-0.012	0.013	3.37E-01
2.828516271	0.006	0.012	6.19E-01	-0.033	0.019	7.63E-02
8.733581	0.001	0.010	9.03E-01	-0.014	0.015	3.65E-01
8.43382	0.002	0.009	8.10E-01	-0.008	0.014	5.72E-01
5.455382	0.003	0.008	6.72E-01	-0.004	0.012	7.42E-01
8.78842	0.004	0.009	6.20E-01	-0.001	0.013	9.42E-01
5.992328	0.000	0.009	9.65E-01	-0.010	0.014	4.64E-01
5.428467	0.004	0.009	6.19E-01	-0.010	0.013	4.22E-01
3.978779	-0.003	0.012	7.88E-01	-0.001	0.018	9.52E-01
2.787471495	-0.005	0.012	6.87E-01	-0.054	0.018	2.65E-03
8.844604	0.004	0.010	6.95E-01	-0.002	0.015	9.19E-01
3.684064	-0.002	0.004	7.17E-01	0.010	0.007	1.28E-01
7.804018	-0.006	0.009	5.56E-01	-0.011	0.014	4.39E-01
8.80726	-0.004	0.009	6.52E-01	-0.026	0.014	5.29E-02
2.529091265	0.006	0.012	6.13E-01	0.003	0.017	8.56E-01
8.659902	0.003	0.010	7.63E-01	-0.016	0.015	2.77E-01
6.03842	-0.004	0.010	6.89E-01	0.008	0.015	6.00E-01
1.818545632	0.003	0.010	7.34E-01	0.001	0.015	9.22E-01
6.611364	0.003	0.008	7.39E-01	-0.007	0.012	5.45E-01

8.694555	-0.003	0.009	7.51E-01	-0.003	0.014	8.32E-01
7.605859	0.001	0.012	9.62E-01	0.002	0.018	9.00E-01
7.093136	-0.002	0.010	8.10E-01	0.010	0.015	4.94E-01
7.29735	-0.002	0.010	8.53E-01	0.011	0.015	4.63E-01
6.847876	-0.003	0.009	7.18E-01	0.002	0.014	8.89E-01
5.001871	0.004	0.010	6.58E-01	0.003	0.015	8.57E-01
4.02117	-0.004	0.014	7.62E-01	0.007	0.021	7.41E-01
2.809003181	-0.006	0.014	6.78E-01	-0.069	0.021	1.41E-03
2.081635919	-0.001	0.006	8.15E-01	0.010	0.008	2.10E-01
7.132498	0.006	0.010	5.58E-01	-0.022	0.014	1.22E-01
6.199907	0.005	0.008	4.98E-01	0.012	0.012	3.19E-01
5.691558	0.003	0.008	7.68E-01	-0.009	0.013	4.63E-01
7.383477	-0.003	0.009	7.54E-01	0.021	0.013	1.12E-01
7.026859	-0.009	0.010	3.41E-01	-0.031	0.014	3.27E-02
2.329250306	0.000	0.005	9.26E-01	-0.010	0.008	1.85E-01
5.939845	0.000	0.010	9.69E-01	-0.003	0.015	8.30E-01
7.170179	-0.005	0.010	5.75E-01	-0.020	0.014	1.70E-01
6.479146	0.002	0.008	8.40E-01	-0.017	0.012	1.52E-01
2.821787619	-0.005	0.016	7.56E-01	-0.067	0.024	4.78E-03
7.879715	0.003	0.008	6.70E-01	0.007	0.011	5.61E-01
2.831207732	0.007	0.012	5.54E-01	-0.028	0.018	1.25E-01
6.944769	-0.004	0.009	6.46E-01	0.021	0.013	1.07E-01
5.051663	0.003	0.009	7.09E-01	0.006	0.013	6.33E-01
2.804293125	-0.006	0.014	6.73E-01	-0.067	0.021	1.48E-03
1.031966234	0.000	0.006	9.29E-01	0.015	0.008	7.83E-02
2.033526058	0.003	0.004	5.37E-01	0.007	0.006	2.39E-01
8.991625	-0.003	0.007	6.70E-01	0.012	0.011	2.59E-01
1.868337656	-0.003	0.009	7.47E-01	-0.009	0.013	4.89E-01
5.428131	0.004	0.007	5.56E-01	-0.014	0.011	1.95E-01
1.791631025	-0.003	0.009	7.39E-01	0.002	0.013	8.58E-01
1.756305603	-0.001	0.008	9.26E-01	0.011	0.012	3.81E-01
8.43752	-0.004	0.006	5.22E-01	-0.018	0.009	5.18E-02
5.972479	0.004	0.010	6.53E-01	-0.016	0.015	2.63E-01
6.844176	0.001	0.009	8.90E-01	-0.012	0.013	3.52E-01
6.473763	-0.003	0.007	6.69E-01	-0.015	0.011	1.82E-01
6.062643	0.002	0.009	8.46E-01	-0.001	0.013	9.13E-01
2.268692439	0.003	0.006	6.50E-01	0.007	0.009	4.18E-01
8.184187	-0.009	0.010	3.74E-01	-0.024	0.015	1.18E-01
8.772607	-0.002	0.007	8.01E-01	-0.016	0.011	1.49E-01
5.859774	0.002	0.009	8.11E-01	-0.011	0.014	4.43E-01
6.106715	-0.005	0.010	6.19E-01	-0.023	0.015	1.12E-01
8.54787	0.004	0.010	7.22E-01	-0.017	0.016	2.87E-01
5.487679	0.002	0.008	8.09E-01	-0.006	0.011	5.95E-01

8.330198	0.000	0.006	9.92E-01	0.002	0.010	8.26E-01
8.739973	-0.004	0.009	6.39E-01	-0.013	0.013	3.17E-01
8.880266	0.005	0.009	6.11E-01	0.011	0.013	4.21E-01
6.733826	0.002	0.011	8.63E-01	-0.002	0.017	9.09E-01
3.78634	-0.002	0.012	8.42E-01	0.029	0.018	1.08E-01
3.227525	-0.003	0.006	5.75E-01	0.014	0.009	1.25E-01
8.041203	-0.005	0.009	6.00E-01	-0.021	0.014	1.31E-01
2.582247614	0.004	0.011	7.51E-01	-0.008	0.017	6.37E-01
2.845674333	0.000	0.009	9.55E-01	0.009	0.013	5.05E-01
8.607082	0.000	0.007	9.62E-01	-0.020	0.011	6.18E-02
0.527990213	-0.007	0.011	5.30E-01	-0.014	0.016	3.84E-01
6.858306	0.003	0.009	7.02E-01	0.021	0.013	1.03E-01
2.079280891	0.000	0.004	9.57E-01	0.009	0.005	1.13E-01
8.932413	-0.001	0.007	8.60E-01	0.001	0.010	9.01E-01
7.429568	-0.007	0.009	4.87E-01	-0.010	0.014	4.60E-01
7.907303	0.004	0.009	6.08E-01	0.006	0.013	6.51E-01
3.635954	-0.001	0.004	7.53E-01	0.009	0.005	8.50E-02
5.691221	0.002	0.009	7.92E-01	-0.008	0.014	5.72E-01
1.742848299	-0.005	0.006	4.03E-01	-0.001	0.010	9.55E-01
8.009578	-0.004	0.008	6.72E-01	0.009	0.013	4.54E-01
0.992267189	-0.004	0.006	5.42E-01	0.012	0.009	1.98E-01
6.704556	-0.003	0.008	6.87E-01	-0.011	0.012	3.63E-01
2.725567898	-0.005	0.009	5.43E-01	-0.008	0.013	5.53E-01
4.018142	-0.005	0.014	7.07E-01	0.022	0.021	2.86E-01
6.357021	0.002	0.008	8.28E-01	-0.009	0.012	4.52E-01
8.338609	0.004	0.008	6.36E-01	0.003	0.012	7.75E-01
6.746274	-0.004	0.008	6.59E-01	-0.007	0.012	5.51E-01
4.015787	0.004	0.013	7.73E-01	0.022	0.019	2.33E-01
4.285942	0.006	0.011	5.79E-01	0.019	0.016	2.26E-01
8.963028	-0.004	0.010	7.14E-01	-0.006	0.014	6.72E-01
4.355584	-0.004	0.014	7.67E-01	0.010	0.021	6.18E-01
8.431801	0.003	0.007	7.19E-01	0.006	0.011	5.79E-01
7.028877	-0.001	0.010	9.50E-01	-0.026	0.014	7.40E-02
7.065548	-0.001	0.008	8.84E-01	-0.014	0.012	2.29E-01
4.114025	0.000	0.010	9.84E-01	0.013	0.015	3.69E-01
6.992879	0.004	0.007	6.10E-01	-0.002	0.011	8.79E-01
7.913022	0.004	0.009	6.87E-01	-0.002	0.013	8.57E-01
8.264931	-0.002	0.009	7.75E-01	0.000	0.013	9.73E-01
5.764563	0.001	0.008	8.74E-01	0.009	0.013	4.67E-01
5.727556	-0.004	0.009	6.57E-01	-0.017	0.014	2.24E-01
2.985294	-0.002	0.004	7.16E-01	0.005	0.006	3.96E-01
3.515512	0.002	0.008	8.02E-01	0.022	0.011	5.29E-02
7.244867	-0.006	0.009	5.29E-01	0.011	0.014	4.28E-01

5.520986	-0.003	0.007	6.54E-01	-0.010	0.011	3.53E-01
7.632101	-0.003	0.013	8.24E-01	0.005	0.019	7.88E-01
6.50707	-0.005	0.010	6.44E-01	-0.026	0.015	7.96E-02
1.828975043	-0.006	0.010	5.51E-01	-0.002	0.015	8.70E-01
8.932076	0.003	0.008	7.11E-01	-0.005	0.012	6.74E-01
8.652837	-0.003	0.008	7.52E-01	-0.013	0.012	2.88E-01
7.531844	0.002	0.009	8.06E-01	0.009	0.014	5.32E-01
0.640022266	-0.005	0.009	5.36E-01	0.001	0.013	9.24E-01
6.024962	0.002	0.008	7.93E-01	-0.020	0.012	7.95E-02
7.105247	-0.003	0.009	7.16E-01	0.001	0.013	9.37E-01
5.241747	-0.002	0.004	6.84E-01	0.000	0.006	9.89E-01
3.734193	-0.006	0.008	4.38E-01	0.002	0.012	8.76E-01
7.893845	0.004	0.007	5.21E-01	0.015	0.010	1.44E-01
3.16865	-0.003	0.006	6.07E-01	0.005	0.009	6.00E-01
8.372925	0.003	0.007	7.13E-01	0.023	0.011	3.54E-02
6.926265	-0.005	0.009	5.92E-01	0.009	0.014	5.24E-01
3.933361	-0.001	0.006	8.80E-01	0.011	0.010	2.49E-01
6.383599	0.001	0.009	8.90E-01	0.021	0.014	1.30E-01
6.912808	-0.002	0.008	7.86E-01	0.001	0.012	9.22E-01
4.004685	0.002	0.010	8.77E-01	0.028	0.016	7.50E-02
8.031783	-0.004	0.007	5.27E-01	-0.005	0.010	6.36E-01
7.40871	-0.005	0.008	5.39E-01	-0.015	0.011	1.92E-01
0.637667238	0.003	0.009	7.26E-01	0.014	0.014	3.02E-01
6.498323	0.005	0.009	5.66E-01	-0.021	0.014	1.31E-01
7.565151	-0.005	0.008	5.03E-01	-0.008	0.011	4.63E-01
5.012973	0.004	0.009	6.84E-01	-0.004	0.014	7.68E-01
5.49138	0.001	0.010	9.18E-01	-0.019	0.015	2.14E-01
4.047412	-0.001	0.010	9.10E-01	-0.018	0.014	2.07E-01
6.783281	-0.004	0.007	5.38E-01	-0.002	0.011	8.60E-01
5.800898	-0.006	0.010	5.17E-01	-0.020	0.014	1.51E-01
6.940732	0.006	0.010	5.35E-01	0.030	0.015	4.32E-02
7.194066	0.004	0.008	6.73E-01	0.000	0.012	9.99E-01
1.779183019	0.002	0.010	8.39E-01	0.011	0.014	4.39E-01
2.818423293	-0.007	0.016	6.71E-01	-0.081	0.024	7.83E-04
8.317078	0.002	0.009	8.41E-01	0.015	0.014	2.99E-01
0.800500612	0.000	0.006	9.53E-01	0.005	0.009	5.61E-01
8.362832	0.002	0.008	8.15E-01	-0.003	0.012	7.89E-01
2.737679471	0.003	0.011	8.09E-01	-0.018	0.016	2.59E-01
0.598304624	0.004	0.009	6.19E-01	0.012	0.013	3.73E-01
8.421708	0.003	0.006	5.96E-01	-0.018	0.010	5.73E-02
8.680761	-0.003	0.009	7.49E-01	0.013	0.013	3.47E-01
5.538481	0.002	0.006	7.86E-01	0.010	0.009	2.73E-01
6.504379	0.000	0.007	9.52E-01	-0.011	0.011	3.30E-01

0.573408613	0.003	0.007	7.21E-01	0.007	0.011	5.27E-01
4.099559	-0.002	0.011	8.41E-01	0.002	0.016	8.98E-01
3.753706	0.002	0.011	8.69E-01	0.007	0.016	6.90E-01
2.280131148	-0.001	0.006	7.97E-01	-0.005	0.009	5.29E-01
6.559217	-0.003	0.008	7.12E-01	0.004	0.012	7.42E-01
8.515573	0.003	0.009	7.31E-01	0.005	0.013	6.98E-01
6.443148	0.005	0.008	5.02E-01	0.009	0.012	4.38E-01
6.982113	-0.004	0.010	6.76E-01	-0.013	0.015	3.74E-01
4.114362	0.000	0.010	9.71E-01	0.013	0.014	3.57E-01
8.499424	0.003	0.007	7.00E-01	0.007	0.011	5.06E-01
0.65617103	-0.007	0.011	5.18E-01	-0.001	0.017	9.40E-01
7.45581	0.003	0.007	6.23E-01	0.001	0.010	8.89E-01
6.744255	-0.007	0.011	5.45E-01	-0.030	0.017	7.29E-02
3.804507	0.003	0.006	6.01E-01	0.010	0.008	2.15E-01
6.873445	0.000	0.009	9.80E-01	-0.006	0.013	6.76E-01
6.899014	0.003	0.008	7.39E-01	-0.001	0.012	9.46E-01
0.79377196	-0.004	0.005	4.22E-01	-0.001	0.008	9.49E-01
0.908159041	-0.002	0.006	7.76E-01	-0.021	0.008	1.32E-02
8.75915	0.001	0.009	8.90E-01	-0.012	0.014	3.95E-01
6.590842	-0.002	0.010	8.21E-01	-0.014	0.014	3.21E-01
0.586865916	-0.003	0.010	7.67E-01	-0.040	0.015	7.00E-03
7.538236	0.002	0.009	8.42E-01	-0.008	0.014	5.55E-01
1.671188158	-0.002	0.004	6.66E-01	0.003	0.006	5.81E-01
6.890603	0.005	0.009	5.74E-01	0.000	0.014	1.00E+00
4.197124	-0.003	0.012	8.15E-01	0.013	0.018	4.48E-01
6.538695	0.003	0.010	7.46E-01	0.012	0.014	3.89E-01
7.921769	0.002	0.011	8.21E-01	-0.002	0.016	8.82E-01
8.805578	0.005	0.009	6.23E-01	-0.013	0.014	3.34E-01
3.300531	-0.001	0.013	9.06E-01	0.009	0.019	6.45E-01
0.826069489	-0.005	0.007	4.98E-01	0.004	0.011	7.01E-01
5.690212	0.002	0.008	7.95E-01	-0.017	0.013	1.64E-01
3.927978	-0.001	0.008	9.18E-01	0.015	0.013	2.48E-01
6.198225	0.004	0.008	6.61E-01	0.011	0.012	3.74E-01
3.437123	-0.003	0.011	7.88E-01	0.015	0.017	3.79E-01
5.748751	0.001	0.011	9.13E-01	0.017	0.016	2.90E-01
7.019793	-0.002	0.008	8.27E-01	-0.023	0.012	5.83E-02
8.707339	0.002	0.007	7.67E-01	-0.016	0.010	1.21E-01
0.747344262	-0.003	0.006	5.79E-01	-0.008	0.009	3.44E-01
0.528663078	0.003	0.010	7.80E-01	-0.023	0.014	1.04E-01
7.965506	0.001	0.009	8.87E-01	0.004	0.013	7.39E-01
8.721133	-0.002	0.008	8.16E-01	-0.005	0.013	7.14E-01
1.68969195	-0.002	0.004	5.86E-01	0.002	0.005	7.73E-01
5.658924	0.002	0.009	8.10E-01	-0.018	0.014	2.03E-01

0.708990947	0.005	0.009	5.95E-01	-0.001	0.014	9.16E-01
3.762117	0.004	0.008	6.55E-01	0.021	0.013	9.85E-02
6.533985	-0.003	0.007	6.59E-01	0.011	0.010	2.77E-01
7.313163	-0.006	0.008	4.80E-01	0.017	0.012	1.81E-01
6.948133	-0.002	0.009	8.17E-01	0.009	0.013	4.86E-01
2.584602643	0.002	0.011	8.64E-01	0.013	0.016	4.31E-01
2.818086861	-0.007	0.016	6.78E-01	-0.078	0.024	1.08E-03
1.89087864	0.006	0.008	4.86E-01	-0.007	0.012	5.88E-01
3.806189	0.001	0.006	7.99E-01	0.011	0.008	1.93E-01
3.300195	0.000	0.012	9.70E-01	0.010	0.018	5.94E-01
8.967402	0.007	0.010	4.76E-01	-0.021	0.015	1.53E-01
1.437703939	0.003	0.004	4.62E-01	0.001	0.006	8.35E-01
3.189508	-0.002	0.006	7.04E-01	0.012	0.009	2.06E-01
4.180639	-0.004	0.011	7.34E-01	0.002	0.016	8.87E-01
7.869959	0.005	0.008	5.50E-01	-0.007	0.012	5.84E-01
6.106379	-0.006	0.009	4.81E-01	-0.017	0.013	2.10E-01
8.240707	-0.002	0.008	8.41E-01	0.001	0.011	9.55E-01
8.985906	0.002	0.010	8.16E-01	0.015	0.014	3.05E-01
2.65054343	-0.005	0.015	7.51E-01	0.020	0.023	3.78E-01
6.142377	0.003	0.006	6.35E-01	-0.005	0.009	5.91E-01
7.411065	-0.005	0.008	4.76E-01	-0.011	0.011	3.27E-01
4.143295	-0.005	0.014	7.28E-01	0.010	0.020	6.07E-01
2.35448275	0.006	0.008	4.41E-01	0.019	0.011	1.02E-01
1.45856276	0.002	0.004	6.63E-01	0.000	0.005	9.44E-01
5.502819	0.003	0.010	8.00E-01	-0.016	0.015	2.94E-01
7.157394	-0.004	0.007	5.58E-01	-0.017	0.011	1.17E-01
4.10696	-0.003	0.012	8.11E-01	0.019	0.018	2.88E-01
2.426479325	-0.003	0.009	7.03E-01	0.016	0.013	2.29E-01
3.614759	-0.001	0.007	8.29E-01	0.008	0.010	4.08E-01
6.327079	0.005	0.009	5.40E-01	-0.022	0.013	8.46E-02
2.571145339	0.005	0.010	5.80E-01	0.004	0.014	8.03E-01
5.44899	0.005	0.009	5.85E-01	0.010	0.013	4.71E-01
8.52432	0.002	0.009	8.30E-01	0.013	0.013	3.13E-01
6.820289	-0.005	0.010	6.22E-01	0.013	0.014	3.69E-01
5.288511	-0.004	0.009	6.25E-01	-0.025	0.014	6.21E-02
5.740677	-0.003	0.009	7.05E-01	-0.004	0.014	7.70E-01
7.698042	-0.004	0.010	7.17E-01	0.014	0.015	3.69E-01
5.998721	0.003	0.009	7.15E-01	-0.014	0.014	3.02E-01
3.77221	-0.001	0.009	9.46E-01	0.018	0.014	1.86E-01
7.932199	-0.002	0.009	8.16E-01	-0.017	0.013	1.82E-01
2.54793149	-0.006	0.014	6.75E-01	-0.015	0.021	4.58E-01
7.0965	-0.002	0.009	8.00E-01	-0.009	0.014	5.38E-01
2.32454025	0.004	0.008	6.34E-01	-0.011	0.012	3.75E-01

3.89097	0.001	0.005	7.76E-01	0.021	0.007	3.44E-03
8.627605	-0.003	0.008	7.39E-01	-0.012	0.012	3.21E-01
1.866991926	-0.002	0.009	8.20E-01	-0.013	0.013	3.47E-01
5.960031	0.004	0.008	6.11E-01	0.001	0.012	9.43E-01
2.091056031	-0.001	0.005	7.94E-01	0.013	0.008	1.01E-01
4.376779	-0.005	0.014	7.09E-01	0.020	0.021	3.41E-01
4.354238	-0.004	0.014	7.94E-01	0.025	0.021	2.42E-01
7.251259	-0.005	0.010	6.09E-01	0.012	0.015	4.44E-01
8.025054	-0.003	0.009	7.09E-01	-0.008	0.013	5.47E-01
2.737343039	0.002	0.011	8.15E-01	-0.017	0.016	2.95E-01
0.786370443	0.001	0.004	8.88E-01	0.000	0.007	9.49E-01
7.928161	-0.007	0.009	4.75E-01	-0.009	0.014	5.29E-01
0.504776364	-0.004	0.009	6.64E-01	-0.013	0.013	2.96E-01
7.4511	0.003	0.006	6.02E-01	0.007	0.009	4.34E-01
2.726240763	-0.005	0.009	5.65E-01	-0.009	0.013	4.74E-01
8.63938	0.001	0.007	9.20E-01	-0.014	0.011	1.97E-01
6.632896	-0.003	0.009	7.27E-01	-0.013	0.014	3.46E-01
0.788389038	0.000	0.005	9.51E-01	0.002	0.007	8.06E-01
1.885159286	-0.006	0.008	4.27E-01	-0.013	0.012	2.92E-01
8.039857	0.003	0.007	6.69E-01	0.005	0.010	6.44E-01
0.592921703	0.007	0.010	4.94E-01	-0.011	0.014	4.30E-01
8.514227	0.002	0.008	7.54E-01	-0.003	0.012	8.19E-01
0.542793247	-0.005	0.009	5.65E-01	0.002	0.014	9.00E-01
8.175776	-0.005	0.006	3.85E-01	-0.009	0.009	3.29E-01
6.759395	0.007	0.010	4.88E-01	0.013	0.014	3.70E-01
0.821023	0.000	0.006	9.55E-01	0.011	0.009	2.18E-01
5.442598	0.003	0.008	6.66E-01	-0.008	0.011	4.78E-01
8.671341	-0.003	0.007	7.04E-01	-0.020	0.010	4.31E-02
3.688102	-0.002	0.004	7.06E-01	0.006	0.007	3.44E-01
8.770589	0.004	0.008	6.50E-01	-0.003	0.012	8.18E-01
8.42911	-0.005	0.011	6.60E-01	0.004	0.016	8.21E-01
6.150452	-0.002	0.007	7.95E-01	-0.001	0.011	9.37E-01
6.800103	0.004	0.010	7.16E-01	0.012	0.015	4.39E-01
3.275299	-0.006	0.012	6.14E-01	0.016	0.017	3.53E-01
6.281324	-0.001	0.011	9.53E-01	-0.005	0.016	7.29E-01
7.170852	0.001	0.009	8.63E-01	0.004	0.013	7.39E-01
6.628186	0.005	0.010	5.72E-01	-0.015	0.014	3.06E-01
5.675745	0.001	0.009	9.51E-01	0.011	0.014	4.42E-01
5.559003	0.001	0.007	8.61E-01	-0.007	0.011	4.92E-01
8.557627	-0.001	0.007	9.10E-01	0.011	0.010	3.00E-01
1.777837289	-0.002	0.008	7.64E-01	0.000	0.012	9.77E-01
7.232082	-0.003	0.009	7.07E-01	0.000	0.013	9.73E-01
8.043221	0.003	0.009	7.09E-01	-0.010	0.013	4.51E-01

3.328792	0.005	0.010	6.13E-01	0.012	0.015	4.26E-01
0.993949352	0.002	0.006	7.12E-01	0.010	0.008	2.40E-01
2.51933472	0.009	0.026	7.26E-01	-0.003	0.039	9.39E-01
7.110967	0.003	0.007	6.61E-01	-0.005	0.010	6.32E-01
5.432168	0.004	0.010	6.79E-01	0.010	0.015	4.88E-01
5.418711	0.003	0.008	6.87E-01	-0.023	0.011	4.56E-02
0.854666259	-0.003	0.007	7.00E-01	-0.011	0.011	3.20E-01
6.032027	0.003	0.007	6.55E-01	0.016	0.011	1.42E-01
2.355492048	-0.001	0.007	8.84E-01	0.013	0.010	1.95E-01
8.755449	-0.002	0.009	8.46E-01	-0.005	0.014	7.26E-01
7.010037	-0.006	0.009	4.83E-01	-0.015	0.014	2.66E-01
5.739667	-0.003	0.008	7.53E-01	-0.002	0.012	8.95E-01
2.324203817	-0.002	0.008	7.98E-01	-0.011	0.011	3.39E-01
4.291998	0.005	0.012	6.53E-01	0.013	0.017	4.43E-01
4.19376	-0.004	0.011	7.23E-01	0.022	0.016	1.80E-01
7.606868	-0.002	0.014	9.04E-01	0.005	0.020	8.01E-01
1.774472963	-0.004	0.011	6.99E-01	0.003	0.016	8.55E-01
6.473091	-0.005	0.007	4.77E-01	0.001	0.011	9.48E-01
8.488995	-0.002	0.009	8.16E-01	-0.001	0.014	9.14E-01
2.80496599	-0.005	0.014	7.09E-01	-0.068	0.021	1.43E-03
8.192934	-0.004	0.009	6.59E-01	-0.002	0.013	8.78E-01
3.563621	0.003	0.006	6.16E-01	0.017	0.009	6.26E-02
7.108948	0.004	0.009	6.24E-01	0.014	0.013	3.11E-01
0.980155615	0.002	0.007	7.54E-01	0.014	0.010	1.70E-01
8.972112	0.003	0.008	6.70E-01	-0.001	0.011	9.35E-01
5.946574	-0.003	0.007	6.40E-01	-0.017	0.011	1.15E-01
7.153694	0.002	0.007	7.92E-01	-0.018	0.010	7.92E-02
2.853412283	-0.003	0.009	7.79E-01	-0.006	0.014	6.66E-01
6.614392	0.000	0.010	9.81E-01	0.014	0.015	3.33E-01
5.973152	0.000	0.008	9.98E-01	-0.006	0.012	6.16E-01
6.207309	-0.003	0.007	6.61E-01	-0.005	0.010	5.99E-01
2.366594323	0.010	0.012	3.94E-01	0.043	0.017	1.35E-02
8.839557	0.005	0.009	5.74E-01	-0.020	0.014	1.54E-01
7.552366	-0.002	0.007	7.25E-01	-0.010	0.010	3.32E-01
8.107144	-0.002	0.008	8.24E-01	-0.016	0.011	1.65E-01
7.346133	0.005	0.011	6.26E-01	0.013	0.016	4.00E-01
1.746549058	-0.001	0.007	9.06E-01	0.009	0.011	3.94E-01
3.995601	-0.003	0.009	6.97E-01	0.017	0.013	1.97E-01
3.053253	-0.003	0.008	7.56E-01	0.008	0.013	5.15E-01
8.764533	-0.005	0.009	5.75E-01	0.028	0.013	3.98E-02
8.732235	-0.002	0.007	7.75E-01	0.005	0.010	6.00E-01
6.0327	0.001	0.010	9.08E-01	0.027	0.014	5.94E-02
6.811205	-0.006	0.011	5.55E-01	-0.007	0.016	6.83E-01

8.681098	0.004	0.009	6.33E-01	0.023	0.013	8.07E-02
5.051999	0.002	0.007	7.55E-01	0.000	0.010	9.65E-01
8.519274	0.007	0.007	3.45E-01	-0.004	0.011	6.97E-01
8.553926	-0.002	0.007	8.24E-01	-0.006	0.011	5.90E-01
6.588823	-0.001	0.008	9.33E-01	-0.005	0.012	6.65E-01
4.292334	0.005	0.012	6.61E-01	0.012	0.018	4.84E-01
8.304293	0.000	0.007	9.89E-01	-0.010	0.010	2.87E-01
4.209236	-0.002	0.013	9.02E-01	0.017	0.019	3.74E-01
6.053896	0.001	0.008	8.81E-01	0.003	0.011	8.24E-01
0.678039149	0.000	0.011	9.77E-01	-0.008	0.016	6.03E-01
2.527745535	0.004	0.011	7.09E-01	0.020	0.016	2.12E-01
7.775085	-0.006	0.011	5.94E-01	-0.010	0.016	5.53E-01
4.123445	-0.001	0.009	9.52E-01	-0.003	0.014	8.30E-01
8.172748	0.004	0.009	6.92E-01	-0.015	0.013	2.62E-01
8.872528	-0.005	0.010	6.25E-01	0.023	0.015	1.19E-01
3.92495	-0.002	0.009	8.32E-01	0.022	0.013	7.64E-02
0.677029851	-0.005	0.009	5.76E-01	-0.013	0.013	3.44E-01
6.5175	-0.002	0.009	8.39E-01	-0.018	0.014	1.99E-01
7.990065	-0.007	0.011	5.13E-01	-0.011	0.016	4.78E-01
5.902837	-0.008	0.008	3.47E-01	0.010	0.013	4.35E-01
6.988505	0.004	0.007	5.59E-01	0.009	0.010	3.42E-01
0.755082212	-0.002	0.005	6.45E-01	-0.001	0.008	9.03E-01
5.759517	0.003	0.009	7.36E-01	-0.004	0.014	7.68E-01
7.94902	-0.006	0.009	4.78E-01	-0.004	0.013	7.86E-01
3.579097	0.000	0.006	9.37E-01	0.012	0.009	1.79E-01
6.273922	0.003	0.007	6.65E-01	-0.001	0.010	9.28E-01
8.003859	-0.003	0.010	7.86E-01	-0.019	0.014	1.89E-01
6.511444	-0.006	0.010	5.22E-01	-0.013	0.015	3.84E-01
6.17131	-0.005	0.009	5.98E-01	0.004	0.014	7.57E-01
0.998659408	-0.001	0.006	8.20E-01	0.009	0.009	3.10E-01
2.738688769	0.003	0.011	8.18E-01	-0.018	0.016	2.63E-01
7.065212	-0.001	0.011	9.53E-01	-0.027	0.016	8.24E-02
0.574081478	0.001	0.009	8.90E-01	0.020	0.013	1.29E-01
8.035147	0.003	0.006	6.39E-01	-0.004	0.009	6.98E-01
2.355155615	0.005	0.007	4.73E-01	0.010	0.010	3.34E-01
2.324876682	0.004	0.008	6.53E-01	-0.013	0.013	3.15E-01
7.205168	0.001	0.006	8.67E-01	-0.004	0.010	6.48E-01
6.176021	0.005	0.008	5.13E-01	-0.004	0.013	7.76E-01
7.238475	-0.006	0.009	5.03E-01	-0.007	0.014	6.01E-01
6.033037	-0.004	0.010	6.45E-01	0.012	0.014	4.08E-01
5.765236	-0.003	0.010	7.38E-01	-0.017	0.015	2.35E-01
7.585673	0.007	0.009	4.64E-01	0.025	0.014	6.94E-02
3.298849	-0.002	0.012	8.78E-01	0.017	0.017	3.27E-01

7.70578	0.004	0.012	7.02E-01	0.014	0.017	4.33E-01
5.295913	0.005	0.012	6.88E-01	-0.026	0.018	1.44E-01
6.601271	-0.005	0.009	6.04E-01	-0.005	0.014	7.16E-01
6.200244	-0.002	0.008	7.79E-01	-0.001	0.013	9.68E-01
6.182749	0.005	0.009	5.63E-01	-0.005	0.013	7.13E-01
0.968044042	-0.002	0.005	6.51E-01	0.014	0.008	8.48E-02
8.139441	-0.007	0.009	4.76E-01	-0.011	0.014	4.21E-01
4.304446	-0.006	0.013	6.22E-01	0.008	0.019	6.54E-01
8.626259	-0.001	0.007	8.36E-01	-0.006	0.010	5.39E-01
8.356777	0.002	0.008	8.33E-01	0.013	0.013	3.18E-01
6.745937	-0.005	0.008	5.67E-01	-0.014	0.012	2.59E-01
2.082981649	-0.002	0.007	8.30E-01	0.013	0.011	2.26E-01
8.152562	0.001	0.008	9.36E-01	-0.002	0.012	8.77E-01
6.347265	0.007	0.009	4.52E-01	-0.017	0.013	1.96E-01
7.834633	-0.003	0.010	7.56E-01	0.017	0.015	2.44E-01
6.116472	-0.004	0.011	6.86E-01	-0.012	0.016	4.63E-01
2.836927086	0.007	0.010	4.86E-01	-0.002	0.015	9.20E-01
1.13390531	0.003	0.007	6.35E-01	-0.018	0.011	1.03E-01
8.843258	-0.001	0.008	9.14E-01	-0.005	0.011	6.64E-01
4.046066	0.008	0.010	4.36E-01	-0.008	0.015	5.77E-01
3.878859	0.002	0.005	7.49E-01	0.017	0.007	1.35E-02
6.452232	0.004	0.008	6.05E-01	-0.015	0.012	2.08E-01
6.836101	-0.006	0.010	5.58E-01	-0.016	0.015	2.90E-01
7.384823	0.001	0.008	8.48E-01	0.005	0.012	6.74E-01
5.697277	-0.002	0.007	8.31E-01	-0.017	0.011	1.21E-01
5.580535	0.003	0.007	7.11E-01	-0.009	0.011	3.73E-01
7.477342	0.004	0.007	6.25E-01	-0.015	0.011	1.66E-01
8.582523	0.003	0.007	6.80E-01	0.007	0.011	5.38E-01
6.450886	0.003	0.007	6.42E-01	0.002	0.011	8.24E-01
2.171463421	0.005	0.009	6.16E-01	-0.007	0.014	6.04E-01
6.190151	0.003	0.007	6.20E-01	-0.009	0.010	3.35E-01
2.814722535	-0.006	0.016	7.12E-01	-0.073	0.023	1.69E-03
0.512514314	-0.005	0.008	5.85E-01	0.006	0.012	6.06E-01
2.54658576	-0.008	0.023	7.28E-01	-0.034	0.034	3.24E-01
6.269549	0.000	0.008	9.80E-01	-0.001	0.011	8.98E-01
8.042885	0.001	0.009	9.29E-01	-0.010	0.013	4.28E-01
6.168955	-0.006	0.008	5.00E-01	0.000	0.012	9.71E-01
8.736945	-0.006	0.009	5.29E-01	0.002	0.014	9.11E-01
4.095858	-0.001	0.010	9.34E-01	0.010	0.014	4.92E-01
4.033618	-0.003	0.014	8.15E-01	-0.012	0.020	5.61E-01
8.535422	-0.001	0.007	9.29E-01	-0.016	0.010	1.05E-01
6.014196	0.005	0.008	5.10E-01	-0.002	0.012	8.64E-01
5.567414	0.004	0.007	5.85E-01	0.000	0.010	9.74E-01

7.597112	0.008	0.010	4.59E-01	0.006	0.015	6.96E-01
0.794781258	-0.004	0.005	4.50E-01	0.002	0.008	7.92E-01
1.481103744	0.006	0.007	3.85E-01	-0.003	0.010	7.48E-01
2.819096159	-0.006	0.016	7.05E-01	-0.076	0.024	1.81E-03
8.00285	0.003	0.007	6.67E-01	-0.012	0.011	2.78E-01
7.938927	-0.002	0.008	8.26E-01	-0.005	0.012	6.54E-01
7.468595	0.002	0.009	8.09E-01	-0.014	0.013	2.89E-01
7.843717	0.005	0.010	6.08E-01	0.005	0.014	7.35E-01
5.653541	0.006	0.009	5.28E-01	-0.009	0.013	4.81E-01
8.531385	0.005	0.009	6.08E-01	0.019	0.014	1.74E-01
2.840964277	-0.001	0.010	9.25E-01	-0.006	0.014	6.53E-01
1.659076584	0.003	0.004	5.56E-01	0.003	0.007	6.01E-01
2.828852704	0.006	0.012	6.46E-01	-0.034	0.018	6.50E-02
5.90923	-0.006	0.009	5.00E-01	-0.027	0.013	4.16E-02
4.20789	-0.001	0.013	9.10E-01	0.013	0.020	5.15E-01
4.094176	0.007	0.012	5.30E-01	0.017	0.018	3.33E-01
5.910239	0.001	0.009	9.01E-01	0.009	0.014	5.27E-01
1.063927331	0.002	0.006	7.99E-01	-0.007	0.010	4.60E-01
2.573500367	-0.002	0.010	8.60E-01	-0.014	0.015	3.39E-01
3.892316	-0.002	0.005	7.35E-01	0.017	0.007	1.11E-02
7.628736	0.000	0.011	9.68E-01	0.015	0.017	3.85E-01
6.161218	0.004	0.008	6.10E-01	-0.008	0.012	5.08E-01
4.229085	-0.003	0.013	8.17E-01	0.022	0.020	2.65E-01
8.760159	0.002	0.009	8.11E-01	0.001	0.013	9.60E-01
5.444616	-0.002	0.008	7.94E-01	-0.001	0.012	9.66E-01
8.357113	0.002	0.006	7.80E-01	0.010	0.009	3.07E-01
5.445625	0.005	0.009	6.01E-01	0.003	0.014	8.44E-01
0.524289454	0.006	0.010	5.36E-01	0.012	0.014	4.09E-01
1.751595547	0.003	0.007	6.51E-01	0.001	0.011	9.18E-01
6.546769	-0.004	0.010	7.15E-01	0.003	0.015	8.54E-01
1.850170296	-0.001	0.008	8.92E-01	0.000	0.012	9.80E-01
6.15819	0.006	0.009	5.18E-01	0.003	0.014	8.51E-01
5.938163	0.005	0.007	5.28E-01	-0.017	0.011	1.26E-01
5.533434	0.002	0.008	7.85E-01	0.008	0.013	5.22E-01
6.524565	0.001	0.007	9.42E-01	-0.012	0.011	2.74E-01
8.500433	0.001	0.010	8.94E-01	0.019	0.014	1.85E-01
5.672045	0.003	0.009	7.71E-01	0.000	0.014	9.72E-01
7.468258	-0.001	0.008	8.62E-01	-0.006	0.012	6.44E-01
5.9856	-0.002	0.007	7.98E-01	0.001	0.011	9.61E-01
7.957431	-0.003	0.010	7.38E-01	0.009	0.014	5.17E-01
2.543894299	-0.017	0.030	5.80E-01	-0.005	0.044	9.14E-01
8.983887	-0.001	0.008	8.73E-01	-0.023	0.012	6.22E-02
0.903112552	0.004	0.007	5.70E-01	-0.019	0.010	5.52E-02

6.114117	0.003	0.008	6.96E-01	0.005	0.011	6.60E-01
5.938499	0.005	0.010	5.98E-01	-0.033	0.015	2.34E-02
2.528081967	0.004	0.011	7.31E-01	0.015	0.017	3.76E-01
1.778173722	-0.002	0.010	8.41E-01	-0.009	0.015	5.45E-01
7.651277	0.008	0.010	4.12E-01	0.001	0.015	9.43E-01
8.36586	0.004	0.009	6.60E-01	-0.008	0.013	5.57E-01
1.704158552	-0.002	0.004	7.28E-01	0.001	0.007	9.12E-01
4.088456	0.007	0.010	4.77E-01	0.002	0.015	9.03E-01
8.040194	0.004	0.008	6.60E-01	-0.005	0.012	6.68E-01
7.14461	-0.002	0.009	7.89E-01	-0.009	0.014	5.07E-01
7.93489	0.006	0.008	4.76E-01	0.000	0.013	9.84E-01
8.90348	-0.003	0.007	6.60E-01	0.004	0.010	6.93E-01
6.540377	0.005	0.009	5.92E-01	0.015	0.013	2.43E-01
2.849375092	-0.003	0.008	7.63E-01	-0.012	0.012	3.45E-01
7.63782	0.002	0.009	8.52E-01	0.015	0.013	2.54E-01
5.777684	0.004	0.010	6.50E-01	-0.016	0.014	2.52E-01
7.672473	0.003	0.009	6.94E-01	0.004	0.013	7.81E-01
4.005021	0.002	0.010	8.70E-01	0.028	0.016	7.32E-02
5.998384	0.003	0.010	7.71E-01	0.002	0.015	9.19E-01
0.857021287	0.003	0.008	6.67E-01	-0.008	0.012	5.19E-01
0.602678248	0.004	0.008	5.90E-01	-0.019	0.011	1.01E-01
6.714649	0.002	0.010	8.40E-01	-0.022	0.015	1.46E-01
1.76370712	-0.002	0.009	8.15E-01	0.007	0.014	6.14E-01
8.83081	0.003	0.009	7.00E-01	-0.004	0.013	7.31E-01
5.469849	-0.004	0.010	6.87E-01	-0.029	0.014	4.10E-02
1.463609249	0.000	0.004	9.33E-01	-0.002	0.006	8.04E-01
2.558697333	-0.008	0.011	4.64E-01	0.004	0.016	8.15E-01
8.808606	0.003	0.008	7.24E-01	-0.024	0.012	4.42E-02
8.826773	0.002	0.007	7.84E-01	-0.003	0.011	7.50E-01
3.751351	-0.006	0.011	5.74E-01	0.031	0.017	6.45E-02
4.206544	-0.002	0.012	8.86E-01	0.017	0.018	3.41E-01
6.344237	0.004	0.008	6.09E-01	-0.006	0.012	6.50E-01
7.54631	-0.004	0.010	6.56E-01	0.004	0.014	7.75E-01
6.790683	0.000	0.009	9.81E-01	0.006	0.013	6.30E-01
1.765389283	-0.003	0.009	7.71E-01	0.008	0.014	5.76E-01
7.816802	-0.002	0.010	8.01E-01	0.015	0.014	3.06E-01
1.134241742	0.003	0.007	6.35E-01	-0.019	0.011	7.68E-02
8.605064	-0.003	0.006	6.18E-01	0.002	0.009	8.10E-01
7.635465	-0.001	0.012	9.42E-01	0.026	0.018	1.49E-01
8.640389	0.005	0.010	6.07E-01	-0.040	0.015	5.81E-03
6.058942	-0.001	0.007	9.36E-01	0.003	0.010	7.90E-01
8.510863	-0.003	0.008	7.35E-01	0.004	0.012	7.58E-01
6.908771	0.000	0.008	9.97E-01	0.004	0.011	7.19E-01

4.209908	-0.002	0.014	8.69E-01	0.021	0.022	3.40E-01
8.000158	0.004	0.009	6.51E-01	0.014	0.013	3.09E-01
4.221011	-0.005	0.014	7.26E-01	0.009	0.021	6.66E-01
7.339404	-0.003	0.010	7.65E-01	-0.014	0.015	3.60E-01
6.967646	-0.006	0.010	5.52E-01	0.003	0.014	8.22E-01
6.111089	-0.002	0.009	8.23E-01	-0.014	0.013	2.93E-01
7.699051	0.002	0.008	7.61E-01	0.019	0.012	1.11E-01
0.763493027	-0.001	0.005	8.48E-01	-0.013	0.008	1.02E-01
7.991747	0.004	0.008	5.99E-01	-0.014	0.011	2.26E-01
0.59897749	-0.006	0.008	4.96E-01	-0.004	0.012	7.59E-01
6.149779	0.003	0.009	7.30E-01	-0.008	0.014	5.66E-01
2.781752141	-0.005	0.012	7.00E-01	-0.045	0.018	1.35E-02
2.073561537	0.000	0.003	8.75E-01	0.008	0.005	9.93E-02
6.997925	-0.001	0.008	9.35E-01	-0.008	0.011	4.86E-01
0.747680695	-0.003	0.006	6.13E-01	-0.011	0.009	1.83E-01
8.756795	0.004	0.009	6.61E-01	-0.009	0.014	4.98E-01
2.830198434	0.007	0.012	5.37E-01	-0.033	0.018	6.41E-02
3.372528	-0.001	0.011	9.13E-01	-0.006	0.017	7.05E-01
1.003705897	0.004	0.009	6.17E-01	0.001	0.013	9.60E-01
8.211438	-0.004	0.008	6.72E-01	0.009	0.012	4.67E-01
8.965383	-0.002	0.008	7.78E-01	0.006	0.012	6.21E-01
3.05359	-0.002	0.009	7.90E-01	0.007	0.013	6.05E-01
6.857633	-0.003	0.008	7.05E-01	-0.002	0.012	8.85E-01
3.616778	-0.001	0.006	8.46E-01	0.002	0.009	8.03E-01
1.83772229	-0.001	0.010	9.51E-01	-0.013	0.015	3.75E-01
5.880633	-0.002	0.007	7.34E-01	-0.017	0.011	1.14E-01
8.808942	0.003	0.010	7.66E-01	-0.017	0.014	2.44E-01
1.863291167	-0.002	0.008	8.35E-01	0.008	0.012	5.20E-01
4.302764	-0.005	0.015	7.26E-01	0.017	0.022	4.28E-01
5.775666	0.002	0.007	8.17E-01	-0.001	0.011	9.05E-01
4.220001	-0.005	0.013	7.03E-01	0.016	0.019	4.02E-01
5.513921	-0.001	0.007	9.41E-01	-0.007	0.011	5.26E-01
6.21	0.001	0.009	9.41E-01	-0.007	0.013	6.02E-01
4.164827	-0.003	0.010	7.80E-01	-0.002	0.016	9.08E-01
8.370907	-0.005	0.008	4.75E-01	0.014	0.011	2.27E-01
2.725904331	-0.005	0.009	5.72E-01	-0.010	0.013	4.44E-01
6.255755	0.004	0.009	6.38E-01	-0.013	0.014	3.36E-01
7.240493	-0.007	0.010	4.65E-01	-0.030	0.015	5.30E-02
6.298818	-0.005	0.008	5.45E-01	-0.008	0.011	4.93E-01
8.300256	0.003	0.007	6.90E-01	0.000	0.011	9.96E-01
1.808116222	-0.006	0.010	5.85E-01	-0.007	0.015	6.46E-01
6.70422	0.002	0.011	8.83E-01	-0.019	0.017	2.48E-01
4.192414	-0.004	0.010	7.04E-01	0.010	0.015	4.96E-01

8.98456	-0.003	0.006	6.08E-01	-0.018	0.010	6.52E-02
7.062184	0.006	0.009	5.31E-01	-0.014	0.013	3.10E-01
0.800164179	-0.001	0.006	9.26E-01	0.005	0.009	5.67E-01
4.124791	0.001	0.009	8.96E-01	0.008	0.013	5.32E-01
4.132193	-0.003	0.012	7.94E-01	0.014	0.017	3.98E-01
3.996947	-0.004	0.009	6.46E-01	0.016	0.013	2.23E-01
5.000189	0.002	0.008	8.58E-01	0.000	0.013	9.97E-01
8.908526	0.004	0.008	6.44E-01	0.010	0.012	4.01E-01
6.611701	-0.003	0.007	6.63E-01	-0.003	0.010	7.94E-01
2.585275508	0.009	0.013	4.76E-01	0.015	0.019	4.39E-01
3.329801	-0.003	0.010	7.88E-01	0.014	0.015	3.67E-01
6.840475	-0.005	0.009	5.94E-01	0.009	0.014	5.34E-01
8.213456	0.002	0.007	7.64E-01	0.009	0.010	3.39E-01
6.052886	0.001	0.008	8.83E-01	0.007	0.012	5.67E-01
8.940487	0.000	0.007	9.64E-01	-0.003	0.010	7.50E-01
8.55729	0.005	0.009	5.57E-01	0.005	0.013	6.83E-01
7.345797	-0.003	0.011	7.95E-01	0.001	0.017	9.75E-01
7.279183	0.000	0.008	9.85E-01	-0.002	0.012	8.41E-01
0.645405187	-0.006	0.010	5.70E-01	-0.019	0.015	1.99E-01
7.079006	0.000	0.009	9.76E-01	0.014	0.013	2.94E-01
8.316405	0.002	0.011	8.29E-01	-0.022	0.016	1.67E-01
6.946788	0.001	0.009	8.67E-01	0.002	0.013	8.52E-01
3.648402	0.002	0.004	6.01E-01	0.013	0.006	4.10E-02
7.446726	0.000	0.008	9.88E-01	0.007	0.012	5.30E-01
7.870632	0.005	0.010	6.06E-01	-0.004	0.015	8.00E-01
8.454678	0.003	0.010	7.84E-01	-0.006	0.015	6.63E-01
8.920974	0.000	0.006	9.59E-01	0.000	0.010	9.70E-01
1.923849034	0.001	0.004	8.64E-01	0.007	0.006	2.31E-01
3.984835	-0.005	0.008	5.87E-01	0.006	0.012	6.58E-01
1.436358209	0.003	0.004	4.59E-01	-0.001	0.006	8.35E-01
5.521323	0.003	0.010	7.90E-01	-0.014	0.014	3.25E-01
6.612037	-0.002	0.007	7.84E-01	0.000	0.011	9.64E-01
7.726975	0.004	0.010	7.04E-01	-0.005	0.014	7.53E-01
2.806648153	0.004	0.015	7.65E-01	-0.067	0.022	2.04E-03
1.690028383	-0.002	0.004	6.08E-01	0.001	0.005	8.75E-01
6.98144	-0.001	0.010	8.91E-01	-0.015	0.015	3.20E-01
3.751014	-0.006	0.011	5.93E-01	0.036	0.017	3.02E-02
6.022607	0.007	0.009	4.39E-01	-0.019	0.014	1.53E-01
7.888126	-0.002	0.008	8.19E-01	0.017	0.011	1.40E-01
8.365187	0.003	0.010	7.27E-01	0.003	0.015	8.23E-01
1.747894788	-0.003	0.007	7.16E-01	0.003	0.011	7.56E-01
7.697705	-0.004	0.010	6.84E-01	0.006	0.015	7.04E-01
3.193546	0.002	0.007	7.70E-01	0.015	0.010	1.26E-01

4.376443	-0.005	0.016	7.47E-01	0.017	0.023	4.62E-01
4.213946	0.004	0.012	7.38E-01	0.016	0.018	3.73E-01
2.749454612	-0.004	0.008	5.83E-01	-0.016	0.012	1.94E-01
8.428773	0.006	0.010	5.54E-01	0.003	0.014	8.09E-01
8.782364	0.006	0.009	5.18E-01	0.015	0.013	2.63E-01
8.347356	-0.001	0.008	9.12E-01	-0.005	0.013	7.16E-01
7.863566	0.001	0.010	9.06E-01	0.002	0.015	8.87E-01
8.824081	0.006	0.008	4.51E-01	0.017	0.012	1.39E-01
8.512208	-0.001	0.010	9.29E-01	-0.018	0.014	2.07E-01
8.343992	-0.003	0.010	7.28E-01	0.017	0.015	2.47E-01
3.605003	0.002	0.009	8.14E-01	-0.006	0.014	6.59E-01
3.574051	-0.001	0.006	8.46E-01	0.011	0.009	2.04E-01
3.578088	0.005	0.007	4.81E-01	0.012	0.010	2.46E-01
6.017224	0.002	0.009	8.39E-01	0.009	0.013	4.84E-01
7.703425	0.004	0.011	6.96E-01	0.021	0.016	1.92E-01
5.12265	0.003	0.010	7.94E-01	-0.013	0.015	4.03E-01
6.245326	-0.003	0.010	7.90E-01	-0.019	0.015	2.23E-01
2.819432591	-0.005	0.016	7.38E-01	-0.076	0.024	1.80E-03
6.374852	-0.003	0.009	7.71E-01	-0.017	0.014	2.28E-01
7.913358	0.003	0.009	7.11E-01	-0.008	0.014	5.54E-01
0.607724737	0.000	0.009	9.77E-01	-0.006	0.013	6.61E-01
3.912166	0.002	0.008	8.41E-01	0.028	0.011	1.27E-02
6.667885	0.001	0.009	9.16E-01	-0.028	0.013	3.25E-02
6.344573	-0.003	0.008	7.30E-01	-0.004	0.012	7.10E-01
0.67568412	0.001	0.010	9.54E-01	-0.002	0.016	9.17E-01
2.520344018	-0.007	0.017	6.84E-01	-0.014	0.025	5.87E-01
7.083043	0.000	0.008	9.86E-01	-0.004	0.011	7.36E-01
7.236456	0.000	0.011	9.96E-01	-0.020	0.017	2.28E-01
4.204189	0.006	0.014	6.84E-01	0.019	0.021	3.72E-01
2.326222413	-0.001	0.006	8.45E-01	-0.010	0.009	2.68E-01
2.367940054	0.000	0.008	9.76E-01	0.026	0.013	3.92E-02
6.51649	-0.005	0.007	4.57E-01	-0.018	0.011	9.95E-02
8.612802	0.002	0.009	7.80E-01	0.015	0.013	2.49E-01
6.8721	-0.003	0.008	7.44E-01	-0.007	0.013	5.76E-01
7.473305	0.004	0.007	5.67E-01	-0.004	0.011	7.35E-01
7.265053	-0.007	0.011	4.85E-01	-0.008	0.016	6.35E-01
8.939814	-0.001	0.007	8.81E-01	-0.006	0.011	5.91E-01
1.05080646	0.003	0.006	6.57E-01	-0.010	0.009	2.70E-01
8.211101	-0.004	0.009	6.57E-01	0.002	0.013	9.06E-01
7.984682	-0.001	0.007	8.88E-01	-0.004	0.011	7.40E-01
7.452782	-0.001	0.008	8.64E-01	0.008	0.013	5.11E-01
1.739820406	-0.002	0.007	7.06E-01	0.017	0.010	7.27E-02
6.473427	-0.004	0.008	6.13E-01	-0.008	0.011	4.99E-01

6.458624	0.003	0.007	6.29E-01	0.018	0.011	8.71E-02
8.374271	0.002	0.009	8.01E-01	-0.017	0.013	1.84E-01
4.111334	-0.002	0.010	8.41E-01	-0.002	0.015	9.07E-01
7.967188	0.003	0.007	6.92E-01	-0.010	0.011	3.32E-01
8.094023	-0.001	0.008	8.56E-01	-0.029	0.012	1.51E-02
3.898372	-0.002	0.005	7.11E-01	0.017	0.007	1.94E-02
7.000617	0.005	0.011	6.18E-01	-0.011	0.016	4.98E-01
1.464282114	0.000	0.005	9.81E-01	0.004	0.007	5.95E-01
8.15088	-0.001	0.009	8.85E-01	0.020	0.013	1.21E-01
0.948194519	0.002	0.005	6.76E-01	-0.002	0.008	7.86E-01
6.902379	0.004	0.008	6.74E-01	0.007	0.012	5.87E-01
3.555547	-0.006	0.026	8.09E-01	-0.021	0.039	5.97E-01
4.125127	0.001	0.009	9.48E-01	0.009	0.013	4.95E-01
3.209358	-0.006	0.013	6.27E-01	0.007	0.019	7.29E-01
8.622895	-0.006	0.008	4.63E-01	0.016	0.012	1.91E-01
7.698378	0.003	0.010	7.95E-01	0.021	0.015	1.53E-01
2.092065329	-0.002	0.005	7.69E-01	0.010	0.008	2.00E-01
6.884211	0.007	0.009	4.74E-01	0.011	0.014	4.25E-01
8.460398	-0.002	0.006	6.86E-01	-0.012	0.009	1.82E-01
8.677733	0.003	0.011	7.54E-01	0.004	0.016	8.07E-01
6.28637	-0.001	0.009	8.86E-01	0.013	0.014	3.40E-01
5.985936	-0.001	0.010	8.91E-01	-0.018	0.015	2.32E-01
6.113108	-0.004	0.008	6.15E-01	0.002	0.011	8.62E-01
7.407364	-0.004	0.008	6.23E-01	-0.011	0.012	3.62E-01
7.650268	0.001	0.008	9.29E-01	-0.007	0.012	5.45E-01
6.93905	-0.004	0.009	6.93E-01	0.018	0.014	1.98E-01
0.61075263	-0.003	0.010	7.34E-01	0.006	0.014	6.61E-01
7.427886	0.002	0.009	7.85E-01	-0.013	0.013	3.31E-01
3.782303	0.001	0.011	9.04E-01	0.009	0.016	5.69E-01
8.432474	-0.003	0.010	7.78E-01	-0.022	0.015	1.45E-01
8.689172	-0.002	0.006	7.71E-01	0.004	0.009	6.15E-01
8.547534	-0.005	0.010	5.98E-01	-0.036	0.015	2.09E-02
7.084725	0.004	0.008	6.21E-01	0.005	0.011	6.76E-01
4.016123	0.003	0.013	7.95E-01	0.018	0.019	3.48E-01
0.786706875	0.000	0.004	9.61E-01	-0.001	0.007	8.97E-01
5.972815	0.000	0.008	9.56E-01	-0.008	0.013	5.28E-01
3.723091	0.002	0.008	8.32E-01	0.006	0.011	5.93E-01
6.75603	-0.001	0.009	8.95E-01	-0.004	0.013	7.53E-01
7.963823	0.000	0.007	9.77E-01	0.002	0.010	8.70E-01
7.025849	0.000	0.010	9.96E-01	-0.026	0.014	7.14E-02
7.595093	-0.004	0.010	6.67E-01	0.008	0.014	5.89E-01
1.05215219	0.003	0.006	6.11E-01	-0.015	0.010	1.07E-01
8.554599	0.004	0.007	5.94E-01	-0.005	0.010	6.45E-01

8.719451	0.003	0.008	6.88E-01	0.014	0.012	2.42E-01
8.334908	-0.004	0.010	7.03E-01	-0.017	0.014	2.22E-01
7.871977	-0.002	0.009	8.02E-01	-0.030	0.014	3.34E-02
2.740707365	0.002	0.011	8.36E-01	-0.016	0.016	3.16E-01
5.871549	-0.003	0.010	8.03E-01	-0.011	0.015	4.80E-01
5.791815	0.003	0.009	7.45E-01	0.016	0.013	2.38E-01
8.945534	0.000	0.009	9.66E-01	-0.011	0.014	4.42E-01
8.920638	-0.003	0.006	6.07E-01	-0.002	0.009	8.08E-01
1.926204062	0.001	0.004	8.13E-01	0.007	0.006	2.51E-01
7.541937	-0.003	0.010	7.54E-01	-0.003	0.015	8.60E-01
4.287288	0.005	0.011	6.08E-01	0.009	0.016	5.57E-01
7.335031	0.000	0.006	9.44E-01	0.000	0.009	9.99E-01
8.812643	-0.001	0.009	9.52E-01	-0.002	0.013	8.54E-01
1.648647174	0.003	0.005	5.41E-01	0.008	0.007	3.01E-01
5.077568	0.005	0.009	5.85E-01	-0.013	0.013	2.98E-01
7.869286	-0.002	0.008	8.22E-01	0.001	0.012	9.21E-01
6.275268	0.000	0.010	9.60E-01	-0.005	0.014	7.39E-01
7.841698	0.001	0.008	8.74E-01	-0.010	0.012	4.12E-01
8.468472	-0.001	0.009	9.01E-01	-0.013	0.013	2.98E-01
8.535759	0.000	0.010	9.61E-01	-0.021	0.015	1.55E-01
7.198439	0.003	0.009	7.09E-01	-0.006	0.014	6.76E-01
3.551173	0.001	0.014	9.60E-01	0.060	0.021	4.61E-03
5.016337	0.003	0.009	7.81E-01	0.000	0.014	9.95E-01
8.123292	-0.003	0.008	7.22E-01	0.002	0.012	8.97E-01
3.794751	0.003	0.006	5.73E-01	0.006	0.009	5.22E-01
7.724283	0.003	0.010	7.46E-01	-0.006	0.014	6.68E-01
6.817261	-0.003	0.010	7.79E-01	-0.003	0.016	8.55E-01
6.846531	0.001	0.009	9.16E-01	0.024	0.013	6.00E-02
6.019916	-0.003	0.008	7.24E-01	-0.001	0.013	9.66E-01
6.864698	-0.002	0.010	8.28E-01	0.006	0.015	6.84E-01
8.034474	-0.001	0.007	8.63E-01	0.015	0.011	1.72E-01
6.319004	-0.002	0.008	8.37E-01	-0.012	0.012	3.31E-01
8.357449	0.003	0.008	6.96E-01	0.013	0.011	2.43E-01
4.031263	0.006	0.014	6.65E-01	0.005	0.021	8.17E-01
6.818607	0.001	0.010	9.29E-01	0.007	0.015	6.65E-01
3.877513	-0.002	0.004	6.74E-01	0.014	0.007	3.29E-02
6.134303	-0.003	0.007	6.95E-01	-0.009	0.011	4.11E-01
2.037563249	0.003	0.004	5.65E-01	0.010	0.007	1.23E-01
8.722479	0.003	0.009	7.84E-01	-0.011	0.014	4.21E-01
2.843655738	0.000	0.008	9.67E-01	-0.020	0.012	9.63E-02
5.796861	0.002	0.010	8.29E-01	-0.002	0.015	8.98E-01
4.034964	0.005	0.013	7.34E-01	-0.010	0.020	6.11E-01
6.240279	0.003	0.006	6.09E-01	-0.003	0.009	7.83E-01

7.08237	0.006	0.008	4.79E-01	-0.004	0.012	7.39E-01
8.762514	-0.005	0.010	5.86E-01	0.013	0.015	3.99E-01
2.077262295	0.000	0.004	9.67E-01	0.009	0.005	1.03E-01
4.020497	-0.003	0.010	7.97E-01	0.008	0.015	6.10E-01
5.423757	0.002	0.009	8.59E-01	-0.016	0.013	2.18E-01
2.862496	-0.003	0.008	7.41E-01	-0.004	0.012	7.12E-01
5.763218	0.000	0.011	9.89E-01	-0.004	0.016	7.85E-01
2.737006606	0.002	0.011	8.47E-01	-0.016	0.016	3.15E-01
6.999944	0.004	0.010	6.61E-01	0.002	0.015	9.07E-01
7.937245	0.002	0.008	7.71E-01	0.003	0.012	8.10E-01
3.171678	-0.003	0.006	6.17E-01	0.009	0.009	2.87E-01
2.517652557	0.007	0.023	7.41E-01	-0.002	0.034	9.53E-01
5.518631	0.000	0.009	9.64E-01	-0.009	0.013	4.88E-01
8.017653	-0.004	0.007	5.83E-01	-0.009	0.011	3.73E-01
6.17703	0.006	0.009	5.10E-01	-0.006	0.013	6.24E-01
6.156844	0.000	0.008	1.00E+00	0.003	0.012	7.74E-01
7.147974	0.001	0.007	8.87E-01	0.001	0.011	9.52E-01
7.097509	0.002	0.010	8.16E-01	0.000	0.014	9.72E-01
3.435777	0.004	0.015	7.85E-01	0.019	0.023	4.14E-01
8.060716	0.006	0.011	6.02E-01	-0.028	0.016	8.46E-02
5.458073	0.001	0.007	8.44E-01	-0.008	0.011	4.75E-01
1.05349792	0.002	0.006	7.32E-01	-0.008	0.009	3.65E-01
4.200152	-0.002	0.012	8.57E-01	0.026	0.018	1.57E-01
7.320901	-0.004	0.006	5.32E-01	0.002	0.009	7.80E-01
7.534535	0.000	0.008	9.94E-01	0.002	0.012	8.88E-01
6.178376	0.004	0.010	6.98E-01	-0.013	0.015	3.63E-01
7.539582	0.003	0.007	7.03E-01	-0.010	0.011	3.51E-01
7.443699	-0.002	0.007	7.67E-01	0.016	0.010	1.14E-01
7.501901	-0.002	0.008	7.67E-01	0.012	0.012	3.04E-01
3.554874	-0.006	0.025	8.12E-01	0.009	0.037	8.04E-01
7.399626	0.004	0.008	6.23E-01	-0.010	0.012	3.75E-01
5.53949	0.007	0.009	4.49E-01	-0.009	0.013	4.74E-01
7.247222	0.001	0.010	9.28E-01	-0.027	0.015	8.02E-02
5.774656	-0.002	0.009	7.96E-01	-0.003	0.013	8.21E-01
3.7241	0.000	0.008	9.72E-01	-0.004	0.012	7.39E-01
7.770375	-0.007	0.013	5.92E-01	-0.021	0.019	2.85E-01
0.547166871	0.004	0.008	5.71E-01	0.016	0.011	1.53E-01
7.548666	-0.003	0.007	6.58E-01	0.007	0.011	5.35E-01
2.791172253	-0.004	0.012	7.46E-01	-0.058	0.018	1.55E-03
2.790835821	-0.004	0.012	7.17E-01	-0.054	0.018	2.83E-03
6.343564	-0.001	0.007	9.25E-01	0.003	0.010	7.51E-01
8.219849	0.001	0.009	9.01E-01	0.008	0.013	5.38E-01
7.148647	-0.001	0.007	9.39E-01	0.005	0.011	6.70E-01

6.789001	0.007	0.009	4.60E-01	0.013	0.013	3.11E-01
0.60032322	0.004	0.007	6.02E-01	0.007	0.011	5.06E-01
4.134548	-0.005	0.014	6.91E-01	0.017	0.020	4.17E-01
4.284933	0.005	0.010	6.06E-01	0.013	0.016	3.92E-01
3.682046	-0.001	0.005	8.62E-01	0.015	0.007	2.99E-02
5.07925	0.005	0.009	5.72E-01	-0.006	0.013	6.52E-01
3.614423	-0.002	0.007	8.28E-01	0.012	0.010	2.35E-01
7.924797	0.002	0.008	8.07E-01	-0.005	0.013	6.99E-01
5.820075	-0.006	0.009	5.00E-01	-0.003	0.014	8.06E-01
3.306923	0.001	0.008	8.97E-01	0.007	0.013	5.65E-01
7.435288	0.003	0.011	8.00E-01	-0.004	0.016	8.24E-01
8.462753	0.003	0.009	7.25E-01	-0.007	0.013	6.14E-01
8.260557	0.002	0.007	7.91E-01	-0.013	0.011	2.27E-01
6.663848	0.003	0.008	7.49E-01	0.010	0.012	4.13E-01
6.138004	0.001	0.010	9.51E-01	-0.036	0.015	1.32E-02
5.704678	-0.003	0.008	6.72E-01	-0.018	0.012	1.20E-01
6.340873	0.003	0.008	7.15E-01	0.003	0.011	8.17E-01
0.967371177	-0.004	0.006	5.50E-01	0.015	0.009	8.95E-02
6.169628	-0.005	0.009	5.93E-01	-0.005	0.013	6.85E-01
4.042365	0.007	0.014	6.04E-01	-0.014	0.021	5.16E-01
5.969115	-0.004	0.008	6.18E-01	-0.011	0.012	3.71E-01
3.892653	-0.002	0.005	7.40E-01	0.018	0.007	8.38E-03
8.555272	-0.001	0.006	8.09E-01	-0.007	0.009	4.32E-01
8.76857	0.001	0.009	8.97E-01	-0.017	0.013	2.08E-01
7.567169	0.001	0.008	9.35E-01	-0.003	0.012	8.17E-01
4.124455	0.002	0.009	8.32E-01	0.006	0.013	6.47E-01
6.233214	-0.002	0.009	7.81E-01	-0.009	0.013	5.07E-01
5.802917	0.000	0.009	9.95E-01	-0.014	0.013	2.86E-01
3.596255	-0.003	0.007	6.47E-01	0.018	0.010	8.45E-02
4.026553	0.004	0.012	7.36E-01	0.011	0.019	5.58E-01
7.502574	0.003	0.009	7.71E-01	-0.012	0.013	3.50E-01
8.599681	0.002	0.008	7.87E-01	0.017	0.012	1.64E-01
7.763646	-0.003	0.012	8.19E-01	-0.025	0.018	1.75E-01
7.938591	-0.001	0.010	9.31E-01	-0.006	0.015	7.00E-01
5.796525	-0.004	0.010	7.00E-01	0.005	0.015	7.58E-01
8.306312	-0.005	0.008	5.68E-01	-0.025	0.012	3.27E-02
0.825733056	-0.005	0.007	5.31E-01	0.005	0.011	6.61E-01
8.918955	0.006	0.008	4.68E-01	-0.003	0.011	7.75E-01
0.783678982	0.001	0.004	8.26E-01	0.006	0.006	3.67E-01
1.803406166	-0.002	0.009	8.71E-01	0.012	0.014	3.83E-01
6.744928	-0.005	0.008	5.37E-01	-0.008	0.012	5.08E-01
0.954586738	0.002	0.005	6.78E-01	0.009	0.007	2.40E-01
0.983183509	0.004	0.006	5.68E-01	0.008	0.010	4.27E-01

5.934798	-0.003	0.010	8.00E-01	-0.040	0.016	1.01E-02
0.787043308	0.000	0.004	9.27E-01	0.001	0.007	8.20E-01
7.591056	-0.003	0.009	7.25E-01	-0.005	0.014	7.24E-01
5.944891	0.003	0.010	7.75E-01	-0.002	0.015	8.93E-01
0.672656227	0.003	0.009	7.68E-01	-0.009	0.014	5.10E-01
7.661707	0.004	0.009	6.75E-01	-0.009	0.013	4.56E-01
5.552275	0.002	0.009	8.49E-01	-0.019	0.014	1.68E-01
2.781415708	-0.004	0.012	7.25E-01	-0.046	0.018	1.15E-02
7.204831	-0.001	0.007	8.39E-01	-0.004	0.010	6.58E-01
1.78961243	-0.001	0.010	9.34E-01	0.000	0.014	9.85E-01
3.032394	-0.002	0.006	7.70E-01	0.012	0.009	2.05E-01
0.627237827	0.006	0.010	5.60E-01	0.003	0.014	8.57E-01
3.635618	-0.001	0.004	7.85E-01	0.008	0.006	1.51E-01
3.771873	-0.001	0.009	9.01E-01	0.013	0.013	3.15E-01
6.053559	0.001	0.009	8.98E-01	0.006	0.013	6.67E-01
4.200488	-0.002	0.012	8.42E-01	0.022	0.018	2.42E-01
5.610141	-0.002	0.007	7.58E-01	-0.010	0.011	3.67E-01
8.961346	-0.006	0.008	4.50E-01	0.018	0.012	1.42E-01
1.479421581	0.000	0.007	9.71E-01	-0.003	0.010	7.91E-01
8.320105	0.000	0.008	9.55E-01	-0.003	0.013	8.20E-01
6.28267	0.004	0.010	7.13E-01	0.020	0.014	1.63E-01
1.865309763	-0.003	0.009	7.75E-01	-0.009	0.013	5.17E-01
6.85696	-0.003	0.009	7.62E-01	0.002	0.013	8.64E-01
8.942842	-0.003	0.009	7.57E-01	-0.016	0.013	2.33E-01
7.808055	0.001	0.009	8.94E-01	-0.003	0.014	8.23E-01
8.835857	-0.003	0.008	6.45E-01	0.009	0.011	4.40E-01
2.523371911	-0.002	0.012	8.55E-01	0.004	0.019	8.26E-01
6.328761	0.001	0.008	8.87E-01	-0.004	0.013	7.32E-01
6.31564	0.002	0.009	8.55E-01	-0.026	0.013	5.19E-02
8.84965	0.004	0.008	6.28E-01	-0.009	0.012	4.50E-01
5.933116	-0.002	0.008	8.47E-01	-0.010	0.013	4.34E-01
7.131826	-0.006	0.010	5.26E-01	-0.028	0.015	5.81E-02
8.877238	0.001	0.007	9.29E-01	0.001	0.011	9.60E-01
6.754685	-0.001	0.011	9.40E-01	0.001	0.016	9.35E-01
6.826681	-0.002	0.007	7.58E-01	0.008	0.010	4.22E-01
5.872222	0.004	0.009	6.37E-01	-0.018	0.014	1.92E-01
8.412961	-0.002	0.008	8.35E-01	0.001	0.012	9.31E-01
7.810074	-0.004	0.010	6.66E-01	-0.009	0.015	5.59E-01
2.745417421	-0.005	0.009	6.22E-01	-0.018	0.014	1.95E-01
7.837998	0.003	0.008	7.07E-01	0.017	0.013	1.64E-01
1.811816981	0.006	0.011	5.83E-01	-0.001	0.017	9.59E-01
1.897607291	-0.002	0.006	7.27E-01	-0.001	0.009	8.70E-01
0.547503303	0.003	0.009	7.22E-01	0.015	0.013	2.65E-01

8.109835	0.003	0.010	7.63E-01	0.008	0.016	6.15E-01
0.560624174	-0.005	0.008	5.30E-01	-0.005	0.012	6.99E-01
2.857113	-0.001	0.007	8.88E-01	-0.011	0.011	3.16E-01
2.269028872	0.002	0.006	6.96E-01	0.010	0.009	2.35E-01
0.66525471	0.002	0.010	8.33E-01	-0.041	0.015	5.25E-03
7.265726	-0.006	0.010	5.89E-01	0.001	0.015	9.31E-01
0.997313678	0.003	0.006	6.25E-01	0.014	0.009	1.16E-01
7.372711	0.003	0.010	7.44E-01	-0.007	0.015	6.57E-01
2.346408368	0.006	0.008	4.62E-01	0.018	0.012	1.17E-01
5.505847	0.000	0.010	9.69E-01	-0.013	0.015	4.02E-01
2.613199413	-0.005	0.013	6.64E-01	0.038	0.019	4.10E-02
5.641429	0.002	0.008	7.61E-01	0.002	0.011	8.82E-01
5.505174	0.000	0.008	9.92E-01	-0.026	0.011	2.31E-02
8.523647	-0.003	0.009	7.52E-01	-0.012	0.013	3.50E-01
8.871182	0.002	0.008	7.82E-01	0.003	0.012	7.79E-01
0.781996819	0.001	0.004	8.10E-01	0.001	0.007	8.39E-01
5.604758	-0.003	0.009	7.63E-01	-0.014	0.014	3.13E-01
8.333899	0.005	0.008	5.20E-01	0.002	0.012	8.45E-01
0.821359432	0.000	0.006	9.93E-01	0.011	0.009	2.37E-01
8.178131	0.001	0.008	8.65E-01	-0.003	0.012	8.12E-01
6.542732	-0.005	0.010	6.39E-01	-0.009	0.015	5.25E-01
3.650421	-0.001	0.004	8.22E-01	0.011	0.007	8.97E-02
8.873201	-0.004	0.008	6.71E-01	0.015	0.012	2.32E-01
3.985844	-0.004	0.008	6.23E-01	0.008	0.011	4.82E-01
8.167365	0.005	0.009	6.03E-01	-0.002	0.013	8.85E-01
6.650727	0.001	0.009	9.38E-01	-0.006	0.014	6.50E-01
0.653479569	-0.006	0.009	5.25E-01	-0.005	0.013	7.03E-01
6.526247	0.003	0.009	7.73E-01	-0.021	0.014	1.37E-01
8.389074	0.004	0.011	7.24E-01	0.013	0.017	4.50E-01
6.631214	-0.003	0.009	7.12E-01	0.010	0.014	4.64E-01
3.936389	0.001	0.006	8.55E-01	0.020	0.010	3.88E-02
7.469267	0.002	0.007	7.41E-01	0.005	0.010	6.47E-01
2.808330316	-0.005	0.015	7.40E-01	-0.066	0.022	2.51E-03
4.011413	0.004	0.013	7.61E-01	0.009	0.020	6.58E-01
4.125464	0.000	0.009	9.92E-01	0.011	0.013	4.18E-01
2.52976413	0.005	0.013	6.99E-01	-0.001	0.019	9.76E-01
7.502238	-0.002	0.008	8.02E-01	0.019	0.013	1.29E-01
3.69483	0.002	0.005	6.92E-01	0.018	0.007	1.57E-02
6.64097	0.003	0.007	6.76E-01	0.002	0.010	8.51E-01
8.18957	-0.004	0.008	6.00E-01	-0.003	0.012	7.70E-01
5.690885	0.002	0.010	8.63E-01	-0.021	0.014	1.38E-01
1.792640323	0.004	0.009	6.21E-01	0.013	0.013	3.27E-01
6.189478	-0.004	0.009	6.76E-01	-0.009	0.013	4.83E-01

7.700397	-0.004	0.008	6.15E-01	0.029	0.012	1.76E-02
4.124118	0.002	0.009	8.36E-01	0.001	0.013	9.50E-01
6.69951	0.005	0.010	5.73E-01	0.027	0.014	6.08E-02
0.639012968	-0.002	0.009	8.21E-01	0.000	0.013	9.84E-01
8.204709	-0.001	0.007	9.05E-01	0.009	0.010	3.65E-01
8.416998	0.000	0.010	9.98E-01	-0.015	0.015	3.15E-01
5.596684	-0.005	0.009	5.58E-01	-0.013	0.013	3.20E-01
3.514166	0.002	0.008	8.36E-01	0.020	0.012	1.02E-01
8.834511	-0.003	0.010	7.61E-01	-0.019	0.015	1.86E-01
7.935563	0.006	0.009	5.21E-01	0.010	0.013	4.43E-01
8.336927	0.003	0.008	6.89E-01	-0.002	0.012	8.66E-01
8.288144	-0.002	0.008	7.75E-01	0.002	0.013	8.86E-01
8.171066	0.002	0.007	7.75E-01	0.010	0.010	2.88E-01
5.92336	-0.004	0.008	6.74E-01	0.003	0.012	7.89E-01
7.203486	-0.002	0.009	8.41E-01	0.002	0.013	8.59E-01
5.024412	0.002	0.008	7.73E-01	-0.013	0.013	2.91E-01
2.822796917	-0.005	0.015	7.37E-01	-0.065	0.023	3.73E-03
3.76178	0.003	0.008	7.10E-01	0.022	0.012	6.54E-02
6.213365	0.004	0.007	6.23E-01	-0.013	0.011	2.37E-01
8.027746	-0.001	0.010	9.21E-01	0.011	0.016	4.99E-01
8.82139	-0.003	0.010	7.53E-01	-0.004	0.015	7.85E-01
7.265389	-0.005	0.010	6.06E-01	-0.001	0.015	9.22E-01
6.824663	-0.004	0.009	6.73E-01	0.025	0.013	5.96E-02
7.447063	0.005	0.008	5.42E-01	-0.012	0.011	2.80E-01
6.318668	-0.004	0.010	6.69E-01	-0.036	0.015	1.38E-02
6.485202	-0.002	0.008	7.54E-01	-0.021	0.011	6.94E-02
7.42284	-0.004	0.007	5.82E-01	-0.010	0.011	3.37E-01
6.412533	0.003	0.008	7.38E-01	0.003	0.012	8.01E-01
6.778571	0.005	0.009	5.38E-01	0.001	0.013	9.35E-01
8.653847	0.007	0.010	5.01E-01	-0.020	0.015	1.73E-01
7.193393	0.002	0.009	7.73E-01	-0.003	0.013	7.96E-01
4.008722	0.002	0.010	8.28E-01	0.014	0.015	3.75E-01
3.227862	0.001	0.006	9.33E-01	0.017	0.009	5.87E-02
3.683055	-0.001	0.004	8.16E-01	0.012	0.007	6.14E-02
7.776767	0.003	0.011	7.77E-01	-0.011	0.016	4.81E-01
6.146414	0.004	0.009	6.29E-01	-0.018	0.014	1.86E-01
8.865799	-0.003	0.008	7.03E-01	0.003	0.012	7.84E-01
0.523953022	0.004	0.008	5.86E-01	-0.004	0.012	7.60E-01
6.647699	0.001	0.009	8.63E-01	-0.004	0.013	7.34E-01
8.281752	-0.002	0.010	8.43E-01	-0.011	0.014	4.60E-01
6.403449	-0.001	0.008	9.24E-01	0.002	0.012	8.37E-01
7.128461	0.002	0.008	7.89E-01	-0.014	0.013	2.73E-01
2.74911818	-0.004	0.008	6.06E-01	-0.017	0.012	1.57E-01

3.51854	0.003	0.007	7.02E-01	0.023	0.010	2.02E-02
7.868949	0.003	0.008	6.94E-01	-0.007	0.012	5.52E-01
6.448531	-0.004	0.008	6.23E-01	-0.020	0.012	1.14E-01
7.41342	-0.004	0.008	6.28E-01	-0.003	0.011	7.62E-01
6.097632	0.000	0.009	9.67E-01	-0.001	0.014	9.66E-01
3.239973	-0.003	0.006	5.92E-01	0.012	0.009	1.95E-01
8.854024	0.002	0.009	8.63E-01	0.008	0.013	5.46E-01
7.386505	0.001	0.008	9.44E-01	-0.012	0.012	3.12E-01
3.20633	0.002	0.010	8.23E-01	0.015	0.014	3.03E-01
0.508477123	-0.005	0.009	5.89E-01	0.012	0.013	3.43E-01
8.7393	-0.003	0.008	7.38E-01	0.009	0.013	4.48E-01
8.430455	0.001	0.007	8.51E-01	0.005	0.011	6.29E-01
6.607664	-0.005	0.008	4.96E-01	-0.003	0.011	7.70E-01
7.126443	0.006	0.008	4.51E-01	0.006	0.011	5.90E-01
8.345001	0.002	0.008	7.70E-01	0.002	0.012	8.74E-01
5.123323	0.002	0.008	8.14E-01	-0.007	0.012	5.43E-01
6.830382	0.002	0.008	7.66E-01	0.001	0.012	9.44E-01
5.897791	-0.002	0.009	7.87E-01	-0.010	0.014	4.62E-01
2.831544164	0.006	0.012	5.88E-01	-0.020	0.018	2.66E-01
8.67807	0.001	0.009	9.25E-01	-0.004	0.014	7.76E-01
3.172687	-0.002	0.006	7.06E-01	0.008	0.009	3.54E-01
4.005357	0.002	0.010	8.65E-01	0.018	0.014	1.98E-01
8.660912	-0.002	0.007	7.23E-01	-0.005	0.011	6.30E-01
8.830137	0.000	0.009	9.67E-01	-0.012	0.013	3.35E-01
2.736670174	0.002	0.010	8.72E-01	-0.015	0.016	3.44E-01
6.851577	0.003	0.008	7.31E-01	-0.004	0.012	7.62E-01
1.000678003	0.002	0.007	7.41E-01	0.002	0.011	8.47E-01
7.841025	0.004	0.008	6.35E-01	0.000	0.012	9.86E-01
3.723427	0.001	0.008	9.06E-01	0.002	0.012	8.73E-01
8.71104	0.004	0.009	6.98E-01	0.017	0.013	2.10E-01
5.881978	-0.004	0.007	6.07E-01	0.001	0.010	8.91E-01
6.950488	-0.003	0.009	7.71E-01	0.003	0.013	8.38E-01
5.79316	0.002	0.008	8.09E-01	0.000	0.012	9.88E-01
6.387973	0.005	0.009	5.66E-01	-0.009	0.014	5.20E-01
0.720766088	-0.003	0.007	6.78E-01	0.005	0.011	6.67E-01
7.69064	-0.003	0.010	7.53E-01	0.001	0.014	9.52E-01
6.868735	0.005	0.009	5.49E-01	-0.011	0.013	3.98E-01
2.330259604	0.003	0.005	5.95E-01	-0.010	0.008	2.02E-01
7.061848	0.000	0.010	9.84E-01	-0.014	0.016	3.69E-01
0.532027404	-0.002	0.009	8.34E-01	0.034	0.013	8.38E-03
5.793833	0.002	0.009	8.11E-01	-0.003	0.013	7.91E-01
5.779367	0.002	0.009	8.44E-01	-0.008	0.013	5.63E-01
8.028755	-0.001	0.009	8.92E-01	-0.012	0.013	3.72E-01

8.254501	0.004	0.010	7.21E-01	0.008	0.015	5.88E-01
5.962386	0.001	0.010	8.93E-01	-0.022	0.014	1.34E-01
5.995356	-0.003	0.010	7.40E-01	-0.019	0.015	2.07E-01
8.733245	-0.005	0.009	6.17E-01	-0.013	0.014	3.50E-01
1.049124297	0.002	0.010	8.38E-01	-0.019	0.015	1.92E-01
8.573439	0.002	0.009	8.20E-01	0.003	0.013	7.91E-01
8.806251	0.006	0.008	4.18E-01	0.000	0.012	9.84E-01
8.564019	-0.004	0.007	5.93E-01	-0.005	0.011	6.55E-01
7.606195	0.001	0.012	9.36E-01	0.005	0.017	7.88E-01
3.687429	-0.001	0.005	7.69E-01	0.006	0.007	3.60E-01
2.419750673	-0.001	0.017	9.49E-01	-0.013	0.025	6.12E-01
7.664735	0.003	0.009	7.16E-01	-0.004	0.014	8.01E-01
1.042395645	0.000	0.007	9.59E-01	0.022	0.011	3.28E-02
6.855278	0.002	0.008	8.44E-01	0.011	0.013	3.64E-01
8.752421	0.005	0.009	5.36E-01	-0.010	0.013	4.58E-01
4.122772	-0.002	0.009	8.66E-01	0.006	0.013	6.69E-01
6.01386	-0.002	0.008	8.06E-01	-0.005	0.011	6.36E-01
0.543466112	0.003	0.009	7.33E-01	0.019	0.013	1.42E-01
6.625494	-0.001	0.009	9.53E-01	-0.010	0.013	4.46E-01
2.033862491	0.002	0.004	5.90E-01	0.008	0.006	2.15E-01
5.551938	-0.005	0.011	6.69E-01	-0.032	0.016	4.25E-02
3.879195	0.001	0.005	7.98E-01	0.017	0.007	1.53E-02
1.436694642	0.003	0.004	5.21E-01	0.002	0.006	7.75E-01
6.294445	-0.003	0.009	7.13E-01	-0.005	0.014	7.44E-01
8.151216	-0.002	0.008	7.79E-01	0.012	0.012	2.99E-01
5.590291	0.006	0.011	5.87E-01	0.001	0.016	9.49E-01
6.055914	0.004	0.009	6.86E-01	-0.021	0.013	1.18E-01
6.398402	0.001	0.007	8.58E-01	-0.008	0.010	4.38E-01
4.228076	0.004	0.012	7.62E-01	0.023	0.017	1.94E-01
1.18975312	0.003	0.009	7.61E-01	-0.001	0.013	9.24E-01
0.61983631	0.004	0.007	5.88E-01	0.005	0.010	6.30E-01
1.665132371	-0.001	0.004	7.57E-01	0.013	0.006	3.07E-02
7.155712	-0.001	0.007	8.60E-01	-0.006	0.010	5.59E-01
7.286248	-0.008	0.009	4.02E-01	-0.003	0.014	8.15E-01
6.060288	0.001	0.006	8.45E-01	-0.008	0.008	3.41E-01
6.049522	0.000	0.008	9.90E-01	-0.026	0.011	2.43E-02
5.675072	-0.004	0.009	6.58E-01	-0.014	0.014	3.17E-01
4.126473	-0.001	0.008	8.96E-01	0.013	0.012	2.99E-01
1.919138977	-0.002	0.005	6.46E-01	-0.010	0.007	1.94E-01
6.864025	-0.001	0.008	8.79E-01	-0.003	0.012	8.07E-01
2.726577196	-0.005	0.009	6.12E-01	-0.011	0.013	4.28E-01
0.719083925	0.001	0.008	8.57E-01	0.005	0.011	6.37E-01
6.207645	-0.003	0.007	7.07E-01	0.002	0.011	8.38E-01

2.07658943	0.000	0.003	9.96E-01	0.006	0.005	2.01E-01
7.271109	-0.006	0.011	5.58E-01	0.002	0.016	8.98E-01
7.479024	0.003	0.009	7.35E-01	-0.001	0.013	9.24E-01
7.335367	-0.004	0.006	5.13E-01	-0.001	0.009	9.05E-01
2.821451187	-0.004	0.016	7.85E-01	-0.073	0.024	2.26E-03
3.180425	-0.001	0.005	8.81E-01	0.010	0.008	1.88E-01
8.500097	0.001	0.008	8.90E-01	0.018	0.012	1.45E-01
6.357694	-0.004	0.009	6.61E-01	-0.001	0.014	9.39E-01
5.080596	0.006	0.011	6.15E-01	0.002	0.017	9.06E-01
6.133966	-0.004	0.008	5.95E-01	-0.002	0.011	8.36E-01
8.171739	-0.002	0.007	7.78E-01	0.008	0.011	4.49E-01
3.795424	0.003	0.006	6.12E-01	0.010	0.008	2.41E-01
0.784351847	-0.002	0.004	7.11E-01	-0.001	0.006	8.32E-01
3.318699	0.002	0.012	8.43E-01	0.021	0.018	2.63E-01
8.810624	0.002	0.009	8.33E-01	-0.026	0.013	5.40E-02
8.904152	-0.003	0.006	5.64E-01	0.019	0.009	3.08E-02
5.698286	0.004	0.008	5.89E-01	-0.007	0.012	5.83E-01
8.599345	0.000	0.008	9.57E-01	0.020	0.012	1.04E-01
4.136903	-0.004	0.013	7.78E-01	0.014	0.019	4.59E-01
6.134639	-0.001	0.008	9.47E-01	-0.023	0.012	5.82E-02
8.4715	0.004	0.009	6.46E-01	-0.024	0.014	9.26E-02
3.189172	-0.002	0.006	7.46E-01	0.011	0.009	2.34E-01
5.887361	-0.004	0.009	6.17E-01	-0.013	0.013	3.20E-01
2.325549547	0.003	0.007	6.57E-01	-0.007	0.011	5.10E-01
6.202935	0.002	0.008	8.53E-01	-0.004	0.012	7.23E-01
2.27003817	0.003	0.006	5.98E-01	0.006	0.009	5.03E-01
6.797748	0.000	0.010	9.82E-01	0.007	0.015	6.39E-01
6.806832	0.001	0.009	9.35E-01	-0.013	0.013	3.36E-01
0.597631759	0.003	0.008	7.18E-01	-0.003	0.013	8.08E-01
5.295576	0.004	0.012	7.34E-01	-0.026	0.017	1.34E-01
5.584572	0.002	0.007	7.57E-01	0.002	0.011	8.65E-01
5.587264	0.003	0.010	7.73E-01	-0.019	0.014	1.78E-01
8.07821	0.001	0.009	8.91E-01	0.006	0.014	6.55E-01
6.987832	0.002	0.006	7.18E-01	0.008	0.010	4.24E-01
7.517714	0.006	0.009	5.10E-01	-0.010	0.013	4.55E-01
7.937918	0.003	0.007	6.57E-01	-0.004	0.010	7.19E-01
8.47621	0.001	0.009	9.22E-01	-0.038	0.014	6.53E-03
6.941068	0.005	0.009	5.78E-01	0.029	0.014	3.76E-02
6.837447	-0.001	0.008	8.76E-01	0.007	0.012	5.54E-01
6.757713	-0.001	0.005	8.96E-01	-0.001	0.008	8.67E-01
1.651675067	0.001	0.005	8.02E-01	0.006	0.007	4.26E-01
2.53750208	-0.005	0.013	6.75E-01	-0.003	0.019	8.89E-01
6.894977	-0.002	0.010	8.62E-01	0.013	0.015	4.08E-01

8.952262	-0.001	0.007	9.05E-01	-0.017	0.011	1.25E-01
6.113444	-0.004	0.008	6.60E-01	0.006	0.012	6.36E-01
6.240616	0.004	0.007	6.02E-01	0.001	0.011	9.08E-01
7.153357	0.000	0.007	9.52E-01	-0.010	0.010	3.41E-01
8.061389	-0.002	0.011	8.58E-01	-0.006	0.016	7.11E-01
0.797136286	-0.004	0.006	5.24E-01	0.002	0.009	8.46E-01
8.81668	-0.001	0.009	8.84E-01	0.001	0.013	9.10E-01
4.286952	0.005	0.011	6.52E-01	0.014	0.016	3.73E-01
8.009915	-0.001	0.010	9.25E-01	-0.003	0.014	8.21E-01
6.790346	0.007	0.008	4.24E-01	0.008	0.013	5.30E-01
6.487221	0.004	0.009	6.85E-01	-0.001	0.013	9.26E-01
8.92232	0.001	0.007	8.63E-01	0.011	0.010	2.89E-01
7.847081	-0.004	0.011	7.03E-01	-0.006	0.016	7.09E-01
5.661615	0.003	0.007	6.29E-01	-0.003	0.010	7.63E-01
7.332339	-0.004	0.006	5.22E-01	-0.007	0.009	4.30E-01
8.277715	0.003	0.010	7.69E-01	-0.027	0.015	6.75E-02
6.059615	-0.002	0.008	7.73E-01	-0.002	0.011	8.45E-01
7.607541	-0.002	0.015	8.86E-01	-0.003	0.022	8.97E-01
5.602403	0.004	0.010	6.77E-01	-0.001	0.016	9.57E-01
8.854697	-0.005	0.008	5.65E-01	-0.007	0.012	5.79E-01
3.927305	0.000	0.009	9.94E-01	0.017	0.013	1.90E-01
0.762147296	0.000	0.005	9.28E-01	0.000	0.008	9.79E-01
3.924614	-0.003	0.009	7.68E-01	0.023	0.013	6.62E-02
8.252146	0.006	0.009	5.13E-01	0.009	0.013	5.03E-01
0.79242623	-0.003	0.005	5.53E-01	0.002	0.008	8.03E-01
7.866258	-0.004	0.010	7.13E-01	-0.018	0.016	2.52E-01
5.60173	0.004	0.009	6.42E-01	0.006	0.014	6.48E-01
3.79576	0.003	0.005	6.11E-01	0.014	0.008	8.33E-02
6.913144	-0.002	0.009	8.05E-01	-0.007	0.014	6.27E-01
8.972448	-0.001	0.006	9.14E-01	-0.002	0.010	8.49E-01
1.188407389	-0.005	0.015	7.14E-01	0.002	0.022	9.10E-01
6.293772	0.001	0.009	9.08E-01	0.007	0.014	6.00E-01
1.729054563	-0.002	0.005	7.11E-01	0.004	0.008	6.15E-01
4.101577	0.002	0.011	8.24E-01	0.010	0.016	5.36E-01
6.151125	-0.002	0.009	8.19E-01	0.003	0.013	8.28E-01
8.820381	-0.004	0.010	7.24E-01	-0.006	0.015	6.75E-01
6.316986	0.000	0.008	9.59E-01	-0.018	0.012	1.44E-01
6.529275	0.003	0.010	7.20E-01	0.003	0.015	8.25E-01
3.751687	-0.006	0.011	6.00E-01	0.026	0.017	1.23E-01
8.85436	-0.006	0.008	4.63E-01	0.002	0.012	8.95E-01
6.501014	0.006	0.010	5.66E-01	0.010	0.015	5.29E-01
3.723763	0.000	0.008	9.81E-01	-0.002	0.012	8.65E-01
6.813224	-0.003	0.009	7.17E-01	0.012	0.014	3.94E-01

6.178712	-0.002	0.008	8.17E-01	-0.016	0.012	1.86E-01
8.697583	0.002	0.007	8.12E-01	-0.025	0.011	2.41E-02
1.819218498	-0.003	0.010	7.41E-01	0.002	0.015	9.05E-01
7.587355	-0.005	0.009	5.84E-01	-0.007	0.014	6.25E-01
5.885679	0.001	0.007	9.16E-01	-0.001	0.010	9.14E-01
7.459511	0.004	0.009	6.94E-01	-0.008	0.014	5.54E-01
6.92391	0.000	0.011	9.67E-01	0.004	0.016	7.85E-01
5.511566	0.001	0.010	8.97E-01	-0.021	0.015	1.43E-01
6.945105	0.000	0.009	9.69E-01	0.022	0.013	1.08E-01
6.654428	-0.001	0.010	9.54E-01	0.001	0.015	9.49E-01
5.764227	0.000	0.008	9.59E-01	-0.005	0.012	6.55E-01
4.191405	-0.003	0.014	8.07E-01	0.021	0.020	2.93E-01
8.076865	0.000	0.010	9.63E-01	0.013	0.014	3.68E-01
5.043925	0.000	0.009	9.60E-01	-0.003	0.013	7.85E-01
6.728106	-0.005	0.009	5.66E-01	0.000	0.013	9.90E-01
8.885649	0.001	0.009	9.53E-01	0.005	0.013	7.15E-01
8.339619	0.000	0.008	9.86E-01	0.003	0.013	8.15E-01
6.19116	0.003	0.009	7.75E-01	0.004	0.014	7.92E-01
8.873537	0.001	0.009	8.70E-01	-0.004	0.014	7.61E-01
7.774075	0.000	0.011	9.71E-01	-0.009	0.016	5.57E-01
8.235324	-0.001	0.008	9.28E-01	-0.028	0.012	2.15E-02
3.552183	-0.007	0.014	5.95E-01	0.059	0.021	4.68E-03
6.394702	0.001	0.009	9.33E-01	-0.009	0.013	4.80E-01
7.539918	0.006	0.010	5.66E-01	-0.033	0.015	3.26E-02
7.450427	0.001	0.008	8.93E-01	0.000	0.013	9.82E-01
1.037012723	0.001	0.005	8.16E-01	0.004	0.008	6.27E-01
6.215383	-0.001	0.008	9.26E-01	-0.018	0.011	1.21E-01
7.894518	-0.002	0.009	8.68E-01	-0.015	0.014	2.70E-01
5.817383	-0.003	0.008	7.29E-01	-0.018	0.012	1.28E-01
7.521078	0.003	0.006	6.01E-01	-0.009	0.009	3.48E-01
8.064753	0.002	0.007	8.21E-01	0.004	0.011	6.83E-01
7.145283	0.001	0.010	9.45E-01	-0.016	0.014	2.69E-01
8.577476	-0.004	0.007	5.33E-01	-0.003	0.011	7.68E-01
8.299583	-0.001	0.008	8.94E-01	0.000	0.012	9.84E-01
7.225017	-0.008	0.010	4.13E-01	0.005	0.015	7.31E-01
4.291662	0.005	0.011	6.87E-01	0.014	0.017	4.20E-01
6.867053	0.003	0.008	6.55E-01	0.009	0.012	4.14E-01
5.489698	0.001	0.009	9.18E-01	-0.007	0.014	6.23E-01
8.877574	-0.003	0.008	7.42E-01	0.004	0.012	7.24E-01
6.299828	-0.001	0.009	9.31E-01	-0.022	0.014	1.19E-01
2.806984585	0.003	0.015	8.17E-01	-0.070	0.022	1.44E-03
2.8153954	-0.005	0.015	7.21E-01	-0.065	0.023	4.80E-03
8.904489	-0.003	0.008	7.00E-01	0.011	0.012	3.84E-01

2.175164179	0.005	0.011	6.65E-01	-0.018	0.016	2.66E-01
5.569433	0.002	0.008	8.01E-01	-0.008	0.012	5.14E-01
7.903938	-0.005	0.011	6.56E-01	-0.009	0.016	5.80E-01
6.055241	0.003	0.008	7.51E-01	-0.006	0.012	6.16E-01
5.436542	0.001	0.007	8.47E-01	-0.002	0.011	8.72E-01
7.129471	0.004	0.011	7.02E-01	-0.001	0.017	9.47E-01
3.332492	0.001	0.010	9.20E-01	0.006	0.015	6.99E-01
6.667549	0.001	0.011	9.60E-01	-0.038	0.016	1.66E-02
8.616839	-0.003	0.008	7.08E-01	-0.015	0.012	2.17E-01
6.279642	0.001	0.006	9.35E-01	-0.001	0.009	8.83E-01
5.243093	-0.005	0.004	2.87E-01	-0.003	0.007	6.39E-01
3.796096	0.003	0.005	6.28E-01	0.014	0.008	7.08E-02
2.069524345	0.000	0.004	9.15E-01	0.011	0.006	9.16E-02
0.510832151	-0.001	0.009	8.80E-01	-0.008	0.014	5.67E-01
6.509762	0.002	0.008	7.69E-01	0.003	0.012	7.76E-01
5.542854	-0.002	0.009	7.96E-01	-0.031	0.014	2.57E-02
8.555945	0.003	0.009	7.43E-01	-0.001	0.013	9.09E-01
1.752941277	0.000	0.007	9.80E-01	0.006	0.010	5.48E-01
3.301204	0.006	0.012	6.25E-01	0.028	0.018	1.27E-01
7.981318	-0.002	0.007	7.58E-01	0.007	0.011	4.90E-01
7.84439	0.002	0.009	7.84E-01	0.003	0.013	8.20E-01
8.458043	0.002	0.009	8.10E-01	-0.009	0.013	4.96E-01
7.290285	0.000	0.010	9.95E-01	0.005	0.015	7.22E-01
6.083838	0.003	0.009	7.14E-01	-0.002	0.013	8.63E-01
0.792762662	-0.003	0.005	5.21E-01	0.000	0.008	9.93E-01
7.35589	-0.003	0.010	7.71E-01	-0.009	0.015	5.63E-01
6.298482	-0.005	0.009	5.66E-01	0.011	0.013	3.91E-01
7.73404	0.000	0.011	9.91E-01	-0.016	0.017	3.32E-01
7.146292	0.000	0.008	9.84E-01	0.004	0.011	6.94E-01
8.278724	-0.003	0.007	6.64E-01	-0.008	0.011	4.82E-01
6.036737	-0.004	0.010	7.10E-01	-0.009	0.014	5.33E-01
6.247008	0.002	0.009	8.55E-01	0.002	0.014	8.66E-01
6.044475	0.002	0.006	7.74E-01	-0.005	0.009	5.61E-01
6.547779	0.004	0.009	7.01E-01	-0.008	0.014	5.66E-01
5.885343	-0.003	0.007	7.11E-01	-0.001	0.011	9.51E-01
6.435747	0.003	0.008	7.20E-01	-0.006	0.012	6.05E-01
8.205046	0.003	0.007	6.16E-01	0.012	0.010	2.43E-01
1.814172009	0.000	0.011	9.84E-01	0.009	0.017	5.91E-01
5.90822	-0.002	0.010	8.15E-01	-0.034	0.015	1.93E-02
8.04995	-0.002	0.008	8.06E-01	-0.001	0.011	9.38E-01
1.759333496	-0.001	0.008	8.89E-01	0.019	0.013	1.21E-01
4.188377	-0.003	0.014	8.10E-01	0.021	0.021	3.24E-01
8.545179	0.001	0.009	8.76E-01	-0.007	0.014	6.15E-01

6.054232	-0.004	0.009	6.51E-01	-0.006	0.013	6.52E-01
1.443423293	0.002	0.004	6.29E-01	0.008	0.006	1.83E-01
7.468931	0.001	0.008	9.02E-01	-0.009	0.012	4.51E-01
8.151553	-0.003	0.009	7.33E-01	0.001	0.013	9.67E-01
5.786095	0.002	0.007	8.33E-01	-0.005	0.011	6.33E-01
5.662624	0.002	0.008	8.29E-01	0.000	0.012	9.82E-01
7.689294	-0.007	0.009	4.61E-01	0.005	0.014	6.97E-01
4.126137	-0.001	0.008	9.40E-01	0.013	0.012	2.80E-01
0.999332273	-0.001	0.006	8.32E-01	0.006	0.009	5.31E-01
3.275972	-0.005	0.011	6.40E-01	0.016	0.017	3.63E-01
7.495173	0.004	0.010	6.69E-01	0.014	0.015	3.63E-01
7.114667	-0.001	0.010	9.21E-01	-0.002	0.015	8.89E-01
6.824326	-0.002	0.010	8.61E-01	0.011	0.014	4.38E-01
7.631428	-0.003	0.011	7.66E-01	-0.010	0.017	5.61E-01
5.839252	-0.003	0.008	6.71E-01	0.001	0.012	9.23E-01
1.638217764	0.002	0.006	7.01E-01	-0.008	0.009	3.47E-01
1.829984341	-0.003	0.011	7.77E-01	-0.005	0.016	7.43E-01
4.143631	-0.003	0.012	8.08E-01	0.011	0.019	5.68E-01
8.620876	-0.003	0.007	6.71E-01	0.007	0.011	4.89E-01
7.943974	-0.003	0.009	6.97E-01	0.000	0.013	9.89E-01
6.567965	-0.003	0.008	6.98E-01	-0.019	0.012	1.04E-01
8.290499	-0.002	0.010	8.66E-01	-0.019	0.015	2.08E-01
1.627115488	0.002	0.006	7.38E-01	-0.005	0.008	5.70E-01
4.030926	0.006	0.015	6.61E-01	0.006	0.022	7.96E-01
6.591178	-0.003	0.007	6.46E-01	-0.007	0.011	5.15E-01
5.651186	0.004	0.008	6.06E-01	-0.011	0.011	3.59E-01
3.633263	-0.001	0.004	8.32E-01	0.010	0.006	7.20E-02
8.915928	0.000	0.009	1.00E+00	-0.039	0.013	2.71E-03
7.235783	-0.002	0.011	8.81E-01	0.005	0.016	7.46E-01
5.503828	-0.003	0.007	6.75E-01	-0.021	0.011	5.80E-02
3.750005	0.002	0.010	8.55E-01	0.043	0.015	3.66E-03
4.021506	-0.004	0.013	7.89E-01	0.010	0.020	6.19E-01
3.310288	-0.006	0.014	6.72E-01	0.009	0.021	6.46E-01
8.675715	0.001	0.007	8.31E-01	-0.014	0.010	1.50E-01
8.047931	-0.003	0.006	5.73E-01	0.015	0.009	9.03E-02
2.537838512	0.004	0.013	7.59E-01	0.010	0.019	6.11E-01
3.798788	0.004	0.006	4.99E-01	0.013	0.009	1.29E-01
8.190579	-0.005	0.010	6.04E-01	-0.003	0.014	8.41E-01
8.514563	-0.003	0.008	6.99E-01	-0.002	0.012	8.85E-01
5.96104	0.002	0.010	8.31E-01	0.018	0.015	2.52E-01
6.609682	-0.003	0.010	8.08E-01	0.001	0.015	9.51E-01
5.643784	0.002	0.007	7.55E-01	-0.010	0.010	3.10E-01
8.32246	-0.006	0.009	4.76E-01	0.022	0.013	9.54E-02

5.637392	0.003	0.009	7.46E-01	-0.004	0.013	7.50E-01
3.74294	-0.003	0.009	7.27E-01	0.004	0.013	7.82E-01
6.336499	0.002	0.009	7.69E-01	-0.031	0.013	1.41E-02
0.762483729	0.000	0.005	9.93E-01	-0.004	0.008	5.72E-01
0.559951309	0.002	0.008	8.20E-01	-0.004	0.012	7.42E-01
4.201161	-0.002	0.011	8.91E-01	0.015	0.017	3.65E-01
2.06750575	0.003	0.004	4.74E-01	0.012	0.006	4.06E-02
6.42599	0.003	0.008	6.60E-01	0.003	0.011	8.20E-01
6.706911	0.003	0.008	7.07E-01	-0.011	0.013	3.98E-01
7.081361	-0.003	0.008	7.23E-01	-0.012	0.013	3.46E-01
6.759058	0.005	0.008	5.04E-01	0.012	0.012	3.33E-01
7.480033	0.001	0.009	9.21E-01	-0.011	0.014	4.57E-01
0.604696844	-0.004	0.008	6.11E-01	-0.006	0.012	6.44E-01
7.333349	0.001	0.006	8.29E-01	-0.003	0.009	6.96E-01
7.108275	-0.001	0.010	8.94E-01	0.020	0.014	1.58E-01
4.239515	0.005	0.012	6.92E-01	-0.009	0.018	6.01E-01
2.741716663	0.002	0.011	8.85E-01	-0.016	0.016	3.07E-01
0.541447517	0.005	0.007	4.73E-01	0.009	0.011	4.16E-01
0.958287497	-0.002	0.005	6.90E-01	0.009	0.007	2.10E-01
8.266613	0.006	0.009	5.11E-01	-0.028	0.013	2.75E-02
7.234101	-0.001	0.011	9.19E-01	0.013	0.016	4.24E-01
8.079893	0.000	0.009	9.84E-01	-0.006	0.013	6.70E-01
3.89198	-0.001	0.005	7.90E-01	0.018	0.007	8.77E-03
0.501412038	0.003	0.008	7.01E-01	-0.007	0.013	5.60E-01
4.221684	-0.003	0.014	8.01E-01	0.008	0.020	7.11E-01
6.884884	0.007	0.009	4.37E-01	0.010	0.013	4.25E-01
0.580137264	0.003	0.009	7.46E-01	-0.005	0.014	6.95E-01
5.65926	0.003	0.010	7.74E-01	-0.013	0.015	3.91E-01
6.903051	0.004	0.009	6.58E-01	0.019	0.014	1.59E-01
4.012759	-0.002	0.012	8.80E-01	0.002	0.018	8.97E-01
2.741043797	0.002	0.011	8.69E-01	-0.017	0.016	3.02E-01
7.322246	-0.004	0.006	5.27E-01	-0.006	0.009	4.84E-01
7.504929	0.002	0.010	8.44E-01	-0.005	0.014	7.01E-01
1.786584536	-0.001	0.008	8.99E-01	0.004	0.011	7.51E-01
8.766215	0.004	0.009	6.77E-01	-0.007	0.013	5.90E-01
4.017805	-0.005	0.014	7.29E-01	0.017	0.021	4.17E-01
0.976454857	0.001	0.006	8.63E-01	0.016	0.010	9.51E-02
3.264533	0.001	0.008	9.09E-01	0.008	0.012	5.05E-01
8.278051	-0.002	0.009	8.43E-01	-0.023	0.013	8.27E-02
8.282761	0.002	0.008	7.61E-01	-0.009	0.011	4.56E-01
6.782609	-0.001	0.008	8.56E-01	0.010	0.012	3.71E-01
6.776889	0.000	0.007	9.68E-01	-0.016	0.010	1.22E-01
2.359865672	0.005	0.009	5.95E-01	0.016	0.013	2.10E-01

6.491931	0.001	0.009	8.76E-01	-0.006	0.013	6.48E-01
8.140787	0.006	0.008	4.34E-01	0.013	0.011	2.64E-01
6.083165	0.004	0.008	6.48E-01	0.014	0.012	2.48E-01
8.674033	0.006	0.010	5.52E-01	-0.012	0.014	3.97E-01
7.028541	-0.001	0.009	9.14E-01	-0.008	0.013	5.68E-01
0.640695131	-0.002	0.009	7.86E-01	-0.011	0.014	4.32E-01
5.959694	0.004	0.008	5.63E-01	0.004	0.012	7.00E-01
5.423084	0.002	0.006	7.42E-01	-0.007	0.010	4.41E-01
2.823469782	-0.004	0.014	7.68E-01	-0.055	0.021	8.40E-03
1.8636276	-0.001	0.008	8.74E-01	0.007	0.012	5.51E-01
5.659597	0.002	0.007	7.74E-01	-0.006	0.011	6.03E-01
0.508813555	-0.002	0.008	7.71E-01	-0.008	0.013	5.03E-01
8.342983	0.003	0.011	7.68E-01	0.014	0.016	3.80E-01
8.430792	0.001	0.007	8.42E-01	0.024	0.010	2.10E-02
0.617817715	0.001	0.006	8.45E-01	0.011	0.010	2.57E-01
4.186022	-0.002	0.014	8.83E-01	0.006	0.021	7.54E-01
0.525298752	0.000	0.009	9.85E-01	-0.017	0.014	2.19E-01
3.188836	-0.002	0.006	7.60E-01	0.011	0.009	2.28E-01
3.437459	-0.001	0.010	9.04E-01	0.014	0.015	3.47E-01
8.07451	0.003	0.009	7.09E-01	-0.006	0.014	6.52E-01
6.877146	0.005	0.009	5.98E-01	0.012	0.013	3.62E-01
5.121641	0.002	0.010	8.08E-01	-0.005	0.014	7.21E-01
2.736333741	0.001	0.010	8.95E-01	-0.014	0.015	3.65E-01
2.037226817	0.002	0.004	6.07E-01	0.010	0.006	1.17E-01
3.938407	0.004	0.007	5.43E-01	0.012	0.010	2.23E-01
7.127452	0.003	0.008	6.72E-01	0.000	0.011	9.75E-01
7.913695	0.002	0.011	8.50E-01	-0.019	0.016	2.45E-01
4.196451	-0.002	0.011	8.46E-01	0.016	0.016	3.15E-01
8.440548	0.001	0.008	8.90E-01	-0.002	0.012	8.76E-01
8.01698	0.003	0.007	6.86E-01	-0.009	0.010	3.57E-01
5.551602	-0.005	0.010	6.17E-01	-0.025	0.015	8.54E-02
8.412288	-0.003	0.007	7.20E-01	-0.003	0.011	7.48E-01
8.441558	-0.001	0.009	9.30E-01	-0.022	0.014	1.08E-01
3.576406	0.005	0.008	4.93E-01	0.017	0.012	1.38E-01
6.14877	0.005	0.009	5.32E-01	0.001	0.013	9.10E-01
8.023708	-0.003	0.008	6.92E-01	-0.002	0.012	8.72E-01
5.642775	0.003	0.007	6.28E-01	-0.004	0.010	6.81E-01
6.923574	-0.005	0.011	6.58E-01	0.000	0.017	9.96E-01
4.113352	0.002	0.010	8.49E-01	0.014	0.015	3.33E-01
5.653877	0.004	0.008	6.33E-01	-0.010	0.012	4.04E-01
8.5149	-0.002	0.009	7.94E-01	-0.004	0.013	7.76E-01
2.738352337	0.002	0.011	8.76E-01	-0.019	0.016	2.51E-01
5.748415	-0.006	0.010	5.62E-01	0.007	0.015	6.33E-01

6.067016	-0.001	0.007	9.28E-01	-0.002	0.010	8.21E-01
6.98716	0.002	0.008	8.35E-01	0.013	0.012	2.81E-01
3.438469	0.003	0.008	6.84E-01	0.019	0.012	1.17E-01
5.986273	0.005	0.009	5.90E-01	-0.025	0.014	7.32E-02
6.955198	-0.001	0.007	8.75E-01	-0.002	0.010	8.26E-01
2.815058967	-0.006	0.016	7.21E-01	-0.074	0.023	1.47E-03
5.145864	0.002	0.009	8.29E-01	-0.016	0.014	2.53E-01
6.876137	0.008	0.010	4.47E-01	0.020	0.015	1.70E-01
8.712386	0.002	0.009	8.14E-01	0.007	0.014	6.11E-01
7.125433	-0.001	0.008	8.98E-01	-0.018	0.011	1.12E-01
0.749362858	0.001	0.006	8.64E-01	-0.009	0.008	2.89E-01
1.867328358	-0.001	0.009	9.06E-01	-0.009	0.013	5.13E-01
0.779641791	0.001	0.004	8.61E-01	0.002	0.007	7.78E-01
6.007468	-0.003	0.010	7.37E-01	-0.009	0.014	5.34E-01
8.847295	-0.003	0.006	6.63E-01	0.004	0.010	6.45E-01
3.788695	0.011	0.012	3.87E-01	0.030	0.019	1.02E-01
4.01175	0.004	0.013	7.39E-01	0.019	0.019	3.23E-01
6.172656	-0.001	0.006	8.89E-01	-0.012	0.009	1.82E-01
8.881948	-0.004	0.010	6.77E-01	-0.001	0.015	9.52E-01
0.567016393	0.003	0.008	7.26E-01	-0.017	0.013	1.78E-01
0.74969929	-0.002	0.006	6.99E-01	-0.007	0.008	3.96E-01
8.843595	-0.001	0.008	9.40E-01	0.000	0.012	9.98E-01
5.532761	0.002	0.007	8.06E-01	-0.003	0.011	7.89E-01
2.810012479	-0.004	0.014	7.76E-01	-0.068	0.021	1.59E-03
5.478259	-0.001	0.007	8.52E-01	-0.013	0.010	1.93E-01
7.400635	-0.002	0.008	8.14E-01	0.008	0.012	5.16E-01
7.440334	0.000	0.009	9.70E-01	-0.008	0.013	5.51E-01
7.397271	0.000	0.007	9.83E-01	-0.006	0.010	5.77E-01
6.474436	0.002	0.008	7.71E-01	0.000	0.011	9.80E-01
8.468136	0.004	0.010	6.95E-01	-0.007	0.015	6.61E-01
6.107725	0.003	0.009	7.24E-01	-0.009	0.013	4.86E-01
7.720583	-0.006	0.012	6.35E-01	-0.016	0.018	3.84E-01
2.809339613	-0.005	0.014	7.36E-01	-0.068	0.021	1.46E-03
6.055578	0.003	0.007	6.68E-01	-0.009	0.010	3.97E-01
0.517897235	0.005	0.009	5.94E-01	0.010	0.013	4.13E-01
3.998292	0.001	0.009	9.14E-01	0.008	0.013	5.46E-01
3.193209	0.002	0.007	7.87E-01	0.016	0.010	1.09E-01
0.622864204	-0.001	0.008	8.73E-01	0.006	0.012	6.34E-01
5.78374	0.001	0.010	9.19E-01	-0.019	0.015	2.30E-01
3.737557	-0.002	0.009	8.06E-01	0.039	0.013	3.74E-03
1.701803523	-0.001	0.004	8.70E-01	0.005	0.006	4.61E-01
1.681954	0.001	0.004	7.18E-01	0.010	0.006	7.76E-02
6.404122	-0.001	0.008	8.63E-01	-0.002	0.011	8.28E-01

3.898708	-0.002	0.005	7.28E-01	0.017	0.007	1.77E-02
8.368552	0.006	0.009	5.02E-01	0.007	0.014	6.06E-01
3.317689	-0.005	0.013	6.74E-01	0.008	0.019	6.77E-01
4.361303	-0.004	0.014	7.78E-01	0.004	0.021	8.58E-01
7.242512	-0.004	0.010	7.06E-01	-0.010	0.015	4.89E-01
0.825396623	-0.004	0.007	5.61E-01	0.005	0.011	6.19E-01
7.556403	-0.002	0.007	7.56E-01	-0.005	0.010	6.30E-01
8.917273	-0.004	0.009	6.41E-01	-0.017	0.014	2.14E-01
4.032609	-0.002	0.014	8.62E-01	-0.007	0.021	7.51E-01
2.082645217	-0.001	0.007	9.29E-01	0.012	0.011	2.77E-01
0.973090531	0.001	0.005	8.69E-01	0.018	0.007	1.41E-02
8.868491	-0.001	0.009	9.13E-01	0.002	0.014	9.05E-01
7.16345	0.005	0.009	6.13E-01	-0.007	0.014	6.24E-01
8.156936	0.001	0.007	9.05E-01	0.014	0.011	1.70E-01
2.784780034	-0.004	0.012	7.54E-01	-0.043	0.018	1.74E-02
6.983459	-0.002	0.010	8.33E-01	-0.018	0.015	2.44E-01
6.998935	-0.001	0.008	8.76E-01	-0.003	0.011	7.96E-01
7.34546	0.001	0.011	9.18E-01	-0.005	0.016	7.72E-01
1.467310007	0.000	0.007	9.70E-01	-0.001	0.011	9.37E-01
7.151675	-0.003	0.007	6.47E-01	-0.014	0.011	1.82E-01
8.334572	0.002	0.008	8.47E-01	-0.017	0.012	1.59E-01
5.95162	-0.003	0.008	7.05E-01	-0.021	0.012	8.90E-02
7.871304	-0.004	0.010	7.13E-01	-0.028	0.015	6.50E-02
5.438897	-0.002	0.006	7.62E-01	-0.011	0.009	2.03E-01
8.498078	-0.007	0.009	4.68E-01	0.013	0.014	3.48E-01
7.236793	-0.002	0.012	8.90E-01	-0.011	0.018	5.24E-01
7.653969	0.000	0.009	9.84E-01	0.001	0.014	9.20E-01
6.911462	0.004	0.007	5.79E-01	0.002	0.010	8.19E-01
0.903448985	0.003	0.006	6.30E-01	-0.019	0.010	4.78E-02
8.084266	-0.001	0.010	8.88E-01	0.019	0.014	1.91E-01
0.79511769	-0.004	0.006	5.09E-01	0.002	0.008	8.24E-01
8.996335	0.000	0.009	9.77E-01	0.001	0.014	9.63E-01
6.271904	0.001	0.008	9.17E-01	-0.002	0.012	8.67E-01
5.689539	-0.004	0.006	5.32E-01	-0.014	0.009	1.30E-01
7.763982	-0.002	0.012	8.73E-01	-0.027	0.018	1.47E-01
3.555211	-0.004	0.025	8.72E-01	-0.003	0.038	9.30E-01
7.417457	0.002	0.008	7.62E-01	0.000	0.012	9.97E-01
7.387178	0.001	0.009	9.06E-01	-0.018	0.014	2.07E-01
0.586193051	0.005	0.010	6.05E-01	-0.005	0.015	7.37E-01
6.158862	0.000	0.006	9.57E-01	0.019	0.009	3.20E-02
6.875464	-0.002	0.008	8.15E-01	-0.001	0.013	9.63E-01
8.023372	-0.002	0.009	8.16E-01	-0.012	0.013	3.41E-01
2.278448985	-0.003	0.006	6.87E-01	-0.003	0.009	7.42E-01

8.190915	0.000	0.009	9.82E-01	0.001	0.014	9.29E-01
1.030620504	0.000	0.007	9.83E-01	0.020	0.010	6.03E-02
3.596592	0.002	0.007	7.93E-01	0.020	0.010	3.80E-02
7.898555	0.003	0.009	7.54E-01	0.000	0.014	9.77E-01
2.826161243	-0.003	0.013	8.34E-01	-0.046	0.019	1.63E-02
6.291753	-0.003	0.008	6.92E-01	0.013	0.012	2.66E-01
0.719420357	-0.003	0.008	6.88E-01	-0.002	0.011	8.55E-01
0.683758503	0.007	0.013	6.18E-01	-0.010	0.020	6.00E-01
8.710704	0.003	0.008	6.99E-01	0.019	0.012	1.14E-01
4.047748	0.000	0.010	9.90E-01	-0.017	0.015	2.59E-01
6.396384	0.000	0.008	9.96E-01	0.003	0.011	7.94E-01
8.621885	-0.003	0.007	6.47E-01	0.004	0.011	7.17E-01
6.829709	0.000	0.009	9.84E-01	0.010	0.013	4.47E-01
6.648035	-0.004	0.009	6.31E-01	0.000	0.013	9.76E-01
6.441466	-0.003	0.010	7.49E-01	-0.015	0.014	3.01E-01
2.278785417	-0.002	0.006	6.91E-01	-0.004	0.009	6.26E-01
0.522607291	0.000	0.009	9.58E-01	-0.004	0.013	7.83E-01
8.271659	-0.005	0.009	6.09E-01	0.008	0.014	5.64E-01
4.026889	0.003	0.014	8.16E-01	0.005	0.020	7.95E-01
6.13834	-0.004	0.008	5.70E-01	-0.013	0.012	2.76E-01
7.281202	-0.006	0.011	5.60E-01	0.003	0.016	8.40E-01
1.86631906	0.003	0.009	6.97E-01	-0.010	0.013	4.35E-01
8.032456	-0.004	0.009	6.16E-01	-0.019	0.013	1.66E-01
7.988383	0.002	0.008	8.32E-01	-0.020	0.013	1.14E-01
5.606104	-0.002	0.006	7.09E-01	0.001	0.010	9.20E-01
4.015114	-0.004	0.012	7.46E-01	0.025	0.018	1.73E-01
7.17859	0.005	0.010	5.93E-01	0.015	0.015	3.26E-01
6.914154	-0.003	0.009	7.27E-01	-0.006	0.013	6.80E-01
1.772117935	0.003	0.009	7.39E-01	0.038	0.013	2.57E-03
6.389655	-0.003	0.008	6.84E-01	-0.005	0.012	6.51E-01
5.461101	-0.003	0.010	7.82E-01	-0.007	0.015	6.30E-01
8.23667	0.002	0.010	8.67E-01	-0.022	0.015	1.51E-01
8.903816	-0.005	0.008	5.91E-01	0.025	0.013	4.61E-02
8.633661	0.000	0.010	9.96E-01	0.001	0.014	9.67E-01
6.082829	0.003	0.007	6.53E-01	0.016	0.011	1.35E-01
8.196635	0.004	0.006	5.07E-01	-0.010	0.009	2.91E-01
2.586284805	0.005	0.009	5.78E-01	0.010	0.014	4.60E-01
4.118062	-0.002	0.012	8.65E-01	0.024	0.018	1.66E-01
7.99713	-0.004	0.009	6.85E-01	-0.009	0.014	5.08E-01
8.933422	0.003	0.009	7.19E-01	0.006	0.013	6.24E-01
7.153021	-0.003	0.007	6.65E-01	-0.004	0.011	6.88E-01
2.331268901	0.002	0.006	7.01E-01	-0.008	0.008	3.50E-01
2.522026181	-0.004	0.012	7.55E-01	0.013	0.018	4.77E-01

2.067842183	0.003	0.004	4.54E-01	0.013	0.006	2.99E-02
8.742665	0.001	0.009	9.24E-01	-0.006	0.013	6.52E-01
6.666203	-0.003	0.009	7.62E-01	-0.006	0.013	6.58E-01
7.917059	0.000	0.009	9.64E-01	-0.028	0.013	2.69E-02
1.739483974	-0.001	0.006	8.72E-01	0.015	0.009	1.23E-01
7.670791	0.001	0.008	8.80E-01	-0.008	0.012	5.14E-01
8.877911	0.005	0.009	5.98E-01	0.006	0.014	6.57E-01
8.266949	0.003	0.009	6.92E-01	-0.030	0.013	1.77E-02
6.542396	-0.001	0.009	9.52E-01	-0.005	0.013	6.96E-01
4.094512	0.006	0.011	5.85E-01	0.010	0.017	5.49E-01
6.60329	0.003	0.008	7.32E-01	0.003	0.012	7.78E-01
6.939723	-0.002	0.010	8.24E-01	0.028	0.014	4.71E-02
1.814508441	-0.001	0.011	9.08E-01	-0.001	0.016	9.61E-01
6.955535	-0.002	0.007	8.00E-01	-0.001	0.010	9.06E-01
3.858336	0.000	0.006	9.47E-01	0.011	0.008	2.00E-01
7.5416	-0.007	0.009	4.30E-01	-0.007	0.014	6.02E-01
7.077996	0.002	0.008	7.85E-01	0.017	0.013	1.88E-01
8.874883	0.002	0.010	8.64E-01	-0.029	0.015	5.21E-02
8.97413	-0.003	0.008	6.84E-01	0.007	0.011	5.45E-01
2.74138023	0.002	0.011	8.82E-01	-0.017	0.016	2.85E-01
2.813376805	0.005	0.015	7.34E-01	-0.063	0.023	6.61E-03
0.728167605	-0.005	0.007	4.67E-01	-0.004	0.011	6.89E-01
5.913267	0.001	0.007	8.44E-01	0.012	0.011	2.71E-01
6.100996	-0.002	0.009	8.16E-01	-0.015	0.013	2.53E-01
6.208991	-0.002	0.008	8.47E-01	0.002	0.012	8.72E-01
0.795454123	-0.004	0.006	5.21E-01	0.000	0.008	9.88E-01
6.832737	0.001	0.009	9.05E-01	0.002	0.013	9.07E-01
7.817812	0.004	0.009	7.06E-01	0.018	0.014	1.99E-01
2.649870565	-0.006	0.019	7.65E-01	0.011	0.029	7.12E-01
3.181098	0.003	0.005	6.07E-01	0.011	0.008	1.41E-01
6.168283	0.001	0.010	9.14E-01	0.010	0.015	4.78E-01
7.902256	-0.003	0.010	7.49E-01	-0.019	0.015	2.13E-01
8.042549	-0.002	0.009	8.08E-01	-0.025	0.014	7.17E-02
3.93908	0.000	0.007	9.85E-01	0.011	0.010	2.96E-01
7.773739	0.005	0.012	6.57E-01	-0.013	0.018	4.71E-01
2.807321018	0.004	0.015	8.08E-01	-0.069	0.022	1.73E-03
6.490585	0.002	0.007	7.68E-01	-0.018	0.011	8.43E-02
8.483275	-0.003	0.009	7.49E-01	-0.007	0.014	6.02E-01
3.617114	-0.001	0.006	8.38E-01	0.000	0.009	9.83E-01
3.636964	-0.001	0.004	8.65E-01	0.008	0.005	1.19E-01
7.16816	0.006	0.009	5.32E-01	0.003	0.014	8.26E-01
5.680119	-0.006	0.009	5.17E-01	-0.026	0.014	5.95E-02
7.912686	-0.002	0.010	8.57E-01	-0.019	0.014	1.97E-01

1.839740886	-0.002	0.010	8.08E-01	-0.013	0.015	3.69E-01
2.539520675	-0.004	0.015	7.79E-01	-0.007	0.023	7.49E-01
0.569034989	0.006	0.009	5.07E-01	0.013	0.013	3.19E-01
7.536218	0.001	0.007	8.76E-01	-0.004	0.011	6.76E-01
5.556312	-0.002	0.010	8.49E-01	-0.024	0.016	1.25E-01
5.409291	0.003	0.007	7.12E-01	-0.013	0.011	2.42E-01
5.294904	0.003	0.011	7.67E-01	-0.026	0.017	1.23E-01
8.104789	-0.002	0.008	7.90E-01	-0.006	0.012	6.46E-01
3.30154	0.000	0.011	9.68E-01	0.020	0.017	2.51E-01
1.6520115	0.001	0.005	8.37E-01	0.000	0.007	9.76E-01
6.926602	-0.004	0.012	7.01E-01	0.009	0.017	6.05E-01
5.457064	0.003	0.010	7.99E-01	-0.020	0.016	1.92E-01
5.974834	-0.001	0.008	8.51E-01	-0.001	0.011	9.44E-01
7.577599	-0.001	0.010	9.50E-01	-0.013	0.014	3.60E-01
6.105706	-0.008	0.010	4.49E-01	-0.013	0.015	3.72E-01
7.126779	0.001	0.008	9.38E-01	0.002	0.011	8.51E-01
2.329586738	0.000	0.005	9.32E-01	-0.010	0.008	2.15E-01
8.826437	0.001	0.007	8.61E-01	-0.008	0.010	4.46E-01
3.128278	-0.005	0.009	5.80E-01	0.000	0.014	9.91E-01
1.8182092	-0.003	0.009	7.07E-01	-0.009	0.013	4.77E-01
8.766888	-0.003	0.009	7.19E-01	0.017	0.014	1.96E-01
0.72110252	-0.002	0.007	7.75E-01	0.013	0.011	2.58E-01
7.390206	0.002	0.008	7.67E-01	-0.014	0.012	2.12E-01
8.941496	-0.004	0.008	5.94E-01	-0.017	0.011	1.27E-01
3.256122	0.003	0.010	7.96E-01	0.012	0.015	4.23E-01
3.682382	-0.001	0.004	9.04E-01	0.013	0.007	4.80E-02
5.579862	0.005	0.010	5.89E-01	-0.028	0.014	5.38E-02
6.147087	0.001	0.007	8.70E-01	-0.012	0.011	3.00E-01
0.968380475	-0.002	0.005	7.58E-01	0.013	0.008	1.04E-01
6.380572	0.003	0.009	7.15E-01	0.000	0.013	9.84E-01
2.162716173	-0.004	0.011	7.29E-01	-0.020	0.017	2.41E-01
8.345674	-0.003	0.008	7.30E-01	-0.002	0.011	8.37E-01
3.631581	-0.001	0.004	7.86E-01	0.005	0.006	3.93E-01
7.60384	0.009	0.013	4.74E-01	0.008	0.019	6.78E-01
7.846072	-0.001	0.010	8.84E-01	-0.002	0.015	9.14E-01
1.77144507	0.000	0.010	9.69E-01	0.024	0.015	1.03E-01
6.194524	0.005	0.009	5.95E-01	-0.005	0.014	7.23E-01
5.830504	0.003	0.009	7.28E-01	0.010	0.013	4.22E-01
2.092401762	-0.002	0.005	7.40E-01	0.006	0.008	4.59E-01
0.513860044	0.000	0.006	9.89E-01	-0.002	0.010	8.55E-01
4.023525	0.005	0.013	6.95E-01	0.013	0.020	4.98E-01
6.294108	-0.004	0.010	7.20E-01	-0.005	0.015	7.56E-01
6.444494	-0.002	0.008	7.81E-01	-0.013	0.012	2.76E-01

1.504654025	0.001	0.006	8.41E-01	-0.014	0.008	1.11E-01
8.360814	-0.006	0.010	5.56E-01	0.000	0.015	9.96E-01
2.738015904	0.002	0.011	8.89E-01	-0.018	0.016	2.53E-01
1.666141669	0.001	0.004	8.45E-01	0.013	0.006	2.63E-02
7.089099	0.000	0.010	9.85E-01	0.000	0.015	9.82E-01
2.855431	-0.001	0.008	8.63E-01	-0.009	0.011	4.39E-01
7.847754	0.002	0.009	8.25E-01	0.001	0.013	9.56E-01
6.456605	0.000	0.008	9.66E-01	-0.011	0.012	3.88E-01
8.200672	0.001	0.008	8.55E-01	-0.010	0.012	4.21E-01
6.766796	0.004	0.008	6.07E-01	0.000	0.012	9.78E-01
3.191527	-0.001	0.007	8.55E-01	0.017	0.010	9.34E-02
8.778327	0.003	0.007	6.96E-01	-0.008	0.010	4.17E-01
1.187061659	0.002	0.008	7.85E-01	0.007	0.013	5.60E-01
3.515848	0.001	0.007	8.71E-01	0.020	0.011	6.61E-02
6.954526	0.000	0.007	9.59E-01	-0.006	0.010	5.46E-01
3.87785	-0.002	0.005	7.19E-01	0.015	0.007	2.57E-02
5.29524	0.003	0.012	7.65E-01	-0.026	0.017	1.34E-01
7.631092	-0.004	0.012	7.45E-01	0.015	0.017	3.83E-01
8.632651	-0.003	0.010	7.28E-01	0.002	0.015	8.83E-01
4.340781	-0.003	0.013	8.28E-01	0.016	0.020	4.27E-01
8.557963	0.000	0.007	9.60E-01	0.000	0.010	9.64E-01
1.002696599	0.004	0.008	6.51E-01	-0.006	0.013	6.34E-01
6.775207	-0.002	0.010	8.37E-01	-0.009	0.015	5.44E-01
3.999638	0.002	0.010	8.50E-01	0.011	0.015	4.87E-01
1.681281135	-0.001	0.004	7.32E-01	0.005	0.006	4.16E-01
0.543129679	-0.002	0.007	8.28E-01	0.003	0.011	7.62E-01
1.853871055	0.002	0.008	8.30E-01	-0.007	0.012	5.49E-01
2.742389528	0.001	0.011	9.08E-01	-0.017	0.016	2.92E-01
5.902164	-0.007	0.010	5.25E-01	-0.017	0.016	2.84E-01
8.378981	-0.001	0.009	8.92E-01	-0.029	0.013	2.73E-02
7.259333	-0.006	0.011	6.17E-01	0.006	0.016	6.96E-01
0.598641057	-0.003	0.009	6.96E-01	0.009	0.013	4.80E-01
4.198806	-0.001	0.012	9.33E-01	0.022	0.018	2.34E-01
3.331483	0.002	0.010	8.51E-01	0.011	0.015	4.61E-01
1.198163934	0.001	0.009	8.81E-01	0.014	0.014	3.12E-01
7.246885	-0.005	0.009	5.63E-01	-0.038	0.014	5.92E-03
3.634945	-0.001	0.004	8.21E-01	0.008	0.006	1.93E-01
8.753094	0.000	0.010	9.65E-01	0.007	0.014	6.30E-01
7.069922	-0.002	0.010	8.64E-01	-0.005	0.016	7.58E-01
6.509425	-0.002	0.009	8.62E-01	0.008	0.013	5.50E-01
7.555058	-0.003	0.008	7.54E-01	0.009	0.012	4.64E-01
5.112557	-0.003	0.008	7.31E-01	-0.011	0.012	3.39E-01
8.089986	-0.004	0.009	6.79E-01	-0.024	0.013	6.50E-02

5.615187	-0.005	0.008	5.62E-01	-0.021	0.012	6.76E-02
7.92076	0.001	0.008	9.41E-01	-0.016	0.012	2.03E-01
3.319035	0.002	0.011	8.34E-01	0.021	0.017	2.21E-01
1.740156839	-0.003	0.007	6.82E-01	0.016	0.010	1.01E-01
8.359468	-0.004	0.010	6.76E-01	-0.016	0.015	2.83E-01
7.026522	-0.009	0.010	3.41E-01	-0.027	0.014	5.99E-02
6.74661	0.002	0.009	8.26E-01	-0.009	0.014	5.32E-01
6.981104	-0.001	0.010	9.45E-01	-0.005	0.015	7.23E-01
1.50532689	0.001	0.006	8.28E-01	-0.012	0.009	1.69E-01
5.757498	0.003	0.009	7.18E-01	-0.003	0.013	7.94E-01
0.797809151	-0.003	0.006	5.56E-01	0.001	0.009	8.67E-01
7.568851	0.005	0.009	5.50E-01	-0.020	0.013	1.24E-01
8.891032	0.002	0.007	7.47E-01	0.005	0.011	6.50E-01
2.066496452	0.003	0.004	4.95E-01	0.011	0.006	4.98E-02
6.183086	0.001	0.006	8.68E-01	-0.006	0.009	5.15E-01
4.196115	-0.001	0.010	9.06E-01	0.025	0.016	1.10E-01
8.276706	-0.002	0.007	7.88E-01	0.023	0.011	3.10E-02
7.531507	0.000	0.010	9.74E-01	0.000	0.015	9.92E-01
3.209022	-0.005	0.012	6.89E-01	0.008	0.019	6.72E-01
0.855002691	-0.002	0.007	7.93E-01	-0.010	0.011	3.46E-01
8.905162	-0.005	0.010	5.87E-01	-0.013	0.015	3.79E-01
7.936909	-0.001	0.009	8.79E-01	0.002	0.014	8.91E-01
6.830046	0.002	0.010	8.08E-01	-0.002	0.014	8.86E-01
7.258324	-0.004	0.010	6.84E-01	-0.014	0.015	3.27E-01
8.737618	-0.002	0.008	7.62E-01	-0.006	0.012	6.30E-01
4.026216	0.005	0.013	7.03E-01	0.011	0.019	5.47E-01
2.332951064	0.003	0.006	6.39E-01	-0.004	0.009	6.03E-01
6.021934	0.001	0.009	9.47E-01	-0.003	0.014	8.46E-01
6.506734	0.001	0.010	9.26E-01	-0.026	0.014	6.63E-02
5.725201	0.000	0.009	9.65E-01	-0.011	0.014	4.21E-01
5.68382	0.003	0.010	7.70E-01	-0.015	0.015	3.18E-01
8.558636	-0.003	0.007	6.07E-01	-0.010	0.010	3.31E-01
0.620845608	0.000	0.009	9.64E-01	-0.028	0.014	4.99E-02
4.037655	0.005	0.015	7.34E-01	-0.014	0.022	5.40E-01
6.256092	-0.001	0.010	9.32E-01	-0.016	0.015	2.69E-01
8.509181	0.001	0.008	9.12E-01	-0.009	0.012	4.82E-01
6.345246	-0.001	0.009	8.75E-01	0.008	0.013	5.54E-01
8.58656	0.005	0.009	6.11E-01	0.011	0.014	4.16E-01
1.131886714	0.004	0.008	6.42E-01	-0.011	0.011	3.11E-01
0.90412185	0.003	0.006	6.77E-01	-0.018	0.009	5.10E-02
4.039337	-0.001	0.015	9.47E-01	-0.018	0.022	4.12E-01
4.013095	-0.001	0.012	9.08E-01	-0.003	0.018	8.59E-01
5.556985	-0.001	0.009	8.90E-01	-0.019	0.013	1.42E-01

8.541815	0.001	0.009	9.53E-01	0.009	0.014	5.01E-01
8.683789	0.002	0.009	8.55E-01	-0.003	0.014	8.51E-01
8.002177	0.003	0.009	7.79E-01	-0.009	0.013	5.15E-01
8.649473	-0.003	0.009	7.13E-01	0.008	0.013	5.69E-01
4.035636	0.005	0.014	7.17E-01	-0.009	0.022	6.76E-01
0.798145584	-0.003	0.006	5.73E-01	0.001	0.009	8.93E-01
8.721806	-0.005	0.009	6.07E-01	-0.005	0.014	7.32E-01
5.011964	-0.002	0.012	8.70E-01	-0.006	0.018	7.33E-01
0.821695865	0.000	0.006	9.63E-01	0.010	0.009	2.58E-01
3.31668	-0.007	0.013	6.20E-01	0.017	0.020	3.86E-01
4.016796	0.002	0.013	8.50E-01	0.002	0.019	9.16E-01
0.904458282	0.002	0.006	6.97E-01	-0.018	0.009	5.07E-02
2.726913629	-0.004	0.009	6.44E-01	-0.012	0.013	3.60E-01
8.333226	-0.001	0.007	9.12E-01	-0.004	0.010	7.06E-01
2.64785197	-0.007	0.021	7.35E-01	0.021	0.031	5.02E-01
3.648739	0.002	0.004	6.80E-01	0.012	0.006	4.18E-02
7.297014	0.000	0.010	9.67E-01	0.005	0.015	7.10E-01
7.168497	0.000	0.011	9.72E-01	-0.006	0.016	6.90E-01
2.550286518	-0.004	0.012	7.31E-01	-0.014	0.017	4.20E-01
6.309921	0.000	0.007	9.81E-01	-0.005	0.010	6.24E-01
5.911921	-0.003	0.009	7.51E-01	-0.008	0.013	5.53E-01
7.176235	-0.003	0.010	7.39E-01	0.003	0.016	8.27E-01
2.857449	-0.001	0.008	9.12E-01	-0.010	0.011	3.94E-01
6.764778	0.001	0.008	9.16E-01	-0.002	0.012	8.39E-01
6.011841	0.005	0.008	5.21E-01	0.007	0.013	5.94E-01
8.351057	-0.004	0.010	7.07E-01	0.000	0.015	9.88E-01
8.217157	0.003	0.008	6.82E-01	-0.005	0.012	6.48E-01
2.785789332	-0.003	0.012	8.13E-01	-0.049	0.018	6.75E-03
7.001963	0.000	0.007	9.56E-01	-0.009	0.010	3.65E-01
8.792793	-0.005	0.007	4.69E-01	0.010	0.011	3.67E-01
0.761137999	0.002	0.005	6.88E-01	0.005	0.008	5.03E-01
7.909321	0.003	0.011	7.52E-01	0.000	0.016	9.97E-01
8.160636	0.003	0.007	6.57E-01	0.000	0.010	9.97E-01
5.614178	0.002	0.008	7.99E-01	0.002	0.013	8.74E-01
8.914245	0.004	0.008	6.44E-01	0.001	0.012	9.66E-01
6.303865	-0.005	0.009	6.00E-01	0.000	0.013	9.98E-01
5.771292	0.000	0.008	9.64E-01	-0.007	0.012	5.39E-01
6.30622	0.003	0.008	7.30E-01	0.020	0.012	1.03E-01
5.493735	0.000	0.010	9.99E-01	0.001	0.015	9.49E-01
7.669108	-0.004	0.008	6.69E-01	-0.025	0.012	4.56E-02
3.636291	-0.001	0.004	8.45E-01	0.010	0.005	7.77E-02
1.463272816	0.000	0.004	9.66E-01	-0.002	0.006	7.54E-01
8.584878	-0.004	0.009	6.14E-01	0.001	0.013	9.63E-01

4.199143	-0.001	0.012	9.11E-01	0.024	0.018	1.81E-01
7.928498	0.003	0.009	7.58E-01	-0.010	0.013	4.52E-01
8.695228	0.004	0.008	6.49E-01	0.012	0.012	3.10E-01
4.215291	-0.002	0.014	8.58E-01	-0.002	0.020	9.29E-01
5.564723	0.001	0.009	8.77E-01	-0.015	0.014	2.92E-01
2.551295816	0.001	0.010	9.32E-01	-0.011	0.015	4.78E-01
3.804844	0.002	0.006	7.23E-01	0.009	0.008	2.59E-01
8.537441	-0.003	0.008	6.62E-01	0.005	0.012	6.94E-01
4.36635	-0.003	0.015	8.32E-01	0.009	0.022	6.93E-01
7.270772	-0.005	0.010	6.26E-01	-0.006	0.015	6.78E-01
2.073897969	0.000	0.003	9.53E-01	0.007	0.005	1.16E-01
6.714313	-0.003	0.010	7.87E-01	-0.003	0.015	8.43E-01
4.099895	0.000	0.011	9.67E-01	0.001	0.016	9.71E-01
2.819769024	-0.004	0.016	8.21E-01	-0.073	0.024	2.76E-03
4.209572	-0.001	0.014	9.54E-01	0.022	0.021	2.82E-01
3.576069	0.004	0.007	5.53E-01	0.015	0.011	1.80E-01
5.493399	0.000	0.007	9.91E-01	-0.003	0.010	8.05E-01
8.952599	0.003	0.009	7.32E-01	-0.022	0.013	8.24E-02
6.877483	0.004	0.008	6.09E-01	0.015	0.012	2.32E-01
3.782639	-0.005	0.011	6.41E-01	0.000	0.017	9.82E-01
7.868613	-0.003	0.009	7.76E-01	-0.018	0.013	1.72E-01
2.516306827	-0.005	0.016	7.37E-01	-0.002	0.024	9.43E-01
8.115891	0.003	0.008	7.01E-01	-0.010	0.012	3.84E-01
4.133202	-0.003	0.015	8.55E-01	0.017	0.022	4.41E-01
2.748781747	-0.004	0.008	6.47E-01	-0.019	0.012	1.19E-01
8.722815	0.001	0.010	9.48E-01	-0.015	0.015	3.07E-01
7.254287	-0.003	0.011	7.62E-01	-0.004	0.016	8.28E-01
0.797472718	-0.003	0.006	5.58E-01	0.001	0.009	8.69E-01
8.48092	0.001	0.008	8.59E-01	0.008	0.012	4.94E-01
0.686786396	0.003	0.015	8.15E-01	-0.023	0.022	2.91E-01
0.505112797	-0.003	0.008	6.70E-01	-0.009	0.012	4.60E-01
6.782945	-0.002	0.007	7.53E-01	0.011	0.011	2.96E-01
5.935471	-0.002	0.008	8.16E-01	-0.024	0.011	3.35E-02
7.073623	-0.003	0.009	7.49E-01	-0.011	0.013	4.01E-01
6.449204	-0.001	0.008	8.64E-01	-0.020	0.013	1.09E-01
6.025299	0.001	0.009	8.72E-01	-0.012	0.013	3.31E-01
6.876473	0.007	0.009	4.37E-01	0.029	0.013	3.03E-02
6.983122	-0.001	0.010	9.34E-01	-0.020	0.015	1.79E-01
4.119408	0.000	0.012	9.85E-01	0.021	0.018	2.52E-01
8.793466	-0.003	0.008	7.38E-01	-0.016	0.012	1.87E-01
4.019824	-0.003	0.011	7.95E-01	0.011	0.017	5.39E-01
7.813102	0.001	0.008	8.93E-01	0.009	0.012	4.34E-01
6.032364	0.001	0.010	9.21E-01	0.026	0.014	6.75E-02

8.571757	-0.003	0.008	7.27E-01	0.002	0.012	8.48E-01
6.810196	-0.002	0.008	7.77E-01	0.001	0.012	9.33E-01
0.799827747	-0.001	0.006	8.62E-01	0.004	0.009	6.36E-01
6.076773	0.002	0.007	7.36E-01	-0.008	0.011	4.73E-01
8.258875	0.000	0.007	9.88E-01	-0.007	0.011	5.06E-01
0.790744067	-0.003	0.005	5.14E-01	-0.001	0.007	9.08E-01
3.938071	0.004	0.007	5.49E-01	0.013	0.010	1.95E-01
6.146751	0.001	0.007	8.30E-01	-0.017	0.010	9.58E-02
6.863689	-0.001	0.008	9.13E-01	0.000	0.012	9.75E-01
6.110416	-0.003	0.010	7.87E-01	-0.011	0.015	4.73E-01
1.188743822	-0.004	0.014	7.62E-01	0.000	0.021	9.98E-01
4.12109	-0.001	0.011	9.60E-01	0.017	0.017	3.21E-01
4.11167	0.000	0.010	9.69E-01	-0.005	0.015	7.58E-01
5.724864	0.001	0.010	8.88E-01	-0.004	0.014	7.86E-01
6.145405	0.005	0.010	6.05E-01	-0.010	0.014	4.81E-01
6.371151	-0.001	0.008	9.45E-01	0.007	0.012	5.43E-01
6.484529	-0.002	0.010	8.11E-01	-0.022	0.014	1.26E-01
8.283434	0.004	0.008	6.14E-01	-0.003	0.012	7.97E-01
4.090811	0.008	0.011	4.70E-01	0.011	0.017	4.90E-01
2.071542941	0.002	0.004	5.85E-01	0.012	0.005	2.79E-02
6.758385	0.001	0.008	8.78E-01	0.015	0.012	2.40E-01
7.333012	-0.002	0.006	6.94E-01	-0.003	0.009	7.06E-01
6.660147	0.005	0.009	5.87E-01	-0.011	0.014	4.07E-01
5.839588	-0.003	0.007	6.66E-01	-0.013	0.010	1.82E-01
4.287624	0.004	0.011	6.80E-01	0.011	0.016	4.97E-01
8.678743	-0.003	0.009	7.59E-01	-0.001	0.014	9.52E-01
4.287961	0.004	0.011	6.78E-01	0.015	0.016	3.56E-01
2.54019354	-0.008	0.019	6.99E-01	0.008	0.029	7.74E-01
8.262239	-0.001	0.006	8.50E-01	-0.003	0.009	7.36E-01
3.993582	-0.005	0.011	6.60E-01	0.003	0.016	8.57E-01
6.339527	-0.003	0.008	6.85E-01	0.010	0.013	4.32E-01
8.156599	-0.001	0.007	8.99E-01	0.000	0.010	9.87E-01
8.704648	0.002	0.008	8.44E-01	0.013	0.012	2.76E-01
1.188070957	-0.004	0.014	7.48E-01	0.005	0.021	8.01E-01
2.782088574	-0.004	0.012	7.67E-01	-0.045	0.018	1.31E-02
0.552213359	0.000	0.009	9.87E-01	0.006	0.014	6.65E-01
7.473641	0.005	0.007	5.42E-01	-0.021	0.011	6.46E-02
8.659566	-0.003	0.008	6.82E-01	-0.036	0.012	3.02E-03
8.093013	0.004	0.009	6.80E-01	0.002	0.013	8.95E-01
4.288297	0.004	0.011	6.78E-01	0.013	0.016	4.13E-01
5.288848	-0.003	0.009	7.21E-01	-0.025	0.014	6.50E-02
8.613475	-0.004	0.009	6.23E-01	0.007	0.013	5.80E-01
0.629256423	0.001	0.010	9.13E-01	-0.023	0.015	1.23E-01

8.293864	-0.005	0.010	6.45E-01	-0.017	0.015	2.70E-01
1.743521165	0.000	0.006	9.84E-01	0.006	0.009	5.39E-01
0.640358698	0.001	0.010	8.99E-01	0.010	0.015	5.08E-01
6.456269	0.001	0.009	8.83E-01	-0.016	0.013	2.27E-01
8.16972	-0.002	0.010	8.55E-01	-0.022	0.014	1.17E-01
8.660239	0.002	0.009	8.46E-01	-0.003	0.013	7.86E-01
6.163909	0.004	0.009	6.10E-01	-0.027	0.013	3.84E-02
5.484315	0.003	0.009	7.57E-01	-0.006	0.014	6.77E-01
7.551357	0.002	0.010	8.26E-01	0.017	0.015	2.46E-01
8.334236	0.002	0.009	8.07E-01	-0.007	0.013	5.90E-01
0.954923171	-0.002	0.005	7.60E-01	0.011	0.008	1.86E-01
1.672870321	0.001	0.004	7.97E-01	0.004	0.006	4.62E-01
7.900574	-0.001	0.009	8.99E-01	-0.008	0.014	5.80E-01
6.982449	-0.003	0.010	7.25E-01	-0.002	0.015	9.13E-01
8.204036	0.002	0.007	8.07E-01	-0.003	0.011	7.74E-01
7.866931	-0.002	0.007	7.49E-01	-0.012	0.011	2.62E-01
8.275023	0.002	0.007	8.14E-01	0.006	0.010	5.64E-01
5.819738	-0.005	0.010	5.85E-01	-0.006	0.014	6.80E-01
3.795087	0.003	0.006	6.42E-01	0.006	0.008	4.70E-01
6.785973	0.006	0.010	5.77E-01	0.000	0.015	9.85E-01
8.191252	0.001	0.008	9.18E-01	0.012	0.012	3.03E-01
3.631917	-0.001	0.004	7.85E-01	0.007	0.006	2.08E-01
5.542518	-0.002	0.008	8.01E-01	-0.009	0.012	4.80E-01
8.002513	0.001	0.008	9.03E-01	-0.002	0.012	8.39E-01
2.519671152	0.005	0.023	8.40E-01	-0.007	0.034	8.36E-01
7.685594	0.005	0.010	6.12E-01	0.002	0.015	9.04E-01
8.749393	0.004	0.009	6.56E-01	0.009	0.014	5.04E-01
5.910575	-0.003	0.008	7.46E-01	-0.006	0.012	5.88E-01
6.492267	-0.003	0.010	7.32E-01	-0.017	0.015	2.43E-01
8.302611	-0.001	0.008	9.02E-01	0.009	0.012	4.23E-01
7.655315	-0.001	0.010	8.87E-01	-0.010	0.015	4.93E-01
6.097295	-0.001	0.008	9.06E-01	0.004	0.012	7.47E-01
7.382804	0.001	0.007	8.72E-01	-0.002	0.010	8.40E-01
7.473977	0.006	0.008	4.63E-01	-0.014	0.013	2.53E-01
5.61586	0.002	0.009	8.52E-01	-0.012	0.013	3.74E-01
6.511107	-0.006	0.009	5.06E-01	-0.013	0.013	3.28E-01
6.058606	-0.003	0.009	6.90E-01	0.006	0.013	6.46E-01
0.754745779	-0.002	0.005	7.67E-01	-0.001	0.008	8.68E-01
3.320044	0.003	0.013	7.86E-01	0.001	0.019	9.69E-01
4.03059	0.005	0.015	7.19E-01	-0.003	0.022	8.96E-01
5.758171	0.002	0.008	8.07E-01	-0.001	0.012	9.56E-01
1.461590653	-0.001	0.004	8.85E-01	0.001	0.006	8.05E-01
5.53007	0.001	0.009	9.39E-01	0.001	0.013	9.59E-01

1.779519452	0.000	0.011	9.69E-01	0.007	0.016	6.81E-01
0.660544654	-0.008	0.010	4.60E-01	-0.003	0.016	8.50E-01
5.053681	0.002	0.009	8.37E-01	-0.003	0.013	8.09E-01
0.555241253	0.003	0.008	7.14E-01	-0.003	0.012	7.95E-01
4.288634	0.005	0.011	6.60E-01	0.012	0.016	4.54E-01
3.544108	-0.004	0.010	7.27E-01	-0.004	0.015	7.72E-01
2.365248593	0.009	0.014	5.19E-01	0.038	0.021	6.90E-02
8.036156	-0.004	0.009	6.60E-01	0.005	0.014	6.98E-01
6.673941	0.000	0.009	9.68E-01	-0.018	0.013	1.77E-01
8.182505	-0.006	0.009	4.90E-01	-0.013	0.013	3.09E-01
8.532731	0.002	0.006	7.25E-01	-0.013	0.009	1.56E-01
2.840627844	0.000	0.010	9.91E-01	0.003	0.015	8.32E-01
7.892836	0.004	0.009	6.63E-01	0.014	0.013	2.95E-01
6.83341	0.004	0.009	6.58E-01	0.006	0.013	6.70E-01
3.936725	0.001	0.007	8.63E-01	0.017	0.010	7.98E-02
1.501289699	0.001	0.005	8.76E-01	-0.007	0.008	3.43E-01
6.404458	0.002	0.010	8.16E-01	-0.021	0.015	1.46E-01
5.41501	0.004	0.009	6.80E-01	-0.011	0.013	4.01E-01
7.095491	0.003	0.010	7.44E-01	-0.013	0.015	4.14E-01
2.78679863	-0.003	0.012	8.31E-01	-0.052	0.018	3.84E-03
8.954617	0.002	0.007	8.01E-01	0.017	0.011	1.09E-01
2.075580132	0.001	0.003	6.64E-01	0.007	0.005	1.59E-01
8.861425	0.000	0.008	9.75E-01	0.002	0.013	8.76E-01
5.977189	0.002	0.009	7.90E-01	-0.002	0.013	9.05E-01
7.554721	-0.002	0.007	8.09E-01	0.012	0.011	2.80E-01
7.894182	-0.001	0.007	9.07E-01	0.004	0.010	6.95E-01
4.205535	-0.001	0.013	9.14E-01	0.018	0.020	3.70E-01
5.128033	0.002	0.007	7.84E-01	-0.003	0.010	7.66E-01
8.034138	-0.001	0.010	9.44E-01	0.012	0.015	4.22E-01
7.892163	0.000	0.009	9.99E-01	0.014	0.014	3.12E-01
2.742053095	0.001	0.011	9.26E-01	-0.017	0.016	2.85E-01
3.131306	-0.005	0.009	5.71E-01	-0.017	0.013	2.04E-01
0.503094201	0.001	0.009	9.22E-01	0.019	0.013	1.54E-01
4.205198	-0.001	0.014	9.52E-01	0.018	0.021	3.74E-01
3.188499	-0.002	0.006	7.92E-01	0.011	0.009	2.45E-01
8.731899	-0.001	0.006	8.70E-01	0.004	0.009	6.23E-01
6.279305	0.005	0.009	5.47E-01	-0.013	0.013	3.18E-01
8.549889	-0.002	0.006	7.51E-01	0.004	0.010	6.60E-01
5.963732	0.002	0.009	8.56E-01	-0.020	0.013	1.13E-01
7.397607	0.005	0.009	5.96E-01	-0.003	0.013	7.98E-01
8.630633	0.000	0.008	9.80E-01	-0.002	0.013	8.80E-01
6.033373	0.002	0.010	8.17E-01	0.006	0.015	6.96E-01
2.843319305	0.000	0.008	9.96E-01	-0.014	0.012	2.52E-01

2.036890384	0.002	0.004	6.48E-01	0.010	0.006	1.12E-01
3.799797	0.003	0.006	6.11E-01	0.007	0.009	4.11E-01
1.161156349	0.003	0.005	5.57E-01	-0.016	0.008	5.18E-02
3.206667	0.002	0.010	8.79E-01	0.014	0.015	3.47E-01
7.810747	-0.003	0.009	7.34E-01	0.004	0.013	7.47E-01
8.897087	-0.004	0.008	6.15E-01	-0.010	0.011	3.69E-01
8.70633	0.003	0.009	7.63E-01	-0.021	0.014	1.24E-01
6.595552	0.002	0.008	7.67E-01	-0.007	0.012	5.81E-01
7.305761	0.002	0.011	8.74E-01	0.013	0.016	4.11E-01
7.47095	0.001	0.006	8.56E-01	0.012	0.010	2.06E-01
7.414092	-0.005	0.010	5.84E-01	-0.006	0.015	6.63E-01
5.93278	-0.003	0.008	7.28E-01	0.007	0.013	5.52E-01
6.592861	-0.003	0.009	7.19E-01	-0.021	0.014	1.31E-01
6.466025	-0.005	0.010	6.58E-01	-0.019	0.015	2.17E-01
5.01331	0.003	0.009	7.62E-01	0.002	0.013	8.82E-01
3.514839	0.001	0.008	8.49E-01	0.015	0.012	1.88E-01
4.358948	-0.002	0.012	8.66E-01	0.010	0.018	5.61E-01
0.621182041	0.006	0.009	5.40E-01	-0.027	0.014	5.38E-02
2.785452899	-0.003	0.012	8.21E-01	-0.045	0.018	1.21E-02
7.165805	0.003	0.008	7.49E-01	0.004	0.012	7.27E-01
0.507804257	0.002	0.007	7.17E-01	-0.002	0.010	8.47E-01
1.813162711	-0.004	0.011	7.49E-01	0.002	0.016	9.10E-01
8.205382	-0.002	0.007	8.32E-01	0.003	0.011	7.77E-01
8.243062	0.000	0.007	9.62E-01	0.001	0.011	8.95E-01
0.789734769	-0.003	0.005	5.32E-01	0.000	0.007	9.78E-01
6.662166	0.001	0.010	8.95E-01	-0.002	0.015	8.81E-01
5.991655	-0.004	0.008	6.19E-01	-0.011	0.012	3.44E-01
7.408373	-0.004	0.007	6.27E-01	-0.013	0.011	2.49E-01
5.661279	0.002	0.007	7.53E-01	-0.005	0.010	6.06E-01
6.978076	-0.002	0.010	8.20E-01	0.017	0.015	2.86E-01
4.294017	-0.002	0.012	8.54E-01	0.013	0.018	4.50E-01
5.475231	0.002	0.007	7.77E-01	-0.008	0.011	4.52E-01
5.886016	0.000	0.007	9.92E-01	-0.011	0.010	2.76E-01
5.981899	0.001	0.009	9.28E-01	-0.014	0.013	2.91E-01
6.081147	0.005	0.009	5.74E-01	0.016	0.013	2.15E-01
8.530039	-0.001	0.009	9.24E-01	0.018	0.013	1.69E-01
8.089313	-0.003	0.008	7.52E-01	-0.012	0.012	3.04E-01
7.72462	-0.002	0.008	8.36E-01	-0.004	0.012	7.41E-01
4.17963	0.004	0.013	7.35E-01	0.007	0.019	7.04E-01
8.168038	0.002	0.009	8.64E-01	-0.001	0.013	9.49E-01
7.097846	-0.004	0.010	7.18E-01	-0.005	0.015	7.16E-01
6.566955	-0.001	0.008	9.04E-01	-0.010	0.012	4.13E-01
6.235906	0.003	0.008	7.18E-01	-0.003	0.012	8.31E-01

8.623231	0.001	0.009	9.46E-01	0.019	0.013	1.45E-01
6.494286	-0.001	0.010	9.12E-01	-0.008	0.015	6.09E-01
8.545852	-0.001	0.007	8.27E-01	0.001	0.010	9.15E-01
7.649932	0.002	0.008	8.01E-01	-0.010	0.012	3.96E-01
8.290836	0.000	0.009	9.73E-01	-0.017	0.013	2.00E-01
4.032272	-0.002	0.014	9.07E-01	-0.011	0.021	5.99E-01
5.984927	0.003	0.008	6.70E-01	0.002	0.011	8.89E-01
5.553284	0.004	0.008	6.43E-01	-0.020	0.012	1.09E-01
6.794047	0.005	0.008	5.13E-01	-0.005	0.011	6.34E-01
6.842157	0.000	0.007	9.98E-01	0.008	0.011	4.89E-01
1.862618302	-0.001	0.008	8.76E-01	0.014	0.012	2.26E-01
2.076925862	0.000	0.003	8.89E-01	0.008	0.005	1.41E-01
7.622681	-0.006	0.012	5.75E-01	0.018	0.017	2.92E-01
8.027409	-0.002	0.009	8.61E-01	0.003	0.014	8.25E-01
7.601149	0.007	0.011	5.21E-01	-0.002	0.016	9.14E-01
0.713364571	-0.002	0.008	8.30E-01	-0.007	0.011	5.61E-01
5.111548	0.002	0.008	7.67E-01	-0.004	0.012	7.66E-01
8.861762	0.002	0.008	8.36E-01	0.001	0.012	9.66E-01
2.851393687	0.002	0.008	7.48E-01	-0.009	0.012	4.51E-01
5.765573	0.001	0.009	9.49E-01	-0.025	0.014	6.28E-02
6.912471	0.000	0.009	9.86E-01	-0.001	0.013	9.34E-01
7.065885	0.004	0.009	7.06E-01	-0.001	0.014	9.44E-01
3.562949	-0.001	0.006	9.20E-01	0.018	0.010	6.01E-02
8.230614	-0.005	0.009	5.31E-01	0.000	0.013	9.71E-01
6.34491	-0.002	0.010	8.20E-01	0.000	0.015	9.82E-01
6.442475	0.006	0.010	5.29E-01	-0.031	0.015	4.31E-02
7.845399	-0.001	0.010	8.91E-01	0.001	0.015	9.65E-01
5.294567	0.003	0.011	7.98E-01	-0.026	0.017	1.16E-01
6.120846	-0.002	0.009	7.90E-01	-0.014	0.014	3.22E-01
5.908557	-0.002	0.007	7.81E-01	-0.014	0.011	2.00E-01
8.411952	-0.001	0.008	8.73E-01	-0.007	0.011	5.30E-01
3.6373	0.000	0.004	9.29E-01	0.009	0.005	8.51E-02
2.811694642	0.004	0.015	7.82E-01	-0.063	0.022	4.38E-03
8.939141	0.001	0.009	9.55E-01	0.010	0.014	4.65E-01
8.751748	-0.001	0.007	8.66E-01	0.002	0.011	8.22E-01
6.031355	0.000	0.009	1.00E+00	0.000	0.014	9.91E-01
8.926693	-0.001	0.007	8.99E-01	-0.001	0.011	9.44E-01
8.28007	-0.003	0.007	6.45E-01	0.001	0.011	9.16E-01
8.811633	0.004	0.009	6.04E-01	-0.003	0.013	7.99E-01
8.239025	0.003	0.009	7.04E-01	0.001	0.013	9.56E-01
4.356257	-0.002	0.013	8.80E-01	0.017	0.019	3.74E-01
2.745753854	-0.004	0.009	6.83E-01	-0.017	0.014	2.17E-01
5.457737	0.001	0.007	9.32E-01	-0.008	0.011	4.95E-01

4.302091	-0.004	0.014	8.02E-01	0.016	0.021	4.59E-01
4.233122	0.002	0.013	8.52E-01	0.005	0.020	8.17E-01
6.781936	0.000	0.008	9.80E-01	0.001	0.013	9.13E-01
6.286707	0.004	0.010	6.47E-01	-0.008	0.014	5.61E-01
8.987924	-0.004	0.010	7.20E-01	-0.003	0.015	8.22E-01
8.506153	-0.003	0.010	7.41E-01	-0.002	0.014	9.16E-01
5.078577	0.003	0.009	7.30E-01	-0.014	0.013	2.70E-01
8.52903	0.005	0.009	5.43E-01	0.017	0.013	1.88E-01
5.817047	-0.001	0.008	9.34E-01	-0.015	0.013	2.23E-01
0.825060191	-0.004	0.007	5.92E-01	0.006	0.010	5.85E-01
8.896751	-0.003	0.007	6.75E-01	0.007	0.010	4.83E-01
6.472754	-0.007	0.009	4.27E-01	0.001	0.013	9.61E-01
1.328026915	0.007	0.009	4.65E-01	0.000	0.014	9.82E-01
5.961713	-0.001	0.010	9.37E-01	-0.022	0.014	1.27E-01
1.159810619	0.004	0.005	4.73E-01	-0.009	0.007	2.45E-01
3.232908	-0.002	0.006	7.72E-01	0.014	0.009	1.17E-01
0.968716907	-0.002	0.005	7.46E-01	0.010	0.008	1.81E-01
6.182076	-0.003	0.006	6.61E-01	0.001	0.009	9.39E-01
8.433483	0.002	0.008	8.42E-01	-0.009	0.012	4.89E-01
2.174827747	0.004	0.010	7.20E-01	-0.013	0.015	4.18E-01
7.862894	-0.004	0.007	5.74E-01	0.008	0.011	4.71E-01
3.752024	-0.005	0.011	6.35E-01	0.021	0.017	2.19E-01
8.662594	-0.001	0.006	8.03E-01	0.003	0.009	7.39E-01
6.20529	-0.002	0.008	7.91E-01	-0.001	0.012	9.42E-01
6.43541	0.001	0.007	8.82E-01	-0.004	0.011	6.88E-01
3.304905	-0.004	0.010	6.81E-01	-0.004	0.014	7.70E-01
1.688682652	-0.001	0.004	8.39E-01	0.003	0.006	5.78E-01
0.959296795	0.001	0.005	8.04E-01	0.011	0.008	1.43E-01
3.899381	0.001	0.005	9.02E-01	0.020	0.007	8.41E-03
0.538083191	0.003	0.009	7.56E-01	0.022	0.014	1.01E-01
5.87996	0.002	0.009	7.77E-01	-0.016	0.013	2.16E-01
0.801173477	-0.001	0.006	8.66E-01	0.005	0.009	5.35E-01
3.89568	0.001	0.005	8.08E-01	0.018	0.007	1.19E-02
7.972234	-0.001	0.009	8.91E-01	0.020	0.014	1.55E-01
3.947155	-0.002	0.007	7.99E-01	0.007	0.010	4.96E-01
6.685043	0.001	0.008	9.01E-01	-0.024	0.012	4.56E-02
7.302397	-0.005	0.011	6.46E-01	0.005	0.017	7.47E-01
6.861334	0.004	0.010	6.67E-01	0.008	0.015	6.23E-01
5.964068	-0.002	0.010	8.25E-01	-0.011	0.014	4.49E-01
5.632682	-0.001	0.010	9.41E-01	-0.016	0.015	2.69E-01
7.334694	-0.003	0.006	6.73E-01	-0.003	0.009	7.63E-01
2.821114754	-0.004	0.016	8.25E-01	-0.080	0.024	9.00E-04
0.856684854	0.002	0.008	7.65E-01	-0.008	0.012	4.74E-01

0.587538782	0.000	0.009	9.67E-01	-0.013	0.013	3.42E-01
8.105125	-0.001	0.007	8.61E-01	-0.011	0.010	2.66E-01
2.787135062	-0.003	0.012	8.32E-01	-0.052	0.018	3.93E-03
7.177917	0.004	0.010	7.17E-01	0.022	0.016	1.58E-01
8.120601	-0.002	0.008	8.41E-01	0.005	0.012	6.84E-01
5.608459	-0.002	0.008	7.90E-01	-0.004	0.012	7.69E-01
1.702812821	-0.001	0.004	8.50E-01	0.005	0.007	4.72E-01
7.203822	0.002	0.006	6.94E-01	0.002	0.009	8.04E-01
7.855492	0.000	0.007	9.53E-01	0.007	0.010	4.67E-01
8.621549	0.001	0.009	9.28E-01	0.006	0.013	6.36E-01
2.422778566	0.008	0.012	4.96E-01	0.000	0.018	9.85E-01
5.144855	0.002	0.009	8.05E-01	-0.020	0.013	1.16E-01
1.049460729	-0.002	0.008	8.03E-01	-0.019	0.012	1.28E-01
0.551876927	0.005	0.009	5.87E-01	-0.002	0.013	8.46E-01
5.294231	0.003	0.011	8.05E-01	-0.026	0.017	1.12E-01
6.203608	-0.002	0.009	8.54E-01	-0.009	0.014	5.03E-01
6.145742	0.004	0.009	6.27E-01	-0.005	0.013	6.82E-01
6.387637	0.001	0.009	9.08E-01	-0.028	0.014	3.81E-02
3.186481	0.002	0.006	7.69E-01	0.013	0.009	1.33E-01
2.367267189	0.002	0.010	8.34E-01	0.036	0.015	1.48E-02
6.654091	-0.004	0.007	6.14E-01	-0.009	0.011	4.32E-01
7.516368	-0.004	0.009	6.69E-01	-0.023	0.013	7.60E-02
6.264166	0.005	0.008	5.00E-01	-0.007	0.011	5.52E-01
4.293344	0.004	0.012	7.47E-01	0.018	0.018	3.02E-01
2.74272596	0.001	0.010	9.36E-01	-0.016	0.016	2.94E-01
2.541202838	0.004	0.028	8.77E-01	0.019	0.041	6.49E-01
8.891368	0.004	0.010	7.30E-01	0.003	0.015	8.61E-01
7.025513	-0.006	0.010	5.52E-01	-0.029	0.014	4.11E-02
7.630082	-0.002	0.013	8.78E-01	0.025	0.019	1.84E-01
4.295362	-0.002	0.012	8.46E-01	0.006	0.018	7.62E-01
3.169659	0.001	0.006	9.34E-01	0.006	0.009	4.81E-01
6.497314	0.004	0.009	6.63E-01	-0.010	0.013	4.36E-01
8.235661	0.000	0.008	9.81E-01	-0.025	0.012	4.29E-02
3.322063	0.002	0.011	8.62E-01	0.015	0.016	3.50E-01
6.031018	0.001	0.008	8.77E-01	-0.001	0.012	9.17E-01
8.633997	0.004	0.010	7.12E-01	-0.011	0.014	4.36E-01
7.879042	-0.004	0.008	6.52E-01	0.002	0.012	8.73E-01
1.002360166	0.003	0.008	6.71E-01	-0.007	0.012	5.66E-01
6.593197	0.001	0.008	8.78E-01	-0.023	0.012	6.87E-02
1.823255689	-0.001	0.010	9.02E-01	0.012	0.015	4.01E-01
6.303529	-0.002	0.009	8.58E-01	-0.004	0.013	7.33E-01
8.576804	-0.002	0.008	7.45E-01	-0.008	0.011	4.68E-01
3.931679	-0.001	0.007	9.33E-01	0.015	0.011	1.69E-01

7.438988	-0.001	0.006	9.31E-01	0.009	0.010	3.61E-01
0.681739907	0.003	0.012	7.79E-01	-0.019	0.018	2.77E-01
6.86739	0.005	0.010	6.22E-01	0.006	0.014	7.00E-01
6.41657	0.000	0.010	9.90E-01	-0.020	0.014	1.51E-01
3.799124	0.003	0.006	5.71E-01	0.011	0.009	1.99E-01
6.120509	0.000	0.008	9.96E-01	-0.006	0.012	6.19E-01
7.543619	-0.004	0.010	6.70E-01	-0.002	0.015	8.87E-01
4.19746	-0.001	0.011	9.10E-01	0.022	0.017	2.04E-01
8.638034	0.002	0.007	8.17E-01	-0.006	0.011	5.87E-01
0.615799119	-0.003	0.008	7.01E-01	0.000	0.011	9.74E-01
6.878492	0.005	0.009	5.80E-01	-0.015	0.013	2.62E-01
6.393692	-0.002	0.009	8.37E-01	0.001	0.013	9.61E-01
6.857297	-0.002	0.010	8.13E-01	0.003	0.015	8.57E-01
8.449296	0.001	0.009	9.45E-01	-0.010	0.013	4.51E-01
8.696574	0.000	0.009	9.93E-01	0.001	0.014	9.27E-01
0.514869342	0.001	0.009	8.90E-01	0.002	0.013	8.70E-01
6.999608	-0.001	0.008	9.04E-01	-0.001	0.012	9.54E-01
1.750586249	-0.001	0.007	8.39E-01	0.004	0.010	7.17E-01
1.666814534	0.000	0.004	9.51E-01	0.010	0.006	1.07E-01
1.921494005	-0.002	0.006	7.83E-01	-0.005	0.008	5.40E-01
3.927642	0.001	0.009	9.40E-01	0.017	0.013	1.91E-01
8.297901	0.003	0.008	6.80E-01	-0.006	0.012	5.94E-01
7.277837	-0.005	0.009	5.99E-01	0.001	0.014	9.70E-01
8.689845	-0.002	0.007	7.62E-01	0.014	0.010	1.46E-01
8.929048	-0.005	0.009	5.97E-01	-0.008	0.013	5.49E-01
8.179477	-0.005	0.007	5.29E-01	-0.009	0.011	4.24E-01
4.031936	0.004	0.014	7.47E-01	-0.001	0.020	9.75E-01
6.761077	0.003	0.009	7.52E-01	-0.011	0.013	4.26E-01
6.929293	-0.005	0.011	6.76E-01	-0.014	0.017	4.13E-01
2.071206508	0.002	0.004	5.87E-01	0.012	0.006	3.80E-02
5.650849	0.004	0.009	6.38E-01	-0.014	0.013	2.96E-01
6.204617	-0.002	0.008	8.10E-01	-0.008	0.012	5.03E-01
7.66608	0.003	0.010	7.74E-01	-0.002	0.016	9.23E-01
8.935777	0.003	0.009	7.49E-01	0.000	0.013	9.86E-01
6.365096	-0.002	0.011	8.54E-01	-0.001	0.016	9.46E-01
1.789275997	0.005	0.011	6.32E-01	0.004	0.017	8.34E-01
2.748445314	-0.004	0.008	6.56E-01	-0.019	0.012	1.22E-01
5.24242	-0.005	0.004	2.81E-01	-0.002	0.007	7.78E-01
4.017133	-0.005	0.013	7.18E-01	0.007	0.019	7.07E-01
1.328363347	0.007	0.009	4.26E-01	-0.002	0.014	8.86E-01
8.293191	-0.005	0.009	5.87E-01	-0.020	0.014	1.47E-01
6.580076	-0.001	0.009	9.09E-01	-0.021	0.014	1.17E-01
7.953057	0.002	0.008	8.34E-01	-0.009	0.012	4.58E-01

2.33362393	0.001	0.006	8.23E-01	-0.001	0.009	9.46E-01
5.751779	-0.001	0.010	8.93E-01	-0.015	0.015	3.29E-01
6.970001	-0.005	0.011	6.43E-01	-0.005	0.016	7.59E-01
7.64825	0.000	0.010	9.63E-01	0.003	0.015	8.48E-01
1.682290433	0.001	0.004	7.95E-01	0.010	0.006	7.58E-02
6.270222	-0.002	0.008	8.48E-01	0.017	0.012	1.69E-01
5.968778	0.000	0.009	9.81E-01	-0.004	0.014	7.70E-01
2.784443602	-0.003	0.012	8.08E-01	-0.046	0.018	1.16E-02
3.785331	-0.003	0.011	8.13E-01	0.027	0.017	1.13E-01
6.350966	-0.003	0.010	7.67E-01	-0.007	0.015	6.65E-01
0.991930756	-0.003	0.006	6.06E-01	0.012	0.010	2.07E-01
5.875586	0.002	0.010	8.05E-01	-0.005	0.014	7.26E-01
6.8758	-0.001	0.006	9.13E-01	0.002	0.009	8.07E-01
7.687612	-0.002	0.010	8.45E-01	-0.007	0.015	6.32E-01
6.4307	0.000	0.009	9.94E-01	0.000	0.013	9.73E-01
4.039001	-0.001	0.015	9.38E-01	-0.017	0.022	4.56E-01
6.976394	0.006	0.011	6.15E-01	0.005	0.017	7.82E-01
7.308116	-0.004	0.010	6.92E-01	-0.013	0.016	4.00E-01
1.724008074	-0.002	0.005	7.40E-01	0.002	0.007	8.06E-01
7.635802	0.000	0.012	9.75E-01	0.029	0.018	1.14E-01
6.299155	0.001	0.009	9.46E-01	-0.024	0.013	7.08E-02
2.276430389	0.001	0.014	9.42E-01	0.012	0.021	5.90E-01
7.159077	0.003	0.007	7.29E-01	0.011	0.011	3.23E-01
0.532363837	-0.003	0.010	7.49E-01	0.013	0.015	3.73E-01
7.492481	0.002	0.009	8.50E-01	0.006	0.013	6.24E-01
1.923176168	0.000	0.004	9.52E-01	0.001	0.006	8.88E-01
2.069860778	0.001	0.004	8.74E-01	0.011	0.006	8.73E-02
1.674888916	0.000	0.004	9.95E-01	0.003	0.006	6.32E-01
7.077323	-0.002	0.009	8.20E-01	0.000	0.013	9.76E-01
5.689875	-0.006	0.009	5.12E-01	-0.023	0.014	9.94E-02
0.905131148	0.002	0.006	7.46E-01	-0.014	0.009	1.28E-01
7.899228	-0.002	0.007	7.78E-01	-0.001	0.011	9.30E-01
0.621518473	0.000	0.009	9.79E-01	-0.012	0.014	3.92E-01
4.294353	-0.002	0.012	8.80E-01	0.019	0.018	2.92E-01
6.558544	0.002	0.007	8.25E-01	0.006	0.011	5.66E-01
1.457889895	-0.001	0.003	8.08E-01	0.002	0.005	6.29E-01
8.523984	0.001	0.006	8.28E-01	0.004	0.009	6.90E-01
5.985263	0.000	0.008	9.79E-01	-0.003	0.012	7.91E-01
3.784658	-0.003	0.010	7.66E-01	0.024	0.015	9.44E-02
7.901247	0.002	0.009	7.91E-01	-0.005	0.013	7.10E-01
6.035728	0.002	0.007	7.44E-01	-0.019	0.011	8.17E-02
8.215138	0.003	0.007	7.05E-01	0.006	0.011	5.64E-01
6.844849	-0.001	0.011	9.22E-01	-0.018	0.016	2.55E-01

1.839404453	0.003	0.010	7.45E-01	0.002	0.015	8.77E-01
1.882804257	-0.001	0.008	9.11E-01	-0.011	0.012	3.67E-01
7.416784	-0.004	0.009	6.38E-01	-0.010	0.013	4.60E-01
0.907486176	0.002	0.006	7.88E-01	-0.018	0.009	3.68E-02
7.751871	0.000	0.009	9.91E-01	-0.007	0.014	5.94E-01
2.782425006	-0.003	0.012	7.77E-01	-0.044	0.018	1.51E-02
3.879532	0.001	0.005	8.58E-01	0.018	0.007	1.17E-02
6.744592	-0.003	0.007	6.22E-01	-0.013	0.010	2.19E-01
8.238689	-0.003	0.008	6.88E-01	0.005	0.012	6.96E-01
8.352067	0.004	0.010	7.19E-01	0.003	0.014	8.52E-01
0.967034744	-0.003	0.007	6.05E-01	0.017	0.010	8.12E-02
6.475782	0.001	0.010	8.81E-01	-0.005	0.015	7.46E-01
2.421432836	0.006	0.014	6.79E-01	-0.016	0.020	4.35E-01
2.651216296	-0.002	0.013	8.87E-01	0.022	0.020	2.57E-01
6.035055	-0.002	0.008	8.50E-01	-0.023	0.012	5.89E-02
8.493705	-0.001	0.008	9.16E-01	-0.002	0.011	8.88E-01
7.113995	-0.001	0.008	8.92E-01	-0.004	0.012	7.33E-01
8.716423	0.000	0.009	9.92E-01	-0.009	0.014	5.33E-01
0.787379741	-0.002	0.004	6.58E-01	0.001	0.007	8.68E-01
6.469726	0.002	0.008	7.51E-01	-0.017	0.012	1.52E-01
6.21572	0.000	0.007	9.52E-01	-0.028	0.011	1.11E-02
1.659413017	0.001	0.004	7.65E-01	0.001	0.006	9.04E-01
6.352648	-0.002	0.009	8.51E-01	-0.014	0.014	3.08E-01
2.849711524	-0.002	0.008	8.30E-01	-0.009	0.012	4.47E-01
6.941405	0.000	0.009	9.82E-01	0.022	0.013	1.02E-01
4.036646	-0.002	0.015	9.02E-01	-0.015	0.022	5.02E-01
6.494622	-0.001	0.009	9.53E-01	-0.006	0.014	6.52E-01
6.863352	-0.001	0.009	8.91E-01	0.004	0.013	7.70E-01
6.667212	0.002	0.010	8.77E-01	-0.008	0.015	5.91E-01
7.602831	0.004	0.013	7.27E-01	0.035	0.019	7.02E-02
6.287716	0.002	0.009	8.46E-01	-0.006	0.014	6.69E-01
2.849038659	-0.002	0.009	8.59E-01	-0.017	0.013	1.91E-01
8.181159	-0.005	0.009	5.64E-01	0.017	0.014	2.23E-01
1.066955224	-0.002	0.007	7.44E-01	-0.005	0.010	6.01E-01
5.700641	0.002	0.007	7.35E-01	-0.011	0.010	2.52E-01
6.34962	0.000	0.006	9.57E-01	-0.010	0.009	2.47E-01
6.275941	0.005	0.010	5.78E-01	-0.003	0.014	8.18E-01
4.0353	0.004	0.014	7.53E-01	-0.014	0.021	5.19E-01
6.037747	0.000	0.007	9.57E-01	0.010	0.011	3.47E-01
6.676632	0.002	0.008	7.50E-01	0.006	0.011	6.30E-01
4.211254	0.000	0.012	1.00E+00	0.006	0.017	7.12E-01
0.617481282	-0.002	0.007	7.99E-01	0.008	0.011	4.61E-01
6.859315	-0.001	0.008	8.75E-01	0.002	0.012	8.46E-01

5.491044	0.000	0.008	9.68E-01	-0.017	0.012	1.64E-01
0.53539173	-0.003	0.009	7.11E-01	-0.012	0.013	3.68E-01
6.74762	-0.004	0.012	7.71E-01	-0.023	0.018	2.08E-01
8.84124	0.002	0.009	8.08E-01	-0.008	0.013	5.55E-01
5.62999	0.001	0.007	8.39E-01	-0.004	0.011	7.44E-01
0.610416198	0.002	0.008	7.64E-01	0.000	0.012	9.85E-01
2.162379741	-0.003	0.011	7.67E-01	-0.015	0.016	3.73E-01
3.331147	-0.004	0.010	7.02E-01	0.014	0.015	3.40E-01
4.214282	0.003	0.012	8.04E-01	0.017	0.017	3.35E-01
5.98459	-0.003	0.007	7.27E-01	0.011	0.011	3.10E-01
7.975262	0.000	0.009	9.59E-01	-0.004	0.013	7.52E-01
7.482388	0.000	0.008	9.89E-01	-0.005	0.012	6.88E-01
8.319433	-0.002	0.007	8.35E-01	0.001	0.011	9.18E-01
6.040775	0.001	0.008	8.61E-01	0.010	0.012	3.93E-01
3.665561	0.000	0.005	9.84E-01	0.015	0.008	4.79E-02
6.404795	0.001	0.010	9.14E-01	-0.023	0.014	1.12E-01
2.635403964	0.000	0.009	9.56E-01	-0.009	0.013	4.71E-01
3.032731	0.001	0.007	8.60E-01	0.018	0.010	9.20E-02
0.52765378	0.000	0.008	9.70E-01	-0.006	0.012	5.98E-01
6.561909	0.003	0.009	7.17E-01	0.010	0.014	4.63E-01
1.778510154	-0.001	0.011	9.20E-01	-0.002	0.017	9.06E-01
2.573163934	0.000	0.010	9.88E-01	-0.010	0.014	4.73E-01
8.879256	0.000	0.010	9.84E-01	0.014	0.015	3.39E-01
6.834419	0.000	0.008	9.90E-01	0.002	0.013	8.46E-01
7.024167	-0.002	0.009	8.60E-01	-0.028	0.013	3.43E-02
7.636138	0.005	0.013	7.02E-01	0.038	0.019	4.30E-02
7.1399	-0.005	0.011	6.67E-01	-0.007	0.016	6.46E-01
7.109285	0.004	0.010	7.26E-01	0.017	0.015	2.56E-01
4.038664	-0.001	0.015	9.40E-01	-0.017	0.022	4.51E-01
3.615096	0.000	0.006	9.80E-01	0.010	0.010	2.84E-01
8.694891	0.003	0.007	6.78E-01	-0.004	0.011	7.06E-01
4.230094	-0.002	0.013	8.82E-01	0.016	0.019	4.15E-01
6.562582	0.002	0.007	7.25E-01	-0.004	0.011	7.33E-01
1.458226327	0.000	0.003	9.34E-01	-0.001	0.005	8.41E-01
7.340077	-0.005	0.009	5.51E-01	0.000	0.013	9.83E-01
7.410728	-0.003	0.007	6.91E-01	-0.010	0.011	3.83E-01
8.883294	0.004	0.010	6.71E-01	-0.002	0.015	8.90E-01
6.486548	-0.002	0.010	8.78E-01	0.000	0.015	9.85E-01
2.291906288	0.000	0.004	9.95E-01	-0.014	0.007	3.51E-02
7.818484	-0.003	0.010	7.48E-01	-0.004	0.014	7.61E-01
7.52377	0.002	0.009	8.59E-01	-0.018	0.013	1.73E-01
5.994347	0.000	0.010	9.60E-01	0.008	0.014	5.88E-01
3.636627	0.000	0.004	8.91E-01	0.010	0.005	7.75E-02

7.997467	0.001	0.011	9.13E-01	0.000	0.016	9.75E-01
7.544965	0.002	0.010	8.07E-01	-0.001	0.015	9.37E-01
8.081575	0.000	0.006	9.42E-01	0.000	0.010	9.64E-01
0.789398336	-0.003	0.005	5.40E-01	0.000	0.007	9.51E-01
3.309278	-0.003	0.012	8.25E-01	-0.011	0.019	5.43E-01
4.046739	0.001	0.009	9.12E-01	-0.014	0.014	3.01E-01
4.100231	0.001	0.010	9.28E-01	-0.003	0.016	8.37E-01
8.613138	0.001	0.009	9.35E-01	0.014	0.014	3.33E-01
6.659811	0.000	0.009	9.85E-01	0.008	0.013	5.35E-01
4.231777	-0.002	0.013	8.47E-01	0.008	0.019	6.61E-01
8.256856	-0.001	0.009	9.46E-01	-0.005	0.013	7.33E-01
8.0264	-0.002	0.008	8.02E-01	-0.017	0.011	1.41E-01
2.276766822	0.001	0.013	9.65E-01	0.011	0.019	5.72E-01
7.423849	0.000	0.008	9.64E-01	0.006	0.011	6.11E-01
5.242757	-0.005	0.004	3.01E-01	-0.002	0.007	8.20E-01
0.796463421	-0.003	0.006	5.97E-01	0.002	0.008	8.26E-01
0.684431368	0.003	0.013	8.05E-01	-0.007	0.020	7.45E-01
8.590261	0.001	0.006	8.45E-01	0.002	0.010	8.12E-01
7.482052	-0.003	0.010	7.45E-01	-0.010	0.015	5.19E-01
3.632927	-0.001	0.004	8.72E-01	0.010	0.006	7.09E-02
4.023861	0.005	0.013	7.14E-01	0.009	0.019	6.35E-01
8.149534	0.000	0.007	9.51E-01	0.008	0.010	4.11E-01
2.172472718	0.004	0.009	6.93E-01	-0.028	0.014	5.14E-02
1.673543186	-0.001	0.004	7.73E-01	0.007	0.006	2.19E-01
8.527348	-0.002	0.009	8.59E-01	0.009	0.013	5.10E-01
8.629287	-0.002	0.007	7.73E-01	0.005	0.010	6.52E-01
6.760404	0.000	0.010	9.80E-01	-0.021	0.015	1.46E-01
8.752758	-0.001	0.009	9.54E-01	-0.001	0.014	9.51E-01
6.144396	0.002	0.008	7.99E-01	-0.002	0.012	8.55E-01
8.473519	-0.001	0.009	9.36E-01	-0.036	0.013	5.22E-03
4.133538	-0.003	0.013	8.11E-01	0.013	0.019	5.00E-01
3.997956	0.000	0.008	9.59E-01	0.008	0.012	5.16E-01
3.18177	0.002	0.005	6.40E-01	0.013	0.008	9.89E-02
7.305425	-0.005	0.011	6.57E-01	0.005	0.016	7.46E-01
6.451222	0.002	0.008	7.93E-01	0.008	0.012	4.65E-01
3.986181	-0.003	0.008	6.85E-01	0.007	0.011	5.29E-01
6.940395	0.000	0.009	9.58E-01	0.027	0.013	4.55E-02
7.547993	-0.002	0.010	8.05E-01	-0.021	0.014	1.54E-01
3.324754	0.001	0.010	9.41E-01	0.019	0.015	2.14E-01
3.992909	-0.003	0.011	7.67E-01	0.005	0.016	7.64E-01
0.504439931	0.001	0.008	8.66E-01	-0.012	0.012	2.99E-01
6.758049	0.001	0.007	9.43E-01	0.006	0.011	6.12E-01
6.049858	0.001	0.008	8.54E-01	-0.018	0.011	1.23E-01

7.176571	0.000	0.010	9.98E-01	0.007	0.014	6.06E-01
1.832675801	-0.001	0.009	9.56E-01	0.000	0.014	9.93E-01
6.793711	0.002	0.010	8.47E-01	-0.017	0.015	2.64E-01
8.74973	0.001	0.008	9.24E-01	0.019	0.013	1.34E-01
7.290622	-0.001	0.010	9.21E-01	0.011	0.015	4.45E-01
5.837569	0.001	0.009	8.88E-01	-0.001	0.013	9.59E-01
6.854269	-0.004	0.007	6.24E-01	-0.008	0.011	4.82E-01
4.095521	0.001	0.010	9.38E-01	0.016	0.015	2.70E-01
6.889931	0.006	0.009	4.85E-01	-0.007	0.013	5.73E-01
3.170668	-0.003	0.006	6.62E-01	0.005	0.009	5.49E-01
1.857571813	-0.001	0.008	9.08E-01	0.010	0.012	4.07E-01
6.892958	0.004	0.009	6.94E-01	0.008	0.014	5.76E-01
2.533464889	0.002	0.009	8.08E-01	-0.001	0.014	9.34E-01
5.653204	0.003	0.007	6.72E-01	-0.008	0.011	4.38E-01
1.85723538	-0.001	0.008	8.56E-01	0.001	0.012	9.02E-01
6.119836	-0.002	0.008	7.69E-01	0.013	0.012	2.76E-01
3.322736	0.002	0.011	8.60E-01	0.017	0.016	2.92E-01
7.139563	0.001	0.010	9.15E-01	-0.007	0.015	6.34E-01
8.5583	0.000	0.007	9.73E-01	-0.002	0.010	8.35E-01
5.61115	0.002	0.010	8.76E-01	-0.014	0.015	3.46E-01
6.233887	0.002	0.007	7.80E-01	-0.013	0.011	2.41E-01
3.694494	0.001	0.005	7.88E-01	0.018	0.007	1.23E-02
6.883538	0.005	0.009	5.90E-01	0.010	0.014	4.88E-01
2.536156349	-0.005	0.011	6.54E-01	-0.005	0.017	7.66E-01
3.63831	0.002	0.004	5.92E-01	0.011	0.006	6.95E-02
6.205963	-0.003	0.007	6.40E-01	0.002	0.011	8.23E-01
8.685135	0.003	0.006	5.78E-01	-0.005	0.009	5.72E-01
6.836438	-0.004	0.010	6.95E-01	-0.003	0.014	8.26E-01
8.565701	-0.003	0.009	7.22E-01	-0.016	0.014	2.37E-01
1.438040372	0.002	0.004	5.90E-01	0.000	0.006	9.97E-01
3.743613	-0.007	0.010	4.32E-01	-0.008	0.014	5.81E-01
3.993246	-0.004	0.011	7.46E-01	0.005	0.016	7.57E-01
8.352403	0.002	0.009	7.94E-01	0.006	0.014	6.97E-01
2.07793516	0.001	0.004	8.17E-01	0.009	0.005	7.90E-02
8.650482	0.002	0.008	8.17E-01	0.008	0.011	4.77E-01
2.811358209	0.004	0.015	7.87E-01	-0.067	0.022	2.37E-03
6.970338	-0.005	0.011	6.18E-01	0.002	0.016	8.90E-01
5.82714	0.004	0.008	6.66E-01	0.005	0.012	7.02E-01
7.127788	-0.001	0.008	9.07E-01	-0.008	0.012	4.95E-01
6.375525	0.005	0.008	5.21E-01	0.006	0.012	5.74E-01
8.523311	0.001	0.009	9.40E-01	0.004	0.013	7.40E-01
7.121396	0.001	0.009	8.76E-01	-0.018	0.013	1.70E-01
6.771843	0.001	0.010	9.30E-01	-0.017	0.014	2.33E-01

5.69223	0.004	0.010	7.31E-01	0.010	0.015	4.99E-01
1.055516516	0.002	0.006	7.86E-01	-0.015	0.009	8.42E-02
7.558422	0.000	0.009	9.67E-01	-0.008	0.013	5.38E-01
5.688193	-0.001	0.007	9.14E-01	-0.010	0.011	3.71E-01
3.937062	0.001	0.007	8.39E-01	0.015	0.010	1.47E-01
7.433269	-0.004	0.010	6.99E-01	-0.015	0.015	3.20E-01
8.90819	-0.001	0.008	9.01E-01	0.004	0.011	6.91E-01
3.436114	0.003	0.015	8.60E-01	0.016	0.022	4.64E-01
7.449418	0.000	0.006	9.65E-01	-0.004	0.009	6.28E-01
3.255786	-0.002	0.010	8.49E-01	0.009	0.015	5.57E-01
0.59258527	0.002	0.009	7.89E-01	-0.017	0.013	1.82E-01
2.727250061	-0.004	0.009	6.80E-01	-0.014	0.014	3.05E-01
7.333685	0.001	0.006	8.47E-01	-0.002	0.009	8.11E-01
8.220858	0.004	0.008	6.33E-01	0.010	0.011	3.60E-01
5.705015	0.001	0.008	9.11E-01	0.001	0.012	9.00E-01
2.783434304	-0.003	0.012	7.95E-01	-0.047	0.018	9.53E-03
1.199509665	0.001	0.009	9.30E-01	0.008	0.014	5.35E-01
5.97147	0.005	0.010	5.79E-01	-0.015	0.015	2.90E-01
7.957768	-0.006	0.010	5.88E-01	0.000	0.015	9.81E-01
3.24031	0.000	0.006	9.58E-01	0.015	0.009	1.15E-01
8.669659	0.006	0.009	5.06E-01	-0.009	0.013	5.19E-01
7.868276	0.001	0.009	8.62E-01	-0.014	0.013	2.69E-01
1.663113775	0.001	0.004	7.37E-01	0.001	0.006	8.38E-01
0.503430634	-0.004	0.009	7.05E-01	-0.005	0.014	7.34E-01
5.635037	-0.001	0.011	9.26E-01	-0.012	0.016	4.53E-01
5.637728	0.003	0.009	7.78E-01	0.005	0.013	7.26E-01
5.969451	0.000	0.007	9.85E-01	-0.010	0.011	3.68E-01
6.678651	-0.003	0.007	6.80E-01	0.015	0.011	1.62E-01
5.079587	0.004	0.008	6.44E-01	-0.005	0.012	6.67E-01
4.018478	-0.003	0.013	8.46E-01	0.026	0.020	2.00E-01
8.966392	-0.001	0.007	8.65E-01	-0.010	0.010	3.30E-01
7.493154	-0.004	0.010	6.97E-01	-0.013	0.014	3.84E-01
5.563377	0.002	0.009	8.05E-01	-0.030	0.013	2.47E-02
7.961132	0.000	0.007	9.47E-01	0.001	0.011	9.33E-01
0.755418644	0.001	0.005	8.26E-01	0.002	0.008	7.75E-01
8.781355	0.003	0.008	7.18E-01	0.003	0.012	8.11E-01
7.277164	-0.004	0.009	6.93E-01	-0.009	0.014	5.30E-01
6.077109	0.001	0.009	9.27E-01	-0.013	0.013	3.01E-01
7.650941	0.001	0.007	8.53E-01	-0.008	0.010	4.03E-01
8.348366	-0.002	0.007	7.67E-01	-0.005	0.011	6.48E-01
6.594543	-0.001	0.007	8.74E-01	-0.009	0.011	4.19E-01
8.639044	0.001	0.009	9.35E-01	-0.015	0.013	2.48E-01
1.504990458	0.001	0.006	8.81E-01	-0.014	0.009	9.97E-02

7.966851	-0.003	0.008	6.76E-01	-0.020	0.012	9.65E-02
5.065457	0.001	0.007	9.23E-01	-0.004	0.011	6.84E-01
7.128125	0.002	0.008	8.26E-01	-0.017	0.012	1.39E-01
6.495295	-0.003	0.008	6.86E-01	-0.007	0.012	5.54E-01
8.110172	-0.001	0.010	9.47E-01	0.003	0.015	8.60E-01
0.966698312	-0.003	0.007	6.40E-01	0.017	0.010	1.05E-01
7.318546	-0.003	0.006	6.74E-01	0.001	0.009	9.42E-01
7.056801	-0.002	0.011	8.80E-01	-0.015	0.016	3.32E-01
6.974375	-0.005	0.011	6.61E-01	0.006	0.016	7.22E-01
3.318362	0.000	0.011	1.00E+00	0.006	0.017	7.17E-01
6.672931	0.001	0.009	9.24E-01	-0.010	0.013	4.69E-01
3.169995	-0.002	0.006	7.37E-01	0.005	0.009	5.52E-01
6.932994	-0.005	0.010	5.94E-01	0.000	0.015	9.76E-01
6.458287	0.002	0.008	7.86E-01	0.030	0.012	1.49E-02
2.151613898	-0.002	0.008	7.72E-01	-0.003	0.012	8.08E-01
3.18278	0.002	0.005	6.49E-01	0.013	0.008	8.61E-02
8.654856	-0.003	0.009	7.37E-01	-0.013	0.013	3.34E-01
0.709327379	0.004	0.009	6.78E-01	-0.004	0.013	7.61E-01
6.132621	-0.002	0.009	8.49E-01	0.008	0.013	5.25E-01
8.863444	-0.001	0.009	9.15E-01	-0.001	0.013	9.09E-01
2.152623195	-0.003	0.008	7.50E-01	-0.002	0.012	8.54E-01
7.609896	-0.003	0.016	8.69E-01	0.001	0.024	9.70E-01
8.342646	-0.003	0.011	7.95E-01	0.003	0.016	8.51E-01
6.696482	-0.003	0.010	7.51E-01	0.018	0.015	2.29E-01
6.058269	-0.002	0.009	7.94E-01	0.005	0.013	7.30E-01
2.820441889	0.004	0.016	7.93E-01	-0.081	0.024	9.98E-04
3.372864	0.004	0.011	7.13E-01	-0.004	0.017	8.22E-01
4.179293	0.003	0.012	8.13E-01	0.005	0.018	7.91E-01
0.793435527	-0.003	0.005	5.30E-01	-0.003	0.008	7.18E-01
5.293894	0.002	0.011	8.36E-01	-0.027	0.016	1.04E-01
8.810961	0.001	0.009	8.72E-01	-0.039	0.014	3.94E-03
8.111854	-0.003	0.009	6.97E-01	-0.008	0.013	5.26E-01
8.044567	-0.002	0.007	8.12E-01	-0.001	0.011	9.11E-01
8.665958	0.001	0.007	9.12E-01	0.001	0.011	9.25E-01
8.936114	0.002	0.008	7.93E-01	-0.017	0.012	1.62E-01
7.496518	-0.005	0.009	5.44E-01	0.011	0.013	4.05E-01
5.652195	-0.001	0.008	8.85E-01	-0.011	0.013	3.69E-01
8.472846	0.002	0.009	8.51E-01	-0.011	0.014	4.31E-01
7.274809	-0.006	0.010	5.46E-01	0.012	0.015	4.26E-01
4.122436	-0.001	0.009	9.35E-01	0.009	0.013	5.05E-01
6.854605	0.000	0.008	9.75E-01	0.003	0.012	8.07E-01
6.454587	-0.001	0.009	9.01E-01	-0.027	0.014	5.33E-02
7.418803	-0.002	0.008	7.76E-01	-0.013	0.012	2.58E-01

2.783770736	-0.003	0.012	8.11E-01	-0.050	0.018	6.45E-03
6.91449	-0.002	0.009	8.02E-01	-0.009	0.014	5.13E-01
4.204526	0.004	0.014	7.69E-01	0.020	0.020	3.21E-01
5.57246	-0.002	0.007	7.41E-01	-0.009	0.011	4.09E-01
8.429446	-0.001	0.009	9.37E-01	0.018	0.014	1.83E-01
7.982327	0.000	0.009	9.60E-01	0.003	0.014	8.26E-01
6.58344	-0.003	0.010	8.02E-01	-0.012	0.015	4.22E-01
5.535116	0.001	0.009	9.30E-01	-0.020	0.013	1.28E-01
8.554935	0.001	0.006	8.38E-01	-0.006	0.009	4.98E-01
7.893509	0.004	0.008	6.61E-01	0.026	0.013	4.15E-02
7.686603	0.003	0.007	6.17E-01	0.003	0.010	7.58E-01
8.510526	0.003	0.009	7.13E-01	0.009	0.013	4.77E-01
7.399962	0.002	0.008	8.48E-01	-0.003	0.012	8.27E-01
0.761810864	-0.001	0.005	9.15E-01	0.001	0.008	8.94E-01
1.052825055	0.002	0.006	7.02E-01	-0.010	0.010	2.86E-01
1.757987766	0.003	0.008	7.15E-01	0.001	0.012	9.50E-01
6.0761	0.003	0.009	7.05E-01	-0.011	0.013	4.09E-01
4.187031	-0.002	0.013	8.54E-01	0.016	0.019	3.86E-01
1.836040127	0.000	0.010	9.94E-01	-0.005	0.014	7.46E-01
8.931403	0.000	0.008	9.92E-01	-0.020	0.011	7.59E-02
8.646109	0.000	0.009	9.71E-01	0.001	0.013	9.42E-01
8.855706	0.002	0.008	8.12E-01	-0.010	0.012	3.80E-01
7.084389	0.001	0.009	8.81E-01	0.016	0.014	2.46E-01
8.872191	-0.001	0.009	9.45E-01	0.020	0.013	1.33E-01
6.764441	0.004	0.009	6.72E-01	-0.009	0.014	5.45E-01
7.855828	-0.002	0.007	8.08E-01	0.007	0.011	5.58E-01
8.264258	0.001	0.010	9.36E-01	-0.017	0.015	2.48E-01
7.914368	0.002	0.009	7.89E-01	-0.029	0.013	2.14E-02
3.437796	0.000	0.010	9.89E-01	0.016	0.014	2.52E-01
5.063102	0.002	0.009	8.02E-01	-0.013	0.013	3.03E-01
3.918221	-0.002	0.010	8.76E-01	0.026	0.015	7.81E-02
8.101088	0.000	0.008	9.50E-01	-0.014	0.012	2.18E-01
6.834083	0.000	0.009	9.71E-01	0.007	0.013	5.82E-01
3.635282	-0.001	0.004	8.73E-01	0.008	0.006	1.76E-01
5.86011	-0.002	0.010	8.19E-01	-0.003	0.015	8.49E-01
0.566679961	-0.001	0.008	9.50E-01	-0.008	0.013	5.28E-01
0.790407634	-0.003	0.005	5.89E-01	0.002	0.007	8.18E-01
8.061725	0.000	0.007	9.62E-01	0.001	0.010	9.17E-01
8.714741	-0.003	0.009	7.06E-01	0.002	0.013	9.10E-01
7.00028	0.003	0.011	7.62E-01	-0.011	0.016	4.90E-01
8.697246	0.002	0.009	8.41E-01	-0.023	0.014	1.10E-01
7.404672	0.001	0.009	8.84E-01	0.017	0.013	1.98E-01
0.686113531	0.003	0.013	8.23E-01	-0.018	0.019	3.57E-01

2.543557866	0.003	0.031	9.34E-01	0.006	0.047	9.07E-01
8.582859	0.003	0.009	7.05E-01	0.014	0.013	2.63E-01
6.666539	0.000	0.008	9.63E-01	-0.007	0.012	5.28E-01
6.977067	0.000	0.011	9.95E-01	-0.007	0.016	6.46E-01
8.311022	0.002	0.008	7.59E-01	0.012	0.011	2.91E-01
2.557351603	-0.003	0.011	7.42E-01	-0.015	0.016	3.43E-01
4.179966	-0.002	0.014	9.12E-01	0.016	0.021	4.42E-01
5.994683	-0.008	0.010	4.36E-01	-0.008	0.015	5.93E-01
8.887331	-0.003	0.008	7.56E-01	-0.001	0.012	9.31E-01
3.309615	0.003	0.010	8.03E-01	-0.010	0.015	5.35E-01
7.607205	0.001	0.014	9.59E-01	0.000	0.021	9.96E-01
3.307933	-0.002	0.009	8.10E-01	0.016	0.014	2.31E-01
5.878614	0.001	0.010	8.93E-01	-0.020	0.014	1.59E-01
5.775329	0.002	0.006	8.07E-01	-0.006	0.010	5.21E-01
4.106624	-0.001	0.012	9.30E-01	0.015	0.018	3.98E-01
8.430119	0.001	0.007	8.74E-01	0.012	0.010	2.62E-01
6.701528	-0.001	0.008	8.76E-01	-0.010	0.012	4.36E-01
6.19587	0.001	0.008	8.81E-01	-0.018	0.012	1.42E-01
6.856624	0.000	0.011	9.73E-01	-0.002	0.016	8.79E-01
8.6054	0.000	0.009	9.85E-01	0.007	0.013	5.84E-01
6.188805	-0.005	0.009	5.82E-01	-0.019	0.013	1.62E-01
0.542456814	-0.003	0.009	7.28E-01	0.006	0.013	6.44E-01
6.28166	-0.001	0.009	8.75E-01	0.006	0.013	6.53E-01
2.731623685	0.000	0.010	9.77E-01	-0.015	0.015	3.07E-01
8.930058	0.000	0.008	9.79E-01	0.003	0.012	7.97E-01
7.400299	-0.002	0.009	8.45E-01	-0.001	0.013	9.38E-01
8.025727	-0.001	0.008	8.75E-01	-0.019	0.012	1.06E-01
1.134578175	0.003	0.007	6.95E-01	-0.018	0.010	8.64E-02
1.926540494	0.001	0.004	8.91E-01	0.005	0.006	3.84E-01
7.690304	-0.002	0.009	8.18E-01	0.011	0.014	4.54E-01
2.746426719	-0.003	0.009	7.24E-01	-0.015	0.013	2.57E-01
7.996794	-0.003	0.011	7.53E-01	0.003	0.016	8.41E-01
5.459083	0.001	0.010	9.07E-01	-0.011	0.015	4.60E-01
3.73352	-0.002	0.008	8.39E-01	0.006	0.012	6.42E-01
0.587202349	0.004	0.010	6.72E-01	-0.018	0.014	2.11E-01
1.457553462	-0.001	0.003	7.14E-01	0.004	0.005	3.91E-01
0.64708735	0.002	0.010	8.09E-01	-0.012	0.014	3.92E-01
5.598029	0.001	0.008	8.65E-01	-0.004	0.012	6.99E-01
0.800837044	-0.001	0.006	8.28E-01	0.004	0.009	6.10E-01
8.860753	-0.001	0.009	9.36E-01	-0.017	0.014	2.17E-01
7.347479	-0.003	0.011	7.81E-01	-0.024	0.017	1.57E-01
0.500739173	0.001	0.008	8.99E-01	-0.003	0.012	7.82E-01
1.660085882	0.000	0.004	9.30E-01	0.001	0.006	8.63E-01

2.735997309	0.000	0.010	9.71E-01	-0.013	0.015	3.85E-01
2.826497676	-0.001	0.013	9.49E-01	-0.050	0.019	8.39E-03
8.554263	0.002	0.007	7.21E-01	-0.004	0.010	6.98E-01
8.335581	0.004	0.008	6.47E-01	-0.005	0.012	6.46E-01
1.134914607	0.004	0.007	6.04E-01	-0.021	0.010	3.77E-02
6.175348	0.004	0.009	6.58E-01	-0.007	0.013	5.66E-01
8.365524	0.000	0.009	9.71E-01	-0.001	0.013	9.20E-01
2.785116467	-0.003	0.012	8.37E-01	-0.044	0.018	1.63E-02
3.319708	0.002	0.012	8.45E-01	0.001	0.018	9.69E-01
0.597968192	0.002	0.007	8.09E-01	0.009	0.011	3.93E-01
0.731195498	0.002	0.007	8.16E-01	-0.002	0.010	8.37E-01
1.819891363	-0.002	0.010	8.78E-01	0.000	0.015	9.91E-01
5.060074	0.001	0.008	8.73E-01	-0.016	0.012	1.96E-01
8.586224	0.001	0.010	9.51E-01	0.008	0.014	5.63E-01
7.155039	0.001	0.007	9.23E-01	-0.010	0.010	3.13E-01
5.974161	0.004	0.009	6.72E-01	0.007	0.013	6.11E-01
3.913175	-0.002	0.008	7.79E-01	0.027	0.012	2.13E-02
7.145956	-0.006	0.010	5.65E-01	0.001	0.016	9.26E-01
7.938254	0.001	0.008	8.87E-01	-0.010	0.012	3.86E-01
8.142469	0.002	0.008	8.32E-01	0.003	0.012	7.65E-01
2.325213115	0.003	0.008	7.54E-01	-0.012	0.012	3.32E-01
6.587478	0.001	0.008	9.32E-01	0.001	0.013	9.59E-01
1.815517739	0.004	0.009	6.53E-01	-0.006	0.013	6.61E-01
8.350721	-0.005	0.009	5.94E-01	-0.002	0.013	8.65E-01
6.858642	0.002	0.009	8.42E-01	0.010	0.014	4.83E-01
6.923237	-0.005	0.011	6.49E-01	0.002	0.016	9.15E-01
7.372038	0.002	0.010	8.26E-01	0.012	0.015	4.20E-01
8.580841	0.000	0.006	9.57E-01	0.002	0.010	8.08E-01
6.549124	0.000	0.010	9.70E-01	0.009	0.014	5.37E-01
2.076252997	0.000	0.003	9.45E-01	0.007	0.005	1.67E-01
1.46764644	0.005	0.007	5.36E-01	-0.006	0.011	6.11E-01
2.833899193	0.004	0.011	7.13E-01	-0.020	0.017	2.32E-01
6.321023	0.002	0.009	8.54E-01	0.018	0.014	1.80E-01
6.272913	0.000	0.009	9.64E-01	-0.016	0.013	2.23E-01
7.012056	0.001	0.009	9.30E-01	-0.023	0.014	1.05E-01
3.633936	0.000	0.004	9.49E-01	0.012	0.006	4.52E-02
2.782761439	-0.003	0.012	8.06E-01	-0.044	0.018	1.60E-02
6.475446	-0.003	0.009	7.24E-01	-0.011	0.014	4.32E-01
3.186817	0.001	0.006	8.13E-01	0.013	0.009	1.28E-01
6.500678	0.005	0.010	5.90E-01	0.003	0.014	8.20E-01
8.392775	0.002	0.009	8.13E-01	0.003	0.014	7.97E-01
7.817475	0.002	0.010	8.49E-01	0.016	0.014	2.75E-01
3.208685	-0.004	0.012	7.50E-01	0.009	0.018	6.13E-01

1.79230389	0.003	0.010	7.58E-01	0.014	0.014	3.48E-01
2.034198923	0.002	0.004	6.72E-01	0.008	0.006	2.03E-01
6.813897	0.000	0.011	9.67E-01	-0.009	0.016	5.56E-01
3.168986	-0.002	0.006	7.51E-01	0.008	0.009	3.99E-01
0.822032298	-0.001	0.006	9.10E-01	0.010	0.009	2.80E-01
6.109407	0.002	0.010	8.20E-01	0.000	0.015	9.97E-01
8.024381	-0.003	0.008	6.48E-01	-0.014	0.011	2.03E-01
7.290958	-0.002	0.010	8.60E-01	0.010	0.015	4.99E-01
2.727586494	-0.004	0.009	6.94E-01	-0.015	0.014	2.76E-01
3.17235	-0.001	0.006	8.01E-01	0.009	0.009	2.87E-01
3.859009	0.000	0.005	9.80E-01	0.011	0.008	1.71E-01
6.205627	-0.002	0.006	7.54E-01	0.002	0.010	8.43E-01
7.059493	-0.003	0.010	7.39E-01	-0.005	0.015	7.20E-01
6.984132	0.002	0.010	8.32E-01	0.012	0.015	4.21E-01
5.871885	0.002	0.008	8.23E-01	-0.010	0.013	4.28E-01
8.230951	-0.003	0.009	7.17E-01	0.013	0.013	3.25E-01
4.132529	-0.002	0.010	8.07E-01	0.009	0.015	5.63E-01
6.047503	-0.002	0.008	7.47E-01	-0.001	0.011	9.24E-01
8.897424	-0.003	0.008	7.30E-01	-0.013	0.013	3.12E-01
3.892989	-0.001	0.005	8.52E-01	0.019	0.007	6.91E-03
8.606073	-0.005	0.008	5.27E-01	0.001	0.011	9.58E-01
1.838731588	-0.001	0.010	9.50E-01	-0.004	0.015	7.97E-01
1.922503303	0.000	0.005	9.97E-01	-0.002	0.007	7.56E-01
6.748292	0.003	0.011	8.07E-01	-0.013	0.016	4.17E-01
1.036676291	0.001	0.005	8.72E-01	0.007	0.008	3.71E-01
7.485416	-0.002	0.010	8.64E-01	-0.002	0.015	8.71E-01
0.904794715	0.002	0.006	7.54E-01	-0.016	0.009	7.05E-02
7.603504	0.002	0.011	8.52E-01	0.022	0.016	1.65E-01
2.421096403	0.005	0.015	7.22E-01	-0.020	0.022	3.68E-01
6.644671	-0.003	0.010	7.61E-01	-0.007	0.015	6.39E-01
8.633324	-0.001	0.008	9.05E-01	0.007	0.012	5.70E-01
8.774962	-0.004	0.008	6.16E-01	-0.016	0.012	1.85E-01
6.244316	-0.001	0.009	9.44E-01	-0.017	0.013	2.08E-01
3.188163	-0.001	0.006	8.48E-01	0.011	0.009	2.10E-01
4.100568	0.002	0.010	8.09E-01	-0.005	0.015	7.40E-01
7.23511	-0.002	0.011	8.89E-01	0.014	0.017	4.17E-01
0.533036702	-0.003	0.007	7.25E-01	-0.013	0.011	2.36E-01
4.177611	-0.002	0.014	8.96E-01	0.018	0.020	3.82E-01
6.357358	0.000	0.008	9.56E-01	0.002	0.012	8.53E-01
7.908648	0.000	0.009	9.85E-01	-0.016	0.014	2.55E-01
7.895528	-0.003	0.009	7.75E-01	0.026	0.013	5.28E-02
4.217646	-0.002	0.013	9.03E-01	0.024	0.019	2.16E-01
0.638676535	-0.002	0.010	8.51E-01	-0.009	0.014	5.19E-01

2.746090286	-0.003	0.009	7.18E-01	-0.014	0.013	2.94E-01
2.363229998	0.002	0.008	8.34E-01	0.023	0.012	6.17E-02
7.278847	-0.004	0.008	6.61E-01	0.004	0.013	7.80E-01
7.470613	-0.004	0.008	5.89E-01	0.002	0.012	8.94E-01
2.783097871	-0.003	0.012	8.17E-01	-0.046	0.018	1.24E-02
0.903785417	0.002	0.006	7.13E-01	-0.019	0.009	4.33E-02
3.985172	-0.003	0.008	7.16E-01	0.008	0.012	5.11E-01
7.898219	-0.001	0.008	9.28E-01	0.003	0.013	7.83E-01
8.597662	0.000	0.008	9.62E-01	-0.013	0.013	3.07E-01
8.411279	-0.001	0.005	9.01E-01	-0.004	0.008	6.47E-01
0.595276731	-0.001	0.007	8.54E-01	-0.004	0.011	6.90E-01
5.414674	0.002	0.010	8.05E-01	-0.011	0.015	4.56E-01
6.467708	0.004	0.009	6.16E-01	-0.022	0.013	8.05E-02
8.757804	0.000	0.006	9.70E-01	-0.009	0.009	3.23E-01
1.674216051	0.000	0.004	9.69E-01	0.006	0.006	3.43E-01
5.939508	0.000	0.009	9.86E-01	-0.006	0.013	6.65E-01
6.348274	-0.003	0.008	6.91E-01	-0.028	0.012	2.07E-02
7.138554	0.001	0.009	9.24E-01	-0.012	0.014	4.13E-01
8.152226	-0.004	0.009	6.77E-01	-0.001	0.013	9.52E-01
3.733856	-0.005	0.008	5.69E-01	0.003	0.012	8.14E-01
6.195197	0.000	0.010	9.94E-01	-0.010	0.015	5.06E-01
7.573225	-0.001	0.007	9.20E-01	-0.010	0.010	3.38E-01
0.724130414	0.000	0.007	9.84E-01	0.019	0.011	7.96E-02
8.267286	-0.003	0.008	6.96E-01	-0.021	0.012	8.99E-02
0.991257891	-0.001	0.007	8.53E-01	0.014	0.010	1.94E-01
7.972571	-0.001	0.008	9.17E-01	0.010	0.011	3.96E-01
5.789123	0.001	0.008	8.68E-01	-0.008	0.013	5.20E-01
5.530743	-0.001	0.008	8.71E-01	-0.003	0.012	8.36E-01
7.186328	0.004	0.009	6.78E-01	-0.018	0.014	1.84E-01
7.699387	-0.002	0.009	7.92E-01	0.022	0.013	8.52E-02
8.167701	-0.001	0.010	8.77E-01	-0.004	0.014	7.93E-01
4.216974	-0.003	0.012	8.17E-01	0.008	0.017	6.55E-01
2.164734769	0.002	0.011	8.21E-01	-0.016	0.016	3.16E-01
8.149198	-0.001	0.007	9.32E-01	0.007	0.011	5.03E-01
6.699846	0.002	0.009	8.12E-01	0.014	0.013	3.01E-01
0.795790555	-0.003	0.006	5.88E-01	0.002	0.008	8.53E-01
6.573011	-0.003	0.007	6.12E-01	-0.010	0.010	3.13E-01
5.666662	0.001	0.009	9.01E-01	0.001	0.014	9.52E-01
6.507407	-0.004	0.009	6.16E-01	-0.007	0.013	6.12E-01
7.688285	0.000	0.009	9.81E-01	0.006	0.013	6.68E-01
0.982847076	0.003	0.007	6.42E-01	0.006	0.010	5.31E-01
7.746488	0.004	0.011	6.73E-01	-0.004	0.016	8.07E-01
7.566496	0.001	0.007	8.95E-01	0.001	0.010	8.92E-01

6.710948	0.004	0.009	6.90E-01	-0.004	0.013	7.91E-01
6.865371	0.002	0.008	8.18E-01	0.029	0.013	2.24E-02
6.230186	-0.003	0.008	7.19E-01	-0.014	0.013	2.51E-01
7.396934	0.000	0.007	9.50E-01	-0.008	0.011	4.44E-01
8.039521	-0.002	0.009	8.39E-01	-0.008	0.013	5.59E-01
5.890726	0.000	0.007	9.69E-01	0.007	0.010	5.22E-01
3.895008	0.001	0.005	8.55E-01	0.020	0.007	4.42E-03
6.57974	-0.005	0.009	5.68E-01	-0.017	0.014	2.11E-01
2.304354294	-0.002	0.005	7.23E-01	-0.003	0.007	6.59E-01
2.540866406	0.002	0.025	9.38E-01	0.023	0.037	5.33E-01
8.580168	0.001	0.010	9.21E-01	-0.007	0.015	6.37E-01
7.444371	0.004	0.008	6.38E-01	0.014	0.012	2.41E-01
8.091331	-0.001	0.007	8.53E-01	-0.003	0.011	7.97E-01
8.726516	0.000	0.008	9.76E-01	-0.005	0.012	6.44E-01
7.867267	0.002	0.008	8.21E-01	-0.003	0.011	8.13E-01
5.611487	0.003	0.010	7.39E-01	-0.033	0.014	2.20E-02
5.698959	0.004	0.010	6.82E-01	-0.021	0.014	1.44E-01
8.847968	-0.002	0.007	7.83E-01	0.002	0.011	8.64E-01
5.988628	-0.001	0.009	8.89E-01	-0.004	0.014	7.45E-01
6.487557	0.002	0.006	7.54E-01	-0.003	0.009	7.75E-01
0.824723758	-0.003	0.007	6.30E-01	0.006	0.010	5.48E-01
6.274595	0.005	0.010	6.09E-01	-0.013	0.014	3.58E-01
1.79869611	0.000	0.010	9.80E-01	-0.004	0.015	8.06E-01
0.604360411	-0.001	0.008	9.13E-01	-0.025	0.012	4.24E-02
8.730217	0.003	0.009	7.17E-01	-0.022	0.013	9.57E-02
8.397149	0.003	0.007	6.92E-01	-0.006	0.010	5.66E-01
6.807841	-0.001	0.010	9.50E-01	0.012	0.015	4.27E-01
8.210428	0.002	0.009	8.70E-01	-0.009	0.014	5.04E-01
7.072277	-0.001	0.008	8.87E-01	-0.021	0.012	7.50E-02
0.533709567	0.002	0.009	8.03E-01	-0.014	0.013	3.11E-01
8.586897	0.000	0.008	9.78E-01	0.002	0.011	8.72E-01
2.731287252	0.000	0.010	9.97E-01	-0.015	0.015	3.13E-01
8.956299	0.002	0.007	8.09E-01	-0.006	0.010	5.48E-01
6.298146	-0.001	0.007	9.30E-01	0.017	0.011	1.18E-01
7.022821	-0.002	0.009	8.04E-01	-0.033	0.014	1.69E-02
8.364851	-0.003	0.008	7.37E-01	0.001	0.013	9.20E-01
7.625709	0.001	0.012	8.99E-01	0.006	0.017	7.37E-01
7.364973	0.001	0.008	9.32E-01	-0.007	0.011	5.15E-01
5.293558	0.002	0.011	8.55E-01	-0.026	0.016	1.00E-01
4.357939	-0.003	0.014	8.30E-01	0.015	0.021	4.73E-01
7.34647	-0.001	0.011	9.30E-01	0.014	0.017	3.94E-01
4.112007	0.002	0.010	8.75E-01	-0.007	0.015	6.57E-01
5.293221	0.002	0.011	8.63E-01	-0.026	0.016	1.00E-01

7.970888	-0.002	0.008	7.76E-01	-0.012	0.012	3.10E-01
1.758660631	-0.001	0.008	8.98E-01	0.010	0.013	4.30E-01
5.632345	0.001	0.009	9.10E-01	-0.024	0.014	8.43E-02
6.023617	0.003	0.009	7.50E-01	-0.008	0.013	5.65E-01
4.230431	-0.001	0.013	9.16E-01	0.012	0.019	5.29E-01
3.633599	0.000	0.004	9.52E-01	0.012	0.006	3.34E-02
7.849436	-0.003	0.009	7.88E-01	0.015	0.014	2.85E-01
5.705688	0.001	0.010	8.95E-01	-0.006	0.015	6.88E-01
6.638952	-0.003	0.009	7.72E-01	-0.002	0.014	8.83E-01
6.321359	0.007	0.010	4.97E-01	0.019	0.015	1.91E-01
8.170057	0.000	0.010	9.94E-01	0.000	0.015	9.95E-01
3.514502	-0.002	0.008	8.26E-01	0.018	0.012	1.32E-01
8.673696	0.003	0.010	7.25E-01	-0.002	0.014	8.73E-01
8.955627	0.002	0.007	8.04E-01	0.005	0.010	6.34E-01
3.895344	0.001	0.005	8.55E-01	0.019	0.007	7.38E-03
7.773402	0.002	0.012	8.85E-01	-0.005	0.018	7.96E-01
8.846622	0.002	0.007	8.30E-01	0.003	0.011	7.62E-01
7.438652	0.006	0.009	4.96E-01	0.011	0.013	3.82E-01
8.163664	0.002	0.007	8.03E-01	0.027	0.011	1.11E-02
8.350048	-0.003	0.009	7.19E-01	-0.007	0.013	6.20E-01
8.527012	0.002	0.007	8.24E-01	-0.001	0.011	9.05E-01
6.44954	0.002	0.008	7.71E-01	-0.003	0.012	8.07E-01
3.172014	-0.002	0.006	7.45E-01	0.008	0.009	3.87E-01
1.884822853	-0.004	0.008	6.31E-01	-0.010	0.012	4.14E-01
7.977281	-0.001	0.008	8.80E-01	0.009	0.012	4.59E-01
7.86794	0.002	0.008	7.66E-01	-0.009	0.012	4.50E-01
0.572735747	0.005	0.010	6.22E-01	0.024	0.016	1.29E-01
5.749424	-0.004	0.009	7.08E-01	0.011	0.014	4.30E-01
5.886352	-0.002	0.008	8.11E-01	-0.011	0.012	3.44E-01
1.850506729	0.004	0.008	6.40E-01	0.013	0.012	2.92E-01
8.105461	-0.001	0.007	8.88E-01	-0.017	0.011	1.29E-01
3.335857	0.001	0.010	9.21E-01	0.025	0.014	7.68E-02
2.269701737	0.002	0.006	6.96E-01	0.014	0.009	1.12E-01
8.148525	0.002	0.010	8.09E-01	0.010	0.015	5.19E-01
3.33552	0.002	0.009	8.37E-01	0.021	0.014	1.30E-01
7.152012	-0.001	0.007	8.43E-01	-0.009	0.011	3.91E-01
4.00704	0.000	0.011	9.85E-01	0.014	0.016	3.74E-01
6.044812	0.002	0.007	8.22E-01	-0.009	0.011	4.42E-01
2.595368485	0.004	0.020	8.44E-01	0.000	0.030	9.93E-01
8.224895	-0.003	0.008	6.81E-01	-0.016	0.012	1.87E-01
5.830841	-0.002	0.008	8.46E-01	0.003	0.012	8.22E-01
3.191863	-0.001	0.007	9.17E-01	0.017	0.011	1.01E-01
1.460581356	0.001	0.004	8.49E-01	0.002	0.005	6.97E-01

3.333502	0.001	0.009	9.19E-01	0.011	0.013	3.96E-01
8.331208	0.000	0.009	9.97E-01	0.000	0.013	9.94E-01
7.084052	0.001	0.009	9.45E-01	0.018	0.014	1.86E-01
6.601608	-0.004	0.008	6.37E-01	0.002	0.012	8.91E-01
7.603168	0.004	0.012	7.27E-01	0.032	0.017	6.51E-02
1.889196477	0.000	0.007	9.54E-01	0.002	0.010	8.83E-01
7.032914	-0.003	0.009	7.61E-01	0.001	0.014	9.22E-01
6.535667	-0.004	0.008	5.94E-01	-0.002	0.012	8.63E-01
6.452568	0.000	0.007	9.75E-01	-0.010	0.010	3.31E-01
1.469328603	0.004	0.007	5.63E-01	-0.001	0.010	9.14E-01
8.650146	0.001	0.007	9.01E-01	0.009	0.011	3.95E-01
8.96101	-0.007	0.009	4.32E-01	0.005	0.014	6.97E-01
5.603412	0.001	0.010	9.21E-01	-0.001	0.015	9.55E-01
0.730859065	-0.001	0.007	8.99E-01	0.000	0.011	9.96E-01
6.657119	0.003	0.010	7.67E-01	-0.005	0.015	7.44E-01
5.829495	-0.001	0.009	8.83E-01	0.012	0.014	3.90E-01
4.121763	0.003	0.010	7.38E-01	0.017	0.015	2.63E-01
6.405131	0.002	0.011	8.34E-01	0.003	0.016	8.31E-01
5.792824	-0.001	0.009	8.78E-01	0.011	0.014	4.22E-01
5.602067	0.004	0.010	7.14E-01	0.017	0.015	2.65E-01
6.3439	0.001	0.007	8.91E-01	0.000	0.010	9.99E-01
8.875892	0.002	0.008	8.05E-01	0.000	0.012	9.72E-01
0.597295327	0.004	0.007	5.52E-01	0.006	0.011	5.85E-01
2.812367507	-0.002	0.015	8.91E-01	-0.061	0.023	6.80E-03
6.776553	-0.001	0.007	8.92E-01	-0.016	0.011	1.59E-01
5.504838	-0.003	0.008	7.56E-01	-0.026	0.012	3.80E-02
8.622222	-0.002	0.008	7.71E-01	0.006	0.013	6.53E-01
6.909444	0.003	0.007	7.16E-01	-0.006	0.011	6.01E-01
6.94847	-0.001	0.009	8.80E-01	0.002	0.013	8.58E-01
8.886994	-0.002	0.010	8.18E-01	0.001	0.015	9.24E-01
5.482633	0.000	0.007	1.00E+00	0.008	0.011	4.59E-01
1.479758013	0.004	0.007	5.66E-01	-0.007	0.011	5.40E-01
5.763891	0.000	0.009	9.58E-01	-0.008	0.014	5.36E-01
5.430822	0.002	0.008	7.88E-01	-0.010	0.011	3.90E-01
7.296678	-0.001	0.010	9.07E-01	0.009	0.015	5.69E-01
8.31405	-0.003	0.006	6.80E-01	-0.002	0.010	8.32E-01
6.904061	0.001	0.011	9.00E-01	0.021	0.016	1.89E-01
3.89669	0.001	0.005	8.81E-01	0.018	0.007	8.50E-03
5.836224	0.003	0.010	7.62E-01	0.005	0.016	7.28E-01
7.624363	0.001	0.012	9.07E-01	0.001	0.018	9.72E-01
6.174675	0.002	0.007	7.69E-01	-0.004	0.010	7.06E-01
6.480156	0.000	0.008	9.63E-01	0.002	0.012	8.51E-01
7.179263	0.003	0.010	7.85E-01	0.005	0.014	7.19E-01

6.408495	0.003	0.008	7.02E-01	-0.006	0.013	6.47E-01
8.666631	-0.001	0.007	9.08E-01	-0.011	0.010	2.80E-01
2.036553952	0.002	0.004	7.00E-01	0.010	0.006	1.17E-01
4.088793	0.006	0.010	5.52E-01	-0.002	0.015	8.89E-01
1.770435772	0.001	0.010	9.22E-01	0.018	0.015	2.29E-01
8.785728	-0.005	0.010	6.04E-01	0.011	0.015	4.51E-01
8.073164	-0.001	0.009	9.30E-01	0.001	0.014	9.45E-01
8.160973	0.000	0.009	9.82E-01	0.000	0.013	9.79E-01
7.05882	0.001	0.011	9.25E-01	-0.005	0.016	7.41E-01
7.727311	0.002	0.010	7.96E-01	0.007	0.014	6.06E-01
7.350507	-0.002	0.010	8.25E-01	-0.002	0.015	8.76E-01
6.112435	-0.003	0.008	7.21E-01	-0.004	0.011	7.48E-01
5.763554	-0.001	0.009	9.49E-01	-0.005	0.013	7.33E-01
8.921647	-0.002	0.010	8.70E-01	0.025	0.014	8.16E-02
3.315334	0.000	0.011	9.76E-01	0.005	0.016	7.74E-01
4.029917	-0.001	0.014	9.20E-01	-0.005	0.021	8.22E-01
5.918986	0.001	0.010	9.41E-01	-0.005	0.015	7.54E-01
4.189386	-0.001	0.013	9.24E-01	0.020	0.019	2.84E-01
3.931342	0.002	0.007	7.55E-01	0.017	0.011	1.20E-01
0.976791289	0.000	0.007	9.62E-01	0.013	0.010	2.00E-01
3.898035	-0.001	0.005	8.54E-01	0.017	0.007	2.01E-02
2.813713237	0.003	0.015	8.24E-01	-0.070	0.023	2.51E-03
5.841607	-0.001	0.008	8.59E-01	-0.023	0.012	5.04E-02
1.700794226	-0.001	0.004	8.96E-01	0.007	0.006	2.56E-01
5.952629	-0.001	0.008	9.01E-01	0.004	0.012	7.41E-01
6.168619	-0.004	0.009	6.61E-01	0.007	0.014	6.26E-01
2.363902863	0.000	0.010	9.91E-01	0.033	0.015	2.69E-02
8.885312	0.000	0.009	9.98E-01	0.012	0.013	3.57E-01
7.283557	0.001	0.010	8.79E-01	-0.004	0.014	7.69E-01
5.005235	0.001	0.011	9.02E-01	-0.006	0.016	7.22E-01
5.487007	-0.001	0.007	8.78E-01	-0.010	0.011	3.58E-01
8.67235	-0.001	0.007	8.93E-01	0.006	0.011	5.59E-01
1.772454368	0.001	0.008	9.16E-01	0.043	0.012	3.10E-04
2.649534133	-0.004	0.022	8.44E-01	0.012	0.033	7.18E-01
2.784107169	-0.002	0.012	8.44E-01	-0.048	0.018	7.99E-03
6.269885	0.002	0.008	7.82E-01	0.016	0.011	1.69E-01
2.743062393	0.000	0.010	9.90E-01	-0.016	0.015	3.00E-01
2.074234402	0.000	0.003	9.92E-01	0.007	0.005	1.36E-01
8.427427	0.003	0.010	7.46E-01	0.010	0.015	5.07E-01
7.44168	0.004	0.010	7.19E-01	0.017	0.015	2.61E-01
7.702752	-0.001	0.008	8.69E-01	-0.009	0.012	4.48E-01
4.214619	-0.002	0.012	8.67E-01	0.006	0.018	7.23E-01
1.50398116	0.000	0.006	9.45E-01	-0.014	0.008	8.88E-02

2.82313335	-0.002	0.014	8.69E-01	-0.054	0.021	1.04E-02
7.548329	-0.002	0.009	8.62E-01	-0.006	0.014	6.50E-01
3.641001	0.000	0.004	9.84E-01	0.008	0.005	1.55E-01
3.986854	-0.002	0.007	7.35E-01	0.011	0.011	3.08E-01
2.813040372	0.003	0.015	8.36E-01	-0.057	0.023	1.22E-02
8.775299	-0.004	0.009	6.55E-01	-0.031	0.013	2.21E-02
4.295026	-0.001	0.012	9.21E-01	0.013	0.018	4.88E-01
8.316068	0.001	0.009	8.73E-01	-0.007	0.013	6.02E-01
2.836590653	0.006	0.010	5.47E-01	-0.012	0.015	4.41E-01
8.50548	-0.001	0.010	9.25E-01	-0.001	0.014	9.39E-01
8.146843	-0.001	0.007	9.10E-01	0.008	0.010	4.20E-01
8.075519	0.004	0.009	6.69E-01	0.008	0.013	5.28E-01
2.163725471	0.002	0.011	8.47E-01	-0.025	0.017	1.45E-01
2.0752437	0.001	0.003	7.19E-01	0.007	0.005	1.47E-01
1.882467825	0.002	0.008	7.67E-01	-0.007	0.012	5.69E-01
7.706116	-0.001	0.011	9.50E-01	-0.003	0.017	8.44E-01
7.546647	0.000	0.010	9.70E-01	-0.014	0.015	3.25E-01
2.276093956	0.000	0.015	9.88E-01	0.011	0.023	6.40E-01
6.092922	0.001	0.008	8.89E-01	0.003	0.012	8.11E-01
8.381	-0.003	0.008	6.86E-01	0.017	0.012	1.59E-01
7.502911	0.001	0.009	9.28E-01	-0.019	0.013	1.62E-01
1.743184732	-0.003	0.006	6.52E-01	0.000	0.009	9.70E-01
8.991288	-0.005	0.008	5.21E-01	0.001	0.012	9.24E-01
8.708012	-0.001	0.009	8.73E-01	-0.020	0.013	1.26E-01
4.093839	0.001	0.012	9.17E-01	0.017	0.018	3.44E-01
5.448653	0.003	0.006	6.00E-01	0.003	0.010	7.47E-01
3.893325	-0.001	0.005	8.72E-01	0.018	0.007	7.52E-03
5.562368	0.001	0.010	9.01E-01	-0.008	0.016	5.89E-01
1.503644727	0.001	0.005	8.74E-01	-0.013	0.008	1.04E-01
3.666906	0.000	0.005	9.68E-01	0.011	0.007	1.17E-01
8.73089	0.002	0.008	7.78E-01	-0.022	0.011	4.98E-02
8.83552	-0.001	0.008	9.33E-01	-0.001	0.011	9.63E-01
1.500953266	0.000	0.005	9.76E-01	-0.009	0.008	2.62E-01
8.088303	0.000	0.009	9.96E-01	-0.001	0.014	9.70E-01
1.845123807	-0.001	0.009	8.85E-01	0.009	0.014	5.10E-01
2.152286763	0.001	0.008	9.12E-01	0.002	0.012	8.98E-01
3.371518	0.005	0.011	6.68E-01	0.011	0.017	5.14E-01
8.711377	0.000	0.008	9.89E-01	-0.001	0.012	9.23E-01
7.41712	0.000	0.008	9.64E-01	-0.009	0.012	4.80E-01
5.98089	0.000	0.007	9.84E-01	0.004	0.011	7.10E-01
3.43645	0.002	0.014	8.87E-01	0.017	0.020	3.97E-01
7.517377	0.003	0.007	7.14E-01	-0.021	0.010	4.29E-02
3.928651	0.003	0.008	7.11E-01	0.018	0.012	1.42E-01

3.604666	-0.002	0.008	7.84E-01	-0.003	0.013	7.88E-01
7.433606	-0.004	0.010	6.66E-01	-0.010	0.015	4.83E-01
6.036065	0.000	0.008	9.89E-01	-0.019	0.012	1.11E-01
8.848305	-0.001	0.008	8.54E-01	0.002	0.011	8.68E-01
6.772179	0.002	0.010	8.23E-01	-0.010	0.016	5.33E-01
5.689203	0.001	0.007	8.94E-01	-0.011	0.010	2.47E-01
3.891643	-0.001	0.005	8.77E-01	0.019	0.007	6.16E-03
7.790561	-0.002	0.008	8.22E-01	0.016	0.012	1.86E-01
7.601822	0.004	0.011	7.02E-01	0.012	0.017	4.73E-01
8.411615	0.001	0.005	8.85E-01	-0.002	0.008	8.15E-01
6.419261	0.001	0.007	8.80E-01	0.008	0.010	4.09E-01
1.031293369	0.000	0.006	9.68E-01	0.016	0.009	8.25E-02
6.374179	0.000	0.007	9.74E-01	-0.011	0.011	3.32E-01
2.278112552	-0.002	0.007	7.94E-01	-0.001	0.010	8.90E-01
0.684094935	0.004	0.014	7.46E-01	-0.012	0.020	5.40E-01
6.603963	-0.001	0.011	9.23E-01	-0.003	0.016	8.50E-01
4.206208	0.000	0.014	9.93E-01	0.018	0.021	3.92E-01
7.111303	0.002	0.007	8.16E-01	-0.010	0.011	3.57E-01
7.922106	0.001	0.008	8.76E-01	0.004	0.012	7.64E-01
1.000341571	0.001	0.007	8.85E-01	-0.001	0.010	8.94E-01
7.409382	-0.001	0.008	9.07E-01	-0.008	0.011	4.65E-01
6.111425	0.001	0.008	9.23E-01	-0.019	0.012	9.96E-02
6.280988	0.001	0.009	9.49E-01	-0.004	0.013	7.70E-01
0.566007096	0.004	0.008	6.03E-01	0.000	0.012	9.84E-01
8.373598	0.000	0.009	9.80E-01	0.015	0.013	2.44E-01
4.292671	0.003	0.012	8.25E-01	0.013	0.017	4.70E-01
8.649809	0.002	0.007	8.19E-01	0.010	0.010	3.06E-01
4.101241	0.004	0.010	6.93E-01	0.007	0.015	6.72E-01
4.038328	-0.001	0.014	9.44E-01	-0.012	0.021	5.67E-01
6.30151	0.004	0.009	6.93E-01	-0.010	0.014	4.63E-01
6.10907	0.002	0.010	8.49E-01	0.011	0.015	4.85E-01
6.938713	0.001	0.010	8.83E-01	0.006	0.014	6.74E-01
6.141704	-0.002	0.009	8.49E-01	-0.019	0.014	1.72E-01
2.588303401	0.007	0.011	5.66E-01	0.012	0.017	4.84E-01
3.207003	0.001	0.010	9.48E-01	0.013	0.016	3.91E-01
7.897883	-0.001	0.009	9.14E-01	-0.007	0.014	6.07E-01
6.392683	-0.005	0.011	6.75E-01	-0.017	0.016	3.02E-01
0.52496232	-0.001	0.009	8.67E-01	-0.007	0.013	6.08E-01
6.328088	-0.003	0.010	7.41E-01	-0.012	0.015	4.27E-01
8.026063	-0.001	0.007	8.67E-01	-0.013	0.011	2.14E-01
8.864117	-0.001	0.008	9.22E-01	0.009	0.013	4.73E-01
2.174491314	0.003	0.010	7.75E-01	-0.013	0.015	4.06E-01
8.531722	0.000	0.010	9.92E-01	0.017	0.014	2.37E-01

6.248017	0.002	0.007	7.85E-01	-0.012	0.010	2.09E-01
7.727984	0.001	0.008	8.72E-01	-0.003	0.012	8.18E-01
2.164398336	-0.001	0.011	9.36E-01	-0.017	0.016	2.94E-01
6.050531	0.002	0.009	8.37E-01	-0.005	0.014	6.98E-01
5.912257	0.000	0.007	9.70E-01	-0.006	0.011	5.82E-01
7.085061	0.000	0.006	9.42E-01	-0.006	0.009	4.97E-01
7.232755	0.000	0.010	9.83E-01	0.000	0.014	9.78E-01
8.566711	-0.003	0.007	6.90E-01	-0.004	0.010	6.50E-01
7.922779	0.000	0.008	1.00E+00	-0.004	0.012	7.51E-01
6.93434	0.000	0.010	9.91E-01	-0.003	0.014	8.20E-01
6.520528	0.001	0.008	8.70E-01	0.012	0.012	3.00E-01
7.552703	-0.001	0.007	8.59E-01	-0.017	0.011	1.24E-01
6.006795	0.002	0.010	8.45E-01	0.007	0.014	6.17E-01
8.473182	-0.002	0.010	8.20E-01	-0.031	0.015	3.67E-02
6.418588	-0.001	0.008	8.89E-01	0.001	0.012	9.34E-01
6.427336	0.003	0.008	6.63E-01	0.005	0.011	6.41E-01
8.871855	-0.003	0.009	7.34E-01	0.013	0.013	3.08E-01
3.985508	-0.003	0.008	7.19E-01	0.009	0.012	4.50E-01
0.558942011	0.000	0.009	9.59E-01	-0.006	0.013	6.46E-01
5.966759	0.001	0.009	8.76E-01	-0.010	0.013	4.25E-01
3.750678	-0.004	0.011	7.44E-01	0.041	0.016	1.14E-02
2.841637142	0.001	0.009	8.85E-01	-0.007	0.013	6.11E-01
5.510557	-0.002	0.008	7.41E-01	-0.007	0.011	5.59E-01
2.548267923	-0.003	0.013	7.96E-01	-0.023	0.020	2.44E-01
0.641031563	0.000	0.010	9.86E-01	-0.023	0.015	1.15E-01
6.383263	-0.002	0.007	7.41E-01	-0.008	0.010	4.45E-01
6.197216	0.000	0.007	9.99E-01	0.006	0.011	6.03E-01
3.299185	0.000	0.012	9.95E-01	0.021	0.019	2.69E-01
3.182443	0.002	0.005	7.11E-01	0.013	0.008	8.58E-02
8.297564	0.000	0.007	9.75E-01	-0.003	0.010	7.77E-01
3.995937	-0.002	0.009	8.23E-01	0.021	0.013	1.20E-01
1.792976756	0.000	0.009	9.80E-01	0.022	0.014	1.03E-01
8.482939	0.000	0.007	9.70E-01	-0.012	0.011	2.56E-01
8.141123	0.002	0.009	8.32E-01	0.010	0.013	4.45E-01
8.572093	-0.001	0.008	8.82E-01	0.017	0.011	1.43E-01
5.738995	0.002	0.009	8.17E-01	-0.012	0.014	4.01E-01
1.751931979	0.002	0.007	7.77E-01	0.001	0.011	9.57E-01
3.436786	-0.002	0.012	9.03E-01	0.015	0.019	4.22E-01
6.445503	0.004	0.009	6.38E-01	-0.017	0.014	2.18E-01
3.169322	-0.002	0.006	7.67E-01	0.007	0.009	4.33E-01
7.854819	0.001	0.007	9.31E-01	0.000	0.010	9.85E-01
7.234437	-0.002	0.010	8.34E-01	0.022	0.015	1.43E-01
3.858673	0.000	0.006	9.98E-01	0.009	0.008	2.53E-01

6.819953	-0.001	0.009	9.21E-01	0.007	0.014	6.24E-01
2.6491977	-0.005	0.024	8.25E-01	0.010	0.036	7.77E-01
3.329464	0.001	0.010	9.27E-01	0.017	0.015	2.58E-01
6.911126	0.003	0.007	6.58E-01	0.003	0.010	7.84E-01
6.127238	0.000	0.008	9.74E-01	-0.002	0.011	8.30E-01
3.318026	-0.002	0.012	8.44E-01	0.010	0.018	5.91E-01
1.67152459	0.000	0.004	9.21E-01	0.002	0.006	6.65E-01
6.553161	0.004	0.009	6.55E-01	-0.017	0.013	1.89E-01
1.91947541	0.000	0.005	9.50E-01	-0.010	0.008	2.18E-01
8.344329	0.001	0.010	8.85E-01	0.017	0.015	2.55E-01
7.596439	0.002	0.008	8.07E-01	0.012	0.012	3.29E-01
6.115463	0.001	0.008	8.82E-01	0.032	0.012	8.24E-03
6.026981	-0.003	0.009	7.20E-01	-0.024	0.014	7.48E-02
6.098977	-0.001	0.009	9.09E-01	-0.004	0.014	7.67E-01
6.290744	-0.003	0.011	8.03E-01	-0.005	0.016	7.31E-01
8.26594	0.001	0.009	8.94E-01	-0.024	0.013	6.47E-02
6.675959	-0.001	0.007	9.36E-01	0.011	0.011	3.10E-01
4.142958	-0.002	0.013	8.74E-01	0.006	0.020	7.76E-01
8.628278	-0.005	0.008	5.82E-01	-0.010	0.012	4.20E-01
1.674552483	0.000	0.004	9.79E-01	0.003	0.006	6.03E-01
6.614729	-0.003	0.009	7.74E-01	0.005	0.013	7.12E-01
7.088426	0.003	0.010	7.48E-01	-0.002	0.015	9.11E-01
6.333471	0.000	0.010	9.65E-01	0.013	0.016	3.96E-01
2.364239295	0.001	0.011	9.21E-01	0.036	0.016	2.38E-02
8.206055	-0.001	0.007	9.30E-01	-0.001	0.010	9.57E-01
6.283006	0.001	0.008	9.45E-01	0.008	0.012	4.93E-01
7.703088	0.002	0.007	7.92E-01	0.010	0.010	3.32E-01
6.547106	-0.002	0.009	8.15E-01	-0.002	0.014	8.75E-01
6.256764	0.001	0.009	9.55E-01	-0.009	0.013	4.81E-01
6.375862	0.002	0.007	7.65E-01	-0.009	0.010	3.92E-01
1.703822119	-0.001	0.004	8.83E-01	0.003	0.007	6.68E-01
7.001626	0.001	0.007	9.31E-01	-0.008	0.010	4.28E-01
7.306434	-0.002	0.011	8.25E-01	0.014	0.017	4.01E-01
6.97673	0.000	0.011	9.91E-01	0.000	0.016	9.98E-01
3.753369	-0.001	0.011	9.05E-01	0.004	0.017	8.00E-01
6.755357	0.001	0.007	8.59E-01	0.001	0.011	9.34E-01
7.668772	0.000	0.010	9.97E-01	-0.017	0.016	2.69E-01
5.915285	0.000	0.010	9.83E-01	-0.017	0.015	2.56E-01
6.634242	0.002	0.010	8.46E-01	-0.024	0.015	1.03E-01
5.799889	-0.002	0.009	8.31E-01	-0.015	0.013	2.34E-01
5.988964	0.001	0.008	9.13E-01	-0.002	0.012	8.44E-01
8.57714	-0.002	0.009	8.24E-01	-0.012	0.013	3.80E-01
6.04212	0.000	0.007	9.48E-01	0.024	0.011	3.01E-02

7.726638	0.002	0.009	8.52E-01	-0.004	0.014	7.53E-01
8.560318	0.002	0.008	8.46E-01	0.001	0.012	9.43E-01
7.013065	0.002	0.010	8.36E-01	-0.019	0.015	1.97E-01
0.761474431	-0.001	0.005	8.68E-01	0.003	0.008	7.04E-01
7.581636	0.003	0.009	7.64E-01	0.014	0.013	2.97E-01
1.132896012	0.000	0.008	9.76E-01	-0.016	0.012	1.61E-01
5.604422	0.001	0.011	9.33E-01	-0.004	0.016	8.23E-01
5.991319	0.001	0.008	9.41E-01	-0.013	0.012	2.64E-01
1.132559579	0.001	0.008	9.24E-01	-0.013	0.011	2.65E-01
7.927825	-0.002	0.009	8.22E-01	-0.008	0.014	5.46E-01
7.686266	0.001	0.007	9.05E-01	0.001	0.010	9.26E-01
5.289184	-0.002	0.009	8.04E-01	-0.025	0.014	7.06E-02
7.65296	0.004	0.009	6.69E-01	0.017	0.014	2.19E-01
8.383018	0.003	0.009	7.10E-01	-0.020	0.014	1.43E-01
7.160422	0.004	0.009	6.78E-01	0.023	0.013	8.14E-02
8.981532	0.002	0.008	7.54E-01	-0.003	0.012	8.28E-01
3.997283	0.001	0.008	9.34E-01	0.018	0.013	1.53E-01
8.886658	0.000	0.008	9.64E-01	-0.003	0.012	7.94E-01
0.679721311	-0.003	0.011	7.76E-01	-0.034	0.017	4.60E-02
3.332829	-0.003	0.010	7.42E-01	0.005	0.015	7.44E-01
7.255296	-0.004	0.011	7.24E-01	0.011	0.016	5.07E-01
6.942414	-0.001	0.008	8.76E-01	0.009	0.012	4.59E-01
0.544811842	0.001	0.009	8.91E-01	0.022	0.013	1.05E-01
1.189080254	-0.002	0.013	8.50E-01	-0.001	0.019	9.43E-01
0.548176168	0.003	0.010	7.58E-01	-0.001	0.015	9.70E-01
6.013187	0.002	0.009	7.85E-01	0.008	0.013	5.64E-01
6.12421	-0.002	0.009	8.66E-01	-0.018	0.014	2.06E-01
7.436633	0.000	0.010	9.65E-01	0.001	0.014	9.25E-01
8.866472	0.001	0.007	9.26E-01	0.003	0.011	7.58E-01
2.731960117	0.000	0.010	9.79E-01	-0.015	0.015	2.92E-01
6.356012	-0.001	0.008	9.18E-01	-0.002	0.012	8.64E-01
6.599926	-0.001	0.008	8.68E-01	-0.001	0.012	9.21E-01
2.5284184	-0.001	0.012	9.03E-01	0.007	0.018	6.78E-01
0.855339124	-0.001	0.007	8.92E-01	-0.010	0.011	3.78E-01
6.179721	-0.001	0.009	9.29E-01	-0.033	0.013	9.76E-03
5.825458	0.002	0.010	8.05E-01	0.012	0.015	4.11E-01
8.93645	-0.002	0.010	8.77E-01	-0.015	0.015	2.93E-01
2.364575728	0.002	0.012	8.81E-01	0.037	0.018	3.73E-02
2.163052606	-0.002	0.011	8.94E-01	-0.019	0.017	2.64E-01
7.505939	-0.002	0.008	8.46E-01	0.001	0.012	9.24E-01
6.659138	-0.001	0.009	9.10E-01	0.005	0.013	6.89E-01
2.816404698	-0.004	0.015	7.80E-01	-0.063	0.023	5.90E-03
8.507835	0.002	0.009	8.60E-01	0.035	0.013	8.49E-03

7.434951	-0.005	0.011	6.24E-01	-0.007	0.016	6.47E-01
1.920484708	-0.001	0.006	8.35E-01	-0.010	0.009	2.55E-01
7.597448	0.003	0.010	7.42E-01	-0.002	0.015	9.01E-01
3.936052	0.002	0.006	7.63E-01	0.022	0.009	2.31E-02
7.154367	0.001	0.007	8.44E-01	-0.014	0.010	1.62E-01
7.654305	0.002	0.008	7.71E-01	0.001	0.012	9.40E-01
0.746671397	0.001	0.006	8.51E-01	-0.004	0.008	6.37E-01
1.03398483	0.001	0.005	8.80E-01	0.005	0.007	4.69E-01
2.162043308	-0.002	0.010	8.55E-01	-0.012	0.016	4.47E-01
0.754072914	-0.001	0.005	8.26E-01	-0.009	0.008	2.75E-01
6.998598	0.000	0.008	9.70E-01	-0.005	0.012	6.53E-01
3.649075	0.001	0.004	7.74E-01	0.012	0.006	5.41E-02
0.724803279	0.001	0.007	9.07E-01	0.010	0.011	3.59E-01
2.073225104	0.000	0.003	9.88E-01	0.009	0.005	5.76E-02
6.041111	0.000	0.008	9.66E-01	0.010	0.011	3.82E-01
5.622253	-0.001	0.010	9.47E-01	0.004	0.015	7.70E-01
0.82236873	-0.001	0.006	8.81E-01	0.010	0.009	3.07E-01
0.602341816	-0.001	0.009	9.25E-01	-0.020	0.013	1.27E-01
6.835428	-0.001	0.011	9.28E-01	-0.022	0.016	1.59E-01
7.413756	-0.001	0.010	9.21E-01	0.000	0.015	9.98E-01
4.028235	0.002	0.014	8.64E-01	0.003	0.021	8.76E-01
7.885098	-0.003	0.009	7.69E-01	0.026	0.013	3.87E-02
4.22202	-0.002	0.013	8.75E-01	0.004	0.019	8.43E-01
4.100904	0.004	0.010	6.99E-01	0.000	0.015	9.93E-01
4.18804	-0.002	0.013	8.66E-01	0.019	0.019	3.18E-01
5.512575	-0.001	0.006	7.98E-01	-0.013	0.009	1.43E-01
0.790071201	-0.002	0.005	6.05E-01	0.000	0.007	9.96E-01
6.150115	-0.001	0.010	9.29E-01	-0.021	0.015	1.45E-01
4.113016	0.004	0.010	6.98E-01	0.013	0.015	3.93E-01
0.575763641	-0.002	0.009	8.08E-01	0.007	0.013	5.85E-01
0.652806704	-0.004	0.011	7.05E-01	-0.022	0.016	1.79E-01
6.229513	-0.001	0.008	8.85E-01	-0.010	0.012	3.91E-01
2.854085148	0.004	0.009	6.80E-01	-0.004	0.014	7.94E-01
5.968442	0.000	0.007	9.52E-01	-0.001	0.010	8.91E-01
6.110753	0.002	0.010	8.29E-01	-0.019	0.015	1.87E-01
2.517316124	0.003	0.020	8.68E-01	-0.003	0.030	9.11E-01
8.355094	0.003	0.009	6.87E-01	-0.007	0.013	5.66E-01
8.377299	0.001	0.007	8.46E-01	-0.013	0.010	2.27E-01
5.731593	0.002	0.010	8.66E-01	-0.006	0.015	6.83E-01
7.419812	-0.001	0.007	8.38E-01	-0.003	0.011	8.09E-01
1.88448642	-0.003	0.008	7.26E-01	-0.010	0.012	4.11E-01
6.505052	-0.001	0.008	8.94E-01	-0.004	0.011	7.23E-01
5.779703	-0.001	0.009	9.17E-01	0.009	0.013	4.75E-01

7.727648	0.002	0.008	8.13E-01	-0.005	0.012	7.03E-01
6.784291	0.000	0.008	9.55E-01	-0.017	0.012	1.48E-01
5.7649	-0.001	0.009	9.46E-01	0.004	0.013	7.60E-01
6.467371	0.004	0.008	6.58E-01	-0.021	0.013	9.33E-02
7.437643	0.002	0.010	8.15E-01	0.003	0.016	8.53E-01
8.397821	-0.001	0.010	9.28E-01	-0.007	0.014	6.43E-01
0.760801566	0.002	0.005	6.75E-01	0.001	0.008	8.59E-01
5.077905	0.003	0.009	7.30E-01	-0.009	0.013	4.84E-01
5.628308	-0.002	0.008	8.41E-01	-0.039	0.011	5.87E-04
2.727922926	-0.003	0.009	7.18E-01	-0.016	0.014	2.34E-01
5.957339	-0.001	0.008	9.37E-01	-0.001	0.012	9.36E-01
5.953975	-0.003	0.009	7.71E-01	-0.011	0.014	4.18E-01
1.673879618	0.000	0.004	9.51E-01	0.008	0.006	1.60E-01
6.109743	-0.003	0.009	7.74E-01	-0.004	0.014	7.94E-01
3.634609	0.001	0.004	8.12E-01	0.009	0.006	1.24E-01
7.233765	-0.005	0.010	6.22E-01	-0.014	0.015	3.25E-01
6.140022	0.000	0.008	9.53E-01	-0.014	0.012	2.49E-01
3.315671	-0.003	0.011	7.61E-01	-0.001	0.017	9.33E-01
3.75236	-0.005	0.011	6.78E-01	0.014	0.017	4.12E-01
3.737221	-0.007	0.009	4.68E-01	0.035	0.014	1.21E-02
7.347142	0.000	0.012	9.83E-01	-0.009	0.018	5.88E-01
7.383141	-0.005	0.008	5.19E-01	0.016	0.012	1.83E-01
8.219512	0.000	0.008	9.56E-01	0.001	0.011	8.99E-01
6.208318	-0.001	0.008	9.40E-01	0.009	0.011	4.15E-01
1.832339369	-0.004	0.010	6.93E-01	-0.007	0.015	6.33E-01
5.292885	0.001	0.011	8.88E-01	-0.026	0.016	9.74E-02
8.348702	-0.001	0.008	8.80E-01	-0.011	0.012	3.38E-01
3.607694	0.000	0.008	9.85E-01	-0.014	0.011	2.14E-01
7.224681	-0.003	0.010	7.90E-01	0.004	0.015	8.10E-01
4.291325	0.003	0.011	7.79E-01	0.012	0.017	4.94E-01
5.980553	0.000	0.007	9.91E-01	0.006	0.010	5.34E-01
7.8925	0.000	0.009	9.60E-01	0.006	0.014	6.85E-01
7.238138	-0.006	0.010	5.38E-01	-0.017	0.016	2.86E-01
6.072063	0.000	0.012	9.77E-01	-0.033	0.017	5.46E-02
3.933024	0.001	0.006	9.34E-01	0.011	0.010	2.60E-01
4.193423	-0.002	0.011	8.81E-01	0.017	0.017	3.20E-01
7.901583	0.003	0.009	7.68E-01	0.000	0.014	9.87E-01
2.809676046	-0.003	0.014	8.40E-01	-0.070	0.021	1.16E-03
0.942475165	0.003	0.006	6.50E-01	-0.014	0.008	8.58E-02
7.441007	-0.003	0.009	6.92E-01	-0.011	0.013	3.96E-01
6.170974	-0.003	0.007	7.11E-01	0.008	0.011	4.93E-01
8.047595	-0.001	0.007	9.01E-01	0.008	0.011	4.55E-01
1.501626132	0.000	0.005	9.53E-01	-0.009	0.008	2.56E-01

7.933544	0.002	0.008	7.95E-01	-0.002	0.011	8.67E-01
2.530100563	0.003	0.012	7.97E-01	-0.001	0.018	9.55E-01
6.082156	0.000	0.008	9.87E-01	0.017	0.012	1.46E-01
2.067169317	0.002	0.004	5.74E-01	0.011	0.006	4.69E-02
8.799522	0.001	0.007	8.83E-01	0.002	0.010	8.79E-01
8.100751	0.000	0.009	9.76E-01	-0.008	0.014	5.46E-01
1.469665035	0.001	0.007	8.52E-01	0.001	0.010	9.56E-01
6.031691	0.003	0.008	6.69E-01	0.009	0.012	4.59E-01
7.818148	0.000	0.009	9.97E-01	0.005	0.014	7.45E-01
6.371488	0.002	0.008	8.29E-01	-0.001	0.012	9.15E-01
6.666876	-0.001	0.010	9.03E-01	0.000	0.016	9.85E-01
6.408832	0.001	0.008	8.82E-01	0.000	0.012	9.75E-01
7.937582	0.000	0.009	9.88E-01	0.007	0.014	6.31E-01
8.605737	-0.002	0.008	8.45E-01	0.006	0.013	6.33E-01
8.796158	0.002	0.008	8.19E-01	0.005	0.012	6.90E-01
5.550929	0.000	0.009	9.90E-01	-0.011	0.013	4.11E-01
0.784015415	-0.001	0.004	8.08E-01	0.002	0.006	7.22E-01
6.339863	0.001	0.008	8.73E-01	0.006	0.012	6.17E-01
6.501351	-0.001	0.009	8.86E-01	0.006	0.013	6.35E-01
4.004012	-0.001	0.010	9.46E-01	0.014	0.015	3.36E-01
7.537227	-0.001	0.009	8.92E-01	0.009	0.013	4.90E-01
6.093258	-0.002	0.009	8.62E-01	0.011	0.013	4.31E-01
4.214955	-0.001	0.014	9.37E-01	-0.002	0.021	9.30E-01
6.947124	-0.002	0.009	8.49E-01	0.007	0.013	6.15E-01
8.017989	-0.003	0.007	7.34E-01	-0.002	0.011	8.61E-01
4.186358	0.001	0.014	9.53E-01	0.008	0.021	7.00E-01
3.299522	0.002	0.012	8.53E-01	0.014	0.018	4.24E-01
8.349712	-0.001	0.009	9.36E-01	-0.003	0.014	8.05E-01
7.587692	-0.003	0.008	7.25E-01	-0.022	0.012	7.76E-02
6.536003	-0.001	0.008	8.81E-01	0.000	0.012	9.73E-01
8.660575	-0.001	0.008	8.55E-01	-0.005	0.012	6.59E-01
6.183759	-0.001	0.008	8.88E-01	-0.016	0.013	2.13E-01
7.449754	0.000	0.006	9.79E-01	-0.012	0.009	1.91E-01
5.799216	0.000	0.008	9.56E-01	-0.006	0.012	6.14E-01
4.23144	-0.002	0.012	8.82E-01	0.008	0.018	6.51E-01
8.779	0.000	0.008	9.99E-01	-0.014	0.012	2.55E-01
6.569647	-0.006	0.009	5.24E-01	-0.009	0.014	5.17E-01
3.55151	-0.002	0.014	9.01E-01	0.059	0.020	3.86E-03
8.984896	-0.001	0.007	8.69E-01	-0.019	0.011	6.82E-02
7.488444	0.000	0.007	9.65E-01	-0.007	0.011	5.16E-01
5.443943	-0.002	0.008	8.07E-01	-0.005	0.012	6.90E-01
3.878522	0.000	0.005	9.19E-01	0.017	0.007	1.32E-02
8.501779	0.003	0.009	7.71E-01	0.010	0.013	4.72E-01

4.340444	-0.001	0.014	9.25E-01	0.016	0.021	4.48E-01
8.172412	0.000	0.007	9.81E-01	-0.014	0.010	1.84E-01
7.421494	0.000	0.007	9.78E-01	-0.014	0.011	1.83E-01
3.325764	-0.001	0.009	8.73E-01	0.015	0.014	2.83E-01
7.466576	0.000	0.009	9.68E-01	-0.011	0.013	3.92E-01
4.093503	0.004	0.010	6.91E-01	0.012	0.016	4.58E-01
3.932688	0.000	0.007	9.79E-01	0.012	0.010	2.25E-01
4.212264	0.004	0.011	6.98E-01	0.012	0.017	4.95E-01
2.069187913	0.001	0.004	7.74E-01	0.012	0.006	6.51E-02
6.209327	0.001	0.008	8.98E-01	0.003	0.012	7.98E-01
6.071054	0.000	0.009	9.91E-01	-0.008	0.014	5.53E-01
8.350384	-0.004	0.010	6.91E-01	-0.009	0.015	5.41E-01
6.111762	-0.001	0.008	8.75E-01	-0.025	0.012	3.99E-02
7.503247	-0.001	0.008	8.72E-01	-0.020	0.013	1.07E-01
8.943515	-0.001	0.007	9.15E-01	-0.013	0.011	2.10E-01
6.787319	0.003	0.010	7.82E-01	-0.001	0.015	9.74E-01
1.822919256	0.004	0.009	6.54E-01	0.012	0.014	3.73E-01
0.732204796	-0.003	0.007	6.26E-01	0.011	0.010	2.82E-01
8.455015	-0.004	0.010	7.06E-01	0.001	0.015	9.36E-01
8.483612	0.001	0.009	9.46E-01	-0.014	0.014	2.99E-01
3.192873	0.001	0.007	8.76E-01	0.017	0.010	1.05E-01
1.891551505	0.001	0.008	9.40E-01	-0.015	0.012	2.04E-01
6.847204	-0.001	0.008	8.93E-01	-0.001	0.012	9.03E-01
3.299858	0.003	0.011	8.21E-01	0.000	0.017	9.91E-01
2.070870076	0.002	0.004	6.19E-01	0.011	0.006	5.92E-02
5.461438	0.001	0.010	9.49E-01	-0.003	0.014	8.18E-01
3.575397	0.000	0.006	9.38E-01	0.010	0.009	2.96E-01
7.352525	-0.003	0.010	7.32E-01	-0.023	0.015	1.18E-01
6.977739	0.000	0.010	9.85E-01	0.007	0.014	6.21E-01
5.077232	0.002	0.009	8.23E-01	-0.016	0.013	2.25E-01
7.240157	-0.005	0.010	6.25E-01	-0.021	0.015	1.78E-01
6.975384	-0.001	0.010	8.80E-01	-0.004	0.015	7.69E-01
4.216301	-0.001	0.010	8.90E-01	0.004	0.015	8.16E-01
0.992940054	-0.002	0.006	7.79E-01	0.010	0.008	2.26E-01
2.589985564	0.001	0.016	9.37E-01	0.020	0.023	3.95E-01
6.326742	0.000	0.008	9.63E-01	-0.023	0.011	4.86E-02
5.560012	0.003	0.009	7.05E-01	-0.004	0.014	7.50E-01
8.982205	0.001	0.010	8.90E-01	0.001	0.015	9.44E-01
5.079923	0.003	0.009	7.41E-01	-0.007	0.013	5.81E-01
6.026308	-0.001	0.009	8.70E-01	0.005	0.013	7.16E-01
7.908985	0.002	0.011	8.23E-01	0.001	0.016	9.71E-01
6.446176	0.003	0.009	7.75E-01	-0.023	0.014	1.03E-01
8.346347	0.000	0.009	9.68E-01	-0.004	0.014	7.60E-01

2.357174211	0.006	0.007	4.42E-01	0.012	0.011	2.65E-01
7.148984	-0.004	0.007	5.90E-01	0.000	0.011	9.96E-01
5.607449	0.000	0.006	9.54E-01	-0.005	0.010	6.05E-01
5.616533	0.000	0.007	9.58E-01	0.007	0.011	5.03E-01
8.62525	0.000	0.007	9.48E-01	-0.016	0.011	1.27E-01
6.824999	-0.002	0.010	8.64E-01	0.029	0.016	6.63E-02
3.859682	-0.002	0.005	7.21E-01	0.010	0.008	2.02E-01
8.026736	-0.002	0.009	8.21E-01	-0.023	0.014	1.00E-01
0.559614876	0.001	0.009	9.41E-01	-0.002	0.014	8.60E-01
7.021476	-0.004	0.009	6.65E-01	-0.021	0.014	1.39E-01
1.754287008	-0.002	0.008	7.60E-01	0.017	0.011	1.44E-01
2.82447908	0.001	0.014	9.21E-01	-0.039	0.020	5.08E-02
6.196879	0.001	0.009	9.14E-01	0.010	0.013	4.42E-01
6.537349	-0.006	0.009	4.53E-01	-0.010	0.013	4.43E-01
8.195625	-0.003	0.008	7.20E-01	-0.010	0.012	3.71E-01
6.180058	-0.001	0.007	8.62E-01	-0.034	0.011	2.08E-03
7.423176	0.000	0.007	9.87E-01	-0.007	0.011	5.40E-01
6.331789	-0.001	0.009	9.43E-01	-0.006	0.013	6.72E-01
2.84130071	0.003	0.009	7.39E-01	-0.005	0.014	6.92E-01
7.91605	0.002	0.009	8.11E-01	-0.016	0.014	2.54E-01
0.733550526	0.000	0.007	9.50E-01	0.000	0.010	9.87E-01
6.283679	0.001	0.010	9.42E-01	0.003	0.014	8.31E-01
8.158954	0.001	0.009	8.83E-01	-0.009	0.014	4.95E-01
6.426326	-0.001	0.009	9.04E-01	0.003	0.013	8.01E-01
8.544506	0.002	0.009	8.31E-01	-0.004	0.014	7.91E-01
0.672319794	0.000	0.009	9.68E-01	-0.005	0.013	7.11E-01
0.998322975	0.001	0.006	8.18E-01	0.013	0.009	1.41E-01
8.960337	-0.001	0.008	8.68E-01	-0.003	0.012	8.28E-01
8.162991	0.002	0.009	8.55E-01	0.012	0.013	3.49E-01
1.851179594	0.003	0.008	7.53E-01	0.005	0.012	6.98E-01
3.786676	0.002	0.012	8.70E-01	0.031	0.017	7.87E-02
3.894335	0.000	0.005	9.27E-01	0.019	0.007	7.28E-03
7.12577	-0.001	0.008	8.80E-01	-0.009	0.012	4.29E-01
6.590506	-0.004	0.009	6.61E-01	-0.007	0.013	6.21E-01
8.476547	-0.001	0.009	9.01E-01	-0.037	0.014	9.01E-03
1.83637656	0.001	0.010	8.91E-01	-0.001	0.015	9.64E-01
0.822705163	-0.001	0.006	8.70E-01	0.009	0.010	3.39E-01
8.761169	0.003	0.007	7.18E-01	-0.004	0.010	7.15E-01
2.079617323	0.001	0.004	7.79E-01	0.007	0.006	2.10E-01
0.760465133	0.000	0.005	9.49E-01	-0.002	0.008	7.67E-01
1.691374113	0.000	0.004	9.55E-01	0.002	0.005	6.48E-01
1.81955493	-0.001	0.010	9.29E-01	0.000	0.015	9.85E-01
8.737282	-0.002	0.008	7.71E-01	-0.001	0.011	9.45E-01

8.182168	-0.007	0.010	4.66E-01	-0.009	0.015	5.40E-01
8.855033	-0.002	0.009	8.32E-01	-0.006	0.014	6.64E-01
8.784719	0.002	0.008	8.42E-01	-0.006	0.012	6.09E-01
4.048084	0.004	0.011	6.87E-01	-0.015	0.016	3.58E-01
0.560287742	0.001	0.010	8.92E-01	-0.012	0.015	4.47E-01
6.282333	0.002	0.009	8.54E-01	0.018	0.014	1.80E-01
0.753736482	-0.002	0.005	7.74E-01	-0.006	0.008	4.84E-01
8.022699	0.003	0.009	7.56E-01	-0.008	0.014	5.40E-01
5.876259	0.003	0.010	8.03E-01	0.004	0.015	8.13E-01
2.550959383	0.000	0.009	9.75E-01	-0.010	0.013	4.29E-01
5.468503	-0.001	0.007	8.69E-01	0.013	0.010	2.07E-01
5.292212	0.001	0.010	9.15E-01	-0.026	0.015	9.23E-02
8.044231	-0.001	0.006	8.74E-01	0.006	0.010	4.98E-01
6.066007	0.000	0.008	9.98E-01	-0.023	0.011	3.80E-02
7.95945	0.000	0.008	9.61E-01	-0.020	0.012	8.94E-02
1.774809396	0.001	0.010	9.36E-01	-0.013	0.014	3.74E-01
3.336866	-0.002	0.010	8.32E-01	0.007	0.015	6.30E-01
6.844512	0.001	0.011	9.38E-01	-0.020	0.016	2.03E-01
0.799491314	-0.002	0.006	7.82E-01	0.003	0.009	6.96E-01
7.532517	0.000	0.010	9.87E-01	-0.004	0.014	7.62E-01
8.957645	0.001	0.010	8.90E-01	-0.025	0.014	8.39E-02
5.494072	0.000	0.009	9.58E-01	-0.006	0.014	6.67E-01
8.919965	-0.001	0.005	8.56E-01	-0.003	0.008	6.94E-01
7.069586	0.001	0.010	9.37E-01	0.005	0.015	7.34E-01
7.342096	-0.001	0.010	8.97E-01	-0.015	0.015	3.29E-01
0.618827012	0.002	0.008	8.11E-01	0.013	0.011	2.39E-01
5.292549	0.001	0.010	9.09E-01	-0.026	0.016	9.73E-02
7.405682	-0.006	0.011	6.03E-01	0.003	0.016	8.43E-01
8.617512	-0.002	0.007	7.60E-01	0.008	0.011	4.66E-01
6.184768	-0.002	0.008	8.19E-01	0.005	0.012	6.84E-01
2.827506973	0.003	0.013	7.87E-01	-0.053	0.019	4.59E-03
5.44428	-0.002	0.007	8.15E-01	0.009	0.011	4.20E-01
7.035942	-0.005	0.010	6.33E-01	-0.022	0.016	1.60E-01
6.027317	-0.001	0.007	8.59E-01	-0.021	0.011	5.39E-02
5.865493	0.000	0.006	9.74E-01	-0.013	0.009	1.61E-01
8.72988	-0.003	0.007	7.11E-01	-0.017	0.011	1.12E-01
0.947521654	-0.001	0.005	9.11E-01	-0.004	0.008	6.15E-01
6.063988	-0.003	0.008	6.90E-01	-0.021	0.012	7.91E-02
2.072888671	0.000	0.003	9.54E-01	0.010	0.005	3.18E-02
6.437429	0.001	0.010	9.18E-01	0.006	0.015	7.07E-01
7.625036	-0.006	0.013	6.66E-01	0.011	0.019	5.61E-01
6.644335	-0.002	0.008	8.14E-01	-0.004	0.011	7.17E-01
7.343778	0.003	0.012	7.90E-01	0.016	0.017	3.42E-01

5.557321	-0.001	0.007	9.28E-01	-0.007	0.010	5.13E-01
5.819402	-0.004	0.009	6.32E-01	-0.002	0.013	9.05E-01
6.998262	0.000	0.008	9.56E-01	-0.010	0.012	4.03E-01
8.584205	-0.005	0.008	5.23E-01	-0.003	0.012	8.08E-01
8.267958	-0.003	0.008	7.45E-01	-0.001	0.012	9.45E-01
0.64574162	-0.001	0.010	9.51E-01	0.004	0.015	7.67E-01
3.666233	0.002	0.005	6.30E-01	0.013	0.007	7.27E-02
0.966025447	0.000	0.007	9.93E-01	0.018	0.010	8.69E-02
7.867604	0.003	0.008	7.38E-01	0.001	0.012	9.60E-01
5.990646	0.002	0.007	8.00E-01	-0.003	0.011	8.14E-01
7.32191	-0.002	0.006	7.35E-01	-0.003	0.009	7.06E-01
2.786125765	-0.001	0.012	9.08E-01	-0.052	0.018	4.35E-03
2.827843406	0.003	0.012	7.91E-01	-0.052	0.018	5.30E-03
6.777898	0.004	0.011	6.95E-01	0.001	0.016	9.50E-01
7.092799	0.003	0.009	7.01E-01	0.011	0.013	4.17E-01
8.300592	0.002	0.006	7.91E-01	-0.001	0.010	9.00E-01
8.4244	-0.002	0.008	8.10E-01	-0.007	0.011	5.07E-01
2.73229655	0.000	0.010	9.71E-01	-0.015	0.015	3.05E-01
1.658403719	0.001	0.005	7.83E-01	0.006	0.007	3.56E-01
4.178957	0.000	0.011	9.68E-01	0.010	0.017	5.42E-01
7.96416	-0.003	0.008	6.92E-01	0.013	0.012	2.78E-01
7.857174	0.000	0.008	9.82E-01	0.004	0.011	7.16E-01
0.824387326	-0.003	0.007	6.66E-01	0.007	0.010	5.18E-01
6.997589	0.003	0.008	7.26E-01	0.002	0.012	8.42E-01
7.277501	-0.003	0.009	7.81E-01	-0.001	0.014	9.34E-01
6.512453	-0.002	0.010	8.09E-01	0.021	0.015	1.42E-01
7.379776	0.004	0.011	7.20E-01	-0.005	0.016	7.57E-01
7.096164	-0.001	0.010	9.14E-01	-0.001	0.015	9.32E-01
5.988291	0.001	0.009	9.46E-01	0.007	0.014	6.32E-01
7.342432	0.001	0.011	9.27E-01	0.011	0.016	4.88E-01
6.314631	0.000	0.006	9.76E-01	-0.020	0.010	3.36E-02
8.797503	0.001	0.008	8.61E-01	-0.018	0.011	1.15E-01
0.734223391	0.001	0.007	9.00E-01	-0.004	0.010	6.83E-01
8.179813	-0.001	0.008	9.17E-01	-0.021	0.012	8.29E-02
6.746947	0.002	0.009	8.55E-01	-0.010	0.013	4.61E-01
8.984223	-0.004	0.008	6.20E-01	-0.031	0.012	8.93E-03
8.460734	0.002	0.008	8.15E-01	-0.002	0.012	8.51E-01
2.329923171	0.001	0.005	8.37E-01	-0.009	0.008	2.84E-01
3.746641	0.000	0.009	9.67E-01	0.014	0.014	3.18E-01
2.845001468	-0.001	0.009	9.51E-01	-0.006	0.013	6.32E-01
6.097968	0.001	0.009	9.39E-01	0.004	0.014	7.91E-01
3.305914	-0.002	0.009	8.25E-01	0.008	0.013	5.37E-01
7.178253	0.003	0.011	7.95E-01	0.017	0.016	2.93E-01

2.805638855	0.001	0.014	9.34E-01	-0.069	0.021	1.37E-03
7.133508	-0.001	0.007	8.42E-01	-0.009	0.011	4.04E-01
0.541111084	0.004	0.009	6.56E-01	0.012	0.013	3.64E-01
0.788725471	-0.002	0.005	6.28E-01	-0.001	0.007	9.07E-01
5.138462	-0.002	0.009	8.08E-01	-0.004	0.013	7.83E-01
4.012086	0.000	0.012	9.69E-01	0.010	0.018	5.91E-01
2.807993883	-0.002	0.015	9.03E-01	-0.065	0.022	3.41E-03
6.627513	0.000	0.008	9.76E-01	-0.015	0.012	1.92E-01
5.552947	0.002	0.008	7.95E-01	-0.023	0.011	4.16E-02
6.066344	0.002	0.008	8.36E-01	-0.013	0.012	2.72E-01
7.596103	0.001	0.009	8.69E-01	0.011	0.013	4.04E-01
8.173084	0.003	0.009	7.83E-01	-0.013	0.014	3.40E-01
2.805302422	-0.002	0.014	8.85E-01	-0.073	0.021	6.84E-04
3.644702	0.001	0.004	7.42E-01	0.014	0.006	1.92E-02
0.972417666	0.000	0.005	9.83E-01	0.015	0.007	4.03E-02
7.627054	-0.004	0.012	7.62E-01	-0.004	0.018	8.21E-01
5.242084	-0.003	0.004	4.74E-01	-0.001	0.006	9.01E-01
1.461927086	0.000	0.004	9.38E-01	0.002	0.006	7.02E-01
7.591729	0.002	0.011	8.53E-01	-0.006	0.016	6.92E-01
6.062306	-0.002	0.006	7.83E-01	-0.001	0.009	8.73E-01
1.764379985	0.001	0.010	8.95E-01	0.015	0.015	2.89E-01
5.882315	-0.001	0.007	8.75E-01	0.003	0.011	7.78E-01
6.932657	-0.001	0.009	8.91E-01	0.007	0.013	5.90E-01
8.244408	0.000	0.010	9.66E-01	0.002	0.016	8.81E-01
6.377544	0.001	0.011	9.17E-01	0.014	0.016	4.04E-01
8.672014	-0.002	0.007	8.34E-01	0.005	0.011	6.70E-01
3.575733	0.003	0.007	6.77E-01	0.011	0.010	2.77E-01
8.243399	0.002	0.007	7.89E-01	0.008	0.010	4.01E-01
4.210918	0.001	0.012	9.07E-01	0.023	0.017	1.93E-01
8.106807	-0.001	0.008	9.16E-01	-0.019	0.012	1.07E-01
6.685379	-0.001	0.008	8.93E-01	-0.010	0.011	3.82E-01
7.692995	0.001	0.007	9.33E-01	-0.006	0.010	5.83E-01
6.630205	0.000	0.010	9.71E-01	0.015	0.015	3.05E-01
7.318882	-0.002	0.006	7.36E-01	-0.001	0.009	9.21E-01
7.976271	-0.004	0.009	6.28E-01	0.007	0.014	6.29E-01
1.755969171	0.003	0.008	6.72E-01	0.009	0.012	4.62E-01
7.026186	-0.005	0.009	5.60E-01	-0.023	0.014	9.12E-02
7.434615	-0.005	0.010	5.82E-01	-0.005	0.014	7.39E-01
7.205504	-0.003	0.006	6.67E-01	-0.014	0.009	1.47E-01
6.476791	0.000	0.008	9.53E-01	-0.011	0.011	3.32E-01
4.28897	0.001	0.011	9.52E-01	0.014	0.016	3.77E-01
4.289979	0.000	0.011	9.72E-01	0.010	0.016	5.56E-01
5.016674	-0.001	0.009	8.67E-01	-0.016	0.013	2.34E-01

5.614851	-0.003	0.008	7.32E-01	-0.006	0.013	6.30E-01
8.437857	-0.001	0.008	8.93E-01	-0.020	0.011	8.23E-02
0.95862393	-0.001	0.005	8.53E-01	0.011	0.007	1.47E-01
7.453792	0.003	0.009	7.49E-01	0.003	0.013	7.97E-01
5.977525	0.000	0.010	9.93E-01	0.002	0.015	9.14E-01
5.959358	0.000	0.009	9.96E-01	-0.009	0.013	4.65E-01
2.387116712	-0.006	0.016	7.09E-01	-0.003	0.024	8.95E-01
6.935685	0.001	0.009	9.41E-01	0.014	0.014	3.23E-01
8.164337	-0.001	0.006	8.77E-01	-0.003	0.009	7.28E-01
8.631306	0.001	0.008	9.26E-01	-0.005	0.012	6.62E-01
5.880296	-0.001	0.008	9.14E-01	-0.024	0.012	4.29E-02
2.082308784	0.000	0.007	9.44E-01	0.010	0.010	3.44E-01
5.530406	0.000	0.008	9.71E-01	0.001	0.011	9.34E-01
6.700183	-0.001	0.010	9.12E-01	-0.003	0.015	8.49E-01
1.755632738	0.002	0.008	7.98E-01	0.008	0.012	5.18E-01
6.928957	-0.004	0.009	6.94E-01	-0.013	0.014	3.43E-01
6.537013	-0.002	0.007	7.58E-01	-0.023	0.011	3.46E-02
5.608795	-0.002	0.008	8.06E-01	-0.020	0.012	9.46E-02
8.044904	-0.001	0.007	9.21E-01	-0.008	0.011	4.51E-01
2.575518963	0.002	0.009	8.61E-01	-0.003	0.014	8.03E-01
2.748108882	-0.003	0.008	7.45E-01	-0.020	0.012	1.11E-01
3.894671	0.000	0.005	9.17E-01	0.019	0.007	6.73E-03
8.61583	-0.002	0.010	8.63E-01	0.016	0.014	2.63E-01
5.618215	0.002	0.009	8.62E-01	-0.020	0.014	1.41E-01
3.644029	0.000	0.004	9.80E-01	0.012	0.006	3.33E-02
8.056679	0.003	0.009	7.67E-01	0.012	0.013	3.33E-01
5.841943	0.002	0.008	7.78E-01	-0.024	0.012	5.65E-02
6.03741	-0.004	0.008	6.42E-01	0.000	0.013	9.93E-01
7.450091	0.001	0.006	8.96E-01	-0.001	0.009	9.03E-01
7.076987	0.002	0.009	8.48E-01	0.007	0.013	5.65E-01
1.817536335	0.000	0.011	9.75E-01	0.008	0.016	6.13E-01
0.749026425	-0.001	0.006	8.72E-01	-0.015	0.008	7.39E-02
6.595216	-0.002	0.009	8.03E-01	-0.018	0.013	1.54E-01
5.069494	0.001	0.007	9.39E-01	-0.013	0.010	2.02E-01
3.916539	-0.002	0.009	8.07E-01	0.029	0.013	2.74E-02
2.826834108	0.001	0.013	9.51E-01	-0.052	0.019	6.34E-03
1.034321263	0.001	0.005	8.97E-01	0.002	0.007	8.27E-01
5.452354	-0.001	0.009	8.82E-01	-0.013	0.014	3.37E-01
5.083624	0.001	0.008	9.41E-01	-0.006	0.012	5.99E-01
0.572399315	0.001	0.008	9.42E-01	0.003	0.011	7.84E-01
2.564416687	-0.001	0.009	8.79E-01	0.008	0.013	5.39E-01
7.159413	0.000	0.007	9.68E-01	0.013	0.011	2.33E-01
2.516979692	0.002	0.018	9.19E-01	-0.004	0.027	8.78E-01

6.537686	-0.006	0.009	4.74E-01	0.003	0.013	8.47E-01
6.386291	0.003	0.007	6.97E-01	-0.003	0.010	7.93E-01
2.747772449	-0.003	0.008	7.48E-01	-0.019	0.012	1.24E-01
4.112343	0.004	0.010	7.17E-01	-0.002	0.015	9.11E-01
7.6284	-0.001	0.010	9.03E-01	0.010	0.015	4.76E-01
8.155253	0.002	0.011	8.15E-01	-0.020	0.016	2.11E-01
8.059034	0.001	0.009	8.87E-01	0.000	0.013	9.82E-01
6.603626	0.000	0.007	9.62E-01	-0.009	0.011	4.04E-01
5.705351	-0.002	0.009	7.76E-01	-0.003	0.013	8.07E-01
7.114331	0.001	0.008	8.64E-01	0.002	0.012	8.67E-01
2.743398826	0.000	0.010	9.65E-01	-0.017	0.015	2.79E-01
6.908434	0.001	0.008	9.07E-01	0.005	0.011	6.49E-01
8.480584	0.001	0.010	8.90E-01	0.033	0.015	3.21E-02
5.961377	0.000	0.009	9.74E-01	-0.002	0.013	8.87E-01
6.51178	-0.003	0.008	7.01E-01	-0.010	0.012	3.93E-01
8.458716	0.000	0.008	9.97E-01	0.002	0.012	8.81E-01
7.083716	0.000	0.008	9.76E-01	0.003	0.012	8.16E-01
6.159535	-0.001	0.007	9.27E-01	0.005	0.011	6.40E-01
8.327171	-0.001	0.009	9.16E-01	-0.030	0.014	3.06E-02
8.214802	0.001	0.007	8.77E-01	0.004	0.010	6.65E-01
7.838334	0.001	0.007	9.12E-01	0.015	0.011	1.78E-01
2.071879374	0.001	0.003	6.80E-01	0.012	0.005	1.74E-02
6.47881	-0.003	0.008	7.27E-01	-0.023	0.012	5.11E-02
7.915041	0.001	0.008	9.45E-01	-0.026	0.012	2.40E-02
4.033281	0.001	0.014	9.54E-01	-0.013	0.021	5.19E-01
3.916876	-0.003	0.009	7.76E-01	0.030	0.013	2.27E-02
6.890267	0.002	0.009	8.07E-01	-0.006	0.013	6.71E-01
8.168374	0.000	0.009	9.72E-01	-0.021	0.014	1.38E-01
2.729941522	-0.001	0.010	9.57E-01	-0.016	0.014	2.57E-01
4.021843	0.002	0.013	8.56E-01	0.024	0.019	1.98E-01
6.368796	-0.001	0.007	9.36E-01	-0.010	0.010	3.29E-01
4.037319	0.004	0.015	8.13E-01	-0.018	0.022	4.32E-01
6.566619	0.001	0.007	8.69E-01	-0.004	0.010	6.76E-01
8.37057	-0.001	0.009	8.77E-01	0.012	0.013	3.61E-01
6.285698	0.001	0.008	8.54E-01	0.005	0.011	6.86E-01
1.770772205	-0.001	0.010	8.87E-01	0.025	0.015	1.04E-01
2.333960362	0.002	0.006	7.75E-01	0.002	0.009	7.97E-01
5.935808	0.000	0.008	1.00E+00	-0.025	0.012	4.10E-02
1.922839736	0.000	0.004	9.61E-01	-0.001	0.006	8.83E-01
7.465567	0.001	0.010	8.84E-01	-0.013	0.014	3.75E-01
2.566435283	-0.002	0.008	7.78E-01	0.007	0.012	5.36E-01
5.800225	-0.002	0.008	7.64E-01	-0.005	0.011	6.75E-01
8.578822	-0.003	0.008	6.71E-01	-0.013	0.012	2.88E-01

8.030437	0.000	0.009	9.60E-01	0.017	0.013	1.88E-01
0.519242966	0.000	0.010	9.90E-01	-0.016	0.014	2.74E-01
7.097173	-0.001	0.010	8.97E-01	0.003	0.015	8.29E-01
7.414765	0.000	0.007	9.46E-01	-0.013	0.010	1.82E-01
6.935349	0.000	0.009	9.99E-01	0.007	0.014	6.36E-01
6.441129	0.000	0.009	9.86E-01	0.009	0.014	5.10E-01
6.333807	0.001	0.010	9.09E-01	0.014	0.015	3.54E-01
8.124302	0.000	0.010	9.91E-01	0.031	0.015	3.34E-02
8.602709	0.001	0.009	9.18E-01	-0.015	0.014	2.79E-01
8.655192	-0.001	0.008	8.51E-01	-0.008	0.012	5.06E-01
1.031629802	-0.001	0.006	8.70E-01	0.016	0.009	7.14E-02
5.905865	0.001	0.007	8.40E-01	-0.014	0.011	1.93E-01
0.760128701	0.001	0.005	8.17E-01	-0.003	0.008	6.93E-01
8.374608	0.002	0.009	8.51E-01	-0.014	0.013	2.91E-01
2.036217519	0.001	0.004	7.40E-01	0.010	0.006	1.28E-01
8.424063	-0.002	0.009	8.56E-01	-0.004	0.013	7.31E-01
1.67017886	-0.001	0.004	8.49E-01	0.001	0.006	8.69E-01
5.45269	0.001	0.008	9.46E-01	-0.021	0.012	9.53E-02
2.820105456	-0.001	0.016	9.43E-01	-0.078	0.024	1.54E-03
6.204954	-0.001	0.006	9.01E-01	-0.007	0.010	4.75E-01
4.202507	0.001	0.012	9.66E-01	0.013	0.018	4.51E-01
5.13779	-0.001	0.007	8.48E-01	-0.002	0.011	8.69E-01
2.612526548	-0.004	0.013	7.68E-01	0.043	0.019	2.41E-02
7.316191	-0.002	0.006	7.63E-01	0.000	0.009	9.85E-01
0.567689259	0.002	0.008	7.98E-01	0.003	0.012	7.80E-01
8.371243	-0.003	0.008	6.95E-01	0.011	0.012	3.29E-01
5.675409	-0.003	0.010	7.29E-01	0.011	0.014	4.33E-01
7.105584	0.001	0.011	9.58E-01	0.006	0.016	7.31E-01
6.810532	-0.005	0.009	6.08E-01	0.003	0.013	7.95E-01
5.784077	-0.004	0.011	7.37E-01	-0.024	0.016	1.31E-01
5.632009	0.002	0.009	8.55E-01	-0.031	0.014	2.88E-02
2.829189136	0.001	0.012	9.33E-01	-0.031	0.018	8.16E-02
8.339282	0.000	0.009	9.90E-01	0.010	0.013	4.49E-01
2.73095082	-0.001	0.010	9.42E-01	-0.016	0.014	2.82E-01
6.036401	0.002	0.010	8.42E-01	-0.008	0.015	5.89E-01
2.728259359	-0.003	0.009	7.45E-01	-0.017	0.014	2.16E-01
3.208349	-0.003	0.012	8.13E-01	0.010	0.018	5.55E-01
7.933208	0.002	0.007	8.06E-01	-0.013	0.010	1.94E-01
0.734559824	-0.001	0.007	9.37E-01	-0.008	0.010	4.03E-01
7.346806	-0.002	0.011	8.37E-01	0.005	0.016	7.50E-01
6.177703	0.004	0.009	6.79E-01	-0.011	0.013	4.07E-01
6.171647	-0.002	0.010	8.55E-01	-0.002	0.014	9.08E-01
0.992603621	-0.002	0.006	7.48E-01	0.011	0.009	2.18E-01

6.482847	0.000	0.008	9.98E-01	-0.012	0.012	3.28E-01
6.356685	-0.003	0.009	7.64E-01	-0.022	0.013	9.76E-02
4.027562	-0.001	0.013	9.16E-01	-0.006	0.019	7.44E-01
7.279519	-0.005	0.009	5.72E-01	0.000	0.014	9.88E-01
3.897026	0.000	0.005	9.17E-01	0.018	0.007	1.15E-02
4.227739	0.001	0.011	9.48E-01	0.019	0.016	2.57E-01
3.665897	0.002	0.005	6.91E-01	0.014	0.008	5.62E-02
3.232572	0.000	0.006	9.68E-01	0.015	0.009	7.57E-02
6.706575	0.002	0.009	8.08E-01	-0.003	0.013	8.23E-01
8.410269	0.003	0.008	7.35E-01	0.003	0.013	7.98E-01
7.856838	-0.002	0.007	8.01E-01	0.000	0.010	9.82E-01
8.079556	0.002	0.008	7.62E-01	0.005	0.012	6.43E-01
2.635067531	0.001	0.009	9.17E-01	-0.013	0.013	2.94E-01
6.366441	0.001	0.007	8.74E-01	0.009	0.011	3.89E-01
6.988842	0.002	0.007	7.55E-01	0.005	0.010	6.01E-01
2.81404967	0.002	0.016	8.96E-01	-0.073	0.023	1.69E-03
1.891215072	0.003	0.008	6.89E-01	-0.011	0.012	3.40E-01
0.856348422	0.001	0.008	8.70E-01	-0.009	0.011	4.29E-01
5.463456	0.000	0.007	9.57E-01	-0.013	0.010	1.82E-01
2.504868118	-0.005	0.013	7.13E-01	-0.009	0.020	6.51E-01
6.609009	-0.001	0.008	9.26E-01	-0.004	0.012	7.68E-01
8.014961	-0.001	0.010	9.33E-01	-0.021	0.015	1.51E-01
2.848702227	0.001	0.009	9.47E-01	-0.018	0.013	1.62E-01
7.320228	-0.003	0.006	6.35E-01	0.003	0.009	7.06E-01
8.650819	-0.001	0.008	8.91E-01	0.015	0.012	1.91E-01
0.605706141	-0.003	0.009	6.99E-01	0.002	0.013	8.94E-01
0.623537069	0.000	0.009	9.57E-01	0.022	0.013	1.08E-01
5.914949	-0.001	0.009	9.19E-01	-0.022	0.014	1.06E-01
7.175898	0.000	0.010	9.78E-01	-0.007	0.015	6.26E-01
6.673268	0.002	0.008	7.98E-01	-0.012	0.012	3.03E-01
8.211774	-0.003	0.008	7.28E-01	0.014	0.012	2.51E-01
6.027654	0.000	0.007	9.64E-01	-0.017	0.010	1.12E-01
2.280804013	0.002	0.005	6.91E-01	-0.003	0.008	6.77E-01
6.710612	-0.001	0.010	9.16E-01	0.002	0.014	8.87E-01
3.897363	0.001	0.005	9.06E-01	0.017	0.007	1.57E-02
8.576467	0.001	0.008	9.38E-01	-0.009	0.013	4.50E-01
8.173757	-0.001	0.008	9.29E-01	0.000	0.012	9.94E-01
3.925287	0.001	0.009	9.19E-01	0.022	0.013	8.43E-02
1.921830438	0.000	0.005	9.38E-01	-0.002	0.008	8.34E-01
1.133568877	0.002	0.008	7.74E-01	-0.015	0.011	1.77E-01
5.631673	0.003	0.009	7.52E-01	-0.035	0.014	1.28E-02
6.047167	0.000	0.007	9.44E-01	0.006	0.010	5.60E-01
6.014869	-0.001	0.008	8.97E-01	0.010	0.013	4.47E-01

1.675561781	0.000	0.004	9.82E-01	0.001	0.006	8.99E-01
6.048849	-0.001	0.009	9.08E-01	-0.017	0.013	1.95E-01
7.543283	-0.003	0.008	7.65E-01	0.001	0.013	9.11E-01
6.841821	0.002	0.010	8.60E-01	0.010	0.015	4.94E-01
4.205871	0.000	0.014	9.94E-01	0.023	0.021	2.68E-01
8.301265	0.002	0.008	8.03E-01	0.000	0.012	9.82E-01
8.834847	-0.001	0.008	9.42E-01	-0.012	0.011	2.84E-01
6.493949	-0.003	0.008	7.39E-01	-0.006	0.012	5.96E-01
5.473549	-0.002	0.009	8.30E-01	0.000	0.014	9.86E-01
7.459847	0.000	0.010	9.69E-01	-0.011	0.015	4.80E-01
7.983336	0.003	0.010	7.77E-01	-0.022	0.015	1.27E-01
8.501443	0.001	0.009	8.99E-01	0.000	0.014	9.83E-01
7.166142	0.000	0.008	9.87E-01	-0.004	0.012	7.43E-01
8.017316	0.001	0.009	9.06E-01	-0.008	0.014	5.46E-01
1.187734524	-0.002	0.012	8.70E-01	0.007	0.018	7.08E-01
7.347815	-0.002	0.011	8.91E-01	-0.021	0.017	1.96E-01
0.823041595	-0.001	0.006	8.50E-01	0.009	0.010	3.70E-01
2.066832885	0.002	0.004	6.01E-01	0.011	0.006	6.18E-02
7.706789	-0.001	0.011	9.61E-01	-0.010	0.017	5.55E-01
3.649412	0.001	0.004	8.18E-01	0.012	0.006	5.40E-02
8.691863	0.002	0.008	8.45E-01	0.002	0.012	8.54E-01
3.784994	-0.002	0.010	8.63E-01	0.027	0.015	7.70E-02
1.463945681	0.001	0.004	8.42E-01	0.001	0.007	8.55E-01
3.798451	0.003	0.006	6.05E-01	0.013	0.009	1.64E-01
0.501748471	0.000	0.009	9.88E-01	0.007	0.014	6.15E-01
7.151339	-0.003	0.007	6.58E-01	-0.017	0.011	1.08E-01
2.420423538	0.005	0.017	7.74E-01	-0.018	0.025	4.84E-01
1.750922682	0.000	0.007	9.64E-01	-0.002	0.010	8.09E-01
0.561633472	-0.001	0.007	9.17E-01	0.000	0.010	9.88E-01
6.279978	0.002	0.007	8.24E-01	-0.001	0.010	9.42E-01
2.831880597	0.004	0.012	7.59E-01	-0.015	0.018	3.93E-01
2.586621238	0.005	0.013	7.01E-01	-0.001	0.020	9.45E-01
2.745080989	-0.003	0.010	7.87E-01	-0.019	0.014	1.96E-01
1.664123073	0.000	0.004	9.90E-01	0.006	0.006	3.74E-01
8.271996	-0.003	0.008	7.54E-01	0.015	0.013	2.38E-01
7.771384	-0.003	0.012	8.26E-01	-0.021	0.018	2.46E-01
5.076895	0.001	0.008	9.25E-01	-0.010	0.012	3.98E-01
6.158526	0.001	0.007	8.54E-01	0.007	0.011	4.92E-01
4.290652	0.002	0.011	8.46E-01	0.013	0.017	4.32E-01
0.590903107	0.000	0.009	9.94E-01	-0.009	0.013	4.84E-01
1.897270859	-0.001	0.006	8.62E-01	0.000	0.009	9.80E-01
4.165163	0.000	0.010	9.67E-01	0.000	0.014	9.82E-01
8.625586	-0.001	0.008	8.80E-01	-0.008	0.012	5.12E-01

2.528754832	0.003	0.013	8.32E-01	0.010	0.019	5.88E-01
1.701467091	0.001	0.004	8.17E-01	0.005	0.006	3.92E-01
6.640298	-0.002	0.008	7.60E-01	-0.017	0.011	1.28E-01
5.906874	-0.001	0.009	8.95E-01	-0.031	0.013	1.74E-02
8.422717	-0.001	0.007	8.77E-01	-0.012	0.010	2.00E-01
5.994011	0.002	0.009	8.54E-01	0.008	0.014	5.47E-01
0.685777098	-0.001	0.013	9.43E-01	-0.011	0.020	5.95E-01
7.905284	-0.001	0.010	8.80E-01	0.007	0.014	6.17E-01
8.030101	-0.001	0.008	9.30E-01	0.012	0.012	3.31E-01
8.63467	0.003	0.010	7.68E-01	-0.021	0.015	1.54E-01
5.513585	-0.003	0.007	7.28E-01	0.003	0.011	7.90E-01
0.74700783	0.001	0.006	9.21E-01	-0.001	0.009	9.13E-01
6.791692	0.002	0.011	8.26E-01	0.005	0.016	7.36E-01
7.156722	0.001	0.007	9.17E-01	-0.008	0.011	4.82E-01
8.970766	-0.002	0.009	7.99E-01	-0.025	0.014	7.19E-02
4.1221	0.000	0.009	9.99E-01	0.014	0.014	3.17E-01
3.578761	0.002	0.006	7.56E-01	0.013	0.009	1.49E-01
2.824815513	0.001	0.013	9.12E-01	-0.042	0.020	3.52E-02
6.270558	0.000	0.007	9.95E-01	0.009	0.011	4.23E-01
2.423114999	0.005	0.011	6.74E-01	0.005	0.017	7.66E-01
6.246671	-0.002	0.009	8.45E-01	0.002	0.013	8.81E-01
6.750647	0.004	0.012	7.59E-01	0.025	0.017	1.61E-01
1.003369464	0.003	0.008	7.21E-01	-0.003	0.013	8.32E-01
8.016643	0.000	0.007	9.64E-01	-0.007	0.010	4.86E-01
8.543497	0.002	0.007	7.80E-01	0.007	0.010	4.94E-01
2.744744556	-0.001	0.010	9.30E-01	-0.018	0.015	2.29E-01
0.736578419	0.001	0.007	8.39E-01	-0.010	0.010	2.98E-01
5.873231	0.001	0.008	9.28E-01	-0.020	0.012	1.02E-01
8.584541	-0.005	0.008	4.97E-01	-0.008	0.012	4.74E-01
7.706452	-0.002	0.011	8.95E-01	0.000	0.017	9.83E-01
6.983795	0.000	0.010	9.94E-01	-0.004	0.015	8.14E-01
6.657792	-0.001	0.008	9.21E-01	-0.010	0.011	3.88E-01
8.459052	-0.003	0.008	7.19E-01	-0.007	0.012	5.23E-01
1.883477123	-0.001	0.009	9.38E-01	-0.014	0.013	2.69E-01
6.93804	0.001	0.009	9.37E-01	0.008	0.014	5.78E-01
1.681617568	-0.001	0.004	8.37E-01	0.009	0.006	1.21E-01
4.036982	0.001	0.015	9.64E-01	-0.022	0.022	3.35E-01
8.036829	-0.002	0.011	8.63E-01	0.026	0.016	1.08E-01
8.794812	0.002	0.009	7.95E-01	-0.027	0.013	3.71E-02
5.0123	-0.001	0.011	9.21E-01	-0.003	0.017	8.60E-01
5.291876	0.001	0.010	9.45E-01	-0.026	0.015	9.01E-02
1.884149988	-0.002	0.008	7.93E-01	-0.010	0.013	4.17E-01
6.199234	0.001	0.008	9.06E-01	0.014	0.011	2.08E-01

1.753950575	-0.001	0.007	8.89E-01	0.013	0.011	2.44E-01
8.542487	0.001	0.010	9.21E-01	0.021	0.015	1.66E-01
2.815731833	-0.002	0.015	8.72E-01	-0.063	0.023	5.84E-03
6.189141	-0.001	0.008	9.17E-01	-0.007	0.012	5.31E-01
2.59469562	0.001	0.019	9.41E-01	0.005	0.029	8.53E-01
3.893998	0.000	0.005	9.54E-01	0.019	0.007	6.94E-03
3.799461	0.002	0.006	6.75E-01	0.009	0.008	2.64E-01
1.816527037	0.002	0.009	7.85E-01	-0.004	0.013	7.88E-01
8.903143	0.000	0.009	9.96E-01	0.013	0.013	3.18E-01
3.305578	0.000	0.009	9.87E-01	0.015	0.013	2.51E-01
7.976608	-0.005	0.009	5.40E-01	0.006	0.013	6.68E-01
8.941833	-0.001	0.006	8.69E-01	-0.015	0.009	1.21E-01
0.709663812	0.001	0.009	9.08E-01	0.001	0.013	9.44E-01
8.328516	0.002	0.006	6.95E-01	-0.007	0.010	4.78E-01
2.579892586	0.004	0.011	7.34E-01	0.026	0.016	1.20E-01
2.848029361	0.003	0.009	7.67E-01	0.003	0.013	8.49E-01
2.333287497	0.002	0.006	7.81E-01	-0.006	0.009	5.00E-01
8.587906	0.000	0.008	9.53E-01	0.004	0.011	7.02E-01
6.170301	-0.002	0.007	7.90E-01	0.013	0.011	2.45E-01
8.451651	-0.004	0.011	6.76E-01	0.000	0.016	9.90E-01
7.881061	0.002	0.009	8.31E-01	0.023	0.013	7.97E-02
8.142133	0.001	0.006	8.47E-01	0.001	0.009	8.98E-01
6.329097	0.000	0.008	9.65E-01	-0.007	0.013	5.81E-01
8.92131	-0.003	0.008	6.68E-01	0.020	0.012	9.03E-02
7.77643	0.002	0.009	8.51E-01	-0.014	0.013	2.87E-01
5.788787	0.000	0.011	9.98E-01	0.003	0.016	8.30E-01
5.620234	0.000	0.009	9.64E-01	0.011	0.013	4.08E-01
6.515145	0.000	0.008	9.67E-01	-0.034	0.011	2.70E-03
8.982541	-0.001	0.010	9.10E-01	-0.015	0.015	3.10E-01
8.066099	0.003	0.008	6.68E-01	0.016	0.012	1.57E-01
5.042243	-0.001	0.008	9.37E-01	0.000	0.012	9.85E-01
4.040346	0.004	0.015	8.03E-01	-0.019	0.022	3.81E-01
8.820044	-0.002	0.010	8.75E-01	-0.003	0.014	8.46E-01
2.034535356	0.001	0.004	7.45E-01	0.008	0.006	1.92E-01
6.065671	-0.003	0.009	7.40E-01	-0.016	0.013	2.17E-01
3.650085	0.000	0.004	9.96E-01	0.012	0.006	5.65E-02
6.360049	0.003	0.009	7.54E-01	-0.020	0.014	1.47E-01
0.723793981	-0.001	0.007	8.75E-01	0.010	0.011	3.46E-01
6.452905	-0.003	0.010	7.63E-01	-0.024	0.016	1.15E-01
5.485997	0.000	0.009	9.59E-01	0.000	0.013	9.72E-01
6.457278	0.000	0.009	9.56E-01	0.002	0.013	8.99E-01
7.089771	0.000	0.009	9.76E-01	-0.001	0.013	9.39E-01
3.891307	0.000	0.005	9.46E-01	0.019	0.007	5.10E-03

8.684798	0.003	0.009	7.55E-01	-0.007	0.014	5.93E-01
0.720429655	0.000	0.007	9.91E-01	-0.008	0.011	4.70E-01
6.170638	-0.001	0.006	8.79E-01	0.009	0.010	3.53E-01
2.83356276	0.003	0.011	7.89E-01	-0.025	0.017	1.36E-01
7.629073	0.000	0.010	9.71E-01	0.011	0.016	4.68E-01
6.042793	0.003	0.009	7.85E-01	0.012	0.014	4.14E-01
2.070197211	0.001	0.004	7.58E-01	0.011	0.006	7.52E-02
6.304874	-0.001	0.007	9.03E-01	0.003	0.011	7.96E-01
5.890389	-0.002	0.009	8.60E-01	-0.002	0.013	8.90E-01
5.661952	0.001	0.007	8.74E-01	-0.001	0.011	9.36E-01
7.428223	0.001	0.010	9.04E-01	-0.003	0.015	8.60E-01
7.507957	0.002	0.009	8.61E-01	-0.008	0.014	5.50E-01
7.547656	0.002	0.010	8.09E-01	-0.009	0.015	5.23E-01
6.7419	-0.001	0.011	9.05E-01	-0.031	0.017	6.15E-02
8.148861	0.000	0.009	9.66E-01	0.004	0.013	7.85E-01
3.637973	0.001	0.004	7.07E-01	0.010	0.006	6.87E-02
6.164245	0.003	0.009	7.70E-01	-0.023	0.014	8.70E-02
5.57347	0.001	0.008	9.08E-01	-0.009	0.012	4.78E-01
6.384272	0.002	0.010	8.57E-01	0.003	0.015	8.22E-01
7.268417	-0.002	0.010	8.24E-01	-0.012	0.014	4.25E-01
8.78371	0.002	0.008	7.93E-01	0.006	0.012	5.95E-01
0.907822608	0.000	0.006	9.80E-01	-0.020	0.008	1.83E-02
4.21731	-0.001	0.013	9.21E-01	0.021	0.019	2.85E-01
3.274962	0.000	0.011	9.74E-01	0.012	0.017	4.76E-01
8.871518	-0.002	0.008	8.46E-01	-0.002	0.012	8.53E-01
0.599986787	0.001	0.010	9.46E-01	-0.010	0.014	5.06E-01
6.237924	0.002	0.010	8.49E-01	-0.023	0.014	1.01E-01
7.602495	0.009	0.013	4.84E-01	0.029	0.019	1.29E-01
6.747283	-0.002	0.010	8.57E-01	-0.009	0.016	5.69E-01
8.369225	0.002	0.007	7.59E-01	0.005	0.011	6.45E-01
6.306893	0.001	0.008	9.42E-01	0.009	0.012	4.74E-01
5.50551	-0.004	0.009	6.71E-01	-0.031	0.013	1.77E-02
0.612434793	0.004	0.010	7.15E-01	0.010	0.015	5.17E-01
6.935012	0.000	0.009	9.89E-01	0.001	0.013	9.33E-01
5.555302	0.000	0.010	1.00E+00	-0.018	0.014	2.16E-01
3.187826	-0.001	0.006	9.20E-01	0.012	0.009	1.77E-01
7.292977	-0.003	0.011	7.75E-01	-0.017	0.016	2.74E-01
8.231287	-0.004	0.010	6.77E-01	0.012	0.014	3.97E-01
0.616471984	0.001	0.009	9.56E-01	0.013	0.014	3.38E-01
0.57307218	0.000	0.008	9.66E-01	0.020	0.012	1.04E-01
2.366930756	0.006	0.011	5.77E-01	0.040	0.016	1.40E-02
8.204373	0.001	0.009	9.51E-01	0.002	0.013	8.67E-01
6.013524	-0.001	0.009	9.45E-01	-0.005	0.013	7.01E-01

0.993612919	-0.001	0.005	9.16E-01	0.009	0.008	2.48E-01
8.048604	-0.002	0.005	7.46E-01	0.006	0.008	4.81E-01
8.286799	0.002	0.008	7.81E-01	0.001	0.011	9.18E-01
6.92492	-0.002	0.012	8.79E-01	0.000	0.017	9.84E-01
2.274075361	0.000	0.012	9.85E-01	-0.003	0.017	8.61E-01
6.103351	0.003	0.010	7.79E-01	-0.023	0.014	1.10E-01
4.090475	0.006	0.011	5.58E-01	0.012	0.016	4.44E-01
7.371366	-0.001	0.010	9.19E-01	-0.002	0.015	8.97E-01
7.503584	-0.003	0.009	7.10E-01	-0.020	0.014	1.40E-01
3.787013	0.005	0.010	6.41E-01	0.027	0.016	8.30E-02
1.799705407	-0.001	0.010	9.06E-01	0.010	0.015	5.37E-01
8.106471	0.004	0.009	6.94E-01	-0.024	0.014	8.36E-02
3.207339	0.000	0.011	9.93E-01	0.013	0.016	4.34E-01
0.67299266	-0.002	0.008	8.46E-01	-0.003	0.012	8.08E-01
0.716392464	0.000	0.008	9.56E-01	-0.005	0.012	6.55E-01
4.201834	0.001	0.011	9.19E-01	0.019	0.017	2.52E-01
6.857969	0.000	0.008	9.71E-01	0.011	0.012	3.66E-01
1.759669929	0.000	0.008	9.89E-01	0.018	0.013	1.53E-01
8.129348	0.001	0.009	9.36E-01	0.002	0.014	8.93E-01
6.317322	0.000	0.009	9.71E-01	-0.028	0.014	4.47E-02
8.930394	-0.001	0.009	9.01E-01	-0.001	0.013	9.68E-01
1.187398092	0.001	0.010	9.46E-01	0.008	0.015	6.00E-01
6.829373	-0.001	0.007	9.22E-01	0.006	0.010	5.54E-01
2.648861267	-0.004	0.025	8.66E-01	0.015	0.037	6.97E-01
8.752085	0.001	0.008	9.35E-01	0.010	0.011	3.97E-01
6.352984	0.000	0.009	9.76E-01	-0.005	0.014	7.11E-01
7.334022	-0.001	0.006	8.38E-01	-0.006	0.009	5.02E-01
5.011627	0.001	0.009	9.06E-01	-0.003	0.013	8.11E-01
5.875923	-0.001	0.009	9.27E-01	0.007	0.014	5.96E-01
7.856165	0.000	0.006	9.47E-01	-0.004	0.010	6.71E-01
6.160881	0.001	0.007	8.76E-01	0.001	0.011	9.62E-01
2.61286298	-0.001	0.012	9.45E-01	0.036	0.018	3.74E-02
5.131061	0.001	0.007	8.91E-01	-0.010	0.011	3.45E-01
8.811297	0.000	0.006	9.52E-01	-0.008	0.009	3.78E-01
8.309676	-0.002	0.010	8.47E-01	0.015	0.014	2.85E-01
6.070717	0.000	0.009	9.87E-01	-0.008	0.014	5.83E-01
3.917549	0.000	0.009	9.87E-01	0.030	0.014	2.87E-02
2.814386102	0.001	0.016	9.31E-01	-0.066	0.023	4.50E-03
8.590597	0.002	0.006	6.88E-01	0.004	0.009	6.95E-01
7.790224	-0.004	0.009	6.97E-01	0.007	0.014	5.87E-01
1.723671642	0.000	0.005	9.64E-01	0.000	0.007	9.84E-01
8.163328	0.003	0.009	7.40E-01	0.018	0.013	1.70E-01
3.300868	0.003	0.013	8.21E-01	0.015	0.020	4.64E-01

7.599467	0.000	0.011	9.99E-01	0.009	0.016	5.86E-01
7.524779	0.001	0.007	8.89E-01	-0.002	0.010	8.29E-01
7.301388	-0.007	0.011	5.35E-01	-0.023	0.016	1.56E-01
0.677366283	0.001	0.010	8.99E-01	-0.003	0.015	8.20E-01
0.550194764	0.002	0.009	8.61E-01	0.006	0.013	6.23E-01
7.927152	-0.004	0.008	6.44E-01	-0.015	0.013	2.40E-01
2.747099584	-0.001	0.009	8.94E-01	-0.016	0.013	1.99E-01
8.08393	0.001	0.009	9.21E-01	0.001	0.014	9.37E-01
6.734162	-0.002	0.010	8.35E-01	-0.006	0.016	6.79E-01
8.185196	-0.002	0.009	8.35E-01	-0.018	0.014	1.92E-01
4.019151	-0.001	0.013	9.20E-01	0.010	0.019	5.81E-01
8.181832	-0.006	0.009	5.24E-01	-0.008	0.014	5.51E-01
4.027226	0.001	0.014	9.67E-01	-0.003	0.020	9.02E-01
6.697155	-0.001	0.006	9.22E-01	0.009	0.009	3.46E-01
7.811756	-0.002	0.008	7.55E-01	0.004	0.012	7.48E-01
6.098641	0.002	0.009	7.90E-01	-0.001	0.013	9.08E-01
0.78603401	-0.001	0.004	8.38E-01	0.000	0.006	9.62E-01
8.688836	-0.001	0.007	8.98E-01	0.008	0.010	4.20E-01
8.644426	-0.002	0.007	7.98E-01	0.017	0.011	1.29E-01
5.636046	0.000	0.009	9.59E-01	-0.022	0.014	1.15E-01
0.947858087	0.001	0.005	8.80E-01	-0.003	0.008	7.47E-01
2.846347198	0.003	0.009	7.31E-01	-0.009	0.013	5.07E-01
7.625372	-0.003	0.013	8.15E-01	0.014	0.020	4.76E-01
0.512850746	-0.001	0.008	9.45E-01	0.006	0.011	6.11E-01
1.030956937	-0.001	0.007	9.04E-01	0.018	0.010	6.51E-02
2.812703939	0.002	0.015	9.06E-01	-0.058	0.023	1.07E-02
0.643723024	-0.001	0.010	9.55E-01	-0.006	0.015	7.05E-01
6.341545	-0.001	0.007	8.34E-01	-0.010	0.010	2.98E-01
6.359713	0.001	0.009	9.26E-01	-0.016	0.013	2.09E-01
8.488658	0.000	0.008	9.87E-01	-0.004	0.012	7.52E-01
0.653143137	-0.003	0.010	7.88E-01	-0.007	0.015	6.57E-01
0.555577685	0.003	0.009	7.09E-01	-0.001	0.013	9.08E-01
8.935441	0.000	0.007	9.69E-01	-0.004	0.011	6.97E-01
3.316007	-0.002	0.012	8.79E-01	0.002	0.018	9.13E-01
5.718809	-0.002	0.009	8.60E-01	-0.017	0.013	1.91E-01
5.588609	-0.001	0.008	8.61E-01	-0.008	0.012	4.95E-01
6.041448	0.000	0.009	9.83E-01	0.018	0.013	1.83E-01
6.391337	-0.001	0.010	9.58E-01	-0.007	0.014	6.16E-01
0.681067042	-0.001	0.012	9.58E-01	-0.009	0.018	6.17E-01
6.348947	-0.001	0.009	9.00E-01	0.000	0.013	9.98E-01
7.138891	-0.001	0.009	9.26E-01	-0.019	0.014	1.73E-01
1.659749449	-0.001	0.004	8.80E-01	0.001	0.006	8.83E-01
7.454464	-0.001	0.009	9.54E-01	-0.009	0.013	4.81E-01

6.762759	0.003	0.008	7.38E-01	-0.001	0.011	9.31E-01
2.823806215	0.001	0.014	9.70E-01	-0.049	0.021	1.87E-02
3.317353	-0.001	0.013	9.39E-01	0.016	0.019	4.08E-01
5.406599	0.001	0.009	9.04E-01	-0.010	0.013	4.63E-01
7.318209	-0.002	0.006	7.08E-01	-0.007	0.009	4.44E-01
3.640665	0.001	0.004	7.65E-01	0.009	0.006	1.16E-01
0.993276486	-0.001	0.006	8.60E-01	0.010	0.008	2.48E-01
8.529367	0.004	0.009	6.10E-01	0.017	0.013	1.99E-01
4.29469	0.000	0.012	1.00E+00	0.019	0.018	2.96E-01
2.420087105	0.002	0.017	8.87E-01	-0.015	0.026	5.70E-01
8.973458	-0.001	0.010	9.08E-01	0.009	0.015	5.16E-01
7.374057	0.000	0.009	1.00E+00	0.013	0.014	3.71E-01
8.520283	0.000	0.007	9.74E-01	-0.014	0.010	1.54E-01
3.171005	-0.002	0.006	7.52E-01	0.010	0.009	2.68E-01
0.515205774	0.000	0.009	9.86E-01	0.006	0.014	6.63E-01
7.348825	-0.001	0.010	9.39E-01	-0.007	0.016	6.30E-01
0.521261561	0.001	0.009	9.29E-01	-0.003	0.014	8.17E-01
5.78845	-0.001	0.010	9.56E-01	0.008	0.015	6.04E-01
3.912502	0.000	0.008	9.72E-01	0.027	0.011	1.87E-02
5.731257	0.000	0.011	1.00E+00	-0.003	0.017	8.81E-01
5.794506	0.000	0.008	9.70E-01	0.001	0.011	9.65E-01
6.336162	0.002	0.009	7.86E-01	-0.027	0.013	4.13E-02
2.728595792	-0.003	0.009	7.66E-01	-0.017	0.014	2.29E-01
1.69103768	0.000	0.004	9.12E-01	0.001	0.005	7.99E-01
8.732908	-0.001	0.009	9.16E-01	-0.015	0.014	2.98E-01
6.39773	-0.002	0.009	8.13E-01	-0.011	0.013	4.31E-01
3.637637	0.001	0.004	7.90E-01	0.010	0.005	6.15E-02
8.628951	-0.002	0.008	8.33E-01	0.002	0.013	8.70E-01
3.912839	-0.002	0.008	8.25E-01	0.027	0.012	2.05E-02
7.332003	-0.003	0.006	6.36E-01	-0.003	0.009	7.09E-01
5.571451	0.005	0.009	5.89E-01	-0.005	0.014	7.36E-01
5.752788	-0.005	0.010	5.96E-01	0.000	0.015	9.80E-01
8.896414	-0.003	0.009	7.78E-01	0.007	0.014	5.93E-01
4.041019	0.005	0.015	7.37E-01	-0.015	0.022	4.94E-01
7.176908	0.003	0.010	7.59E-01	0.015	0.015	3.11E-01
0.551540494	0.005	0.009	5.46E-01	0.001	0.013	9.29E-01
5.773311	0.001	0.008	8.71E-01	-0.010	0.012	3.85E-01
7.77172	0.000	0.012	9.72E-01	-0.012	0.018	5.24E-01
6.323378	-0.002	0.008	8.00E-01	0.007	0.012	5.54E-01
3.1922	0.000	0.007	9.86E-01	0.017	0.011	1.06E-01
4.01545	0.001	0.013	9.39E-01	0.030	0.019	1.14E-01
2.734315146	-0.002	0.010	8.15E-01	-0.014	0.015	3.51E-01
7.687276	0.001	0.008	9.22E-01	-0.005	0.012	6.80E-01

8.654519	-0.001	0.009	8.72E-01	-0.022	0.013	8.61E-02
6.796066	0.002	0.009	8.01E-01	-0.002	0.014	8.90E-01
8.41868	0.000	0.009	9.84E-01	0.013	0.013	2.91E-01
7.620662	-0.004	0.013	7.63E-01	0.014	0.020	4.79E-01
6.902715	0.001	0.009	9.01E-01	0.013	0.013	3.32E-01
5.697613	0.002	0.007	8.04E-01	-0.005	0.011	6.52E-01
8.112863	0.000	0.009	1.00E+00	-0.007	0.013	6.00E-01
7.341759	0.001	0.009	8.67E-01	-0.021	0.013	1.08E-01
2.420759971	0.004	0.016	8.01E-01	-0.020	0.024	4.01E-01
0.511505016	-0.001	0.008	9.27E-01	-0.011	0.012	3.90E-01
2.387453144	-0.002	0.018	9.29E-01	-0.005	0.027	8.45E-01
7.933881	0.002	0.009	8.44E-01	0.008	0.014	5.39E-01
5.785086	-0.003	0.009	7.13E-01	-0.010	0.013	4.52E-01
2.85173012	0.001	0.008	9.08E-01	-0.007	0.012	5.44E-01
8.642408	-0.002	0.009	8.16E-01	-0.024	0.014	8.93E-02
5.289521	-0.002	0.009	8.67E-01	-0.025	0.014	7.79E-02
2.540529973	-0.001	0.022	9.74E-01	0.015	0.033	6.45E-01
5.655223	0.000	0.008	9.87E-01	0.007	0.012	5.25E-01
5.406936	0.001	0.010	9.03E-01	-0.009	0.015	5.73E-01
0.623873501	0.000	0.009	9.63E-01	0.028	0.013	3.43E-02
2.28046758	0.001	0.005	8.89E-01	-0.004	0.008	6.24E-01
4.232113	-0.001	0.013	9.46E-01	0.008	0.019	6.90E-01
8.759823	-0.002	0.008	8.40E-01	0.000	0.011	9.98E-01
1.460244923	0.000	0.004	8.92E-01	0.003	0.005	6.13E-01
3.371182	0.009	0.012	4.58E-01	0.015	0.017	3.82E-01
5.73092	-0.001	0.010	9.39E-01	0.003	0.015	8.47E-01
5.96575	0.002	0.010	8.77E-01	0.008	0.015	5.78E-01
0.674001957	0.000	0.010	9.99E-01	0.008	0.015	5.74E-01
8.393784	-0.001	0.008	9.50E-01	-0.004	0.012	7.26E-01
8.471837	0.002	0.009	8.40E-01	-0.021	0.013	1.16E-01
8.770252	0.000	0.008	9.61E-01	-0.008	0.011	4.64E-01
1.45721703	0.000	0.004	9.00E-01	0.005	0.005	3.11E-01
5.642102	0.001	0.008	9.22E-01	-0.012	0.011	2.87E-01
2.648188402	-0.003	0.023	8.83E-01	0.019	0.035	5.78E-01
8.815671	0.001	0.008	9.44E-01	-0.024	0.012	4.86E-02
7.007009	0.001	0.009	9.25E-01	-0.019	0.014	1.72E-01
8.19428	0.000	0.009	9.68E-01	-0.008	0.013	5.43E-01
8.502452	0.000	0.006	9.70E-01	0.002	0.009	8.12E-01
5.597693	-0.001	0.009	9.28E-01	-0.008	0.013	5.61E-01
7.845736	0.000	0.010	9.81E-01	-0.001	0.015	9.35E-01
2.834572058	0.002	0.011	8.72E-01	-0.036	0.016	3.10E-02
8.846959	-0.001	0.007	8.59E-01	-0.009	0.011	4.20E-01
7.951375	0.001	0.006	8.73E-01	-0.006	0.009	5.38E-01

8.43281	-0.001	0.009	9.24E-01	-0.001	0.014	9.68E-01
2.80765745	-0.001	0.015	9.58E-01	-0.069	0.022	1.68E-03
8.96	0.002	0.007	7.48E-01	-0.004	0.011	7.22E-01
8.994653	0.000	0.007	9.67E-01	0.004	0.010	7.06E-01
2.520007585	0.000	0.019	9.95E-01	-0.012	0.029	6.69E-01
7.645895	0.005	0.011	6.63E-01	-0.011	0.016	5.21E-01
5.43856	0.002	0.007	8.06E-01	0.004	0.010	6.96E-01
0.632284316	0.000	0.009	9.58E-01	0.002	0.014	8.71E-01
4.293007	0.000	0.012	9.81E-01	0.016	0.017	3.64E-01
7.854483	0.000	0.008	9.93E-01	0.010	0.011	3.68E-01
2.581238317	0.004	0.011	7.10E-01	0.010	0.016	5.15E-01
7.369347	0.000	0.010	9.97E-01	0.012	0.014	3.83E-01
6.233551	0.001	0.007	8.82E-01	-0.009	0.010	3.52E-01
8.613811	-0.001	0.008	8.96E-01	0.007	0.012	5.53E-01
2.035881086	0.001	0.004	7.69E-01	0.009	0.006	1.40E-01
8.788756	0.000	0.009	9.61E-01	-0.010	0.013	4.37E-01
3.798115	0.003	0.006	6.26E-01	0.013	0.009	1.61E-01
2.277103254	-0.001	0.011	9.18E-01	0.012	0.016	4.64E-01
0.733886959	0.000	0.007	9.88E-01	-0.004	0.010	7.23E-01
2.729605089	-0.001	0.009	9.15E-01	-0.016	0.014	2.71E-01
2.827170541	0.002	0.013	9.05E-01	-0.051	0.019	7.05E-03
2.817077563	0.000	0.015	9.78E-01	-0.061	0.022	6.12E-03
6.665194	0.005	0.009	5.33E-01	0.017	0.013	1.91E-01
0.966361879	-0.002	0.007	7.67E-01	0.017	0.011	1.10E-01
2.810348911	0.002	0.014	9.03E-01	-0.065	0.022	2.74E-03
6.277623	0.001	0.007	8.97E-01	-0.022	0.010	2.79E-02
8.706667	0.000	0.008	9.51E-01	-0.016	0.012	1.90E-01
7.90091	0.003	0.010	7.17E-01	0.002	0.014	8.68E-01
7.433942	-0.004	0.010	6.84E-01	-0.020	0.015	1.67E-01
8.15559	0.001	0.011	9.48E-01	-0.006	0.016	7.03E-01
5.727892	0.001	0.011	9.58E-01	-0.014	0.017	3.91E-01
8.359805	-0.001	0.009	8.95E-01	-0.012	0.013	3.50E-01
7.655651	0.002	0.010	8.69E-01	0.003	0.015	8.34E-01
6.93333	-0.002	0.010	8.28E-01	-0.002	0.014	9.00E-01
8.410942	-0.001	0.005	9.03E-01	0.003	0.008	6.89E-01
8.512881	0.000	0.008	1.00E+00	0.009	0.011	4.40E-01
7.905621	0.002	0.009	8.48E-01	0.014	0.013	2.97E-01
4.204862	0.001	0.014	9.50E-01	0.014	0.020	5.05E-01
6.349956	0.002	0.007	8.09E-01	-0.007	0.010	4.83E-01
7.843044	-0.001	0.008	9.25E-01	0.006	0.012	6.29E-01
5.778021	0.001	0.009	9.32E-01	-0.022	0.014	1.05E-01
7.520069	-0.001	0.007	9.02E-01	0.000	0.010	9.66E-01
7.144946	-0.001	0.010	9.05E-01	-0.012	0.014	4.14E-01

7.816466	0.001	0.010	9.42E-01	0.013	0.014	3.56E-01
2.273402496	0.001	0.008	9.38E-01	-0.007	0.012	5.80E-01
3.743276	-0.005	0.009	5.99E-01	-0.002	0.014	8.94E-01
0.855675557	0.000	0.007	9.63E-01	-0.009	0.011	3.92E-01
6.640634	-0.001	0.007	9.33E-01	-0.001	0.010	9.58E-01
7.007345	-0.001	0.010	9.37E-01	-0.014	0.014	3.25E-01
5.56304	0.001	0.011	9.36E-01	-0.041	0.017	1.29E-02
8.349039	-0.001	0.008	9.32E-01	0.004	0.013	7.72E-01
1.504317592	0.000	0.006	9.86E-01	-0.016	0.008	6.27E-02
3.563285	0.001	0.006	8.23E-01	0.018	0.009	5.84E-02
1.663786641	0.001	0.004	8.87E-01	0.006	0.006	3.61E-01
6.647363	-0.001	0.009	9.11E-01	-0.010	0.013	4.36E-01
5.415683	0.000	0.009	9.59E-01	-0.015	0.013	2.63E-01
7.70477	-0.001	0.009	8.86E-01	0.006	0.013	6.31E-01
1.701130658	0.000	0.004	9.97E-01	0.006	0.006	2.88E-01
5.68281	0.000	0.009	9.57E-01	-0.001	0.014	9.52E-01
7.730339	0.002	0.011	8.69E-01	0.012	0.016	4.57E-01
2.812031074	0.002	0.015	9.01E-01	-0.064	0.022	4.12E-03
8.776644	0.001	0.008	8.56E-01	0.014	0.011	2.20E-01
3.180761	0.001	0.005	7.79E-01	0.010	0.008	1.91E-01
4.165499	0.000	0.011	9.70E-01	0.001	0.016	9.42E-01
6.39201	0.000	0.008	9.52E-01	-0.011	0.012	3.62E-01
5.616197	-0.001	0.008	9.10E-01	0.007	0.013	5.70E-01
6.911799	0.000	0.009	9.82E-01	-0.004	0.014	7.75E-01
4.041356	0.004	0.014	7.72E-01	-0.017	0.021	4.32E-01
1.160819917	0.002	0.006	6.82E-01	-0.018	0.008	3.50E-02
7.532853	0.001	0.009	8.78E-01	0.008	0.013	5.61E-01
8.115554	0.001	0.009	9.26E-01	0.003	0.014	8.34E-01
1.672533888	0.000	0.004	9.92E-01	0.005	0.006	3.48E-01
8.958318	-0.001	0.009	8.77E-01	-0.035	0.014	1.18E-02
3.182107	0.002	0.005	7.70E-01	0.014	0.008	6.88E-02
7.91134	0.000	0.008	9.71E-01	0.008	0.012	5.06E-01
3.248384	-0.001	0.007	8.69E-01	0.013	0.010	1.91E-01
5.968105	-0.002	0.009	8.43E-01	-0.002	0.013	8.58E-01
7.807382	0.001	0.008	9.09E-01	-0.007	0.012	5.47E-01
7.786523	-0.002	0.007	7.39E-01	-0.005	0.011	6.21E-01
7.701069	-0.001	0.009	9.44E-01	-0.002	0.014	8.88E-01
2.854421581	0.003	0.009	7.13E-01	-0.004	0.013	7.70E-01
7.349497	-0.001	0.011	9.07E-01	-0.006	0.016	7.23E-01
5.1462	0.001	0.009	9.55E-01	-0.012	0.014	3.81E-01
8.964374	0.003	0.007	7.15E-01	-0.002	0.011	8.71E-01
8.237343	-0.003	0.008	6.95E-01	-0.013	0.013	2.87E-01
1.502635429	-0.001	0.005	8.78E-01	-0.009	0.008	2.32E-01

8.813989	0.000	0.009	9.77E-01	-0.005	0.013	6.97E-01
6.691772	0.002	0.010	8.79E-01	0.016	0.015	2.92E-01
0.727831172	-0.003	0.007	6.45E-01	-0.010	0.010	3.40E-01
3.187153	0.001	0.006	9.17E-01	0.013	0.009	1.29E-01
7.55674	0.000	0.007	9.66E-01	-0.004	0.010	6.97E-01
3.314998	-0.001	0.011	9.35E-01	0.004	0.016	8.07E-01
5.607786	-0.001	0.008	9.36E-01	-0.004	0.012	7.71E-01
6.373843	0.000	0.007	9.83E-01	-0.008	0.011	4.40E-01
3.551846	-0.004	0.014	7.52E-01	0.059	0.021	4.00E-03
8.338273	-0.001	0.009	9.14E-01	-0.008	0.013	5.36E-01
8.574785	0.001	0.008	8.58E-01	0.002	0.011	8.36E-01
2.078944458	0.001	0.004	7.07E-01	0.010	0.005	7.30E-02
0.71975679	-0.002	0.008	7.95E-01	-0.007	0.011	5.59E-01
4.203516	0.003	0.012	7.97E-01	0.021	0.019	2.70E-01
8.714068	0.000	0.008	9.65E-01	-0.013	0.012	2.60E-01
6.974039	-0.003	0.010	7.90E-01	-0.008	0.016	6.18E-01
8.048268	-0.002	0.007	7.72E-01	0.011	0.010	2.59E-01
8.068454	-0.001	0.010	9.22E-01	0.000	0.014	9.95E-01
5.146873	-0.001	0.008	9.33E-01	-0.013	0.012	2.69E-01
1.672197455	0.000	0.004	9.39E-01	0.006	0.006	3.39E-01
8.287135	0.001	0.009	9.14E-01	-0.002	0.014	8.57E-01
1.442413996	0.001	0.004	8.14E-01	0.007	0.006	2.40E-01
3.66657	0.001	0.005	8.03E-01	0.012	0.007	1.05E-01
0.679384879	-0.001	0.011	9.59E-01	-0.038	0.017	2.30E-02
4.022179	0.000	0.014	9.86E-01	0.024	0.021	2.51E-01
3.274626	0.000	0.010	9.85E-01	0.015	0.016	3.46E-01
3.30255	0.002	0.014	8.72E-01	0.014	0.020	4.76E-01
2.277439687	-0.003	0.009	7.66E-01	0.005	0.014	7.01E-01
2.387789577	-0.001	0.018	9.62E-01	-0.007	0.027	7.88E-01
5.766582	0.001	0.009	9.47E-01	-0.008	0.013	5.31E-01
8.716759	-0.002	0.009	8.30E-01	-0.017	0.013	1.77E-01
8.682107	-0.001	0.009	9.41E-01	0.003	0.013	8.24E-01
6.124546	-0.002	0.010	8.53E-01	-0.007	0.015	6.17E-01
8.879593	0.001	0.008	8.74E-01	0.008	0.012	4.87E-01
0.579127967	0.001	0.008	8.64E-01	0.013	0.012	2.60E-01
4.228749	0.000	0.012	9.98E-01	0.018	0.019	3.23E-01
6.165255	-0.001	0.008	9.30E-01	0.004	0.012	7.36E-01
8.963365	0.000	0.009	9.99E-01	0.010	0.013	4.64E-01
7.664398	0.002	0.010	8.06E-01	-0.017	0.015	2.50E-01
0.718747492	-0.001	0.008	9.12E-01	0.008	0.011	4.58E-01
0.58350159	-0.001	0.008	9.43E-01	0.010	0.012	3.97E-01
3.859346	-0.001	0.005	8.56E-01	0.012	0.008	1.48E-01
6.858979	0.001	0.008	9.19E-01	0.003	0.012	8.15E-01

6.041784	0.000	0.008	9.51E-01	0.017	0.012	1.53E-01
4.121427	0.003	0.011	7.93E-01	0.019	0.016	2.39E-01
2.733305848	-0.001	0.010	9.17E-01	-0.014	0.015	3.40E-01
8.516582	-0.001	0.007	8.94E-01	-0.006	0.011	5.92E-01
8.583196	-0.001	0.008	8.91E-01	0.024	0.012	4.43E-02
8.419017	0.000	0.009	9.61E-01	0.009	0.013	4.68E-01
1.723335209	-0.001	0.005	8.65E-01	-0.001	0.007	8.82E-01
6.453577	-0.002	0.009	7.78E-01	-0.006	0.013	6.65E-01
2.858795	0.000	0.008	9.55E-01	-0.004	0.012	7.11E-01
5.432505	-0.002	0.009	8.43E-01	0.007	0.013	5.83E-01
7.669445	-0.002	0.010	8.60E-01	-0.014	0.015	3.75E-01
5.427122	0.001	0.006	8.80E-01	-0.009	0.009	3.49E-01
3.928314	0.001	0.008	8.82E-01	0.017	0.013	1.73E-01
4.112679	0.005	0.010	6.26E-01	0.007	0.015	6.23E-01
4.040683	0.004	0.015	7.99E-01	-0.014	0.022	5.16E-01
7.966178	-0.004	0.009	6.70E-01	-0.005	0.014	7.14E-01
8.549552	-0.001	0.006	8.54E-01	0.001	0.009	9.09E-01
6.276614	0.000	0.007	9.52E-01	-0.004	0.011	7.31E-01
7.95003	0.001	0.008	8.84E-01	-0.010	0.012	4.02E-01
7.525452	0.000	0.009	9.80E-01	-0.045	0.013	8.92E-04
0.999668706	0.001	0.006	8.38E-01	0.005	0.010	6.12E-01
8.918619	0.002	0.009	8.50E-01	-0.003	0.013	8.36E-01
6.324051	0.000	0.008	9.76E-01	0.007	0.013	5.92E-01
5.62057	-0.003	0.009	7.78E-01	0.002	0.014	8.96E-01
2.081299486	0.000	0.005	9.29E-01	0.010	0.008	1.80E-01
4.037991	0.002	0.014	8.68E-01	-0.013	0.022	5.53E-01
2.078271593	0.002	0.004	6.75E-01	0.009	0.005	8.03E-02
8.588579	-0.002	0.007	8.12E-01	0.006	0.010	5.56E-01
3.56564	0.001	0.006	8.32E-01	0.022	0.009	2.24E-02
7.551693	0.002	0.008	7.89E-01	-0.002	0.012	8.49E-01
3.29784	0.001	0.011	9.31E-01	0.003	0.016	8.71E-01
7.319891	-0.002	0.006	7.18E-01	-0.004	0.009	6.83E-01
2.533128456	0.000	0.011	9.95E-01	0.007	0.017	6.90E-01
0.823378028	-0.001	0.007	8.20E-01	0.008	0.010	3.94E-01
4.022516	0.001	0.013	9.33E-01	0.018	0.020	3.71E-01
6.386627	0.000	0.007	9.80E-01	-0.007	0.011	5.39E-01
1.673206753	-0.001	0.004	8.69E-01	0.003	0.006	6.32E-01
8.306985	-0.001	0.008	9.50E-01	-0.005	0.012	6.83E-01
6.494959	-0.001	0.009	9.14E-01	-0.003	0.013	8.21E-01
8.307321	-0.001	0.009	8.78E-01	0.000	0.013	9.91E-01
8.059707	0.001	0.007	9.02E-01	0.006	0.011	5.64E-01
6.177366	0.004	0.009	6.37E-01	-0.021	0.013	1.06E-01
5.146537	-0.001	0.008	8.95E-01	-0.014	0.012	2.50E-01

4.289307	0.002	0.011	8.42E-01	0.016	0.016	3.17E-01
8.003522	0.001	0.006	8.66E-01	-0.007	0.009	4.11E-01
3.878186	-0.001	0.005	8.98E-01	0.017	0.007	1.57E-02
6.257437	0.000	0.009	9.98E-01	-0.016	0.014	2.47E-01
4.200825	0.003	0.012	8.07E-01	0.017	0.018	3.31E-01
7.512667	-0.001	0.009	9.07E-01	-0.002	0.013	8.53E-01
6.703883	0.001	0.009	9.06E-01	-0.015	0.013	2.39E-01
2.32588598	0.001	0.007	8.34E-01	-0.007	0.010	5.05E-01
7.255969	-0.003	0.010	7.47E-01	0.020	0.015	1.86E-01
6.400758	0.001	0.007	9.29E-01	-0.005	0.010	6.59E-01
7.144274	0.000	0.009	9.75E-01	0.001	0.014	9.40E-01
8.239362	0.001	0.009	8.78E-01	-0.012	0.014	3.80E-01
0.75609151	0.002	0.005	7.65E-01	-0.001	0.008	8.66E-01
8.32818	0.003	0.006	6.70E-01	-0.004	0.009	6.35E-01
8.848977	-0.004	0.009	6.33E-01	-0.014	0.014	3.30E-01
8.582186	-0.001	0.007	8.83E-01	0.012	0.010	2.59E-01
6.696145	-0.002	0.008	8.01E-01	0.002	0.013	8.82E-01
1.837049425	0.002	0.010	8.05E-01	-0.010	0.014	4.87E-01
3.687765	0.000	0.004	9.12E-01	0.006	0.007	3.45E-01
6.173666	0.002	0.009	8.12E-01	0.003	0.014	8.43E-01
4.008385	-0.003	0.010	7.90E-01	0.012	0.015	4.33E-01
0.824050893	-0.002	0.007	7.13E-01	0.007	0.010	4.76E-01
5.818056	-0.001	0.008	8.72E-01	-0.006	0.013	6.11E-01
8.023035	-0.001	0.009	9.42E-01	-0.012	0.014	3.99E-01
6.799767	0.001	0.009	9.48E-01	-0.006	0.014	6.58E-01
6.514135	0.003	0.008	7.04E-01	-0.009	0.012	4.35E-01
8.972785	0.002	0.008	8.13E-01	-0.006	0.011	5.78E-01
6.770497	0.003	0.009	7.73E-01	-0.013	0.013	3.34E-01
5.078241	0.001	0.009	9.35E-01	-0.010	0.013	4.70E-01
5.829159	0.001	0.008	9.19E-01	0.012	0.012	3.44E-01
8.327507	0.000	0.007	9.98E-01	-0.019	0.010	5.78E-02
6.829036	-0.001	0.008	9.15E-01	0.000	0.012	9.73E-01
4.232786	0.000	0.013	9.78E-01	0.005	0.020	7.83E-01
6.350629	-0.001	0.008	9.49E-01	-0.010	0.013	4.45E-01
8.56301	-0.001	0.007	9.07E-01	-0.002	0.010	8.51E-01
7.438316	0.003	0.007	6.57E-01	0.009	0.011	3.89E-01
5.533098	0.000	0.008	9.87E-01	0.001	0.012	9.22E-01
4.089129	0.006	0.010	5.56E-01	-0.002	0.015	8.76E-01
0.720093222	-0.002	0.007	8.05E-01	-0.012	0.011	2.73E-01
6.076437	0.002	0.008	7.64E-01	-0.001	0.012	9.17E-01
6.128584	-0.001	0.009	9.55E-01	-0.026	0.014	6.35E-02
5.684493	0.000	0.008	9.60E-01	-0.003	0.012	8.27E-01
7.608887	0.002	0.012	8.90E-01	-0.004	0.017	8.38E-01

2.829862001	0.005	0.012	6.88E-01	-0.035	0.017	4.10E-02
6.652073	0.005	0.010	6.03E-01	-0.005	0.014	7.21E-01
1.663450208	0.000	0.004	9.67E-01	0.001	0.006	8.77E-01
7.477005	0.000	0.008	9.54E-01	-0.007	0.011	5.62E-01
1.668496697	-0.002	0.004	6.35E-01	0.003	0.006	6.83E-01
8.010924	-0.001	0.007	8.37E-01	0.010	0.011	3.69E-01
0.568698556	0.005	0.009	5.98E-01	0.006	0.014	6.77E-01
0.687122828	0.003	0.015	8.37E-01	-0.024	0.022	2.74E-01
7.817139	0.000	0.010	9.99E-01	0.019	0.014	1.84E-01
7.533526	0.002	0.010	8.62E-01	0.010	0.014	4.61E-01
5.793497	-0.001	0.008	8.51E-01	-0.008	0.012	4.73E-01
3.937398	0.003	0.007	6.56E-01	0.014	0.011	1.74E-01
7.23612	-0.004	0.010	6.90E-01	-0.009	0.015	5.35E-01
2.81674113	-0.002	0.015	8.94E-01	-0.062	0.022	5.57E-03
2.846010766	0.005	0.009	5.70E-01	0.001	0.013	9.38E-01
2.732969415	-0.001	0.010	9.12E-01	-0.015	0.015	3.18E-01
2.810685344	0.001	0.015	9.41E-01	-0.068	0.022	2.02E-03
7.578608	0.000	0.010	9.65E-01	-0.009	0.015	5.41E-01
7.319218	-0.001	0.006	8.95E-01	-0.003	0.009	7.41E-01
8.592952	0.001	0.008	8.65E-01	0.007	0.012	5.46E-01
7.156049	-0.001	0.007	9.07E-01	-0.003	0.010	7.81E-01
8.737955	-0.001	0.009	9.38E-01	0.006	0.014	6.79E-01
7.319555	-0.002	0.006	8.05E-01	-0.005	0.009	5.99E-01
3.516521	0.000	0.007	9.81E-01	0.014	0.011	1.95E-01
7.397944	0.002	0.010	8.43E-01	0.002	0.015	9.00E-01
6.850904	-0.002	0.008	8.01E-01	0.001	0.012	9.49E-01
7.225354	-0.003	0.010	7.99E-01	-0.006	0.016	6.85E-01
0.756764375	0.002	0.005	7.47E-01	0.005	0.008	5.31E-01
8.140114	-0.002	0.007	8.24E-01	-0.001	0.011	9.48E-01
8.311358	0.001	0.007	8.47E-01	0.005	0.010	6.62E-01
3.63259	0.000	0.004	9.98E-01	0.009	0.006	1.21E-01
7.686939	0.002	0.007	8.06E-01	0.005	0.011	6.47E-01
4.030253	0.002	0.014	8.63E-01	-0.002	0.021	9.39E-01
8.084939	0.000	0.009	9.71E-01	0.011	0.014	4.22E-01
1.502971862	0.000	0.005	9.97E-01	-0.012	0.008	1.43E-01
6.101333	0.002	0.010	8.38E-01	-0.041	0.015	6.02E-03
4.027898	0.001	0.013	9.30E-01	-0.003	0.019	8.85E-01
7.86323	-0.004	0.011	7.13E-01	0.000	0.016	9.87E-01
1.676907512	0.000	0.004	9.78E-01	0.002	0.006	6.94E-01
8.571421	-0.001	0.006	8.79E-01	0.018	0.009	5.54E-02
4.134211	-0.001	0.013	9.14E-01	0.017	0.019	3.85E-01
0.972754098	0.000	0.005	9.77E-01	0.017	0.007	1.88E-02
2.811021776	0.002	0.015	9.17E-01	-0.068	0.022	1.78E-03

8.556281	0.000	0.008	9.59E-01	-0.005	0.011	6.79E-01
8.16501	-0.002	0.009	8.38E-01	0.002	0.013	8.50E-01
6.482511	-0.001	0.009	9.12E-01	-0.012	0.013	3.58E-01
6.139349	-0.001	0.008	9.36E-01	-0.006	0.012	5.90E-01
8.516919	0.001	0.010	8.98E-01	-0.005	0.015	7.56E-01
7.704434	-0.002	0.011	8.86E-01	0.007	0.016	6.46E-01
7.980309	0.001	0.010	9.03E-01	0.023	0.015	1.22E-01
7.268754	-0.002	0.011	8.44E-01	-0.023	0.016	1.40E-01
5.022057	0.001	0.010	9.57E-01	-0.005	0.015	7.61E-01
6.229177	0.003	0.008	7.51E-01	-0.006	0.013	6.11E-01
2.805975287	0.001	0.014	9.66E-01	-0.067	0.022	1.91E-03
5.838242	-0.002	0.007	7.93E-01	-0.003	0.011	8.11E-01
5.112221	0.000	0.008	9.64E-01	-0.001	0.012	9.15E-01
1.048114999	-0.005	0.014	7.30E-01	-0.015	0.020	4.73E-01
2.074907267	0.001	0.003	8.36E-01	0.007	0.005	1.40E-01
6.925592	-0.003	0.012	8.25E-01	0.015	0.018	3.95E-01
0.681403474	-0.001	0.012	9.19E-01	-0.015	0.018	4.13E-01
3.935716	0.003	0.006	6.80E-01	0.023	0.009	1.39E-02
6.514472	0.002	0.009	8.60E-01	-0.010	0.014	4.65E-01
8.011597	-0.001	0.010	8.89E-01	0.007	0.014	6.09E-01
6.217738	0.001	0.009	8.99E-01	-0.006	0.013	6.74E-01
4.004348	-0.001	0.010	8.93E-01	0.020	0.015	1.92E-01
2.829525569	0.004	0.012	7.48E-01	-0.034	0.017	4.92E-02
7.935227	0.004	0.008	6.59E-01	0.003	0.012	7.81E-01
7.891827	0.002	0.008	8.06E-01	0.014	0.012	2.14E-01
7.294659	0.002	0.011	8.61E-01	-0.006	0.016	7.07E-01
7.888799	0.001	0.009	9.22E-01	0.022	0.014	1.20E-01
6.186114	0.001	0.008	9.35E-01	0.004	0.012	7.38E-01
7.971225	0.001	0.009	9.54E-01	-0.005	0.014	7.35E-01
1.649992904	0.000	0.005	9.17E-01	-0.002	0.007	7.27E-01
7.472968	0.004	0.009	6.49E-01	-0.019	0.013	1.45E-01
6.403785	0.002	0.008	8.20E-01	0.006	0.012	6.17E-01
6.215047	0.001	0.008	9.44E-01	0.002	0.012	8.91E-01
8.272332	-0.001	0.009	9.31E-01	0.020	0.014	1.47E-01
5.612832	-0.002	0.009	7.91E-01	-0.011	0.013	4.12E-01
0.723121116	-0.001	0.007	8.56E-01	-0.001	0.011	9.19E-01
6.548115	0.001	0.008	9.48E-01	0.001	0.012	9.23E-01
2.273738928	0.000	0.010	9.69E-01	-0.005	0.014	7.45E-01
6.190487	0.001	0.009	8.95E-01	-0.013	0.014	3.25E-01
6.751657	0.000	0.012	9.99E-01	-0.022	0.018	2.10E-01
2.550622951	0.000	0.009	9.61E-01	-0.015	0.014	2.98E-01
0.593931001	-0.001	0.009	9.41E-01	-0.004	0.013	7.51E-01
1.460917788	0.000	0.004	9.77E-01	0.000	0.006	9.60E-01

8.540805	-0.002	0.009	7.82E-01	-0.003	0.013	7.88E-01
3.937735	0.003	0.007	6.80E-01	0.013	0.011	2.10E-01
5.946237	-0.001	0.008	9.14E-01	-0.016	0.012	1.68E-01
8.513554	0.002	0.009	8.44E-01	0.005	0.013	7.17E-01
5.543527	0.001	0.009	9.21E-01	-0.012	0.013	3.51E-01
8.238352	-0.004	0.008	6.28E-01	-0.006	0.011	6.04E-01
7.880388	0.001	0.010	8.83E-01	0.011	0.014	4.61E-01
2.735324443	-0.002	0.010	8.31E-01	-0.013	0.015	4.10E-01
1.481440176	0.002	0.007	7.19E-01	-0.002	0.010	8.76E-01
7.669781	-0.001	0.009	9.07E-01	-0.014	0.013	2.81E-01
8.577813	-0.002	0.008	7.91E-01	-0.007	0.012	5.37E-01
5.490707	-0.002	0.008	7.81E-01	-0.007	0.012	5.24E-01
8.065762	0.003	0.008	6.98E-01	0.012	0.013	3.43E-01
6.203272	-0.001	0.008	8.60E-01	-0.005	0.011	6.35E-01
8.990616	-0.003	0.007	6.99E-01	0.006	0.010	5.78E-01
0.798482016	-0.002	0.006	6.83E-01	0.003	0.009	7.22E-01
5.457401	-0.001	0.007	9.32E-01	-0.019	0.011	6.98E-02
1.189416687	0.000	0.011	9.72E-01	-0.001	0.016	9.35E-01
4.180975	-0.001	0.011	9.43E-01	0.008	0.017	6.56E-01
7.108612	0.002	0.009	8.59E-01	0.010	0.013	4.53E-01
8.248782	0.002	0.010	8.74E-01	-0.003	0.014	8.55E-01
8.538787	-0.003	0.007	7.10E-01	0.010	0.011	3.80E-01
5.460765	0.002	0.010	8.81E-01	0.008	0.015	6.13E-01
1.839068021	0.002	0.010	8.23E-01	-0.006	0.015	7.03E-01
3.917212	-0.001	0.009	8.84E-01	0.031	0.014	2.09E-02
7.878369	-0.002	0.009	8.45E-01	-0.006	0.013	6.47E-01
2.275757524	-0.002	0.016	9.15E-01	0.007	0.025	7.66E-01
8.989943	-0.002	0.008	8.27E-01	0.016	0.012	1.87E-01
4.2126	0.003	0.012	8.07E-01	0.008	0.018	6.65E-01
6.663511	-0.002	0.010	8.40E-01	0.007	0.015	6.41E-01
1.88314069	0.000	0.008	1.00E+00	-0.015	0.013	2.22E-01
7.445044	0.002	0.009	8.20E-01	-0.012	0.013	3.52E-01
5.757835	0.000	0.008	9.68E-01	-0.004	0.012	7.69E-01
2.839618547	0.004	0.009	6.86E-01	-0.002	0.013	9.08E-01
5.058728	-0.001	0.008	8.74E-01	-0.016	0.012	1.87E-01
6.116136	-0.001	0.009	9.15E-01	0.011	0.013	3.97E-01
8.490004	0.001	0.008	8.89E-01	-0.014	0.011	2.18E-01
2.734988011	-0.002	0.010	8.20E-01	-0.013	0.015	3.92E-01
3.99762	0.000	0.008	9.78E-01	0.011	0.012	3.69E-01
7.705107	-0.001	0.011	9.24E-01	0.030	0.017	7.25E-02
2.277776119	-0.002	0.008	8.17E-01	0.002	0.012	8.93E-01
7.992757	0.001	0.006	8.76E-01	-0.013	0.010	1.88E-01
7.294322	-0.001	0.011	9.39E-01	0.013	0.016	4.40E-01

2.081972351	0.001	0.006	8.71E-01	0.009	0.009	3.05E-01
7.432933	0.000	0.010	9.76E-01	-0.005	0.016	7.46E-01
7.99242	0.000	0.006	9.95E-01	-0.012	0.009	1.67E-01
7.684584	0.000	0.007	9.58E-01	-0.002	0.010	8.70E-01
6.204281	-0.001	0.008	9.40E-01	-0.021	0.012	6.43E-02
8.869836	0.003	0.009	7.06E-01	-0.015	0.014	2.55E-01
7.42183	-0.002	0.007	7.36E-01	-0.012	0.011	2.61E-01
8.761505	0.001	0.007	9.16E-01	-0.006	0.011	5.84E-01
2.728932224	-0.002	0.009	8.00E-01	-0.017	0.014	2.38E-01
4.211591	0.002	0.010	8.69E-01	0.000	0.015	9.98E-01
0.763829459	0.000	0.005	9.52E-01	-0.002	0.008	8.11E-01
5.773647	0.001	0.007	8.80E-01	-0.001	0.010	9.01E-01
8.185532	-0.003	0.011	8.11E-01	-0.015	0.016	3.39E-01
7.916723	0.003	0.010	7.46E-01	-0.027	0.015	8.47E-02
0.710000245	0.002	0.008	8.01E-01	-0.004	0.013	7.40E-01
4.360967	-0.001	0.013	9.65E-01	0.005	0.020	8.09E-01
6.669231	-0.001	0.008	9.14E-01	-0.014	0.012	2.22E-01
0.544138977	0.003	0.010	7.51E-01	0.012	0.014	3.87E-01
4.089466	0.006	0.010	5.19E-01	0.003	0.015	8.30E-01
0.787716173	-0.002	0.004	7.18E-01	0.001	0.007	9.11E-01
2.068515048	0.002	0.004	6.18E-01	0.012	0.006	4.41E-02
5.958349	0.000	0.009	9.91E-01	-0.021	0.013	1.18E-01
8.763524	0.001	0.008	8.73E-01	0.002	0.011	8.89E-01
8.679079	-0.001	0.007	8.31E-01	0.000	0.010	9.81E-01
7.293313	-0.003	0.010	7.97E-01	-0.018	0.016	2.53E-01
4.090138	0.005	0.011	6.13E-01	0.009	0.016	5.53E-01
4.034627	0.001	0.016	9.39E-01	-0.019	0.023	4.22E-01
6.863016	0.000	0.009	9.85E-01	-0.001	0.013	9.68E-01
6.535331	-0.003	0.009	6.98E-01	-0.008	0.013	5.55E-01
6.668894	-0.001	0.009	9.25E-01	-0.026	0.014	5.77E-02
7.601485	0.006	0.011	5.81E-01	0.015	0.016	3.52E-01
7.022485	-0.003	0.009	7.72E-01	-0.021	0.014	1.34E-01
8.377635	0.002	0.007	8.24E-01	-0.005	0.010	6.02E-01
7.862557	-0.003	0.009	7.70E-01	0.016	0.013	2.17E-01
3.893662	0.000	0.005	9.42E-01	0.019	0.007	7.14E-03
5.570442	0.000	0.007	9.61E-01	-0.004	0.010	6.83E-01
7.806709	0.001	0.009	9.01E-01	-0.018	0.013	1.66E-01
7.897546	0.000	0.009	9.90E-01	-0.008	0.013	5.50E-01
6.356348	0.000	0.009	9.90E-01	-0.017	0.013	1.94E-01
5.51594	0.000	0.007	9.96E-01	-0.026	0.011	1.62E-02
7.247895	-0.001	0.009	9.07E-01	0.000	0.014	9.98E-01
8.835184	-0.002	0.006	8.10E-01	-0.004	0.010	6.58E-01
5.112894	-0.001	0.008	9.11E-01	-0.010	0.011	4.02E-01

5.565059	0.000	0.008	9.68E-01	-0.031	0.013	1.33E-02
3.295148	0.001	0.012	9.25E-01	0.011	0.017	5.15E-01
2.734651578	-0.002	0.010	8.28E-01	-0.014	0.015	3.59E-01
0.980828481	0.000	0.006	9.42E-01	0.012	0.010	2.12E-01
6.18174	-0.002	0.009	8.63E-01	0.011	0.013	4.25E-01
7.095827	0.000	0.009	9.91E-01	-0.005	0.014	6.90E-01
2.572154637	0.001	0.010	8.87E-01	0.007	0.014	6.32E-01
5.291539	0.000	0.010	9.93E-01	-0.026	0.015	8.86E-02
6.268203	0.005	0.009	5.54E-01	0.010	0.013	4.60E-01
5.110539	0.001	0.008	8.55E-01	-0.014	0.012	2.16E-01
2.817413996	-0.002	0.015	9.16E-01	-0.067	0.022	2.43E-03
6.102342	-0.001	0.008	9.09E-01	0.013	0.012	2.77E-01
2.072215806	0.001	0.003	7.62E-01	0.011	0.005	2.08E-02
6.367787	-0.002	0.009	8.16E-01	0.010	0.013	4.34E-01
6.386964	0.002	0.007	8.19E-01	-0.009	0.011	4.17E-01
6.892286	0.004	0.009	6.50E-01	-0.004	0.013	7.39E-01
0.622527771	-0.001	0.010	9.18E-01	0.018	0.015	2.18E-01
8.338946	0.002	0.009	8.45E-01	0.017	0.013	1.98E-01
0.793099095	-0.003	0.005	6.01E-01	-0.003	0.008	7.41E-01
6.061633	-0.002	0.009	8.51E-01	0.001	0.013	9.20E-01
5.727219	-0.002	0.010	8.14E-01	-0.026	0.015	8.58E-02
3.322399	0.001	0.011	9.59E-01	0.017	0.016	2.84E-01
8.704984	0.000	0.009	9.92E-01	0.010	0.013	4.66E-01
7.670118	-0.002	0.008	8.09E-01	-0.020	0.012	9.29E-02
7.847418	0.000	0.008	9.89E-01	0.009	0.012	4.59E-01
7.320564	-0.002	0.006	6.93E-01	0.002	0.009	8.29E-01
4.186695	0.001	0.014	9.29E-01	0.013	0.021	5.25E-01
0.558269146	0.002	0.009	8.38E-01	0.001	0.013	9.15E-01
3.336529	-0.001	0.009	9.19E-01	0.013	0.013	3.23E-01
2.855094	0.000	0.008	9.66E-01	-0.005	0.012	6.44E-01
0.796126988	-0.002	0.006	7.08E-01	0.003	0.008	7.57E-01
5.9422	0.001	0.009	9.46E-01	-0.021	0.014	1.21E-01
5.48667	0.001	0.008	8.76E-01	-0.006	0.011	5.94E-01
6.48352	-0.001	0.008	8.83E-01	-0.005	0.011	6.45E-01
8.258538	0.003	0.009	7.25E-01	-0.009	0.013	4.85E-01
7.270436	-0.003	0.008	7.17E-01	0.001	0.012	9.07E-01
6.776216	0.000	0.009	9.59E-01	-0.024	0.014	8.33E-02
5.291203	0.000	0.010	9.98E-01	-0.025	0.015	8.94E-02
8.990279	-0.001	0.009	8.86E-01	0.011	0.013	4.00E-01
6.155835	-0.002	0.007	7.47E-01	-0.004	0.011	7.08E-01
5.838915	0.000	0.008	9.92E-01	0.007	0.012	5.76E-01
3.329128	0.003	0.010	8.04E-01	0.012	0.015	4.30E-01
8.80154	0.002	0.006	7.65E-01	0.000	0.009	9.92E-01

0.589220944	0.003	0.009	7.27E-01	0.018	0.014	1.84E-01
7.432596	0.000	0.009	9.96E-01	-0.003	0.014	8.40E-01
2.304017862	-0.001	0.005	8.45E-01	-0.006	0.007	4.07E-01
5.991992	-0.003	0.009	7.51E-01	-0.021	0.013	1.09E-01
0.655834598	-0.003	0.011	7.67E-01	-0.008	0.016	6.29E-01
3.632254	0.001	0.004	8.91E-01	0.009	0.006	1.38E-01
4.042029	0.004	0.014	7.95E-01	-0.013	0.021	5.33E-01
5.522332	-0.002	0.007	8.23E-01	-0.012	0.011	2.52E-01
7.445381	0.002	0.009	8.38E-01	-0.021	0.014	1.36E-01
7.178926	0.003	0.010	7.75E-01	0.008	0.014	5.93E-01
8.34702	0.000	0.008	9.76E-01	-0.008	0.011	4.74E-01
2.844328603	0.000	0.008	9.72E-01	-0.015	0.012	2.40E-01
7.485753	0.001	0.008	8.91E-01	-0.007	0.013	5.74E-01
6.628522	0.001	0.008	9.34E-01	-0.008	0.013	5.29E-01
1.003033032	0.002	0.008	8.50E-01	-0.006	0.012	6.10E-01
1.756978468	0.003	0.008	7.09E-01	0.021	0.011	6.25E-02
1.866655493	0.000	0.009	9.77E-01	-0.010	0.013	4.53E-01
4.290316	0.002	0.011	8.65E-01	0.014	0.016	3.93E-01
7.13048	-0.001	0.012	9.02E-01	-0.020	0.017	2.47E-01
5.128369	0.000	0.007	9.72E-01	-0.003	0.010	7.97E-01
2.274411793	-0.002	0.014	8.82E-01	-0.003	0.020	8.78E-01
7.385159	-0.001	0.007	9.44E-01	0.000	0.011	9.99E-01
5.939172	-0.001	0.010	9.41E-01	-0.012	0.014	3.86E-01
7.962141	0.001	0.006	9.18E-01	0.006	0.010	5.24E-01
7.903602	-0.001	0.008	9.05E-01	-0.018	0.012	1.27E-01
5.778357	0.002	0.011	8.74E-01	-0.012	0.016	4.71E-01
7.951039	0.001	0.007	9.35E-01	-0.001	0.011	9.37E-01
6.866717	0.000	0.009	9.77E-01	0.004	0.013	7.59E-01
0.571053585	0.002	0.008	7.90E-01	0.003	0.012	7.79E-01
3.208012	-0.002	0.011	8.72E-01	0.011	0.017	5.16E-01
6.465689	0.002	0.009	8.41E-01	-0.001	0.014	9.42E-01
7.170515	-0.002	0.007	7.97E-01	-0.003	0.011	8.02E-01
7.450764	-0.001	0.008	8.78E-01	0.000	0.013	9.73E-01
2.075916565	0.001	0.003	7.77E-01	0.007	0.005	1.43E-01
1.132223147	0.002	0.008	8.41E-01	-0.014	0.011	2.16E-01
1.813499144	-0.001	0.011	9.55E-01	-0.006	0.016	7.17E-01
1.75327771	0.000	0.007	9.59E-01	0.012	0.011	2.78E-01
5.666998	0.001	0.010	9.22E-01	0.010	0.016	5.02E-01
3.307596	-0.001	0.010	8.80E-01	0.006	0.014	6.95E-01
0.557596281	0.002	0.010	8.41E-01	0.012	0.014	4.14E-01
6.277287	0.000	0.009	9.74E-01	-0.030	0.014	3.00E-02
3.753033	-0.003	0.011	7.92E-01	0.005	0.017	7.52E-01
2.729268657	-0.001	0.009	8.77E-01	-0.016	0.014	2.51E-01

0.798818449	-0.003	0.006	6.60E-01	0.004	0.009	6.23E-01
6.513126	-0.002	0.009	8.26E-01	0.004	0.013	7.26E-01
4.29368	0.001	0.012	9.06E-01	0.016	0.018	3.77E-01
8.600354	0.003	0.008	7.41E-01	-0.004	0.012	7.21E-01
6.687398	0.003	0.010	7.89E-01	-0.002	0.015	9.04E-01
8.388738	0.002	0.010	8.22E-01	0.015	0.015	2.91E-01
7.112649	0.000	0.009	9.88E-01	-0.002	0.013	8.77E-01
6.868062	0.001	0.008	9.39E-01	-0.011	0.011	3.45E-01
5.5876	0.000	0.006	9.81E-01	-0.002	0.010	8.25E-01
0.572062882	0.001	0.009	9.23E-01	0.023	0.014	9.37E-02
5.608122	-0.001	0.008	8.96E-01	0.001	0.011	9.19E-01
2.034871789	0.001	0.004	8.05E-01	0.008	0.006	1.85E-01
0.713028138	0.000	0.008	9.54E-01	0.010	0.012	4.01E-01
4.036309	0.002	0.015	8.76E-01	-0.007	0.022	7.58E-01
7.869622	0.001	0.008	8.81E-01	-0.004	0.011	7.43E-01
4.094849	0.003	0.011	7.98E-01	0.007	0.016	6.71E-01
5.876932	0.001	0.011	9.40E-01	-0.010	0.017	5.50E-01
4.222356	-0.001	0.013	9.19E-01	0.016	0.019	3.78E-01
8.313377	-0.002	0.009	7.92E-01	0.001	0.013	9.62E-01
8.045576	-0.002	0.009	8.64E-01	0.009	0.014	5.18E-01
0.982510644	0.002	0.006	7.82E-01	0.008	0.010	4.18E-01
0.615462687	-0.002	0.010	8.17E-01	0.001	0.015	9.35E-01
8.981195	-0.001	0.009	9.35E-01	-0.008	0.013	5.16E-01
6.601944	-0.005	0.009	5.71E-01	0.004	0.013	7.80E-01
0.991594323	-0.003	0.007	6.91E-01	0.012	0.010	2.20E-01
6.478473	-0.001	0.010	8.91E-01	-0.037	0.014	9.53E-03
2.743735258	-0.001	0.010	9.02E-01	-0.017	0.015	2.67E-01
6.623476	0.001	0.009	8.88E-01	-0.003	0.014	8.27E-01
7.974253	-0.003	0.009	7.15E-01	-0.009	0.013	5.01E-01
8.086621	0.000	0.008	9.60E-01	0.008	0.012	4.63E-01
2.733978713	-0.002	0.010	8.48E-01	-0.013	0.015	3.84E-01
2.388126009	-0.002	0.018	9.29E-01	-0.004	0.026	8.64E-01
7.807719	-0.001	0.009	9.22E-01	-0.009	0.014	5.41E-01
3.130296	-0.004	0.009	6.97E-01	-0.015	0.014	2.56E-01
6.633232	0.000	0.009	9.81E-01	-0.013	0.014	3.64E-01
0.569707854	0.002	0.009	8.46E-01	-0.014	0.014	2.97E-01
8.927703	0.001	0.008	8.71E-01	0.017	0.012	1.66E-01
0.581482995	-0.002	0.008	8.54E-01	0.009	0.013	4.84E-01
6.328425	-0.004	0.008	6.15E-01	-0.006	0.012	6.15E-01
2.275084659	-0.002	0.016	9.12E-01	0.005	0.024	8.29E-01
5.795852	-0.001	0.007	8.66E-01	0.002	0.011	8.54E-01
1.503308295	0.000	0.005	9.86E-01	-0.014	0.008	7.87E-02
0.659198923	-0.003	0.010	7.44E-01	-0.006	0.014	6.70E-01

6.480492	0.000	0.008	9.81E-01	-0.005	0.012	6.45E-01
8.608092	0.000	0.009	9.74E-01	0.002	0.013	8.87E-01
8.242726	0.000	0.005	9.30E-01	0.001	0.008	8.70E-01
7.279856	-0.003	0.009	7.24E-01	-0.013	0.014	3.39E-01
6.562245	0.003	0.009	7.74E-01	-0.002	0.014	8.93E-01
7.067567	0.000	0.008	9.53E-01	0.004	0.012	7.49E-01
6.324724	0.001	0.010	9.21E-01	-0.014	0.015	3.44E-01
8.462416	-0.002	0.006	8.00E-01	0.013	0.009	1.64E-01
6.234223	0.001	0.008	9.48E-01	-0.011	0.012	3.71E-01
4.019488	0.000	0.012	9.71E-01	0.006	0.018	7.36E-01
6.55989	-0.003	0.010	7.56E-01	0.005	0.014	7.04E-01
8.235997	0.001	0.008	9.27E-01	0.001	0.011	9.45E-01
1.703485686	-0.001	0.004	9.08E-01	0.003	0.006	6.96E-01
3.311633	-0.006	0.013	6.28E-01	0.006	0.019	7.72E-01
2.786462197	0.000	0.012	9.92E-01	-0.053	0.018	3.49E-03
1.850843161	0.000	0.008	9.78E-01	0.005	0.012	6.61E-01
6.269212	0.001	0.008	8.65E-01	-0.019	0.013	1.29E-01
5.882651	-0.002	0.007	8.13E-01	-0.004	0.011	6.82E-01
4.032945	0.002	0.014	8.72E-01	-0.011	0.021	6.10E-01
7.465903	-0.001	0.010	9.25E-01	-0.016	0.015	2.84E-01
5.872895	0.000	0.009	9.74E-01	-0.026	0.014	5.74E-02
2.732632983	-0.001	0.010	9.06E-01	-0.015	0.015	3.21E-01
7.467249	-0.003	0.008	7.65E-01	-0.018	0.013	1.61E-01
3.649748	0.001	0.004	8.90E-01	0.012	0.006	5.62E-02
4.02588	0.001	0.013	9.21E-01	0.010	0.019	6.17E-01
2.364912161	0.005	0.013	7.06E-01	0.037	0.020	6.01E-02
2.571818204	-0.001	0.008	9.17E-01	-0.002	0.011	8.27E-01
2.735660876	-0.001	0.010	9.09E-01	-0.013	0.015	4.01E-01
1.063254465	-0.001	0.006	8.46E-01	-0.014	0.009	1.32E-01
8.31304	-0.004	0.009	6.13E-01	0.011	0.013	4.05E-01
6.206299	-0.001	0.007	8.95E-01	-0.007	0.010	5.25E-01
6.557199	0.000	0.008	9.60E-01	0.010	0.012	4.33E-01
6.218075	-0.001	0.010	8.95E-01	-0.011	0.015	4.65E-01
2.635740396	0.002	0.009	8.07E-01	-0.001	0.013	9.08E-01
3.578425	0.003	0.006	6.91E-01	0.012	0.009	1.89E-01
6.41186	-0.001	0.007	8.74E-01	-0.002	0.011	8.22E-01
5.86112	0.000	0.008	9.89E-01	-0.012	0.012	3.00E-01
2.5583609	-0.002	0.011	8.43E-01	0.014	0.016	3.78E-01
6.504042	-0.003	0.009	7.67E-01	-0.020	0.013	1.27E-01
7.991411	0.003	0.009	7.70E-01	-0.002	0.014	8.58E-01
8.331881	0.003	0.008	7.41E-01	-0.009	0.013	4.55E-01
7.025176	-0.003	0.010	7.36E-01	-0.028	0.014	4.77E-02
6.526583	-0.001	0.008	9.03E-01	-0.005	0.012	6.82E-01

5.456728	0.000	0.010	9.61E-01	-0.011	0.015	4.74E-01
5.290866	0.000	0.010	9.86E-01	-0.026	0.015	8.06E-02
6.180394	0.001	0.008	8.48E-01	-0.030	0.011	8.59E-03
5.671035	-0.003	0.008	7.32E-01	-0.012	0.012	3.36E-01
7.627391	-0.004	0.014	7.94E-01	0.022	0.020	2.85E-01
6.860324	0.000	0.009	9.63E-01	0.000	0.013	9.92E-01
6.912135	0.000	0.010	9.75E-01	-0.015	0.014	3.14E-01
1.758324199	0.000	0.008	9.70E-01	0.002	0.013	8.82E-01
8.28478	-0.004	0.009	6.41E-01	0.001	0.013	9.17E-01
6.436083	0.001	0.010	9.51E-01	-0.023	0.014	1.16E-01
6.803804	-0.001	0.009	9.00E-01	-0.015	0.013	2.53E-01
5.609132	-0.002	0.007	7.78E-01	-0.018	0.010	7.85E-02
5.851027	0.002	0.010	8.61E-01	-0.006	0.014	6.61E-01
2.817750428	0.000	0.015	9.97E-01	-0.069	0.022	1.71E-03
2.820778322	0.001	0.016	9.63E-01	-0.078	0.024	1.50E-03
0.726149009	-0.003	0.007	6.95E-01	-0.004	0.011	6.83E-01
0.731531931	0.000	0.007	9.80E-01	0.001	0.010	9.55E-01
5.80359	-0.001	0.009	8.79E-01	-0.006	0.013	6.49E-01
6.8287	0.000	0.008	9.62E-01	0.007	0.012	5.55E-01
2.068178615	0.002	0.004	5.75E-01	0.012	0.006	3.77E-02
1.690364815	-0.001	0.004	7.49E-01	0.001	0.005	8.05E-01
6.070381	-0.001	0.011	9.15E-01	0.002	0.017	8.87E-01
4.232449	0.000	0.013	9.79E-01	0.006	0.019	7.45E-01
6.877819	0.003	0.008	7.18E-01	0.010	0.013	4.37E-01
7.457492	0.001	0.009	9.14E-01	0.008	0.013	5.32E-01
8.077538	0.001	0.009	9.17E-01	0.020	0.013	1.29E-01
7.582309	0.001	0.009	8.90E-01	0.011	0.014	4.24E-01
6.937704	0.000	0.009	9.99E-01	0.018	0.014	1.75E-01
6.66553	0.004	0.009	6.92E-01	0.006	0.014	6.63E-01
6.533312	0.000	0.008	9.62E-01	0.010	0.011	4.00E-01
2.824142647	0.000	0.014	9.91E-01	-0.042	0.020	3.97E-02
8.591607	-0.001	0.009	9.18E-01	0.020	0.013	1.16E-01
5.825794	0.000	0.009	9.62E-01	0.010	0.013	4.59E-01
6.639288	0.001	0.009	8.95E-01	-0.009	0.014	5.27E-01
8.72046	0.001	0.009	8.64E-01	0.008	0.013	5.09E-01
1.198500367	-0.001	0.010	9.24E-01	0.013	0.016	4.14E-01
6.385954	0.003	0.008	7.07E-01	-0.005	0.011	6.64E-01
6.87109	-0.002	0.009	8.60E-01	-0.009	0.013	5.04E-01
8.112527	-0.002	0.009	8.51E-01	-0.016	0.014	2.57E-01
5.898464	-0.001	0.009	9.35E-01	0.003	0.013	8.41E-01
1.442750428	0.001	0.004	7.23E-01	0.005	0.006	3.78E-01
4.202171	0.002	0.013	8.62E-01	0.017	0.020	4.02E-01
2.532455591	0.000	0.010	9.96E-01	-0.005	0.015	7.06E-01

3.336193	-0.002	0.009	8.38E-01	0.025	0.014	6.19E-02
8.655865	-0.002	0.010	8.43E-01	-0.021	0.015	1.51E-01
1.133232444	0.002	0.008	8.17E-01	-0.017	0.012	1.46E-01
8.644763	-0.001	0.007	8.38E-01	0.016	0.010	9.86E-02
3.897699	0.000	0.005	9.67E-01	0.017	0.007	1.87E-02
8.505143	0.000	0.009	9.90E-01	-0.005	0.014	6.94E-01
8.769916	0.001	0.007	9.04E-01	-0.005	0.011	6.25E-01
8.301938	-0.001	0.007	8.43E-01	0.005	0.010	6.61E-01
8.409597	0.001	0.008	8.70E-01	0.006	0.012	6.52E-01
8.517928	0.002	0.007	7.62E-01	0.011	0.011	3.13E-01
7.53218	-0.001	0.009	9.09E-01	0.008	0.014	5.63E-01
5.927397	0.001	0.010	9.09E-01	-0.013	0.015	3.99E-01
2.070533643	0.002	0.004	6.75E-01	0.011	0.006	7.99E-02
7.862221	-0.002	0.009	8.29E-01	0.006	0.013	6.47E-01
2.648524835	-0.003	0.025	9.17E-01	0.017	0.037	6.44E-01
5.897454	0.003	0.010	7.82E-01	0.007	0.014	6.26E-01
1.502298997	0.000	0.005	9.50E-01	-0.010	0.008	1.90E-01
7.969879	0.002	0.011	8.19E-01	-0.004	0.016	7.84E-01
6.878155	0.005	0.009	5.93E-01	-0.011	0.013	3.91E-01
8.237679	-0.004	0.010	6.61E-01	-0.014	0.015	3.66E-01
7.364637	-0.002	0.007	7.52E-01	-0.010	0.011	3.75E-01
5.078914	0.002	0.009	8.43E-01	-0.014	0.013	2.83E-01
5.654886	0.000	0.007	9.63E-01	0.014	0.010	1.54E-01
8.518937	0.005	0.009	5.52E-01	-0.006	0.013	6.84E-01
8.019671	-0.003	0.008	6.82E-01	-0.025	0.012	4.52E-02
7.57558	0.004	0.008	6.27E-01	0.014	0.012	2.42E-01
6.358367	-0.001	0.008	9.23E-01	0.017	0.011	1.27E-01
1.80004184	0.001	0.010	9.39E-01	0.012	0.015	4.43E-01
8.171402	-0.001	0.007	9.31E-01	0.012	0.011	2.55E-01
6.412196	-0.001	0.009	9.38E-01	-0.005	0.013	7.05E-01
6.367451	0.001	0.008	9.17E-01	0.002	0.012	8.90E-01
5.642438	0.002	0.007	8.06E-01	-0.001	0.011	9.00E-01
7.149656	-0.003	0.007	7.11E-01	0.001	0.011	9.31E-01
8.966729	-0.001	0.006	9.20E-01	-0.011	0.009	2.62E-01
5.874577	-0.002	0.009	8.61E-01	-0.019	0.014	1.70E-01
6.840811	-0.002	0.009	8.02E-01	0.010	0.013	4.43E-01
7.666753	-0.001	0.010	9.48E-01	0.001	0.015	9.22E-01
3.752697	-0.004	0.011	7.48E-01	0.008	0.017	6.20E-01
5.580198	0.001	0.007	8.46E-01	-0.019	0.010	6.26E-02
2.281140445	0.002	0.005	7.61E-01	-0.002	0.008	7.79E-01
7.443026	0.002	0.009	8.35E-01	-0.009	0.014	5.10E-01
5.902501	-0.006	0.009	5.22E-01	-0.010	0.014	4.74E-01
8.362496	-0.001	0.006	8.77E-01	-0.002	0.009	8.25E-01

5.828486	0.001	0.007	8.63E-01	0.021	0.010	3.47E-02
8.676724	0.001	0.009	8.75E-01	-0.024	0.014	9.38E-02
2.744408123	-0.002	0.010	8.79E-01	-0.017	0.015	2.51E-01
8.51961	0.002	0.007	8.29E-01	-0.018	0.011	8.61E-02
7.807046	0.000	0.009	9.82E-01	-0.024	0.013	7.29E-02
7.770711	-0.001	0.012	9.18E-01	-0.016	0.018	4.00E-01
7.00129	0.001	0.010	9.30E-01	-0.010	0.014	4.83E-01
8.54316	0.000	0.007	9.92E-01	0.016	0.011	1.41E-01
7.777103	0.001	0.010	9.00E-01	-0.008	0.015	5.74E-01
0.616808417	0.001	0.010	9.32E-01	0.021	0.015	1.55E-01
6.361058	0.002	0.010	8.17E-01	-0.002	0.014	8.98E-01
7.806373	-0.001	0.008	9.15E-01	-0.017	0.012	1.39E-01
5.683147	-0.001	0.009	9.35E-01	0.003	0.014	8.25E-01
4.231104	0.000	0.012	9.76E-01	0.012	0.018	5.15E-01
7.209541	-0.002	0.007	8.20E-01	-0.011	0.010	2.97E-01
7.074632	0.000	0.009	9.65E-01	-0.009	0.013	5.24E-01
8.457706	-0.003	0.009	7.70E-01	-0.013	0.014	3.41E-01
1.160483484	0.002	0.005	7.15E-01	-0.018	0.008	2.77E-02
7.705443	0.000	0.012	9.83E-01	0.028	0.017	1.07E-01
5.71679	-0.004	0.009	6.87E-01	-0.038	0.013	4.08E-03
4.230767	0.000	0.012	9.71E-01	0.009	0.019	6.22E-01
7.55203	0.002	0.008	7.94E-01	-0.013	0.012	2.86E-01
1.665468804	0.000	0.004	9.73E-01	0.016	0.006	8.57E-03
7.699724	0.000	0.009	9.68E-01	0.022	0.013	8.91E-02
1.675225349	0.000	0.004	9.67E-01	0.006	0.006	3.47E-01
1.199173232	-0.001	0.010	9.21E-01	0.009	0.016	5.54E-01
8.776981	-0.001	0.008	9.26E-01	-0.001	0.011	9.08E-01
7.812092	-0.004	0.008	5.91E-01	0.005	0.012	6.84E-01
7.908312	0.002	0.010	8.38E-01	-0.024	0.015	1.05E-01
5.418374	0.000	0.008	9.74E-01	-0.029	0.012	1.53E-02
0.943484463	0.001	0.005	7.69E-01	-0.007	0.007	3.33E-01
8.065426	0.004	0.008	6.01E-01	0.026	0.012	3.60E-02
3.750342	-0.001	0.011	9.08E-01	0.042	0.016	7.02E-03
7.948347	-0.003	0.010	7.34E-01	0.004	0.014	7.78E-01
7.842035	0.001	0.008	8.98E-01	-0.002	0.012	8.64E-01
8.039184	0.000	0.008	9.77E-01	-0.018	0.011	1.10E-01
7.966515	-0.003	0.009	7.29E-01	-0.018	0.013	1.67E-01
2.744071691	-0.002	0.010	8.82E-01	-0.017	0.015	2.53E-01
7.157058	0.001	0.007	9.10E-01	-0.007	0.011	5.28E-01
6.433055	0.001	0.010	9.44E-01	-0.001	0.014	9.72E-01
3.924277	0.000	0.009	9.72E-01	0.025	0.013	5.29E-02
6.065334	-0.002	0.008	8.39E-01	-0.017	0.012	1.53E-01
3.306251	-0.002	0.009	8.17E-01	0.002	0.013	8.98E-01

8.336591	0.000	0.009	9.92E-01	0.006	0.013	6.55E-01
2.582584047	0.006	0.012	6.46E-01	-0.014	0.018	4.59E-01
3.192536	0.001	0.007	9.44E-01	0.017	0.011	1.07E-01
6.272577	-0.004	0.008	6.24E-01	-0.012	0.012	2.83E-01
8.245081	0.001	0.007	8.81E-01	0.009	0.010	3.65E-01
6.945442	-0.002	0.008	7.87E-01	0.016	0.012	2.03E-01
5.951284	0.000	0.010	9.84E-01	-0.013	0.014	3.74E-01
7.981654	0.001	0.009	9.10E-01	0.003	0.013	8.15E-01
7.627727	-0.003	0.009	7.29E-01	0.018	0.014	1.97E-01
6.213701	0.000	0.008	9.71E-01	-0.022	0.012	6.05E-02
4.01646	0.000	0.013	9.96E-01	0.013	0.020	5.02E-01
5.941191	0.000	0.008	9.95E-01	0.003	0.012	7.74E-01
6.988169	0.001	0.007	8.77E-01	0.010	0.010	3.27E-01
8.616166	-0.003	0.008	7.47E-01	0.008	0.012	5.03E-01
2.274748226	-0.001	0.015	9.25E-01	0.001	0.022	9.69E-01
7.165469	0.001	0.008	9.43E-01	0.000	0.013	9.78E-01
8.77799	0.000	0.008	9.95E-01	-0.010	0.012	4.13E-01
8.250464	0.000	0.009	9.83E-01	-0.018	0.014	1.83E-01
8.982878	0.000	0.010	9.80E-01	-0.006	0.014	6.80E-01
8.102097	0.000	0.010	9.70E-01	-0.005	0.015	7.48E-01
6.808514	-0.002	0.008	8.41E-01	-0.004	0.012	7.42E-01
8.113199	0.001	0.009	8.91E-01	-0.002	0.013	8.76E-01
8.713732	0.000	0.008	9.94E-01	-0.009	0.012	4.73E-01
8.891704	0.000	0.009	9.58E-01	-0.011	0.013	4.02E-01
6.758722	0.004	0.009	6.79E-01	0.027	0.013	3.73E-02
6.846867	-0.001	0.009	9.08E-01	0.017	0.014	2.23E-01
3.919904	0.005	0.011	6.74E-01	0.029	0.016	6.72E-02
0.980492048	0.000	0.006	9.94E-01	0.012	0.010	2.20E-01
7.448409	-0.001	0.006	8.78E-01	-0.005	0.009	6.12E-01
8.772271	0.001	0.006	8.45E-01	-0.007	0.010	4.52E-01
2.074570834	0.000	0.003	8.88E-01	0.007	0.005	1.34E-01
8.928039	0.000	0.008	9.57E-01	0.022	0.011	5.37E-02
3.59861	0.000	0.007	9.82E-01	0.005	0.010	5.91E-01
0.62891999	-0.003	0.009	7.23E-01	-0.017	0.014	2.23E-01
7.401981	0.002	0.009	7.93E-01	-0.014	0.013	3.00E-01
6.174002	0.000	0.009	9.89E-01	-0.004	0.014	7.60E-01
6.639961	-0.003	0.010	7.37E-01	-0.011	0.014	4.59E-01
6.514808	-0.001	0.007	9.39E-01	-0.016	0.011	1.25E-01
4.039674	0.002	0.015	8.92E-01	-0.020	0.022	3.59E-01
7.698714	-0.001	0.010	9.53E-01	0.019	0.015	2.23E-01
7.774748	0.001	0.010	8.78E-01	-0.013	0.015	3.64E-01
2.730277954	-0.001	0.010	8.80E-01	-0.016	0.014	2.65E-01
8.090658	-0.004	0.010	7.20E-01	-0.016	0.015	2.78E-01

0.531690971	-0.002	0.009	8.11E-01	0.036	0.014	8.52E-03
0.981501346	0.000	0.006	9.73E-01	0.012	0.009	2.17E-01
8.215811	0.001	0.008	8.84E-01	-0.004	0.012	7.63E-01
8.400849	-0.001	0.006	8.63E-01	0.014	0.009	1.35E-01
5.517958	0.001	0.009	9.39E-01	-0.022	0.013	9.43E-02
5.654214	0.000	0.008	9.54E-01	0.004	0.012	7.40E-01
7.101547	-0.001	0.009	9.32E-01	0.001	0.014	9.62E-01
7.507621	0.002	0.009	8.62E-01	-0.013	0.013	3.14E-01
6.353657	-0.002	0.008	7.99E-01	0.002	0.012	8.94E-01
3.18749	0.000	0.006	9.88E-01	0.012	0.009	1.63E-01
3.516184	0.000	0.007	9.70E-01	0.017	0.011	1.17E-01
6.115126	0.001	0.009	9.53E-01	0.023	0.013	7.88E-02
6.826345	0.000	0.007	9.98E-01	0.008	0.010	4.38E-01
1.883813555	0.000	0.009	9.94E-01	-0.011	0.013	3.68E-01
7.962478	0.001	0.008	9.41E-01	0.007	0.012	5.36E-01
1.807443357	-0.001	0.011	9.58E-01	-0.009	0.017	6.07E-01
8.568729	0.001	0.010	8.94E-01	-0.005	0.014	7.12E-01
2.388462442	0.001	0.016	9.58E-01	-0.006	0.024	7.90E-01
8.674705	0.000	0.009	9.71E-01	-0.014	0.013	2.81E-01
8.080229	-0.001	0.010	9.08E-01	-0.015	0.014	2.90E-01
8.985569	0.002	0.008	8.44E-01	0.015	0.012	2.18E-01
4.216637	0.000	0.012	9.81E-01	0.008	0.017	6.42E-01
5.859437	0.001	0.011	9.32E-01	-0.005	0.016	7.65E-01
7.842371	0.001	0.009	8.85E-01	0.008	0.014	5.78E-01
0.713701003	0.001	0.008	8.65E-01	0.000	0.012	9.83E-01
1.812153413	0.002	0.010	8.54E-01	0.013	0.015	3.68E-01
8.953272	0.000	0.007	9.69E-01	-0.012	0.011	2.71E-01
4.033954	0.002	0.013	9.00E-01	-0.008	0.020	6.79E-01
5.619897	-0.001	0.009	9.04E-01	-0.002	0.014	8.91E-01
6.012178	0.003	0.009	7.43E-01	0.008	0.014	5.61E-01
7.958104	-0.004	0.010	7.16E-01	-0.036	0.015	1.64E-02
7.442689	0.003	0.009	7.41E-01	0.002	0.014	8.60E-01
8.72618	-0.001	0.007	8.46E-01	0.002	0.011	8.22E-01
4.290989	0.001	0.011	9.28E-01	0.009	0.017	5.73E-01
1.803069733	0.002	0.009	8.61E-01	0.013	0.014	3.45E-01
7.970216	0.002	0.010	7.96E-01	-0.007	0.014	6.07E-01
0.722448251	0.001	0.007	8.86E-01	0.005	0.011	6.55E-01
5.56405	0.000	0.009	9.82E-01	-0.029	0.014	3.22E-02
7.553039	0.001	0.006	8.91E-01	-0.015	0.009	1.04E-01
0.656507463	-0.002	0.011	8.47E-01	-0.014	0.017	4.02E-01
7.893172	0.001	0.008	8.61E-01	0.010	0.012	4.15E-01
4.089802	0.006	0.010	5.55E-01	0.006	0.015	6.77E-01
8.665285	-0.001	0.008	8.49E-01	0.002	0.011	8.47E-01

8.545515	0.000	0.010	9.63E-01	-0.007	0.015	6.45E-01
7.81041	-0.002	0.010	8.53E-01	-0.006	0.015	7.12E-01
2.516643259	-0.001	0.017	9.55E-01	-0.007	0.025	7.92E-01
8.305975	-0.001	0.010	9.02E-01	-0.019	0.015	2.18E-01
7.733703	0.002	0.011	8.75E-01	0.001	0.017	9.58E-01
5.289857	-0.001	0.010	9.22E-01	-0.025	0.014	8.39E-02
2.747436017	-0.002	0.008	8.11E-01	-0.019	0.013	1.24E-01
8.602372	0.000	0.009	9.95E-01	-0.016	0.014	2.47E-01
7.48979	0.000	0.007	9.71E-01	0.000	0.011	9.91E-01
4.04001	0.002	0.015	8.89E-01	-0.021	0.022	3.34E-01
8.753431	0.000	0.009	9.85E-01	0.015	0.014	2.76E-01
2.36356643	0.000	0.009	9.63E-01	0.029	0.013	3.07E-02
1.727708833	0.000	0.005	9.38E-01	0.008	0.007	2.61E-01
7.39828	0.002	0.007	7.57E-01	-0.010	0.010	3.29E-01
3.207676	-0.001	0.011	9.23E-01	0.012	0.017	4.80E-01
7.514349	0.000	0.006	9.58E-01	0.010	0.009	2.74E-01
0.644732322	-0.001	0.009	9.50E-01	0.002	0.013	9.08E-01
0.981164913	0.000	0.006	9.80E-01	0.012	0.009	1.97E-01
6.009823	-0.001	0.008	9.50E-01	-0.007	0.012	5.81E-01
5.590628	0.003	0.010	7.79E-01	-0.013	0.015	3.95E-01
6.748629	0.001	0.012	9.14E-01	0.015	0.017	3.78E-01
8.341301	-0.001	0.010	9.57E-01	-0.006	0.015	6.77E-01
7.021812	-0.004	0.009	7.04E-01	-0.024	0.014	9.21E-02
2.80631172	0.000	0.014	9.82E-01	-0.067	0.022	1.81E-03
0.799154881	-0.002	0.006	7.01E-01	0.005	0.009	5.78E-01
2.06885148	0.002	0.004	6.26E-01	0.012	0.006	4.82E-02
2.164061904	0.000	0.011	9.80E-01	-0.026	0.017	1.24E-01
3.438132	0.002	0.009	8.55E-01	0.017	0.013	1.99E-01
0.614789821	-0.001	0.008	9.21E-01	-0.007	0.012	5.21E-01
5.142163	0.001	0.007	9.24E-01	-0.014	0.011	2.03E-01
3.333165	-0.002	0.009	8.63E-01	0.009	0.014	5.35E-01
1.160147052	0.002	0.005	6.60E-01	-0.016	0.008	4.22E-02
8.631642	-0.001	0.011	8.92E-01	0.001	0.016	9.54E-01
2.15195033	0.000	0.008	9.74E-01	0.001	0.012	9.09E-01
0.540774651	0.002	0.009	8.47E-01	0.009	0.013	4.76E-01
1.778846587	0.000	0.009	9.97E-01	0.004	0.014	7.97E-01
7.972907	0.000	0.006	9.93E-01	0.002	0.009	8.67E-01
5.29053	0.000	0.010	9.60E-01	-0.025	0.015	8.22E-02
1.690701248	-0.001	0.004	7.68E-01	0.001	0.005	9.16E-01
6.411523	-0.001	0.007	9.27E-01	-0.016	0.011	1.20E-01
0.525635185	0.001	0.010	9.01E-01	-0.011	0.014	4.34E-01
7.879379	0.000	0.008	9.63E-01	0.009	0.012	4.64E-01
6.476455	-0.003	0.010	7.66E-01	-0.021	0.016	1.78E-01

0.726485442	-0.003	0.007	6.57E-01	0.001	0.011	9.17E-01
6.568637	-0.001	0.008	8.88E-01	-0.015	0.011	1.72E-01
2.275421091	-0.003	0.017	8.58E-01	0.005	0.025	8.45E-01
6.944433	0.000	0.008	9.83E-01	0.018	0.013	1.48E-01
6.409505	0.000	0.008	9.73E-01	-0.001	0.012	9.60E-01
7.898892	0.000	0.008	9.94E-01	-0.004	0.013	7.68E-01
8.405896	-0.002	0.008	8.06E-01	-0.004	0.013	7.67E-01
4.034291	0.002	0.016	9.18E-01	-0.019	0.023	4.21E-01
8.401522	0.000	0.010	9.75E-01	-0.010	0.014	5.02E-01
5.908893	-0.003	0.008	7.39E-01	-0.015	0.012	1.99E-01
2.730614387	-0.002	0.010	8.61E-01	-0.016	0.014	2.65E-01
1.682626866	0.002	0.004	6.55E-01	0.008	0.006	1.58E-01
0.551204062	0.004	0.009	6.98E-01	-0.004	0.014	7.68E-01
7.110294	0.000	0.009	9.77E-01	-0.002	0.013	8.52E-01
7.024504	-0.003	0.009	7.37E-01	-0.025	0.014	7.37E-02
5.860783	0.002	0.008	7.86E-01	-0.003	0.013	7.94E-01
1.923512601	-0.001	0.004	8.78E-01	0.002	0.006	6.87E-01
6.57873	-0.002	0.009	8.22E-01	-0.014	0.014	3.12E-01
3.899045	0.000	0.005	9.31E-01	0.019	0.007	1.11E-02
4.211927	0.002	0.010	8.06E-01	0.007	0.015	6.14E-01
3.917885	0.000	0.010	9.78E-01	0.026	0.014	6.41E-02
1.1988368	-0.001	0.011	8.93E-01	0.011	0.016	5.01E-01
6.54105	0.001	0.010	9.17E-01	-0.002	0.015	9.08E-01
7.662043	-0.001	0.009	9.49E-01	-0.013	0.014	3.36E-01
6.925929	-0.003	0.012	8.25E-01	0.013	0.017	4.55E-01
5.08026	0.002	0.010	8.74E-01	-0.006	0.014	6.51E-01
8.590934	0.001	0.006	9.16E-01	0.007	0.009	4.13E-01
6.175684	0.002	0.009	8.08E-01	-0.015	0.014	2.80E-01
0.981837778	0.001	0.006	8.86E-01	0.010	0.010	2.88E-01
2.035544654	0.001	0.004	8.26E-01	0.008	0.006	1.63E-01
4.041692	0.003	0.014	8.30E-01	-0.011	0.021	6.16E-01
3.319371	0.001	0.011	9.08E-01	0.008	0.017	6.22E-01
5.796188	0.000	0.010	9.94E-01	0.008	0.015	5.70E-01
5.952966	0.004	0.011	7.18E-01	0.012	0.016	4.53E-01
8.493368	-0.002	0.008	8.00E-01	-0.001	0.012	9.34E-01
8.218166	0.000	0.006	9.36E-01	-0.005	0.009	5.68E-01
0.510159286	-0.002	0.007	8.32E-01	-0.022	0.011	4.67E-02
2.281476878	0.001	0.005	7.69E-01	-0.004	0.007	6.15E-01
3.938744	0.002	0.007	7.72E-01	0.012	0.010	2.40E-01
0.673665525	-0.001	0.010	9.12E-01	0.007	0.015	6.60E-01
6.319341	0.002	0.011	8.41E-01	0.007	0.017	6.92E-01
4.046402	0.003	0.009	7.36E-01	-0.011	0.014	4.19E-01
7.274473	-0.004	0.010	7.11E-01	0.016	0.015	3.13E-01

7.481043	0.001	0.009	9.43E-01	-0.003	0.013	8.20E-01
5.936481	0.001	0.008	8.52E-01	-0.005	0.011	6.33E-01
0.534046	0.001	0.010	8.96E-01	-0.009	0.014	5.16E-01
7.602158	0.007	0.011	5.33E-01	0.021	0.016	1.94E-01
6.275605	0.003	0.009	7.60E-01	-0.004	0.013	7.56E-01
5.967769	-0.001	0.009	9.09E-01	0.008	0.014	5.72E-01
6.448867	-0.001	0.009	9.33E-01	-0.017	0.014	2.19E-01
0.982174211	0.001	0.006	8.34E-01	0.011	0.010	2.61E-01
6.891949	0.007	0.009	4.54E-01	-0.004	0.014	7.72E-01
2.035208221	0.001	0.004	8.33E-01	0.008	0.006	1.75E-01
1.500616834	-0.001	0.005	8.92E-01	-0.009	0.008	2.39E-01
7.854146	-0.003	0.008	7.51E-01	0.019	0.012	1.26E-01
0.723457548	-0.002	0.007	7.88E-01	0.000	0.011	9.65E-01
0.685440666	0.001	0.014	9.24E-01	-0.001	0.021	9.60E-01
5.907211	-0.001	0.009	9.48E-01	-0.018	0.013	1.71E-01
3.634272	0.000	0.004	9.15E-01	0.009	0.006	1.13E-01
1.675898214	0.001	0.004	9.04E-01	0.003	0.006	6.16E-01
7.145619	-0.002	0.010	8.37E-01	-0.004	0.015	7.90E-01
2.851057255	0.001	0.008	9.05E-01	-0.004	0.011	7.13E-01
8.878247	0.002	0.009	8.03E-01	-0.010	0.013	4.36E-01
0.722784683	0.001	0.007	9.36E-01	0.003	0.011	7.56E-01
7.112985	0.000	0.009	9.69E-01	0.001	0.014	9.62E-01
7.920087	-0.002	0.009	8.46E-01	-0.001	0.013	9.42E-01
7.624699	-0.002	0.014	8.90E-01	0.006	0.021	7.66E-01
5.290194	-0.001	0.010	9.46E-01	-0.025	0.014	8.57E-02
0.507467825	0.000	0.008	9.66E-01	0.005	0.012	6.54E-01
8.799185	-0.001	0.006	8.61E-01	0.004	0.010	6.78E-01
6.32035	0.002	0.009	8.32E-01	0.002	0.013	8.46E-01
2.163389038	0.001	0.011	9.64E-01	-0.020	0.017	2.46E-01
6.247681	0.000	0.006	9.35E-01	-0.006	0.009	5.19E-01
6.351302	-0.004	0.010	7.03E-01	-0.003	0.015	8.41E-01
4.35592	0.001	0.015	9.44E-01	0.010	0.022	6.54E-01
7.489453	-0.001	0.009	8.85E-01	-0.004	0.013	7.59E-01
2.537165647	-0.002	0.013	8.54E-01	0.009	0.019	6.35E-01
1.818882065	-0.002	0.010	8.74E-01	0.011	0.015	4.45E-01
4.366013	0.000	0.015	9.76E-01	0.011	0.023	6.39E-01
6.122191	0.000	0.010	9.69E-01	-0.013	0.015	3.86E-01
6.737527	-0.002	0.010	8.28E-01	-0.007	0.014	6.12E-01
3.694157	0.000	0.005	9.54E-01	0.017	0.007	2.16E-02
5.145191	0.000	0.009	9.62E-01	-0.020	0.014	1.43E-01
5.976852	0.001	0.009	9.46E-01	-0.020	0.013	1.11E-01
1.501962564	0.000	0.005	9.26E-01	-0.009	0.008	2.40E-01
6.808177	-0.001	0.009	8.83E-01	0.002	0.013	8.85E-01

0.539428921	0.001	0.007	9.13E-01	0.030	0.011	5.71E-03
8.012943	-0.001	0.010	8.89E-01	-0.017	0.015	2.46E-01
6.020252	0.000	0.009	9.96E-01	-0.011	0.013	3.75E-01
8.49976	0.000	0.008	9.94E-01	0.013	0.012	2.65E-01
7.306098	-0.001	0.011	9.32E-01	0.002	0.016	8.83E-01
0.856011989	0.000	0.008	9.73E-01	-0.010	0.011	3.97E-01
3.317016	-0.003	0.013	8.13E-01	0.016	0.019	3.88E-01
7.296341	0.001	0.009	9.40E-01	-0.006	0.014	6.74E-01
6.38831	0.002	0.009	8.22E-01	0.007	0.014	6.17E-01
0.82371446	-0.002	0.007	7.68E-01	0.008	0.010	4.39E-01
6.008814	0.001	0.008	8.56E-01	-0.007	0.012	5.79E-01
2.072552239	0.001	0.003	8.15E-01	0.011	0.005	2.63E-02
6.577721	-0.001	0.011	9.55E-01	-0.007	0.016	6.78E-01
1.765725716	0.001	0.010	8.89E-01	0.008	0.015	5.82E-01
4.289643	0.001	0.011	9.08E-01	0.011	0.016	4.84E-01
6.163573	0.001	0.009	9.17E-01	-0.006	0.013	6.26E-01
6.849895	-0.001	0.009	9.36E-01	0.001	0.013	9.52E-01
2.746763151	-0.002	0.009	8.28E-01	-0.016	0.013	2.25E-01
5.783404	-0.001	0.009	9.41E-01	-0.018	0.014	1.99E-01
1.849833863	0.001	0.008	9.22E-01	0.004	0.012	7.31E-01
8.14045	0.002	0.010	8.30E-01	0.013	0.015	3.89E-01
8.400176	-0.001	0.008	8.49E-01	0.004	0.011	6.93E-01
2.078608025	0.002	0.004	6.42E-01	0.010	0.005	5.35E-02
3.171341	-0.001	0.006	8.55E-01	0.012	0.009	1.73E-01
5.90351	-0.006	0.008	4.63E-01	-0.018	0.012	1.56E-01
8.542824	0.000	0.009	9.94E-01	0.024	0.013	6.36E-02
3.644365	0.001	0.004	8.71E-01	0.013	0.006	2.54E-02
8.953944	0.001	0.009	9.40E-01	-0.001	0.013	9.12E-01
8.05937	0.001	0.007	8.32E-01	0.000	0.010	9.78E-01
1.882131392	0.001	0.008	8.74E-01	-0.006	0.012	5.86E-01
4.018815	0.000	0.013	9.92E-01	0.016	0.020	4.13E-01
5.962049	0.000	0.008	9.94E-01	-0.012	0.012	2.96E-01
0.631947884	-0.001	0.009	8.96E-01	0.013	0.013	3.53E-01
7.909994	-0.003	0.009	7.84E-01	0.000	0.014	9.82E-01
2.73364228	-0.002	0.010	8.69E-01	-0.013	0.015	3.75E-01
6.635587	0.001	0.007	8.36E-01	-0.008	0.010	4.61E-01
0.958960362	0.000	0.005	9.62E-01	0.011	0.007	1.41E-01
8.343656	-0.001	0.009	9.31E-01	0.031	0.014	2.60E-02
4.095185	0.003	0.010	7.78E-01	0.011	0.015	4.73E-01
8.392438	-0.001	0.009	9.12E-01	0.001	0.014	9.20E-01
5.965414	0.000	0.007	9.69E-01	-0.008	0.010	4.28E-01
2.816068265	-0.002	0.015	8.97E-01	-0.068	0.023	2.96E-03
5.145528	-0.001	0.009	9.25E-01	-0.017	0.014	2.23E-01

8.806923	0.000	0.006	9.41E-01	-0.012	0.008	1.46E-01
6.393356	-0.001	0.009	9.28E-01	-0.002	0.013	9.04E-01
6.060624	-0.001	0.008	9.12E-01	-0.029	0.013	2.26E-02
0.596622461	0.000	0.009	9.98E-01	0.004	0.013	7.49E-01
8.205718	0.002	0.007	8.32E-01	0.002	0.011	8.23E-01
6.36846	-0.001	0.006	8.50E-01	-0.003	0.009	7.62E-01
8.848641	-0.003	0.008	6.91E-01	-0.002	0.011	8.81E-01
6.038083	-0.003	0.008	7.27E-01	0.002	0.012	8.50E-01
5.646476	0.000	0.008	9.57E-01	-0.016	0.012	1.67E-01
6.151461	0.001	0.010	9.32E-01	0.015	0.015	2.99E-01
8.083593	0.000	0.009	9.93E-01	0.000	0.014	9.89E-01

Minimally adjusted models control for age, sex, race/ethnicity, and data collection site

Fully adjusted models control for age, sex, race/ethnicity, data collection site, smoking status, income leve

Supplementary Table 4: Pearson correlations Between Spectral Features Showing Significant Associations with Unprocessed Meat Intake

ppm	2.46382334	2.46315048	2.46348691	2.46281405	2.46415978	7.052427
2.46382334	-					
2.46315048	0.93	-				
2.46348691	0.97	0.97	-			
2.46281405	0.89	0.97	0.93	-		
2.46415978	0.97	0.88	0.93	0.84	-	
7.052427	0.43	0.36	0.38	0.35	0.45	-
7.05276389	0.51	0.45	0.48	0.44	0.55	0.98

Abbreviations: ppm: chemical shift in parts per million

Supplementary Table 5: Standardized Parameter Estimates from Linear Regression Models Examining the Associations Between Six Markers of Inflammation with Spectral Features

	Glutamine / proline betaine ppm: 2.464159775			Histidine ppm: 7.052427453		
	β	SE	P	β	SE	P
C-Reactive Protein	**-0.18**	**0.02**	**$<2.0*10^{-16}$**	**-0.16**	**0.02**	**$<2.0*10^{-16}$**
Interleukin-2	0.02	0.03	0.4	**-0.12**	**0.03**	**0.0001**
Interleukin-6	**-0.07**	**0.02**	**0.0001**	**-0.14**	**0.02**	**$1.8*10^{-14}$**
Fibrinogen	-0.03	0.02	0.06	**-0.22**	**0.02**	**$<2.0*10^{-16}$**
Total Homocysteine	0.01	0.02	0.63	**-0.07**	**0.02**	**$2.8*10^{-5}$**
TNF-α	-0.04	0.03	0.17	**-0.13**	**0.03**	**$1.2*10^{-5}$**

Note: **Significant results (P<.004) in bold**.

Abbreviations: ppm: chemical shift in parts per million

All models controls for age, sex, race/ethnicity, data collection site, smoking status, education level, income level, total energy intake, and physical activity levels

Supplementary Table 6: Parameter Estimates for Associations Between Sentinel Spectral Features and Food Groups

Food group	Glutamine / proline betaine ppm: 2.43823342			Histidine ppm: 7.052427453		
	b	SE	P	*b*	SE	P
Fats and Oils	-0.01	0.00	0.00	0.00	0.00	0.16
Green Leafy Vegetables	0.03	0.01	0.01	0.01	0.01	0.14
Other Alcohol	-0.01	0.01	0.02	-0.01	0.00	0.01
Legumes	-0.02	0.01	0.03	-0.01	0.01	0.03
Tomatoes	-0.01	0.01	0.04	-0.01	0.00	0.03
Refined Grains	-0.01	0.00	0.04	-0.01	0.00	0.01
Fried Potatoes	-0.05	0.02	0.04	-0.06	0.02	0.00
Other Soups	-0.03	0.01	0.05	-0.04	0.01	0.00
Soy Foods and Beverages	0.01	0.01	0.06	0.01	0.01	0.31
Meal Rpleacement Drinks	0.03	0.02	0.11	0.01	0.01	0.36
Ice Cream	-0.03	0.02	0.11	0.01	0.01	0.59
Whole Grains	0.01	0.00	0.11	0.01	0.00	0.01
High Fat Chinese Dishes	-0.04	0.02	0.12	-0.03	0.02	0.08
Yellow Vegetables	0.01	0.01	0.12	0.00	0.01	0.80
Avocado and Guacamole	-0.04	0.03	0.17	-0.01	0.02	0.63
Coffee	0.00	0.00	0.19	0.00	0.00	0.07
Sweet Breads	0.02	0.02	0.22	0.00	0.01	0.89
Hot Chocolate	-0.02	0.02	0.23	-0.04	0.02	0.03
Poultry	-0.01	0.01	0.25	-0.02	0.01	0.03
Tea	0.00	0.00	0.26	0.00	0.00	0.27
Low-fat Dairy	0.00	0.00	0.27	0.00	0.00	0.13
Coffee Creamers	0.00	0.00	0.28	0.00	0.00	0.68
Pizza	0.05	0.05	0.29	0.01	0.04	0.79
Sweet Extras	0.00	0.00	0.36	0.01	0.00	0.00
Diet Sodas	0.00	0.00	0.36	0.00	0.00	0.25
High Fat Dairy	-0.01	0.01	0.39	-0.01	0.01	0.12
Starchy Vegetables	-0.01	0.02	0.40	-0.02	0.01	0.26
Seeds, Nuts and Peanut Butter	0.01	0.01	0.40	0.01	0.01	0.06
Beer	-0.01	0.01	0.41	0.00	0.01	0.66
Cottage Cheese	-0.02	0.03	0.41	-0.01	0.02	0.72
Low-fat Dairy Desserts	0.01	0.02	0.45	0.00	0.01	0.86
Whole Milk	0.00	0.01	0.46	0.01	0.00	0.07
Starchy Foods with Mayonnaise	-0.02	0.03	0.46	-0.04	0.02	0.11
Sweets and Desserts	0.01	0.01	0.46	0.00	0.01	0.75
Protein Foods with Mayonniase	-0.02	0.04	0.53	-0.04	0.03	0.23
Other Vegetables	0.00	0.01	0.55	0.00	0.00	0.93
Cream Based Soups	-0.02	0.03	0.55	-0.02	0.03	0.49

Salty Snacks	0.01	0.01	0.57	0.00	0.01	0.87
Fruit Juice	0.00	0.01	0.57	0.00	0.00	0.52
Fish	0.00	0.01	0.66	-0.01	0.01	0.37
Cruciferous Vegetables	0.00	0.01	0.72	0.00	0.01	0.60
Yogurt	0.00	0.01	0.84	-0.01	0.01	0.43
Non-diet Soda	0.00	0.00	0.86	0.00	0.00	0.69
Fruit Juice	0.00	0.00	0.86	0.00	0.00	0.83
Eggs	0.00	0.01	0.93	0.00	0.01	0.95

Abbreviations: ppm: chemical shift in parts per million

Supplementary Table 7: Standardized Parameter Estimates from Linear Regression Models Examining the Associations Between BMI and Red Meat Intake, Sentinel Spectral Features and Six Markers of Inflammation

	B	SE	P
Red meat intake			
Unprocessed red meat	**0.08**	**0.01**	**$3.9*10^{-7}$**
Processed red meat	**0.08**	**0.02**	**$2.8*10^{-6}$**
Sentinel Spectral Features			
Glutamine / Proline betaine	**-0.18**	**0.02**	**$<2.0*10^{-16}$**
Histidine	**-0.2**	**0.02**	**$<2.0*10^{-16}$**
Markers of Inflammation			
C-Reactive Protein	**0.35**	**0.02**	**$<2.0*10^{-16}$**
Interleukin-2	**0.12**	**0.03**	**$1.5*10^{-4}$**
Interleukin-6	**0.35**	**0.02**	**$<2.0*10^{-16}$**
Fibrinogen	**0.26**	**0.02**	**$<2.0*10^{-16}$**
Homocysteine	**0.09**	**0.2**	**$1.2*10^{-7}$**
TNF-a	**0.24**	**0.03**	**$3.2*10^{-16}$**

Note: **Significant results (P<.005; Bonferroni corrected) in bold**.

All models controls for age, sex, race/ethnicity, data collection site, smoking status, education level, income level, total energy intake, and physical activity levels,
